# Synthetic Lethality between Cohesin and WNT Signaling Pathways in Diverse Cancer Contexts

**DOI:** 10.3390/cells13070608

**Published:** 2024-03-30

**Authors:** Maria Michela Pallotta, Maddalena Di Nardo, Antonio Musio

**Affiliations:** Institute for Biomedical Technologies (ITB), National Research Council (CNR), 56124 Pisa, Italy; mariamichela.pallotta@itb.cnr.it (M.M.P.); maddalena.dinardo@itb.cnr.it (M.D.N.)

**Keywords:** cohesin, WNT, β-catenin, cancer, synthetic lethality, LY2090314, c-MYC

## Abstract

Cohesin is a highly conserved ring-shaped complex involved in topologically embracing chromatids, gene expression regulation, genome compartmentalization, and genome stability maintenance. Genomic analyses have detected mutations in the cohesin complex in a wide array of human tumors. These findings have led to increased interest in cohesin as a potential target in cancer therapy. Synthetic lethality has been suggested as an approach to exploit genetic differences in cancer cells to influence their selective killing. In this study, we show that mutations in *ESCO1*, *NIPBL*, *PDS5B*, *RAD21*, *SMC1A*, *SMC3*, *STAG2*, and *WAPL* genes are synthetically lethal with stimulation of WNT signaling obtained following LY2090314 treatment, a GSK3 inhibitor, in several cancer cell lines. Moreover, treatment led to the stabilization of β-catenin and affected the expression of *c-MYC,* probably due to the occupancy decrease in cohesin at the *c-MYC* promoter. Finally, LY2090314 caused gene expression dysregulation mainly involving pathways related to transcription regulation, cell proliferation, and chromatin remodeling. For the first time, our work provides the underlying molecular basis for synthetic lethality due to cohesin mutations and suggests that targeting the WNT may be a promising therapeutic approach for tumors carrying mutated cohesin.

## 1. Introduction 

Cohesin is a member of the larger family of SMC (structural maintenance of chromosomes) complexes. The core of cohesin is composed of four subunits: SMC1A, SMC3, RAD21, and STAG1/2. Its activity is closely regulated by many accessory factors. Cohesin loading onto chromatin, mediated by the complex NIPBL–MAU2, occurs in G1 in yeast or at the end of the telophase of the previous cell cycle in mammalian cells. ESCO1/2 factors allow cohesin establishment in the S phase whereas PDS5 proteins ensure its maintenance. Once loaded onto the cohesin ring, the interaction of SMC3 head domain with DNA stimulates SMC3–SMC1A interface ATPase activity, responsible for opening of the cohesin ring. This allows WAPL to open the SMC3–RAD21 interface, totally releasing the DNA. Cohesin is phosphorylated at RAD21 and STAG subunits by polo-like kinase 1 during prophase and prometaphase; this process permits the removal of cohesin from chromosome arms. A modest amount of cohesin remains on chromosomes, especially at centromeres. Centromeric cohesin is certainly preserved by SGO1–PP2A complex, which stores cohesin in a hypophosphorylated state and maintains centromeric cohesion. At the metaphase–anaphase transition, the remaining cohesin is dissolved by the endopeptidase separase (ESPL1), which cleaves the cohesin’s RAD21 subunit. This cleavage permits opening of the cohesin ring, causing it to dissociate from chromosomes [1,2].

Ample experimental evidence highlights the role of cohesin as a pivotal player in various aspects of chromosome biology: correct chromosome segregation, DNA repair, genome stability, chromatin structure, 3D genome organization, and gene expression regulation. Considering its roles, it is not surprising that cancer genome and exome sequencing have revealed that cohesin subunits undergo a wide spectrum of somatic mutations in cancer. In fact, cohesin mutations have been identified in breast cancer [3,4], urothelial bladder carcinoma [5,6,7,8,9], lung carcinoma [10], Ewing’s sarcoma [11,12,13], colorectal carcinoma [14,15,16,17,18], glioblastoma [19,20], melanoma [21], and myeloid neoplasms [22,23,24,25]. According to the COSMIC (Catalogue of Somatic Mutations in Cancer, URL https://cancer.sanger.ac.uk/cosmic accessed on 01 August 2023), *STAG1* (5%), *NIPBL* (4.9%), *STAG2* (3.4%), and *PDS5B* (3.4%) are the most frequently mutated in cancer [26]. The multifaceted roles of cohesin may aid in hampering the growth of cohesin-mutant cancer cells by interfering with pathways that depend on correct cohesin function. Synthetic lethality indicates a genetic interaction where the concomitant dysfunction of two genes causes cell death, whereas with the alteration of a single gene, the cell remains viable. For instance, *STAG2* shows a strong synthetic lethal interaction with its paralog *STAG1* [27,28] while the inhibition of *poly-ADP ribose polymerase* (PARP) displays synthetic lethality with cohesin variants [29,30]. Recently, we showed that cohesin-mutant cancer cells were highly sensitive to treatment with LY2090314, a GSK3 inhibitor that acts as an agonist of the WNT signaling pathway. In particular, our investigation revealed that the stabilization of β-catenin in response to mutated cohesin probably plays a significant role in the heightened sensitivity of WNT target genes [31]. However, an outstanding question remains regarding the precise mechanisms underlying β-catenin stabilization and WNT activation in cohesin-mutant cells. Furthermore, to date, only a limited number of cohesin mutations have been tested for synthetic lethality. A larger number of genetic interactions can identify key processes that result in synthetic lethality when altered, providing a therapeutic strategy for cohesin-mutant cancer. In this study we first performed whole exome sequencing (WES) in cells belonging to the NCI-60 cancer cell line panel in order to identify mutations in cohesin genes. Via this approach, we detected fifteen mutations on both core cohesin and regulatory cohesin genes, namely *ESCO1*, *ESPL1*, *NIPBL*, *PDS5B*, *RAD21*, *SMC1A*, *SMC3*, *STAG2*, and *WAPL* genes. Next, cell lines harboring cohesin gene variants were treated with LY2090314. We found that treatment led to a strong decrease in cell viability and β-catenin stabilization in all cell lines except for that carrying *ESPL1* mutation. Of note, the combination of LY2090314 treatment and cohesin deficiency caused a decrease in c*-MYC* (from hereon *MYC*) expression, likely to have been triggered by the occupancy decrease in both cohesin and β-catenin on the *MYC* promotor. Finally, RNA-seq analysis showed that LY2090314 treatment caused gene expression dysregulation affecting pathways related to chromatin remodeling, regulation of transcription, regulation of cell proliferation, and intracellular signal transduction. All together, these results indicate that all mutated cohesin genes, except for *ESPL1*, are synthetically lethal with stimulation of WNT signaling and this may offer a potential therapeutic target in tumors where cohesin is mutated.

## 2. Materials and Methods

### 2.1. Cell Culture and Treatment

The NCI-60 cancer cell line panel was purchased from the National Cancer Institute (USA). Cells were grown in RPMI supplemented with 10% FBS, 100 U/mL penicillin, 0.1 mg/mL streptomycin, and 1% L-glutamine in a humidified 5% CO_2_ atmosphere at 37 °C. Cells were treated with LY2090314 (Merck, Darmstadt, Germany) 100 nM for 24 h.

### 2.2. Whole Exome Sequencing (WES)

Genomic DNA was isolated from a diverse panel of NCI-60 cancer cell lines utilizing the PureDireX-Genomin DNA Isolation Kit (Bio-Helix, New Taipei City, Taiwan) employing a nonorganic extraction procedure. Subsequently, library preparation and exome enrichment were performed using the Nextera Flex for Enrichment solution (Illumina, San Diego, CA, USA) in conjunction with ‘SureSelect Human All Exon V7’ probes (Agilent Technologies, Santa Clara, CA, USA), targeting 35 Mb of human exonic content. Quantification and quality assessment of the samples were conducted utilizing the Qubit 2.0 Fluorometer (Thermo Fisher Scientific, Waltham, MA, USA) and Agilent 2100 Bioanalyzer (Agilent Technologies, Santa Clara, CA, USA). The constructed libraries were subjected to sequencing on the NovaSeq 6000 platform (Illumina, San Diego, CA, USA) in 150 pair-end mode. Raw sequencing data underwent initial processing, including format conversion and de-multiplexing, using the Bcl2Fastq 2.0.21 version of the Illumina pipeline. Adapter sequences were subsequently masked from the raw fastq data utilizing Cutadapt v1.112 (accessed on 01 September 2023), employing parameters such as --anywhere (on both adapter sequences), --overlap 5, --times 2, --minimum-length 35, and --mask-adapter.

Further analysis was carried out using the Illumina DRAGEN Somatic Pipelines 3.5.7, (https://emea.illumina.com/products/by-type/informatics-products/basespacesequencehub/apps/edico-genome-inc-dragen-somatic-pipeline.html), which facilitated read mapping to the GRCh38/hg38 assembly and variant identification. Variants were functionally annotated using Annovar [32] (version Build #21596031) and SnpEff [33] (version 5.2). SNP and indel summary reports were generated, containing comprehensive information such as variant coordinates, base pair changes, amino acid change annotation, and functional annotations, including clinical significance, population frequencies, and various scores (SIFT, PolyPhen, LRT, MutationTaster), as well as HPO and other pertinent information essential for variant prioritization.

### 2.3. MTT Assay

HCT116, HCT15, A549, CCRF CEM, SF539, RFX393, UO31, IGROV1, HCC2998 cancer cell lines, along with MCF7 as the control cell line, were seeded onto 96-well plates at a concentration of 2 × 10^3^ cells/well. Subsequently, MTT (3-(4,5-dimethyl-thiazol-2-yl)-2,5-diphenyltetrazolium bromide) solution at a concentration of 5 mg/mL was added to each well, and the plates were incubated at 37 °C for 4 h to allow the formation of formazan crystals. Following the incubation period, the formazan crystals were dissolved by adding acidified isopropanol (in 0.01 M HCl) to each well. The optical density of the resulting solution was measured using a microplate reader at a wavelength of 595 nm, allowing the quantification of cell viability.

### 2.4. Co-Immunoprecipitation (Co-IP)

For co-immunoprecipitation (co-IP) experiments, 800 μg of total protein extract from MCF7 cells was dissolved in 1 mL of incubation buffer. The solution underwent preclearing by incubation with 20μL of Dynabeads protein A (Invitrogen) for 1 h to reduce non-specific binding. Following preclearing, the supernatants were then incubated overnight at 4 °C with 7 μg of β-catenin antibody coupled to 40μL of Dynabeads protein A, facilitating the specific immunoprecipitation of target proteins. Subsequently, the immunocomplexes were boiled in sample buffer to dissociate the bound proteins and proteins were separated by SDS-PAGE. The Western blotting procedure involved probing the membrane with specific antibodies against NIPBL, RAD21, SMC1A, andSMC3.

### 2.5. Western Blotting

The harvested cell pellets were resuspended in lysis buffer containing tris HCl pH 8.0 (25 μM), NaCl (55 μM), EDTA (1 μM), and Protease Inhibitor Cocktail (Merck, Darmstadt, Germany). The protein concentration in the lysates was determined using the Bradford Protein Assay (Thermo Fisher Scientific, Waltham, MA, USA). Subsequently, proteins were loaded at a concentration of 5μg per lane and separated by SDS-PAGE. Following electrophoresis, proteins were transferred onto nitrocellulose membranes (Amersham, Marlborough, MA, USA) for subsequent immunoblotting. Non-specific binding was blocked with freshly prepared 5% BSA for 1 h on a shaking platform at room temperature. The membranes were then incubated with primary antibodies at a dilution of 1:1000, unless otherwise indicated, allowing specific detection of target proteins. After washing to remove unbound primary antibodies, the membranes were incubated with secondary antibody–peroxidase conjugate (Merck, Darmstadt, Germany) to facilitate detection. Chemiluminescence detection (Amersham, Marlborough, MA, USA) was employed to visualize the protein bands on the membranes, and images were captured using a Chemidoc imaging system (Bio-Rad, Hercules, CA, USA). To ensure equal loading of protein samples, anti-tubulin antibody was used as a loading control.

### 2.6. Antibodies

Primary antibodies were as follows: anti-γ-tubulin (T5326, 1:5000, Sigma-Aldrich), anti-β-catenin (#9562, CST), anti-GSK3 beta antibody [3D10] (ab93926, 1:1000, Abcam, Cambridge, UK), anti-SMC1A (A700-018, 1:1000, Fortis Life Sciences, Waltham, MA, USA), anti-NIPBL (A301-779A, 1:1000, Fortis Life Sciences, Waltham, MA, USA), anti-RAD21 (A300-080A, 1:1000, Fortis Life Sciences), and anti-SMC3 (A300-060A, 1:1000, Fortis Life Sciences, Waltham, MA, USA).

### 2.7. Chromatin Immunoprecipitation (ChIP)

To investigate the localization of β-catenin, NIPBL, RAD21, SMC1A, and SMC3 at the *MYC* locus, chromatin immunoprecipitation (ChIP) assays were conducted following previously established protocols with minor adjustments [34,35]. Initially, cells were crosslinked with 1% formaldehyde for 15 min, and the crosslinking reaction was quenched with the addition of 125 mM glycine. The crosslinked cells were then lysed successively with lysis buffer 1 (50 mM HEPES-KOH, pH 7.5, 140 mM NaCl, 1 mM EDTA, 10% glycerol, 0.5% NP-40, and 0.25% Triton X-100) and lysis buffer 2 (10 mM Tris-HCl, 100 mM NaCl, and 1 mM EDTA), and sonicated in lysis buffer 3 (10 mM Tris-HCl, 100 mM NaCl, 1 mM EDTA, 0.1% sodium deoxycholate, and 0.5% sarkosyl) to obtain chromatin fragments ranging from 500 to 200 base pairs. The sheared chromatin samples were then immunoprecipitated using Dynabeads protein A (Invitrogen) pre-bound with 10 μg of antibodies against β-catenin, NIPBL, RAD21, SMC1A, and SMC3. Mouse IgG (Sigma) served as a negative control. Following immunoprecipitation, the beads were washed successively with low-salt buffer (20 mM tris–HCl, pH 8, 150 mM NaCl, 0.5 mM EDTA, 0.1% SDS, 1% Triton X-100), high-salt buffer (20 mM tris–HCl, pH 8, 500 mM NaCl, 0.5 mM EDTA, 0.1% SDS, 1% Triton X-100), and RIPA buffer (50 mM HEPES-KOH, pH 7.5, 0.5 mM EDTA, 10% NP-40, 0.7% sodium deoxycolate and 0.5 M LiCl) to remove non-specifically bound proteins. The immunoprecipitated chromatin was eluted from the beads overnight at 65 °C and subsequently treated with proteinase K. DNA was then extracted using phenol-chloroform extraction, followed by purification with the QIAquick Purification Kit (Qiagen, Hilden, Germany). For each cell line, three independent ChIP assays were performed and analyzed via quantitative PCR (qPCR). Each sample was run in duplicate and repeated three times to ensure reproducibility. Specific primers targeting either the promoter or the first exon of *MYC* gene were utilized for qPCR analysis (Supplementary Table S1).

The results were expressed using the percent input method, where signals obtained from the ChIP were normalized to signals obtained from an input sample, representing the amount of chromatin used in the ChIP.

### 2.8. RNA-Sequencing (RNA-seq)

For RNA-seq analysis, two samples from each cell line, both before and after LY2090314 treatment, underwent individual processing as previously described [35,36]. Library preparation was performed using the Universal Plus mRNA-Seq kit (Tecan Genomics, Redwood City, CA, USA) according to the manufacturer’s instructions (library type: fr-secondstrand). RNA samples were quantified and subjected to quality assessment using either the Agilent 2100 Bioanalyzer RNA assay (Agilent Technologies, Santa Clara, CA, USA) or the Caliper LabChip GX (PerkinElmer, Waltham, MA, USA). Final libraries were evaluated for quality using both the Qubit 2.0 Fluorometer (Thermo Fisher Scientific, Waltham, MA, USA) and either the Agilent Bioanalyzer DNA assay or the Caliper LabChip GX (PerkinElmer, Waltham, MA, USA). Subsequently, the libraries were prepared for sequencing and sequenced in single-end 75 bp mode on the NextSeq 500 (Illumina, San Diego, CA, USA)/paired-end 150 bp mode on the NovaSeq 6000 (Illumina, San Diego, CA, USA). Raw sequencing data underwent initial processing using the Illumina BCL Convert v3.9.31 pipeline for format conversion and de-multiplexing. The reads were then aligned to the reference hg38-iGenomes genome using STAR, a splice junction mapper for RNA-seq reads [37]. This alignment method facilitated the identification of splice junctions between exons. Preprocessing of RNA-seq data for differential expression analysis was performed using Htseq-count to count the overlap of reads with genes. DESeq2 [38,39] was subsequently employed to compare expression levels of genes and transcripts by fitting a generalized linear model (GLM) for each gene. Normalization was conducted using the median-of-ratios method [38], and statistical significance was determined using a Wald test.

Only protein-coding genes were considered for analysis, and gene-level expression values were determined using fragments per kilobase million (FPKM) mapped. Genes with FPKM > 1 were considered expressed and analyzed, with a false discovery rate (FDR) threshold of <0.001 for statistical significance.

### 2.9. Pathway Analysis and Function

To gain insights into the biological significance of the differentially expressed genes, functional analysis was conducted using the Database for Annotation, Visualization, and Integrated Discovery (DAVID) v2023q2 [40] (URL “https://david.ncifcrf.gov” accessed on 09 October 2023). This online tool enables the annotation and visualization of gene lists, providing valuable information on enriched biological processes. For each term identified by DAVID, a *p*-value was calculated, and terms with a significance level of *p* < 0.05 were considered enriched.

### 2.10. cDNA Synthesis and Quantitative Real-Time PCR (qPCR)

Total RNA extraction was carried out using the RNAeasy Mini-kit (Qiagen), following the manufacturer’s protocol. Subsequently, cDNA synthesis was performed using SuperScript™ II reverse transcriptase in conjunction with oligo-dT primers (Invitrogen). PCR analyses were conducted using the Rotor Gene 3000 (Corbett, Sydney, Australia). qPCR reactions were run in triplicate to ensure reproducibility and reliability of the results. To normalize the qPCR data, expression levels were referenced to the housekeeping gene HPRT. Specific primer pairs utilized for the validation of RNA-seq data and *MYC* expression are listed in Supplementary Table S2.

### 2.11. Statistical Analyses

Statistical analyses were conducted using the IBM SPSS statistical package (version 28.0) for Windows. Results were reported as mean ± standard deviation (SD). Differences between continuous variables were assessed using Student’s *t*-test. A *p*-value < 0.05 was considered statistically significant.

## 3. Results

### 3.1. Identification of Mutated Cohesin Genes in Cancer Cell Lines

The list of cell lines in the NCI-60 panel and their tissue origins are given in Supplementary Table S3. The panel consists of fifty-five cell lines: two from the prostate, four from blood, six each from the colorectal system, breast, ovary, and central nervous system, eight each from the kidney and the lung and nine from melanoma. The identification of mutations in cohesin genes was performed via WES. Although a limitation of cell line sequencing is the lack of available normal-matched tissue for comparison, the NCI-60 panel does allow comparisons between cell lines from nine distinct tissues of origin. We detected fifteen mutations in nine cell lines each carrying at least one cohesin mutation. In particular, one was derived from each of lung, ovary, blood, and central nervous system, two from kidney, and three from colon cancer (Table 1). Identified variants were mapped on both core cohesin and regulatory cohesin genes, involving *ESCO1*, *ESPL1*, *NIPBL*, *PDS5B*, *RAD21*, *SMC1A*, *SMC3*, *STAG2*, and *WAPL* genes. Most mutations (80%, 12 out of 15) were nonsense or frameshift leading to premature stop codons. The remaining three mutations were missense, the first two (c.3288G > T, c.5038A > G) affecting *ESPL1* and the last (c.2028G > T) involving *SMC1A* (Table 1). Overall, WES analysis showed that 16% (9 out of 55) of cell lines belonging to the NCI-60 panel carry at least one mutation in cohesin or regulatory cohesin genes.

### 3.2. Effect of LY2090314 Treatment on Cancer Cell Lines Carrying Mutations in Cohesin Genes

Since the availability of cancer cell lines harboring mutations in cohesin genes is very rare, this finding allowed us to investigate whether all identified cohesin mutations were synthetically lethal with stimulation of WNT signaling and whether targeting the WNT pathway may prove to be a novel therapeutic strategy for cohesin-mutant cancers. To this aim, all cohesin-mutated cancer cells were treated with LY2090314 (100 nM for 24 h), a GSK3 inhibitor and stimulator of the WNT signaling pathway. The activation of WNT signaling via ligand binding or GSK3 inhibition releases β-catenin, thus allowing it to accumulate in the nucleus and activate transcription of WNT target gene/s [41,42].

**Figure 1:**
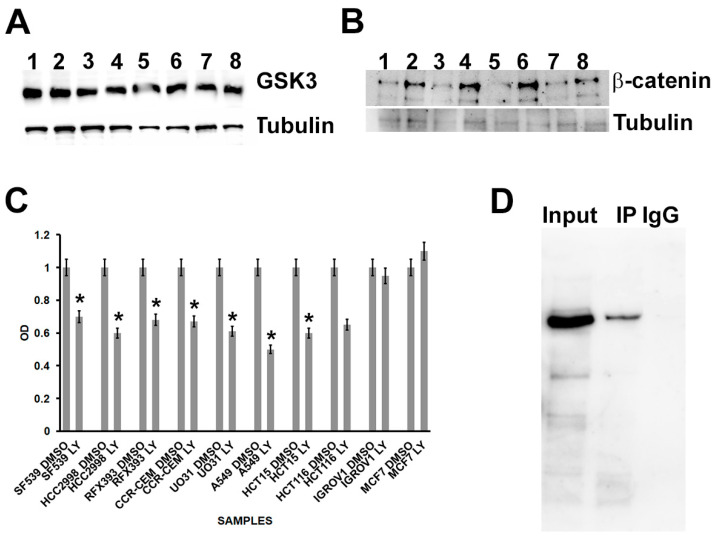
Effects of LY2090314 (LY) treatment in cancer cell lines carrying mutations in cohesin genes. (**A**) Western blotting showing that GSK3 levels did not change in MCF7 (1, untreated; 2, plus LY); HCT116 (3, untreated; 4, plus LY2090314); HCT15 (5, untreated; 6, plus LY), and IGROV1 (7, untreated; 8, plus LY4) cell lines. (**B**) LY2090314 treatment caused an increase in the expression of β-catenin in MCF7 (1, untreated; 2, plus LY); HCT116 (3, untreated; 4, plus LY); HCT15 (5, untreated; 6, plus LY), and IGROV1 (7, untreated; 8, plus LY) cell lines. (**C**) MTT assay shows that LY2090314 treatment caused the reduction of viability in all cell lines except for MCF7, the control cell lines without mutation in cohesin genes, and IGROV1 carrying mutation in the *ESPL1* gene. (**D**) β-catenin was found to be co-precipitated with SMC1A, whereas no SMC1A signal was detected in the IPs using IgG-coated beads. * *p* < 0.05.

After treatment, we analyzed GSK3 and β-catenin protein levels via Western blotting. We found that there was no difference in GSK3 levels between cohesin-mutated and control cells after treatment of cells with LY2090314 (Figure 1A and Figure S1A). This was somewhat expected since, upon administration, LY2090314 binds to and inhibits GSK-3 in an ATP-competitive manner but treatment has no effect on GSK3 protein level, as previously shown [43]. However, this result contrasts with what has been previously described [31]. It is possible that this discrepancy arises from the combination of several factors. In fact, in our work, the treatment with LY2090314 was applied for 24 h and the total proteins were subsequently extracted, while in the previous work, the treatment lasted 6 h and then the proteins were extracted from the cytoplasmic or membrane fraction [31].

Instead, LY2090314 treatment markedly increased β-catenin in all mutated cells compared with controls (Figure 1B and Figure S1B). Next, we investigated the cell viability after WNT stimulation by using MTT assay. Interestingly, we found that LY2090314 inhibited the growth of all cohesin-mutated cell lines apart from the MCF7 control cell line and IGROV1 carrying an *ESPL1* mutation. It is interesting to note that cells carrying multiple cohesin mutations exhibited reduced viability compared with those with a single mutation. For instance, the A549 cell line, which harbors mutations in both *WAPL* and *NIPBL* genes, showed diminished viability compared with the CCRFCEM cell line, which only has a mutation in *NIPBL*, highlighting the contribution of *WAPL* to synthetic lethality. In addition, the MTT assay allowed discrimination of the role of the *SMC1A* mutation. Indeed, the IGROV1 cell line, which carries the mutation in *ESPL1*, showed no differences in viability compared with the control line. In contrast, the HCT15 cell line, which harbors mutations in both *ESPL1* and *SMC1A*, exhibited growth delay after GSK3 inhibitor treatment (Figure 1C). In order to investigate a possible interaction between cohesin and β-catenin, we performed co-IP between cohesin members and β-catenin. We found that NIPBL, RAD21, SMC1A, and SMC3 were detected in IP–β-catenin precipitates, while no signal was detected in the control Western blotting using IgG-coated beads (Figure 1D and Figure S2).

These results suggest that β-catenin is stabilized following WNT activation when cohesin activity is perturbed. Furthermore, for the first time, we have demonstrated that β-catenin interacts with cohesin factors.

### 3.3. The Interplay between β-Catenin, Cohesin and MYC

It is well known that the accumulation of β-catenin promotes the transcription of many oncogenes such as *MYC* [44]. In addition, previous studies have shown that *MYC* transcription is positively regulated by cohesin in *Drosophila*, zebrafish, mouse, and human cells [45,46,47,48]. Therefore, we analyzed the expression of *MYC* in cohesin-mutated cancer cells after LY2090314 treatment.

**Figure 2:**
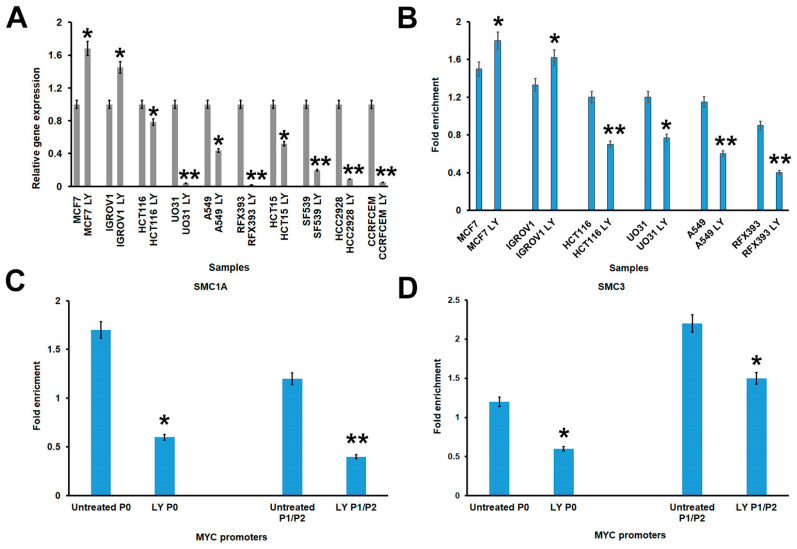
Effects of LY2090314 (LY) treatment on *MYC* locus. (**A**) *MYC* was downregulated in all cell lines carrying mutation in cohesin genes, apart from IGROV1 harboring *ESPL1* mutation and MCF7, the control cell line. (**B**) LY treatment caused decreased binding of β-catenin at P1/P2 promoters of *MYC* locus in all cell lines carrying a single cohesin mutation, again except for IGROV1 and MCF7 cell lines. (**C**,**D**) ChIP experiments with antibodies against SMC1A or SMC3 showed decreased cohesin binding at both P0 and P1/2 promoters of the *MYC* gene following LY treatment in UO31 and HCT116, respectively. ** *p* < 0.01, * *p* < 0.05.

We found that treatment caused a strong decrease in *MYC* expression level in all cell lines, except for IGROV1 and MCF7 cells, which on the contrary displayed a significant increase. In particular, SF539, HCC2998, RFX393, CCRFCEM, and UO31 showed a decrease of < 80%, A549 and HCT15 almost 50%, while IGROV1 and MCF7 displayed increases of 45% and 68%, respectively (Figure 2A).

WNT signalling was previously shown to induce enhancer-promoter looping at the *MYC* gene in cancer cells [49]. Therefore, we investigated both cohesin and β-catenin binding at *MYC* promoters via ChIP-qPCR analysis. In order to investigate the effect of gene mutation on the binding of β-catenin, we focused on cell lines harboring a single cohesin mutation. The *MYC* locus contains three exons and four distinct promoters. P0 transcripts begin at many initiation sites, while both P1 and P2 are placed at the beginning of the first exon. Finally, P3 maps between the first and second exon. Exon 1, a large noncoding exon, contains an enhancer element and a region that oversees the elongation of RNA transcripts [50,51]. We found that β-catenin did not bind to P0 promoter while its recruitment at the first exon showed a significant decrease in cell lines with a single cohesin mutation. In contrast, β-catenin binding displayed a significant increase in MCF7 and IGROV1 cell lines (Figure 2B). Next, we also investigated the recruitment of core cohesin members and regulatory factors at *MYC* promoters. ChIP data showed that binding of NIPBL, RAD21, SMC1A, and SMC3 decreased significantly at both P0 and P1/P2 promoters in CCRFCEM, RFX393, UO31, and HCT116 cell lines, respectively, whereas ESPL1 showed a significant increase in the IGROV1 cell line (*p* < 0.05) (Figure 2C,D and Figure S3).

All together, these results suggest that LY2090314 treatment leads to decreased *MYC* expression, and this probably depends on the reduced binding of both cohesin and β-catenin at the first exon of *MYC*.

### 3.4. LY2090314 Treatment Causes Gene Expression Dysregulation

In order to investigate the effect of LY2090314 treatment on gene expression against a cohesin-mutated background, we performed RNA-seq analysis. Treatment caused the dysregulated expression of many genes ranging from 6162 in RFX393 to 3253 in HCT116 cell lines (FDR < 0.001, Table 2).

**Figure 3:**
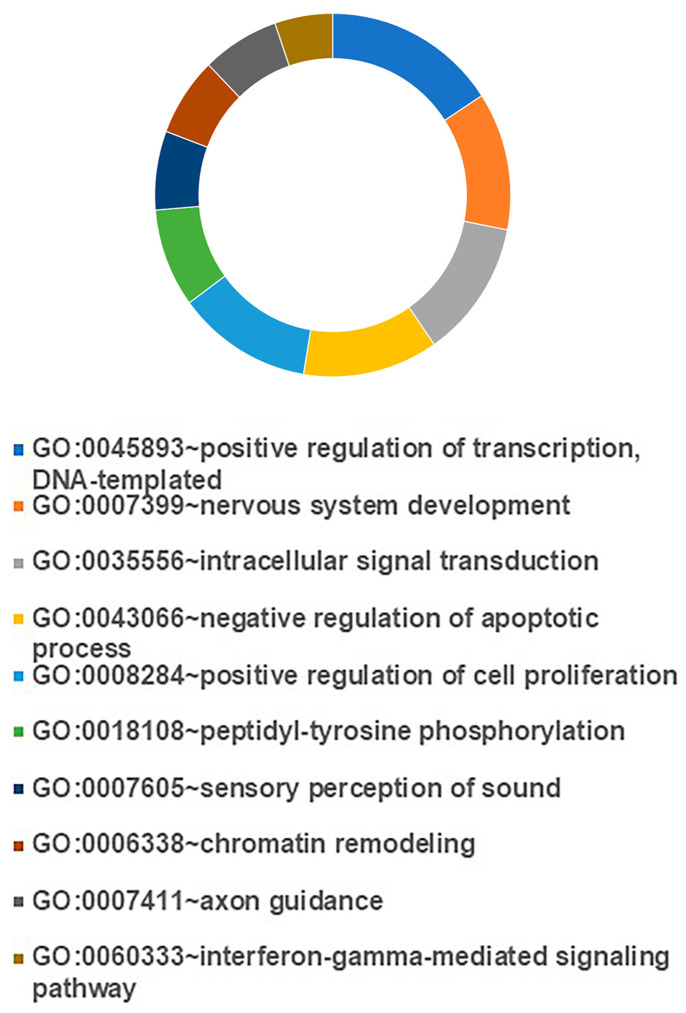
Effects of LY2090314 treatment on gene expression. GO term enrichment analysis of biological process that were significantly over-represented when considering differentially expressed genes following LY2090314 treatment.

In the RFX393, SF539, and UO31 cell lines carrying mutations in the core cohesin genes, *RAD21*, SMC1A, and *STAG2* showed the highest number of dysregulated genes. The complete list of dysregulated genes is reported in DataSet. Cell lines shared 133 dysregulated genes (Supplementary Table S4). The classification of dysregulated genes according to molecular function and biological process via the DAVID tool (URL “https://david.ncifcrf.gov” accessed on 09 October 2023) showed that identified pathways are related to positive regulation of transcription (GO:0045893), positive regulation of cell proliferation (GO:0008284), chromatin remodeling (GO:0006338), and intracellular signal transduction (GO:0035556). The first ten pathways for a number of genes are shown in Figure 3, while the remaining pathways are listed in Supplementary Table S5.

**Figure 4:**
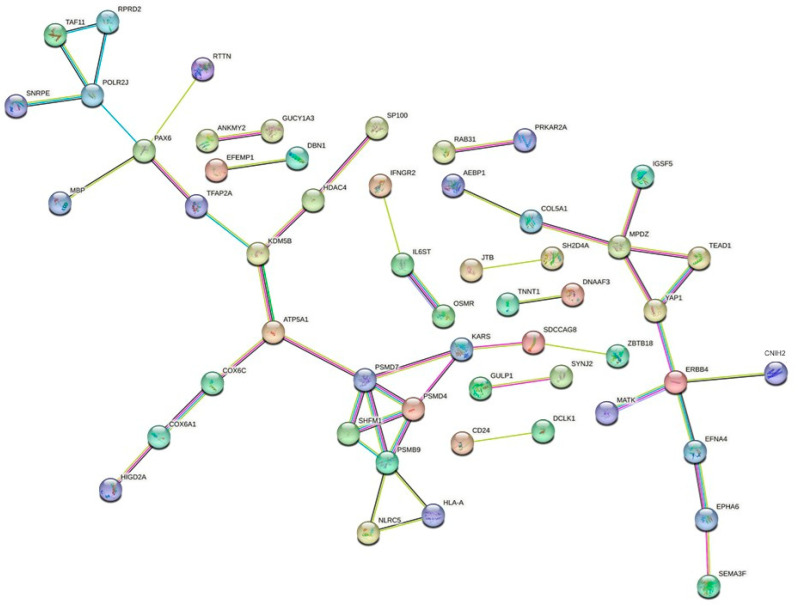
Effects of LY2090314 treatment on gene expression. STRING network analysis of the 133 dysregulated proteins. They were grouped in two different clusters.

To find a comprehensive relationship between the 133 dysregulated proteins, the STRING network analysis tool (URL “https://string-db.org” accessed on 10 October 2023) was applied. The protein interactions were filtered, so that unconnected root nodes and indirect interactions were discarded. This analysis predicts that the dysregulated proteins are related to two clusters, centered on KDM5B, ATP5A1, and YAP1 and ERBB4, respectively, whose members are involved in proteosome and cancer progression (Figure 3. The heat map related to the core of the two clusters (see Figure 4) showed that 97% of genes maintained the same trend, though to a different extent (Supplementary Figure S4, Supplementary Table S6). RNA-seq data were validated in five genes, namely *PSMD7*, *PSMD4*, *PSMB9*, *SFHM1*, and *PAX6*, via quantitative RT-PCR experiments (Supplementary Figure S5). These genes were chosen because most of them are involved in cancer development [52,53,54,55] and their differential expression could explain the decreased viability after LY2090314 treatment.

Collectively, following treatment with LY2090314, the cell lines shared 133 dysregulated genes involved in fundamental biological processes whose changes in gene expression could underlie synthetic lethality in cells with mutated cohesin genes.

## 4. Discussion

Advances in cancer genomics have allowed the identification of potential therapeutic targets in relation to genomic defects that were formerly disregarded due to their non-driver mutation classification [56,57]. The cohesin complex is frequently mutated in cancer and it is thought that cohesin dysregulation plays a significant role in cancer development and progression [26]. These findings have attracted increased interest supporting the idea of cohesin as a possible therapeutic target [17,58]. Recently, we showed that cohesin subunits SMC3, RAD21, and STAG2 are more responsive to treatment with LY2090314, a GSK3 inhibitor and an agonist of WNT signaling [31]. This phenomenon is called synthetic lethality, where the occurrence of a single genetic event is tolerable for cell survival, whereas the co-occurrence of a second event results in cell death. To date, only a limited number of cohesin gene mutations have been tested for synthetic lethality and the mechanism underlying synthetic lethality is still under debate.

Since the availability of cell lines carrying cohesin mutations is limited, we first performed WES for an NCI-60 cancer panel. We identified at least one cohesin mutation in nine cell lines derived from colon, kidney, lung, ovary, blood, and central nervous system cancers. Core cohesin subunits and regulatory factors were both represented. In fact, we detected missense, frameshift, or nonsense mutations in *ESCO1*, *ESPL1*, *NIPBL*, *PDS5B*, *RAD21*, *SMC1A*, *SMC3*, *STAG2*, and *WAPL* genes.

Next, these cell lines were treated with LY2090314 compound to test its effect on cell viability in cells carrying cohesin mutation deriving from different cancer contexts. We found no difference in GSK3 levels between MCF7 cells, a control cell line without cohesin mutations, and mutant cohesin cells. In contrast, we found that β-catenin was stabilized in all mutant cohesin cells following LY2090314 treatment. It is worth noting that HCT116 and HCT15 cell lines are constitutively active for WNT signaling. In fact, HCT116 cells carry a three-base deletion in the β-catenin encoding gene causing an in-frame deletion at Ser45 while HCT15 harbors a truncated mutation in the *APC* gene [59,60,61,62]. The migration of β-catenin into the nucleus represents a pivotal event in the canonical WNT pathway [63,64]. This process hinges on the destabilization of the β-catenin destruction complex, a crucial assembly comprising CK1, GSK3, Axin, and APC, culminating in the potentiation of WNT signaling [65,66,67,68]. These findings indicate that mutations in *APC* or *β-catenin* thwart the destruction complex’s ability to bind to β-catenin, leading to aberrant accumulation of β-catenin within the cell. In addition, our results suggest that β-catenin normally targeted for degradation is stabilized in cohesin-deficient cells and is available in the active form following inhibition of GSK3, which in turn enhances WNT signaling. The WNT pathway and β-catenin are involved in establishing the body axis during embryonic growth [69,70,71]. In adults, they contribute to tissue stem cell maintenance, homeostasis, apoptosis, and cell renewal, proliferation, and repair [72,73]. The aberrant expression of WNT signaling/β-catenin has been widely considered to play a pivotal role in tumorigenesis, cancer progression, and metastasis (reviewed in [74]). Furthermore, we found that all mutated cells showed growth delay after treatment, with the only exception being a cell line harboring *ESPL1* mutation. This result could reflect the different roles of cohesin proteins under investigation. RAD21, SMC1A, SMC3 belong to the core cohesin complex, while NIPBL and WAPL have an antagonist role. NIPBL is involved in the loading of cohesin onto chromosomes [75]. Once loaded, cohesin can translocate along the chromosome or be removed by the cohesin-releasing factor WAPL [76,77,78]. Instead, ESPL1, although essential for sister chromatid cohesion dissolution, is not a member of the cohesin complex [79]. Therefore, unlike other proteins, ESPL1 intervenes only in the final phase of the cohesin life cycle and, thus, its mutations have no effect on sensitivity to treatment with LY2090314.

It is well known that the accumulation of β-catenin promotes the transcription of many oncogenes, such as *MYC* [44,80]. *MYC* is a transcription factor that promotes the rapid down- and upregulation of about 15% of human genes and, as consequence, it is thought to coordinate the plethora of transcriptional changes that foster cell proliferation [81,82].

**Figure 5:**
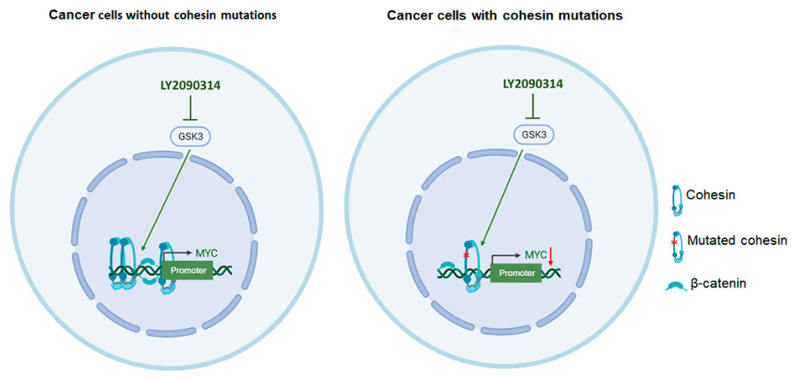
Model for the synthetic lethal interaction between LY2090314 treatment and mutated cohesin. In cancer cells with and without cohesin gene mutations, LY2090314 inhibits GSK3, which acts as an agonist of the WNT signaling pathway. In cells without cohesin mutation, this leads to an increase in both cohesin and β-catenin binding at *MYC* promoters, stimulating cell growth. Instead, in the presence of mutated cohesin, the recruitment of both β-catenin and cohesin at the *MYC* locus is decreased and *MYC* expression is downregulated, leading to decreased viability.

Here, we found that activation of the WNT pathway caused a considerable decrease in both cell viability and *MYC* expression level in all cell lines, apart from that carrying *ESPL1* mutation, as previously stated. This data indicates that LY2090314 exerts its effects through the downregulation of *MYC*, due to β-catenin stabilization and enhancement of WNT signaling, consequently affecting cell growth (Figure 5). This study suggests that WNT enhancement may be effective for the treatment of tumors containing cohesin mutations.

Based on the results presented in this study, we show that SMC1A, RAD21, and SMC3 bind all promoter regions of *MYC*. This result indicates that *MYC* is positively regulated by cohesin, further supporting this notion as previously observed in *Drosophila*, zebrafish, and mouse cells [45,48]. The recruitment of cohesin on *MYC* promoter regions showed a significant decrease following LY2090314 treatment. Interestingly, we found that β-catenin recruitment also decreases at the *MYC* locus and β-catenin interacts with cohesin complex. This suggests that the regulation of *MYC* expression is finely regulated, involving both cohesin and β-catenin. As an effect of *MYC* dysregulation, we found the alteration of the expression of hundreds of genes. Cell lines carrying mutations in *RAD21*, *SMC1A*, and *STAG2* displayed the highest numbers of dysregulated genes. This observation further confirms the role of cohesin in regulating gene expression, suggesting that altered cohesin activity leads to aberrant transcription [83]. We hypothesize that this points to changes in gene expression as a cancer-promoting mechanism. This notion is supported by the finding that *RAD21*, *SMC1A*, and *STAG2* have been thought to play a pivotal role in tumorigenesis [84,85,86]. All cell lines carrying cohesin mutations shared 133 genes. These genes were involved in several biological processes, such as regulation of transcription, intracellular signal transduction, negative regulation of apoptotic process, positive regulation of cell proliferation, and chromatin remodeling. In addition, STRING analysis showed that dysregulated proteins belong to two clusters, centered on KDM5B, ATP5A1 and YAP1 and ERBB4. which are involved in proteosomes and tumorigenesis [87,88,89,90,91,92]. The altered expression of these proteins could underlie the synthetic lethal phenomenon exerted by LY2090314 treatment in a genetic environment with mutated cohesin.

## 5. Conclusions

In summary, we identified the molecular mechanism underlying synthetic lethality due to cohesin mutations. Moreover, synthetic lethality offers a promising approach for developing valuable therapeutic interventions in cancer when direct targeting of driver genes is impractical. Thus, our findings indicate that targeting the WNT pathway may be effective in treating cancers that harbor cohesin mutations.

## Figures and Tables

**Table 1:** Cohesin gene mutations identified in the NCI-60 cancer cell line panel.

Cell Line	Tissue	Gene	DNA Mutation	Protein Mutation
HCT116	Colon	*SMC3*	c.2425C > T	p.Gln809 *
HCT15	Colon	*ESPL1*	c.3288G > T	p.Glu1096Asp
		*SMC1A*	c.2028G > T	p.Glu676Asp
A549	Lung	*NIPBL*	c.7201G > T	p.Gly2401 *
		*WAPL*	c.916C > T	p.Gln306 *
CCRFCEM	Blood	*NIPBL*	c.8212C > T	p.Gln2738 *
SF539	Central nervous system	*STAG2*	c.775C > T	p.Arg259 *
RFX393	Kidney	*RAD21L1*	c.31C > T	p.Arg11 *
UO31	Kidney	*SMC1A*	c.855_856ins	p.Glu286fs
IGROV1	Ovary	*ESPL1*	c.5038A > G	p.Arg1680Gly
HCC2998	Colon	*SMC3*	c.2830C > T	p.Arg944 *
		*ESCO1*	c.1369G > T	p.Glu457 *
		*ESCO1*	c.925G > T	p.Glu309 *
		*STAG2*	c.1147G > T	p.Glu383 *
		*PDS5B*	c.2740G > T	p.Glu914 *

* premature stop codon.

**Table 2:** Numbers of dysregulated genes in cancer cells carrying mutations in cohesin genes.

Cell Line	Dysregulated Genes	Upregulated Genes	Downregulated Genes
A549	4808	2240	2568
CCRFCEM	5403	3124	2279
HCC2998	4242	2177	2065
HCT15	3693	2000	1693
HCT116	3253	1604	1649
IGROV1	2747	1333	1414
RFX393	6162	2826	3336
SF539	5855	2921	2934
UO31	5936	2631	3305

## Data Availability

The relevant data supporting the findings of this study are available in this article and its Supplementary Information files. All NGS raw files have been deposited in the NCBI Sequence Read Archive under accession number PRJNA1019503.

## Supplementary Materials

The following supporting information can be downloaded at: https://www.mdpi.com/article/10.3390/cells13070608/s1, Table S1: Primers used to investigate cohesin loading at the *MYC* locus. Table S2: Primers used to validate RNA-seq data and *MYC* expression by RT-PCR. Table S3: NCI-60 cancer panel cell used in this work. Table S4: Dysregulated genes in common among cohesion-mutated cancer cells. Table S5: Classification of dysregulated genes by molecular function and biological process through the DAVID tool. Table S6: Fold change of the core genes as identified through STRING analysis. Figure S1: Effects of LY2090314 (LY) treatment in cancer cell lines carrying mutation in cohesin genes. Figure S2: β-catenin and cohesin interaction. Figure S3. Effects of LY2090314 (LY) treatment on the *MYC* locus. Figure S4: Heatmap of dysregulated genes which were held in common among cancer cell lines carrying mutation in cohesin genes. Figure S5: RNA-seq data was validated by RT-PCR.
