# Supplementary material for: Synthetic Lethality between Cohesin and WNT Signaling Pathways in Diverse Cancer Contexts
Source: Cells. 2024 Mar 30;13(7):608. doi: 10.3390/cells13070608 (PMC11011321; doi:10.3390/cells13070608)
Supplement: Supplementary file 1 [file cells-13-00608-s001.zip › cells-2914227-supplementary.pdf]

Table S1. Primers used to investigate the cohesin loading at *MYC* locus.

MYC promoter	Forward	Reverse
P0	TGCCAGTAGAGGGCACACTT	CCAATCGCTATGCTGGATTT
P1/P2	GTATAAAAGCCGGTTTTCGGG	CTCTGCCTCTCGCTGGAATTA

Table S2. Primers used to validate RNA-seq data and *MYC* expression by RT-PCR.

Gene	Nucleotide sequence
PSMD7 F	TCGAACAGTTTTGCAGTTCCT
PSMD7 R	CTGTGTGGTACCAGCCAACT
PSMD4 F	CCTTGGCCCTTCGTGTATCT
PSMD4 R	GGCATCGTCTGAGTCTCTTTCA
PSMB9 F	CGCTTCACCACAGACGCTAT
PSMB9 R	TGCCCAAGATGACTCGATGG
SFHM1 F	AGTAGGAGAGCCCTGTGGAG
SFHM1 R	TTTGTTACGCAAACGCCAGG
PAX6 F	AGTGAATCAGCTCGGTGGTG
PAX6 R	AATTCGGGAAATGTCGCACG
HPRT F	AGCCAGACTTTGTTGGATTTG
HPRT R	TACTAAGCAGATGGCCACAGA
MYC F	TTCGGGTAGTGGAAAACCAG
MYC R	TAGAAATACGGCTGCACCGA

Table S3. NCI-60 cancer panel cell used in this work.

Cell line	Cancer
786-0	renal adenocarcinoma
A498	renal carcinoma
A549	lung adenocarcinoma
ACHN	renal carcinoma
BT549	breast
CAKI1	renal adenocarcinoma
CCRFCEM	prioheral blood acute lymphoblastic leukemia
COLO225	colon adenocarcinoma
DU145	prostate carcinoma
EKVX	lung adenocarcinoma
HCC2998	colon adenocarcinoma
HCT116	colon carcinoma
HCT15	colon adenocarcinoma
HOP62	lung adenocarcinoma
HOP92	lung carcinoma
HS587T	Breast carcinoma
HT29	recto-sigmoid colon adenocarcinoma
IGROV1	left ovary cysto adenocarcinoma
K562	pleural effusion crhonic myelogeous leukemia
LOXIHVI	lymphnode metastasis malignant amelanotic melanoma
M14	amelanotic melanoma
MALME 3M	malignant melanoma
MCF7	breast adenocarcinoma
MDA 231	breast
MDA 468	breast
MDA435	pleural effusion melanoma
NCI ADR RES	ovarian adenocarcinoma
NCI H322	lung bronchi alveolar carcinoma
NCI H522	Lung adenocarcinoma
NCI H226	lung squamous cell
NCI H23	lung adenocarcinoma
OVCAR3	ovarian carcinoma
OVCAR5	ovarian carcinoma
OVCAR8	ovarian carcinoma
PC3	prostate carcinoma
RFX393	renal hypernephroma
RPMI	peripheral blood multiple myeloma
SF268	plastic astocytoma
SF295	cns gliosarcoma
SF539	cns gliosarcoma
SK MEL 2	malignant melanoma
SKMEL28	malignant melanoma
SKMEL5	skin metastasis-thigh malignant melanoma
SKOV3	ovary adenocarcinoma
SNB 75	cns astrocytoma
SNB19	cns glioblastoma
SNC12C	renal carcinoma

SR	immunoblastic large cell lymphoma
SW60	colon adenocarcinoma
T47D	breast carcinoma
TK-10	renal carcinoma
U251	cns glioblastoma
UACC257	malignant melanoma
UACC62	malignant melanoma
UO31	renal carcinoma

Table S4. Dysregulated genes in common among cohesin mutated cancer cells.

AEBP1
AKR1C2
ALKBH4
ANKMY2
ANO6
ARHGDIG
ATP5A1
BOLA1
C12orf56
C7orf73
CAB39
CALML5
CBS
CCDC82
CD24
CNIH2
COL5A1
COX6A1
COX6C
CRYBG3
CTXN1
CYP4V2
DBN1
DCLK1
DDAH2
DGKG
DLGAP3
DNAAF3
EFEMP1
EFHD1
EFNA4
EFR3B
EHF
EPHA6
ERBB4
EVL
FAHD1
FAR2P1
FERMT1
FOXI3
FRAS1
FRG1CP
FUT9
GPR27
GUCY1A3
GULP1
HDAC4
HIGD2A
HLA-A

HMGB3
HSD17B12
IFNGR2
IFT22
IGSF5
IL6ST
JTB
KARS
KCNMA1
KDM5B
KIF13B
LAMTOR4
LGALS3
LINC00885
LINC01006
LRRK1
LYPD1
MAPKBP1
MATK
MBP
MDFIC
MIER1
MPDZ
MRPL41
MTIF3
MYLIP
MYO10
NLRC5
NPR2
OSMR
PAX6
PCDHB13
PCDHB4
PDZD4
PLA2G3
PMAIP1
POLR2J
PRKAR2A
PRR36
PRTFDC1
PSMB9
PSMD4
PSMD7
RAB31
RIMS4
ROGDI
RPRD2
RTTN
SDCCAG8
SEMA3F
SGK223

SH2D4A
SHFM1
SLC30A7
SNAR-E
SNRPE
SP100
SSFA2
STEAP1
SYNE4
SYNJ2
TAF11
TBC1D20
TCEAL8
TEAD1
TENM1
TFAP2A
TMEM183B
TMEM191A
TMEM254
TMEM51
TNNT1
TRIM47
TUB
UBASH3B
VPS72
YAP1
ZAK
ZBTB18
ZNF175
ZNF433
ZNF480
ZNF704
ZSCAN21

Table S5. Classification of dysregulated genes by molecular function and biological process through DAVID tool

**GO Term**	**Gene count**	**Pvalue**
GO:0018108~peptidyl-tyrosine phosphorylation	5	0,008175
GO:0060333~interferon-gamma-mediated signaling pathway	3	0,010238
GO:0006535~cysteine biosynthetic process from serine	2	0,011333
GO:0001958~endochondral ossification	3	0,013365
GO:0019343~cysteine biosynthetic process via cystathionine	2	0,016952
GO:0001974~blood vessel remodeling	3	0,020681
GO:0043418~homocysteine catabolic process	2	0,02254
GO:0010749~regulation of nitric oxide mediated signal transduction	2	0,02254
GO:0061026~cardiac muscle tissue regeneration	2	0,02254
GO:0070814~hydrogen sulfide biosynthetic process	2	0,02254
GO:0019448~L-cysteine catabolic process	2	0,02254
GO:0019344~cysteine biosynthetic process	2	0,02254
GO:0021587~cerebellum morphogenesis	2	0,028095
GO:0043506~regulation of JUN kinase activity	2	0,028095
GO:0045794~negative regulation of cell volume	2	0,028095
GO:0007399~nervous system development	7	0,030231
GO:0034097~response to cytokine	3	0,030461
GO:0019346~transsulfuration	2	0,03362
GO:0006565~L-serine catabolic process	2	0,03362
GO:1902041~regulation of extrinsic apoptotic signaling pathway via death domain receptors	2	0,03362
GO:0007173~epidermal growth factor receptor signaling pathway	3	0,034056
GO:0038165~oncostatin-M-mediated signaling pathway	2	0,039113
GO:0006563~L-serine metabolic process	2	0,039113
GO:0042262~DNA protection	2	0,039113
GO:0035556~intracellular signal transduction	7	0,043447
GO:0050667~homocysteine metabolic process	2	0,044575
GO:0060351~cartilage development involved in endochondral bone morphogenesis	2	0,050007
GO:0048861~leukemia inhibitory factor signaling pathway	2	0,050007
GO:0045893~positive regulation of transcription, DNA-templated	9	0,053463
GO:0051593~response to folic acid	2	0,055408
GO:0060576~intestinal epithelial cell development	2	0,055408

Table S6. Fold change of the core genes as identified through STRING analysis.

GENE	A549	CCRFCEM	HCC2998	HCT15	HCT116	RFX393	SF539	UO31
AEBP1	8,781881	-7,2421136	6,2582193	4,331768	8,651378	-6,29959	8,893221	4,609508
ATP5A1	1,506823	0,99769598	1,396185	1,041263	1,166753	1,798665	1,693419	1,159415
CNIH2	4,810471	2,14625663	5,2538755	5,33844	4,979673	4,565897	5,335135	2,813232
COL5A1	-3,72704	4,42277437	9,9132061	9,736137	7,73084	-7,59382	-7,111	-3,49548
COX6A1	0,948685	1,0035897	1,2598939	1,138598	0,997369	0,768972	1,132275	1,111442
COX6C	2,085412	1,2770899	2,0955803	2,600954	1,541953	0,806694	1,428309	2,051815
EFNA4	1,857483	2,74234841	1,1912141	2,145185	1,984843	3,915648	2,557189	2,092331
EPHA6	5,553663	10,0259143	10,404797	10,22366	9,674417	3,236467	9,912908	5,271792
ERBB4	9,965668	10,1899532	7,1704213	10,38949	6,180831	9,765947	10,07289	5,14034
HDAC4	-3,90296	-4,5422614	-3,669352	-2,71021	-2,94275	-2,55449	-3,82672	-2,40194
HIGD2A	1,026246	2,69673853	2,1332292	1,307718	1,469376	1,158269	1,321822	2,045639
HLA-A	-1,62468	-3,159552	-4,843231	-4,25773	-3,32619	-5,81819	-3,56923	-3,74
IGSF5	5,452931	9,11757733	9,4967212	5,850408	4,011027	7,310567	9,006076	2,828348
KARS	1,307147	1,6323852	1,1615521	1,567462	1,248558	0,769304	1,120598	0,787161
KDM5B	1,908905	1,92963578	2,9365567	3,661349	2,450383	2,710088	1,423219	1,92559
MATK	13,41421	8,50223566	14,015534	10,40087	13,28504	5,562463	8,909455	9,728849
MBP	-3,48624	8,70492606	-4,449248	-4,05842	-3,79233	-5,062	5,096394	-5,32066
MPDZ	-1,94657	10,9709312	11,350166	11,16784	10,6193	10,55379	-1,56829	-2,39327
NLRC5	-3,67689	-5,7224071	-7,794282	-6,3077	-4,13293	-7,25047	-6,05032	-5,62313
PAX6	-9,43533	-10,439776	-7,479587	-9,0772	-9,45952	-13,1605	-11,6155	-13,2967
POLR2J	1,637573	1,76048453	1,7592206	2,348731	1,465465	1,442296	1,723672	1,49771
PSMB9	-3,11822	-6,6563643	-3,653017	-3,76306	-3,17268	-7,91417	-5,25622	-6,61754
PSMD4	1,28967	1,55368227	1,1703388	1,629802	1,123902	1,340787	0,637512	1,415487
PSMD7	0,821862	1,18968592	1,0216243	1,180327	1,11296	0,998745	1,228027	0,640761
RPRD2	1,305857	0,97806286	1,1912296	1,147184	1,513213	1,950583	1,067892	1,59466
RTTN	-3,09737	-3,5924349	-3,27124	-2,68151	-3,32017	-2,69378	-2,46833	-2,6328
SDCCAG8	-1,79874	-1,3995031	-1,731946	-1,88981	-1,79565	-1,36481	-1,50522	-1,67822
SEMA3F	2,767987	7,62693093	1,5095825	2,783946	1,713493	2,451309	2,946458	3,012534
SHFM1	1,115161	1,26447387	1,0540022	1,603245	1,176263	1,251091	1,0144	0,649253
SNRPE	1,516899	1,11611427	1,6649296	1,852026	1,251715	1,375647	0,755952	1,672792
SP100	-3,66922	-4,3994037	-3,813757	-3,57286	-3,77314	-5,54397	-5,02581	-4,91081
TAF11	2,100474	1,2653927	1,2957028	1,782188	1,607644	1,536219	0,827676	2,220905
TEAD1	-1,54112	13,2429457	-1,310147	-1,05479	-1,44303	-1,10197	-1,06496	-2,01676
TFAP2A	1,235104	15,1973756	5,2677044	2,773967	2,022446	5,17402	3,16382	3,558483
YAP1	-1,61659	6,96097813	-2,260392	-1,85507	-2,18588	-0,99808	-1,20363	-2,48089
ZBTB18	-6,56115	-7,7809039	-5,966399	-6,22223	-4,17612	-5,33338	-7,20582	-8,01148

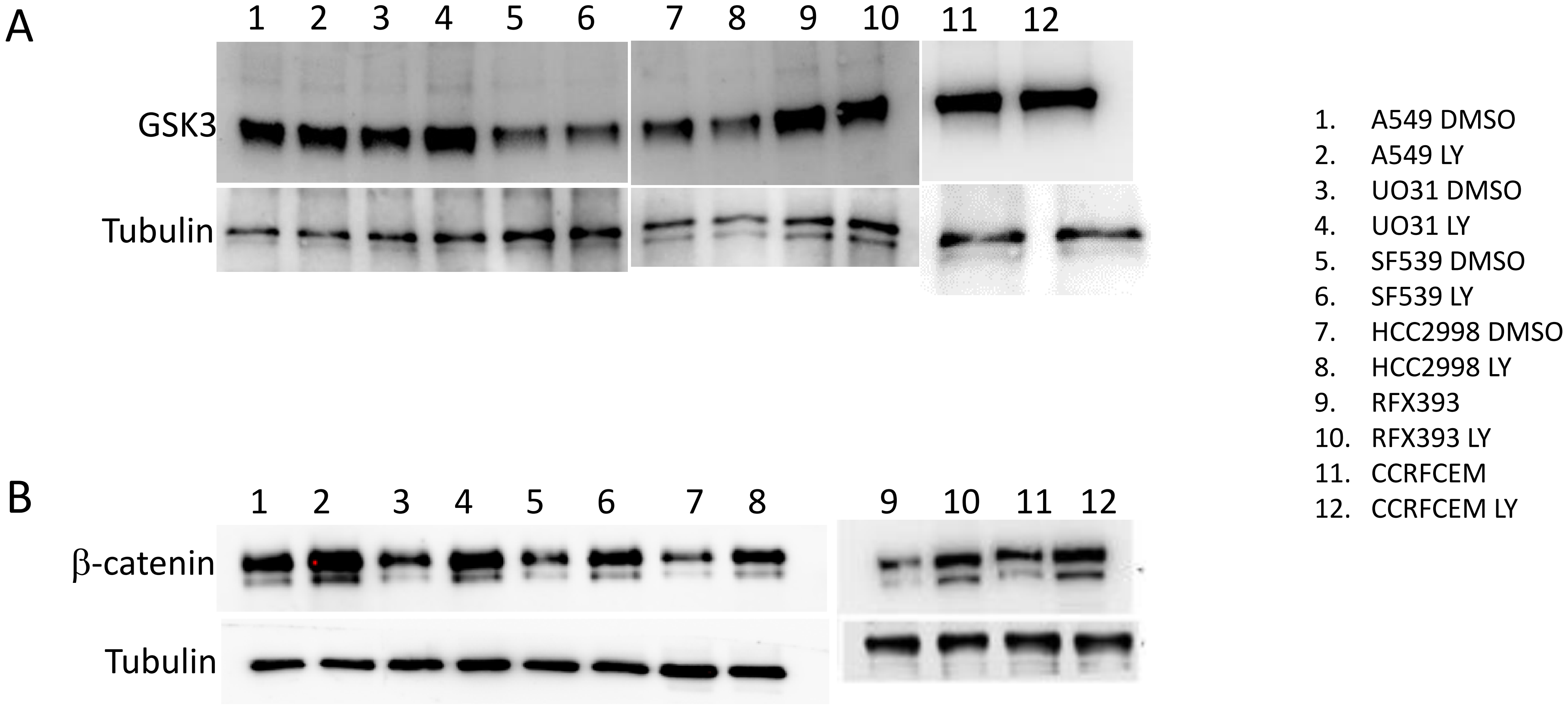

Figure S1. Effects of LY2090314 (LY) treatment in cancer cell lines carrying mutation in cohesin genes.

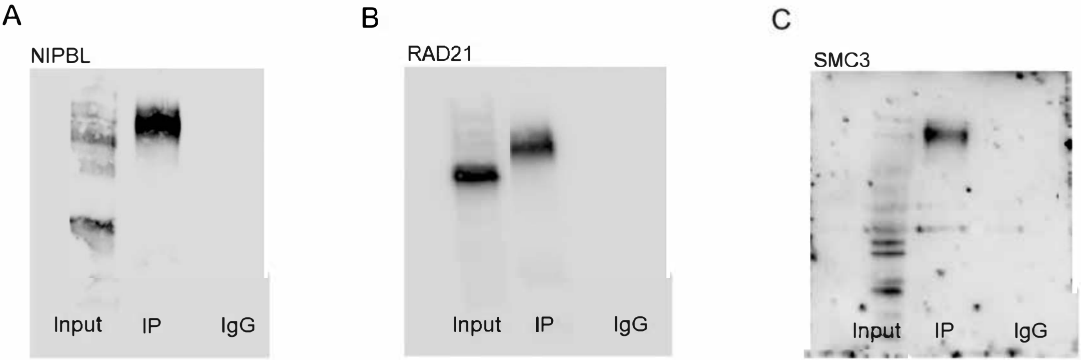

Figure S2. β–catenin and cohesin interaction.

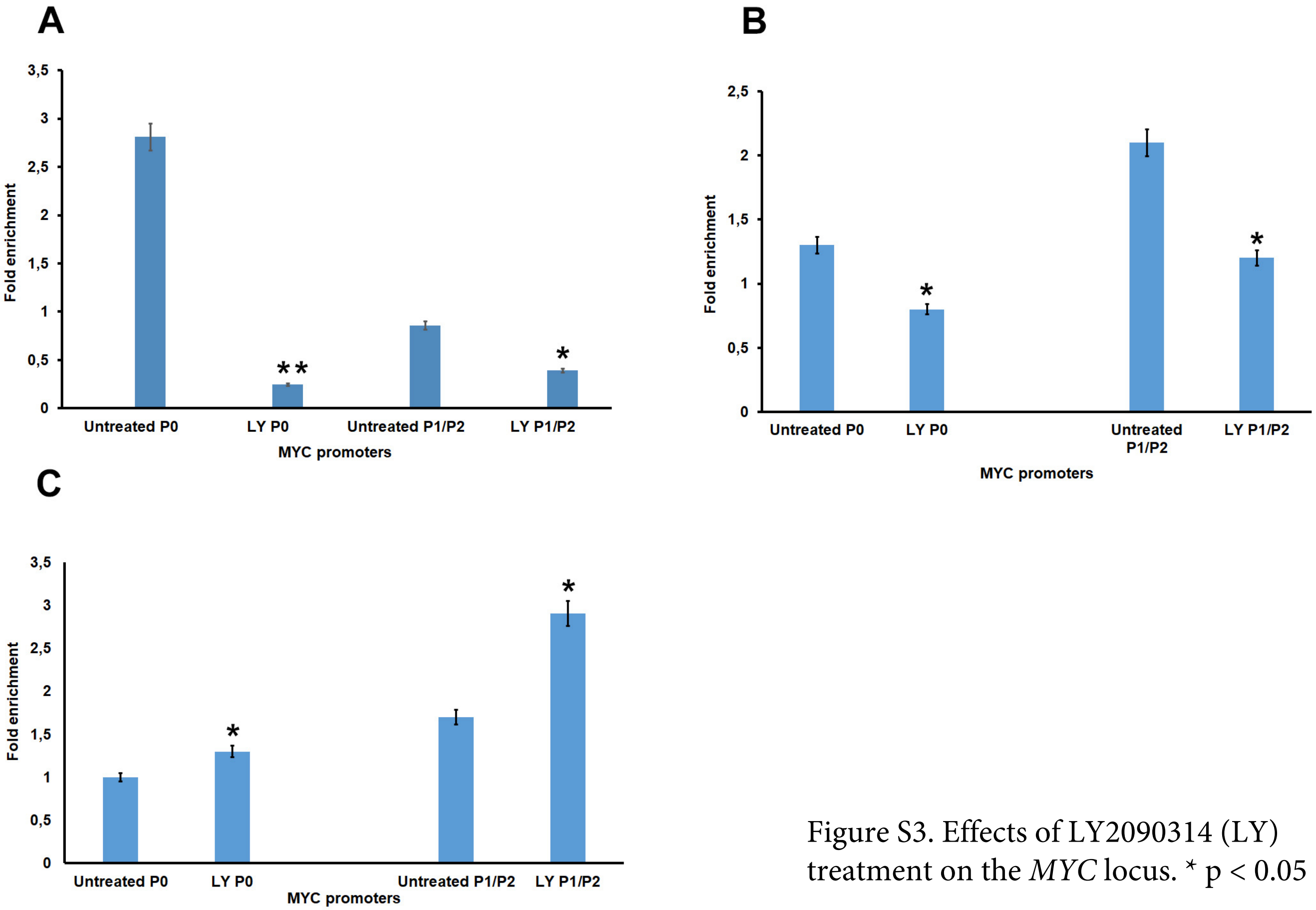

Figure S3. Effects of LY2090314 (LY) treatment on the *MYC* locus. * $p < 0.05$

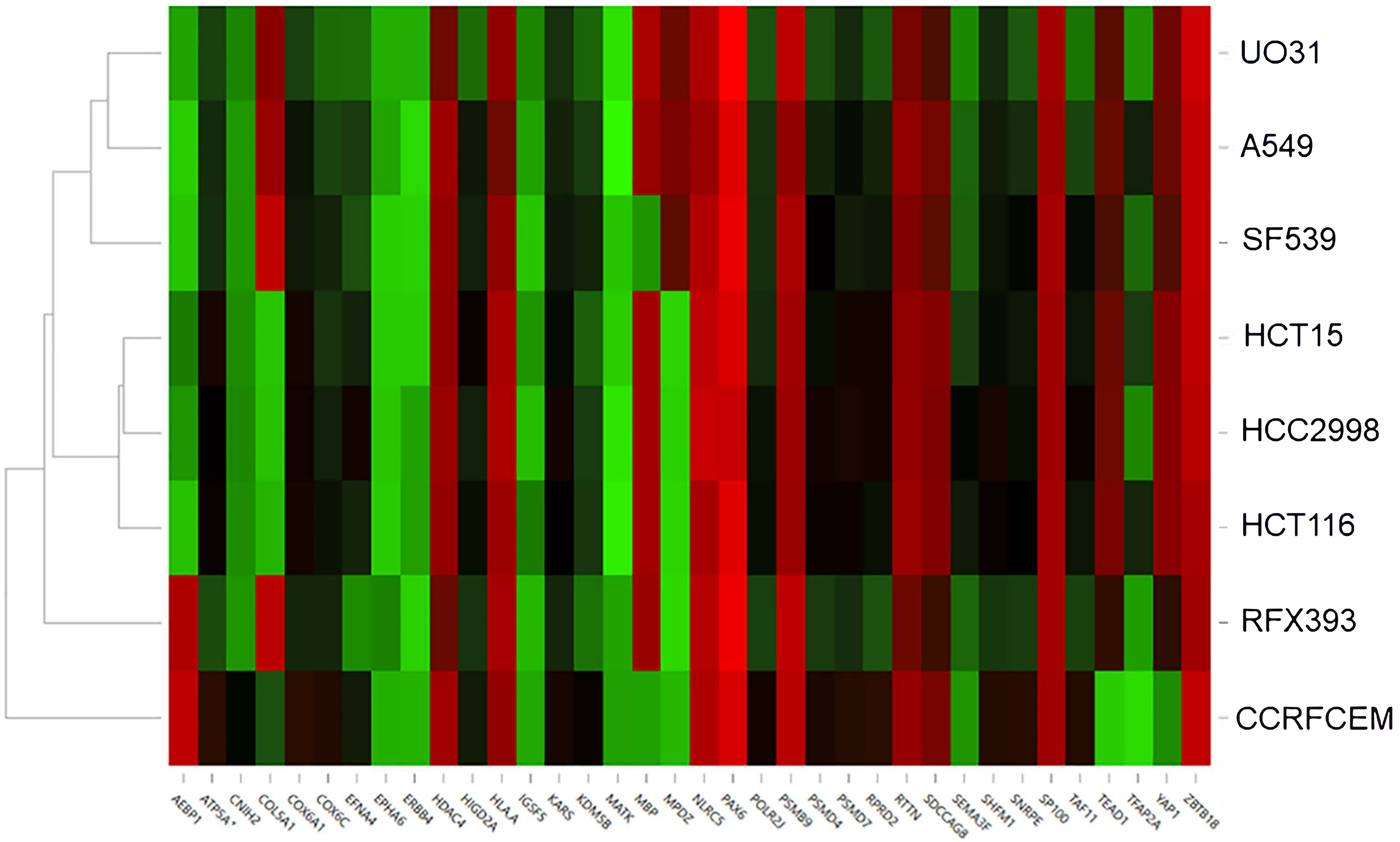

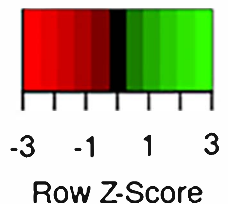

Figure S4. Heatmap of dysregulated genes which were held in common among cancer cell lines carrying mutation in cohesin genes.

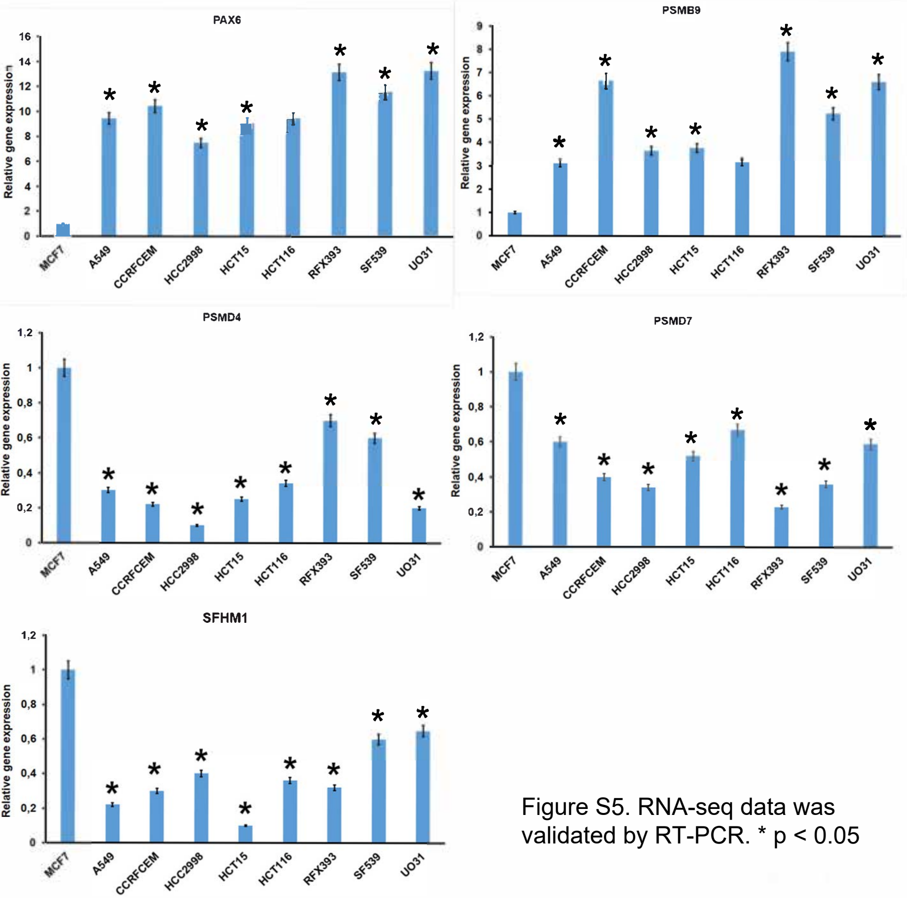

Figure S5. RNA-seq data was validated by RT-PCR. * $p < 0.05$

DataSet

Gene	log2FoldChange	FDR	A549
FBP1	14,83041042	1,16E-22	
PRMT6	14,62005523	3,01E-22	
TACSTD2	14,1320197	1,31E-20	
RAB25	13,94198259	4,28E-20	
ESR1	13,54465197	4,74E-19	
MATK	13,41420953	1,11E-18	
BGN	13,03006642	5,27E-06	
DSCAM-AS	12,99903837	3,03E-10	
GGT6	12,6265048	2,07E-15	
PRSS8	12,54176389	1,30E-16	
ADAMTS19	12,446067	4,40E-16	
APCDD1	12,40906493	1,86E-71	
ZNF253	12,33136668	8,13E-16	
FXYD3	12,31128069	1,52E-08	
ALDH3B2	12,2652071	1,26E-14	
FREM2	12,0110289	1,74E-42	
ZNF93	11,91133862	1,04E-14	
RBBP8NL	11,89876632	1,73E-14	
DLX5	11,85182118	1,66E-14	
ZNF100	11,8094264	1,83E-14	
MAL2	11,73008956	2,24E-40	
FAR2	11,71831126	2,99E-14	
PLXDC2	11,67111965	5,53E-14	
PRDX2	11,57218469	6,77E-167	
NCAM2	11,49779772	3,52E-13	
SMIM22	11,35793171	3,62E-13	
EPHA1	11,32788026	4,25E-13	
ZNF429	11,30010511	3,64E-13	
BSPRY	11,28981192	2,14E-13	
JPH1	11,28650522	4,75E-12	
PCP4	11,22517096	6,42E-13	
MBNL3	11,18423118	2,82E-13	
ESRP1	11,1796724	4,67E-65	
ASCL4	11,17762107	8,26E-13	
OVOL1	11,10960529	3,40E-12	
EFS	11,04998154	1,72E-12	
TDRD1	11,03233132	1,62E-12	
ILDR1	11,02676272	1,71E-12	
GRHL2	10,95825971	2,36E-33	
PGR	10,84690151	7,39E-06	
DYNC1I1	10,79875822	2,20E-12	
FAM3B	10,78903026	6,60E-12	
AP4B1-AS1	10,75384802	8,22E-12	
COLEC12	10,7514003	1,54E-11	
NAALADL2	10,73321652	1,35E-11	
TSTD1	10,6768621	4,21E-12	
S100A14	10,64761245	5,17E-65	
ESRP2	10,60179046	2,58E-32	
SPESP1	10,59619128	3,25E-11	

CCDC64B	10,57424625	3,71E-11
PROM2	10,54686299	4,16E-08
TMEM125	10,53290735	2,71E-11
ZNF433	10,52293406	3,00E-11
ODAM	10,45076285	4,28E-11
CEACAM21	10,4412263	4,96E-11
ZNF83	10,4347698	1,84E-11
TSPEAR	10,40768338	1,34E-10
PCDHA10	10,32226235	1,14E-10
ZNF347	10,24882037	1,98E-10
CDH3	10,24492045	5,53E-124
SPATC1L	10,21405667	5,29E-11
HLA-DQB1	10,14768265	1,17E-08
RNF223	10,14166989	3,14E-10
ZNF785	10,11865613	2,72E-10
ZNF682	10,07156341	3,73E-10
LINC00052	10,05833253	6,32E-09
C18orf63	10,05154707	5,76E-10
ERBB4	9,965667541	1,06E-09
ZNF732	9,901721262	1,94E-09
BATF	9,879758951	2,49E-09
ZNF737	9,834656474	2,69E-17
ZNF681	9,823772551	3,09E-10
CDKN2B	9,823614992	2,67E-09
CXCL12	9,772918139	2,14E-09
ELF5	9,769088325	1,77E-08
ZNF888	9,739908544	5,07E-10
ZNF818P	9,729028372	2,41E-09
LCP1	9,708760872	1,42E-26
GALNT3	9,70494672	1,11E-26
MACC1	9,688912049	2,84E-08
CHST8	9,683750692	3,04E-09
HLA-DRB1	9,662291349	1,35E-08
NUP210L	9,612554421	9,63E-10
BEND5	9,571107541	7,92E-09
SLC2A10	9,560054365	7,20E-09
TUBA3E	9,551723937	1,21E-08
SPINT1	9,509444793	8,01E-84
ZNF492	9,493532896	4,81E-08
GUCY1A3	9,488880467	1,81E-09
SPINK5	9,484146943	5,15E-08
CBLC	9,482769011	1,16E-08
ZNF790	9,479910883	1,36E-08
ACTG2	9,47650021	1,98E-07
TYRP1	9,465833765	1,09E-08
PRLR	9,44860426	4,27E-25
THNSL2	9,447244425	3,37E-09
SLITRK4	9,438807435	1,65E-08
MAPK13	9,425293645	3,85E-55
KIAA1324L	9,388807667	6,27E-25

TMPRSS2	9,36377236	3,17E-08
ZNF793	9,349966905	3,34E-09
FUT9	9,332148559	2,03E-08
DDX43	9,319035884	3,09E-08
ST14	9,316630296	1,16E-67
C2orf54	9,254875825	3,89E-07
PLA2G4F	9,251776165	1,84E-06
ZNF85	9,179045737	1,70E-16
ZNF730	9,17094468	5,52E-08
MARCH1	9,166331249	9,83E-08
LYPD6B	9,164200591	5,50E-08
FCGR1A	9,152924489	3,81E-07
CLDN7	9,12765705	9,34E-97
HBA1	9,114209555	1,59E-07
RGS6	9,11160318	1,24E-07
SYK	9,106794427	2,54E-23
ZNF137P	9,091160297	7,95E-08
CNGB3	9,065049482	1,23E-07
LOC339862	9,062887334	1,02E-07
SEMA4A	9,003356912	1,01E-15
LINC00992	8,992022302	1,49E-07
ZNF876P	8,986313291	1,32E-07
ACSS3	8,983670889	7,36E-73
ACTR3C	8,978650414	1,36E-07
ZNF204P	8,9640701	1,86E-07
PTCHD2	8,962682645	8,83E-07
C1orf210	8,929573642	3,42E-07
FOXP2	8,918765776	3,31E-07
GSG1L	8,91198105	2,13E-07
LRRC61	8,873485942	7,75E-32
LINC01132	8,868525622	1,52E-06
AMZ1	8,838348871	6,76E-07
TNFRSF18	8,826572342	2,90E-06
HTR2C	8,820015358	3,27E-07
TMEM163	8,813256365	4,82E-07
KIAA1257	8,803220397	6,26E-07
AEBP1	8,781880523	6,49E-07
STAP2	8,770479719	2,70E-42
KCNMB2-A	8,769377342	4,10E-07
LOC10028[illegible]	8,76929788	4,96E-07
PLA2G3	8,756803372	3,58E-06
JAM2	8,731511772	3,34E-06
TMPRSS4	8,719267345	9,23E-07
CDC42EP5	8,717854853	5,52E-07
TPTE	8,700381588	6,98E-07
DNAJA4	8,696071679	2,99E-20
LOC64491[illegible]	8,687562275	1,02E-06
ERG	8,68448461	6,45E-07
ADAMTS1[illegible]	8,677994105	6,92E-07
DSCAM	8,675508209	1,90E-06

AFF3	8,657011209	1,01E-06
SEMA3D	8,62932204	3,23E-06
LINC00664	8,625251437	2,04E-06
EPPK1	8,620669439	2,80E-32
C4orf19	8,588437573	1,30E-06
MTMR7	8,574084857	1,15E-06
PCDHA6	8,556135663	7,65E-38
KRTAP5-AS	8,541633863	0,000191
DACH1	8,518907637	1,50E-06
PAX5	8,518213966	1,26E-05
KRT17	8,50290761	8,31E-07
SAMD10	8,495123669	4,79E-07
FAM81B	8,491277455	2,52E-06
ZIC4	8,464396038	2,02E-06
ZNF486	8,460311561	2,30E-07
PDZRN3	8,450499434	2,13E-06
ALOX15	8,440487764	5,38E-15
KDF1	8,42491246	4,35E-19
RFTN2	8,408479654	5,10E-06
TMEM132E	8,393316481	0,000203
FAM83B	8,393112138	3,70E-25
C5AR2	8,380655082	5,71E-12
ZNF736	8,354177223	4,82E-19
SYNE4	8,338549821	1,55E-06
HLA-DRB5	8,334913548	6,70E-06
RASL11B	8,330938414	3,44E-13
NMUR2	8,313928667	4,40E-06
TCHHL1	8,308746194	0,000319
ZNF563	8,267646706	5,36E-06
HPN	8,242725734	2,19E-12
NODAL	8,238767773	1,21E-05
ZNF610	8,232102299	6,51E-06
RAMP1	8,199869708	1,14E-05
ZNF98	8,171776139	1,92E-05
HOXD12	8,168215722	4,60E-05
HS6ST3	8,146051609	1,17E-05
DNALI1	8,135183555	1,18E-05
F7	8,128493987	5,30E-05
BST2	8,108503901	3,01E-21
MAPK4	8,107109971	6,76E-06
ZIC1	8,10504593	1,05E-20
C2CD4D	8,097641749	1,50E-05
ZNF709	8,092340505	1,27E-05
TRPS1	8,067979948	5,09E-17
B3GALNT1	8,067570231	8,40E-22
TRIL	8,053720831	2,75E-05
GPR68	8,049848995	1,74E-05
FLT3	8,04129708	1,65E-05
TRPV6	8,038225858	1,78E-05
RENBP	8,031831954	2,83E-05

NPAS3	8,028116868	3,15E-05
CCDC110	8,024958438	4,10E-05
TMPRSS13	8,008894666	3,39E-16
FOXI3	7,998684963	2,30E-05
ADIRF	7,990448625	3,79E-21
POU6F2	7,98359163	2,52E-05
GFRA3	7,963203278	0,0001
RDH16	7,957702927	1,53E-16
HIST1H3G	7,956552419	2,64E-05
CDH18	7,955065638	3,12E-05
GHR	7,954923934	2,55E-05
ZNF442	7,950850984	2,51E-05
TTYH1	7,923392024	0,000144
ZFP2	7,919198424	6,37E-05
SLIT2	7,910641703	3,05E-05
ZNF345	7,907283357	3,14E-05
XKR7	7,899483453	6,68E-05
CYP1A1	7,843074429	5,06E-11
VAMP5	7,8213863	0,000224
ICOS	7,815981104	5,38E-05
ARL11	7,814917018	5,32E-05
LOC440461	7,81401817	0,000119
BARX2	7,809457739	0,000252
LAD1	7,807514084	5,80E-103
EMCN	7,801230297	5,93E-05
TMEM30B	7,800130436	6,31E-59
LINC00885	7,793159707	0,000321
HOPX	7,791270704	0,000265
BEX5	7,772639197	0,000212
ARHGEF34I	7,767242843	4,86E-06
GNAS-AS1	7,750936418	6,68E-05
LEF1	7,749065909	6,69E-92
CHRM1	7,726259495	8,97E-10
SEPP1	7,710620354	0,000324
CALML5	7,700700032	0,000153
SGCG	7,69197486	0,000236
TLX1NB	7,68104446	8,88E-05
CBFA2T3	7,673801792	7,07E-33
LOC101929	7,637671746	0,000142
ARHGEF38	7,636647632	0,00015
SPTSSB	7,631683273	1,77E-26
CAPN9	7,621159061	0,00015
TFF3	7,617133808	2,88E-10
DEFB126	7,612758432	0,000122
ACTL10	7,611351406	7,65E-06
LRRN1	7,608945242	9,16E-06
AATBC	7,605317577	0,000137
FAM109B	7,600133304	1,55E-10
ZNF441	7,592806835	0,000335
OXGR1	7,584372464	0,000197

LOC100132	7,57249996	0,000504
KDR	7,572400806	0,000169
CELF3	7,561609831	0,000245
NRG3	7,534299835	1,53E-05
IBSP	7,522312632	0,000186
GRID2IP	7,509961224	0,000189
GNG13	7,497170312	2,47E-05
RASGRF1	7,461159018	6,33E-31
EDIL3	7,460955263	0,000807
SAMD12-A	7,444510042	0,000317
FAM83F	7,417324474	0,000689
GRIK3	7,408737157	5,50E-05
PKP1	7,371365235	2,07E-15
C8orf46	7,367596428	0,000363
ZNF793-AS	7,333708463	0,000401
CALCR	7,330792299	3,15E-05
MDK	7,329280415	8,49E-228
CDYL2	7,306026717	6,69E-13
MPZL2	7,295618508	3,57E-12
EPN3	7,2826831	1,29E-80
SERPINB5	7,280236538	0,00025
LRFN5	7,270260311	0,000522
MPPED2	7,266708945	8,15E-05
PACRG	7,239594438	0,000618
ENG	7,238045033	0,000803
SOX11	7,232646995	0,000685
HS6ST2	7,229214726	1,76E-35
LRRC2	7,214852499	0,000862
MALRD1	7,212976414	1,61E-08
TENM1	7,212127908	5,72E-05
STMND1	7,212103084	4,02E-05
LOC103091	7,199779518	0,000688
OAS2	7,198058661	0,000315
IZUMO1	7,191504247	0,000797
HAGLROS	7,177359941	0,000751
ZNF600	7,126512054	7,57E-20
ABCB4	7,118691368	0,000178
RAB17	7,11011167	1,80E-14
CARD14	7,078809261	9,44E-05
ZG16B	7,07832301	4,19E-07
PKIB	7,070730183	1,89E-72
ENPP5	7,070085369	4,23E-08
PCDHB3	7,05446232	3,78E-32
MYRFL	7,053231097	0,000509
CCL5	7,039264703	2,75E-06
RTN1	6,961328324	0,000124
PRSS22	6,93865583	8,39E-08
TTC29	6,91362291	0,000123
LRP2	6,910383024	2,77E-06
PVALB	6,877403456	0,000299

ALG1L	6,871080414	4,51E-20
TUBA3D	6,867444483	1,97E-07
TFF1	6,865682269	1,72E-49
DTNA	6,847313504	8,31E-29
LINC00341	6,833344351	0,000412
IFITM1	6,811160127	1,88E-11
IRF6	6,801426994	1,99E-24
WIF1	6,796959046	0,000739
C12orf56	6,788019918	4,81E-11
SLC6A14	6,774293991	0,000211
ESPN	6,732996072	7,17E-17
PCDH18	6,713099848	8,42E-17
TARP	6,678649321	0,000856
GLDN	6,672419746	0,000327
PLEKHG6	6,664794941	1,05E-15
PTPRO	6,632778017	7,57E-20
INHA	6,6303522	0,00016
PCDHGA10	6,613810193	0,000485
SLC1A3	6,590804847	0,000389
CYP2J2	6,57240289	1,71E-21
SLC27A3	6,559140853	4,98E-67
GLI3	6,552948611	2,22E-18
ZFP69	6,547506036	2,77E-09
LINC00925	6,532813428	9,31E-12
YBX2	6,515444569	8,25E-52
RAB9B	6,488122817	0,000602
SHISA2	6,467103046	2,37E-06
TCEA3	6,448826413	5,85E-58
SPSB4	6,435596296	4,54E-06
AARD	6,430288504	1,56E-14
ERC2	6,425396772	1,46E-09
DNAJC15	6,421501319	1,54E-66
TDRD5	6,39332679	3,06E-15
RPRM	6,389094704	8,63E-06
EPCAM	6,377018631	2,03E-167
PCDHB9	6,373900079	2,69E-14
SH3GL3	6,356069435	3,03E-06
SLC9A4	6,335273598	4,13E-09
CLIC3	6,329227295	9,69E-07
LOC14870[illegible]	6,314051121	1,59E-08
FIRRE	6,28080176	9,13E-08
SUSD3	6,27383037	1,51E-14
ADGRB1	6,200154019	2,16E-39
BMP7	6,184188752	2,22E-108
ZNF91	6,171995639	5,49E-46
C19orf57	6,167351559	2,18E-05
SLC24A3	6,143904596	4,19E-13
MCTS2P	6,13637906	8,62E-24
NRXN3	6,134164675	1,51E-11
MPP7	6,103119652	1,18E-55

ZNF525	6,099002372	5,05E-42
MARVELD3	6,092755173	2,16E-32
GADD45G	6,079016868	1,13E-13
C15orf59	6,069454368	5,68E-24
MAN1A1	6,068593759	4,97E-31
TMEM154	6,051395481	8,28E-07
ASS1	6,044900384	9,40E-52
B3GALT4	6,036186256	7,08E-06
DEF6	6,031605117	9,63E-17
ZNF808	6,007533098	7,76E-18
ZNF816	5,995890587	1,62E-21
PCDHB16	5,970615944	1,70E-30
MYRIP	5,965754524	2,90E-13
CCM2L	5,95157401	5,47E-08
PACSIN1	5,949095291	3,25E-19
SCN4A	5,938280394	5,34E-12
COL9A2	5,934416912	8,07E-15
TEX14	5,932995707	0,000127
CLDN4	5,920921972	2,67E-60
MFAP2	5,919547915	1,71E-21
LNX1	5,896161085	0,000589
PCDHB14	5,890687652	3,41E-30
SYT7	5,871970522	2,94E-62
MAFB	5,871700811	9,03E-23
CDS1	5,848730034	1,75E-58
PSTPIP2	5,848126736	1,79E-24
CRISPLD1	5,840080017	3,49E-22
ZSCAN12P1	5,832889491	1,47E-12
CRABP2	5,826674353	1,68E-30
EFEMP1	5,797022524	4,97E-51
KLHDC7B	5,781377452	1,90E-10
GATA3	5,776426147	1,28E-102
CLCA2	5,770434805	0,000981
TMEM229B	5,763737401	2,47E-17
NFATC4	5,759147193	3,96E-10
ZNF117	5,75694458	9,02E-24
ATP2C2	5,74386306	8,51E-47
SEMA6A	5,734032471	1,26E-10
FAM178B	5,719694848	0,000115
OGDHL	5,719548051	7,61E-27
HIST1H2BH	5,715259331	0,00012
SMARCA4	5,70742558	3,08E-116
RORC	5,707248179	0,000153
ICA1	5,707127962	5,80E-52
TXNIP	5,705164737	6,13E-05
PAH	5,675742535	0,000181
BIK	5,652469505	7,41E-09
ARHGDIG	5,638704936	8,05E-27
LPAR2	5,634530708	2,74E-57
ZBED6CL	5,611117664	5,12E-10

SECTM1	5,610321391	2,70E-19
PCDHB13	5,599447839	3,45E-22
LYPD6	5,595760254	1,99E-11
PPM1E	5,58701164	1,78E-20
SAMD12	5,577199654	1,55E-24
RORA	5,576672513	1,55E-31
IRX2	5,56643445	4,58E-38
EPHA6	5,553663132	7,42E-13
GNG7	5,552573557	1,05E-08
ARHGEF35	5,533251525	2,67E-17
SERHL2	5,525407507	8,06E-05
BMP2	5,514819598	2,64E-42
KRTCAP3	5,494779294	1,48E-28
TP73	5,486190943	3,31E-11
C10orf82	5,481940177	4,22E-09
AMOT	5,479846482	2,00E-42
KREMEN2	5,463306284	5,96E-56
ZNF701	5,457880934	1,85E-29
ZNF493	5,456753268	3,94E-09
IGSF5	5,452930914	2,07E-07
ZMAT1	5,452215211	0,00041
GATA3-AS1	5,435280462	0,000529
ZNF468	5,416840303	2,31E-46
FAM46C	5,401532672	6,77E-14
PCDHA11	5,387866268	4,98E-36
BMF	5,370544305	4,68E-06
PCDHGB7	5,369537259	0,000673
OVOL2	5,357992661	7,91E-09
PRDM6	5,356139201	6,64E-09
DENND2D	5,350816163	2,46E-54
GPC4	5,338294687	1,31E-12
LRRC10B	5,331171117	0,000859
SPDEF	5,330018154	6,80E-07
BEST1	5,317284797	0,00059
FAM160A1	5,289782832	1,01E-06
ELMO3	5,270462484	5,11E-53
ZNF836	5,269009638	9,48E-28
C5orf38	5,265811835	4,20E-24
CCSER1	5,261316299	0,000688
N4BP3	5,259829608	3,42E-33
EDAR	5,250442537	1,72E-05
ZNF440	5,233749905	6,28E-13
BLNK	5,229732928	0,000115
LOC10272З	5,203567545	0,000136
PCDHA7	5,196685206	4,92E-09
SYTL1	5,190488409	9,98E-31
PCDH1	5,183260244	2,81E-36
MYT1	5,180608033	2,25E-10
PCDHA13	5,168406282	1,82E-06
KCNN2	5,16414258	2,83E-06

DLL1	5,153937005	2,09E-06
TIMP3	5,145814019	4,89E-12
STARD10	5,139347904	2,09E-45
LINC00649	5,125895933	0,000191
RET	5,123271367	9,66E-13
CNKSR1	5,122783386	7,93E-15
MSI1	5,094031586	4,33E-27
IGSF9	5,072873907	5,56E-62
AIF1L	5,055960622	1,30E-119
SCX	5,051045549	8,24E-05
ZNF608	5,047423228	3,96E-40
SNTB1	5,044210249	1,35E-61
ABAT	5,037055893	7,58E-14
FAR2P1	5,036413681	1,04E-07
ADAMTS17	5,031365386	1,29E-05
CMYA5	5,027073419	0,00036
PTPRG-AS1	5,009501707	1,94E-13
DUSP2	5,001344841	7,03E-11
CLDN3	4,997205832	2,54E-94
EGR3	4,994333032	1,19E-05
REPS2	4,99070384	1,21E-06
KIAA1324	4,984397766	1,90E-21
PRR15L	4,931968792	3,59E-30
COLGALT2	4,930403624	0,000339
IFI27	4,921821682	1,21E-05
LINC00665	4,92072504	4,43E-33
APOA1	4,891460919	0,000261
CNTNAP2	4,889918853	3,11E-14
FAM46B	4,88803436	1,27E-20
FAM83H-A	4,876921829	5,15E-75
CXCR4	4,867005778	1,94E-14
HOXC-AS3	4,865035624	4,49E-07
FRMPD1	4,851824618	2,94E-06
GUCY1A2	4,847074825	3,67E-31
MECOM	4,845004455	8,90E-28
IFITM3	4,8200885	1,00E-27
CNIH2	4,810470697	2,36E-17
GLS2	4,806486514	2,43E-12
IRX4	4,798144078	9,20E-09
TMEM139	4,787336381	1,62E-05
TMEM150C	4,785630179	8,58E-31
FGF12	4,776838345	4,67E-18
DLX3	4,77617894	2,36E-31
LOC728739	4,760257817	0,000148
PCDHB10	4,759841689	4,26E-07
ANXA9	4,747108086	4,46E-21
AP3B2	4,729971521	1,05E-05
SDC2	4,727574446	2,68E-25
POU3F3	4,720000736	2,88E-07
GPM6B	4,71871808	0,000523

EXPH5	4,713207179	2,47E-25
KCNU1	4,713150893	0,000618
IRX3	4,694323827	6,28E-56
NPY1R	4,693879639	1,11E-26
PCDH19	4,686961676	8,49E-05
PGBD5	4,662481685	2,84E-07
CIART	4,657969799	1,80E-19
LYPD3	4,652451765	2,00E-20
SEMA7A	4,650591286	9,39E-22
MARK1	4,624023996	0,000285
NDNF	4,621262159	0,000855
PCDHB8	4,591189461	1,12E-30
CCDC83	4,582804189	7,95E-06
ZNF738	4,580475989	1,50E-15
PCDHB4	4,576658232	5,68E-06
ANO9	4,569303708	2,12E-11
TENM4	4,558515975	3,18E-08
NANOS1	4,557269138	8,19E-33
PRR36	4,551518142	4,68E-118
FAAH	4,550645608	5,56E-28
PTK7	4,536419318	6,54E-87
FHDC1	4,52897571	1,24E-12
ZNF790-AS	4,528369477	9,09E-13
GSTO2	4,517398771	7,92E-39
PIP5K1B	4,51649174	4,92E-05
DSC2	4,504815043	3,60E-68
OASL	4,504641302	0,000708
KCNK15	4,497692281	1,33E-21
SLC1A2	4,484904596	2,66E-31
GUCY1B3	4,458440976	8,40E-05
ENPEP	4,447823368	1,28E-07
AP1M2	4,429050626	5,28E-49
DEGS2	4,423472645	2,10E-11
GDPD3	4,421242033	1,11E-06
WASF3	4,419413473	2,25E-08
CHRM3	4,417565309	7,38E-10
UPK2	4,411408044	2,21E-06
ENTPD1	4,407378321	1,56E-10
TRPV4	4,390706107	1,17E-08
GPR158	4,383967675	1,82E-09
ZNF165	4,382822746	5,44E-32
MUC1	4,379689796	1,01E-13
TMC4	4,378400332	1,44E-32
DLGAP3	4,366705538	1,58E-12
NPW	4,348117324	1,07E-06
LDHD	4,346571315	5,79E-06
POTEF	4,344696395	0,000885
FAM227B	4,33934069	1,90E-13
RUNDC3A-/	4,339094979	3,23E-08
C14orf37	4,336180078	3,93E-05

SNCAIP	4,319654608	1,41E-06
CGN	4,313703097	4,63E-27
FAM221A	4,295611922	6,71E-08
HOXC11	4,292147598	1,29E-08
ERBB3	4,2814789	8,83E-83
TMEM45B	4,280726636	4,79E-21
CPLX1	4,267482513	8,55E-18
SOCS1	4,226145319	2,80E-16
ARTN	4,22583631	2,53E-05
RPS6KL1	4,224068313	1,67E-20
NIPAL1	4,223570373	6,71E-07
ERICH2	4,214426162	2,68E-17
TRIM36	4,210215136	2,01E-30
SHANK1	4,195746407	1,72E-05
DDX26B	4,177422697	1,17E-10
ARRDC4	4,170560998	1,99E-11
ARMCX1	4,167058591	0,000161
BNIPL	4,163102292	5,98E-05
LOXL3	4,145040093	1,05E-15
GREB1	4,144552092	4,49E-54
GFRA1	4,143632006	9,92E-25
RTN4RL1	4,128400732	1,05E-52
C3orf14	4,126933769	1,38E-50
NMU	4,125414871	2,44E-10
PRRG2	4,106314836	1,26E-10
PCDH7	4,097166023	1,54E-11
CKMT1B	4,095131411	5,89E-23
MEF2C	4,093316656	0,000361
TNFSF13	4,092052326	2,45E-07
PARP8	4,072202529	6,09E-14
LOXL1	4,072052731	1,45E-07
BDH1	4,064577007	3,60E-58
ZNF391	4,059756697	1,08E-09
GRAMD3	4,051262496	2,86E-16
PPL	4,042232789	1,34E-33
BCAS1	4,039722132	3,58E-06
SNAR-E	4,010549659	0,000411
LIN7A	4,004782362	5,58E-26
SIAH2	3,993929674	4,24E-49
SLC15A2	3,992939193	1,01E-05
GJB2	3,987166281	5,92E-12
ZNF616	3,984561394	1,37E-38
LAMP3	3,976949563	5,39E-07
NUPR1	3,966466825	0,000386
PAX8-AS1	3,965252367	1,62E-12
CDK14	3,956422981	2,13E-05
HOXC10	3,953105767	9,79E-47
WNT6	3,952213668	9,91E-05
FUT1	3,945369637	1,33E-06
LSR	3,940984454	7,39E-44

SBK1	3,933955311	4,79E-63
GRHL1	3,924327405	1,93E-16
GLUL	3,919342765	1,35E-28
CDH1	3,905117718	4,10E-64
SH3BGR	3,903970758	9,69E-10
FBXL16	3,897396633	5,50E-17
BEX4	3,892726409	6,78E-05
PCBP3	3,886635021	1,29E-11
CRLF1	3,882929453	3,62E-07
SYT12	3,879739534	1,15E-17
ASCL1	3,837626721	4,42E-09
STC1	3,836429433	1,60E-30
ACE	3,829838015	0,000384
TRIM2	3,82721153	4,50E-16
QPRT	3,82716319	1,98E-13
ACKR3	3,82514454	8,35E-46
RNF208	3,814024905	2,14E-19
TNFAIP8L3	3,806091648	8,30E-14
IL17RE	3,795336081	2,86E-07
CRACR2B	3,776206992	5,15E-23
LOC10099€	3,768428478	2,70E-08
HSPB8	3,754206095	8,30E-23
GALNT16	3,752563718	1,36E-06
MPZL3	3,750017186	1,93E-16
GYLTL1B	3,742572224	1,14E-30
SLC37A1	3,739374349	8,08E-16
AIM1	3,735130785	1,07E-17
KRTAP3-1	3,727938956	5,39E-08
ARHGEF5	3,72245082	1,09E-21
KCNH2	3,72183225	6,89E-17
RNF125	3,714773237	6,12E-06
ZNF829	3,709699598	2,08E-17
DHRS2	3,706801444	4,19E-16
ZNF718	3,705410438	7,50E-19
KCNC3	3,697428039	4,28E-27
MMP11	3,688084169	8,76E-06
HOTAIR	3,676098714	0,00012
IFI30	3,675709455	2,22E-28
ISG15	3,672052552	8,28E-06
UNC5A	3,66793285	0,000885
ANKRD34A	3,666002996	4,57E-16
KLC3	3,663169036	3,55E-05
IFIH1	3,660706747	7,52E-08
IGFBP5	3,648680451	1,72E-17
ETNK2	3,639555605	3,49E-16
HOOK2	3,632938995	3,63E-58
PRICKLE2-A	3,618682874	0,000109
DLG3	3,607228977	7,07E-44
ZNF467	3,605886339	8,55E-27
HOOK1	3,6027988	1,28E-27

VIPR1	3,59174282	1,45E-05
SP6	3,590172692	6,31E-14
TGM1	3,585087254	0,000684
HID1	3,579169221	2,14E-18
GJA3	3,561266401	1,68E-09
IRX5	3,557846914	3,59E-24
SULF2	3,556889393	2,53E-35
GPR37L1	3,551974524	0,000658
KCNJ8	3,547483322	7,82E-34
ADGRV1	3,539949841	1,41E-07
ARVCF	3,538022603	7,55E-31
LINC01389	3,535512904	0,00073
TJP3	3,526879457	2,03E-08
LOC729737	3,515610545	0,000347
TBC1D30	3,505730393	6,59E-40
FLJ23867	3,496501234	2,01E-21
ARG2	3,494718499	1,49E-12
PDE5A	3,492061084	3,82E-06
PSMD6	3,486575728	4,61E-57
CEL	3,483922882	0,000361
TJP2	3,483053847	5,56E-21
KRT23	3,481700964	2,67E-05
ZNF43	3,476039486	1,48E-39
CHGA	3,470913768	7,29E-14
PDGFB	3,468314051	9,28E-11
CKMT1A	3,460311105	1,30E-23
XBP1	3,459393925	2,77E-17
FAM84B	3,456803643	3,36E-18
IDH2	3,456584968	5,91E-46
ATP6V1C2	3,455952653	1,35E-13
SH3YL1	3,454099411	4,68E-34
SLC44A2	3,452321569	7,01E-53
PDE4DIP	3,449267762	1,09E-24
BRIP1	3,447050662	2,25E-59
MB	3,446419539	1,11E-17
ABLIM2	3,431906468	1,36E-05
SOWAHB	3,413419381	0,000355
TINCR	3,411423707	9,27E-09
TMEM191A	3,396892746	3,54E-20
LOC389834	3,385744641	1,74E-07
KCNIP3	3,370818668	8,85E-08
AGAP2	3,369331292	7,91E-18
TFAP2C	3,361847755	3,49E-41
FGD3	3,339430989	1,72E-08
SLC26A4-A	3,33780832	0,00022
GALNT6	3,331929834	2,75E-19
ITPKB	3,331447018	1,04E-21
DDAH2	3,327653165	2,29E-35
CDK18	3,325447948	1,51E-13
ZNF311	3,317658326	1,09E-05

VGF	3,308826383	1,64E-15
C6orf132	3,306070346	2,90E-30
EMID1	3,302924388	4,52E-06
KIAA0040	3,301036085	2,02E-13
KCNK6	3,296231426	1,31E-10
ASCL5	3,293576788	0,000287
HMHA1	3,293310513	2,08E-12
PLEKHF2	3,284719718	6,58E-44
ZNF596	3,273781089	1,14E-06
SHISA9	3,269461363	1,09E-09
FRAT1	3,268138586	0,000179
PRIMA1	3,262025823	8,03E-06
KNDC1	3,255926119	6,06E-07
USP32	3,253688782	7,78E-28
RGS16	3,249190434	1,05E-06
NYNRIN	3,243143929	0,000252
TMEM198	3,242926293	2,11E-06
RND2	3,242765011	4,38E-12
IFI6	3,235243938	6,61E-06
CD8A	3,228946123	3,67E-07
ARHGEF16	3,221109901	1,08E-20
WNT11	3,210263087	8,65E-06
PGM5	3,206105972	0,000306
APPBP2	3,205771024	4,88E-67
ZNF813	3,205438074	4,77E-14
ZNF850	3,203855721	1,39E-06
ZNF28	3,202488395	4,18E-09
GSTM3	3,202176508	3,90E-12
MAPT	3,202150936	1,99E-20
LOC104968	3,195916211	1,95E-08
ASAH2	3,192193409	1,71E-14
GAD1	3,191092918	7,52E-13
PRPH	3,185711453	6,56E-06
LINC00886	3,18394509	5,10E-07
MYO1D	3,182347219	6,62E-22
RNF144B	3,180082786	1,56E-05
RASD1	3,179779299	0,000231
LINC01006	3,17527942	0,000762
GCNT1	3,174992324	2,39E-20
PIK3R3	3,168966541	2,15E-20
HIC1	3,16892032	4,04E-05
PRRT4	3,167464232	0,000938
ERV3-1	3,163752403	3,13E-21
TGIF2	3,159193049	1,50E-22
GMPR	3,156017869	0,000361
ANK3	3,155414414	6,79E-23
RAP1GAP2	3,155201396	5,62E-18
CSTA	3,153870022	7,28E-06
EFNA1	3,150679223	2,92E-12
BCAS2	3,1492152	4,88E-63

PLEKHO1	3,139593012	1,72E-24
NSUN7	3,135652193	4,69E-08
TRIM37	3,126424028	5,37E-67
ARHGAP4	3,116490889	1,17E-05
WNT5A	3,110090063	3,84E-14
SULT1A1	3,100710482	8,82E-06
SDK1	3,100099122	1,95E-07
PARD6B	3,09982801	6,74E-43
RNF182	3,092850914	1,52E-08
EN2	3,09261165	1,13E-05
ZNF700	3,092496824	1,49E-08
SH3BP2	3,087453429	6,07E-09
DENND2C	3,082621365	0,000211
CCDC170	3,082492832	1,73E-07
TLE2	3,070164415	9,86E-28
EEF1A2	3,069776355	3,07E-26
PPIC	3,06682397	1,50E-30
KLHDC9	3,065885734	3,02E-05
NEB	3,050323495	2,63E-16
SH3D21	3,043339396	0,000127
SLC8A1	3,036345957	5,41E-10
ATAD3B	3,035304827	2,31E-08
NXPH4	3,030655546	6,74E-12
SIDT1	3,020383002	5,69E-09
CSDE1	3,01693793	4,42E-40
IPO5P1	3,016806752	6,07E-09
AIM1L	3,01519276	0,000198
CAMK2B	3,008526233	6,98E-08
ZNF695	3,003252236	7,72E-05
KRT19	2,991845031	8,50E-18
MARVELD2	2,984697056	1,07E-23
GPR160	2,97737938	5,91E-11
PLEKHB1	2,974658564	1,14E-07
TSPAN13	2,964132227	5,18E-30
TNK1	2,96390386	2,15E-19
ZNF572	2,959656002	1,15E-07
CXXC4	2,959349309	5,17E-05
GRTP1	2,946842123	2,69E-13
KCNQ1OT1	2,931956676	3,91E-05
TMEM254-	2,922861091	8,67E-05
CAMK2N2	2,922760121	1,93E-11
HSD11B2	2,922404245	2,09E-08
INSIG1	2,918713404	3,78E-16
LNX2	2,916469963	1,06E-39
LINC00959	2,911802724	9,66E-05
VAMP8	2,910816181	9,33E-11
LRRC26	2,910741923	3,78E-09
KCNJ3	2,908141197	0,000113
RGMA	2,907067621	7,26E-10
PPM1D	2,904269698	9,96E-34

RPS6KA2	2,904099026	0,000274
FOXA1	2,903597295	7,07E-53
C1orf233	2,903287223	1,73E-22
CTXN1	2,903247528	3,95E-31
PITPNM3	2,902264408	1,31E-09
GAL	2,896223114	1,47E-14
ADM2	2,893413529	0,000126
RHOD	2,892597262	1,04E-12
HCN4	2,892277509	3,15E-09
OXCT1	2,889995769	6,25E-18
MMP16	2,88865668	3,43E-05
REEP1	2,886748499	0,000708
METRNL	2,88028653	8,61E-21
PFDN4	2,871364217	3,87E-49
DCLK1	2,870575477	3,18E-21
PTK6	2,865289805	5,48E-06
FAM117A	2,863631944	1,40E-15
MEX3A	2,859659448	4,98E-55
KCNMB4	2,858163525	0,000712
CCDC149	2,856789425	4,73E-15
ADAMTS13	2,853196257	0,00011
ZNF354A	2,84987555	1,35E-17
HOMER2	2,840127853	1,19E-27
C1orf226	2,839733618	9,54E-08
RGL2	2,836396723	4,56E-25
LOC28333	2,835152309	8,23E-13
MAPK8IP2	2,831491804	6,51E-07
NDC80	2,826785941	4,59E-13
JHDM1D-A	2,820976028	1,42E-10
PCAT7	2,820910418	3,81E-05
PLXNA4	2,81901736	1,33E-06
ARHGEF19	2,816044027	1,17E-15
MYLIP	2,815188517	4,81E-17
FRAT2	2,814420706	4,28E-11
DTWD1	2,809252208	7,28E-19
HENMT1	2,808228544	6,97E-20
SERTAD4	2,800568169	2,02E-11
CYB561	2,800284763	5,96E-56
MPC1	2,799389571	3,41E-10
MSRB2	2,795633189	4,31E-26
SIX4	2,795160085	1,05E-13
RIC3	2,795022104	0,000201
CRMP1	2,79388554	2,29E-12
NOTCH3	2,791442507	1,49E-32
ZNF425	2,789222349	5,17E-12
SH3BP5	2,78653604	5,98E-39
PAIP2B	2,783726285	5,09E-13
ENPP4	2,782237768	4,77E-07
CAMSAP3	2,775636783	1,96E-31
PPDPF	2,770431647	8,28E-36

LINC00858	2,768108891	0,000775
SEMA3F	2,767986674	7,36E-18
ADGRL1	2,759064828	3,82E-38
PLCB1	2,758629265	2,69E-13
SIKE1	2,744055739	1,74E-42
P2RY2	2,743243828	2,31E-14
JUP	2,74298681	5,60E-14
TEAD3	2,739013695	2,58E-20
HES2	2,732358527	0,000506
EFHD1	2,732222672	7,02E-10
RASGEF1A	2,718467824	9,41E-14
BCAS3	2,716843938	9,08E-24
NIPAL2	2,710676064	4,01E-10
RHOB	2,709061203	6,22E-24
PAQR8	2,704965578	1,57E-09
PTP4A3	2,702129798	1,38E-15
CBS	2,701623379	1,43E-12
GPR27	2,701257575	4,65E-05
C2orf15	2,698128281	1,26E-08
TM7SF2	2,695994196	1,62E-10
STON2	2,694974411	2,04E-24
CPE	2,692092185	8,26E-08
GSTM4	2,687842253	1,19E-27
FAM171A2	2,687326704	2,91E-14
SLC29A2	2,687020741	2,83E-17
FRMD6-AS:	2,681496177	0,0005
CREB3L4	2,675197227	1,16E-19
ASCL2	2,668206393	5,01E-08
CALHM2	2,665957015	6,91E-08
SMPDL3B	2,664846897	4,12E-13
CACNA1D	2,659965322	4,22E-10
DHCR24	2,658043024	3,95E-19
PCLO	2,655012757	6,89E-17
ADAM22	2,652630524	1,16E-17
ALDOC	2,647417276	1,97E-11
GCAT	2,64272333	7,37E-12
PVRL4	2,638088855	5,78E-18
KATNAL2	2,637430606	5,47E-06
GPNMB	2,63690342	1,52E-05
RTN4R	2,63288772	5,07E-08
YIPF2	2,630901521	1,13E-25
NPL	2,629719951	3,83E-08
ADGRG1	2,62727411	1,48E-20
ZNF561-AS	2,626111666	0,000284
NRAS	2,620314488	4,42E-33
FAM174B	2,618801776	3,08E-08
OAZ3	2,615962857	0,000288
TRIM33	2,613884523	3,38E-36
FRMD6	2,607337706	1,40E-22
DCLRE1B	2,606150023	3,74E-23

ALDH6A1	2,602985358	5,21E-16
STARD13	2,602151644	4,10E-13
OLFM1	2,590436411	1,61E-18
MYB	2,586352457	0,00023
PARP10	2,581158948	0,000426
AK7	2,57662642	6,74E-20
COL9A3	2,574454156	0,000558
CASKIN1	2,569002923	3,32E-10
TENM3	2,565113702	1,12E-20
SLC16A14	2,562004599	0,000163
DAAM1	2,560887344	9,30E-25
BCL2	2,560824222	6,66E-06
ACOX3	2,552381837	5,22E-14
RUNX2	2,55238131	3,56E-20
B3GNT4	2,549633769	7,73E-05
SLC7A8	2,549338687	1,34E-08
THOC7	2,546353607	7,16E-37
ARSG	2,541425059	8,59E-10
ZNF554	2,539807958	0,000122
BCL11B	2,53695102	1,37E-22
ACBD7	2,530223659	3,72E-07
HUNK	2,524575682	1,90E-18
GPR153	2,524312729	1,34E-08
DIRAS1	2,52373788	4,21E-17
DUSP9	2,513553335	6,59E-05
ABCD1	2,512648655	1,05E-06
TUBD1	2,498643025	1,34E-15
ZFP3	2,498109849	2,00E-15
ABHD11	2,490505602	7,50E-20
SP140L	2,485862808	3,02E-09
PITPNC1	2,478874605	2,02E-30
PLXNB3	2,478815395	6,22E-10
TGFBR3L	2,475517354	5,85E-05
KIAA1217	2,462423558	3,58E-05
SLC12A8	2,453652151	4,00E-07
RNASEH2A	2,45354608	2,88E-32
CCNG2	2,450252805	5,37E-07
TLE3	2,449518445	7,57E-21
CDC42BPG	2,449129755	5,20E-10
ZNF138	2,448653216	3,40E-17
ZBTB42	2,448547064	2,21E-19
GPR89B	2,447809995	1,59E-06
NTN1	2,446021879	0,00022
NUDT12	2,440638235	4,79E-11
ZNF630	2,437536848	2,36E-05
MYO5C	2,436725547	7,32E-22
GTF2IRD2B	2,435301555	1,65E-11
DDR1	2,42858223	2,45E-09
ARAP2	2,428402275	2,27E-05
ARID5B	2,427301919	1,38E-22

LFNG	2,416636696	9,94E-09
ZHX2	2,414562837	4,88E-19
BAIAP3	2,412182221	0,000655
PDZD4	2,409898461	1,45E-06
DUSP8	2,409571238	8,20E-05
MYH14	2,405892964	6,91E-10
TBC1D9	2,402117949	1,32E-23
SOX13	2,389643373	1,76E-09
ZFYVE28	2,386540448	1,37E-09
ZNF860	2,381123163	0,000848
CASZ1	2,378058575	1,48E-09
ATXN7L2	2,377734706	2,26E-09
ZNF704	2,37066693	8,94E-16
HBP1	2,369118921	5,12E-08
IL17RB	2,368516657	1,70E-05
GCH1	2,365381244	2,60E-14
RHOV	2,362682747	1,48E-07
PLEKHH1	2,355451483	1,73E-22
FAM129A	2,355111155	0,000306
MACROD1	2,345849637	1,78E-07
FIBCD1	2,34547124	0,000448
KLHDC3	2,344216135	6,33E-18
LOC81691	2,343204833	2,88E-06
ZNF217	2,342334387	5,64E-26
ZNF595	2,332518587	8,53E-12
ZNF490	2,330861512	9,84E-09
GATSL3	2,327007019	0,000961
RIMS4	2,322252578	3,91E-05
SLC50A1	2,321382013	3,15E-23
RNASEL	2,320298372	3,69E-10
SLC22A23	2,317811602	1,38E-20
ZBTB12	2,317361141	8,79E-07
CCDC71L	2,316594092	8,49E-08
LINGO1	2,315011913	1,03E-09
GABPB2	2,309359235	5,23E-11
ZNF708	2,308041288	1,24E-07
PHLPP1	2,304492343	1,07E-15
L1CAM	2,304125257	1,87E-08
RAB3IP	2,303826774	2,07E-27
ZNF184	2,302423814	1,90E-18
CRIP2	2,301973125	5,71E-20
LYRM9	2,29277629	0,000409
FUOM	2,290891367	8,39E-08
ZNF44	2,286279158	2,90E-06
SFXN2	2,282294631	1,69E-15
DSP	2,274810511	9,44E-29
TMCO4	2,271363581	4,68E-10
RHOBTB1	2,269336323	3,72E-31
ZNF443	2,2644221	4,17E-08
RSBN1	2,259239491	5,46E-17

MCM3AP-A	2,253761027	2,44E-09
GPX4	2,253655753	1,14E-37
CCDC183-A	2,253595146	7,13E-05
RRBP1	2,249376168	8,67E-14
LINC00960	2,248486967	0,000244
RAB30	2,246267626	3,06E-09
NAPG	2,24046019	1,70E-14
NRXN2	2,237644259	3,87E-09
HIPK1	2,236853196	4,29E-25
DNMT3B	2,234869127	2,00E-19
MTIF3	2,23431439	3,44E-14
FZD1	2,233793601	1,61E-15
WDR83OS	2,233115399	1,63E-28
ZFP62	2,232970408	4,86E-26
JPH3	2,23223697	7,71E-05
EFNB3	2,229144445	2,69E-07
ZNF841	2,226982298	1,52E-07
ZC3H6	2,223676981	2,44E-07
ZNF611	2,222250631	4,13E-09
RAD51C	2,221983567	3,85E-31
RPS6KB1	2,220048311	1,68E-35
TXNDC16	2,218129954	4,11E-06
LRRC37A3	2,21533081	5,63E-07
TBC1D10A	2,214136377	4,05E-10
TRIP6	2,209635395	9,72E-27
PPP1R13B	2,209595264	5,55E-15
ZNF703	2,208890346	1,80E-23
C9orf152	2,208041702	0,000652
DNASE2	2,201706786	1,63E-23
RCOR2	2,199485734	4,21E-05
TMOD2	2,195749595	1,76E-06
NKAIN1	2,194779435	4,54E-11
FBXO27	2,189034305	1,34E-11
FIS1	2,188814195	3,46E-18
CRISPLD2	2,186579322	2,55E-06
FAM43A	2,185513373	1,11E-07
DBN1	2,181449386	4,17E-30
SYNPO2	2,172876894	7,83E-06
SLC7A5	2,168983713	4,97E-09
SLC25A29	2,168827275	3,79E-19
TTC9	2,168520974	8,70E-17
MAPK11	2,16590107	8,16E-07
PRDM4	2,161143922	4,34E-34
BAMBI	2,158932512	1,50E-32
MEPCE	2,152031404	1,76E-34
PRKCH	2,15202524	3,92E-08
ATP1A1-AS	2,151516561	1,83E-06
RANBP17	2,147214203	1,82E-07
PAQR4	2,14612525	1,23E-28
DLG1	2,145991328	7,00E-23

ZNF783	2,142881863	3,78E-10
HEATR6	2,132466161	6,95E-24
ZNF837	2,125563104	0,000146
KANSL1L	2,117189542	1,45E-07
LOC11323(	2,115967785	5,66E-09
GABPB1	2,115031013	1,65E-14
ABCA11P	2,111969928	0,000138
C6orf48	2,111520247	6,71E-07
MMAB	2,109691633	1,43E-24
TEX19	2,108895125	0,00022
ZBTB22	2,104066254	3,45E-11
HMGN1	2,103774151	2,31E-29
FHIT	2,101193335	0,000206
KIF26A	2,100951579	1,78E-06
TAF11	2,100473705	3,72E-20
SALL4	2,096687044	0,000255
KDM7A	2,095612472	4,76E-06
ZNF879	2,095004365	0,000948
ZNF519	2,093648346	2,00E-06
ZNF385A	2,090020909	6,62E-21
EZH2	2,089109422	1,05E-10
TDRKH	2,087679123	1,73E-06
COX6C	2,085412195	2,81E-23
SPTBN2	2,083056186	4,20E-13
N6AMT2	2,081954756	1,15E-06
TMEM178E	2,078698292	2,51E-07
MGMT	2,074830895	2,43E-10
PDCD4	2,071592163	2,46E-13
FBXW9	2,071480927	2,10E-13
ZNF107	2,068080672	2,07E-17
WDR83	2,064433584	4,03E-10
RAB3D	2,064303089	1,04E-11
P2RX4	2,064177818	6,60E-17
C2CD2L	2,06053894	1,20E-07
DHRS13	2,060537405	2,09E-09
FRMD4A	2,056433216	1,51E-06
KIF1A	2,053816557	1,86E-11
TMEM54	2,053466349	7,41E-17
TEF	2,052883502	4,65E-07
ZNF239	2,049958017	0,000191
MED12L	2,04593598	4,99E-05
MYL12B	2,045172989	4,09E-29
MVD	2,044283959	8,89E-08
LMX1B	2,040919081	7,41E-06
VPS52	2,040760932	3,31E-26
PSRC1	2,040519404	7,10E-11
TNNT1	2,035718485	2,06E-19
F11R	2,034162983	2,20E-19
XYLT1	2,027649688	1,34E-05
ADAP1	2,027266626	0,000146

FAM102B	2,025864673	6,96E-17
CCZ1B	2,02584136	1,16E-15
ETS2	2,022973062	8,04E-16
FAM127C	2,020509669	3,60E-15
F12	2,019860239	2,82E-19
DFNB31	2,019167269	6,15E-08
FAM214A	2,018631693	0,000148
TMEM184/	2,016751226	1,39E-06
TC2N	2,015533913	1,53E-08
CRYL1	2,015420912	2,57E-11
LINC01004	2,014503333	0,000216
CSPG5	2,011853844	6,79E-05
CRB3	2,010583588	5,73E-05
GSTZ1	2,009663218	4,87E-13
HDAC10	2,008880241	1,10E-08
C1QTNF9B-	2,005372571	7,42E-05
ZNF627	2,004424658	6,30E-15
EMB	2,004116539	1,14E-13
ZSCAN16	2,001681778	2,18E-09
HMGB2	1,999702614	8,38E-09
ZMYM5	1,999583969	3,65E-08
SLC2A11	1,999483096	1,33E-06
NUDT3	1,997100976	1,83E-09
ARF5	1,996222545	3,33E-29
ELL3	1,991516142	1,03E-05
EIF4EBP2	1,990456016	2,56E-11
SLC9A3R1	1,989675394	2,12E-11
DTNBP1	1,984899448	9,92E-10
RMND5B	1,980678414	9,94E-20
HMG20B	1,979176686	4,43E-25
KALRN	1,978022336	0,000543
NPDC1	1,976175774	3,09E-17
ICK	1,973683144	3,54E-15
DHTKD1	1,972929229	3,79E-17
ZNF256	1,971938439	4,82E-05
LOC10012S	1,969354773	2,23E-05
MIR600HG	1,968520079	9,58E-05
AP1S1	1,966155379	6,05E-09
KRT8	1,965918718	3,38E-09
FAM134B	1,965605506	0,000157
TP53TG1	1,96130137	7,02E-09
TRIM45	1,958884651	1,71E-06
NUDT4	1,956152564	5,55E-13
C21orf33	1,955421472	1,55E-08
TMEM183E	1,953409975	2,90E-07
RAB27A	1,953301386	8,14E-12
TNFRSF19	1,953128706	8,41E-14
HSD3B7	1,952242176	6,28E-11
RNF165	1,952194857	0,000775
FUT8	1,951261655	4,21E-19

GLB1L2	1,949216917	2,44E-11
ZCWPW1	1,948333534	8,66E-05
PROSER1	1,947822064	9,22E-23
ZNF76	1,938134154	1,89E-10
BRPF3	1,936434621	3,32E-18
SOX4	1,934144927	4,79E-18
PLOD3	1,930099499	6,26E-19
ZNF485	1,928448035	1,27E-05
RNASET2	1,92759738	4,50E-09
GRK6	1,925486463	4,77E-21
VPS9D1	1,922325273	0,000201
EDC3	1,918855488	8,55E-14
BCAR1	1,91722274	5,49E-08
MARC1	1,916812634	1,61E-09
ZNF3	1,913193773	1,01E-17
DGKZ	1,909376938	2,02E-16
PPM1L	1,909297441	7,63E-05
KDM5B	1,908905436	9,13E-11
GPSM2	1,906482236	4,76E-19
SIPA1L1	1,903112599	4,13E-20
ADRA2C	1,903008113	9,77E-11
BOLA1	1,902512432	3,90E-10
ADARB1	1,899644082	1,69E-07
SERINC5	1,89916434	4,91E-07
STK19	1,896951891	1,07E-05
CD82	1,892419838	0,000127
ONECUT2	1,887298321	1,52E-08
CORO1B	1,887277213	3,20E-07
MROH1	1,886707833	1,53E-10
ALKBH4	1,884332022	1,44E-11
PLXNA3	1,882523709	4,24E-10
PCSK6	1,881733471	4,40E-08
MAPK3	1,880373672	3,80E-08
ITPR2	1,879048752	2,24E-07
ASNA1	1,878493938	4,82E-25
MYL12A	1,875922621	1,17E-18
HDAC5	1,875710719	4,11E-06
SORT1	1,870722018	2,12E-11
KIFC1	1,869009839	2,30E-12
FGFR3	1,864847382	9,74E-06
CCNE2	1,861929936	6,25E-09
SUV420H2	1,860903247	1,83E-07
MREG	1,859043429	1,57E-11
SSBP3	1,857626133	1,97E-11
EFNA4	1,857483124	1,10E-09
SIRT5	1,853484434	4,30E-12
DYM	1,851863872	5,19E-19
GNB2	1,851837139	8,47E-20
TMEM168	1,851817005	1,25E-15
GTF3A	1,851758718	9,06E-24

CENPU	1,85150746	5,55E-09
ISYNA1	1,851484552	3,90E-15
ZNF687	1,848122997	2,42E-19
SPPL2A	1,847231008	4,12E-17
SEMA4C	1,846869219	2,34E-11
REEP6	1,838361156	2,03E-09
ZNF431	1,8351493	1,24E-08
MARCH9	1,8322987	7,83E-09
NRARP	1,831844088	0,000147
MB21D2	1,831516304	3,57E-07
SP5	1,830999802	2,95E-09
THNSL1	1,830628421	6,82E-14
SYDE2	1,830100863	0,000136
TMEM52	1,828826736	0,000114
SUMO3	1,828246272	3,15E-22
GCDH	1,819530643	1,82E-14
MAST1	1,816762973	0,000457
C19orf43	1,815028407	1,21E-22
SLC9A3R2	1,814251649	1,22E-14
NPIPB5	1,809802117	4,12E-06
DCP2	1,809301865	5,67E-12
TMEM183A	1,808905399	7,16E-17
ZKSCAN1	1,80872353	4,06E-17
FAM110A	1,8082731	4,87E-09
PSMG3-AS1	1,808220464	1,58E-05
NCOA3	1,802915535	5,04E-13
ZFYVE21	1,801419418	3,17E-13
PNPLA8	1,801231962	5,54E-05
TNPO2	1,80069351	1,16E-07
CACNB3	1,800340768	6,82E-12
TEAD2	1,799669952	1,27E-09
PRSS16	1,798172877	1,44E-05
ENSA	1,797371777	7,31E-23
SH3BP1	1,796736014	2,06E-06
GSE1	1,790330202	6,08E-21
IQCH-AS1	1,78727421	1,64E-06
FRS2	1,782430862	1,28E-08
CBX2	1,779989113	3,09E-16
TBX2	1,779776282	6,26E-09
SELENBP1	1,779519876	1,58E-08
RHBDF1	1,779131746	1,70E-11
CCDC64	1,77904904	1,02E-06
MGAT4A	1,778006486	4,58E-05
GMIP	1,777090586	3,50E-07
RALGDS	1,776349076	1,15E-12
PLEKHG5	1,772839345	0,000543
RNF168	1,771670924	1,03E-15
WDR53	1,771148437	3,38E-08
LOC220729	1,770089512	4,19E-05
A1BG-AS1	1,770018839	0,000117

DGCR6L	1,766406253	2,17E-15
TMEM134	1,763391585	3,73E-08
PLEKHN1	1,762791527	0,000296
SEC22B	1,762682296	7,59E-12
ZNF232	1,758428287	6,33E-05
IDNK	1,75815492	2,43E-05
SETDB1	1,75751223	1,85E-12
GLCCI1	1,753382935	7,42E-05
ZNF710	1,751546904	6,56E-12
ZNF713	1,749254334	3,01E-05
ZNF304	1,748869667	2,70E-09
C11orf80	1,747285269	2,40E-07
MAZ	1,746071445	1,11E-17
ZNF430	1,741171783	2,06E-06
TRAPPC2L	1,741150871	1,42E-13
TPD52L1	1,740747824	6,22E-07
MYO6	1,739450458	3,06E-19
EMP2	1,736198441	3,60E-08
MNX1-AS1	1,735842426	0,000109
ICAM3	1,734220139	2,97E-05
TRIM39	1,731996293	2,97E-06
ZNRF1	1,730510272	1,90E-17
THOC6	1,729949554	1,32E-06
VPS72	1,729803637	7,31E-09
CHPF	1,729666423	1,31E-05
PRADC1	1,729535501	1,72E-05
ATXN7L3B	1,728935661	1,80E-23
ADAM15	1,727495736	1,65E-16
PKMYT1	1,726812354	7,59E-11
TRPM7	1,725999479	2,07E-20
CIRBP	1,725658759	5,88E-07
GAS5	1,724330068	0,000261
ZNF714	1,721676024	4,13E-08
SMTN	1,721576215	9,68E-15
EFNA3	1,721390601	1,07E-07
UBL3	1,716095962	5,98E-09
FAM53B	1,715752579	1,15E-17
ZNF821	1,71529262	1,11E-05
PACSIN3	1,715036443	2,89E-14
ZNF74	1,714599705	4,61E-13
RFXAP	1,713774602	0,000544
RNF115	1,712985094	4,60E-16
SMARCD2	1,712130812	2,41E-21
TP53INP2	1,709874387	0,001011
GTF2IRD1	1,707533752	7,54E-13
TNRC6C	1,7031664	6,58E-13
ISOC2	1,701603929	6,50E-19
CPT1A	1,69533654	4,53E-17
DUS4L	1,693716874	6,88E-09
MEST	1,693193648	2,00E-20

ISOC1	1,69019797	6,33E-16
RAB11FIP4	1,689064617	1,69E-12
LPIN3	1,688756864	6,47E-08
PRUNE	1,688418196	4,88E-14
NELFCD	1,687419097	1,90E-14
MPND	1,68740221	1,34E-08
FBXO31	1,686775283	5,49E-15
TRIM62	1,6834757	1,61E-05
ZNF136	1,67548157	0,000607
BRCA2	1,674501036	1,80E-05
TCF19	1,674418639	1,74E-10
CUL7	1,673827848	8,16E-07
C1orf21	1,673703474	2,23E-05
DECR1	1,672889689	2,02E-13
USP20	1,6724634	1,07E-08
EPHB4	1,670927283	2,86E-17
SLC35B2	1,667550501	1,66E-09
POGZ	1,667143024	8,23E-09
FAM193B	1,66193578	1,20E-06
ZNF688	1,657502548	1,16E-05
PLA2G12A	1,657377491	4,71E-09
SLC24A1	1,656971304	1,18E-06
ZNF721	1,656293968	6,74E-12
KRBA1	1,656165537	7,04E-07
METTL4	1,653433247	5,36E-06
PFKL	1,651012236	3,76E-12
COBLL1	1,650509119	5,62E-07
PIAS2	1,649998602	1,20E-12
MED28	1,649138509	1,61E-10
CGREF1	1,648034211	5,56E-09
CD276	1,647255556	4,89E-09
GNAS	1,645752681	1,76E-20
NHP2	1,645181046	2,00E-19
MTL5	1,643349457	4,40E-05
FAM63A	1,642896889	0,000792
CASP6	1,642609486	2,72E-09
CCDC122	1,642002479	9,56E-06
RMDN1	1,640394445	2,57E-09
NFIX	1,639712691	1,56E-05
PPCDC	1,639383335	2,14E-06
ASNS	1,637675376	0,00089
POLR2J	1,637573017	9,36E-11
LLGL2	1,636721055	6,58E-13
HMGN2	1,635539635	3,89E-14
PI4K2B	1,63521932	6,97E-09
RBM8A	1,630721809	1,06E-17
EHMT2	1,629017055	6,08E-20
MXD3	1,626871587	5,05E-07
KCTD20	1,625766572	3,40E-17
SAYSD1	1,621477404	7,04E-11

LRIG1	1,619917165	4,12E-05
MRPL41	1,619589904	9,64E-12
SRD5A3	1,61927828	8,46E-09
ZSCAN21	1,613437443	7,64E-09
C7orf43	1,611506378	4,38E-07
ATP5D	1,610116583	1,64E-14
POLR3C	1,609579302	1,90E-05
ZNF778	1,606522712	1,01E-07
AP4M1	1,602051849	5,04E-09
C6orf47	1,601562584	5,61E-13
CABLES2	1,600706449	1,21E-08
ZNF316	1,599875337	7,46E-15
FAM50B	1,599422671	3,79E-07
CTNNBIP1	1,595070626	2,34E-06
PCDHB2	1,593719349	6,81E-07
GABBR1	1,591649593	0,000382
XXYLT1	1,591324993	1,01E-11
FGD4	1,589279306	3,04E-05
ZCCHC14	1,588959908	3,91E-13
PPP1R13L	1,587287603	2,12E-06
CDC42SE1	1,58709755	4,51E-12
XPOT	1,584767911	8,39E-08
ZFP69B	1,584624755	0,000848
CACFD1	1,584485323	2,87E-07
ARHGEF37	1,582564958	0,000759
ABHD16A	1,581651027	8,44E-12
PEX26	1,581114877	6,02E-13
DHCR7	1,58064779	3,88E-08
SLC37A4	1,580571867	1,24E-14
OARD1	1,578698518	4,51E-11
MEX3B	1,577584145	2,20E-10
LONRF1	1,577455651	0,000394
ANO8	1,576294846	7,11E-06
RMND5A	1,576177853	1,42E-11
DNAJC19	1,574044238	2,17E-07
DVL3	1,571408025	7,63E-17
ZNF273	1,570043918	2,17E-05
TRAPPC6A	1,568873042	1,34E-08
CDH24	1,564792197	2,18E-07
MOSPD3	1,56440619	3,67E-05
PXDN	1,563229916	9,97E-08
S100A13	1,559916047	1,03E-08
SLC25A39	1,557167681	2,20E-19
PPP2R3A	1,554300839	8,71E-06
CPSF4	1,554093231	8,07E-06
SEZ6L2	1,553268233	1,71E-06
ZNF680	1,552328991	6,45E-07
GLUD2	1,551671778	0,000186
ZNF14	1,550634924	0,000345
PHGDH	1,550268574	0,000607

APH1A	1,547307133	3,14E-14
LSS	1,545651124	4,45E-08
SLC25A1	1,542634464	1,84E-08
RAC3	1,542564919	6,82E-07
MEAF6	1,541441781	1,20E-06
CHD6	1,541406534	3,79E-16
RTKN2	1,540114043	6,18E-06
GADD45GII	1,538266018	4,00E-07
ILVBL	1,53813994	6,52E-12
DLD	1,536857008	7,15E-08
PNRC2	1,535884218	1,37E-07
FAM83H	1,534689606	9,40E-12
NEURL1B	1,533865705	9,58E-05
SUV420H1	1,529798533	7,62E-13
RALBP1	1,529448835	2,38E-09
VDR	1,524831516	0,000178
CC2D1A	1,523506488	2,18E-08
ZNF564	1,522852677	0,000317
FAM222A	1,519139863	7,44E-06
LMNB1	1,516926873	6,22E-12
SNRPE	1,51689948	6,00E-17
ITFG2	1,515330574	0,000303
MAN2B1	1,511690888	2,13E-09
GPRIN1	1,510967694	1,06E-10
AMDHD2	1,509398514	1,76E-07
KRT18	1,509038413	8,26E-05
SOX2	1,507993645	1,59E-05
ARMC10	1,50790238	1,19E-11
ATP5A1	1,506823099	1,93E-15
RPS18	1,506378397	1,82E-06
DHPS	1,505480155	2,80E-11
MEX3D	1,5002235	0,000138
EXO5	1,499307872	0,000375
TRIM24	1,499100123	3,81E-12
MYBL2	1,498712347	5,50E-17
TDG	1,498397888	3,31E-15
SCARB2	1,496975206	5,04E-10
VPS4B	1,495382286	1,07E-11
MDM4	1,494401962	4,22E-05
VTI1B	1,493281435	2,38E-08
RBM4	1,492151044	2,54E-11
ZMYM2	1,49159086	9,38E-13
AHR	1,491255485	3,38E-13
STUB1	1,490973096	4,25E-13
DDX49	1,489828277	4,73E-12
ZNF394	1,487037546	0,000118
ELOVL7	1,486604736	1,04E-07
PSMC2	1,485823376	8,39E-14
SRPK2	1,483649405	1,28E-13
ANXA6	1,48289837	0,000114

RAD23A	1,482881705	3,69E-13
SPATA2L	1,482599495	1,10E-07
MIF-AS1	1,477220568	3,49E-06
KIF20A	1,472185844	1,91E-08
ERMARD	1,472082843	0,000102
MAPK6	1,466932113	4,87E-15
ZFP1	1,466657922	1,69E-08
PATZ1	1,46415175	1,14E-06
GAS6	1,459227592	0,001008
TSC22D4	1,458642921	1,60E-05
ZNF212	1,458172363	3,17E-07
MVK	1,457409856	1,91E-05
APRT	1,457304254	4,04E-14
SLC9A2	1,456927794	1,33E-05
GTF2IP1	1,456906336	0,000191
NOP14-AS1	1,455006923	2,05E-05
TRIM26	1,454952296	3,92E-11
TMEM254	1,454756822	2,18E-07
KRIT1	1,454470565	7,86E-10
TSEN15	1,453535836	0,000169
AP4B1	1,451843142	1,78E-09
ARPC1A	1,451794612	1,42E-09
H2AFX	1,449411638	6,32E-15
SLC43A2	1,449370142	1,19E-05
PLEKHA7	1,448836189	2,71E-07
LRCH4	1,448584586	2,40E-10
ARHGAP19	1,448361522	7,38E-05
NDUFA13	1,447389317	5,31E-12
SSH3	1,44619659	7,84E-07
ZNF252P	1,44527264	7,14E-11
SLC12A9	1,443157099	0,000204
RGS14	1,442860957	0,000559
PLCG1	1,440821108	9,24E-12
PPP1R35	1,438258078	1,40E-06
FYTTD1	1,437557965	1,25E-07
PODXL2	1,436994812	1,35E-10
CHST15	1,436165079	2,53E-07
GPR89A	1,436070006	0,000108
PAK2	1,436007189	8,95E-13
DNLZ	1,434470285	7,24E-09
MMACHC	1,434191555	2,69E-05
PKP3	1,433945312	7,23E-07
PRICKLE2	1,433268687	0,000727
MSL2	1,432543345	0,000166
PCYOX1L	1,432092381	0,000106
CLOCK	1,43146896	6,21E-09
ZNF548	1,431333466	0,000315
DYNLL2	1,42727625	5,81E-11
CBLB	1,427018723	1,09E-07
TMEM63A	1,425624463	0,000422

ZNF544	1,424181033	2,63E-10
RPL35A	1,423720833	2,64E-08
SRF	1,421477963	5,82E-12
ARHGAP10	1,421385365	1,52E-07
ZNF791	1,421141182	1,19E-05
RPL17	1,419810186	9,06E-07
C22orf29	1,419550421	1,03E-06
ZNF197	1,419416889	6,84E-06
NADK2	1,417219056	1,84E-08
HDHD2	1,415168955	1,30E-05
BRWD1	1,415081233	1,16E-10
HAX1	1,414691744	8,97E-13
NCBP2	1,413793384	5,00E-15
CCDC88C	1,409609994	5,88E-05
LINC00998	1,409542146	0,000689
COPE	1,407072954	2,00E-12
PDRG1	1,406536095	0,000539
GALNT12	1,405813148	2,84E-06
RUSC1	1,40554405	9,23E-09
SH2B2	1,403857823	0,000191
HADH	1,402063527	1,47E-05
CLTB	1,399562372	4,52E-05
PEX1	1,397550073	1,09E-07
SETMAR	1,396776716	1,29E-07
MPV17L2	1,395449421	1,82E-08
ARRDC1	1,395104613	6,64E-09
TUFT1	1,393840115	0,000175
PCYT2	1,393564957	3,88E-06
HYOU1	1,392996346	1,76E-09
ATPAF1	1,392037207	8,50E-08
CCDC167	1,391283073	0,000522
ABCC5	1,391079386	6,25E-05
FAM21A	1,390501111	1,23E-10
CHTF18	1,390425183	5,69E-06
LSM4	1,389251548	3,86E-13
MRPL11	1,388854221	1,39E-11
PDCD10	1,387338292	1,51E-08
PACS2	1,386889225	8,21E-11
WWP1	1,386546961	0,00024
SLC2A4RG	1,385540877	4,12E-10
ZNF839	1,385339533	0,000233
TSPYL5	1,382372011	2,53E-13
MEA1	1,38069121	1,14E-12
GOLPH3L	1,380535644	0,000132
LHX4	1,380186587	2,36E-07
RNF44	1,378800519	3,40E-09
LYSMD2	1,378045555	1,26E-05
FBXW4	1,375797471	2,33E-07
SLC39A6	1,375544961	3,38E-06
ZNF675	1,375481501	7,18E-06

FKBPL	1,375433708	1,06E-05
CSK	1,372413992	7,28E-06
ARPC1B	1,372243989	1,27E-06
KCTD13	1,37204319	1,73E-06
ZNHIT1	1,370236208	0,000193
RING1	1,368451018	3,71E-09
MRPS18A	1,368089062	1,59E-09
RAB1B	1,367120495	4,42E-05
STK40	1,3668479	4,16E-05
BCL7C	1,365945725	1,09E-09
TET3	1,365793199	7,74E-12
VAV3	1,364991059	0,000167
PTPMT1	1,363474949	1,05E-11
MOAP1	1,362424717	5,42E-06
RNASEH2C	1,362312482	3,42E-05
TTC39C	1,362215911	8,71E-06
GCA	1,361513938	0,000219
NFKBIL1	1,361499892	5,69E-07
TET2	1,359102426	1,22E-08
NELFE	1,357306661	4,59E-13
FOXN3	1,35632151	0,00041
FDX1L	1,356061556	9,76E-07
RFWD2	1,35559882	5,37E-10
ATF6B	1,350480103	1,71E-08
TATDN1	1,347320716	3,92E-10
TMEM161/	1,344960811	1,70E-05
NSD1	1,344868831	1,20E-14
KRCC1	1,344624201	0,000128
GAB1	1,344521682	0,000237
ESD	1,343958655	2,53E-10
PPP1R3D	1,342998295	8,46E-05
FAM195A	1,342289513	8,64E-09
CELSR2	1,341404506	9,20E-09
MUT	1,340460901	3,27E-06
MTSS1L	1,33814895	7,92E-07
HSBP1L1	1,336514033	0,000169
ZNF276	1,33568763	1,12E-06
UCP2	1,33450853	9,13E-09
ARMCX6	1,333370127	9,15E-07
MTERF1	1,332538452	0,000162
RAB6A	1,330554047	7,41E-07
PTTG1IP	1,329608188	4,10E-13
PPP1R14B	1,32942841	9,91E-12
LTBP1	1,329241941	3,58E-08
TMEM265	1,328414736	7,71E-05
PIEZO1	1,326433502	6,49E-11
RHPN1	1,324128957	0,000374
SNX27	1,323184452	6,40E-10
TELO2	1,322931094	4,88E-10
NDRG2	1,322796653	0,000689

ZBTB10	1,322785426	2,08E-06
DBF4	1,322597314	3,87E-08
KIAA0226	1,320860061	3,34E-08
DHRS7B	1,320464047	2,89E-06
PIK3R2	1,319730103	2,44E-05
ARFGAP2	1,317534633	4,27E-10
DNPH1	1,31716321	1,17E-07
OBSL1	1,317048888	0,000107
ZNF607	1,316895139	4,77E-05
LRRC1	1,315523996	2,09E-06
TBCC	1,315232881	7,51E-06
TMC6	1,311848402	2,50E-05
LIMK2	1,309772778	0,000301
PREX1	1,309672775	3,06E-08
DCXR	1,30913244	1,44E-08
FBXO45	1,308930932	9,95E-07
ZNF398	1,308928075	3,39E-10
NFYA	1,307919507	1,18E-06
KARS	1,307147078	5,05E-11
CYBA	1,306881999	1,03E-07
RPRD2	1,305857044	4,82E-11
CBLL1	1,302740794	4,13E-09
AK4	1,301754914	1,09E-07
PBX2	1,300986672	2,22E-11
ADCK2	1,300044247	6,75E-06
IVD	1,297263479	4,24E-07
SEPT3	1,297102241	0,000522
NECAB3	1,296773849	9,20E-05
CSNK2B	1,296703391	6,03E-11
PRR14	1,296508692	6,53E-09
DXO	1,295497275	3,02E-05
HMGB3	1,295131999	2,93E-12
PYCARD	1,29427364	0,000972
INPPL1	1,291453401	6,34E-11
SF3B4	1,291175975	9,49E-14
RHOBTB3	1,291132315	4,64E-09
ATP6V0E2	1,290606575	0,000168
CYB5R1	1,290377781	0,000193
AFG3L2	1,290139581	9,69E-12
PSMD4	1,289669582	5,50E-14
OLFM2	1,289137464	8,38E-05
GRINA	1,28841561	0,000317
METRN	1,288320245	3,31E-07
DDX41	1,283601505	2,31E-10
SMDT1	1,282439894	0,000303
UBN2	1,282207352	2,81E-06
MSX2	1,281847223	0,000153
MFSD2A	1,280759781	0,000247
HSDL1	1,279222957	1,15E-05
C14orf80	1,279028678	2,98E-07

ZMIZ1	1,275762399	2,34E-06
AKT1	1,275356506	1,59E-06
RINT1	1,275128087	2,39E-05
PCCB	1,27473925	1,20E-08
NDUFB9	1,274300654	7,18E-07
CNPY4	1,273330797	0,000231
AARS2	1,270795924	3,13E-08
SPATA17	1,269527649	0,00015
DOCK6	1,267897957	1,45E-07
ZNF33B	1,267029159	0,000831
DDX20	1,266341751	1,24E-07
C7orf50	1,266179458	4,56E-08
SFI1	1,26334502	9,82E-05
PVRL1	1,262030075	0,000734
PGM2L1	1,259425001	2,38E-05
MMP17	1,258468103	0,000894
SATB2	1,257772583	0,000235
PUSL1	1,257383848	5,59E-05
GAMT	1,256803019	5,25E-05
IDI1	1,256089258	5,28E-05
ATP5L	1,255441832	1,43E-09
HSPB1	1,252741647	1,31E-06
MCCC1	1,251119072	0,000126
TCF25	1,250994998	3,60E-07
NUCKS1	1,249609097	4,23E-11
ZFHX3	1,24894895	6,34E-07
RPS25	1,248534913	1,98E-07
RFXANK	1,248043127	3,36E-05
PRKCZ	1,247876093	0,00011
CTIF	1,247813235	1,53E-05
TDP1	1,246565773	2,41E-08
TIGD6	1,244659418	0,000566
SPATA33	1,243879076	4,13E-06
WRB	1,243689064	1,64E-05
PSMB4	1,241995836	2,60E-08
AFG3L1P	1,240959384	2,72E-05
PRRC2A	1,239416696	2,77E-11
KBTBD7	1,238547041	0,000117
CNOT2	1,238211474	7,55E-11
CSRNP2	1,23696558	5,07E-07
RBM38	1,235210816	1,17E-08
TFAP2A	1,235103509	1,30E-11
JTB	1,234002921	1,92E-09
NES	1,233927999	0,000775
CMC2	1,23113776	1,59E-06
SARS	1,230144167	3,96E-05
PPP4C	1,228004257	3,46E-11
DNAL4	1,227678033	0,000392
BUD31	1,227111292	3,50E-11
CACNA1H	1,226862743	8,63E-07

GLTSCR1L	1,226236485	4,18E-06
COMTD1	1,224925185	1,29E-06
FNBP1	1,223741509	3,03E-08
UQCR10	1,222902625	1,39E-06
IFT46	1,222680215	1,25E-07
ZNF205	1,220845204	3,36E-05
TMBIM4	1,220298928	9,24E-06
CD47	1,219669471	8,42E-08
GGCT	1,218863864	6,32E-10
ZNF397	1,218799223	8,15E-05
ATXN7L1	1,21557037	9,12E-05
PRMT2	1,215329665	7,42E-05
NDST1	1,214180957	9,09E-07
ATMIN	1,213160866	5,57E-10
MDC1	1,213136372	1,34E-06
TROAP	1,213098126	0,000695
TMEM128	1,211810981	0,000951
MAPKAPK2	1,210288689	2,52E-10
BCL9	1,209110899	2,41E-06
TERF2IP	1,207556118	1,47E-07
MCUR1	1,207132891	2,28E-05
ZNF764	1,206348785	4,37E-05
FAM185A	1,206296536	0,000491
MTMR4	1,205934839	5,23E-09
ATP5F1	1,20547367	2,82E-08
CUX1	1,204324394	1,35E-08
PI4KB	1,20428892	6,76E-08
PEX10	1,203678774	3,44E-07
CUL9	1,202329053	8,33E-06
TCF3	1,201443832	1,43E-09
MAD2L1BP	1,201337244	3,96E-05
POLR3GL	1,20118375	0,000187
ANKS1A	1,199773705	7,49E-08
MARCKSL1	1,199185196	6,90E-12
PIAS3	1,198686118	4,83E-09
SMAGP	1,198683445	0,000198
RPL26L1	1,197919145	4,17E-06
MCM7	1,195742337	4,49E-08
STMN1	1,19571987	0,00042
NDUFB5	1,192804921	6,35E-05
SUPT20H	1,19218194	0,000816
SMG7	1,190925769	1,27E-11
SLC35B3	1,18879956	8,26E-05
SGPL1	1,188470235	1,30E-07
EFCAB11	1,18680129	0,000375
NUDT16L1	1,18649597	6,17E-06
HNRNPAB	1,185127431	8,30E-11
GPANK1	1,184877933	7,68E-05
SSR2	1,184003481	7,78E-06
TAMM41	1,183335761	0,00016

NACC1	1,181560852	9,51E-09
USP8	1,180279507	3,13E-10
RRP1	1,17758296	1,98E-05
PPP1R2	1,177566679	1,21E-06
MLYCD	1,175151717	0,000263
NR2F2	1,175032872	1,81E-06
PTGFRN	1,174139567	2,90E-08
C7orf73	1,173467606	1,34E-08
NDUFB7	1,172841436	5,40E-09
ROGDI	1,172838245	2,16E-05
POLR3B	1,170495679	6,42E-06
MED20	1,169858032	0,000715
ZBTB9	1,169541662	5,29E-06
RBPJ	1,16913058	4,94E-07
OTUD7B	1,169035602	1,06E-08
RP9	1,166816769	0,000411
PKNOX1	1,166542322	0,000151
GATAD1	1,166455083	0,000189
ZNF322	1,166401928	0,000321
RPS3	1,164176433	4,36E-06
DUS1L	1,16403822	3,59E-06
PAN3	1,16393559	0,000333
DNPEP	1,163917049	4,54E-09
TBC1D9B	1,162645182	1,76E-09
TMEM141	1,161899849	6,47E-07
LYSMD1	1,160564141	0,000171
AGAP3	1,15980204	1,31E-06
SLC25A10	1,159241746	6,09E-05
IMPACT	1,158336906	5,37E-06
C19orf52	1,158330637	0,000151
PPP6R2	1,157266332	0,000113
PDE7A	1,156816276	0,000335
PCGF3	1,153862926	2,69E-08
KMT2C	1,15331557	1,31E-06
ZNF23	1,153146829	0,000133
FGFR1OP	1,153122153	0,000343
PKN1	1,152140538	4,34E-08
BCYRN1	1,151441208	0,000565
MAML1	1,150689475	8,76E-06
COPG2	1,15061265	1,49E-05
RNF43	1,150290858	1,74E-06
ZNF786	1,148144434	1,45E-06
NBN	1,146904071	3,46E-09
TARS2	1,145255124	1,32E-05
AP5Z1	1,14399897	2,44E-05
ATP5I	1,143875958	4,25E-08
GINS2	1,143682143	5,09E-06
NT5DC2	1,142676274	1,06E-08
RPP40	1,142443441	4,16E-05
SLC25A13	1,140638757	1,23E-05

MRPS25	1,140115636	1,07E-07
NMRAL1	1,13927116	4,36E-06
STK38	1,13853745	1,63E-07
TOP1	1,138346762	2,42E-10
TMEM64	1,136272217	1,12E-08
SQLE	1,136054023	7,00E-07
ATP8B1	1,134950651	1,17E-05
UGCG	1,134413584	5,76E-05
SECISBP2	1,133166361	7,59E-07
ZNF652	1,133054565	9,82E-07
SEC11C	1,132357994	0,000483
RANBP9	1,13142984	9,08E-07
ACSF3	1,131053822	1,48E-05
MRPL9	1,129467536	1,57E-05
OSBPL10	1,12844989	4,74E-06
RPL18A	1,128253885	1,02E-06
ACTR6	1,128060701	1,33E-05
PTPN2	1,127065019	3,13E-07
LDOC1	1,12694928	0,000146
ATP1A1	1,126409818	8,89E-08
ZMYND8	1,124504321	4,25E-08
RHEB	1,124392373	3,92E-06
PSPC1	1,122984126	9,25E-06
EXD2	1,122651226	1,64E-07
SKP2	1,121591103	5,25E-07
ATP2C1	1,120950909	1,07E-07
CCDC112	1,120119649	0,00063
DNAJC2	1,119407708	4,10E-08
AP4E1	1,11821056	3,75E-05
CDC45	1,117538297	0,000189
ZNF480	1,115547498	9,21E-05
SHFM1	1,115160626	4,71E-09
BRD2	1,115046611	5,87E-05
PEX11B	1,114990132	0,000112
FARSA	1,114747629	1,30E-05
PELI3	1,114491441	0,00016
FLRT3	1,113144905	2,90E-06
HMGB1	1,112331665	6,19E-11
THAP5	1,111553799	2,04E-07
KLHDC2	1,108978553	0,000572
DNAAF3	1,108915707	0,000172
DBP	1,108336321	0,000559
USP38	1,108051613	0,000163
COX5B	1,105973344	6,87E-08
IMPDH1	1,10555886	1,39E-06
MIS18A	1,10480064	0,000252
ATXN7	1,103820807	4,52E-05
MRPL4	1,103819815	2,14E-08
WIBG	1,103432307	6,82E-05
SMARCB1	1,101651743	1,34E-06

WIZ	1,101206975	5,18E-09
SYNE2	1,101070863	0,000178
SLC39A7	1,100630461	4,90E-06
ZNF33A	1,100415014	1,77E-05
PRKAB2	1,09968928	0,000238
TMEM167B	1,098749485	0,000339
VGLL4	1,09833812	5,69E-06
RPL13	1,097489402	7,60E-06
SMAD1	1,097434095	4,62E-06
NCK2	1,09677507	6,18E-08
ADD3	1,096596529	1,03E-05
AKAP9	1,095950691	7,72E-06
B4GALT7	1,09532618	3,37E-05
NDUFC1	1,094850393	1,71E-05
BCR	1,094645054	0,000921
CTNNA1	1,094268657	1,04E-09
CRTC1	1,093656024	7,90E-05
RPL14	1,093646861	1,03E-07
ZNF32	1,092020292	8,02E-05
RPSAP58	1,091768226	1,94E-07
ALDH5A1	1,090738811	1,58E-05
IFT22	1,089917339	5,91E-07
PPP1R11	1,088822354	5,03E-06
TUG1	1,087498126	3,37E-08
CDYL	1,086534483	1,18E-06
SRGAP2	1,086077707	3,73E-08
PRELID1	1,084775886	4,13E-08
VPS9D1-AS	1,083308534	3,19E-05
CFDP1	1,082189954	7,01E-07
ANKRD50	1,080938807	9,67E-08
OSTC	1,079246391	2,47E-05
HCG18	1,078853097	5,68E-06
SERPINH1	1,077830955	8,31E-07
TRAPPC6B	1,077587503	2,38E-05
RPL21	1,076503034	4,49E-06
COX4I1	1,075809014	1,95E-07
FAM89B	1,074605238	2,17E-06
SURF1	1,074600897	0,000773
NCAPG2	1,073765915	9,24E-06
EBAG9	1,07349799	0,000216
CLIC1	1,072743497	1,27E-05
TOR3A	1,070720526	1,06E-07
ITFG3	1,070518678	0,000234
DBNDD1	1,070147442	1,72E-05
ZBTB1	1,069901216	2,38E-06
TPT1	1,06923943	4,82E-05
C6orf106	1,068864163	0,000391
MRPS18C	1,065812279	5,53E-05
MCL1	1,06310542	5,07E-09
RPL30	1,062982447	0,000305

POMP	1,062005961	0,000567
CNOT6	1,061430238	4,02E-06
COG6	1,060197208	0,00078
YIPF3	1,059718674	7,10E-06
SUMF1	1,057669319	0,000532
TMEM251	1,057234922	3,04E-05
TOMM6	1,054867143	1,31E-08
UBAC1	1,054522378	1,40E-06
VANGL1	1,053711803	5,96E-05
NUSAP1	1,051935535	0,000344
RAB24	1,051547917	0,000645
TRAK2	1,051343028	3,57E-06
HAUS1	1,050730581	0,00042
AIMP2	1,049948232	2,64E-07
ECSIT	1,048523649	7,35E-05
LAMTOR4	1,048409805	3,96E-06
CMTR1	1,043870241	7,13E-06
PNMA1	1,042964573	3,39E-05
NSMCE2	1,042626926	7,33E-05
NFE2L3	1,04259968	8,80E-05
STYXL1	1,042319998	0,000614
ECHS1	1,04133194	1,14E-06
STX16	1,040018376	0,001015
ZNF254	1,038999865	0,000192
SIVA1	1,038757111	5,15E-05
NR1D2	1,038616261	2,39E-05
PIAS4	1,037229354	0,000234
BAG6	1,036551579	4,19E-06
MRPL14	1,03619389	1,51E-06
SNRPC	1,033856456	1,34E-08
RMND1	1,032597687	4,04E-06
DDX6	1,030804467	8,00E-08
RPL21P28	1,03006454	0,000888
CCDC124	1,029242853	1,06E-05
GUK1	1,029166223	0,000566
ADAT1	1,028847673	6,47E-06
HIGD2A	1,026245761	4,05E-05
VPS45	1,025259961	1,17E-05
ZKSCAN5	1,023181628	4,05E-05
MAP1LC3B	1,023110535	0,000981
MARK3	1,023025165	0,000107
TRPM4	1,022282082	6,05E-05
FZD6	1,021761606	0,0002
ZNF282	1,020437996	2,92E-05
BSDC1	1,020300878	0,000295
SENP5	1,020232608	3,97E-06
SERP1	1,019574059	4,25E-08
UQCC2	1,019251745	0,000639
SAP130	1,018427699	4,27E-08
KLHDC4	1,018222055	2,09E-05

PPFIA3	1,018011416	1,16E-05
MBD1	1,016868111	1,24E-06
MICU2	1,016801537	1,35E-05
PDAP1	1,014627663	5,18E-07
ZC3H13	1,013481658	1,74E-05
TMEM241	1,012058014	0,000177
ZNRF3	1,010408598	1,29E-06
EPB41L4B	1,010117961	0,000268
POLR1D	1,009853906	5,60E-05
DAXX	1,009543472	2,31E-06
ATP13A1	1,007839709	1,58E-06
TMED10	1,007728796	6,44E-06
NUFIP1	1,007363168	3,69E-05
YWHAZ	1,005575909	2,38E-05
WBP4	1,005469051	0,000533
UPF1	1,004727353	3,52E-07
ARMT1	1,003823671	5,96E-06
CEP170B	1,002539932	5,71E-07
MRPS18B	0,998502838	7,59E-06
RAB13	0,997181138	0,000465
RAD21	0,996575065	2,60E-08
TMEM205	0,995701873	0,000454
NDUFAF6	0,995037298	0,000139
ZBTB41	0,994215905	5,92E-05
C7orf26	0,993938643	4,84E-06
SOS2	0,99307999	0,000195
ABT1	0,991735458	9,69E-05
NDUFAB1	0,990495297	0,000726
ERI2	0,989914346	0,000651
SDAD1	0,989518362	0,000189
RFC2	0,988676324	2,77E-07
TYRO3	0,988667055	0,000139
HARS2	0,988645261	8,78E-06
MKS1	0,988283957	0,000556
CSTB	0,987176659	1,57E-05
ZFAND2B	0,986947882	0,00031
KDM6B	0,985996517	0,00064
UBL7	0,983391584	5,01E-05
COPS6	0,982016107	4,41E-07
LSG1	0,9817954	1,12E-05
RAB22A	0,981517884	1,24E-06
ESYT2	0,980955806	1,80E-07
N4BP2L2	0,980477976	7,74E-05
PSMB1	0,980464144	1,28E-05
TUBA1A	0,978906213	7,71E-06
UBAP2L	0,97840977	0,000675
BAK1	0,977880797	1,32E-05
STX6	0,976915247	3,59E-06
CDK8	0,976584028	2,41E-05
TPX2	0,975682159	1,40E-06

SEH1L	0,975556971	7,60E-07
GATAD2A	0,975302694	1,39E-06
PUS7	0,974020476	0,000341
CBR4	0,971804775	0,000395
MIDN	0,971552548	2,11E-05
ANGEL1	0,970636458	1,90E-05
DCTN4	0,969057749	9,29E-06
SEPHS1	0,968560196	0,000189
ABCA2	0,968234681	0,000987
PSMC3	0,964506259	4,69E-08
PYCR1	0,964019677	0,000289
KAT5	0,962879713	8,47E-05
AP2M1	0,961901549	0,000239
ZFP90	0,961463646	2,37E-05
DIAPH1	0,960457504	0,000114
BTBD6	0,957401226	6,59E-05
C9orf114	0,95690856	0,000251
MRFAP1	0,956786866	1,63E-06
VPS11	0,953744955	0,000623
CARM1	0,951769374	3,08E-06
TRAPPC10	0,949928981	2,29E-05
VAPB	0,949717811	0,000106
ZNF768	0,949683058	3,21E-05
MTMR3	0,949611898	1,18E-05
GID8	0,948695055	5,55E-07
COX6A1	0,948684642	2,07E-06
RIPK1	0,948560697	0,000218
BLVRA	0,94795564	0,000669
RIOK3	0,947100471	0,00099
LEO1	0,946231	2,06E-06
LINC00674	0,945440408	0,000537
KMT2E	0,944547486	6,91E-05
GLRX5	0,944264267	9,98E-05
SLC7A2	0,943462408	0,000433
UBR2	0,941525047	4,69E-05
CHMP1A	0,940216151	0,00035
BCAP29	0,939196198	0,000137
SPIRE1	0,939101531	4,53E-05
WRNIP1	0,938826416	0,000292
ADRBK1	0,938738592	0,000115
SMG5	0,935979302	6,53E-06
SLC19A1	0,935053861	4,05E-05
SNX18	0,934819487	0,000713
LMAN2	0,934679054	2,75E-05
CARKD	0,933446013	0,000422
ZNF84	0,930932678	0,000263
CNOT4	0,930447279	0,000319
TACC3	0,929263403	0,000864
TRIM41	0,925449765	1,87E-05
SEPT8	0,924522623	0,000133

LARS	0,924081418	4,10E-05
PMPCB	0,924081011	2,75E-05
TAF1C	0,924030338	0,000183
DERL1	0,922536294	0,000433
PDCL3	0,920992217	0,000482
TIMM44	0,920599251	7,86E-05
DCAF16	0,919577484	0,000968
SHROOM3	0,914982732	0,000455
ACTR10	0,913115358	0,000204
C19orf60	0,912861641	0,000458
DOPEY2	0,908157338	0,000122
CYTH2	0,907903126	0,000458
SRSF9	0,907895927	7,52E-06
MRPL57	0,906299411	1,01E-05
PPP2R5E	0,905080083	3,66E-05
EVL	0,904220444	0,000296
NBPF15	0,904086233	0,000298
ALG5	0,902550088	0,000206
RBM17	0,90203211	0,000133
AGPAT3	0,901754575	0,000255
KDM2A	0,901386044	2,27E-05
PHKG2	0,900355471	0,000111
INTS3	0,897487682	2,91E-06
RNF130	0,897109471	0,000459
SMURF1	0,896457809	3,82E-05
HYAL2	0,896176614	0,00012
ACTR3B	0,895900379	0,000739
MDH2	0,894292754	6,22E-05
FKBP8	0,893353707	0,000956
CPNE3	0,893300452	2,60E-05
GPATCH2	0,892538454	0,000229
KIAA1522	0,892469878	1,10E-05
KLHL36	0,890167609	0,000314
NCBP2-AS2	0,884802337	0,000941
UFD1L	0,883794021	1,73E-05
WHSC1	0,882445505	8,54E-05
NDUFV3	0,882287438	1,72E-05
RABIF	0,879910787	0,000449
MTCH1	0,879749315	0,000146
STK11	0,877935311	4,15E-05
RTKN	0,877880573	0,000665
PYGO2	0,877648332	6,93E-05
SCFD1	0,87744508	5,22E-05
GCC1	0,876052046	0,000394
CDK2AP2	0,876012988	0,000136
ARF3	0,876003351	0,000102
STAG1	0,875327396	2,17E-05
SAMD1	0,875100121	0,00065
ACAT2	0,875020044	0,000129
OAZ1	0,873421511	2,80E-05

RPS2	0,87174412	9,28E-06
PPP4R3A	0,871680946	2,08E-05
MRPS10	0,870427129	5,19E-05
ASCC2	0,865116781	0,000214
FBXL19	0,864445223	0,000158
FOXP4	0,863297608	0,000668
RNF40	0,861531502	0,000129
ZKSCAN8	0,861027266	0,000403
SLC52A2	0,86017945	0,000125
PPIL1	0,85950481	6,37E-05
TRIM27	0,855866726	0,000756
BCKDK	0,855574476	0,000148
CEP63	0,855040343	0,000987
SLC3A2	0,854563366	1,30E-05
NUMA1	0,854166767	4,04E-05
LEMD2	0,853318497	5,59E-05
GAK	0,85285052	1,69E-05
CYP51A1	0,852361194	3,67E-05
CCNF	0,850995041	2,21E-05
LCMT1	0,85057831	0,000415
TMEM9	0,849765761	6,05E-05
ENOPH1	0,848839504	7,98E-05
C6orf89	0,847850643	0,000102
SURF2	0,847242629	0,000463
CHMP4B	0,847014018	5,26E-06
ZDHHC4	0,846156025	0,000226
FAM207A	0,844579125	0,000485
SNRPD3	0,844533583	7,09E-05
CDCA3	0,844360299	0,000344
GCSH	0,842373766	0,000502
GLCE	0,842067007	5,60E-05
BCL7A	0,841876264	0,000618
HDAC3	0,840965622	0,000198
STX7	0,840781998	0,000727
PPP1CA	0,840373003	2,91E-06
FBRS	0,838416744	5,18E-06
SEPHS2	0,836377555	9,14E-06
SS18	0,835434365	0,000143
STAG2	0,834391525	7,52E-06
TRIP11	0,833243118	0,000417
ACAA2	0,832668075	6,52E-05
LEMD3	0,831878167	0,00034
FAM120AC	0,831126757	0,000387
RPUSD1	0,82945637	0,00055
TBC1D10B	0,82939139	4,18E-05
ZMIZ2	0,827488368	1,21E-05
TSEN54	0,826103152	0,000904
FZR1	0,824762639	0,00042
GNAI3	0,82473303	0,000107
GNB2L1	0,822468617	6,09E-05

PSMD7	0,821862284	7,56E-06
CTBP1	0,82183206	9,16E-05
PRR7	0,819515917	0,000516
ARNT	0,819088037	7,25E-05
CDC5L	0,818474906	7,03E-05
PPT1	0,818150791	0,000958
RNF114	0,816576677	0,000128
ACTL6A	0,81613134	0,000143
MCM2	0,816011261	1,27E-05
EARS2	0,815443161	0,000433
FAHD1	0,812392831	0,000118
C16orf13	0,810848802	0,00011
TRAF4	0,810819216	1,72E-05
SPINT2	0,810289462	3,48E-05
TNIP2	0,808861043	0,000165
TRRAP	0,800889462	5,50E-05
H2AFZ	0,798790784	0,000379
NFRKB	0,798618621	0,00042
CAND1	0,798249984	0,000126
CANX	0,797503341	7,26E-05
DCAF15	0,795701557	0,00087
YIF1A	0,794204919	7,60E-05
MRPS21	0,792776662	8,26E-05
GNL1	0,791318506	0,000291
SREBF2	0,786336993	0,000988
COX7C	0,785818964	6,00E-05
ARMC6	0,784712772	0,000499
TAF6	0,784405578	0,000255
SAP18	0,780984561	0,00011
RAF1	0,778632373	0,00016
MAP2K2	0,777634661	0,000635
TMEM106(	0,776570553	0,000158
WDR73	0,775659744	0,000412
PABPC1	0,774858761	1,26E-05
PPIL2	0,773907784	0,000326
MRPL2	0,771813728	0,000259
CXXC1	0,771734521	0,000996
EPB41L5	0,771506803	0,000569
PANK3	0,771425473	0,000285
NOL7	0,768343986	0,000433
TEX2	0,766932347	0,000268
PPP2R5C	0,766648179	0,000313
TACO1	0,766160368	0,000108
DHX38	0,766155384	0,000289
NELFA	0,765467201	0,000672
TLCD1	0,765332077	0,00078
MORC2	0,764741168	6,53E-05
SLC25A24	0,764665629	0,000239
SMIM7	0,764186021	0,000576
KNOP1	0,763157198	0,00018

RAE1	0,760172001	0,000965
H3F3B	0,759582159	2,17E-05
RNF139	0,759162242	0,000285
SYPL1	0,75624972	0,001015
FDPS	0,755231544	5,08E-05
MRFAP1L1	0,755071296	0,000292
IMP4	0,75255653	6,38E-05
SNRPA1	0,751902976	0,000423
C14orf2	0,751875384	0,000775
PPIF	0,748698523	6,84E-05
SMCHD1	0,746357012	0,000658
RRP36	0,742786237	0,000167
CTBP2	0,740221571	0,000818
ZMYND11	0,738642855	0,000595
MTA2	0,73445575	5,80E-05
PTPN18	0,725963545	0,000943
CASP2	0,72439561	0,00035
RPL8	0,723706563	0,000122
CCNI	0,720473533	7,85E-05
LAPTM4B	0,718332101	0,000137
SMPD4	0,717232212	0,000153
CCNA2	0,714324714	0,000285
VARS	0,712443421	5,33E-05
YWHAH	0,709524699	0,000206
ANKIB1	0,709430201	0,000367
KMT2D	0,700916676	0,000674
AVL9	0,700433425	0,00031
ITPK1	0,697197182	0,000182
ADK	0,687662251	0,000786
YY1	0,687228966	0,000967
FBRSL1	0,685227611	0,000328
XPO5	0,681644693	0,000264
TADA3	0,679901	0,000916
PRCC	0,679370156	0,000437
GUCD1	0,677448256	0,000871
SLC44A1	0,674520283	0,000566
DLGAP5	0,671140577	0,000866
CENPN	0,670769492	0,00087
RBM15B	0,647169217	0,000486
BPTF	0,621311867	0,000864
ILF2	0,614464184	0,00073
YWHAQ	-0,615805196	0,00102
PSMD8	-0,618283043	0,000635
NAP1L4	-0,634832197	0,000885
AEN	-0,634931801	0,000861
RPL23	-0,638373365	0,000747
DNM1L	-0,63945966	0,000895
EBNA1BP2	-0,660055921	0,000735
NME4	-0,663290458	0,000321
WNK1	-0,664126607	0,000416

TMX2	-0,665984556	0,000751
DSG2	-0,66772735	0,000822
RBCK1	-0,672340416	0,000617
DCTN1	-0,676725379	0,000759
COPZ1	-0,679547019	0,000385
CHMP2B	-0,682598648	0,000859
PITPNA	-0,684886017	0,000965
UBQLN1	-0,689331975	0,000457
SRP68	-0,701757293	0,000284
NASP	-0,704239511	0,000172
TFAM	-0,707300232	0,000404
SLC31A1	-0,710547164	0,00035
COX7B	-0,7142806	0,000714
KIF4A	-0,714860174	0,000714
C11orf58	-0,725512534	9,72E-05
NCL	-0,726901273	2,48E-05
STK25	-0,729866019	0,000392
PPP1CB	-0,733117821	0,000693
FAM199X	-0,733450403	0,000353
DDX47	-0,733525111	0,000471
IPO5	-0,733705228	0,000221
PAFAH1B2	-0,734793018	0,000248
ESF1	-0,736833143	0,000369
POLRMT	-0,737051572	0,000459
MDN1	-0,738538154	0,000268
PFN2	-0,740538061	0,000105
PRPF8	-0,741955326	0,000509
ZFP91	-0,742185365	0,000536
BROX	-0,744335137	0,000791
RCN1	-0,748346929	0,000129
PUM2	-0,751028285	7,98E-05
GALNT1	-0,751453662	0,000101
XRN2	-0,753269517	0,000164
KPNA6	-0,755062063	0,000414
NSFL1C	-0,755244245	0,000472
PAFAH1B1	-0,757831769	6,03E-05
IKBKAP	-0,75890065	0,00036
SEPT7	-0,760150858	0,000237
NPTN	-0,763144396	0,000116
KIAA0232	-0,764107972	0,000582
CCT4	-0,764323019	1,05E-05
VEZF1	-0,764800912	0,000443
EXOSC2	-0,766717224	0,000291
DHX40	-0,76706401	8,06E-05
PHRF1	-0,767949693	5,33E-05
CCT6A	-0,770515707	2,84E-05
ICMT	-0,772835514	3,95E-05
RBM42	-0,773761013	0,00025
PHACTR4	-0,774559856	0,000132
LANCL1	-0,774951898	0,000355

ATXN2	-0,775669381	0,000618
PCGF5	-0,777940188	0,000402
DDHD2	-0,777982578	0,00089
DDX3X	-0,778193048	2,37E-05
ACBD3	-0,779070322	0,000266
SMAD2	-0,781112084	5,38E-05
CSNK2A2	-0,784748071	0,000361
VMA21	-0,784847754	0,000678
KIF3B	-0,785876021	0,001011
POLR2C	-0,785990469	7,34E-05
GRIPAP1	-0,786086481	0,000264
OGFOD1	-0,7861782	0,000218
TTC17	-0,786894397	0,000938
SHMT1	-0,788817501	0,000417
SUPT5H	-0,791259896	0,000454
CNOT1	-0,792881385	4,47E-05
KAT6A	-0,793473522	0,000243
CASC3	-0,794781895	0,000164
FAM83G	-0,795532151	0,000543
BAZ2A	-0,796483285	7,03E-05
EML1	-0,796561098	0,000491
IDH3A	-0,797048711	2,80E-05
AUP1	-0,797085886	6,93E-05
NUP98	-0,797223833	1,15E-05
ZZEF1	-0,798535317	0,000149
COPB1	-0,79903482	4,33E-05
ENTPD6	-0,799108593	6,35E-05
DDX55	-0,801208104	0,00036
SNHG16	-0,80128069	0,000238
POLD1	-0,801354623	0,000399
TRAF3IP1	-0,801777985	0,000631
CDK16	-0,802781964	1,49E-05
WBP11	-0,80310758	2,46E-05
CFLAR	-0,805557754	0,0002
TSR1	-0,805831967	6,11E-06
PFN1	-0,806419879	2,15E-06
ICE1	-0,810174627	0,000163
PTP4A2	-0,812685959	9,54E-05
PRPF4	-0,813589413	5,82E-05
IMPAD1	-0,814040967	3,67E-05
UBA1	-0,814148794	0,000502
CALM2	-0,815265537	7,27E-06
AKAP13	-0,816733811	3,52E-05
UQCRFS1	-0,817069918	1,11E-05
RSPRY1	-0,817760497	0,00032
NT5C3B	-0,820435218	8,17E-05
SMC5	-0,82110688	0,000696
PDCD5	-0,821293847	3,83E-05
MELK	-0,822071739	5,69E-05
SUPT6H	-0,822343879	0,000457

GLUD1	-0,8246912	1,16E-05
NAA35	-0,827230346	0,000333
SLC27A2	-0,828729111	0,000317
ECT2	-0,829093725	1,23E-05
NDEL1	-0,829384886	0,000474
ZYG11B	-0,82939773	0,00065
SLC25A22	-0,8298654	0,000642
FAM49B	-0,83029425	3,07E-05
PACSIN2	-0,831199145	0,000953
TMEM248	-0,831311231	0,000151
UBAC2	-0,833493191	0,000789
STRN	-0,833666351	7,98E-05
AATF	-0,833792543	5,66E-06
PROSER3	-0,83421459	0,000906
MRPS12	-0,835318672	0,00042
ERCC6L2	-0,835847658	0,000601
VPS37A	-0,836191151	0,000305
ANKS6	-0,836200772	0,000259
FOCAD	-0,838518424	0,000415
COA6	-0,838691128	0,000987
SMU1	-0,843020382	2,13E-05
TTC7A	-0,843199424	0,000609
NPLOC4	-0,843876538	6,44E-06
ALDH3A2	-0,844429871	7,43E-06
RHOF	-0,844964371	0,000135
NLGN2	-0,847520671	0,000776
CD151	-0,847943843	1,14E-05
ALS2	-0,84912157	0,000462
ARHGAP11	-0,852228568	0,000168
INTS7	-0,852580033	0,000133
RAPGEF6	-0,853446898	0,000412
FAM129B	-0,854375967	0,000112
WDR12	-0,85513409	2,70E-05
RNGTT	-0,855255788	0,000395
IER3	-0,855519298	0,000438
MIA3	-0,856712653	8,60E-05
C14orf119	-0,858468091	0,000835
NDUFA10	-0,858644395	4,68E-05
PSENEN	-0,859837187	0,00012
TBCA	-0,860963787	0,000241
RNASEH1	-0,861454912	7,13E-05
GCC2	-0,862195747	0,000968
BFAR	-0,864211793	2,93E-05
CTDNEP1	-0,865125621	5,08E-05
ATL2	-0,868738908	2,73E-05
NMT1	-0,869751094	1,58E-05
GIGYF2	-0,869972168	1,03E-05
STAT5B	-0,870507917	7,78E-05
HUWE1	-0,870826121	9,10E-07
TVP23B	-0,870974903	0,000369

HIAT1	-0,871258207	0,000178
PTP4A1	-0,872040859	1,00E-05
HMG20A	-0,872083756	0,000285
NCKAP1	-0,872768051	3,47E-05
TMED7	-0,873084074	6,52E-05
EFTUD2	-0,874155921	8,40E-05
FNDC3A	-0,874309281	0,000113
KIAA1551	-0,875052173	0,000545
PRKDC	-0,876494398	0,000193
GGH	-0,87693641	0,000931
SBNO1	-0,877148381	4,72E-05
IPO9	-0,878269061	0,000243
KMT2B	-0,878777647	1,97E-05
SNX9	-0,879130218	0,000392
TMEM181	-0,880549019	0,000582
EDEM2	-0,883189518	0,000383
PARD3	-0,883794256	7,35E-05
XPC	-0,884124908	7,42E-05
ZFAND6	-0,884296038	0,000138
EXTL3	-0,885060717	9,06E-05
RFC5	-0,885342173	0,00054
SLC35A2	-0,886260578	0,000411
POLR1E	-0,888268795	0,000226
STRAP	-0,888295394	7,88E-05
LINC01420	-0,888457904	0,000384
CD24	-0,888490001	0,000486
HEBP1	-0,888989377	0,000864
GOPC	-0,893928509	8,34E-05
ATP6V1B2	-0,89559927	1,89E-05
MTFMT	-0,895620119	0,000533
RECQL	-0,896619604	9,64E-05
SMCR8	-0,89776349	1,00E-05
COX15	-0,898197295	8,28E-06
MRPL44	-0,898493991	9,20E-05
CHUK	-0,901446522	0,000316
PSMA1	-0,902104356	6,46E-06
ZDHHC5	-0,902161803	1,38E-06
MRPS26	-0,902456259	0,000127
GEMIN7	-0,903976778	0,000955
PSMC4	-0,907342319	3,11E-07
SAAL1	-0,909573833	5,35E-05
FASTKD5	-0,910324897	0,000304
BECN1	-0,911934458	1,92E-05
SHB	-0,91215614	1,24E-05
PSMB3	-0,914548599	1,90E-06
GIT1	-0,920866187	1,34E-06
UBE2R2	-0,920976739	9,07E-06
MYCBP2	-0,921060609	0,000196
GNB5	-0,922431762	0,000195
PSEN2	-0,922725266	0,00056

OSBP	-0,92378496	2,93E-05
HMGA1	-0,927376175	2,59E-05
ARFGEF1	-0,933677864	6,68E-05
LYPLA1	-0,934288139	3,05E-05
MEN1	-0,935267564	3,20E-06
ARL14EP	-0,936651086	0,000321
DHX33	-0,938294384	2,07E-05
NPTXR	-0,938839121	0,000725
NUP85	-0,940974666	0,000448
USP24	-0,941037152	9,09E-05
PPP3CA	-0,945433384	7,59E-05
HIP1	-0,945714245	4,89E-06
CAD	-0,94665571	7,05E-05
GPATCH1	-0,946690618	0,000143
DDX21	-0,947908187	0,000116
CD81	-0,948330828	2,19E-07
TRIM44	-0,949707115	2,81E-07
RAB11FIP2	-0,949828484	0,000749
RAPGEF1	-0,950615564	0,00014
VSIG10	-0,9516154	0,000132
AGPAT5	-0,951918518	4,70E-05
DDX51	-0,95271156	1,51E-05
DNAJC16	-0,953266068	0,00098
TSG101	-0,953845862	0,000133
NCOR1	-0,954431054	2,36E-07
SOAT1	-0,956204876	0,000483
ARIH2	-0,957541547	6,15E-06
TTL	-0,959640381	5,69E-05
TRAM2	-0,959880085	2,96E-06
NAT14	-0,961968843	0,00099
NSMAF	-0,962437367	1,63E-05
ARID1B	-0,962600144	4,58E-05
MYH10	-0,963437287	1,60E-06
TMCC1	-0,963454774	4,58E-05
NOC4L	-0,965301199	1,07E-05
KIAA1143	-0,967617616	0,000794
CTNS	-0,96792933	0,000473
TIMM50	-0,968765763	1,34E-07
ATM	-0,969248206	0,00023
DDX17	-0,969300907	3,35E-06
CYP20A1	-0,970921043	8,50E-05
CRLS1	-0,971055194	5,70E-05
MAGT1	-0,971448421	1,31E-06
RPS16	-0,971968571	0,000323
SUZ12	-0,972038494	5,25E-06
URB2	-0,972289412	3,12E-05
TRIM32	-0,972941801	6,75E-05
SLC16A1	-0,974071835	1,08E-05
API5	-0,974100191	4,82E-06
SNTB2	-0,975221555	0,000264

AMD1	-0,975439816	0,000103
LAPTM4A	-0,975639745	4,41E-07
PHF8	-0,975756268	0,000133
LEPROT	-0,975805316	0,000142
FN3KRP	-0,977579542	1,85E-05
CARS2	-0,978518928	7,37E-05
TRAF3	-0,980217977	1,64E-05
TOMM20	-0,980380532	1,14E-05
PIN4	-0,981487059	0,000168
AMOTL1	-0,981493835	3,41E-05
UTP18	-0,981895205	6,37E-06
BOP1	-0,983072394	5,77E-08
MRPS27	-0,983270727	4,62E-07
OPA3	-0,984943648	0,000129
PQBP1	-0,98564126	0,000124
TRMT61B	-0,986619958	0,00049
ZNF503	-0,987259276	1,30E-06
PSMD11	-0,987509265	2,17E-07
ARAF	-0,987946494	0,00011
KIAA1033	-0,988058056	4,57E-05
SH3PXD2B	-0,988633495	9,16E-05
EMC9	-0,988769695	0,000823
WIPF2	-0,989305418	4,26E-06
TYW3	-0,989478355	3,38E-05
ACTR5	-0,990709795	0,000625
AR	-0,990779535	0,000111
CNP	-0,991471958	2,86E-05
PSMD1	-0,992533084	4,32E-06
NFU1	-0,992758512	0,000245
DESI2	-0,993242811	2,02E-05
ZFR	-0,996598889	0,000656
NXN	-0,99678682	0,000609
GTF2A2	-0,997653635	5,49E-06
WDR44	-0,998186096	0,000471
EIF3M	-0,998315551	9,93E-07
MYEOV2	-0,998949847	4,38E-05
ARL3	-0,999285052	1,01E-05
AFF1	-1,000010125	4,71E-05
GEMIN4	-1,00129503	1,82E-07
NR3C1	-1,001723987	2,52E-06
SLC7A11	-1,003252356	0,000913
SERTAD2	-1,003758802	5,12E-06
PPP1R9B	-1,004491898	7,69E-08
GSK3A	-1,004873239	4,22E-07
DDX10	-1,006089324	8,25E-07
COL18A1	-1,006182757	9,48E-08
URI1	-1,006751858	2,79E-08
LINC00094	-1,008224419	0,00035
CEBPZOS	-1,010050983	9,17E-05
EPT1	-1,010660393	6,46E-05

NSMF	-1,010732407	1,02E-06
MPP6	-1,011804262	8,29E-05
CASP8	-1,012402548	6,24E-05
KATNB1	-1,012467591	0,000288
RASAL2	-1,012849322	6,31E-05
TNKS	-1,013521212	7,18E-06
PCM1	-1,015059839	8,09E-07
LONP2	-1,015298563	1,78E-06
WDR43	-1,017141392	5,05E-05
RAB9A	-1,017589019	0,000336
ARHGEF1	-1,01866925	1,82E-07
EMC7	-1,019027358	0,000213
QSER1	-1,019694769	5,95E-07
CCDC34	-1,02176366	5,29E-05
EXOC8	-1,022678178	0,00011
MPZL1	-1,022900067	5,74E-08
CCDC86	-1,023136585	2,36E-08
VRK2	-1,023371766	8,28E-05
LSM14A	-1,023782496	7,80E-05
SIX5	-1,023960808	0,000707
MED8	-1,024924876	0,000343
MAP2K4	-1,024951063	6,41E-05
ALCAM	-1,025613357	8,52E-05
PLEKHA3	-1,026318164	0,00089
LGALSL	-1,026497198	0,000665
MBOAT2	-1,026613296	0,000289
RIF1	-1,02819853	5,85E-05
LMF2	-1,028523167	0,000292
STXBP5	-1,028700146	0,000679
TTC31	-1,029182582	0,000513
PTMS	-1,030021053	8,02E-05
CSPP1	-1,031778341	0,000722
PPP1R12B	-1,032519884	0,000204
TMEM261	-1,032830149	1,29E-05
NSL1	-1,033669236	2,55E-05
PTPN21	-1,03479245	0,000849
CLTA	-1,035548909	1,08E-05
TWISTNB	-1,035600353	2,84E-05
TACC1	-1,036042309	1,24E-06
PDDC1	-1,037711388	0,000398
SSX2IP	-1,039342246	1,62E-05
CYFIP1	-1,040108986	8,94E-08
VPS54	-1,04093028	0,000362
METTL16	-1,042745966	4,68E-07
PLCB4	-1,044527426	0,00089
PPTC7	-1,047530283	5,96E-05
PPP2CB	-1,048361575	1,36E-06
ERC1	-1,051552367	5,99E-08
TBCB	-1,052750429	4,18E-07
WDFY1	-1,055466697	1,72E-06

ELOVL5	-1,056419794	2,90E-08
SLFN5	-1,056498081	0,00088
SPOP	-1,056832821	3,83E-05
FAR1	-1,057031063	1,55E-05
MOB1A	-1,059086523	9,20E-06
ATP11A	-1,060514288	0,000229
ALAS1	-1,062409895	0,000125
CORO1C	-1,063084638	5,20E-07
MIER1	-1,063473221	4,10E-06
NFKBIB	-1,063757202	0,000724
ACLY	-1,063985657	1,64E-05
ABR	-1,064677571	8,27E-07
DRAM1	-1,065014602	0,000653
RGP1	-1,065265908	0,000385
PRCP	-1,0664224	2,32E-05
TACC2	-1,06645684	1,11E-05
C2orf44	-1,066538765	0,000745
TSKU	-1,067563	2,24E-05
BAG4	-1,067803093	6,02E-06
BIRC6	-1,068506068	3,66E-05
TOMM5	-1,068672589	6,13E-07
OCIAD2	-1,069156795	0,000213
MACF1	-1,069232761	1,87E-07
RAI1	-1,069868344	0,000722
COX11	-1,070821744	0,000491
KDM6A	-1,071599148	0,000899
MED1	-1,072098144	1,08E-08
TUBG2	-1,072240695	0,000955
ELP3	-1,073153586	7,14E-07
ARL13B	-1,073428719	1,40E-05
MBOAT7	-1,077396524	1,81E-05
SLC25A6	-1,077553318	2,40E-06
SNRPB	-1,079361031	4,24E-05
TMEM147	-1,079942296	4,70E-06
EFR3B	-1,080277758	6,44E-06
PCYOX1	-1,08067963	5,05E-05
TALDO1	-1,084293032	1,90E-06
CRTAP	-1,084605514	1,74E-06
SPATA20	-1,085405376	0,000746
MPRIP	-1,086087729	1,30E-06
NGLY1	-1,089188588	5,95E-05
PNO1	-1,091792187	0,000503
LOC100506	-1,091845339	0,000181
RAB10	-1,094745941	7,60E-09
CAB39	-1,095121417	6,65E-08
URB1	-1,096247207	4,26E-07
SLC30A1	-1,096327794	0,000148
AHCTF1	-1,098039817	3,15E-05
MRPL10	-1,098417798	4,62E-06
NETO2	-1,101382072	1,09E-08

ACAT1	-1,101654076	0,000954
CYB5D1	-1,102173077	8,76E-06
SCRN1	-1,105876722	4,46E-09
HIF1A	-1,108083751	1,05E-08
SLC35F6	-1,10983898	1,14E-05
HR	-1,110553022	0,000878
ADRBK2	-1,110694592	5,07E-05
EIF2B4	-1,112901598	6,76E-08
MRPL33	-1,113287208	4,06E-05
PPME1	-1,113610524	1,10E-05
NLE1	-1,114329299	0,000244
SH3D19	-1,115759703	6,20E-07
MYO19	-1,116765917	1,31E-05
ABHD17B	-1,117013236	3,51E-05
TAOK1	-1,117081754	3,46E-09
MKNK2	-1,117425358	3,01E-06
MSL3	-1,117740161	4,32E-06
UAP1L1	-1,118275799	0,000915
CALCOCO2	-1,11883913	1,26E-05
AGFG1	-1,119112694	5,45E-07
KATNBL1	-1,12032325	5,99E-05
BICD2	-1,120564505	3,27E-08
RETSAT	-1,121216366	9,65E-06
GBE1	-1,1213136	0,000493
AIDA	-1,121850873	0,000148
NME1	-1,121977403	7,36E-08
KCTD9	-1,123776027	2,33E-06
ADAM17	-1,124235469	3,31E-07
DCDC2	-1,124309486	7,47E-05
ANKFY1	-1,126720307	4,34E-08
CCDC25	-1,127925075	7,75E-07
COPS7B	-1,12819863	4,82E-06
HIATL1	-1,128978517	5,30E-07
FTSJ1	-1,129468107	5,39E-08
PLEKHA5	-1,12959734	1,97E-05
BOD1L1	-1,130463868	3,06E-09
ELOVL2	-1,130507211	0,000348
SGCB	-1,132193529	1,38E-05
NEK2	-1,133864983	1,87E-05
SUCLA2	-1,134869858	4,13E-05
NOL6	-1,136856213	9,95E-06
ZBED5	-1,138800191	1,26E-05
IARS2	-1,139816994	6,07E-10
PTPN13	-1,140617023	1,01E-05
NBAS	-1,14075191	2,28E-06
CDC27	-1,141040651	1,08E-09
NUP43	-1,141289195	4,52E-05
ERICH1	-1,142417993	1,25E-05
ITPA	-1,14401148	6,45E-06
POFUT1	-1,14440891	1,19E-06

FAM8A1	-1,144442831	5,26E-05
RAB11FIP1	-1,146036358	6,01E-08
ZNF343	-1,14605633	2,49E-05
PYGB	-1,147363638	8,56E-11
TMEM51	-1,148324218	2,89E-05
BET1L	-1,149494438	4,01E-06
ADSS	-1,149737979	8,20E-07
SUV39H1	-1,150492619	7,68E-06
TMEM123	-1,151152353	7,11E-11
LRRFIP1	-1,152386794	3,08E-09
FOPNL	-1,153444225	6,13E-07
RNF144A	-1,154961718	0,000356
PNPO	-1,155983013	1,41E-08
JADE2	-1,156338038	9,33E-06
CSTF3	-1,156491541	3,32E-05
BAX	-1,158095509	0,000114
CKB	-1,158139239	1,86E-05
LENG8	-1,159152681	4,11E-07
KLHL2	-1,160232883	0,000717
OTUD5	-1,160402855	1,76E-06
COPS8	-1,161161191	7,21E-07
EBLN3	-1,161211806	1,27E-07
LOC101927	-1,161659161	0,000556
LAMA3	-1,161668798	0,000396
TSPAN3	-1,162147774	1,83E-07
DTX2	-1,162956188	6,53E-05
BNIP2	-1,163365868	6,60E-06
FAM160B2	-1,164002386	1,30E-06
TSNAX	-1,16510223	4,21E-06
NONO	-1,16536242	4,02E-07
SPAST	-1,166436281	1,61E-07
TGOLN2	-1,166590085	9,85E-08
LRFN1	-1,166623403	0,000151
MAP4K3	-1,167414742	1,07E-06
KIF21A	-1,168158704	6,90E-09
RAB3GAP2	-1,169673802	6,85E-08
PRKAA1	-1,170493248	5,94E-05
GINS4	-1,170784626	2,28E-07
TRMT6	-1,172460951	3,34E-08
ARRB2	-1,173133414	9,43E-10
DYRK3	-1,177515895	0,000906
GPI	-1,178565861	3,22E-06
ABCD3	-1,179450933	1,59E-07
ERO1A	-1,179745433	3,86E-08
FAM195B	-1,180104079	0,000591
SPIN3	-1,18022972	8,77E-05
MINPP1	-1,180254251	7,31E-06
RAB36	-1,181824237	0,000689
ACTN4	-1,183274551	5,07E-06
TNS2	-1,183391421	3,27E-07

PPAP2C	-1,183514212	8,46E-07
DTL	-1,184133021	1,92E-07
NOL4L	-1,185962175	1,56E-06
MRPL45	-1,186296325	1,22E-06
LRPPRC	-1,18759387	7,30E-12
UBA2	-1,189463335	4,03E-08
CAPNS1	-1,192363415	0,000386
DIS3L	-1,19334	1,48E-05
ZCCHC7	-1,193497361	8,59E-05
MRM1	-1,193933166	6,41E-05
ZBED1	-1,194679045	3,77E-06
CPNE1	-1,195506241	3,31E-09
SEC14L1	-1,19703044	5,18E-07
NUDT19	-1,197392704	1,58E-06
TFB2M	-1,199168344	0,000226
SLC25A37	-1,200954737	0,000103
PTPN3	-1,201449226	4,43E-06
TANGO6	-1,201517903	5,22E-05
SGPP1	-1,202817071	7,66E-06
RPTOR	-1,203013218	2,00E-10
GNL3L	-1,203117871	1,32E-08
DHX37	-1,20315065	5,62E-11
BAG2	-1,205156879	1,77E-06
NSRP1	-1,205401647	2,44E-07
TLN1	-1,206620801	6,99E-09
CKAP4	-1,208286224	2,36E-11
SHPRH	-1,209580904	0,000671
PRDX3	-1,211954878	2,73E-07
MBTPS2	-1,213316986	8,70E-07
LRRC14	-1,213462003	1,44E-06
PAPD5	-1,21374678	2,78E-07
LTA4H	-1,213803615	2,76E-07
NUP133	-1,214019326	2,40E-08
THTPA	-1,214141856	0,000479
CSNK2A1	-1,214264815	3,67E-09
TEX261	-1,214530715	3,03E-10
ATE1	-1,21490063	6,09E-07
CAST	-1,21540062	1,21E-08
KDM5C	-1,215586439	9,33E-11
PTPRA	-1,216522558	5,05E-06
RIC1	-1,21657255	8,01E-05
MCU	-1,2166589	2,31E-06
SSH1	-1,217253141	3,19E-07
BDP1	-1,218602223	1,70E-06
SLC5A3	-1,218722255	2,39E-06
SAMD8	-1,218894717	1,74E-05
MAP4K2	-1,219160451	0,000244
VKORC1L1	-1,221612361	4,31E-10
TYMS	-1,223145393	4,67E-09
PECR	-1,223437984	0,000231

MEGF8	-1,223457007	2,07E-08
PI4K2A	-1,223667443	3,32E-05
LGALS8	-1,223747657	2,07E-05
NBPF1	-1,224464224	4,97E-06
FSCN1	-1,225004284	6,07E-09
ERCC6L	-1,226293864	3,68E-07
NFIA	-1,227272423	7,51E-06
GEMIN6	-1,227395288	1,81E-06
GPX8	-1,229341963	2,53E-06
TOP2A	-1,232094367	8,81E-06
MTRF1L	-1,232415763	8,50E-07
STK17A	-1,23294858	5,80E-05
PEAK1	-1,234483627	3,62E-07
C9orf3	-1,234933863	1,52E-05
FOXN2	-1,235047278	0,000137
C10orf32	-1,236113483	0,00023
EIF2AK4	-1,237244588	4,18E-09
EPS8L2	-1,237386504	0,000223
ITSN2	-1,239765427	4,98E-06
AEBP2	-1,239783868	7,14E-06
TSHZ1	-1,241781809	0,000182
SLC20A1	-1,244640193	9,92E-06
TERF2	-1,24526716	6,86E-07
BBIP1	-1,249163428	2,37E-06
GALNT7	-1,253062404	0,000386
QSOX2	-1,254710239	4,43E-05
ENTPD4	-1,254726919	2,31E-07
CDK12	-1,254889041	2,28E-11
MTRR	-1,255051892	0,000447
HAUS6	-1,257324737	3,49E-08
PTPRG	-1,257780221	5,05E-06
IBTK	-1,25827348	4,05E-10
DYNC2H1	-1,258714221	1,28E-05
LDAH	-1,259425834	2,00E-06
ZNF580	-1,26170559	1,52E-06
MGST3	-1,262802777	0,000276
MC1R	-1,264580406	0,000135
THADA	-1,267595125	1,07E-09
FZD5	-1,26924959	6,63E-07
MED29	-1,269503733	2,85E-11
GNPAT	-1,270048001	8,84E-10
RABAC1	-1,27023887	1,49E-05
FTO	-1,271596154	4,61E-08
UBE2G1	-1,273481237	6,45E-08
KIF18A	-1,273923323	8,48E-06
PKM	-1,27411376	2,21E-09
LRRC37BP1	-1,274569401	9,85E-05
ACOT9	-1,278913007	1,69E-06
CARD8	-1,278982945	1,96E-05
ICE2	-1,282501765	2,17E-09

POMZP3	-1,28272373	5,10E-05
CES2	-1,283725415	5,66E-07
METTL17	-1,285659637	0,00038
PHLDB3	-1,285950741	0,000989
TWF2	-1,286384427	1,31E-09
SMYD3	-1,287420813	0,000461
SHKBP1	-1,287528589	5,21E-06
PXDC1	-1,289558867	0,000544
JMJD6	-1,290434067	0,000147
LIN7C	-1,291578527	2,78E-05
IFNAR2	-1,293968112	4,91E-05
PPFIBP1	-1,294484735	5,21E-08
PIGW	-1,295020401	2,52E-05
B4GALT4	-1,295310226	2,29E-07
SHCBP1	-1,295765493	1,60E-08
MZF1	-1,300083313	6,83E-05
SHC1	-1,300432321	1,72E-13
IFNGR2	-1,301684994	0,000129
POLR3D	-1,301906159	1,64E-05
UTP6	-1,302222977	1,65E-10
SLC41A2	-1,30404372	0,000162
ANXA11	-1,304197122	3,11E-09
ABHD2	-1,305272289	2,03E-06
ATRN	-1,308671936	3,05E-08
APEX2	-1,310622917	1,15E-06
ADCK3	-1,310982839	8,31E-05
INHBB	-1,311392358	0,000419
ESCO2	-1,312463963	1,08E-06
DUSP3	-1,313178251	4,13E-09
BTBD3	-1,316207615	2,52E-07
COQ10A	-1,316495818	0,00016
ARHGAP12	-1,317191542	1,30E-05
PDHX	-1,319735649	9,12E-08
TAF1	-1,321558029	1,01E-06
TNFRSF10A	-1,32159718	0,000186
RNF111	-1,32404279	2,00E-10
RPGRIP1L	-1,324297851	7,65E-06
MT1X	-1,324648172	5,98E-05
STARD3NL	-1,325819009	0,000145
CNEP1R1	-1,326371193	0,000186
BBX	-1,326874157	1,53E-07
IGF2BP2	-1,327009075	1,92E-05
UTRN	-1,32806868	6,97E-05
TPP1	-1,331259065	2,54E-06
FKBP1A	-1,332275149	4,79E-09
RBFOX2	-1,334585622	7,28E-12
IKBKB	-1,335375736	3,28E-06
NF1	-1,335741889	5,74E-07
PGK1	-1,336531487	1,55E-11
ASB1	-1,33732112	5,18E-09

LACTB2	-1,339566221	1,51E-05
TBCE	-1,339665437	1,14E-07
AKAP10	-1,340209741	3,16E-05
SPAG9	-1,341824098	7,91E-08
TMEM245	-1,34221185	3,74E-09
MAPKBP1	-1,342606243	0,000772
CEP89	-1,345968229	1,86E-06
CYSTM1	-1,347039792	1,06E-09
PKI55	-1,34961384	0,000169
ACADVL	-1,352201655	1,52E-06
AGFG2	-1,352684056	9,42E-06
MOSPD2	-1,353189008	5,88E-05
LAMA5	-1,356441787	6,37E-15
RHOT1	-1,357054796	5,80E-10
ERI1	-1,359339727	1,30E-09
APP	-1,359366211	4,16E-15
IGBP1	-1,359602392	3,70E-06
RPL23AP53	-1,360649177	0,000739
GLA	-1,360831677	0,000549
TOM1L2	-1,360921601	5,47E-06
RPS6KA3	-1,361261819	1,39E-11
PDLIM1	-1,363025391	5,67E-12
PSD3	-1,363771036	1,36E-08
RICTOR	-1,36428351	1,78E-06
RAB2B	-1,364328239	1,03E-06
RASSF3	-1,364872111	1,14E-06
MGST1	-1,36633906	6,00E-08
BCL2L2	-1,370758213	2,29E-06
USP28	-1,371660943	7,69E-08
EML4	-1,371789409	5,49E-15
BLMH	-1,372644713	7,15E-11
LRRC49	-1,372982986	1,92E-06
HDHD1	-1,374248065	3,12E-05
SLC41A1	-1,375894505	8,66E-09
SPATS2L	-1,376145764	4,65E-09
PDE8A	-1,377519818	9,97E-08
ENTHD2	-1,379353129	0,000529
CLU	-1,383154096	0,000315
NIPA2	-1,383264638	3,71E-08
TMEM2	-1,383445011	2,27E-08
ATP7B	-1,388962466	0,000225
MID1IP1	-1,389753438	0,000131
MLLT3	-1,390747819	1,12E-06
PTAR1	-1,391030824	3,29E-08
SH3BP4	-1,393586145	4,24E-08
FCHO2	-1,394074007	0,000102
DENND4C	-1,39408203	1,17E-09
ARV1	-1,394842008	3,11E-06
PCNXL2	-1,394848821	3,59E-07
PHC1	-1,395964869	1,15E-07

CHMP2A	-1,397628083	0,000274
PNKD	-1,401064775	7,57E-07
CHST14	-1,402840259	6,80E-06
ETV6	-1,405125414	4,99E-08
ATP5SL	-1,405411772	3,20E-09
RHBDF2	-1,406309125	1,23E-10
HPS5	-1,406713364	1,49E-08
AP2A2	-1,407393177	1,54E-11
CNST	-1,408195042	0,000118
STK4	-1,408418299	1,44E-12
ETAA1	-1,408550795	2,90E-06
PBK	-1,410033579	6,01E-06
NOP10	-1,414210642	1,23E-13
GPKOW	-1,415063042	1,73E-06
KYNU	-1,415853348	0,000316
SOCS5	-1,416202295	3,54E-07
EFR3A	-1,419042202	2,07E-05
TMEM164	-1,419130624	3,96E-12
WWC2	-1,419302461	8,75E-07
ARMC9	-1,419400941	4,00E-08
B9D2	-1,420991266	8,39E-05
EXOC6B	-1,42107586	1,34E-06
PLEK2	-1,422621055	2,53E-05
PRICKLE3	-1,422706101	2,27E-06
TBC1D25	-1,422829785	0,000702
FUZ	-1,423860434	9,21E-05
ERCC1	-1,424267765	1,97E-11
FLVCR1	-1,42446126	9,12E-05
LCLAT1	-1,427675049	4,83E-11
PRMT3	-1,428505386	1,23E-07
CDC42BPA	-1,429958218	1,30E-06
PLEKHG2	-1,430839343	1,79E-10
MAP7D1	-1,431741322	2,54E-08
IPO7	-1,432350967	7,84E-10
ZNF584	-1,432536418	1,72E-08
CD59	-1,433286177	0,000137
ZNF780A	-1,433813142	3,08E-08
SEPN1	-1,435871852	2,59E-14
RHOBTB2	-1,435934227	8,36E-06
FLNA	-1,438116956	5,78E-11
RAI14	-1,438270988	2,06E-07
RTN4	-1,444137601	1,96E-12
CD99	-1,444339857	1,91E-11
COPRS	-1,445123027	3,43E-12
NRBP2	-1,44757961	4,28E-05
MFHAS1	-1,448304833	4,35E-14
HEXIM2	-1,449144704	0,000332
GOSR1	-1,450032028	9,30E-11
SNHG3	-1,450889391	3,40E-05
ATP6AP2	-1,451943018	9,18E-09

EDEM1	-1,452452666	6,11E-09
HEXIM1	-1,4524929	1,59E-08
ADCY3	-1,45398297	5,59E-14
TNPO1	-1,456091705	4,17E-11
PPP2R3B	-1,456655091	0,000622
C5	-1,458348764	0,000617
TFE3	-1,458690864	6,58E-13
SNX12	-1,458826918	2,00E-05
SLC46A1	-1,459034005	5,18E-11
ABTB2	-1,459146227	1,80E-08
TMEM206	-1,460753088	0,00041
C11orf54	-1,462004081	0,000345
TMEM120E	-1,463904663	3,09E-08
PMAIP1	-1,464331587	0,000403
TBC1D20	-1,465984226	3,33E-10
DIEXF	-1,466763763	3,60E-06
SMC1A	-1,467289387	1,75E-11
FHOD1	-1,468116902	1,04E-12
LPGAT1	-1,468408165	5,38E-10
POLR2L	-1,470921574	6,74E-12
FZD7	-1,471108511	1,12E-07
MEF2D	-1,476066461	1,09E-05
SLC12A7	-1,477401578	9,60E-10
ST6GALNA(	-1,479643563	3,02E-07
SERGEF	-1,47998492	8,45E-05
ADCY7	-1,481220197	7,08E-08
YIPF6	-1,4819893	1,33E-10
STX1A	-1,48423699	8,53E-07
SEPT2	-1,485649412	4,61E-15
ASRGL1	-1,487151402	0,000105
PCCA	-1,48726186	7,76E-06
GRHPR	-1,491468899	3,14E-07
ACBD5	-1,49235345	9,03E-09
METTL3	-1,492861916	2,82E-11
EFHD2	-1,495333651	6,57E-07
PLP2	-1,495352038	3,62E-12
CIC	-1,497259435	4,21E-14
LAS1L	-1,498164389	2,03E-10
ADCK4	-1,498176347	2,10E-07
CACNA2D1	-1,501377698	2,94E-07
SPRED2	-1,50220177	2,73E-13
NEDD4L	-1,50240335	4,31E-07
TXLNA	-1,502796586	4,49E-10
GCLC	-1,504077243	1,65E-10
POLR3G	-1,504688077	1,54E-06
FUT10	-1,505178198	0,000932
ARL2BP	-1,506374673	3,41E-11
TTC21B	-1,506819177	2,08E-05
ZFAND5	-1,509055584	1,15E-13
SLC30A7	-1,509718261	2,47E-07

NAV2	-1,509983598	1,92E-05
BACH1	-1,511943407	2,98E-07
ROCK2	-1,512206259	2,48E-10
TMEM147-	-1,512490891	2,96E-05
PARVB	-1,514404754	2,63E-08
CTSC	-1,515395175	9,93E-09
MED14	-1,515453974	1,28E-14
SMARCD3	-1,516085048	5,44E-06
CELSR1	-1,518769824	2,81E-14
TMEM41B	-1,521335362	4,53E-09
UBQLN2	-1,522317315	5,63E-14
ASAP1	-1,522335216	2,14E-14
SYMPK	-1,523340141	4,41E-17
ITPRIPL2	-1,524722633	4,35E-10
ZIC2	-1,526375326	0,000206
KSR2	-1,527623608	0,000174
DOCK9	-1,52815618	2,80E-08
TRAF5	-1,529014529	4,52E-05
GALM	-1,529615068	0,000289
IRF1	-1,52981743	2,90E-05
C1GALT1	-1,530858504	2,52E-11
WWC3	-1,531177301	1,05E-10
IRS2	-1,532074924	0,000209
CDR2L	-1,532827189	1,75E-05
VOPP1	-1,533338207	2,73E-09
STXBP4	-1,533777845	4,30E-07
NT5DC3	-1,536910063	2,31E-06
ITM2C	-1,538325432	1,09E-08
SKIL	-1,540399839	7,46E-09
TTC33	-1,541005434	2,97E-05
TEAD1	-1,541119484	1,07E-11
CCDC113	-1,542101602	5,12E-06
RPS6KA5	-1,542464242	0,000146
CBR1	-1,542797136	4,24E-08
CWC25	-1,542820877	1,73E-10
ID2	-1,543762128	4,00E-05
CDCA7	-1,543882463	0,000524
EXT1	-1,5444812	1,15E-07
EPN2	-1,547041731	6,54E-13
PNP	-1,547373819	1,17E-14
C20orf96	-1,547640775	0,000882
APH1B	-1,547686368	0,000296
FERMT2	-1,551902174	3,12E-10
MARVELD1	-1,552321975	8,35E-09
PRAF2	-1,553156442	8,44E-05
SSH2	-1,55343478	8,51E-08
AKAP17A	-1,554785895	1,77E-06
SH2B3	-1,55527668	3,93E-05
ME2	-1,556615066	1,76E-12
GPATCH11	-1,556669233	3,55E-07

GNG12	-1,558020562	1,21E-10
PEPD	-1,558225077	1,06E-11
ALDH1B1	-1,5588461	2,55E-17
MYO1B	-1,560865485	1,69E-17
SLC17A5	-1,562476636	0,000284
NCEH1	-1,564037848	8,19E-07
PIP4K2B	-1,566166558	2,09E-15
IRS1	-1,5705276	2,59E-16
HCCS	-1,577537256	2,12E-09
MALT1	-1,578005733	6,05E-12
USP11	-1,579282799	1,71E-14
TARBP1	-1,58022166	1,35E-07
TPM1	-1,581677568	1,73E-07
CYB5B	-1,58219333	3,68E-09
LINC01128	-1,584546497	0,000227
PCNA	-1,584614436	1,18E-19
SH3RF1	-1,586175273	7,95E-05
CAPRIN2	-1,586265028	2,18E-06
LGALS3	-1,58855	0,00022
CCND3	-1,588672157	0,000481
USP9X	-1,589546534	9,91E-12
SAT2	-1,590985682	7,23E-07
SWAP70	-1,591814587	2,68E-10
EHD3	-1,592995346	1,79E-05
LITAF	-1,593395501	1,85E-07
LOC72874З	-1,594022837	0,000168
MAP3K10	-1,594577698	4,43E-08
GBAS	-1,596921867	0,000138
RASA3	-1,597316607	3,03E-06
RNF135	-1,597820857	1,13E-06
AQR	-1,598244519	1,99E-17
ECHDC1	-1,599683916	5,15E-08
EGLN1	-1,600865662	4,59E-14
ANTXR1	-1,603100505	3,57E-19
PFAS	-1,604801887	1,43E-19
CD46	-1,606864074	1,93E-13
AVPI1	-1,609537621	1,77E-07
FRG1HP	-1,611491831	0,000287
FNTA	-1,613414319	2,99E-13
PANK2	-1,614556889	8,10E-12
MCM8	-1,615035986	1,11E-13
YAP1	-1,616587319	5,73E-13
IDS	-1,618150142	2,09E-06
HLA-A	-1,624676828	1,35E-09
UGGT2	-1,624753397	3,86E-07
LRP8	-1,628736708	9,28E-11
MAPKAPK5	-1,62928973	2,49E-10
CASP9	-1,630889229	0,00049
KLF3	-1,632123452	1,00E-08
TMEM104	-1,635111789	6,12E-08

ASXL2	-1,640178463	1,83E-13
CASK	-1,641962506	3,85E-15
SAMHD1	-1,642255	0,000673
PLEKHA2	-1,642590545	6,43E-05
HEATR1	-1,644300948	1,46E-13
RCAN3	-1,644641717	0,00049
DOK4	-1,645424244	6,00E-11
LAMB1	-1,645612365	1,96E-17
FEZ2	-1,646297839	1,70E-14
SAT1	-1,647004148	1,31E-08
C16orf70	-1,650107384	1,90E-08
FGD6	-1,656199447	3,67E-12
RAB34	-1,659634245	4,87E-10
ATP5G1	-1,663349179	3,88E-13
DHRSX	-1,665578564	9,83E-08
WDR54	-1,666490198	5,45E-09
GANC	-1,669741473	1,73E-05
CASC4	-1,670680598	1,22E-15
DDX52	-1,674001591	2,63E-15
RBPMS	-1,674823575	5,22E-15
BMP1	-1,675351202	2,33E-11
LDLRAD3	-1,675355833	9,73E-09
MLPH	-1,676371389	1,05E-15
NUAK1	-1,679268799	3,20E-12
STK17B	-1,679407399	3,63E-08
NEIL2	-1,679494235	1,64E-11
LMO7	-1,68042288	5,65E-11
NIPA1	-1,682374827	1,51E-15
PCED1A	-1,684706819	5,88E-09
ARSK	-1,688226066	9,81E-05
PM20D2	-1,692972787	5,32E-07
HERC2	-1,69407679	9,90E-21
DENND5A	-1,694155189	1,06E-07
TEP1	-1,694310035	1,68E-06
PSAT1	-1,695567955	0,000755
CD99L2	-1,698978623	1,23E-08
CLTCL1	-1,700527389	0,000236
PLD1	-1,700984609	1,48E-07
SYT1	-1,701216858	5,30E-07
ID1	-1,702851199	2,44E-05
PFKP	-1,70553494	6,78E-11
KIAA0930	-1,705807424	2,33E-09
STIM1	-1,708274641	1,02E-15
AP1S2	-1,708935821	1,09E-09
MB21D1	-1,713007748	8,36E-05
TOX2	-1,714201094	6,95E-09
SLC4A7	-1,71471726	1,14E-10
PHF19	-1,715531329	1,93E-16
FAM219A	-1,716600747	9,04E-05
PLS3	-1,717516746	2,24E-15

GALC	-1,721237104	2,46E-05
CRYZ	-1,721413956	1,77E-07
CTSB	-1,721856009	4,18E-08
ABHD15	-1,723376625	2,49E-08
HGSNAT	-1,725073197	7,73E-07
COASY	-1,729305868	3,15E-18
PRKAR2A	-1,729384774	3,81E-09
ARHGEF28	-1,729682604	2,30E-10
TPCN1	-1,732472698	1,80E-13
ATP2B1	-1,732847295	2,12E-16
SESTD1	-1,736235618	1,78E-07
AP2B1	-1,737690649	1,28E-15
WDR81	-1,738055406	2,10E-09
FRAS1	-1,738064337	2,03E-12
ATP8B2	-1,738888321	2,31E-12
HDAC6	-1,742480542	9,50E-11
HACD1	-1,745965611	7,25E-06
PLIN2	-1,74658769	1,64E-06
DNAJC18	-1,746940449	6,75E-05
SEMA4B	-1,749875125	1,59E-13
PITPNM1	-1,75168531	2,42E-09
GPR137B	-1,752436511	6,70E-06
PEX6	-1,755942581	0,000916
MYADM	-1,758385078	1,21E-06
IFT81	-1,760411989	6,53E-14
AKIP1	-1,763194778	4,31E-11
LGALS1	-1,764471088	5,93E-08
ULK2	-1,766553732	1,33E-05
LRP6	-1,77284123	3,64E-17
PDZD8	-1,77330563	9,77E-20
PLD3	-1,774335905	3,06E-10
KCTD7	-1,775221947	1,71E-05
TUBGCP5	-1,777019493	3,49E-13
PARVA	-1,779912657	6,03E-11
SEC23A	-1,783434265	1,49E-14
SPTBN1	-1,785485318	1,38E-26
CDC42EP4	-1,787684427	2,02E-10
P3H1	-1,788750999	1,85E-10
RELB	-1,789553789	4,14E-05
RANGRF	-1,789631223	1,58E-09
TRMT11	-1,789846138	0,000893
FARP2	-1,793019592	1,07E-09
IFNGR1	-1,793943677	2,38E-13
PTDSS2	-1,793985556	1,61E-06
GALNT14	-1,795905617	1,56E-16
SDCCAG8	-1,798735939	5,81E-08
STOX2	-1,800382616	0,000138
HSPA2	-1,80057969	7,63E-05
PLOD2	-1,801731104	1,35E-20
PATL1	-1,805216039	2,72E-16

TLN2	-1,807382169	1,96E-07
FABP5	-1,808316959	6,78E-21
SNHG25	-1,809044927	1,93E-11
HOXB7	-1,809735751	1,65E-10
FLNB	-1,810415772	8,62E-11
B4GALT6	-1,810819789	4,16E-07
CPPED1	-1,811293592	3,93E-14
PITPNM2	-1,813354734	2,69E-08
NAB1	-1,816900272	2,57E-09
GOLGA7B	-1,817601805	0,000925
CDC14B	-1,818668824	3,59E-10
P3H2	-1,822713825	4,45E-11
ATAD5	-1,823704593	3,91E-07
ADA	-1,827392993	3,61E-07
GFPT2	-1,828044853	1,24E-05
EFNA5	-1,828515849	1,99E-12
MAP2K6	-1,829320715	2,03E-10
MAP3K14	-1,830015664	0,000339
MARCKS	-1,830223431	4,22E-14
WLS	-1,830380971	4,39E-06
ZCCHC24	-1,831395004	0,000658
TRIM5	-1,832437963	5,45E-05
SLC35B4	-1,832982396	7,19E-21
ENO2	-1,83696129	1,00E-06
VCL	-1,83827484	3,31E-08
KIF3C	-1,840315887	2,64E-11
ELL2	-1,840355889	1,80E-07
ANKMY2	-1,840649248	2,00E-09
SLC39A10	-1,841619864	1,73E-15
RNF145	-1,84447694	3,30E-06
PRTG	-1,850249785	1,61E-05
SEMA4F	-1,850465987	0,000475
KLHL15	-1,85259595	4,45E-06
PLAGL1	-1,853327647	2,78E-06
STX2	-1,855084111	8,10E-13
DAGLA	-1,857202789	5,03E-05
CRLF3	-1,857758097	7,73E-08
LBR	-1,859514993	1,33E-16
C19orf12	-1,862028105	1,82E-13
CDON	-1,862034737	0,000535
EXTL2	-1,863196479	3,99E-07
CITED4	-1,866200711	2,55E-06
DEDD2	-1,866718787	1,58E-12
ACACA	-1,866860515	7,24E-26
TCIRG1	-1,868922834	8,86E-10
MYO10	-1,871461001	5,53E-17
FNDC3B	-1,871862031	9,50E-22
MAML3	-1,873578674	3,70E-09
ADGRE5	-1,873921652	8,53E-07
KLF12	-1,874630933	3,62E-07

MCFD2	-1,875616628	6,37E-23
SNAP25	-1,877203442	0,000306
APOBEC3B	-1,878215942	3,14E-08
USP40	-1,878454161	2,91E-12
ANO6	-1,879995862	1,98E-14
SNHG10	-1,8803793	0,000367
ADCY9	-1,880983235	7,71E-10
ZNF319	-1,883277919	0,000324
TMEM106E	-1,884265603	8,83E-18
ITPR1	-1,885390757	1,55E-09
LINC00641	-1,88862155	2,07E-05
CPOX	-1,888622609	7,41E-07
BCL3	-1,890195811	3,99E-10
CARNMT1	-1,896450682	5,18E-11
FUT11	-1,896942712	5,57E-08
TJP1	-1,902026983	3,54E-11
LTBP2	-1,906781983	0,000347
SNAI1	-1,916480893	0,000808
LHFPL2	-1,918178397	6,94E-17
RAB27B	-1,92063752	1,34E-14
IMPA1	-1,92175894	5,14E-12
FAM169A	-1,925189015	0,000176
CEP170	-1,926713899	5,20E-22
SORBS3	-1,927049216	1,03E-21
ABCB10	-1,92836782	3,15E-12
ABCB7	-1,937458986	4,44E-10
WNT7B	-1,940082379	3,38E-07
PLEC	-1,9404649	4,39E-06
IER5L	-1,940669875	1,26E-07
MPDZ	-1,946574282	2,57E-10
SPIN4	-1,948938679	7,40E-08
ACER3	-1,952843901	2,33E-21
TNFRSF10E	-1,954141224	8,66E-20
DIAPH2	-1,95528438	5,73E-12
GATA2	-1,956724759	5,07E-09
GAREML	-1,956868657	5,04E-07
TXNRD1	-1,963784157	1,14E-08
CA11	-1,964360122	8,96E-05
GID4	-1,966099472	1,60E-10
SELM	-1,96699303	2,72E-07
EVI5	-1,967468872	4,53E-09
DMTN	-1,967570399	1,22E-17
BICD1	-1,968209493	8,60E-13
TREX1	-1,968231976	6,16E-06
JAK1	-1,972371846	3,34E-16
PIM2	-1,972865192	1,49E-12
BASP1	-1,976973042	0,000362
RDX	-1,978487247	6,23E-17
TXN	-1,981320441	3,61E-11
NR2F1	-1,981798515	2,36E-08

KIAA0355	-1,984411351	5,18E-14
SLC9A7	-1,984962432	1,57E-06
PTPN14	-1,985968006	3,32E-12
FAM161A	-1,988511075	1,66E-06
PTPRJ	-1,99006897	1,05E-15
PLS1	-1,991509185	1,14E-14
WTIP	-1,995283934	0,00025
CAMK1D	-1,999239538	1,74E-10
SLC39A14	-2,008902968	1,01E-06
FN3K	-2,013039019	0,000969
SIPA1L3	-2,014930527	1,01E-27
FOSL2	-2,017574683	1,83E-11
USB1	-2,017891546	7,96E-09
HFE	-2,020454263	0,000151
FAM46A	-2,02177754	3,95E-05
GLIS2	-2,024169235	2,97E-19
DPYSL2	-2,025916877	1,11E-10
SYDE1	-2,029468907	3,26E-09
EPHB2	-2,033700811	9,48E-20
STXBP1	-2,033803018	3,83E-19
CASC10	-2,03416681	2,01E-05
VAMP2	-2,038230837	4,68E-17
TRIM16L	-2,040591557	2,41E-05
SRGAP2D	-2,042150444	1,19E-05
ITPRIP	-2,042873958	3,38E-05
GNPDA2	-2,043463723	1,72E-06
IL6ST	-2,048621492	2,65E-17
CHORDC1	-2,050058514	6,49E-20
STEAP3	-2,053866365	6,98E-13
ELF3	-2,058943832	0,000131
IFIT5	-2,065663701	1,76E-05
TYMP	-2,066829869	8,88E-05
PVR	-2,067725744	3,68E-08
LDHA	-2,070492277	3,00E-32
CCNJL	-2,072319326	3,36E-07
DYNLT3	-2,074192817	7,48E-06
PRPS1	-2,078758947	5,18E-18
ADGRB2	-2,085770917	1,29E-07
KCTD17	-2,088166835	9,22E-07
NUMBL	-2,088860703	9,22E-23
SHISA4	-2,089676761	0,000805
EID2	-2,090988157	1,72E-18
FUT4	-2,091635651	1,01E-05
STAT3	-2,093435436	1,19E-09
LOC28445[illegible]	-2,096033919	0,00062
TCF12	-2,097484248	2,18E-13
SDC1	-2,100044417	3,16E-22
CPEB1	-2,101870045	0,000236
ARNT2	-2,1036601	2,53E-17
KIAA0319	-2,107474341	0,000447

PGD	-2,113527306	1,72E-11
PRKAA2	-2,116527031	2,78E-15
SIM2	-2,116797235	4,30E-09
KCNAB2	-2,121467026	0,000103
PTCH1	-2,121757568	2,09E-09
ATP1B1	-2,122580588	7,52E-13
ZDHHC1	-2,12500203	0,000111
LASP1	-2,128248719	9,11E-15
FBXO17	-2,130060965	8,95E-10
HSPG2	-2,130449704	1,03E-16
DNAJC24	-2,130620812	7,70E-12
SYNJ2	-2,132120889	3,09E-19
CDS2	-2,135851584	1,93E-21
PRTFDC1	-2,138102366	3,01E-08
THBS1	-2,140866332	6,31E-08
DLGAP1-AS	-2,144254388	0,000198
CAPG	-2,144705251	6,96E-18
IL13RA1	-2,147149791	1,20E-16
FAM107B	-2,148838783	1,37E-07
TRAM1	-2,150709171	7,27E-12
PLCD3	-2,150878406	3,65E-08
HSD17B12	-2,151702164	5,72E-29
MAP3K12	-2,157191961	2,99E-07
LPCAT2	-2,158170388	1,36E-08
SERPINB1	-2,160889742	8,89E-16
GRB10	-2,162943655	0,000443
NEDD4	-2,164596902	1,16E-25
CARD10	-2,166040474	1,42E-11
AHI1	-2,170112786	4,55E-14
SFMBT2	-2,171446084	1,07E-06
TCF7	-2,172470338	9,16E-14
RASSF10	-2,173202777	3,78E-07
GALT	-2,17338393	0,000415
YPEL2	-2,176087197	0,000839
CPNE2	-2,17828855	1,36E-08
PMEPA1	-2,183043691	6,05E-15
ARHGAP29	-2,185102293	1,18E-08
ADPRH	-2,189376374	0,000384
PLXND1	-2,190058797	1,79E-12
MTMR10	-2,191381194	6,03E-14
ATG16L1	-2,193676739	4,68E-26
CAPN10	-2,196467466	8,79E-11
AP1S3	-2,19666235	4,48E-13
DIXDC1	-2,199356544	3,75E-13
HNRNPU-A	-2,201208333	0,000184
GCLM	-2,205115798	2,53E-09
ZAK	-2,206127585	5,86E-30
DGCR5	-2,20882555	1,87E-05
APLP1	-2,208976219	1,91E-06
BHLHE40	-2,211849444	4,88E-20

ANXA4	-2,213708536	6,30E-15
ABHD14A	-2,214354882	0,000689
GALNT18	-2,218088068	4,53E-16
EPB41L1	-2,218206688	4,74E-21
CDKL1	-2,218874716	5,61E-06
RNF157	-2,219600418	4,10E-12
RBM24	-2,220060873	2,81E-07
CYFIP2	-2,220432832	1,95E-08
HJURP	-2,221265172	2,71E-15
RNF170	-2,22227403	3,34E-12
RNF213	-2,22318742	2,59E-11
LAMC3	-2,223539944	2,79E-06
SDCBP	-2,224391013	8,23E-13
BRSK2	-2,224919846	6,15E-06
CPD	-2,225885835	7,38E-31
ADAM10	-2,230517562	4,10E-24
SLC16A3	-2,237337887	3,82E-06
GSR	-2,239186552	1,76E-23
CTSF	-2,2399567	4,21E-10
TTC28	-2,240028436	4,47E-15
L3MBTL3	-2,241919633	2,34E-09
EID2B	-2,242188017	4,93E-06
SCARA3	-2,2434334	2,27E-15
TMC5	-2,243690911	1,08E-15
PAK1	-2,24707866	2,77E-11
KCNQ5	-2,250433543	0,000169
LZTS3	-2,250668698	0,000185
IKBIP	-2,254439392	3,47E-07
FKBP14	-2,255146701	2,67E-17
TMX3	-2,257811834	2,80E-19
ITGB4	-2,263565967	3,63E-16
UPP1	-2,267128009	0,000581
HIPK2	-2,274135816	3,52E-11
ZNF512	-2,276912575	5,97E-22
ACCS	-2,27941561	0,000525
CFL2	-2,285645909	1,25E-22
PRKCE	-2,290353975	3,00E-05
ANK2	-2,293866137	0,000592
TSPAN5	-2,296749438	4,26E-08
WDR35	-2,302458147	1,51E-15
WDR90	-2,302759843	1,55E-07
THRA	-2,30578153	1,08E-08
SYP	-2,309548862	0,000372
CNTLN	-2,316537509	7,37E-11
C17orf104	-2,316861933	4,87E-05
ETHE1	-2,319089812	6,23E-12
MAFG	-2,32188122	1,91E-31
LPCAT4	-2,326678032	1,20E-10
TMEM17	-2,328231809	1,15E-08
GNB4	-2,328558669	7,60E-19

PBX1	-2,328995574	6,47E-08
RAB31	-2,329077699	3,57E-08
ABHD4	-2,329245018	4,40E-06
FAT1	-2,329637814	1,99E-14
ANXA2	-2,330746247	3,08E-45
MET	-2,335696518	2,16E-40
FAM131C	-2,343511831	4,36E-08
TNNI3	-2,348024979	8,65E-05
CCDC82	-2,352682865	5,25E-07
TNFRSF1A	-2,352768424	2,06E-20
TBX3	-2,354831689	4,25E-35
SH2D4A	-2,357669942	2,80E-27
C12orf75	-2,369793329	4,21E-23
SEMA3B	-2,370029045	4,09E-08
DSTNP2	-2,370588663	5,26E-06
ANXA2P2	-2,370844525	2,43E-08
CPEB2	-2,372963177	2,68E-09
FMNL1	-2,373219006	4,79E-17
IL4R	-2,379371751	3,64E-08
TGFB1	-2,381476927	8,55E-26
PHKA1	-2,384547231	7,59E-24
CAMKK1	-2,386361714	1,44E-13
DEGS1	-2,392580498	1,44E-16
CLIP2	-2,397485647	9,54E-15
ID3	-2,401148867	5,18E-11
SSFA2	-2,401293442	1,11E-34
ABHD6	-2,403420475	1,29E-08
RPL13P5	-2,412522741	2,30E-05
FBXO2	-2,413724331	1,07E-05
NCOA7	-2,414302323	4,52E-24
FGFBP3	-2,418348699	7,34E-09
RNPEPL1	-2,4242428	4,81E-20
PORCN	-2,426111215	2,48E-09
A4GALT	-2,42725596	9,25E-05
FAM63B	-2,431994557	2,58E-07
TRIP10	-2,432676987	3,49E-13
SLC25A12	-2,433221121	6,50E-21
SLC35G1	-2,437424286	8,88E-08
GALNT2	-2,440758635	6,52E-34
METTL7B	-2,442039065	2,14E-10
CHM	-2,442187102	1,58E-08
KBTBD11	-2,442307584	1,99E-07
RILPL2	-2,443062189	1,61E-05
IQSEC2	-2,44335333	5,67E-11
PHYH	-2,443965175	6,96E-19
LYPD1	-2,447913557	3,30E-12
BRWD3	-2,450505207	4,80E-09
DOCK1	-2,450836356	1,58E-24
COTL1	-2,451400877	3,32E-24
SLC23A2	-2,451801958	1,18E-23

PKD2	-2,461933241	2,85E-19
NBEA	-2,463944774	5,59E-14
UNC13A	-2,47098578	0,000576
ADORA2B	-2,47184561	1,12E-08
VASH1	-2,473022375	4,91E-08
ABCC4	-2,473667738	8,25E-31
REEP2	-2,476229517	1,15E-10
CTSL	-2,480767482	2,39E-13
KIAA1804	-2,481810113	9,05E-10
C1orf106	-2,484204344	4,74E-10
OAF	-2,487696924	6,65E-13
GLS	-2,491836843	2,05E-23
PRDM11	-2,492296532	3,07E-07
MFI2	-2,494290325	1,10E-24
DCBLD1	-2,499822743	1,43E-14
CYP4V2	-2,50095507	0,000381
FKBP7	-2,501362491	3,41E-11
ITGAV	-2,502950377	2,44E-26
CASC15	-2,503498422	5,86E-05
TNFAIP3	-2,50490909	9,73E-05
SGTB	-2,505020514	8,31E-16
TSPAN14	-2,508375892	9,01E-32
ECE1	-2,51058738	5,62E-31
HEYL	-2,512970087	1,52E-09
FAM81A	-2,513181715	4,83E-13
NRIP1	-2,516169105	3,79E-15
STOM	-2,516272947	1,96E-35
ZNF81	-2,520836778	3,14E-07
SRXN1	-2,524740719	5,05E-12
ITGB1	-2,52529863	1,48E-47
GOLM1	-2,528165851	1,29E-38
C19orf33	-2,529411553	3,34E-16
FSTL3	-2,535562893	6,19E-11
GPAT3	-2,544829379	1,24E-07
NCR3LG1	-2,546639805	9,73E-07
RDH10	-2,547014626	3,16E-15
B4GALT5	-2,548305827	3,88E-31
ACBD4	-2,553015357	2,00E-08
ARL4D	-2,555567409	4,98E-11
TK2	-2,562810851	2,43E-13
ZBED5-AS1	-2,568913917	3,38E-10
FTL	-2,570932756	5,20E-13
SLC12A2	-2,577618794	1,80E-28
LINC01604	-2,578573145	4,37E-05
ADORA1	-2,579232637	6,85E-08
PON2	-2,589241837	1,97E-16
SBF2	-2,591566926	8,73E-10
KCNH3	-2,592906162	3,25E-10
PGM1	-2,593781375	1,53E-18
MAP1A	-2,602027834	3,04E-05

WBSCR27	-2,602448164	2,90E-05
AFAP1L2	-2,608043905	6,55E-09
CHD9	-2,614383951	1,09E-28
C20orf196	-2,615924318	8,29E-06
GNG4	-2,618250743	5,95E-07
TSPAN4	-2,622063114	1,66E-14
LYST	-2,623454305	3,83E-07
TCP11L1	-2,623835622	9,14E-21
SIPA1	-2,624125983	6,36E-16
SCCPDH	-2,624946868	4,05E-26
CDK6	-2,627933655	1,03E-06
SOCS6	-2,632500991	5,28E-28
CYP2S1	-2,636283768	6,28E-10
CCDC88B	-2,639999743	5,07E-07
COL4A4	-2,641337732	0,000431
EHF	-2,642630779	1,32E-09
PRPS2	-2,650344179	1,05E-29
EDA2R	-2,65884519	3,74E-10
TBC1D8	-2,659475858	2,04E-08
AREG	-2,666492998	1,77E-11
ST6GAL1	-2,668587739	1,55E-06
GBX2	-2,677636394	0,000866
SLC6A6	-2,680006763	1,07E-36
SLC1A1	-2,686447728	0,001015
STRIP2	-2,686681925	5,56E-10
TMCC1-AS1	-2,693017289	4,91E-07
PALLD	-2,693143702	2,30E-18
PYGO1	-2,697363372	6,78E-10
VPS13C	-2,699114961	1,41E-23
PCDHAC2	-2,70096235	8,94E-06
DNMBP	-2,705117072	2,80E-19
GYG2	-2,708674209	3,31E-08
TNFSF9	-2,712487356	8,55E-05
TGFBR3	-2,713365903	0,00024
HOOK3	-2,717833399	3,08E-23
ARHGAP24	-2,719720658	1,72E-05
RPH3AL	-2,725609943	7,68E-13
LOC145783	-2,728230377	1,39E-06
BTN3A1	-2,73654328	0,000312
MATN3	-2,745508677	7,35E-05
MXRA7	-2,748743962	7,12E-16
SFXN3	-2,749555339	8,18E-20
LOC339803	-2,758122273	2,87E-08
LOC100130	-2,762264628	0,000419
CMIP	-2,763623575	1,59E-37
KLHL5	-2,764722222	5,01E-40
PCOLCE2	-2,765982512	4,56E-14
GRAMD1A	-2,767591578	8,94E-28
EGLN3	-2,770009441	1,22E-16
PRKD1	-2,774445342	3,55E-08

ACO1	-2,775316418	3,14E-16
MTUS1	-2,776045692	4,59E-32
MAP3K8	-2,783396326	2,63E-06
FADS1	-2,78404692	1,60E-23
C15orf41	-2,788392695	2,52E-13
LIF	-2,789389814	1,79E-06
SPRY2	-2,79247235	4,07E-11
SUSD1	-2,795344804	4,29E-18
SRC	-2,795457169	3,67E-41
EHBP1	-2,797995239	1,42E-38
GALNT10	-2,80386621	2,10E-37
RASD2	-2,812307287	2,17E-05
LRP1	-2,813071842	2,30E-07
GGT1	-2,818555128	4,56E-40
KLF13	-2,82416595	6,02E-24
KCNF1	-2,828578169	1,31E-09
PELI1	-2,831500485	3,58E-09
EML2-AS1	-2,831943038	5,94E-05
GRK5	-2,836476196	1,68E-09
LRRN2	-2,836897248	3,46E-06
MUC5B	-2,845783695	1,58E-21
SLCO3A1	-2,845800882	1,30E-23
ADAM9	-2,84849873	3,76E-46
FXYD5	-2,852235302	5,89E-29
NT5M	-2,855845538	1,64E-10
TAGLN	-2,856720979	8,02E-05
KIF13B	-2,862613519	1,43E-09
DNAJB4	-2,867950928	5,04E-07
NGEF	-2,86959755	2,04E-13
BTN3A2	-2,872318111	0,00015
ARHGEF10	-2,882176343	4,27E-16
NR2F1-AS1	-2,888191542	5,90E-12
NFAT5	-2,890842297	7,23E-22
FAM135A	-2,894210537	1,07E-14
FERMT1	-2,895333932	8,51E-40
DENND3	-2,895366497	2,77E-07
ITGA2	-2,896672787	9,62E-33
RPGR	-2,89944104	4,12E-06
ZBTB38	-2,900392667	1,50E-39
KLRG1	-2,903808171	0,000579
WNT3	-2,912156878	1,32E-07
PTHLH	-2,915049614	4,17E-11
GPRC5B	-2,917244233	7,96E-09
ABHD8	-2,91831183	0,000835
DUSP28	-2,919247026	7,28E-05
CLCF1	-2,923273467	5,50E-05
DPY19L2P2	-2,926718855	0,0001
MICAL2	-2,936122556	2,83E-29
PPARG	-2,936516551	5,79E-13
CCND2	-2,936604527	0,00012

GHDC	-2,936938621	3,79E-21
SASH1	-2,939835656	6,20E-11
ADGRL2	-2,94566724	6,35E-29
ACSL4	-2,953063426	4,19E-30
FAM72C	-2,9575849	0,000173
ABCC1	-2,960483529	4,79E-50
SPOCK1	-2,961568875	1,47E-08
ST3GAL5	-2,963255542	4,39E-09
FCGBP	-2,963661754	4,58E-05
SGK223	-2,968142847	8,51E-38
HNF4G	-2,977176327	0,000126
EVC2	-2,977947249	4,23E-07
LOC171391	-2,978364799	1,14E-06
LINC00294	-2,981170442	2,03E-12
PTRF	-2,982103663	2,19E-27
MCOLN2	-2,985565775	1,02E-05
GPCPD1	-2,986894671	1,36E-17
BCAR3	-2,987447756	2,52E-26
ACSM3	-2,987758949	8,32E-10
FKBP1B	-2,993067509	5,45E-06
NDRG1	-2,994640994	2,48E-27
KSR1	-2,998470224	1,22E-14
AKAP7	-2,998493019	4,91E-06
TIMP1	-3,00086058	1,82E-08
VANGL2	-3,00111973	1,53E-11
HYAL3	-3,003692008	1,62E-07
CMTM3	-3,021723393	6,83E-16
PRR5L	-3,042960663	4,92E-12
MGC70870	-3,048040715	0,000457
RBM20	-3,05118445	7,39E-08
ARNTL2	-3,056134005	1,08E-21
CNFN	-3,067873422	4,79E-15
IL1R1	-3,071833119	1,99E-06
LOC730102	-3,075369222	2,11E-05
ADGRG6	-3,083031395	1,01E-42
CAMK2D	-3,086291182	1,52E-28
CHIC1	-3,090741575	3,07E-14
RTTN	-3,097374691	7,03E-19
IFFO2	-3,099344807	2,53E-32
TYW1B	-3,100021856	1,16E-05
ANO7	-3,103316482	0,00011
SMAD3	-3,103524902	4,77E-23
DNAJC22	-3,104857002	5,73E-29
TIMP2	-3,105660572	1,38E-27
EPHX1	-3,108874475	1,75E-20
TUBB4A	-3,115558501	1,38E-17
PSMB9	-3,118218365	3,52E-06
SYNPO	-3,127136428	2,57E-10
CLCN4	-3,131584094	7,01E-11
DDX60L	-3,138365466	9,81E-14

HOXC4	-3,14390568	1,37E-12
NUDT11	-3,144938984	3,70E-06
FGFR1	-3,146487106	3,19E-57
PPAP2B	-3,147972901	8,00E-20
FAM89A	-3,150831354	4,39E-10
TOX3	-3,155207677	1,20E-05
SORCS2	-3,167256483	4,81E-08
CEMIP	-3,168171274	8,37E-14
FRMD5	-3,170876697	7,34E-19
PIK3CD	-3,176220735	3,42E-08
ADGRF4	-3,179704278	5,68E-06
NUAK2	-3,184017351	1,43E-20
ST6GALNA(	-3,190241737	8,02E-13
STARD9	-3,192852355	2,47E-06
LOC100507	-3,195833914	0,000814
GPR39	-3,197723457	1,21E-12
HOTAIRM1	-3,198869775	8,57E-05
LIPH	-3,201491527	1,44E-07
ZNF532	-3,201800209	5,24E-38
HTR1D	-3,203411644	7,45E-08
SPTB	-3,215199911	0,000323
FSTL4	-3,218360636	2,06E-13
DSE	-3,21939713	2,17E-22
GOLGA8B	-3,224779116	1,26E-06
LRP4	-3,228397998	1,18E-17
MFGE8	-3,234698026	1,16E-41
JAZF1	-3,235465562	0,00037
ANXA3	-3,235811475	4,82E-19
RAB3B	-3,238104179	2,99E-12
MST1R	-3,243684649	4,62E-09
RHPN2	-3,258470219	2,15E-58
CHST3	-3,262431462	1,41E-07
DOCK5	-3,262455098	1,39E-36
LGR4	-3,270254572	1,60E-27
CENPV	-3,270872524	6,82E-14
PRNP	-3,27115845	2,31E-32
ELFN2	-3,271594132	1,69E-08
TMEM56	-3,272842757	1,85E-27
OSMR	-3,283578511	2,49E-21
LRRK2	-3,288910384	1,20E-13
LOC344887	-3,295200043	0,000752
GRIN2D	-3,307059495	5,41E-18
SOWAHC	-3,317412694	9,29E-23
COL27A1	-3,318805675	8,22E-12
NXPE3	-3,319379757	2,13E-11
KIAA1549L	-3,320921652	4,53E-16
PLK2	-3,321623028	5,33E-23
LOC389831	-3,334586725	3,80E-23
HLF	-3,339545602	3,34E-09
FAHD2B	-3,344733193	7,50E-18

F2R	-3,348285188	1,14E-50
FBLN5	-3,350313299	6,87E-06
C6orf141	-3,353825171	2,21E-11
SLC47A1	-3,356943096	1,06E-25
QPCT	-3,360041355	1,01E-15
SDK2	-3,364449426	5,29E-12
RASSF4	-3,372000432	1,50E-05
CYP4F3	-3,378138531	0,000199
C16orf45	-3,38522245	2,25E-24
PDLIM5	-3,390797565	1,96E-36
SQRDL	-3,391941641	2,49E-16
CREG2	-3,393906131	0,000673
PDGFRL	-3,397555266	6,69E-19
WWTR1	-3,398927209	6,91E-60
B3GNT9	-3,398955687	3,50E-06
THSD4	-3,411411336	2,66E-28
ADAMTS9	-3,413570203	1,76E-08
SH3BGRL	-3,418160978	5,10E-36
SLFN11	-3,418476219	4,01E-22
CUEDC1	-3,419939482	5,31E-64
DNAJC12	-3,425723788	7,94E-06
PIK3AP1	-3,427591229	3,21E-05
BCL2L15	-3,432367471	2,62E-09
HOXA7	-3,440471398	3,38E-05
DCLK2	-3,443721406	1,26E-13
KIAA1462	-3,448335265	5,96E-11
LINC01468	-3,46121666	4,46E-08
CHST6	-3,466257524	0,000417
VLDLR	-3,47426875	1,19E-10
SH3RF2	-3,479715165	8,69E-12
SRPX	-3,484949726	2,07E-09
HDX	-3,485707592	0,000112
MBP	-3,486238849	4,93E-23
FAM171A1	-3,488466347	1,89E-39
C3orf52	-3,506477859	4,49E-05
LRRC8C	-3,50694548	1,09E-19
COL21A1	-3,510307519	6,02E-06
ZNF264	-3,514312325	6,39E-20
EPB41L4A	-3,52082005	2,37E-15
ANXA2P3	-3,525398929	1,98E-13
COL4A5	-3,536340057	4,18E-19
CCDC68	-3,538038726	1,41E-06
RBMS2	-3,542062665	4,02E-33
UGDH	-3,543224252	1,10E-48
DLGAP1-AS	-3,5438839	4,91E-06
EPDR1	-3,545062073	4,68E-32
PLA2R1	-3,547234235	0,000223
PCED1B	-3,551324742	9,85E-14
RUSC2	-3,557992981	1,60E-25
MAP3K15	-3,581457828	1,02E-09

FAM13A	-3,589370201	1,92E-14
AMDHD1	-3,591031744	3,62E-12
FAM101B	-3,591758253	4,86E-27
LINC00152	-3,593148692	3,46E-15
RTN4RL2	-3,596051011	1,33E-10
RIN3	-3,620281211	5,46E-25
APOL1	-3,621025751	0,000373
HOGA1	-3,627701066	1,08E-09
HSD17B11	-3,627817464	3,64E-18
CDH23	-3,629662491	0,000509
BMP4	-3,633621934	5,28E-28
TUB	-3,646368295	1,91E-39
VIM-AS1	-3,652738335	1,30E-18
RRAS	-3,653091492	2,43E-23
NPAS2	-3,657212049	3,38E-35
PAQR5	-3,662314478	1,15E-53
MYLK	-3,66649102	4,09E-34
SP100	-3,669218466	1,26E-05
SLC16A4	-3,672604922	7,13E-26
NLRC5	-3,67688517	1,15E-07
TRPC1	-3,678943087	1,04E-15
LAMC2	-3,68230282	2,14E-12
CA12	-3,68415122	1,14E-12
GAS6-AS1	-3,69337918	0,00074
ZNF488	-3,699518632	8,22E-10
ANKRD29	-3,705164027	2,21E-07
S100A6	-3,706154175	1,83E-29
CPS1	-3,710860752	4,01E-53
ARPIN	-3,713563801	7,84E-33
GPC1	-3,723699383	5,13E-68
COL5A1	-3,727043237	6,62E-13
COCH	-3,730706666	7,44E-22
MGLL	-3,744497147	3,04E-18
KCNMA1	-3,747044346	2,12E-16
OLFML2A	-3,749268925	2,39E-13
HSPA12A	-3,751659801	2,21E-41
CAMK2N1	-3,75347989	5,97E-26
RASSF2	-3,756192772	6,70E-26
CD68	-3,758379673	5,93E-11
PIK3CD-AS:	-3,767851468	3,92E-07
ANKDD1A	-3,794516031	0,000298
TRABD2A	-3,797700408	0,000831
NRCAM	-3,801667352	1,42E-29
PAPSS2	-3,802226664	3,03E-07
MMD	-3,805744489	5,26E-24
CST1	-3,807616703	5,87E-41
ARHGEF40	-3,818415067	2,52E-11
RAB11FIP5	-3,823868728	1,15E-19
BOC	-3,825839321	9,08E-30
TRIM7	-3,830257893	9,77E-21

SERPINF2	-3,834947548	0,000841
CHST7	-3,838054056	6,15E-23
PRAME	-3,846932695	2,86E-18
FBN2	-3,85033524	1,38E-21
FHL1	-3,862091744	5,92E-21
C3orf80	-3,868773518	2,13E-06
THBD	-3,870063059	5,94E-39
RASGRF2	-3,875268123	4,37E-06
TMSB4X	-3,881138002	7,34E-113
CEACAM19	-3,886266663	1,50E-11
MORC4	-3,889363335	1,65E-56
ELMO1	-3,899727089	4,39E-10
HDAC4	-3,902959639	4,83E-12
PLCXD2	-3,90401293	1,23E-10
CCDC88A	-3,907782332	9,49E-82
HTATSF1P2	-3,912463254	1,15E-19
HEG1	-3,914581049	3,50E-05
DGKD	-3,917738033	2,18E-75
CDC42EP3	-3,925187295	7,14E-50
RARB	-3,926752143	8,54E-14
STEAP1	-3,932909477	1,38E-14
PLAUR	-3,93406433	5,13E-13
SNX7	-3,943639731	8,44E-29
CNTNAP1	-3,948293014	8,06E-29
LOC103908	-3,950246246	1,08E-08
MTCL1	-3,958062794	1,54E-37
SIRPA	-3,963746957	1,06E-61
ITGA5	-3,966979792	3,04E-35
BTBD11	-3,967748071	1,78E-46
BDKRB2	-3,970790664	5,94E-06
DUSP6	-3,971664901	7,12E-05
KIF7	-3,97757222	5,65E-06
SLC30A4	-3,979378683	3,33E-07
CMTM7	-3,98151949	1,02E-30
SLC12A4	-3,992752726	9,04E-12
MAPRE2	-4,001351375	3,94E-40
SEPT10	-4,006120047	8,34E-22
ITGA3	-4,006998377	1,73E-113
EHD2	-4,018918613	3,63E-84
ADAM19	-4,022231498	1,91E-30
GLRX	-4,02416803	7,41E-07
CEP112	-4,039273419	2,12E-27
CRIM1	-4,043659153	3,91E-45
C16orf62	-4,049034373	4,31E-68
DENND5B	-4,0635956	4,50E-50
SGK1	-4,07378676	2,14E-46
CD44	-4,083736839	8,90E-50
C3orf18	-4,091858401	2,78E-09
TRPM6	-4,092396644	1,24E-13
RHCG	-4,095978812	6,17E-06

MN1	-4,09650253	0,000147
GNAL	-4,096878116	2,40E-27
CNTNAP3	-4,099149313	1,32E-29
NPHS1	-4,100210583	1,31E-05
IGFBP3	-4,10389365	1,58E-13
ITGA6	-4,109396992	3,43E-61
CPM	-4,117761397	2,42E-14
MITF	-4,118916245	3,17E-09
ENO3	-4,121988012	6,11E-10
ELK3	-4,123011365	1,95E-32
COL12A1	-4,126485057	5,75E-12
NTN4	-4,131241592	7,83E-33
CCDC184	-4,131979799	1,88E-05
EEPD1	-4,133720514	2,32E-14
CDKL5	-4,135849714	1,52E-16
ANGPT1	-4,138301293	7,33E-09
LCAT	-4,138379148	0,000933
ENPP1	-4,139596745	8,85E-16
FCGRT	-4,145869481	9,20E-18
CYP4F11	-4,147368977	2,64E-27
EMILIN2	-4,147573431	4,83E-13
EVA1A	-4,149908226	1,86E-10
DLG4	-4,177388429	9,49E-22
NCALD	-4,178342658	4,34E-09
ANO1	-4,181963891	0,000254
FADS2	-4,184195261	2,25E-85
F2RL1	-4,188862756	1,93E-54
DDX11L2	-4,196771334	5,38E-08
STAMBPL1	-4,197036757	6,70E-16
CAV2	-4,199738379	1,31E-57
ARHGAP23	-4,214507694	7,35E-18
LPAR6	-4,217720476	0,000159
C1QL4	-4,217735797	0,000695
PFKFB3	-4,219438981	4,82E-35
AMIGO2	-4,220893607	5,90E-66
BMPR1B	-4,227171132	1,02E-35
TMEM92	-4,229577387	7,83E-06
FSIP1	-4,247423724	2,40E-05
RPS6KA6	-4,249901731	4,12E-20
CNNM1	-4,262017052	1,21E-42
RASSF9	-4,263893086	0,000787
FHOD3	-4,280046126	9,78E-12
ARL4C	-4,285823687	1,90E-86
VWA5B2	-4,295107998	1,86E-06
APOL6	-4,296268596	1,02E-17
ARHGAP26	-4,308991468	1,03E-20
SOX9	-4,310106382	1,97E-20
ANKRD2	-4,332213485	0,000215
FGG	-4,332348832	4,58E-06
C14orf159	-4,333034194	6,12E-31

CGNL1	-4,350600208	8,13E-11
LINC01503	-4,353108513	4,23E-05
CSF1	-4,376294191	1,44E-28
ARAP3	-4,376358071	5,08E-25
TMEM145	-4,377995423	0,000415
PDE4D	-4,384830128	2,59E-103
ADM	-4,386293708	1,11E-12
GULP1	-4,387066542	4,60E-44
CACNG8	-4,38836635	0,000294
FAM124A	-4,393565082	1,05E-09
MGAT5B	-4,396404788	3,39E-36
WIPF1	-4,399046052	1,32E-45
ADAMTSL4	-4,404205641	1,96E-05
VEGFC	-4,407339362	5,01E-15
RIN2	-4,41098654	8,27E-31
ADAM23	-4,41346179	1,61E-15
MPP1	-4,425101959	2,07E-12
PCDHB5	-4,436098047	2,29E-09
MSRB3	-4,453560616	2,09E-39
TGFBR2	-4,455673006	1,05E-56
XDH	-4,462934582	0,000418
GATA4	-4,463217266	2,15E-10
PALD1	-4,464240016	6,97E-41
DGKA	-4,464879871	1,29E-17
ZNF469	-4,47630182	2,16E-14
MAP1B	-4,478204013	7,23E-34
ATP8B3	-4,483401558	3,81E-07
FHL2	-4,491469625	7,89E-14
DHRS3	-4,497358442	5,79E-40
PELI2	-4,503902017	1,14E-05
NOSTRIN	-4,504813741	2,88E-07
LUCAT1	-4,541716743	1,24E-11
SH3PXD2A	-4,543728271	8,14E-33
LGR6	-4,5457003	2,80E-20
GLIPR1	-4,551357426	3,42E-15
IL22RA1	-4,584290192	3,67E-06
EPHA2	-4,584341597	3,01E-37
FAM189A1	-4,588598371	1,55E-06
TRIM47	-4,59280337	1,61E-55
PCDH9	-4,595923305	1,45E-28
ASB9	-4,610869248	2,49E-08
LOC101927	-4,613923064	5,11E-09
TESC	-4,614770977	6,10E-47
SYNGR4	-4,620900562	0,000245
C1R	-4,626927951	2,91E-28
AKAP12	-4,628432764	1,22E-53
HHEX	-4,646912652	3,95E-38
CEACAM6	-4,656351127	1,31E-11
PDE1A	-4,659451514	1,33E-06
PLCXD1	-4,662522566	3,19E-62

SERPINB9	-4,666016272	1,82E-35
HOXB6	-4,67624745	1,21E-09
MOB3B	-4,68930039	0,000123
DENND1C	-4,69105658	4,52E-07
TMEM98	-4,696192052	2,64E-27
WIPF3	-4,697817713	0,000326
ANKRD22	-4,700040355	0,000248
ST3GAL2	-4,702077903	3,72E-43
PDE6G	-4,705560347	0,000951
ROR1	-4,711343815	2,34E-57
LOC254896	-4,737500671	0,00089
FBN1	-4,739152737	4,54E-33
CALD1	-4,752491393	7,48E-22
APBB1	-4,759174686	2,09E-35
YOD1	-4,768046192	1,65E-18
ZNF513	-4,771614955	1,34E-18
HOXC8	-4,77486251	3,10E-12
AADAC	-4,791521802	0,000218
FZD8	-4,821675006	5,07E-17
TUBA4A	-4,825836587	3,28E-18
FLJ32255	-4,826651682	0,000306
LINC00511	-4,83872671	0,000492
PRG4	-4,843572731	0,000588
FOSL1	-4,855994872	1,80E-30
MYO1E	-4,862036159	2,18E-45
IGFBP4	-4,863326066	1,83E-48
ZC3H12C	-4,864732427	1,14E-21
SPRED1	-4,873618489	5,55E-36
TNS3	-4,876159595	2,49E-66
FILIP1	-4,889155254	2,23E-12
SLITRK5	-4,900275999	6,69E-06
LGR5	-4,90466065	1,98E-05
TRNP1	-4,907683685	4,84E-84
C17orf51	-4,912608374	8,28E-30
EPHA7	-4,926332645	0,0003
CIB2	-4,927251887	9,46E-28
MRC2	-4,942131918	5,35E-30
EPB41L2	-4,949476264	1,82E-59
CLDND2	-4,949997644	2,70E-07
BAAT	-4,952574781	1,68E-24
GNG2	-4,961084389	1,66E-06
C2orf72	-4,984297536	3,12E-06
GLDC	-4,999314527	3,29E-64
DNM1	-5,009728913	1,62E-117
AHNAK2	-5,01083406	2,39E-129
NEXN	-5,041253731	0,000669
LRRK1	-5,062718867	9,45E-52
RAB32	-5,071528699	5,22E-34
SEC16B	-5,079683588	0,000483
TBX1	-5,086569724	9,04E-06

TOR4A	-5,087949323	3,41E-71
TRIM6	-5,088648446	4,42E-12
PDE8B	-5,089873839	5,02E-11
RGS2	-5,099573838	3,27E-37
GNAI1	-5,101215221	4,63E-57
LINC01504	-5,10189969	0,000776
SLITRK6	-5,109039301	2,00E-05
SYT11	-5,114554225	4,66E-05
HOXA-AS2	-5,121041471	6,07E-11
PROCR	-5,127755256	4,57E-06
SVEP1	-5,128252191	3,71E-08
CYP26B1	-5,12909868	2,56E-16
INPP1	-5,133960236	1,55E-10
FOXA2	-5,139070616	4,27E-79
CAV1	-5,146903605	1,31E-75
SYCE1L	-5,189138835	7,63E-07
BNC2	-5,193839872	2,16E-05
UBASH3B	-5,196451679	7,58E-47
IL18	-5,201041254	7,18E-14
HAS2	-5,20391033	8,76E-06
CP	-5,209495278	5,67E-12
GRIP2	-5,237581865	4,61E-05
DOCK10	-5,238613614	2,19E-46
SYNE1	-5,2487767	4,61E-37
MESP1	-5,259297194	7,23E-06
ASPH	-5,26342544	6,67E-89
DPP4	-5,265530185	1,97E-07
F5	-5,26639012	4,82E-06
IGFBPL1	-5,272814994	7,45E-17
SMARCA1	-5,275462519	5,13E-47
C15orf52	-5,277560419	5,54E-06
SLC45A1	-5,282132771	3,94E-05
CRAT	-5,28348558	1,37E-38
HOXB13	-5,302716304	6,29E-17
ARHGAP22	-5,303264377	3,48E-10
CRYBG3	-5,30697382	3,04E-35
GJA1	-5,315714619	2,06E-20
PDE7B	-5,349900901	1,40E-17
LOXL2	-5,362994625	2,61E-22
ITGAM	-5,367517753	4,70E-05
PYGL	-5,374703568	3,74E-05
PMP22	-5,376227714	4,62E-42
TGFB2	-5,380370313	6,47E-23
MYH15	-5,401947588	7,15E-11
RIN1	-5,40662495	4,22E-43
LIX1L	-5,410749716	1,66E-67
COL5A2	-5,414736045	2,00E-110
SEMA3A	-5,416118552	4,81E-20
SLC2A3	-5,426355734	2,36E-17
LYN	-5,426862912	3,39E-42

MYO15B	-5,429259738	1,62E-74
LIMCH1	-5,431779462	2,95E-32
SIK1	-5,441931567	0,000206
RGS11	-5,443854081	0,000736
TBX20	-5,447140627	1,80E-06
PRRT3-AS1	-5,463219662	6,75E-07
CASP10	-5,469403367	1,39E-17
SRSF12	-5,493314578	7,24E-06
PPIEL	-5,518231932	0,000617
GPRIN3	-5,530299296	8,69E-12
TMEM9B-A	-5,532777695	2,75E-05
EDARADD	-5,553727325	6,78E-32
SYNM	-5,566381572	2,14E-92
RBP1	-5,573363979	5,94E-18
TNS4	-5,581355187	1,00E-27
ERO1B	-5,60099644	3,04E-14
RHBDL3	-5,650189984	4,64E-43
DNM3	-5,655870126	2,65E-16
SPX	-5,659962691	4,01E-05
MLLT11	-5,667002517	3,85E-57
CAPN2	-5,670270472	4,74E-42
DCBLD2	-5,676329047	2,20E-117
SPRY4	-5,700984728	4,16E-43
DKK3	-5,709782595	8,61E-51
SLC16A2	-5,719265742	7,47E-37
TMEM47	-5,724070138	0,000188
HAPLN3	-5,731573908	1,50E-09
SOX8	-5,772917776	1,89E-05
COL7A1	-5,773856552	3,24E-49
NRG2	-5,776025589	3,07E-09
CCDC106	-5,788410053	2,17E-46
C10orf54	-5,820207762	3,33E-11
TGFB1I1	-5,83269486	0,000104
MGAM	-5,837272163	0,000246
APOC1	-5,841799293	6,54E-50
ACOX2	-5,858947479	3,15E-06
EGFR	-5,859844023	7,43E-112
BIRC3	-5,878648899	8,00E-27
HLX	-5,902939267	0,000116
ABCC2	-5,903072623	1,22E-28
ABCC3	-5,911883854	0,00022
PCDHA3	-5,934717691	1,69E-06
ZDHHC2	-5,941140173	4,17E-33
PTGR1	-5,951789554	2,39E-79
JAG1	-5,970665306	1,55E-185
POPDC3	-5,975135042	1,63E-21
MMP24	-5,979359604	2,35E-31
ANKRD33B	-5,981206838	3,08E-22
SERPINE2	-5,98788729	1,86E-118
BST1	-6,021102274	5,88E-07

THSD1	-6,035623182	4,00E-12
PDE4B	-6,045034888	1,13E-05
TBXAS1	-6,045402514	3,78E-27
FRMD3	-6,112733586	3,60E-68
BRINP1	-6,112875983	2,03E-05
LINC00261	-6,148855206	1,64E-05
DUSP4	-6,195718621	2,95E-07
BIN1	-6,203394986	2,73E-61
ERICH5	-6,205648102	3,91E-09
IL15	-6,233309996	1,73E-07
PROS1	-6,246950822	7,31E-37
CYGB	-6,252998599	9,20E-06
EVA1C	-6,255369945	1,72E-85
DPYSL3	-6,27320185	7,64E-102
CECR7	-6,285196483	7,01E-06
TP63	-6,37955558	2,00E-08
MYEOV	-6,382456464	3,32E-96
CD14	-6,401759103	5,59E-06
TLE6	-6,411156897	4,56E-06
ALDH3B1	-6,415697168	6,12E-39
TRIML2	-6,416556644	3,47E-24
CD109	-6,441374472	3,29E-122
PAX7	-6,457543068	1,17E-06
PLA2G16	-6,458505735	1,90E-80
CA9	-6,466777632	1,19E-07
PLCL2	-6,480200568	4,44E-14
GPX2	-6,483178776	0,000121
STRA6	-6,490081489	5,10E-07
TRIM9	-6,490868066	3,87E-28
C2orf88	-6,508852622	0,00067
FOXC2	-6,519491036	0,000715
RBFOX3	-6,52960923	1,39E-29
ZNF717	-6,541011717	1,26E-06
CLMP	-6,547018334	2,22E-11
CACNG6	-6,549582193	2,47E-08
RASSF6	-6,56105742	3,07E-08
ZBTB18	-6,561148061	3,66E-40
PRKCA	-6,567368569	1,87E-147
SLC19A3	-6,568259056	5,29E-09
TM4SF1	-6,56888982	2,19E-106
CHGB	-6,587705647	4,71E-20
ME3	-6,588148088	8,33E-30
SERPINE1	-6,589784092	5,78E-05
SUCLG2-AS	-6,614966518	0,000452
AKR1C2	-6,641494988	1,62E-40
REREP3	-6,648333128	0,000407
ARHGAP31	-6,6500181	0,000425
PKDCC	-6,652533635	2,06E-36
ETV4	-6,65531902	1,94E-08
EID3	-6,671088906	0,000491

FSTL1	-6,671251218	1,45E-44
FLRT2	-6,678919565	0,000739
PLCE1	-6,704555929	2,25E-43
C10orf90	-6,713520999	0,000329
ZBTB20	-6,7420993	1,96E-20
CCDC144A	-6,746341878	0,000284
ETV5	-6,766815411	1,42E-67
CCDC80	-6,807263377	1,74E-71
C1S	-6,821408927	3,34E-24
EMP1	-6,839407612	1,63E-07
PLSCR4	-6,850534618	0,000207
HS3ST1	-6,859849049	0,000193
TSPAN18	-6,864334783	0,000229
VTN	-6,865183669	1,69E-07
BANK1	-6,86564829	0,000183
MYRF	-6,871827957	4,68E-06
KRT7	-6,904686532	7,60E-14
BEAN1	-6,948251832	0,00017
SFRP4	-6,950134084	1,05E-10
TNC	-6,968673651	5,41E-27
PNMA2	-7,002636207	5,54E-60
IGFBP6	-7,021136694	3,87E-59
BDNF	-7,064245195	9,66E-49
FAR2P2	-7,090796285	9,11E-05
FMN2	-7,093417355	7,57E-18
S100A4	-7,116218545	3,42E-87
HNF4A	-7,118130526	3,24E-41
CPA4	-7,125221766	4,80E-11
DAB2	-7,138272316	2,65E-37
KLHDC7A	-7,142069375	1,64E-08
TTLL6	-7,155134786	6,15E-08
GATM	-7,199631513	2,61E-12
CYP24A1	-7,202230368	5,53E-266
OSBPL3	-7,208629059	1,85E-43
CDH2	-7,223979277	9,31E-64
CACNG7	-7,230680156	4,27E-08
KIFC3	-7,235256138	9,20E-54
SOX21	-7,245743521	0,000947
EPHA5-AS1	-7,247370293	0,000965
LINC01234	-7,276812295	3,95E-38
PPEF1	-7,288467999	3,62E-05
AKR1C1	-7,289454746	6,32E-114
USP44	-7,302778445	0,000951
LACAT8	-7,305913028	0,000746
FAM27E3	-7,305913028	0,000746
LIPE	-7,323635321	3,62E-09
KCNT2	-7,330931367	0,000933
G0S2	-7,331861461	0,000774
BAIAP2L2	-7,332370284	5,16E-20
CRYM	-7,341162427	2,89E-05

PPP1R3C	-7,343759543	3,28E-09
SYT13	-7,351394606	5,22E-08
STAT4	-7,356978904	2,86E-09
RNF144A-A	-7,360971006	0,000606
LINC01085	-7,361271778	0,000594
LINC00488	-7,361872544	0,000584
PDE10A	-7,385855949	1,70E-22
GRIN2B	-7,386837039	0,000688
SLAMF7	-7,387432647	0,000614
RIMBP3	-7,390089849	0,000534
NRP2	-7,3968404	2,07E-90
KIAA2012	-7,414872246	0,000513
CYP27C1	-7,415452645	0,000482
ZC4H2	-7,420797113	2,91E-09
KIRREL3	-7,431565727	3,68E-05
ADAMTS16	-7,440655904	0,000514
PWARSN	-7,441228326	0,000467
LINC00312	-7,441228326	0,000467
ADAM12	-7,445126059	4,27E-25
ROBO1	-7,452791889	6,36E-96
PLA2G4A	-7,454732497	1,63E-06
NEK9	-7,464831776	1,23E-05
C3	-7,469417969	1,68E-35
CARD11	-7,470183145	0,00041
CRIP1	-7,470722303	9,40E-125
RAET1E	-7,494734541	0,000339
ARX	-7,499285634	6,61E-10
SLCO1B7	-7,517252788	0,000379
ZEB1	-7,517430625	1,67E-59
FGFBP1	-7,523757069	0,000717
NTS	-7,533749627	1,09E-21
ITGA1	-7,541871704	1,48E-05
HBQ1	-7,54315017	0,000284
VSX1	-7,543944858	0,000272
LOC101927	-7,545003279	0,000284
ACOT2	-7,549137071	5,83E-45
TIMD4	-7,566759991	0,000277
MAP3K7CL	-7,56988729	0,000277
LAMA5-AS1	-7,570410132	0,000305
BCHE	-7,592043801	0,00022
FXYD2	-7,59332369	0,000236
LINC01322	-7,594351563	0,000282
HOXB8	-7,596248149	2,87E-17
SOGA3	-7,599603896	3,16E-22
IFI16	-7,615562927	5,13E-10
SBK3	-7,615630874	0,000199
BMPER	-7,616638146	0,00021
USP32P1	-7,621294745	9,24E-06
MYOM2	-7,637349142	0,000199
LOC102724	-7,638590745	0,00018

MAGI2-AS3	-7,639086233	0,000181
NID2	-7,658486407	0,000257
NCMAP	-7,664374325	0,000257
FAM133A	-7,682237847	0,000168
CSGALNAC	-7,682496705	2,39E-31
HAND2	-7,683202137	0,00015
CCK	-7,683442508	0,000148
FOXQ1	-7,692379857	6,15E-23
LINC00473	-7,705433091	7,29E-15
TMSB4Y	-7,706065695	0,000133
TLR3	-7,724113798	0,000229
PEAR1	-7,726369654	1,34E-05
IGFBP1	-7,728106794	0,000123
PACERR	-7,747748726	0,000118
SLC4A4	-7,76341504	1,04E-23
ITIH5	-7,788703477	0,000115
EPAS1	-7,80885744	2,81E-126
RARRES1	-7,812613071	9,34E-05
MRAP2	-7,812613071	9,34E-05
UGT2B7	-7,813276218	0,000108
KLHL4	-7,816482095	5,85E-11
KRT81	-7,837121632	2,38E-14
SLC10A4	-7,842891571	5,54E-11
RARA-AS1	-7,848132803	0,000124
LINC01273	-7,852008825	7,09E-05
GGT3P	-7,852008825	7,09E-05
DSC3	-7,859706768	2,89E-11
PRDM1	-7,867454106	6,28E-11
UNC5D	-7,871944354	6,71E-05
GALNT9	-7,873216801	8,79E-05
SPARC	-7,88994262	5,73E-05
SLC6A17	-7,903648304	3,36E-11
MYL9	-7,906255586	9,56E-05
C1QTNF1	-7,91069587	1,71E-05
GACAT2	-7,912045102	8,28E-05
KIAA1644	-7,92872953	4,84E-05
TNFAIP2	-7,942410039	8,38E-77
PHYHIP	-7,947839427	4,67E-05
ANXA1	-7,955155203	6,04E-278
AGMO	-7,96551353	4,02E-05
ANKS4B	-7,967297183	5,38E-05
SP9	-7,977789235	1,43E-25
LONRF3	-7,981235969	1,83E-11
F2	-7,983949902	3,75E-05
ACOT1	-8,014706272	1,60E-06
LOC101928	-8,019559694	3,15E-05
MAP7D2	-8,031144442	3,69E-27
EPS8L3	-8,037134373	2,92E-05
CA13	-8,038455664	3,77E-05
HACD4	-8,053196698	2,65E-05

ITGA11	-8,053568538	2,60E-05
PLA1A	-8,05580379	3,54E-05
KCNE4	-8,070736124	2,39E-05
ZNF215	-8,07110328	2,40E-05
LAMA4	-8,090992024	4,76E-05
HOXB3	-8,099851541	5,56E-17
MIR100HG	-8,117269755	1,03E-06
GREM2	-8,120158921	1,94E-05
FST	-8,16499402	8,16E-07
DAPK1	-8,176052154	3,60E-54
ST6GALNA(	-8,198657518	1,70E-05
SCG2	-8,200848001	1,26E-05
IQGAP2	-8,208421363	9,15E-53
SLC6A15	-8,215028872	1,26E-05
TMBIM1	-8,232491251	1,62E-76
TMEM220	-8,24591235	1,02E-05
RAB38	-8,246725273	1,01E-05
GPC6	-8,252496186	4,76E-18
LOC10013(	-8,275829139	8,70E-06
LINC01186	-8,289768258	8,76E-06
ZNF583	-8,291819748	8,80E-06
PDCD6IPP2	-8,320190229	7,12E-06
SYT15	-8,321119813	8,41E-06
C11orf70	-8,324609999	4,15E-07
CNTNAP3B	-8,32588655	2,71E-23
EBI3	-8,334322313	6,57E-06
SERPINB4	-8,336479034	1,16E-05
MICU3	-8,34755975	6,07E-06
NPR2	-8,359406854	3,50E-07
ZFP82	-8,360372423	6,83E-06
AFAP1L1	-8,372287285	4,69E-13
LINC00857	-8,384822578	4,38E-13
SNPH	-8,397843315	6,82E-19
PLCXD3	-8,404146444	5,25E-06
TFCP2	-8,42625713	9,65E-05
POU4F1	-8,454231236	4,42E-06
THY1	-8,467228889	2,46E-07
BATF3	-8,47958685	1,87E-07
HTR2B	-8,495562203	3,28E-06
CLIP4	-8,509495607	1,11E-31
TFPI2	-8,52259241	1,39E-06
CXCL3	-8,522924849	2,01E-07
C1RL	-8,530600367	4,99E-25
LOC10192(	-8,531202797	2,38E-06
RNF212	-8,554875316	2,45E-06
LOC72997(	-8,5559286	2,05E-06
LOC101927	-8,557372403	2,31E-06
ETV1	-8,5629529	1,56E-07
PSMB8	-8,565253127	3,78E-23
PROC	-8,581788514	2,26E-06

CRTAC1	-8,582309288	2,70E-06
HHIP-AS1	-8,591792336	1,70E-06
CSF2RA	-8,601846264	2,46E-06
SUSD5	-8,603127675	1,69E-06
MKRN3	-8,604016801	1,58E-06
GGT8P	-8,604016801	1,58E-06
TMEM156	-8,610289905	5,81E-14
CCAT1	-8,616390614	1,59E-06
ADH6	-8,618296671	3,02E-06
ST6GAL2	-8,629270537	2,59E-07
CDA	-8,649200653	1,36E-06
ANKRD30A	-8,651292629	1,44E-06
ADRA1B	-8,660828986	1,22E-06
LHX8	-8,661926718	1,21E-06
PHLDB2	-8,666796947	3,99E-41
RUNX3	-8,668516815	1,22E-26
NR1H4	-8,675780159	2,90E-06
FOXL2NB	-8,685009153	1,23E-06
PEG10	-8,719858816	2,39E-16
C8orf88	-8,727062328	8,46E-07
HPGD	-8,728782301	5,98E-87
GAPLINC	-8,728811822	1,00E-06
BICC1	-8,748010139	3,01E-149
EREG	-8,751175328	5,94E-09
ITGAX	-8,760554909	8,04E-07
CD70	-8,765112996	2,92E-14
AK5	-8,779874871	6,42E-07
EDNRA	-8,782354229	9,73E-07
TGM2	-8,798065812	8,29E-91
STAC	-8,800603197	5,67E-07
TNFRSF9	-8,80104604	5,59E-07
PRDM13	-8,80104604	5,59E-07
FBLN1	-8,803525261	1,16E-06
EBF1	-8,820928644	5,09E-07
ZFPM2-AS1	-8,830387157	5,30E-07
MMP2	-8,834852678	6,24E-21
PLAT	-8,840971701	4,57E-07
ALX4	-8,850836266	4,35E-07
P3H3	-8,851379777	2,34E-08
MAP1LC3A	-8,859887349	4,82E-07
CTSZ	-8,867022957	4,07E-85
TRPA1	-8,871631442	4,13E-07
RRN3P1	-8,880136455	3,74E-07
ITIH2	-8,892139589	5,30E-07
ZNF559	-8,899171804	3,47E-07
CYB5R2	-8,899378956	3,38E-07
FAM71E1	-8,910705087	3,92E-07
COL4A2	-8,916554407	1,74E-91
LOC38828[illegible]	-8,933823823	1,18E-06
GLIS3	-8,959238903	9,17E-07

C11orf45	-8,964924019	2,33E-07
FBXL7	-8,965417983	2,33E-07
SLIT3	-8,9724057	6,65E-28
PADI1	-8,983075101	2,11E-07
KCNJ2	-8,992889946	2,08E-07
A1CF	-9,00128975	1,90E-07
PTPN20	-9,008580162	2,47E-07
ZNF542P	-9,009160258	2,06E-07
FAM43B	-9,027146572	1,73E-07
CHMP1B2F	-9,02724138	1,71E-07
UCHL1	-9,034402942	9,22E-19
RNF217	-9,039675476	1,73E-22
SLC7A7	-9,042404092	1,03E-20
MAGEA6	-9,057130583	1,22E-08
STK33	-9,062457818	1,37E-07
ADRA1D	-9,065270963	1,20E-08
ZNF502	-9,095209486	1,25E-07
NRIP3	-9,097016793	1,29E-07
USH1C	-9,104680196	1,57E-08
LBH	-9,124984763	3,79E-15
TPM2	-9,126228074	1,60E-169
CD40	-9,128471775	9,90E-08
SDPR	-9,131191014	2,15E-37
APOBEC3C	-9,1376624	1,01E-49
SCN9A	-9,149019049	4,56E-16
LOC10272З	-9,16253964	9,96E-08
STEAP2	-9,167211822	6,36E-07
KCNJ16	-9,168719653	7,86E-08
KRT222	-9,170697851	1,03E-07
CFI	-9,177609158	7,52E-08
DDC	-9,184917147	6,96E-08
GCNT2	-9,20718121	9,08E-24
ARSJ	-9,223655664	8,10E-24
UGT1A7	-9,223889009	5,66E-08
TMEFF2	-9,225300193	8,18E-08
TM4SF20	-9,229726169	7,54E-08
PRKCDBP	-9,231706618	5,53E-08
SOX17	-9,238909799	5,17E-08
GLI2	-9,251194125	4,86E-38
ALDH2	-9,25733174	1,56E-104
ZNF71	-9,260292845	1,24E-16
ALX1	-9,262644343	5,86E-08
GREM1	-9,266956527	6,87E-08
PWAR5	-9,304592837	3,85E-08
SMO	-9,310849575	2,83E-24
DGKG	-9,315766108	1,41E-16
LAYN	-9,318332217	4,24E-08
IGF2BP1	-9,31892959	1,23E-130
UGT1A1	-9,321126023	4,58E-08
KCNK3	-9,324047351	1,73E-16

CNTNAP3P	-9,376057634	2,49E-08
SLC17A3	-9,38928707	2,25E-08
GABRE	-9,392295329	1,36E-07
AADACP1	-9,402834302	2,09E-08
FAT3	-9,409306186	2,04E-08
AKR1C3	-9,415412884	2,41E-07
CYP3A5	-9,425351598	4,17E-08
FGF2	-9,428118658	9,77E-33
PAX6	-9,435334076	1,95E-08
PCSK5	-9,435813317	1,50E-32
SALL1	-9,443581814	1,85E-08
ANXA8L1	-9,448599742	1,75E-08
ANKRD1	-9,456132063	5,26E-09
MDFIC	-9,465443859	1,89E-25
TGFBI	-9,47634008	4,67E-91
ZEB2	-9,486431652	1,87E-08
C1RL-AS1	-9,487541355	1,34E-08
SRPX2	-9,487747995	1,31E-08
MID1	-9,500584196	3,81E-173
C11orf86	-9,514786358	1,70E-08
CDH6	-9,553737777	1,20E-09
APBB1IP	-9,569900958	1,25E-08
LINC00941	-9,573583132	8,81E-09
PRSS3	-9,588228246	1,39E-08
TLE4	-9,59223833	1,05E-09
CXCL1	-9,593592277	1,02E-08
FIGN	-9,597087297	8,26E-09
PAQR9	-9,603255374	7,38E-09
FLNC	-9,606818776	2,33E-08
BARX1	-9,615006058	6,82E-09
RBP4	-9,626473573	6,57E-09
LOC102723	-9,632890656	5,82E-09
TNFSF12	-9,645206999	6,08E-09
CFH	-9,660527206	2,31E-18
HOXB5	-9,666242625	5,36E-09
C7orf31	-9,67187897	5,15E-09
GJC1	-9,682204611	1,77E-34
SMIM6	-9,684711463	5,08E-09
PDK4	-9,713030876	5,75E-06
ZC3HAV1L	-9,742402579	3,97E-09
ANPEP	-9,75695175	9,52E-09
COL4A1	-9,757136416	5,29E-27
ARSI	-9,765097835	2,87E-09
MT1A	-9,775775619	2,82E-09
FYB	-9,780018712	3,46E-10
VCAN	-9,80814943	8,53E-141
MSC-AS1	-9,820600242	2,21E-09
ZFP28	-9,839885432	2,60E-09
UPK1B	-9,84161919	1,83E-09
FAM230C	-9,866674858	1,72E-09

LINC01279	-9,879503225	1,79E-09
AGPS	-9,880127021	1,94E-60
TTTY15	-9,894878453	1,34E-09
GIP	-9,895345964	1,36E-09
UTY	-9,903553015	1,54E-09
UGT1A9	-9,915418962	1,66E-09
NALCN	-9,922949061	1,20E-09
SMOC1	-9,923153276	1,16E-09
EMP3	-9,960856511	2,63E-93
PCDHA4	-9,980906633	1,76E-09
LINC00842	-9,982437485	9,46E-10
S100A3	-10,00449068	1,11E-10
PRKY	-10,04074852	5,80E-10
JAM3	-10,04093639	5,82E-10
FOXL2	-10,05695026	6,56E-10
BVES	-10,0574645	5,58E-10
RGS20	-10,06646671	5,02E-10
LINC01021	-10,07055543	4,95E-10
LINC00707	-10,07323942	8,72E-10
PHYHIPL	-10,08802376	4,55E-10
LINC00667	-10,09187049	4,34E-10
LSAMP	-10,10670336	1,06E-09
TIMP4	-10,11022886	6,54E-11
NLGN4Y	-10,13313786	3,44E-10
SAMD5	-10,13460119	5,32E-10
ZNF470	-10,14080425	3,39E-10
ADAMTS10	-10,14194518	3,69E-10
PPP1R16B	-10,14367084	5,98E-11
ZNF175	-10,14908449	3,16E-10
CXCL2	-10,14912809	3,15E-10
SLCO2B1	-10,16115682	2,94E-10
ADGRE1	-10,16250207	3,98E-10
ZNF300	-10,18084231	2,64E-10
SLC23A1	-10,19583073	1,13E-09
TNFSF15	-10,20061307	2,54E-09
MUC13	-10,20250987	4,71E-10
DACT2	-10,23576662	2,14E-10
GDA	-10,2373529	4,98E-10
ZNF773	-10,24241638	1,86E-10
MALL	-10,25275207	6,19E-10
IPW	-10,25773146	1,69E-10
CDIP1	-10,26099018	1,69E-10
SRGN	-10,26133041	2,94E-11
FOXA3	-10,27780014	2,64E-10
SGK2	-10,30895757	1,26E-10
C14orf105	-10,32184736	2,99E-10
RSPO3	-10,33603593	1,04E-66
LYNX1	-10,33688025	1,13E-10
SLFN13	-10,34088249	1,05E-10
ZNF229	-10,38899147	8,14E-11

HABP2	-10,42622588	4,03E-10
SCARA5	-10,43041705	7,30E-11
HRH1	-10,44255101	5,92E-11
NKX2-5	-10,46244582	5,27E-11
INSL4	-10,46625618	5,77E-11
MIR31HG	-10,46935563	5,47E-11
NID1	-10,51339309	3,97E-11
HHIP	-10,52829952	3,71E-11
HKDC1	-10,5345566	3,27E-32
PPP1R14A	-10,56323942	6,74E-12
SPP1	-10,58202798	2,70E-07
LIN28B	-10,58297431	2,67E-11
HOXA10	-10,58407519	3,06E-11
ZNF280A	-10,59291822	2,85E-11
EPHA5	-10,60376381	2,34E-11
TWIST2	-10,61953992	2,72E-11
MPV17L	-10,62536166	2,67E-11
PLD5	-10,62542522	2,75E-11
LDHB	-10,65127597	0,000297
CPLX2	-10,65303675	1,10E-26
BCO1	-10,67620575	1,79E-11
ALDH3A1	-10,683511	1,28E-22
DLL3	-10,69248262	1,47E-11
FOXE1	-10,70284658	1,34E-11
SLC35G2	-10,72417456	1,29E-11
ZNF280B	-10,73983239	1,39E-11
WT1	-10,76237429	9,62E-12
CACNA1G	-10,76555783	1,11E-11
LGSN	-10,77219283	9,15E-12
DPYD	-10,78312885	8,43E-12
SEC14L4	-10,79373123	8,31E-12
HECW1	-10,79409667	9,31E-12
PRKCQ-AS1	-10,80135362	7,83E-12
LOC100505	-10,81736515	9,35E-12
GLP2R	-10,83970972	1,06E-11
MARCH4	-10,8692058	9,31E-12
GSTP1	-10,87639444	7,46E-164
MSN	-10,88627819	1,01E-10
TCF4	-10,89738944	4,33E-12
HORMAD1	-10,9094453	4,06E-12
CARD6	-10,91273555	5,16E-12
GALNT5	-10,95505611	3,91E-12
ANO5	-10,98368756	2,72E-12
NT5E	-10,9877719	2,57E-12
HNF1A	-10,98885261	3,32E-12
MSC	-10,99060587	4,91E-12
PRKCQ	-11,00141147	2,38E-12
ZFHX4	-11,01083736	3,81E-12
CDH4	-11,02203664	3,18E-12
SPAG16	-11,0352691	2,51E-12

GNG11	-11,04032699	1,76E-24
LOC400655	-11,05736734	1,90E-12
ZFY	-11,07287044	1,56E-12
ATP10A	-11,07707844	1,52E-12
GAL3ST1	-11,07936262	4,41E-12
IGFBP7	-11,1277483	1,68E-12
SLC35F3	-11,16470184	9,76E-13
AXL	-11,16934016	1,47E-35
DEPDC7	-11,17660211	9,06E-13
NPY4R	-11,18765614	9,19E-13
TLR6	-11,24348572	7,09E-13
KDM5D	-11,2745397	4,89E-13
FOXF2	-11,29181065	5,08E-13
ASB4	-11,3007182	5,08E-13
HMGA2	-11,30877407	1,24E-13
USP9Y	-11,32230084	4,47E-13
THSD7A	-11,33877423	5,49E-13
SFRP5	-11,33900402	3,23E-12
NEFL	-11,34830377	3,28E-13
GSPT2	-11,34983341	3,13E-13
ZBED2	-11,35174828	4,91E-12
C2CD2	-11,35264011	3,27E-13
APOH	-11,35873688	8,18E-13
OSR1	-11,37490798	7,47E-14
GPRIN2	-11,37591047	2,71E-13
PDE1C	-11,38095019	2,60E-13
C5orf42	-11,44782549	1,75E-13
ANXA13	-11,52101099	9,06E-13
DFNA5	-11,53291566	1,10E-13
TXLNGY	-11,62972842	6,12E-14
TSPAN7	-11,63219993	6,74E-14
ZNF518B	-11,6437141	6,24E-14
SDHAF3	-11,6524805	5,50E-14
CDH17	-11,66287943	2,99E-13
PON3	-11,66550951	7,60E-14
TDRP	-11,66571392	4,80E-14
FGL1	-11,67172881	1,14E-13
FGA	-11,69539062	4,44E-14
CES1	-11,71254828	5,80E-14
FRG1CP	-11,7328433	3,20E-14
NIPAL4	-11,76511532	2,81E-14
CPNE8	-11,81559621	2,23E-14
TNFRSF10D	-11,88317218	1,33E-14
MYOM3	-11,91812856	2,31E-14
ETS1	-11,94245703	1,16E-14
PDE3A	-11,95003361	8,91E-15
PNMAL1	-11,95929039	1,11E-14
SLCO1B3	-11,98506365	7,71E-15
GALNT13	-12,01346458	5,95E-15
EVC	-12,0820133	4,53E-15

TCEAL8	-12,09028266	4,10E-15
ABCA1	-12,09918558	3,54E-15
AKR1B10	-12,13610948	2,37E-29
PTGS2	-12,15652632	6,15E-57
EIF1AY	-12,16519943	2,37E-15
VGLL3	-12,17483744	2,63E-15
SGCD	-12,18297354	2,85E-15
SFRP1	-12,21872758	2,48E-15
EYA4	-12,22044304	1,75E-15
CNKSR2	-12,39211203	6,72E-16
COL4A6	-12,46119536	1,51E-16
LPAR1	-12,47125555	3,64E-16
CYBRD1	-12,47597273	3,52E-16
SIGMAR1	-12,49942016	1,55E-31
ZCCHC11	-12,51813002	2,82E-16
IGF2BP3	-12,54857538	2,26E-16
SGCE	-12,58165204	1,85E-16
ALPK2	-12,58921504	3,33E-16
PDGFD	-12,61463782	1,65E-16
DZIP1	-12,63323567	1,39E-16
F2RL2	-12,63460828	1,36E-16
NR0B1	-12,64145996	1,48E-16
WBP5	-12,67251918	1,20E-16
GABRA5	-12,81484126	5,28E-17
CNN3	-12,83149393	1,50E-17
GNE	-12,85254518	3,43E-17
NNMT	-12,87156386	3,34E-17
HAVCR1	-12,88142234	2,92E-17
AKT3	-12,88683176	2,91E-17
GABRB3	-12,90253532	1,03E-17
PLAU	-12,90527539	2,69E-17
TMEM246	-12,9065904	2,51E-17
STK32B	-12,98713566	1,59E-17
TM4SF18	-12,99579493	2,38E-17
TBX18	-13,02883031	1,29E-17
PTPRM	-13,12770682	7,57E-18
PVRL3	-13,19559675	4,23E-18
AKR1B1	-13,30521402	7,67E-100
DDX3Y	-13,30780865	2,22E-18
MMP7	-13,36861061	4,55E-18
KIRREL	-13,51470088	6,40E-19
GPX1	-13,58806295	3,90E-19
NLRP2	-13,61670414	2,77E-19
INA	-13,64222856	2,33E-19
CXCL5	-13,66109354	2,46E-19
ARSE	-13,79658144	1,26E-19
SLC22A3	-13,97683561	2,51E-20
GRAMD1B	-14,03928964	1,97E-20
ADD2	-14,06950793	1,37E-20
GCNT3	-14,22440669	7,12E-21

HNF1B	-14,24690845	6,21E-21
PAPPA	-14,37392932	1,67E-21
EMC10	-14,38449147	1,57E-21
CNTN1	-14,45039623	6,09E-22
NRG1	-14,81406133	1,16E-22
NTRK3	-15,07357431	1,34E-23
RPS4Y1	-15,10364072	1,18E-23
ALDH1A1	-15,1695285	8,67E-128
CLDN2	-16,1057909	3,50E-26

CCRF CEM

Gene	log2FoldCh	FDR
KRT18	18,51615	1,26E-35
KRT19	18,18038	1,02E-32
DSP	17,42377	4,18E-31
CYP1B1	17,15096	3,83E-14
CDH1	16,3574	2,10E-27
BMP7	15,89041	4,39E-26
LAPTM4B	15,85446	5,87E-26
KRT8	15,63682	2,38E-200
SEMA3C	15,56502	4,68E-25
MYH14	15,46491	8,54E-24
BCAR1	15,45553	7,72E-24
S100A16	15,43406	7,64E-24
TRPS1	15,42867	1,44E-24
LAMC1	15,35392	2,52E-24
S100A14	15,31161	1,63E-23
FREM2	15,26203	4,59E-24
TFAP2A	15,19738	6,65E-24
SULF2	15,07961	1,51E-24
FBP1	15,05246	2,82E-23
AHR	15,02705	2,51E-23
EMP2	14,99027	1,10E-22
MAL2	14,98054	3,29E-23
AP1M2	14,9564	4,98E-23
STC2	14,92736	9,19E-08
CDC42EP1	14,86987	7,01E-13
TPD52L1	14,80713	5,09E-22
PTPRS	14,72197	2,29E-22
TFF1	14,71965	3,13E-21
CLDN3	14,69582	2,92E-22
PKP3	14,69019	5,58E-22
SP5	14,67589	5,96E-22
LAD1	14,6423	5,78E-22
LDOC1	14,64139	7,33E-22
ST14	14,56582	1,90E-21
PRICKLE2	14,51747	9,96E-21
TSPYL5	14,51162	8,29E-22
CD276	14,47934	2,25E-21
SERPINB6	14,44954	1,27E-21
TFAP2C	14,44625	2,00E-21
LTBR	14,36799	2,15E-21
TACSTD2	14,35407	3,49E-21
LAMA5	14,31598	3,08E-21
LINC00674	14,23226	9,80E-21
GRHL2	14,21003	1,53E-20
RAB25	14,16411	1,17E-20
C3orf14	14,11293	1,98E-20
NR2F2	14,08434	1,96E-20
TNFRSF19	14,08171	2,39E-20

STC1	14,05315	3,32E-20
ZCCHC14	14,02077	2,33E-20
MYO5B	14,01732	3,44E-20
DSC2	13,97851	4,09E-20
SPDEF	13,92611	6,24E-11
SYTL2	13,90274	7,99E-20
GDF15	13,88992	1,22E-12
TJP1	13,88286	1,30E-19
PRSS23	13,86001	7,94E-26
EPN3	13,85371	9,79E-20
PPP2R2C	13,8101	9,37E-20
MISP	13,76527	1,33E-18
RNF130	13,76028	1,89E-19
MGST1	13,74427	2,34E-19
NFIB	13,68009	3,10E-19
ATP2C2	13,65958	1,21E-18
DKK1	13,65883	8,06E-09
PLS3	13,6093	4,26E-19
ADRA2C	13,56806	9,32E-19
ME1	13,5165	3,85E-18
SOX13	13,50967	3,79E-18
LRP3	13,46739	1,05E-18
CCND1	13,4587	1,47E-65
FAM83H-A	13,41254	1,24E-18
SRXN1	13,40231	8,37E-18
TMEM30B	13,37273	2,09E-18
ZNF503	13,30989	2,55E-18
RBM47	13,30335	5,09E-18
MSI1	13,29654	3,83E-18
CTBP2	13,29073	1,78E-19
IMPACT	13,27893	4,06E-18
DOCK6	13,27273	3,69E-18
CBR1	13,27125	5,77E-18
BGN	13,25814	3,18E-06
GYLTL1B	13,2481	7,84E-18
TEAD1	13,24295	3,80E-18
KITLG	13,22645	5,11E-18
SLC7A2	13,22341	6,19E-18
DSCAM-AS	13,21847	9,67E-11
RHOD	13,19143	2,26E-17
AGAP1	13,12737	9,72E-18
MAP7	13,12558	8,01E-18
MSX2	13,07628	2,74E-17
PMEPA1	13,06526	1,78E-17
HOXC10	13,05353	1,51E-17
SELENBP1	13,04542	1,84E-17
HSPB8	12,99472	6,28E-17
GAL	12,98983	1,92E-17
BAIAP2L1	12,95784	2,63E-17
CYB5A	12,92483	5,78E-17

MLPH	12,89886	3,87E-17
FAM127C	12,87746	4,40E-17
PBX1	12,86361	5,11E-16
GGT6	12,84646	6,51E-16
BASP1	12,83757	3,64E-09
WDR72	12,83035	5,63E-17
SYT12	12,8302	2,60E-16
PTK2	12,8292	3,19E-18
KCNJ8	12,82906	6,51E-17
PCDHA6	12,80737	6,76E-17
AFAP1	12,79925	1,01E-16
PRSS8	12,76838	4,74E-18
INADL	12,7615	8,60E-17
FGFR4	12,76046	1,59E-17
TBX3	12,70983	1,21E-16
PRLR	12,69977	1,62E-16
CA12	12,68942	4,37E-09
ACSS3	12,67222	9,19E-18
LINGO1	12,67138	1,64E-16
KCNG1	12,66449	1,19E-15
PRR15L	12,6635	3,89E-16
EEF1A2	12,63661	1,84E-125
PKP2	12,61244	9,44E-16
PPP1R26	12,59384	3,25E-16
IRX2	12,5883	2,89E-16
MARVELD3	12,56409	1,42E-15
HTRA1	12,56293	3,30E-16
CYP1A1	12,54748	8,88E-17
FXYD3	12,53046	5,64E-09
SHROOM3	12,50753	4,13E-16
SPTSSB	12,4645	6,87E-15
DYNC1I1	12,46434	5,41E-16
ELF3	12,45258	1,32E-14
CTSL	12,4488	2,08E-15
PPP1R9A	12,4464	7,55E-16
ARHGAP39	12,4458	1,18E-15
KIF1A	12,42522	7,76E-16
COLEC12	12,41457	5,75E-15
CDH3	12,39311	1,03E-57
IGFBP5	12,38006	2,49E-15
LRP2	12,37994	3,22E-05
APCDD1	12,37845	1,61E-81
NEBL	12,36335	1,17E-15
AREG	12,36208	7,21E-15
SYK	12,35697	1,18E-15
PLEKHA6	12,33736	1,33E-15
ESR1	12,33191	9,28E-17
THBS1	12,329	7,69E-15
GNG12	12,32498	1,51E-15
DUSP23	12,32405	1,34E-15

SH3D19	12,31592	1,45E-15
PLOD2	12,31197	1,40E-15
KIAA1671	12,30931	1,54E-15
CACNG4	12,30361	1,89E-15
DCLK1	12,29887	1,69E-15
STEAP3	12,29656	2,72E-15
KYNU	12,28447	7,42E-15
RHBDF1	12,28426	1,64E-15
GPD1L	12,26612	2,55E-15
SPG20	12,25089	2,13E-15
ADIRF	12,23746	5,59E-14
CCDC50	12,231	3,37E-15
KRTAP5-AS	12,21872	1,40E-05
ANK3	12,21383	3,37E-15
SIX4	12,20777	3,07E-15
PCDH18	12,19331	9,70E-14
CLU	12,19203	5,13E-19
LOC101927	12,18592	1,07E-14
NEK6	12,18512	3,33E-15
PCDHA11	12,1819	5,22E-15
SECTM1	12,18019	6,15E-14
DIRAS1	12,17753	3,32E-15
MARC1	12,1759	3,24E-15
SDC1	12,17182	2,56E-16
TSPAN6	12,16036	3,58E-15
CADM1	12,13663	4,53E-15
PCLO	12,13489	5,54E-15
IL13RA1	12,12479	6,70E-15
RBBP8NL	12,12283	7,21E-15
PPL	12,12137	1,79E-27
DTNA	12,09743	1,05E-14
DLX5	12,07542	7,16E-15
UNC13B	12,07404	9,77E-15
LIN7A	12,06416	1,11E-14
STARD13	12,06099	1,17E-14
FAM83B	12,05878	6,78E-15
C15orf59	12,05288	3,50E-14
C9orf152	12,04599	1,99E-14
FBXO27	12,03307	8,44E-15
MYOF	12,03072	1,69E-26
GALNT16	12,0229	1,24E-05
PCDH1	12,01945	1,16E-14
PERP	12,0093	1,98E-15
NHS	12,00149	3,13E-14
CCDC170	12,00096	1,57E-13
NEB	11,984	1,07E-14
SLC1A2	11,98377	1,18E-14
ARHGEF5	11,97137	2,04E-14
THEM4	11,96514	1,17E-14
C14orf132	11,93543	1,44E-14

IGSF9	11,93418	2,78E-28
PCSK1N	11,9307	1,56E-14
ALDH3A2	11,92995	2,10E-28
RASEF	11,92785	1,69E-14
OLFM1	11,92128	1,55E-14
KLF11	11,90703	2,70E-13
PLK2	11,83897	8,42E-14
VWA1	11,83851	4,59E-14
SKAP2	11,83813	3,05E-14
WNT5A	11,81851	6,78E-14
NUDT16	11,81696	4,63E-14
GPX8	11,81012	3,95E-14
FAM50B	11,80919	3,31E-14
HOOK1	11,8078	3,13E-14
PVRL4	11,7956	3,37E-14
SERTAD4	11,79059	7,81E-14
ABCA3	11,78856	1,21E-25
NPY1R	11,78779	5,34E-14
ZIC1	11,77298	1,02E-13
EPHB2	11,76664	4,01E-14
OAT	11,76459	2,02E-13
ERICH2	11,76307	1,29E-13
CAP2	11,76027	4,22E-14
RAB3IL1	11,744	2,74E-13
CHMP4C	11,73214	1,10E-13
CLDN4	11,73026	7,62E-26
SNX9	11,72336	5,66E-14
ASAP2	11,71522	3,46E-15
ESPN	11,71433	5,35E-13
ARFGEF3	11,71012	1,23E-13
DOCK1	11,7069	7,71E-14
DIP2C	11,69664	6,18E-14
ALOX15	11,68684	1,52E-12
KDF1	11,6769	8,54E-14
ERRFI1	11,67351	2,36E-13
KIF16B	11,66894	1,46E-13
PSD3	11,66843	8,10E-14
GPRC5C	11,66807	8,63E-14
AR	11,65388	8,40E-14
CEMIP	11,6441	2,39E-13
GRHL3	11,6283	1,35E-13
P2RY2	11,60711	1,14E-14
TBX2	11,58989	1,22E-13
PARVA	11,57995	1,77E-13
SMIM22	11,57988	1,76E-13
NUPR1	11,5793	5,74E-10
CYP2J2	11,55625	4,12E-13
IRS2	11,53132	7,70E-13
SLC2A8	11,51409	3,44E-13
MECOM	11,51187	2,16E-13

TMEM37	11,50285	2,72E-13
CC2D2A	11,49915	2,54E-13
MATN2	11,48804	2,66E-13
TMEM54	11,48792	1,30E-14
ITPRIPL2	11,47796	2,64E-13
WNT7B	11,47671	6,82E-13
IRF6	11,46702	3,36E-13
PALM	11,46217	3,03E-13
THSD4	11,46106	4,68E-13
ARNT2	11,45887	3,23E-13
EPHB3	11,45768	4,42E-13
C5orf38	11,45545	4,87E-13
PCP4	11,44742	3,34E-13
SDC2	11,43898	3,10E-13
INHBB	11,42692	4,73E-13
MNX1-AS1	11,41911	8,91E-13
RASAL2	11,41693	3,64E-13
P2RY6	11,4105	0,000213
CCDC149	11,40415	4,26E-13
RHOB	11,38919	4,37E-89
ETNK2	11,3746	9,74E-14
SCARA3	11,35396	7,84E-13
FAM84B	11,34459	6,89E-33
SRGAP1	11,33887	1,06E-12
OVOL1	11,33452	1,73E-12
WASF3	11,32761	5,91E-13
NUDT12	11,32577	7,52E-13
SYT17	11,318	6,26E-13
ENTPD2	11,30987	1,69E-12
GRTP1	11,29903	9,32E-13
EFS	11,2736	9,71E-13
CRMP1	11,2659	1,20E-12
KRT7	11,26375	3,53E-11
TDRD1	11,25549	9,36E-13
ILDR1	11,25003	9,90E-13
PHLDA2	11,2436	1,01E-11
EDN1	11,23605	4,14E-12
DLX3	11,23105	1,60E-13
PLEKHA1	11,22036	1,36E-12
HOXC13	11,19725	2,18E-12
FAM46B	11,19593	2,43E-12
PTGES	11,19333	9,05E-12
TMC4	11,19264	1,42E-13
MAFIP	11,19047	1,94E-12
NUAK1	11,18851	1,49E-12
RANBP17	11,18031	2,47E-12
LAMB1	11,16855	3,25E-46
TEAD2	11,16429	1,11E-43
PRSS16	11,15806	2,89E-12
DPYSL5	11,13234	2,91E-12

KCNK15	11,11781	2,59E-12
B3GNT3	11,11376	2,44E-11
THNSL2	11,11118	4,17E-12
WDR35	11,10741	3,21E-12
FIBCD1	11,10458	3,77E-11
LDLRAD3	11,10191	3,39E-12
EFHD1	11,09877	4,05E-12
TMEM132/	11,09169	2,60E-10
KLF4	11,09139	3,13E-12
PGR	11,07427	1,14E-10
WWC2	11,06974	3,02E-12
MIPOL1	11,06711	2,96E-12
TMEM144	11,0554	6,43E-12
NXPH4	11,05475	1,99E-13
TUSC1	11,05437	3,16E-12
CST1	11,0504	3,79E-12
MID2	11,04632	4,42E-12
MFAP2	11,04524	3,57E-12
MB	11,04265	3,38E-12
MBNL2	11,02726	3,71E-12
NETO2	11,02237	7,03E-24
TMEM51	11,01316	4,39E-12
FAM3B	11,01229	4,20E-12
SH2D4A	11,00398	4,30E-12
GPRC5A	10,99	2,08E-10
RNF182	10,98612	1,59E-11
PANX2	10,97237	1,41E-10
MPDZ	10,97093	7,95E-12
KNDC1	10,96897	6,24E-11
ARHGEF10I	10,96271	5,56E-12
DCDC2	10,96037	6,28E-12
NES	10,9596	6,24E-12
WNK2	10,9436	4,13E-13
SDC4	10,93372	5,58E-13
ITGB5	10,93115	8,47E-16
C1orf115	10,91671	7,76E-12
HPN	10,90638	1,15E-11
MET	10,89082	1,88E-23
RET	10,87253	7,15E-11
EPPK1	10,86809	3,24E-20
PPFIBP2	10,86724	1,06E-11
FOXA1	10,85615	1,15E-53
GLTSCR1	10,84876	5,99E-13
C7orf13	10,82602	1,30E-11
KIF26A	10,82483	2,05E-12
SPESP1	10,81783	2,10E-11
LINC01296	10,80333	1,98E-11
MBOAT2	10,80284	1,47E-11
CCDC64B	10,79587	2,41E-11
AKR1C2	10,78875	2,55E-10

OSMR	10,78361	4,12E-11
GPX3	10,77591	0,000145
MLF1	10,76834	2,15E-11
TMEM125	10,75566	1,95E-11
GADD45G	10,74748	1,36E-10
FAM198B	10,74134	2,17E-11
KALRN	10,73844	8,47E-11
HES2	10,73431	7,13E-10
FAM114A1	10,72302	1,09E-10
WNT11	10,71032	3,06E-10
ATOH8	10,69233	7,73E-11
DGAT2	10,68214	3,74E-11
ODAM	10,67355	3,20E-11
CMTM4	10,67284	3,76E-32
PTPRG-AS1	10,67171	3,64E-11
MYO1E	10,65802	1,02E-10
ARHGEF35	10,65744	4,97E-11
DMKN	10,6544	3,61E-11
LRRC75B	10,64987	4,09E-11
CNKSR3	10,64446	4,36E-11
LHX4	10,62997	1,94E-12
TSPEAR	10,62884	9,10E-11
SPECC1	10,62456	1,60E-31
TTC12	10,62058	4,40E-11
RAB36	10,6185	5,60E-11
TMC5	10,60644	6,01E-11
LINC00886	10,60089	2,36E-10
SLC9A2	10,59679	6,12E-40
EFNB3	10,59628	5,33E-11
TBC1D12	10,56878	6,15E-11
DUXAP8	10,56066	6,31E-11
FERMT1	10,55087	6,79E-11
CPE	10,54833	7,72E-11
EHF	10,54811	1,27E-10
BAMBI	10,54602	4,65E-76
PCDHA10	10,54429	8,67E-11
SULT2B1	10,50616	5,98E-12
CELSR1	10,50233	1,57E-21
FBN2	10,48959	2,96E-10
P3H2	10,48735	1,08E-10
LRRC49	10,48145	1,04E-10
CASC9	10,47211	1,08E-10
LRIG3	10,46339	1,42E-10
SHISA9	10,45879	1,74E-10
WSCD1	10,44024	1,62E-10
HSPG2	10,43908	7,64E-12
PAQR5	10,43893	1,49E-10
CDS1	10,43867	4,28E-21
MEIS3P1	10,43545	1,80E-10
SNTB1	10,43338	3,54E-21

SLC12A8	10,4234	1,91E-10
CACNA2D1	10,42211	1,48E-10
RBPMS	10,41511	6,33E-12
CACHD1	10,40505	8,32E-10
CHRM1	10,39072	6,14E-10
MORN4	10,3897	1,98E-10
HIST3H2A	10,38683	1,82E-10
LMCD1	10,38578	9,96E-09
HCN4	10,36633	2,10E-10
HLA-DQB1	10,36614	7,28E-09
RNF223	10,36555	2,62E-10
CNTNAP2	10,36447	2,90E-10
IGFBP3	10,35669	3,42E-09
ERBB3	10,35425	1,01E-07
ELOVL2	10,35329	2,38E-10
HOXC11	10,34814	3,69E-10
ZNF467	10,33957	5,96E-30
SPIN3	10,33575	2,46E-10
NSUN7	10,3232	2,81E-10
PLD1	10,31485	3,20E-10
ARHGEF37	10,31312	3,58E-10
TNS1	10,31094	5,53E-10
KRTAP3-1	10,29714	1,25E-09
BCAS1	10,29517	2,38E-09
EXD3	10,28631	5,65E-10
ZNF532	10,28442	3,37E-10
TFF3	10,28278	4,37E-10
ZYG11A	10,28203	1,04E-09
LINC00052	10,27757	4,27E-09
C18orf63	10,27563	4,93E-10
RERG	10,26471	3,85E-10
OTUB2	10,26299	3,28E-09
SALL4	10,25412	7,25E-10
ERVMER34	10,24829	1,07E-09
NMNAT2	10,23804	5,72E-10
THAP10	10,23432	8,67E-10
PXDC1	10,2295	5,48E-10
FKBP10	10,22614	2,23E-06
ALDH1A3	10,22389	1,97E-08
EN2	10,21414	5,10E-10
UGGT2	10,20854	5,90E-10
GOLT1A	10,20203	5,73E-10
DUSP9	10,1981	6,13E-10
LINC00925	10,19722	6,33E-10
OVOL2	10,19675	7,71E-09
ERBB4	10,18995	9,17E-10
MLXIPL	10,18632	9,65E-10
LPIN3	10,17608	1,46E-19
ZBED9	10,17489	6,44E-10
NPAS2	10,16143	1,05E-09

SLC24A3	10,1313	8,52E-10
ZNF732	10,12637	1,68E-09
GREB1L	10,10877	1,45E-08
GALNT18	10,09061	1,15E-09
ASCL1	10,09036	2,24E-09
FAM110B	10,08991	1,49E-09
PLEKHN1	10,08655	1,20E-09
BLVRB	10,084	2,38E-08
PCDH7	10,08396	1,29E-09
CAMK2B	10,06505	1,24E-09
ADGRL2	10,06371	1,46E-09
LYPD1	10,05952	2,17E-09
SDK1	10,05082	1,82E-09
CDKN2B	10,04813	2,42E-09
C12orf56	10,03844	1,58E-09
EPHA6	10,02591	1,62E-09
FAR2P1	10,02434	1,08E-08
EPHA2	10,02037	3,87E-09
TEAD4	10,01548	5,51E-26
ADGRV1	10,01533	4,29E-09
LGR4	10,01466	2,15E-09
KCNC3	10,00401	6,14E-19
ANKRD18B	10,00212	2,15E-09
SLC9A4	9,999145	1,06E-08
SLC26A4-A	9,998752	1,92E-09
ARC	9,990127	2,96E-08
ELF5	9,988684	1,40E-08
CSTA	9,985364	4,57E-09
MEIS3	9,98523	7,95E-09
UPK2	9,98102	3,32E-08
TMEM136	9,978068	3,02E-09
PRIMA1	9,973736	9,19E-09
WWTR1	9,964371	4,82E-19
SAMD4A	9,960849	2,01E-09
NMU	9,960411	3,02E-09
TBX2-AS1	9,942289	3,96E-09
NCKAP5	9,934449	3,08E-09
PCSK6	9,934409	1,08E-37
C19orf33	9,931567	1,47E-10
NAV2	9,913448	3,56E-09
SMAD9	9,913277	8,03E-08
NRP1	9,912312	1,68E-77
PNPLA4	9,912202	3,18E-09
CHST8	9,906542	3,24E-09
CRIP2	9,894457	4,26E-64
ZNF311	9,893708	1,55E-08
CCL2	9,889241	1,69E-08
HLA-DRB1	9,882708	1,20E-08
RNF157	9,874439	4,06E-09
CYP4F11	9,873256	1,09E-08

ARHGDIG	9,869457	1,42E-10
GATA6	9,863909	6,30E-09
ULK2	9,862734	7,66E-09
BOK	9,856444	1,54E-10
PARD6G	9,851041	4,50E-09
P4HA2	9,848623	1,13E-17
LYPD6	9,847104	4,60E-09
PTGR1	9,840801	9,52E-09
PPIC	9,83634	4,29E-40
WLS	9,834077	8,10E-09
GAD1	9,817353	2,82E-10
APP	9,807836	2,03E-111
MPP3	9,806672	5,97E-09
SOWAHC	9,798947	1,04E-08
CARD10	9,796063	7,52E-09
SLC2A10	9,782149	7,90E-09
PEX11A	9,781951	1,12E-08
VIPR1	9,777978	4,33E-08
TUBA3E	9,776343	1,25E-08
ARHGEF28	9,768911	2,31E-10
MAPK4	9,768153	9,29E-08
PTGFRN	9,760393	1,11E-62
EPHA10	9,754373	8,04E-09
TRPV4	9,753706	1,19E-08
GALNT11	9,751194	7,37E-18
NFATC4	9,74593	9,43E-09
LINC00960	9,745275	1,35E-08
PCDHB6	9,742903	1,11E-08
SPATA18	9,741312	1,28E-07
ENPP5	9,732536	1,63E-08
C10orf82	9,731967	1,47E-08
EDA2R	9,725915	2,34E-08
SPATA17	9,721145	2,96E-10
MUC5B	9,720102	1,02E-08
SEPT10	9,712052	3,54E-08
FAXC	9,711466	1,09E-08
SPINK5	9,710087	4,86E-08
CBLC	9,704723	1,31E-08
ICOSLG	9,702527	1,10E-08
TYRP1	9,688178	1,28E-08
MRAS	9,682314	1,66E-08
ERC2	9,675398	1,28E-08
LINC00963	9,670125	6,69E-10
SLITRK4	9,662981	1,90E-08
SLC47A1	9,661977	2,09E-08
REPS2	9,656906	5,20E-08
ALDH1L2	9,655257	9,17E-07
PLEK2	9,654519	1,47E-08
LIFR	9,645031	9,04E-10
LSR	9,641215	1,35E-90

HMOX1	9,637589	1,40E-08
EML1	9,637485	1,58E-25
IRX4	9,635053	3,18E-08
LURAP1L	9,63055	1,87E-08
KRT15	9,612484	4,66E-07
PRRG1	9,609441	1,00E-07
PRSS22	9,602329	3,95E-08
SYTL4	9,597391	4,13E-08
OPHN1	9,596807	2,06E-08
LDHD	9,595454	1,36E-07
LINC01116	9,587397	2,20E-08
TMPRSS2	9,585074	3,57E-08
ACOT4	9,584223	2,86E-08
HSBP1L1	9,581887	6,64E-10
C1orf106	9,581141	4,13E-08
KCNS3	9,575132	7,28E-08
PLBD1	9,569223	3,79E-08
SOX2	9,557764	7,09E-10
FUT9	9,555039	2,62E-08
RBPMS2	9,547515	2,90E-08
TENM4	9,544322	5,00E-08
DDX43	9,540774	3,72E-08
GNG7	9,538906	5,10E-08
GPR27	9,534525	3,62E-08
TEAD3	9,534384	1,64E-30
NAALADL2	9,523916	1,26E-09
LPCAT2	9,515369	4,80E-08
CYP24A1	9,514791	3,43E-08
RBM24	9,512804	5,94E-08
ARNTL	9,506426	6,86E-08
NOV	9,505339	2,36E-06
FAM43A	9,492867	1,23E-09
TJP3	9,487876	7,65E-06
CACNA1H	9,480785	1,94E-31
KLF9	9,474374	1,56E-07
C2orf54	9,473916	3,66E-07
PLA2G4F	9,469557	1,55E-06
ALDH3A1	9,468089	1,11E-06
LOC100506	9,463473	8,74E-08
MMP16	9,460583	6,72E-08
NDST1	9,45496	4,94E-109
PCDHA7	9,447899	4,99E-08
PRPH	9,43801	7,49E-08
ARHGEF34I	9,43365	6,95E-08
GPX2	9,428422	7,55E-06
B3GNT4	9,424589	6,38E-08
SYNPO	9,422672	1,99E-07
HSPA12A	9,417816	7,01E-08
ANKRD18A	9,403299	6,73E-08
FGFR2	9,399125	6,63E-08

TC2N	9,397026	3,62E-33
WISP2	9,39572	3,25E-06
IL1R1	9,394927	7,53E-07
LYPD6B	9,386527	7,52E-08
TMEM117	9,376235	7,51E-08
FCGR1A	9,372466	3,93E-07
FAM83H	9,371955	7,22E-128
OGFRL1	9,362567	2,34E-09
LINC01123	9,355445	8,68E-08
KCNMA1	9,351246	2,83E-07
ID4	9,346262	5,24E-06
CPT1C	9,34379	2,48E-07
EYA2	9,342619	3,24E-07
PRAME	9,337414	1,08E-08
HOXC-AS3	9,334355	1,10E-07
SYDE1	9,332786	1,04E-07
ZNF860	9,331874	1,44E-07
NUAK2	9,32493	1,10E-07
LGR6	9,31986	1,57E-07
FGB	9,314201	1,95E-05
B3GALNT1	9,312075	5,38E-16
RASD1	9,306441	3,81E-07
COL4A5	9,297688	2,35E-07
CDKN1A	9,288598	1,27E-12
CNGB3	9,286705	1,66E-07
INHA	9,286589	1,02E-05
C1orf116	9,286441	2,91E-07
SIM2	9,281174	1,44E-07
GMPR	9,278086	5,30E-07
FAM201A	9,277934	1,34E-07
RIC3	9,26876	2,36E-07
TGIF1	9,265305	9,22E-40
TMC7	9,259301	1,72E-07
TMEM45B	9,254503	5,63E-16
SHH	9,250075	2,35E-07
OSBPL10	9,245801	8,74E-30
CLDN7	9,244643	1,56E-14
PCDHB4	9,239217	6,59E-07
RASSF10	9,237769	2,67E-07
EFEMP1	9,227512	2,19E-58
FSTL4	9,225357	3,06E-07
ZNF703	9,214068	1,90E-155
DNAH3	9,209748	2,75E-07
NIPAL1	9,209748	2,75E-07
PROM2	9,204316	3,63E-51
MYRIP	9,203601	1,00E-08
SLC25A43	9,199208	6,42E-29
PPP2R3A	9,19829	9,63E-22
SHROOM2	9,198085	2,22E-07
NRG3	9,197784	3,31E-07

EFNA5	9,192112	5,32E-09
UNC5B	9,191521	9,54E-08
PCAT7	9,187055	2,26E-07
TUB	9,180463	2,35E-07
L1CAM	9,179532	5,32E-21
THBD	9,174525	2,94E-07
CDC42BPB	9,173263	1,65E-110
CHGA	9,162987	6,29E-09
TNFRSF11A	9,162612	2,89E-07
C1orf210	9,150645	4,61E-07
MMP17	9,145194	1,63E-15
FZD9	9,14488	4,06E-07
COL6A1	9,140321	1,15E-15
AIM1L	9,13782	5,51E-07
GSG1L	9,135606	3,28E-07
TRPV3	9,13382	3,07E-07
PGM5	9,120861	4,74E-07
ADAMTS9	9,119237	2,07E-06
IGSF5	9,117577	3,91E-07
PRR34-AS1	9,116898	6,38E-07
ABCA12	9,111465	8,58E-26
RAB31	9,110813	2,35E-08
FAM110C	9,109723	1,56E-06
MARC2	9,107719	3,81E-07
CLDN23	9,096712	3,79E-07
COL6A2	9,093521	9,60E-09
SOWAHB	9,083742	4,89E-07
GRIK3	9,078372	1,76E-06
TEKT4P2	9,075179	4,27E-07
FEZ1	9,07365	6,25E-07
MAOB	9,063665	1,49E-06
SP6	9,062704	2,06E-08
HOXC13-AS	9,060495	6,07E-07
RPRM	9,054175	1,23E-06
HTR2C	9,043409	5,29E-07
LHX1	9,037292	6,16E-07
S100A9	9,036367	6,64E-05
AGR2	9,035703	7,51E-06
INTU	9,023778	5,71E-07
HOXA4	9,016694	1,40E-06
NHLRC1	9,015111	1,69E-06
RADIL	9,013523	2,07E-06
KIAA1522	9,010672	4,59E-108
CNNM1	9,008189	6,24E-07
PCDHA5	9,00544	6,61E-07
PCDHB3	9,001015	6,24E-21
DLC1	8,997454	6,94E-15
CALCR	8,994436	9,35E-07
FBXO16	8,991252	7,89E-07
LOC10028	8,991252	7,89E-07

SNCAIP	8,986079	7,09E-07
DFNB31	8,985552	1,41E-14
KCNV1	8,978609	2,07E-06
PLA2G3	8,975903	4,09E-06
FOS	8,965947	1,96E-05
CYR61	8,96308	0,000387
LCN2	8,957774	3,62E-06
JAM2	8,950815	3,96E-06
CAV2	8,942861	9,46E-07
LINC01270	8,941994	1,35E-05
TMPRSS4	8,940438	1,38E-06
CDC42EP5	8,940405	9,35E-07
PPARG	8,939611	1,31E-06
PRODH	8,931906	6,48E-05
SLC30A3	8,93012	1,25E-06
VEPH1	8,927401	5,08E-06
TPTE	8,924286	1,15E-06
FZD4	8,917265	1,72E-06
XK	8,917212	1,15E-06
ZCCHC12	8,91304	1,07E-06
MYO6	8,912772	4,10E-90
LOC644919	8,912169	1,58E-06
FTCDNL1	8,908853	1,14E-06
CD24	8,905695	1,21E-138
DSCAM	8,89586	2,63E-06
ZNF112	8,886438	1,39E-06
HSD11B2	8,88442	3,54E-08
A4GALT	8,878141	4,96E-06
STMND1	8,876595	1,39E-06
PPAP2C	8,876382	1,69E-20
SLC25A21-A	8,871538	1,39E-05
PRICKLE2-A	8,867539	1,52E-06
FAM86B1	8,856665	5,71E-08
TACC2	8,856308	1,01E-06
SEMA3D	8,855277	4,29E-06
CLIC3	8,853699	2,08E-06
ADCY6	8,852147	7,62E-26
KRT23	8,847978	1,54E-06
FRMPD1	8,840943	1,86E-06
PCDHA13	8,83346	1,74E-06
CCDC83	8,83172	2,47E-06
KCNN2	8,828581	1,72E-06
PCOLCE2	8,825478	1,85E-06
ARPIN	8,823681	2,05E-06
LHX2	8,820543	2,49E-06
MUC3A	8,812944	2,42E-06
C4orf19	8,810284	2,19E-06
TRIP6	8,808076	8,46E-113
AK4	8,805051	6,64E-38
MTMR7	8,796727	2,06E-06

TRNP1	8,791723	2,18E-06
PARD3B	8,79041	5,34E-08
PDGFB	8,766109	6,36E-14
LOC100134	8,764622	2,69E-06
EPN2	8,762468	4,28E-26
ZNF503-AS	8,757654	3,08E-06
EPS8	8,75075	3,70E-42
KIF5C	8,750186	3,53E-06
LOC728739	8,744518	3,03E-06
CARD14	8,743156	4,25E-06
WDFY3-AS2	8,740783	3,67E-06
KRT17	8,736977	3,07E-07
HOXB9	8,729938	4,67E-05
GFPT2	8,716988	3,31E-06
FAM81B	8,712684	4,23E-06
SERPINA1	8,708816	3,60E-06
MEST	8,707189	8,11E-104
LOC90768	8,706867	3,81E-06
FCHO2	8,706084	7,48E-08
MBP	8,704926	4,24E-06
TFAP2A-AS	8,69864	3,80E-06
EFNA2	8,693295	3,59E-06
SCIN	8,689954	7,42E-06
ZIC4	8,686948	3,76E-06
HOXC5	8,684344	1,25E-05
IGF1	8,680069	4,14E-06
PDZRN3	8,673666	4,00E-06
PTPRO	8,673197	2,41E-13
VAX2	8,672196	4,67E-06
PCDH19	8,669773	7,63E-06
KRT80	8,666322	6,28E-74
HOTAIR	8,662255	4,39E-06
CERS1	8,656126	1,38E-05
SGPP2	8,655797	6,00E-06
LINC01006	8,651254	4,82E-06
FANK1	8,64895	2,15E-05
ANK1	8,646109	6,46E-05
SNAP25	8,644236	5,79E-06
PDGFRL	8,644236	5,79E-06
EPHA4	8,641264	9,62E-06
FAM20C	8,632669	3,01E-13
C5AR2	8,632282	3,69E-12
SYBU	8,623389	2,01E-07
C16orf45	8,621353	5,76E-06
EGR2	8,616319	7,94E-05
PPP1R26-A	8,610457	1,23E-05
RAI2	8,598675	9,91E-06
SLC41A2	8,593204	1,39E-07
GRB14	8,589022	3,26E-19
CYB561	8,584943	3,66E-78

LAMB2	8,583275	2,03E-38
CEBPA-AS1	8,582337	1,18E-05
SHC2	8,579669	1,51E-05
TTC29	8,579243	6,90E-06
TOM1L1	8,565347	1,34E-05
PDGFC	8,565209	7,38E-07
FAM131C	8,563859	7,25E-06
LNX1	8,560405	5,36E-05
GSTO2	8,558007	3,92E-24
HLA-DRB5	8,555878	1,12E-05
SPATA6	8,552025	1,31E-05
FBXL2	8,549399	8,39E-06
PAX2	8,544433	0,000147
SLC22A11	8,544158	2,81E-05
ASCL5	8,543456	8,03E-06
IFNLR1	8,542915	8,08E-06
BMP8B	8,541831	8,24E-06
PVALB	8,540857	2,02E-05
XKRX	8,537012	1,47E-05
NMUR2	8,536412	8,49E-06
RMDN2	8,531509	8,67E-06
MPP2	8,5301	4,01E-07
FAM95C	8,527147	9,17E-06
ESRP1	8,525894	2,00E-14
TCHHL1	8,525487	0,000308
GUCY1B2	8,525365	2,54E-05
KIAA1324	8,516142	2,18E-26
BMP2	8,507046	1,19E-32
CRIM1	8,502482	2,53E-07
MATK	8,502236	2,43E-65
SOX18	8,500091	1,02E-05
KCNQ1OT1	8,487938	6,01E-07
SNAR-E	8,485441	2,33E-05
GATA5	8,484959	1,21E-05
ARHGEF16	8,481703	6,57E-28
STOX2	8,47876	1,13E-05
CREB5	8,471553	0,00015
IGFL1	8,469042	0,000304
LRRK2	8,468004	1,64E-05
GJA1	8,467182	5,58E-05
WIF1	8,466014	6,50E-05
NECAB2	8,462737	2,81E-05
SERPINA3	8,462346	2,52E-05
NODAL	8,459452	2,04E-05
RIPPLY3	8,453078	1,37E-05
LOC102723	8,451924	1,43E-05
PLEKHG6	8,45025	9,55E-12
CD101	8,443863	1,70E-05
CRABP2	8,439823	5,32E-64
SLC6A14	8,439788	1,48E-05

RIN2	8,43575	2,14E-05
MAP2	8,433039	3,76E-07
CLCA2	8,430824	0,000162
SHISA4	8,428787	3,12E-05
LOC10023:	8,420033	3,00E-07
MUCL1	8,411241	1,63E-05
FAM196A	8,408787	4,61E-05
NOL3	8,408406	2,24E-37
UPK3B	8,401734	7,99E-05
HOXD12	8,38729	6,33E-05
GPR37L1	8,384722	2,17E-05
LOX	8,383492	2,02E-05
JPH3	8,379678	3,47E-07
SMPDL3A	8,378098	2,98E-05
HOXA5	8,376797	2,87E-05
FOXA2	8,376231	1,94E-05
HS6ST3	8,370135	2,28E-05
MAPK15	8,360264	4,41E-05
DNAH5	8,35912	2,34E-05
F7	8,355261	7,33E-05
NELL2	8,352928	3,26E-05
GRB7	8,352306	3,40E-05
RHPN2	8,352034	4,91E-22
LINC01124	8,341158	3,40E-05
PAH	8,33748	2,86E-05
HLA-F	8,333804	5,01E-05
SLC52A3	8,329374	3,69E-05
DNAJC22	8,329009	4,44E-07
LRRK1	8,326845	3,24E-05
HOXC6	8,32495	3,00E-05
ALDH3B2	8,311479	0,000107
STOM	8,308738	3,78E-26
TBC1D8B	8,307921	5,20E-05
AMBP	8,304242	0,000126
MAMLD1	8,303715	1,12E-06
LOC38983-	8,302399	5,05E-07
SERPINH1	8,297632	2,42E-173
EPHB4	8,294463	1,08E-186
ESRP2	8,289146	3,74E-60
PART1	8,287342	3,58E-05
SHTN1	8,286086	4,16E-73
NKX6-1	8,284728	3,50E-05
ADAP2	8,284099	3,27E-05
NCAM2	8,282747	1,93E-20
DNM1P35	8,277144	0,000118
DGCR5	8,272384	3,68E-05
NACC2	8,258752	8,57E-05
SLC1A3	8,256653	3,61E-05
TRPM4	8,256062	1,98E-36
NPAS3	8,253297	5,55E-05

CCDC110	8,250622	6,76E-05
ACTG2	8,247106	5,17E-06
WFDC2	8,242793	6,52E-05
GPR39	8,23943	5,37E-05
MIR4458H(	8,233382	4,30E-05
SEMA4C	8,226937	3,78E-66
FOXI3	8,220589	4,63E-05
RHOV	8,216209	2,76E-21
MANSC4	8,215138	2,36E-05
POU6F2	8,20766	5,07E-05
SHANK2	8,20514	4,93E-06
GRID1	8,204916	5,72E-05
KCNN1	8,202949	7,86E-05
CPLX2	8,199681	0,0002
ANKS1B	8,199391	8,09E-05
AMOTL2	8,198659	1,25E-39
COLCA1	8,195348	5,49E-05
HOXA6	8,183055	0,000157
CDH18	8,179399	6,15E-05
GHR	8,17733	5,36E-05
MARVELD2	8,171575	3,24E-35
PRDM6	8,156443	1,20E-06
NOXA1	8,154725	0,000139
KCNJ3	8,145379	1,68E-06
ZP3	8,144747	0,000118
ADAM32	8,141215	0,000146
RNF180	8,134854	8,93E-05
SLIT2	8,133456	6,50E-05
ZFHX3	8,127191	1,83E-84
TGFBI	8,125667	0,000176
CLDN9	8,118336	0,000106
NFIA	8,117538	6,17E-20
SEMA6A	8,114238	2,45E-11
SLC52A1	8,111883	0,000195
MGC32805	8,111142	0,000184
TUBA3D	8,10476	2,35E-06
KRT16	8,098716	0,000233
PDE2A	8,086626	0,000164
RAB6B	8,081451	2,03E-06
TBC1D30	8,080921	3,20E-69
GULP1	8,080781	8,87E-05
KIF26B	8,080034	8,77E-05
RIBC2	8,07981	0,00031
LPPR3	8,079047	0,000339
RIMS4	8,076834	3,37E-14
TH	8,074848	0,000129
NYNRIN	8,068065	2,62E-08
ADAMTS7	8,067639	5,65E-10
LIMCH1	8,064377	0,000179
ADAMTSL3	8,064253	0,000121

SNCB	8,059268	0,000455
CCDC40	8,055846	0,000216
CCDC3	8,051422	0,000103
TMEM105	8,045367	0,00015
LOC10027(	8,044597	0,000144
GFRA1	8,032619	1,02E-46
SAMHD1	8,030861	7,21E-10
SASH1	8,025341	0,00013
EMCN	8,023008	0,000122
PDZD4	8,021273	9,63E-11
PCDHAC1	8,017631	0,000198
EWSAT1	8,015935	0,000186
CKMT1B	8,015226	9,29E-06
HOPX	8,009938	0,000376
GSC	8,0088	0,000328
KCNF1	8,007382	0,00014
BOC	8,004265	0,000124
PKD1L2	8,000326	0,000123
SFN	7,995855	3,45E-60
GOLGA7B	7,994768	2,19E-12
PRKAG2	7,988609	1,20E-26
NXPH3	7,98373	0,000194
ZDHHC1	7,979715	0,000164
GNAS-AS1	7,973289	0,000143
ACCS	7,970005	0,000228
KRTAP4-1	7,96851	0,000213
KCNU1	7,963609	0,000159
MARCH1	7,959274	4,33E-06
CALB2	7,957237	0,000298
LOC730102	7,945065	0,000423
BCAM	7,938679	1,82E-59
EGR1	7,936272	2,11E-07
CYP4F22	7,932598	0,000208
BACE2	7,930568	1,30E-47
CCSER1	7,928417	0,000207
CALML5	7,921022	0,000273
SGCG	7,918348	0,000376
GJB5	7,916192	0,000678
ADORA1	7,913467	0,000206
NRK	7,91278	0,000498
PRKD1	7,910912	0,000236
TLX1NB	7,904218	0,000193
HOXC9	7,895782	0,000216
NRCAM	7,885644	8,37E-26
OLFML3	7,882212	4,66E-05
CD36	7,873658	0,000388
HOXA1	7,871919	0,000353
NDNF	7,870992	0,000225
CLDN1	7,867622	5,96E-05
TTC6	7,865802	0,000249

KLB	7,865802	0,000249
IER5L	7,865423	8,43E-08
SSPN	7,864097	0,000261
C8orf48	7,863228	0,000256
LOC101929	7,862325	0,000281
ARHGEF38	7,861453	0,000292
TNS3	7,853323	6,67E-14
KIF12	7,851028	0,000255
SCNN1A	7,842193	5,68E-08
NR2F1-AS1	7,841392	0,000259
TGFA	7,836938	1,86E-09
DEFB126	7,836099	0,000265
LOC441666	7,830813	0,000312
DHX32	7,829272	7,50E-43
RHBDF2	7,819571	3,12E-40
SNAR-G1	7,814779	0,000351
OXGR1	7,809334	0,000377
SPINT1	7,807703	6,95E-101
CDC42EP4	7,805983	6,39E-05
THRB	7,803961	0,000301
HOXA11	7,797447	0,000824
KDR	7,794008	0,000349
TM4SF1	7,793096	0,000342
NPTX1	7,789564	0,000673
SEL1L3	7,787998	0,000459
LINC00992	7,787687	7,25E-06
CELF3	7,782172	0,000454
FRK	7,772142	0,000758
MAP3K13	7,766907	3,85E-20
FZD3	7,753876	2,70E-28
NKILA	7,746868	0,000393
WNT9A	7,745976	0,000534
IBSP	7,745927	0,000399
TNFRSF12A	7,737806	8,97E-46
ZNF204P	7,735734	8,95E-06
COL26A1	7,735693	0,000899
SLC5A5	7,73281	0,000754
GRID2IP	7,732714	0,00041
SLCO5A1	7,723217	0,000501
FOXP2	7,716344	1,30E-05
CEACAM5	7,714683	0,000842
DAPK2	7,702009	0,000568
SETBP1	7,696138	0,000756
NAP1L2	7,693368	0,000802
C19orf45	7,693289	0,000486
CNTNAP3	7,690358	0,000503
ZNF516	7,689317	7,96E-23
PLIN5	7,684507	0,000575
CA4	7,683483	0,000606
GSN	7,681015	0,000312

SYCP2	7,675543	0,000898
CACNA1I	7,675543	0,000898
LFNG	7,672021	3,33E-16
SAMD12-A	7,665753	0,000633
CACNA1D	7,663389	3,69E-17
TDRD5	7,655623	7,79E-13
TRIO	7,649953	3,13E-63
TUFT1	7,649351	2,38E-45
LMNA	7,644569	1,27E-90
RASGEF1A	7,641693	4,94E-22
HAP1	7,633651	0,000966
HOXC4	7,629503	0,00068
SEMA3F	7,626931	1,42E-50
COL3A1	7,611104	0,000945
ARHGEF17	7,597573	3,48E-22
SIX1	7,593567	9,37E-20
C8orf46	7,591308	0,000772
SYNE4	7,581167	9,93E-44
MIR4664	7,576593	0,000794
RNF207	7,575535	0,000796
FGF12	7,56619	2,65E-15
NGFR	7,562799	0,000913
STBD1	7,561729	0,000898
CXCL12	7,560836	2,06E-09
ZNF793-AS	7,556371	0,000863
ANKDD1B	7,548839	0,000933
LHX6	7,548839	0,000933
CDC14B	7,544706	1,47E-12
TMEM150C	7,537981	0,000228
RHOBTB1	7,537732	5,00E-163
SERPINB5	7,521011	0,000168
CDK14	7,515214	3,28E-05
TLCD1	7,510149	2,30E-52
TNFAIP8L3	7,500546	1,82E-24
LOC283335	7,489581	6,93E-20
FLT4	7,488181	6,12E-05
ADGRB1	7,486807	0,000102
ELOVL7	7,47681	0,00054
MALRD1	7,47604	2,96E-08
RNF43	7,457199	2,16E-113
NTN4	7,456175	3,86E-05
GSTM3	7,455986	2,32E-31
TSPAN12	7,451261	5,83E-12
DENND5A	7,443239	0,000192
WWC1	7,437486	1,48E-68
CXCL16	7,435329	1,43E-20
RIPK4	7,42293	4,15E-12
MARK1	7,416309	0,000164
ATXN1	7,409406	2,81E-18
RND3	7,408705	2,54E-38

GUCY1A3	7,402359	1,63E-19
BCAS4	7,400253	5,32E-21
SELM	7,395917	3,89E-05
MYO10	7,395312	1,52E-59
PCSK9	7,392746	2,69E-07
LENG9	7,388493	3,38E-28
LMX1B	7,388031	3,86E-15
CABYR	7,37879	2,15E-13
PRTG	7,370904	4,39E-05
SLC6A8	7,352812	4,39E-27
PRRG4	7,319279	8,00E-05
ID1	7,30414	3,65E-53
C1QTNF9B·	7,303048	2,06E-16
NUP210L	7,302089	2,68E-19
TTC39A	7,280854	2,47E-20
DHRS2	7,277364	8,37E-29
GPAT3	7,271041	2,35E-07
SCN4A	7,258994	2,42E-13
FKBP9	7,245274	1,13E-96
STARD10	7,220011	5,20E-76
C16orf74	7,219817	2,55E-08
RAB20	7,212022	5,06E-07
RTN1	7,200173	0,000109
TLCD2	7,195037	7,75E-14
WNT6	7,190415	9,42E-05
LOXL3	7,189319	4,44E-15
ATP8B1	7,185617	1,52E-48
POU3F3	7,154774	2,91E-07
F2RL1	7,151771	6,60E-13
NRARP	7,144869	1,45E-24
CEBPA	7,141171	1,58E-24
SLC9A3R2	7,120234	1,11E-70
TPBG	7,118258	3,49E-82
COL16A1	7,100097	0,000184
CDYL2	7,095874	2,03E-13
CKMT1A	7,084098	7,43E-29
ZBTB7B	7,084083	1,23E-100
APBB2	7,0813	4,75E-31
SH3BGRL2	7,072847	5,60E-12
SEMA3B	7,059212	0,000184
CMBL	7,055353	5,33E-31
VPS37D	7,045693	5,28E-07
CRISPLD2	7,018965	8,13E-16
KLRG2	7,01024	1,67E-07
RAMP1	7,001266	0,000258
METRNL	6,999214	5,08E-33
CD9	6,999183	3,98E-103
C17orf104	6,988694	2,66E-06
LONRF2	6,982516	4,75E-21
TNNT1	6,979775	6,05E-101

EPB41L1	6,977228	6,54E-65
YAP1	6,960978	1,12E-38
ENTPD1	6,95014	9,10E-16
KSR2	6,946923	7,24E-10
KIAA1217	6,942007	1,83E-27
ASCL4	6,916132	2,54E-20
SIRPA	6,913195	6,04E-07
TBC1D9	6,910064	4,39E-87
PKIB	6,884629	1,12E-64
PHLDA3	6,884185	3,26E-28
PHLDA1	6,879018	3,83E-47
KDELR3	6,876037	1,40E-15
MACC1	6,865558	2,38E-08
TRIL	6,856845	0,000494
HBA1	6,849316	1,58E-06
RPP25	6,846583	1,12E-50
LGALS3	6,823482	2,73E-10
HMCN1	6,816422	0,000423
GAS2L3	6,811516	7,49E-43
FHDC1	6,797715	1,43E-18
SPATA20	6,782822	3,32E-54
HID1	6,782585	1,46E-53
JUP	6,77629	3,39E-65
PCDHB2	6,766082	3,51E-27
SCML1	6,762626	2,92E-13
CCDC122	6,761286	3,07E-17
LRRC73	6,760096	9,36E-09
LATS2	6,750773	5,03E-18
SEMA6B	6,745008	6,97E-19
MSRB3	6,743805	0,000585
EPCAM	6,73978	5,53E-161
ARVCF	6,733789	5,30E-37
CRABP1	6,730786	9,04E-06
ZFP2	6,724162	0,000909
LACC1	6,714883	2,06E-09
SLC8A1	6,712639	2,14E-12
IL17RB	6,707815	3,58E-10
PVRL2	6,706077	2,32E-58
B4GALNT4	6,695691	5,23E-29
SLC4A3	6,692303	3,03E-06
FBXO44	6,688162	8,39E-17
NAT8L	6,677343	2,17E-34
FBXO17	6,677318	5,97E-27
TNFRSF18	6,675123	3,44E-05
AMZ1	6,671113	8,02E-06
NIPAL2	6,663191	4,19E-17
RAI14	6,661359	6,31E-24
COBL	6,655265	7,16E-23
CAMSAP2	6,653902	2,45E-84
TMEM163	6,647421	5,98E-06

SYT7	6,644507	1,26E-91
MUC1	6,615409	2,77E-31
LAMA3	6,615188	7,87E-16
DRAM1	6,585507	5,64E-21
GPSM1	6,575564	1,97E-34
F12	6,565561	2,78E-87
PLOD1	6,555994	5,27E-65
SH3BP4	6,554959	2,42E-50
GLIS2	6,550655	1,05E-25
SH3PXD2B	6,550199	6,28E-37
FLVCR2	6,528785	5,65E-10
S100P	6,514162	9,83E-06
IL17RD	6,512224	2,88E-15
ACE	6,510111	1,88E-05
MAFB	6,508823	1,27E-23
CEBPD	6,49057	3,65E-15
CIART	6,473362	9,46E-28
MAP3K5	6,469615	8,05E-07
PC	6,462552	8,80E-05
MYO5C	6,460699	1,63E-85
RIPK2	6,455259	3,41E-72
ZFYVE28	6,450405	7,16E-15
SLC35D2	6,446034	1,72E-18
PCDHA12	6,445333	1,65E-11
NRXN3	6,436383	1,10E-09
GPNMB	6,434627	3,15E-22
AHNAK2	6,433655	1,34E-14
LGALSL	6,430943	6,82E-15
AHNAK	6,430734	1,11E-78
DUSP8	6,427596	2,52E-20
UST	6,425901	5,46E-11
CHST15	6,401798	5,98E-59
PREX1	6,390321	1,76E-70
ERBB2	6,359575	3,20E-40
FOXC1	6,357343	2,89E-19
CTSF	6,345889	2,15E-09
DEPTOR	6,342537	1,15E-06
RDH16	6,338374	2,35E-21
KIAA1324L	6,337934	9,28E-50
GALNT14	6,33013	7,07E-23
TMPRSS13	6,326284	1,32E-19
FAM171B	6,321828	5,21E-33
LARP6	6,310799	3,20E-10
TIMP3	6,292722	4,52E-09
ABLIM3	6,291073	4,67E-05
SORBS1	6,290603	3,91E-05
MPZL2	6,289825	3,55E-14
SESN3	6,282493	3,33E-16
SMAD7	6,280564	6,71E-27
LCA5L	6,270394	1,69E-06

KLC3	6,266232	8,62E-07
ZNRF3	6,261816	5,15E-120
CEP170B	6,259462	1,29E-90
P3H4	6,256097	7,57E-69
CCL5	6,254285	4,10E-06
MAPRE3	6,251359	1,38E-07
NGEF	6,25114	6,27E-05
KRT81	6,232982	1,91E-10
ECHDC3	6,223682	3,95E-08
ENAH	6,215435	5,09E-95
NKD1	6,199221	8,90E-60
RSPH1	6,196258	5,93E-06
WNT10B	6,182504	6,05E-09
TEX40	6,182214	7,38E-05
VDR	6,170114	6,32E-27
TRIM16L	6,158395	5,85E-29
MYO1D	6,145478	4,11E-53
HECW2	6,145254	5,83E-15
ZNF165	6,135403	1,10E-38
ARSD	6,132022	2,70E-31
ADCY1	6,131118	6,58E-34
SEPT11	6,129103	2,07E-73
PTK6	6,125417	1,01E-08
SLC27A3	6,118043	2,19E-24
GRIP1	6,113621	5,22E-15
CHDH	6,113203	1,07E-15
FAM81A	6,093641	4,90E-09
SDSL	6,078039	7,05E-24
PODXL2	6,064673	3,19E-75
TSPAN15	6,057417	4,95E-25
S100A13	6,056839	4,40E-43
MTERF2	6,045533	4,96E-11
NSMF	6,028024	1,86E-41
TRPM2-AS	6,021218	0,000182
DLX1	6,020962	3,51E-07
C15orf65	6,011457	3,77E-07
IFI27	6,007341	1,40E-06
NOTUM	5,973145	3,73E-16
RARG	5,97227	2,53E-49
GJA3	5,970164	1,82E-08
ALDH3B1	5,954325	7,64E-05
GREB1	5,945723	1,49E-74
PRRG2	5,945635	2,84E-09
ARRDC4	5,945164	5,90E-21
DBNDD1	5,933075	4,09E-55
CDR2L	5,926917	2,72E-31
ROR1	5,921756	0,000339
SUOX	5,919421	1,62E-15
RNF144B	5,918612	5,75E-07
FAM160A1	5,912449	2,80E-06

HPSE	5,909054	1,24E-06
MAPT	5,903376	1,29E-36
SIAE	5,90255	3,13E-38
PLXDC2	5,90081	7,26E-30
ABCB6	5,898977	4,55E-05
NTN1	5,896537	5,81E-12
RASGRF1	5,883801	8,66E-25
C2CD4D	5,882519	0,000171
HSD3B7	5,877595	1,47E-35
RAB17	5,868842	1,41E-16
EPB41L4B	5,865707	9,14E-37
PTPN21	5,864551	2,51E-20
HS6ST2	5,863165	4,92E-35
PRKCZ	5,862807	2,76E-38
FIRRE	5,858313	1,77E-06
RGS6	5,858275	4,13E-07
DPYSL4	5,852453	1,00E-12
COL1A1	5,849883	3,31E-14
SETD6	5,84628	1,17E-20
KLF5	5,845149	6,79E-68
SHOX2	5,828622	3,11E-06
ZNF433	5,824231	2,72E-10
N4BP3	5,814577	2,41E-29
C14orf37	5,813523	3,15E-05
SCARF2	5,813482	0,000524
NQO1	5,813267	8,54E-27
SDC3	5,812428	4,97E-30
C6orf132	5,81163	1,11E-40
LTBP4	5,792632	1,82E-36
LOC10192S	5,788182	5,53E-07
C10orf35	5,781497	8,23E-12
MYRFL	5,77994	0,000729
ITGA2B	5,776085	3,65E-07
FLRT3	5,770874	6,29E-44
FGD1	5,759209	8,05E-21
BEND7	5,757584	3,54E-05
VSTM2L	5,748985	0,000613
SUSD2	5,730426	3,96E-11
TCF7L1	5,729855	1,19E-09
SLC26A11	5,724111	2,76E-09
PLEKHH3	5,720794	1,04E-22
MOCOS	5,708215	1,04E-12
TNFRSF10E	5,701972	6,28E-19
IFIT3	5,690026	0,000177
GAS6	5,680645	3,71E-09
BLNK	5,660097	0,000344
BCAS3	5,643205	2,40E-84
LOC10050E	5,641182	0,000267
ANXA9	5,63834	1,28E-22
CTTN	5,63809	4,28E-67

RNF32	5,633785	1,35E-05
BAIAP2	5,632058	1,70E-39
ABCC3	5,629903	0,000257
GJB2	5,623454	4,44E-15
FN1	5,618679	1,08E-06
PSEN2	5,609161	9,23E-24
SMPDL3B	5,608236	1,25E-45
PARVB	5,607175	3,08E-18
ST5	5,591658	2,53E-12
LITAF	5,575742	1,47E-47
DDR1	5,566425	6,84E-42
ASCL2	5,554096	4,02E-13
INPP4B	5,553588	2,46E-38
LOC14870‹	5,541456	5,77E-09
CAMK1	5,537312	1,23E-06
MDK	5,531206	2,36E-111
CD55	5,522366	8,96E-31
BBC3	5,519997	4,26E-11
IFI30	5,513906	6,87E-48
PLXNA4	5,508627	1,77E-16
RAB30	5,505622	1,34E-32
SPATS2L	5,505389	2,87E-40
AK7	5,481894	2,88E-45
C1QTNF6	5,476163	1,26E-07
CEACAM1	5,472832	6,54E-07
EFNA1	5,472685	1,19E-18
TNFSF13	5,468394	2,19E-08
TSKU	5,459518	6,13E-41
HUNK	5,443629	1,53E-31
LARGE	5,441173	8,18E-24
TMEM52	5,435063	3,07E-10
NR4A1	5,427687	3,24E-21
PCDHGB5	5,42211	1,32E-06
SH3BP5	5,4217	2,10E-95
LRRC8E	5,410592	4,64E-15
PTP4A3	5,403133	1,35E-35
TNFRSF10A	5,389508	3,52E-17
MAPK8IP2	5,366468	3,69E-15
ID2	5,3569	1,66E-26
PHLDB1	5,351331	4,95E-27
CNKSR1	5,347675	1,95E-07
SYNPO2	5,338178	1,31E-15
TSHZ3	5,337906	4,92E-09
SMKR1	5,335765	1,20E-15
IFITM3	5,328272	1,76E-34
MPP7	5,323351	7,97E-36
ZG16B	5,322724	2,55E-07
KREMEN2	5,319225	8,13E-49
ZNF205	5,306733	6,67E-25
FZD7	5,293319	7,40E-19

PTGER2	5,28809	0,000889
FLJ23867	5,259281	7,25E-26
DENND2C	5,252752	6,24E-06
VGF	5,248111	1,31E-28
ZNF114	5,236011	7,17E-25
KLF3	5,22966	9,02E-18
PAX8-AS1	5,221436	3,58E-27
MAPK13	5,2144	2,12E-82
DEGS2	5,213832	1,41E-10
PITX1	5,205043	5,65E-39
PRELID2	5,198972	3,88E-10
CRLF1	5,18407	2,85E-05
TMEM178I	5,183876	4,25E-17
SPEF2	5,18314	1,04E-08
ITGB4	5,180959	1,74E-55
ALDOC	5,177996	5,49E-23
CST3	5,161439	1,68E-53
NFE2	5,156647	2,14E-07
NRGN	5,153607	1,17E-11
MAML2	5,152249	2,17E-10
INPP5J	5,134923	0,000375
CKAP4	5,133239	2,54E-83
STON2	5,128947	4,74E-47
MYO7A	5,127919	0,000386
OASL	5,115109	0,000182
GPRIN1	5,111623	1,63E-50
GUCY1B3	5,096941	0,000669
MYLIP	5,092302	2,09E-34
AK1	5,087606	8,19E-12
PKP1	5,080958	6,97E-13
TBC1D16	5,078211	2,29E-73
RTN4RL1	5,066145	5,72E-51
BCL3	5,063464	2,09E-22
IQSEC2	5,06293	4,30E-10
AKR1E2	5,062665	5,84E-08
BCAR3	5,061022	1,71E-06
TMEM62	5,056079	4,87E-12
TEX15	5,051669	5,40E-09
SYDE2	5,049097	9,19E-12
RASAL1	5,042599	9,69E-05
GRIN2D	5,04243	5,88E-10
MTUS1	5,039767	3,25E-39
SYT3	5,038663	6,94E-05
CTIF	5,038142	5,30E-15
PROSER2	5,018951	6,44E-13
PRR15	5,013636	1,74E-10
DLL1	5,00873	0,000209
TBC1D8	4,999679	1,83E-11
STEAP1	4,993984	0,000104
JDP2	4,987458	1,62E-19

TINCR	4,9858	3,53E-10
NDRG2	4,984782	5,73E-13
ZBTB46	4,97852	0,000261
HOMER2	4,974571	8,12E-65
ZNF630	4,973455	1,05E-08
KIF13A	4,963912	3,76E-45
IGSF3	4,962345	2,66E-30
OBSL1	4,955626	4,99E-26
C11orf63	4,947602	0,000452
PRKAA2	4,944833	2,37E-12
SGMS2	4,943694	5,19E-05
MNX1	4,932519	1,36E-20
ZBTB7C	4,931884	6,62E-06
PYGO1	4,930924	6,13E-06
LYPD3	4,92967	3,26E-20
SPR	4,929584	8,91E-59
SSH3	4,923911	1,46E-18
MFAP3L	4,920771	1,90E-15
LRRC26	4,917952	2,47E-09
PDLIM1	4,917127	3,33E-35
AIFM2	4,910547	2,47E-29
PACSIN3	4,906097	1,18E-45
EHHADH	4,900593	5,24E-05
CBS	4,894393	1,56E-36
AGAP2-AS1	4,89011	1,99E-33
HOMER3	4,885329	1,03E-39
NEIL2	4,881217	1,42E-07
ABTB2	4,878949	9,25E-19
OSBPL6	4,87513	3,16E-11
AMOTL1	4,869982	1,35E-38
TSPAN9	4,866068	9,02E-11
AP4B1-AS1	4,862704	3,72E-18
METTL7B	4,862653	0,000379
B4GALNT3	4,858171	0,000516
MTMR11	4,841643	4,30E-05
SLC22A5	4,826923	5,21E-12
FBLIM1	4,822133	2,88E-12
RGAG4	4,822063	5,83E-07
SCARB2	4,820448	3,09E-63
S100A10	4,811005	5,59E-86
CCM2L	4,809592	3,70E-07
KREMEN1	4,796814	4,59E-32
MB21D2	4,790576	1,46E-22
BFSP1	4,789136	1,72E-11
NFE2L3	4,788723	9,30E-36
AGRN	4,786577	5,19E-68
PLCB4	4,785415	1,67E-17
SRRM3	4,779945	3,92E-06
PVT1	4,778877	5,58E-25
TMEM121	4,776293	1,46E-08

DNAH14	4,773592	5,30E-09
SPIRE1	4,764763	5,17E-53
KIAA0319	4,764151	0,000393
MGAT5B	4,758775	0,000939
GPR161	4,758642	1,76E-11
B4GALNT1	4,754666	1,02E-19
ABCB4	4,750298	0,000181
ONECUT1	4,747813	0,000524
FJX1	4,740956	3,91E-13
NPR3	4,739177	2,02E-17
ADCY9	4,733116	5,15E-05
GAREM	4,716696	1,23E-08
DLGAP3	4,709187	1,63E-11
GCA	4,705885	9,25E-14
CD151	4,691338	9,62E-61
SHROOM1	4,689824	2,27E-11
C3orf67	4,687932	0,000866
GOLIM4	4,687695	8,17E-51
ADGRG6	4,680003	1,61E-17
LLGL2	4,675108	1,43E-52
NANOS1	4,670742	8,03E-27
GPC4	4,665419	9,24E-07
VASN	4,664318	0,000514
CBARP	4,658365	2,05E-06
GTF2IRD1	4,646021	4,03E-63
NR2F1	4,642615	3,76E-08
MAPK8IP1	4,638852	1,46E-39
STXBP6	4,62836	2,49E-10
DDAH1	4,628337	3,74E-15
NPTXR	4,619537	2,25E-20
SLC29A2	4,617781	3,36E-33
OLFM2	4,617192	1,51E-30
SMTN	4,606443	4,60E-67
SLC27A2	4,603584	3,21E-46
GAA	4,597868	1,45E-49
GNG13	4,580945	1,17E-05
PACSIN1	4,575172	2,52E-14
HSD17B14	4,573624	3,91E-05
ABHD4	4,564071	5,06E-09
GPR158	4,551005	7,19E-07
CNFN	4,549809	0,000373
PFN2	4,543671	2,77E-75
LRP1	4,537812	5,65E-09
PCBD1	4,533739	5,55E-74
TP53INP1	4,521414	3,82E-14
IGFBP4	4,520898	4,33E-09
ZNF618	4,52083	7,57E-69
NEURL1B	4,514261	3,30E-32
DUSP4	4,5018	0,000403
URB1-AS1	4,501664	1,24E-05

RUNX2	4,498905	2,08E-33
OCLN	4,49184	6,18E-17
FGD4	4,487073	1,61E-15
ZNF296	4,486692	6,92E-20
EFR3B	4,486474	2,49E-20
EGR3	4,480879	8,34E-05
FLT3	4,480371	0,000524
NINJ1	4,467231	1,25E-20
STX3	4,460803	2,80E-24
SAMD12	4,458494	6,59E-17
ENC1	4,456212	1,40E-24
BMF	4,452773	7,86E-06
FZD2	4,452044	2,07E-23
AVPI1	4,44704	1,98E-16
EPS8L2	4,425498	3,42E-27
COL5A1	4,422774	4,99E-05
HOOK2	4,421331	2,21E-32
IFI44L	4,413334	0,000623
S100A11	4,406186	4,06E-43
GSTA4	4,402766	2,15E-11
TNS2	4,397696	2,73E-23
TENM3	4,370937	3,37E-54
NHSL1	4,366433	9,00E-19
CSRP2	4,365945	3,75E-15
MRPL41	4,363447	9,75E-60
FAAH	4,354689	1,88E-15
FUT1	4,351911	6,68E-10
PLEKHG5	4,345658	9,13E-13
SLC46A1	4,342565	2,07E-12
ERMP1	4,336979	1,67E-20
ARHGEF4	4,331136	1,47E-05
EHD4	4,330381	4,31E-19
GNAZ	4,327037	4,50E-07
ATP1B1	4,321881	6,87E-53
KIAA1549	4,303915	4,94E-26
REEP6	4,30153	1,23E-27
CXADR	4,300735	9,09E-25
RAB13	4,298919	6,17E-47
BACE1	4,295777	2,39E-23
GOLM1	4,291854	1,03E-20
C6orf52	4,288467	2,79E-05
LTBP2	4,288274	7,55E-05
PTPRJ	4,286662	9,21E-16
MYO1C	4,285455	1,00E-37
CAMSAP3	4,284224	1,18E-29
SGSH	4,279255	7,14E-22
PRR36	4,276902	4,32E-70
GPR176	4,267803	0,000482
ACTA2	4,262606	1,17E-06
TMEM184/	4,259323	2,04E-22

ZNF185	4,258523	4,68E-23
TMEM191/	4,257557	6,67E-17
HEYL	4,25739	0,000129
UNC93B1	4,255295	1,43E-35
ASAH2	4,252661	2,20E-15
CHST3	4,252163	4,44E-05
ZNF793	4,248631	2,55E-09
ANKRD34A	4,243976	5,46E-11
TXNIP	4,241314	1,81E-12
MERTK	4,239828	2,02E-05
MAGI1	4,228252	4,50E-24
CABLES1	4,227358	3,90E-12
ZNF385A	4,224981	1,04E-49
HSPA1A	4,222813	1,54E-23
ABLIM2	4,217064	2,08E-05
AGMAT	4,215463	4,97E-13
PTPRU	4,211864	7,83E-28
TMEM150/	4,192146	4,19E-16
HIP1	4,189994	2,04E-46
TUBB2B	4,189271	5,63E-11
HFE	4,180716	0,000275
BOD1	4,173048	1,03E-32
RAB11FIP5	4,172796	8,81E-06
APOE	4,166859	4,71E-08
EPS8L1	4,147699	1,44E-08
ALG1L	4,141075	6,23E-11
TOB2P1	4,132592	0,000103
NAV1	4,127265	8,25E-05
NBPF15	4,108737	4,39E-21
PARD6B	4,107041	6,55E-25
SRCIN1	4,104695	2,74E-05
ABCC2	4,102596	3,16E-05
LRRC16A	4,094414	1,39E-21
HCN2	4,09238	7,74E-16
PWWP2B	4,083006	2,77E-21
TRIM45	4,08276	9,48E-13
CRACR2B	4,074749	3,67E-19
IFITM10	4,070531	0,000529
ICA1	4,063091	4,28E-30
ARHGAP44	4,061878	5,56E-13
CFAP53	4,060304	9,83E-05
EGLN3	4,056515	5,94E-06
GAB1	4,049854	3,75E-14
SLMO1	4,043447	1,06E-07
GFOD1	4,041105	9,64E-10
KCNJ11	4,023756	7,04E-08
NUDT14	4,017239	1,99E-16
CTSO	4,014815	2,54E-06
RAB26	4,014374	1,24E-06
GATA2	4,008899	3,63E-08

LOC389641	4,008085	0,000182
USP2	3,995146	1,07E-05
CASC10	3,991427	4,95E-07
CYP2S1	3,991152	6,41E-11
FRAS1	3,98015	6,96E-21
HR	3,973897	3,09E-29
MORC4	3,969104	2,24E-20
IER3	3,967031	2,93E-38
SEMA4G	3,966419	6,58E-06
SOCS3	3,962655	7,03E-08
LMTK3	3,957005	1,94E-11
SQSTM1	3,954741	6,58E-27
ZNF608	3,949325	2,58E-23
ASS1	3,946505	1,52E-21
FARP1	3,944499	6,34E-36
CFAP45	3,944143	1,90E-05
CDC42BPG	3,942165	7,16E-08
KLHDC8B	3,940282	4,51E-07
ACKR3	3,937601	1,38E-32
FMO5	3,925187	0,000198
OSGIN1	3,922841	3,20E-15
TLDC1	3,917828	1,26E-24
KRT86	3,914212	0,000877
RUNDC3A-/	3,913529	0,000365
BCYRN1	3,903579	1,21E-31
CCNO	3,903439	1,19E-05
NCOA3	3,901779	1,17E-56
PALM3	3,893151	0,000196
BMPR2	3,886106	3,22E-36
NPDC1	3,883854	5,33E-64
GRHL1	3,883052	2,42E-11
FAM83G	3,88168	6,15E-19
HAGLR	3,880339	0,000107
SUSD4	3,874233	0,000238
PADI2	3,869412	1,70E-05
CDK20	3,866843	4,24E-06
NOTCH3	3,859547	9,43E-47
TNK1	3,856362	5,22E-22
ZBTB38	3,856208	8,73E-23
ADAM11	3,853549	4,93E-10
CGN	3,851985	1,15E-33
ADAM15	3,844301	1,78E-51
GRINA	3,840857	3,20E-24
RASL11B	3,819149	4,35E-09
USP32	3,817496	2,18E-34
CKB	3,816635	1,81E-25
CSPG5	3,809498	1,82E-08
CCDC71L	3,807505	4,31E-16
AJUBA	3,80584	1,16E-15
CDYL	3,805659	4,89E-46

TCEA3	3,803245	6,27E-33
RUSC2	3,802133	5,01E-05
TUBB6	3,798404	7,58E-35
ASAP3	3,7972	5,69E-12
ARRB1	3,79032	1,77E-10
PCAT6	3,786584	0,000876
FUCA2	3,784883	1,74E-25
CTNNA1	3,78438	3,07E-55
ALDH7A1	3,783798	5,80E-40
SEPT3	3,77786	1,58E-15
ZNF239	3,773893	1,04E-08
CFD	3,769192	5,86E-08
CALD1	3,761599	6,98E-05
PVR	3,757249	1,05E-13
CAV1	3,754355	6,21E-06
MGAT4B	3,740349	5,46E-59
C1orf233	3,740194	2,09E-30
FAM213A	3,722981	1,74E-25
TSPAN17	3,721805	4,86E-36
AMOT	3,717635	1,80E-28
STAP2	3,714468	6,02E-31
C9orf116	3,702819	9,21E-07
PVRL1	3,696231	7,91E-21
RP9P	3,69216	9,51E-07
ELMO3	3,674265	1,41E-09
FAM212B	3,674004	5,65E-12
OSR2	3,670292	1,95E-10
JHDM1D-A	3,667192	2,17E-10
NLGN2	3,666709	1,18E-26
LPAR2	3,663594	4,72E-21
C1orf226	3,644911	2,74E-09
FAM174B	3,640756	2,34E-11
MANSC1	3,638285	8,76E-12
MLLT4	3,637844	3,49E-27
FAM86JP	3,637171	1,23E-06
C2orf15	3,632474	9,99E-19
HEATR6	3,624311	7,61E-54
EVI5	3,623112	1,30E-06
RORA	3,621671	1,08E-17
HSPA4L	3,620381	5,17E-13
UBTD1	3,616165	1,12E-06
DST	3,615123	7,97E-30
TIAM2	3,602748	0,000824
TRIM37	3,598611	2,82E-71
CCPG1	3,59806	3,02E-17
EIF4E3	3,595264	4,96E-11
TOX2	3,5888	3,37E-06
PLOD3	3,588095	8,07E-52
COL18A1	3,586433	7,78E-23
CGREF1	3,585664	1,55E-21

CAMK2N1	3,572275	6,17E-08
USP43	3,570567	2,65E-07
TMEM56	3,565736	0,00034
C9orf3	3,559733	3,86E-21
ZNF354A	3,556975	2,03E-23
TTLL7	3,550998	5,44E-08
FRMD6-AS:	3,54454	0,000121
FAM109B	3,542391	2,48E-09
HIST1H2BK	3,538409	1,67E-09
DLX2	3,53378	6,69E-05
SPTBN2	3,533576	1,65E-29
SMARCD3	3,531739	1,70E-06
FAM127A	3,529251	1,87E-15
IQCE	3,528903	3,02E-39
ZNF790-AS	3,527749	2,48E-08
PMEL	3,52428	0,000988
CLCC1	3,522874	2,56E-31
TTC23	3,520178	3,44E-16
KANK1	3,519456	5,72E-15
ABCA2	3,513296	5,04E-30
SKIDA1	3,508967	5,32E-07
RXRA	3,508068	3,51E-24
CDC42EP2	3,507389	1,51E-07
FAM109A	3,506641	3,70E-15
PLXNB2	3,504829	4,87E-31
FAM171A2	3,502111	5,95E-24
GLUD2	3,490558	2,10E-10
ZNF619	3,488673	4,31E-08
PLCD3	3,487616	8,41E-14
ZNF697	3,485993	2,20E-06
FMNL2	3,479557	2,60E-18
APPBP2	3,476857	2,09E-61
AKAP12	3,476214	6,16E-05
RGS3	3,469835	5,13E-18
SAMD11	3,468498	7,16E-07
PAQR7	3,465406	2,41E-12
TMEM254-	3,463872	2,73E-05
AXIN2	3,463443	1,24E-47
CDH24	3,459966	8,99E-15
TPD52	3,451198	6,01E-41
AIF1L	3,443158	3,81E-48
FAM222A	3,44236	7,22E-25
CADM4	3,434139	4,65E-14
MEX3A	3,434101	2,42E-57
TRIM16	3,431476	4,73E-13
MTSS1	3,430806	6,61E-06
ARHGEF19	3,429155	1,87E-17
TP53I3	3,427195	4,04E-08
LINC00467	3,425887	0,000117
HAGHL	3,42181	1,40E-17

PRKAR2A	3,420902	2,06E-09
PRR7	3,416923	4,07E-26
IL6ST	3,41204	7,43E-17
FBXO6	3,410709	0,000127
MGLL	3,408663	4,72E-07
KLF10	3,397023	8,64E-17
CITED4	3,395793	0,000121
ADPRHL1	3,390532	5,14E-13
MT1F	3,381137	5,86E-05
RTN2	3,379216	8,22E-09
CPEB2	3,377261	9,02E-09
HSPB1	3,371525	2,50E-32
GCNT1	3,356012	1,95E-16
RBFOX2	3,35438	6,89E-26
EFNA3	3,352291	2,12E-15
FAM19A5	3,328854	0,000588
PDGFA	3,327261	1,82E-12
ADGRL1	3,326567	5,25E-31
PSMD6	3,325738	7,55E-39
RAB11FIP4	3,325504	5,02E-25
CMTM8	3,322191	1,25E-13
TCTN1	3,307833	8,87E-19
ACVR2A	3,303331	1,09E-09
SH3BGR	3,298706	4,46E-05
LINC00858	3,298215	0,000436
PCGF2	3,292789	2,00E-37
UBE2Q2P2	3,291022	0,000121
TGM1	3,285778	0,000292
NFIX	3,285011	1,56E-16
ZSCAN31	3,281572	7,18E-12
SNX33	3,280635	1,82E-12
TSC22D1	3,278324	8,14E-27
ADGRG1	3,277306	4,07E-26
KIAA1257	3,271322	0,000436
BEX2	3,270171	1,69E-06
CRYL1	3,268917	1,05E-17
AP1S1	3,267016	4,27E-22
TNIK	3,26493	7,47E-10
PTMS	3,26123	6,69E-26
PYCR1	3,253999	1,87E-27
GLIDR	3,245671	9,81E-07
ARHGEF12	3,244451	6,18E-25
TYRO3	3,237715	4,61E-20
PFDN4	3,231499	1,10E-40
RAB27B	3,22708	3,51E-15
RILPL1	3,223603	3,81E-07
MMP15	3,220404	3,75E-19
PCDHB14	3,2203	5,63E-11
BSPRY	3,220033	2,84E-24
ABHD17C	3,217781	1,26E-11

DNAJC6	3,214988	0,000211
CCDC113	3,212492	2,44E-07
LOC11323(	3,192028	5,56E-13
ACTRT3	3,191804	0,00045
SPHK1	3,189866	3,98E-07
SYNGR3	3,187639	0,000274
ADSSL1	3,187628	0,000183
GLS2	3,186486	4,21E-07
GLCE	3,184178	1,45E-35
HDAC11	3,180762	7,42E-06
SERTAD3	3,176212	5,44E-16
FITM2	3,175128	3,59E-10
BCL9L	3,169381	4,39E-23
NR2F6	3,165273	3,56E-16
CUEDC1	3,159026	1,73E-10
CCT6B	3,156308	0,000132
SLC25A13	3,153079	3,48E-30
RPS6KB1	3,15141	2,84E-43
SPATA2L	3,137416	4,86E-21
FGFR3	3,133078	1,82E-13
H1F0	3,13275	3,04E-12
LOXL1	3,125465	7,16E-05
LZTS2	3,125081	7,20E-19
CHN1	3,123908	6,28E-13
LPPR2	3,122389	9,61E-08
FOXO6	3,118667	0,000188
NDRG4	3,101656	1,08E-06
GNA11	3,0973	1,22E-24
ARHGEF11	3,093947	7,28E-18
HHAT	3,091541	5,30E-06
TMEM64	3,088348	2,40E-32
SPPL2A	3,087012	2,66E-36
HSPA2	3,081047	1,15E-06
ZSWIM5	3,079065	8,66E-05
ADAMTS19	3,063133	1,95E-22
FA2H	3,054089	0,000764
JUNB	3,049241	3,53E-14
RTKN	3,044634	2,04E-22
ZNF425	3,04257	1,30E-10
C16orf46	3,040916	3,39E-06
RRBP1	3,025681	4,59E-21
TSC22D3	3,021781	3,21E-05
PLD2	3,021236	1,65E-06
EIF5AL1	3,013404	1,58E-22
KATNAL2	3,009112	4,24E-06
ZDHHC14	3,007885	0,000202
WIPI1	3,00571	7,51E-08
SLC45A3	2,998213	1,51E-05
HOXB7	2,996618	7,28E-09
ADAP1	2,992519	2,41E-06

C9orf172	2,990599	0,000134
CBR3	2,984261	0,000264
ABHD11	2,977869	3,10E-23
MGST2	2,974864	3,64E-09
NHP2	2,973675	1,27E-30
DOK4	2,97089	1,21E-13
BLVRA	2,967468	7,68E-21
NPL	2,964393	1,88E-05
EPB41L5	2,963665	4,67E-24
C2orf81	2,962969	7,03E-07
GUCY1A2	2,957491	1,92E-13
AGAP3	2,956902	2,09E-28
AMIGO1	2,95613	8,51E-05
HIST1H2BD	2,949666	1,89E-06
KCTD1	2,943008	5,42E-18
UFSP1	2,941411	5,15E-05
NR1D2	2,939776	4,15E-20
TMTC2	2,938875	1,02E-08
TSPAN13	2,937212	8,73E-24
SLC22A18	2,930704	3,86E-10
FAM127B	2,925293	1,08E-34
TMEM231	2,924574	5,22E-12
PCDHB13	2,923883	2,04E-07
TMCC2	2,916578	8,02E-06
BMP4	2,916401	8,41E-08
C11orf74	2,911238	1,18E-05
TST	2,906178	6,28E-11
PPM1D	2,901321	6,98E-33
TUBB3	2,901159	3,73E-30
PDE5A	2,901098	0,00041
RGS16	2,896322	2,05E-05
TP53TG1	2,890421	1,46E-11
CHCHD10	2,874939	3,69E-33
HABP4	2,874049	2,52E-11
NRSN2-AS1	2,873945	6,88E-08
S100A6	2,867961	7,53E-12
TSPO	2,861731	2,46E-25
SPAG1	2,857152	1,68E-05
HSPA1B	2,856492	1,29E-11
COBLL1	2,85507	2,38E-15
PLAGL1	2,8536	0,000781
PPFIBP1	2,830032	1,18E-19
C17orf97	2,828225	2,46E-07
PRDM4	2,822199	2,08E-38
CDK18	2,821401	4,48E-08
MOCS1	2,817749	2,88E-05
AUTS2	2,81625	3,00E-16
KIF1C	2,814705	1,36E-22
GPT2	2,803903	1,56E-08
C14orf79	2,795449	1,20E-07

NBPF20	2,793033	3,60E-06
SLC22A31	2,787436	0,000473
HIST2H2BE	2,777777	0,000141
NUDT19	2,776864	1,29E-17
KCNK1	2,773299	4,73E-08
FRMD6	2,769703	1,20E-21
IGF1R	2,766445	2,85E-24
RMND5B	2,762889	1,78E-28
TOB1	2,76173	2,59E-18
TWF1	2,744539	1,21E-24
EFNA4	2,742348	1,02E-19
WASL	2,737039	5,77E-16
CCDC103	2,721151	0,00016
FNBP1L	2,717888	9,98E-30
CTXN1	2,71438	3,17E-19
B4GALT2	2,714297	2,57E-25
DUSP1	2,708855	0,000327
GSTZ1	2,706215	2,45E-22
CERCAM	2,702585	3,80E-09
TMEM2	2,699388	2,33E-20
ZDHHC9	2,6974	3,42E-18
HIGD2A	2,696739	6,99E-24
SUMF1	2,68789	2,23E-14
ARL4A	2,68276	4,91E-23
GAS2L1	2,682314	2,32E-18
VCL	2,676786	4,41E-11
ECI1	2,675083	1,38E-24
LYRM9	2,673314	0,000151
RAPH1	2,669695	5,67E-13
DECR2	2,667555	5,30E-16
MTL5	2,665399	1,18E-07
TTC9	2,660598	5,85E-19
LHFPL2	2,653324	1,82E-11
TATDN1	2,651757	6,31E-24
CCNJL	2,643048	0,00027
PON2	2,640315	6,83E-11
REEP5	2,637469	1,28E-17
TLE2	2,634878	2,74E-17
PHACTR2	2,631291	1,64E-11
BAIAP2-AS:	2,629192	3,18E-05
ANXA4	2,627813	2,05E-18
CLMN	2,627151	1,03E-11
IFNGR2	2,619991	0,000105
TP53I11	2,618945	2,56E-17
C11orf24	2,617109	8,90E-16
RALB	2,614438	2,20E-06
BCAS2	2,613072	1,57E-33
PDE4DIP	2,609004	1,00E-12
IL17RC	2,605513	0,000317
STK32C	2,600006	9,10E-12

GLUL	2,596216	2,50E-30
TUBD1	2,591997	3,26E-16
MYCL	2,588472	0,000254
CCDC24	2,587886	8,39E-06
KIF21A	2,587346	4,38E-11
CAST	2,585622	1,53E-15
TBC1D9B	2,585395	1,16E-28
NME3	2,577825	5,41E-07
LOC100129	2,576905	9,66E-07
METRN	2,576813	9,00E-17
NUDT4	2,571725	1,42E-19
ACOT9	2,570268	2,57E-10
BHLHE40	2,568412	7,01E-16
ZFP36L1	2,567718	4,46E-19
TMEM63B	2,567289	4,19E-15
ZNF35	2,565776	1,57E-07
KCNIP3	2,564923	3,55E-05
LRRC37A3	2,562932	3,06E-06
VPS37C	2,559553	5,10E-17
MSRB2	2,558971	4,39E-18
VPS9D1-AS	2,558108	5,28E-20
PLEKHB1	2,557468	2,77E-05
NEK3	2,548311	1,02E-05
DNAJC15	2,546348	9,80E-17
TMEM38A	2,544813	0,000139
AKT1S1	2,543487	8,57E-16
SGPL1	2,541782	9,30E-20
SPATC1L	2,540355	3,36E-11
DYRK1B	2,539614	3,30E-11
GALNT3	2,536527	1,99E-15
COQ2	2,535299	3,20E-12
EPB41L4A-/	2,534201	1,01E-08
RAB34	2,528264	1,21E-12
PFKM	2,524629	3,96E-15
ZCWPW1	2,523461	2,08E-05
TANC2	2,518974	3,19E-14
PCDHGC3	2,517327	2,49E-07
CRISPLD1	2,516473	2,10E-06
RRAS2	2,515458	6,82E-08
ABCG1	2,510204	4,32E-10
BATF	2,505279	0,000683
SERINC2	2,50511	3,62E-07
COTL1	2,504854	2,61E-13
AAED1	2,500685	0,000174
PRKCD	2,499758	1,18E-09
FAM195A	2,497627	3,05E-17
MYO1B	2,49661	2,79E-26
CASZ1	2,494148	1,56E-09
YBX2	2,487387	6,11E-16
WWP1	2,484647	2,25E-11

MYL5	2,480527	0,000407
CASKIN1	2,475997	6,20E-06
ELF4	2,470606	3,73E-10
GPR153	2,465669	2,54E-08
PRELID1	2,462545	1,16E-18
SPRYD3	2,462206	1,35E-19
PODXL	2,461062	2,42E-16
ACSL1	2,460665	2,52E-12
NOP16	2,458899	7,77E-27
MAFG-AS1	2,458237	6,38E-06
ISOC2	2,457596	1,31E-14
TM7SF2	2,456464	1,07E-07
FCHSD1	2,456003	3,91E-05
EXPH5	2,455755	1,71E-12
PPP1R13L	2,452562	6,38E-09
ACTN1	2,451875	6,23E-15
SLC50A1	2,443989	4,12E-21
ZNF783	2,442588	4,79E-06
DYRK2	2,442516	7,35E-11
SRGAP2B	2,438895	5,95E-06
C17orf58	2,435159	0,000779
ATP6V1E2	2,432822	0,000132
NMB	2,426238	5,75E-06
MICA	2,425808	1,50E-06
SLC7A5	2,42449	8,02E-19
GOLGA2P7	2,422129	1,57E-05
DTWD1	2,421131	5,55E-13
C6orf1	2,419102	2,28E-09
IDH1	2,411309	2,90E-30
MPZL3	2,408871	1,81E-07
GGA2	2,407005	7,32E-17
LRP5	2,405615	1,33E-16
SLC39A4	2,402283	1,02E-11
RPL17	2,399199	8,20E-13
BRIP1	2,3954	6,61E-24
TMEM183I	2,392067	5,93E-07
ATP6V1C2	2,389595	5,60E-05
CTAGE5	2,389569	1,65E-06
KIAA0040	2,38933	3,42E-07
KANK2	2,388883	1,39E-14
SERINC5	2,387714	2,21E-12
GRN	2,386883	2,87E-10
MYBL1	2,384082	2,38E-05
PRMT6	2,383733	2,88E-23
KIAA1191	2,380588	1,49E-24
SYNGR2	2,380024	1,45E-12
PPFIA3	2,378918	7,54E-15
PQLC3	2,378798	9,68E-08
MAP3K6	2,376035	2,54E-13
ZNF620	2,375615	4,79E-07

ZSCAN12P1	2,369422	0,000282
DLG5	2,369041	4,47E-25
CSDE1	2,365221	7,22E-19
SEZ6L2	2,362676	1,48E-11
SPA17	2,359472	2,94E-06
DUSP3	2,355476	2,72E-16
IGF2BP2	2,351035	6,36E-05
TXNRD3	2,347849	0,000453
ZSCAN16	2,344905	7,06E-11
PAIP2B	2,344868	1,24E-08
ABCB9	2,343744	8,73E-05
NSD1	2,336226	8,44E-30
PDLIM7	2,333354	3,19E-16
GAS8	2,332942	4,04E-12
GPC1	2,332219	4,17E-06
ZNF572	2,327907	8,86E-05
RAB3D	2,325245	1,39E-13
LY6E	2,32448	4,77E-09
TTC39B	2,324464	0,000533
SLC12A9	2,319851	1,57E-08
PELI3	2,316108	4,21E-10
PXN	2,315516	4,86E-14
B4GALT7	2,313494	2,41E-08
F11R	2,309275	2,92E-24
PFKFB2	2,309104	2,74E-09
DNAAF3	2,309049	4,99E-08
P2RX4	2,308035	1,80E-10
IDNK	2,301288	3,22E-05
FUOM	2,298551	3,15E-06
KDM1B	2,28982	2,73E-07
SEC11A	2,288061	2,86E-26
GRAMD3	2,286854	3,04E-06
ANXA5	2,282527	4,02E-20
TBK1	2,279727	1,22E-09
CTSB	2,278628	6,85E-09
LOC728554	2,278525	1,22E-09
LYPLAL1	2,275528	7,81E-08
NRSN2	2,272602	3,88E-09
SLC22A23	2,271001	1,46E-19
ALCAM	2,264385	9,89E-13
FAM102B	2,263146	5,47E-11
TMCO4	2,262263	1,58E-07
YES1	2,262002	1,25E-20
MT2A	2,261669	2,95E-13
TNKS1BP1	2,25889	6,07E-22
FBXO31	2,252405	1,46E-15
ELL3	2,252067	2,59E-07
FIS1	2,248581	1,01E-20
TSEN34	2,247082	9,73E-15
VAV2	2,246961	4,20E-15

GATAD1	2,246437	3,15E-08
MYL12B	2,246267	5,48E-25
VSIG10L	2,243556	5,32E-06
COL9A2	2,243007	0,000116
UNKL	2,241225	2,02E-05
JOSD2	2,239335	4,00E-06
CREB3L4	2,237779	1,21E-13
STYXL1	2,233487	3,35E-13
ASNS	2,230146	1,12E-07
OPLAH	2,23001	6,76E-09
FAM131A	2,228852	0,000284
FZD5	2,226855	2,20E-07
SLC2A11	2,219396	1,07E-07
DNLZ	2,216273	1,16E-11
ATXN7L1	2,214065	1,36E-08
ZNF217	2,212557	4,60E-24
THG1L	2,21155	1,27E-15
TPMT	2,210207	5,24E-06
TPM1	2,209783	1,29E-14
TMED9	2,209376	3,07E-10
FHIT	2,207686	0,000789
TRAPPC2L	2,20693	1,36E-18
TBL1X	2,199735	5,18E-09
KDELR2	2,198976	1,21E-17
FRS2	2,195377	1,38E-11
KCTD15	2,192046	4,65E-07
SLC25A39	2,182387	1,68E-20
SPINT2	2,177898	2,04E-24
ANKRD42	2,174559	3,20E-06
DTX4	2,174169	6,14E-05
DBN1	2,17405	5,62E-20
FAM86EP	2,171735	0,000237
PUS7	2,167925	2,02E-12
DOC2A	2,164561	0,000165
ROGDI	2,164405	4,92E-10
CLIP2	2,164212	3,37E-05
RAB24	2,157374	8,76E-07
GLMP	2,153158	2,36E-06
SH2B2	2,149436	2,14E-06
CNIH2	2,146257	3,15E-05
SLC52A2	2,142099	1,32E-14
MICALL2	2,141636	1,52E-07
MAPK12	2,141008	8,04E-05
B9D1	2,138493	5,97E-05
PTGR2	2,134965	1,16E-06
TRIM2	2,131093	8,18E-07
CHPF	2,126416	1,42E-06
ZSCAN21	2,117161	4,14E-12
RAC3	2,114233	2,02E-10
DYNLT1	2,112129	8,99E-12

NRBP1	2,111904	8,43E-19
FNDC3B	2,108807	9,94E-12
RNLS	2,108296	0,000468
SLC35B3	2,104882	1,71E-07
PIAS3	2,104628	6,30E-19
MSX1	2,103827	4,52E-05
THOC3	2,102831	9,68E-11
ACOX3	2,102569	1,26E-08
SIAH2	2,09937	4,27E-13
GADD45A	2,095003	2,44E-08
DSTYK	2,094099	2,12E-12
ZSCAN9	2,086666	1,24E-05
PLXNB1	2,078116	1,93E-06
DNASE2	2,078058	2,18E-11
KNOP1	2,077226	6,05E-16
HYAL2	2,072416	8,57E-16
PRCP	2,071639	1,57E-10
PPCDC	2,069247	1,39E-07
RARA	2,068252	2,43E-12
ZNF395	2,061578	9,54E-12
EIF3E	2,058378	5,80E-13
ORC5	2,058153	3,09E-08
GALNS	2,057103	1,78E-07
DTD1	2,057041	1,17E-10
ASNA1	2,056704	1,44E-14
RHOBTB3	2,05657	5,94E-15
ZFP62	2,056509	1,05E-16
EXD2	2,056027	1,02E-15
ATP9A	2,052515	1,26E-17
ICK	2,052252	2,94E-12
RUSC1	2,051954	6,75E-15
ATP6V0E1	2,050372	2,03E-08
CD63	2,050289	5,30E-10
OSCP1	2,048691	0,000819
PER3	2,048083	0,000503
RUFY1	2,047696	4,23E-14
UEVLD	2,045737	0,000134
SNX24	2,044607	1,90E-06
DAG1	2,04319	1,32E-12
ZNF485	2,039627	0,000193
GIPC1	2,038946	1,15E-10
SMURF1	2,038359	4,62E-15
KCTD3	2,037843	3,74E-17
CTTNBP2NI	2,037159	9,67E-10
ARHGAP10	2,036239	3,83E-11
ISYNA1	2,035948	3,57E-14
TMEM9	2,033915	3,30E-17
SNX27	2,033463	1,22E-13
ID3	2,03315	5,95E-06
C7orf50	2,029052	4,52E-11

APRT	2,02883	1,50E-12
CPLX1	2,025532	3,06E-05
LMAN2	2,02384	1,06E-11
USP8	2,023325	1,03E-19
CELSR2	2,023279	1,27E-13
ANO10	2,020196	6,85E-06
IER2	2,014347	8,69E-05
OSBPL1A	2,010952	3,85E-12
KLHL22	2,008793	2,29E-06
C4orf48	2,005191	1,35E-06
SNX18	2,00356	1,85E-10
IQSEC1	2,003257	1,78E-12
RAD51C	1,997582	4,69E-20
FAF2	1,99713	5,27E-19
USP31	1,996081	1,83E-08
LTBP3	1,990633	3,05E-12
HARS2	1,98967	1,61E-15
FTH1	1,985734	1,86E-07
ANXA2	1,984334	4,14E-17
FAM129B	1,98312	2,05E-12
ARHGAP12	1,982867	1,95E-06
LDLRAP1	1,980934	0,00026
HNRNPAB	1,977358	2,40E-19
MEPCE	1,973047	7,25E-15
VPS72	1,972077	7,07E-10
MYL12A	1,968622	1,28E-17
HEATR5A	1,967794	7,96E-10
C5	1,967231	0,000559
TRAPPC6A	1,964882	9,80E-08
ACACB	1,959299	4,95E-08
TMOD3	1,958007	9,81E-20
POLR3C	1,956652	5,33E-07
TMTC3	1,955918	1,38E-09
GSTM4	1,95454	1,23E-12
GNB2	1,95051	2,66E-15
NAAA	1,950375	3,82E-06
DNAJA4	1,950346	1,49E-06
GCSH	1,949564	2,29E-14
NBPF9	1,946154	2,96E-06
TRAF4	1,943589	1,93E-14
HEBP1	1,942929	9,20E-09
DDAH2	1,937674	2,15E-11
KHK	1,936154	1,59E-09
NAMPT	1,933522	5,46E-17
KDM5B	1,929636	3,00E-10
HS1BP3	1,928572	0,000215
KLHL36	1,928344	5,28E-13
DUS4L	1,926907	8,08E-09
SLC16A13	1,925784	0,000534
NRAV	1,923028	5,76E-07

SRD5A1	1,922843	8,60E-07
MFSD3	1,92224	1,96E-12
THOC7	1,921512	1,42E-13
RNF208	1,920684	2,58E-06
GPSM2	1,919449	2,39E-14
SAPCD2	1,917438	1,27E-09
SLC7A11	1,913377	2,90E-09
FSCN1	1,911618	1,73E-09
MAPK6	1,911183	1,27E-18
ERF	1,910231	2,93E-07
CORO1B	1,90939	1,26E-06
DYNC2H1	1,907388	6,15E-05
HTATIP2	1,90026	1,38E-07
DHTKD1	1,899731	2,25E-13
TMSB10	1,899365	1,28E-10
SLC3A2	1,896433	1,01E-16
CANX	1,895731	9,67E-16
CDC42SE1	1,895236	5,97E-13
TRIM3	1,893438	0,000368
RPS6KL1	1,892957	0,000369
IMP4	1,892705	6,22E-18
PLBD2	1,887409	3,46E-07
LTBP1	1,887118	1,31E-11
AGTRAP	1,881971	1,73E-06
ATP11A	1,880161	3,55E-06
IQCK	1,877337	8,92E-06
PCYOX1	1,876807	2,44E-09
ALKBH2	1,875532	2,28E-08
TTC8	1,869912	2,85E-08
FAM227B	1,869401	0,000211
SLC35B2	1,867366	1,82E-09
MARVELD1	1,866656	4,65E-07
LNX2	1,861283	3,95E-14
ECHS1	1,860965	2,09E-10
CCDC6	1,857749	1,74E-13
VMP1	1,857694	4,97E-13
RAB12	1,857647	3,94E-10
RGL2	1,856931	6,74E-05
TMEM241	1,853832	1,60E-06
ADCK1	1,853222	8,62E-06
CNNM4	1,851102	2,35E-09
VTI1B	1,850684	1,74E-09
SLC4A2	1,850421	1,04E-15
ANKEF1	1,849492	3,27E-07
DDX41	1,849106	5,25E-17
CYP51A1	1,848237	1,30E-15
CLDN12	1,848174	1,37E-05
ANKRD50	1,846818	2,55E-14
CYB5R1	1,838843	2,22E-06
TP53INP2	1,833244	0,000366

BPHL	1,822595	1,66E-07
ATMIN	1,822505	3,40E-15
KANSL1L	1,819918	0,000874
HES4	1,817268	0,000315
GNPNAT1	1,813997	3,76E-12
RHOC	1,813916	3,56E-07
FAM63A	1,813071	0,000307
ADI1	1,81254	8,69E-12
TMED3	1,804151	2,06E-09
GNB2L1	1,803625	8,80E-12
SLC44A1	1,80313	7,64E-14
MGST3	1,801296	8,12E-06
COMTD1	1,800736	9,14E-09
NCKAP1	1,799229	8,69E-12
SLC39A11	1,795094	5,14E-05
MRPL24	1,794068	3,41E-09
CRTAP	1,792038	2,03E-11
NPM1	1,790059	4,62E-15
C1GALT1C1	1,78922	0,000131
ABCD1	1,786642	0,000795
SIRT5	1,784023	4,28E-08
FBXW9	1,77854	2,77E-09
MAML1	1,777726	3,19E-10
SOX4	1,776872	1,55E-12
IMPDH1	1,776529	9,21E-12
B4GALT1	1,772425	2,03E-11
EGFL7	1,76919	7,27E-07
SLC29A3	1,768482	0,000623
NDUFB9	1,768455	3,58E-09
ITPKC	1,768037	1,82E-07
ETS2	1,767145	3,46E-10
AEN	1,765436	7,14E-13
POLR2J	1,760485	2,59E-10
CYB5D2	1,758297	3,15E-05
STRA13	1,758257	1,48E-13
BMPR1A	1,756095	1,10E-08
CYFIP1	1,754003	1,96E-13
ITFG3	1,753639	2,35E-07
DLD	1,752615	2,81E-09
PPP1R16A	1,751617	1,27E-10
UBL3	1,748478	4,84E-08
APLP2	1,740871	5,89E-16
ARPC1B	1,740292	2,06E-09
FAM185A	1,738997	0,00027
MORN2	1,737878	0,000403
AKTIP	1,735471	0,000116
MAP3K9	1,735043	2,63E-07
SPG21	1,733853	3,57E-13
PYCARD	1,73377	0,000153
SYAP1	1,732194	3,66E-11

PNRC1	1,729324	1,66E-07
OCRL	1,725617	1,49E-07
ZNF687	1,721023	1,45E-13
NEU1	1,719258	0,000385
TMEM251	1,71794	6,42E-08
GABPB1-AS	1,717407	0,000489
CLTB	1,716208	1,73E-05
SMAD3	1,716046	0,000426
XPOT	1,714029	3,09E-10
GATSL2	1,711863	2,68E-08
CACNB3	1,711712	3,37E-11
WDR83OS	1,710454	6,06E-13
AIMP2	1,70949	1,52E-10
TACO1	1,709474	1,86E-09
PICK1	1,705587	4,33E-05
FRMD4A	1,704951	6,28E-05
HBP1	1,703608	0,000157
DLGAP4	1,703029	3,84E-09
ZBTB12	1,702798	0,000639
ARPC1A	1,700596	1,32E-10
ACTR3B	1,697739	3,21E-06
IFT22	1,696669	8,48E-09
AK3	1,695462	2,11E-08
RNF103	1,695456	2,51E-06
PHTF1	1,695451	0,000392
RPP40	1,695219	8,76E-07
RMND1	1,693265	5,02E-10
FAM65A	1,691866	4,39E-10
ITGA2	1,691673	4,16E-05
SMARCD2	1,691069	8,86E-13
TMEM5	1,690062	1,86E-09
DCAF4	1,688446	0,000151
TRAK2	1,688223	6,71E-11
ARMC10	1,684134	7,57E-12
FAHD1	1,684026	1,52E-09
ZBTB41	1,683172	3,28E-07
DCLRE1B	1,682057	4,68E-09
NDUFV3	1,678027	5,37E-12
ZNF3	1,677977	1,54E-10
BRPF3	1,677158	1,29E-11
GGCX	1,674451	4,76E-08
DPCD	1,673772	6,75E-05
PTRH2	1,670848	7,76E-09
CPNE3	1,670335	1,00E-12
TCEAL4	1,6701	3,68E-12
PDXK	1,668159	1,64E-09
HPCAL1	1,664853	2,02E-05
FAM102A	1,663729	1,62E-11
ERI2	1,661649	5,21E-07
SH3YL1	1,659777	3,48E-06

DIRC2	1,659023	0,000303
ZNF768	1,657941	4,85E-11
GAS5	1,657568	0,000969
CEBPZOS	1,657509	3,71E-07
SLC44A2	1,652677	6,41E-10
NPC2	1,652163	9,41E-08
DNAJC2	1,652035	1,13E-12
TMEM102	1,648836	8,49E-05
BUD31	1,648786	7,83E-11
RNF44	1,646388	5,52E-10
NGFRAP1	1,645649	8,78E-13
IFRD1	1,645319	0,000836
DNAJC1	1,645018	2,77E-09
TSPAN31	1,644609	0,0004
PPDPF	1,644153	1,76E-07
C12orf66	1,642898	0,000383
CFL2	1,641238	0,000161
PIGB	1,640869	7,84E-06
LYSMD2	1,639317	2,65E-06
CYBA	1,638581	1,03E-07
PPP1R37	1,636879	0,000321
TRPM7	1,636285	2,41E-08
SCRIB	1,635161	1,33E-09
FAM86C1	1,634482	2,62E-06
STUB1	1,633495	3,56E-11
KARS	1,632385	1,96E-09
RNF122	1,631524	0,000367
GIN1	1,627993	0,000328
PHLPP1	1,626128	3,97E-10
ATG101	1,625546	3,99E-08
SDCCAG3	1,625015	0,000818
TRIM33	1,623884	1,77E-11
ZKSCAN1	1,622987	5,72E-13
AP2S1	1,622921	1,32E-09
TIGD5	1,622783	0,000318
PLEKHF2	1,622125	9,22E-10
SLC37A1	1,621751	0,000568
CHCHD5	1,621725	5,76E-08
SGK223	1,620567	1,18E-05
TVP23B	1,620441	3,27E-06
TMEM134	1,618455	4,95E-06
NAPG	1,618152	1,40E-06
MANBAL	1,617899	3,27E-08
PIP4K2C	1,616431	4,16E-07
BTG2	1,611756	0,000398
CCDC47	1,611573	1,97E-12
LAMTOR4	1,611057	4,15E-11
MTIF3	1,609839	3,70E-06
FAM50A	1,60699	2,64E-10
CYC1	1,605585	7,20E-09

ZNF219	1,603483	1,34E-07
SMURF2	1,602748	1,89E-07
ZNF778	1,599922	9,74E-07
HILPDA	1,599088	3,02E-06
RDH13	1,597491	1,69E-07
MOSPD3	1,596098	0,000631
GPR89A	1,595283	3,23E-05
TMBIM4	1,594029	7,93E-07
SLC35E3	1,59253	3,74E-06
MDM2	1,591937	3,14E-05
NRAS	1,590777	1,91E-10
IFT43	1,589247	6,07E-06
AGPAT3	1,587047	6,81E-09
HRSP12	1,585168	5,99E-05
SLC25A24	1,583891	1,01E-10
BNIP3	1,583478	6,88E-09
ATG14	1,581801	0,000555
NUBP1	1,578876	3,09E-05
SCARB1	1,576463	7,71E-07
POR	1,575869	1,90E-10
CALM1	1,575776	8,93E-09
RHOQ	1,575628	2,64E-06
CLOCK	1,575539	4,48E-08
RNF115	1,568621	9,83E-08
LOC642852	1,566099	0,000171
BCAP31	1,56451	6,48E-08
ITPK1	1,563378	2,44E-08
C12orf45	1,562611	8,19E-06
ZNF786	1,562526	1,48E-06
CLN3	1,561745	3,04E-05
EPHX1	1,559897	0,001015
MRPS21	1,559756	7,91E-12
LLPH	1,556938	7,41E-11
PRDX4	1,556391	4,25E-07
PRDX2	1,554877	4,23E-08
HSCB	1,554837	3,40E-05
NAB2	1,553778	0,000373
PSMD4	1,553682	6,54E-14
TMEM192	1,553109	1,96E-06
GLRX2	1,550178	2,60E-05
PNMA1	1,54782	1,02E-08
APH1A	1,547485	3,03E-11
ZDHHC21	1,546643	0,000202
IMPA2	1,544536	3,79E-07
GBA	1,543074	1,80E-05
FLAD1	1,542251	5,80E-11
ILVBL	1,539495	5,17E-08
LEO1	1,535061	1,61E-12
BRI3	1,534563	1,02E-07
SLC19A2	1,532016	0,000284

SMAGP	1,531767	4,46E-07
TRAP1	1,530174	4,01E-08
BOLA1	1,529471	3,21E-06
ZNF627	1,527652	1,59E-06
ZNF629	1,527448	2,17E-08
SLC25A10	1,527095	3,07E-06
RBAK	1,525599	1,17E-05
THNSL1	1,525027	1,54E-07
SIL1	1,525018	2,12E-05
PAM	1,522979	2,11E-05
TMEM168	1,518232	4,61E-08
DYNLL2	1,518149	4,78E-12
TIMM9	1,515675	2,28E-05
YIF1A	1,514666	1,56E-10
WIBG	1,514027	8,81E-08
IQCH-AS1	1,513697	0,000164
TAF13	1,513058	3,94E-05
TMEM14A	1,510311	5,61E-05
HMG20B	1,508701	1,08E-10
ANKIB1	1,508467	6,27E-08
TIGD6	1,505229	0,000336
FBXW4	1,504518	2,04E-06
IER5	1,504305	1,49E-06
RNASET2	1,503521	0,000191
USP3	1,502456	8,92E-10
MDH2	1,501817	8,87E-08
SLC39A6	1,50136	1,07E-06
TRIM24	1,499819	8,66E-10
OTUD7B	1,498359	1,65E-08
CCDC112	1,498161	3,73E-05
ARPP19	1,495216	4,36E-11
ARRDC3	1,493257	5,08E-05
ACOT13	1,492961	6,83E-07
KDELR1	1,490497	9,84E-09
SIMC1	1,487332	0,000679
TRMT12	1,486723	9,26E-06
TRMT5	1,483955	3,68E-08
TPRN	1,481973	1,61E-06
EBAG9	1,481905	2,66E-06
MGC72080	1,481407	1,86E-05
NIT2	1,481369	6,06E-08
METTL8	1,479558	5,54E-08
BAG3	1,477726	0,000255
MTCH1	1,477652	3,21E-10
TKFC	1,476742	1,03E-07
RP9	1,476272	1,77E-05
DAAM1	1,475696	2,26E-07
PAK4	1,475589	7,84E-08
PDCD4	1,475508	4,26E-07
SLC25A30	1,47459	2,07E-06

AFG3L2	1,47421	2,03E-11
NR2C2AP	1,473447	0,000787
RNF13	1,468237	2,57E-08
STX7	1,468133	3,61E-05
PAWR	1,467924	3,55E-05
KLF7	1,467201	0,00036
NSMCE2	1,46262	8,90E-07
GDE1	1,461951	1,10E-06
VRK2	1,45659	3,08E-05
CRTC1	1,454171	5,26E-07
PTPN18	1,451674	9,22E-07
SRGAP2	1,450894	2,65E-10
ARL6IP1	1,449268	4,95E-11
PPP1R15A	1,447541	0,000143
TMEM205	1,446418	4,81E-05
EFCAB11	1,444752	0,000161
DNPH1	1,444372	4,99E-07
PPP1R3D	1,443388	0,000376
MED21	1,442432	6,03E-06
SEMA4B	1,440445	2,16E-05
ZMIZ2	1,44007	7,33E-11
SLC35F2	1,436611	9,09E-05
SNHG16	1,435611	1,73E-08
ZBTB4	1,435378	8,22E-09
TMEM159	1,432902	0,00027
FUT8	1,432162	1,42E-09
ZMIZ1	1,430254	2,15E-07
MPDU1	1,429669	9,83E-07
HIPK1	1,428048	6,80E-08
PSMB5	1,427908	2,23E-08
PTEN	1,424975	9,73E-09
INPPL1	1,424892	2,52E-09
ARF3	1,421318	3,47E-08
ZNF74	1,421106	1,22E-07
MIPEP	1,420795	1,23E-06
TMEM183A	1,419527	1,05E-06
BCL2L11	1,417491	1,38E-05
NMD3	1,417248	2,93E-07
DHCR7	1,415543	2,53E-05
STK36	1,415406	5,24E-05
MRFAP1	1,414993	1,04E-08
PSEN1	1,413366	1,45E-07
RWDD2B	1,411449	4,19E-05
CD3EAP	1,409149	1,13E-05
GABPB1	1,406968	3,36E-06
COX5B	1,406201	6,88E-09
SPOPL	1,402499	2,12E-05
GPATCH2	1,401973	5,94E-06
GNS	1,401511	0,000559
MFSD10	1,39572	1,61E-07

ESRRA	1,39566	1,07E-07
MXD4	1,394457	0,001013
TIGAR	1,393007	3,30E-05
OSGIN2	1,392351	4,74E-06
ACAA2	1,391852	3,48E-05
BCAP29	1,390186	1,12E-06
PSMC2	1,390047	2,04E-09
NTMT1	1,387835	4,07E-08
SLC39A1	1,387611	7,87E-05
ZNF787	1,387369	6,34E-07
ENSA	1,386356	7,84E-12
TOMM7	1,384766	1,07E-06
ARFIP2	1,38449	3,81E-06
ALDH1B1	1,380067	3,62E-07
PIK3R2	1,37896	5,61E-05
KIF20A	1,378892	1,53E-07
FKBPL	1,377681	0,000177
TNIP2	1,376207	6,24E-08
SLC25A1	1,375212	2,94E-05
CARM1	1,374885	5,26E-07
KRCC1	1,372927	7,51E-05
MGAT1	1,372396	3,50E-06
GTF2IRD2B	1,371907	0,000565
TES	1,371035	0,000195
PTPMT1	1,371002	1,12E-06
MRPS2	1,367544	9,91E-08
NBN	1,367307	2,72E-10
ARL8A	1,366093	2,81E-07
METTL2A	1,365909	7,99E-06
COPS6	1,365459	2,25E-07
KDM6B	1,365147	2,60E-07
ATP5J2	1,363248	6,53E-06
C5orf30	1,362962	1,92E-06
FAM177A1	1,362826	2,89E-05
ASAH1	1,360368	0,000123
ENDOV	1,360151	0,000103
UIMC1	1,359128	0,000358
SWI5	1,357365	1,31E-05
PYCRL	1,35671	5,18E-06
TNPO2	1,3559	0,000522
CNOT6	1,353709	2,11E-08
ACTN4	1,352529	6,39E-06
RPL26L1	1,347391	4,30E-05
GOLGA5	1,346393	1,88E-05
RHEB	1,345284	3,35E-07
OGG1	1,344886	3,23E-05
SCAMP1	1,343543	1,85E-06
MCL1	1,342561	9,26E-11
LGALS1	1,342401	0,000756
KIF3B	1,342306	1,80E-06

SAT1	1,341889	4,22E-05
RAB1A	1,33925	7,31E-06
MTERF1	1,335725	0,000233
SMC6	1,334113	5,34E-06
CENPB	1,333778	7,70E-08
NFIC	1,332528	3,71E-08
JUND	1,331575	0,00042
ALKBH4	1,328761	5,41E-06
TAGLN2	1,328738	2,57E-06
TIPARP	1,326479	0,000289
SLC29A4	1,323861	0,000169
SLIRP	1,323231	7,15E-06
NUMA1	1,322705	1,12E-09
UQCC3	1,322663	2,58E-06
SIPA1L1	1,322662	6,82E-08
KCMF1	1,322607	1,64E-06
DNAJC30	1,32237	4,56E-05
ARF5	1,321948	3,82E-08
TCTN3	1,320822	9,42E-06
RAB3IP	1,320721	1,45E-08
TSTA3	1,315143	1,71E-07
PRDX6	1,314907	4,59E-06
TAMM41	1,313512	3,24E-05
YIPF2	1,308829	1,41E-06
PLP2	1,308764	1,21E-05
COX4I1	1,308619	1,20E-07
DNAAF2	1,308066	3,15E-06
DHRS7B	1,307009	6,47E-05
SLC35F5	1,305509	0,000726
TBCC	1,30447	3,33E-05
PABPC1	1,304254	2,04E-07
PEX13	1,303558	9,82E-06
RBSN	1,301577	7,90E-06
MRPS25	1,300009	3,33E-06
PSRC1	1,298655	0,00027
ATP6V0E2	1,297221	0,000379
TMEM141	1,295593	4,94E-07
SLC24A1	1,295401	3,44E-05
NLN	1,294683	4,13E-08
DARS2	1,292713	4,26E-07
SUV420H1	1,291941	1,91E-07
HSBP1	1,290924	1,82E-08
MED13L	1,290741	1,64E-06
NGRN	1,288507	4,53E-08
CRK	1,288012	2,14E-08
DUSP16	1,287984	1,50E-06
HDDC2	1,28789	4,72E-07
TMEM120A	1,287618	0,000254
CLTC	1,285303	6,82E-06
BLOC1S3	1,284653	0,000144

CERS2	1,282729	2,02E-08
SHB	1,282272	0,000485
TFG	1,281317	1,26E-07
DCXR	1,280824	3,94E-06
C20orf27	1,280484	8,81E-06
NQO2	1,280303	2,99E-05
WDR73	1,279347	0,000741
CHCHD4	1,27814	0,000542
PTK7	1,277313	5,22E-07
COX6C	1,27709	4,80E-08
FAM3A	1,274302	1,24E-05
BCL2L1	1,271042	0,000176
ZNF252P	1,270595	5,98E-07
NACC1	1,26997	3,12E-07
SLC10A3	1,26863	0,000511
ATP5H	1,266764	4,20E-06
PDAP1	1,266724	3,18E-08
MAP3K1	1,266641	7,07E-05
ZBTB42	1,266526	1,89E-05
FOSL2	1,266451	0,000355
TAF11	1,265393	7,52E-07
COA4	1,264717	7,95E-07
SHFM1	1,264474	1,76E-08
EARS2	1,2641	3,18E-06
TMEM127	1,264048	8,76E-06
MCCC2	1,263074	2,20E-06
KLHDC10	1,261086	7,88E-07
WDYHV1	1,259159	0,000439
ZNF480	1,257215	2,82E-05
FKBP4	1,253838	8,69E-05
ATP5D	1,253279	2,21E-06
MRPS12	1,253164	5,28E-06
RMDN1	1,251834	5,33E-06
RNF149	1,251721	7,89E-06
RINT1	1,251463	7,97E-05
UBE3C	1,247648	1,99E-06
RPL30	1,247488	8,98E-05
CBLL1	1,246816	3,99E-06
ZNF623	1,246344	3,47E-05
MPP5	1,246325	1,01E-06
TEX2	1,245697	2,96E-07
NDUFB10	1,242941	1,32E-05
ECE2	1,241076	0,000764
TMEM43	1,239009	5,47E-05
MAPK9	1,237438	6,92E-07
SYPL1	1,23553	5,00E-07
CNOT4	1,234823	1,10E-05
HMGB3	1,234822	3,69E-09
FAM83D	1,233783	4,86E-06
MLKL	1,233525	0,000523

CHURC1	1,233449	0,000131
DLG3	1,232295	1,41E-06
RBM8A	1,231705	6,49E-08
PEA15	1,231642	1,71E-05
PTPN12	1,229779	3,04E-06
SFXN4	1,229605	7,52E-06
PLCB3	1,227992	0,000111
ZNF398	1,227973	6,71E-07
MRPL14	1,22782	5,07E-06
PITPNC1	1,227416	2,28E-06
DSTN	1,22707	0,00012
TARS2	1,226081	2,61E-05
CUX1	1,225623	1,28E-06
ATP1A1	1,22511	4,14E-06
NANS	1,222446	0,000335
C6orf47	1,221213	2,61E-06
SF3B4	1,220983	9,23E-07
CBX2	1,220275	1,36E-06
GCC1	1,220224	8,46E-06
RNF14	1,220045	0,000122
AVL9	1,218866	1,07E-07
SETDB1	1,217902	2,72E-06
TBL2	1,216717	2,45E-05
NEO1	1,216582	7,57E-06
EMC2	1,215569	0,000143
C21orf33	1,215361	0,000227
HDDC3	1,213456	0,000179
SOX12	1,213283	1,11E-05
PIK3C2A	1,212936	0,000254
NPC1	1,210725	7,70E-06
ZBTB9	1,204243	0,000257
EEFSEC	1,203353	0,000325
TMEM87B	1,200912	0,00059
SLC37A4	1,199896	2,07E-06
RPL12	1,199791	0,000193
HEBP2	1,196898	0,000787
PSMB4	1,195779	2,71E-06
ZNF277	1,195291	0,00013
MREG	1,192551	4,67E-05
POLR1D	1,191314	4,64E-08
PSMD7	1,189686	2,34E-08
CSTB	1,187441	4,84E-06
NFKBIL1	1,182931	5,36E-05
PTTG1IP	1,182912	9,65E-07
PRKCI	1,18216	2,65E-06
BCKDK	1,180503	6,74E-05
NDUFS2	1,178776	4,70E-05
CHPT1	1,178399	6,87E-05
FAM220A	1,177271	0,000863
DPY19L1	1,177167	0,000597

GGCT	1,177023	1,05E-06
MCU	1,175645	0,00049
EMC3	1,174979	0,000523
AP4E1	1,174565	7,59E-05
PPIL1	1,172993	4,40E-06
RHBDD2	1,171968	0,001013
ZNF710	1,170862	8,93E-05
TMEM184I	1,170254	0,000125
SMUG1	1,170009	0,000786
FZD1	1,16909	0,000106
ALDH6A1	1,168882	9,07E-06
BRMS1	1,168832	3,37E-05
PRKAG1	1,168704	0,000168
SURF2	1,168022	7,27E-05
ATP6AP1	1,167192	0,000121
MRPL42	1,167099	3,67E-06
ATN1	1,165406	5,05E-07
TUBB4B	1,165376	2,58E-05
CETN2	1,165316	0,000807
ANGEL1	1,164597	2,51E-05
ASAP1	1,16314	2,11E-06
ASH1L	1,160903	3,64E-05
CUL7	1,16015	0,000295
PDCD10	1,159434	1,53E-05
PFKL	1,15726	8,33E-05
AARS	1,15686	0,000304
FBRS	1,155899	1,99E-07
AURKA	1,155172	6,24E-06
CMC2	1,154774	7,00E-05
ZNF579	1,154646	7,45E-05
AFF4	1,152277	2,08E-07
ZNF581	1,15015	0,000362
G6PC3	1,147612	0,000449
ACTR10	1,147118	0,000169
FBXO22	1,146995	4,36E-05
SNAPC4	1,146659	0,000191
H2AFJ	1,146381	0,000952
TMEM11	1,141594	3,40E-05
GCLC	1,141205	0,000738
LAMTOR1	1,140008	0,00011
UBAC1	1,137921	5,36E-06
RNF121	1,135981	0,000373
TRIAP1	1,135759	0,000152
HSPA9	1,130322	6,01E-05
NFS1	1,127315	0,00012
STX4	1,126197	0,000367
PACS2	1,125746	1,13E-05
HSD17B4	1,125408	5,84E-05
COQ9	1,124743	1,86E-05
MBTPS2	1,122862	0,000725

C19orf43	1,12022	7,13E-07
VIMP	1,11941	3,73E-05
SLC2A4RG	1,119046	9,68E-05
RAD23B	1,118905	2,61E-07
HCFC1R1	1,117688	6,86E-05
FAM189B	1,117026	0,000331
PEX10	1,116724	0,000197
TCF25	1,11667	1,20E-06
SNRPE	1,116114	5,04E-07
PSMD12	1,111306	0,000149
TMEM254	1,110665	0,000547
PDCD6	1,10997	0,000285
ZNF704	1,108194	0,000877
C8orf82	1,107691	0,000317
DOLK	1,107643	0,000787
RPS3	1,107008	0,000236
JTB	1,106735	3,88E-05
KIAA0196	1,105396	7,82E-06
XXYLT1	1,103102	0,000105
PTGES2	1,102912	1,48E-05
DLG1	1,102862	5,95E-06
UNG	1,102201	1,21E-05
MTFR1	1,100843	0,000285
ARRDC1	1,099566	7,59E-05
LSG1	1,098925	1,66E-05
NPTN	1,09845	1,57E-05
CREG1	1,097839	0,000172
FAM53C	1,097406	0,000274
PDCL3	1,097194	0,000133
MRPS33	1,096253	0,000215
DNPEP	1,095833	1,54E-05
MTCH2	1,094134	0,000777
IFT46	1,092713	0,00024
LOC90784	1,092292	8,55E-05
FTSJ3	1,091307	8,61E-05
NDUFC1	1,09124	0,000315
ZFP1	1,090374	0,000275
THAP5	1,090363	3,15E-05
NDFIP1	1,089395	5,55E-06
SLC39A7	1,087026	3,21E-05
RARS	1,086777	5,47E-05
TUBA1C	1,079577	6,90E-05
MIDN	1,07871	1,62E-05
DERL1	1,078052	0,000122
RPL35A	1,076884	0,000528
RPL8	1,073928	0,000206
OSER1	1,073688	0,000307
SEL1L	1,071435	2,04E-06
CTPS2	1,071283	0,000111
PSMA3	1,070815	0,000444

TTC39C	1,070743	0,000545
SIKE1	1,069727	0,00026
ABCF3	1,067204	0,000234
RPL13	1,067007	0,000827
CSNK1G3	1,066438	7,05E-05
PMVK	1,066425	0,000845
PHAX	1,065473	1,62E-05
PLA2G12A	1,064445	0,000784
C7orf73	1,063209	6,54E-06
RPS14	1,06319	0,00039
SPTSSA	1,061993	0,000746
WIZ	1,060987	7,96E-06
HSPA4	1,059691	0,00054
C5orf15	1,059648	8,93E-05
COG4	1,056658	0,000201
CSRP1	1,053601	0,000415
KRTCAP2	1,051487	6,37E-06
RAB6A	1,051366	0,000412
TANC1	1,04921	0,000358
CHMP1B	1,047975	0,000441
PHF23	1,047736	0,000168
ABLIM1	1,045261	0,000376
OS9	1,044553	0,000161
DHRS7	1,042501	0,000221
TRIP4	1,041895	0,000555
MMAB	1,041363	0,00031
GPX4	1,039367	0,00027
SEC62	1,037971	1,07E-05
MRPS18B	1,036812	0,0002
PI4KB	1,036047	4,73E-05
RNF139	1,034668	2,89E-05
LAMP1	1,034586	0,000203
MRPL30	1,033435	2,33E-05
CENPN	1,033065	7,51E-06
ZNF12	1,032992	0,000166
LAPTM4A	1,03294	3,63E-05
CCDC9	1,030289	0,000562
EXOSC4	1,029427	0,000125
SPNS1	1,029143	0,000634
PPP1R11	1,028427	0,000249
RNASEH2A	1,027811	7,00E-05
TRIM26	1,027294	0,000168
NELFCD	1,024806	2,27E-05
DNMT3B	1,023597	0,000285
WBSCR22	1,023255	6,60E-05
DGKZ	1,022904	0,000222
PLK1	1,022524	0,000895
TRIM41	1,020452	0,00097
HMBS	1,019738	0,00022
SFXN1	1,019198	9,57E-05

MTIF2	1,018488	3,81E-05
CNIH1	1,017004	0,000202
VAPB	1,016898	0,000187
CDKN3	1,01596	0,000869
TMEM97	1,014167	7,88E-05
NFKBIA	1,012941	0,001009
GTPBP10	1,012293	0,000878
RRAGA	1,011708	0,000155
PWP1	1,010858	3,83E-05
PKP4	1,010686	8,20E-05
SPATS2	1,006385	6,95E-05
RRP1	1,006126	0,000818
PTPRF	1,00446	4,13E-05
COX6A1	1,00359	0,000158
PPCS	1,003367	0,00062
PAQR4	1,003357	0,000418
TPT1	1,002877	0,000895
TMEM14C	1,001926	0,000385
TOMM40	1,000426	0,000364
PEMT	0,999944	0,001011
UBQLN4	0,999817	2,43E-05
MRRF	0,999305	0,000138
PSMC1	0,998942	1,99E-05
ATP5A1	0,997696	6,04E-06
HEXB	0,9969	0,000476
NFE2L2	0,99552	0,000182
JAGN1	0,994405	0,000742
LAMTOR2	0,990314	0,000111
BTBD7	0,988971	0,000229
BRWD1	0,987982	0,000296
EEF2K	0,987181	0,000146
ASPSCR1	0,983986	0,000229
LMTK2	0,982302	0,000282
AHSA1	0,981712	2,41E-05
IRAK1	0,979893	0,000456
DCTPP1	0,97876	0,00051
RPRD2	0,978063	8,55E-05
ATXN7L3B	0,974713	7,76E-06
WDFY3	0,974356	0,000808
ZMYND11	0,972873	5,36E-05
HACD3	0,970839	1,65E-05
MAP1LC3B	0,969761	0,000306
RBM4	0,968436	0,00099
PPP1R14B	0,964982	0,000838
ANKS1A	0,9618	0,000282
TADA3	0,961406	0,000408
NUCKS1	0,959986	2,19E-05
PDCD5	0,959294	9,68E-05
TARBP2	0,959255	0,000349
SPTLC2	0,958334	0,000346

RRP15	0,958156	0,000174
PRR13	0,957661	0,000275
NDUFB7	0,954075	7,30E-05
TMEM147	0,951862	0,000623
KDM2A	0,95039	6,08E-05
STK38	0,948555	0,000489
ARNT	0,946852	7,29E-05
HDGF	0,946137	5,78E-05
MRPL15	0,944922	9,20E-05
DCAF13	0,94079	0,000772
PSMC5	0,939484	0,000427
PUF60	0,936573	0,000476
RCN1	0,933638	9,09E-05
PPP1R2	0,932841	0,000667
TIMM10	0,930636	0,000192
TSPAN14	0,930211	0,000762
TMED10	0,925435	0,00038
FAM89B	0,924501	0,000911
CAMSAP1	0,920566	5,84E-05
SCD	0,920376	4,76E-05
USP6NL	0,920231	0,000565
NSUN5	0,920169	0,000461
TUFM	0,918821	0,000135
STX6	0,916381	0,000173
MRPS23	0,914407	0,000457
LRFN4	0,911534	0,000425
ZNF652	0,911041	0,000493
RPE	0,908421	0,000686
CDC25B	0,907446	0,000564
EIF2AK1	0,90524	4,17E-05
GRK6	0,901121	0,000695
BRK1	0,899139	0,000399
TIMMDC1	0,895754	0,00068
NCBP2	0,893035	3,96E-05
KPNA2	0,880941	0,000311
UBE2Z	0,879879	0,000124
TRIP11	0,874977	0,00092
MTMR4	0,872133	0,000214
DSG2	0,869831	0,000232
NOTCH2	0,866571	0,000769
RNF7	0,855324	0,000859
VPS25	0,852239	0,000673
CCT2	0,851754	0,000331
CUL1	0,849301	0,000667
SOS1	0,845649	0,000758
MED13	0,836427	0,000107
FAM91A1	0,834823	0,000421
PAPOLA	0,834178	0,000154
VPS4B	0,830039	0,000983
VPS52	0,828681	0,000466

SMG7	0,826364	0,000244
YIPF3	0,825346	0,000765
TCOF1	0,818454	0,000503
CLUH	0,818211	0,000131
PDIA4	0,817047	8,26E-05
TAX1BP1	0,816346	0,000439
HECTD1	0,816009	0,000321
TAF6	0,815987	0,0009
MLF2	0,813776	0,000347
PFDN5	0,812407	0,00095
GEMIN5	0,811586	0,000632
PICALM	0,8094	0,000793
PPP4R3A	0,803962	0,000458
HMGN1	0,799889	0,00035
TOMM6	0,795889	0,000801
CACUL1	0,79556	0,000799
EIF2S1	0,793321	0,000852
TOR3A	0,792909	0,0009
ERAL1	0,791998	0,000765
DBI	0,784721	0,000359
CAND1	0,781615	0,000884
UBE2Q1	0,774477	0,000967
KIF5B	0,771237	0,000302
PROSER1	0,767053	0,000635
MORF4L2	0,751948	0,000839
H3F3B	0,723022	0,000734
ABCE1	-0,72912	0,000954
THRAP3	-0,73542	0,000751
ANP32A	-0,74525	0,000493
ACACA	-0,77334	0,001009
TTC3	-0,79272	0,000289
CHD7	-0,79408	0,000887
GNB1	-0,79783	0,000527
ELAVL1	-0,80059	0,001004
SEPT2	-0,80138	0,000461
RTF1	-0,80548	0,000444
HCFC1	-0,80995	0,000144
ITCH	-0,81631	0,000587
HMGCR	-0,81743	0,000624
STAG2	-0,82156	0,000129
CCT8	-0,82324	0,000431
MLLT1	-0,82582	0,000821
COPB1	-0,82585	0,000274
LRRFIP1	-0,83997	0,000221
APPL1	-0,84029	0,000582
SEP15	-0,84677	0,000355
WBP11	-0,84723	0,000228
NAP1L4	-0,84856	0,000913
FAAP100	-0,84856	0,000873
DIS3	-0,85513	0,000802

MLLT6	-0,85943	0,000233
MTG2	-0,85971	0,001024
PSMA1	-0,86449	0,000207
TOP2B	-0,86601	5,62E-05
STT3B	-0,86838	0,000979
QARS	-0,86985	0,000796
DCAF15	-0,87253	0,000447
KPNA6	-0,87354	0,000292
UGP2	-0,87452	0,000546
MKI67	-0,87521	0,000551
REXO1	-0,87537	0,000456
ADD1	-0,87548	0,000604
TCF20	-0,87678	0,00027
ZNF644	-0,87973	0,000456
SMAD2	-0,88497	0,000112
UCK2	-0,88533	0,000787
PCGF5	-0,89001	0,000442
CNOT1	-0,89153	0,000193
ARHGAP11	-0,89221	0,000529
NSMAF	-0,89379	0,000603
PDE12	-0,8943	0,00086
BRD7	-0,89645	0,000187
HNRNPUL2	-0,8974	0,000321
NCAPH	-0,90014	0,000126
NCOR1	-0,90122	6,59E-05
DHX30	-0,90405	3,30E-05
KIAA0430	-0,9043	0,000347
CDK12	-0,90433	0,000104
AP3M1	-0,90748	0,000119
AQR	-0,90964	0,000378
PPP6R1	-0,91046	0,000398
SFXN2	-0,91199	0,000743
ANKLE2	-0,91234	0,000406
MED1	-0,91249	4,51E-05
CENPO	-0,91518	0,000321
ST13	-0,91684	0,000238
TRAM2	-0,91685	0,000164
GRAMD1A	-0,91725	0,000122
AK2	-0,91843	0,000154
TGS1	-0,91895	0,000345
SF3A2	-0,91936	0,00103
ASXL1	-0,92108	4,53E-05
WHSC1	-0,92268	0,000148
ZZEF1	-0,92287	0,00019
PRKDC	-0,92454	0,000215
NECAP2	-0,92571	0,000712
CRLS1	-0,92724	0,000748
ATP6V1B2	-0,92731	0,000161
SUB1	-0,92973	0,000157
USP33	-0,93095	0,000455

RFC1	-0,93126	7,66E-05
AGL	-0,93325	0,000529
CHML	-0,93485	0,000758
COPS8	-0,93675	0,000762
SNRNP40	-0,937	0,000574
CPD	-0,94158	0,000413
ASPM	-0,94748	0,000686
PHF3	-0,94752	0,000442
ASXL2	-0,94867	0,0006
FTSJ1	-0,94992	0,000267
NUP93	-0,95042	0,000309
LANCL1	-0,95073	0,000124
ATXN10	-0,95089	0,000154
TRIM25	-0,95108	0,000267
SRRM1	-0,95135	0,000246
RAB27A	-0,95153	0,000476
API5	-0,95303	6,24E-05
POLA1	-0,95432	0,000555
RBX1	-0,955	0,000944
IPO5	-0,95696	0,000139
UBE2G2	-0,9579	3,18E-05
MCM10	-0,95924	6,11E-05
CASP2	-0,96177	2,25E-05
MSH2	-0,96202	2,96E-05
WDR82	-0,96289	2,60E-05
MAFK	-0,9629	0,000649
CSNK2A1	-0,96371	4,32E-05
KIF11	-0,96392	8,24E-06
NBAS	-0,9645	0,000695
BBX	-0,96451	0,000916
DNTTIP1	-0,96676	0,000846
ABCC1	-0,96914	3,84E-05
DENND2D	-0,97439	0,000158
SRP9	-0,97466	3,41E-05
BRD3	-0,97529	0,000248
DYNC1H1	-0,97529	0,000122
OSBP	-0,97614	0,000218
LAS1L	-0,97637	0,000623
SH3BP5L	-0,97786	0,000369
SON	-0,97788	2,45E-05
CASC4	-0,97927	0,000126
NIPBL	-0,98112	3,27E-05
KIF2C	-0,9814	0,000406
RBCK1	-0,98485	6,39E-06
BICD2	-0,98486	0,000116
RRM1	-0,98519	0,000206
ZNF367	-0,98671	0,000952
PHRF1	-0,98834	6,53E-06
KPNA3	-0,98884	0,000392
C2CD3	-0,98933	9,88E-05

ABI2	-0,99395	9,35E-06
CSTF2T	-0,99399	0,000173
GIT2	-0,99458	4,70E-05
C22orf39	-0,99488	0,000971
ATPIF1	-0,99798	5,25E-05
WDR24	-0,99834	0,000461
ALG9	-0,9987	0,000282
DDHD1	-0,99895	0,00018
PPP1R18	-1,00253	0,000459
DHFR	-1,00336	3,15E-05
BLM	-1,00336	0,00055
CDK11B	-1,00571	3,59E-05
SLC16A1	-1,00774	0,000191
CEP104	-1,00849	0,000368
LIN54	-1,00981	6,74E-05
L3MBTL2	-1,00985	0,000102
MTMR1	-1,0121	0,000102
RAB3GAP2	-1,01308	0,000572
SLBP	-1,01388	0,000106
FAM111B	-1,01523	0,000205
CHCHD2	-1,01679	3,64E-05
CCDC34	-1,01996	0,001017
CTR9	-1,02056	0,000921
DESI2	-1,02117	0,000105
PATL1	-1,02157	1,91E-05
EXOSC9	-1,02266	0,000209
SAAL1	-1,02745	0,000164
TRIM44	-1,02923	0,000141
PSME1	-1,02995	6,27E-05
C4orf27	-1,0307	0,000494
TTI1	-1,03295	2,45E-05
MRE11A	-1,0359	0,000997
SGOL2	-1,03689	5,84E-05
OSBPL8	-1,03741	7,86E-06
XAB2	-1,0375	0,000367
HMGN2	-1,03798	3,05E-05
COL4A3BP	-1,03888	0,000325
MCM5	-1,04039	0,000264
DCTN3	-1,04048	0,000833
HIATL1	-1,04145	0,000522
CHORDC1	-1,04219	0,000163
FRYL	-1,0437	5,55E-05
ZNF747	-1,04575	0,000514
ARID1A	-1,04875	1,19E-06
ALAD	-1,05014	0,000301
GBA2	-1,05204	0,000821
EIF2AK4	-1,05215	7,53E-05
MEN1	-1,05378	1,50E-05
ADSS	-1,05473	5,84E-05
UBA5	-1,05484	0,000266

CENPF	-1,05509	1,94E-05
ITM2B	-1,05542	3,12E-05
DDA1	-1,05748	7,29E-05
ERCC6L	-1,05804	0,000364
EP300	-1,0583	0,000107
VPS16	-1,05878	0,000357
MAP3K4	-1,0594	0,000386
ZMYM3	-1,06006	7,39E-06
UBQLN2	-1,06112	7,75E-05
ARL2BP	-1,06149	0,000765
PRPS1	-1,06216	0,000335
WNK1	-1,06294	2,07E-06
CHD1	-1,06345	3,87E-06
RALGAPA2	-1,06723	0,000359
IFNAR1	-1,06729	0,000447
CDC6	-1,06793	0,000158
ACAT2	-1,06801	5,58E-05
TRPC4AP	-1,06802	3,79E-05
VPS36	-1,06839	0,000102
PPIP5K2	-1,07028	8,51E-05
TPP1	-1,07086	0,000541
HERC1	-1,07182	1,91E-05
TACC1	-1,07412	1,23E-05
PARP1	-1,07606	9,94E-07
ERCC6L2	-1,07663	0,00011
KHNYN	-1,0767	0,00045
PNP	-1,07678	1,51E-05
POT1	-1,07885	0,000466
MGA	-1,08019	5,50E-05
HMGN3	-1,0805	4,93E-05
MKL1	-1,0807	6,56E-05
SEC22C	-1,08148	0,000164
LARS2	-1,08234	6,26E-05
SUN2	-1,08335	2,27E-05
CACTIN	-1,08401	0,000167
JARID2	-1,08505	2,92E-06
NF2	-1,08584	0,000107
DDX3X	-1,0864	1,73E-07
C22orf46	-1,08714	0,000203
RNF138	-1,08731	7,84E-05
KANSL1	-1,0874	0,000277
DIDO1	-1,08741	7,00E-07
CEPT1	-1,088	0,00012
ATG4B	-1,09051	3,29E-05
SMARCAD1	-1,09264	6,40E-05
MAPKAPK3	-1,09273	0,000897
SOAT1	-1,09296	0,000271
HSD17B12	-1,09321	8,15E-05
UBE3A	-1,09342	3,79E-06
MOB1A	-1,09368	7,05E-05

ALAS1	-1,0939	0,000362
FEN1	-1,09459	3,40E-06
AES	-1,09525	7,62E-06
ERO1A	-1,09726	3,28E-05
SREK1IP1	-1,09804	2,91E-05
SGSM3	-1,09912	0,000421
ZNF275	-1,09959	0,000251
WASF2	-1,10064	9,94E-06
OXSR1	-1,10241	2,68E-05
PJA2	-1,10325	2,18E-06
ZNF335	-1,10326	0,000271
TMED5	-1,10353	0,000124
MTA3	-1,10498	1,50E-06
MIER2	-1,10545	0,00061
INTS7	-1,106	2,89E-05
SLC25A40	-1,10718	0,000588
NCAPG	-1,10946	1,20E-05
MFAP1	-1,1106	0,000212
AKR1A1	-1,11204	2,29E-05
VRK1	-1,11566	1,15E-05
KDM2B	-1,11615	2,83E-06
OTUD5	-1,1162	0,000179
LRP12	-1,11698	0,000114
INPP5K	-1,11758	0,000646
ZSWIM1	-1,1201	0,000193
RHOT1	-1,12129	3,41E-05
DNMT3A	-1,12306	4,46E-06
PGK1	-1,12345	2,43E-06
LIMD1	-1,1253	0,00013
NONO	-1,12571	2,42E-06
BAG2	-1,12812	9,99E-05
RPAP1	-1,12837	8,66E-06
NDUFB8	-1,12999	0,000243
C1orf112	-1,13015	0,000294
ZNF37A	-1,13219	9,20E-06
ATF2	-1,13274	6,85E-06
FAM98B	-1,1338	6,95E-05
CD44	-1,13485	3,95E-05
TYW3	-1,13712	2,69E-05
TRIM35	-1,13718	0,000726
ZC3H7B	-1,13838	2,74E-06
EXO1	-1,13862	2,09E-05
ZNF343	-1,13888	0,000483
CTPS1	-1,13982	1,95E-06
HMGXB4	-1,13985	8,72E-06
RHOG	-1,14373	0,00015
HIRA	-1,1451	3,50E-07
ARIH2	-1,1459	7,72E-07
FPGS	-1,14606	0,000115
LRRC41	-1,14661	7,04E-06

ICE2	-1,14816	3,11E-06
VPRBP	-1,1484	1,88E-06
RAB11B	-1,14923	1,20E-05
TUBGCP3	-1,15088	2,29E-05
PHKA2	-1,15133	5,68E-05
C11orf57	-1,15166	2,86E-05
ASH2L	-1,15262	1,96E-05
ZNF740	-1,15359	0,000133
SF3A3	-1,15444	0,000486
TMEM237	-1,15612	6,39E-05
MAGOH	-1,15708	3,49E-06
POLDIP3	-1,15768	0,000104
MTMR2	-1,15808	4,68E-05
RPRD1B	-1,15811	3,67E-05
CDC40	-1,15962	0,000154
RNF38	-1,15981	0,000273
ASCC3	-1,16154	0,000992
MED4	-1,16168	5,69E-05
URB2	-1,16244	4,64E-05
ARID4B	-1,16274	0,000318
MBNL3	-1,1628	4,47E-07
FKBP1A	-1,16296	6,26E-06
OSBPL2	-1,16346	1,94E-05
USE1	-1,16353	0,000628
MFSD6	-1,16631	0,00047
NDUFAF3	-1,16811	9,21E-05
HTRA2	-1,16833	7,07E-05
VPS13D	-1,16859	8,81E-06
BNIP2	-1,16933	3,22E-05
UBR7	-1,17027	2,20E-07
JADE2	-1,17071	0,000168
HMGA1	-1,17251	0,000106
RRM2	-1,17539	6,99E-08
TAP2	-1,17544	0,000282
EBLN3	-1,17691	9,28E-06
NCAPD2	-1,17763	4,55E-06
RPTOR	-1,17779	5,23E-05
TRMT6	-1,17803	3,36E-06
NOP9	-1,17932	1,09E-05
FAM217B	-1,18035	1,40E-05
RAD51	-1,18189	6,74E-05
DENND1B	-1,18195	0,000658
DNMT1	-1,183	1,58E-05
CLEC16A	-1,18375	7,51E-06
RNF123	-1,18405	9,16E-06
INTS9	-1,18422	3,58E-05
EID2	-1,18431	0,000329
SNRNP48	-1,18712	8,68E-06
PLK4	-1,1873	6,42E-05
ORAI2	-1,18839	0,000337

EDEM3	-1,19249	5,36E-05
GSG2	-1,19405	0,000333
PTPN6	-1,19776	0,000162
ZCCHC9	-1,19827	0,000247
H2AFV	-1,19912	1,23E-06
FNTA	-1,19975	7,39E-05
AGBL5	-1,20137	0,000492
STAM	-1,20218	0,000138
GXYLT1	-1,20263	1,21E-05
SIVA1	-1,20498	4,03E-06
TMX4	-1,2064	1,12E-05
PRDM2	-1,20644	4,50E-05
PSMB2	-1,20755	1,25E-06
TOP2A	-1,2086	1,97E-05
PPWD1	-1,21	0,000783
PI4KA	-1,21089	2,50E-07
TTC31	-1,21152	0,000162
PIKFYVE	-1,21193	0,000611
FH	-1,21223	2,18E-07
SMYD4	-1,21366	0,000388
TNRC6C	-1,21483	9,78E-08
CHAF1B	-1,21501	0,00015
PLAGL2	-1,21502	1,32E-06
CTDP1	-1,21612	0,000216
MID1IP1	-1,21647	0,000935
LRRCC1	-1,21943	0,000334
NVL	-1,2198	1,70E-05
CHEK1	-1,22162	2,74E-07
MDN1	-1,22188	2,24E-06
TMEM230	-1,22351	8,72E-07
SMAD4	-1,22374	9,79E-06
GPKOW	-1,2238	0,000228
MIA3	-1,22391	3,63E-07
LMF2	-1,22409	5,10E-05
RNF219	-1,22665	3,85E-05
CPSF1	-1,22858	3,42E-05
CALML4	-1,22895	0,000633
SRSF11	-1,23047	4,92E-05
DCAF12	-1,23071	2,48E-07
C9orf78	-1,23089	6,32E-07
LDHA	-1,23126	2,09E-06
PHF2	-1,23233	0,000233
HEATR5B	-1,23361	5,18E-05
DONSON	-1,23938	0,000235
ST6GALNA(	-1,23949	0,000747
HS6ST1	-1,24182	1,85E-06
KIAA1147	-1,24277	2,74E-05
GLI3	-1,24318	0,000459
PREP	-1,24325	8,96E-05
SFI1	-1,24573	0,001029

GTF2B	-1,24869	0,000429
TFDP1	-1,24882	8,37E-09
SLC20A1	-1,24956	1,13E-08
ASB1	-1,25	2,43E-05
CBL	-1,25098	5,19E-06
TERF2	-1,25237	5,72E-06
PCIF1	-1,25347	1,08E-06
MPRIP	-1,25593	9,94E-07
FAM122A	-1,25772	0,000212
CEP78	-1,25936	3,00E-06
ATG2B	-1,26081	4,35E-05
ERI1	-1,26112	4,99E-06
SCAF4	-1,26288	0,000207
AP1M1	-1,26473	0,000145
ORMDL3	-1,26501	0,000193
NLK	-1,26616	4,72E-06
MED8	-1,26679	4,96E-05
VPS37B	-1,26973	4,03E-06
BAP1	-1,26999	3,37E-08
TAF1	-1,27181	9,22E-05
UFL1	-1,27273	3,46E-05
TRAF3IP2	-1,27286	0,000153
FANCD2	-1,27471	6,01E-06
GMPPB	-1,27629	3,09E-05
IPO5P1	-1,27665	0,000243
ZBED4	-1,27763	2,72E-05
NR2C2	-1,27789	1,37E-06
PDE6D	-1,28262	0,000246
C2orf44	-1,28297	0,000184
STAT3	-1,28319	0,000572
C12orf4	-1,28339	0,000206
USP16	-1,28357	2,58E-07
RBM26	-1,28373	1,98E-06
TBC1D20	-1,28396	7,63E-06
CEP295	-1,28531	0,000868
WDR54	-1,28544	8,03E-06
CAMK2D	-1,28615	0,000655
RTCA	-1,28736	2,13E-06
MCM6	-1,29053	6,86E-08
PRNP	-1,29057	8,59E-05
TAF5L	-1,29075	9,49E-07
RNF213	-1,29268	0,000221
SMIM10L1	-1,29823	6,25E-07
TAF5	-1,29826	0,000228
FBXW7	-1,29866	1,19E-05
TMEM201	-1,29905	5,49E-05
BTAF1	-1,30128	3,17E-05
ITSN2	-1,30381	1,62E-05
DOT1L	-1,30572	5,96E-08
BIN3	-1,30725	0,000216

ARHGAP19	-1,30784	2,09E-05
CEP97	-1,30935	0,000187
DSN1	-1,31052	2,55E-06
GNPTAB	-1,31075	1,38E-08
NOTCH1	-1,31674	9,42E-10
PTPN3	-1,31682	6,15E-07
TUBGCP5	-1,31763	0,000269
PHF6	-1,32033	8,18E-06
CSGALNAC	-1,32086	0,000487
TAZ	-1,32226	0,000647
TAF9B	-1,32236	0,000712
SUZ12	-1,3224	2,04E-09
TET1	-1,3254	0,000164
FTO	-1,32665	5,57E-07
TGFBR1	-1,32685	1,05E-05
POLD1	-1,33006	2,14E-09
HDAC1	-1,33033	8,85E-10
IGF2R	-1,3318	2,84E-07
TMEM123	-1,33327	2,11E-10
DBNL	-1,33356	8,31E-05
NCAPH2	-1,3341	1,05E-08
SEPT7	-1,33479	1,84E-09
PAFAH2	-1,33631	7,69E-05
ARRB2	-1,33677	4,43E-08
STK26	-1,33774	2,71E-08
HDHD1	-1,3392	0,00046
KLHL7	-1,34163	0,000114
PCMTD2	-1,34424	0,000315
MAP3K7	-1,34687	6,51E-08
ZAK	-1,34795	4,44E-07
JADE3	-1,34935	3,56E-06
HNRNPD	-1,34943	5,82E-10
DCAF11	-1,34949	2,40E-05
SUCLA2	-1,35197	8,40E-07
ERCC1	-1,35506	6,38E-08
TBRG1	-1,35514	6,43E-05
KRI1	-1,35571	7,08E-10
LINC00938	-1,35719	0,000303
ARHGEF1	-1,35953	9,76E-10
RBBP4	-1,36079	9,39E-11
POMGNT2	-1,36093	0,000396
CHAMP1	-1,36269	9,20E-06
CDK2	-1,3641	4,75E-08
TOPBP1	-1,36472	1,07E-07
CNST	-1,36528	0,000758
CWC25	-1,36788	6,54E-05
SPTAN1	-1,36839	4,99E-07
FAM49B	-1,37053	1,06E-09
NSL1	-1,37129	5,06E-07
HIPK2	-1,37192	0,000406

PYCR2	-1,37436	5,28E-05
USB1	-1,37529	0,000199
FBXO33	-1,37582	0,000813
SUV39H1	-1,37635	1,78E-05
TAPT1	-1,37656	5,40E-06
LGALS8	-1,37697	8,67E-06
TCP11L1	-1,37802	0,000428
GNPAT	-1,38321	1,33E-06
USP24	-1,38334	2,03E-08
ZFAT	-1,38434	1,54E-05
TRAM1	-1,38498	1,97E-05
PBX3	-1,38522	2,06E-05
CCNE2	-1,38675	1,03E-06
TLE1	-1,38698	1,34E-06
RRP7A	-1,38727	4,03E-08
LRRC20	-1,39202	0,000975
SPEN	-1,39239	7,95E-07
REV3L	-1,39295	1,52E-06
LYRM7	-1,39305	2,09E-06
UTP14C	-1,39441	3,17E-05
NISCH	-1,39884	1,92E-06
SDCCAG8	-1,3995	0,000223
PARP11	-1,40089	0,000524
TYK2	-1,40204	2,87E-05
AEBP2	-1,40513	1,64E-05
FNDC3A	-1,4066	8,53E-07
ADARB1	-1,40661	1,85E-06
SYNRG	-1,4085	4,99E-06
ZKSCAN2	-1,41168	1,42E-05
RPA1	-1,41184	4,97E-10
WEE1	-1,41256	0,000533
GINS3	-1,41299	0,000402
FAM126B	-1,41486	0,000228
SLFN5	-1,4161	2,28E-05
UBR1	-1,41625	0,000622
SSBP3	-1,41759	4,13E-08
TTK	-1,41798	3,57E-08
ZNF92	-1,41917	3,28E-05
VPS26B	-1,41926	2,58E-06
GABPA	-1,41988	1,44E-05
TBC1D2B	-1,4209	1,24E-07
DNAJC24	-1,42093	0,000961
NNT	-1,4217	1,18E-07
CTBP1-AS2	-1,42308	2,43E-07
FIGNL1	-1,42503	2,73E-06
HDAC7	-1,42829	4,56E-10
RBL2	-1,42887	1,52E-06
CARNMT1	-1,43001	0,000193
AMIGO2	-1,43031	0,000397
NUF2	-1,43192	0,000223

ADCY7	-1,43212	3,60E-05
USP1	-1,43217	6,12E-07
JAK1	-1,43286	2,46E-07
LRP8	-1,43452	3,33E-05
BLOC1S6	-1,43513	5,96E-08
CAB39	-1,43612	6,49E-10
HIAT1	-1,43842	2,07E-08
RELT	-1,4387	0,000432
STK40	-1,43973	1,78E-05
IKBKB	-1,44212	0,000183
CD47	-1,44281	1,08E-09
SCML2	-1,44292	0,000307
ADRBK1	-1,44664	7,57E-09
C4orf46	-1,44674	1,31E-05
FAM117B	-1,44693	2,81E-08
PASK	-1,4485	1,35E-05
ZNF512B	-1,45415	2,05E-06
SDR39U1	-1,4556	5,12E-06
FAM195B	-1,45704	0,000273
RPIA	-1,45761	1,43E-08
SLX4	-1,46259	5,21E-07
DCP1A	-1,46265	2,48E-05
E2F8	-1,46666	0,000614
INSR	-1,46809	0,000654
YEATS2	-1,46821	0,000395
GOPC	-1,46843	1,68E-07
SKP2	-1,46965	3,50E-11
UBE2J1	-1,46971	2,79E-09
TBC1D10A	-1,47446	2,23E-06
AIP	-1,47471	1,67E-07
DSCR3	-1,47738	2,99E-07
PPP2R1B	-1,47832	7,43E-06
RNGTT	-1,48013	2,23E-08
GBAS	-1,48039	0,000933
ARID5A	-1,48084	0,00016
CXorf38	-1,48085	1,20E-05
TMPO-AS1	-1,48105	2,14E-05
NEK7	-1,48243	2,08E-07
MMS22L	-1,48374	1,51E-05
DNA2	-1,48576	2,57E-05
KAT6B	-1,48706	3,99E-10
XPNPEP1	-1,48715	1,85E-07
PHIP	-1,48794	5,11E-10
MYO18A	-1,48931	1,59E-05
PRPF38A	-1,48946	1,94E-11
WDR7	-1,49172	5,89E-07
PFAS	-1,49478	1,03E-09
DHRS4L2	-1,49487	2,83E-06
ACOT7	-1,49494	1,88E-06
SPC24	-1,49554	7,95E-07

CARHSP1	-1,49567	8,57E-11
IRAK1BP1	-1,49624	0,000946
PRAF2	-1,4968	0,00021
INPP4A	-1,49728	6,09E-09
CDADC1	-1,50007	0,000909
SNAP23	-1,50105	2,41E-06
NASP	-1,50432	6,38E-12
KIFAP3	-1,50599	1,71E-05
HSF2	-1,50787	2,40E-06
MED12	-1,50859	4,33E-09
POLE	-1,51131	6,36E-10
LNPEP	-1,51289	2,16E-06
AAK1	-1,51367	2,39E-06
PHF8	-1,51676	2,04E-08
PROSC	-1,51739	1,18E-08
APIP	-1,51762	1,37E-08
PHF11	-1,51812	6,59E-05
DMAP1	-1,51925	7,94E-05
NUDT21	-1,51988	6,69E-12
NUP50	-1,52001	1,91E-10
ZGPAT	-1,5321	1,73E-07
XRRA1	-1,53276	1,57E-05
CMIP	-1,53289	4,01E-09
XKR8	-1,53354	0,000983
SESTD1	-1,53366	6,04E-06
ZBTB44	-1,53432	2,92E-07
PCNXL2	-1,53445	5,84E-06
KDM5A	-1,53733	3,57E-06
ELOVL5	-1,54034	1,66E-13
GK5	-1,5405	1,72E-07
CTNNBL1	-1,54072	9,62E-10
TMEM164	-1,54154	1,15E-09
TCFL5	-1,54198	5,85E-06
DEDD2	-1,54216	2,09E-06
ZBED6CL	-1,54461	0,00041
CKAP2L	-1,54618	3,81E-05
LPGAT1	-1,54652	3,40E-09
DR1	-1,54769	9,95E-11
MED14	-1,54822	1,02E-10
C21orf91	-1,55051	2,81E-06
RINL	-1,55052	0,000359
PANK2	-1,55091	4,99E-08
EPS15	-1,55105	2,09E-09
PXMP2	-1,55473	1,27E-05
PSMB10	-1,55768	7,24E-06
KIAA1551	-1,55786	2,98E-11
DUT	-1,55841	2,03E-10
HSPA13	-1,56129	1,13E-09
MBOAT1	-1,5615	0,000396
OSGEP	-1,57036	9,36E-07

LRRC40	-1,57047	8,34E-08
HP1BP3	-1,57073	1,05E-05
EYA3	-1,57081	3,09E-09
SMC2	-1,57279	2,64E-09
CFAP20	-1,57339	1,39E-06
SRSF4	-1,57446	6,44E-10
ARFRP1	-1,57465	9,83E-11
IFFO2	-1,57515	6,39E-05
SLC35E2B	-1,57655	1,47E-11
ATM	-1,57675	0,000451
PITPNM1	-1,57738	2,36E-06
FCHSD2	-1,57938	8,18E-07
TRPM2	-1,57961	1,13E-06
ASF1B	-1,5816	5,27E-10
RNF144A	-1,58262	3,75E-06
CRYBB2P1	-1,58326	7,64E-05
MAD2L2	-1,58468	2,81E-05
BUD13	-1,58561	1,46E-06
ATAD3B	-1,58687	0,000455
PSMD5-AS:	-1,587	6,59E-06
H6PD	-1,58831	4,71E-07
S100PBP	-1,59068	4,14E-06
CENPP	-1,59074	1,20E-05
BLMH	-1,59645	1,21E-09
KIAA0754	-1,59648	4,93E-05
DGKD	-1,59714	8,87E-07
FGFR1	-1,59947	4,80E-11
NUP133	-1,59998	4,21E-12
CPNE1	-1,60159	2,02E-12
AGTPBP1	-1,60331	2,86E-08
SNX29	-1,60372	0,000266
SNRK	-1,60436	3,80E-09
MYO9B	-1,60495	1,24E-07
ZNF711	-1,60573	0,000119
EVL	-1,60589	3,14E-10
ZNF292	-1,6065	2,06E-09
TOE1	-1,60733	2,87E-05
LIMK2	-1,60962	3,17E-07
ARID1B	-1,61049	2,16E-10
TWF2	-1,61081	1,02E-08
ARFGEF1	-1,61176	1,32E-10
HACD1	-1,61384	0,000272
STXBP5	-1,61416	9,29E-08
FAM120C	-1,6146	8,72E-06
GID4	-1,61811	3,78E-05
CARS2	-1,61846	2,15E-09
ZNF549	-1,62079	0,000689
CCNDBP1	-1,62106	9,38E-07
TMEM110	-1,6219	3,19E-05
C21orf2	-1,62391	2,26E-07

PIP4K2B	-1,62527	3,64E-12
CENPC	-1,6293	2,37E-05
TINF2	-1,63042	3,40E-10
TARBP1	-1,63082	3,97E-05
PDE7A	-1,63504	3,11E-08
SDE2	-1,63617	0,000164
TRNAU1AP	-1,63712	2,50E-06
GNAI2	-1,63716	1,64E-06
CERK	-1,63768	2,15E-05
DIAPH3	-1,63895	4,37E-05
TMEM206	-1,64326	0,000291
NUP43	-1,64857	2,41E-08
PTPRA	-1,6488	1,68E-09
DYRK3	-1,65013	0,000108
PGBD5	-1,65425	0,000491
SHCBP1	-1,6547	1,76E-11
ETHE1	-1,65575	5,50E-05
IRF2	-1,65769	2,80E-08
GTF3C5	-1,65971	4,53E-14
SPRED2	-1,6607	3,60E-12
ESCO2	-1,66125	6,78E-09
SREBF2	-1,66213	3,47E-13
NGLY1	-1,6625	1,31E-09
ZNF462	-1,66436	1,31E-06
NDST2	-1,66538	2,53E-08
MED30	-1,66543	2,32E-05
TRAF3	-1,66782	1,49E-11
TGFB1	-1,66797	3,84E-07
CHFR	-1,66851	1,29E-09
ZNF101	-1,66967	4,09E-08
SLC23A2	-1,66967	9,98E-09
MAP2K6	-1,67006	1,60E-06
SLC36A4	-1,67057	0,000119
SNX25	-1,67077	2,32E-05
N4BP2	-1,67331	1,69E-09
ETV6	-1,67726	2,82E-08
GTPBP1	-1,67729	1,37E-09
UPRT	-1,67784	0,000844
MOB1B	-1,67811	1,09E-06
VPS39	-1,67905	5,52E-10
IL17RA	-1,67911	1,05E-10
MSI2	-1,68096	2,77E-09
KAT6A	-1,68428	1,66E-11
ARHGEF7	-1,6884	1,90E-09
FAM171A1	-1,68921	5,65E-05
FKBP5	-1,69456	1,10E-10
PTAR1	-1,69465	1,20E-07
SPOP	-1,70112	7,61E-09
RUFY3	-1,70112	5,80E-09
TMEM55A	-1,70299	2,87E-05

PSD4	-1,70723	5,36E-10
USP4	-1,7098	1,51E-12
ELF1	-1,71044	1,48E-14
ZNF70	-1,7107	0,000781
LINC00294	-1,71091	0,000984
TXLNA	-1,71276	1,35E-08
CBFB	-1,71282	1,39E-11
STK17A	-1,71485	3,49E-09
POLM	-1,71505	4,41E-06
KIAA1143	-1,71633	4,09E-09
KDM5C	-1,71637	5,49E-16
NFATC3	-1,71644	1,63E-11
METTL17	-1,71785	3,57E-05
SYNJ2	-1,71891	3,50E-10
PPM1F	-1,71914	1,75E-05
TBC1D1	-1,72047	9,29E-08
SGTB	-1,72354	3,79E-05
DTL	-1,72358	4,42E-13
SCAPER	-1,72367	6,72E-05
CRY1	-1,72403	3,73E-10
ZNF827	-1,72404	0,000122
CNTLN	-1,72807	0,000168
PCNXL4	-1,72922	3,17E-13
CRYZL1	-1,72956	6,64E-06
CCBL1	-1,73069	0,00044
EFHD2	-1,73148	1,05E-08
SCAI	-1,73197	5,58E-07
TLN1	-1,73272	3,23E-09
ARRDC1-AS	-1,73428	8,21E-06
HMGCS1	-1,73487	1,01E-08
SCD5	-1,73488	1,23E-09
RCBTB1	-1,73715	9,00E-07
BDH2	-1,73756	9,27E-05
EPM2A	-1,73892	0,000127
AKAP17A	-1,74251	1,67E-05
ATP2B4	-1,74336	5,69E-06
PIN1	-1,74541	4,11E-12
RAD51AP1	-1,74625	0,000379
TCEANC2	-1,74801	3,06E-06
FBXO5	-1,74853	3,75E-06
MIR4442	-1,74923	0,000998
GNAQ	-1,75047	8,28E-16
KIF24	-1,75772	7,86E-06
PHC1	-1,75986	3,97E-10
LDAH	-1,76145	3,10E-10
KDELC1	-1,76167	1,20E-05
AGAP2	-1,7653	1,16E-09
NEU3	-1,76603	4,62E-09
COPS7B	-1,76697	1,74E-11
ATP9B	-1,7672	3,89E-06

RAD51D	-1,76775	0,000174
HAUS5	-1,76803	2,40E-06
RNF170	-1,77203	1,07E-05
SLC35A3	-1,77262	1,45E-09
PLEKHG4	-1,77322	7,17E-05
FDFT1	-1,77552	1,89E-10
ARL6IP6	-1,77581	1,44E-05
CTDSPL2	-1,77672	5,41E-10
IL4R	-1,77733	0,000768
RNF145	-1,77798	1,57E-05
ZNF589	-1,77876	0,000542
INAFM2	-1,77996	0,000985
ZNF792	-1,77998	0,000474
ORC1	-1,78265	8,63E-10
FBXO41	-1,78273	5,88E-08
PBRM1	-1,78487	1,20E-16
LIG4	-1,78852	3,19E-09
LINC00909	-1,78873	0,000839
DOPEY1	-1,78929	8,66E-06
THYN1	-1,79044	2,01E-10
CEP57	-1,79111	6,52E-09
CEP170	-1,79451	2,62E-13
STARD4	-1,79487	8,22E-06
KDELC2	-1,79488	1,82E-10
PHF19	-1,79501	1,16E-13
PPP1R3E	-1,79592	0,000328
KLF13	-1,79751	3,26E-13
RAD54L2	-1,79766	6,09E-07
C9orf40	-1,79946	5,48E-09
MANEA	-1,80153	4,82E-06
CLSPN	-1,80425	6,66E-16
SPTBN1	-1,81	4,13E-16
PEX6	-1,81026	0,000831
VAMP2	-1,81039	1,53E-09
RBL1	-1,81122	3,33E-08
STAU2	-1,8141	1,60E-14
SSFA2	-1,81484	1,78E-11
AFF1	-1,81535	7,80E-11
KLHL15	-1,81707	3,60E-05
TTF1	-1,81781	6,40E-13
ANKRD49	-1,81853	3,65E-05
LOC648987	-1,81996	2,63E-05
MTR	-1,82195	1,35E-07
SCP2	-1,82373	2,52E-07
LRRC14	-1,82602	1,32E-09
FAT1	-1,82664	4,91E-09
RAPGEF1	-1,83175	2,48E-10
SACM1L	-1,83319	1,16E-09
CCDC28A	-1,83361	5,62E-05
GPR180	-1,83568	4,49E-08

CEP152	-1,8384	4,93E-05
SHQ1	-1,84056	5,13E-11
PLEKHG2	-1,84276	2,24E-13
MAP7D1	-1,84316	1,11E-10
RICTOR	-1,84539	2,09E-06
CHAF1A	-1,84692	1,53E-13
FES	-1,84793	0,000796
BNIP3L	-1,8486	2,15E-07
LOC72874З	-1,84887	0,000349
ZGRF1	-1,85041	0,000481
RRN3P3	-1,85447	0,000958
PDDC1	-1,85573	3,01E-08
PRPS2	-1,85828	2,90E-10
DHX35	-1,86122	1,57E-06
EPB41	-1,86199	9,21E-13
CDK19	-1,86327	1,49E-11
XYLT1	-1,86461	2,95E-08
ZNF486	-1,8647	2,93E-07
ZSCAN16-A	-1,86727	5,07E-05
ARHGEF18	-1,86763	3,50E-13
SLC30A7	-1,8681	3,82E-06
TPP2	-1,87137	6,32E-14
MAP4K2	-1,87331	5,42E-09
TEX30	-1,87721	4,58E-07
SBF1	-1,87768	2,10E-13
C18orf54	-1,8783	7,25E-08
ENO2	-1,87837	2,75E-07
CASC5	-1,88048	7,51E-16
HIC2	-1,88779	2,12E-05
B4GALT5	-1,88798	3,24E-15
CLDND1	-1,88853	2,40E-11
CYTH1	-1,88912	2,01E-10
LEF1	-1,8962	1,92E-10
PDXP	-1,89696	1,95E-10
ZBED1	-1,90243	3,99E-10
MMD	-1,90555	0,000646
BUB1B	-1,90805	2,90E-08
HMGB2	-1,90821	6,47E-08
TMEM135	-1,91293	1,06E-08
NIN	-1,91682	1,32E-15
BCKDHB	-1,91727	3,67E-07
ARGLU1	-1,91807	0,00029
TBC1D4	-1,91825	2,46E-05
RANGRF	-1,91875	8,64E-09
PM20D2	-1,91939	1,04E-07
TCF19	-1,9194	4,28E-16
HDAC6	-1,92	1,08E-10
RAB8B	-1,92098	7,56E-07
GLS	-1,92421	2,60E-07
SNTA1	-1,92479	1,30E-07

PCNA	-1,92534	1,32E-21
ACSL4	-1,92701	2,26E-10
SENP7	-1,93154	0,000215
GTSE1	-1,93163	2,30E-14
CPOX	-1,9325	5,96E-06
SERGEF	-1,93498	1,51E-06
SYNGR1	-1,93556	3,25E-05
OCIAD2	-1,93633	9,92E-11
EPHX2	-1,93691	2,25E-07
SMC5	-1,93879	6,80E-14
ASIC1	-1,94303	7,88E-05
FAM169A	-1,94533	0,000277
BACH1	-1,946	2,02E-09
ME2	-1,95335	9,77E-15
MACF1	-1,95336	9,62E-21
SLC25A12	-1,95583	1,59E-09
HLA-C	-1,95914	0,000451
PCBP3	-1,95935	3,16E-06
KCNC4	-1,96066	0,000104
MYH10	-1,96304	1,32E-14
XRN1	-1,96422	1,62E-07
IFNAR2	-1,96463	4,79E-09
ANKRD10	-1,96531	1,37E-07
HOMER1	-1,96886	2,48E-12
MIER1	-1,97182	5,42E-16
LINC00641	-1,97339	0,000492
HBS1L	-1,97525	8,79E-19
NUPL2	-1,97582	0,00053
ABCB7	-1,97644	1,73E-10
FAM160B1	-1,97661	1,20E-07
ITPRIP	-1,97845	0,000254
ACOT8	-1,97879	2,59E-07
LOC730101	-1,97893	5,13E-08
FRG1	-1,98428	4,85E-10
FBXO4	-1,98488	8,44E-05
CEP135	-1,98504	1,19E-05
LOC100996	-1,98674	2,51E-05
LZTFL1	-1,98698	3,86E-05
KPNA5	-1,99586	0,000203
SLC27A1	-1,99607	0,000103
IRF1	-1,99808	6,48E-08
FABP5	-1,99808	1,91E-18
CHCHD7	-2,00163	1,73E-08
BTN2A1	-2,00184	0,000247
HBEGF	-2,00271	0,000265
ZNF141	-2,00297	1,13E-05
INPP5B	-2,00469	2,23E-08
SMYD3	-2,00517	5,66E-07
ZNF230	-2,00568	0,000345
DDX17	-2,00581	2,15E-09

FBXL12	-2,00621	4,18E-09
MOB3A	-2,00622	2,20E-13
GLCCI1	-2,00764	2,21E-10
GGT7	-2,00768	3,43E-05
CEP128	-2,0086	1,32E-13
CEP85	-2,01069	6,71E-07
SASS6	-2,01078	1,03E-05
GRAMD4	-2,01489	2,10E-06
ZNF22	-2,01513	1,23E-06
CASP3	-2,01811	9,44E-09
EFCAB7	-2,0184	0,000444
ATP11B	-2,01928	4,61E-19
MAGEE1	-2,02139	7,12E-05
KIF15	-2,02535	5,13E-09
NIPAL3	-2,03028	5,58E-06
NPAT	-2,0305	4,10E-12
RAP1GDS1	-2,03228	2,38E-16
PCED1A	-2,03399	4,75E-05
TECPR1	-2,03512	6,00E-11
ECHDC1	-2,03591	6,99E-11
HECA	-2,03649	2,12E-06
MYCBP2	-2,03878	4,43E-17
ANO6	-2,0419	1,93E-14
ZNF37BP	-2,04514	1,14E-05
SZT2	-2,0453	4,24E-10
CASP8AP2	-2,04577	1,21E-10
RAD54L	-2,04619	3,74E-11
ABHD3	-2,04712	1,59E-07
PXYLP1	-2,05	3,46E-10
BICD1	-2,05485	2,94E-10
GNPDA2	-2,05497	4,68E-05
GBE1	-2,05639	2,25E-11
CDC25A	-2,05653	6,24E-15
SLC25A20	-2,05795	2,12E-05
PDE4D	-2,05811	1,02E-13
KIAA0922	-2,0592	1,63E-07
ABCD3	-2,06012	4,63E-16
ZNF493	-2,06071	1,85E-06
GATSL3	-2,06844	0,000105
SNX10	-2,07064	1,18E-06
PIDD1	-2,07172	5,38E-10
PCMTD1	-2,07375	3,52E-07
PTP4A2	-2,07513	9,72E-19
TRIM14	-2,07574	6,46E-07
SNHG3	-2,07645	8,06E-07
CENPW	-2,08515	3,58E-10
MCAM	-2,08625	2,64E-05
CHRNB1	-2,0875	2,60E-05
ASF1A	-2,09366	5,57E-16
ago-04	-2,09585	4,15E-10

FBXO25	-2,09659	3,71E-10
NAB1	-2,09676	1,76E-09
ZCCHC3	-2,09913	3,83E-11
FADS3	-2,10168	4,04E-14
MCM8	-2,10305	3,98E-16
PAXIP1-AS2	-2,10428	6,38E-05
SLCO3A1	-2,10606	3,95E-08
CARD8	-2,10729	1,47E-08
CHD9	-2,10751	3,97E-12
ZSWIM3	-2,10931	3,63E-05
SLC35D1	-2,11336	7,71E-11
APOBEC3F	-2,11378	0,000101
ARL11	-2,11417	0,000241
TAF4B	-2,11536	4,66E-08
FMNL3	-2,11788	3,08E-15
STK4	-2,12141	2,51E-21
TTC7A	-2,12182	4,43E-17
SH3KBP1	-2,12326	3,33E-09
STK39	-2,12467	3,38E-08
POMK	-2,12712	2,24E-05
KAT2B	-2,12754	2,83E-05
TGFBR2	-2,1283	2,09E-09
ITPR1	-2,1294	5,94E-10
SMAP2	-2,13244	6,28E-07
LINC01128	-2,13282	2,31E-06
LOC100506	-2,13547	0,000779
SLC16A10	-2,14504	2,89E-05
EXOG	-2,14545	0,00051
PIP4K2A	-2,15072	3,87E-20
HSD17B11	-2,15669	0,00012
CRLF3	-2,15716	5,46E-09
NCAPD3	-2,15774	3,03E-16
DDIT4L	-2,16205	5,21E-09
CCSAP	-2,16253	2,31E-10
C15orf41	-2,16454	6,07E-06
ARFGAP1	-2,16536	1,28E-23
DCK	-2,16569	4,07E-20
USP51	-2,16845	0,000231
TTC38	-2,16935	1,01E-09
EPDR1	-2,16992	1,60E-08
SOBP	-2,17274	9,74E-08
UTRN	-2,17282	7,76E-12
PRKACB	-2,17322	2,57E-15
FADS1	-2,17486	9,78E-13
ABHD17B	-2,17566	2,49E-15
OGT	-2,18163	4,76E-07
ARID3A	-2,1822	3,93E-05
KATNB1	-2,18385	3,99E-14
MAPKBP1	-2,18476	4,53E-08
PLIN2	-2,18684	9,12E-11

SLC8B1	-2,19132	3,09E-09
GANC	-2,19447	1,90E-07
ULBP1	-2,19478	0,000234
TTC28	-2,19631	1,15E-10
ST6GALNA(	-2,19836	6,37E-06
SCN8A	-2,20093	0,000318
SEPT9	-2,20114	3,49E-08
WDR76	-2,20286	3,75E-12
ING5	-2,20775	4,80E-08
CD83	-2,21055	1,38E-07
SORL1	-2,21198	6,34E-14
USP11	-2,21423	1,12E-14
FAM111A	-2,2162	4,10E-15
LRRC37B	-2,21836	1,36E-05
CLCN6	-2,21936	0,000161
RP2	-2,22212	2,16E-07
FUZ	-2,22311	7,79E-10
FAF1	-2,22427	4,07E-09
IFI27L1	-2,22802	7,35E-08
ZNF853	-2,23049	0,000287
ATP1A3	-2,23239	2,21E-06
FAM69A	-2,23591	0,000114
ZNF605	-2,23653	4,61E-06
L3MBTL3	-2,23819	8,08E-06
NAPRT	-2,23847	4,28E-07
GPATCH11	-2,23961	1,32E-09
ICAM3	-2,2414	2,66E-09
CTSC	-2,24186	1,42E-19
BRWD3	-2,2426	1,68E-06
SLC10A7	-2,24313	3,40E-06
MGAT4A	-2,2462	4,72E-14
FNBP1	-2,24913	5,51E-26
CEP19	-2,25108	1,14E-05
CNTRL	-2,25434	1,50E-09
CD82	-2,26336	1,71E-12
CDS2	-2,26615	1,36E-20
RPL23AP53	-2,2678	4,07E-09
CYP2U1	-2,27056	5,13E-07
P3H1	-2,27114	4,27E-14
ZNF445	-2,27258	2,12E-12
HIVEP2	-2,2764	9,85E-16
TRAF5	-2,27872	1,31E-07
BMP2K	-2,28189	2,92E-18
SPIN4	-2,28379	1,32E-07
TREX1	-2,2845	3,42E-06
ECE1	-2,28482	1,84E-17
GNB4	-2,28485	5,24E-13
PAQR3	-2,28546	2,99E-15
RASSF2	-2,28651	4,19E-07
SARM1	-2,29188	5,04E-09

ATP8B2	-2,29286	3,59E-13
RAB9B	-2,30237	2,42E-05
LOC285074	-2,30985	4,37E-05
DNMBP	-2,31715	8,03E-11
RRAGD	-2,31822	2,55E-08
KCTD17	-2,32285	1,22E-07
ABCB10	-2,32314	3,00E-14
SNHG10	-2,32429	1,68E-06
SMC1A	-2,32553	4,57E-23
HSPB11	-2,32741	1,87E-13
ZNF772	-2,3278	5,01E-12
C11orf54	-2,32797	5,91E-09
ANKMY2	-2,33538	1,55E-12
RCBTB2	-2,33659	3,12E-08
PLCH1	-2,33847	1,02E-26
STMN1	-2,33931	7,93E-13
IL1RAP	-2,33983	0,000242
STRIP2	-2,35091	0,000327
TTC21B	-2,35551	4,10E-11
IKBKE	-2,35803	3,84E-05
FRG1HP	-2,36334	8,78E-08
SLC1A4	-2,36352	2,02E-07
RIN3	-2,36612	2,94E-07
AMIGO3	-2,36993	1,18E-09
CDK5RAP3	-2,37364	6,44E-07
ZMYM6NB	-2,37378	3,16E-06
TMEM170B	-2,37499	5,46E-09
SLC5A3	-2,3752	6,64E-20
PHF7	-2,38154	2,46E-05
GHDC	-2,38324	1,15E-09
CDPF1	-2,38458	7,86E-07
TUBGCP6	-2,39237	6,27E-05
TMX3	-2,39607	4,60E-12
CCDC15	-2,40074	1,04E-05
LOC101927	-2,4008	0,000315
DUSP7	-2,4027	4,76E-08
DACH1	-2,40352	9,12E-09
SPSB1	-2,40356	1,91E-06
ANKRD13A	-2,40606	1,21E-10
STS	-2,41413	3,03E-10
CCDC18	-2,41695	2,29E-06
RNPEPL1	-2,42017	5,94E-14
RB1	-2,42272	4,23E-26
CHST14	-2,43201	1,68E-13
TMEM106A	-2,43448	1,67E-06
APBA1	-2,43609	1,34E-06
RHOU	-2,44075	1,44E-07
TESC	-2,44997	1,71E-06
GK	-2,45512	2,19E-10
KCTD7	-2,46367	3,49E-09

CHM	-2,46424	1,04E-07
FAM135A	-2,47272	6,69E-08
ICA1L	-2,47654	4,86E-07
HACE1	-2,48016	1,70E-08
CARD8-AS1	-2,49241	3,16E-05
DGKE	-2,4925	5,87E-11
KIF13B	-2,50098	1,05E-06
C21orf58	-2,50406	1,50E-06
TNFAIP8	-2,51081	5,14E-17
ZNF512	-2,51184	3,02E-20
EMB	-2,51364	5,27E-30
TIGD7	-2,51664	2,91E-08
ZEB1-AS1	-2,51723	4,58E-07
PARP8	-2,52021	5,02E-15
TSPAN5	-2,52241	9,22E-09
CCDC109B	-2,52278	9,05E-15
EVI5L	-2,52438	3,54E-10
ADGRB2	-2,531	4,16E-10
PDE3B	-2,53101	1,21E-17
RBMS2	-2,53444	1,64E-10
ATHL1	-2,54527	0,000405
PLEKHM3	-2,55103	1,22E-10
SLAIN1	-2,55208	3,08E-12
MFGE8	-2,56043	2,79E-16
B3GNTL1	-2,56382	2,30E-08
ST3GAL3	-2,56544	3,52E-10
CPQ	-2,56652	6,15E-05
FCGBP	-2,56666	0,000594
SOCS1	-2,58014	1,95E-16
TRIM46	-2,58275	0,000124
GNG4	-2,58353	8,94E-06
CROCCP2	-2,60057	0,000161
SMIM3	-2,60377	8,52E-18
SYNJ1	-2,60426	9,25E-10
TTBK2	-2,60723	1,09E-07
PMAIP1	-2,6088	2,18E-11
KDM6A	-2,61247	2,43E-17
B2M	-2,61596	1,03E-15
TSHZ1	-2,62332	6,88E-14
NREP	-2,62366	3,84E-14
HOOK3	-2,62429	1,84E-19
TRAF3IP2-A	-2,62588	4,61E-08
C12orf75	-2,63152	2,08E-25
DPYSL2	-2,63677	1,11E-16
OGDHL	-2,63738	1,10E-16
DLG4	-2,64065	1,72E-06
NFATC1	-2,64653	1,54E-16
ARFGAP3	-2,6499	7,31E-21
PRKXP1	-2,66407	0,000737
RGS19	-2,66425	2,17E-15

NFATC2	-2,67228	7,74E-13
STAT5B	-2,67592	5,18E-35
C9orf9	-2,68225	5,94E-06
CLIP3	-2,68322	5,27E-08
MVB12B	-2,68324	3,41E-19
OXCT1	-2,687	2,21E-28
PLTP	-2,68765	5,69E-06
DIAPH2	-2,69364	9,95E-18
NEIL3	-2,69575	2,18E-07
SP4	-2,69662	4,28E-10
EXTL2	-2,69834	3,58E-10
LETM2	-2,70049	0,000637
USP49	-2,70078	2,35E-14
ANKH	-2,70256	2,33E-23
STIM1	-2,70445	1,27E-30
ACADM	-2,70574	1,61E-30
MFHAS1	-2,70738	2,58E-25
CAPG	-2,7074	1,09E-20
BIVM	-2,71333	9,87E-07
ARL4C	-2,71854	1,38E-29
RASA2	-2,71936	6,67E-13
ZNF155	-2,72081	2,21E-06
KCNQ5	-2,73617	1,72E-06
HIVEP3	-2,73729	0,000143
TTC33	-2,74085	1,62E-15
C20orf196	-2,75092	3,46E-06
NXPE3	-2,7512	1,13E-07
STMN3	-2,75137	3,89E-32
PLA2G6	-2,75224	4,50E-07
SEC14L2	-2,75689	4,15E-08
NDRG1	-2,75874	4,83E-14
TRIM47	-2,76006	1,51E-11
MTMR10	-2,76289	3,01E-19
GNAO1	-2,76793	0,000774
TRIB2	-2,77108	5,39E-12
TCF12	-2,77647	4,11E-21
MTF2	-2,79508	1,86E-23
PRKCH	-2,79868	6,11E-21
TFPI	-2,79914	2,21E-06
PPARA	-2,80185	4,26E-17
CNOT6L	-2,80208	1,79E-33
USP20	-2,82465	1,51E-33
RECK	-2,82627	1,90E-05
SLC12A4	-2,82803	2,28E-05
TMED8	-2,83731	3,56E-11
SERPINF1	-2,8427	0,000253
GALNT7	-2,84775	7,33E-18
CDON	-2,84895	1,30E-08
SSH2	-2,85181	2,94E-25
BAHCC1	-2,85261	2,94E-11

PGM1	-2,85896	7,44E-16
KHDC1	-2,8624	4,72E-07
PAK1	-2,86593	4,49E-16
SCMH1	-2,90758	6,07E-10
TYMS	-2,91979	8,68E-40
DUSP10	-2,92028	2,96E-05
SHPRH	-2,92492	3,45E-15
BZRAP1-AS	-2,9259	1,91E-07
C19orf38	-2,9329	0,000284
ZNF880	-2,93587	0,000691
ZNF702P	-2,93853	6,15E-06
PELI1	-2,94292	9,50E-09
ITM2C	-2,94339	9,50E-24
ATAD5	-2,94352	5,96E-12
CD8A	-2,94533	3,40E-21
PRTFDC1	-2,94605	3,17E-15
ZNF81	-2,95172	1,20E-08
TERT	-2,95801	1,94E-05
NMI	-2,95816	6,24E-13
CHST7	-2,9585	6,82E-08
NMNAT3	-2,96082	1,92E-06
ARAP2	-2,96665	7,31E-21
CECR6	-2,96895	5,50E-06
SMCO4	-2,96999	9,39E-09
C16orf62	-2,97054	2,27E-20
CMTM1	-2,97625	0,000548
RNASEH2B	-2,98084	5,27E-24
FXYD5	-2,98455	2,80E-21
GPR63	-2,9874	3,44E-07
SLC2A3	-2,99096	0,000304
TSPYL4	-2,99614	1,36E-26
NEDD9	-2,99836	0,0008
ASRGL1	-3,00722	2,18E-20
ADAMTS17	-3,00829	6,39E-11
GCHFR	-3,0125	1,42E-15
PARP10	-3,01556	1,15E-12
ZNF266	-3,01589	4,77E-09
GALT	-3,01734	2,55E-07
RORC	-3,02056	4,19E-11
GSDMD	-3,02338	3,19E-10
LRRN1	-3,02467	9,27E-16
ARID3B	-3,03179	1,93E-13
ADAM10	-3,03406	4,34E-44
PPP2R3B	-3,03492	1,34E-16
TMEM8B	-3,04595	2,10E-11
UBALD2	-3,04754	1,15E-11
ZNF469	-3,05418	0,000488
CCND3	-3,05863	1,10E-12
YPEL1	-3,05964	1,63E-10
DDX60L	-3,06131	9,63E-11

MICB	-3,06669	1,73E-14
CRACR2A	-3,07558	1,58E-05
PTK2B	-3,09142	2,50E-22
RIMS2	-3,09194	1,03E-08
LRRC37A4F	-3,1114	1,25E-13
GRK5	-3,11563	6,54E-10
SUSD1	-3,11581	3,17E-22
COCH	-3,12045	7,76E-12
ACO1	-3,12542	1,81E-17
BISPR	-3,12851	5,95E-07
RAPGEF5	-3,1382	1,51E-11
CHST11	-3,13842	1,70E-25
MAP10	-3,14385	3,79E-12
STK17B	-3,15591	7,82E-31
RPGR	-3,15642	9,96E-07
HLA-A	-3,15955	1,69E-24
CAPN10	-3,1624	2,48E-15
PPM1M	-3,17095	1,17E-11
CCDC82	-3,18211	7,11E-11
UHRF1	-3,18507	8,48E-27
AGPAT4	-3,18977	3,08E-07
ATL1	-3,19255	8,76E-07
KBTBD11	-3,20539	4,19E-12
LRP4	-3,23683	3,46E-16
DAAM2	-3,23858	6,65E-12
ZNF814	-3,24084	2,46E-06
SCCPDH	-3,24415	1,02E-32
NT5M	-3,24474	8,62E-13
SH2D2A	-3,25295	4,72E-11
OSBPL5	-3,26237	6,68E-26
CMTM7	-3,26277	1,49E-12
FLVCR1-AS	-3,26474	2,76E-06
S100A4	-3,26826	7,88E-16
CDKN2C	-3,26943	7,18E-41
ANKRD36C	-3,27534	8,03E-06
LNP1	-3,2792	1,51E-06
CMTM3	-3,28037	6,92E-17
DLEU2	-3,30933	2,93E-10
DEF6	-3,32196	3,60E-37
JAK3	-3,32226	2,08E-21
CCDC28B	-3,32284	4,53E-12
CDCA7	-3,32615	1,29E-15
ARHGAP26	-3,32915	4,23E-11
TMSB4X	-3,33258	1,04E-52
DHX58	-3,33331	9,45E-06
ERG	-3,34056	2,41E-22
SLC25A42	-3,35195	7,50E-20
LOC283788	-3,3541	2,17E-21
ASB9	-3,35693	0,000599
PLEKHG1	-3,35804	1,52E-28

ITGA5	-3,36046	5,32E-17
PIK3CD-AS1	-3,36371	0,000329
LINC00885	-3,36659	1,14E-05
AKNA	-3,37456	8,36E-11
RASA3	-3,3803	1,61E-31
HYI	-3,38106	1,17E-14
E2F2	-3,38626	5,25E-16
SYNE1	-3,38898	3,52E-07
SPRED1	-3,391	6,13E-10
CENPV	-3,3995	1,82E-14
SSBP2	-3,40483	5,53E-13
ASMTL-AS1	-3,40977	0,000792
CD109	-3,40992	7,78E-13
ABTB1	-3,41246	0,000138
SH2B3	-3,4364	5,37E-29
PPP1R1C	-3,4473	2,75E-07
RASL10B	-3,4527	2,70E-18
STXBP1	-3,45593	1,30E-39
SLC39A8	-3,45787	2,34E-25
VAMP1	-3,46732	2,92E-05
SIPA1	-3,47322	1,36E-23
GALNT2	-3,47397	5,10E-55
FNIP2	-3,47572	6,83E-11
SHANK1	-3,47861	1,93E-13
LINC01237	-3,48208	6,46E-06
SEMA4D	-3,4905	1,30E-28
HMHA1	-3,4999	2,06E-45
FILIP1L	-3,50182	3,71E-13
ARL17A	-3,50532	0,000318
ATP2A1	-3,51295	0,000129
GLIPR1	-3,52251	8,59E-08
NCR3LG1	-3,52291	2,19E-13
TPM2	-3,53367	0,000761
AMPD3	-3,53694	3,67E-12
GLIPR2	-3,54218	7,05E-20
UBE2L6	-3,54825	4,27E-13
LBR	-3,55918	4,32E-52
CSF1	-3,56247	3,88E-12
NUCB2	-3,5701	4,23E-16
RTTN	-3,59243	1,25E-24
C2orf48	-3,61216	0,000143
VAMP5	-3,61898	2,37E-07
APOL1	-3,62088	0,000489
FLT1	-3,63444	0,00013
PDCD4-AS1	-3,63876	0,000844
EDEM1	-3,64173	1,16E-35
PSMB8-AS1	-3,64684	2,41E-05
NOS3	-3,66094	4,00E-06
BEX4	-3,66864	1,68E-25
IFITM1	-3,69719	5,61E-11

DENND3	-3,69793	7,22E-12
CCDC136	-3,70632	9,48E-16
LINC01089	-3,71035	1,21E-06
HLA-B	-3,71525	1,45E-14
CD99	-3,73517	1,42E-48
HNRNPU-A	-3,74447	3,80E-06
FAM78A	-3,75517	1,55E-18
MICAL1	-3,76056	3,25E-22
WDR90	-3,77962	5,32E-11
LINC00342	-3,78075	6,44E-06
FYN	-3,78849	3,31E-21
BTBD11	-3,79074	6,15E-30
PITPNM2	-3,80527	1,48E-36
FLT3LG	-3,81626	1,02E-09
CXorf57	-3,84244	1,73E-11
FAM46A	-3,84644	3,80E-22
PALD1	-3,85969	1,14E-20
KIF7	-3,86027	2,20E-05
ACSS1	-3,86345	2,13E-30
MRC2	-3,87086	2,79E-16
CYFIP2	-3,87613	2,82E-22
PABPC4L	-3,88365	9,57E-16
HERC3	-3,88479	1,53E-22
GPRASP1	-3,89178	1,04E-13
EEPD1	-3,90698	1,01E-10
JAZF1	-3,9105	2,37E-06
ZNF513	-3,91214	8,28E-08
NKD2	-3,9152	9,10E-52
ERAP1	-3,92329	7,44E-61
STRA6	-3,93638	1,65E-06
SH3BGRL	-3,94055	2,43E-42
TIAM1	-3,94555	7,92E-41
RIMS3	-3,96638	9,21E-21
CEP85L	-3,99088	2,46E-20
TENM1	-3,99241	1,20E-22
LOC100506	-3,99407	4,91E-14
CORO2A	-3,99887	5,45E-27
C19orf66	-4,00016	2,34E-11
CD99P1	-4,00326	1,64E-08
ITGA6	-4,00365	4,58E-48
AKAP7	-4,0075	4,92E-11
RUNX1	-4,01404	9,75E-88
EML5	-4,02025	2,92E-14
SERHL2	-4,02455	6,51E-11
ZNF382	-4,04748	3,57E-07
VWCE	-4,05377	3,79E-12
AFF3	-4,05676	9,45E-31
VAV1	-4,05769	1,80E-35
CASP4	-4,06011	0,000118
GLRX	-4,08297	5,99E-07

FUT4	-4,0841	1,09E-28
CUBN	-4,09529	0,000579
HHEX	-4,09686	2,31E-19
GPC3	-4,10026	1,41E-10
VIM-AS1	-4,12408	6,82E-20
RILPL2	-4,14638	1,03E-17
VPS13C	-4,14953	4,67E-41
PLCXD1	-4,15417	5,77E-33
STARD9	-4,15821	1,57E-06
PXK	-4,17627	3,71E-17
AKR1C3	-4,18141	6,56E-05
EMILIN2	-4,18773	5,69E-11
ELOVL4	-4,19941	5,44E-20
IL21R	-4,21048	9,38E-08
SH3TC1	-4,22543	1,17E-33
HERC2P3	-4,22785	0,000262
CCDC88A	-4,23457	5,14E-73
LYST	-4,23688	2,87E-18
GYG2	-4,2369	3,21E-25
LCP1	-4,24531	1,42E-19
KCNAB2	-4,2556	1,91E-21
PHEX	-4,27477	2,19E-13
KBTBD8	-4,27582	1,98E-16
RASA4CP	-4,27779	4,59E-12
CRYBG3	-4,28148	6,88E-15
FADS2	-4,29014	7,52E-58
CD72	-4,29501	6,18E-10
HTATSF1P2	-4,29965	5,80E-22
PLEKHA2	-4,30502	2,10E-41
ATP8A2	-4,31613	4,71E-12
RASA4	-4,32075	8,55E-06
GSDMB	-4,32856	3,94E-05
PIK3R5	-4,33802	8,24E-15
LRRC8C	-4,35736	5,33E-30
GXYLT2	-4,35926	3,91E-28
PCDH9	-4,36357	1,39E-22
TIMP2	-4,37699	3,43E-61
ATP8B3	-4,37956	3,89E-06
RASSF4	-4,38643	5,74E-10
SP100	-4,3994	2,26E-08
CAMK1D	-4,42315	2,64E-61
KLF12	-4,43654	1,20E-58
TFDP2	-4,44433	2,09E-104
CDKN2D	-4,46812	7,74E-26
BTN3A1	-4,46945	1,28E-12
INPP1	-4,47337	1,73E-06
CYP4V2	-4,48817	1,12E-15
ABHD8	-4,49825	6,39E-10
KIAA0226L	-4,52931	2,80E-09
LEF1-AS1	-4,53124	1,76E-13

B4GALT6	-4,534	8,27E-63
HDAC4	-4,54226	1,35E-16
TMBIM1	-4,58144	1,58E-08
BIRC3	-4,5897	5,18E-10
CACNA2D4	-4,60328	6,40E-09
CASC15	-4,63398	3,80E-24
ADRBK2	-4,65225	4,16E-106
FMNL1	-4,65841	2,02E-65
C6orf223	-4,66099	3,63E-11
BTN3A2	-4,66907	4,76E-15
ZNF264	-4,67047	1,57E-38
FAM63B	-4,67775	6,52E-39
TSPAN2	-4,72599	1,53E-07
APBB1	-4,7297	8,97E-27
TNF	-4,75046	2,06E-05
CTHRC1	-4,75951	1,06E-20
XAF1	-4,76061	1,29E-07
FBN1	-4,76326	2,07E-26
DOCK10	-4,76673	1,13E-30
BTN3A3	-4,77232	9,79E-15
STAMBPL1	-4,80479	6,21E-21
FHL1	-4,82525	1,91E-34
KHDRBS3	-4,84202	8,98E-07
DDX11L2	-4,8448	4,25E-10
ALDH2	-4,84915	5,88E-14
PPM1K	-4,86257	7,61E-25
ANKRD44	-4,86409	2,86E-21
TBXAS1	-4,87119	1,29E-11
MGC70870	-4,87929	1,98E-12
DOCK8	-4,88175	2,48E-83
ATP8A1	-4,89456	1,27E-29
GAS6-AS1	-4,90875	8,32E-07
SCN2A	-4,90918	9,53E-05
ANTXR2	-4,93898	1,30E-08
GRIK5	-4,98473	1,88E-08
CIB2	-4,99883	1,18E-19
PIK3CD	-5,0101	1,98E-25
CCDC69	-5,01543	8,04E-32
KCNAB3	-5,01632	1,05E-05
SEPT1	-5,0164	2,39E-53
BIN1	-5,01738	3,96E-24
HERC5	-5,01863	6,98E-11
RASSF5	-5,02555	1,16E-31
POU2AF1	-5,04201	1,27E-05
PCDHGB4	-5,04603	3,66E-10
ARHGAP23	-5,05053	1,80E-28
C14orf159	-5,05211	2,84E-46
CXCR4	-5,06749	3,98E-103
ARHGAP4	-5,07293	4,35E-68
GBP5	-5,09952	7,01E-05

BZRAP1	-5,10605	0,000307
MX2	-5,10884	1,45E-36
CMPK2	-5,11585	1,96E-21
PDE1B	-5,1259	1,22E-05
TRIM22	-5,13336	1,32E-10
GBP1	-5,14345	7,08E-08
C17orf51	-5,15049	3,84E-32
SARDH	-5,16652	3,37E-21
ST6GAL1	-5,1697	1,64E-32
CAMKV	-5,17176	5,74E-06
MAL	-5,18713	2,42E-08
SLFN11	-5,1924	1,12E-61
CPVL	-5,19289	1,77E-17
UBASH3B	-5,21732	1,14E-46
ZNF423	-5,24534	1,14E-60
TNFAIP2	-5,2697	5,40E-13
DPY19L2P2	-5,28439	3,86E-16
CHN2	-5,29299	2,89E-06
FGF9	-5,30828	3,62E-05
SATB1-AS1	-5,3139	0,000576
LIMD2	-5,32093	4,11E-52
PLCL1	-5,32534	3,37E-38
IL9R	-5,32787	8,60E-17
SPRY1	-5,33491	1,16E-30
FAM101B	-5,34065	1,16E-68
DLEU7-AS1	-5,39343	3,22E-06
FAM57B	-5,40311	1,98E-11
ST3GAL2	-5,40332	7,06E-48
MRVI1	-5,40675	4,85E-06
TAPBPL	-5,42276	1,77E-30
EPB41L2	-5,4597	1,14E-99
EMBP1	-5,46074	4,96E-13
ANGPTL6	-5,48056	0,000339
HMGN5	-5,54852	1,15E-05
CCDC88B	-5,59443	1,12E-50
GSTM2	-5,61639	0,000165
YOD1	-5,63311	7,77E-28
MAP1A	-5,63878	3,94E-29
LIX1L	-5,65442	2,57E-48
SNPH	-5,67097	7,21E-05
SLC16A7	-5,67365	1,84E-24
LOC153684	-5,67453	2,21E-08
LINC01550	-5,67864	5,67E-22
LPAR6	-5,69178	5,27E-08
BCL2A1	-5,69578	0,000796
PURG	-5,71041	0,000762
TRANK1	-5,71202	9,92E-22
ACSL5	-5,71792	6,31E-17
NLRC5	-5,72241	1,03E-26
VIPR2	-5,72621	4,27E-09

TSPY26P	-5,76973	1,18E-07
RGCC	-5,77011	1,04E-23
LOC72968З	-5,80152	5,31E-09
RCAN1	-5,80308	4,75E-88
GRASP	-5,80648	2,74E-05
RAB32	-5,80943	5,39E-40
LIME1	-5,83459	7,78E-28
RASGRP2	-5,83751	1,94E-22
BST1	-5,84102	3,43E-05
ANGPT1	-5,84202	6,68E-26
DISP2	-5,85209	0,000546
AMN	-5,85467	5,28E-05
C1RL	-5,87636	1,26E-06
ZNF415	-5,90183	6,82E-10
SH2D3C	-5,91933	6,68E-17
DPP4	-5,9195	5,32E-11
HKDC1	-5,92042	1,33E-05
LPXN	-5,92654	2,15E-107
CLIP4	-5,95834	7,38E-10
MLLT11	-5,96438	6,62E-63
TTN	-5,97401	0,000229
CHRNA3	-5,98608	9,05E-30
MAPRE2	-5,99349	1,26E-94
KIAA2022	-5,99523	3,66E-18
RLTPR	-6,00993	7,17E-130
CORO6	-6,01283	3,27E-07
ZNF578	-6,03105	2,89E-08
N4BP2L1	-6,0436	1,33E-14
P2RY1	-6,04498	0,000137
NOG	-6,05404	1,73E-14
DCHS1	-6,13467	2,70E-13
TM6SF1	-6,1371	1,13E-09
ADGRF3	-6,13724	8,21E-05
RFTN1	-6,14055	6,01E-63
PRG4	-6,15543	9,29E-08
CCDC181	-6,16689	4,04E-05
IL16	-6,20057	7,10E-48
NXF3	-6,20838	6,23E-05
C10orf25	-6,22139	6,43E-12
CLEC11A	-6,22761	8,09E-75
ADA	-6,23355	6,28E-91
ANKLE1	-6,24553	3,35E-12
PAG1	-6,32062	7,88E-97
ZNF491	-6,3388	2,48E-05
IRGM	-6,41358	1,64E-05
SCARF1	-6,43393	1,18E-13
GJC2	-6,43618	1,27E-05
WAS	-6,44393	1,41E-51
SYT11	-6,45142	7,10E-12
RGPD3	-6,45793	1,19E-17

CORO1A	-6,47047	1,16E-81
ACOT2	-6,47556	1,44E-20
SLC17A9	-6,50119	5,10E-48
TRPV2	-6,51165	5,72E-29
PTPN22	-6,51691	9,18E-29
HOXB3	-6,52179	7,65E-07
SELL	-6,56372	3,94E-26
ERVH48-1	-6,56638	1,03E-17
CDH23	-6,57282	3,27E-22
PDE6G	-6,58461	8,37E-09
HEMGN	-6,59316	4,81E-06
ZNF528	-6,59463	7,55E-21
NAP1L5	-6,5964	8,62E-14
IKZF2	-6,62535	9,16E-44
MYB	-6,62718	2,68E-147
LTB	-6,62779	1,75E-06
WIPF1	-6,64048	2,06E-122
SEPT6	-6,64789	1,34E-66
ARMCX4	-6,64986	2,67E-11
TMEM173	-6,65136	8,57E-15
LOC102723	-6,65328	1,49E-07
PSMB9	-6,65636	2,69E-59
MIR142	-6,6641	2,24E-05
DUSP6	-6,67673	1,10E-05
SYN1	-6,71463	1,14E-26
EPSTI1	-6,71981	1,96E-06
TRO	-6,725	3,14E-13
ITGA1	-6,72903	0,000759
ST3GAL6	-6,73737	0,000674
TRIM9	-6,73835	1,85E-27
HPGD	-6,73851	3,25E-38
TCF7	-6,7396	1,30E-121
FAM184A	-6,744	5,36E-12
NAP1L3	-6,75209	2,46E-06
ZDHHC2	-6,79426	5,37E-48
HOXB4	-6,80821	3,26E-13
APOL3	-6,81041	4,90E-11
CAMK4	-6,82727	3,02E-45
APOL6	-6,84055	2,68E-61
GPSM3	-6,84544	2,73E-48
FSTL1	-6,85003	3,67E-42
SLC18A2	-6,85877	2,77E-31
FLJ32255	-6,87824	1,37E-13
LINC00461	-6,90615	0,000402
ZNF844	-6,90773	0,000353
ACOT1	-6,91377	0,000369
KCNH8	-6,92267	0,000345
C1orf228	-6,92587	1,30E-35
CRIP1	-6,93031	4,15E-95
CASP10	-6,93339	7,03E-32

ELMO1	-6,94788	1,16E-51
CDK6	-6,94923	1,03E-50
CCR7	-6,95786	3,80E-23
IL32	-6,96093	1,66E-07
SLC4A4	-6,96288	3,65E-14
STAG3	-6,96447	1,53E-14
SPOCK2	-6,98012	1,98E-53
TNFAIP3	-6,98677	2,25E-88
CD79A	-7,02269	1,02E-22
UGT3A2	-7,04393	3,98E-07
FAM129C	-7,049	1,27E-21
NLRP1	-7,0963	0,00023
LINC01138	-7,10305	4,04E-07
TRIM38	-7,10401	0,000633
SLC44A5	-7,1238	0,000243
EMP3	-7,12503	1,18E-31
DGKA	-7,12607	3,62E-55
PILRA	-7,15482	0,000144
GZMA	-7,1625	7,25E-08
CD79B	-7,16712	6,37E-08
TMEM156	-7,19099	3,37E-08
PTGDR2	-7,20022	6,58E-08
TNFRSF8	-7,21166	4,20E-11
AEBP1	-7,24211	6,77E-112
ANXA1	-7,27196	2,92E-185
ARHGEF6	-7,33312	6,64E-124
DPEP1	-7,3369	6,83E-08
RUNX3	-7,34963	6,82E-15
OSBPL3	-7,37791	1,66E-35
DBH-AS1	-7,39007	3,67E-15
JAKMIP2	-7,40171	1,98E-08
JAG2	-7,40786	0,000642
P3H3	-7,42432	2,91E-18
FERMT3	-7,42477	7,63E-87
LINC01260	-7,42839	9,14E-09
GFI1	-7,44434	1,57E-40
PNMA2	-7,465	4,08E-62
BIN2	-7,47256	1,39E-57
NEK9	-7,47431	1,10E-05
CDH2	-7,50339	3,25E-60
GZMM	-7,50561	4,66E-09
HCST	-7,5097	9,71E-20
C2orf88	-7,51707	3,01E-05
TP73-AS1	-7,52842	0,000534
CSTF3-AS1	-7,52988	0,000953
PLAG1	-7,54023	2,49E-12
KCNC2	-7,54049	0,000945
RGL4	-7,55927	3,52E-09
IL12RB2	-7,56389	0,000866
PCDH10	-7,56626	1,20E-44

HEPACAM	-7,57912	0,001007
C10orf54	-7,58581	1,59E-27
GCNT4	-7,58775	0,000889
CCDC26	-7,59412	2,34E-05
MICU3	-7,59699	0,000945
ETV5	-7,59886	6,26E-94
ARHGAP19	-7,60548	0,001015
SLC1A7	-7,62575	0,000647
MLC1	-7,63698	1,79E-05
LOC403323	-7,64199	2,37E-05
CRYGN	-7,65759	0,000574
ADAMTSL2	-7,66059	0,000563
ZNF683	-7,66431	0,000586
HIST1H3C	-7,67918	0,000528
CCR8	-7,69898	0,000478
NBAT1	-7,70044	0,000486
LOC101929	-7,7149	0,000486
ZNF426	-7,71524	1,55E-20
SV2A	-7,72357	4,41E-77
C16orf86	-7,7494	0,000741
C3AR1	-7,75054	0,000417
HVCN1	-7,76313	5,28E-10
DYNAP	-7,77423	0,000355
LOC728024	-7,78043	0,000346
ZBTB18	-7,7809	5,79E-57
ZNF826P	-7,78385	0,000367
LOC100505	-7,80175	8,31E-18
ZNF788	-7,81322	2,31E-14
LOC100996	-7,84114	0,000284
REC8	-7,84563	2,02E-52
SMO	-7,84977	4,50E-14
FBLN7	-7,86534	0,000238
TUSC5	-7,86988	0,000222
DFNA5	-7,87053	0,000227
LOC100130	-7,87633	0,000233
RGPD4	-7,89523	5,64E-06
C11orf96	-7,9013	0,000233
ZNF655	-7,90359	0,000758
LOC101929	-7,92138	0,000176
TMEM26	-7,9276	0,000182
LOC101929	-7,93133	0,000176
ZNF677	-7,94118	0,000192
PTPRCAP	-7,95739	1,42E-109
GOLGA8N	-7,99499	6,83E-15
TNFSF10	-8,00036	3,76E-06
PON1	-8,00043	0,000153
CHRNA9	-8,00334	0,000136
NPR2	-8,02269	2,52E-05
LOC439933	-8,02847	0,000109
PELI2	-8,03405	8,69E-08

LAT	-8,04437	6,06E-08
TTC16	-8,04977	4,21E-06
RASGRP1	-8,06125	3,93E-148
LGALS9	-8,06746	2,03E-49
ANTXRLP1	-8,07013	0,00011
EGFR	-8,07405	8,71E-05
LIPE	-8,07562	3,74E-11
F13A1	-8,07976	9,60E-05
ILDR2	-8,08028	3,70E-33
GP5	-8,09863	2,36E-06
ARHGAP9	-8,10131	2,72E-27
RFX8	-8,12367	0,000102
LOC100505	-8,13741	8,31E-05
FAM27E3	-8,13868	0,000114
GBGT1	-8,14599	0,000177
FAM163B	-8,16661	5,53E-05
LDLRAD4	-8,16767	3,19E-75
CARD16	-8,17346	5,88E-05
LINC01221	-8,18077	5,26E-05
GJC1	-8,18324	4,83E-21
SLAMF6	-8,20932	1,02E-11
LINC00884	-8,20973	5,30E-05
MPP1	-8,22573	1,08E-83
CFTR	-8,22597	4,07E-05
LINC00920	-8,24256	3,92E-05
GBP3	-8,24627	2,25E-06
ACAP1	-8,2535	2,05E-82
CFP	-8,27017	1,01E-06
RTP5	-8,27076	3,61E-05
TAGAP	-8,27142	5,07E-05
NPTX2	-8,27176	3,25E-05
ARHGDIB	-8,27433	3,52E-07
HAR1A	-8,27853	4,07E-05
PCSK5	-8,28099	1,26E-21
ZNF439	-8,29237	2,38E-11
TNNT3	-8,29989	2,83E-05
C20orf203	-8,33031	2,43E-05
RAB38	-8,33179	3,11E-05
MEI1	-8,34205	2,58E-05
ERAP2	-8,34441	3,16E-96
ACSL6	-8,3471	5,32E-05
ZNF626	-8,34807	2,38E-05
ST6GALNA(	-8,35317	2,43E-05
LINC01225	-8,35938	3,99E-05
SNORD116	-8,37655	2,41E-05
PLCL2	-8,38084	1,33E-27
LINC00689	-8,3825	6,36E-07
GLP1R	-8,38373	2,86E-05
ARHGAP31	-8,3943	8,35E-07
LOC100506	-8,40234	1,83E-32

DENND1C	-8,40256	3,85E-50
SLIT1	-8,41561	1,55E-10
CDO1	-8,41614	1,69E-05
CD7	-8,41872	1,27E-56
LINC01096	-8,4548	2,08E-05
SOGA3	-8,46285	3,05E-26
TIE1	-8,47096	1,34E-05
IL2RB	-8,47479	3,82E-07
NCR3	-8,48375	1,43E-05
RASGRP4	-8,48453	1,08E-05
POU3F2	-8,487	1,95E-23
MAB21L1	-8,49925	1,01E-05
LCN10	-8,50512	1,13E-05
LINC00526	-8,50865	1,64E-05
LOC10037[illegible]	-8,52002	9,38E-06
CACNA1C-[illegible]	-8,52813	1,27E-05
APOBEC3C	-8,53932	4,50E-38
TSIX	-8,54946	9,39E-05
HMHB1	-8,55184	7,84E-06
VILL	-8,55828	1,04E-18
CD52	-8,56393	3,09E-13
SCN9A	-8,57572	3,92E-13
PWARSN	-8,58138	6,48E-06
ZEB1	-8,58575	5,30E-82
DGKG	-8,58763	2,75E-13
SRGN	-8,61781	1,88E-07
GLB1L3	-8,62726	5,90E-06
STAT5A	-8,6404	1,88E-08
RGS18	-8,65309	4,57E-06
CD244	-8,66292	5,74E-06
RORB	-8,66667	1,32E-13
ABCA13	-8,66955	4,05E-06
RTP4	-8,67553	4,91E-06
TFCP2	-8,68924	4,65E-05
PRF1	-8,68949	3,72E-06
TNFRSF14	-8,6933	1,99E-13
LOC101927[illegible]	-8,69498	3,57E-06
LSAMP	-8,69753	3,46E-06
CELF2-AS1	-8,70697	3,68E-06
IFFO1	-8,71538	1,56E-18
MYO18B	-8,7218	3,76E-06
NLRC3	-8,73399	4,41E-05
SLC23A1	-8,73557	2,27E-05
KIRREL2	-8,74121	6,47E-14
LOC101926[illegible]	-8,74204	3,08E-06
FGF17	-8,74277	2,73E-06
POU4F1	-8,74453	2,78E-06
C2orf40	-8,74595	2,91E-06
LCT	-8,74632	7,85E-14
IQGAP2	-8,75158	4,19E-65

SCG2	-8,75543	2,53E-06
MDFIC	-8,75573	6,70E-20
BATF3	-8,78296	8,40E-08
MAP4K1	-8,80934	4,06E-69
BEND4	-8,82189	3,15E-14
FAM27B	-8,85132	1,50E-06
GIMAP1	-8,85438	2,75E-06
ABI3	-8,85869	2,79E-14
TMC8	-8,90569	5,17E-88
REG4	-8,92947	1,07E-06
C2CD2	-8,93404	1,33E-06
GRAP	-8,9381	5,54E-21
EPHB6	-8,95376	1,29E-239
DAPK1	-8,97324	5,73E-64
LOC101928	-8,98436	8,72E-07
TSLP	-8,9858	7,65E-07
CCL28	-8,98728	8,04E-07
SLC40A1	-8,99903	4,00E-05
TSKS	-9,01301	6,71E-07
WT1-AS	-9,03021	5,65E-07
PSMA8	-9,04347	5,29E-07
FGF14-AS2	-9,04892	4,91E-07
GCSAM	-9,05177	5,02E-07
GOLGA8O	-9,11564	7,53E-07
ZNF577	-9,11615	1,31E-06
LY75	-9,1219	7,36E-07
KRT1	-9,12414	3,89E-07
CXCR3	-9,14626	3,85E-07
FGD5	-9,14948	3,99E-07
LINC00654	-9,16369	2,99E-07
NEXN	-9,16889	1,33E-22
RUNX1T1	-9,17002	2,70E-07
SLC38A5	-9,17633	4,43E-55
ZNF831	-9,18129	2,31E-07
CCRL2	-9,19274	2,43E-07
NID2	-9,19343	2,91E-07
MZB1	-9,19831	4,26E-116
SLFN12	-9,20091	2,74E-07
ZP1	-9,20765	2,10E-07
LINC01163	-9,2138	6,19E-07
NDST3	-9,23239	1,05E-08
RAB33A	-9,25334	2,08E-07
PECAM1	-9,28149	8,03E-23
ZNF528-AS	-9,28581	1,78E-07
VIM	-9,28586	8,07E-200
ADAMTS10	-9,29014	3,13E-07
PWAR5	-9,29225	2,17E-07
C1RL-AS1	-9,29275	3,40E-07
CACNA1C-A	-9,29455	2,72E-07
ATCAY	-9,29974	2,82E-07

TRG-AS1	-9,31304	1,15E-07
ZNF521	-9,31956	2,66E-30
ZNF69	-9,32466	1,00E-07
SPTA1	-9,32773	1,01E-07
CCDC141	-9,33504	1,09E-07
PIK3R6	-9,34156	9,84E-08
ZNF71	-9,34831	3,58E-16
C1orf186	-9,36793	7,93E-08
ITGB2	-9,37181	9,59E-96
CST7	-9,37182	7,69E-08
GNGT2	-9,3741	7,51E-08
AFF2	-9,37614	5,91E-06
PCAT18	-9,38229	7,21E-08
TMEM25	-9,39153	8,05E-08
XPNPEP2	-9,40055	6,51E-08
AKR1B1	-9,40177	4,40E-43
IL12RB1	-9,41171	6,09E-08
ICAM4	-9,44134	1,33E-07
MAP3K7CL	-9,45182	5,09E-08
CLEC2B	-9,45507	4,71E-08
SPARC	-9,47116	4,28E-08
MYO7B	-9,5026	3,55E-08
ZDHHC15	-9,54599	2,90E-08
TMEM71	-9,56369	2,53E-08
STAP1	-9,56711	2,69E-08
BLK	-9,57306	2,35E-08
ITGB2-AS1	-9,57619	2,77E-07
CECR1	-9,58493	1,97E-32
TLX2	-9,59496	2,06E-08
THSD7B	-9,60378	2,34E-08
TMEM204	-9,60591	2,22E-08
JAG1	-9,61339	1,91E-08
SELPLG	-9,63877	2,44E-32
NEGR1	-9,64535	1,65E-08
ZNF583	-9,6563	1,50E-08
C14orf39	-9,67396	5,22E-08
PTGDR	-9,68597	1,40E-08
LOC100996	-9,6881	9,43E-10
LRRC34	-9,69416	2,44E-08
TNFSF14	-9,70117	1,14E-08
LY6H	-9,71909	1,09E-08
NPM2	-9,72733	1,03E-08
LINC01226	-9,74208	5,54E-08
LOC101927	-9,76765	7,52E-09
C11orf21	-9,77981	1,03E-08
HSF5	-9,81202	7,47E-09
DTX1	-9,8258	7,56E-09
MYOM2	-9,83119	5,02E-09
LOC100130	-9,84213	5,03E-10
PTCRA	-9,84961	6,28E-09

PLCB2	-9,85368	2,02E-26
SMPD3	-9,86019	3,46E-127
ZC3H12B	-9,86824	4,56E-09
ZNF135	-9,88403	4,18E-09
LOC63930	-9,89791	3,76E-09
LOC101927	-9,91501	3,60E-09
LBH	-9,91661	6,37E-19
SLFN13	-9,93552	2,71E-09
RNASE6	-9,94242	3,72E-09
LRMP	-9,95452	3,81E-35
PNMAL1	-9,95808	2,37E-09
SLC35G2	-9,98375	2,19E-09
LIPC	-9,9955	1,92E-09
ZC4H2	-10,0086	2,21E-19
MYT1L	-10,0144	3,45E-09
ADAMTS1	-10,0197	2,63E-10
LINC01366	-10,0257	1,56E-09
AGPS	-10,031	1,27E-59
GSTP1	-10,031	1,95E-106
RIMBP3	-10,0335	1,92E-09
CD226	-10,0412	1,51E-09
SLC32A1	-10,05	1,67E-09
GRAMD1B	-10,0519	1,38E-09
ZNF665	-10,0522	1,53E-09
SLC43A3	-10,0532	2,53E-05
ZNF154	-10,0609	2,63E-09
ARHGAP30	-10,0799	6,50E-79
ZNF660	-10,0911	1,29E-09
PODN	-10,1065	1,09E-09
GIMAP7	-10,1076	9,92E-10
LY9	-10,116	1,08E-09
PRDM8	-10,1284	9,90E-10
RGPD1	-10,1362	9,00E-10
ST6GALNA(	-10,1431	8,64E-10
GVINP1	-10,1518	8,27E-10
HACD4	-10,1689	8,97E-10
GNA15	-10,1708	1,93E-56
LDHB	-10,1753	0,000514
RRN3P1	-10,1815	1,96E-08
SNX20	-10,1947	6,69E-10
RAB44	-10,2035	6,83E-10
PCED1B-AS	-10,2038	6,13E-10
IGF2BP3	-10,2079	5,49E-10
C20orf197	-10,2089	6,07E-10
ST18	-10,2252	5,27E-10
LST1	-10,2255	4,87E-10
GPR65	-10,2266	4,69E-10
CD27	-10,2322	4,81E-10
ZNF501	-10,2623	3,94E-10
CD1D	-10,2714	5,17E-28

ZNF470	-10,2781	3,82E-10
FRG1CP	-10,295	3,11E-10
MME	-10,3115	7,68E-10
NRROS	-10,319	3,08E-38
GNG2	-10,3239	1,82E-55
ZNF773	-10,3269	2,70E-10
LINC00528	-10,353	2,39E-10
NKG7	-10,3907	2,70E-10
PARP15	-10,3915	6,60E-10
GPA33	-10,4004	1,66E-10
RHOH	-10,4015	4,56E-09
SATB1	-10,4102	1,11E-78
CD8B	-10,4188	1,49E-10
LSP1	-10,4358	2,28E-11
PAX6	-10,4398	1,30E-10
MIR646HG	-10,4482	1,24E-10
ARHGAP25	-10,4639	1,16E-10
ZFP28	-10,474	1,55E-10
LINC00426	-10,5384	7,77E-11
ZNF90	-10,5397	7,61E-11
ZNF649	-10,5434	1,13E-10
CYTH4	-10,5729	5,87E-11
CD48	-10,6126	4,66E-11
LAX1	-10,6281	4,26E-11
ICAM2	-10,6441	5,61E-12
LTA	-10,6486	4,57E-11
LRRC38	-10,6526	3,61E-11
ZKSCAN7	-10,6674	9,03E-11
LINC00539	-10,7038	3,36E-11
TNFRSF1B	-10,7112	2,52E-11
LIMS2	-10,713	1,47E-40
PDCD1	-10,7208	2,39E-11
SPAG16	-10,7255	2,56E-11
LYL1	-10,7516	3,09E-11
CREB3L3	-10,7572	2,36E-11
KLHL3	-10,7717	4,26E-11
FOXB1	-10,7852	1,61E-11
CD37	-10,809	1,11E-22
KCNK17	-10,8121	1,51E-11
C7orf31	-10,8514	1,52E-11
SIGLEC10	-10,8514	1,10E-11
ITPRIPL1	-10,8552	2,28E-12
LOC101927	-10,8787	9,81E-12
ZNF280B	-10,8853	1,33E-11
CD69	-10,8859	5,67E-11
PSMB8	-10,8956	4,75E-43
APOBEC3D	-10,9023	7,84E-12
NLGN4X	-10,9045	8,14E-12
ZFP82	-10,9095	7,54E-12
LAPTM5	-10,9143	7,99E-42

CTSW	-10,9173	1,59E-12
ZNF542P	-10,9211	7,76E-12
CNKSR2	-10,9231	6,92E-12
PDE6B	-10,9234	6,97E-12
P2RX1	-10,9271	1,37E-11
EVI2A	-10,95	5,88E-12
LINC01224	-10,9583	5,86E-12
TTC24	-10,9616	1,56E-11
ZNF671	-10,9734	6,77E-12
ZNF559	-10,9877	5,15E-12
BHLHE23	-11,0233	3,85E-12
PINLYP	-11,0452	3,40E-12
CNN3	-11,0645	6,02E-13
SDHAF3	-11,0676	2,91E-12
LOC10099€	-11,1524	1,72E-12
PSTPIP1	-11,1681	1,80E-12
GIMAP2	-11,1701	1,59E-12
PYHIN1	-11,1728	2,47E-12
MSN	-11,1819	3,10E-11
DOK2	-11,2582	1,34E-12
SIGLEC12	-11,2606	1,54E-12
HS3ST4	-11,2724	8,74E-13
APOBEC3G	-11,2792	8,08E-13
SIGMAR1	-11,298	5,60E-25
LINC00977	-11,3316	6,11E-13
VPREB1	-11,3379	7,56E-13
ZC3HAV1L	-11,3486	5,34E-12
BCHE	-11,3543	6,35E-13
IPW	-11,3569	2,90E-12
WBP5	-11,39	4,39E-13
TREML2	-11,4108	3,57E-13
STXBP5L	-11,4216	3,33E-13
GSPT2	-11,4273	3,65E-13
SIX6	-11,4418	5,20E-13
MDFI	-11,4768	2,62E-13
TCF4	-11,4786	2,86E-13
C8orf88	-11,49	2,19E-13
DLEU7	-11,4907	2,52E-13
RAB37	-11,4977	2,11E-13
FAM65B	-11,5049	4,70E-14
CD300A	-11,5159	2,10E-13
CTSG	-11,5197	1,88E-13
ANGPTL2	-11,5346	2,12E-13
CMAHP	-11,5415	5,74E-12
GDF10	-11,5904	1,34E-13
PVRIG	-11,5943	6,77E-26
MKRN3	-11,5949	4,68E-13
TLE4	-11,6417	2,04E-14
DAB1	-11,6597	8,68E-14
IGLL1	-11,663	8,81E-14

ZNF175	-11,6774	6,96E-14
WT1	-11,713	6,27E-14
TNFSF8	-11,7537	4,40E-14
NSG1	-11,7824	3,75E-14
ZNF506	-11,7974	3,36E-14
LIN28B	-11,8008	3,31E-14
LOC10013(	-11,8161	3,21E-14
PEX5L	-11,8424	2,50E-14
SMIM24	-11,8531	8,76E-15
CD34	-11,8809	3,15E-14
ZIK1	-11,8851	2,04E-14
TLR9	-11,8996	8,41E-15
C5orf42	-11,9356	1,82E-14
AASS	-11,9717	1,68E-14
LINC00667	-11,9776	2,04E-14
SP140	-11,9836	1,06E-14
GPR174	-11,9976	1,15E-14
ZNF320	-12,0438	1,09E-14
PRKCQ-AS1	-12,0474	7,35E-15
LOC100507	-12,063	6,58E-15
ZFPM2	-12,0676	7,40E-15
CD38	-12,0761	6,84E-15
USP44	-12,0763	6,20E-15
TMIGD2	-12,0791	6,22E-15
JAM3	-12,1335	4,20E-15
PTPRD	-12,1625	3,76E-15
PVRL3	-12,1776	3,46E-15
SLC7A3	-12,1859	3,37E-15
ZSCAN18	-12,1901	3,92E-15
SKAP1	-12,1937	3,54E-15
TDRD9	-12,1943	1,28E-15
CYBRD1	-12,2002	2,77E-15
CD84	-12,2145	2,77E-15
LINC01215	-12,2227	2,88E-15
GNE	-12,2451	2,09E-15
NINJ2	-12,247	2,55E-15
FLI1	-12,2668	5,73E-16
NDN	-12,2734	1,90E-15
FBXL7	-12,3214	1,44E-15
PPP1R16B	-12,3545	5,21E-16
HCLS1	-12,371	2,97E-30
KCNA3	-12,395	1,17E-15
UBA7	-12,3958	8,86E-16
CD2	-12,3994	9,91E-16
CD1B	-12,4082	7,82E-16
C16orf54	-12,453	6,48E-16
OGN	-12,4648	5,38E-16
GIMAP6	-12,4836	6,09E-16
LOC101927	-12,487	5,06E-16
ZNF518B	-12,4877	4,99E-16

SLA2	-12,5271	1,93E-16
EMC10	-12,5286	3,94E-16
CD96	-12,5436	1,15E-16
LZTS1	-12,5466	3,44E-16
GPX7	-12,5681	4,71E-16
S1PR4	-12,5779	4,53E-16
KLHL6	-12,6135	2,22E-16
GAS7	-12,6135	2,59E-31
PKIA	-12,6453	1,88E-16
TAL1	-12,6518	1,80E-16
DOCK2	-12,6725	1,36E-46
TCEAL8	-12,7377	9,63E-17
CD93	-12,7436	1,22E-16
TSHR	-12,7904	7,92E-17
TPTEP1	-12,7991	7,40E-17
TBC1D10C	-12,8144	8,23E-17
PIK3CG	-12,8329	6,25E-17
ITGAL	-12,8402	1,74E-17
HHIP-AS1	-12,8538	1,12E-16
NRN1	-12,9066	4,26E-17
KIRREL	-12,9241	2,98E-17
AKT3	-12,9395	2,65E-17
GPX1	-12,9539	3,86E-17
CD6	-12,9732	3,26E-17
DPYD	-12,9928	2,24E-17
LINC00202	-13,0026	2,23E-05
INA	-13,0087	2,43E-17
SAMSN1	-13,0087	2,03E-17
EVI2B	-13,195	5,15E-18
NETO1	-13,2017	4,87E-18
IL7R	-13,2148	4,48E-18
CD53	-13,2658	3,38E-18
TRAF3IP3	-13,2776	3,33E-18
CD247	-13,3197	2,46E-18
SCN3A	-13,3271	2,53E-18
CARD11	-13,3339	2,54E-18
ADAM33	-13,4053	9,50E-18
RAG1	-13,4054	1,21E-83
GMFG	-13,4154	1,51E-18
PARVG	-13,4372	1,05E-18
S1PR1	-13,4603	9,29E-19
SLA	-13,4769	8,44E-19
TRAT1	-13,5033	6,87E-19
RASAL3	-13,5222	6,71E-19
ZCCHC11	-13,5406	7,54E-19
FBLN2	-13,5596	4,93E-19
SPNS3	-13,5977	5,37E-19
UBASH3A	-13,7594	1,27E-19
MFNG	-13,7735	2,11E-19
AIF1	-13,8254	9,62E-20

BCL11A	-13,8814	5,60E-20
GTSF1	-13,8913	5,70E-20
CHI3L2	-13,9587	4,02E-20
THEMIS	-14,0415	2,83E-20
TESPA1	-14,0737	1,79E-20
SIT1	-14,0835	1,79E-20
CD28	-14,1145	1,24E-20
ITK	-14,1158	1,24E-20
RAG2	-14,1219	1,17E-20
LAIR1	-14,1597	9,21E-21
LCP2	-14,1665	8,57E-21
GYPC	-14,1724	1,57E-20
CD3G	-14,216	6,55E-21
IFI16	-14,2246	1,63E-40
PRKCQ	-14,2683	5,94E-21
FYB	-14,2735	1,74E-21
RCSD1	-14,296	4,53E-21
CDH4	-14,3475	7,97E-21
PTPRM	-14,3747	5,80E-21
CD1C	-14,5072	8,27E-22
HHIP	-14,5155	1,11E-21
NCKAP1L	-14,5741	7,98E-22
P2RY8	-14,575	9,73E-22
KIR3DL2	-14,7	4,90E-22
CD1A	-14,7151	2,22E-22
PRKCB	-14,7507	2,42E-22
APBB1IP	-14,7597	1,76E-22
ARPP21	-14,8344	9,73E-23
DNTT	-14,8607	7,43E-23
CHST2	-14,873	8,14E-23
NKX2-5	-14,9716	4,04E-23
MYO1G	-14,9859	3,16E-23
TOX	-15,0301	2,15E-23
CELF2	-15,0873	1,76E-23
PTPN7	-15,1137	1,22E-23
GRAP2	-15,1997	6,89E-24
CD4	-15,2189	9,17E-24
CD1E	-15,2277	1,16E-23
SASH3	-15,3399	3,27E-24
IL2RG	-15,3525	3,18E-24
PTPRC	-15,4525	1,20E-24
RAC2	-15,4682	1,47E-24
ZAP70	-15,6555	2,81E-25
XIST	-15,7087	1,45E-08
CD3D	-15,7419	1,73E-25
SH2D1A	-15,8149	7,53E-26
LCK	-15,8473	9,28E-26
SPN	-16,207	6,12E-27
TSPAN7	-16,2123	4,83E-27
CD3E	-16,5052	6,57E-28

ETS1	-16,5332	3,88E-28
IKZF1	-16,6221	2,45E-28
ITGA4	-17,1723	2,36E-30
ITM2A	-17,2557	2,21E-30
ALDH1A2	-17,6951	4,41E-32

HCC998

Gene	log2FoldCh	FDR
LDOC1	15,02067	1,05E-22
TSPYL5	14,89026	1,22E-22
C3orf14	14,49209	3,13E-21
SPDEF	14,3064	3,22E-11
EFEMP1	14,19703	1,67E-20
MATK	14,01553	5,58E-20
GFRA1	13,80921	6,07E-19
BGN	13,63471	2,29E-06
S1PR3	13,61444	2,39E-11
DSCAM-AS	13,59866	4,78E-11
ACKR3	13,4312	2,28E-18
RTN4RL1	13,38226	3,29E-18
PXDN	13,25621	1,10E-17
BASP1	13,2179	2,07E-09
MYEF2	13,21538	2,63E-17
ZNF43	13,17183	1,18E-17
VAV3	13,15812	1,46E-17
PRLR	13,07816	2,79E-17
AMOTL1	13,03444	3,17E-17
HTRA1	12,94143	5,77E-17
ABCA12	12,92665	2,15E-16
COLEC12	12,79441	1,02E-15
CHST11	12,78896	2,68E-16
EMB	12,77568	1,49E-16
FRMD4A	12,71299	7,12E-16
TFF1	12,69871	2,25E-28
SPG20	12,62977	3,97E-16
GSTM3	12,52983	1,84E-16
PRICKLE2	12,4937	1,17E-27
DTNA	12,47557	1,94E-15
DLX5	12,45386	1,34E-15
FBP1	12,43989	5,01E-43
C15orf59	12,43259	6,36E-15
GALNT16	12,39955	8,70E-06
SLC1A2	12,36273	2,24E-15
C14orf132	12,31425	2,75E-15
ZNF304	12,26446	3,92E-15
MSI1	12,24651	1,17E-16
ZNF85	12,22294	4,72E-15
EVL	12,20139	1,27E-16
NPY1R	12,16606	9,98E-15
ZIC1	12,15098	1,87E-14
CRISPLD1	12,13311	4,50E-14
BCAS1	12,10375	6,76E-05
ACSS3	12,07637	1,85E-28
STC1	12,0239	1,73E-27
GALNT14	12,00203	1,85E-14
ZNF569	11,99968	3,70E-14

TBX2	11,96873	2,33E-14
C5orf38	11,83362	9,20E-14
PCP4	11,82643	6,46E-14
FAM127C	11,82218	1,28E-15
SDC2	11,81758	5,97E-14
MAP9	11,78223	1,14E-13
ASCL4	11,7789	8,52E-14
ZFP30	11,73708	9,97E-14
EFS	11,65203	1,88E-13
TCEA3	11,64927	3,56E-15
TMPRSS13	11,63844	2,79E-13
TDRD1	11,63412	1,82E-13
ST8SIA4	11,62612	2,23E-12
ZNF331	11,49764	2,15E-12
KCNK15	11,49622	5,03E-13
LIFR	11,46712	1,16E-12
RIMS4	11,46459	2,36E-12
PGR	11,45126	2,08E-11
MIPOL1	11,44575	5,75E-13
MB	11,42139	6,55E-13
GLI3	11,40553	1,04E-12
ZNF793	11,39418	8,37E-13
FAM3B	11,39088	8,19E-13
ZNF548	11,38803	9,76E-13
RGMA	11,37579	1,37E-12
LRP2	11,3638	8,30E-07
MPDZ	11,35017	1,54E-12
FGF12	11,34341	1,82E-12
MOCS1	11,28034	4,40E-12
ZNF606	11,26892	3,14E-12
CHST10	11,25254	6,08E-12
RET	11,2525	1,35E-11
ZNF14	11,23012	2,86E-12
SPESP1	11,1971	4,09E-12
SLC7A2	11,1936	8,05E-24
OSMR	11,16314	7,91E-12
GPX3	11,15676	0,000109
ESR1	11,15562	1,76E-34
PCDH18	11,12746	1,73E-12
ZNF433	11,12437	4,19E-12
ZNF93	11,08539	1,19E-13
EFNB3	10,97484	1,03E-11
NHS	10,95329	7,15E-13
DDIT4L	10,9162	2,54E-11
SLC8A1	10,91227	1,43E-11
CASC9	10,85088	2,08E-11
SHISA9	10,83701	3,32E-11
EHD3	10,83165	4,16E-11
MEIS3P1	10,81462	3,46E-11
AARD	10,79535	2,96E-11

ZNF829	10,78589	1,11E-12
ZNF570	10,77631	3,44E-11
ZFP3	10,77346	8,03E-13
CHRM1	10,77044	1,21E-10
ZNF287	10,76751	3,43E-11
ZNF256	10,75648	3,80E-11
ELOVL2	10,73224	4,60E-11
PPM1E	10,70047	1,64E-18
ZNF682	10,67299	6,37E-11
ZYG11A	10,65982	2,03E-10
C18orf63	10,65387	9,57E-11
NMNAT2	10,61635	1,11E-10
GUCY1A2	10,59375	2,33E-12
LINC00925	10,57567	1,24E-10
TIMP3	10,51054	2,81E-10
ZNF486	10,50546	2,11E-10
ZNF732	10,50439	3,28E-10
BATF	10,48033	4,14E-10
FAM110B	10,46914	2,94E-10
ASCL1	10,46833	4,39E-10
MAGEH1	10,44813	3,59E-10
ADGRL2	10,44211	2,87E-10
LYPD1	10,43762	4,25E-10
C12orf56	10,41741	3,11E-10
EPHA6	10,4048	3,19E-10
HSPB8	10,39286	2,68E-27
RAB39B	10,38165	0,000991
SLC9A4	10,37915	2,11E-09
CXCL12	10,375	4,18E-10
MTSS1	10,37222	2,72E-10
PRIMA1	10,35133	1,80E-09
TBX2-AS1	10,3216	7,76E-10
NCKAP5	10,31348	6,02E-10
SYT3	10,3125	1,91E-09
CHST8	10,28533	6,31E-10
DCLK1	10,27077	7,20E-20
CCL2	10,26676	3,35E-09
SYT12	10,23191	1,33E-25
ZNF529	10,20745	1,01E-09
BEND5	10,17209	1,65E-09
ZNF568	10,1655	5,30E-09
SLC2A10	10,16123	1,54E-09
TRPV4	10,13302	2,35E-09
IRX3	10,12342	2,44E-28
PCDHB6	10,1221	2,18E-09
GUCY1A3	10,10669	3,93E-11
EDA2R	10,10556	4,65E-09
ZNF492	10,09695	9,03E-09
TYRP1	10,06715	2,50E-09
SLITRK4	10,04119	3,71E-09

MANSC4	10,01817	2,87E-07
TEX15	10,01486	3,17E-09
CALHM2	10,0135	1,15E-10
IRX4	10,013	6,23E-09
FAM20C	10,00223	7,09E-11
FAM19A5	9,989433	5,77E-09
IRX2	9,979434	1,01E-26
LINC01116	9,966234	4,27E-09
PCDHGB5	9,953704	4,54E-09
FUT9	9,933783	5,09E-09
POU3F3	9,930664	1,12E-08
TENM4	9,9238	9,91E-09
DDX43	9,919997	7,31E-09
GPR27	9,913681	7,08E-09
COL5A1	9,913206	1,63E-08
ALDH3B2	9,894236	1,94E-22
ZNF30	9,878616	9,03E-09
ZNF549	9,865583	1,15E-08
GGT6	9,850192	9,29E-27
IGFBP2	9,830465	8,16E-06
SPTSSB	9,819617	2,73E-22
PRPH	9,816154	1,48E-08
ZNF879	9,782138	1,30E-08
ZNF354C	9,779577	1,46E-08
WISP2	9,776691	7,69E-07
PROM2	9,770735	1,68E-54
C17orf104	9,754095	6,90E-08
FCGR1A	9,752584	8,22E-08
KCNMA1	9,731135	5,83E-08
EYA2	9,72015	6,66E-08
RGS6	9,712256	3,13E-08
CA8	9,697605	3,22E-08
ONECUT1	9,696075	4,29E-08
ALOX15	9,681996	1,77E-14
COL4A5	9,67728	4,76E-08
CNGB3	9,665961	3,30E-08
LOC339862	9,665045	2,86E-08
SFMBT2	9,653294	4,47E-08
RIC3	9,64675	4,73E-08
C2orf74	9,604596	3,61E-08
BLNK	9,590241	1,06E-06
TUB	9,559155	4,64E-08
THBD	9,5528	5,88E-08
TMEM150C	9,514586	1,12E-23
SHISA2	9,514277	9,95E-08
LINC00858	9,51216	1,41E-07
CPE	9,502472	1,19E-09
PGM5	9,500237	9,65E-08
ADAMTS9	9,499543	4,63E-07
RAB31	9,499046	3,34E-09

IGSF5	9,496721	7,88E-08
SLC22A17	9,49438	7,22E-08
CRABP1	9,494295	2,09E-07
SPSB4	9,483238	1,58E-07
GRIK3	9,455727	3,84E-07
HOXC13-AS	9,438673	1,24E-07
PCDHB3	9,437384	9,91E-23
RNF150	9,433937	1,10E-07
ADAMTS7	9,430021	5,67E-09
HTR2C	9,421943	1,06E-07
TMEM163	9,415766	1,48E-07
SH3GL3	9,402797	1,41E-07
CALCR	9,373794	1,93E-07
ZNF550	9,371308	9,41E-07
SNCAIP	9,364852	1,43E-07
PLA2G3	9,3562	9,38E-07
JAM2	9,331028	9,03E-07
MPPED2	9,313587	6,95E-07
ELOVL4	9,309188	5,10E-07
HOXC11	9,302718	4,86E-09
TPTE	9,302609	2,37E-07
MLPH	9,290466	1,74E-41
TENM3	9,275716	1,10E-82
DSCAM	9,275646	5,76E-07
NTN4	9,261591	3,78E-07
AFF3	9,259456	3,40E-07
STMND1	9,255604	2,87E-07
LOC339803	9,250437	6,59E-07
SLC25A21-A	9,248358	3,40E-06
A1BG-AS1	9,247949	4,91E-09
ZNF704	9,247657	2,45E-39
PRICKLE2-A	9,245919	3,14E-07
LINC00664	9,228294	6,52E-07
LINC00052	9,207401	4,05E-08
HAGLR	9,153835	4,86E-07
CACNA1D	9,112495	6,17E-15
ZNF530	9,09584	7,32E-07
SERPINA1	9,087902	7,69E-07
EFHD1	9,080078	2,55E-14
ZIC4	9,065833	8,03E-07
PDZRN3	9,052295	8,56E-07
VAX2	9,051393	1,01E-06
C5AR2	9,03241	2,59E-13
KCNG1	9,027783	8,49E-32
ANK1	9,027005	1,72E-05
SNAP25	9,023529	1,27E-06
CAMK2B	9,020235	1,51E-08
ARMCX1	9,019409	1,92E-06
RFTN2	9,011391	1,84E-06
TMEM132E	8,998563	5,70E-05

ZNF618	8,991527	5,18E-83
SLFN11	8,98255	1,51E-06
FAR2P1	8,982513	9,47E-08
DOCK10	8,978304	2,24E-06
RAI2	8,978304	2,24E-06
LOC728392	8,962452	3,68E-06
TTC29	8,958226	1,50E-06
ELF5	8,949841	1,19E-07
PVALB	8,920841	4,75E-06
NMUR2	8,915326	1,86E-06
HRK	8,911386	3,49E-05
TCHHL1	8,90676	9,48E-05
ZNF726	8,903932	5,35E-06
UNC5C	8,900774	6,05E-06
KCNQ5	8,885436	4,52E-06
ITGA2B	8,884791	9,51E-08
GATA5	8,863292	2,68E-06
SLC9A2	8,859171	4,23E-77
LRRK2	8,847464	3,71E-06
GJA1	8,844207	1,42E-05
ZNF610	8,833965	2,86E-06
LOC100128	8,82488	2,96E-06
ZNF595	8,823963	7,92E-33
GUCY1B3	8,82321	3,82E-06
SLC6A14	8,81885	3,29E-06
CLCA2	8,811747	4,54E-05
FAM196A	8,788932	1,13E-05
ADAMTS19	8,780495	5,39E-40
IGFBP5	8,778477	1,59E-05
ZNF98	8,77494	7,47E-06
HOXD12	8,767594	1,60E-05
TUBA3E	8,73329	1,15E-07
TMEM106A	8,727912	5,89E-06
TARP	8,719868	2,16E-05
NCAM2	8,718227	6,75E-22
ZG16B	8,705074	4,59E-07
BMP2	8,690692	4,33E-38
NUP210L	8,679497	1,37E-18
SPINK5	8,669108	3,64E-07
LOC100996	8,660182	9,21E-06
TRIL	8,656679	1,15E-05
CEACAM21	8,646834	6,40E-13
FLT3	8,64314	7,82E-06
SLC1A3	8,63559	8,26E-06
NPAS3	8,631101	1,33E-05
WFDC2	8,622582	1,59E-05
MIR4458HG	8,612508	9,90E-06
FOXI3	8,599741	1,07E-05
ZNF543	8,597352	1,48E-11
SPOCK1	8,595894	1,28E-05

ZNF736	8,594924	3,42E-23
GPR158	8,591964	1,58E-07
POU6F2	8,585918	1,18E-05
GRID1	8,58298	1,35E-05
AR	8,569033	1,45E-23
GFRA3	8,562623	3,88E-05
CDH18	8,557548	1,46E-05
HDGFRP3	8,548923	4,47E-45
VIM-AS1	8,547515	1,49E-05
IGSF11	8,538476	1,33E-05
RASSF8-AS1	8,53796	1,90E-05
LOC148709	8,524247	2,24E-07
KIAA1324	8,522206	8,85E-32
ZNF418	8,518152	1,67E-05
RNF180	8,512753	2,18E-05
ZNF345	8,509154	1,57E-05
CRLF1	8,50405	2,90E-07
XKR7	8,4996	2,89E-05
SLC52A1	8,49215	5,27E-05
GATA3-AS1	8,476296	2,06E-05
NYNRIN	8,475578	2,94E-09
CYP24A1	8,472555	2,86E-07
ADAMTSL3	8,442187	2,99E-05
XG	8,429003	0,000256
ICOS	8,41825	2,70E-05
GXYLT2	8,417665	2,78E-05
ARL11	8,416019	2,68E-05
LOC440461	8,413876	5,23E-05
LOC101927	8,410804	3,88E-05
ZNF423	8,395554	2,75E-05
LINC00885	8,392123	0,000129
HOPX	8,390407	0,000108
KCNF1	8,385504	3,47E-05
PKD1L2	8,379019	2,99E-05
IGSF1	8,376384	0,000135
MARCH1	8,347751	8,44E-07
GPM6B	8,347093	3,52E-05
KCNU1	8,341908	3,94E-05
KCNJ8	8,324998	4,61E-52
CYP4F22	8,312061	5,35E-05
FHOD3	8,307795	0,000141
CALML5	8,300819	7,32E-05
SGCG	8,29567	0,000106
CPEB1	8,295425	5,71E-05
NRK	8,293176	0,000146
ALOX12P2	8,292017	0,000147
PRKD1	8,288868	6,16E-05
ZNF264	8,274056	5,41E-05
INHA	8,260231	3,39E-05
C8orf48	8,242479	6,64E-05

DRD1	8,236932	0,00036
CAPN9	8,2238	7,91E-05
DYNC1I1	8,212721	2,41E-41
PCDHGB1	8,21223	1,54E-11
LOC441666	8,210199	8,26E-05
PCDHB4	8,203273	3,59E-06
SMTNL2	8,190413	8,40E-05
KDR	8,173282	9,26E-05
CELF3	8,161871	0,000126
NRG3	8,158925	2,04E-06
ZNF204P	8,147438	1,73E-06
PHLDA3	8,146372	6,04E-11
IBSP	8,124372	0,000107
PABPC4L	8,116276	0,000139
RECK	8,108808	0,000176
PLEKHO1	8,106736	1,65E-78
VAV1	8,106392	2,01E-10
SYNPO2	8,10455	9,39E-29
PAPLN	8,097206	0,000155
GSG1L	8,09512	2,04E-06
RAB34	8,092566	7,22E-05
LINC00494	8,090328	0,000121
ARHGEF6	8,084129	0,000137
ABCG1	8,076043	5,92E-33
SETBP1	8,073675	0,000221
NAP1L2	8,073375	0,000236
ZNF813	8,072138	6,12E-19
DAAM2	8,070913	0,000133
CNTNAP3	8,068823	0,000137
CACNA1I	8,052953	0,000269
SUSD2	8,02192	6,25E-13
SCD5	8,011803	1,28E-22
SDK1	8,004071	1,48E-10
ARMCX2	8,002293	0,000229
UPK2	7,99768	2,29E-08
ANKRD18B	7,994776	2,98E-10
BRINP2	7,994224	0,000326
ZNF529-AS	7,993837	0,000188
PCDHB2	7,976507	2,40E-36
GPC3	7,972626	0,000431
C8orf46	7,969714	0,000217
CNNM1	7,96851	3,54E-06
KCNMB2-A	7,952828	3,82E-06
NPR3	7,950304	8,87E-19
ZNF793-AS	7,93521	0,000244
CACNG4	7,918135	1,16E-40
TFAP2C	7,91749	3,27E-109
ZNF311	7,897972	6,50E-09
MIR99AHG	7,895665	0,000741
INPP5D	7,890391	0,000141

WDR72	7,876668	2,83E-58
FLT4	7,875746	1,46E-05
LRFN5	7,871704	0,000325
ERG	7,868279	5,77E-06
DPY19L2P1	7,860023	0,000417
FHL1	7,855166	0,000374
SOX11	7,833519	0,000417
PRUNE2	7,825654	0,000734
JPH3	7,820818	1,00E-09
MIR2052H(	7,820315	0,000608
LRRC2	7,815301	0,000509
C19orf81	7,814472	7,54E-06
ST8SIA1	7,809036	0,000446
PANX3	7,803248	0,000899
C18orf61	7,796867	0,000658
SCUBE3	7,796462	0,000464
CCDC83	7,795145	1,13E-05
KCNN2	7,790019	8,34E-06
FGF13	7,788841	0,000579
TSHZ3	7,788664	1,25E-13
AK8	7,787563	0,00061
NEFH	7,779515	0,000962
PTPRQ	7,776472	0,000488
GATA3	7,776088	2,36E-127
SUSD4	7,770043	9,73E-06
GREB1	7,765709	4,02E-97
RFTN1	7,761384	0,000537
MTMR7	7,758597	9,67E-06
ABCC8	7,756199	0,000528
ST8SIA6	7,747478	0,000734
HCAR1	7,744847	0,000679
MYCN	7,736954	0,000591
SERTAD4-A	7,701789	0,000996
AMOT	7,695159	4,78E-57
NOX5	7,692263	0,00076
SYTL5	7,690903	0,000745
ATP6V1B1	7,690472	0,000983
DOCK8	7,688869	3,33E-09
EVC2	7,67216	0,000942
FAM171A2	7,668868	1,87E-55
TFAP2A-AS	7,661004	1,64E-05
ZNF772	7,639444	4,69E-09
HOTAIR	7,624825	1,85E-05
LDHD	7,612329	1,25E-07
SYT10	7,611063	0,000984
RHOBTB1	7,577975	4,03E-167
MYLK	7,575539	2,32E-05
AP4B1-AS1	7,571421	5,59E-20
EML1	7,552678	6,06E-44
SLC24A3	7,548041	2,89E-12

PRKAA2	7,533779	6,50E-13
ZNF790-AS	7,51893	4,91E-12
PREX1	7,493683	1,37E-68
ZNF737	7,467495	1,74E-30
CALD1	7,458892	8,12E-10
PLXNA4	7,447603	1,24E-17
ZNF681	7,439409	1,68E-29
WIF1	7,434652	0,00018
RIPPLY3	7,417224	4,84E-05
GNB4	7,392777	1,81E-11
CRMP1	7,389575	0,00062
EMID1	7,363448	5,39E-08
CYFIP2	7,349853	3,35E-24
PCDHA6	7,33462	4,15E-55
ANTXR1	7,330373	4,18E-60
LINC00665	7,329018	1,10E-35
RNF182	7,320738	2,27E-18
RERG	7,275784	1,03E-13
ACTA2	7,27102	1,21E-13
RASSF8	7,258937	2,46E-47
OGDHL	7,247019	9,09E-23
FAM109B	7,243978	2,08E-14
CHGA	7,235828	7,52E-18
SEMA3E	7,228582	0,000433
BST2	7,21485	6,38E-05
SCN1B	7,207078	0,000279
GPR68	7,188181	0,000118
TRPV6	7,176456	0,000121
ERBB4	7,170421	2,64E-13
ANKS1B	7,166859	0,000216
ZNF790	7,131837	8,20E-10
DLX3	7,123845	1,65E-44
SCARA3	7,102126	3,44E-25
RASGEF1A	7,101369	4,27E-31
SLIT2	7,10033	0,000178
ZNF876P	7,098426	1,74E-07
SLC47A1	7,09304	1,53E-09
GHR	7,091039	0,000167
CHRNB4	7,055207	0,00049
FRMD6	7,046092	3,70E-86
TNFAIP8L3	7,041312	5,41E-29
PRAME	7,037049	9,78E-14
SNCB	7,035751	0,000947
CCDC3	7,019545	0,00026
CCM2L	7,001187	1,22E-09
SULF2	6,996305	7,39E-53
HOXC10	6,953238	1,78E-65
FBXO15	6,936258	1,17E-06
PTPN13	6,934679	2,97E-36
SEMA4A	6,93265	1,14E-34

MAFB	6,92537	2,04E-27
OBSL1	6,918156	6,07E-51
NEURL1B	6,912698	1,43E-54
TLE2	6,909141	1,04E-43
PDZD4	6,879944	1,03E-14
MAPT	6,877663	4,34E-41
PCDHA13	6,848575	2,28E-06
COL9A2	6,838313	6,99E-15
CBFA2T3	6,837361	1,05E-44
NUPR1	6,835696	6,81E-29
PCDHB8	6,828096	2,30E-30
NAALADL2	6,804651	4,76E-20
FREM2	6,801726	5,48E-49
SEMA6B	6,791715	9,91E-23
MEX3B	6,78399	1,28E-38
PAX5	6,781135	3,56E-05
FOXP1	6,77248	1,33E-40
HOXC-AS3	6,771946	2,43E-08
LOC284581	6,717772	6,10E-08
KCNJ4	6,690664	0,000934
LOC10013(	6,631209	1,13E-05
SIDT1	6,622284	4,68E-14
VIM	6,60453	1,11E-05
TFPI	6,573539	4,02E-05
PRR36	6,569977	2,78E-38
C14orf37	6,558088	6,12E-07
S100A9	6,543337	0,000301
CYP1B1	6,542504	1,90E-32
TNFRSF19	6,540662	2,62E-67
ERC2	6,52357	2,49E-09
SNAR-E	6,518019	5,25E-05
KLHL13	6,511762	6,63E-10
FZD2	6,501492	6,78E-33
NFATC2	6,499418	2,80E-10
LHX1	6,483655	3,90E-07
CAMK1D	6,468028	1,15E-11
SLC25A21	6,456282	3,14E-05
PCDHA12	6,39316	4,32E-16
PACSIN1	6,380908	6,92E-19
TRPS1	6,338882	8,56E-116
HCN4	6,332647	3,82E-17
LINC00649	6,326097	2,25E-05
AEBP1	6,258219	2,31E-06
PTCHD2	6,249279	1,83E-06
AK7	6,207543	2,56E-28
ACTG2	6,204727	5,84E-07
LOC72873(	6,198715	4,10E-06
CDK14	6,192017	4,45E-06
RAB9B	6,189049	0,00015
CERCAM	6,1879	2,35E-39

DIRAS1	6,18067	2,42E-41
A1BG	6,168623	8,03E-10
ARHGEF17	6,155999	7,34E-29
TMEM136	6,155282	7,65E-13
MGAT5B	6,15259	5,34E-06
HLA-DRB1	6,151757	5,62E-09
PPP1R9A	6,141527	5,05E-50
SALL4	6,134368	5,55E-13
TP53INP1	6,133238	1,18E-23
ZNF572	6,122728	2,58E-13
MED12L	6,115241	3,73E-20
PCDHB16	6,107535	6,22E-30
TDRD5	6,10746	3,71E-15
EFR3B	6,090076	1,84E-29
SKIDA1	6,087695	4,57E-12
LIN7A	6,082378	3,50E-36
LCP1	6,074578	1,15E-30
EMCN	6,0627	0,000291
PAX2	6,047439	0,000682
UNC5B	6,045647	0,0001
KCNIP3	6,030484	3,10E-12
AGAP2	6,02887	6,33E-20
RASSF2	6,019785	1,63E-08
NREP	6,019497	1,17E-30
TUBA3D	6,015121	2,85E-07
ARNT2	6,011031	9,00E-31
APBA1	6,007081	0,000201
EOMES	5,986797	4,38E-10
TMEM121	5,968884	5,20E-11
HPN	5,962563	6,94E-22
ST3GAL5	5,961133	1,21E-05
RIMS2	5,961052	0,000551
FAM81B	5,955839	1,96E-05
CRIP2	5,947829	2,08E-106
PCDHA10	5,9369	2,53E-18
TENM1	5,934966	4,42E-06
ITGA5	5,930507	1,93E-06
SEMA3D	5,921978	1,44E-05
PDE9A	5,921567	3,03E-07
KLB	5,909587	0,000616
TMEM37	5,898962	3,41E-23
SERTAD4	5,887991	1,90E-28
BCAT1	5,876122	5,20E-05
FAM92A1	5,867823	1,67E-10
EFCAB6	5,855288	0,000769
FZD1	5,848969	2,18E-37
DCDC2	5,846172	9,82E-24
TACSTD2	5,8362	3,35E-76
FES	5,829763	5,29E-05
TP73	5,802755	7,34E-09

NDRG2	5,799418	2,12E-19
TINCR	5,785377	1,19E-15
CPQ	5,763506	0,00102
DLGAP3	5,749514	5,32E-16
NRXN2	5,721699	1,83E-18
WNT5A	5,691117	4,07E-28
CACNA1H	5,683928	4,40E-43
ETNK2	5,670038	4,76E-29
COLCA1	5,669701	0,000182
RORA	5,665765	3,94E-33
FOXA1	5,664251	4,32E-98
FOXO6	5,642924	1,11E-08
RGS17	5,629693	8,75E-05
PCDHA7	5,628619	6,92E-10
RTN1	5,621732	5,62E-06
GPLD1	5,621103	5,47E-08
KCNN1	5,596852	0,000125
FBXO17	5,589271	1,85E-24
LINC00341	5,583743	0,000193
SPEG	5,555755	0,000307
KYNU	5,547324	5,64E-30
TNS1	5,536725	4,06E-14
ATP2A3	5,532488	2,76E-05
AMZ1	5,521297	2,58E-05
METTL7A	5,502656	8,14E-06
ATRNL1	5,499077	7,03E-05
ZNF329	5,495939	1,86E-21
TGM1	5,489321	5,05E-08
PBX1	5,486562	7,58E-28
PKIB	5,483303	6,99E-56
TMCC2	5,476731	1,01E-08
PCDHGA5	5,474994	0,000133
RASSF10	5,464319	1,52E-07
BMP7	5,464109	1,05E-46
C1orf115	5,449669	5,64E-16
SOX18	5,440319	8,38E-06
MRC2	5,437823	8,88E-06
PRKAR2B	5,435158	6,44E-21
PCDHGA4	5,406775	0,000158
TFF3	5,391887	1,68E-14
MAPK4	5,389758	3,92E-06
DKK1	5,387622	9,63E-19
LRRN1	5,386697	1,84E-08
TLX1NB	5,386204	0,000762
RUNDC3A	5,378221	7,04E-05
HOXC13	5,376712	5,82E-23
SOBP	5,360705	8,52E-06
RAB27B	5,347071	6,03E-21
EDN1	5,335994	1,80E-19
HECW2	5,318945	3,67E-18

BEND7	5,318238	5,28E-05
LOC90768	5,298414	3,49E-06
SALL2	5,287805	5,50E-11
SCN4A	5,283844	3,75E-11
TFAP2A	5,267704	1,21E-113
CNIH2	5,253875	5,34E-24
DIP2C	5,252018	3,70E-28
LINC01006	5,242588	4,93E-06
ZFP2	5,230917	0,000807
TOX2	5,230143	2,49E-09
LHX4	5,196891	1,93E-28
ZDBF2	5,165989	2,54E-08
LAMC3	5,139259	0,000676
ZNF563	5,131398	1,08E-05
ASAP3	5,113202	2,07E-21
TUBB4A	5,110985	3,20E-08
LOX	5,078282	4,45E-05
ODAM	5,077867	1,21E-18
PHLDB1	5,07498	6,43E-25
SYTL2	5,073312	2,16E-60
IGSF9	5,070851	6,66E-76
RDH16	5,068877	9,45E-21
ATP8B2	5,06563	2,49E-15
MRAS	5,05615	1,92E-10
TNFRSF18	5,02375	1,17E-05
GPC4	5,021648	1,06E-12
KLHDC7B	5,013342	1,62E-11
LINGO1	4,96926	3,58E-21
ZNF391	4,968052	1,10E-10
LYSMD2	4,963355	7,14E-40
CERS1	4,963319	0,000516
NTN1	4,955941	2,97E-09
ABCA3	4,951011	3,08E-27
FRMD6-AS:	4,935116	5,50E-05
ABCB4	4,928198	3,45E-05
SPATA17	4,927835	1,06E-16
LONRF2	4,927798	6,89E-23
REPS2	4,926057	2,10E-08
LRRC75B	4,924503	7,90E-08
TGFBR3L	4,921782	3,54E-09
LARP6	4,917192	8,19E-10
RNF165	4,913363	7,03E-05
CADM1	4,897241	1,14E-38
EPHA4	4,882977	6,57E-05
TP53	4,849375	1,99E-30
ADAM32	4,845566	0,000712
TUSC3	4,836625	8,30E-44
MAST1	4,833614	6,99E-15
WSCD1	4,826726	1,34E-15
SLCO3A1	4,794794	1,27E-08

TBC1D9	4,779957	1,17E-51
HID1	4,768699	3,67E-31
PRDM6	4,752531	8,62E-10
FAM46C	4,748671	2,97E-12
FEZ1	4,742223	8,85E-06
TET1	4,714883	1,47E-11
RASL11B	4,711811	2,47E-10
BMF	4,705077	5,35E-07
FARP1	4,6825	6,86E-22
RPRM	4,678249	4,14E-05
BCAS3	4,676764	4,61E-52
NXPH4	4,674437	1,32E-25
SOX2	4,664417	1,22E-17
ZNF362	4,638846	3,08E-31
ADGRB1	4,622913	2,23E-22
ALG1L	4,607292	1,02E-16
DPYSL5	4,602126	7,01E-17
DNALI1	4,599464	0,000186
FLVCR2	4,595563	3,43E-10
EWSAT1	4,594228	0,000931
MUC5B	4,592407	1,74E-10
KIF1A	4,589605	9,48E-31
SUSD3	4,587369	3,12E-11
OGFRL1	4,586969	1,53E-17
TCEAL3	4,585979	4,33E-24
FAM134B	4,585154	1,54E-10
ZNF185	4,575686	2,01E-23
SYT17	4,570557	3,42E-23
RUNDC3A-/	4,567833	1,67E-07
IRX5	4,565692	1,28E-29
ZNF836	4,561731	2,75E-14
LOXL2	4,548841	2,71E-05
XKRX	4,535316	0,000556
SLC22A23	4,519086	7,97E-61
SYNGR1	4,511685	1,85E-10
GLDC	4,494997	0,000723
RIMKLB	4,488087	6,13E-28
TMOD2	4,480643	7,50E-17
SPATA18	4,472045	3,10E-06
ZBTB10	4,466462	8,08E-39
FLRT3	4,462918	2,23E-27
NEO1	4,454664	5,41E-33
TUBA1A	4,454247	5,32E-65
NKAIN1	4,437615	9,09E-19
PTPRG-AS1	4,431666	9,77E-07
IGSF10	4,430641	0,000171
GPNMB	4,425842	1,28E-23
TIAM2	4,425809	0,000106
CEL	4,422717	3,58E-06
ENTPD1	4,386912	1,81E-08

ZNF461	4,363395	5,45E-08
CDH3	4,358288	4,84E-77
ZNF544	4,338758	2,28E-38
MT1F	4,328796	9,99E-07
SOX4	4,326201	2,78E-66
THNSL2	4,318546	1,44E-13
CIART	4,306328	5,42E-12
HLA-DQB1	4,304344	9,07E-07
EXO5	4,300858	3,50E-15
PACSIN3	4,300296	3,08E-44
NEB	4,297578	2,19E-15
C10orf82	4,296451	0,000234
TANC2	4,287206	4,70E-31
ZCCHC12	4,286018	2,47E-06
MDH1B	4,283069	5,92E-05
SYNE3	4,277814	9,86E-05
TRIB2	4,272892	1,82E-05
MAMLD1	4,267255	3,03E-05
ZNF137P	4,257916	6,83E-05
RGAG4	4,245061	3,77E-07
UCP2	4,24079	5,84E-15
LINC01132	4,228442	8,87E-05
RPS6KA2	4,223879	1,20E-08
KRT81	4,213545	3,36E-06
THSD4	4,203162	1,18E-17
SERPINA3	4,202073	0,000335
NOTCH3	4,196798	1,92E-56
PDGFRL	4,193585	5,50E-05
GRIP1	4,187697	7,69E-11
QPCT	4,182634	5,09E-05
AK4	4,17254	1,10E-37
GNG13	4,144234	1,56E-05
ARHGAP4	4,139802	1,26E-09
C1QL1	4,139549	0,00041
GLIS2	4,134539	1,14E-18
P2RX4	4,13289	7,14E-40
TAF15	4,126972	8,42E-15
HEATR6	4,1237	2,86E-66
LCA5L	4,12297	0,000595
LINC00992	4,1082	6,57E-07
NKX3-1	4,107863	5,54E-05
RBPMS2	4,092412	2,37E-08
DMC1	4,088597	0,000376
APPBP2	4,083429	9,16E-71
PFDN4	4,078863	4,55E-72
KRTAP5-AS	4,077875	1,63E-07
SLC26A4-A:	4,061913	5,08E-06
GLUD2	4,060401	2,96E-15
CMYA5	4,053345	0,000273
ALDH5A1	4,051948	1,15E-22

PROCA1	4,045179	0,000399
PRKCH	4,032917	8,75E-22
CCNG2	4,03043	5,37E-15
ZNF83	4,015756	8,39E-28
KREMEN2	4,010571	1,20E-29
BACE2	4,003597	4,77E-28
MID2	4,00207	4,98E-17
PPP2R3A	4,001163	3,08E-16
TUBB2B	4,00088	1,51E-15
LYPD3	4,000823	5,72E-12
TRIM37	3,992522	7,77E-78
MAP10	3,991093	0,000301
CCDC74B	3,990957	0,000132
NUP210	3,964997	5,14E-33
DNAH5	3,946467	0,000648
SIX4	3,941048	6,24E-17
ADAMTS19	3,93981	9,32E-05
DBN1	3,936569	1,97E-53
CENPF	3,930794	2,88E-20
TRAPPC6A	3,921714	1,07E-26
BCL11B	3,907685	3,90E-40
LRP3	3,903826	3,30E-27
ZNF419	3,901773	1,11E-09
TMEM254-	3,900988	1,55E-06
GATS	3,880031	4,10E-14
KNDC1	3,876653	4,77E-09
NCOA3	3,869672	2,02E-51
NODAL	3,864341	0,000436
CTXN1	3,86369	6,19E-22
PPP2R2C	3,860183	2,32E-51
LMCD1	3,858246	4,46E-05
PKIG	3,854807	3,66E-20
BRIP1	3,85372	1,66E-55
FAM213A	3,837256	1,37E-32
EMP2	3,836016	1,93E-22
PBXIP1	3,833958	2,24E-10
SHANK2	3,830778	8,68E-14
FAM69B	3,82742	4,32E-06
ACBD7	3,823734	7,02E-13
NUAK1	3,8205	1,17E-18
RASSF5	3,819554	8,31E-05
ZNF701	3,818534	2,83E-20
GCNT1	3,809994	2,08E-22
FGFR2	3,802247	0,000129
DHTKD1	3,800078	1,52E-45
NANOS1	3,799337	1,32E-21
KSR2	3,790668	1,37E-08
RBM24	3,781744	9,12E-07
USP32	3,776734	1,86E-31
SERHL2	3,776234	0,001004

HLA-DRB5	3,767918	0,000354
N4BP3	3,763073	3,80E-19
ARHGAP44	3,759288	3,10E-15
ZNF730	3,744287	9,07E-07
ZNF347	3,744054	3,87E-05
IFITM3	3,741253	1,93E-19
PPM1D	3,73826	2,48E-53
ZNF354A	3,736206	1,69E-26
OSR2	3,733517	7,53E-12
ATP9A	3,705801	6,35E-42
PGBD5	3,703144	6,28E-06
SECTM1	3,701678	2,97E-09
SIAH2	3,699057	4,17E-38
HCN2	3,691728	4,07E-14
TTLL7	3,690754	3,28E-06
RHOV	3,689775	1,26E-11
LTBP2	3,6888	0,000103
ZFYVE28	3,679165	1,00E-14
SMKR1	3,677918	7,73E-11
ZMIZ1	3,656902	1,09E-39
REEP2	3,652033	0,000532
PIAS3	3,635875	1,69E-46
PALM	3,633318	2,17E-12
ACACB	3,632531	2,56E-22
FAM46B	3,629309	4,78E-08
MVB12B	3,62543	3,97E-17
SPEF2	3,622356	4,68E-07
PLCG1-AS1	3,610758	0,000195
AIF1L	3,602814	1,71E-31
NGFRAP1	3,602228	2,17E-35
TTC25	3,58829	0,000172
BCAS4	3,581032	1,14E-13
BOLA1	3,571767	5,07E-21
TEAD2	3,567779	1,65E-23
KLRG2	3,564764	2,06E-06
KRBA2	3,564348	9,65E-05
HSD17B14	3,548252	6,72E-05
KRTAP3-1	3,544726	1,66E-07
PGM2L1	3,53513	5,82E-26
SEPT3	3,534524	7,76E-11
GMPR	3,534474	0,000149
TPBG	3,520187	9,57E-37
SEMA3C	3,516113	2,71E-56
SMAD9	3,515815	0,000181
SCIN	3,512597	0,000595
GAMT	3,512565	2,79E-10
STARD13	3,510438	2,76E-15
LYRM9	3,507442	7,29E-06
RND2	3,50726	3,80E-10
PGBD1	3,498906	1,64E-12

NRSN2	3,496312	3,18E-10
TSPEAR	3,491174	4,24E-06
NOVA1	3,48613	1,56E-07
SSBP2	3,485068	7,06E-07
NSUN7	3,477035	2,77E-07
CERS4	3,472483	9,79E-14
ZNF217	3,470225	1,80E-52
IGFBP4	3,464506	6,85E-14
CREB3L4	3,459205	1,62E-20
PRRT3	3,454306	2,41E-06
PLOD2	3,448753	1,51E-25
MARC2	3,44589	0,000123
FAM200A	3,439047	1,60E-13
CXCR4	3,4389	2,23E-09
AMOTL2	3,435307	1,45E-12
SRRM3	3,426154	0,000186
ESPN	3,422075	5,83E-10
C16orf74	3,421662	8,58E-06
CCDC74A	3,417418	6,05E-06
NRXN3	3,392851	6,87E-07
LOC10192[illegible]	3,391135	8,26E-06
MYH7B	3,390372	0,000749
SLAIN1	3,373638	1,14E-07
DNAJC6	3,371039	0,000144
P2RY2	3,369116	1,79E-22
TP53TG1	3,367419	1,45E-19
EFCAB11	3,363009	3,18E-15
HSPB1	3,361526	6,44E-37
COL5A2	3,360114	0,000288
DFNB31	3,35604	1,26E-15
H2AFY2	3,354626	2,16E-25
ARRB1	3,352901	3,12E-08
STARD10	3,348332	7,23E-16
PVRL4	3,34615	1,70E-20
MSRB2	3,333633	1,24E-29
MPP7	3,329544	5,20E-28
DYNC2H1	3,328349	2,81E-10
GADD45G	3,326015	1,83E-06
FAM102B	3,313243	8,60E-19
NEK11	3,308425	3,97E-08
IPO5P1	3,303694	7,39E-12
PRSS27	3,286459	0,000313
FIBCD1	3,275318	5,41E-06
GGT7	3,274343	2,23E-08
LMX1B	3,272542	6,03E-13
GSTA4	3,26758	3,01E-11
MMP11	3,25673	0,000178
PNRC1	3,255557	1,25E-20
MCAM	3,253561	5,08E-06
ISM1	3,249082	3,42E-06

GSTM4	3,248234	2,04E-13
LRRC37A8F	3,246597	0,000122
CNTNAP2	3,240128	3,71E-06
TOB2P1	3,236712	0,000687
SIX1	3,235503	3,40E-16
FXYD3	3,22392	3,03E-08
NR3C1	3,223591	1,38E-32
TMEM132A	3,223147	1,11E-21
BCL2	3,223014	4,45E-08
SCAMP5	3,213437	1,44E-11
DDB2	3,211523	1,78E-15
C1QTNF9B-	3,208989	8,04E-10
CA12	3,206875	2,16E-07
C1QTNF6	3,19642	0,000661
PCDHB14	3,192614	4,11E-15
PSMD6	3,192208	3,39E-38
ATP6V1C2	3,191885	1,43E-09
PNMA1	3,191298	1,85E-26
GLUL	3,188266	1,15E-42
CD82	3,187017	2,02E-12
RAB26	3,180629	2,19E-06
DYRK1B	3,179948	2,14E-22
SOCS3	3,17428	4,48E-07
RAD51C	3,172116	8,64E-45
ZNF420	3,169459	1,43E-09
HHAT	3,166945	8,43E-06
C1orf53	3,162992	1,05E-05
SMPDL3B	3,162871	5,93E-24
NPTXR	3,161754	1,23E-09
C15orf65	3,158533	9,81E-06
FZD3	3,154926	8,67E-16
SHANK1	3,138707	0,000975
ZNF160	3,13824	1,03E-20
APCDD1	3,133616	7,54E-20
SAMD11	3,132468	2,01E-06
CDKN1A	3,12997	3,35E-09
ANKRD34A	3,129885	3,22E-05
WNT11	3,114251	7,01E-06
B3GALNT1	3,114232	4,48E-13
ISYNA1	3,113702	9,33E-34
FCMR	3,11322	0,000132
TXNIP	3,112104	3,12E-07
ZNF253	3,106338	1,59E-21
DTX3	3,103245	1,51E-17
ATP5G2	3,100831	4,99E-44
TDRKH	3,098938	1,25E-14
KRT7	3,097467	1,32E-05
H1F0	3,093938	1,08E-10
ZNF17	3,086943	9,20E-07
DHRS2	3,084721	9,76E-15

CASC10	3,079259	5,45E-06
RARG	3,075468	5,57E-21
FGD1	3,070552	1,08E-17
NDRG4	3,066473	2,74E-07
STARD5	3,056778	3,84E-05
RASD1	3,055219	0,000491
PCOLCE	3,048287	9,55E-08
LYPD6B	3,046301	0,000146
PYROXD2	3,044247	3,15E-06
REEP1	3,039725	7,22E-05
FAM214A	3,036617	3,06E-08
FAM227B	3,027737	2,04E-09
ZNF22	3,027504	1,56E-09
ADA	3,014384	1,29E-08
MYLIP	3,012435	4,30E-19
HBP1	3,007389	3,19E-11
BTG2	3,006228	2,77E-11
XYLT1	3,002159	3,76E-08
GAB1	3,001647	1,51E-11
ERV3-1	2,995173	1,05E-15
RBBP8NL	2,990356	1,83E-12
ZNRF1	2,984354	5,87E-23
DUXAP8	2,984337	7,70E-09
PBLD	2,976091	1,75E-08
GPX8	2,97533	3,72E-15
CBLB	2,972157	6,67E-20
TUBD1	2,971367	3,67E-20
ZNF561-AS	2,968359	4,73E-06
GRHL3	2,966025	5,50E-09
ZNF416	2,962409	6,42E-07
MTERF1	2,962204	1,82E-09
RHOB	2,960606	5,07E-21
ZSCAN16	2,956792	3,05E-17
ASAH2	2,954174	8,57E-12
MDK	2,947095	1,55E-36
WNT7B	2,946124	5,19E-10
ZNF493	2,941468	0,000226
CAP2	2,94117	4,43E-16
TEX9	2,940523	9,83E-08
KDM5B	2,936557	3,09E-20
PLD1	2,935034	0,000153
DDAH2	2,934175	1,95E-22
PTP4A3	2,931072	4,18E-10
PCBP3	2,90827	1,92E-06
PKP1	2,906117	5,38E-06
EIF4E3	2,905793	5,41E-09
ZBTB12	2,904559	4,78E-08
SYNE2	2,903208	3,71E-21
ZFP14	2,894019	6,43E-08
PPAP2A	2,893362	1,03E-10

CACNB3	2,884591	6,24E-23
FAM174B	2,883962	6,49E-09
CHD6	2,881746	8,01E-29
MATN2	2,88107	2,11E-13
GALNT6	2,877504	5,44E-14
PITPNC1	2,874877	2,00E-31
PTGES	2,874375	3,72E-06
CCDC170	2,868838	1,95E-06
ARMCX6	2,866482	8,45E-07
CD24	2,860954	4,93E-23
TTC7B	2,860696	3,13E-09
UTRN	2,860351	2,71E-08
FTCDNL1	2,85735	0,000472
ARHGEF28	2,854524	4,38E-12
TRIM24	2,848741	1,73E-30
WASF3	2,847842	2,68E-12
DNAH3	2,846155	0,000609
ARSG	2,845817	7,58E-10
ZNF816	2,841899	4,09E-09
FERMT2	2,838863	3,69E-14
SLC4A3	2,834216	0,000781
CRNDE	2,832147	8,43E-08
ZNF134	2,83106	9,76E-12
HOMER2	2,828664	1,50E-14
C2orf76	2,816085	1,92E-07
ZNF821	2,815238	1,54E-07
SOX13	2,808846	1,08E-12
BLVRA	2,808435	2,72E-19
FAXC	2,805654	9,16E-05
CRISPLD2	2,805106	4,04E-07
MAN2B1	2,802596	3,80E-22
GSTZ1	2,792411	3,28E-20
PARD6G	2,792119	5,77E-05
TCF7L1	2,791079	2,58E-06
CDYL2	2,787374	6,04E-06
PDE3B	2,787257	2,16E-07
IDH2	2,787106	6,55E-19
HK1	2,786604	8,75E-13
LRRC73	2,77915	9,86E-06
FBXO27	2,779073	4,62E-10
SIPA1L2	2,77562	7,58E-17
WWTR1	2,769239	8,47E-12
FAM129A	2,768904	4,70E-06
PDK3	2,766403	2,92E-08
NAAA	2,764698	2,14E-12
ZSCAN2	2,762822	1,79E-19
SOCS1	2,76062	8,99E-09
HES2	2,756902	0,000365
ADIRF	2,749415	2,05E-06
ZNF211	2,740162	1,24E-05

C20orf194	2,739144	9,53E-05
TUBB6	2,735056	5,92E-20
SLC9A6	2,734531	9,83E-08
SLC25A29	2,732661	6,09E-16
PRSS23	2,729069	7,25E-17
ZNF467	2,726141	2,93E-21
RPS6KB1	2,725349	1,48E-32
FAM212B	2,718456	6,59E-09
CCT6B	2,715586	0,000402
WDR83OS	2,715547	1,17E-28
PARD6B	2,715358	2,85E-19
PYCARD	2,714908	2,48E-09
SEMA7A	2,713161	8,26E-11
RAB13	2,696139	1,45E-18
MAP3K12	2,692694	1,52E-05
ECI2	2,679857	3,54E-09
RAB36	2,678356	0,000223
SBK1	2,675387	6,18E-18
RBM43	2,666837	8,15E-05
FGFR1	2,665442	3,87E-09
ZNF808	2,665439	1,54E-07
GNAS	2,654304	1,33E-33
PCSK6	2,651624	3,15E-14
BCL6	2,643224	4,05E-12
PCDHB9	2,642044	9,23E-07
TCTN1	2,639291	7,17E-14
LITAF	2,635207	1,87E-15
FGD3	2,631148	2,10E-06
BCAS2	2,628694	2,13E-32
IFT22	2,627567	2,04E-18
SHOX2	2,62008	0,000507
LINC00674	2,616771	6,32E-19
BCORL1	2,614606	2,52E-11
ZNF219	2,613443	4,48E-16
TSPAN31	2,612744	1,02E-08
SNHG5	2,612563	1,11E-06
SELENBP1	2,611229	1,90E-10
ARHGEF37	2,611034	0,000154
B4GALNT1	2,607819	2,05E-07
ZNF254	2,606751	1,45E-14
SLC29A4	2,603275	2,28E-07
RNF122	2,583732	9,20E-05
YBX2	2,581425	2,98E-15
GPSM1	2,566609	8,74E-08
NHP2	2,564659	3,18E-32
ULK1	2,559608	8,53E-21
ENDOV	2,55769	5,62E-13
TMEM191A	2,556919	2,43E-10
ZNF749	2,554531	1,17E-05
KRT18	2,552295	1,33E-09

CBX2	2,551435	3,34E-23
TTC28	2,543847	0,000117
RAB3D	2,53657	3,65E-15
SNTA1	2,535813	1,04E-06
CYB561	2,532358	2,15E-30
ARHGEF25	2,530634	0,000275
ZSWIM4	2,52912	1,30E-07
SNX24	2,529106	5,42E-10
DHRS13	2,528563	1,10E-10
ITPKB	2,525349	4,62E-10
ZNF818P	2,520988	1,03E-05
COL18A1	2,516747	6,90E-08
LINC00886	2,515911	6,63E-05
ZNF417	2,515303	5,68E-06
ABAT	2,507121	0,000156
INPP4B	2,501784	1,80E-11
METRN	2,500346	8,49E-15
MT2A	2,499964	6,78E-11
GATSL2	2,496212	6,46E-16
DNLZ	2,486373	2,48E-16
ZNF713	2,484095	9,42E-06
ZNF776	2,483155	5,23E-10
ZNF805	2,482935	1,36E-07
RIMKLA	2,482478	0,000121
LTBP1	2,48068	5,24E-20
USP30	2,480328	6,28E-08
ZNF117	2,466058	1,46E-05
MYT1	2,46501	0,000258
HNRNPA1L	2,464782	5,17E-07
SDSL	2,463112	1,06E-08
ZBTB42	2,461828	6,36E-12
CSDE1	2,454135	1,86E-18
C11orf80	2,451352	2,01E-09
DHPS	2,450524	1,99E-16
NFATC4	2,445749	0,000141
ALDH4A1	2,440316	8,20E-06
ITPR2	2,438	2,35E-11
ARHGDIG	2,432534	5,75E-06
KRT8	2,431907	7,21E-10
CCDC176	2,429598	0,000336
DNPH1	2,425328	1,77E-23
PTK7	2,421709	1,60E-10
CASKIN1	2,411922	2,33E-08
SLC27A3	2,404896	3,93E-17
TMEM183E	2,40258	3,20E-08
ZNF431	2,399969	6,32E-05
CAMK2N2	2,399208	1,18E-06
WDYHV1	2,391546	1,38E-11
PTPRS	2,391068	3,56E-15
DNMT3B	2,387811	2,13E-16

DISP1	2,386692	2,92E-07
FAM117A	2,38522	3,96E-08
MEX3A	2,377623	4,28E-24
WDR83	2,375892	1,93E-14
DLC1	2,374266	2,69E-09
PRMT6	2,365528	9,44E-23
DTWD1	2,365255	9,69E-11
IDNK	2,363868	3,19E-07
TLE1	2,362141	1,47E-11
HMG20B	2,359172	2,96E-26
PLXNB3	2,356226	9,47E-08
LGALS1	2,349806	6,60E-11
TNNT1	2,348936	2,66E-18
NR4A1	2,347358	6,31E-06
UST	2,346484	9,10E-05
ZFHX3	2,34641	8,60E-13
ZBTB22	2,345303	6,46E-10
PRDX2	2,34525	9,93E-22
SLC4A8	2,337022	0,000958
NPDC1	2,336853	3,05E-24
GABPB1	2,334416	1,25E-14
FBXW9	2,333834	9,86E-15
FYTTD1	2,331897	1,04E-16
DTD1	2,329833	3,75E-17
GAA	2,325095	2,36E-14
MXD4	2,324049	4,88E-06
ZNF627	2,323765	1,10E-13
HKR1	2,320513	1,02E-10
MFSD3	2,313093	3,19E-17
PLEKHF2	2,312427	2,35E-17
MXD3	2,308563	5,82E-09
RPS27L	2,308343	2,48E-05
HDAC5	2,307794	1,88E-05
MPP2	2,304446	0,000728
CTNNBIP1	2,301807	8,13E-12
CIR1	2,301055	3,15E-10
TRERF1	2,299908	6,24E-08
ZNF429	2,298158	3,45E-10
SLC35E3	2,292109	2,34E-11
SRD5A3	2,291593	1,14E-09
STON2	2,284443	7,96E-13
CSRP2	2,28367	1,64E-06
WDR91	2,279584	8,64E-06
DNAJC15	2,279567	1,84E-17
ALDH7A1	2,27936	5,73E-18
MOSPD3	2,277415	1,46E-07
ILDR1	2,276773	1,06E-08
GKAP1	2,270517	0,000226
ZNF250	2,262772	3,56E-06
NME4	2,253647	8,24E-20

RNF130	2,248504	9,11E-13
SH3BP5	2,240427	1,86E-13
SH3BGR	2,238998	0,000137
FDXR	2,237613	2,38E-05
EPN3	2,23577	4,35E-12
MAPK8IP1	2,232994	1,79E-06
CKAP4	2,232416	3,36E-12
POGZ	2,228105	4,61E-13
NDST1	2,227985	4,49E-16
LAMTOR4	2,22004	2,73E-15
ZNF260	2,215268	1,60E-13
GJA3	2,210487	7,03E-05
SAMD15	2,207832	0,000736
FAM127A	2,207774	8,35E-08
LYPLAL1	2,199205	1,87E-06
RAB27A	2,198021	2,24E-09
ANAPC15	2,197509	8,51E-16
RHOD	2,191067	6,98E-06
EFNA1	2,188957	4,49E-06
ASS1	2,187336	9,86E-08
VPS72	2,185589	1,79E-11
GSE1	2,185574	1,75E-23
GTF2IRD2B	2,185307	7,41E-09
PMF1	2,181226	4,13E-07
FBXL16	2,181189	2,39E-07
SLC29A3	2,180647	8,56E-07
KLHL24	2,179288	0,000479
MFAP2	2,167298	2,16E-05
PEX11B	2,153761	9,70E-13
DCLRE1B	2,15372	1,11E-13
SRGAP2B	2,153058	2,21E-05
ZNF490	2,151701	6,76E-06
RHPN1	2,151224	2,13E-05
ISOC2	2,151222	3,33E-18
LOC10496[illegible]	2,144513	4,31E-05
THOC7	2,143429	1,02E-20
ZNF552	2,142129	2,74E-06
FUT8	2,137003	2,06E-18
SLC25A43	2,136744	3,43E-11
SPIRE1	2,133833	1,09E-12
HIGD2A	2,133229	1,11E-17
F2R	2,132232	5,15E-05
FLYWCH2	2,131743	3,66E-12
FAM86B1	2,131722	0,000234
PLXND1	2,13164	1,10E-06
HOMER3	2,130591	2,42E-13
OS9	2,129536	2,15E-18
SLC37A4	2,122622	4,42E-17
GPR161	2,121128	0,00013
THBS3	2,120819	0,000546

EPB41L4A-,	2,118958	2,70E-06
C6orf48	2,118355	1,75E-06
SPPL2A	2,115339	2,49E-17
ZNF738	2,109887	5,20E-05
COX6C	2,09558	2,21E-11
PUS10	2,086103	0,000217
LRFN1	2,082239	6,40E-06
LRRC26	2,080896	1,40E-05
CLU	2,079642	5,49E-07
BCAM	2,077868	3,98E-12
KRT10	2,075739	2,82E-09
PDGFA	2,075346	2,15E-08
ISL2	2,072901	0,000584
PLCG1	2,072271	8,39E-18
CCDC24	2,070105	5,88E-05
ENSA	2,067002	3,40E-22
TMEM187	2,065908	6,05E-06
CUL9	2,065166	8,32E-07
ESCO1	2,062777	3,78E-05
ARG2	2,061054	1,74E-05
ARVCF	2,055878	4,55E-10
ZNF8	2,050378	6,05E-07
PCDHB13	2,048269	1,16E-06
NARF	2,045654	4,68E-07
PXN-AS1	2,045486	8,11E-06
S100A13	2,038286	6,76E-12
PDCD4	2,032306	3,45E-12
IFT46	2,030156	1,95E-13
CCZ1B	2,030033	7,67E-14
CCPG1	2,029847	1,41E-06
CADM4	2,025767	2,24E-07
PHPT1	2,02538	1,32E-15
BBC3	2,023078	5,20E-05
ZNF32	2,022669	5,13E-08
NRSN2-AS1	2,022614	8,45E-06
MYO5A	2,021996	5,06E-10
LRRC49	2,021718	2,44E-05
PRSS8	2,020649	7,00E-18
HMGB3	2,017863	6,09E-18
LY6E	2,016406	2,99E-09
ZCWPW1	2,014113	0,000557
RNF115	2,014	1,28E-14
PIK3R3	2,013701	2,21E-08
ASNA1	2,01055	1,09E-18
SLC43A2	2,007973	3,55E-09
ZNF468	2,006876	8,19E-11
BFSP1	2,005436	0,000529
KCTD1	2,000728	4,21E-09
ACOX3	1,9977	2,49E-07
TMEM64	1,997377	4,90E-15

RPS6KL1	1,996785	1,35E-05
CELSR2	1,995212	2,99E-13
DAAM1	1,988142	1,31E-12
TEAD3	1,9826	5,69E-10
TJP3	1,979691	9,07E-06
PPP2R5E	1,974249	3,74E-17
RPS2	1,97079	8,11E-16
VPS9D1	1,970183	6,92E-05
HRAS	1,967106	1,35E-08
MRPS21	1,959055	2,79E-14
KLHDC2	1,958773	4,06E-08
CCDC167	1,958528	6,35E-06
YPEL5	1,958386	6,42E-05
COPG2	1,958028	1,10E-10
ZNF428	1,949948	5,78E-07
XBP1	1,946981	6,90E-06
PPP1R21	1,945156	8,92E-07
ITFG2	1,944346	6,66E-06
ZNF771	1,938278	1,10E-05
SFXN2	1,928422	1,20E-08
CHMP6	1,927689	1,63E-07
TRIQK	1,927487	2,33E-07
RNF44	1,92263	1,80E-12
RGS12	1,921546	4,56E-09
ARID2	1,919605	6,62E-12
ANK3	1,91607	2,22E-06
FAM13B	1,910682	9,10E-05
SOS2	1,910009	1,30E-09
LRRC27	1,908426	0,000221
PPOX	1,907633	0,000688
RPL22L1	1,905042	1,59E-06
SRGAP2	1,903865	1,30E-11
ZNF512B	1,903693	6,55E-10
C21orf33	1,901426	3,41E-09
ZNF525	1,900837	2,38E-07
KCTD15	1,900239	1,48E-05
ATXN3	1,899224	4,70E-08
PLXDC2	1,892896	1,69E-07
RRBP1	1,892411	2,79E-08
TBCK	1,891562	2,07E-09
BHLHB9	1,890292	0,000381
DSP	1,889995	6,30E-12
BCYRN1	1,882195	8,94E-08
ZNF669	1,881574	3,21E-05
NEBL	1,880728	1,12E-08
PLCH1	1,878498	9,57E-11
OARD1	1,878241	5,50E-09
STMN1	1,877624	7,39E-08
RNASEL	1,871787	2,39E-06
NRAS	1,87171	1,70E-13

THOC6	1,871195	3,04E-07
ZNF766	1,870138	5,70E-12
SEMA4C	1,868815	1,18E-08
PELI3	1,865626	6,57E-08
MLLT3	1,864453	0,000186
PAIP2	1,863248	4,18E-10
RPL13	1,861926	1,98E-10
C14orf80	1,858738	2,14E-09
TM7SF2	1,857057	6,66E-05
ATF6B	1,854086	2,94E-08
LINC01296	1,853758	0,000186
SP140L	1,85289	0,000175
SYNE4	1,852068	2,03E-10
DRAM1	1,848992	2,66E-06
PVT1	1,842361	1,09E-06
ATP2B4	1,83648	2,63E-05
TMEM183/	1,830298	1,18E-12
SLC27A5	1,829656	4,38E-08
ZNF608	1,827736	1,31E-07
GALNT18	1,827388	0,000938
BTG1	1,825917	1,49E-10
RGS10	1,823068	2,49E-05
TCTN2	1,822854	3,04E-06
VTI1B	1,822588	2,20E-09
ADAM11	1,822478	6,94E-05
ANXA6	1,822149	0,000186
TRIM36	1,819036	2,98E-06
HILPDA	1,815788	3,03E-07
RMND5B	1,814768	2,18E-13
HEXDC	1,814297	4,04E-06
ABCA2	1,812458	5,24E-09
DST	1,811883	4,25E-09
LSM11	1,80746	6,22E-06
MRPL41	1,805504	4,62E-10
CCDC71L	1,800447	6,06E-05
ZNF425	1,799261	9,29E-06
IQCK	1,795836	1,11E-06
DTNB	1,793332	7,72E-08
FBXO31	1,792741	2,07E-11
TCF25	1,791742	1,48E-14
ZSCAN21	1,790628	2,53E-09
LOC11323(	1,787731	1,47E-06
HMGN1	1,784359	2,24E-13
LRRC23	1,780944	0,000504
RPSAP58	1,780536	1,03E-11
APRT	1,776202	6,47E-13
FBXW4	1,769278	3,07E-07
C2orf68	1,763822	3,14E-09
FRG1BP	1,761233	0,000121
FAM89B	1,760789	4,57E-11

CYP2J2	1,760427	0,000116
POLR2J	1,759221	1,97E-10
SYDE2	1,75916	0,000136
SF3B4	1,757877	5,31E-13
SLC44A2	1,755179	1,57E-09
ZSCAN26	1,754245	8,04E-05
ZNF444	1,754199	2,74E-08
ATP11B	1,750227	0,000196
ZNF611	1,74641	3,68E-06
ZNF212	1,743672	1,93E-07
HSD17B8	1,74344	7,23E-05
VPS50	1,736904	1,25E-05
COBLL1	1,736375	3,77E-07
ZNF197	1,734832	1,30E-06
FAH	1,731429	3,45E-09
ZNF616	1,730874	8,65E-09
IQCH-AS1	1,729956	2,18E-05
HCFC1R1	1,729864	1,92E-05
TMEM9	1,729673	5,54E-11
PPDPF	1,727185	2,26E-06
TARS2	1,720409	1,15E-09
NSMCE4A	1,719778	8,82E-07
RRM2B	1,718841	6,27E-05
AP5S1	1,717811	1,93E-05
NSMCE2	1,716573	7,48E-09
TSPAN17	1,7159	4,60E-09
JHDM1D-A	1,715483	9,40E-05
ZNF16	1,715479	0,000361
GAS5	1,715253	0,000993
LAPTM4B	1,712006	1,78E-14
SETDB1	1,711099	5,64E-11
ZKSCAN4	1,708236	0,000167
NFIB	1,708034	6,98E-09
AP4E1	1,707147	1,68E-08
TRIM45	1,703681	0,000151
ALDH6A1	1,700447	1,90E-10
TSTD1	1,695855	4,12E-08
ENO2	1,695636	0,000325
HIPK1	1,695251	1,45E-12
SLC2A4RG	1,693645	1,29E-07
C7orf60	1,692841	0,000118
TGIF2	1,691702	1,82E-05
TNRC6C	1,69157	3,62E-10
ZNF687	1,691319	8,17E-08
C2orf15	1,689401	1,89E-06
SSH3	1,689077	4,93E-06
SIVA1	1,687504	6,09E-09
FNBP1	1,687103	3,45E-10
CHD3	1,686402	8,90E-12
TRIM52-AS	1,683916	0,000203

DNAJC4	1,680037	1,05E-05
PPL	1,677764	3,61E-05
TAMM41	1,677247	1,27E-07
KIF13A	1,674381	3,45E-09
MDM4	1,67343	2,29E-06
PKN1	1,672815	2,21E-06
RPS18	1,67184	1,47E-07
VPS52	1,671525	7,72E-12
UBE2Q2	1,670509	1,08E-07
WWP1	1,670227	1,03E-05
ZNF888	1,669097	1,59E-05
APPL2	1,668611	1,85E-06
GCSH	1,666286	1,74E-10
CCDC92	1,665442	1,98E-05
SNRPE	1,66493	1,52E-12
FAM127B	1,664172	7,91E-12
TRIM33	1,663756	2,63E-11
ILVBL	1,663161	5,06E-10
EPHB3	1,663109	0,000458
TTC26	1,659232	0,000179
CYTH2	1,657679	2,08E-11
POLD4	1,657366	0,000289
CD47	1,657148	1,12E-08
FKBP10	1,656699	4,84E-13
ENY2	1,652711	5,57E-07
NR2F2	1,650378	7,80E-10
KLF7	1,649707	1,27E-05
ZNF184	1,649363	4,04E-08
SUV420H2	1,648323	4,49E-05
POLR3C	1,637918	2,86E-05
IFT43	1,637577	7,14E-07
BNIP3	1,636473	4,02E-09
KCNC3	1,635683	1,82E-05
ZNF585A	1,635372	0,000329
FAM63A	1,633655	0,000873
LHPP	1,63274	2,54E-05
SERTAD3	1,631672	1,95E-05
LPIN3	1,630516	0,000528
HIST2H2BE	1,629812	0,000696
ZNF385A	1,62946	1,21E-08
VPS45	1,627482	8,47E-09
TMEM231	1,624393	3,89E-06
MAP4K4	1,622147	8,10E-13
MMAB	1,621735	5,73E-11
MORN2	1,618728	0,000836
ICAM3	1,618701	0,000166
CHCHD5	1,618269	4,51E-08
ZDHHC24	1,618035	9,26E-05
ZNF138	1,616292	4,85E-06
PDE4A	1,613151	0,000596

NUDT4	1,608888	4,21E-08
RPL30	1,607811	1,66E-07
ZNF252P	1,605916	6,29E-10
TMPO-AS1	1,605726	0,000211
ADARB1	1,605392	0,000146
ZNF35	1,605304	0,00054
NCK2	1,602623	2,79E-09
TUFT1	1,601587	2,49E-07
FBXW8	1,601468	5,80E-08
ZNF480	1,600252	2,36E-07
METTL6	1,599946	1,81E-05
PDE4DIP	1,59939	2,72E-05
ZNF791	1,59824	7,35E-05
AUTS2	1,5979	4,48E-07
IQSEC1	1,597762	3,57E-09
CDKN2C	1,593458	7,27E-06
ZNF503	1,593412	2,23E-09
DGCR6L	1,591504	9,94E-05
DNAAF3	1,591157	2,47E-06
PPCDC	1,590243	2,03E-05
RBM8A	1,589993	1,55E-11
IMPDH1	1,589471	2,05E-07
AGGF1	1,589414	1,28E-08
RINT1	1,589318	2,41E-06
C11orf49	1,588763	1,97E-06
NUCKS1	1,588139	2,46E-12
ERGIC1	1,587585	4,73E-08
TGIF1	1,584061	1,81E-06
TMEM223	1,583357	1,50E-07
ZNF607	1,58245	3,26E-06
INPPL1	1,581604	6,67E-11
MREG	1,579406	1,44E-07
DENND2D	1,579321	5,95E-08
MZT2B	1,579227	3,33E-08
ALG13	1,578646	7,49E-06
NDUFB7	1,577205	1,41E-11
ZNF268	1,576304	7,64E-07
ATPAF1	1,571215	2,21E-08
EAPP	1,57074	5,28E-07
TLE3	1,569167	4,11E-09
SUPT4H1	1,569126	2,68E-10
PACS2	1,567865	1,53E-08
H1FX	1,567129	9,78E-06
SMDT1	1,563098	2,58E-05
RALGPS2	1,555213	5,91E-07
EIF2D	1,554245	7,42E-09
ZNF140	1,553575	3,98E-06
DECR1	1,553376	4,71E-09
IER5	1,550956	1,35E-06
TATDN1	1,550668	3,67E-09

PATZ1	1,549405	1,59E-06
FLOT2	1,549217	1,11E-05
GRHL2	1,547815	9,10E-07
DDT	1,54601	1,95E-06
KIF20A	1,545637	1,17E-08
ZNF84	1,544425	1,54E-07
MDM2	1,544323	9,78E-05
TMOD3	1,543956	3,26E-12
EGFL7	1,542492	9,91E-05
HOOK2	1,542218	7,24E-10
QKI	1,540506	1,77E-05
MTA3	1,531114	1,23E-08
FAM172A	1,529776	0,000146
CIRBP	1,529711	2,26E-05
ITGB5	1,526796	0,000215
SOGA1	1,524433	2,21E-05
NXT2	1,52022	0,000282
ZFP1	1,518543	1,47E-06
RMI2	1,516928	6,58E-06
AGAP2-AS1	1,516677	0,000238
MKS1	1,514153	6,76E-06
LTBP3	1,513772	3,24E-06
PIGT	1,513063	7,68E-08
RPS27	1,511235	8,32E-06
SEMA3F	1,509582	0,000147
RPL10	1,508294	5,08E-07
RB1	1,506447	9,18E-06
CCDC101	1,503267	1,73E-05
PSMG3-AS:	1,502988	0,000726
ZNF688	1,501035	0,000247
ATMIN	1,499076	5,47E-10
PIGB	1,496509	7,74E-06
MBD5	1,495115	0,000485
CHD1L	1,493187	1,07E-06
IDS	1,492168	0,000111
STRADA	1,491904	8,84E-06
FAM53B	1,490848	2,51E-08
ATXN7L1	1,49042	2,15E-05
RPS3	1,488144	2,46E-08
GOLPH3L	1,486702	0,000103
SLC50A1	1,484887	5,96E-07
EXPH5	1,484548	4,94E-05
DSTYK	1,484371	1,19E-07
TRPM7	1,483521	7,52E-07
MNAT1	1,482497	8,38E-06
PTPRU	1,481423	4,98E-06
C17orf62	1,480933	1,02E-08
RPL8	1,477112	2,81E-11
COMMD7	1,475739	7,43E-05
DCXR	1,475402	1,68E-06

IFT172	1,475007	0,000362
TMEM205	1,473983	6,55E-06
RNF5	1,473318	1,47E-05
NMT2	1,470933	0,000275
DYNLL2	1,469821	8,77E-10
FAM210B	1,469108	9,05E-05
PQLC3	1,46882	0,000415
RABIF	1,467708	3,02E-07
APH1A	1,465736	1,62E-10
CCDC149	1,465607	0,000752
SPECC1	1,465127	5,05E-06
PRDM4	1,464477	6,05E-11
RBM4	1,463687	6,52E-08
PCNX	1,46018	6,49E-09
SEZ6L2	1,458527	0,000143
SERPINH1	1,457958	1,31E-09
PRUNE	1,457716	2,19E-07
TLCD1	1,457356	7,72E-05
KIAA1549	1,457118	2,09E-05
TMEM254	1,454475	3,25E-06
GCDH	1,454327	6,10E-08
MTCH2	1,453655	2,74E-06
CACFD1	1,450899	0,000292
RUNX2	1,450193	2,01E-06
LEF1	1,449869	6,29E-06
PRR3	1,446715	0,00021
TCEAL4	1,444742	3,20E-08
RNF139	1,442579	2,87E-08
RPL18A	1,441439	7,53E-07
MCM7	1,437198	1,35E-08
LEO1	1,436833	3,05E-10
NOL3	1,434785	2,48E-06
RING1	1,43447	2,41E-07
DANCR	1,434155	3,86E-06
LRRC37BP1	1,433776	0,000824
PLCB1	1,433723	0,000222
OXCT1	1,4328	6,77E-05
C9orf3	1,432595	7,03E-05
RAD23A	1,432022	5,98E-08
GALNT12	1,430254	0,000496
RPL35A	1,43021	5,49E-07
TIGD6	1,426887	0,000642
SAMD1	1,425865	1,34E-05
C9orf152	1,425171	0,000827
TSPAN13	1,423793	7,33E-06
TEF	1,423332	0,000686
PAIP2B	1,422978	0,000214
ARL8A	1,422619	2,50E-08
GABARAPL	1,419485	0,000547
PPM1A	1,418827	4,20E-05

NAT8L	1,416697	0,000472
ZNF286A	1,415199	3,80E-07
NDUFB9	1,411086	2,03E-07
C19orf43	1,410989	8,86E-09
SMARCD2	1,410104	3,07E-10
FAM3A	1,409069	0,000105
RAB24	1,407237	0,000117
CINP	1,407209	0,000458
BMPR2	1,404865	3,68E-06
NRP1	1,404057	0,000948
ACSF3	1,402345	9,00E-08
UQCRC2	1,399481	2,10E-07
TPD52	1,398235	2,83E-08
TMEM14C	1,397306	5,96E-07
ATP5A1	1,396185	5,68E-11
MACROD1	1,396011	5,76E-06
ARHGEF19	1,395058	0,00014
ECSIT	1,394509	1,83E-05
SNX2	1,39414	7,39E-06
NCOA2	1,393739	0,000122
LPAR2	1,392595	8,68E-07
MTSS1L	1,39102	2,50E-05
PHLPP1	1,388919	1,32E-07
SPTBN2	1,386815	0,000129
CTDSP2	1,386125	9,36E-06
ZNF510	1,385913	0,000105
ZNF778	1,384027	1,68E-05
OLFM1	1,382628	3,00E-05
ZNF12	1,381722	7,74E-07
ZNF675	1,381188	8,30E-05
ZNF45	1,380098	0,000516
ATXN7L3B	1,377835	3,53E-10
PACS1	1,37749	7,32E-05
ARNT	1,377149	2,15E-08
DSTN	1,376171	1,49E-05
ZKSCAN1	1,373294	7,82E-09
CCDC57	1,372851	0,000352
DCP1B	1,372766	0,000469
COX4I1	1,370566	1,37E-10
ATP6V0E2	1,367477	0,000478
SRPK2	1,367321	2,97E-08
MRPL11	1,36504	3,18E-08
MAPK6	1,36498	4,86E-09
RPS19	1,363615	1,99E-07
BCL9	1,362792	4,26E-06
NDUFA3	1,361349	3,92E-06
KDELC2	1,36126	8,96E-05
RALGPS1	1,360926	0,000228
RXRA	1,359932	5,06E-05
ZFYVE21	1,359374	1,21E-05

TPD52L1	1,358863	0,000146
SLC9A3R1	1,358633	2,22E-05
RPL32	1,357517	1,51E-07
CRTC1	1,35702	0,000464
RBM4B	1,356539	0,00038
TIMM9	1,356282	0,000223
TSEN15	1,35496	0,000417
TSPAN6	1,353887	1,96E-05
KIAA0101	1,352818	1,92E-06
NUDT3	1,35253	0,000404
PMS2P1	1,351186	0,000119
FUNDC2	1,349091	5,46E-07
EPB41L1	1,348514	4,77E-06
TMC4	1,348307	0,000181
TAOK3	1,348302	8,11E-07
GNB5	1,345252	7,33E-05
S100A10	1,343723	3,05E-07
ZNF282	1,34348	1,51E-06
POLR2K	1,341302	0,000253
NDUFS8	1,339111	2,04E-06
PRCP	1,337171	1,65E-05
MB21D2	1,336735	0,00055
COA5	1,336125	3,03E-05
BANF1	1,336029	4,16E-07
FAM86C1	1,334131	7,42E-05
ZNF765	1,332645	0,000663
GLB1	1,331738	1,09E-05
IFT57	1,331002	4,10E-05
MED13L	1,330162	2,58E-06
ROGDI	1,329984	0,000463
DGKZ	1,328694	1,35E-05
BOD1	1,326473	3,06E-05
CFAP36	1,326465	2,06E-05
RPS14	1,325966	1,37E-07
CAMSAP1	1,325871	1,52E-08
NADSYN1	1,325354	2,50E-05
HARS2	1,324254	2,14E-07
NIPSNAP1	1,323328	9,13E-07
CARHSP1	1,323116	0,000127
JTB	1,320032	1,17E-06
JUND	1,317769	0,000806
ZNF3	1,317759	2,42E-06
ZMAT2	1,317303	1,11E-06
PDCL3	1,317159	3,83E-06
CUL7	1,316893	2,26E-05
UBTD2	1,31652	8,47E-07
SEC11A	1,315265	4,25E-09
COX5B	1,314794	9,05E-09
RPL12	1,313706	1,61E-06
COQ2	1,313262	1,24E-05

PPP1R13B	1,312476	2,87E-05
MYL12B	1,309321	4,30E-09
AP4B1	1,308023	7,87E-05
ATP5I	1,306405	4,18E-06
CETN2	1,305042	1,87E-06
RECQL5	1,304665	0,000461
SNAPIN	1,302362	6,91E-05
ZNF100	1,301981	0,000319
RNF181	1,301779	9,64E-06
ZSWIM7	1,300495	0,000248
RNASEH2A	1,300062	4,88E-06
TRAK2	1,299338	1,13E-06
GLTSCR2	1,299203	2,90E-05
EHMT2	1,297797	1,52E-06
AHR	1,297529	5,66E-08
SSNA1	1,296208	9,31E-05
TAF11	1,295703	2,74E-06
HMBS	1,295412	2,22E-05
NUDT14	1,290271	2,59E-05
SOS1	1,289928	3,08E-07
TAF3	1,2885	2,05E-05
KAT5	1,28827	3,58E-06
EEF1A2	1,287066	0,00039
ASB8	1,28632	0,000944
COMTD1	1,286297	0,000131
GRB14	1,285789	0,000221
OAZ2	1,285703	1,66E-05
SUMO3	1,284756	3,48E-08
TMEM65	1,283998	0,000459
CCDC34	1,28287	0,000163
GPR89A	1,282523	0,00042
ZNF777	1,280927	0,001014
ZNF845	1,277636	0,000372
JARID2	1,275837	3,40E-07
FLJ10038	1,275307	0,000224
SQLE	1,274916	5,53E-06
ARL6IP4	1,274406	1,95E-07
ASCC2	1,273193	3,21E-06
KLHDC3	1,272871	9,48E-05
SIRT5	1,270147	5,68E-05
MRPS28	1,266984	0,000189
SAYSD1	1,264972	1,14E-05
AKT1	1,264573	2,50E-05
SMIM7	1,264023	1,66E-06
RPS25	1,263699	6,75E-06
CTPS2	1,262593	3,27E-06
TK1	1,260526	0,000101
COX6A1	1,259894	1,46E-08
EIF2AK3	1,257893	0,000531
NDUFA2	1,257548	1,49E-05

SLC9A3R2	1,257462	9,95E-05
KLHDC4	1,256603	1,86E-06
NSA2	1,249954	3,26E-07
USP8	1,249603	4,32E-08
PPIC	1,245906	1,88E-05
EPHB4	1,24351	3,15E-07
IMPACT	1,239786	4,20E-05
EBAG9	1,235105	9,31E-05
VKORC1	1,232874	0,000196
PAAF1	1,232799	2,15E-05
FAM110A	1,232218	0,00026
ABHD16A	1,229957	7,61E-06
SUV420H1	1,22993	1,40E-06
RPS29	1,226514	7,67E-05
RPL21P28	1,225594	0,000273
UQCC3	1,225289	2,21E-05
C10orf76	1,223965	0,000319
TRAF4	1,223268	3,14E-06
TTC9	1,22325	0,000194
C14orf2	1,222446	1,99E-05
TOP2B	1,221189	9,61E-08
ZNF579	1,220045	1,92E-05
UQCC2	1,218958	0,000201
TACO1	1,217451	9,36E-05
ALKBH7	1,217427	2,49E-05
MED21	1,216717	7,44E-05
THG1L	1,216631	1,75E-05
MTIF3	1,216222	9,89E-05
ALKBH4	1,214689	0,000168
ANKRD50	1,214297	9,54E-07
RPL28	1,21287	4,31E-06
MYO5C	1,212673	0,000219
ZNF768	1,212357	3,77E-06
FAM83H-A	1,210512	8,57E-06
DLG1	1,209466	2,13E-06
ZNF107	1,207865	0,000705
SLC25A39	1,20728	2,27E-07
TMEM168	1,207083	0,000127
DBNDD1	1,205695	0,000604
COX14	1,20568	0,000257
FAF2	1,205563	9,45E-07
PRMT2	1,205526	0,0003
VPS28	1,205052	9,50E-06
RAE1	1,204445	5,14E-06
PPP1R35	1,201896	0,000516
CBX1	1,201702	2,77E-06
PRKD3	1,201199	0,000875
ARL2	1,20106	0,000277
PRPSAP1	1,197767	2,46E-05
CBS	1,197307	0,000191

INTS2	1,19634	0,000128
GNB2L1	1,195884	6,74E-06
ARF3	1,194338	8,43E-06
HLTF	1,19368	3,65E-05
SCAMP1	1,191661	7,84E-05
SSBP3	1,191594	0,00036
RPRD2	1,19123	8,28E-06
EFNA4	1,191214	7,48E-05
FZD6	1,189761	0,000241
RPS15A	1,188854	9,07E-06
CUTA	1,188696	2,37E-06
YIPF3	1,18531	1,78E-06
HR	1,185286	0,000351
DTWD2	1,184316	0,000734
C7orf73	1,183978	7,26E-06
TMEM263	1,183779	0,000926
C16orf58	1,182054	3,20E-06
TMEM63B	1,180684	0,00061
CRBN	1,179926	0,000874
ATP5L	1,179059	1,41E-06
RPLP1	1,178268	0,000392
MEPCE	1,176403	0,00016
SIKE1	1,171814	0,000335
PSMD4	1,170339	7,31E-07
IGF1R	1,169959	1,61E-05
OTUD7B	1,169801	0,000242
LMAN2	1,169538	2,70E-05
SARS2	1,16692	0,000128
RPS17	1,165063	7,24E-05
NT5DC2	1,16347	3,40E-05
GALK1	1,1633	0,000417
PSMB4	1,163063	3,57E-06
MAP2K5	1,163008	0,0001
GZF1	1,162069	0,000385
TMEM258	1,162015	0,000111
KARS	1,161552	1,08E-05
TMBIM4	1,16103	0,000148
MRPS18B	1,160657	8,07E-06
SLC29A2	1,160302	0,000631
MTX1	1,158474	0,000221
CTCF	1,157234	6,87E-05
GDE1	1,155616	0,000138
SLC25A24	1,154875	7,03E-05
MIF	1,151847	1,34E-06
SNX18	1,151753	0,000929
C19orf60	1,151344	0,000224
RSBN1	1,15014	0,000466
PIK3C2B	1,150078	0,000845
MRPL27	1,146683	3,58E-05
TRMT12	1,146182	0,000196

BCKDHA	1,145522	0,000336
PRPF3	1,14493	0,000646
RPL4	1,144273	3,60E-06
DBI	1,144017	0,00013
RGL2	1,143174	5,72E-05
TRPM4	1,142546	0,000847
DNMT3A	1,142199	1,22E-05
PTTG1IP	1,141425	5,04E-07
SSR2	1,1411	6,02E-05
RPL37A	1,13728	0,000132
NSD1	1,136545	4,87E-05
IDH3G	1,136499	4,71E-06
CNOT2	1,136386	7,77E-06
AP5M1	1,135589	0,000235
B4GALT7	1,133072	0,000298
XPOT	1,129973	9,03E-05
WRB	1,128811	0,000755
NDUFA13	1,125375	1,69E-05
SERPINB6	1,123678	3,04E-06
ATAD2	1,123307	0,000572
ARPP19	1,121754	9,59E-07
ECI1	1,121547	3,67E-05
NUDT16L1	1,121143	0,000291
AP4M1	1,118412	0,000282
BDH1	1,118175	4,81E-05
MYO6	1,117935	7,49E-06
TMEM14B	1,116317	0,0004
CMC2	1,115425	0,000145
PIGU	1,114556	0,000105
SIPA1L1	1,114199	7,02E-06
RHNO1	1,114125	0,000995
DLGAP5	1,112358	1,98E-05
VIPAS39	1,112201	0,000204
CREB1	1,110187	3,88E-05
PPP2R1A	1,11017	0,00037
CDK2AP1	1,106424	0,000425
ZNF205	1,105976	0,000432
RPL31	1,105414	1,88E-05
TBC1D5	1,105028	3,24E-05
SCD	1,104844	0,000559
RPLP0	1,101273	1,29E-05
MRPL24	1,099867	0,000158
RTFDC1	1,099354	0,000199
MTMR4	1,099344	8,75E-06
REEP5	1,098903	0,000919
LAMA5	1,097799	3,05E-05
ZMYND11	1,094308	9,35E-06
MRPL9	1,093738	0,000189
MYL12A	1,092993	1,01E-05
SUCLG2	1,092139	0,000399

DMXL2	1,089154	2,13E-05
GLRX5	1,089153	6,33E-05
CCNI	1,088774	8,28E-05
RMDN1	1,08592	0,000111
C9orf64	1,085803	0,000607
ATP13A1	1,085537	1,94E-05
SEC22B	1,085164	0,000172
TM7SF3	1,084991	0,000416
SART1	1,084442	7,31E-05
DDX41	1,083899	5,48E-06
RPS23	1,083577	0,000138
BCL7C	1,082235	9,33E-05
ATP5D	1,080279	0,000243
ZKSCAN8	1,078585	0,000211
STUB1	1,076117	2,26E-05
TRMT112	1,075663	0,00012
PFKL	1,074194	0,000277
ZNF561	1,074005	0,000336
ZFP62	1,07313	2,31E-05
RPRD1A	1,072582	1,22E-05
YIPF5	1,071163	0,000191
PYCRL	1,070946	0,000925
ATP5H	1,070372	6,09E-05
CUX1	1,069014	4,18E-05
NCS1	1,06864	6,53E-05
RPL26L1	1,067776	8,67E-05
CSRNP2	1,067608	6,11E-05
STK38	1,066805	0,000129
ECD	1,066735	0,000156
ISCU	1,064714	0,000438
NBN	1,061605	3,57E-06
RPL13A	1,061085	0,000145
SHFM1	1,054002	8,33E-06
CNOT8	1,053832	0,00017
AP2S1	1,05373	4,45E-05
TAF6	1,053485	0,000151
VMP1	1,052211	0,000125
DYM	1,051617	8,89E-05
DARS2	1,050279	2,27E-05
ERBB3	1,049769	8,91E-06
HNRNPH3	1,049654	0,000918
TKFC	1,049239	8,61E-05
COX7C	1,049056	0,000138
MRPS34	1,048854	6,90E-05
STX6	1,048551	0,000155
KLHL36	1,047918	0,00013
SCAF11	1,046306	4,30E-05
TRIAP1	1,045857	0,000758
RPL37	1,043718	0,000281
TRIP6	1,043471	3,36E-05

EPB41L5	1,042732	8,90E-05
MYL6	1,039907	2,12E-06
GPX4	1,038686	0,000189
FIS1	1,038179	9,57E-05
MAZ	1,037029	0,000278
SYT7	1,035101	0,000305
ATRAID	1,033007	0,000241
LNX2	1,032511	7,45E-05
CELSR1	1,032494	0,000241
PRKAG1	1,029978	0,000167
ERGIC3	1,02802	5,54E-05
DAZAP2	1,026149	0,000614
PPP1R37	1,024715	0,00016
MTMR3	1,021826	0,000104
PSMD7	1,021624	1,89E-05
MAPKAPK2	1,020537	0,000197
CLCC1	1,020488	0,000427
PPIG	1,017798	0,000546
TMEM5	1,017566	0,000203
LLPH	1,017492	0,000102
C5orf30	1,016942	0,000284
ANKIB1	1,016853	4,68E-05
DDX6	1,014834	2,10E-05
JAGN1	1,013604	0,000306
USP6NL	1,013537	0,000261
ADAR	1,013266	8,53E-05
LDB1	1,01296	4,37E-05
CBLL1	1,011326	0,000287
BCAP29	1,011129	0,000784
ARF5	1,008666	5,94E-05
CCDC124	1,00752	0,000516
BCAP31	1,006858	0,000996
NDUFA1	1,006299	0,000758
TOP1	1,00556	7,11E-06
PI4KB	1,005225	0,000218
TMEM251	1,002075	0,0009
BRK1	1,000331	5,65E-05
TET2	1,00032	0,000861
SIRT1	0,999291	0,000925
ARID5B	0,999214	0,000666
RANBP9	0,998716	0,000198
ZNF395	0,997365	0,000809
CCDC90B	0,997024	0,000551
ANAPC16	0,995985	7,00E-05
NDUFA12	0,992438	0,000994
ARMC10	0,992373	0,000212
CCND1	0,992067	0,000928
NCBP2	0,991072	0,00012
SCYL2	0,990839	8,16E-05
CASP2	0,98981	0,00012

SNX27	0,989243	7,73E-05
WDR73	0,988617	0,000491
PWP1	0,988436	2,71E-05
VPS9D1-AS	0,988257	0,000855
XXYLT1	0,987506	0,000528
MCL1	0,987363	1,47E-05
CCSER2	0,986459	0,000393
NELFCD	0,985325	0,000261
CFL1	0,984245	0,000168
HNRNPA0	0,983309	0,000158
ADAT1	0,9828	0,000194
PRKACA	0,978225	0,000639
MPHOSPH9	0,977433	0,000414
RNF114	0,976174	7,63E-05
LAMB2	0,973888	0,000992
PCCB	0,973307	4,53E-05
ESRP2	0,972157	0,000324
PRR11	0,972111	0,000123
SOX12	0,9705	0,001005
SLC35C2	0,967043	0,000735
DNASE2	0,965876	0,000177
ADI1	0,96521	0,00026
RBM38	0,960775	0,0005
HSP90AA1	0,960542	0,000946
F12	0,959039	0,000432
RPL41	0,958777	0,000164
AURKA	0,95771	0,000345
COX6B1	0,956655	0,000323
TBC1D30	0,956442	0,000275
ARFGAP2	0,956334	0,000372
THNSL1	0,954383	0,000738
C19orf53	0,951059	0,000151
RPL35	0,948409	6,63E-05
STX17	0,947849	0,000947
SNRPC	0,947623	3,34E-05
PTOV1	0,94344	0,000368
CIAO1	0,940882	0,000721
CNOT6	0,940309	0,000362
PAQR4	0,937091	0,000117
PSMC3	0,935808	0,000629
RPL18	0,932102	0,000226
ZMYM2	0,931651	0,000259
PPP1R14B	0,931454	0,000786
ZNF33A	0,9156	0,000719
CRTAP	0,915109	5,62E-05
EIF4B	0,914825	0,000147
AAAS	0,910153	0,000483
SEL1L	0,909299	0,000111
RPS11	0,90844	0,000901
NDUFB2	0,90659	0,000822

SNRPF	0,902676	0,000266
CENPN	0,901107	0,000391
PPP4C	0,898712	0,000353
CDC42SE1	0,89486	0,000876
EEF1D	0,892502	0,000243
C14orf166	0,887791	0,000117
COPE	0,88728	0,000368
BPTF	0,886972	0,000125
NAP1L1	0,883449	0,000412
FAHD1	0,883323	0,00074
RFWD2	0,881221	0,000576
ZMYND8	0,878116	0,000709
PABPC1	0,877179	0,000197
OST4	0,876047	0,000902
PEBP1	0,875026	0,000168
TET3	0,865746	0,000766
IMP4	0,862398	0,000376
CPNE3	0,860237	0,000736
ATP5C1	0,860121	0,000459
PTPN18	0,859713	0,000923
RPL15	0,858116	0,000341
RPL7A	0,849546	0,000105
URI1	0,848833	0,000607
CTNNA1	0,843021	0,000334
RPS13	0,842025	0,000324
NGRN	0,834346	0,000861
RPSA	0,826937	0,000443
H3F3B	0,816439	0,000351
ILF2	0,80409	0,000385
SEC63	-0,79323	0,000796
PRPF40A	-0,79846	0,000493
NSUN2	-0,7985	0,000794
PARP1	-0,81044	0,000554
COLGALT1	-0,81258	0,000746
ANXA2	-0,81683	0,000241
CNPY2	-0,84093	0,000665
LTBR	-0,84164	0,000438
NCL	-0,84846	0,000348
TNPO1	-0,86057	0,00034
RPA2	-0,86293	0,000866
TCP1	-0,86484	0,00016
EIF4E	-0,8657	0,000353
CDC123	-0,86692	0,000463
SKIV2L2	-0,86804	0,000271
NASP	-0,86962	0,000703
RER1	-0,87483	0,000484
MPHOSPH8	-0,87668	0,000271
PUM1	-0,87737	0,00045
MED1	-0,87979	0,000219
ABR	-0,87986	0,000694

CHMP7	-0,8815	0,000724
EPS8	-0,88236	0,00072
ELF1	-0,88255	0,000541
LAMP1	-0,89247	0,000764
USP16	-0,8931	0,000993
NAA15	-0,8936	0,000127
MGAT2	-0,90074	0,000696
RRM2	-0,90085	0,000137
RANBP2	-0,90088	0,0003
MSL1	-0,90197	0,000577
TLK1	-0,90503	0,00026
TMEM123	-0,90582	0,000121
UBN1	-0,90663	0,000466
IDH3A	-0,90829	0,000139
MIB1	-0,90865	0,00016
ST13	-0,90884	0,00016
WDR12	-0,90984	0,000371
TMEM33	-0,91043	0,000414
ARFGEF2	-0,91382	0,000148
ATXN10	-0,9139	0,000162
WNK1	-0,91458	0,000561
PPP1R8	-0,91624	0,000392
GAPVD1	-0,91755	0,000182
MOB1A	-0,91756	0,001005
HBS1L	-0,91816	0,000293
CTPS1	-0,91862	0,000328
PSMA1	-0,9195	0,000455
PHRF1	-0,91962	0,000287
MAP3K2	-0,92422	0,00041
NCLN	-0,92468	0,000466
SDHB	-0,92739	0,000422
HSPA13	-0,92805	0,00065
ADAM17	-0,93025	0,00079
AKR1A1	-0,93085	0,000312
TMEM259	-0,93114	0,000259
TMEM248	-0,93129	0,000227
RCC1	-0,93143	0,000103
ACBD3	-0,93241	0,000414
SH3D19	-0,93384	0,000942
TAF12	-0,93487	0,000735
CD2AP	-0,93621	0,00031
TMEM230	-0,93778	0,000377
RNF111	-0,93829	0,000696
DIMT1	-0,93899	0,000297
RBM28	-0,93905	0,000229
SON	-0,94394	0,000295
AKAP11	-0,94573	6,99E-05
C21orf59	-0,94772	0,000447
GOLM1	-0,95057	0,000584
EXOSC2	-0,95087	0,000154

UBAC2	-0,95106	0,000696
CAPZA1	-0,95172	0,000107
COPS2	-0,9534	0,000266
YKT6	-0,9586	0,000156
CLEC16A	-0,95907	0,000782
COPS8	-0,95985	0,000793
SLC6A6	-0,96071	0,000107
PIM3	-0,96079	0,000816
BNIP2	-0,9615	0,000982
CSNK2A1	-0,96445	4,42E-05
NSRP1	-0,96486	0,00065
SIRT7	-0,96515	0,000471
USP48	-0,96569	0,000268
ATP13A3	-0,96728	0,00069
CMTM6	-0,96746	0,000261
TYW1	-0,97127	0,000566
WDR43	-0,97218	0,000482
IPO7	-0,97332	2,34E-05
UBQLN2	-0,97353	0,000361
STRAP	-0,97366	0,000164
AGAP1	-0,9737	0,000925
MAGT1	-0,97395	0,000156
UBQLN1	-0,97475	3,18E-05
DDX55	-0,97624	0,000525
RNGTT	-0,97764	0,001016
OFD1	-0,97828	0,000376
GIGYF2	-0,97841	0,000128
ITSN1	-0,97854	0,000403
STK25	-0,98033	9,17E-05
HDAC1	-0,98134	3,98E-05
IPO9	-0,98137	0,000358
PPIP5K2	-0,98337	0,000342
FHOD1	-0,985	0,00045
ANKRD26	-0,98534	0,000518
GINS4	-0,98717	0,000489
ERI1	-0,98743	0,000695
SSX2IP	-0,98912	0,000681
JMJD1C	-0,98955	0,000535
LRFN4	-0,98973	0,000185
PTPN11	-0,99033	1,45E-05
SMIM12	-0,99042	0,000241
SLC7A6	-0,99142	0,000599
TPP1	-0,99228	0,000738
FAM49B	-0,99432	5,99E-05
LRRFIP1	-0,99618	4,46E-05
AMD1	-0,99689	0,000605
TXNDC12	-1,00011	5,69E-05
NOP9	-1,00164	0,000281
GARS	-1,00317	8,20E-05
ZDHHC5	-1,00325	8,01E-05

NAA25	-1,00362	8,14E-05
NUS1	-1,00485	0,000236
RDX	-1,00934	0,000508
ICE2	-1,0098	0,000111
ATXN2	-1,00984	8,32E-05
ADSS	-1,01044	0,000381
CDC6	-1,01294	0,000489
SEPT7	-1,0132	0,000132
ABHD5	-1,01373	0,000794
CNP	-1,01428	0,000702
ACD	-1,01529	0,000243
PLEKHA6	-1,01601	0,000256
RSPRY1	-1,01603	0,000108
TBRG4	-1,01614	0,000854
PDCD11	-1,01874	0,000334
WBP11	-1,01972	1,17E-05
PEX5	-1,02143	0,000222
HSPH1	-1,02158	3,01E-05
RPF2	-1,02403	0,000352
PICK1	-1,02491	0,000479
SNRNP40	-1,02646	0,000192
MAP2K1	-1,02766	7,55E-05
DESI1	-1,02904	0,000155
BCCIP	-1,0301	4,73E-05
RAB10	-1,03029	4,61E-06
NOLC1	-1,03214	2,29E-05
FAM111B	-1,03389	0,000235
HSPA14	-1,03407	1,80E-05
APEX2	-1,03475	0,000295
RAPGEF6	-1,03719	0,000221
UBR4	-1,03823	0,000631
MCMBP	-1,03854	3,79E-05
DNAJC30	-1,0402	0,000317
ADO	-1,04108	0,000736
SHROOM3	-1,04131	0,000396
MFAP1	-1,04158	0,000651
ERLIN2	-1,04245	8,38E-05
ENTPD4	-1,04356	0,000422
COPS7B	-1,04377	0,00029
NME6	-1,04652	0,000561
EIF4G3	-1,04698	9,03E-06
GTF2H1	-1,05041	5,26E-05
RLF	-1,05122	0,000172
DHX30	-1,05124	4,37E-05
HELLS	-1,05172	0,000222
REXO1	-1,05177	8,24E-05
PDIA3	-1,05198	3,31E-06
SPG7	-1,05235	0,000202
ATP2B1	-1,0527	4,65E-05
BLOC1S6	-1,05297	0,00017

SPRED2	-1,05329	7,84E-05
UHRF1	-1,05352	0,000899
EIF3D	-1,05723	0,0001
MARS2	-1,05748	0,000401
INTS7	-1,0598	0,000239
APTX	-1,06446	0,000335
HIPK3	-1,06493	0,000165
MPRIP	-1,06579	5,56E-05
DCAKD	-1,06708	0,00087
PGM2	-1,0679	0,000118
BROX	-1,06967	2,77E-05
NUFIP2	-1,06993	7,14E-06
COASY	-1,07294	2,21E-05
KIAA0368	-1,07379	5,87E-05
LPGAT1	-1,07439	0,000151
TFAM	-1,07532	1,08E-05
FAM155B	-1,07745	0,000855
ABHD17B	-1,07976	0,00069
MAP7	-1,08181	2,24E-05
FAM111A	-1,08214	0,000134
RNF216P1	-1,08595	0,00094
CUL5	-1,08805	8,43E-05
ARHGAP11	-1,08819	3,74E-05
PLEKHG2	-1,08848	0,000167
EPT1	-1,08863	0,000104
ZNFX1	-1,09156	0,000951
VPS37A	-1,09164	6,20E-05
MAGOH	-1,09236	0,000128
CHEK1	-1,09241	1,40E-05
PROSC	-1,09379	6,82E-05
TMEM2	-1,09548	0,000271
NOTCH1	-1,09562	0,000133
DESI2	-1,09576	4,80E-05
PSMD1	-1,09676	0,000269
GLYR1	-1,09983	5,06E-05
ST7	-1,10218	0,000947
NUDT19	-1,10249	0,000366
PPTC7	-1,10252	0,000273
PCNA	-1,10259	1,85E-06
ERMP1	-1,10286	0,000551
STXBP5	-1,10568	0,000917
CEP170	-1,10641	8,14E-05
AP2B1	-1,10685	1,51E-05
DOK4	-1,10692	0,000205
TMF1	-1,10737	0,000365
MGA	-1,10772	6,29E-05
ACBD5	-1,10784	0,000453
YARS	-1,10942	0,000157
STK24	-1,11129	4,09E-06
ZNF367	-1,11181	0,000246

EMC3-AS1	-1,11242	0,000286
LACTB2	-1,11336	0,000891
SLC4A7	-1,11632	0,000549
INTS10	-1,11785	0,000126
CHAF1A	-1,11858	8,14E-05
LMNB2	-1,11913	0,00062
EPS15	-1,12044	5,08E-05
CASC5	-1,12083	2,63E-05
MAFK	-1,12117	7,34E-05
GFM1	-1,1214	5,14E-06
PARVB	-1,1218	0,000328
CYSTM1	-1,12293	0,000854
TRABD	-1,12361	3,65E-06
AASDHPPT	-1,12443	0,000109
MANF	-1,12683	2,36E-06
HAUS5	-1,12699	0,000373
PPP2CB	-1,12707	3,79E-05
PRPS1	-1,12796	0,000218
MPP6	-1,12834	0,000203
CMTM4	-1,12844	1,34E-06
PLP2	-1,12879	1,18E-05
NDC1	-1,13031	1,38E-06
EBLN3	-1,13135	0,000278
SHCBP1	-1,13206	2,68E-05
MLX	-1,13237	2,12E-06
SBDSP1	-1,13307	9,41E-05
CBY1	-1,13417	0,000329
PRKAA1	-1,13426	0,000663
DNAJA1	-1,13537	0,000237
LONP2	-1,13597	1,01E-05
ITPR3	-1,13599	4,54E-05
M6PR	-1,13616	7,78E-05
PPIP5K1	-1,138	0,000151
GNA11	-1,13824	7,80E-06
HIATL1	-1,13932	0,000191
SBNO2	-1,1398	1,95E-05
SNX14	-1,14031	4,01E-05
PITPNB	-1,14111	3,17E-05
TBC1D20	-1,14134	3,06E-05
CNDP2	-1,14237	4,56E-05
HCCS	-1,14476	0,000346
ITGB1	-1,14669	0,000154
PPP2R2A	-1,14901	2,65E-06
NDUFAF4	-1,15024	0,000201
AK2	-1,15144	3,20E-07
PYCR2	-1,15168	0,000921
GTPBP1	-1,15198	0,000226
TOPBP1	-1,15237	2,78E-06
FBXO41	-1,15249	0,000373
PSMB2	-1,15249	3,47E-07

TMEM51	-1,1542	0,000305
RPAP1	-1,15591	2,52E-05
HERC2	-1,1561	2,83E-06
WDR36	-1,15705	0,000108
DCUN1D5	-1,15809	9,38E-06
CTNS	-1,15819	0,000224
MALSU1	-1,1589	0,000948
ABCC1	-1,16182	3,99E-06
EMC1	-1,16223	1,25E-06
SERTAD2	-1,16239	1,22E-05
CAPZA2	-1,16336	0,000125
MICA	-1,16483	0,000375
BTAF1	-1,16555	0,000156
MRPS30	-1,16602	2,62E-05
NCKAP1	-1,16632	1,24E-06
MCPH1	-1,16644	7,40E-05
PGM3	-1,16836	0,00067
CAP1	-1,16933	9,72E-06
ARHGEF7	-1,16958	0,000369
ZMPSTE24	-1,17015	4,14E-05
TLN1	-1,17097	0,000329
SLC41A1	-1,17288	4,55E-05
FUCA2	-1,17336	0,000221
PPFIBP1	-1,17371	7,22E-06
SPEN	-1,17581	0,00011
DTX2	-1,17607	0,000401
MELK	-1,17808	8,69E-06
ORC1	-1,17847	6,68E-05
PTPRK	-1,17952	2,29E-06
NUDT15	-1,17969	0,000185
CCBL2	-1,18151	6,23E-05
FAM210A	-1,18151	0,000856
GPD2	-1,18267	1,13E-05
SKP2	-1,18285	1,00E-06
ZFP36	-1,18329	0,000193
DTL	-1,18399	1,93E-05
PDXP	-1,18477	0,000738
POLK	-1,18522	0,000432
MLXIP	-1,1855	1,25E-06
APP	-1,18555	5,60E-07
ARID1B	-1,18641	3,96E-05
IKBKAP	-1,18714	8,71E-07
CCM2	-1,18775	0,000218
REL	-1,18932	8,80E-05
L3MBTL2	-1,18966	3,50E-05
TTLL12	-1,19317	2,59E-06
GCLC	-1,19475	1,22E-05
KANK1	-1,19544	0,000239
PAFAH1B2	-1,1963	2,31E-05
UPF3A	-1,19676	0,000151

CORO1C	-1,19875	6,53E-07
CPPED1	-1,19881	0,000159
PTER	-1,19936	4,88E-05
SORBS3	-1,20038	0,000802
MALT1	-1,20105	7,61E-06
MIER2	-1,20111	0,000271
STK38L	-1,20305	1,63E-05
ELOVL6	-1,20518	0,000437
EXOSC9	-1,20597	9,69E-06
LRCH1	-1,20809	1,05E-05
UCHL3	-1,20974	0,00062
MAD2L1	-1,21056	3,26E-06
ABCD3	-1,21205	6,59E-06
PELO	-1,21222	1,74E-05
KATNBL1	-1,21283	0,000352
LRRC42	-1,21403	1,04E-05
KIAA0232	-1,21479	4,94E-06
AKIRIN1	-1,21624	1,88E-06
TBC1D14	-1,21657	1,56E-07
FOXRED2	-1,21788	0,000614
NETO2	-1,21882	1,38E-07
SPATA5L1	-1,21887	5,61E-05
PANK4	-1,21939	0,00019
SNTB1	-1,22168	2,85E-05
CHAF1B	-1,22181	3,04E-05
HEXB	-1,22182	3,29E-05
AKIRIN2	-1,22271	0,000234
CCT8	-1,2232	5,16E-08
LRRC47	-1,22356	4,56E-05
NUP50	-1,22436	2,39E-06
LNPEP	-1,22462	0,000331
ARHGEF39	-1,22773	0,000671
GEMIN4	-1,22911	2,96E-05
UBE3A	-1,22965	2,62E-07
TOMM34	-1,23038	6,11E-07
USP40	-1,23198	2,36E-05
PAQR8	-1,23388	0,000983
GXYLT1	-1,23602	1,66E-05
ZNF532	-1,23614	0,000651
SECISBP2L	-1,23678	0,000752
URB1	-1,23787	5,84E-07
MAK16	-1,23877	0,000455
UTP11L	-1,24239	2,79E-06
TWF2	-1,24311	0,000163
C12orf4	-1,24438	0,000282
ARHGEF18	-1,24511	1,74E-05
KAT6A	-1,24569	3,20E-05
FTO	-1,24637	9,41E-06
RAB3GAP2	-1,24714	9,20E-06
FNDC3B	-1,24739	5,41E-07

TTLL4	-1,24783	2,27E-05
LHFPL2	-1,2489	1,11E-05
ADRBK2	-1,24974	3,08E-05
NUP155	-1,2505	5,97E-06
NUPL1	-1,25332	2,12E-05
DHDDS	-1,25334	0,000614
LPCAT1	-1,25348	8,35E-07
SPATS2L	-1,25378	3,38E-06
IRF6	-1,2539	8,94E-05
FAAP20	-1,25489	1,95E-05
TMEM59	-1,25788	0,000155
ICMT	-1,25931	1,28E-06
BAP1	-1,25952	3,42E-06
EZR	-1,25986	0,000124
CYB5R3	-1,26033	6,06E-05
CLDN4	-1,26125	0,000173
DNA2	-1,26171	8,34E-05
TOM1L2	-1,26207	0,000298
NIPA1	-1,2629	2,29E-05
TAF5L	-1,26392	1,60E-06
POR	-1,26461	2,55E-05
SNX9	-1,26531	5,21E-06
ADIPOR2	-1,26631	7,87E-08
EYA3	-1,26736	2,16E-05
DCAF12	-1,26776	1,20E-06
MLKL	-1,26842	3,49E-05
DVL1	-1,26964	1,89E-06
MARCKS	-1,27056	1,31E-06
RBBP8	-1,27083	8,29E-05
APPL1	-1,2714	1,67E-07
SAMD8	-1,27197	0,000428
GART	-1,27299	3,37E-08
ARHGAP18	-1,2734	0,000145
ST6GALNA(	-1,27352	0,000276
CBR1	-1,27463	1,93E-05
DIEXF	-1,27474	0,000291
SNAPC3	-1,27535	0,000418
AKIP1	-1,27686	0,000139
LTV1	-1,27692	0,000538
EPB41	-1,27806	1,20E-05
MTF1	-1,27827	1,17E-05
ILKAP	-1,27835	0,000203
ATP1B1	-1,27967	8,45E-07
PEX14	-1,27987	2,81E-05
SLC30A1	-1,28011	0,000102
NXN	-1,28046	0,000209
SF3A3	-1,28178	6,41E-05
TMUB1	-1,28193	1,62E-06
RGP1	-1,28269	0,000264
MAPKAPK3	-1,28443	3,96E-05

MKLN1	-1,28459	8,89E-08
STARD3NL	-1,285	0,000601
PRPF4	-1,28599	1,00E-07
GPATCH11	-1,28648	0,000973
SDC1	-1,28864	6,98E-07
KCTD9	-1,29038	7,01E-06
SLC26A6	-1,29072	6,85E-06
PDPR	-1,29102	1,22E-06
COA7	-1,29155	0,000124
FAM122A	-1,29183	9,95E-05
MED4	-1,29202	8,34E-05
TOMM40L	-1,29226	0,000102
SLC35A2	-1,29244	4,46E-06
SPTY2D1	-1,29436	2,11E-05
BRI3BP	-1,29517	3,10E-05
PPIL4	-1,29613	1,99E-06
JOSD1	-1,29673	7,70E-08
ago-03	-1,29956	0,000376
TRAK1	-1,3	2,46E-06
ATP6V1B2	-1,30003	1,64E-07
MAP2K3	-1,30027	0,000348
SLBP	-1,30041	6,24E-07
SPTBN1	-1,30203	9,92E-09
ZYX	-1,30308	1,88E-05
EDRF1	-1,30404	4,22E-05
NUMBL	-1,30405	2,84E-05
KDM4C	-1,30625	0,000234
NECAP1	-1,30641	1,11E-06
PTPN14	-1,30662	0,000743
CCT6A	-1,30668	3,59E-09
DDI2	-1,3081	0,000702
GOSR1	-1,3098	1,28E-06
LDHA	-1,30993	5,54E-08
TEAD1	-1,31015	1,67E-06
STK17B	-1,31079	0,000519
PKP2	-1,31137	0,000855
ROCK2	-1,31138	7,69E-07
CALU	-1,31161	5,66E-08
ACADVL	-1,3122	0,000437
SEC23A	-1,31279	9,55E-06
DNTTIP2	-1,31311	2,85E-07
ATP13A2	-1,31438	0,000147
LRRC8B	-1,31586	3,21E-06
WDFY1	-1,31619	3,59E-07
NF2	-1,31632	3,76E-05
TBPL1	-1,31716	9,41E-05
SMCR8	-1,31947	6,41E-08
MESDC1	-1,3209	2,34E-05
TNFRSF21	-1,32206	2,44E-06
HIGD1A	-1,32288	8,33E-07

ACADM	-1,32685	0,000287
USPL1	-1,32799	0,000591
ZBTB38	-1,32827	3,06E-06
FZD5	-1,32897	7,36E-06
INF2	-1,33073	6,87E-06
BIRC6	-1,33193	9,36E-07
OTUD4	-1,33209	5,34E-06
MANEAL	-1,33239	2,16E-05
RNF38	-1,33525	8,47E-06
C6orf132	-1,33602	8,10E-06
ZNF770	-1,33616	0,000229
NPRL2	-1,33631	2,19E-05
LAD1	-1,33685	2,89E-06
TXLNG	-1,33706	4,24E-06
KCNH2	-1,33838	0,000864
UFL1	-1,33853	4,80E-05
LIMA1	-1,34137	0,000745
SIRT2	-1,34157	0,00035
RRP7A	-1,34226	3,66E-08
TST	-1,34313	0,000102
C6orf203	-1,34339	0,000355
DCAF17	-1,34554	0,000152
PTDSS2	-1,34958	0,000915
SLC41A2	-1,35081	0,000469
EPRS	-1,3515	6,54E-10
ADCY3	-1,35215	1,25E-07
PHKA1	-1,35469	7,68E-06
SEPT2	-1,35545	1,56E-07
ACAP3	-1,35552	8,31E-05
LCOR	-1,35734	4,20E-06
KLHL2	-1,35746	0,000274
TYW3	-1,3576	7,36E-06
KDM5C	-1,35882	4,68E-09
KPNA6	-1,36079	2,15E-08
BUB1B	-1,36099	0,000184
XPNPEP3	-1,36299	4,33E-07
PFKFB3	-1,36331	0,000706
MAP3K10	-1,36333	4,30E-05
AEBP2	-1,36394	1,45E-05
FUCA1	-1,364	0,000159
BTBD3	-1,3654	2,34E-06
ZFR	-1,36548	6,31E-06
KIAA0020	-1,36724	6,88E-05
ZNF106	-1,36804	3,80E-09
SLC18B1	-1,36884	0,000337
PPARGC1B	-1,36887	0,000835
NF1	-1,36978	7,92E-06
WRN	-1,37011	0,000128
RAB29	-1,37012	8,89E-05
MED8	-1,37093	1,63E-05

UTP6	-1,37169	1,64E-07
XPO4	-1,3724	8,60E-09
FSCN1	-1,37291	6,35E-05
ARFGEF3	-1,37356	0,000181
FOXN2	-1,374	0,000435
HSF2	-1,37644	2,01E-05
ME1	-1,37708	0,000452
FOXO3	-1,37727	0,000845
CHAMP1	-1,37727	9,06E-06
ASCC3	-1,3776	8,40E-05
PIDD1	-1,37784	8,34E-05
NDUFAF1	-1,37857	0,00037
MORC3	-1,37874	0,000158
TAP2	-1,38066	0,000173
MTRR	-1,38104	0,000227
EFNA5	-1,38138	0,000561
DSCR3	-1,38158	3,23E-06
RUNX1	-1,38273	4,53E-06
ACAT1	-1,38279	0,000745
MTRF1L	-1,38457	2,94E-06
TMEM120I	-1,38513	2,25E-05
SSH2	-1,38543	2,00E-05
NIPA2	-1,38578	1,18E-06
ANLN	-1,38584	4,19E-10
DDX21	-1,38704	2,35E-07
POM121	-1,38736	3,26E-05
PTS	-1,38794	0,000296
CLCN3	-1,38875	2,09E-09
DDX52	-1,38898	8,04E-08
NBEAL2	-1,39052	5,69E-06
LRRC41	-1,39116	3,77E-07
ACACA	-1,39208	1,04E-09
VPS37B	-1,39241	5,60E-08
ATP8B1	-1,39441	2,53E-07
YRDC	-1,3952	2,74E-06
PALLD	-1,39567	0,000763
ZCCHC7	-1,39567	9,02E-06
LIMD1	-1,39633	3,34E-06
TMEM201	-1,39682	7,96E-05
SLC45A4	-1,39688	0,000145
FBXO28	-1,39968	8,56E-05
ELOVL5	-1,39998	2,92E-10
RNF219	-1,40039	8,33E-07
BET1L	-1,40253	4,22E-07
RAD54L	-1,40352	3,10E-05
CARS2	-1,40365	3,51E-07
CTDSPL2	-1,40903	2,49E-06
PHF19	-1,40927	1,65E-07
CFAP97	-1,41019	2,23E-06
PDDC1	-1,41121	0,000172

IPO11	-1,41222	3,07E-06
YIPF6	-1,41283	3,83E-07
ANKRD10	-1,41314	0,000393
SLC23A2	-1,41378	7,63E-07
BAG4	-1,41397	3,81E-06
PIM2	-1,41851	5,77E-05
FIGNL1	-1,41979	4,99E-07
BAZ2A	-1,42	1,66E-09
PDP1	-1,42122	3,99E-07
SIPA1L3	-1,42355	1,62E-06
PPM1F	-1,42514	0,000855
NUP98	-1,42616	1,66E-09
NGLY1	-1,42667	1,12E-06
SH3KBP1	-1,42985	5,69E-05
C9orf40	-1,43034	8,50E-06
SRSF4	-1,43057	4,93E-08
PPP1R26	-1,43125	1,72E-07
SLC39A10	-1,43155	1,78E-06
FAM8A1	-1,43305	7,32E-05
DOCK1	-1,43315	2,48E-06
DEDD2	-1,43338	9,20E-05
TRIP12	-1,4356	5,13E-05
SMC5	-1,43736	4,65E-08
ANKRD17	-1,43746	1,18E-09
PTPN3	-1,43918	5,57E-08
RGS19	-1,44078	0,000786
RHPN2	-1,44156	4,66E-07
CHUK	-1,44181	1,88E-07
CMIP	-1,4421	9,22E-07
DNAJC16	-1,44277	8,23E-06
VKORC1L1	-1,44302	5,03E-10
PHF8	-1,44334	1,00E-07
USP24	-1,44364	4,12E-08
MCM8	-1,44445	3,21E-08
USP45	-1,44601	0,000244
IRF1	-1,44751	0,000296
TEX30	-1,44824	0,000228
PRMT3	-1,44998	4,28E-05
XYLB	-1,45031	0,000446
DR1	-1,4504	9,37E-09
ADCY7	-1,45251	0,000261
TXNRD1	-1,45352	5,46E-05
CASP8AP2	-1,45398	1,11E-05
PRELID2	-1,45438	4,06E-05
CCDC51	-1,45631	3,77E-07
RABGGTB	-1,45639	0,000209
OSGEP	-1,4572	5,10E-06
MTUS1	-1,4582	3,00E-07
PAPD5	-1,46308	8,10E-08
NFAT5	-1,46312	0,000158

JAG2	-1,4644	2,51E-07
NUDC	-1,46585	1,44E-05
FARP2	-1,46781	0,000118
TMEM180	-1,46794	5,01E-05
RBM26	-1,46828	9,86E-08
KIAA0754	-1,46878	0,00062
CTSB	-1,47043	6,45E-05
GALK2	-1,47146	5,28E-06
TRMT6	-1,47287	7,96E-09
SLC25A19	-1,47342	5,64E-06
NUP133	-1,47354	1,72E-09
PMAIP1	-1,47365	0,000673
SPAG9	-1,47388	7,00E-08
PTPRJ	-1,47774	7,88E-07
STAG3L2	-1,481	0,000325
TRMU	-1,48116	1,09E-06
CAB39	-1,4813	6,85E-10
RAD54L2	-1,48238	5,54E-05
MTMR2	-1,4828	1,34E-06
RPL23AP53	-1,48355	0,000695
ATG16L1	-1,4842	1,91E-07
ARFGEF1	-1,4868	1,38E-08
MANEA	-1,48684	0,00026
TIPARP	-1,48842	4,06E-06
TUBGCP3	-1,48921	1,67E-08
UTP14C	-1,49021	6,01E-06
SLC20A2	-1,49104	2,78E-09
ABHD10	-1,49144	6,04E-07
CLCN5	-1,49236	4,58E-06
VCL	-1,4927	3,06E-05
RIF1	-1,49361	1,06E-07
P3H1	-1,49445	4,17E-06
NAB1	-1,49787	2,84E-05
ARIH2	-1,4985	2,29E-10
NABP1	-1,49917	1,69E-05
INTS6	-1,50039	1,49E-05
CMPK1	-1,50092	4,34E-06
CNOT1	-1,50123	1,16E-09
MINK1	-1,50294	1,09E-05
NPAS2	-1,50309	0,000589
KDM6A	-1,50376	2,53E-05
COL4A3BP	-1,50554	4,01E-08
DIS3	-1,50609	1,04E-09
FAR1	-1,50914	2,76E-06
KPNA3	-1,50944	5,07E-08
EDEM1	-1,50949	2,76E-05
KLF13	-1,51	1,33E-09
TANGO6	-1,51087	9,65E-06
ENPP4	-1,51125	0,000673
TRIM44	-1,51132	1,60E-10

PIGW	-1,51234	5,64E-06
DNAJC24	-1,51249	0,000167
TLN2	-1,51501	0,000535
LRRC16A	-1,51667	2,16E-05
FOXK1	-1,51767	4,60E-08
SUZ12	-1,51863	2,08E-11
NFIA	-1,51865	3,03E-06
CUL4A	-1,51963	3,72E-09
SLC5A3	-1,51992	3,46E-07
PIBF1	-1,52059	0,000358
CBFB	-1,5214	5,23E-10
VPS13C	-1,52296	4,42E-06
NKTR	-1,52595	6,11E-05
PFKFB2	-1,52716	2,44E-07
TOE1	-1,52839	3,87E-05
HIAT1	-1,52856	3,88E-08
PCSK7	-1,53002	4,91E-05
MIEF1	-1,53136	9,36E-08
C1GALT1	-1,53156	2,35E-07
FNTA	-1,53166	1,11E-08
AGL	-1,53415	1,28E-09
MAP7D1	-1,53575	4,27E-06
ITGB4	-1,5359	9,41E-07
API5	-1,53709	7,01E-11
RCAN1	-1,54013	0,000466
TPCN1	-1,54029	2,12E-08
RMDN3	-1,54096	1,34E-06
RNF126	-1,54156	4,12E-06
NEIL2	-1,54255	1,73E-07
DFFA	-1,54363	7,40E-10
SLC16A1	-1,54462	3,76E-10
TPP2	-1,54611	2,98E-08
DEGS1	-1,55233	8,17E-06
SERPINB1	-1,55316	5,02E-09
IFNGR2	-1,55389	1,20E-05
ZNF827	-1,55472	0,000559
MON1A	-1,55553	8,01E-05
FAM83G	-1,55569	3,63E-08
TMED4	-1,55809	3,13E-10
FNIP1	-1,5627	3,87E-09
MYO19	-1,56394	1,79E-10
TERF2	-1,56415	1,41E-08
CASC4	-1,56461	3,61E-08
GATA6	-1,56503	0,000284
HK2	-1,56583	2,16E-05
USB1	-1,56795	2,05E-05
KIAA1468	-1,56913	6,81E-07
NAPEPLD	-1,57266	5,30E-05
MYO5B	-1,57366	9,72E-09
GCFC2	-1,57748	0,00053

PLEKHA1	-1,57817	1,32E-06
ERO1A	-1,58101	4,38E-09
NRARP	-1,58151	0,000167
PP7080	-1,58234	2,09E-07
PLSCR1	-1,58387	0,000438
FAM213B	-1,58411	2,21E-08
RNPEPL1	-1,58434	1,21E-05
RHOBTB2	-1,58582	0,000238
GNPDA1	-1,58886	1,12E-07
NEDD4	-1,58916	4,74E-09
TCIRG1	-1,58922	1,66E-05
ADGRG1	-1,58993	3,14E-07
FUT10	-1,59098	0,000737
CC2D1B	-1,59284	6,89E-10
SUGT1	-1,59358	5,11E-08
SIM2	-1,59653	0,000258
TCF20	-1,59981	5,50E-11
STK4	-1,60093	5,26E-11
SHROOM1	-1,60247	4,92E-06
S100A14	-1,60297	3,41E-06
URB2	-1,60509	3,73E-10
SNRK	-1,60565	5,64E-08
JMJD6	-1,6061	2,01E-05
TTC33	-1,60683	0,000116
CPD	-1,60755	4,06E-10
LGALSL	-1,60765	1,18E-06
CCNE1	-1,60927	0,000137
DONSON	-1,61527	9,25E-08
JRKL	-1,61654	0,000918
ZBED6CL	-1,61864	0,000108
SHC1	-1,61874	1,98E-12
PHIP	-1,61935	5,54E-10
CLSPN	-1,62015	1,19E-11
SFN	-1,62153	9,06E-06
SLC25A25	-1,62313	4,94E-05
USP9X	-1,62328	2,67E-08
CTSH	-1,62387	1,98E-05
UBA3	-1,62716	4,24E-08
HAUS6	-1,62775	1,60E-10
CSPP1	-1,62863	1,95E-05
DNMBP	-1,63007	5,69E-05
RTCA	-1,63082	8,08E-10
RPS6KA1	-1,63245	7,23E-06
GOLT1A	-1,63442	5,18E-06
KIF1B	-1,63482	1,36E-07
POMZP3	-1,63692	0,000129
EXT1	-1,63755	4,57E-07
NOL9	-1,64083	9,09E-09
IL6ST	-1,64093	1,12E-08
SASS6	-1,64123	0,000294

PDZD8	-1,64434	1,34E-07
FAM76A	-1,64501	0,000863
U2SURP	-1,64578	1,04E-12
CLN5	-1,64813	0,000551
SEPN1	-1,64833	1,52E-10
SLC12A8	-1,65017	2,97E-06
MAPK12	-1,65036	7,87E-06
DHRS1	-1,65265	0,000402
TMED8	-1,6539	0,000988
HSPG2	-1,65887	3,90E-07
PDP2	-1,66204	5,96E-09
GMDS	-1,66371	2,29E-10
FUT11	-1,66533	8,39E-05
VPS54	-1,66614	2,20E-08
CTNNB1	-1,66685	4,10E-05
MTMR10	-1,66847	3,91E-06
KLHL21	-1,66884	1,18E-08
HES4	-1,67056	1,40E-06
IER3	-1,67084	6,65E-10
HOXB7	-1,67234	2,56E-07
SERPINB8	-1,67252	0,000948
RNF213	-1,67413	1,49E-06
UGGT2	-1,6742	0,000335
NSUN5P1	-1,67534	0,000472
CASK	-1,67548	2,64E-11
TCF12	-1,67566	1,29E-07
SLC30A7	-1,6779	2,01E-06
C4orf32	-1,67878	0,000116
XKR8	-1,6789	0,000101
BCL2L2	-1,68071	0,000383
EHD4	-1,68189	2,13E-08
TMED5	-1,68202	8,66E-08
TCF7	-1,68223	2,75E-05
ARHGEF16	-1,68236	1,83E-08
MKNK2	-1,68256	7,39E-12
ANO6	-1,68282	6,43E-10
ATP7B	-1,68387	4,11E-05
ZNF697	-1,68676	1,40E-05
TRMT2A	-1,68692	2,41E-09
ETNK1	-1,68795	6,21E-11
GK	-1,68816	0,000561
PGM1	-1,68846	1,34E-06
TMEM63A	-1,68961	3,23E-07
PDLIM5	-1,69079	5,94E-08
OSBP	-1,69188	1,44E-12
LIN7C	-1,69239	1,58E-05
C10orf12	-1,69419	1,98E-05
MYO18A	-1,69496	4,56E-07
SZT2	-1,69534	2,72E-07
DENND4C	-1,69831	6,99E-11

SLC35A3	-1,69869	1,14E-05
FAM81A	-1,69902	6,66E-05
CXorf38	-1,69917	1,67E-07
CHORDC1	-1,69981	3,67E-08
PLK3	-1,70233	0,000715
NFKB1	-1,70565	9,61E-07
BIRC2	-1,7061	1,30E-05
IPO5	-1,70631	5,67E-16
HECA	-1,70843	0,000516
ALDH1B1	-1,71132	8,16E-13
TMEM45B	-1,71159	1,94E-10
GATA2	-1,71373	8,11E-06
SGK223	-1,71374	2,41E-08
SORL1	-1,71534	3,42E-06
ST14	-1,71683	1,10E-07
GOPC	-1,71818	1,27E-10
GLB1L2	-1,72339	9,34E-07
STAT3	-1,72545	2,15E-06
SLC25A20	-1,72573	0,000477
PSAT1	-1,72636	0,00017
FAM126A	-1,7267	3,26E-06
ITGA2	-1,72737	1,56E-07
HSPA12A	-1,72828	6,99E-05
TSPO	-1,72958	1,75E-13
MYO1B	-1,73068	2,24E-15
SDCCAG8	-1,73195	4,99E-07
FAM219A	-1,73317	0,000132
RRAGD	-1,73389	0,000147
TMEM62	-1,73426	5,78E-09
NOCT	-1,73709	1,04E-06
ZBED1	-1,73861	3,18E-08
TUBGCP4	-1,74331	5,46E-11
SGK1	-1,74372	0,000448
DNAJC22	-1,74377	4,38E-05
ACSL4	-1,74558	9,07E-07
NAA16	-1,75078	0,000286
SACM1L	-1,75107	1,13E-07
DDX10	-1,75202	2,04E-13
TCEB3	-1,75282	1,27E-12
RNF145	-1,75556	3,87E-05
SLC35D1	-1,75608	1,42E-07
ETHE1	-1,75748	1,97E-06
FTL	-1,75784	9,27E-06
STK40	-1,76184	1,57E-08
HMGA1	-1,7647	4,23E-09
SLC8B1	-1,76509	2,31E-06
GALNT7	-1,77057	2,33E-07
FAM221A	-1,77869	3,28E-05
TOLLIP	-1,781	2,03E-06
SUCLA2	-1,78115	4,36E-11

GAS6	-1,78246	9,83E-08
CDC42EP5	-1,78254	0,000142
LIMK2	-1,78476	1,02E-08
PACSIN2	-1,7851	5,42E-12
ASCL2	-1,78549	4,98E-05
NCEH1	-1,78784	4,40E-07
CDC42BPA	-1,78988	3,58E-09
DYNLT3	-1,79017	0,000357
PATL1	-1,79158	2,17E-13
SYNJ2	-1,79227	6,92E-11
LRP8	-1,79578	9,20E-10
LMBRD2	-1,79587	3,49E-07
MIB2	-1,79657	5,49E-08
KLC3	-1,79707	0,000375
PINK1-AS	-1,79736	2,38E-05
TUBE1	-1,79859	0,000116
MAP2	-1,79868	3,52E-05
INPP5B	-1,79977	4,55E-07
SLC10A7	-1,80099	0,000133
FBXO30	-1,80845	3,55E-10
TXN	-1,8097	7,86E-09
ZNF37BP	-1,81049	3,98E-08
TMEM135	-1,81071	7,37E-07
NT5DC3	-1,81246	8,05E-08
HOOK1	-1,81542	2,58E-11
VWA1	-1,81817	1,59E-06
SMC1A	-1,81874	3,03E-13
KLHL18	-1,82297	2,93E-11
LACC1	-1,82389	0,000367
PPP1R18	-1,82544	1,96E-11
MROH6	-1,82813	1,85E-08
SBDS	-1,82857	3,55E-14
PRPS2	-1,82862	1,42E-09
MFSD2A	-1,83168	6,32E-06
SLC7A11	-1,83176	3,03E-11
FAM3C	-1,83599	3,03E-12
CYB5B	-1,8368	1,38E-10
NCAPD3	-1,83807	7,42E-10
HDHD1	-1,83831	8,60E-08
CORO1A	-1,83921	3,52E-05
PNP	-1,84142	2,45E-13
FURIN	-1,84253	1,50E-12
SLC39A14	-1,84351	5,95E-06
DNAJA4	-1,84409	4,51E-09
MIER1	-1,8446	2,41E-13
DOPEY1	-1,84919	1,32E-06
CTSC	-1,84932	2,05E-11
NDRG1	-1,84947	7,45E-06
SLC7A8	-1,85031	0,000136
ADAM10	-1,85129	1,70E-12

PVR	-1,85217	2,08E-06
IMPA1	-1,85257	4,93E-08
FRAS1	-1,85575	4,33E-12
ARID3A	-1,85715	0,001013
CPEB4	-1,85755	0,000387
PNPLA4	-1,86256	4,08E-06
IRF7	-1,86305	0,000119
ADAT2	-1,86425	6,23E-05
EFHD2	-1,86625	4,90E-10
UBR1	-1,86718	2,58E-06
QSOX2	-1,869	8,35E-08
KCNC4	-1,86994	0,000125
PANK2	-1,87605	4,42E-10
JAK1	-1,87621	5,69E-12
NR6A1	-1,87776	3,27E-06
SH2D3A	-1,88135	7,64E-07
GALNT2	-1,88222	6,42E-15
MYO1E	-1,88271	1,96E-05
GSR	-1,88363	1,76E-13
WDR81	-1,88568	1,08E-10
DOCK9	-1,88749	1,80E-11
MAPKBP1	-1,88868	6,22E-06
ABHD3	-1,88872	1,93E-05
EIF2AK4	-1,88893	1,89E-14
FAM126B	-1,89656	3,75E-08
RELT	-1,89748	1,88E-08
RCBTB1	-1,89914	4,19E-07
ADCY9	-1,90006	3,92E-08
CLCN2	-1,90064	0,000139
TMEM55A	-1,90224	1,62E-06
RPP25	-1,90234	1,13E-11
GFPT1	-1,90847	4,33E-18
NSUN5P2	-1,91444	0,000728
INPP5J	-1,9149	3,45E-05
LPCAT4	-1,91549	1,38E-05
FZD9	-1,91881	0,000982
PRTFDC1	-1,92004	1,83E-05
AP1S3	-1,92129	1,57E-08
CYP4F11	-1,92225	6,72E-05
SDE2	-1,92384	6,33E-06
SYBU	-1,9305	9,69E-06
SLC25A22	-1,9315	6,99E-15
MYO10	-1,93559	2,33E-11
LPIN2	-1,93769	9,08E-11
CDK12	-1,93839	3,63E-15
EOGT	-1,93862	1,53E-06
LOC100506	-1,9421	4,97E-05
NUP43	-1,94228	1,80E-11
ABCB7	-1,94343	3,40E-10
NEK3	-1,94439	1,35E-10

HES6	-1,945	2,52E-06
ABLIM1	-1,94599	6,07E-13
TFDP1	-1,94721	1,11E-18
SLC25A12	-1,95144	1,04E-07
MORC4	-1,95339	3,21E-13
CD83	-1,95657	4,93E-06
TMEM181	-1,95765	3,03E-09
LGR4	-1,9648	9,10E-07
CHD9	-1,96496	1,78E-11
PDE12	-1,96586	1,66E-15
CRLF3	-1,96736	1,63E-07
MDN1	-1,97038	4,06E-14
GCLM	-1,97047	4,95E-07
EHBP1	-1,97647	3,41E-14
ATAD5	-1,97837	3,34E-07
THRA	-1,97857	5,34E-06
ARFGAP3	-1,98199	1,63E-08
FAM73A	-1,98483	2,78E-08
KLHL15	-1,98491	2,37E-06
LASP1	-1,98515	3,30E-08
XPNPEP1	-1,9866	4,61E-13
GALE	-1,98706	6,65E-09
OAT	-1,99199	4,66E-06
NDFIP2	-1,9927	7,54E-12
TGDS	-1,99336	0,000516
NIPAL3	-1,99394	5,39E-06
FABP5	-1,99806	8,77E-18
AKAP17A	-1,99866	2,28E-09
MAP1B	-1,99942	3,07E-05
MYCBP2	-1,99952	4,10E-14
LRIG1	-2,00043	1,22E-11
SYNJ1	-2,0029	1,59E-06
TRPM2	-2,00379	5,73E-11
TMSB4X	-2,00821	1,44E-13
COL9A3	-2,01185	2,27E-06
BIK	-2,01573	2,06E-05
ECHDC1	-2,01854	6,88E-11
OAF	-2,01925	7,04E-06
TNS3	-2,02068	5,98E-08
GLYCTK	-2,02437	0,000368
BMP2K	-2,02663	7,08E-14
CD46	-2,02721	1,39E-09
DFFB	-2,02747	4,01E-06
NRCAM	-2,02792	2,43E-08
CPOX	-2,03953	2,33E-06
PARP12	-2,04172	0,000143
NUP50-AS1	-2,04186	1,05E-05
NMI	-2,04512	4,34E-06
AHI1	-2,04698	7,70E-09
ENDOD1	-2,05186	2,75E-15

CDC14B	-2,05191	1,58E-10
SPIN4	-2,05266	6,17E-06
SPRYD7	-2,0577	4,95E-10
MED14	-2,0602	4,70E-19
DLG4	-2,06437	0,000166
MICAL1	-2,06484	9,38E-06
TMX3	-2,06688	1,94E-10
ZNF589	-2,06995	1,05E-06
KRT23	-2,07157	0,000239
NFKB2	-2,07262	2,88E-13
COBL	-2,07435	6,97E-09
DGKD	-2,08007	2,94E-12
SQRDL	-2,08158	6,41E-05
RAB11FIP1	-2,0855	1,20E-18
POLR3G	-2,09206	1,54E-07
FBXO10	-2,09354	6,83E-09
POLM	-2,09806	1,48E-10
NCOA7	-2,09814	1,94E-13
ATP6AP1L	-2,10173	0,000957
PLEKHB1	-2,10331	0,000137
UBASH3B	-2,10484	0,000598
CPNE7	-2,10573	4,02E-05
SUSD1	-2,11439	2,30E-08
PAOX	-2,11457	0,000961
FAF1	-2,11552	1,23E-08
SH2D4A	-2,11716	1,46E-14
RNF170	-2,11993	4,66E-09
PORCN	-2,12058	1,11E-05
SEL1L3	-2,12382	2,44E-13
AHSA2	-2,12502	0,000291
KLF4	-2,13097	5,11E-13
CLIP2	-2,13583	5,40E-13
POMK	-2,13585	7,18E-06
DCP1A	-2,13942	3,23E-11
KLF3	-2,14623	7,65E-12
PLS1	-2,15079	1,11E-14
RAPGEF5	-2,15175	0,00012
PAM	-2,15278	5,55E-16
FAHD2B	-2,15655	0,000329
INSR	-2,15707	5,67E-09
SH3RF1	-2,159	1,17E-07
CEP72	-2,1594	9,36E-06
ZAK	-2,15983	4,61E-18
ADAM9	-2,16733	2,39E-10
ADAMTSL5	-2,16745	1,21E-06
MAFG	-2,16754	1,02E-17
SMCO4	-2,16904	0,000393
TARBP1	-2,17078	7,03E-10
PAK1	-2,17323	2,50E-09
MICAL2	-2,17589	4,38E-12

OPTN	-2,1786	0,000181
TERT	-2,19099	0,00049
PPFIBP2	-2,19513	1,17E-14
TXLNA	-2,19544	5,91E-16
ABCB10	-2,196	5,72E-12
RELB	-2,20392	3,61E-07
CDC25A	-2,20474	2,01E-15
LINC01123	-2,20877	3,14E-09
CDCA7	-2,21088	5,46E-07
FNDC3A	-2,21369	9,79E-16
ACO1	-2,21797	1,04E-08
CASP3	-2,23035	1,37E-11
RASL10B	-2,2364	4,59E-05
MAPK8IP3	-2,24472	0,000789
DUSP7	-2,24621	2,51E-07
C15orf41	-2,24689	7,47E-07
COTL1	-2,24864	1,84E-13
SHPRH	-2,24972	2,46E-07
KCNJ14	-2,25007	1,36E-06
HAS3	-2,25263	1,04E-05
CEP85	-2,25474	5,61E-09
YAP1	-2,26039	5,63E-20
STK31	-2,26189	0,000201
EXTL2	-2,27259	2,77E-07
PRR5L	-2,27534	0,000194
LMO7	-2,27608	1,56E-14
TWISTNB	-2,27611	2,18E-19
CALML4	-2,28086	8,37E-13
MFI2	-2,28237	6,94E-15
SRXN1	-2,28489	7,88E-09
EPHX1	-2,28738	3,12E-07
PERP	-2,29126	6,04E-11
ARHGAP32	-2,29718	1,71E-13
ARPIN	-2,2982	3,75E-07
TBC1D4	-2,30026	1,54E-07
PRKCE	-2,30401	4,98E-05
GPR180	-2,30447	5,78E-13
TGFA	-2,30586	1,48E-07
DCBLD1	-2,31237	1,81E-09
MIR22HG	-2,31262	8,05E-05
CAPN10	-2,31373	3,22E-08
KCNK5	-2,31572	2,12E-05
PRNP	-2,32698	2,68E-14
LINC01128	-2,33319	1,37E-08
SELM	-2,33832	1,70E-06
KIAA1551	-2,34497	2,85E-19
ME2	-2,35114	4,65E-20
NDNF	-2,35528	9,75E-05
SFXN3	-2,35644	2,63E-10
RASSF3	-2,35924	9,42E-16

LAMA3	-2,36601	6,83E-15
HOOK3	-2,37146	2,93E-13
OTUB2	-2,37327	2,26E-05
SIPA1	-2,37409	3,02E-08
PAQR3	-2,37617	6,43E-15
CORO2A	-2,37801	4,36E-07
LOC284023	-2,38057	7,72E-08
DSG2	-2,38345	9,41E-26
GANC	-2,38403	3,26E-10
PPARA	-2,38765	5,00E-12
PLCXD1	-2,39594	4,84E-10
SLCO4A1	-2,40914	1,31E-10
DMKN	-2,40968	6,20E-19
ARHGAP26	-2,40993	9,38E-06
ALAS1	-2,41326	5,93E-17
LIME1	-2,41386	0,000964
RAMP1	-2,41562	2,29E-06
ALS2CL	-2,41901	0,000387
NHSL1	-2,42028	1,90E-12
FAM101B	-2,42416	1,57E-08
B4GALT6	-2,42471	4,23E-09
CNTLN	-2,43002	1,75E-09
BACH1	-2,43241	1,24E-16
GHDC	-2,43479	3,26E-11
SLC16A3	-2,43686	6,11E-07
SAMHD1	-2,43908	3,28E-07
LHX6	-2,43928	3,66E-05
UGDH	-2,44066	6,63E-18
PCSK9	-2,44079	0,000389
DIAPH2	-2,44113	3,55E-12
TRIM2	-2,44716	4,87E-17
PHLDA1	-2,4507	3,74E-08
PPAP2C	-2,45194	1,91E-21
CARD10	-2,45342	5,03E-12
RP2	-2,45592	8,01E-10
PRRG1	-2,45655	1,10E-05
FLVCR1	-2,45854	5,68E-13
ABHD2	-2,46416	2,41E-15
RILPL2	-2,46517	2,98E-05
SYNM	-2,47141	3,31E-07
PEX6	-2,47143	5,61E-07
TNFRSF10A	-2,474	1,93E-13
TMPRSS2	-2,47429	7,83E-10
SMIM3	-2,47965	3,51E-16
MYB	-2,49064	2,16E-13
FXYD5	-2,50201	1,34E-17
ZNF292	-2,50777	8,29E-25
MUC3A	-2,5084	1,79E-08
MOCOS	-2,51222	1,01E-09
TFR2	-2,52641	4,57E-12

LBR	-2,5278	1,30E-18
AGMAT	-2,53504	6,98E-17
KCNMB4	-2,53507	1,08E-07
LNP1	-2,53738	0,000588
TMC7	-2,53789	2,75E-10
RALGAPA2	-2,54038	3,41E-22
FBLIM1	-2,54223	1,59E-11
HSD17B12	-2,54605	1,22E-26
FAM89A	-2,55067	9,12E-06
CBLC	-2,55128	8,60E-13
B4GALT5	-2,55295	1,18E-26
KIAA1804	-2,55744	1,36E-09
UAP1L1	-2,55954	5,07E-16
C1orf106	-2,56215	3,00E-09
DTX4	-2,56536	1,85E-17
STS	-2,56735	1,06E-12
GNAL	-2,57404	3,76E-05
RDH10	-2,5776	5,91E-13
PRDM5	-2,5867	7,31E-05
ZIC5	-2,5959	0,000469
KLHL17	-2,59688	1,36E-06
INAFM2	-2,59793	4,70E-10
ARAP2	-2,60017	8,27E-15
IFIT5	-2,60104	7,50E-08
DDX60L	-2,6023	0,000205
SH2D2A	-2,60416	2,00E-07
MCF2L-AS1	-2,60562	3,32E-05
C1orf116	-2,61591	3,99E-08
ISG20	-2,61639	1,29E-08
SLC12A2	-2,62026	6,44E-34
UPP1	-2,62081	3,01E-05
RAB11FIP5	-2,62337	4,78E-08
HPDL	-2,63195	5,27E-20
SLC12A7	-2,63603	5,39E-25
MB21D1	-2,6371	4,12E-11
SERAC1	-2,64857	9,11E-13
PIP5K1B	-2,65181	1,20E-07
FLNB	-2,65217	5,75E-18
HHEX	-2,6535	1,47E-05
IGF2BP2	-2,65524	6,03E-21
FSTL3	-2,65857	8,47E-12
SNHG4	-2,662	2,37E-16
ARHGEF38	-2,6621	2,82E-06
ABHD6	-2,66211	8,24E-10
CEACAM5	-2,66354	0,000695
ECE1	-2,68543	5,55E-24
TAGLN	-2,68549	0,000458
TBC1D8B	-2,6888	7,45E-06
SSFA2	-2,69382	3,84E-24
DNAJC3	-2,69915	1,00E-17

TAPBPL	-2,70159	0,000324
GJB2	-2,70723	3,81E-15
ABCA7	-2,71178	1,36E-12
LIPH	-2,71386	0,000342
VGF	-2,7178	1,98E-13
RBMS2	-2,7195	2,76E-13
LOC171391	-2,725	0,000358
NEDD4L	-2,72543	7,62E-19
GNPDA2	-2,73327	6,04E-11
CARNMT1	-2,73519	2,04E-19
ARNTL2	-2,7422	6,50E-14
KIAA1671	-2,74377	2,68E-28
S100A6	-2,75583	1,17E-16
SLC17A5	-2,75846	8,93E-11
MET	-2,76636	5,88E-39
SLC6A8	-2,76649	2,81E-12
PLEKHA4	-2,76714	0,000144
HBEGF	-2,77097	2,81E-10
ANO9	-2,77389	6,72E-06
PTK6	-2,77471	1,01E-16
SYK	-2,77668	5,49E-15
IL4R	-2,77819	2,14E-10
FGFRL1	-2,79002	6,60E-17
ALDH1A3	-2,80187	5,52E-05
RPLP0P2	-2,80312	6,38E-05
TNFSF9	-2,80601	4,02E-05
CTH	-2,80836	1,82E-10
HYAL3	-2,81269	1,14E-05
LOC100288	-2,81553	1,22E-09
SLC12A4	-2,82114	1,47E-05
SERPINI1	-2,82276	2,22E-08
HNMT	-2,82416	0,00016
DNAJC3-AS	-2,82417	0,00023
DAGLA	-2,82748	4,09E-12
TMC5	-2,82918	9,30E-21
SLC35G1	-2,82969	2,00E-09
TMEM106B	-2,82979	9,48E-29
BAAT	-2,8439	0,000327
NSMF	-2,84555	6,79E-28
TREX1	-2,84925	4,87E-12
LBX2-AS1	-2,85057	3,14E-06
FRK	-2,85201	8,05E-05
MXD1	-2,86851	4,57E-19
FN3K	-2,87077	6,17E-07
CHIC1	-2,8741	4,25E-10
RBM26-AS1	-2,88228	0,000482
HLA-C	-2,88348	1,28E-07
FBXO2	-2,8921	1,23E-06
CD27-AS1	-2,90616	0,000773
SOCS6	-2,9083	4,48E-28

ERVK13-1	-2,91553	1,44E-06
HLA-F	-2,92272	4,01E-06
CHRNB1	-2,92299	7,85E-12
TRIP10	-2,92477	8,96E-18
NMNAT3	-2,93135	3,79E-07
SPRY1	-2,93324	7,08E-07
B3GNT3	-2,93606	2,26E-09
PRKAR2A	-2,93628	7,62E-19
TTC38	-2,93752	5,73E-18
EFNB1	-2,93778	3,55E-24
HIC2	-2,94162	1,40E-16
FAM135A	-2,94357	6,03E-14
GPC1	-2,94503	5,12E-12
TMEM80	-2,94886	7,31E-20
EPHB2	-2,95632	9,55E-20
MIRLET7BH	-2,95785	0,000384
METTL7B	-2,96983	1,51E-14
RAB20	-2,97298	3,43E-09
PM20D2	-2,97351	2,04E-18
NUPL2	-2,9764	2,15E-09
SLC2A1	-2,97656	1,05E-14
ANKMY2	-2,98214	6,24E-22
GRK5	-2,98869	1,81E-09
ZBTB47	-2,99448	6,78E-06
BTN3A3	-2,99861	0,000934
CENPV	-3,00098	2,53E-11
GPR63	-3,00725	6,52E-08
PTPRG	-3,0181	1,36E-30
ATP11A	-3,02183	2,65E-28
DACH1	-3,02648	6,43E-14
POLR2J2	-3,02871	0,001002
PITX2	-3,02972	7,00E-08
TYMP	-3,03309	8,19E-10
NAPRT	-3,03754	1,48E-16
CHM	-3,04147	1,12E-11
NKPD1	-3,0418	0,000194
C19orf66	-3,05349	2,14E-05
SH3BGRL2	-3,06048	1,18E-12
S100A4	-3,06163	4,27E-15
STX3	-3,07778	3,06E-22
MGAT5	-3,07865	7,39E-23
PRSS22	-3,08277	1,54E-12
ANK2	-3,08815	3,12E-07
CLCN4	-3,09099	1,36E-06
CCDC82	-3,10004	4,05E-11
SH2B3	-3,10477	8,01E-23
MAPRE2	-3,11059	7,11E-15
GPAT3	-3,11316	1,39E-11
TRAF5	-3,1168	2,55E-19
TRPV1	-3,11981	0,000358

TIAM1	-3,12543	3,76E-21
SH2D5	-3,1273	3,79E-09
SETDB2	-3,12962	1,47E-18
AHNAK2	-3,13714	7,59E-23
L3MBTL3	-3,16094	9,58E-15
ADGRE5	-3,16871	3,29E-18
PLEKHA2	-3,17433	2,83E-19
PPARG	-3,17515	1,49E-13
WLS	-3,17675	1,89E-19
NOTUM	-3,17869	1,40E-17
SLC22A15	-3,18037	0,000181
FAM46A	-3,18457	8,75E-14
ARHGEF10	-3,18607	3,56E-18
HNRNPU-A	-3,18717	1,44E-05
MCF2L	-3,19017	1,00E-21
PCDHGA10	-3,19042	3,27E-09
SNX7	-3,19519	7,12E-13
CA2	-3,20364	0,000734
KIF13B	-3,21511	7,16E-12
DDAH1	-3,22411	2,01E-19
SERINC2	-3,24938	1,62E-17
RTTN	-3,27124	3,08E-18
FAT1	-3,28295	2,92E-24
CLDN15	-3,29096	2,39E-14
EGLN3	-3,29342	2,34E-14
MYRFL	-3,31646	2,00E-05
XK	-3,32454	1,86E-20
LINC-PINT	-3,33162	6,81E-05
CYP4F3	-3,34755	0,00021
PTPRE	-3,36931	6,50E-07
LOC73010	-3,37336	7,46E-07
CAMK2D	-3,38234	3,53E-33
LPCAT2	-3,39085	2,78E-22
GNAI1	-3,39293	5,55E-13
DNAJB4	-3,39478	7,02E-11
TNFAIP2	-3,40044	0,000167
SPRY2	-3,40479	1,45E-15
A4GALT	-3,40921	1,22E-09
KBTBD11	-3,41808	3,39E-15
SASH1	-3,41873	7,08E-14
DPY19L2P2	-3,42751	1,45E-06
AREG	-3,43312	5,00E-18
HOXC8	-3,43524	0,000266
CEMIP	-3,44049	9,97E-23
TRMT11	-3,46795	7,22E-14
SOX15	-3,47039	8,63E-05
TMEM51-A	-3,47683	0,000335
STEAP1	-3,47921	2,26E-10
TRNP1	-3,4838	1,45E-20
IL6R	-3,49058	1,18E-06

FHL2	-3,492	1,17E-07
TPPP	-3,49344	4,61E-15
FERMT1	-3,49851	9,98E-46
ZNF469	-3,51299	4,04E-06
LINC01232	-3,51393	3,33E-05
TNNI3	-3,5211	5,39E-12
RIN2	-3,52731	5,48E-15
NLRP6	-3,52958	0,000551
GRIN2D	-3,54953	3,77E-18
DPYSL3	-3,54992	6,13E-08
SIK1	-3,55755	0,000243
SH3RF2	-3,55805	9,86E-12
MAN1A1	-3,56221	7,75E-39
PLIN2	-3,56441	8,03E-24
SEPT10	-3,57033	9,96E-16
ELFN1	-3,57638	1,45E-11
ERVMER34	-3,59651	1,45E-21
CMPK2	-3,60457	0,000178
DLGAP1-AS	-3,60683	5,33E-08
SHH	-3,60713	3,00E-18
AATK	-3,61045	0,000102
KBTBD8	-3,61389	3,52E-10
WDR90	-3,61719	8,69E-09
EVA1C	-3,62249	1,76E-12
MX2	-3,62689	1,15E-05
PLEK2	-3,62831	4,42E-40
ULBP3	-3,62972	2,69E-07
ST6GALNAC	-3,6353	1,37E-17
MYO15B	-3,63952	3,26E-23
PYGL	-3,64449	8,64E-07
PSMB9	-3,65302	1,17E-08
FNIP2	-3,65865	6,38E-13
ACOT11	-3,65914	3,01E-09
C14orf159	-3,65919	3,77E-17
HDAC4	-3,66935	1,62E-10
SEMA3B	-3,6809	1,56E-19
FRRS1	-3,69361	1,68E-11
PTGR1	-3,71369	5,47E-24
SORBS2	-3,72124	8,87E-06
PLA2G4A	-3,73021	0,000319
ARHGAP23	-3,73074	3,41E-12
CPXM2	-3,73583	9,06E-09
CYP4V2	-3,75116	1,73E-10
CXorf57	-3,75454	1,37E-12
C16orf62	-3,75624	6,72E-36
LINC01315	-3,76858	1,51E-05
NTSR1	-3,78552	0,00072
EGF	-3,79049	0,000625
KCNAB2	-3,7985	1,98E-16
STAMBPL1	-3,80038	1,92E-11

SLC52A3	-3,80153	9,30E-16
PHLDA2	-3,80508	1,48E-16
TNFAIP3	-3,8132	2,92E-14
SP100	-3,81376	5,76E-06
CIB2	-3,82094	1,75E-06
EFNB2	-3,82165	3,00E-47
FAM131B	-3,82239	0,000909
HTR1D	-3,82738	9,35E-12
CREG2	-3,82776	8,20E-05
MAFF	-3,85776	0,000549
BTN3A2	-3,86376	9,20E-10
GLS	-3,86476	6,65E-55
HSD17B11	-3,86643	3,01E-20
SNAI1	-3,895	7,52E-21
CYBB	-3,89666	2,23E-06
SOWAHC	-3,89807	2,89E-30
LGALS3	-3,91201	1,40E-23
C3orf52	-3,91218	2,07E-06
PPM1K	-3,92464	2,67E-13
C3orf80	-3,92839	8,05E-06
HERC5	-3,93357	1,89E-06
DCBLD2	-3,93449	1,80E-46
PTPRH	-3,93992	1,84E-06
LRRC56	-3,95787	3,56E-21
ASPH	-3,96055	2,29E-41
ATAD3B	-3,96073	9,66E-26
ABCC2	-3,97519	4,46E-13
PTCH1	-3,97899	8,41E-36
PROCR	-3,98466	6,81E-09
ADM	-3,99772	2,43E-08
TYW1B	-3,99867	5,02E-11
CMTM7	-4,00069	7,87E-24
BHLHA15	-4,03507	2,46E-11
CD68	-4,03508	3,27E-14
SERPINB5	-4,04064	8,47E-09
IGFBP3	-4,04666	5,06E-13
TGFBR2	-4,05692	1,14E-40
HOXA7	-4,068	3,33E-07
BDKRB2	-4,06808	4,72E-06
FCGRT	-4,08131	8,10E-10
ANO7	-4,09223	3,00E-09
SLC17A9	-4,09702	8,34E-09
RHOU	-4,1008	1,33E-29
UCHL1	-4,10812	2,64E-08
LRAT	-4,11476	5,43E-14
PLBD1	-4,12771	4,76E-31
ZNF513	-4,14351	2,63E-10
FRMD5	-4,15675	7,39E-34
TNFRSF11E	-4,16245	4,93E-16
PELI1	-4,1654	2,57E-25

TCN2	-4,16926	0,000697
GJB3	-4,18125	3,25E-15
ETV4	-4,18718	2,45E-08
SH3TC2	-4,19452	2,43E-08
NELL2	-4,1981	7,44E-24
AKR1C2	-4,20728	6,86E-15
KHDRBS3	-4,20998	3,98E-05
ITGB8	-4,21416	5,39E-05
PLA2G16	-4,22188	6,46E-21
NES	-4,22548	5,90E-30
FOXO1	-4,23054	1,13E-26
LIF	-4,23746	6,70E-12
CREB3L1	-4,24366	8,65E-08
CDK6	-4,26547	3,64E-18
AIM1L	-4,26622	2,72E-27
ZBTB20	-4,27141	0,000192
ANTXR2	-4,27646	8,87E-06
SEMA3A	-4,28951	1,72E-09
SERPINE2	-4,29707	2,54E-33
MLLT11	-4,30846	9,12E-25
MAOA	-4,30853	3,59E-05
HTATSF1P2	-4,32492	7,34E-23
SPON1	-4,32649	0,000756
PRKCA	-4,32964	4,32E-35
PLTP	-4,33981	1,24E-26
ECM1	-4,34286	1,26E-11
DSEL	-4,34877	1,37E-16
TRIM47	-4,36047	1,19E-39
TUBAL3	-4,3781	0,000333
DACT1	-4,37847	4,40E-07
LRRK1	-4,38512	1,74E-30
ADORA2B	-4,38617	2,10E-38
C10orf54	-4,40503	8,51E-05
LCN2	-4,41976	1,34E-14
PLLP	-4,42888	3,01E-12
APOLD1	-4,43194	6,64E-08
ICAM1	-4,43422	1,59E-21
PARM1	-4,44184	1,51E-19
MBP	-4,44925	1,66E-38
CAPN12	-4,45557	2,30E-10
IGFBP6	-4,46789	2,59E-15
TMC8	-4,4808	8,98E-11
STAT5A	-4,49078	3,62E-06
PAK6	-4,50039	1,14E-15
FAM84A	-4,50473	9,96E-21
MSN	-4,50734	5,17E-06
SMPD3	-4,52526	4,23E-12
SATB1	-4,53561	2,03E-05
SLC16A7	-4,54281	3,16E-12
PLAUR	-4,55589	1,70E-17

NRP2	-4,56573	2,37E-16
DCHS1	-4,61099	1,97E-05
BCL2L15	-4,61398	4,62E-20
SLC22A18A	-4,61702	6,68E-05
ILDR2	-4,62169	5,37E-05
LUCAT1	-4,628	6,79E-11
ST3GAL2	-4,65051	1,16E-32
GULP1	-4,6698	1,83E-39
LZTS3	-4,70025	1,80E-25
XKR6	-4,71876	1,23E-05
CCNI2	-4,74969	1,83E-25
NAP1L5	-4,782	3,91E-05
STRIP2	-4,79645	1,93E-19
DUSP6	-4,8047	1,73E-08
B3GNT9	-4,80669	1,73E-15
MACC1	-4,81503	7,67E-24
CNTD2	-4,82047	2,36E-14
KCNQ1	-4,83565	2,97E-28
CRAT	-4,84117	2,62E-23
EPAS1	-4,84175	6,89E-23
HLA-A	-4,84323	5,43E-73
BMP4	-4,84937	7,57E-42
F3	-4,89197	2,54E-39
LOC10192[illegible]	-4,90218	0,000625
GOLGA8B	-4,92362	1,54E-08
CRYBG3	-4,93467	1,33E-25
PRRT3-AS1	-4,93718	0,000167
TRIM38	-4,93738	1,79E-05
GSDMB	-4,95041	4,34E-13
HPGD	-4,9755	2,50E-12
HNF4G	-4,98367	3,73E-17
PMP22	-4,98834	4,11E-31
DUSP4	-4,99372	6,33E-05
NCR3LG1	-5,00797	8,20E-33
GATA4	-5,01107	1,17E-13
ELFN1-AS1	-5,01427	5,48E-10
HLA-B	-5,02166	3,01E-26
ANXA3	-5,03106	1,58E-58
HDAC9	-5,0329	4,83E-08
ANO1	-5,03429	6,92E-07
COL6A1	-5,03609	7,07E-108
BLACAT1	-5,04202	9,10E-09
EHF	-5,0495	1,50E-59
MPP1	-5,05083	2,96E-17
FAM83F	-5,05092	4,49E-20
PKDCC	-5,05832	4,14E-13
CAPN2	-5,06569	2,13E-33
EPHA2	-5,08436	2,77E-46
IL20RA	-5,08595	3,30E-27
FUT4	-5,0991	6,50E-58

ANKRD22	-5,1103	1,48E-05
SLPI	-5,12056	1,00E-06
EMP3	-5,12469	1,03E-11
C17orf51	-5,13923	3,09E-34
EEPD1	-5,1466	1,06E-25
MUC20	-5,15736	9,13E-14
EGFR	-5,16004	1,16E-61
GCNT2	-5,16398	0,00067
LOC10013(	-5,16523	5,02E-20
RASGRF2	-5,16944	1,51E-14
HAPLN3	-5,17193	3,21E-06
PLCB2	-5,17228	0,000251
PTGS2	-5,23179	7,59E-05
CRIP1	-5,23235	6,64E-31
RIN1	-5,23629	1,87E-37
RAB32	-5,25611	3,85E-32
MYH15	-5,25635	2,74E-08
CADPS2	-5,25985	7,81E-14
TESC	-5,26122	5,04E-41
KLK1	-5,26152	1,39E-13
B3GNT5	-5,27091	1,48E-16
ADAP1	-5,2864	6,48E-66
NOXO1	-5,29837	1,67E-17
KIAA0226L	-5,304	1,14E-16
GNB3	-5,30739	5,55E-06
PID1	-5,31309	9,75E-05
MYEOV	-5,31788	1,63E-47
CNTFR	-5,32283	3,41E-15
ITGB6	-5,32289	2,49E-06
SOX6	-5,33055	1,98E-05
HOXB4	-5,33213	1,60E-05
SEC16B	-5,38382	0,000702
EPB41L2	-5,38394	6,68E-83
ETV5	-5,39707	2,48E-28
DDN	-5,44679	5,13E-37
LOC10012§	-5,45137	3,74E-08
LYN	-5,45356	5,28E-36
TRIM31	-5,45558	2,10E-09
IL22RA1	-5,4567	4,84E-10
MX1	-5,46577	4,23E-18
GAS6-AS1	-5,47743	1,31E-10
EMILIN2	-5,48255	1,57E-29
FAM222A-/	-5,4891	2,34E-14
TMEM173	-5,50631	8,04E-08
CALB2	-5,5277	4,28E-31
CSF1R	-5,54088	1,29E-05
ATG9B	-5,54543	1,22E-11
SPNS2	-5,55258	5,53E-55
SPRED1	-5,55299	1,59E-44
JAG1	-5,55857	9,70E-96

TUBA4A	-5,56822	9,72E-26
MTAP	-5,58537	3,41E-28
CASP10	-5,60949	1,16E-16
PIK3AP1	-5,62203	3,33E-23
AOC1	-5,63325	1,88E-08
NAGS	-5,63633	2,52E-23
ARSJ	-5,64015	3,83E-05
CD22	-5,64131	4,99E-07
SLCO4A1-A	-5,64229	1,10E-05
CLIP4	-5,66174	1,28E-08
SPTBN5	-5,67004	8,25E-14
GABRB2	-5,71745	0,000437
DENND1C	-5,71794	6,83E-14
TOR4A	-5,73994	4,19E-88
FOSL1	-5,74299	4,20E-47
MSLN	-5,74469	2,13E-23
TRIM29	-5,76766	0,00016
MEIS2	-5,77576	4,68E-10
ADAMTS2	-5,81011	0,000247
HOXB9	-5,81024	2,45E-05
SNPH	-5,81837	9,78E-06
PALD1	-5,83864	2,40E-82
GAL3ST2	-5,85008	8,67E-09
TM4SF1	-5,85788	1,26E-72
C6orf223	-5,88348	2,48E-28
GPX2	-5,89315	0,00063
VILL	-5,90325	6,15E-06
CHN2	-5,90797	1,08E-09
PEG10	-5,91265	1,70E-06
CEACAM6	-5,9206	7,94E-24
ULBP2	-5,94199	5,07E-21
WNT10A	-5,94592	3,80E-16
ZBTB18	-5,9664	1,15E-24
COL17A1	-5,98474	0,000807
ME3	-6,01091	3,98E-21
ACOT2	-6,0205	5,87E-18
TGFBI	-6,04962	1,10E-26
ANXA1	-6,05079	2,24E-114
ITGA6	-6,06202	2,61E-103
MCTP2	-6,07549	4,05E-14
TMEM150E	-6,0908	5,40E-08
KRT6A	-6,22205	4,02E-05
LAMC2	-6,22361	5,79E-73
NOS3	-6,23221	4,55E-07
LINC01133	-6,25928	6,40E-07
HAS2	-6,27855	5,16E-10
CASP4	-6,29288	0,000474
PNPLA3	-6,29663	6,50E-07
MOB3B	-6,32513	2,80E-13
DISP2	-6,3281	1,49E-05

YOD1	-6,33302	8,00E-44
ROBO1	-6,34619	1,18E-36
MST1R	-6,34789	7,32E-44
EPHA7	-6,35563	3,20E-09
P2RY1	-6,35778	2,93E-05
BIRC3	-6,39938	1,98E-22
DNM3	-6,40486	2,33E-23
COX6B2	-6,45291	8,30E-06
MYRF	-6,47265	2,07E-05
FUT3	-6,4895	3,06E-47
ADRA2A	-6,52539	1,02E-22
HOXB6	-6,53411	4,48E-27
GAS7	-6,5418	3,46E-05
SNORA52	-6,54996	1,74E-05
STEAP2	-6,56205	2,41E-14
PLBD1-AS1	-6,5695	1,43E-08
HMX3	-6,57937	0,000947
COL4A2	-6,60131	1,36E-31
ZC4H2	-6,60212	1,41E-06
AKR1C1	-6,60835	3,27E-53
APOL6	-6,62744	1,30E-58
BIN1	-6,64859	3,74E-57
PHLDB2	-6,74097	3,15E-19
PTPRB	-6,7496	1,49E-33
TRABD2A	-6,76755	2,82E-05
SYT8	-6,79117	0,000417
MID1	-6,79592	7,97E-61
NEK9	-6,79651	9,63E-05
INPP1	-6,80721	2,07E-06
EPSTI1	-6,82305	3,85E-06
TWIST1	-6,86481	4,48E-09
LINC01138	-6,87492	3,45E-07
ZNF717	-6,8861	5,48E-07
CA9	-6,90869	4,43E-11
TNS4	-6,92837	6,62E-51
C1QTNF1	-6,95752	5,45E-17
SPRY4	-7,05008	2,08E-78
TMEM74B	-7,08837	0,000144
PEAR1	-7,09024	0,000695
LAMB3	-7,09684	1,63E-06
LGALS9	-7,11575	7,20E-37
TBX20	-7,12324	4,44E-16
LINC00673	-7,131	5,78E-05
SMIM24	-7,13207	0,000116
HOXB3	-7,15201	2,17E-10
ETV1	-7,20569	8,59E-05
RIMBP2	-7,37683	4,92E-05
LGR5	-7,39201	9,42E-20
AKR7A3	-7,39698	6,81E-09
UGT1A10	-7,3999	0,000942

LANCL3	-7,4005	0,000933
IL32	-7,41841	2,93E-08
ZNF71	-7,42002	6,49E-09
TRIM15	-7,42569	7,86E-37
C14orf105	-7,42861	0,00101
ZNF502	-7,43941	0,000865
PCSK5	-7,43979	1,65E-15
PRDM1	-7,45598	4,10E-09
SSTR5	-7,45939	0,000815
HOXB-AS3	-7,46681	0,000704
LIPE	-7,46769	3,56E-09
PAX6	-7,47959	0,000894
GRAMD2	-7,48843	0,00091
LINC01322	-7,50138	0,000871
DGKG	-7,50669	8,06E-09
BHLHE41	-7,50984	2,61E-05
KCNH8	-7,51664	9,16E-05
HLA-H	-7,51773	7,03E-09
PRAP1	-7,59283	2,25E-43
DUSP5P1	-7,59975	0,000921
ZNF577	-7,62086	0,000601
SLC2A12	-7,62358	1,74E-05
DSG3	-7,6259	0,000549
PIWIL4	-7,65428	3,01E-05
AKR1C3	-7,65464	5,74E-05
RRN3P1	-7,65941	0,000391
RUNX3	-7,6619	1,13E-15
TIMP4	-7,67701	1,41E-05
C1RL	-7,68396	6,42E-18
CES4A	-7,68883	0,000284
LINC00520	-7,7067	0,000312
SKAP1	-7,72333	0,000247
PLAU	-7,73348	0,000331
PROC	-7,7576	0,000255
PDCD1	-7,75959	0,000213
TM4SF5	-7,7611	0,000219
ZNF69	-7,77462	0,000194
EREG	-7,7809	2,36E-07
NLRC5	-7,79428	4,42E-70
ANKLE1	-7,8031	0,000435
TMED6	-7,81505	0,00028
GALNT9	-7,81601	0,000347
TINAG	-7,82413	0,000189
LOC101927	-7,84988	0,000138
PRDM13	-7,86073	0,000132
NTRK2	-7,86273	0,000431
ZNF583	-7,86914	0,00014
PTPRR	-7,8932	0,000117
NEURL3	-7,8934	0,000278
SOX9	-7,89385	7,70E-93

AKR1B10	-7,89469	8,23E-12
DKFZp434J	-7,91512	0,000106
FAM27B	-7,91512	0,000106
SLC44A4	-7,91813	8,50E-19
MGAT3	-7,92066	1,08E-09
RAET1L	-7,9248	4,32E-06
MYOM2	-7,94291	0,000113
SLC2A9	-7,94816	9,18E-05
C15orf52	-7,95643	7,22E-06
MYO1A	-7,97008	9,78E-05
BAIAP2L2	-8,0161	5,90E-25
SPNS3	-8,01806	7,19E-05
LRRC66	-8,03065	6,81E-05
ABHD12B	-8,03108	6,11E-05
TPRXL	-8,03829	3,74E-22
OSR1	-8,03947	2,67E-06
MALL	-8,05409	3,40E-06
C2orf70	-8,07787	5,50E-05
CTAGE15	-8,08593	5,23E-05
KRT20	-8,08645	1,66E-43
DNAH12	-8,08769	7,49E-05
GREM1	-8,09231	5,50E-05
LINC01356	-8,0999	4,42E-05
LPAR1	-8,11248	0,000101
HCP5	-8,11822	7,56E-38
ARHGAP6	-8,13256	7,00E-05
MDFIC	-8,14911	2,49E-16
C4BPB	-8,16514	4,48E-05
LINC00857	-8,18096	1,30E-11
LCAL1	-8,18998	3,07E-15
CARD6	-8,19575	2,79E-05
TM4SF20	-8,20019	3,15E-05
RNF128	-8,20648	5,08E-05
KLHL3	-8,21239	2,82E-05
ACOT1	-8,21637	1,26E-06
SCG2	-8,24584	3,92E-05
RRAD	-8,2648	2,21E-05
GJC2	-8,27378	7,93E-12
FAM27E3	-8,27894	1,94E-05
SOX1	-8,283	2,87E-05
EPHA1-AS1	-8,28637	4,30E-05
MT1A	-8,29999	2,36E-05
LINC01268	-8,3001	2,28E-05
SLC7A7	-8,31436	1,42E-17
NR0B2	-8,31593	2,16E-05
LINC00526	-8,3246	1,50E-05
CYB5R2	-8,32564	1,77E-05
VGLL3	-8,33349	2,36E-05
ZNF773	-8,33509	1,39E-05
SLC9A3	-8,35277	0,00013

SGCE	-8,3539	1,27E-05
CSMD3	-8,36754	1,26E-05
SLC22A3	-8,38377	3,33E-05
MYL9	-8,42429	1,14E-05
HNF4A	-8,42684	1,41E-59
SNHG18	-8,42993	8,77E-06
PLB1	-8,43384	1,17E-05
TNC	-8,44545	2,35E-44
PRSS12	-8,44853	2,78E-18
GAL3ST1	-8,45544	1,01E-05
NPR2	-8,46067	9,97E-07
TMEM246	-8,48032	7,21E-06
NT5E	-8,50096	6,03E-06
DLL3	-8,50278	6,00E-06
TM4SF4	-8,5161	0,000459
MT1G	-8,52433	6,78E-06
ZNF470	-8,53251	6,42E-06
TLDC2	-8,54012	6,59E-06
LHX5	-8,54255	5,61E-06
GALNT8	-8,546	6,09E-06
GBP3	-8,54916	4,68E-07
AASS	-8,55362	4,63E-06
A1CF	-8,55625	4,57E-06
CHST13	-8,56116	4,42E-06
CREB3L3	-8,57003	7,01E-06
GUCY2C	-8,58368	5,78E-06
SFTA2	-8,58553	3,95E-06
HBQ1	-8,59293	4,64E-06
CDA	-8,60471	3,89E-06
TMBIM1	-8,61297	2,13E-77
BATF2	-8,62272	2,78E-18
KLK10	-8,62367	3,27E-25
AMN	-8,6242	2,55E-19
LINC00431	-8,63102	6,10E-06
MMP7	-8,67264	2,52E-06
PDZK1IP1	-8,69467	2,22E-06
ZNF503-AS	-8,7122	2,12E-06
ELAVL2	-8,71846	2,05E-06
ZNF518B	-8,72587	2,99E-06
LINC01559	-8,72751	5,59E-06
LOC10106(	-8,72878	1,89E-06
LRRN4	-8,74397	3,35E-06
CDHR5	-8,7449	1,72E-06
OSBPL3	-8,79375	3,50E-68
ZNF320	-8,81007	1,32E-06
SPP1	-8,81453	2,58E-05
TFCP2	-8,8181	4,84E-05
LOC10192(	-8,82096	1,15E-06
LINC00668	-8,84372	1,01E-06
REG1A	-8,87158	7,16E-06

KLK12	-8,88587	8,36E-07
LONRF3	-8,88872	1,64E-14
HOXB8	-8,89412	8,40E-27
VSNL1	-8,90186	1,00E-13
LYZ	-8,91601	2,58E-07
P3H3	-8,94113	3,73E-08
HCN1	-8,94663	6,06E-07
DPP10	-8,95143	9,51E-15
CCL24	-8,97766	7,38E-07
EMP1	-8,97885	7,07E-15
ITGA1	-8,97993	8,11E-08
RASSF6	-8,98142	1,63E-21
LY75	-8,987	4,96E-07
DIO3	-8,99229	5,96E-23
GABRE	-9,00214	2,98E-06
ZNF175	-9,0069	5,01E-07
ACSL5	-9,01237	8,52E-82
GPR35	-9,04203	8,92E-41
CXCL3	-9,06779	7,46E-08
SLCO2B1	-9,06794	3,01E-07
CDKN2A	-9,06871	5,38E-62
C1RL-AS1	-9,06938	2,74E-06
PROM1	-9,08231	4,42E-07
NKX2-5	-9,15232	3,15E-07
HCG26	-9,1557	2,16E-07
C7orf31	-9,15615	2,20E-07
DMRTA2	-9,19567	1,26E-08
TRIM10	-9,19981	1,64E-07
GATA2-AS1	-9,22479	1,07E-08
PCDHA4	-9,23838	1,52E-07
CTSZ	-9,24178	3,78E-79
GBGT1	-9,24407	1,15E-07
ALDH2	-9,29847	3,92E-109
CFTR	-9,3181	2,69E-07
UGT1A1	-9,32289	7,68E-08
FAM155A	-9,32587	9,32E-08
TNFRSF14	-9,33669	3,48E-16
SLC39A5	-9,35148	1,07E-07
DPP4	-9,3749	6,14E-43
DKK4	-9,38465	5,95E-08
DEPDC7	-9,39259	5,08E-08
THSD7A	-9,42397	4,92E-08
UGT8	-9,43996	1,97E-06
DSG4	-9,44558	7,20E-08
CXCL2	-9,47846	1,89E-06
MAP7D2	-9,48274	2,86E-38
TMEM200A	-9,48902	2,93E-08
ZNF506	-9,49139	3,44E-08
NPM2	-9,49977	3,92E-08
C1orf61	-9,57609	2,41E-08

MOGAT3	-9,57611	1,83E-08
GRIN2B	-9,59233	1,64E-08
FGF19	-9,59782	0,000121
CNN3	-9,60202	2,17E-09
TCN1	-9,61003	2,56E-08
FOXQ1	-9,68838	1,13E-11
SFRP5	-9,71913	3,54E-08
GABRB3	-9,72954	9,38E-10
POU4F1	-9,73676	9,07E-09
SULT4A1	-9,77483	8,09E-09
HOXA10	-9,80324	4,93E-09
PDX1	-9,81802	5,14E-09
ISL1	-9,83262	4,22E-09
LOC389602	-9,84416	4,23E-09
TDO2	-9,84485	5,30E-09
KCNE3	-9,85194	6,59E-10
CCND2	-9,85526	2,41E-137
AGPS	-9,86315	3,18E-57
CALB1	-9,94435	4,72E-10
CD40	-9,96295	2,12E-09
SLC38A5	-9,99038	7,96E-68
LINC00941	-9,99283	1,99E-09
ONECUT3	-10,0111	3,28E-09
ATP10B	-10,0278	5,34E-28
WBP5	-10,0544	1,49E-09
G0S2	-10,0587	1,19E-09
SOX8	-10,0591	4,09E-27
ETS1	-10,0902	1,07E-09
GDA	-10,0986	9,21E-10
ADAMTSL2	-10,1632	2,53E-09
CACNA1E	-10,1929	5,77E-10
FAM71E1	-10,2324	8,36E-10
PSMB8	-10,2455	8,88E-38
IRF8	-10,2786	5,16E-10
LDHB	-10,2914	0,000549
TLR6	-10,2943	3,02E-10
SLC6A20	-10,3178	4,76E-10
C6orf15	-10,3417	2,12E-09
CXCL1	-10,4069	9,15E-10
CYP3A5	-10,4133	3,34E-10
TDRD12	-10,4166	5,13E-21
LOC100507	-10,4253	1,93E-10
TTC22	-10,4288	1,40E-10
MAP1LC3A	-10,4662	1,12E-10
CDKN1C	-10,4823	1,95E-09
C5orf42	-10,4856	1,48E-10
PADI1	-10,5142	8,44E-11
CLRN3	-10,5223	8,07E-11
REG3A	-10,5306	1,54E-09
TCEAL8	-10,5639	7,07E-11

GJB1	-10,573	5,17E-10
ZNF300	-10,5732	1,32E-10
SBSPON	-10,5801	9,02E-11
HS3ST1	-10,6001	2,56E-11
HOXB5	-10,6276	4,46E-11
ZNF649	-10,6497	3,94E-11
CADPS	-10,7057	3,47E-22
GSPT2	-10,7074	3,05E-11
RAC2	-10,7348	2,50E-11
FRG1CP	-10,7431	2,30E-11
SLC5A1	-10,8114	1,98E-11
NUDT16P1	-10,8842	1,30E-11
HRH1	-10,8993	1,22E-11
ZNF215	-10,901	1,52E-11
SEMA5A	-10,914	9,15E-12
MYH4	-10,9294	1,23E-11
BCL11A	-10,933	7,64E-12
MYO7B	-10,9387	8,00E-12
LOC11511(	-10,9746	7,86E-12
ANXA13	-10,9805	5,82E-12
IPW	-10,9978	1,00E-11
ANKS4B	-11,0015	5,26E-12
GIPC2	-11,014	4,68E-12
UCA1	-11,0174	7,03E-44
KLK6	-11,0526	5,79E-45
CYP2W1	-11,074	9,79E-12
ZC3HAV1L	-11,1092	1,27E-11
LGALS4	-11,1148	4,16E-24
CCAT1	-11,1181	2,54E-12
CDIP1	-11,1571	4,84E-12
ALDH1A1	-11,1852	7,74E-65
ZNF280B	-11,2167	1,78E-12
GSTP1	-11,2361	1,70E-136
ADGRG5	-11,2691	1,05E-12
CDH11	-11,3121	1,13E-12
MT1E	-11,3247	1,01E-12
KCNJ2	-11,3492	8,37E-13
TNFSF15	-11,3685	2,20E-12
HMGA2	-11,4013	1,34E-13
PHGR1	-11,4116	1,31E-12
MDFI	-11,4126	4,49E-13
TNFRSF10D	-11,4771	1,39E-12
SGK2	-11,5123	2,53E-13
CA13	-11,5508	1,36E-12
KIRREL	-11,6004	1,61E-13
SDHAF3	-11,6316	2,34E-13
USH1C	-11,7058	2,29E-14
SLCO1B3	-11,7381	4,34E-13
DPEP1	-11,767	6,84E-27
C2CD2	-11,8585	4,76E-14

PVRL3	-11,8805	3,09E-14
FUT6	-11,9421	1,94E-14
CDX1	-12,0106	1,26E-14
LINC00667	-12,0268	2,08E-14
GNE	-12,064	9,35E-15
HKDC1	-12,0803	1,37E-41
TNFRSF1B	-12,1107	1,79E-14
PRSS3	-12,1595	5,46E-15
DPP10-AS1	-12,2617	3,16E-15
FGFBP1	-12,3204	3,23E-15
MMP1	-12,3263	3,30E-14
KLK11	-12,3488	3,16E-15
FOXA3	-12,3767	1,80E-15
PROX1	-12,3802	1,76E-15
HNF1A	-12,3888	1,29E-15
MGAM2	-12,4117	1,27E-15
PPP1R1B	-12,4244	1,45E-15
ZCCHC11	-12,4642	8,44E-16
GRAMD1B	-12,5182	1,05E-15
FUT2	-12,5408	5,15E-16
MYOM3	-12,57	4,32E-16
SSTR1	-12,6904	1,02E-15
HEPH	-12,6905	2,66E-16
SIGMAR1	-12,7302	1,02E-31
GCNT3	-12,7503	1,53E-16
EPS8L3	-12,8443	8,14E-17
PIWIL1	-13,0069	3,48E-17
GPA33	-13,1721	1,37E-17
CES1	-13,2664	1,30E-17
TSPAN8	-13,4141	2,35E-18
TINAGL1	-13,5457	1,28E-18
DDC	-13,5966	9,30E-19
IGF2BP3	-13,6638	6,43E-19
SAMD5	-13,7191	3,96E-19
GALNT5	-13,7696	2,28E-19
MUC17	-13,7966	1,57E-18
EMC10	-13,8489	1,37E-19
GPX1	-14,2001	2,27E-20
CLDN2	-14,6936	5,15E-22
CDH17	-14,8429	1,94E-22
XIST	-14,8789	6,68E-10
VIL1	-15,1111	2,81E-23
MUC13	-15,3276	6,28E-24
FLNC	-16,1695	2,21E-26

HCT15

Gene	log2FoldCh	FDR
BMP7	16,08871	3,59E-26
FREM2	15,46054	3,87E-24
LDOC1	14,83828	6,13E-22
TSPYL5	14,70998	7,20E-22
FAM127B	14,51422	2,69E-21
C3orf14	14,31005	1,67E-20
STC1	14,25015	2,78E-20
EFEMP1	14,01727	8,73E-20
PPP2R2C	14,00795	7,87E-20
ELOVL5	14,00236	7,89E-20
ESR1	13,96691	1,24E-19
FAM127A	13,79486	2,13E-18
BNIP3	13,67158	8,23E-19
GFRA1	13,63107	3,08E-18
OBSL1	13,6066	2,92E-18
ANTXR1	13,54043	1,64E-18
TFF1	13,47215	3,11E-18
S1PR3	13,4291	4,38E-11
DSCAM-AS	13,41322	8,68E-11
HSPB8	13,19107	4,78E-17
H2AFJ	13,16881	2,80E-17
FAM127C	13,0751	3,43E-17
PXDN	13,07411	4,88E-17
MYEF2	13,03724	1,22E-16
BASP1	13,03202	3,44E-09
PCDHA6	13,00594	5,35E-17
ZNF43	12,99129	5,32E-17
VAV3	12,97832	6,68E-17
ACSS3	12,86763	1,40E-17
KCNG1	12,86512	9,23E-16
IRX2	12,78701	2,23E-16
BST2	12,77299	4,68E-08
MAP7D3	12,70177	3,22E-16
ZNF737	12,69345	2,11E-15
SPDEF	12,6842	1,65E-15
ALDH3B2	12,68083	3,16E-15
HS6ST2	12,67379	9,63E-16
DYNC1I1	12,66238	4,13E-16
COLEC12	12,61011	4,23E-15
ZNF595	12,6088	1,05E-15
EMB	12,59489	6,32E-16
KYNU	12,48043	5,49E-15
CBS	12,46973	1,39E-15
ANO6	12,45716	1,86E-15
SPG20	12,44866	1,63E-15
L1CAM	12,40309	6,19E-15
PCDH18	12,39491	1,93E-08
B4GALNT4	12,38271	6,77E-15

PCDHA11	12,37883	3,92E-15
ZNF22	12,30551	2,24E-14
NRCAM	12,29366	1,24E-14
RPL39L	12,28571	4,28E-15
DLX5	12,27423	5,50E-15
LIN7A	12,26351	8,47E-15
C15orf59	12,2487	2,49E-14
PCDHB3	12,2372	8,33E-15
GALNT16	12,22578	1,50E-05
PYCARD	12,18902	1,64E-14
SLC1A2	12,18134	8,84E-15
ADCY1	12,15314	1,26E-14
ADGRB1	12,15092	9,63E-15
C14orf132	12,13333	1,08E-14
SCN4A	12,13316	3,29E-05
FBXO17	12,1052	3,99E-14
ZNF85	12,04229	1,83E-14
ZNF829	12,0339	3,49E-14
WNT5A	12,01504	4,77E-14
FAM50B	12,00773	2,44E-14
MTERF1	12,00101	2,79E-14
ZIC1	11,97284	7,33E-14
CRISPLD1	11,95601	1,77E-13
BCAS1	11,91574	9,55E-05
PCDHB8	11,91281	4,46E-14
DTX3	11,89689	4,42E-14
AR	11,85234	6,04E-14
GUCY1A2	11,84118	7,17E-14
GALNT14	11,82153	6,96E-14
ZNF736	11,80418	8,71E-14
MMP17	11,7645	9,78E-14
PCDHGC3	11,72563	1,94E-13
CHST15	11,72186	1,55E-14
C5orf38	11,65494	3,42E-13
SDC2	11,63741	2,18E-13
KRBA1	11,60867	2,90E-13
P2RY6	11,60332	0,000234
MAP9	11,59992	4,03E-13
ASCL4	11,59737	3,03E-13
ZNF134	11,55249	4,41E-13
SYT1	11,49205	9,23E-13
TCEA2	11,4889	5,12E-13
EFS	11,47244	6,78E-13
CRMP1	11,46301	8,16E-13
ZNF813	11,37073	1,47E-12
GUCY1A3	11,35146	1,32E-12
ZNF331	11,32069	7,64E-12
PTPRO	11,30728	2,52E-12
SUSD3	11,30037	1,97E-11
LIFR	11,28903	4,09E-12

RIMS4	11,28752	8,31E-12
PGR	11,27648	7,34E-11
MIPOL1	11,26546	1,97E-12
TUSC1	11,25237	2,09E-12
FAM155B	11,23424	2,60E-12
ZNF548	11,20858	3,37E-12
SOX2	11,19864	2,88E-12
RGMA	11,1934	4,60E-12
MPDZ	11,16784	5,18E-12
FGF12	11,16081	6,10E-12
CRIP2	11,09976	1,21E-44
ZNF512	11,09569	5,60E-12
ZNF606	11,09051	1,07E-11
CHST10	11,07506	2,09E-11
LMX1B	11,06323	6,62E-12
TSHZ3	11,04687	1,55E-10
SLFN5	11,04478	8,53E-12
SPESP1	11,01466	1,33E-11
EVL	11,0054	3,05E-23
OSMR	10,97983	2,58E-11
ATP2A3	10,8654	1,70E-11
CEACAM21	10,86124	2,30E-11
DTNA	10,85915	3,37E-12
TDRD5	10,84308	3,75E-11
CHGA	10,79427	3,20E-11
DPYSL4	10,76235	9,77E-11
EXO5	10,74576	4,79E-11
PCDHA10	10,7415	5,36E-11
DDIT4L	10,73782	8,44E-11
RAB31	10,73773	1,55E-10
COL6A2	10,73532	5,14E-11
ZNF347	10,67226	9,73E-11
CASC9	10,67012	6,71E-11
SHISA9	10,65819	1,09E-10
EHD3	10,64886	1,32E-10
SYNGR1	10,6155	2,98E-10
AARD	10,61437	9,48E-11
PCDHA12	10,59859	1,45E-10
ZNF570	10,59639	1,11E-10
HPDL	10,58731	1,19E-10
ZNF287	10,58692	1,10E-10
STC2	10,57244	7,61E-05
ELOVL2	10,55088	1,44E-10
CXCR4	10,53595	1,59E-10
NSUN7	10,52181	1,70E-10
RNLS	10,49489	1,92E-10
ZNF682	10,49182	1,95E-10
ZYG11A	10,48245	6,24E-10
TFF3	10,48196	2,63E-10
C18orf63	10,47496	2,96E-10

LINC00052	10,47208	2,43E-09
RERG	10,46316	2,31E-10
ERVMER34	10,44851	6,39E-10
ZNF572	10,43944	3,80E-10
CHFR	10,40148	2,45E-11
MATK	10,40087	2,98E-38
LINC00925	10,39606	3,77E-10
DUSP9	10,39559	3,62E-10
ERBB4	10,38949	5,48E-10
ZNF569	10,38374	4,58E-11
PRKAA2	10,38041	3,69E-10
ZBED9	10,37291	3,80E-10
BEX2	10,35968	9,66E-10
ZNF486	10,32628	6,34E-10
ZNF732	10,32627	9,76E-10
SALL2	10,32364	3,48E-11
GREB1L	10,30301	8,00E-09
ASCL1	10,29038	1,29E-09
FAM110B	10,2868	8,52E-10
MAGEH1	10,26559	1,04E-09
ADGRL2	10,26267	8,46E-10
SDK1	10,25012	1,04E-09
C12orf56	10,23597	8,98E-10
ZNF350	10,23214	9,15E-10
EPHA6	10,22366	9,23E-10
FERMT2	10,22036	6,04E-11
CXCL12	10,19548	1,21E-09
SLC9A4	10,19419	5,83E-09
ELF5	10,18351	7,66E-09
PRIMA1	10,17464	5,10E-09
GNB4	10,17118	1,22E-09
ZNF818P	10,15072	1,40E-09
NCKAP5	10,13181	1,70E-09
IGFBP5	10,12845	1,65E-18
SYT3	10,12824	5,25E-09
CHST8	10,10453	1,79E-09
HLA-DRB1	10,07831	6,49E-09
MALRD1	10,07242	5,08E-09
CKMT1B	10,07012	3,77E-19
NOVA1	10,06058	3,44E-09
ARHGDIG	10,05956	1,45E-10
ZNF525	10,04886	6,96E-19
ZNF529	10,02715	2,84E-09
ADIRF	9,993416	5,14E-16
BEND5	9,989825	4,50E-09
ZNF568	9,988893	1,44E-08
TUBA3E	9,976225	6,78E-09
CTSF	9,954454	5,89E-09
TRPV4	9,950384	6,35E-09
PCDHB6	9,939866	5,90E-09

ZG16B	9,937022	3,42E-08
EDA2R	9,921779	1,23E-08
ZNF492	9,920583	2,42E-08
ZNF615	9,919655	6,94E-09
MUC5B	9,91782	5,39E-09
SPINK5	9,91124	2,60E-08
SYNPO2	9,901092	1,13E-16
ZNF790	9,898411	7,88E-09
TYRP1	9,885693	6,72E-09
ERC2	9,873483	6,76E-09
PRLR	9,872352	4,74E-26
MED12L	9,868654	8,73E-10
SLITRK4	9,862422	9,98E-09
RNF165	9,840588	9,16E-09
ZNF772	9,840342	8,21E-09
IRX4	9,835134	1,67E-08
TEX15	9,834264	8,42E-09
GPR158	9,832135	9,08E-09
FAM19A5	9,806613	1,51E-08
MFAP2	9,805429	5,79E-10
CST1	9,804459	5,99E-10
C5AR2	9,802418	1,64E-09
ACKR3	9,80135	2,10E-33
ZNF630	9,79352	1,07E-08
GLI3	9,786185	7,98E-10
NRXN2	9,778399	6,43E-10
ZNF793	9,775445	6,75E-10
PCDHGB5	9,773252	1,20E-08
WDR17	9,767539	1,25E-08
FAM3B	9,767518	6,66E-10
RTN4RL1	9,758581	3,28E-33
FUT9	9,753136	1,34E-08
RASL11B	9,752092	7,24E-10
TENM4	9,740581	2,57E-08
DDX43	9,73766	1,90E-08
COL5A1	9,736137	4,28E-08
TUBA3D	9,733453	2,95E-08
GPR27	9,731577	1,84E-08
ZNF549	9,687153	3,02E-08
PYGO1	9,661519	5,68E-08
MMP16	9,657177	3,41E-08
PCDHA7	9,645748	2,52E-08
KLRG2	9,644041	3,10E-08
ZDBF2	9,617959	5,92E-08
ZNF879	9,602217	3,35E-08
ZNF354C	9,600252	3,77E-08
ARHGEF25	9,597458	3,85E-08
BGN	9,589008	0,000134
WISP2	9,588515	1,78E-06
C17orf104	9,578011	1,72E-07

RAB34	9,569217	1,75E-23
FCGR1A	9,567202	2,03E-07
ID4	9,549858	2,87E-06
NPY1R	9,548937	1,43E-16
EYA2	9,543705	1,66E-07
AKR1C2	9,535713	1,72E-08
SMARCA1	9,534441	1,12E-07
HOXC-AS3	9,531524	5,52E-08
SYDE1	9,530235	5,18E-08
PGBD5	9,526925	6,72E-08
CA8	9,519356	8,17E-08
POF1B	9,517578	2,48E-21
ONECUT1	9,51245	1,07E-07
SIRPA	9,508806	5,47E-08
COL4A5	9,493659	1,17E-07
LOC339862	9,485725	7,19E-08
CNGB3	9,4835	8,21E-08
INHA	9,478977	5,77E-06
SFMBT2	9,475249	1,12E-07
RIC3	9,468758	1,18E-07
NCAM2	9,464568	1,06E-15
PCDHB4	9,434149	3,33E-07
LINC00992	9,414908	1,08E-07
CXXC4	9,405229	1,23E-07
NRG3	9,394136	1,63E-07
PPM1E	9,379547	2,77E-06
TUB	9,378697	1,14E-07
THBD	9,373821	1,45E-07
SHISA2	9,336291	2,42E-07
GSG1L	9,334491	1,60E-07
CRABP1	9,317725	4,98E-07
PGM5	9,317352	2,32E-07
ADAMTS9	9,313517	1,07E-06
SLC22A17	9,312854	1,75E-07
SPSB4	9,30596	3,77E-07
GRIK3	9,279889	8,96E-07
HOXC13-AS	9,260037	2,95E-07
TNFRSF18	9,255547	1,65E-06
AMZ1	9,254918	4,48E-07
RNF150	9,254693	2,62E-07
RPRM	9,249577	6,08E-07
HTR2C	9,242047	2,55E-07
CACNA1H	9,240967	1,19E-07
TMEM163	9,237309	3,52E-07
S100A9	9,228318	3,92E-05
NHLRC1	9,216129	8,44E-07
RADIL	9,214822	1,04E-06
C3orf67	9,209012	8,21E-07
CNNM1	9,206388	2,97E-07
PCDHA5	9,204132	3,15E-07

SLC12A5	9,191678	3,22E-07
CALCR	9,19097	4,50E-07
KCNMB2-A	9,190703	3,25E-07
SNCAIP	9,184104	3,36E-07
PLEKHA8P1	9,173467	9,84E-07
PLA2G3	9,170193	2,11E-06
MPPED2	9,137487	1,57E-06
ELOVL4	9,124756	1,16E-06
PCDHB16	9,124317	1,16E-20
TPTE	9,123467	5,52E-07
FBXO15	9,121688	4,82E-07
LOC644919	9,112071	7,64E-07
MGAT5B	9,103718	8,15E-07
ADAMTS19	9,098045	5,60E-07
DSCAM	9,091345	1,29E-06
CIART	9,077921	2,91E-23
BEX4	9,076557	6,39E-07
SLC25A21-/	9,074296	7,40E-06
TENM1	9,071485	1,01E-06
PRICKLE2-A	9,066576	7,25E-07
LOC339803	9,06613	1,48E-06
HOMER2	9,061482	2,38E-12
SEMA3D	9,056499	2,16E-06
LINC00664	9,051103	1,47E-06
MB21D1	9,049926	7,96E-07
KRT23	9,045971	7,26E-07
A1BG-AS1	9,045527	2,80E-08
PCDHA13	9,030977	8,19E-07
LINC00665	9,020751	1,11E-20
PCDHGB1	9,019905	3,15E-08
PRTG	8,994931	9,73E-07
MTMR7	8,994558	9,69E-07
CEACAM1	8,952554	4,20E-08
GNG4	8,945217	2,82E-06
MAGEE1	8,944729	1,49E-06
LOC728739	8,943651	1,44E-06
MAP10	8,930739	2,02E-06
ZNF530	8,916482	1,62E-06
FAM81B	8,909213	2,02E-06
ZIC4	8,884681	1,77E-06
SLC24A3	8,884634	5,79E-08
VAX2	8,869128	2,21E-06
SNAP25	8,840921	2,75E-06
ANK1	8,839004	3,57E-05
EPHA4	8,836724	4,75E-06
ARMCX1	8,835459	4,15E-06
RFTN2	8,833901	3,99E-06
TMEM132E	8,826407	0,00011
SLFN11	8,800151	3,26E-06
DOCK10	8,794527	4,82E-06

RAI2	8,794527	4,82E-06
LOC728392	8,777575	7,90E-06
TTC29	8,776723	3,24E-06
ANKRD18B	8,76368	1,22E-07
HLA-DRB5	8,751955	5,43E-06
EGFL7	8,74991	9,64E-07
PAX2	8,736957	8,36E-05
PVALB	8,735846	1,01E-05
NMUR2	8,734073	3,98E-06
ZNF726	8,727807	1,13E-05
TCHHL1	8,717545	0,000184
UNC5C	8,715441	1,29E-05
SOX18	8,698495	4,78E-06
SNAR-E	8,686466	1,16E-05
GATA5	8,684125	5,72E-06
TNNT1	8,672667	5,76E-13
GJA1	8,669515	2,91E-05
WIF1	8,668554	3,43E-05
USP51	8,66771	9,14E-06
PCDH9	8,667105	9,92E-06
LRRK2	8,664269	7,90E-06
IGFL1	8,661223	0,00018
LOC100128	8,64432	6,30E-06
GUCY1B3	8,640412	8,13E-06
SLC6A14	8,637063	7,01E-06
RET	8,628736	1,09E-11
PCP4	8,606194	1,86E-18
FAM196A	8,603386	2,36E-05
ZNF98	8,59805	1,57E-05
F7	8,557288	3,80E-05
DLX3	8,535824	2,08E-05
TARP	8,53361	4,41E-05
PCDHGA4	8,532653	1,37E-05
MAPK4	8,511034	2,31E-06
PACSIN1	8,491847	3,76E-16
C10orf82	8,48449	5,84E-07
PART1	8,484019	1,72E-05
TRIL	8,479313	2,37E-05
FLT3	8,463026	1,62E-05
TRAM1L1	8,454765	4,31E-05
ACTG2	8,453803	3,66E-06
WFDC2	8,438235	3,24E-05
SPOCK1	8,417745	2,63E-05
FOXI3	8,417631	2,21E-05
POU6F2	8,407022	2,42E-05
MYT1	8,405425	6,03E-07
GRID1	8,40477	2,76E-05
EDN1	8,388377	2,04E-15
CDH18	8,379038	2,95E-05
HIST1H3G	8,376053	2,59E-05

GHR	8,374908	2,54E-05
DIRAS1	8,373828	3,34E-30
EFNB3	8,370232	5,64E-12
VIM-AS1	8,365007	3,01E-05
MSRB3	8,363273	2,84E-05
ZNF512B	8,351877	8,11E-45
OLFM1	8,347818	4,05E-27
RNF180	8,335124	4,38E-05
SLIT2	8,331474	3,08E-05
ZNF345	8,329112	3,16E-05
XKR7	8,315214	5,79E-05
MAFB	8,291527	2,40E-20
FIRRE	8,286017	2,47E-06
RIBC2	8,282362	0,000168
LPPR3	8,281734	0,000184
PCDHGA5	8,279224	4,40E-05
SPEG	8,276772	4,15E-05
TH	8,270516	6,45E-05
FBN2	8,265952	9,91E-11
ADAMTSL3	8,264437	5,92E-05
SNCB	8,252471	0,000257
XG	8,240529	0,000477
ICOS	8,239214	5,37E-05
ARL11	8,234025	5,34E-05
LOC101927	8,233508	7,57E-05
LOC440461	8,228883	0,000101
EMCN	8,219916	5,89E-05
EWSAT1	8,216821	9,36E-05
CBFA2T3	8,215246	2,92E-05
ZNF423	8,214804	5,47E-05
PCDHAC1	8,212788	0,000101
GSC	8,210957	0,000174
KCNF1	8,207095	6,83E-05
LINC00885	8,205069	0,000244
ZNF93	8,203395	3,08E-05
IGSF1	8,189363	0,000255
BEX5	8,186029	0,000172
NXPH3	8,179393	9,70E-05
TP53INP1	8,177881	2,02E-24
GPM6B	8,166847	6,91E-05
PCBP3	8,166465	4,16E-11
KCNU1	8,16287	7,67E-05
ZNF730	8,156274	2,14E-06
CYP4F22	8,128845	0,000103
CCSER1	8,128303	0,000102
SGCG	8,120034	0,000198
ALOX12P2	8,117241	0,000271
CALML5	8,116434	0,000139
CPEB1	8,112287	0,000109
RIMS2	8,112287	0,000109

GJB5	8,109574	0,000386
NRK	8,106751	0,000273
PCDHB9	8,102737	4,88E-11
C20orf194	8,099435	2,53E-06
WASF3	8,086964	1,12E-20
ZNF155	8,079296	0,000128
SARDH	8,070754	0,000245
NDNF	8,069411	0,000109
CD36	8,068599	0,000204
DRD1	8,063615	0,00064
C8orf48	8,060014	0,000126
SULF2	8,05757	2,51E-126
CAPN9	8,04568	0,000149
DEFB126	8,034688	0,000129
LOC441666	8,027253	0,000156
SNAR-G1	8,010961	0,000177
PHF21B	7,996037	0,000766
PNMA6A	7,992385	0,000203
KDR	7,990736	0,000174
ZFP3	7,98958	7,19E-28
B4GALNT1	7,969386	2,17E-20
HOXC13	7,968536	1,53E-18
LRP2	7,967447	5,32E-17
FLRT3	7,957453	2,43E-63
BLNK	7,945738	5,12E-05
NKILA	7,945582	0,000195
IBSP	7,94481	0,000198
RDH16	7,944641	3,35E-18
PABPC4L	7,938604	0,000256
SLC5A5	7,927442	0,000409
PARVB	7,921669	4,77E-23
PAPLN	7,919696	0,000285
IFI44L	7,918364	6,51E-05
KCNJ4	7,916388	0,000233
LINC00494	7,910079	0,000225
PMEPA1	7,909712	4,23E-53
CEACAM5	7,909187	0,000461
LRRIQ1	7,907705	0,000235
DAAM2	7,890793	0,000246
NAP1L2	7,888258	0,000434
CA4	7,883551	0,000311
ZNF790-AS	7,877029	4,39E-10
ZNF547	7,873679	0,000262
EDIL3	7,873623	0,000692
TIMP3	7,868399	7,09E-10
VAV1	7,853819	8,16E-10
ZNF304	7,846697	2,68E-30
ALOX15	7,840508	1,73E-18
PTPRG-AS1	7,839038	1,66E-14
NDRG4	7,83144	1,31E-12

ZNF529-AS	7,813502	0,000342
BRINP2	7,809125	0,000589
COL3A1	7,806554	0,000504
GPC3	7,797252	0,000757
PIK3C2G	7,793659	0,000941
C8orf46	7,790297	0,000394
FAR2P1	7,786166	9,55E-09
MB	7,778151	1,14E-17
PCDHB10	7,75852	1,18E-05
ZNF793-AS	7,754229	0,000443
PCDHGB3	7,752468	0,000445
ARHGDIB	7,746177	0,000286
C17orf51	7,721037	0,000672
FILIP1L	7,691498	0,000582
LRFN5	7,690579	0,000579
DPY19L2P1	7,6765	0,000742
FHL1	7,672767	0,000668
FNBP1	7,670933	4,39E-54
PACRG	7,66248	0,000677
PLXNA4	7,652195	2,27E-15
SOX11	7,650953	0,000739
PDIA2	7,643364	0,000703
KRT81	7,638751	1,38E-10
MEIS1	7,635725	0,000854
ST8SIA1	7,626879	0,000787
C19orf81	7,619254	2,32E-05
TNFRSF11B	7,619164	0,000783
FGF13	7,611388	0,001003
DCLK1	7,606463	4,56E-40
JPH3	7,605847	3,79E-09
HAGLROS	7,59848	0,000839
PTPRQ	7,596517	0,000855
SLCO4C1	7,591592	0,000962
PDZD4	7,583032	1,25E-12
ABCC8	7,575503	0,000921
LHX2	7,568875	3,48E-05
GATA3	7,541784	7,07E-67
KIF5C	7,49482	4,58E-05
SALL4	7,456777	2,38E-11
AMOTL1	7,449595	8,61E-40
CCL5	7,430154	6,93E-07
MAP1B	7,412889	1,54E-24
PCDH19	7,411947	8,33E-05
ZNF614	7,364365	3,09E-28
MYLK	7,3594	6,76E-05
LOC10013C	7,356492	7,43E-05
FBXO27	7,353658	7,74E-34
FCHO1	7,339099	5,90E-67
CRLF1	7,327358	5,00E-08
RGAG4	7,309785	8,19E-08

PCDHGB2	7,275853	0,000113
DNAJA4	7,252774	9,99E-33
SEMA3C	7,243898	2,39E-132
ATRNL1	7,176054	0,000143
SLC25A21	7,172936	0,000148
MARCH1	7,171825	2,55E-07
UPK3B	7,167938	0,000494
RIMKLB	7,164873	6,36E-34
TUSC3	7,163633	1,24E-45
AP4B1-AS1	7,151144	2,34E-20
SPTSSB	7,125687	5,26E-29
CERS4	7,118084	3,55E-17
PCDHB2	7,116907	4,92E-38
TMEM37	7,109778	7,43E-26
KCNMA1	7,106614	4,83E-07
DNALI1	7,1043	0,000197
ADAMTS19	7,091058	1,13E-47
TBC1D9	7,084568	1,31E-90
DHRS2	7,039216	6,60E-39
HIST1H2AE	7,032101	0,000765
PRICKLE2	6,999566	1,31E-51
KIAA1324	6,969253	4,80E-06
CYP1A1	6,948864	8,81E-09
DPYSL5	6,907482	2,56E-21
PREX1	6,893009	4,86E-78
APOA1	6,880846	0,000466
ZNF467	6,864893	1,55E-45
GATA3-AS1	6,864765	0,00052
NUP210L	6,8636	6,61E-25
ZNF302	6,808967	6,23E-27
MRI1	6,794372	1,68E-36
WIPF1	6,754245	0,000702
SH3GL3	6,751788	2,39E-06
LDHD	6,716967	3,84E-07
GNAS-AS1	6,713782	0,000798
CCL2	6,696965	1,08E-08
CSTA	6,696502	1,28E-09
TDRD1	6,691593	1,05E-25
SYT5	6,68305	0,00091
TLX1NB	6,67414	0,000962
RASSF2	6,650467	2,17E-08
FLT4	6,632761	1,82E-05
RND2	6,627384	6,77E-18
PTPRS	6,553692	2,18E-50
HBA1	6,546274	3,01E-07
HSPA1A	6,539755	6,22E-27
FAM101B	6,530443	8,54E-06
CHST11	6,527232	3,35E-41
PCSK1N	6,523186	1,16E-37
MDK	6,500897	1,19E-62

ECHDC3	6,488805	1,38E-10
CAMK2B	6,478044	3,40E-12
NFATC1	6,469721	5,12E-12
SLC8A1	6,451777	6,15E-15
IGF1	6,439017	9,44E-06
OLFM2	6,429342	1,32E-23
C2orf74	6,410526	6,73E-08
RNF182	6,408051	6,47E-18
PCED1B	6,400561	2,98E-05
VIM	6,377832	2,83E-05
UPK2	6,375548	2,93E-08
HCN2	6,369543	1,61E-26
BCL11B	6,365305	3,26E-53
BMP2	6,32244	6,73E-47
TIGD7	6,319351	3,94E-05
GNG13	6,316984	6,33E-07
SHANK1	6,303675	2,87E-06
SHC2	6,29574	9,38E-05
TUBB4A	6,294287	8,72E-09
GREB1	6,264173	1,03E-75
NODAL	6,256146	0,000102
WDR72	6,245963	3,43E-27
CD24	6,234964	1,68E-79
FGD1	6,229035	2,35E-23
ETNK2	6,220881	2,28E-37
KCNN4	6,211672	2,51E-10
ST8SIA4	6,210086	1,98E-16
RHOBTB1	6,181665	5,28E-129
HOXC10	6,179927	1,55E-39
HPN	6,178262	3,70E-18
ZNF610	6,173175	7,21E-05
INPP4B	6,157576	3,42E-40
NELL2	6,15547	0,000168
UCP2	6,144526	9,20E-100
ZNF114	6,134301	2,40E-23
PCAT6	6,130764	9,11E-05
ZNF550	6,111569	4,20E-05
A1BG	6,109214	6,00E-10
PCLO	6,107469	4,67E-30
LYPD3	6,100447	7,23E-16
SLC22A31	6,066771	3,11E-07
RPP25	6,056751	5,61E-37
IGFBP3	6,027321	2,59E-07
TRPS1	6,021314	4,87E-84
PKIB	6,019833	3,49E-56
VSTM2L	6,017575	0,000115
RENBP	6,007876	0,00015
PRAME	5,999086	1,16E-12
NPR3	5,998584	1,12E-23
CALD1	5,987135	4,60E-09

FAM20C	5,975162	5,27E-20
LCP1	5,910377	1,10E-27
ABCB6	5,90875	9,43E-54
HTRA1	5,902424	5,78E-22
C1orf233	5,900041	2,11E-55
TBX2-AS1	5,897034	1,12E-11
FAM69B	5,888908	1,11E-12
LINC00886	5,88871	8,81E-14
IGSF5	5,850408	3,13E-07
FGFR4	5,850092	4,03E-54
PCDHGA1	5,820413	0,000259
SIDT1	5,818504	1,08E-15
COL9A2	5,811118	6,32E-14
LINC01006	5,809716	8,94E-06
FAM198B	5,809324	1,39E-19
KHDC1	5,756928	0,000906
PALM	5,717439	2,37E-19
DLGAP3	5,713587	3,76E-15
KIF1A	5,695326	1,04E-46
YBX2	5,673939	6,64E-50
ITGA2B	5,670269	1,05E-09
IFI27	5,664103	2,01E-06
ENTPD1	5,65227	5,82E-14
JAM2	5,634845	9,83E-05
TTC28	5,633596	8,77E-12
CYP1B1	5,623458	2,10E-24
LRIG1	5,613511	1,48E-10
C14orf37	5,584641	2,82E-06
MANSC4	5,543078	2,27E-05
NRSN2	5,541817	4,08E-23
TTN-AS1	5,539068	0,00099
ARC	5,535545	1,98E-07
B3GALT4	5,522215	2,63E-05
BCAS3	5,51217	1,55E-60
NPAS3	5,486139	0,0005
HHAT	5,456927	7,72E-07
COL18A1	5,4492	1,48E-58
PLXDC2	5,435939	6,28E-33
TMEM229I	5,425635	1,09E-16
FRMD6-AS	5,414243	6,61E-07
TEX19	5,40905	3,34E-08
LINGO1	5,392236	1,40E-49
KIAA1324L	5,391856	5,38E-47
P3H2	5,34857	2,28E-16
ZBTB7C	5,345057	4,74E-07
CNIH2	5,33844	5,23E-19
PROM2	5,323381	2,98E-28
APCDD1	5,276923	1,87E-18
KLHL13	5,259367	5,21E-09
TNFAIP8L3	5,247228	1,10E-20

GPNMB	5,240145	5,19E-25
MCC	5,234503	4,27E-08
ATP6V0A4	5,212878	0,000677
CYP24A1	5,153249	1,19E-07
MAPK8IP1	5,148213	7,38E-23
RINL	5,147567	9,76E-09
GNG7	5,143712	1,59E-08
PLIN2	5,121788	4,88E-10
TMEM150(	5,120575	2,04E-16
P2RY2	5,111378	9,19E-23
ANKRD6	5,109146	6,17E-05
CACNA2D1	5,09358	7,90E-16
HES2	5,086522	1,93E-09
WNT6	5,061169	1,15E-05
RBMS1	5,053348	2,23E-38
LYPD1	5,050464	5,55E-10
DEF6	5,028628	4,49E-16
FAM109B	5,020481	3,04E-14
MAP2K6	5,017535	1,41E-05
CKB	5,010472	6,71E-27
FAM46C	4,959239	1,28E-12
OGDHL	4,950264	6,12E-21
SPATA17	4,946956	2,94E-11
SIX1	4,916993	1,45E-24
ABLIM3	4,909931	5,94E-05
ECHDC2	4,896349	3,46E-11
ARHGAP4	4,890123	1,04E-10
ERG	4,881965	2,55E-06
HS6ST3	4,87939	0,000818
RIPPLY3	4,875492	5,48E-05
STON2	4,823918	1,13E-34
C16orf45	4,811901	2,12E-05
OAS2	4,81111	0,000316
PCDHGA6	4,807086	0,000694
BMF	4,806099	9,05E-07
CELF5	4,802362	2,76E-07
RGS14	4,791011	3,46E-18
HIST1H2BH	4,774529	0,000228
SLCO3A1	4,772043	8,41E-09
KRT17	4,768338	1,22E-06
SUSD2	4,750213	2,31E-09
HLA-DQB1	4,744778	2,55E-07
SOCS1	4,744096	3,04E-12
RGS17	4,736385	0,000332
ZNF433	4,732872	4,27E-09
EEF1A2	4,695411	2,03E-05
RUNDC3A	4,674135	0,000168
C2CD4C	4,665505	0,000831
HMCN1	4,660331	0,000213
NEB	4,659666	2,85E-33

OLFML3	4,658768	0,000124
TCEA3	4,656823	1,43E-35
LOXL1-AS1	4,653322	2,19E-05
KRT86	4,645182	0,000124
CACNG4	4,641652	5,18E-34
TSPEAR	4,612706	7,76E-08
SEMA6A	4,603548	1,92E-13
TP53TG1	4,603482	7,15E-23
ABCG1	4,601026	8,80E-08
CMTM3	4,594483	1,58E-08
CHRM1	4,591863	1,10E-09
LOX	4,577035	0,000127
GLUD2	4,576987	1,17E-15
ST3GAL1	4,572296	7,87E-20
FOXA2	4,568752	0,000115
ZNF461	4,553474	7,78E-08
HOXD12	4,552545	0,00071
RCN3	4,544247	4,46E-05
MUC1	4,541145	4,01E-15
LONRF2	4,515429	7,15E-21
SERHL2	4,502201	0,000314
CDYL2	4,494359	2,30E-13
LOC72855[illegible]	4,488191	1,62E-21
FBP1	4,479201	3,89E-62
SEC31A	4,461775	1,15E-45
AK7	4,449337	5,37E-34
ISYNA1	4,448357	6,17E-27
FSTL4	4,405357	1,49E-06
CTXN1	4,391017	2,50E-57
TINCR	4,389555	1,93E-11
DDAH2	4,383248	1,42E-31
ARHGEF10	4,382559	1,67E-10
ZNF701	4,359647	2,36E-21
USP32	4,354479	1,79E-34
FXYD3	4,342717	1,55E-11
PFDN4	4,341927	1,88E-62
AEBP1	4,331768	1,53E-05
LMO2	4,319251	0,000739
S100P	4,310627	0,000365
KCNJ3	4,306773	2,44E-07
TUBA1A	4,28584	6,16E-51
STMND1	4,277598	2,89E-05
SERPINI1	4,275834	2,13E-06
HOTAIR	4,272042	4,45E-05
MEX3B	4,264261	2,74E-37
ZNF385A	4,252423	2,37E-54
REPS2	4,247116	7,19E-07
COL6A1	4,235951	3,69E-09
CEL	4,220445	1,28E-05
NOTCH3	4,208004	1,53E-57

HCN4	4,154597	2,09E-07
TFAP2C	4,151628	2,15E-44
SOX4	4,131974	1,98E-54
PADI2	4,119553	2,93E-06
IGSF9	4,117282	2,13E-52
RN7SK	4,108843	2,47E-05
LOC101926	4,103044	0,000852
KLHDC8B	4,072817	1,17E-06
NFE2	4,072811	3,81E-11
ATOH8	4,058612	2,51E-09
FEZ1	4,045256	1,28E-05
PCDHB14	4,036226	2,02E-13
PLCB1	4,03491	2,46E-17
STARD13	4,018847	7,35E-14
KCNH3	4,013305	0,000237
FRMPD1	4,011254	2,91E-05
SMAD6	4,005844	7,10E-14
IGSF10	4,003827	0,000241
RASD1	4,002678	0,00027
SLC30A3	3,988291	2,14E-05
MEX3A	3,974337	2,65E-62
PGM2L1	3,960737	1,24E-29
DNAAF3	3,955156	1,80E-19
SOCS3	3,950272	1,12E-08
AMOT	3,947028	3,69E-26
TMEM254-	3,913301	6,33E-05
PBX1	3,879096	2,25E-07
PLEKHO1	3,877206	1,08E-12
PLXNB3	3,876029	1,26E-14
GRHL3	3,84583	1,46E-18
FRMD6	3,843759	1,00E-28
SHF	3,843294	4,08E-09
HOXC11	3,828864	3,96E-10
MGLL	3,812111	1,11E-08
FAM171A2	3,808234	1,59E-21
RAB30	3,774609	6,30E-20
NREP	3,763561	6,96E-19
RASSF10	3,755065	1,12E-05
GTF2I	3,754515	5,33E-49
AMIGO2	3,744839	1,27E-07
ZMIZ1	3,719394	1,17E-38
FGFR3	3,71562	7,15E-18
AKR1E2	3,70362	2,90E-06
BOLA1	3,700583	5,98E-22
DLX4	3,695904	5,99E-05
MSH6	3,685478	1,03E-38
HECW2	3,67324	2,00E-10
CNPY4	3,668934	2,61E-12
DBN1	3,668313	8,29E-61
KDM5B	3,661349	4,90E-31

MAML3	3,654701	1,01E-07
DMKN	3,649759	5,78E-09
BAMBI	3,639699	2,47E-36
LHX1	3,629231	2,73E-05
ALDOC	3,626278	3,60E-13
NCOA3	3,623819	3,65E-41
PDE4A	3,608944	2,13E-08
CCNG2	3,6038	2,86E-12
HSD17B14	3,597977	0,000129
ASPHD1	3,59734	1,92E-09
SAMD11	3,588226	3,54E-07
PPM1D	3,57981	7,70E-43
GADD45G	3,575317	5,52E-05
NRXN3	3,565532	7,42E-06
ZNF836	3,564832	7,75E-09
SCIN	3,551562	0,000742
HR	3,550387	8,57E-18
FN1	3,546392	0,000278
RASGEF1A	3,545901	1,81E-06
HSPA2	3,538783	1,96E-07
LGALS1	3,498965	2,55E-13
SERTAD4	3,494699	2,62E-14
PSMD6	3,487285	1,17E-46
TBX2	3,482634	1,71E-14
CCDC83	3,479458	0,000355
FAM227B	3,475075	1,93E-07
SIAH2	3,470894	2,20E-29
SEMA4A	3,469472	1,04E-18
PTCHD2	3,467411	0,000549
SLC9A3R2	3,464996	4,71E-32
LTBP2	3,458579	0,000288
SEZ6L2	3,4559	2,67E-18
CRABP2	3,448691	1,24E-14
LFNG	3,445389	1,83E-12
CERCAM	3,410795	2,07E-15
NPW	3,408886	0,00029
PTP4A3	3,403682	1,25E-10
ESRP1	3,40324	4,31E-11
TMEM121	3,389567	2,79E-06
WNT11	3,377137	3,33E-05
RTN1	3,376443	0,000681
FAM72B	3,374941	3,37E-09
STARD10	3,37054	6,40E-19
TNS1	3,346603	5,26E-08
CYP2J2	3,344374	3,58E-09
APOE	3,329936	2,30E-06
RCOR2	3,328888	1,36E-09
GSTA4	3,323097	5,63E-12
ASAH2	3,320009	1,18E-12
ZFYVE28	3,319145	4,39E-10

BRIP1	3,312826	1,10E-38
CACNB3	3,306518	2,31E-32
ATP8B2	3,296884	1,40E-10
NANOS1	3,296503	6,46E-18
PBXIP1	3,264187	1,25E-07
CCDC71L	3,256252	1,35E-12
MUC3A	3,25475	0,000441
SCAMP5	3,253648	2,32E-10
PCDHB13	3,251279	8,95E-10
CCDC170	3,244558	2,82E-07
ZNF704	3,241048	1,03E-18
MEIS3P1	3,229736	9,08E-09
NR3C1	3,227149	5,17E-31
NKAIN1	3,226665	8,30E-16
PRSS23	3,220837	6,41E-18
SYNE4	3,205183	6,26E-25
PLCH1	3,203041	2,80E-22
DAB2IP	3,198741	4,76E-17
APPBP2	3,198527	4,62E-32
PCAT7	3,19382	0,00056
GATS	3,178096	9,69E-10
FUT8-AS1	3,173703	0,000206
ARHGEF19	3,171564	5,06E-13
GLUL	3,164777	2,70E-48
KRT8	3,160935	5,20E-20
TET1	3,152902	6,77E-07
MECOM	3,139919	2,17E-10
C15orf65	3,13038	2,17E-05
DYRK1B	3,128045	4,41E-21
SESN3	3,121744	0,000966
AHR	3,121385	2,40E-28
RUNDC3A-/	3,120855	5,74E-05
TMPRSS13	3,11984	1,59E-10
ADAMTS7	3,111694	8,85E-06
ZNF561-AS	3,108101	6,32E-05
KCNC3	3,094988	2,04E-17
PITPNC1	3,091805	2,34E-30
CPE	3,08921	6,45E-05
SYT17	3,079496	7,51E-12
LEPR	3,0787	0,000419
PRRT3	3,07029	1,31E-05
SLC9A2	3,067826	1,21E-24
TRIM37	3,067492	9,83E-35
MXD4	3,067295	1,83E-12
SLC22A23	3,066029	1,03E-27
ODAM	3,04524	4,34E-08
RASGRF1	3,040461	1,01E-14
KRBA2	3,027438	4,11E-05
ACTL8	3,016977	0,000302
FGD3	2,995601	2,85E-06

ENKD1	2,988601	0,000571
PRMT6	2,974717	1,61E-36
C1QTNF9B-	2,973895	1,48E-08
LINC01116	2,970236	2,12E-05
FAM102B	2,969715	3,48E-27
C7orf13	2,967974	1,82E-10
HEATR6	2,963007	3,25E-32
SLC29A4	2,962819	3,35E-17
MSRB2	2,962748	3,68E-16
FAM46B	2,961937	1,80E-05
HIST1H2BD	2,938742	7,90E-07
TUBB2B	2,93684	1,08E-08
SRGAP2C	2,93017	8,17E-05
PHLDB3	2,911173	3,23E-07
KRT7	2,905324	7,20E-05
GSTZ1	2,902976	1,64E-19
LRRC75B	2,902641	4,05E-09
NEURL1B	2,890767	9,38E-07
COL1A1	2,886645	1,04E-07
RNF122	2,884444	6,01E-10
ADSSL1	2,880815	0,000631
MEPCE	2,872996	1,05E-33
TGFBR3L	2,87132	0,000122
DDB2	2,867714	7,79E-12
MCTP1	2,865381	2,62E-07
TRIQK	2,858281	6,27E-08
POLD4	2,854896	2,17E-06
METRN	2,845581	8,15E-20
LINC01521	2,833603	1,02E-06
ZNF217	2,831973	1,18E-20
C15orf40	2,823084	4,25E-11
ZNF253	2,819718	1,67E-13
ZNF185	2,813847	1,08E-07
IFI6	2,809785	8,81E-05
MLPH	2,808749	3,70E-14
SLC43A2	2,807535	2,42E-16
ZNRF1	2,804153	3,41E-22
LHX4	2,802788	7,20E-17
LAGE3	2,802747	1,76E-21
GGT1	2,801005	4,41E-13
TUBD1	2,795148	1,93E-12
IDH2	2,788991	6,50E-26
SEMA3F	2,783946	1,60E-09
TEAD2	2,783527	4,94E-14
CASC10	2,782826	9,19E-05
LOC10023:	2,782714	5,09E-06
CBLB	2,782369	1,81E-15
ZNF137P	2,777477	0,000168
TUBB3	2,774882	1,59E-27
TFAP2A	2,773967	9,20E-24

IGFLR1	2,772741	6,09E-07
LTBP1	2,76104	2,43E-18
CPS1	2,760692	9,23E-06
CREB3L4	2,759025	1,47E-17
MAML2	2,750403	0,000198
RAB26	2,749224	0,000194
SLC6A8	2,747768	8,41E-09
PIK3R3	2,747598	1,04E-13
GTF2IP1	2,744989	3,06E-09
BCAS2	2,736996	4,17E-28
ZNF841	2,735691	1,51E-10
CKMT1A	2,734473	1,65E-13
GATSL3	2,732449	0,00024
MAPK11	2,729275	1,29E-06
MYLIP	2,728572	3,48E-10
RAB3D	2,727404	3,13E-14
EFR3B	2,721988	5,26E-08
BRSK1	2,721709	7,42E-05
CABYR	2,705934	5,15E-05
TNFRSF19	2,705738	1,22E-15
NHP2	2,70191	1,08E-22
KCNIP3	2,700377	0,000205
PDGFA	2,694711	4,05E-05
FZD2	2,686632	4,86E-09
KLF7	2,685271	2,55E-09
CTSO	2,681382	0,000901
FAM213A	2,677698	1,55E-14
SP6	2,675557	2,73E-11
RPS27L	2,675198	3,26E-06
PRR36	2,673963	3,34E-25
ITPKB	2,673589	4,93E-12
LAPTM4B	2,673011	5,17E-32
ZNF75A	2,666803	6,00E-06
RBBP8NL	2,660834	1,01E-10
MOSPD3	2,658567	1,53E-10
TMEM183E	2,657206	2,79E-09
SLC27A5	2,655741	2,33E-13
AHNAK2	2,654458	1,31E-06
DTWD1	2,653744	3,79E-14
PXN-AS1	2,644129	7,14E-08
ZNF268	2,6282	1,05E-09
APLP1	2,619917	1,86E-05
HBP1	2,616732	1,09E-08
DNLZ	2,61035	5,58E-11
ADAM11	2,601448	4,23E-05
COX6C	2,600954	1,98E-15
CCDC92	2,582561	4,87E-07
MRPS21	2,578293	6,02E-26
KREMEN2	2,575645	6,82E-06
GTF2IRD2B	2,574951	5,03E-09

C6orf52	2,566656	0,000886
RAP2B	2,564386	4,18E-10
KCTD1	2,561208	1,65E-12
ZNF429	2,560384	1,36E-06
RPS6KB1	2,547195	8,47E-27
SHANK2	2,535575	1,53E-09
KRT18	2,533286	1,79E-10
ZBTB42	2,531107	2,70E-14
FAM214A	2,51575	7,95E-06
ADRA2C	2,515029	6,12E-07
FZD1	2,514159	4,40E-10
VGF	2,510396	2,04E-08
CSDE1	2,507477	3,42E-22
C9orf3	2,505908	1,69E-11
SH3BGR	2,503648	3,77E-05
HARS2	2,491896	1,57E-13
DAAM1	2,486114	5,63E-17
N4BP3	2,48438	7,43E-09
ZNF480	2,483982	5,51E-16
TRAPPC6A	2,48269	2,41E-07
PACSIN3	2,475254	4,26E-21
SBK1	2,474692	2,09E-21
TNIK	2,469378	1,24E-06
ATP9A	2,461277	3,91E-17
RHOB	2,457521	6,73E-18
NES	2,457435	9,14E-08
PHLDA3	2,456692	1,57E-05
FLJ23867	2,456412	2,46E-09
CROT	2,443204	0,000108
FAM222A	2,441327	5,78E-14
LRRC24	2,439169	0,000709
PNRC1	2,430847	5,81E-09
ZNF432	2,425041	3,47E-06
LOC14870	2,423091	0,000257
TNKS2	2,422716	4,99E-15
GPRIN1	2,422197	2,88E-18
PVRL4	2,421639	7,23E-07
FRAT2	2,416655	2,59E-05
ERV3-1	2,404414	1,04E-06
S100A13	2,403234	3,60E-15
SYNE2	2,397411	1,13E-09
MNX1-AS1	2,386837	5,52E-06
ARSG	2,376597	4,34E-07
SELENBP1	2,374701	3,54E-13
ZFP14	2,37148	6,56E-07
THOC7	2,367109	2,05E-23
FRMD4A	2,365184	1,80E-06
LOC10496	2,361978	1,29E-05
NRP1	2,356551	1,38E-10
PKP1	2,355715	0,0004

MNS1	2,351588	1,06E-06
CASKIN1	2,350682	3,95E-07
POLR2J	2,348731	2,18E-14
IRX3	2,339271	9,27E-15
TFPT	2,335823	2,16E-09
BCL6	2,334624	1,93E-09
SPPL2A	2,324294	1,12E-19
RNF223	2,324268	1,54E-05
CLDN3	2,321568	6,66E-17
IFT22	2,319629	2,26E-13
TBCK	2,318156	1,02E-10
CBX2	2,316004	3,05E-15
PDCL3	2,313624	9,69E-15
TMEM254	2,311588	7,85E-09
PRKAR2B	2,304774	2,47E-05
NME4	2,304597	4,54E-21
KRT80	2,303699	2,60E-12
RAC3	2,303222	1,08E-07
WWTR1	2,301692	5,70E-13
RGL1	2,30125	5,65E-05
ARID5B	2,297072	8,95E-15
ALKBH2	2,291298	3,03E-13
DHTKD1	2,290889	1,12E-14
EML1	2,282271	2,16E-13
LOC10012‹	2,275918	6,32E-06
TAGLN2	2,270945	3,46E-18
RASA3	2,268366	0,000118
PRMT2	2,267834	2,36E-07
SOS2	2,264486	2,29E-08
F12	2,261407	1,06E-16
WNT7B	2,257137	3,97E-06
DNAJC15	2,256914	3,17E-16
ZSCAN2	2,256555	2,68E-12
SUMO3	2,255926	7,16E-21
ZNF425	2,255357	0,000282
IMMP2L	2,250999	7,42E-06
NINL	2,24891	4,44E-09
WDR83OS	2,239316	1,14E-17
CADM4	2,235474	9,08E-06
LPPR2	2,233879	3,69E-06
PAQR8	2,232041	0,000834
PPFIA3	2,220904	2,58E-13
ALDH4A1	2,218812	0,000187
TMEM183/	2,217742	1,31E-17
RAB13	2,212687	3,35E-13
TMEM168	2,211732	1,53E-14
RAD51C	2,197182	3,13E-20
EFHD1	2,185643	5,08E-06
MSX2	2,185616	2,71E-09
TLE3	2,182663	1,33E-18

EFCAB11	2,181371	1,60E-08
S100A11	2,177321	5,67E-09
FUT8	2,174651	4,87E-12
NXPH4	2,168293	3,13E-05
TMTC2	2,157862	2,27E-05
PPDPF	2,155907	7,23E-11
LIPT2	2,155063	0,000293
C7orf73	2,149084	6,62E-17
RNASEL	2,148772	0,000245
NHS	2,148158	9,92E-06
RASSF8	2,147772	4,24E-07
GOLGA7B	2,147141	0,00099
EFNA4	2,145185	2,45E-12
RRBP1	2,144279	1,71E-10
ANKRD34A	2,142221	2,71E-06
ZNF608	2,140031	2,84E-09
ZNF444	2,136005	1,83E-12
ROGDI	2,13581	1,38E-09
MRPL41	2,133335	2,42E-15
TRMT12	2,133202	2,07E-11
IFT46	2,131964	4,58E-12
PMS2P1	2,126042	1,96E-09
NBPF15	2,122796	3,13E-09
GABPB1-AS	2,116376	1,30E-05
TMEM187	2,11628	6,52E-06
ERMP1	2,114961	3,20E-09
HID1	2,112659	1,35E-06
TRIM45	2,107145	7,75E-06
MAFG-AS1	2,106106	9,45E-05
TSPAN31	2,102939	6,38E-06
TP53I11	2,101767	1,02E-05
ZNF354A	2,101188	3,57E-08
PMVK	2,096245	3,78E-09
VTI1B	2,09518	1,24E-12
MAPT	2,095157	3,93E-09
ZBTB12	2,092676	3,35E-05
LTBP3	2,092319	2,39E-13
HSD17B8	2,085771	1,58E-05
ZNF256	2,085482	0,000108
OARD1	2,084344	6,60E-09
ENTPD2	2,082333	2,91E-05
KRT19	2,080534	1,04E-07
TATDN1	2,079975	1,10E-14
CRISPLD2	2,070953	3,33E-05
ZNF703	2,070931	1,46E-13
TMEM191A	2,069816	1,28E-07
TRIP6	2,067017	3,20E-13
GRAMD1A	2,066487	1,55E-10
GLMP	2,059114	2,35E-05
PPP2R3A	2,057513	8,18E-08

ABCA12	2,056278	1,85E-05
TDRKH	2,055866	4,13E-07
FKBP10	2,054801	2,74E-13
WWP1	2,052467	5,21E-07
CADM1	2,051976	1,08E-08
DCXR	2,050427	8,82E-11
HIST1H2BK	2,050153	0,00053
SARS2	2,046481	1,93E-12
C17orf97	2,039945	1,28E-05
DCLRE1B	2,03847	4,26E-10
COQ2	2,036807	5,89E-10
FAM89B	2,035774	2,82E-13
CHD3	2,034377	2,23E-16
RNF115	2,032703	1,41E-15
MAST1	2,031765	0,00051
HCFC1R1	2,029297	9,41E-13
SYT7	2,028767	3,50E-10
TMEM205	2,027776	2,47E-08
SLC38A6	2,027433	0,000537
OSR2	2,025339	0,000224
ENDOV	2,021541	1,66E-08
PRADC1	2,017269	2,36E-05
NUDT1	2,017222	1,14E-08
DBNDD1	2,014362	2,13E-07
HKR1	2,013952	7,92E-06
ECH1	2,012774	1,70E-10
LOC101927	2,011551	0,00019
SPIRE2	2,010604	0,000576
SLC9A3R1	2,00879	6,37E-10
ZSCAN21	2,007665	5,01E-07
RMDN1	2,007334	9,61E-12
DSP	2,005925	2,80E-16
GNAS	2,005916	2,66E-18
PVRL1	1,99673	3,68E-07
SP5	1,995568	2,73E-09
HMGB3	1,989702	5,09E-14
IGF1R	1,987256	7,23E-14
FBXW9	1,981539	1,30E-08
IQCH-AS1	1,976484	1,48E-06
LRBA	1,973738	3,91E-10
ARL4A	1,970245	9,17E-13
FIS1	1,970241	5,42E-12
ACOX3	1,965444	8,36E-08
THSD4	1,963101	6,91E-05
ENY2	1,961198	5,38E-11
LOC11323(	1,95364	9,02E-06
BCL2L1	1,953534	1,01E-09
DNMT3B	1,952832	7,25E-08
C21orf58	1,95129	0,000128
DNMT3A	1,950203	4,31E-13

SIPA1L2	1,949488	2,45E-08
PACS1	1,948342	1,52E-09
CTNNA1	1,944404	9,23E-13
AGA	1,941804	0,000352
MACROD1	1,941347	7,69E-08
PTMS	1,939811	3,35E-06
KCNJ8	1,93809	7,07E-10
TMOD3	1,93042	2,42E-16
SWI5	1,930162	6,18E-09
ICAM3	1,928823	1,44E-05
TP53INP2	1,922141	0,000325
DUSP23	1,921157	1,24E-09
SMTN	1,918715	1,79E-08
ZNF362	1,91581	0,000295
TIGD6	1,914343	0,000417
CYB561	1,913291	5,69E-17
BBS10	1,913109	3,63E-07
NDUFB9	1,911369	3,16E-10
ZNF45	1,907926	8,07E-05
TBC1D7	1,904742	5,50E-08
NRSN2-AS1	1,90197	0,000172
TRAPPC2L	1,90124	1,28E-12
TLCD1	1,899952	2,80E-11
CINP	1,895453	7,81E-07
RMND5B	1,893666	1,44E-12
RABIF	1,890572	4,23E-08
MAPK6	1,889076	2,36E-12
CD44	1,883597	7,04E-09
FRG1BP	1,882252	0,000126
CDH3	1,882009	5,08E-16
TET2	1,881772	2,40E-11
RNF44	1,879093	9,07E-12
EPHB4	1,878455	1,92E-11
RAB1B	1,875401	1,48E-07
ATMIN	1,873042	6,19E-15
TRMT2B	1,872815	7,49E-09
SOX13	1,868772	2,16E-05
SRGAP2	1,867643	4,08E-13
SLC39A4	1,867285	5,56E-05
TPD52L1	1,866376	9,31E-07
CCDC90B	1,866257	5,03E-09
KANSL1L	1,86337	1,70E-05
ZMAT2	1,861309	3,61E-13
CLU	1,860595	3,97E-06
NBPF3	1,8582	0,000286
MGMT	1,856061	9,75E-07
PCOLCE	1,855709	0,000867
AP4E1	1,853515	2,65E-09
SNRPE	1,852026	4,73E-15
ICK	1,847171	1,17E-10

NRAS	1,844342	5,50E-10
MYL12A	1,840874	8,75E-14
CCDC167	1,839764	3,56E-05
TAOK3	1,839699	1,75E-09
SLC2A4RG	1,837495	5,63E-12
TRIB1	1,837122	6,18E-08
ZKSCAN1	1,830122	1,44E-07
MIR3654	1,828134	2,73E-06
ZSCAN16	1,825506	2,87E-05
VPS72	1,824846	4,77E-07
SMKR1	1,824638	0,000181
KNOP1	1,818542	2,63E-12
APRT	1,813793	4,09E-15
SH3BP5	1,804929	6,10E-12
CPLX1	1,802996	0,000162
MTCH1	1,801262	3,47E-13
NELFCD	1,800225	9,31E-10
ACOT13	1,798676	7,91E-08
ATP6V0E2	1,79801	1,17E-06
TM7SF2	1,797482	0,00024
ZNF117	1,797461	0,000381
DECR1	1,796917	5,26E-09
PDE4DIP	1,796274	1,50E-06
GPR89A	1,793741	2,24E-05
ZFP1	1,792998	3,23E-07
ZNF713	1,790172	0,000171
STRADB	1,788542	1,12E-06
DTD1	1,787222	1,25E-09
FLYWCH2	1,787064	1,10E-07
PVT1	1,785883	7,39E-06
TAF11	1,782188	4,18E-09
NMB	1,781585	0,000632
ATXN7L3B	1,7778	1,24E-12
CPT1A	1,772883	3,92E-09
HSPB1	1,77105	8,61E-06
TMED3	1,768335	2,79E-10
SLC25A13	1,76752	2,20E-05
PAK4	1,767322	1,04E-10
SYAP1	1,764188	9,51E-11
ARMCX6	1,763829	1,58E-07
MARCKSL1	1,763008	1,59E-07
ZNF14	1,761375	0,00016
SSNA1	1,756858	1,14E-08
KITLG	1,755231	3,03E-09
PRUNE	1,751543	1,68E-10
RBM8A	1,749949	2,26E-12
TMEM64	1,748295	3,77E-11
DUS4L	1,747943	2,06E-07
SLC27A3	1,746367	2,36E-09
ANKRD50	1,741919	2,24E-09

ULK1	1,731917	5,96E-10
ZNF3	1,730934	5,62E-10
SIRT5	1,729687	1,14E-07
ISOC2	1,727203	5,27E-12
NSMCE2	1,727129	7,57E-07
GALK1	1,727076	6,31E-07
ALG8	1,726595	5,26E-06
HILPDA	1,725214	3,60E-05
ZNF252P	1,723638	2,32E-06
SKIL	1,720442	1,96E-06
TRIM24	1,719404	5,06E-10
SLC25A29	1,716929	1,06E-06
RP9	1,715468	3,64E-05
ZNF688	1,714818	8,07E-05
ZNHIT1	1,711899	1,87E-05
ZMYND8	1,711583	8,58E-12
NUDT3	1,711059	7,12E-06
PLCG1	1,701562	2,43E-07
STMN3	1,695797	7,34E-06
AUTS2	1,69509	1,16E-06
FAM3A	1,693207	6,84E-09
ALKBH4	1,693076	1,21E-08
SAP130	1,692883	1,46E-12
EPN3	1,692016	0,001005
SRR	1,690589	6,54E-05
LOC28333!	1,689649	0,000245
RASA1	1,687862	2,19E-07
POLR3C	1,684216	0,000372
LEO1	1,684209	3,93E-12
FLJ10038	1,684156	5,79E-06
FOXP1	1,68414	9,56E-08
SSR2	1,683082	1,81E-09
LYSMD1	1,680265	4,04E-06
TC2N	1,679785	1,10E-05
C14orf80	1,679031	1,17E-08
ZNF83	1,677902	2,96E-06
SLC7A5	1,67754	2,48E-09
LAMTOR4	1,672706	2,26E-10
RAB15	1,671831	1,69E-08
LINC01184	1,671599	0,000736
RSU1	1,667938	4,19E-09
CMC2	1,665218	6,02E-08
TSPAN17	1,656475	1,23E-08
PPIC	1,655154	2,67E-08
OAZ2	1,653797	1,11E-07
LY6E	1,65279	2,76E-06
CFDP1	1,652138	1,17E-09
CALHM2	1,65192	0,000722
SAYSD1	1,65137	1,26E-08
CHD6	1,649441	1,14E-11

CACFD1	1,648334	0,000992
ELMO3	1,64724	2,52E-06
ERGIC1	1,646394	1,47E-08
DENND2D	1,64608	9,45E-06
JUP	1,646069	8,99E-05
DZIP3	1,646068	3,85E-05
ATXN7L1	1,64537	6,21E-05
SEPW1	1,642209	2,96E-06
PFN2	1,640737	1,32E-09
REEP6	1,639353	0,000505
ZFP30	1,637171	0,000573
STOM	1,637096	3,19E-06
CORO1B	1,634126	2,71E-05
COA3	1,63393	1,63E-05
ACBD7	1,631314	0,000143
PSMD4	1,629802	7,99E-13
FBXO31	1,629706	1,35E-09
SF3B4	1,626249	1,23E-12
ADCK1	1,626092	4,32E-05
HPS1	1,624647	9,70E-09
PCSK6	1,623689	2,43E-05
ZNF100	1,620059	5,87E-06
TMBIM4	1,620017	1,61E-07
ZNF138	1,615362	0,000102
ZNF808	1,610017	0,000678
SERPINB6	1,608198	1,63E-10
TJP3	1,607084	0,000594
TMEM132A	1,605105	3,07E-05
SHFM1	1,603245	1,30E-10
AP4M1	1,603197	2,57E-06
CETN2	1,601569	5,70E-07
IQCK	1,600595	2,94E-05
C7orf50	1,599733	2,30E-07
SLC25A23	1,595741	7,22E-05
ZNF212	1,595411	6,09E-06
DLGAP5	1,594849	2,09E-10
RNF130	1,594734	8,64E-07
RGL2	1,594456	3,99E-08
SNAPC5	1,593746	4,27E-06
ATP5G2	1,592682	7,07E-09
VIPAS39	1,591401	1,34E-07
SEMA4C	1,58843	9,79E-07
HMG20B	1,58813	1,51E-11
UBL3	1,586849	1,55E-06
NUCKS1	1,586718	1,28E-10
LINC00674	1,586662	1,31E-07
MMAB	1,582781	2,43E-05
KBTBD7	1,580852	0,000145
PSD4	1,580091	0,00014
NDUFAF6	1,579547	5,52E-07

S100A10	1,578931	1,65E-09
THOC6	1,573561	7,56E-05
NDST1	1,572874	6,29E-08
AP5S1	1,572587	4,28E-05
LAMTOR2	1,572064	6,94E-08
HDDC3	1,571566	0,000335
AGAP2	1,571229	0,000265
KARS	1,567462	5,14E-10
GNB2	1,560267	3,29E-10
ARPC1A	1,559848	1,11E-08
KLHDC3	1,556414	9,02E-06
CGN	1,555884	8,13E-06
ENSA	1,555873	3,60E-11
BOD1	1,554741	4,11E-06
TRIM33	1,55451	3,94E-09
ZDHHC12	1,554084	1,26E-06
PARD6B	1,55392	7,55E-07
ATP5J2	1,553045	8,80E-06
IRX5	1,552275	8,65E-07
SMARCD2	1,551821	2,33E-10
GSTK1	1,551699	5,46E-06
C20orf27	1,548047	1,73E-09
SOX12	1,545968	6,35E-10
XXYLT1	1,545358	9,81E-08
ARF5	1,544873	4,40E-11
TMEM65	1,544523	1,24E-05
PCYOX1L	1,543677	0,000235
FRS2	1,543117	3,21E-05
SERPINH1	1,540437	1,28E-08
ARMC10	1,535981	9,15E-09
DHX40	1,535341	1,96E-08
GATSL2	1,534871	2,73E-05
MIS18BP1	1,533675	0,000109
PMF1	1,53324	0,000445
PRDM4	1,532467	8,28E-11
CCDC85B	1,532187	6,29E-08
MRPS11	1,530392	6,50E-06
TSC22D1	1,529458	4,33E-05
SH2B2	1,528365	0,000179
GTF3A	1,527449	9,58E-10
MYO5C	1,52727	1,11E-07
TPBG	1,525621	4,46E-09
DHFRL1	1,522798	0,000542
CAMK2N2	1,521959	0,000471
POGZ	1,52126	2,10E-05
BANF1	1,520673	5,36E-08
DARS2	1,520461	3,64E-09
TRAK2	1,520369	1,36E-09
PRKAG1	1,518826	2,71E-06
TGIF2	1,513994	6,96E-05

NUDT4	1,513296	1,25E-06
DYNLL2	1,512896	6,56E-10
DDX41	1,512372	8,34E-10
CDC42SE1	1,510759	1,85E-08
SLC39A11	1,508769	0,000581
ALDH6A1	1,506708	6,50E-06
ZDHHC4	1,505008	8,22E-08
FOXA1	1,503545	8,81E-06
PIAS3	1,503231	6,60E-05
STRA13	1,502125	6,16E-09
ROMO1	1,501414	4,07E-08
MTIF3	1,499693	1,58E-05
ARPC5	1,498993	2,85E-07
CYB5R4	1,498339	0,000367
KIF20A	1,496801	1,48E-07
NPDC1	1,496212	4,69E-09
MYL12B	1,495802	2,96E-10
TEAD3	1,495619	4,87E-06
ZNF184	1,495085	8,42E-06
AP1S1	1,494236	0,000232
TMEM14C	1,49352	2,42E-07
PLEKHH1	1,493398	1,05E-05
FTSJ3	1,492916	6,03E-07
AP2M1	1,489768	2,72E-07
CDK2AP1	1,488986	3,57E-06
EPHB3	1,488797	0,000511
C11orf73	1,488326	1,87E-06
LEF1	1,487925	3,06E-05
ZNF687	1,486911	1,21E-06
SLC37A4	1,485137	2,18E-08
KIF3C	1,483792	5,16E-05
FAM220A	1,480749	1,87E-05
FAH	1,479959	3,73E-07
USP8	1,478667	2,48E-09
CFL1	1,47562	5,61E-10
DHODH	1,47429	9,43E-05
CABLES2	1,473742	5,39E-06
ALDH7A1	1,47281	6,70E-05
EBAG9	1,471962	0,00069
BRMS1	1,471396	4,35E-07
ZP3	1,470608	5,48E-05
TMEM14B	1,466997	5,95E-06
RPA3	1,465328	5,86E-05
EFNA3	1,465323	9,91E-05
RNF208	1,465078	0,000445
TMEM19	1,464553	3,48E-05
FUNDC2	1,463222	2,32E-07
MCTS2P	1,462279	0,000263
GCNT1	1,459077	6,76E-05
SSH3	1,458788	1,04E-05

KLHDC2	1,457662	0,000119
CHCHD5	1,456325	3,62E-06
DPAGT1	1,45618	1,79E-07
IGFBP2	1,455861	1,56E-05
RALBP1	1,455718	5,09E-06
HIPK1	1,452752	8,34E-09
C19orf43	1,450269	1,74E-10
OPN3	1,449698	0,00063
VMP1	1,448862	1,39E-07
CHMP1B	1,44855	1,29E-06
WDR83	1,448217	7,67E-05
ZNF783	1,447238	0,000192
PSMC2	1,446198	2,31E-09
ATG10	1,44546	0,000393
IFI30	1,445103	0,000972
ZFHX3	1,44415	1,04E-07
GLCE	1,443857	1,58E-06
S100A16	1,437603	0,000649
NSUN5	1,436811	1,89E-07
POLR2K	1,435845	7,47E-05
WDYHV1	1,434975	0,00034
NDUFS6	1,434618	2,80E-08
CBLL1	1,434316	1,90E-06
ARPC1B	1,433311	5,64E-05
C14orf2	1,432212	3,23E-07
MRPS25	1,427624	6,21E-06
MED20	1,426984	1,91E-05
TACO1	1,424266	7,43E-09
BCL2L11	1,421284	2,31E-05
GTF3C1	1,419382	0,000172
MRPL11	1,417368	1,51E-08
GABPB1	1,417232	5,21E-05
CLTB	1,416306	0,000121
CENPBD1	1,41406	9,70E-05
RPL26L1	1,412569	2,26E-06
RNASEH2A	1,411899	4,17E-08
DST	1,409972	1,67E-05
IFT43	1,409263	0,000696
GADD45GII	1,406851	0,000135
BMPR2	1,405259	2,16E-05
CASP6	1,404595	5,27E-05
RUSC1	1,402595	1,75E-05
GCSH	1,402429	1,86E-05
APH1A	1,402396	1,28E-08
GDE1	1,401497	5,63E-07
PTTG1IP	1,400315	1,41E-07
MAN2B1	1,399451	3,00E-06
PRELID1	1,398	1,35E-08
COX4I1	1,397132	6,50E-10
LAMTOR1	1,396515	1,65E-06

TTC37	1,395108	1,40E-07
NDUFV3	1,394474	6,06E-08
NIT2	1,393886	2,70E-05
BUD31	1,393354	8,50E-07
DHX38	1,390368	3,11E-08
SRD5A3	1,389638	0,000396
VKORC1	1,386069	4,50E-05
NCK2	1,38535	1,73E-07
TDP1	1,385165	5,09E-06
TRIAP1	1,385114	2,46E-05
JARID2	1,384885	1,81E-05
ABHD16A	1,384027	1,23E-06
SKAP2	1,381881	0,000132
FAM53B	1,379555	3,86E-08
ASNA1	1,379471	6,35E-08
DSTN	1,379283	1,99E-05
JTB	1,37885	3,34E-08
DGKZ	1,378243	3,30E-07
AIF1L	1,376792	0,000184
POLR3GL	1,376176	9,83E-05
RTFDC1	1,374814	6,51E-07
MBTD1	1,373782	0,000537
RNF5	1,372982	3,93E-05
ACVR1	1,372388	0,000879
ADI1	1,370919	3,14E-06
ZSWIM7	1,370191	0,000245
RPS3	1,36878	1,60E-06
AKT1	1,367573	1,97E-05
NKD1	1,366446	2,13E-05
DHPS	1,365302	1,29E-06
GINS2	1,365284	6,56E-07
SELT	1,364909	1,24E-05
TANC2	1,360954	0,00018
TRPM7	1,360692	2,20E-08
DGCR6L	1,360529	0,000122
SLC35B2	1,36049	1,92E-05
AIMP2	1,359893	1,10E-07
ATAD2B	1,359441	0,000695
RPL13	1,359132	9,31E-06
RFWD2	1,358824	2,02E-05
SIKE1	1,354744	1,01E-07
PACS2	1,352482	1,98E-06
GPATCH2	1,352078	4,66E-06
TTLL5	1,349355	5,60E-07
COIL	1,348032	0,000611
LNX2	1,347407	2,30E-07
RHPN1	1,346807	0,00035
UQCR10	1,344894	1,63E-05
PRR11	1,344288	8,98E-08
NGFRAP1	1,344239	2,76E-08

NIPSNAP1	1,344148	5,96E-07
H2AFY2	1,343632	7,99E-05
GRK6	1,342062	3,23E-08
PDCD10	1,341621	2,11E-06
ZNF277	1,341428	1,94E-05
ZNF254	1,340404	0,000787
COX5B	1,339662	1,37E-08
NT5DC2	1,339046	4,12E-05
SETDB1	1,338272	1,60E-06
APOA1BP	1,337954	1,66E-06
MEST	1,335147	0,000113
BRK1	1,334614	4,19E-07
ZNF260	1,334612	0,000406
ZFYVE21	1,333625	1,46E-05
AURKA	1,333085	3,56E-06
CYTH2	1,332201	1,69E-06
ZNF84	1,331238	0,000996
BLVRA	1,3311	3,07E-05
POLR2I	1,330165	4,08E-07
PIGG	1,327631	3,33E-05
COPS6	1,327035	2,19E-07
EXOC1	1,3246	2,90E-05
RALGAPA1	1,324586	1,21E-05
FMR1	1,321168	0,000196
SPTLC2	1,320757	2,64E-06
SNX27	1,319485	5,82E-06
OST4	1,319435	5,75E-07
MDH2	1,319073	1,90E-06
NGRN	1,317234	5,30E-06
FARSA	1,316821	1,54E-05
MESDC2	1,316708	2,79E-05
PAN2	1,315214	0,000773
SIVA1	1,313283	1,64E-05
WRB	1,310531	7,95E-05
H3F3B	1,310114	6,72E-08
HIGD2A	1,307718	5,95E-08
ZNF766	1,306582	9,63E-05
ESD	1,30632	3,69E-07
SETMAR	1,305597	9,91E-05
C2orf68	1,305094	2,86E-06
HSBP1	1,304965	1,02E-05
MYBL2	1,302929	2,26E-07
VPS28	1,302163	6,01E-05
CNPY3	1,298069	1,45E-05
C1orf43	1,297412	1,23E-05
WBSCR16	1,296791	2,47E-07
MAP3K11	1,296594	0,000433
ATP6V0E1	1,29556	0,00098
AHCY	1,295296	1,15E-05
MRPL9	1,293491	0,000123

MPND	1,290693	0,000291
MED21	1,286018	7,07E-05
RNF181	1,284658	7,10E-06
AARS	1,284502	0,000167
LCMT1	1,282021	7,80E-06
COG4	1,277985	0,000254
SRPK2	1,277903	2,50E-06
CENPB	1,277733	2,04E-07
C5orf30	1,274516	1,27E-05
THAP11	1,273865	0,000246
GSE1	1,272802	7,22E-08
UBAC1	1,272376	0,000258
SPATA33	1,271571	0,000123
ADAT1	1,270524	2,74E-05
BCAP31	1,27044	7,22E-06
GRN	1,26936	0,000576
EMC3	1,267426	0,000182
HMGN1	1,264795	2,57E-07
PHKG2	1,263413	1,20E-05
COX14	1,262372	0,000328
ATL3	1,26112	0,000599
EIF4EBP2	1,260735	0,000177
LAMA5	1,260208	8,25E-06
RGS12	1,260175	0,000396
ZMYM2	1,260086	1,36E-06
TIMM22	1,257512	3,16E-05
CRTC2	1,256069	0,000137
RPP40	1,255635	0,000287
CMAS	1,255568	0,000914
C7orf26	1,254996	4,52E-06
BACE2	1,254208	0,000134
RSBN1	1,252278	5,53E-05
TMEM251	1,252257	8,42E-05
ARF3	1,250908	1,61E-06
ANAPC15	1,249364	1,23E-05
MRPL24	1,248046	2,07E-05
LAMC1	1,24658	1,98E-06
CELSR2	1,244061	0,000108
MLYCD	1,24341	0,000746
MTA2	1,243198	9,90E-08
ZBTB22	1,241316	0,000296
LARS	1,238296	9,27E-07
ORAI2	1,23723	0,000349
RPS19	1,236402	3,79E-05
MIF-AS1	1,236058	0,00068
NDUFB10	1,235767	4,15E-06
SAMM50	1,235704	3,35E-05
NDUFB5	1,232291	0,000223
DPP3	1,2298	4,87E-05
INTS8	1,22836	0,00021

COA5	1,227784	0,000822
TMEM258	1,227226	0,000118
OS9	1,226403	6,95E-05
PSMC5	1,225546	1,19E-05
SCFD1	1,22424	8,82E-06
VPS25	1,223984	1,73E-05
MRPL43	1,222613	0,000224
GRB14	1,222008	0,000627
NCBP2	1,221931	2,09E-05
SMURF1	1,221565	4,05E-06
MAF1	1,220407	3,18E-05
LMAN2	1,219491	2,00E-06
TMEM179E	1,219289	0,000213
ZNF786	1,216019	7,34E-05
CALM1	1,215794	5,09E-05
ZNF219	1,215723	0,00014
OTUD7B	1,215561	7,67E-06
SPTBN2	1,215306	0,000183
TOMM6	1,215075	3,95E-06
RBBP9	1,213898	9,91E-05
LPAR2	1,21355	1,35E-05
NTPCR	1,213198	0,000974
SRA1	1,212915	0,000277
XPOT	1,21009	0,00051
HDGF	1,209846	4,35E-07
ZKSCAN5	1,209579	5,41E-05
COX7C	1,208263	5,34E-07
ZSWIM6	1,205826	0,000591
ACTB	1,205266	0,000721
TARBP2	1,204911	1,67E-05
NUBP2	1,204729	3,97E-06
C8orf82	1,204075	0,000153
DBI	1,201064	3,79E-06
TXNDC9	1,200953	7,22E-05
NOTCH1	1,200526	0,000506
RNF7	1,200464	2,84E-05
GLRX5	1,20009	5,51E-05
DECR2	1,199137	0,000246
C17orf62	1,1989	9,27E-06
UBE2T	1,196232	3,63E-05
PKP3	1,194493	0,000179
NPC1	1,19418	0,000103
SNAPIN	1,194143	0,00014
IKBKG	1,193949	0,000462
TK1	1,193755	2,47E-06
RPL30	1,193488	0,000441
RALGDS	1,192964	5,59E-05
DLD	1,192833	0,000307
SPG21	1,191417	6,43E-06
AP4B1	1,189196	0,000149

MTSS1L	1,188545	0,000489
OTUB1	1,186435	4,39E-06
TTC9	1,185091	0,00012
RNMTL1	1,184387	0,000274
SLC25A43	1,184275	8,40E-05
NRBP1	1,184218	0,00018
DERL1	1,182861	0,00029
ETFA	1,182622	0,000248
AGAP2-AS1	1,18212	0,00079
SART1	1,182043	2,09E-06
PSMD7	1,180327	5,66E-07
FAHD1	1,176774	0,000677
PBX2	1,174574	3,14E-05
ENDOG	1,17257	0,000368
CYC1	1,172103	3,40E-06
ECSIT	1,171856	1,84E-05
TAF6	1,16885	9,38E-06
ARPP19	1,168735	1,25E-06
PSMB4	1,168592	9,40E-06
ABHD12	1,168127	3,75E-05
MCM7	1,168112	1,96E-05
EEF1G	1,167794	3,18E-06
POLE4	1,16727	0,000758
CKAP5	1,166864	3,81E-05
ATP5H	1,166786	1,59E-05
ZNF398	1,16614	7,22E-06
RSRC1	1,164172	0,000112
PRDX2	1,163858	1,18E-05
CD47	1,163344	0,0001
TMEM9	1,161836	5,21E-05
TRIM26	1,161092	2,50E-05
YIF1A	1,159888	2,16E-05
ATP5B	1,157275	7,50E-06
SMG7	1,154996	1,41E-06
PAFAH1B3	1,154149	1,76E-05
MFSD3	1,153696	6,63E-05
SNRPF	1,153479	0,000273
CDC25B	1,151957	0,000461
MZT2B	1,150337	0,000207
KMT2E	1,14956	5,89E-05
FLOT2	1,148721	9,53E-05
RPRD2	1,147184	5,90E-05
CDK5	1,146184	0,000277
SEC11A	1,144002	1,15E-06
MEA1	1,142537	5,54E-06
ESYT2	1,140929	4,47E-06
PAAF1	1,139943	9,14E-05
COX6A1	1,138598	1,89E-06
UBTD2	1,137363	7,74E-05
MREG	1,137184	0,000246

SLC44A2	1,134676	0,00036
RUFY1	1,133205	0,000161
TMEM131	1,131929	4,40E-05
WDR60	1,130749	0,000812
SOGA1	1,130435	0,000132
MAZ	1,126788	1,63E-05
PDAP1	1,126235	9,84E-06
SERP1	1,120583	5,12E-06
G6PC3	1,118029	0,000104
UQCRB	1,116818	0,000114
MIDN	1,114656	0,000241
ZMYND11	1,11253	0,000169
NDUFB2	1,111588	2,50E-05
METTL9	1,110599	9,18E-06
NSD1	1,110145	0,000189
TCEB2	1,106284	0,000114
TMED10	1,104108	2,90E-05
EPB41L5	1,103659	7,67E-05
RPS14	1,103346	6,41E-05
KIF14	1,102437	0,000371
RPS17	1,101195	0,000305
PPP1R14B	1,100621	4,13E-06
EHMT2	1,099958	0,000885
TRAF4	1,099753	9,59E-05
EPN1	1,099571	0,000453
RPL32	1,098999	0,000254
BCL7C	1,097857	8,13E-05
P2RX4	1,095654	0,000532
ANKIB1	1,094538	0,000123
SNRPC	1,094029	5,92E-05
TPX2	1,093119	2,37E-05
SUV420H1	1,089888	0,000221
ZC3H3	1,083688	0,000381
NDUFS8	1,082541	7,72E-05
PLEKHF2	1,082523	0,000161
CST3	1,081429	0,000439
CDKN3	1,081273	0,000177
IMPA2	1,081061	0,000272
COX6B1	1,080597	8,83E-05
TCF25	1,078536	1,34E-05
CENPE	1,078197	0,000355
UBQLN4	1,07586	4,38E-05
PIGT	1,074556	0,000152
PFDN5	1,072165	4,23E-05
IMPDH1	1,071832	0,000928
RNF13	1,071703	0,000406
MPDU1	1,071301	0,00017
RNF139	1,070091	7,37E-05
CERS2	1,069505	1,89E-05
RARA	1,069378	0,000857

ATRAID	1,067573	0,000454
GPAA1	1,067319	0,00027
STX6	1,0664	5,05E-05
CDYL	1,065245	0,000141
LLPH	1,0639	2,72E-05
TMEM141	1,060288	0,000111
MRPS12	1,058865	0,000149
NDUFB4	1,057686	8,99E-05
ALDH3A2	1,053649	2,02E-05
LOC90784	1,05364	0,000672
PHLPP1	1,053546	0,000147
NUCB1	1,052177	0,000185
LRRC45	1,043062	0,000309
ATP5A1	1,041263	4,26E-05
GCC1	1,041257	0,000249
CUL9	1,040863	0,000781
RAB3IP	1,039244	4,72E-05
AP2S1	1,039215	0,000137
FAM195A	1,03706	0,000262
TOP1MT	1,036653	0,000728
ECHS1	1,03432	6,51E-05
IDH3G	1,033441	9,09E-05
SYPL1	1,032682	0,000293
GLTSCR2	1,028248	5,64E-05
RFC2	1,027588	3,19E-05
TDG	1,027117	0,000706
PEX11B	1,026548	0,000923
ZNRF3	1,024904	2,90E-05
DNPH1	1,024556	7,45E-05
VPS52	1,024554	0,000268
ADNP	1,022612	0,000169
CNOT2	1,021947	0,000142
GON4L	1,021654	0,000177
OSTC	1,020862	0,000677
METTL2A	1,020858	0,00072
ANAPC11	1,017775	0,001005
TPGS2	1,017709	0,000306
ZFP62	1,017476	0,000486
ARNT	1,016907	0,00029
C6orf47	1,016459	0,000741
CYBA	1,012695	0,000393
CENPN	1,010042	9,59E-05
PAK2	1,006591	0,000125
KRIT1	1,001478	0,000489
MAPKAP1	0,996664	0,000702
SLC25A39	0,996058	2,90E-05
NCAPG2	0,995039	0,001007
RPS15A	0,992017	0,000601
TUBGCP2	0,991597	0,000174
BCAP29	0,984462	0,00081

CDK2AP2	0,984389	0,000416
ZC3H18	0,980808	0,000242
UBL7	0,978634	0,000988
ATP5I	0,97659	8,26E-05
LMNB1	0,975967	0,000546
MRPL21	0,974143	0,000667
CCZ1B	0,970773	0,000676
PIGX	0,966721	0,000775
NDUFB7	0,96622	0,000102
PPP2R5E	0,965265	0,000203
CHCHD3	0,964102	0,000137
NSDHL	0,960821	0,000742
PRR13	0,95815	0,00033
YWHAZ	0,956842	0,000452
SPATS2	0,95621	0,000495
FBRS	0,955855	8,41E-05
TRIM28	0,954391	5,64E-05
GGCT	0,952785	0,000174
EIF3E	0,952113	0,000667
KRTCAP2	0,951387	0,00048
RPL8	0,951011	0,000273
PPP1CA	0,950711	6,44E-05
HNRNPAB	0,94831	0,00017
ARMT1	0,947934	0,000976
EIF3H	0,938998	0,000115
PI4KB	0,937409	0,00055
HMBS	0,933403	0,000639
ILVBL	0,932742	0,000216
KIAA1429	0,932644	0,00077
NBN	0,93235	0,000144
SGPL1	0,926557	0,000485
CPNE3	0,926097	0,000495
DYM	0,920241	0,000462
MAP4K4	0,915212	0,000845
IMP4	0,91127	0,000896
RAB5A	0,910386	0,000596
GPX4	0,908832	0,000242
ATP5C1	0,906909	0,000768
MRPS34	0,906637	0,00052
MAPKAPK2	0,894728	0,000495
ACAA2	0,894079	0,000566
RALY	0,890634	0,000742
PABPC1	0,883962	0,000106
BPTF	0,876065	0,000534
TOP1	0,872743	0,00031
GNB2L1	0,866841	0,00031
C14orf166	0,861927	0,000749
UQCRQ	0,859634	0,000676
PSMC1	0,854815	0,000484
ITPK1	0,842833	0,000645

KIAA1191	0,841258	0,000929
CHMP4B	0,816357	0,000935
CSNK2A1	-0,83341	0,00078
SLC20A1	-0,85711	0,001001
DNTTIP2	-0,86263	0,000908
UBR4	-0,86574	0,000767
THOP1	-0,86635	0,000599
TIMM13	-0,87576	0,000871
PBRM1	-0,87803	0,000468
NAP1L4	-0,87975	0,000369
MFN2	-0,88066	0,000355
ITCH	-0,88232	0,000882
CLIP1	-0,88714	0,000571
EML4	-0,89213	0,000717
ODC1	-0,90438	0,000263
MAPK1	-0,91361	0,000604
MAP4	-0,923	0,000167
STK25	-0,92328	0,000412
RNF220	-0,93016	0,000438
ZNF106	-0,93039	0,000376
ATP6V1B2	-0,93504	0,000564
GIGYF2	-0,9373	0,000218
PHIP	-0,94081	0,000449
AK2	-0,94168	0,000252
KAT6A	-0,94466	0,000754
CLSTN1	-0,94482	0,000494
PUM1	-0,94566	0,00035
SUN1	-0,9501	0,000511
POLRMT	-0,95017	0,000954
SETX	-0,95276	0,00057
SBDS	-0,95284	0,000445
QSOX1	-0,95314	0,000407
NISCH	-0,95431	0,000625
CCT8	-0,95698	0,000236
ARHGEF12	-0,95791	0,000494
RBM6	-0,95838	0,000619
PITPNB	-0,95869	0,000339
SENP6	-0,96176	0,000972
APPL1	-0,966	0,000639
OGFR	-0,96666	0,00091
ERLIN2	-0,96682	0,000432
CYFIP1	-0,96805	0,000905
VPS37B	-0,9684	0,000746
TXNDC12	-0,97032	0,000627
CNOT1	-0,97038	3,78E-05
THRAP3	-0,97142	0,000171
PRKDC	-0,97204	0,000444
FAM3C	-0,97368	0,000418
CEP104	-0,97675	0,000777
SEPN1	-0,97707	0,0007

ZYX	-0,97726	0,000711
NCL	-0,97928	3,04E-05
WDFY1	-0,98486	0,000543
PCNA	-0,98715	0,000262
TRABD	-0,99053	0,000431
ABLIM1	-0,99112	0,000237
RLIM	-0,99294	0,00032
ALS2	-0,99403	0,000581
MARCKS	-1,00485	0,000694
GINS4	-1,00522	0,000706
URB1	-1,00528	0,000588
MTHFD1L	-1,00661	0,000819
EXOSC10	-1,00674	0,000747
PRKD3	-1,00915	0,000409
TOMM34	-1,01015	0,000293
ACBD3	-1,01059	0,000203
TTLL12	-1,01245	0,000438
TMEM2	-1,01299	0,000695
ETNK1	-1,01569	0,000563
TPCN1	-1,01679	0,001007
NOLC1	-1,02	0,000235
OGFOD1	-1,02132	9,49E-05
WASF2	-1,02351	0,000115
USP48	-1,0245	0,000988
TMED5	-1,02452	0,0009
TMEM259	-1,02469	7,51E-05
CTDSPL	-1,02526	0,000231
NDUFA10	-1,02644	0,000108
CUL5	-1,02828	0,000543
NUDT21	-1,03083	9,40E-05
GCH1	-1,03305	0,000696
GMDS	-1,03384	0,000714
AGPAT5	-1,03469	0,0006
P4HA1	-1,03604	0,000236
GOSR1	-1,03779	0,000625
LRRC41	-1,04221	5,97E-05
IBTK	-1,04457	0,000218
COPS2	-1,04552	0,000149
CDK12	-1,04683	0,000742
NHLRC2	-1,048	0,001003
LMAN1	-1,04998	0,000258
UBE3A	-1,05133	2,16E-05
BLMH	-1,05191	0,000954
NDC1	-1,05418	0,000724
TEAD1	-1,05479	8,97E-05
RFC1	-1,05646	4,50E-05
SLC25A6	-1,05919	0,0001
ZFAND5	-1,05982	7,54E-05
DHX30	-1,06169	0,000112
SLC46A1	-1,06448	0,00084

GPX8	-1,065	0,00096
AGAP1	-1,06623	0,000741
SHROOM3	-1,06967	8,86E-05
TUBGCP3	-1,0718	0,000425
TMC6	-1,07323	0,000603
SRSF11	-1,07492	9,04E-05
FNIP1	-1,07559	0,000204
APBB2	-1,08022	0,000461
CC2D1B	-1,08036	0,000206
NECAP1	-1,08493	0,000182
TCF20	-1,08631	2,24E-05
GTF2H1	-1,08653	4,92E-05
ZNF335	-1,08748	0,000488
YRDC	-1,08806	0,000866
PLEKHA5	-1,08983	0,000871
KHNYN	-1,09002	0,000764
PHF3	-1,09155	0,000135
SIK3	-1,09249	9,89E-05
LRPPRC	-1,09332	3,25E-06
PLP2	-1,09425	4,38E-05
MTF1	-1,09532	0,000172
SHMT1	-1,0968	0,000387
CLEC16A	-1,097	0,000131
ATM	-1,09834	0,000648
RIF1	-1,10291	0,000296
SBNO2	-1,10345	0,000138
GTF2H3	-1,10604	0,000609
TICAM1	-1,10671	0,000745
AP2B1	-1,11322	6,32E-06
EIF3J	-1,11342	2,71E-05
ARHGAP27	-1,11408	0,00044
TRAPPC11	-1,11627	0,000112
VPRBP	-1,11768	3,52E-05
GNA11	-1,11789	1,66E-05
MAST3	-1,11923	0,000546
ZYG11B	-1,12217	0,0005
EBLN3	-1,12354	7,79E-05
RNF138	-1,12528	0,000121
ARFGEF1	-1,12574	8,75E-05
NUP133	-1,12623	0,00046
CKAP4	-1,12653	4,54E-06
PHF13	-1,12705	0,000216
CWC25	-1,12807	0,000533
CELSR1	-1,13278	0,000226
PRPS1	-1,13306	0,000343
EYA3	-1,13363	0,000495
USP9X	-1,13393	0,000479
MARVELD1	-1,13508	0,00041
SERTAD2	-1,1373	0,00021
ORC1	-1,13757	0,000383

ARHGEF18	-1,13812	4,62E-05
ELK1	-1,1384	0,000132
USP1	-1,1393	0,000603
ENTPD4	-1,14001	0,000735
USP25	-1,14291	0,000368
MTERF4	-1,14421	0,000511
TMEM123	-1,14531	5,16E-06
TBC1D20	-1,14585	0,000182
LDHA	-1,14682	0,000591
LPGAT1	-1,14967	9,74E-05
UBR3	-1,15015	0,000263
C17orf96	-1,15186	7,88E-05
BAP1	-1,15465	1,87E-05
ESCO2	-1,15587	0,000255
EIF3M	-1,15672	1,70E-06
MFAP1	-1,15688	0,000239
SMC1A	-1,15763	1,79E-05
HIGD1A	-1,1599	0,000103
HBS1L	-1,16111	0,000581
PTAR1	-1,16158	0,000787
NF1	-1,16298	0,000327
DOCK9	-1,1644	0,000796
RHPN2	-1,16519	0,00092
BAG4	-1,16607	7,26E-05
MANEAL	-1,16614	1,53E-05
NVL	-1,16692	0,000119
FOXK1	-1,16705	0,000181
KIAA1468	-1,16818	0,000328
INTS10	-1,16914	0,00019
BBS7	-1,16986	0,000428
UFL1	-1,17144	0,000375
RCC1	-1,17325	9,65E-06
BTAF1	-1,17471	0,000101
TRIOBP	-1,17522	0,000445
CAB39	-1,17526	7,50E-06
TFDP1	-1,17697	1,27E-06
CHAF1A	-1,17829	0,000482
PPP1R26	-1,18049	0,0002
QRSL1	-1,18076	6,32E-05
MPP6	-1,18141	0,000578
LYRM7	-1,18144	0,000136
CBL	-1,18292	3,94E-05
LSR	-1,1836	0,00071
ASXL2	-1,18374	0,00011
CORO1C	-1,18402	4,35E-05
BOK	-1,18597	0,000271
COLGALT1	-1,18616	9,02E-05
FUCA2	-1,18728	0,000292
FAM83G	-1,18939	2,43E-05
U2SURP	-1,19012	3,06E-06

RBFOX2	-1,19052	7,76E-06
C6orf132	-1,19261	1,86E-05
COPRS	-1,19326	0,000236
ROCK2	-1,19332	2,00E-06
FLNB	-1,19398	6,84E-05
C1orf198	-1,19436	0,000525
CD99	-1,19484	7,50E-06
ACSL1	-1,19611	0,000123
CYTH3	-1,19707	0,000262
CD164	-1,19958	1,16E-05
LRRC42	-1,19978	1,48E-05
AIDA	-1,20067	0,000283
TTL	-1,20221	9,24E-05
CEP97	-1,2032	0,000573
HACD2	-1,20517	0,000883
PIDD1	-1,20582	0,000346
EPRS	-1,20626	1,40E-07
SCARA3	-1,20688	0,000825
TCEB3	-1,20775	7,69E-05
SEPT2	-1,20941	1,19E-06
WDR36	-1,20996	0,000118
LRRC40	-1,21165	0,000494
TRMU	-1,21294	0,00028
CSNK2A2	-1,21354	5,16E-06
TRMT6	-1,21537	4,72E-05
STIM1	-1,21605	3,19E-05
DDX52	-1,21667	5,42E-05
USP40	-1,21742	0,000116
RER1	-1,21988	1,86E-06
CDC6	-1,22287	0,000131
EIF4G3	-1,22509	3,77E-07
CCDC186	-1,22656	0,000537
SH3D19	-1,22658	2,38E-05
DCAKD	-1,22859	1,63E-05
CBWD1	-1,22912	0,000629
BAZ2A	-1,23089	3,48E-05
TAF1	-1,23099	0,000229
HIATL1	-1,23351	9,01E-06
AKR1A1	-1,23369	2,67E-06
XPNPEP1	-1,23512	0,000102
ARMC9	-1,23528	0,000209
QSER1	-1,23606	6,40E-07
KCTD9	-1,23629	2,14E-05
UPF3A	-1,23743	5,67E-05
NIPA1	-1,24377	5,64E-05
FRG1	-1,2452	0,000555
CCDC93	-1,24598	8,17E-05
SYMPK	-1,24691	1,75E-07
KLF16	-1,24695	0,00056
EMC3-AS1	-1,24897	0,000174

AMD1	-1,24941	0,000175
GALNT7	-1,24989	0,00099
FBXO30	-1,25307	0,000453
KDELC2	-1,25367	0,000256
DSCR3	-1,25462	4,86E-05
SRSF4	-1,25503	9,02E-06
PKN2	-1,25541	7,41E-05
RHOBTB3	-1,25602	4,24E-05
DOCK7	-1,25744	1,86E-05
CASK	-1,25796	5,70E-05
EPB41	-1,26195	6,20E-05
WRN	-1,26267	0,00074
KPNA6	-1,26274	0,00014
SPTBN1	-1,26419	3,78E-08
USP28	-1,26547	0,000116
TNFAIP8L1	-1,266	0,000378
DIS3	-1,26653	1,60E-05
NOL6	-1,26665	0,000651
NUP50	-1,26881	7,46E-05
AHNAK	-1,26908	7,88E-05
C9orf40	-1,26969	0,000256
SLC6A6	-1,26984	0,001003
SLC35F2	-1,27013	0,000282
ago-03	-1,27072	0,000858
TNKS	-1,27239	0,000785
APP	-1,27266	6,30E-08
PRMT3	-1,27449	0,000115
ADAM10	-1,27493	1,12E-05
HIP1	-1,27547	5,59E-07
IPO7	-1,27872	6,86E-08
EPS15	-1,27875	8,84E-06
HAUS6	-1,27935	1,79E-05
MAPKAPK3	-1,27995	0,000106
PISD	-1,28001	9,29E-06
LARS2	-1,28019	6,16E-05
MIEF1	-1,28027	8,14E-07
SOAT1	-1,28034	0,000426
PPTC7	-1,2829	1,60E-05
CASP8	-1,28628	2,31E-05
MTR	-1,28703	0,000438
ACADM	-1,28784	1,15E-05
PTPN3	-1,28819	0,000175
DFFA	-1,29026	2,84E-05
RMDN3	-1,29082	0,000899
ITGAV	-1,29202	1,76E-05
C12orf4	-1,29237	0,000489
PYCR2	-1,29405	0,000809
MTO1	-1,29431	2,03E-05
DDX10	-1,29484	5,24E-06
ERI1	-1,29595	1,55E-05

CDC42EP2	-1,29992	0,000733
LONP2	-1,3002	3,10E-07
HIAT1	-1,30071	6,49E-05
PROSC	-1,30234	3,76E-06
MROH6	-1,30476	0,000806
CNOT6L	-1,3059	3,12E-05
CCDC109B	-1,30602	0,000996
DVL1	-1,30634	9,55E-06
SH3PXD2B	-1,30716	0,000351
INPP5F	-1,30831	0,000201
LZTR1	-1,30876	2,98E-05
KIAA0040	-1,30972	5,65E-05
PDIA3	-1,31122	1,32E-07
OSGEP	-1,31152	0,000162
TPP1	-1,31358	2,69E-05
SPAG9	-1,31358	0,000102
TRAF3	-1,31417	2,03E-06
STK4	-1,31469	7,96E-07
FAM111A	-1,32034	0,00073
MAK16	-1,32245	0,000211
PAPD5	-1,32422	1,05E-05
MLLT6	-1,32435	1,91E-08
ARFGAP3	-1,32465	0,000265
NOCT	-1,32481	6,17E-05
ZNF770	-1,32518	0,000482
SGK223	-1,32556	0,00051
NDFIP2	-1,32939	8,92E-06
PLS1	-1,33135	0,000118
SLC35D1	-1,33487	0,000219
CHAF1B	-1,33678	2,62E-05
REXO2	-1,33698	0,00089
FDFT1	-1,33892	4,38E-05
JOSD1	-1,3394	2,17E-07
RBM26	-1,33951	1,24E-05
RPUSD4	-1,34124	1,62E-05
MFHAS1	-1,34204	9,45E-08
LAMA3	-1,34219	0,000592
ATP11B	-1,34365	4,57E-07
TRAK1	-1,34452	2,30E-06
CEP57	-1,34461	0,00035
ZNF37A	-1,34509	1,38E-05
ALAS1	-1,34596	3,33E-05
SEPT11	-1,34703	1,58E-08
BAG2	-1,34785	2,91E-05
MCM8	-1,34851	8,35E-05
TOLLIP	-1,34923	0,000708
PTPRU	-1,3494	1,39E-05
SRRM1	-1,3498	2,22E-06
NOC3L	-1,35069	0,000292
TWISTNB	-1,35219	3,12E-06

DESI2	-1,35323	1,51E-06
BIRC2	-1,35569	0,000264
DCAF12	-1,35657	9,18E-08
VAMP2	-1,36111	1,55E-05
KLHL5	-1,36134	3,19E-05
PIM3	-1,36311	6,31E-05
CLN8	-1,36422	9,29E-06
PPIP5K1	-1,36493	2,19E-06
GSR	-1,36529	7,99E-06
UBA5	-1,36544	4,31E-05
FOXO3	-1,36623	0,000651
RPS6KA1	-1,36665	0,000291
EDEM1	-1,36713	8,55E-06
ANKZF1	-1,36846	0,000375
MGAT5	-1,36869	6,16E-06
ATXN2	-1,36873	1,01E-06
SPOP	-1,36908	5,37E-06
P4HA2	-1,3695	0,000174
PGM2	-1,37267	0,000249
KLHL18	-1,37486	0,000706
BROX	-1,37549	2,95E-07
ERO1A	-1,37974	0,000257
ZMYM6	-1,37981	0,000177
PKD2	-1,37992	6,76E-05
POLM	-1,38174	0,00016
MCFD2	-1,38191	3,65E-07
EHD2	-1,38517	0,000146
CCSAP	-1,38626	0,000188
YIPF6	-1,3864	2,97E-07
SLC35A3	-1,38908	8,39E-06
SSH2	-1,39063	5,73E-05
SACM1L	-1,39187	1,58E-06
ZC3H7B	-1,3939	3,98E-08
DR1	-1,394	1,09E-07
NSRP1	-1,39488	1,59E-05
RANGRF	-1,39563	0,000135
UTP14C	-1,39701	7,85E-05
STX3	-1,39831	4,01E-05
DSG2	-1,39855	1,08E-08
NEMP1	-1,40028	5,23E-06
PBK	-1,40311	8,40E-05
MIA3	-1,40318	9,98E-08
NMNAT1	-1,40763	0,000693
ILKAP	-1,40867	8,97E-05
COASY	-1,40869	3,63E-08
RAB29	-1,41345	8,79E-05
NXN	-1,41493	2,48E-06
KIF1B	-1,41593	4,64E-05
HERC2	-1,41886	5,09E-07
TNPO1	-1,4212	3,03E-09

SLBP	-1,42286	2,01E-06
KREMEN1	-1,42363	2,18E-07
GALNT2	-1,42698	1,18E-05
AGFG2	-1,42866	0,000453
H6PD	-1,42968	8,44E-05
AGTPBP1	-1,42995	3,91E-06
MSI2	-1,43011	3,85E-06
REL	-1,43304	2,23E-06
TBX3	-1,43409	2,36E-07
PNP	-1,43675	4,16E-06
DTX3L	-1,44032	0,000318
CMTM4	-1,4414	3,59E-09
NFIA	-1,44178	3,88E-06
ACBD5	-1,44398	8,38E-06
TMEM181	-1,44651	1,02E-06
DPYSL2	-1,44682	0,000775
BBX	-1,44831	1,60E-06
MKNK2	-1,44862	1,03E-06
MORC3	-1,44997	4,54E-05
SCRN1	-1,45249	2,06E-08
HMGA1	-1,45325	1,14E-06
STK39	-1,454	0,000656
RAD54L2	-1,45414	5,86E-05
TARBP1	-1,4545	0,000407
SZT2	-1,45456	0,000355
TACC1	-1,45653	1,18E-07
CYB5RL	-1,45666	0,000423
SORBS3	-1,45684	1,47E-06
PACSIN2	-1,4574	5,01E-07
PRICKLE3	-1,46473	2,28E-05
ABCD3	-1,46655	3,56E-07
MED8	-1,46946	0,000118
COTL1	-1,46968	1,53E-07
METTL17	-1,47	0,000376
CHD9	-1,4716	1,88E-05
API5	-1,47192	1,33E-07
OTUD3	-1,47252	3,03E-05
WDR81	-1,47327	2,96E-05
B3GALNT2	-1,47486	2,87E-07
RAB3GAP2	-1,47514	3,80E-08
QSOX2	-1,4766	3,64E-05
PQLC1	-1,47747	3,21E-06
SYNJ2	-1,48132	1,47E-05
PGM3	-1,48214	2,12E-05
DNAJC24	-1,4822	0,000833
DOPEY1	-1,48222	0,000338
DDX21	-1,48226	2,59E-07
LOC100506	-1,48471	0,000204
LMF2	-1,48656	2,97E-06
RTCA	-1,4881	2,95E-07

SH3RF1	-1,48818	0,000877
BMP2K	-1,4891	3,10E-05
SFMBT1	-1,49115	9,36E-05
FOXN2	-1,49216	3,83E-05
USP16	-1,49262	1,19E-08
RRAS2	-1,49307	9,48E-06
P3H1	-1,49411	1,20E-05
TFB1M	-1,49464	0,000652
PDE12	-1,49468	2,00E-08
TYMS	-1,49672	1,50E-09
CCBL2	-1,50003	2,80E-07
YARS	-1,50083	5,64E-06
TTC17	-1,50374	5,21E-09
WDR13	-1,50837	0,000135
CDC14B	-1,50923	0,000119
STX2	-1,50966	0,000246
CTNNAL1	-1,51114	0,000208
POLQ	-1,51266	0,000456
PDZD8	-1,51276	4,37E-10
MORN1	-1,51344	9,94E-05
ENO2	-1,5141	0,00026
HMHA1	-1,51852	3,06E-06
RHOBTB2	-1,51907	6,66E-05
PHF8	-1,51907	4,54E-06
MELK	-1,52082	2,05E-09
IPO5	-1,52148	1,71E-08
CDC25A	-1,52399	1,58E-05
DNAJC16	-1,52407	2,03E-06
PIBF1	-1,52407	0,000954
PRKAR2A	-1,52449	3,91E-05
RDX	-1,52652	1,40E-07
NT5DC3	-1,52829	3,12E-05
NPAT	-1,52927	2,60E-06
MYO1B	-1,52968	5,46E-06
ACD	-1,52981	1,89E-07
GPC4	-1,53098	2,96E-05
MCOLN1	-1,53506	2,08E-05
DGKQ	-1,53839	7,22E-05
HLCS	-1,53841	0,000646
S100A6	-1,53848	0,000626
AHCTF1	-1,53966	2,33E-06
OSBP	-1,54299	1,13E-09
GTPBP1	-1,54314	2,04E-07
GART	-1,54367	1,48E-08
ZNF37BP	-1,54547	5,81E-05
CRLF3	-1,54561	0,000531
SARM1	-1,54849	0,000223
GLIS2	-1,54984	6,80E-05
TOE1	-1,5506	8,64E-05
ACACA	-1,55111	1,24E-09

DESI1	-1,55685	4,52E-10
PKP2	-1,55801	8,82E-05
OTUD4	-1,55845	2,56E-07
MYCBP2	-1,56031	5,02E-07
KANK1	-1,56103	1,54E-06
POMZP3	-1,56775	1,81E-05
NKTR	-1,56787	1,39E-07
TGFB1	-1,56821	2,35E-08
TGFBR2	-1,56853	5,59E-05
MGAT4A	-1,57053	0,000101
WRAP73	-1,57288	0,000239
MAP3K10	-1,57354	3,26E-06
RPAP2	-1,57553	5,93E-07
LRP6	-1,57754	1,28E-07
SLC4A3	-1,57875	0,0006
PCNXL2	-1,57994	1,68E-06
TMEM201	-1,5801	5,00E-08
LCOR	-1,58294	6,34E-08
C1orf216	-1,58303	4,86E-07
NUP43	-1,58312	1,75E-06
COBL	-1,58319	4,28E-06
TCF12	-1,5836	1,10E-05
MYSM1	-1,58435	7,25E-05
HDAC6	-1,58454	6,31E-07
RRM2	-1,58497	9,92E-08
CARS2	-1,58632	2,00E-05
GAL	-1,58838	1,21E-07
CBFB	-1,58868	7,98E-09
SSX2IP	-1,58955	2,81E-08
ARIH2	-1,59134	3,98E-09
TIFA	-1,59154	0,000621
TJP1	-1,59173	9,03E-07
ZNF324	-1,59369	2,57E-05
ATF4	-1,59569	1,32E-05
LENG8	-1,59976	2,96E-05
ASRGL1	-1,60255	0,0001
ANKH	-1,60303	6,22E-07
PDP1	-1,60309	8,80E-06
CFAP97	-1,60311	2,29E-06
DONSON	-1,60474	1,07E-05
PIK3C3	-1,60492	7,77E-07
DHRS11	-1,60533	9,47E-05
PARVA	-1,6054	2,45E-06
ACAT1	-1,6062	4,93E-05
FYCO1	-1,61121	9,36E-06
BEND7	-1,61204	0,000906
SYNJ1	-1,61237	0,000232
CLDND1	-1,61275	7,34E-05
STAT3	-1,61909	1,97E-05
PVR	-1,62101	0,000229

FAM213B	-1,62399	5,94E-08
JAK1	-1,62435	6,10E-09
KATNB1	-1,62822	7,78E-09
LRRFIP1	-1,62866	8,77E-12
PAQR3	-1,62926	0,000109
MALT1	-1,63131	1,14E-07
LRRC14	-1,6314	2,62E-08
ADCY3	-1,63469	8,07E-09
TMEM51	-1,6353	1,04E-07
SPRED2	-1,63745	1,66E-10
PITPNM2	-1,64008	0,000155
SWAP70	-1,64072	5,23E-07
C16orf70	-1,64128	2,88E-06
CAPRIN2	-1,6442	0,000416
AKAP17A	-1,64539	5,64E-06
HSPA13	-1,64559	0,000327
SKI	-1,64811	2,52E-10
NFKBIE	-1,64905	4,88E-07
CRELD1	-1,6496	0,000314
LGALSL	-1,65117	7,89E-07
PINK1-AS	-1,65232	0,000134
B4GALT6	-1,65243	0,000452
RELT	-1,65442	1,62E-05
ZSCAN12P1	-1,65604	1,54E-05
ETHE1	-1,65872	8,79E-05
TLN2	-1,65892	7,12E-05
PHF19	-1,66372	2,62E-10
SLC9A7	-1,66648	0,0003
EPHA10	-1,6665	0,000906
MT2A	-1,66714	6,26E-09
DFFB	-1,66894	0,000809
SUZ12	-1,67063	1,93E-12
CSPP1	-1,6707	2,31E-06
TMCO3	-1,67087	1,80E-05
JAG2	-1,67118	5,93E-07
PNKD	-1,67125	4,55E-08
PNPLA6	-1,67435	0,000171
MTMR10	-1,67464	7,21E-06
RUSC2	-1,67474	0,000819
TYW3	-1,67588	5,00E-08
MIER1	-1,68038	6,12E-10
PARP10	-1,68134	0,000549
LPIN1	-1,6827	0,000332
BICD1	-1,68859	2,39E-05
FAR1	-1,68911	5,37E-11
EHD4	-1,68912	6,66E-08
CD46	-1,68925	7,51E-09
MYO10	-1,68949	2,05E-09
WDR76	-1,68988	9,73E-07
MAN1A1	-1,69125	6,16E-08

OGT	-1,6922	1,15E-05
GNL3	-1,69465	2,82E-07
INF2	-1,69789	8,65E-10
ABCB7	-1,70262	1,11E-06
IGFBP4	-1,70808	3,42E-05
ARHGAP29	-1,71072	4,01E-05
CD59	-1,71208	1,27E-05
HEATR1	-1,7178	9,64E-10
MARC2	-1,71969	0,000155
AMIGO3	-1,72227	0,000254
TMEM63A	-1,72787	3,29E-07
SLC16A1	-1,72846	4,01E-11
CALCOCO2	-1,73219	0,000894
OSBPL5	-1,73238	1,58E-06
CROCCP2	-1,73267	0,000564
NMI	-1,73368	0,000427
ING5	-1,73396	4,31E-05
TAP2	-1,73418	3,08E-07
DENND5A	-1,7352	1,28E-06
SAMD8	-1,73674	2,08E-08
CAMK1D	-1,73697	1,08E-05
SLC12A2	-1,74052	1,83E-12
SRXN1	-1,74355	0,000225
C10orf12	-1,74458	8,90E-07
KDM4C	-1,74797	3,59E-08
KLHL21	-1,74834	9,80E-10
AHI1	-1,7507	2,56E-05
MZF1	-1,7522	1,56E-05
RDH10	-1,75366	9,01E-05
ZDHHC23	-1,75571	2,18E-05
KSR2	-1,75635	7,91E-05
GLB1L2	-1,75793	1,97E-08
FAM8A1	-1,75834	7,24E-09
PRSS22	-1,75941	0,000406
POLR3G	-1,75989	0,00071
ARNT2	-1,76192	9,37E-06
NAB1	-1,76381	8,76E-07
RNF170	-1,76554	1,22E-05
RAB11FIP1	-1,76652	1,80E-11
MICAL1	-1,76767	0,000265
FNDC3A	-1,76805	3,36E-12
PTER	-1,77503	9,32E-07
TMEM41B	-1,77804	1,09E-07
TRPM2	-1,78221	5,07E-08
ST5	-1,78602	5,67E-05
BIK	-1,78635	0,000475
ENPP4	-1,78846	8,89E-06
SDCBP	-1,79257	2,69E-05
CASP3	-1,79358	4,18E-06
PODXL	-1,80007	6,17E-10

GANC	-1,80053	9,62E-05
SLC25A25	-1,80414	7,35E-05
BTN2A1	-1,80523	0,000302
HOXB7	-1,80736	8,62E-06
FHL3	-1,81084	8,21E-08
SNX30	-1,81252	1,85E-07
SLC41A2	-1,81303	6,11E-07
MFSD12	-1,81425	7,01E-09
TXNRD1	-1,81757	7,15E-07
NHSL1	-1,81934	0,000492
ZAK	-1,82216	2,19E-11
FUT11	-1,82291	0,000327
CDCA7	-1,83032	8,79E-05
TOX2	-1,83044	7,66E-05
PSIP1	-1,83113	2,51E-06
RAVER2	-1,83359	7,90E-07
ZIC2	-1,83802	3,46E-05
PARP8	-1,83895	0,000226
OSBP2	-1,83976	1,76E-08
RRAGD	-1,84433	3,47E-05
SDE2	-1,8458	3,84E-05
PARP12	-1,8473	0,000186
SLC18B1	-1,84755	2,61E-07
SEMA4F	-1,84767	0,000857
DNAJC3	-1,84841	6,21E-08
UBALD2	-1,85041	0,000818
PTRF	-1,85236	4,30E-11
NOL9	-1,85263	2,24E-07
FNTA	-1,85479	1,09E-11
PPARGC1B	-1,85501	1,70E-05
YAP1	-1,85507	4,29E-12
SCMH1	-1,8588	0,000421
UGGT2	-1,8596	4,63E-07
FKBP14	-1,86041	4,54E-06
FBXO41	-1,86203	2,52E-10
FGFRL1	-1,86259	6,86E-15
SETDB2	-1,86534	5,94E-05
LIN7C	-1,86784	2,69E-08
CCNO	-1,86849	0,000336
DOK3	-1,87049	1,02E-05
DEGS1	-1,8712	4,26E-08
HK2	-1,87557	4,00E-06
TNFRSF10A	-1,87828	3,44E-06
DENND4C	-1,88039	8,58E-12
PDP2	-1,88087	2,99E-07
STARD4	-1,8857	1,20E-07
MICAL2	-1,88587	2,47E-07
CASC4	-1,88768	1,31E-13
ADCK3	-1,88811	7,63E-07
PERP	-1,88849	1,63E-07

LGALS8	-1,88862	6,84E-08
SDCCAG8	-1,88981	1,46E-06
ZNF462	-1,89238	1,06E-08
FABP5	-1,89292	6,62E-07
GPR137B	-1,89409	6,92E-06
EOGT	-1,89673	7,13E-05
ELL2	-1,89703	4,86E-06
EIF2AK4	-1,90306	7,27E-10
IRF1	-1,90394	1,47E-05
DCP1A	-1,90524	3,48E-08
SLC5A3	-1,90886	2,01E-11
RPH3AL	-1,90995	9,23E-06
RABGGTB	-1,91079	1,81E-12
CBARP	-1,9144	1,81E-05
SLC12A8	-1,91897	1,89E-06
ALDH1B1	-1,92259	1,24E-13
ABHD3	-1,92895	3,99E-07
TCIRG1	-1,93014	3,83E-10
SIM2	-1,93472	1,02E-05
TGFA	-1,9383	2,75E-05
STXBP1	-1,93976	4,37E-11
MAFG	-1,94174	5,36E-10
SH2D4A	-1,94292	6,16E-12
HSPA12A	-1,94604	1,00E-05
TUBGCP6	-1,94614	5,57E-08
SHROOM1	-1,94699	9,56E-09
KLF3	-1,94761	9,72E-08
C15orf41	-1,95844	0,000235
MAPK8IP3	-1,96135	0,000768
SQRDL	-1,96165	0,000323
CCDC113	-1,96389	3,89E-08
EXTL2	-1,96503	1,87E-06
SNX9	-1,96509	1,87E-12
NCAPD3	-1,9655	2,63E-10
CBLC	-1,966	3,79E-06
CAMK1	-1,96648	2,26E-05
XRRA1	-1,96722	1,87E-09
APBA3	-1,96768	3,52E-10
FARP2	-1,96799	3,65E-07
PHKA1	-1,96849	4,18E-08
CLTCL1	-1,97156	9,59E-05
SOCS2	-1,97185	0,000563
SMC5	-1,97293	2,15E-12
PIM2	-1,97365	2,80E-08
ELK3	-1,97376	0,000316
TPP2	-1,97498	1,93E-10
ATP11A	-1,97693	1,37E-10
APOBEC3F	-1,97881	0,000295
UGDH	-1,98116	1,11E-06
MFSD2A	-1,98248	3,25E-10

ZNF319	-1,98401	0,000638
OBSCN	-1,98473	3,55E-05
IFFO2	-1,98538	1,36E-05
POMK	-1,98688	6,47E-05
PTCH1	-1,98697	7,25E-06
SERPINB1	-1,98713	4,30E-09
LRP8	-1,98771	1,50E-07
ENDOD1	-1,9892	5,82E-13
VCL	-1,99353	1,05E-07
B4GALT5	-1,99658	1,60E-14
TXLNA	-2,00077	9,06E-13
PATL1	-2,00089	3,99E-14
ANO9	-2,00171	1,70E-06
SOCS6	-2,00205	2,23E-11
TFR2	-2,00549	6,98E-05
NEDD4L	-2,00839	8,00E-10
SATB2	-2,01806	2,13E-11
SERINC2	-2,01866	2,60E-07
EIF4A2	-2,02365	1,65E-07
ADCY7	-2,02437	1,19E-08
TIMP2	-2,02544	1,88E-09
TGDS	-2,02626	0,000136
MFGE8	-2,02635	1,11E-09
ST6GALNA(	-2,02664	4,50E-10
KPNA5	-2,02726	0,000316
LINC00641	-2,02816	0,000199
ZNF57	-2,02941	4,19E-07
DOCK1	-2,02963	6,64E-10
MYO1E	-2,03357	1,60E-05
ICA1L	-2,03412	0,000135
CAMKK1	-2,03883	3,33E-06
ADRBK2	-2,04007	1,45E-13
FLVCR1	-2,04705	1,37E-08
OXCT1	-2,04927	3,95E-12
LPCAT2	-2,05493	2,59E-06
MAPKBP1	-2,05686	1,11E-06
RNPEPL1	-2,05722	1,50E-12
ECHDC1	-2,05887	8,93E-11
CSRNP1	-2,06135	6,69E-08
INPP5J	-2,06397	6,67E-05
TMX3	-2,0655	8,04E-11
SPIN4	-2,07781	1,04E-06
GAS6	-2,07809	4,53E-09
MOB3C	-2,08185	0,000461
GJA3	-2,08458	2,42E-09
TP73	-2,08858	0,000321
RPS6KA2	-2,08977	0,000764
LTN1	-2,09048	3,95E-08
LPIN2	-2,09317	9,38E-12
BACH1	-2,09682	6,21E-11

EPDR1	-2,09963	3,14E-08
GATA6	-2,10082	1,06E-06
SH3BGRL2	-2,10474	3,41E-05
SMCO4	-2,1076	0,00068
PMAIP1	-2,10797	0,000106
EPHX1	-2,11047	3,79E-10
GPR180	-2,11047	1,71E-10
STK17B	-2,11562	1,24E-10
CAV2	-2,11975	7,75E-06
GFPT1	-2,12101	1,08E-06
CARNMT1	-2,12299	5,52E-07
CDKL1	-2,12388	0,000254
KLF9	-2,13118	0,000177
ITGA2	-2,13166	1,41E-08
PSAT1	-2,1337	2,26E-05
ZMYM6NB	-2,13439	5,24E-05
CDC42BPA	-2,13712	9,01E-09
SNHG3	-2,13846	2,00E-09
PAK1	-2,14182	1,97E-08
EFHD2	-2,14375	3,14E-14
TNS3	-2,14578	1,42E-08
HSD17B6	-2,14699	0,000402
SEL1L3	-2,15059	1,16E-12
PANK4	-2,15332	6,10E-12
SLC25A37	-2,15529	1,49E-09
SLC30A7	-2,16006	4,99E-12
ASIC1	-2,16053	3,90E-05
PDDC1	-2,16718	1,26E-13
SLC25A12	-2,16763	6,64E-08
C4orf32	-2,17408	3,65E-07
LDLRAP1	-2,17505	1,06E-07
EVI5	-2,17531	2,78E-08
CPD	-2,17749	2,51E-18
PPARA	-2,17949	5,85E-09
LOC90768	-2,18088	2,43E-05
PTPN14	-2,18113	2,75E-14
RPS6KA5	-2,18596	5,73E-08
FAM89A	-2,18733	0,000623
HERPUD1	-2,18739	0,00049
DGKD	-2,19175	3,60E-14
KLHL15	-2,19404	9,12E-08
SORL1	-2,19908	1,95E-13
MACC1	-2,20028	0,000233
KCNQ4	-2,2007	0,000982
GRB10	-2,20273	0,000822
DIXDC1	-2,20464	3,18E-06
PRTFDC1	-2,20524	1,15E-07
CHM	-2,20803	7,92E-06
CCDC82	-2,21088	3,78E-05
HYI	-2,21899	1,42E-05

TIAM1	-2,2227	9,65E-09
SORBS1	-2,22431	5,09E-07
HSD17B12	-2,22452	6,63E-20
TTBK2	-2,22531	2,11E-05
SBF2	-2,22742	4,25E-06
PLEK2	-2,23215	5,77E-08
DUSP7	-2,23644	4,36E-05
GHDC	-2,23753	1,31E-09
FYN	-2,24886	3,26E-05
RIN3	-2,24894	1,23E-07
CAPN10	-2,25213	1,65E-08
SAMHD1	-2,25383	7,51E-06
SLC35G1	-2,25477	0,00021
FAF1	-2,25649	4,92E-09
ABCB10	-2,25891	4,35E-09
SH2D3A	-2,26073	1,15E-08
CTH	-2,26097	1,23E-05
ATAD5	-2,26456	2,03E-07
SOD2	-2,26528	1,13E-12
FAM92A1	-2,26532	6,99E-05
PTPRG	-2,26543	3,14E-15
KCTD17	-2,26572	2,57E-07
GATA2	-2,2686	1,07E-05
BCL2L2	-2,27085	2,39E-12
ABCC4	-2,27281	7,33E-13
CARD10	-2,27441	6,77E-10
CXCL16	-2,27927	3,06E-16
SNAI1	-2,28002	7,51E-05
CORO1A	-2,28066	1,12E-05
NUPL2	-2,28253	1,84E-05
BMP8B	-2,28714	7,99E-07
DAGLA	-2,28765	1,11E-06
ITGA3	-2,29112	1,00E-20
IFNGR2	-2,29663	7,02E-12
OAT	-2,2987	1,14E-06
SFXN3	-2,30222	1,50E-07
KLHL17	-2,30256	8,64E-06
HOOK1	-2,30447	2,37E-18
DUSP2	-2,30859	1,06E-06
ADGRG6	-2,31599	5,32E-15
DGKE	-2,31772	4,39E-11
ANKMY2	-2,32385	4,69E-12
RAB20	-2,3259	4,55E-05
SLC43A1	-2,32594	5,79E-05
MXRA7	-2,32785	7,57E-10
RCBTB1	-2,33039	6,15E-16
CDK6	-2,3347	3,91E-05
FAM160A1	-2,33951	2,88E-07
MRC2	-2,34043	0,000258
IFNGR1	-2,3445	1,48E-09

SPRY1	-2,34492	0,00053
DCBLD1	-2,35436	4,09E-10
GALT	-2,3612	0,000396
SNX25	-2,36325	7,66E-12
SMIM3	-2,36874	4,63E-12
ZFP36	-2,369	3,62E-09
BRWD3	-2,36931	2,73E-07
POU6F1	-2,36943	0,00014
CDPF1	-2,37138	1,89E-07
RASSF3	-2,37473	9,23E-13
DOCK5	-2,38088	7,54E-15
SSFA2	-2,39261	9,80E-17
IL27RA	-2,40028	7,09E-16
SCCPDH	-2,403	5,64E-15
KIF13B	-2,40374	4,90E-06
ME2	-2,40414	5,44E-17
SH3BP4	-2,40591	3,70E-18
TUBB2A	-2,4063	4,75E-07
TBC1D8B	-2,41703	0,000304
SLC16A14	-2,4194	7,21E-08
LCA5	-2,42774	9,41E-05
GPAT3	-2,43031	3,45E-06
GLS	-2,43293	6,18E-20
WWC2	-2,43578	1,23E-18
ITPKA	-2,4373	1,17E-06
FURIN	-2,43915	3,84E-15
DDAH1	-2,44468	1,62E-09
IL6ST	-2,445	6,06E-20
TNFRSF10B	-2,44568	8,21E-08
ESAM	-2,44629	0,000129
RAPGEFL1	-2,44815	7,79E-07
BTG3	-2,44883	2,15E-07
TUBE1	-2,44947	3,32E-06
SPNS2	-2,44999	0,000162
TUBA8	-2,45068	6,00E-06
RASGRP1	-2,45978	0,000334
TRMT11	-2,46015	3,62E-06
PTGR1	-2,46358	4,19E-09
LIX1L	-2,46457	0,000465
SHROOM2	-2,46898	7,10E-10
FUT1	-2,47071	0,000171
KLHL29	-2,47291	4,50E-06
ADCY9	-2,4779	1,15E-13
NCOA7	-2,47867	2,62E-10
METTL7B	-2,47926	6,01E-09
SLC1A3	-2,48221	8,35E-05
SLC8B1	-2,48358	2,39E-12
GSDMD	-2,48867	1,37E-06
TNRC6C-AS	-2,49004	0,000358
HLA-C	-2,49288	6,60E-06

ADAM9	-2,49602	7,93E-27
NKX3-1	-2,49694	4,16E-08
LBX2-AS1	-2,5025	0,000316
PLEKHG1	-2,50708	0,000783
ACSL4	-2,50783	4,54E-14
KBTBD11	-2,50783	2,87E-07
HPSE	-2,51161	9,18E-07
UNC13D	-2,51212	0,000246
PRNP	-2,51245	3,36E-11
SNORA73A	-2,51625	0,000397
APOBEC3B	-2,518	9,23E-13
CPOX	-2,52658	8,38E-08
ARAP2	-2,53172	2,33E-11
SLCO4A1	-2,53193	1,77E-10
MAMDC4	-2,53587	0,000318
HLA-F	-2,53955	4,99E-05
DIAPH2	-2,53985	1,14E-14
MICB	-2,54665	2,35E-07
ADAP2	-2,54869	1,51E-06
SERPINB8	-2,55944	5,51E-05
CHIC1	-2,56434	9,33E-07
FOXD1	-2,57167	0,000325
HSD17B1	-2,57774	2,95E-10
CAMK2D	-2,57982	4,45E-13
KIAA1804	-2,58011	7,44E-09
LBR	-2,58012	5,65E-17
RTN4RL2	-2,58313	0,000913
SLC52A3	-2,58445	1,25E-05
SLC39A14	-2,58486	1,12E-09
KCNK5	-2,58536	2,66E-06
DCBLD2	-2,5872	1,48E-07
GPC1	-2,59115	9,26E-22
NIPAL3	-2,59295	7,80E-11
VPS13C	-2,60397	4,68E-17
ZBTB47	-2,60437	0,000351
MCF2L	-2,60447	8,53E-10
MCF2L-AS1	-2,60522	0,000454
PRPS2	-2,60582	1,69E-19
GK	-2,60889	1,05E-09
CIRBP-AS1	-2,61801	0,000406
ADGRE5	-2,62016	9,68E-12
MYB	-2,63801	2,50E-10
IFIT5	-2,63849	1,73E-08
PAQR5	-2,64026	7,78E-17
MTCL1	-2,64044	1,19E-08
CYP27B1	-2,64878	0,00054
MLXIPL	-2,65227	0,00019
REEP2	-2,65397	2,25E-09
NFKBIA	-2,66128	2,49E-14
AIM1L	-2,66692	0,000105

GNPDA2	-2,66966	1,30E-08
ZNF589	-2,67172	6,60E-15
SGMS2	-2,67581	4,89E-09
IFNLR1	-2,6764	3,37E-09
RTTN	-2,68151	1,90E-10
CCDC3	-2,68749	1,19E-06
ABHD6	-2,68842	2,36E-09
COCH	-2,69262	9,09E-07
MX1	-2,69422	7,40E-06
ACOT11	-2,69824	0,000254
LRRC75A	-2,70716	3,82E-05
HDAC4	-2,71021	3,33E-05
TK2	-2,71341	2,05E-09
CNTLN	-2,71473	8,47E-11
GJB2	-2,71995	1,60E-14
LOC28402[illegible]	-2,72979	1,70E-06
ATP8A1	-2,73419	5,00E-07
SCNN1A	-2,73479	0,00014
SOWAHC	-2,7375	8,25E-12
OPTN	-2,73816	1,60E-06
HBEGF	-2,74703	0,000175
FHOD3	-2,75206	0,000673
SLC25A22	-2,75922	1,05E-17
SEMA3B	-2,76265	1,05E-09
PM20D2	-2,77115	4,65E-15
FOXO1	-2,77709	1,89E-07
TAPBPL	-2,77882	0,000186
RAB11FIP5	-2,78445	1,19E-08
NFKBID	-2,79229	0,000137
ITPRIPL2	-2,79484	6,20E-26
C1orf106	-2,79535	2,88E-11
SGTB	-2,79858	2,41E-10
PLCXD1	-2,79953	5,55E-12
FRAS1	-2,8084	1,85E-28
RAPGEF5	-2,82033	9,32E-09
PRRG4	-2,82049	2,80E-08
CENPV	-2,82147	3,19E-09
SPRY2	-2,83317	1,97E-09
ARPIN	-2,83718	6,19E-10
SNX10	-2,83828	4,95E-14
SUSD1	-2,8423	4,06E-15
TRAF5	-2,84581	1,47E-17
HOOK3	-2,84585	1,79E-20
DNMBP	-2,84869	2,68E-17
C11orf54	-2,858	1,38E-14
TMEM56	-2,859	6,17E-14
CD27-AS1	-2,86318	0,000674
NDRG1	-2,86498	2,99E-12
NMNAT3	-2,87135	6,50E-06
KBTBD8	-2,87302	2,31E-05

MOCOS	-2,87379	1,35E-10
NUAK2	-2,87514	2,83E-11
ECE1	-2,87558	3,78E-25
CHST3	-2,87728	5,33E-05
KLF12	-2,8832	2,18E-13
GOLM1	-2,88622	4,84E-30
ZC3H12A	-2,89681	6,16E-13
PHLDA2	-2,9009	2,12E-09
DUSP28	-2,90127	0,000711
ANK2	-2,92312	0,000164
TGFBR3	-2,92655	7,77E-05
FERMT1	-2,92751	5,43E-24
SLC16A9	-2,93583	2,40E-14
NFKB1	-2,93841	4,93E-16
CRIM1	-2,94964	5,18E-12
DNHD1	-2,95237	1,77E-05
DSE	-2,95707	4,27E-09
WBSCR27	-2,96198	8,20E-06
INSR	-2,97487	9,52E-19
RAMP1	-2,97538	9,24E-10
TRIP10	-2,97855	7,41E-16
PLIN4	-2,99543	0,000273
ADGRF4	-2,99625	0,000495
LGALS3BP	-2,9996	2,24E-06
CRACR2A	-3,00054	4,45E-05
CDC42EP3	-3,00092	6,65E-19
HHEX	-3,00454	3,64E-06
ATF7IP2	-3,01029	8,79E-05
ZNF264	-3,02699	1,65E-09
OAF	-3,03024	1,16E-13
MMP24	-3,03267	9,94E-05
LIPH	-3,03669	1,84E-05
ALDH3B1	-3,05807	1,74E-06
TBC1D4	-3,06411	1,20E-11
MORC4	-3,06665	1,31E-36
PROCR	-3,0723	0,000126
FXYD5	-3,07312	6,42E-27
C19orf66	-3,07955	1,48E-06
ATHL1	-3,0809	1,28E-09
CD83	-3,08332	1,17E-10
GLS2	-3,09017	5,17E-28
STEAP1	-3,09439	1,31E-05
NFAT5	-3,09756	1,09E-20
LYST	-3,10057	1,94E-10
FAM169A	-3,11048	8,84E-10
FNIP2	-3,1165	2,00E-08
ITGA5	-3,11731	6,75E-18
SLC17A5	-3,11816	2,31E-16
PLEKHA2	-3,12516	1,37E-16
FAM63B	-3,13138	1,41E-11

IGF2BP2	-3,13908	5,26E-21
HSH2D	-3,14921	9,86E-06
F2RL1	-3,14979	2,08E-18
NFKB2	-3,15197	1,31E-32
PLAGL1	-3,18716	8,27E-18
PMP22	-3,20023	2,01E-07
ADM	-3,21282	0,000602
RHEBL1	-3,21288	2,73E-05
FAM171A1	-3,22029	2,03E-20
ATP2A1	-3,22257	0,000617
TIMP1	-3,22288	9,44E-09
PGM1	-3,22685	7,20E-22
XK	-3,22715	2,69E-18
SLC9A5	-3,22719	7,01E-05
IRF5	-3,24035	8,84E-17
IL18	-3,25941	0,000154
HERC5	-3,27179	0,0005
IL15RA	-3,29544	4,92E-13
CBX7	-3,29778	2,25E-07
EHF	-3,29991	1,22E-15
HPGD	-3,3043	0,000968
GULP1	-3,30507	3,19E-14
TAGLN	-3,3057	3,66E-06
BMP4	-3,30753	4,84E-18
PLK3	-3,31056	1,47E-12
VWDE	-3,31341	1,62E-06
ADAP1	-3,32313	4,92E-24
HNRNPU-A	-3,32897	1,37E-08
PLBD1	-3,33387	8,59E-17
CLIP2	-3,33892	3,21E-38
TRAF1	-3,34324	1,18E-07
APC2	-3,34343	6,15E-08
MAPRE2	-3,34568	6,92E-17
HSD17B11	-3,35045	1,65E-13
SNX7	-3,35258	1,31E-13
ANXA1	-3,35367	1,80E-11
RILPL2	-3,35943	1,95E-10
CCDC68	-3,35951	4,76E-05
ACO1	-3,36008	4,67E-21
ALOXE3	-3,37134	1,09E-13
SLC12A4	-3,37478	9,32E-08
TFCP2L1	-3,37772	0,000322
PARM1	-3,38181	2,04E-08
FAHD2B	-3,3833	1,16E-14
ADORA2B	-3,39249	2,43E-15
B3GNT9	-3,39573	8,77E-06
FAM149A	-3,40064	0,000948
RBMS2	-3,40324	3,49E-20
LGALS3	-3,41367	3,94E-19
TMEM51-A	-3,41378	0,000446

CNTD2	-3,41483	7,66E-05
SASH1	-3,42686	4,41E-13
BCL2L15	-3,42965	2,34E-06
CCDC69	-3,43733	8,99E-12
FAM135A	-3,45014	4,66E-21
PDE2A	-3,45442	0,000175
WNT5B	-3,45532	8,06E-08
ZNF814	-3,47568	3,23E-08
SERAC1	-3,49262	2,38E-21
ARNTL2	-3,49858	2,48E-24
SLC22A15	-3,50259	1,07E-05
TSPAN1	-3,51577	3,54E-06
ASPH	-3,51648	2,42E-23
TMEM221	-3,52256	0,000154
UPP1	-3,52501	3,16E-08
GPR63	-3,53582	1,15E-10
SDC4	-3,53921	4,77E-15
FN3K	-3,55129	5,85E-10
SRPX	-3,55196	2,41E-07
RPS6KA6	-3,5536	2,24E-09
ULBP3	-3,55976	4,09E-07
LRRC8C	-3,56105	3,26E-15
FBXO2	-3,56567	2,22E-13
IER3	-3,57213	2,61E-16
SP100	-3,57286	2,59E-05
DPY19L2P2	-3,57774	2,17E-07
SLC30A4	-3,58284	0,000177
C16orf62	-3,58832	5,11E-31
DDN	-3,6028	4,33E-06
IZUMO1	-3,62035	1,18E-12
KLK1	-3,62895	0,000304
ALDH3A1	-3,63151	1,63E-06
SCNN1D	-3,63319	4,12E-09
MCIDAS	-3,6497	3,54E-06
C3	-3,66677	0,000474
CHRNB1	-3,66762	2,05E-12
NAPRT	-3,67062	5,34E-24
C9orf72	-3,68903	1,54E-06
SLC16A2	-3,70363	9,58E-09
HOXA11	-3,71052	1,26E-07
ANXA3	-3,71222	1,16E-20
PLCE1	-3,71761	5,38E-05
TYW1B	-3,72304	3,57E-08
ATAD3B	-3,72631	2,40E-17
SPATA3-AS	-3,72823	5,49E-05
ITGA6	-3,75076	6,42E-27
PSMB9	-3,76306	1,24E-07
HERC2P3	-3,77126	1,11E-06
ZNF702P	-3,77318	2,05E-11
JAG1	-3,77797	1,52E-40

WLS	-3,78813	8,44E-27
STARD9	-3,79887	6,99E-09
LIMCH1	-3,79925	2,10E-11
CPVL	-3,80077	2,05E-07
BHLHA15	-3,80353	3,52E-08
ERRFI1	-3,81226	1,16E-12
FUT4	-3,81387	5,05E-19
IL4R	-3,81622	3,15E-21
HLA-B	-3,83615	1,91E-12
CEACAM19	-3,8399	8,91E-10
RELB	-3,84976	3,62E-20
RASGRF2	-3,85115	2,36E-05
RNF207	-3,85203	1,19E-11
PXK	-3,8564	2,26E-10
DPYSL3	-3,85694	3,20E-15
FRMD5	-3,85803	9,43E-23
FAM124A	-3,86999	9,67E-05
MAP3K8	-3,87189	1,33E-12
ST3GAL2	-3,87221	1,12E-15
NYAP1	-3,8749	1,01E-06
LINC01315	-3,89466	5,21E-06
TRPV1	-3,90082	7,73E-10
ST6GALNA(	-3,908	4,70E-22
PRKCA	-3,90984	8,95E-22
FER1L4	-3,92856	3,74E-09
FAM13A	-3,94867	1,21E-16
TRIM47	-3,94969	3,96E-30
SERPINE2	-3,95866	2,04E-17
EPHX4	-3,97181	7,37E-09
SPTBN5	-3,97793	0,000144
SOX7	-3,98097	7,77E-07
CADPS2	-3,9857	6,19E-06
EML5	-3,99709	9,87E-12
AKAP12	-4,00625	1,54E-21
APOLD1	-4,00773	2,16E-05
CD74	-4,01019	7,40E-07
KCNQ1	-4,03685	1,64E-06
AKAP7	-4,04986	5,60E-09
JAZF1	-4,05751	7,14E-08
MBP	-4,05842	2,21E-28
TNS4	-4,06794	1,93E-07
SEPT10	-4,08229	2,50E-19
WDR90	-4,09486	1,27E-29
FAM222A-/	-4,11737	7,40E-05
CMTM7	-4,12574	2,50E-26
TGFB1I1	-4,13731	2,55E-05
SH2B3	-4,14482	5,04E-48
STX11	-4,14625	2,17E-05
LRRK1	-4,15127	2,81E-24
HES7	-4,15211	8,23E-14

IL20RA	-4,15677	2,16E-14
FUT3	-4,15867	7,49E-09
GABARAPL	-4,17218	6,39E-09
IGFL2	-4,17539	7,01E-12
RGL3	-4,17665	1,52E-06
C3orf52	-4,17721	2,72E-07
C3orf18	-4,18737	9,49E-08
F3	-4,22096	4,52E-24
PLA2G16	-4,2254	7,92E-18
FAT1	-4,23718	2,04E-40
CYP4V2	-4,24917	3,61E-11
HLA-A	-4,25773	2,38E-43
ARHGAP23	-4,27516	1,13E-16
UBASH3B	-4,27805	1,09E-24
FAM83F	-4,32035	1,02E-12
EPHA2	-4,32446	2,40E-28
PPM1K	-4,33079	8,47E-15
QPCT	-4,33256	1,18E-26
BDKRB2	-4,3335	8,84E-07
ANKRD29	-4,34766	3,89E-10
FHL2	-4,3606	1,47E-12
B3GNT5	-4,37197	1,19E-09
NRP2	-4,3783	1,20E-12
PDE8B	-4,37917	6,76E-06
IGFL4	-4,41236	0,000447
SH3RF2	-4,41276	2,40E-20
VWA5B2	-4,41858	0,000445
VWA2	-4,4226	1,56E-13
ZNF513	-4,4409	2,03E-12
HOXA7	-4,4542	4,19E-09
SEC31B	-4,50459	0,000396
RASIP1	-4,52925	5,50E-10
ANKRD33B	-4,55089	1,08E-07
SERPINE1	-4,56709	7,27E-07
LINC00673	-4,57462	5,69E-06
PLAUR	-4,57582	1,55E-14
TESC	-4,58217	1,07E-39
SPRED1	-4,60164	3,31E-24
NR4A3	-4,61093	2,25E-10
STAMBPL1	-4,63879	4,88E-19
KHDRBS3	-4,64457	2,65E-06
PELI1	-4,64988	1,04E-30
C14orf159	-4,65358	8,60E-31
SLC1A1	-4,65977	4,15E-15
SNORD30	-4,66946	0,000857
MYH15	-4,67273	3,09E-05
NOD2	-4,68202	2,26E-08
ARHGAP40	-4,71015	5,66E-05
XDH	-4,71497	0,000457
ULBP2	-4,71536	2,33E-10

HOXC8	-4,73061	3,96E-11
CNTNAP1	-4,74536	4,29E-38
IL22RA1	-4,77275	2,93E-06
MCOLN2	-4,78203	2,69E-18
AMPD3	-4,78806	5,67E-32
LOC10013(	-4,79081	2,62E-15
EVA1C	-4,84	5,85E-26
LAMC2	-4,8471	2,35E-32
CAPN2	-4,8514	2,99E-26
HOXA13	-4,87311	1,35E-05
PDE10A	-4,89094	0,000138
HOXD11	-4,89873	1,23E-07
PITX2	-4,94484	2,43E-31
TUBA4A	-4,95151	1,90E-19
GJB3	-4,975	8,55E-18
SGPP2	-4,98018	2,86E-32
ARHGEF40	-4,986	1,00E-16
DUSP6	-4,99319	5,00E-08
KIFC3	-5,00167	5,15E-18
KLF15	-5,01642	1,59E-14
PDCL3P4	-5,02593	8,19E-06
GNB3	-5,05157	0,000155
TOR4A	-5,05999	4,03E-58
SLC45A1	-5,06775	0,000512
EGFR	-5,07421	4,57E-53
PLAC8	-5,09172	2,89E-05
CORO2B	-5,09953	1,15E-10
TRANK1	-5,11314	3,16E-08
SERPINB9	-5,11534	1,61E-33
HCP5	-5,12198	2,17E-07
HNF4G	-5,13741	1,45E-20
GNAL	-5,16116	6,80E-45
PNMA2	-5,18261	2,70E-18
MID1	-5,18538	1,06E-21
GFI1	-5,21092	2,86E-12
MAFF	-5,21393	7,51E-09
CD274	-5,22275	1,30E-05
LIF	-5,2846	6,19E-18
TNFAIP3	-5,29813	1,72E-40
STK32A	-5,32547	0,000194
TNNC1	-5,32597	6,04E-11
FOSL1	-5,33083	2,72E-38
RIN1	-5,35273	1,89E-37
TMEM98	-5,36971	5,50E-36
DAPK1	-5,37567	3,70E-10
SCG5	-5,38662	1,43E-05
PIK3AP1	-5,40227	3,63E-19
KIF7	-5,4074	0,000259
SATB1	-5,43115	8,27E-10
NCR3LG1	-5,43222	8,97E-43

FAM184A	-5,43231	1,67E-06
NPHS1	-5,44178	3,04E-14
NAP1L5	-5,47704	1,12E-07
PROS1	-5,48901	5,21E-20
CA2	-5,49616	4,63E-16
HOXB13	-5,51505	1,57E-15
SLC2A3	-5,51639	1,59E-17
LAT2	-5,53163	8,06E-08
PLEKHA4	-5,55802	3,56E-27
PCSK5	-5,55836	1,20E-05
MPP1	-5,58069	8,72E-21
SRMS	-5,59012	1,99E-09
ILDR2	-5,59481	4,80E-09
YOD1	-5,60098	7,23E-24
CRYBG3	-5,63652	6,12E-37
EPB41L2	-5,63705	1,52E-79
MEIS2	-5,67206	8,08E-09
PLCL2	-5,67506	5,64E-08
ETV4	-5,67571	4,24E-18
TPM2	-5,6791	4,34E-27
CR2	-5,68404	1,14E-27
ACOT2	-5,69552	3,74E-14
MTAP	-5,71326	5,08E-26
SH3RF3	-5,7168	3,69E-11
PRRT3-AS1	-5,74534	2,56E-07
MOB3B	-5,75927	3,56E-09
TLE6	-5,76722	0,000455
GSTM2	-5,77807	0,000197
ME3	-5,78285	1,11E-17
CX3CL1	-5,7859	2,49E-10
SGSM1	-5,8132	3,57E-09
LYN	-5,84898	4,00E-40
CRAT	-5,85987	1,91E-25
APOL6	-5,8817	3,44E-37
EMP1	-5,89723	0,000791
CASP10	-5,90242	2,40E-20
ANKRD22	-5,90689	1,25E-09
CLMP	-5,91883	3,41E-07
DPP4	-5,92046	2,51E-11
AMN	-5,92084	1,22E-05
GAL3ST2	-5,9316	3,53E-09
C6orf223	-5,93908	9,47E-13
BZRAP1	-5,95224	3,54E-09
EPAS1	-5,95409	1,16E-25
ETV7	-5,965	1,51E-17
MST1R	-5,97141	9,81E-49
ELFN1-AS1	-5,9836	1,31E-17
SPRY4	-6,0046	1,58E-43
RASSF6	-6,01148	3,00E-06
TLR2	-6,03156	1,66E-10

SNPH	-6,03297	3,40E-06
CSF1	-6,06801	3,79E-61
SOX8	-6,09434	2,94E-06
NFKBIZ	-6,09456	1,12E-31
RGS11	-6,10403	7,48E-05
ERAP2	-6,11696	5,29E-36
NPIPA5	-6,17999	1,52E-09
UCA1	-6,18489	0,000283
ARSJ	-6,18637	1,20E-06
HOXB6	-6,20374	2,40E-22
ZBTB18	-6,22223	8,23E-28
DGKG	-6,22389	3,40E-05
PAK6	-6,23015	2,88E-39
PPP1R14C	-6,26818	1,04E-09
INPP1	-6,27813	5,94E-19
HOXD10	-6,29922	2,25E-05
NLRC5	-6,3077	5,40E-29
SPTB	-6,31074	7,60E-25
ZC3H12C	-6,33273	7,30E-41
TRIM38	-6,35262	3,26E-13
HOXB4	-6,41528	8,38E-11
TRABD2A	-6,4191	1,21E-17
CACNB4	-6,41992	2,45E-22
KLK10	-6,42881	6,64E-09
ARMCX4	-6,54363	1,14E-10
HOXB9	-6,56804	1,91E-06
GLI2	-6,60472	1,03E-11
TMEM173	-6,63724	1,72E-15
PALD1	-6,64418	1,25E-110
COL4A2	-6,65634	1,02E-28
FAM65B	-6,68324	0,000753
HNF4A	-6,68926	1,25E-29
MCTP2	-6,71872	7,47E-20
RNF217	-6,74076	3,63E-09
ERO1B	-6,77837	8,73E-24
ACY3	-6,79439	0,000821
OSBPL3	-6,795	1,00E-30
ZDHHC2	-6,79659	3,40E-46
STAT5A	-6,82889	2,68E-05
ZNF71	-6,88119	5,29E-07
BIN1	-6,89757	6,66E-76
LCN2	-6,92761	2,66E-07
BIRC3	-6,93893	1,59E-30
TMEM200E	-6,94078	9,30E-18
LINC01138	-7,02913	1,67E-07
PEAR1	-7,15015	0,000138
RAET1L	-7,17447	0,00013
C1QTNF1	-7,21212	1,91E-16
ZNF426	-7,24091	1,69E-16
APOBEC3C	-7,24194	3,06E-23

USP32P1	-7,27813	8,32E-05
TLE4	-7,2969	9,55E-05
ETV5	-7,32036	3,07E-53
CD14	-7,37847	1,19E-08
JSRP1	-7,3902	0,000119
TNC	-7,40136	8,65E-05
ZC4H2	-7,42415	4,80E-09
TFCP2	-7,42555	0,000816
ZNF717	-7,45689	9,75E-09
ALDH1L1	-7,56361	1,59E-25
LINC00857	-7,5881	3,00E-09
CTGLF12P	-7,59247	0,000563
EPHA1-AS1	-7,606	0,00052
ZNF69	-7,60861	0,00053
CARD6	-7,6145	0,000502
TMEM200/	-7,61538	0,000785
C2orf70	-7,61614	0,000489
NEK9	-7,6174	1,47E-05
IL32	-7,62591	1,11E-08
ERICH5	-7,64403	3,28E-17
DPYD	-7,72141	0,000349
ONECUT3	-7,72414	0,000353
BTBD19	-7,72546	1,32E-05
NPR2	-7,74339	2,22E-05
OSR1	-7,74425	1,24E-05
DEPDC7	-7,76641	0,000258
FOXQ1	-7,78447	1,33E-19
GRIN2B	-7,78938	0,000273
TNFRSF14	-7,79536	3,70E-10
FBLN7	-7,81248	0,000461
HS3ST1	-7,82129	9,19E-06
SLC38A8	-7,86371	0,000421
LOC389602	-7,88205	0,000154
TRIM15	-7,89495	1,23E-35
CA9	-7,90636	6,00E-19
LIPE	-7,92448	1,75E-10
PVRL3	-7,9329	0,000129
ICAM1	-7,93354	8,98E-49
KCNE3	-7,98363	5,11E-06
HOXD-AS2	-7,99688	9,19E-05
ADAM21	-8,01326	0,000101
CTSZ	-8,01832	4,61E-55
RARRES1	-8,01874	0,00015
CD6	-8,03076	0,000138
BHLHE41	-8,04984	5,00E-06
ITGAX	-8,08215	0,000107
ACSL5	-8,08725	2,33E-53
C8orf31	-8,1095	5,49E-05
MIR31HG	-8,11873	5,33E-05
PDZK1IP1	-8,12724	5,52E-05

ALDH2	-8,13648	6,50E-74
MUC13	-8,15354	4,97E-05
HLA-H	-8,16899	1,55E-11
LYPD5	-8,17355	3,18E-06
PTPRD	-8,19554	3,82E-05
HRCT1	-8,20227	3,87E-05
TLR6	-8,20556	3,62E-05
SBSPON	-8,21177	5,34E-05
WNT16	-8,23852	5,06E-05
UGT8	-8,24737	4,94E-05
FGFBP1	-8,26869	2,50E-05
FEZF1-AS1	-8,27696	2,41E-05
MDFIC	-8,28892	1,01E-16
CES4A	-8,30432	2,11E-05
LINC01152	-8,30545	6,47E-05
WIPF3	-8,31139	3,55E-20
CSF2RA	-8,31714	6,24E-05
EBI3	-8,32174	7,48E-05
SOX9	-8,33228	1,25E-80
GPR35	-8,34304	6,31E-31
NEURL3	-8,34779	3,97E-05
NPR1	-8,35184	2,85E-11
CDHR5	-8,37315	0,000113
HOXB8	-8,37974	1,14E-21
P3H3	-8,38897	2,84E-06
C1RL	-8,41005	1,36E-20
LINC00899	-8,43489	1,40E-05
GBP3	-8,48357	4,61E-07
PHLDB2	-8,49304	9,71E-38
TMBIM1	-8,52309	3,13E-69
ICAM4	-8,52957	8,52E-06
TLDC2	-8,53159	6,75E-06
DUSP5P1	-8,54734	7,87E-06
ZNF280A	-8,54888	7,61E-06
CCL26	-8,57952	8,48E-06
ZFP57	-8,58487	1,26E-23
CDH12	-8,59284	5,41E-06
ZC3H12B	-8,62411	4,20E-06
RARRES2	-8,63341	8,19E-06
ADAMTSL2	-8,63781	9,04E-06
ZNF559	-8,69511	5,00E-06
LONRF3	-8,6963	2,35E-13
ZNF470	-8,73891	2,96E-06
ZNF649	-8,74064	2,29E-06
EVX1	-8,75056	9,99E-06
STEAP2	-8,75167	5,01E-06
RRN3P1	-8,77623	1,94E-06
LTB	-8,78645	7,26E-13
VSNL1	-8,82621	5,09E-14
C4BPB	-8,83303	4,78E-06

SLC7A7	-8,83462	3,03E-20
CSF2	-8,842	3,30E-06
TLR3	-8,85649	1,25E-06
CST7	-8,87556	1,25E-06
GIPR	-8,88083	1,08E-06
AKT3	-8,89928	1,07E-06
LRRN4	-8,91101	5,58E-06
KLK6	-8,91989	6,99E-25
LOC72997(	-8,97149	7,01E-07
TMEM220	-8,98193	1,34E-06
LAMA1	-9,01195	5,34E-07
ADGRG5	-9,04198	4,53E-07
HOXD13	-9,04472	7,45E-07
ZNF175	-9,06723	3,99E-07
SERP2	-9,06891	4,22E-07
TIMP4	-9,07039	7,85E-07
PAX6	-9,0772	3,75E-07
DMRTA2	-9,10424	2,53E-08
CDH17	-9,12607	2,19E-06
SLC6A20	-9,12668	3,13E-07
LOC101928	-9,13019	1,12E-06
C11orf45	-9,14255	2,97E-07
HOXB3	-9,15027	4,18E-22
NOS2	-9,15848	1,93E-08
NRN1	-9,17241	2,78E-07
PRKCQ-AS1	-9,17257	2,35E-07
FGF2	-9,18857	7,64E-29
PSMB8	-9,19339	4,86E-27
C7orf31	-9,20478	2,55E-07
GATA2-AS1	-9,21726	1,85E-08
LRRC34	-9,22335	2,14E-07
TNFSF12	-9,22502	1,69E-07
DLX6	-9,22845	4,83E-07
LINC00941	-9,28622	1,22E-07
SH3GL2	-9,29798	3,22E-08
HOXB-AS3	-9,32047	5,86E-07
ABCB1	-9,38316	2,59E-86
CCL28	-9,38556	7,85E-08
FAM27E3	-9,40992	1,78E-07
CELF2	-9,42568	5,61E-08
DMD	-9,43736	5,36E-08
C1RL-AS1	-9,44296	5,16E-08
ICAM2	-9,45128	5,92E-09
HOTTIP	-9,49677	3,92E-08
CADPS	-9,51276	1,25E-14
TMSB4Y	-9,52524	3,51E-08
TMEM52B	-9,52644	2,84E-07
PRKCQ	-9,56007	4,46E-08
LOC11511(	-9,581	4,57E-08
FRG1CP	-9,62767	1,79E-08

MAP1LC3A	-9,68045	1,36E-08
GSDMA	-9,71269	3,89E-07
AGPS	-9,77338	1,50E-54
BCL11A	-9,81767	6,57E-09
CCAT1	-9,86901	9,09E-09
KLK7	-9,90803	2,75E-08
HOXD9	-9,91462	3,64E-09
LINC01224	-9,93851	3,27E-09
PRKY	-10,0719	2,90E-09
TTTY15	-10,0812	1,41E-09
ABO	-10,0842	1,47E-09
NUDT16P1	-10,1288	1,35E-09
DPEP1	-10,1403	7,38E-18
RAB38	-10,156	9,52E-10
CA13	-10,2112	6,86E-10
ETS1	-10,2174	2,20E-09
FAM71E1	-10,2175	8,35E-10
HOXA9	-10,2491	5,52E-10
ARHGAP31	-10,2607	9,02E-11
TRIM10	-10,2821	4,76E-10
PLAT	-10,3271	7,50E-10
EREG	-10,3476	1,00E-13
LDHB	-10,3483	0,000573
ARSE	-10,365	2,83E-10
GPRIN2	-10,4004	2,73E-10
MT1A	-10,4332	1,28E-09
GSTP1	-10,4442	6,66E-122
FOXA3	-10,4469	1,96E-10
PDX1	-10,4553	1,84E-10
HRH1	-10,5017	2,88E-10
HMGA2	-10,523	7,97E-11
UTY	-10,6317	6,54E-11
ZNF215	-10,6922	4,44E-11
TNFRSF1B	-10,694	4,31E-11
NT5E	-10,7104	1,73E-10
GJB6	-10,7497	3,86E-11
EPS8L3	-10,8307	3,08E-11
TRIM58	-10,8347	1,11E-10
ZC3HAV1L	-10,857	2,42E-11
HOXB5	-10,8728	1,56E-11
CYB5R2	-10,893	1,67E-11
ZNF280B	-10,9092	1,34E-11
SDHAF3	-10,989	8,76E-12
ZFY	-11,0521	9,53E-12
CCL20	-11,0895	7,84E-05
GDA	-11,1007	2,87E-11
TNFSF15	-11,1344	1,81E-05
SAMD5	-11,167	3,56E-12
RAC2	-11,1725	7,02E-12
KCNJ5	-11,2668	4,84E-11

HOXA10-AS	-11,402	6,95E-13
AASS	-11,4903	5,85E-13
TNFRSF9	-11,5652	2,73E-13
HOXA10	-11,6178	1,99E-13
TNFRSF10D	-11,6206	2,45E-13
TXLNGY	-11,6929	1,25E-13
PRSS3	-11,7796	7,37E-14
ZNF320	-11,8209	5,82E-14
FUT2	-11,8411	7,23E-14
PLAU	-11,8602	1,06E-13
TCEAL8	-11,8624	4,63E-14
CXCL3	-11,8737	1,58E-12
TINAGL1	-11,8843	6,94E-14
GSPT2	-11,8991	3,93E-14
KDM5D	-11,9224	7,64E-14
ZNF518B	-11,9282	3,13E-14
C5orf42	-11,9633	2,63E-14
CYBRD1	-12,0441	2,02E-14
HNF1B	-12,0528	1,64E-14
KIRREL	-12,0532	1,67E-14
EIF1AY	-12,0881	1,41E-14
USP9Y	-12,0882	1,20E-14
SLCO1B3	-12,1433	1,29E-14
WBP5	-12,2765	4,12E-15
GALNT5	-12,2822	1,17E-05
VIL1	-12,4378	2,88E-15
TMEM246	-12,5519	8,80E-16
GNE	-12,5863	1,26E-15
NLRP2	-12,6137	1,02E-15
CXCL1	-12,6815	3,94E-09
SIGMAR1	-12,7846	5,91E-32
C16orf96	-12,8441	1,28E-16
C2CD2	-12,857	1,75E-16
ZCCHC11	-12,949	7,98E-17
IGF2BP3	-13,1502	1,78E-17
CXCL2	-13,4363	4,70E-08
GPX1	-13,5091	3,40E-18
CXCL5	-13,5715	1,25E-18
CNN3	-13,5781	6,12E-19
DDX3Y	-13,7174	1,60E-18
MT1E	-13,8595	3,61E-19
EMC10	-14,1193	5,21E-20
CLDN2	-14,4213	6,32E-21
OLR1	-15,9408	4,61E-25
AFAP1-AS1	-17,1271	1,52E-29

HCT116

Gene	log2FoldCh	FDR
AKAP12	-8,45032	7,69E-144
TPM2	-9,35571	5,80E-124
GSTP1	-10,5241	8,57E-113
RHOBTB1	7,331974	2,17E-110
ANXA1	-6,75054	3,35E-110
SOX9	-8,57732	7,28E-99
SPRY4	-8,17914	3,72E-88
SERPINE2	-6,38873	4,65E-74
EPB41L2	-5,46721	8,57E-70
PALD1	-6,18134	1,15E-68
FOXA1	5,410203	3,25E-67
HOMER2	7,621033	1,07E-66
RBP1	-9,25166	2,88E-66
CTSZ	-8,88751	4,24E-64
COL12A1	-8,26702	8,61E-63
ETV5	-8,04297	3,99E-61
STC1	6,744312	1,43E-60
BIN1	-6,70767	4,02E-60
ACSL5	-9,20434	5,76E-60
TMBIM1	-8,33587	3,00E-59
EGFR	-5,66357	1,56E-58
TENM3	7,060486	1,96E-58
MAGEB2	-9,56922	2,98E-58
ADAM19	-6,30449	1,37E-57
VCAN	-9,05153	1,60E-57
PRSS23	6,220383	7,12E-57
ZNF703	4,814344	5,70E-56
PHLDB2	-10,4676	4,85E-55
MEX3A	4,343738	5,35E-54
NPW	-4,9696	2,25E-53
FLRT3	6,528264	2,60E-53
OSBPL3	-8,41833	2,44E-51
ANO1	-10,3897	3,99E-51
FAM101B	-4,93268	4,93E-50
IGF2BP2	-4,58798	5,19E-50
PRMT6	4,246137	6,08E-50
AGPS	-9,61246	6,21E-50
MID1	-6,88042	9,15E-50
PIK3R3	6,533346	6,09E-49
SLC38A5	-9,098	1,99E-48
YBX2	7,825385	4,99E-48
CD109	-5,70311	4,82E-47
CD24	5,901124	1,71E-46
CMTM7	-5,36566	2,22E-46
RIN1	-6,09716	2,33E-46
MYEOV	-5,90676	6,06E-46
STAP2	7,046507	2,29E-45
BMP7	3,831037	1,07E-44

FRMD5	-5,08709	3,49E-44
LYN	-6,11729	5,69E-44
ITGA5	-5,3316	8,49E-44
ARHGAP29	-4,90908	1,64E-43
SULF2	4,924137	1,68E-43
FAM213A	5,481121	4,44E-43
JAG1	-5,02959	6,61E-43
CRIP1	-5,91774	1,18E-42
EPAS1	-6,84569	1,55E-42
BAMBI	4,043334	3,76E-42
TNFRSF19	5,473121	4,71E-42
MSX1	-5,20373	5,84E-42
SLC9A2	5,374113	9,99E-42
ALDH2	-7,36833	2,01E-41
UNC13A	-6,58406	1,92E-40
TOR4A	-5,11299	7,05E-40
FBXO2	-5,76396	2,07E-39
PDP1	-4,86571	3,56E-39
EMP3	-7,70822	8,15E-39
SERPINB9	-5,60564	9,30E-39
GATA3	5,742156	1,49E-38
SYTL3	-5,68415	2,22E-38
BCAS3	4,68029	2,56E-38
KLK6	-11,4496	4,87E-38
ASPH	-4,40935	1,10E-37
PSMD6	3,616195	3,79E-37
UCP2	4,821463	9,50E-37
CKB	-3,86434	1,09E-36
NCOA3	3,78466	1,29E-36
PODXL2	4,333654	1,39E-36
TUSC3	6,17928	9,53E-36
ADORA2B	-5,23019	1,13E-35
NDRG1	-6,12467	2,84E-35
TFAP2C	4,486281	9,05E-35
MTAP	-6,5787	1,01E-34
TACSTD2	6,268508	1,31E-34
MCC	-4,30489	1,69E-34
HAS3	-5,53911	3,35E-34
SPRED1	-5,34821	5,66E-34
AKR1B1	-8,7481	5,81E-34
EPHA2	-5,43957	9,43E-34
PAK6	-5,95892	9,76E-34
CDKN2A	-7,7481	1,99E-33
RUNX3	-10,0062	3,46E-33
ADM	-7,65462	3,58E-33
ZDHHC2	-6,24466	6,45E-33
CAV2	-4,54411	7,63E-33
CDC42EP3	-4,2604	1,42E-32
PROM2	6,191901	2,08E-32
CLDND1	-3,53515	2,43E-32

ZBTB20	-7,52767	5,32E-32
GNAI1	-5,68202	1,18E-31
TRIM47	-4,29893	1,30E-31
FOSL1	-6,65976	1,70E-31
F3	-5,33092	2,22E-31
DPYSL3	-4,94325	6,53E-31
DCBLD2	-4,48482	1,06E-30
ANXA3	-4,68788	1,24E-30
ZNF217	3,508752	2,54E-30
BRIP1	3,341347	2,74E-30
ITGA6	-3,73236	2,86E-30
SELM	-4,05125	3,94E-30
M1AP	-7,66513	5,14E-30
ACAA2	3,495835	8,19E-30
GJC1	-9,54859	1,23E-29
IRX5	5,037391	1,31E-29
SH2B3	-3,7852	1,70E-29
WIPF3	-9,50611	3,64E-29
NXN	-3,35205	4,22E-29
IGSF9	3,729142	5,25E-29
NOTCH3	3,436623	5,97E-29
IGFBP5	7,008357	6,75E-29
LIX1L	-4,80686	9,19E-29
SIGMAR1	-12,2412	1,70E-28
DMKN	-3,34494	2,49E-28
CA2	-6,16229	2,66E-28
GREB1	3,683955	2,69E-28
CAV1	-5,09871	3,47E-28
SH3PXD2A	-4,75922	5,47E-28
SSFA2	-4,16056	5,47E-28
DCLK1	5,201957	6,13E-28
LINC01234	-7,1141	7,43E-28
MUC1	6,63167	7,45E-28
GULP1	-4,37101	7,66E-28
SRPX	-5,35103	7,66E-28
DNMBP	-3,74975	8,99E-28
LCP1	6,574197	9,02E-28
TRIM24	3,243025	9,46E-28
RLTPR	-3,82787	9,68E-28
KCNQ2	-6,04813	9,89E-28
CAPN2	-5,34617	1,13E-27
APPBP2	2,972044	1,13E-27
CD44	-3,29639	1,32E-27
LHX4	4,882162	1,37E-27
SIPA1	-3,8601	1,67E-27
ROR2	-7,35609	2,10E-27
APOBEC3C	-8,27721	5,23E-27
ZNF264	-4,48603	5,25E-27
RASEF	6,676843	6,68E-27
SPATC1L	5,778134	7,44E-27

FAM50B	6,034679	7,44E-27
BCL11B	4,528967	7,85E-27
SPDEF	10,11223	1,69E-26
SEMA3C	3,089919	1,91E-26
CUEDC1	-3,40695	2,49E-26
ZC3H12C	-5,57831	2,52E-26
C9orf64	8,309194	6,13E-26
HTR7	-6,25137	6,92E-26
FAT1	-3,74159	1,01E-25
PFDN4	3,240912	1,40E-25
APCDD1	16,04617	1,44E-25
RAB34	8,292254	1,53E-25
MEX3B	3,942088	1,62E-25
CLIP4	-8,4354	2,65E-25
PHF14	3,652511	2,73E-25
RBBP8NL	6,590617	3,06E-25
RTN4RL2	-5,34788	3,18E-25
NEB	6,694938	3,31E-25
TNFAIP2	-6,12292	3,51E-25
AXL	-9,80586	4,23E-25
FHL2	-6,28147	4,47E-25
FGF9	-8,33075	5,48E-25
HOXC10	3,740864	9,04E-25
NAGS	-6,16814	9,72E-25
TFF1	11,92	9,72E-25
MAPRE2	-3,92627	1,00E-24
DSE	-3,87408	2,44E-24
ACSS3	11,35095	3,06E-24
FRMD4A	6,238785	3,37E-24
IGFBP4	8,291469	3,40E-24
ECE1	-3,317	4,10E-24
SEPT10	-4,83452	4,82E-24
GLUL	2,64768	6,94E-24
HSD3B7	3,927364	7,12E-24
PARM1	-4,97611	8,53E-24
DHRS3	-4,21217	1,57E-23
ZNF595	4,431709	2,01E-23
TIAM1	-3,81902	2,08E-23
TNS4	-5,3542	2,15E-23
HUNK	7,685096	2,93E-23
TUBA1A	2,942995	3,26E-23
ELK3	-4,09242	3,30E-23
SLC1A2	7,876029	4,46E-23
LAMC2	-5,75916	5,84E-23
UBASH3B	-4,5867	7,76E-23
F2RL1	-4,0449	8,06E-23
FABP5	-2,71787	8,06E-23
CPOX	-4,8823	8,58E-23
ESR1	10,98328	8,64E-23
P3H3	-8,06862	1,05E-22

POF1B	7,50362	1,13E-22
TRPS1	15,07716	1,20E-22
ANKRD33B	-6,87811	1,51E-22
PMP22	-5,2859	1,75E-22
SOCS6	-3,09343	1,75E-22
AP1S3	-3,59409	1,88E-22
NANOS1	4,492214	2,57E-22
HOXB8	-9,05429	2,89E-22
RTBDN	-7,0152	3,09E-22
KYNU	8,170548	3,12E-22
TMEM98	-5,00143	3,40E-22
ABCA3	4,046015	3,45E-22
SLC17A9	-5,3538	3,45E-22
FREM2	14,91066	3,60E-22
COL6A1	-2,78864	3,60E-22
SLC25A13	3,248782	3,75E-22
ZMYM2	2,884059	4,14E-22
EVA1C	-4,59498	4,32E-22
COL6A2	-3,83169	5,09E-22
P2RY2	3,745829	5,09E-22
ZNF254	3,760248	5,27E-22
TBC1D9	3,201633	5,27E-22
B4GALT5	-3,09881	6,92E-22
S100A4	-4,05627	7,22E-22
IGFBP6	-5,69255	7,73E-22
FBLN1	-7,73845	8,66E-22
BCAR3	-3,54884	9,87E-22
CLDN3	3,728433	1,06E-21
EHD2	-2,95619	1,07E-21
NRCAM	6,497223	1,08E-21
DYNC1I1	9,098525	1,14E-21
SNAI1	-4,07438	1,37E-21
CYP1B1	5,478553	1,46E-21
FLNC	-14,6957	1,86E-21
GOLM1	-2,76429	1,87E-21
MXRA7	-3,50541	1,93E-21
AK7	4,346185	2,41E-21
TEAD2	3,349325	2,61E-21
RIMKLB	6,37893	3,24E-21
CIART	8,242678	3,28E-21
DLX3	9,325012	4,07E-21
GPAT2	-6,88297	4,07E-21
IGF1R	3,351238	4,26E-21
GPX1	-14,5526	4,47E-21
PVRL4	4,594132	4,62E-21
ICAM5	-4,84813	4,97E-21
BDNF	-5,77926	5,15E-21
ETV4	-6,17122	5,47E-21
GLI2	-7,89506	5,62E-21
LEF1	14,6186	6,30E-21

PCSK1N	8,174622	6,44E-21
PRSS12	-9,14011	7,68E-21
ZFHX3	2,851719	8,55E-21
QPRT	8,483407	9,64E-21
IDH2	3,267473	1,15E-20
COBLL1	3,628121	1,26E-20
CILP2	-4,19472	1,37E-20
SUMO3	2,88134	1,57E-20
MT1E	-14,508	1,95E-20
SPPL2A	2,769484	1,98E-20
GJB3	-5,86145	2,19E-20
KLK8	-8,43645	2,19E-20
GPC1	-3,0429	2,29E-20
TGFA	-3,89576	2,40E-20
MAOB	-4,62463	2,40E-20
SMIM22	7,192785	3,60E-20
COTL1	-2,75869	3,63E-20
IRX3	5,668276	3,86E-20
LDOC1	14,28976	4,02E-20
EHF	-3,75755	4,31E-20
GRHL3	5,886222	4,50E-20
STON2	3,350853	4,69E-20
TSPYL5	14,16023	4,86E-20
FOXC1	-2,81584	4,97E-20
TRIP10	-3,40648	7,18E-20
KCNH3	-3,6523	7,24E-20
GTSF1	-14,1016	7,62E-20
MBP	-3,79233	8,03E-20
EMC10	-14,0465	1,20E-19
CRIP2	3,145782	1,81E-19
PREX1	2,92929	1,82E-19
LIN7A	4,133451	1,94E-19
CGREF1	3,509714	2,11E-19
SCN5A	-4,41451	2,14E-19
KIAA1324	5,382971	2,14E-19
MEPCE	2,482219	2,58E-19
SLC16A3	-4,37455	2,93E-19
CHST6	-6,31134	3,08E-19
FBP1	13,27354	3,38E-19
SIRPA	-3,47082	3,88E-19
AMPD3	-4,48464	4,18E-19
LARGE	4,796048	4,60E-19
C15orf59	5,189806	4,91E-19
C14orf159	-4,06448	4,98E-19
ARAP3	-4,56496	5,85E-19
ZNF467	3,240069	6,98E-19
ZNF518B	-13,7419	9,48E-19
C3orf14	13,76133	9,85E-19
MAFB	5,233475	1,01E-18
ARHGAP23	-4,49515	1,20E-18

TMPRSS13	5,777784	1,29E-18
SOX4	3,200378	1,32E-18
USP32	3,509597	1,33E-18
SMARCA1	-3,9803	1,43E-18
OR51B5	-13,6653	1,50E-18
POPDC3	-6,14227	1,60E-18
STAT5B	3,413752	1,63E-18
KIFC3	-5,30279	1,69E-18
HSPB8	4,135698	1,90E-18
WLS	-3,48852	1,98E-18
NT5E	-13,7789	2,30E-18
PRKCA	-3,91116	2,34E-18
RAC2	-13,5908	2,45E-18
MRVI1	-7,88213	3,06E-18
PPP1R14C	-8,25426	3,26E-18
INHBB	4,700124	3,30E-18
DDN	-4,71762	3,36E-18
PLEK2	-3,46651	3,64E-18
RDH16	5,662736	3,67E-18
TINAGL1	-13,5917	4,30E-18
LIF	-4,78528	4,63E-18
EFEMP1	13,46721	4,80E-18
FXYD5	-3,15823	5,21E-18
IER3	-4,70574	5,21E-18
ST6GALNA(	-3,83227	6,41E-18
CRYBG3	-4,6326	6,84E-18
FAM46A	-3,77148	7,03E-18
CXorf57	-4,39055	8,01E-18
GRIN2D	-3,6416	8,38E-18
CLIP2	-2,69686	8,79E-18
PDE9A	-3,44449	1,02E-17
MST1R	-4,87635	1,16E-17
CIB2	-4,78597	1,31E-17
SLC27A3	3,195433	1,32E-17
MATK	13,28504	1,48E-17
HLA-A	-3,32619	1,58E-17
PRPS2	-2,7138	1,64E-17
TNFAIP8L3	5,278145	1,74E-17
TGM2	-5,53891	1,74E-17
HBE1	-13,3432	1,85E-17
ACSL4	-2,82367	1,91E-17
IGF2BP3	-13,2321	2,08E-17
DDAH2	2,922835	2,28E-17
TUBD1	3,132613	2,29E-17
CAMK2D	-3,50962	2,62E-17
SLC6A17	-9,98437	3,02E-17
SPTBN2	2,941214	3,67E-17
TRIM37	2,596188	3,86E-17
ITGA3	-3,32722	4,18E-17
CNN3	-12,9824	4,59E-17

SYTL2	3,153903	5,98E-17
PROCR	-5,39794	7,16E-17
KLK7	-13,2395	7,90E-17
NPR3	4,820671	7,90E-17
STARD10	3,472984	8,07E-17
TMEM30B	13,02118	8,31E-17
PLAU	-13,4944	9,57E-17
HOXB13	-5,69096	9,88E-17
NCOR1	2,344956	1,10E-16
DYRK1B	3,400556	1,14E-16
C1RL	-7,8612	1,21E-16
FRMD6	2,905162	1,31E-16
GFRA1	13,08007	1,32E-16
MSLN	-5,96806	1,38E-16
PLK3	-3,55629	1,39E-16
TUBB4A	-3,51218	1,58E-16
TNNI3	-4,16007	1,59E-16
HLA-B	-4,31868	1,59E-16
FAM13A	-4,15254	1,68E-16
KRBA1	7,23295	1,70E-16
CARD10	-3,1236	1,73E-16
LRRC8C	-3,77083	1,96E-16
PROM1	-12,8746	1,96E-16
C10orf54	-6,5773	2,06E-16
CBR1	12,91963	2,06E-16
SSH1	-2,5055	2,11E-16
GSTM3	13,22374	2,12E-16
ZNF260	12,85028	2,25E-16
ELFN1-AS1	-6,07198	2,44E-16
ABHD10	-2,62265	3,78E-16
ZCCHC11	-12,7687	3,79E-16
PRSS3	-12,835	3,94E-16
FAM171A1	-3,18801	4,06E-16
ARMC4	-5,64916	4,11E-16
CACNA1D	4,483648	4,15E-16
EVL	2,785415	4,24E-16
TRIM7	-4,32342	4,30E-16
SMAP1	3,248835	4,51E-16
PRR36	3,060541	4,54E-16
ACKR3	12,70119	4,82E-16
CADM1	9,344468	4,88E-16
TMEM200/	-12,846	4,96E-16
GEM	-6,26572	5,01E-16
C14orf169	3,279582	5,36E-16
KRT8	3,118281	5,40E-16
PRKAR1B	2,444534	5,62E-16
SEMA6B	-2,53476	6,18E-16
SELENBP1	12,69385	6,26E-16
VWA5B2	-6,33753	6,61E-16
DSC3	-9,41681	7,20E-16

CYP26B1	-5,45396	7,35E-16
MICB	-3,71402	7,53E-16
HSPA12A	-3,35999	7,81E-16
TYMP	-4,11505	9,11E-16
PKIB	3,610183	9,16E-16
TNFRSF10D	-13,1344	9,16E-16
PLA2G16	-4,02256	9,18E-16
ELFN2	-4,51182	9,28E-16
FSTL1	-5,29289	9,60E-16
NLRP2	-12,954	1,02E-15
CREB3L4	3,698574	1,09E-15
PTPRO	7,045422	1,09E-15
MYL9	-12,6756	1,19E-15
GRIP1	6,701999	1,24E-15
TMEM246	-12,5746	1,39E-15
PLAUR	-4,83844	1,43E-15
FAM127C	12,52595	1,44E-15
HMGA2	-12,3969	1,46E-15
THOC7	2,300273	1,55E-15
NFIB	2,656282	1,62E-15
HDGFRP3	12,67636	1,70E-15
RNF217	-8,12313	1,82E-15
TIMP2	-2,55626	1,90E-15
CHD6	2,530226	2,00E-15
DHTKD1	2,492196	2,03E-15
KCNJ8	12,47773	2,05E-15
PCDHA6	12,45601	2,16E-15
ZNF43	12,44169	2,18E-15
LPAR3	-12,4732	2,22E-15
NHP2	2,130014	2,22E-15
PRRX2	-5,40287	2,27E-15
EMP1	-9,43869	2,41E-15
ZNF469	-4,88129	2,44E-15
GPAT3	-3,96932	2,63E-15
VAV3	12,42828	2,64E-15
ADAMTS14	-6,96468	2,64E-15
XXYLT1	2,764025	2,93E-15
ERV3-1	3,507525	3,14E-15
TCEAL8	-12,4197	3,27E-15
KIAA1462	-4,18793	3,43E-15
ADGRB1	3,443055	3,47E-15
CPNE7	-2,57274	3,49E-15
FAM3C	-2,49585	3,62E-15
SNPH	-8,1019	3,80E-15
RAB3B	-3,55996	4,06E-15
MYEF2	12,48624	4,28E-15
BRSK2	-3,55084	4,45E-15
PRLR	12,34846	4,72E-15
NNT	12,32883	5,36E-15
ISOC2	2,510002	5,57E-15

PALLD	-2,91373	5,87E-15
ACO1	-3,17517	6,23E-15
SMO	-8,21074	6,33E-15
GFY	-7,89173	6,35E-15
WBP5	-12,2961	6,45E-15
SP5	3,074371	7,63E-15
MSH3	3,196722	7,77E-15
IRX2	12,23696	8,29E-15
FAM63A	4,384289	8,48E-15
KRT18	3,34224	8,55E-15
PRR15L	12,31181	1,01E-14
POLR3G	-2,75556	1,06E-14
CPA4	-8,69509	1,25E-14
RBP7	-12,1827	1,30E-14
KIRREL	-12,3786	1,32E-14
INPP1	-5,94156	1,62E-14
MCTP2	-6,30175	1,68E-14
TRAPPC6A	3,265039	1,76E-14
HHEX	-3,85866	1,79E-14
PVRL3	-12,1741	1,89E-14
NUCKS1	2,120856	1,98E-14
CSDE1	2,445982	2,05E-14
KIF1A	12,0737	2,14E-14
TRNP1	-3,31737	2,16E-14
FHL1	-4,02923	2,20E-14
MUC3A	-3,64263	2,35E-14
FERMT1	-2,70224	2,46E-14
TRIM72	-12,1187	2,50E-14
ULBP3	-4,59439	2,53E-14
ERBB3	2,369775	3,09E-14
MBNL3	2,648909	3,16E-14
MDFI	-12,1579	3,32E-14
C2CD2	-12,2496	3,33E-14
LRP2	7,50025	3,53E-14
GALNT18	-2,65325	3,59E-14
PIK3AP1	-5,1199	3,60E-14
TMEM200B	-6,68841	3,68E-14
TTYH2	-2,91889	3,77E-14
LRAT	-4,91374	3,98E-14
RMND5B	2,285385	4,07E-14
HOXA10-AS	-12,0262	4,21E-14
MSRB2	2,969371	4,25E-14
WDR83OS	2,227787	4,30E-14
DCDC2	7,239014	4,32E-14
ALG1L	6,186678	4,33E-14
CBS	11,92037	4,83E-14
HIPK1	2,201096	4,84E-14
NRSN2	5,364383	5,21E-14
SERTAD4	9,044891	5,43E-14
LRRK1	-3,67665	5,47E-14

SLC45A1	-8,06192	5,54E-14
SPG20	11,8994	5,56E-14
ERRFI1	-5,19042	6,02E-14
ZNF737	12,1459	6,16E-14
PHLDA2	-4,10805	6,26E-14
S100A6	-2,83843	6,29E-14
ADGRF1	-12,3252	6,29E-14
SOWAHC	-3,71083	6,36E-14
TP53TG1	11,94973	6,41E-14
DOC2B	-8,907	7,09E-14
HOXB6	-5,53873	7,09E-14
DOCK3	-5,41609	7,15E-14
SNX10	-3,17045	7,49E-14
FAM3B	7,665279	7,57E-14
TBX2	8,840533	8,02E-14
EMILIN2	-4,64078	8,30E-14
ALDH3B2	12,13349	8,72E-14
ZBED2	-12,0249	9,03E-14
BCAS2	2,28844	9,11E-14
RTTN	-3,32017	9,75E-14
KLHL29	-3,80573	9,78E-14
PROS1	-4,96408	1,01E-13
FAM135A	-3,13316	1,01E-13
ZNF93	11,78333	1,11E-13
BST2	6,310001	1,12E-13
CNIH2	4,979673	1,21E-13
PCDHA11	11,83027	1,21E-13
PCDHB2	11,81061	1,23E-13
CKMT1B	2,805494	1,24E-13
KLK10	-7,66732	1,24E-13
AUTS2	11,75618	1,24E-13
EFNB3	5,627112	1,27E-13
MUM1L1	-5,11802	1,33E-13
RPL39L	11,73601	1,37E-13
HS3ST1	-11,6202	1,39E-13
GDA	-11,8855	1,41E-13
PCDHB13	7,618172	1,44E-13
CCDC170	9,226247	1,46E-13
PSMG3-AS:	5,032644	1,49E-13
HAPLN3	-6,78316	1,55E-13
ZBTB42	2,723898	1,56E-13
LINC01315	-5,17136	1,56E-13
TARS2	2,609954	1,57E-13
FOXP1	3,161842	1,61E-13
RUNX2	2,895352	1,64E-13
ATXN7L3B	2,081571	1,66E-13
TRIB1	-2,34417	1,67E-13
DLX5	11,72409	1,67E-13
DUOX1	-7,77472	1,85E-13
ZFHX4	-11,9107	1,85E-13

LPAR1	-11,7622	1,85E-13
MALT1	-2,46409	1,87E-13
ERICH5	-7,21705	1,92E-13
HOXA10	-11,8476	1,94E-13
IMPA1	-2,53365	1,98E-13
PCSK5	-7,22714	2,06E-13
PCDHGC3	8,727161	2,10E-13
NREP	3,299718	2,15E-13
C16orf45	-3,81084	2,18E-13
ZNF140	11,67392	2,26E-13
TMEM59L	-5,18766	2,34E-13
IL4R	-3,29361	2,44E-13
PCDHB3	11,68853	2,44E-13
ARL4D	-3,24151	2,45E-13
NAPRT	-3,01841	2,50E-13
SORT1	2,529101	2,50E-13
OLFM1	3,487212	2,51E-13
ULBP2	-5,49527	2,53E-13
S1PR3	11,44113	3,06E-13
SOX8	-7,81824	3,18E-13
AP4B1-AS1	7,567884	3,19E-13
KLC3	-2,92264	3,38E-13
VSNL1	-9,67338	3,44E-13
TCEA3	2,704578	3,51E-13
HEATR6	2,259131	3,58E-13
ADCY1	11,60285	3,61E-13
SDHAF3	-11,7736	3,77E-13
C9orf152	11,69427	3,91E-13
ETS1	-12,1133	3,96E-13
PMAIP1	-3,3779	4,08E-13
ZNF280B	-11,6047	4,38E-13
CORO2A	-3,27404	4,43E-13
DUSP4	-4,98526	4,44E-13
ZFP69B	5,898499	4,49E-13
PYCARD	11,64076	4,50E-13
SPEG	-3,40417	4,55E-13
LTBP1	2,775054	4,61E-13
DGKA	-4,19874	4,70E-13
RPS4Y1	-11,5708	4,79E-13
KREMEN2	2,609274	4,80E-13
NFKB2	-3,33954	4,99E-13
ARHGAP4	-2,93435	5,00E-13
PLXDC2	11,54161	5,03E-13
MCM3AP-A	3,888322	5,54E-13
PCDHB16	11,60451	5,59E-13
CALD1	-3,94791	5,66E-13
ADAMTS19	10,88311	5,74E-13
HENMT1	11,47973	5,83E-13
PGM2L1	2,677797	6,02E-13
TMEM25	-11,5337	6,05E-13

NAALADL2	5,764304	6,29E-13
CD40	-11,6323	6,38E-13
YOD1	-5,00543	7,17E-13
BIRC3	-5,68434	7,39E-13
STK39	-2,92103	8,20E-13
CAPG	-2,27119	8,24E-13
ECHS1	2,128151	8,45E-13
ZNF320	-11,4746	8,45E-13
RAB38	-11,6169	8,53E-13
TRIML2	-5,67261	8,53E-13
TMEM45A	-4,89253	8,55E-13
ZNF608	3,385393	9,06E-13
ZNF829	11,48325	9,10E-13
IL18	-5,14539	9,35E-13
TMEM178E	6,981072	9,44E-13
C1orf56	5,448495	9,74E-13
ARTN	-3,43226	9,81E-13
ZNF253	10,76746	1,02E-12
STEAP1	-3,97064	1,09E-12
RIMS4	7,742497	1,18E-12
MDFIC	-7,60464	1,19E-12
HOXB4	-7,17326	1,21E-12
WNT5A	11,46682	1,23E-12
DTX3	11,34728	1,23E-12
PCDHB8	11,36375	1,23E-12
EFNB2	-3,01028	1,23E-12
MPP1	-5,06117	1,32E-12
CNTNAP1	-3,53213	1,34E-12
NUP210L	8,532645	1,36E-12
TMEM45B	11,32477	1,39E-12
PCDH18	11,84248	1,40E-12
BMP2	10,72257	1,41E-12
CPNE8	-11,4644	1,41E-12
CYB561	1,941339	1,43E-12
CPS1	-2,53059	1,48E-12
GGT6	11,07097	1,48E-12
SCG2	-11,3923	1,48E-12
ZMIZ1	2,366659	1,55E-12
SEMA4A	11,31692	1,56E-12
GLIPR2	-3,19006	1,57E-12
AR	11,3025	1,62E-12
GPNMB	5,079953	1,72E-12
DTNA	3,14926	1,72E-12
ZIC1	11,42183	1,76E-12
CHFR	11,29398	1,80E-12
SIAH2	2,838498	1,81E-12
ARHGAP22	-5,89348	1,85E-12
PPARG	-3,28104	1,86E-12
GUCY1A2	11,29109	1,87E-12
CAP2	2,908784	1,90E-12

ENDOD1	-2,22301	2,11E-12
C17orf51	-3,86885	2,16E-12
ZNF736	11,25414	2,28E-12
AFAP1L1	-8,50849	2,40E-12
NCAM2	11,36713	2,54E-12
GLUD2	4,370908	2,55E-12
NRXN2	3,941501	2,60E-12
ST3GAL2	-3,73564	2,62E-12
SALL2	11,21685	2,62E-12
FAM222A-/	-5,39949	2,93E-12
HOOK2	2,144808	2,95E-12
ICA1	2,7244	2,97E-12
TRIM15	-5,74125	3,00E-12
GRAMD1B	-11,527	3,05E-12
GNE	-11,3374	3,06E-12
KITLG	-2,74975	3,14E-12
GNPDA2	-3,07857	3,15E-12
DLG4	-3,73568	3,16E-12
SLC25A22	-2,32116	3,20E-12
ZNF836	11,22624	3,28E-12
PRNP	-2,92357	3,31E-12
ZNF569	11,27038	3,33E-12
EFHD1	4,834432	3,33E-12
ZNF429	11,17207	3,44E-12
CHN1	3,285688	3,46E-12
ST6GAL1	-3,88869	3,65E-12
CRISPLD1	11,40441	3,73E-12
EREG	-10,5891	3,80E-12
SLC1A1	-4,48019	3,83E-12
RHBDF1	2,586804	3,84E-12
ZNF681	11,13803	3,96E-12
YAP1	-2,18588	4,16E-12
TFAP2A	2,022446	4,20E-12
WNT16	-11,6391	4,20E-12
STAMBPL1	-4,69643	4,30E-12
OR51B4	-11,3375	4,30E-12
SLC10A4	-8,41543	4,30E-12
ZNF3	2,22264	4,52E-12
CABYR	7,77318	4,67E-12
TUBB2B	4,478789	4,69E-12
PPM1D	2,764958	4,78E-12
CACNG4	10,51638	4,80E-12
ELOVL2	5,984108	5,02E-12
PAK1	-2,73746	5,49E-12
FKBP11	3,672787	5,52E-12
KCNJ12	-11,2463	5,52E-12
PCP4	11,09588	5,69E-12
TSPAN18	-10,9152	5,97E-12
RAPGEF5	-3,47953	5,98E-12
ADAM9	-2,24415	6,36E-12

PTRF	-2,18849	6,42E-12
PGM1	-2,56053	6,49E-12
CHD3	2,884938	6,54E-12
DLC1	11,08037	6,64E-12
PTPRM	-11,1105	6,92E-12
CA13	-11,1089	7,16E-12
PDZD4	5,617185	7,23E-12
ASCL4	11,04836	7,36E-12
PKP1	5,01429	7,68E-12
C5orf38	11,10424	7,68E-12
HBEGF	-4,44605	8,11E-12
KDM5B	2,450383	8,22E-12
CNTNAP3B	-6,95317	8,35E-12
TCEAL1	11,08384	8,39E-12
DEF6	-2,35262	8,59E-12
TUBA4A	-4,03402	8,61E-12
OSBP2	-2,35646	8,70E-12
KCTD1	2,617638	8,70E-12
C5	5,168708	8,85E-12
HSD17B11	-3,34304	8,89E-12
TRIM33	2,014988	8,98E-12
MAP9	11,05136	9,23E-12
MIB2	-2,43786	9,29E-12
FAM3A	2,287626	9,29E-12
PLEKHA4	-4,1925	1,01E-11
SH3RF2	-4,49985	1,03E-11
CR2	-4,65141	1,09E-11
ARHGDIG	10,95255	1,16E-11
ABCB1	-5,4807	1,25E-11
NRAS	2,220258	1,25E-11
SYN2	-11,0478	1,28E-11
RNF115	2,030407	1,32E-11
OSMR	-2,97279	1,32E-11
RASSF3	-2,29011	1,33E-11
FGF2	-7,44999	1,34E-11
CFD	-2,59558	1,36E-11
CYTH2	2,043909	1,36E-11
ASCL2	11,11481	1,40E-11
FAM83F	-4,19593	1,40E-11
HOXB7	-2,41256	1,41E-11
TMCC3	-4,1588	1,45E-11
TDRD1	10,9041	1,50E-11
TUG1	2,067018	1,50E-11
CCDC3	-3,22255	1,51E-11
EFS	10,92228	1,52E-11
PTP4A1	-1,86041	1,54E-11
RNF44	2,115074	1,59E-11
ZP3	2,690277	1,61E-11
HMGA1	-2,37136	1,67E-11
EFR3B	3,541953	1,76E-11

CRMP1	10,91431	1,80E-11
ZNF718	10,87713	1,80E-11
ITGB1	-1,95023	1,81E-11
ZNF215	-11,175	1,82E-11
ATP6V0E2	2,550738	1,82E-11
PLEKHO1	3,115719	1,87E-11
IL27RA	-2,4254	1,93E-11
C16orf62	-2,53191	1,96E-11
ELL2	-3,07486	2,01E-11
FGFR2	-2,64953	2,16E-11
MEST	2,213713	2,20E-11
LDHA	-1,88176	2,23E-11
CNPY4	3,793862	2,29E-11
C2orf15	3,155564	2,32E-11
CORO2B	-5,44413	2,33E-11
ZKSCAN1	1,946109	2,37E-11
HOXC11	5,559583	2,46E-11
DOCK11	5,342834	2,51E-11
HIST2H2BE	3,957239	2,53E-11
TSPEAR	7,246436	2,56E-11
RPS6KB1	2,270086	2,57E-11
PAQR5	-2,66229	2,62E-11
SAMD5	-10,8657	2,83E-11
GUCY1A3	10,80245	2,90E-11
ZNF813	10,8221	3,14E-11
ZNF385B	-4,03924	3,21E-11
SPATA17	3,203401	3,33E-11
TDRD5	7,220216	3,44E-11
FLJ10038	2,784476	3,59E-11
CRAT	-3,59059	3,62E-11
KCNK15	10,76651	3,76E-11
DGKG	-8,21844	4,23E-11
MDK	1,878741	4,39E-11
RGL3	-5,19019	4,42E-11
C5AR2	8,267219	4,47E-11
TUSC1	10,70291	4,62E-11
SEPN1	-1,8717	4,67E-11
C1QTNF9B-	4,072267	4,89E-11
APH1A	1,867201	4,93E-11
KLK5	-11,3291	5,02E-11
ANXA9	3,99016	5,03E-11
TRIM9	-5,57122	5,26E-11
GTF2IRD2B	3,039445	5,26E-11
CST1	10,69885	5,35E-11
THNSL2	10,7595	5,54E-11
NOS3	-4,75212	5,67E-11
PLAGL1	-3,1507	5,85E-11
LINC00665	10,04574	5,96E-11
FOXA3	-10,7404	6,07E-11
ZNF793	10,66373	6,08E-11

FAM174B	10,26047	6,09E-11
ZFP3	10,03441	6,13E-11
GPC4	-2,41442	6,25E-11
CYB5R2	-10,7272	6,61E-11
NOD2	-4,74765	6,65E-11
STK33	-10,9046	6,75E-11
FRG1CP	-10,7184	6,76E-11
HCG11	10,65442	7,18E-11
GLI3	10,67472	7,20E-11
RRAS	-3,51107	7,29E-11
CYBRD1	-10,6994	7,73E-11
SKAP1	-10,6893	8,00E-11
TSPAN5	-2,99294	8,18E-11
DPP4	-6,17432	8,24E-11
MAP2K6	5,378467	8,31E-11
IFFO2	-2,62044	8,46E-11
CACNB3	2,138327	9,03E-11
NPY1R	9,999126	9,14E-11
GABRD	-3,9352	9,19E-11
RRBP1	2,351455	9,95E-11
MPDZ	10,6193	1,04E-10
TCEAL4	2,23975	1,04E-10
AMOT	2,432876	1,09E-10
FAM92A1	-3,48553	1,10E-10
LIMA1	-2,64162	1,19E-10
FGF12	10,61243	1,19E-10
ST8SIA4	10,89405	1,25E-10
CHD5	-3,96576	1,26E-10
ZNF331	10,76899	1,29E-10
ITPKA	-3,45364	1,34E-10
NFAT5	-2,40535	1,40E-10
SYK	2,381224	1,44E-10
RERG	5,562196	1,46E-10
CHRM1	5,629867	1,52E-10
HRH1	-10,8233	1,53E-10
FXYD3	4,177195	1,53E-10
FIS1	2,051355	1,53E-10
FAM109B	6,105245	1,54E-10
ENPP1	-4,1837	1,57E-10
CACNA2D1	4,149972	1,60E-10
PDE4A	-2,64381	1,62E-10
LINC00941	-10,9454	1,63E-10
MCOLN2	-4,04906	1,67E-10
HOXA9	-10,6713	1,68E-10
ONECUT3	-10,5921	1,69E-10
SEC14L4	-10,6095	1,72E-10
FZD1	2,297711	1,75E-10
TXNIP	13,33574	1,82E-10
RUSC2	-2,85179	1,84E-10
NETO2	2,188264	1,86E-10

PCDHB14	9,853789	1,87E-10
ZNF606	10,53965	1,96E-10
TFAP2E	-5,61389	2,02E-10
APBB1	-3,59205	2,04E-10
ZNF84	2,232072	2,09E-10
CEACAM21	7,922992	2,09E-10
PTGR1	-2,82058	2,12E-10
SPTB	-5,142	2,13E-10
PER1	-2,41182	2,17E-10
FAM131C	-3,13684	2,21E-10
PLXNA4	10,51262	2,27E-10
TBX18	-10,5103	2,30E-10
ANKMY2	-2,37594	2,36E-10
C7orf50	2,046165	2,38E-10
RORA	9,776096	2,44E-10
ABLIM3	-3,25261	2,65E-10
ZNF433	10,39398	2,70E-10
SGCE	-10,4788	2,72E-10
ANKH	-2,18062	2,74E-10
ZNF513	-4,36109	2,84E-10
PTPRG-AS1	5,598335	2,88E-10
SORBS3	-1,89944	2,95E-10
MIR100HG	-10,0955	3,07E-10
MYRIP	4,309292	3,08E-10
TCEAL3	9,781142	3,12E-10
CAMK2B	5,118112	3,21E-10
ZC4H2	-7,87346	3,30E-10
RNASEH2A	1,991426	3,35E-10
DLGAP3	5,835799	3,55E-10
SCCPDH	10,3586	3,56E-10
TOX2	-3,00156	3,58E-10
FAM110C	-4,12397	3,59E-10
FAHD2B	-3,22365	3,81E-10
ODAM	10,32209	3,92E-10
PLCG1	2,323092	3,93E-10
CDA	-10,5569	4,03E-10
TGIF2	2,426455	4,26E-10
ZMYND8	2,001815	4,29E-10
EXO5	3,85125	4,40E-10
PPP4R4	-5,22528	4,40E-10
SULT2B1	3,222378	4,51E-10
EMP2	2,394297	4,53E-10
ABCA7	-2,22935	4,53E-10
TIMP1	-3,9747	4,54E-10
C3orf52	-4,93347	4,54E-10
SDC2	9,662444	4,59E-10
FAM53B	2,224632	4,75E-10
CACNA2D2	-3,4203	4,96E-10
HOXB3	-7,1358	5,01E-10
AGMAT	10,3097	5,09E-10

ABHD6	-2,92235	5,14E-10
KMT2C	1,916468	5,15E-10
GAS6	-2,46423	5,16E-10
MB	4,820228	5,20E-10
KRT222	-10,5566	5,22E-10
LINC00857	-7,87	5,25E-10
CDC42BPA	-2,15169	5,34E-10
OAF	-2,6213	5,37E-10
SGSM1	-6,06804	5,46E-10
EFNA5	3,590871	5,46E-10
VWDE	-4,06291	5,46E-10
HEYL	-3,08809	5,50E-10
AHR	1,809939	5,63E-10
SEC14L2	-3,12474	5,76E-10
TMEM173	-6,05575	5,76E-10
XBP1	2,856248	5,85E-10
GSTM4	2,057406	5,99E-10
PCDH7	-2,52368	6,17E-10
ARNTL2	-2,6031	6,22E-10
RDX	-1,98493	6,32E-10
GRHL2	2,304149	6,34E-10
P3H1	-2,12499	6,48E-10
LETM2	-4,50214	6,82E-10
PBXIP1	4,062461	7,02E-10
C3orf18	-4,65949	7,09E-10
SLC35F3	-10,3119	7,16E-10
CASC4	-1,84239	7,20E-10
DSCAM-AS	12,86646	7,23E-10
CROT	10,47678	7,30E-10
SERTAD2	-2,09234	7,41E-10
CSAG3	-10,3762	7,74E-10
CDC42SE1	1,893085	7,76E-10
SFXN3	-2,52453	7,96E-10
TCN1	-10,3158	8,12E-10
AKR1C2	5,229524	8,12E-10
CXCL16	-2,01544	8,23E-10
PPDPF	2,06442	8,23E-10
SH3RF1	-2,70766	8,23E-10
SLC8A1	10,18213	8,38E-10
MESP1	-6,33205	8,44E-10
AIF1L	1,880397	8,44E-10
GDF15	-3,53707	8,73E-10
PGR	10,72354	8,94E-10
HTR1D	-3,93408	9,09E-10
ANKIB1	1,849523	9,09E-10
TRIOBP	-2,4532	9,26E-10
PCDHA10	10,19271	9,37E-10
RBMS2	-2,7487	9,46E-10
TWF2	-1,88005	9,71E-10
EDN1	4,679466	9,96E-10

ZNF420	10,18213	1,04E-09
MANSC1	9,598229	1,04E-09
SYNE1	-4,44583	1,05E-09
ENSA	1,939353	1,07E-09
BTG3	-3,01966	1,09E-09
ITPRIPL1	-9,92222	1,09E-09
WDR72	2,399273	1,09E-09
SNX9	-1,91127	1,10E-09
ZFPM2-AS1	-10,2552	1,11E-09
MEIS2	-5,90482	1,12E-09
NR1D1	-2,39084	1,18E-09
AP4E1	2,117218	1,18E-09
ITPRIPL2	-2,38329	1,18E-09
POLE4	2,293708	1,18E-09
ALOX15	9,914015	1,20E-09
TRIP6	1,784935	1,20E-09
LINC00839	-10,327	1,21E-09
FRAT2	3,705513	1,22E-09
CDIP1	-10,2092	1,27E-09
ZNF783	2,562534	1,30E-09
GALNT16	10,25475	1,32E-09
CCDC92	2,854919	1,34E-09
MFI2	-2,56038	1,36E-09
STARD13	2,646804	1,46E-09
HOXB5	-10,2536	1,46E-09
RIN3	-2,65944	1,48E-09
ZNF362	2,599538	1,55E-09
STXBP6	6,178001	1,55E-09
SPRED2	-1,80989	1,56E-09
TTC9	2,592793	1,58E-09
AARD	10,06503	1,62E-09
ZNF117	2,807851	1,62E-09
SH2D5	-3,40676	1,64E-09
EZR	-2,026	1,65E-09
WSCD1	10,08866	1,65E-09
RAD51C	1,814054	1,67E-09
NR4A2	-3,12812	1,68E-09
SHISA9	10,10757	1,73E-09
VTI1B	2,018681	1,78E-09
C7orf13	3,811607	1,79E-09
ZNF287	10,03735	1,86E-09
ZNF570	10,04642	1,86E-09
TSHZ3	10,4942	1,88E-09
P2RY6	8,627831	1,90E-09
ERBB4	6,180831	1,96E-09
GADD45G	4,633238	1,97E-09
HPDL	10,03728	1,98E-09
TDG	1,744736	1,99E-09
GUSBP1	5,011143	2,02E-09
ZNF256	10,02653	2,02E-09

VLDLR	10,4626	2,04E-09
NKAIN1	3,162889	2,05E-09
EHD3	10,10059	2,05E-09
ZNF688	3,280347	2,06E-09
INPP5F	-2,04873	2,09E-09
PPAP2C	-1,8989	2,17E-09
BLVRA	2,100931	2,20E-09
SASH1	-3,19627	2,20E-09
ASPHD2	-2,91914	2,22E-09
FOXRED2	2,011704	2,25E-09
ADGRL2	6,678928	2,29E-09
HNF1B	-10,1716	2,29E-09
PCDHA12	10,05006	2,30E-09
ERI1	-1,85543	2,33E-09
KHDRBS3	-5,43735	2,36E-09
DAB2	-5,11286	2,36E-09
TMEM180	-2,27574	2,37E-09
ZNF785	9,990072	2,39E-09
ITPKB	2,659777	2,44E-09
PLEKHF2	2,113913	2,44E-09
PRDM4	1,894095	2,51E-09
DEPDC7	-10,0856	2,56E-09
PLOD1	-2,09801	2,57E-09
C2orf68	2,25322	2,58E-09
PLCB3	-1,85191	2,58E-09
FAM71E1	-10,0851	2,58E-09
TMEM229I	3,881488	2,60E-09
MAP4K2	-2,18615	2,62E-09
PRDX2	1,791747	2,65E-09
GNAS	1,643304	2,69E-09
PCSK6	2,73556	2,72E-09
NSUN7	9,971846	2,75E-09
LINC00460	-10,1672	2,77E-09
GJB2	-2,93443	2,88E-09
MAZ	1,736031	2,88E-09
ETHE1	-2,57736	2,90E-09
MYLIP	2,813805	2,90E-09
SRPX2	-10,1111	2,93E-09
USH1C	-9,65535	2,97E-09
FAM179B	3,257734	3,05E-09
DEGS2	4,932816	3,08E-09
AP4M1	2,203673	3,11E-09
ZNF514	2,619152	3,11E-09
ZNF682	9,942601	3,17E-09
SLC12A4	-3,7301	3,30E-09
GRK5	-3,20683	3,31E-09
ZNF841	9,260573	3,32E-09
TLR6	-10,035	3,40E-09
PNMA1	2,13076	3,43E-09
A1BG-AS1	9,941424	3,45E-09

GOLGA8B	-4,00872	3,45E-09
GNAL	-3,22038	3,54E-09
DCLRE1B	2,140602	3,60E-09
PLA2G7	-10,037	3,63E-09
PAQR3	-2,13648	3,74E-09
MYOM3	-10,2031	3,79E-09
TYSND1	2,027593	3,84E-09
ALDH6A1	2,261391	3,95E-09
LYPD1	6,716244	3,97E-09
TFF3	9,93152	4,03E-09
DDX60L	-2,98527	4,18E-09
MAN1A1	-2,27309	4,18E-09
ITM2C	-2,38583	4,30E-09
SETMAR	2,228544	4,30E-09
SOX2	9,224737	4,34E-09
TDRKH	2,947813	4,45E-09
C22orf34	-10,0852	4,45E-09
C18orf63	9,924395	4,45E-09
STXBP1	-1,97841	4,47E-09
RASL11B	9,221306	4,54E-09
RAB11FIP5	-3,00058	4,61E-09
NCKAP5	5,526153	4,71E-09
GRAMD2	-10,0555	4,84E-09
TNFSF9	-3,81411	4,87E-09
GALC	9,956345	4,98E-09
CPD	-1,94309	5,00E-09
HSD17B12	-1,75029	5,04E-09
NMNAT2	9,886784	5,15E-09
FAM214A	3,940034	5,19E-09
SHROOM2	-2,44359	5,19E-09
KNOP1	1,887842	5,33E-09
CHPT1	2,382204	5,33E-09
LONRF3	-7,59073	5,50E-09
SLCO1B3	-10,0468	5,54E-09
ETV1	-9,51568	5,56E-09
SMIM3	-2,26626	5,60E-09
LINC00925	9,845905	5,74E-09
TMEM56	-2,72315	6,03E-09
ZNF365	-5,02981	6,39E-09
PDCD4	2,765784	6,49E-09
SSTR5	-9,92169	6,49E-09
TCF7	-2,19724	6,68E-09
TMEM151/	-3,93421	6,69E-09
LZTS1	-9,9183	6,69E-09
EMC3	2,251829	6,78E-09
TESC	-2,89551	6,81E-09
SPTSSB	3,290964	7,01E-09
HNF4A	-4,79706	7,01E-09
B3GNT5	-4,25692	7,05E-09
ALAS1	-1,91799	7,15E-09

LAMA2	-4,1632	7,36E-09
SLC24A3	9,779913	7,64E-09
PLAT	-9,9387	7,73E-09
MCAM	-2,98274	7,95E-09
ENTPD1	4,848459	8,01E-09
ZYG11A	9,930993	8,15E-09
RABIF	2,390583	8,20E-09
GAREML	-2,43865	8,28E-09
ADAP2	-3,04532	8,39E-09
PPP1R13B	2,050257	8,46E-09
EHD4	-1,92183	8,49E-09
GCDH	1,913674	8,54E-09
ZBED9	7,440357	8,65E-09
KLHL2	-2,18635	8,70E-09
WDR90	-2,80533	8,86E-09
SHISA8	-4,01094	8,89E-09
TLE3	1,633884	9,08E-09
HPN	9,114277	9,21E-09
CGN	2,08862	9,23E-09
ZNF790-AS	9,749057	9,40E-09
ZNF747	2,644983	9,43E-09
ACOX2	-6,94886	9,86E-09
FKBP10	1,781273	9,91E-09
ARHGEF10	-2,48991	9,98E-09
SLC6A8	-2,47343	9,99E-09
CPVL	-4,14445	1,02E-08
RDH10	-2,79265	1,02E-08
FUT8	1,975949	1,02E-08
CCAT1	-9,97463	1,03E-08
PAM	-2,06329	1,03E-08
PLEKHA2	-2,55742	1,06E-08
NGFR	-3,63126	1,07E-08
ZNF503	-2,34379	1,09E-08
TLX3	-9,82887	1,10E-08
TRPM7	2,312427	1,10E-08
SKIDA1	4,989646	1,13E-08
TTC30B	4,551786	1,14E-08
GSTA4	4,104191	1,14E-08
C2orf70	-9,8477	1,18E-08
PARD3B	4,348136	1,18E-08
EXOC4	1,892986	1,18E-08
C21orf33	2,00481	1,19E-08
AHI1	-2,19541	1,19E-08
ACOT11	-3,73568	1,21E-08
SNX7	-3,19604	1,23E-08
AP4B1	2,163967	1,24E-08
TNFSF12	-9,80701	1,25E-08
C1orf233	2,740595	1,27E-08
FAM89A	-3,36446	1,27E-08
SLAIN1	9,752107	1,28E-08

DGKD	-2,05299	1,29E-08
ZNF732	9,775242	1,31E-08
C12orf56	9,686908	1,32E-08
HOXA7	-4,53382	1,33E-08
CENPB	1,82637	1,35E-08
EPHA6	9,674417	1,36E-08
SERINC2	-2,6526	1,44E-08
RASGRP2	-4,37511	1,44E-08
RABGGTB	-1,76775	1,44E-08
MAGEH1	9,717176	1,45E-08
CAMK4	-4,09892	1,48E-08
SPESP1	9,043149	1,51E-08
RNF157	4,518528	1,51E-08
TPBG	1,78918	1,52E-08
ARL14EPL	-10,0916	1,56E-08
PHAX	1,667411	1,56E-08
SYT12	3,200889	1,57E-08
SLC26A4-A:	9,647384	1,58E-08
BATF	9,749078	1,58E-08
MAFG	-1,8236	1,58E-08
CD274	-6,77598	1,59E-08
LXN	10,17949	1,62E-08
C6orf223	-5,35127	1,62E-08
JPH3	6,439798	1,63E-08
RGS10	-1,81359	1,64E-08
ZNF486	7,302662	1,64E-08
EFNA4	1,984843	1,66E-08
ASCL1	9,739251	1,68E-08
CYP2J2	3,341183	1,72E-08
CXCL12	9,645271	1,72E-08
ANKRD18B	9,650536	1,72E-08
ADGRE5	-2,3827	1,75E-08
DDX3Y	-9,79609	1,75E-08
SEL1L3	-1,89135	1,75E-08
TRMT2B	2,140339	1,75E-08
TRHDE	-9,93298	1,92E-08
CHST10	9,101843	1,93E-08
NMU	-2,25717	1,97E-08
DCBLD1	-2,38764	1,98E-08
ASRGL1	-2,30575	1,99E-08
FUT4	-2,87907	2,01E-08
GAS7	-7,47853	2,05E-08
NOTUM	-2,68759	2,10E-08
ZNF559	-9,70794	2,20E-08
LINC01405	-9,7163	2,22E-08
DECR2	2,091113	2,27E-08
RNF141	-2,35303	2,27E-08
ANKRD19P	-9,79436	2,27E-08
SLC30A3	-2,61664	2,33E-08
NEDD4L	-2,19001	2,34E-08

MYO5C	1,87486	2,37E-08
TSPAN13	2,046396	2,38E-08
CHST8	9,555086	2,55E-08
LINC00052	9,925515	2,59E-08
ZNF687	1,874562	2,61E-08
ZNF175	-9,6982	2,66E-08
CECR2	-3,91673	2,72E-08
PLCL2	-5,91234	2,77E-08
ELOVL3	-6,58948	2,77E-08
ZFP62	1,753824	2,77E-08
SLC2A1	-2,14455	2,81E-08
HPCAL4	-9,29894	2,85E-08
FAM198B	3,148541	2,87E-08
IFNGR2	-2,11514	2,89E-08
PEAR1	-9,51512	2,89E-08
HEXA	1,686515	2,89E-08
PNRC1	2,131793	2,99E-08
DAPK1	-4,99087	3,06E-08
MAMDC2	-9,68266	3,11E-08
TMED3	1,793289	3,13E-08
LOC28333!	2,792935	3,14E-08
LIPE	-7,37626	3,17E-08
MLH1	2,778959	3,18E-08
RAB15	2,729477	3,23E-08
SDK1	7,223527	3,23E-08
EPN3	2,67355	3,33E-08
BCAR1	2,253433	3,34E-08
CHGA	8,803906	3,39E-08
ME2	-1,73458	3,39E-08
MSN	-9,63986	3,44E-08
C9orf3	2,228483	3,44E-08
ADI1	2,141894	3,48E-08
TNFRSF21	-1,61184	3,57E-08
ADAP1	-2,17598	3,60E-08
KSR1	-2,74078	3,72E-08
SAYSD1	1,864175	3,77E-08
HKR1	2,24124	3,83E-08
RGL2	1,935081	3,90E-08
ZNF529	9,477409	3,91E-08
CRACR2B	2,833831	4,07E-08
CACNB4	-4,77269	4,08E-08
CLN6	1,727275	4,10E-08
DAAM1	2,301614	4,12E-08
ZNF738	2,345098	4,12E-08
HKDC1	-7,31257	4,29E-08
SYT13	-4,87105	4,30E-08
ACSS1	9,525366	4,31E-08
DFNA5	-9,69195	4,32E-08
PLK2	-2,45119	4,34E-08
EDNRA	-9,59826	4,40E-08

NOVA1	9,512227	4,44E-08
TBCK	1,936815	4,49E-08
LRRC75B	3,103118	4,51E-08
PTPRH	-4,58919	4,67E-08
ACOX3	2,268667	4,67E-08
CLCC1	2,014618	4,68E-08
ECHDC1	-1,96399	4,83E-08
RAB27B	2,482879	4,90E-08
ERVMER34	-2,52862	4,91E-08
ATP9A	2,069349	5,05E-08
DDX10	-1,69015	5,08E-08
CBX2	2,24423	5,21E-08
TGFB1	-1,88461	5,22E-08
TMX3	-1,95153	5,29E-08
METTL9	1,708945	5,31E-08
ZNF605	9,566085	5,42E-08
ZNF85	3,03658	5,43E-08
NPM2	-9,5451	5,45E-08
LINC01296	3,074238	5,49E-08
ZNF480	1,960362	5,49E-08
ZFP69	9,446151	5,66E-08
RAB37	-9,63633	5,70E-08
ADGRG5	-9,54377	5,71E-08
AGRN	2,124906	5,71E-08
SLC9A5	-3,90886	5,75E-08
CDYL2	8,780957	5,75E-08
SCARB2	1,717776	5,75E-08
A1BG	5,288871	5,77E-08
BEND5	9,441253	5,82E-08
BACH1	-2,12541	5,98E-08
MALRD1	9,524656	6,03E-08
CBLB	2,239312	6,03E-08
DNER	-6,62186	6,14E-08
HOOK3	-1,98628	6,18E-08
ARMCX6	2,024245	6,20E-08
ZMYND11	1,946272	6,22E-08
PRSS8	1,913119	6,34E-08
RFTN1	-3,27317	6,37E-08
MRPS25	1,743917	6,52E-08
SH2D4A	-1,873	6,74E-08
KIAA1549	2,032946	6,80E-08
DISP2	-7,25807	6,93E-08
AMN1	-2,5042	7,03E-08
ANXA2	-1,48404	7,13E-08
EFHD2	-1,83665	7,16E-08
EN2	3,745561	7,22E-08
RBPMS2	-2,34354	7,36E-08
FAM195A	1,754915	7,41E-08
CTSF	9,406006	7,42E-08
NUDT16P1	-9,50787	7,50E-08

PCDHB6	9,391271	7,52E-08
LUCAT1	-5,503	7,52E-08
SDC4	-2,95833	7,55E-08
CCT8	-1,46849	7,56E-08
ASS1	2,957946	7,56E-08
TTC28	4,016185	7,61E-08
CADPS2	-4,51254	7,61E-08
FAM124A	-4,60249	7,73E-08
LIN7C	-1,97813	7,89E-08
TMEM183A	1,653863	7,90E-08
ZNF347	8,699581	8,01E-08
HECW1	-9,4763	8,03E-08
TUBA3E	9,425213	8,09E-08
ITFG3	2,099152	8,11E-08
KCNQ1	-3,79296	8,16E-08
OSBPL5	-1,97249	8,18E-08
WWOX	2,059285	8,22E-08
SLC22A23	1,63803	8,26E-08
RAB30	2,281207	8,33E-08
CECR7	-7,24748	8,33E-08
GSTZ1	2,141121	8,39E-08
YIF1A	1,762522	8,45E-08
TBX2-AS1	7,109945	8,56E-08
ATP8B3	-4,81593	8,63E-08
CBFB	-1,60534	8,76E-08
ERC2	9,323958	8,76E-08
PITX2	-3,25187	8,78E-08
RAB31	-2,51749	8,93E-08
ZNF311	9,542774	8,94E-08
NF1	1,965234	9,03E-08
PRICKLE2	10,51538	9,06E-08
MEIS3P1	8,661691	9,07E-08
JAK1	-1,7092	9,13E-08
CHRM3	9,315749	9,25E-08
SLC25A24	1,681187	9,38E-08
CCL2	9,538318	9,62E-08
ARPP19	1,538054	9,63E-08
ZNF790	9,34993	9,73E-08
HLA-C	-3,12473	9,73E-08
ZNF710	1,873154	9,76E-08
PTPRE	-3,65169	9,79E-08
XPNPEP1	-1,77098	9,86E-08
NYAP1	-4,17569	9,86E-08
GLT8D2	-9,50065	1,01E-07
PIK3CD	-3,41553	1,01E-07
SH3BP5	1,714315	1,04E-07
NLRC5	-4,13293	1,05E-07
OR51B2	-9,4242	1,06E-07
C19orf43	1,456915	1,06E-07
SIDT1	8,610801	1,07E-07

TEX15	9,284698	1,08E-07
CASP6	1,926381	1,09E-07
TNFSF13	5,758712	1,09E-07
TNFRSF10B	-2,61686	1,10E-07
CKMT1A	2,355711	1,10E-07
ZNF649	-9,47761	1,10E-07
PAX6	-9,45952	1,11E-07
UNC5A	-2,71415	1,11E-07
NYNRIN	6,260787	1,11E-07
SLC39A14	-2,34903	1,12E-07
TMC8	-4,19618	1,12E-07
ALDH5A1	2,111923	1,13E-07
LINC01004	3,927868	1,15E-07
GTF3A	1,733543	1,15E-07
PSD4	2,149147	1,16E-07
APRT	1,665382	1,17E-07
PCDHB9	8,566209	1,18E-07
GRIN2B	-9,73315	1,19E-07
PLCH1	1,835688	1,19E-07
SLITRK4	9,311774	1,19E-07
FRAS1	-1,84247	1,21E-07
SAMD3	-9,39986	1,22E-07
HSPB1	1,741955	1,24E-07
RUNX1	-1,89485	1,27E-07
HLA-DRA	-9,56206	1,28E-07
FOXO3	-2,12285	1,32E-07
ZNF630	9,243833	1,34E-07
LINC01116	9,235918	1,41E-07
SLC17A5	-2,48405	1,41E-07
CYP1A1	6,361402	1,42E-07
RBM8A	1,552107	1,42E-07
DARS2	1,668677	1,42E-07
ADAT1	1,69774	1,45E-07
ZNF568	9,43699	1,47E-07
PCDHGB5	9,2236	1,49E-07
RMI2	2,188479	1,50E-07
RNLS	8,523526	1,51E-07
C5orf42	-9,36039	1,51E-07
AASS	-9,38713	1,51E-07
ITPRIP	-3,85989	1,52E-07
WDR17	9,217789	1,55E-07
ADAMTSL5	-2,47271	1,56E-07
ARHGEF4	-2,3756	1,56E-07
USP8	1,491606	1,56E-07
SLC35E4	-2,66164	1,58E-07
SLC38A2	-2,00339	1,61E-07
LINC01128	-2,39993	1,61E-07
PACSIN3	1,569698	1,65E-07
MKRN3	-9,40991	1,65E-07
FUT9	9,203601	1,65E-07

AKAP9	1,927959	1,66E-07
LYSMD1	2,272687	1,66E-07
ACOT4	9,232998	1,74E-07
FAM19A5	9,258364	1,75E-07
IRF5	-2,56725	1,79E-07
CCDC113	-2,10629	1,80E-07
NRG1	-9,68195	1,81E-07
NNT-AS1	8,650487	1,81E-07
AGPAT4	-3,49739	1,82E-07
LEO1	1,827221	1,82E-07
ZNF432	3,683542	1,82E-07
PCDHGB1	8,484323	1,84E-07
NFIL3	-3,63209	1,86E-07
PRKCQ-AS1	-9,32186	1,87E-07
SYNPO2	3,389678	1,90E-07
RTN4RL1	2,50351	1,91E-07
TBC1D8B	-3,11373	1,92E-07
GABPB1	2,100995	1,92E-07
SULT4A1	-9,33294	1,94E-07
TIPARP	-1,77041	1,94E-07
MORC4	-1,63007	1,96E-07
SCARA3	-2,0367	1,97E-07
BMP4	-3,07711	1,99E-07
NUDT4	1,822538	2,01E-07
TMPRSS2	9,233351	2,07E-07
COL9A3	-2,26361	2,08E-07
DUSP7	-2,48783	2,10E-07
SMAD3	-2,14474	2,12E-07
MET	-1,85896	2,14E-07
EML1	1,977893	2,14E-07
DOCK8	5,693179	2,14E-07
GPR27	9,182907	2,15E-07
GNB2	1,509207	2,18E-07
PLBD1	9,217509	2,19E-07
DDX43	9,189127	2,19E-07
FHAD1	-4,95665	2,19E-07
MATN2	2,619744	2,22E-07
TK2	-2,35708	2,24E-07
SLC35G2	-9,55876	2,24E-07
SEMA4C	1,883226	2,25E-07
USB1	-2,06396	2,25E-07
PKIA	-9,37491	2,26E-07
KHK	1,879917	2,31E-07
ZNF492	9,368543	2,32E-07
ZNF91	1,97455	2,32E-07
TMEM37	3,602128	2,32E-07
GFPT2	-2,44304	2,34E-07
SNX14	-1,59618	2,34E-07
IL12A	-5,85471	2,41E-07
ZNF219	1,927092	2,42E-07

SPINK5	9,359182	2,47E-07
CD99L2	2,684981	2,47E-07
MMAB	2,536147	2,53E-07
PFKP	-1,70791	2,54E-07
RAET1E	-9,28224	2,56E-07
PDE4DIP	2,23327	2,58E-07
STK4	-1,53851	2,61E-07
SSX2IP	-1,80484	2,62E-07
SGK223	-1,90474	2,65E-07
KRT81	4,7064	2,66E-07
RNASEH2C	2,146267	2,71E-07
BHLHE41	-9,04123	2,72E-07
COX6C	1,541953	2,75E-07
HMGB3	1,880214	2,76E-07
LOXL2	-3,6978	2,76E-07
TENM4	9,192565	2,76E-07
KCTD20	1,556077	2,77E-07
MFAP2	-1,86344	2,77E-07
LAMC3	-2,68393	2,77E-07
SLC16A7	-4,05091	2,80E-07
DLGAP1-AS	-4,10966	2,83E-07
GLIS3	-5,93284	2,85E-07
NRXN3	8,609661	2,86E-07
HBP1	3,046594	2,87E-07
L3MBTL3	-2,78943	2,87E-07
TUBB2A	-2,70548	2,90E-07
PCDHA7	9,096418	2,91E-07
EXTL2	-2,23395	2,93E-07
SLC35F5	2,028123	2,93E-07
TRIB2	-2,75099	2,94E-07
SRRM3	-2,54403	2,94E-07
ARHGAP18	-2,03035	2,95E-07
MAPKBP1	-2,2041	2,96E-07
NME4	1,488699	2,99E-07
POU3F3	9,199277	3,02E-07
ZG16B	9,390358	3,04E-07
TGFBR2	-2,05029	3,04E-07
SRMS	-5,09989	3,05E-07
TUBA3D	9,182269	3,05E-07
CD68	-3,25765	3,05E-07
SLC35E3	2,063095	3,06E-07
SUN3	-9,2672	3,07E-07
PRKCDBP	-9,37374	3,08E-07
PTPRU	-1,6887	3,08E-07
RBM4	1,634383	3,09E-07
PEG10	-3,96843	3,10E-07
GABRQ	-5,94736	3,13E-07
CSNK2A1	-1,45333	3,13E-07
TNFRSF9	-9,91175	3,16E-07
ICK	2,152458	3,16E-07

TYRP1	6,962508	3,16E-07
PPIC	1,741756	3,20E-07
ZNF549	9,136305	3,20E-07
RPSAP58	1,583874	3,22E-07
PIK3CD-AS:	-4,1005	3,26E-07
NPAS2	-2,1476	3,27E-07
CRLF3	-2,23457	3,27E-07
SQRDL	-2,67638	3,28E-07
PHLPP1	1,865059	3,28E-07
SERAC1	-2,29861	3,31E-07
PRUNE	1,774061	3,34E-07
LMNB1	1,604023	3,42E-07
RAB11FIP1	-1,55723	3,42E-07
RPRD2	1,513213	3,42E-07
FUT11	-2,25647	3,46E-07
ZNF444	1,713764	3,47E-07
PFKFB4	-2,55463	3,52E-07
AP5S1	1,873426	3,59E-07
HPCAL1	-2,10337	3,62E-07
MMP16	9,108885	3,62E-07
FUT2	-9,19932	3,63E-07
HTT	1,841837	3,65E-07
ZNF697	-2,57515	3,72E-07
ZNF879	9,052249	3,72E-07
ARHGAP31	-8,65447	3,72E-07
ZNRF3	2,114478	3,73E-07
HIGD2A	1,469376	3,83E-07
AIMP2	1,77489	3,85E-07
DSP	1,499621	3,85E-07
CAB39	-1,51179	4,04E-07
ZNF354C	9,049931	4,08E-07
ZNF730	9,04158	4,11E-07
PLS1	-1,736	4,12E-07
MUC5B	4,025338	4,27E-07
SMCO4	-3,09386	4,28E-07
SERPINB5	-3,91067	4,33E-07
SLC4A11	-2,65568	4,33E-07
ATOH8	3,377525	4,35E-07
SLC8B1	-2,04993	4,41E-07
MYL12A	1,61744	4,43E-07
CBLC	-2,51862	4,52E-07
BCL2L2	-1,85565	4,53E-07
GLS2	8,285341	4,64E-07
IAH1	1,907766	4,64E-07
ZNF461	5,754477	4,67E-07
CUX1	1,558002	4,68E-07
ERO1B	-4,99896	4,69E-07
MGMT	2,00252	4,72E-07
DNMT3A	2,081398	4,75E-07
YRDC	-1,7619	4,88E-07

VASH1	-2,72128	4,89E-07
ZDHHC24	2,240921	4,89E-07
FAM110B	8,317516	4,91E-07
INPP5J	-2,64171	5,00E-07
ZSCAN2	1,885709	5,02E-07
CCDC69	-3,25152	5,02E-07
SLC27A6	-3,72359	5,07E-07
ZFP30	2,265902	5,16E-07
LHX5	-9,14195	5,18E-07
LINC00623	-2,81154	5,19E-07
SGMS2	-2,44311	5,19E-07
CD22	-5,89978	5,26E-07
TEAD1	-1,44303	5,31E-07
LAT2	-5,84704	5,41E-07
THNSL1	1,712902	5,42E-07
RABEP2	2,02285	5,43E-07
FAM49B	-1,44666	5,47E-07
NTSR1	-8,97437	5,54E-07
RNASEL	2,318317	5,59E-07
RBM20	-3,32865	5,61E-07
NPL	4,287969	5,67E-07
GPR89A	2,147053	5,85E-07
CHST3	-3,31941	5,98E-07
MECOM	2,553037	6,08E-07
KLHL25	2,073694	6,11E-07
ZNF141	9,005475	6,21E-07
MB21D2	2,36369	6,22E-07
PYGL	-6,4857	6,28E-07
CD99P1	-3,74026	6,41E-07
ANK3	1,959438	6,41E-07
CHRNB1	-2,59371	6,42E-07
NGFRAP1	1,453684	6,55E-07
RGS2	-3,12599	6,64E-07
RAB39B	9,654959	6,70E-07
SLC4A3	-2,26972	6,71E-07
CCDC88A	-1,90661	6,72E-07
RGS20	-9,08341	6,79E-07
C14orf132	3,094059	6,83E-07
VPS37B	-1,86402	6,86E-07
DR1	-1,56417	6,96E-07
GLS	-1,68773	6,96E-07
SUV420H2	2,084258	6,97E-07
INPPL1	1,506216	7,03E-07
MYH15	-7,89818	7,04E-07
ABHD11	1,725303	7,12E-07
GNB4	8,17655	7,12E-07
KIF20A	1,666874	7,13E-07
ACTL10	8,925595	7,16E-07
ATP11A	-1,79009	7,45E-07
CASP10	-4,41288	7,46E-07

PIGM	1,957669	7,48E-07
ADGRE2	-2,49843	7,51E-07
JTB	1,503959	7,52E-07
IDNK	3,918461	7,54E-07
TMEM9	1,507252	7,55E-07
CCDC88B	-2,94288	7,70E-07
POLRMT	-1,71522	7,72E-07
SH3BP4	-1,76953	7,81E-07
MAPK6	1,409954	7,82E-07
FAR2	1,999445	7,87E-07
NFATC1	-2,00133	7,87E-07
PSTPIP2	-1,68456	7,92E-07
CCZ1B	1,68709	8,01E-07
RFX2	-2,21855	8,14E-07
CLDN2	-9,11267	8,19E-07
CNGB3	8,93504	8,24E-07
HES6	-2,4531	8,39E-07
ST6GALNA(	-9,08392	8,44E-07
KIAA1551	-1,49516	8,53E-07
TMEM123	-1,38663	8,57E-07
KLRG2	5,609701	8,62E-07
CCNYL2	-9,10518	8,68E-07
SLCO4A1	-2,1404	8,71E-07
SRXN1	-2,53297	8,80E-07
PRF1	-9,04839	8,87E-07
NR3C1	1,752949	8,90E-07
TET2	2,049921	8,96E-07
C2orf74	8,874604	9,26E-07
PAQR4	1,694541	9,28E-07
SGTB	-2,91078	9,30E-07
SLC1A3	-2,77957	9,52E-07
WIBG	1,751354	9,75E-07
CDKN1C	-6,26863	9,92E-07
PNPLA5	-9,16009	9,93E-07
KCNJ3	5,390524	9,95E-07
PACS2	1,456672	9,96E-07
OLFML2A	-3,23621	1,00E-06
ZNF876P	8,857573	1,00E-06
PRTFDC1	5,374482	1,01E-06
CSF1	-3,17485	1,01E-06
ZC3HAV1L	-9,18038	1,04E-06
HCP5	-5,36149	1,08E-06
BOLA1	1,863883	1,08E-06
ZNF502	-9,03816	1,09E-06
UPK2	7,119217	1,11E-06
RIC3	8,917654	1,11E-06
RND2	2,943894	1,11E-06
LOC100507	-9,04248	1,11E-06
ATP5S	2,666272	1,11E-06
IPO5P1	3,060541	1,11E-06

GNL3	-1,75638	1,12E-06
C10orf82	4,431474	1,12E-06
ECSIT	1,565681	1,12E-06
RPF1	-1,60503	1,14E-06
TUB	8,829049	1,16E-06
ANO5	-8,98757	1,18E-06
PTPRG	-1,91447	1,18E-06
SLC35A4	1,653571	1,18E-06
B3GNT7	-5,34439	1,19E-06
PLEKHA7	2,541842	1,19E-06
ARMC10	1,535503	1,21E-06
NKX1-2	-5,64095	1,21E-06
COL27A1	-2,93941	1,22E-06
ZNF503-AS	-8,97938	1,22E-06
PSMC2	1,431146	1,23E-06
ZNF675	2,042465	1,25E-06
HIC1	6,077444	1,27E-06
HCN2	2,464451	1,27E-06
GCNT3	-8,97114	1,27E-06
PTPRN2	-5,36201	1,28E-06
PDE2A	-3,65184	1,29E-06
TNFAIP3	-3,46244	1,30E-06
LOC100129	2,806201	1,33E-06
ANO6	-1,65875	1,33E-06
ARSJ	-6,36641	1,35E-06
RAVER2	-1,99409	1,38E-06
EYA2	8,991706	1,38E-06
LGALS3	-2,24452	1,39E-06
ST6GALNAC	-1,80035	1,39E-06
C17orf104	9,025804	1,39E-06
ITGA2	-2,07468	1,39E-06
PRDM6	5,140298	1,42E-06
ATF6B	1,556878	1,42E-06
CACFD1	2,045288	1,42E-06
ABCG1	2,662456	1,42E-06
ZNF818P	5,003212	1,43E-06
ZNF618	1,802342	1,45E-06
TNIK	-1,99769	1,45E-06
PBLD	3,14164	1,47E-06
NRG3	8,846041	1,51E-06
HK2	-1,96455	1,51E-06
BRPF3	1,496435	1,53E-06
F12	1,479671	1,54E-06
PELI1	-2,82871	1,56E-06
CTXN1	1,608207	1,58E-06
ACBD7	2,62327	1,62E-06
SHANK3	-2,08262	1,62E-06
AARS2	1,665331	1,62E-06
SMARCA2	1,897539	1,62E-06
ZNF282	1,703791	1,65E-06

ALDH4A1	2,789989	1,66E-06
EFNB1	-1,92207	1,66E-06
CYTH4	-9,02969	1,67E-06
BASP1	8,921334	1,70E-06
CHST2	-8,91119	1,70E-06
TUBGCP3	-1,52776	1,72E-06
MYO1E	-2,22548	1,73E-06
RINL	-2,32194	1,74E-06
ELMOD1	-4,50527	1,74E-06
PLCG2	-1,73128	1,75E-06
BHLHA15	-3,54454	1,76E-06
TMBIM4	1,930866	1,77E-06
KAZN	-1,83738	1,81E-06
ANKRD29	-3,8866	1,81E-06
RAMP1	-2,62425	1,81E-06
POLR2J3	2,601126	1,82E-06
MCTP1	3,660848	1,82E-06
INSR	-2,07757	1,85E-06
UBE2W	-1,60383	1,85E-06
ACOT7	-1,6865	1,87E-06
CCDC68	-3,79829	1,88E-06
SLC2A10	8,010824	1,88E-06
COBL	-1,79315	1,88E-06
DECR1	1,614877	1,90E-06
MALL	-8,74137	1,90E-06
ZEB1	-4,19026	1,92E-06
SLC9A4	8,22982	1,93E-06
IFITM1	3,899939	1,94E-06
SOS2	1,953883	1,95E-06
CTPS2	1,614984	1,96E-06
LINC00491	-8,88698	1,96E-06
CACNA1B	-4,66408	1,98E-06
GMPR	-2,85668	2,01E-06
FZD2	2,098541	2,04E-06
C14orf80	1,655637	2,04E-06
ZNF827	-2,2203	2,07E-06
CPE	-2,31209	2,09E-06
PSEN1	1,513561	2,11E-06
PGM5	8,769142	2,11E-06
SERINC5	1,9414	2,11E-06
DBN1	1,332571	2,14E-06
SHISA2	8,785183	2,15E-06
DOCK5	-1,93327	2,16E-06
AP3S1	-1,71031	2,17E-06
LDLRAP1	-1,96841	2,17E-06
DNLZ	1,674277	2,21E-06
FUNDC2	1,556629	2,25E-06
BICD1	-1,86876	2,25E-06
ACTG2	6,366272	2,25E-06
MFGE8	-1,93706	2,28E-06

FAM89B	1,633534	2,29E-06
KRIT1	1,564076	2,29E-06
ITGB4	-1,39594	2,31E-06
FAM222A	1,75391	2,31E-06
LYST	-2,82665	2,34E-06
SLC41A1	-1,79165	2,38E-06
KSR2	-2,38236	2,40E-06
HTR2C	8,692065	2,41E-06
RNF150	8,704324	2,44E-06
IL20RB	-4,49197	2,53E-06
SF3B4	1,305137	2,54E-06
SLC15A1	-9,02814	2,54E-06
BCKDHA	1,739045	2,55E-06
OSR1	-8,21656	2,56E-06
PCDHB4	8,887223	2,60E-06
SCUBE1	-8,8344	2,60E-06
CEP112	-2,97305	2,61E-06
ASNA1	1,338255	2,63E-06
SPOP	-1,6187	2,68E-06
LINC00858	8,780598	2,80E-06
CNNM1	8,656769	2,82E-06
CHRNA7	-3,46446	2,83E-06
OBSL1	2,045983	2,84E-06
ENO2	-2,28422	2,86E-06
USP53	-1,88709	2,86E-06
COPS6	1,334021	2,94E-06
CXCL2	-9,43556	2,94E-06
PCDHA5	8,654106	2,96E-06
GSAP	6,007495	2,97E-06
C11orf54	-2,15989	2,98E-06
LINC00152	-3,2161	3,00E-06
NAV1	-2,40565	3,00E-06
MAOA	-4,75771	3,00E-06
RASGRF1	2,554569	3,02E-06
SEMA3F	1,713493	3,05E-06
UCA1	-9,19119	3,09E-06
ADGRG6	-1,75112	3,09E-06
OAT	-2,36418	3,10E-06
TMEM163	8,686476	3,11E-06
FAM102B	1,53318	3,14E-06
GSE1	1,389334	3,14E-06
S100A13	1,66505	3,16E-06
PPP3CA	-1,61483	3,17E-06
GATA2-AS1	-8,19409	3,19E-06
MCOLN3	-5,07157	3,23E-06
TUBGCP2	1,389458	3,25E-06
TLE6	-6,88173	3,25E-06
SLC35B2	1,681533	3,28E-06
PROSER1	1,362871	3,33E-06
C1QTNF1	-5,44116	3,35E-06

GLDC	-2,61321	3,45E-06
NHSL1	-1,88585	3,48E-06
FURIN	-1,69132	3,49E-06
SLC2A4RG	1,51454	3,51E-06
TLCD1	1,817714	3,51E-06
CHMP1B	1,534021	3,54E-06
ZNF804A	-8,86825	3,54E-06
GAS6-AS1	-4,68525	3,54E-06
EML2	-1,762	3,58E-06
KRT80	3,113603	3,63E-06
HOXC-AS3	6,616748	3,67E-06
LBR	-1,79401	3,69E-06
AMZ1	8,707235	3,71E-06
EIF4EBP2	1,782781	3,78E-06
PIAS3	1,517865	3,80E-06
SALL4	3,198808	3,83E-06
PPM1E	9,148739	3,84E-06
CRABP1	8,765796	3,85E-06
KATNAL2	3,677577	3,88E-06
SLC47A1	7,892261	3,90E-06
MAFIP	2,283877	3,95E-06
PDXP	-1,51433	3,95E-06
TAGLN2	1,604652	3,96E-06
MARCH1	6,67208	3,96E-06
AEBP1	8,651378	3,96E-06
MRAS	-2,05583	3,97E-06
MCCC1	1,776943	3,97E-06
EDA2R	7,956292	3,98E-06
RAC3	-1,66158	3,98E-06
ARFGAP2	1,438499	4,00E-06
GBAP1	2,487141	4,05E-06
PDZD7	-3,58458	4,15E-06
TJP3	2,283568	4,26E-06
FHDC1	2,673241	4,36E-06
ITFG2	2,3151	4,38E-06
SLC38A6	3,108874	4,49E-06
H2AFJ	2,340529	4,51E-06
FBXO31	1,411238	4,65E-06
TUBE1	-2,38096	4,69E-06
ASAH2	3,057955	4,70E-06
CBX5	2,197094	4,71E-06
STUB1	1,388227	4,75E-06
LPCAT4	-2,21232	4,76E-06
SLC44A2	1,385557	4,83E-06
TPTE	8,573038	4,83E-06
PGBD5	5,649867	4,85E-06
TCF7L1	-1,93949	4,88E-06
HBA1	5,48991	4,93E-06
CHUK	-1,57485	5,00E-06
C17orf58	-2,01668	5,00E-06

ADAMTS19	8,54891	5,00E-06
MTCL1	-2,04216	5,03E-06
WNT3A	-6,6349	5,08E-06
PI4KB	1,499853	5,08E-06
COMMD7	1,641913	5,09E-06
PYCRL	1,635283	5,17E-06
ZNF32	1,871537	5,21E-06
CABLES1	1,992999	5,21E-06
OAS1	6,233914	5,39E-06
HOXD8	-3,6337	5,44E-06
FGFBP1	-8,81414	5,44E-06
ALKBH4	1,702681	5,54E-06
PXK	-2,8654	5,55E-06
RASSF8	2,033504	5,60E-06
ZNF10	8,52212	5,60E-06
CASC9	2,861135	5,67E-06
NOCT	-1,76166	5,67E-06
MAN2B1	1,521365	5,73E-06
PLXNB3	2,225389	5,74E-06
SMAD6	2,823018	5,77E-06
LRRC37A3	2,692554	5,79E-06
CD8A	3,701627	5,81E-06
LRRN1	6,557419	5,81E-06
LMBRD2	3,333561	5,85E-06
TAF6	1,374451	5,94E-06
ZBTB18	-4,17612	6,04E-06
CENPV	-2,55815	6,16E-06
CBARP	-2,26687	6,17E-06
RCBTB1	-1,66115	6,25E-06
PRICKLE2-A	8,516266	6,25E-06
LOC644919	8,561049	6,27E-06
NHLRC1	8,664189	6,27E-06
LINC01132	8,735969	6,30E-06
GRIK3	8,727538	6,34E-06
LRCH4	1,442354	6,54E-06
ATAD3B	-2,27975	6,57E-06
BMP1	-1,60122	6,68E-06
ARHGAP26	-2,75068	6,70E-06
RRAS2	-1,673	6,73E-06
NUPR1	6,177079	6,76E-06
SARM1	3,675925	6,81E-06
FITM2	2,03763	6,81E-06
MB21D1	8,501188	6,82E-06
BANK1	-8,00947	6,94E-06
PORCN	-2,30748	6,96E-06
ANTXR2	-4,48999	6,98E-06
DYNC2H1	2,298876	6,98E-06
KCNN2	8,477195	7,06E-06
NEK11	2,836691	7,08E-06
PCDHA13	8,48192	7,08E-06

ADAMTS9	8,767127	7,18E-06
GATA2	-2,3761	7,29E-06
TMEM74B	-8,01986	7,40E-06
SRPK2	1,492042	7,42E-06
APP	-1,26073	7,43E-06
SNORD25	-3,55195	7,50E-06
KCNV1	8,626659	7,58E-06
NSDHL	1,435904	7,67E-06
SNAI2	-8,8722	7,75E-06
IFT22	1,771363	7,85E-06
PCSK9	-2,79942	7,90E-06
ATXN1L	1,558516	7,91E-06
FRS2	1,870783	7,95E-06
RILPL2	-2,73865	8,01E-06
TENM1	8,523561	8,07E-06
CDC25B	1,401324	8,11E-06
FGFRL1	-1,42863	8,12E-06
PRTG	8,445683	8,33E-06
NDST1	1,904488	8,46E-06
IFNLR1	-2,3979	8,47E-06
TMEM206	-2,1491	8,49E-06
MYBL2	1,271319	8,58E-06
ELOVL4	8,577448	8,63E-06
C4orf19	8,458651	8,69E-06
COX5B	1,272279	8,70E-06
SLC18B1	-1,8234	8,71E-06
DNAJC24	-1,95621	8,75E-06
PBX1	2,666949	8,83E-06
SIKE1	1,335795	8,87E-06
PIM2	-1,75776	8,92E-06
PMVK	1,834344	9,04E-06
RHOB	1,507428	9,04E-06
PDX1	-8,60277	9,10E-06
CBX7	-3,14221	9,15E-06
ECH1	1,501929	9,16E-06
F2R	-1,72413	9,16E-06
MTA2	1,252611	9,29E-06
ZNF212	1,855906	9,29E-06
GABARAPL	-3,64775	9,36E-06
RIT1	2,310416	9,40E-06
CCDC83	8,479962	9,46E-06
MICAL1	-2,15841	9,56E-06
WISP2	9,043356	9,56E-06
DSCAM	8,543961	9,62E-06
SP100	-3,77314	9,71E-06
CRIM1	-2,32449	9,79E-06
FBXW4	1,792166	9,83E-06
HDAC4	-2,94275	9,95E-06
LINC01138	-6,55047	9,95E-06
IL15RA	-2,83076	1,00E-05

SSR2	1,468823	1,00E-05
SH3YL1	1,591226	1,01E-05
MRPL41	1,388165	1,02E-05
TMEM191/	2,244144	1,02E-05
ELFN1	-3,32746	1,02E-05
IMPA2	1,515734	1,03E-05
ARNTL	-2,55442	1,03E-05
ABHD8	-3,75934	1,03E-05
COA3	1,864689	1,04E-05
CDKN2C	1,913765	1,06E-05
EPHB4	1,610479	1,07E-05
DACH1	8,390247	1,08E-05
INPP4B	1,959051	1,08E-05
MPPED2	8,585285	1,09E-05
CHST7	-2,7037	1,09E-05
LINC00664	8,499531	1,09E-05
AP1S1	2,023872	1,09E-05
COL5A1	7,73084	1,09E-05
MCCC2	1,509625	1,10E-05
AMIGO1	7,585775	1,11E-05
ZNF443	2,273701	1,11E-05
AREG	-5,38531	1,12E-05
CCDC82	-2,45203	1,14E-05
ROBO3	-2,85107	1,14E-05
EXTL3	-1,57311	1,14E-05
LOC10272	-8,887	1,15E-05
MEGF11	-5,44611	1,15E-05
C2orf88	-7,86852	1,15E-05
IRAK1	1,290983	1,15E-05
LOC72873	8,393262	1,16E-05
TAF11	1,607644	1,17E-05
FNTA	-1,49621	1,18E-05
LANCL3	-8,54322	1,19E-05
SMARCA4	1,575913	1,19E-05
ZNF107	1,64141	1,19E-05
FAM63B	-2,7185	1,20E-05
WRAP73	-1,96632	1,20E-05
FOXD1	-3,37404	1,20E-05
MT2A	-2,50931	1,20E-05
PLA2G4A	-4,32246	1,21E-05
DUSP16	1,641096	1,21E-05
FKBP1B	-3,22683	1,22E-05
HLA-H	-6,49322	1,22E-05
FMO5	8,363107	1,24E-05
LRRFIP1	-1,27487	1,24E-05
CSRNP1	-2,7327	1,25E-05
PRDM16	-3,02287	1,28E-05
CDK6	-3,07552	1,28E-05
FXYD1	-5,42851	1,30E-05
EFNA2	-2,17101	1,31E-05

SLC39A1	1,788782	1,33E-05
CRLF1	-2,26578	1,34E-05
PLA2G3	8,623793	1,34E-05
STMN3	-1,42731	1,35E-05
LTK	-7,83331	1,35E-05
TPST1	-2,1895	1,36E-05
UPK1A-AS1	-6,62941	1,37E-05
LOC72855(	1,979647	1,37E-05
S100P	5,771672	1,37E-05
SEPHS1	1,483667	1,37E-05
CYP4F11	-2,2902	1,38E-05
C1RL-AS1	-8,53559	1,39E-05
MT1X	-2,28556	1,40E-05
BLACAT1	-4,35168	1,40E-05
LOC37444:	4,377092	1,41E-05
DUSP6	-7,85393	1,41E-05
DNASE2	1,649504	1,41E-05
GFI1	-4,37885	1,41E-05
EPS15	-1,46542	1,42E-05
INAFM2	-2,23211	1,42E-05
ZIC4	8,335446	1,42E-05
LOC90768	8,355633	1,42E-05
NPR2	-7,99235	1,43E-05
NUMA1	1,251075	1,43E-05
TRIM56	1,72109	1,45E-05
PRDM1	-6,48424	1,46E-05
STAT4	-6,69513	1,46E-05
EFR3A	-1,71022	1,47E-05
NIPSNAP3A	2,318927	1,48E-05
NAB1	-1,88504	1,48E-05
TLR3	-8,59153	1,48E-05
SRGAP2D	-2,35094	1,49E-05
MICAL2	-1,94599	1,49E-05
DPP3	1,552689	1,50E-05
PDZRN3	8,32228	1,51E-05
MYL12B	1,279992	1,51E-05
NCMAP	-8,49292	1,52E-05
MAP10	8,379677	1,52E-05
ARSD	1,989769	1,52E-05
STEAP2	-8,3324	1,53E-05
PQLC1	-1,4845	1,53E-05
RPP40	1,624519	1,54E-05
WDR83	1,669967	1,55E-05
BMP8B	-2,20983	1,56E-05
PCLO	2,272117	1,56E-05
ROGDI	1,947309	1,57E-05
CRYM	-7,9214	1,57E-05
RHOV	2,206321	1,57E-05
SNRNP48	-1,36988	1,58E-05
ANKDD1A	-4,42063	1,60E-05

MPP5	1,474774	1,62E-05
HNRNPAB	1,20613	1,62E-05
C7orf31	-8,62723	1,62E-05
HMGN1	1,245043	1,65E-05
PGLS	1,493785	1,66E-05
AQP3	3,435858	1,68E-05
NPDC1	1,334511	1,69E-05
RAB3D	1,941058	1,69E-05
MMD	-2,23207	1,73E-05
RRAGD	-2,04482	1,73E-05
IFI44L	8,830638	1,73E-05
SH2D3A	-1,89923	1,75E-05
VPS72	1,676568	1,77E-05
ATP5G2	1,274874	1,78E-05
FBXO15	5,584093	1,80E-05
KARS	1,248558	1,81E-05
DMTN	-1,51472	1,81E-05
CDON	-2,46998	1,81E-05
ABLIM1	-1,78569	1,82E-05
FRAT1	8,282728	1,84E-05
LTBP4	-1,47128	1,85E-05
KLHL15	-2,03968	1,85E-05
DNAAF3	-1,38288	1,86E-05
LOC10013(	-8,47982	1,86E-05
SYT7	1,282051	1,88E-05
GLRX	-3,79997	1,88E-05
IRAK2	-4,9108	1,88E-05
SETDB1	1,607248	1,92E-05
PPP1R9B	-1,45414	1,92E-05
QPCT	8,344938	1,97E-05
HID1	1,955926	2,02E-05
DNM3	-4,37019	2,03E-05
RPP25	1,632911	2,05E-05
GSR	-1,51095	2,09E-05
RPL17	1,576593	2,10E-05
PITX3	-5,39574	2,10E-05
CCNG2	2,226827	2,10E-05
ABHD16A	1,441088	2,11E-05
MLYCD	1,815852	2,13E-05
EEPD1	-3,13046	2,13E-05
CNOT2	1,264966	2,14E-05
COL4A4	-3,22265	2,14E-05
ZNF701	1,853952	2,15E-05
HIP1R	-1,34167	2,15E-05
WDR73	1,549156	2,16E-05
RECQL4	-1,47208	2,17E-05
AGR2	8,683433	2,18E-05
ATG16L1	-1,40178	2,19E-05
RASL10B	8,260485	2,22E-05
TNFRSF10(	-8,47286	2,23E-05

JAZF1	-3,71489	2,27E-05
LYNX1	-8,42598	2,28E-05
PTGS1	-4,55863	2,28E-05
TNPO2	1,735335	2,28E-05
CDCA3	1,469299	2,31E-05
KIAA1522	1,268727	2,31E-05
TMOD1	-2,82903	2,33E-05
GADD45A	-2,50411	2,33E-05
SAP130	1,261294	2,35E-05
HIPK2	-1,76703	2,35E-05
DVL1	-1,38883	2,36E-05
FES	-2,39117	2,36E-05
KRTAP3-1	4,625566	2,36E-05
SFMBT2	7,508127	2,37E-05
DACT3	-4,32259	2,38E-05
RASSF6	-6,06146	2,41E-05
SMARCD3	-1,81647	2,41E-05
SLFN11	8,251655	2,41E-05
BATF3	-7,94342	2,43E-05
PIK3C2B	1,504952	2,44E-05
SLC22A31	-2,10622	2,45E-05
TET3	1,807276	2,45E-05
TTC29	8,227696	2,45E-05
KREMEN1	-1,3469	2,45E-05
PROCA1	6,153812	2,47E-05
APOBEC3F	-2,32845	2,48E-05
NKX2-8	-5,34666	2,49E-05
DIS3	-1,35342	2,50E-05
ZNF704	2,010345	2,50E-05
P4HA1	-1,8412	2,50E-05
BMF	4,452336	2,53E-05
CTSB	-1,66977	2,53E-05
FLOT2	1,902127	2,54E-05
MIER1	-1,42405	2,56E-05
TXNDC9	1,617286	2,59E-05
NEIL2	-2,04486	2,59E-05
TERF2	-1,81242	2,59E-05
DSG2	-1,31517	2,59E-05
ERO1A	-1,38737	2,60E-05
PYCR2	-1,76508	2,60E-05
TMEM183I	2,07472	2,60E-05
B3GALT4	5,246618	2,61E-05
INHA	8,934155	2,62E-05
MEF2C	-2,51648	2,63E-05
PRKCI	1,296766	2,64E-05
MRPL28	1,486248	2,65E-05
RBFOX2	-1,43746	2,65E-05
ADGRB2	-2,35917	2,67E-05
ST3GAL6	-7,68623	2,68E-05
TAMM41	1,545064	2,68E-05

KLF3	-1,62176	2,69E-05
CDH1	1,803905	2,70E-05
TNFRSF18	-2,90639	2,73E-05
RFTN2	8,282502	2,73E-05
ZNF354A	1,851628	2,73E-05
ABCB4	8,427931	2,80E-05
ARMCX1	8,287873	2,81E-05
SLC16A9	-1,93218	2,81E-05
CASZ1	2,045663	2,85E-05
RAI14	-1,49119	2,86E-05
VPS52	1,24686	2,87E-05
RASSF10	7,431332	2,88E-05
TMEM170I	2,868578	2,89E-05
EIF2AK4	-1,70419	2,89E-05
ZKSCAN5	1,426027	2,90E-05
ZCWPW1	2,644004	2,91E-05
UBL3	1,561985	2,92E-05
LGALS3BP	-2,7812	2,93E-05
NMUR2	8,184898	2,94E-05
TRAPPC2L	1,289844	2,96E-05
DHFRL1	2,087313	2,98E-05
TMEM168	1,439124	3,00E-05
C4orf32	-2,18425	3,04E-05
GAB1	2,108222	3,04E-05
TMEM181	-1,53484	3,05E-05
EXD2	1,394025	3,06E-05
IFIT5	-2,22518	3,08E-05
RPRM	5,322835	3,09E-05
GALNT2	-1,38908	3,11E-05
USP16	-1,35054	3,13E-05
LOC40332	-7,6331	3,17E-05
FCGR1A	7,60662	3,17E-05
BEX2	-2,5006	3,18E-05
ZYX	-1,6074	3,24E-05
RAI2	8,24684	3,24E-05
PRADC1	2,168977	3,25E-05
RAB27A	2,042766	3,26E-05
RYR1	-4,84391	3,26E-05
ADAM21	-8,44711	3,32E-05
SNRPE	1,251715	3,34E-05
ABCC4	-1,63847	3,38E-05
RNF126	-1,51337	3,38E-05
S100A10	1,443627	3,40E-05
FBXW9	1,651992	3,40E-05
SFN	-1,52685	3,41E-05
TBC1D7	1,750189	3,41E-05
BHMG1	-6,48116	3,44E-05
PLOD3	1,260989	3,46E-05
TPPP	-2,45894	3,48E-05
HSPA1A	7,121308	3,49E-05

ZNF563	8,139161	3,61E-05
AP3B2	4,1582	3,61E-05
LLPH-AS1	8,138617	3,62E-05
IL6R	-3,24449	3,68E-05
RARA-AS1	-8,79029	3,70E-05
TTC7B	-1,40316	3,73E-05
ZNF398	1,292258	3,73E-05
GALNT3	2,101619	3,80E-05
LINC01604	-2,77886	3,82E-05
PHTF1	2,138363	3,85E-05
CDH15	-3,46231	3,91E-05
GSDMD	-2,27672	3,99E-05
WBSCR16	1,27295	4,00E-05
ZNF33A	1,345396	4,02E-05
HEATR1	-1,32849	4,03E-05
GATA5	8,133711	4,03E-05
MTG1	1,800333	4,05E-05
ABCA12	2,375515	4,05E-05
RBM47	1,552048	4,05E-05
STARD8	-2,61521	4,08E-05
ZNRF1	1,258661	4,08E-05
PSMA1	-1,2306	4,09E-05
C9orf72	-3,45692	4,13E-05
NIPA1	-1,35756	4,16E-05
WASF3	2,345619	4,20E-05
RFWD2	1,287583	4,22E-05
KIF5A	-3,2283	4,25E-05
BCYRN1	1,286497	4,29E-05
FAH	1,50828	4,31E-05
ZNF610	8,10402	4,33E-05
LIPH	-3,21673	4,35E-05
ZNF577	-8,28258	4,39E-05
ABCG4	-4,67872	4,40E-05
PHLDA1	-2,40259	4,40E-05
HTRA1	8,225693	4,40E-05
MTRF1L	-1,42816	4,42E-05
BAG4	-1,36775	4,42E-05
NRP1	1,976479	4,43E-05
PVR	-1,88715	4,44E-05
LOC100128	8,094733	4,46E-05
ABHD3	-1,79293	4,52E-05
S1PR2	-1,74047	4,52E-05
RIPPLY3	8,101789	4,53E-05
SDCBP	-1,60978	4,55E-05
C17orf97	2,23137	4,56E-05
MPZ	-4,80687	4,58E-05
CYB5A	1,607658	4,60E-05
SMG7	1,152245	4,63E-05
SCAMP5	2,102751	4,64E-05
PSAT1	-2,23387	4,66E-05

GATS	2,940858	4,70E-05
PRKCE	-2,47592	4,74E-05
RAB13	1,439478	4,77E-05
PGF	-3,39538	4,78E-05
CHORDC1	-1,87752	4,80E-05
LPPR4	-8,29102	4,80E-05
TSPAN7	-8,26893	4,81E-05
RHBDL3	-3,22355	4,82E-05
ANKRD22	-5,25539	4,82E-05
SLC6A14	8,088205	4,84E-05
FAF1	-1,81853	4,84E-05
ARID4A	1,892755	4,93E-05
RASA3	-1,62142	4,95E-05
CNTNAP3P	-8,30356	5,01E-05
FLCN	1,438878	5,04E-05
LOC10013(	-3,98417	5,06E-05
CACNA1G	-8,27772	5,06E-05
MROH6	-1,52152	5,08E-05
DDA1	-1,30586	5,15E-05
BTN3A2	-3,12258	5,16E-05
EMC1	-1,18358	5,17E-05
F10	-8,2479	5,18E-05
SPG21	1,217641	5,19E-05
LHFPL2	-1,37121	5,21E-05
TAP2	-1,5323	5,23E-05
THEMIS2	-2,77676	5,27E-05
LPAR2	1,3085	5,30E-05
THEM6	1,529811	5,30E-05
HDHD2	1,682381	5,35E-05
DBI	1,188065	5,36E-05
MYO10	-1,28324	5,37E-05
CST6	-8,28635	5,38E-05
GUCY1B3	8,092151	5,38E-05
SERPINE1	-6,68751	5,38E-05
PPFIBP2	2,194084	5,42E-05
SNX25	-1,95852	5,42E-05
ASB8	1,791802	5,46E-05
PARD6G	-1,89712	5,49E-05
NLRP1	-7,57349	5,58E-05
FHOD3	-3,16836	5,60E-05
CETN2	1,495013	5,62E-05
TRIM45	2,853589	5,64E-05
CITED1	-4,07503	5,69E-05
RHOF	-1,40081	5,73E-05
FRMD6-AS:	4,565219	5,76E-05
SLC16A1	-1,28021	5,78E-05
USP51	8,116321	5,80E-05
PGK1	-1,17705	5,82E-05
ARHGEF40	-2,94532	5,84E-05
KCNQ5	8,15685	5,86E-05

PCDHB10	5,527581	5,94E-05
BMPR2	1,629598	5,95E-05
PVALB	8,188867	5,96E-05
CYP4V2	-2,96516	5,99E-05
EIF1AY	-8,21588	6,02E-05
KCNMA1	5,420729	6,03E-05
ZNF786	1,467551	6,04E-05
LINC01123	-1,88763	6,04E-05
PRTN3	-8,34786	6,04E-05
BVES	-8,32809	6,04E-05
SNCAIP	4,265904	6,08E-05
AKT2	1,322302	6,12E-05
RLF	-1,32101	6,12E-05
SH3GL3	4,305255	6,12E-05
SH3BP1	-1,83681	6,14E-05
LINC00649	8,027765	6,14E-05
PCDH9	8,119576	6,15E-05
CCDC149	1,854708	6,15E-05
EPG5	1,975417	6,16E-05
BCCIP	-1,18315	6,18E-05
SLC25A29	1,543333	6,21E-05
FAHD1	1,262798	6,22E-05
MYH7B	5,384963	6,22E-05
TGDS	-2,19869	6,22E-05
B4GAT1	2,232005	6,25E-05
BAG2	-1,60608	6,26E-05
TPD52L1	1,680552	6,26E-05
AP5Z1	1,402835	6,28E-05
ZDHHC9	1,424303	6,33E-05
LOX	8,032231	6,34E-05
HIST1H2BH	8,0307	6,34E-05
KCNK13	-3,69941	6,38E-05
EDC3	1,375323	6,39E-05
PAPSS2	-3,19486	6,41E-05
RHOT2	1,227298	6,50E-05
ZNF726	8,175617	6,51E-05
BGN	9,666876	6,51E-05
ABCA1	-8,19556	6,55E-05
CSPP1	-1,89347	6,57E-05
ABCB6	1,555614	6,57E-05
AKIRIN1	-1,2308	6,57E-05
GSDMB	-3,40653	6,65E-05
ARAP2	-1,84395	6,66E-05
KLF15	-3,31359	6,72E-05
MPDU1	1,351502	6,74E-05
MORC3	-1,58068	6,76E-05
SIM2	-1,99577	6,77E-05
LAMA4	-8,1844	6,79E-05
MRPL10	-1,22176	6,79E-05
PLCE1	-3,72203	6,80E-05

GPRC5B	-3,04532	6,82E-05
B4GALNT3	-2,43497	6,86E-05
EGLN3	-2,09379	6,87E-05
TM7SF2	1,990882	6,91E-05
BMP2K	-1,41711	6,93E-05
FAM117B	1,66532	6,96E-05
LRRC61	1,820497	7,04E-05
CHML	1,493304	7,07E-05
S100A11	1,672506	7,08E-05
DESI2	-1,26048	7,08E-05
JOSD1	-1,49735	7,10E-05
IQCH-AS1	1,883409	7,15E-05
LOC100506	-4,96249	7,18E-05
PDE4B	-6,0886	7,21E-05
ATP6AP1L	-2,60687	7,21E-05
SPNS1	1,410491	7,25E-05
IFI30	1,603275	7,26E-05
TNNT1	1,176975	7,26E-05
BHLHE40	-2,08858	7,26E-05
SHFM1	1,176263	7,28E-05
CAMK2N1	-1,93411	7,37E-05
KCNQ1OT1	3,300937	7,37E-05
XPOT	1,361868	7,40E-05
NEURL1B	1,775043	7,47E-05
GNA11	-1,21329	7,56E-05
CYP39A1	-6,05525	7,66E-05
PEX10	1,351023	7,70E-05
GDI2	1,178064	7,73E-05
SLC22A11	8,192066	7,77E-05
ABCD3	-1,5865	7,95E-05
MS4A15	-8,24867	7,95E-05
DNAJB4	-2,77754	8,01E-05
CASP4	-4,12262	8,02E-05
EFHC1	2,373475	8,04E-05
NIPSNAP1	1,317142	8,07E-05
LAMTOR2	1,298321	8,09E-05
TMEM51	-1,61842	8,09E-05
KLHDC9	4,895662	8,21E-05
RNF128	-8,00744	8,24E-05
KLHL17	-2,06664	8,29E-05
TRIP11	1,273608	8,29E-05
CFAP74	-8,31747	8,41E-05
ITPR2	1,781885	8,43E-05
DNAJC16	-1,6117	8,51E-05
DSTYK	1,818693	8,63E-05
KBTBD8	-2,85729	8,67E-05
ZIC2	-1,85022	8,71E-05
NSD1	1,701283	8,78E-05
RELL1	-1,63134	8,79E-05
ZNF646	1,31497	8,85E-05

RHBDD1	-1,67052	8,87E-05
RRAD	-8,2414	8,93E-05
CDC14B	-1,79826	8,98E-05
SLC27A1	-2,10504	9,00E-05
LEMD2	1,363421	9,00E-05
TIGD6	1,919054	9,01E-05
DLEU2	-2,63755	9,02E-05
RNPEPL1	-1,45037	9,05E-05
SH3TC2	-3,59309	9,09E-05
ZNF98	8,046301	9,13E-05
CAP1	-1,24732	9,19E-05
RRN3P1	-8,14287	9,32E-05
FBXL16	-1,61416	9,35E-05
ZAK	-1,26705	9,60E-05
NIPAL2	1,961704	9,71E-05
CBWD5	-1,3339	9,74E-05
HOXC13-AS	4,39096	9,74E-05
PAK2	1,196554	9,74E-05
WRB	1,486874	9,76E-05
TMEM63C	-2,7071	9,82E-05
GALNT5	-12,2885	9,82E-05
DMXL2	1,482909	9,94E-05
HDDC3	1,490304	9,94E-05
SEPT2	-1,15972	9,95E-05
NUPL1	-1,40956	0,0001
APOE	2,920654	0,000101
NIT2	1,337467	0,000101
TMPO-AS1	2,008284	0,000102
CTTNBP2	-4,29791	0,000102
ZMAT2	1,222559	0,000103
ULK1	1,738511	0,000104
ZFP1	1,396072	0,000104
VAMP8	2,021231	0,000104
NFE2	2,521933	0,000104
ADAMTS16	-8,23002	0,000104
MED20	1,488322	0,000104
ALOXE3	-2,76291	0,000105
PRPS1	-1,31073	0,000105
LOC100996	3,053108	0,000105
GPR68	7,922437	0,000105
CTNNAL1	-1,57283	0,000105
LDLRAD3	-1,65378	0,000106
PART1	7,935652	0,000106
CORO1B	1,665713	0,000108
CDC27	-1,13814	0,000108
AFF3	7,11611	0,000108
THBS3	2,375641	0,000108
ADCY7	-1,48065	0,000108
PSMD7	1,11296	0,000108
RPTOR	-1,20859	0,000108

GPATCH2	1,348191	0,000109
LOC339803	6,171354	0,000109
NRM	1,737446	0,000109
CPM	-3,00123	0,00011
EXOSC9	-1,3289	0,00011
C15orf52	-5,16265	0,00011
LINC00869	-2,78808	0,000111
SMAD7	2,00805	0,000111
DTD1	1,321262	0,000111
TUBB3	1,093223	0,000112
NEK9	-6,82939	0,000112
THUMPD3-	2,305446	0,000113
MPP7	-1,209	0,000114
CD164L2	-3,28624	0,000115
MTIF3	1,55921	0,000115
MTSS1L	1,343276	0,000115
RAB22A	1,259267	0,000116
HIST1H2BK	2,534228	0,000116
DENND5A	-1,55528	0,000116
CA12	2,661863	0,000116
OLFML3	6,449629	0,000116
HDAC9	-4,4118	0,000118
CASK	-1,24888	0,000119
LPCAT2	-1,87239	0,00012
SEMA7A	-1,62111	0,00012
EVI5	-1,73777	0,00012
GTF2H3	-1,35807	0,00012
TOP2B	1,266157	0,000121
UGT8	-7,88682	0,000121
LEMD1	-8,11845	0,000122
PPL	1,654963	0,000122
TC2N	1,695654	0,000122
MEF2D	-1,53024	0,000122
DVL3	1,146237	0,000123
GJA3	-1,67958	0,000123
JAG2	-1,53185	0,000123
VPS45	1,29332	0,000123
CYB5B	-1,65187	0,000123
ALDH3A2	1,149511	0,000123
FAM196A	8,056727	0,000124
GART	-1,14181	0,000124
PSMB3	-1,12638	0,000124
NT5DC3	-1,55331	0,000124
PPP1R3F	2,58774	0,000125
GAL	-1,15152	0,000125
P3H2	2,253551	0,000125
MIR4458H(	7,881769	0,000125
KHDC1L	-8,05167	0,000126
GRAMD1A	-1,19886	0,000127
KLF4	-1,88473	0,000127

MPG	1,281403	0,000128
SDE2	-1,82639	0,000128
CPNE2	-1,83563	0,000128
SLC7A5	1,186324	0,000128
SPRYD7	-1,72902	0,000128
SHISA4	-2,50452	0,000129
CAPRIN2	-1,6739	0,000129
MARCKS	-1,30644	0,00013
TMEM255B	-4,31954	0,00013
SH3BGRL	-1,74742	0,000131
TFCP2	-8,52861	0,000131
CSPG4	-7,85725	0,000131
DGKQ	-1,61867	0,000132
LOC100506	-1,42337	0,000132
SYNJ2	-1,34469	0,000132
DNAJC2	1,270546	0,000133
GTF2IP1	2,107946	0,000133
FOXI3	7,868964	0,000134
KLHDC3	1,816488	0,000134
PIGN	1,834475	0,000134
CBLL1	1,242221	0,000134
STRIP2	-2,24242	0,000134
TRIM10	-8,03765	0,000135
PIGQ	1,965257	0,000135
LINC00920	-8,03595	0,000135
TRIL	7,927841	0,000135
TUBA1B	1,063615	0,000136
HDGF	1,140424	0,000136
DOCK9	-1,61875	0,000136
OTUD7B	1,183674	0,000136
SMYD3	-1,67141	0,000136
ZNF611	1,667386	0,000137
CCDC90B	1,301752	0,000137
BACE1	1,723937	0,000138
ACTL6A	1,23359	0,000138
KCTD15	1,870751	0,000139
NBPF15	1,768423	0,000139
TMEM61	-8,09055	0,000139
OS9	1,213693	0,000139
TWISTNB	-1,31091	0,00014
RAET1L	-7,82162	0,00014
ASPM	1,35761	0,00014
S100A9	8,683857	0,000141
LIMCH1	-2,6867	0,000141
KIF3C	1,514155	0,000142
DES	-8,09886	0,000142
MGC72080	1,507328	0,000143
NR4A3	-3,05975	0,000143
DENND2D	1,328675	0,000144
ATG2B	1,685977	0,000144

POU6F2	7,856448	0,000144
ARV1	2,068186	0,000144
CFAP97	-1,32914	0,000144
TFB1M	-1,85185	0,000144
ROBO4	-8,51516	0,000144
HADH	1,525641	0,000145
NUDT1	1,702145	0,000146
EPN2	-1,38385	0,000146
GADD45GII	1,454046	0,000146
PON3	-8,07527	0,000147
TXNDC12	-1,39485	0,000147
MYSM1	-1,63893	0,000147
KLHDC2	1,603766	0,000148
HOXA11	-3,03427	0,000148
AMOTL2	2,173817	0,000149
NELFCD	1,515755	0,000149
PDZD8	-1,30249	0,000149
MMP1	-8,21858	0,000149
ITPR3	-1,24177	0,00015
TNFRSF10A	-1,65436	0,00015
ELMO3	1,377384	0,000151
TCF12	-1,51713	0,000151
FRRS1	-2,64311	0,000151
NPAS3	7,902289	0,000151
DBNDD1	1,288131	0,000151
FOXO1	-2,37561	0,000151
ZNF442	7,822419	0,000152
GHR	7,8258	0,000152
SEPT11	-1,13439	0,000152
SNX27	1,477015	0,000152
NADSYN1	1,45897	0,000153
COLCA1	7,843676	0,000154
HIST1H3G	7,827151	0,000154
DUXAP8	2,200388	0,000154
CD83	-1,88703	0,000154
ATP8B1	1,315155	0,000155
REPIN1	1,134402	0,000156
SNRNP25	1,591082	0,000156
RHOBTB2	-1,59203	0,000157
DHDH	-3,63092	0,000159
CYS1	-7,26372	0,00016
WIF1	8,115369	0,00016
HOXD12	8,035163	0,000161
ACTL8	3,387138	0,000161
FAM169A	-2,14705	0,000161
IGSF11	7,80839	0,000161
PSMB2	-1,08611	0,000162
MKNK2	-1,42592	0,000163
CGNL1	-3,38412	0,000163
FLT4	7,150035	0,000163

ALDH1A3	-5,24579	0,000165
PARP2	-1,22298	0,000165
BLNK	7,448747	0,000166
CLCF1	-3,26653	0,000167
IRF2BPL	-1,5648	0,000167
ATP5A1	1,166753	0,000168
PNPLA3	-5,37812	0,000169
TAF12	-1,38702	0,000169
RCN3	6,076797	0,000169
CDH18	7,828236	0,000169
SPATS2L	-1,25753	0,00017
TIMM17B	1,575642	0,000172
PRELID1	1,129336	0,000172
POGZ	1,432596	0,000172
ZFP36L1	-1,43544	0,000173
BMPR1B	7,793532	0,000173
PRRG4	-2,24716	0,000176
C21orf58	2,044335	0,000177
ZBTB1	1,255319	0,000178
PMEPA1	2,875988	0,000178
HOTAIRM1	-3,27593	0,000178
LRRC42	-1,2759	0,000178
ELF5	3,248214	0,000178
MRPL9	1,70521	0,000179
TRAK2	1,218645	0,000179
ZCCHC24	-2,18122	0,000179
TMPRSS4	4,449219	0,00018
GABPB2	1,685298	0,00018
RAB9B	7,801799	0,00018
GBA	1,771914	0,00018
DDX11L2	-3,82707	0,000181
SYNE4	1,300773	0,000182
AXIN2	1,759592	0,000182
EGLN1	-1,2193	0,000183
SLC30A7	-1,54847	0,000183
F7	8,004526	0,000183
ATP6V1C2	1,952745	0,000183
ACTR3C	4,092123	0,000183
SLC2A9	-7,9766	0,000183
C1QL4	-4,50933	0,000184
IL6ST	-1,34	0,000184
ZNF345	7,779217	0,000184
TMEM19	1,476921	0,000184
DNAJC22	-1,82176	0,000185
KRT23	-1,87923	0,000185
LINC00674	1,670943	0,000185
CALCOCO2	-1,83775	0,000185
MRAP2	-8,05432	0,000186
GOLIM4	1,476256	0,000187
LAMB3	-5,84963	0,000187

MBD6	1,384643	0,000187
PSD3	-1,30734	0,000188
PIBF1	-2,01403	0,000189
RNF103	1,595338	0,00019
PARP8	-1,91179	0,000191
ZNF418	7,788684	0,000191
MXI1	-1,43162	0,000192
SCAND1	1,242482	0,000197
SLC12A5	5,063688	0,000197
CABLES2	1,44422	0,000197
TBCC	1,393995	0,000197
PLEKHG1	-1,84723	0,000197
C17orf96	-1,18583	0,000202
CALB2	-2,98246	0,000202
GYG2	7,799461	0,000202
FSTL3	-1,97094	0,000202
CHAF1B	-1,32357	0,000202
GGCT	1,219938	0,000202
MSH6	1,24653	0,000202
ZC3H18	1,140471	0,000203
PEX26	1,210538	0,000203
ABHD14B	-1,2415	0,000203
BBX	-1,28648	0,000203
SLC20A1	-1,13194	0,000205
S100A3	-7,15598	0,000205
TARP	7,987363	0,000206
LNX2	1,396648	0,000207
EFNA1	2,010208	0,000208
SAV1	-1,37985	0,000208
SLC30A4	-3,69214	0,000209
KIAA0040	-1,52203	0,00021
LOC11323(	1,639988	0,00021
CTSO	2,638029	0,00021
NFKB1	-1,54435	0,00021
G6PD	2,428719	0,00021
PDLIM5	-1,5354	0,000211
TRAM1L1	7,908018	0,000211
IGF1	6,915792	0,000212
LGALS1	-1,7706	0,000212
ZNF572	2,365891	0,000212
ALDOC	-1,75615	0,000213
RELB	-2,5584	0,000213
ZBTB12	2,24018	0,000213
MED14	-1,17586	0,000215
RRM2	-1,18776	0,000218
TRIM2	-1,42971	0,00022
ATP6V0E1	1,599937	0,00022
LRRC6	-3,08223	0,00022
MAPKAPK3	-1,27125	0,000221
PPP1R1C	-2,90397	0,000222

GABPB1-AS	2,137004	0,000223
PHC2	-1,53038	0,000225
WIZ	1,097014	0,000225
FUCA1	-1,51076	0,000227
ZNF772	2,821856	0,000227
ZNHIT1	1,576604	0,000229
GATA3-AS1	7,745435	0,00023
ZNF33B	1,376075	0,00023
SYT1	-1,58743	0,00023
ZDHHC1	-2,42808	0,000231
G6PC3	1,273717	0,000231
APPL2	1,30989	0,000232
COLGALT2	5,690752	0,000232
RNF180	7,783805	0,000233
NFATC4	2,616738	0,000234
ADAM10	-1,27151	0,000234
CYFIP2	-1,84772	0,000234
ATP5J2	1,418659	0,000237
LINC01006	6,88718	0,000237
MCF2L	-1,82701	0,000238
NRTN	-3,00181	0,000238
FARS2	1,487976	0,000239
C11orf45	-7,96517	0,000239
INTS3	1,32878	0,00024
AKIP1	-1,48828	0,00024
EPM2AIP1	1,596023	0,00024
CDCA7L	-1,71281	0,000241
STX4	1,431361	0,000241
TOMM20	-1,05454	0,000241
FMNL3	1,744939	0,000241
PCDHGA5	7,730632	0,000242
ADORA1	-2,45996	0,000243
MYADM	-1,55606	0,000243
CNTN1	-7,13439	0,000244
SEC31B	-4,64018	0,000245
LOC101927	-7,97425	0,000245
C7orf73	1,129832	0,000245
FBN2	3,038028	0,000248
MON1B	1,25105	0,000248
MXD4	2,82854	0,000249
DNAJC30	1,573997	0,00025
SFR1	-2,23428	0,00025
SAMHD1	-1,96599	0,00025
PCDHGA6	7,731072	0,000251
CDC42EP5	3,922306	0,000252
AURKA	1,37134	0,000252
SCIN	5,995094	0,000252
HAUS6	-1,19271	0,000253
CHIC1	7,71831	0,000253
TINCR	2,343545	0,000253

SLC12A9	1,76445	0,000255
CHPF	-1,61005	0,000256
RSPH3	-1,60884	0,000256
SEC16A	1,131889	0,000256
PIM3	-1,32006	0,000256
MCPH1	-1,27322	0,000256
METTL15	-1,34715	0,000258
CBFA2T2	1,354565	0,000258
STK31	7,749728	0,000258
LOC100996	-7,49232	0,000259
INPP5B	-1,76882	0,000264
CXCL3	-7,99974	0,000264
RAB24	2,187532	0,000264
KBTBD11	-2,05341	0,000265
RELT	-1,72206	0,000266
NR2F1-AS1	-2,3055	0,000267
TAF7	1,233966	0,000267
DPY19L3	1,61317	0,00027
SEC11A	1,07767	0,00027
DEGS1	-1,52653	0,000271
ABAT	2,450192	0,000272
PSMD5-AS1	2,15913	0,000274
PARP10	2,913883	0,000275
FN1	-2,00156	0,000277
MZT2B	1,257055	0,000277
FLVCR2	2,423763	0,000279
TMEM52	-1,48615	0,000281
ISL1	-7,90777	0,000283
CTSV	-2,03476	0,000283
HSPA2	2,46663	0,000285
LOC389602	-7,91456	0,000286
MLXIPL	-2,11775	0,000287
ARL11	7,685289	0,000287
ICOS	7,688723	0,000288
XKR7	7,767884	0,000288
GPR89B	2,26489	0,000288
SYNE2	1,61963	0,000288
S100A2	-3,38423	0,000291
KRT17	3,577595	0,000292
NDFIP2	-1,26866	0,000297
ZSCAN21	1,497193	0,000299
PLRG1	-1,0812	0,0003
NUP43	-1,40645	0,0003
CDK14	-2,04819	0,000301
PATZ1	1,361898	0,000302
PLP2	-1,14218	0,000302
PTP4A2	-1,14446	0,000303
COMTD1	1,247905	0,000303
MYH16	-7,09048	0,000304
RCAN1	-1,87192	0,000304

ACD	-1,22518	0,000305
ADAMTSL3	7,713189	0,000305
C1GALT1	-1,26071	0,000306
STK24	-1,24829	0,000307
STARD9	-3,01784	0,000316
KCTD9	-1,21642	0,000316
IQSEC2	-1,86421	0,000316
ARF3	1,120852	0,000316
TAF1A	-2,01219	0,000317
CSRP1	1,403305	0,000319
HPS5	-1,36995	0,000319
PLEKHA1	-1,38645	0,000319
FAM27E3	-7,87006	0,000322
TH	7,722977	0,000322
ZNF607	1,466014	0,000322
PRRG1	-2,22126	0,000322
CARS	-1,31569	0,000322
RSBN1	1,358974	0,000323
PTTG1IP	1,270535	0,000324
HIST1H2AE	7,913205	0,000324
NADK2	1,51485	0,000324
RASD1	3,808596	0,000324
ZNF280A	-7,84266	0,000324
CARNMT1	-1,82379	0,000324
MOCOS	-1,93336	0,000324
SLC35F2	-1,36703	0,000327
ESPN	-1,9263	0,000327
RABGAP1	1,275029	0,000327
POLR2J	1,465465	0,00033
SNHG16	-1,10437	0,000331
SLC25A1	1,259903	0,000331
COPG2	1,34166	0,000333
CALM1	1,261528	0,000335
DUS4L	1,721234	0,000336
MDN1	-1,32133	0,000336
TGFB1I1	-3,80246	0,000337
FGF19	-9,25011	0,000338
C12orf75	-1,42815	0,000339
GNB2L1	1,00051	0,000341
MGC50722	-7,85224	0,000343
PRKCQ	-7,86209	0,000345
MYRF	-5,61865	0,000347
DHRS13	1,529105	0,000347
LPIN3	2,301532	0,000347
ADCK1	1,658049	0,00035
ZNF764	1,622793	0,00035
AP5M1	1,298765	0,00035
ZSCAN16	2,441216	0,000351
HSF2	-1,35601	0,000354
FAM185A	2,469464	0,000354

CHRD	-3,5378	0,000355
PSMD4	1,123902	0,000355
PANK2	-1,29087	0,000357
LOC101928	-7,91789	0,000359
KIAA1324L	1,294357	0,000362
ZIC5	-2,77885	0,000362
SPATA18	4,945817	0,000363
UBE2Q2	1,342529	0,000363
SQLE	1,4548	0,000363
SH3GL1	-1,27668	0,000365
GPM6B	7,617074	0,000365
METTL10	-1,46432	0,000366
SRP68	-1,17415	0,000366
OVOL2	3,098504	0,000366
EPHB6	-2,17685	0,000367
PKN3	-1,45426	0,000367
TACO1	1,126641	0,000371
RNF122	1,785468	0,000371
COX14	1,36152	0,000372
LHX6	-2,36834	0,000373
APBB2	-1,257	0,000375
RAB1B	1,439161	0,000376
FLNB	-1,23866	0,000378
PUS10	2,027068	0,000382
TMEM132E	8,27195	0,000382
RNF125	-2,23645	0,000382
NSMF	-1,56897	0,000384
ID4	5,712585	0,000384
CCPG1	2,81537	0,000385
IGSF5	4,011027	0,000387
ZNF571	5,471635	0,000388
UTP6	-1,1439	0,00039
HOXB9	-5,50163	0,000392
AP2A2	-1,11261	0,000392
GIPR	-7,8159	0,000392
SULT1C2	-7,8159	0,000392
FER1L4	-3,01909	0,000395
KCNU1	7,61238	0,000396
POMT1	1,555221	0,000403
SRGAP2	1,069908	0,000405
GSTO2	-1,1609	0,000406
PRRT3-AS1	-4,96134	0,000408
ATP1A3	-1,77301	0,000409
GPX3	10,42365	0,000409
TMEM14C	1,192599	0,000409
ORMDL2	1,700898	0,00041
TMEM254-	3,147194	0,000411
GRB14	1,705775	0,000411
SEC31A	1,265814	0,000412
LCOR	-1,28117	0,000416

GDE1	1,149899	0,000422
DIAPH2	-1,50132	0,000423
BLOC1S3	1,405552	0,000423
CSK	1,482662	0,000424
MGC32805	7,759047	0,000425
AHDC1	-1,37437	0,000427
ZNF100	1,344153	0,000428
APBA3	-1,4078	0,000428
ZFP36	-2,36094	0,000429
TMEM135	-1,50459	0,000432
HLX	-5,99792	0,000435
CEP170	-1,15582	0,000435
UVRAG	1,415553	0,000435
KIAA1804	-1,92788	0,000436
MYD88	1,457596	0,000436
ZNF766	1,171545	0,000436
MAGOH	-1,13801	0,000437
FOXM1	1,071656	0,000437
TMEM221	-3,56153	0,000441
AGBL2	7,570375	0,000442
WDFY1	-1,13084	0,000442
SPX	-6,06185	0,000447
KIAA1841	2,076327	0,000447
SLC52A1	7,759766	0,000447
FGB	5,981685	0,000448
BOP1	-1,01088	0,000448
H3F3AP4	1,180223	0,000448
COASY	-1,05025	0,000448
LINC01098	-7,78901	0,000449
SEC22B	1,212266	0,000449
CD163L1	-3,22018	0,00045
TNKS2	1,301821	0,00045
CAND1	1,079113	0,000454
TXNRD1	-1,44355	0,000455
DNAJC15	1,31981	0,000455
DNASE1L1	1,585317	0,000455
LOC100134	4,465646	0,000456
FAM111B	1,405784	0,000456
EAPP	1,270795	0,00046
BIRC2	-1,63155	0,000461
PM20D2	-1,63996	0,000461
ZNF426	-4,96848	0,000461
ZCCHC7	-1,32453	0,000461
TSPAN31	1,8642	0,000461
C11orf80	1,539044	0,000465
LYPD3	1,805386	0,000466
PDCD11	-1,20714	0,000467
GINS2	1,183061	0,000472
TET1	2,152043	0,000474
BTBD11	-1,70009	0,000476

PPT1	1,272594	0,000481
ADRA2C	1,324482	0,000484
CLSTN1	-1,11953	0,000485
B3GALNT1	1,478649	0,000487
NKX6-1	-2,24822	0,000487
LCMT2	1,5221	0,000488
ZNRF2	1,50141	0,000488
PIP5K1B	6,688401	0,000488
RNF130	1,203287	0,000488
FAM81B	5,211369	0,000489
SIRT5	1,323986	0,00049
XKR8	-1,68945	0,00049
ZC2HC1A	-2,03288	0,00049
FAM178B	5,317604	0,000494
MXD3	1,526651	0,000494
KLHL5	-1,51055	0,000495
COMT	1,603921	0,000497
TMEM92	-3,83539	0,000497
MAK16	-1,35875	0,0005
EHMT2	1,389037	0,000503
ATG13	1,318872	0,000504
ASH1L-AS1	3,183145	0,000506
FKBP14	-1,49876	0,000508
FMN1	2,83861	0,000509
CMTR1	1,135397	0,000509
RPS6KB2	1,204627	0,000517
COX6A1	0,997369	0,000521
SGK1	-1,89882	0,000527
GALNT6	1,504524	0,000527
MYOM2	-7,82138	0,000528
SAXO2	-7,7296	0,000528
MAP3K11	1,578146	0,000529
TBC1D4	-1,98959	0,000529
MAP3K5	-1,99073	0,000531
DPYD	-7,7233	0,000532
RNF145	-1,62178	0,000532
DLG1	1,13687	0,000535
NDUFB2	1,12968	0,000536
MSI2	-1,21928	0,000536
LHX2	-1,96872	0,000537
TFPT	1,818777	0,00054
HLA-DRB5	5,301254	0,000542
ARHGAP24	-2,63245	0,000545
ACACB	1,441445	0,000547
POLD3	1,189763	0,000547
FEZF1-AS1	-7,74739	0,000548
MYL6	1,112655	0,000551
SBF2	-1,84926	0,000554
RNF207	-2,31589	0,000557
ZNF891	7,545464	0,000557

LOC389641	-1,55861	0,000559
PRKD1	7,55984	0,00056
SLC44A5	-6,88423	0,000562
PLEC	-1,65294	0,000568
ATXN7L1	1,527115	0,000568
NBPF11	2,105192	0,00057
DYM	1,228316	0,000572
PPP2R2D	1,146057	0,000576
KRT86	4,023839	0,000577
LYPD5	-6,93532	0,00058
ANGPTL4	-5,68795	0,00058
CSTA	-2,08894	0,000581
CCDC167	1,668751	0,000585
NAA60	1,086328	0,000586
CORIN	-7,7986	0,000586
ENDOG	1,264275	0,000586
PTPN14	-1,85032	0,000588
LAMA5	1,041139	0,000588
CEP85L	-2,16575	0,000593
TCHHL1	8,172988	0,000596
TTC6	7,51459	0,000596
C20orf27	1,031265	0,000596
CHPF2	1,780749	0,000596
IGFL1	8,116563	0,000596
BRD4	1,335718	0,000597
SMKR1	1,773827	0,000599
GLIDR	2,830413	0,000599
SLAMF7	-7,70104	0,000602
LRP8	-1,40892	0,000603
WWP1	1,533205	0,000604
SDCCAG8	-1,79565	0,000604
LRRK2	6,649609	0,000605
AGL	-1,30712	0,000606
CALCR	4,02029	0,000608
C8orf48	7,511556	0,000609
PSMB4	1,075678	0,000617
C21orf59	-1,16055	0,000618
TRIM26	1,11415	0,000618
ARPIN	-2,02245	0,000625
LINC00673	-6,21389	0,000626
FOXA2	5,683235	0,000626
SUV420H1	1,135315	0,00063
SLC16A14	-1,82461	0,000631
NODAL	5,044257	0,000631
TRPM2	-1,39949	0,000631
CALML5	7,569104	0,000631
KIAA1217	2,244667	0,000632
MIR614	-6,88347	0,000632
PPTC7	-1,19078	0,000633
ZNF510	1,543221	0,000633

ZNF268	1,378906	0,000634
KDM2A	1,20125	0,000634
MARCH3	-3,60714	0,000636
CYB5R3	-1,2444	0,000636
FAM46C	2,394627	0,000637
PDIA3	-1,00704	0,000637
ERGIC1	1,146697	0,000637
AGPAT5	-1,28075	0,000648
STX16	1,341112	0,000649
RPL22L1	-1,49503	0,00065
SLC45A3	-1,69467	0,000656
PIP4K2A	-1,13071	0,000656
CYSTM1	-1,32849	0,000656
RSPO4	-5,07233	0,000656
PLXND1	-1,43722	0,000657
CATSPER1	-7,93151	0,000657
IL21R	-3,5792	0,000657
ZC3H4	1,14636	0,000659
IFI6	2,593652	0,000661
TMEM145	-4,38037	0,000661
KCNC3	1,533675	0,000663
PCGF6	-1,66333	0,000665
AHCTF1	-1,20875	0,000667
TBC1D20	-1,13884	0,000667
PSMB9	-3,17268	0,000667
PPAT	-1,3717	0,000668
PLCB1	1,490393	0,000669
CEP85	-1,62786	0,00067
COL4A6	-6,85083	0,000673
DENND3	-2,51937	0,000674
SEL1L	1,168251	0,000677
SLC29A2	1,451889	0,000677
AATBC	7,475633	0,00068
TYW3	-1,40938	0,00068
STK38	1,189965	0,00068
LAMTOR1	1,130218	0,000681
PPM1K	-2,58901	0,000682
EPB41L4A	-2,39193	0,000682
PFKFB3	-1,52106	0,000684
DLD	1,313985	0,000684
PCCB	1,028536	0,000693
PLCXD2	-2,93917	0,000693
CAPN9	7,494649	0,000694
SLC25A37	-1,34766	0,000694
OAS2	5,060692	0,000694
TYMS	-1,3858	0,000694
ZNF519	1,999769	0,000694
PHOSPHO1	-3,08299	0,000695
AOC2	-3,08314	0,000698
EPB41L5	1,57625	0,000699

SNAR-E	5,798747	0,000703
INSIG1	1,699162	0,000703
VPS37A	-1,13657	0,000703
BEX5	7,63965	0,000703
ITGA1	-6,83008	0,000704
MOB3B	-4,70064	0,000705
TMEM134	1,414956	0,000707
LPPR3	7,728433	0,000713
PRKAR2A	-1,74214	0,000713
LOC441666	7,479079	0,000723
ENPP4	-1,57999	0,000723
A4GALT	-2,31884	0,000724
ZNF114	1,549986	0,000729
PLXNB1	1,381617	0,000729
ING4	2,662632	0,00073
TAPT1-AS1	3,96002	0,00073
DIRAS3	-11,5853	0,00073
LINC00880	-7,68517	0,000731
SERPINF2	-4,09541	0,000732
SSBP2	2,650005	0,000732
VAX2	4,18439	0,000732
GAREM	-1,47607	0,000734
CACNA1A	-7,68835	0,000734
FAR2P1	2,869039	0,00074
SLC43A2	1,41194	0,00074
ZSWIM4	1,811731	0,00074
MAP7D1	-1,21277	0,000743
YBX3	-1,01992	0,000744
CTR9	-1,64224	0,000745
WNT10B	-1,55609	0,000745
C2orf54	4,350308	0,000747
SYCE1L	-4,45576	0,000747
UNC5C	5,834953	0,000747
CCDC53	1,478133	0,000755
SUCLG1	1,054226	0,000757
ATP2B1	-1,26251	0,000762
ZKSCAN2	-1,2509	0,000762
PTPN18	1,111526	0,000762
NR6A1	-1,80636	0,000762
SNHG3	-1,43402	0,000762
BTNL9	-3,37271	0,000763
RBM15B	1,065071	0,000763
JAM2	4,038849	0,000766
CDC37L1	-1,30824	0,000767
C3orf80	-3,7604	0,000771
KANSL1L	1,701884	0,000776
PEX11B	1,620089	0,000779
MRC2	-2,28708	0,000781
ARIH2	-1,04358	0,000783
GATSL2	2,207468	0,000783

WWC2	-1,23601	0,000787
SHF	2,045725	0,000787
ZNF394	1,561077	0,000793
TMEM87B	1,338885	0,000795
HOPX	7,657735	0,000797
ACTB	1,302973	0,000797
MRPS11	1,291088	0,000801
CSF2RA	-7,78772	0,000803
ATP6V1B1-	-7,61677	0,000803
TRABD2A	-4,00359	0,000803
PDGFB	-2,0261	0,000809
PROSC	-1,11548	0,000809
FHL3	-1,50711	0,000809
KCTD17	-1,81634	0,00081
AZIN2	-3,21359	0,00081
ARHGAP12	-1,28644	0,00081
PBK	-1,34743	0,00081
GBE1	-1,26305	0,000811
BBS7	-1,28515	0,000818
SGCG	7,567556	0,000818
VGLL4	1,051728	0,000818
ZNF709	5,624394	0,000819
ARC	-2,62213	0,00082
NDUFS8	1,026979	0,000822
DYNLL1	1,001261	0,000826
ZNF627	1,336576	0,000829
YPEL1	4,013251	0,000833
C7orf26	1,078111	0,000835
OPN3	1,836421	0,000836
LAMTOR4	1,12251	0,000838
C2CD4D	6,560771	0,000839
LOC101928	-4,41704	0,00084
P2RY1	-5,76046	0,000843
PPP4R3A	1,147217	0,000847
OXGR1	7,458291	0,00085
CD96	-6,76752	0,00085
GPR63	-2,30801	0,000851
JHDM1D-A	2,800448	0,000852
EPC1	1,122748	0,000853
TMEM254	1,508633	0,000854
IFI27	3,827664	0,000859
RFXANK	1,219726	0,000862
SPRY2	-2,05758	0,000864
SERPINH1	1,255795	0,000864
YIPF6	-1,14464	0,000868
DDX52	-1,1322	0,000869
KIF13B	-1,96708	0,000869
TRMT61A	1,334256	0,000872
VAPB	1,116814	0,000874
RUFY1	1,147307	0,000881

CERS1	4,507423	0,000883
JUP	1,505871	0,000887
FBXO41	-1,51528	0,000887
PPIEL	-5,74967	0,000893
LYSMD2	1,574008	0,000893
C10orf10	-5,73823	0,000895
PNMA6A	7,441092	0,000897
CDK7	-1,06226	0,000899
ZNF74	1,27061	0,000905
LINC00885	7,659283	0,000905
PLA2G4F	4,076638	0,000907
PCDHGA4	6,513609	0,000908
IFT46	1,502763	0,000909
IBSP	7,394631	0,000909
SCD5	-1,31161	0,000927
FER	-1,22687	0,000927
TICAM1	-1,20187	0,000928
SYMPK	-1,0869	0,000928
CCDC88C	1,451313	0,000931
RN7SK	3,335847	0,000933
PIGB	1,632087	0,000939
NDUFA12	1,181625	0,00094
UGGT2	-1,54288	0,000941
BMP8A	-2,61174	0,000941
SAMD15	2,641378	0,000941
ITPR1	-1,67223	0,000943
GUSBP4	2,166702	0,000944
KANK3	-3,20751	0,000946
PITPNM2	-1,50203	0,000954
STX2	-1,49741	0,000955
PAGR1	1,05042	0,000957
RTFDC1	1,083233	0,00096
CACHD1	-1,90027	0,000961
TM4SF18	-7,68446	0,000965
ATF4	-1,30942	0,000966
TBCCD1	-1,29633	0,000969
MLF1	-1,42152	0,000973
CLCN3	-1,22773	0,000975
ATMIN	1,064958	0,000976
NBN	0,999804	0,000978
OSER1	1,148199	0,000981
TARBP2	1,093676	0,000988
PKN2	-1,18313	0,000991
P2RX4	1,685863	0,000992
FAM193B	1,43879	0,000992
NME1	-1,05327	0,000994
TBL1XR1	-1,21948	0,000996
LDHB	-10,0745	0,001001
KCNK3	-5,87716	0,001001
GDPD1	-2,00763	0,001004

FAM149A	-3,45221	0,001004
ONECUT1	2,994159	0,001004
KDM5D	-7,62681	0,001005
PPIE	-1,05426	0,001005
PPP2R5B	-1,8832	0,00101

RFX393

Gene	log2FoldCh	FDR
APCDD1	15,97544	1,65E-26
CDH1	15,9393	3,15E-26
MYH14	15,05027	1,28E-22
GATA3	14,91681	4,62E-23
FBP1	14,63437	3,39E-22
HID1	14,26106	2,81E-20
TSPYL5	14,09069	7,90E-21
GRHL2	13,78605	1,45E-19
KCTD15	13,7853	9,10E-19
ACSS3	13,6889	1,17E-19
IGFBP2	13,51841	4,21E-19
SPDEF	13,51532	3,34E-10
EPN3	13,43565	9,37E-19
TJP3	13,24836	3,01E-17
TMEM30B	12,95434	1,73E-17
ATAD1	12,91144	1,58E-17
MSI1	12,87385	2,84E-17
DSCAM-AS	12,80709	5,38E-10
MSX2	12,65199	1,96E-16
GAL	12,56971	1,32E-16
SLC27A2	12,56716	1,62E-16
CDH3	12,45552	1,28E-45
GGT6	12,43375	5,45E-15
PCDHA6	12,38586	4,35E-16
PPM1E	12,28458	1,73E-09
LINGO1	12,25237	1,08E-15
ADAMTS19	12,24773	1,05E-15
PRR15L	12,24731	2,82E-15
IRX2	12,16643	1,75E-15
FXYD3	12,11972	2,37E-08
ALDH3B2	12,07213	3,28E-14
SPTSSB	12,05193	5,33E-14
EMB	11,97689	4,89E-15
WNK2	11,96096	7,86E-15
POF1B	11,92583	5,29E-14
CACNG4	11,88115	1,09E-14
PCDHA11	11,76471	3,32E-14
PCDHB2	11,74461	3,28E-14
TMEM150C	11,73412	3,75E-14
CBFA2T3	11,72536	2,22E-14
RBBP8NL	11,69948	4,07E-14
TNFAIP8L3	11,66176	2,85E-13
DLX5	11,65328	3,91E-14
GATS	11,64457	3,63E-13
LIN7A	11,64058	6,13E-14
C15orf59	11,63864	2,32E-13
PTP4A3	11,61437	4,42E-14
CCDC170	11,57212	9,57E-13

SLC1A2	11,56487	6,59E-14
S100A14	11,54958	2,36E-10
ADCY1	11,53153	8,79E-14
SULT2B1	11,52374	1,33E-13
PCSK1N	11,50922	8,09E-14
OLFM1	11,50058	8,05E-14
GLB1L2	11,48892	8,86E-14
LRFN4	11,46212	2,97E-16
HENMT1	11,41139	1,40E-13
ZIC1	11,34805	5,27E-13
CRISPLD1	11,32855	1,34E-12
NCAM2	11,30336	8,72E-13
ESPN	11,30165	3,23E-12
ALOX15	11,27582	9,27E-12
TMEM45B	11,25578	3,36E-13
AR	11,23266	3,93E-13
ZNF569	11,19733	9,65E-13
ERBB3	11,16316	2,52E-36
SMIM22	11,16211	8,83E-13
EPHA1	11,13204	1,03E-12
MNX1	11,11334	8,21E-13
ASCL2	11,03969	4,88E-12
C5orf38	11,03155	2,22E-12
PCP4	11,02888	1,55E-12
MNX1-AS1	10,994	4,10E-12
ASCL4	10,98128	2,02E-12
NUP210	10,91268	1,82E-65
PACSIN1	10,89891	1,47E-11
ENTPD2	10,89427	8,15E-12
RNF43	10,88282	9,15E-11
ARHGDIG	10,8827	2,96E-12
NUP210L	10,85694	3,72E-12
EFS	10,85139	4,00E-12
DLX3	10,84673	6,00E-14
TDRD1	10,8345	3,82E-12
ST8SIA4	10,83418	6,28E-11
SBK1	10,82154	1,98E-23
ARHGEF26	10,79318	5,01E-12
RDH16	10,78598	6,73E-12
KRT8	10,77064	6,07E-188
RANBP17	10,7565	1,00E-11
CACNA1D	10,71404	7,49E-12
MED12L	10,68748	2,61E-11
HS6ST2	10,6606	1,75E-13
PGR	10,64303	5,36E-10
CST1	10,63197	1,52E-11
C5AR2	10,62868	6,39E-11
FAM155B	10,6137	1,46E-11
ZNF793	10,59621	1,67E-11
FAM3B	10,59103	1,56E-11

MPDZ	10,55379	3,25E-11
NAALADL2	10,53386	3,17E-11
LAD1	10,51462	1,72E-48
VHL	10,40108	7,51E-30
SPESP1	10,40089	8,06E-11
TMEM125	10,33577	6,63E-11
HES2	10,32485	3,11E-09
ODAM	10,25357	1,05E-10
CEACAM21	10,24467	1,22E-10
PCDHB3	10,22133	1,32E-12
TDRD5	10,21998	1,93E-10
HDHD1	10,2152	1,97E-10
TSPEAR	10,21316	3,33E-10
MYRIP	10,21051	3,13E-10
C9orf152	10,21046	2,48E-12
RTN4RL1	10,1929	6,41E-21
CHGA	10,17584	1,59E-10
EFNB3	10,17486	1,66E-10
DPYSL4	10,15114	5,69E-10
SLC9B2	10,14817	2,25E-10
PCDHA10	10,12635	2,83E-10
ABCG1	10,10235	1,58E-12
KCNJ11	10,10148	7,06E-10
CASC9	10,05205	3,22E-10
NYNRIN	10,04999	2,37E-08
RAB25	10,03756	6,93E-45
FREM2	10,02204	3,32E-91
LRRC26	10,01228	4,02E-10
AARD	9,996854	4,47E-10
EPHX2	9,991915	8,17E-10
NFE2	9,987011	9,13E-10
PCDHA12	9,984583	7,48E-10
MYO5C	9,98282	1,77E-65
CHRM1	9,97669	2,12E-09
ZNF570	9,976164	5,10E-10
HIST3H2A	9,9669	5,20E-10
SIDT1	9,966327	6,43E-10
HLA-DQB1	9,957333	2,71E-08
PTEN	9,948171	3,42E-51
RNF223	9,942623	7,45E-10
KRTAP3-1	9,883615	4,20E-09
PRR15	9,879456	1,26E-09
PRRG2	9,878964	1,81E-09
C18orf63	9,852165	1,37E-09
RERG	9,843537	1,03E-09
SALL4	9,829514	2,07E-09
STS	9,823508	2,25E-09
OVOL2	9,7665	2,49E-08
ERBB4	9,765947	2,49E-09
MLXIPL	9,76218	2,62E-09

ZNF732	9,701412	4,56E-09
ZNF790-AS	9,681709	2,79E-09
RUNDC3A-/	9,676425	1,80E-08
NOTCH3	9,668522	1,03E-124
ASCL1	9,665095	6,03E-09
ADGRL2	9,641219	3,64E-09
CDKN2B	9,623516	6,26E-09
FAR2P1	9,595467	3,13E-08
ELF5	9,577022	4,20E-08
DEGS2	9,576988	5,88E-09
GNB4	9,552122	5,15E-09
STXBP6	9,540514	5,71E-09
ZNF818P	9,531062	5,85E-09
SYT3	9,519156	2,69E-08
ZNF704	9,502241	1,65E-24
CHST8	9,486553	7,44E-09
SYT17	9,480654	5,51E-11
S100P	9,454517	1,29E-06
PCSK9	9,450355	1,15E-07
NOVA1	9,44733	1,56E-08
TMPRSS13	9,42122	1,40E-10
JPH3	9,407293	1,16E-08
KLLN	9,401508	9,10E-08
VIPR1	9,366299	1,17E-07
MAPK4	9,358247	2,60E-07
TUBA3E	9,351443	2,87E-08
ZG16B	9,331266	1,74E-07
SLC16A14	9,32614	4,19E-08
NFATC4	9,323664	2,00E-08
ENPP5	9,316832	3,87E-08
FSD1	9,308084	2,28E-08
C10orf82	9,307468	3,27E-08
MUC5B	9,300814	2,16E-08
DPYSL5	9,293799	2,63E-10
CBLC	9,287005	2,87E-08
PKP2	9,286406	7,71E-21
TEX19	9,28594	2,72E-08
SPINK5	9,281785	1,19E-07
DOCK8	9,273276	2,56E-08
TYRP1	9,26941	2,69E-08
ERC2	9,255166	2,63E-08
SLC47A1	9,237772	4,41E-08
MYT1	9,23213	3,04E-08
MANSC4	9,231071	3,50E-06
TFF1	9,224533	8,98E-53
MB	9,222074	2,69E-10
GPR158	9,215829	3,59E-08
IRX4	9,20963	6,87E-08
FAM19A5	9,193827	6,41E-08
ICA1	9,187911	1,94E-35

GDPD1	9,18196	9,03E-08
PCDHGB5	9,154395	4,52E-08
PBX1	9,148541	1,09E-22
LOC148709	9,145146	5,05E-08
EOMES	9,138573	5,47E-08
DDX43	9,123634	7,62E-08
AQP3	9,123612	1,76E-05
GPR27	9,116953	7,29E-08
VPS9D1-AS	9,108391	4,75E-06
GYLTL1B	9,106885	1,63E-34
C2orf54	9,063702	8,88E-07
PLA2G4F	9,062513	3,92E-06
KCNK5	9,061931	3,42E-07
LRRC3	9,050911	9,59E-08
LMX1B	9,04411	7,40E-10
TC2N	9,042076	1,12E-31
EGR3	9,038839	1,85E-06
KLRG2	9,029619	1,19E-07
ACTL8	9,01931	2,01E-07
ZNF730	8,97477	1,38E-07
SELENBP1	8,97377	4,06E-33
LYPD6B	8,967811	1,37E-07
C17orf104	8,947897	7,17E-07
EMID1	8,937782	1,65E-07
LOC100505	8,932349	2,24E-07
LRP3	8,922783	4,01E-12
HBA1	8,920295	3,84E-07
ONECUT1	8,901736	4,20E-07
LGR6	8,895304	2,87E-07
CA8	8,894837	2,97E-07
BSPRY	8,88377	2,90E-32
COL4A5	8,882935	4,61E-07
KIAA1324L	8,877359	1,01E-27
CNGB3	8,869793	3,02E-07
LOC339862	8,863963	2,49E-07
LRRN1	8,85573	3,02E-07
SFMBT2	8,850203	4,05E-07
RIC3	8,843573	4,28E-07
GALNT16	8,831498	9,16E-05
PCDHB4	8,827213	1,37E-06
PALM3	8,822815	2,78E-07
FSTL4	8,809996	5,71E-07
BLNK	8,802171	1,02E-05
DNAH3	8,793318	4,92E-07
LINC00992	8,793057	3,64E-07
CXXC4	8,791314	4,41E-07
NRG3	8,782026	6,01E-07
ZNF467	8,768198	1,87E-44
PCAT7	8,766369	3,65E-07
TUB	8,759854	3,78E-07

PTCHD2	8,759753	2,01E-06
GNG13	8,747119	1,16E-06
C1orf210	8,735275	8,31E-07
LINC00858	8,718416	1,26E-06
GSG1L	8,713328	5,24E-07
KLHDC9	8,705142	5,81E-07
PGM5	8,704737	8,23E-07
H2AFY2	8,704398	4,25E-50
FAM110C	8,698379	3,11E-06
SLC22A17	8,696749	5,80E-07
CRABP1	8,688865	1,84E-06
ZMAT1	8,6844	1,76E-05
SPSB4	8,678932	1,34E-06
LINC01132	8,67671	3,48E-06
FAM83B	8,66518	6,36E-21
ESR1	8,664982	0,000227
SOWAHB	8,660432	7,81E-07
GRIK3	8,649132	3,38E-06
HOXC13-AS	8,636514	9,77E-07
LOC100996	8,636243	9,78E-07
S100A9	8,632312	0,000146
TNFRSF18	8,622678	6,42E-06
HTR2C	8,621764	8,11E-07
SLC2A4	8,596062	1,01E-06
CNNM1	8,587668	9,39E-07
RADIL	8,584878	3,78E-06
PCDHA5	8,583658	9,98E-07
NPDC1	8,581063	3,46E-12
LRP2	8,578604	9,36E-05
CERS6	8,574557	4,48E-55
LOC100288	8,573547	1,23E-06
KCNMB2-A	8,571721	1,02E-06
TERT	8,568882	4,44E-06
SNCAIP	8,566004	1,06E-06
KCNV1	8,565895	3,74E-06
PLA2G3	8,565589	7,95E-06
METTL7A	8,559011	4,24E-06
ACE	8,537557	5,95E-06
SERPINB5	8,534566	2,74E-05
TMPRSS4	8,524818	2,24E-06
INPP5D	8,509441	0,000109
MPPED2	8,507409	5,57E-06
EDAR	8,502445	5,53E-06
TPTE	8,501247	1,71E-06
LOC644919	8,487253	2,44E-06
ANXA9	8,484291	9,08E-17
FLT4	8,4839	5,93E-06
DSCAM	8,482368	4,49E-06
ADAMTS19	8,481411	1,72E-06
RAB11FIP4	8,480488	9,02E-37

TENM1	8,460122	3,34E-06
STMND1	8,458081	2,03E-06
PVRL4	8,450129	3,87E-19
MARK1	8,448374	2,26E-05
C19orf81	8,431728	2,16E-06
KRT23	8,427986	2,17E-06
SEMA3D	8,426853	7,35E-06
LINC00664	8,423848	4,80E-06
FRMPD1	8,417716	2,67E-06
CCDC83	8,416124	3,84E-06
PCDHA13	8,414683	2,47E-06
KCNN2	8,407571	2,40E-06
C4orf19	8,392872	3,20E-06
ADAMTSL5	8,375062	3,23E-06
TMEM139	8,357632	6,45E-06
GOLT1A	8,353735	3,07E-08
SPATA17	8,325901	2,88E-13
DACH1	8,321702	3,74E-06
LOC72873	8,321603	4,19E-06
FAM81B	8,29645	6,14E-06
TLE2	8,282682	1,90E-28
SUSD2	8,279066	1,77E-09
ZIC4	8,267616	5,05E-06
AK7	8,261966	3,15E-05
PCDH19	8,255853	1,17E-05
VAX2	8,254919	6,46E-06
PDZRN3	8,252642	5,31E-06
MYO7A	8,246231	5,49E-06
FAM83E	8,237969	1,43E-05
CYP1A1	8,229605	5,86E-10
LOC10012	8,218728	6,42E-06
GLS2	8,209653	6,43E-08
RFTN2	8,207418	1,19E-05
WNT6	8,1951	7,05E-06
C12orf56	8,190084	7,89E-08
TMEM132	8,186127	0,000374
RAI2	8,184203	1,44E-05
EDA	8,163528	8,29E-06
SESN3	8,161599	4,21E-11
TTC29	8,16056	8,96E-06
MPP2	8,154055	3,19E-07
SLC22A11	8,133476	4,48E-05
PVALB	8,128652	3,07E-05
ATP2C2	8,127678	2,31E-37
CES3	8,125198	1,32E-05
XKRX	8,123033	2,11E-05
TCHHL1	8,121114	0,000558
NMUR2	8,117267	1,08E-05
PCDH18	8,116304	3,29E-18
ITGA2B	8,109182	4,46E-07

FAM95C	8,108635	1,17E-05
ACBD7	8,108509	2,25E-12
ZNF613	8,075791	1,34E-05
IGFL1	8,064333	0,000539
SNAR-E	8,057571	3,37E-05
KIAA0319	8,057478	2,85E-05
NODAL	8,045129	2,81E-05
WIF1	8,034056	0,000108
LYPD6	8,031415	1,83E-07
RIPPLY3	8,030678	1,70E-05
LOC102723	8,028991	1,78E-05
SLC6A14	8,021629	1,84E-05
KCNH3	8,019082	1,76E-05
PRLR	8,002006	4,00E-32
FAM196A	7,997608	6,90E-05
SCD5	7,996059	2,16E-16
TRPM2-AS	7,98075	2,04E-05
ZNF98	7,970014	4,37E-05
HIST1H2BH	7,964349	2,47E-05
LINC00649	7,95993	2,31E-05
MYO15B	7,952081	2,59E-05
HS6ST3	7,946601	2,79E-05
CKMT1A	7,945939	6,97E-21
PCDHB6	7,932089	4,20E-07
TARP	7,929655	0,000127
F7	7,924704	0,000112
TBC1D30	7,919083	3,39E-65
ATP2A3	7,91755	6,46E-06
KLHL35	7,904701	3,34E-05
FAR2	7,873114	1,15E-24
PART1	7,870717	4,40E-05
SLC16A10	7,867742	3,56E-05
HOXC13	7,864382	5,23E-15
NKX6-1	7,861121	4,21E-05
LOC100996	7,857308	4,90E-05
PRR36	7,853714	3,61E-116
FLT3	7,843298	3,98E-05
ZNF492	7,841094	1,85E-06
TRPV6	7,839458	4,26E-05
FAM117A	7,831434	2,05E-19
MIR4458H(	7,815645	5,06E-05
MPZL2	7,806334	3,38E-10
FOXI3	7,803027	5,46E-05
PLEKHB1	7,783899	5,57E-10
HIST1H3G	7,760431	6,28E-05
GHR	7,758397	6,12E-05
CDH18	7,755186	7,27E-05
IGSF11	7,739097	6,44E-05
TTYH1	7,732668	0,00029
XKR7	7,706455	0,000146

LOC100499	7,703927	8,30E-05
KIF1A	7,701857	2,95E-32
SLC52A1	7,701503	0,000268
MGC32805	7,700419	0,00025
NPY1R	7,696399	9,07E-23
STK31	7,686371	0,000121
GATA3-AS1	7,679904	9,90E-05
LAMC3	7,635508	0,000107
PCDHB13	7,635282	2,29E-14
TMEM105	7,631475	0,000183
LOC440461	7,621685	0,000252
ARL11	7,619124	0,000124
ICOS	7,616717	0,000125
MIR4720	7,616464	0,000498
NRARP	7,610288	2,50E-06
EMCN	7,605789	0,000137
BMP7	7,605634	3,15E-206
PCDHAC1	7,604953	0,000248
LINC00885	7,603152	0,000614
POTEF	7,590039	0,000145
EWSAT1	7,588461	0,00023
IGSF1	7,587363	0,000638
KCNF1	7,582982	0,000159
GSC	7,577927	0,00045
ASAH2	7,575202	8,09E-15
LRRC10B	7,571337	0,000273
NXPH3	7,56973	0,000232
DLG2	7,568322	0,000143
ATP8A1	7,565042	0,000195
GPM6B	7,54746	0,000154
KCNU1	7,540378	0,000174
TFF3	7,533683	1,04E-09
TDGF1	7,526115	0,000981
ALG1L	7,523868	3,19E-16
SEPP1	7,519956	0,000633
CYP4F22	7,51708	0,000237
VAV3	7,512661	1,31E-44
GJB5	7,508207	0,000928
CALML5	7,507674	0,000327
CCSER1	7,503569	0,000234
NRK	7,503225	0,000658
CACNA2D2	7,485417	0,000269
TLX1NB	7,483169	0,000206
ZNF853	7,482133	0,000255
UCP2	7,482108	3,69E-12
CD36	7,461541	0,000474
MISP	7,458585	1,78E-30
NDNF	7,449916	0,000238
MARVELD3	7,449304	4,23E-24
ASIC1	7,447499	1,87E-08

C8orf48	7,446323	0,000278
ARHGEF38	7,435964	0,00033
CAPN9	7,42082	0,000332
LOC441666	7,414795	0,000343
DEFB126	7,41459	0,000278
VANGL2	7,408307	0,000302
SNAR-G1	7,399427	0,00039
OXGR1	7,383426	0,000426
PKIB	7,383362	3,30E-54
NPTX1	7,378993	0,000831
RIMS4	7,373767	1,30E-13
CELF3	7,368203	0,000518
ADIRF	7,339199	0,000436
WNT9A	7,332159	0,000605
IBSP	7,323665	0,000416
SLC5A5	7,321496	0,000896
GRID2IP	7,312824	0,000424
IGSF5	7,310567	8,26E-06
CEACAM5	7,303705	0,001005
KCNJ4	7,301908	0,000485
OVOL1	7,30019	1,65E-16
URAHP	7,297494	0,000536
PARM1	7,293774	0,000674
ZNF350	7,292806	5,45E-09
TMC5	7,288107	2,56E-12
NAP1L2	7,281376	0,000931
RGMA	7,274685	5,94E-15
C19orf45	7,272505	0,000499
PLIN5	7,26845	0,000614
CA4	7,258167	0,000656
SRRM2-AS1	7,255796	0,000791
GRTP1	7,248506	2,05E-18
MYH10	7,239945	3,66E-06
TIMP3	7,236697	8,33E-09
PDE9A	7,219836	1,27E-05
PRKG2	7,210574	0,000652
NPW	7,204505	1,13E-05
HUNK	7,199516	7,61E-31
ZNF385C	7,183095	1,25E-05
NMNAT3	7,177669	0,000957
KDF1	7,15215	4,89E-23
KIAA1257	7,147947	2,08E-05
ZNF793-AS	7,136727	0,000861
RAP1GAP2	7,135852	5,02E-35
EVPL	7,119684	8,46E-68
FA2H	7,082957	5,54E-09
SLC9A4	7,05969	2,05E-07
YBX2	7,056466	2,27E-66
WNT11	7,023685	2,16E-10
MYLIP	7,016305	3,24E-42

DEF6	7,008592	1,49E-11
MUC3A	7,002574	3,70E-05
SYNE4	6,991769	5,37E-36
PPL	6,981754	6,31E-59
TMEM254-	6,975341	1,75E-07
KRT19	6,963958	4,10E-86
CLDN3	6,960838	7,19E-62
SCN4A	6,957427	5,69E-12
CLDN7	6,953666	5,04E-115
IL17RB	6,895841	3,40E-10
SHF	6,85221	3,62E-10
PLEKHG6	6,848796	7,65E-14
RAB26	6,831142	3,18E-10
FRAT1	6,826997	6,35E-05
FIRRE	6,814962	1,16E-06
TUBA3D	6,806951	5,46E-07
FAM83G	6,80323	1,65E-33
NAT8L	6,793479	4,11E-31
PALM	6,778632	3,36E-21
PADI2	6,76915	2,01E-07
MAPT	6,762347	2,12E-36
UPK2	6,738369	4,21E-07
UNC5C	6,730906	0,000278
KCNQ5	6,700083	0,000214
ADSSL1	6,694036	1,30E-06
FCGR1A	6,684635	6,99E-06
NRXN2	6,684038	2,08E-18
PRSS22	6,668443	7,10E-07
MACC1	6,652126	1,88E-07
PDE3B	6,6488	5,13E-14
CRLF1	6,623011	4,88E-07
DBNDD1	6,622369	1,08E-37
LDHD	6,62224	4,71E-06
PCDHB8	6,61743	2,15E-21
B3GNT3	6,60778	3,37E-13
RORC	6,575663	0,000232
HIST1H2AG	6,569431	0,0002
PRSS8	6,567871	3,72E-111
C2orf15	6,564041	1,13E-21
DLGAP3	6,562335	1,15E-11
KIAA1324	6,553657	1,50E-25
FAM46C	6,551078	2,63E-13
HPN	6,53984	1,19E-16
ESRP1	6,500024	1,04E-107
ENPP4	6,484821	1,89E-11
AMIGO1	6,460151	1,02E-06
GLDN	6,446814	0,000329
TFAP2C	6,429285	8,06E-71
IGSF9	6,423122	3,43E-98
SLC22A31	6,422454	1,59E-06

RASAL1	6,402642	7,24E-06
ITGB4	6,402459	3,87E-87
KCNJ8	6,401005	4,08E-48
SLAIN1	6,400853	5,35E-10
COLCA1	6,398284	0,000438
HPDL	6,36353	2,48E-12
GPX2	6,35236	0,000433
RNF150	6,337871	5,60E-06
EPHB3	6,335718	1,23E-22
CALCR	6,290504	1,14E-05
GPC2	6,285325	6,04E-11
C2CD4C	6,274163	0,000834
FUT9	6,260379	5,44E-08
FCHO1	6,244449	2,10E-68
CD8A	6,243818	6,32E-08
KCNJ3	6,209144	5,82E-08
MYB	6,200487	2,50E-09
STK26	6,174214	1,01E-47
HTR6	6,148404	0,000885
SLC7A8	6,137788	4,92E-22
RHOV	6,137754	1,10E-23
SFN	6,131731	3,36E-61
GRHL3	6,130752	4,10E-26
CACNA1H	6,120562	4,96E-55
LRRC37A8P	6,100995	1,08E-05
LDOC1	6,072222	5,29E-75
GSTO2	6,061528	6,25E-37
GREB1	6,054238	9,36E-35
RASGRF1	6,048602	4,17E-25
FAM174B	6,039983	1,26E-20
DLX4	6,019329	1,23E-05
FGD3	6,015655	4,43E-17
ST14	6,00813	2,70E-63
EPPK1	5,992505	1,97E-26
CALHM2	5,98076	3,28E-15
FBXO15	5,958603	2,60E-05
SLC30A3	5,956975	3,66E-05
MYO1D	5,927141	7,10E-49
LYPD3	5,92403	6,39E-23
TACSTD2	5,900003	3,92E-75
CKMT1B	5,894785	4,55E-29
TEX15	5,872006	4,93E-09
SYT7	5,843926	5,41E-101
ALDH3A1	5,782161	0,000184
ACTG2	5,763303	8,44E-06
SLC27A5	5,757769	3,87E-32
WDR72	5,743129	1,40E-19
HOXD12	5,738307	0,000887
SYT1	5,726794	2,69E-20
CNTNAP2	5,719705	1,23E-10

LINC00052	5,719585	8,73E-09
JUP	5,705703	4,95E-48
ENTPD1	5,678805	2,29E-11
PLD6	5,673847	3,17E-20
CADM4	5,638491	1,73E-31
AIF1L	5,635415	2,71E-86
C2CD4D	5,632863	0,000329
TM7SF2	5,628177	5,36E-35
ADRA2C	5,621313	2,07E-37
TIAM1	5,619338	8,64E-07
ALDH6A1	5,619078	7,69E-42
PROM2	5,602315	7,56E-24
NSUN7	5,594387	1,87E-11
FAM171A2	5,578442	6,49E-45
SLC9A2	5,568993	2,45E-68
MATK	5,562463	1,82E-57
C1orf115	5,547056	2,40E-15
HLA-DRB5	5,543358	0,00039
LLGL2	5,538	3,33E-92
RSPH1	5,515934	1,32E-05
SYTL4	5,513617	1,12E-05
FAM83H-A	5,499665	5,32E-54
BCAS3	5,498152	8,67E-76
PCDHA7	5,46731	2,93E-07
SLC15A2	5,465479	7,51E-05
BGN	5,427068	0,000132
KRT18	5,42308	6,89E-48
TUBD1	5,416469	4,88E-45
TMC4	5,412348	7,29E-29
GATSL2	5,407112	4,60E-60
TOX2	5,384028	3,81E-11
LOC11323(	5,383386	7,07E-23
CCDC3	5,378616	0,001003
TEX14	5,374436	0,000322
FOXO4	5,366722	0,000513
ZFP14	5,36657	4,39E-16
ZNF711	5,361319	1,46E-18
GAMT	5,352391	1,41E-25
ZSWIM5	5,350294	1,61E-07
NEURL1B	5,344856	4,39E-24
ABAT	5,337216	4,52E-14
SEMA6B	5,315315	1,10E-17
LRRC73	5,313704	2,46E-09
UNC5A	5,313405	0,000813
CBX2	5,294903	5,74E-100
TINCR	5,286307	4,98E-13
ADGRB1	5,279603	9,76E-24
REEP6	5,264875	4,08E-41
ELOVL4	5,264289	0,000313
CHDH	5,26392	6,60E-14

KCNC3	5,25792	2,87E-33
NKD1	5,250168	3,40E-59
GNG7	5,242476	1,40E-07
ATP1A3	5,200715	6,61E-18
MAP2K6	5,200068	2,27E-09
CGN	5,188384	1,56E-56
FZD9	5,18477	1,02E-05
TFAP2A	5,17402	4,02E-131
SYTL1	5,170581	1,26E-26
ADGRL1	5,13893	4,49E-105
MALRD1	5,1299	4,52E-05
FLRT3	5,12521	3,21E-41
KYNU	5,118848	6,46E-23
VAV1	5,112282	8,27E-11
MBNL3	5,089653	1,94E-56
GPLD1	5,082525	2,06E-05
BCL11B	5,068808	5,28E-48
SP6	5,06322	1,24E-32
TMEM52	5,049082	7,26E-08
PPM1D	5,043954	6,33E-90
TMEM121	5,028557	1,56E-06
ADGRV1	5,011908	6,55E-05
LINC01006	5,009341	0,000114
B3GALT4	5,009023	1,85E-05
C1orf226	5,001692	1,27E-07
ETNK2	4,99316	4,35E-27
LOC28333	4,9891	7,06E-13
AKR1E2	4,978732	8,77E-08
KRT86	4,966228	0,000316
RTN1	4,952543	0,000827
RAPGEF5	4,949417	0,001006
CDC42BPG	4,945263	4,98E-19
ADAM22	4,943616	3,50E-12
RPRM	4,926919	1,39E-05
HCN4	4,909942	1,89E-07
CEACAM1	4,907804	2,95E-12
ATP6V0E2	4,898355	3,58E-42
LMO2	4,874094	0,000486
C17orf97	4,866706	7,47E-17
FGFR3	4,860981	1,36E-17
RCN3	4,857763	0,000301
CACFD1	4,853187	2,88E-13
B4GALNT4	4,849504	1,51E-24
DHRS2	4,847815	5,12E-23
METTL7B	4,843293	0,000104
RAB17	4,842643	2,81E-14
KLHL13	4,839232	1,16E-08
FAM178B	4,82877	0,000904
HSD11B2	4,827987	7,14E-11
TMEM229	4,818356	4,36E-13

ILDR1	4,814599	3,99E-21
EPCAM	4,810609	1,19E-102
FDXR	4,79753	7,03E-18
KREMEN2	4,793282	2,21E-54
FAM222A	4,779299	5,53E-42
COBL	4,750235	2,77E-20
BATF	4,722619	7,86E-09
SOX2	4,715705	9,30E-13
AKR1C2	4,710315	5,69E-09
SLC45A4	4,704796	3,72E-18
DYNC1I1	4,704233	1,57E-31
MEST	4,703507	1,10E-107
TLCD1	4,69639	1,06E-52
SHH	4,685144	1,29E-06
AP4B1-AS1	4,682127	2,45E-13
SLC7A2	4,674546	1,11E-33
MEX3A	4,671064	2,80E-121
C1orf233	4,663559	1,47E-40
NOTUM	4,645438	7,20E-13
SLC4A8	4,643012	3,09E-08
EEF1A2	4,626863	1,99E-54
RHPN1	4,61842	1,66E-32
BRIP1	4,587243	8,39E-70
JAG2	4,568022	2,19E-29
CNIH2	4,565897	8,49E-15
DNAAF3	4,556084	4,92E-22
FLJ10038	4,551074	2,39E-18
TESC	4,545337	0,000782
ZCCHC12	4,544474	5,97E-06
NEB	4,540066	1,46E-32
CLMN	4,533954	1,58E-25
MARC1	4,520978	1,68E-36
FAM69B	4,494782	3,43E-07
RPS6KB1	4,491079	2,70E-117
ARL15	4,490302	1,82E-09
CNFN	4,483039	0,000303
ZDHHC23	4,477086	1,22E-16
ARTN	4,476726	6,50E-05
LHX4	4,463645	2,83E-34
SLC29A2	4,460179	1,38E-35
SCAMP5	4,453413	1,05E-17
SORT1	4,437623	2,74E-36
ALDH5A1	4,430792	9,59E-39
FAM213A	4,425294	3,71E-41
TP53TG1	4,421458	5,36E-27
DHTKD1	4,418975	7,07E-71
ZNF497	4,417796	3,67E-10
CREB3L4	4,416843	6,49E-16
PTPN6	4,416448	5,04E-10
TRIM37	4,403863	1,13E-125

AXIN2	4,402852	7,10E-90
ZNF385A	4,400757	2,27E-66
GREB1L	4,396537	0,000601
ALDH3A2	4,388437	1,63E-83
LINC01003	4,380055	2,52E-07
PCDHGB2	4,378987	0,000165
FRMD6-AS1	4,360294	7,87E-06
PRRT4	4,34984	0,000115
CAMK2B	4,344628	6,27E-11
L2HGDH	4,312912	2,61E-34
SMPDL3B	4,304967	1,03E-36
TMEM97	4,304326	4,77E-65
ZNF879	4,301368	4,09E-06
CA11	4,280363	5,75E-06
PREX1	4,277033	1,65E-51
CELF5	4,27689	7,03E-06
ERV3-1	4,259793	1,60E-22
APPBP2	4,259401	3,15E-112
DTX3	4,247482	7,21E-27
EHF	4,239268	0,00025
LRFN1	4,237592	1,73E-08
USP32	4,230191	2,43E-43
EFHD1	4,226231	3,63E-12
KRTCAP3	4,224448	4,84E-20
PRDM6	4,221887	4,40E-07
LYRM9	4,218861	7,43E-08
BDH1	4,218031	1,33E-54
MTL5	4,215757	6,80E-18
HEATR6	4,200054	8,91E-75
RMI2	4,197429	5,05E-28
FAM171B	4,193317	3,16E-23
RHOBTB1	4,193118	1,03E-80
HOOK1	4,18456	7,00E-16
PCDH7	4,176863	9,84E-09
ADAMTS13	4,171878	2,56E-05
BCAS1	4,167976	1,81E-05
MTMR4	4,154596	4,44E-61
CPT1C	4,15065	1,75E-05
DHFRL1	4,148432	6,95E-15
ADAM11	4,146964	5,24E-07
SP5	4,142276	1,27E-39
LINC00925	4,137209	6,42E-08
TNRC6C	4,133269	2,28E-49
FOXA1	4,131224	3,72E-88
NDRG2	4,129026	1,88E-14
KBTBD7	4,119314	6,97E-11
RAD51C	4,115075	4,55E-74
ELMO3	4,100785	3,30E-41
ARHGAP39	4,089179	2,94E-27
CRMP1	4,070618	5,53E-08

SIM2	4,059076	0,000649
PODXL2	4,054086	4,05E-48
SIX4	4,053182	1,59E-24
CYP1B1	4,041483	1,79E-05
CTSF	4,03573	6,63E-08
PRKCZ	4,027294	2,14E-28
SLC26A4-A:	4,01697	6,54E-10
CXADR	4,01587	7,71E-20
PRICKLE2-A	3,998501	4,43E-05
ASH1L-AS1	3,992103	0,000918
IQCH-AS1	3,987963	3,51E-15
FAM227B	3,98745	4,94E-05
HIST2H2BE	3,986552	3,27E-13
CLU	3,970964	2,11E-24
EFR3B	3,962512	4,70E-19
JMY	3,953759	3,02E-10
GAA	3,936595	4,89E-34
COLEC12	3,924531	1,19E-14
EFNA4	3,915648	1,97E-19
VASH2	3,912098	4,44E-07
ADGRE2	3,910704	6,79E-05
CYP2J2	3,909647	1,27E-14
PARD6B	3,902396	1,97E-28
DCXR	3,888978	4,51E-38
KNDC1	3,853255	9,21E-06
BTG2	3,845164	1,01E-13
PBXIP1	3,840873	4,79E-09
FGFR2	3,833107	2,69E-05
ZNF821	3,826942	7,86E-15
IL17RE	3,813704	2,15E-06
CGREF1	3,808037	2,96E-29
DNMT3A	3,806559	4,39E-50
LOC100506	3,79364	1,72E-09
PRSS23	3,79134	5,38E-33
CEMIP	3,78381	9,90E-15
REEP1	3,771066	1,04E-05
GALNT12	3,762374	1,17E-19
ANKRD34A	3,757896	6,13E-14
SPINT1	3,757106	5,84E-42
HYKK	3,75374	3,35E-06
DGKE	3,753429	8,41E-13
TMEM191A	3,751327	1,14E-14
RAC3	3,746616	2,75E-30
FAM214A	3,725923	4,43E-10
KCNK15	3,724737	1,72E-15
ARHGEF4	3,724649	3,79E-05
PRRT3	3,722094	8,93E-06
FOXO6	3,717833	0,000375
SRRM3	3,714597	0,000427
MOCS1	3,711164	7,80E-09

PITPNC1	3,707705	9,00E-33
BRSK1	3,693827	1,51E-10
PCDHB16	3,688994	5,36E-17
PXN-AS1	3,688119	1,03E-14
TMEM187	3,672888	9,69E-13
ACVR2B	3,67051	1,39E-10
STARD10	3,667832	3,81E-23
CNKSR1	3,666537	0,000444
PCDHB14	3,666126	1,80E-18
AGAP2	3,665993	3,99E-17
ICK	3,665751	1,68E-40
CSPG5	3,665329	1,60E-08
KRBA2	3,659739	4,20E-06
PCDHB9	3,653752	1,23E-10
FASN	3,65103	2,44E-24
PTPRO	3,649014	3,60E-10
MANSC1	3,639365	2,26E-09
SPATA7	3,636598	5,33E-10
OLFM2	3,636255	3,57E-26
SLC46A3	3,634212	0,00019
TEX9	3,633694	7,45E-10
LONRF2	3,601273	6,48E-14
PSMD6	3,600829	5,37E-63
MAFB	3,598815	9,55E-14
CPLX1	3,598445	7,25E-13
IRF6	3,595374	5,35E-17
FAM117B	3,594135	1,68E-22
PCSK6	3,57954	1,73E-25
PPFIBP2	3,55452	7,27E-14
CTXN1	3,53785	2,15E-41
ASCL5	3,529406	0,000433
RGL2	3,52251	8,75E-23
HOXC11	3,522415	7,48E-09
NRGN	3,513856	2,18E-09
HSD17B8	3,508756	4,00E-07
YPEL1	3,507376	0,000684
HSD17B7	3,499974	1,68E-09
PPAPDC2	3,486642	4,95E-10
SUSD3	3,477727	4,37E-07
CFAP53	3,472445	0,000174
FAM102B	3,470913	3,97E-38
CASKIN1	3,462457	1,35E-12
ASS1	3,460361	4,72E-18
CACNA2D1	3,452833	2,70E-10
MAP3K14-/	3,450185	1,93E-07
DUSP9	3,448888	7,36E-09
TSPAN15	3,446096	5,95E-18
LRRC24	3,444403	1,34E-06
ATP5S	3,430793	7,76E-12
PLCH1	3,425678	3,83E-31

EPHB4	3,419799	5,66E-68
FAM72D	3,418325	7,10E-11
XYLT1	3,41826	1,12E-08
C4orf48	3,411681	1,84E-15
FBXL16	3,409745	2,37E-12
BOLA1	3,404325	5,32E-20
LOC101927	3,401406	6,34E-15
PFDN4	3,394908	8,41E-63
TSTD1	3,393185	1,19E-19
P2RY6	3,386927	0,000331
EFNA2	3,376858	0,000338
TP53I11	3,373529	2,50E-34
NKAIN1	3,371403	4,32E-17
C2orf81	3,371363	1,90E-07
FRAS1	3,364255	5,09E-20
TET1	3,347367	1,39E-09
GLUD2	3,33963	1,85E-08
LMTK3	3,339602	1,64E-08
CELSR3	3,331421	5,92E-19
ZNF703	3,330719	3,72E-56
ANKRD18A	3,32568	2,55E-05
TTC25	3,325481	0,000569
SUV420H2	3,323913	1,56E-19
G6PD	3,317617	0,000743
BCAM	3,308112	7,42E-28
SFXN2	3,305444	5,15E-19
SUOX	3,301373	7,00E-11
CHRM3	3,301025	4,55E-05
CRHR1-IT1	3,296608	0,000113
SMIM14	3,294669	1,46E-06
RNF44	3,294247	2,84E-44
DOC2A	3,293501	3,18E-06
KSR2	3,29055	8,55E-06
NCOA3	3,285236	9,65E-45
SOX4	3,283663	4,75E-40
IGFLR1	3,279726	7,41E-08
CDKN1A	3,277401	9,08E-11
NANOS1	3,269817	3,74E-15
ISYNA1	3,26653	2,02E-41
THNSL1	3,265854	3,40E-16
NUDT7	3,265024	0,000414
TMTC2	3,255038	1,97E-09
AP4S1	3,251664	2,26E-09
UNC13D	3,249047	4,72E-05
AP1M2	3,237128	3,01E-42
EPHA6	3,236467	7,54E-08
GDPD3	3,235521	0,000501
SRCIN1	3,233343	0,000111
C11orf71	3,230252	1,08E-05
TBX2-AS1	3,228777	3,47E-05

NIPSNAP1	3,22665	3,32E-42
DDIT4L	3,224486	1,19E-08
C6orf52	3,220141	0,000286
TKFC	3,218874	1,74E-20
C17orf96	3,204066	1,77E-28
ID1	3,199436	2,70E-14
DHCR7	3,199255	9,36E-28
RPRD1A	3,197652	2,89E-45
POLG2	3,197056	3,14E-09
NDRG4	3,196447	3,72E-07
UFSP1	3,190312	1,16E-05
KRT81	3,188341	0,000234
ZBTB42	3,187639	4,45E-27
THNSL2	3,185984	1,29E-10
ALDH4A1	3,185943	3,72E-08
XK	3,185405	0,000199
PIK3C2B	3,183123	3,38E-28
LENG9	3,174205	3,40E-10
CCNG2	3,173829	1,57E-10
PIK3R3	3,17162	8,09E-18
AMZ2P1	3,16836	7,09E-07
TP53INP1	3,164382	8,76E-07
EFNA3	3,157236	1,98E-15
FMO5	3,15639	0,000484
PLEKHH1	3,155548	4,42E-24
MAP7	3,151964	4,07E-33
DYRK1B	3,150195	1,37E-22
SAMD12	3,148388	1,58E-09
SEPT3	3,139472	4,42E-14
RAB30	3,110869	1,65E-17
SEMA4G	3,109343	0,000366
ZSCAN16	3,105809	7,79E-18
IPO5P1	3,096521	9,47E-11
TRAPPC6A	3,094219	5,34E-17
MIR5047	3,090186	1,94E-05
TDRKH	3,084433	6,42E-13
TMC6	3,081516	7,17E-19
ZNF253	3,071887	1,69E-26
EXPH5	3,064038	6,75E-19
ACADSB	3,056451	3,55E-12
PROCA1	3,051769	0,000228
SYNPO2	3,047143	8,21E-09
ATXN7L3B	3,047066	1,75E-46
DHRS13	3,040992	4,58E-10
RALGPS1	3,03936	7,37E-12
UNG	3,035691	2,25E-42
HBP1	3,033918	1,04E-10
ELL3	3,03273	5,19E-12
GABPB1-AS	3,03033	3,71E-09
ZNF837	3,024929	6,62E-07

ZBTB12	3,016013	6,14E-08
SERINC5	3,011158	1,77E-18
NUDT16L1	3,007989	6,03E-15
HR	3,007782	5,49E-13
SLC27A3	3,004443	4,46E-31
DSP	2,990561	1,03E-45
LIPT2	2,989156	0,000772
SLC25A10	2,988291	1,99E-42
ZNF287	2,986738	5,28E-09
SORL1	2,982038	2,27E-09
STOX1	2,970118	0,000122
SOX13	2,968994	2,75E-14
PARD6A	2,967669	0,000154
ARID5B	2,964913	3,08E-27
SH3YL1	2,964035	1,49E-22
PCYOX1L	2,963953	1,44E-12
GLUL	2,962135	2,22E-67
HOOK2	2,959838	2,19E-41
IFT140	2,955675	8,81E-07
MTMR7	2,954551	0,000596
ZNF19	2,948621	4,19E-05
DDR1	2,948465	4,74E-13
SMKR1	2,945895	3,37E-09
FAM131A	2,94445	2,68E-06
ZP3	2,940165	1,04E-14
MDM2	2,934547	4,57E-14
HCN2	2,934166	7,19E-10
FRAT2	2,933956	4,20E-08
PRMT6	2,933145	2,84E-46
ZNF204P	2,931461	0,000205
STRA13	2,930603	2,56E-33
SYNGR1	2,928829	2,74E-05
NIPBL-AS1	2,924041	3,29E-10
TMEM38A	2,917689	1,39E-05
C21orf33	2,916151	1,33E-22
RGS6	2,912671	0,000232
SLC29A4	2,911311	5,42E-14
PLXDC2	2,910112	1,86E-15
NUDT2	2,909024	6,32E-08
MSI2	2,906011	8,89E-15
FAM201A	2,905726	8,47E-05
PDZD4	2,903828	1,27E-06
FBXL20	2,898123	2,44E-06
CCM2L	2,894093	9,03E-05
PLEKHA7	2,892196	2,13E-21
EFNA1	2,888585	1,33E-10
CFAP44	2,888167	0,000979
EMP2	2,884242	4,07E-18
TRPS1	2,878529	8,96E-45
ZNF721	2,878317	3,21E-25

ATXN7L1	2,873738	3,04E-16
ZNF652	2,865432	1,11E-25
UNKL	2,855208	2,71E-13
GDF11	2,852826	2,71E-12
ZNF217	2,847627	2,30E-53
SCML2	2,847216	1,73E-08
BFSP1	2,8465	2,57E-06
MAML3	2,84633	6,72E-06
ZNRF3	2,844808	5,69E-45
SH3BP5	2,841552	3,89E-38
ALKBH4	2,837539	2,00E-23
DUSP2	2,834022	1,47E-05
ADI1	2,832865	3,56E-26
SYNE2	2,831285	9,63E-19
KAZALD1	2,830611	0,000222
PAIP2B	2,824564	7,73E-08
PXMP4	2,818698	6,25E-07
MAN1A1	2,816276	4,30E-13
FAM72A	2,806229	6,55E-05
ATP6V1E2	2,791382	0,000327
CFD	2,788668	2,53E-06
NMNAT2	2,784532	5,26E-06
CYP24A1	2,783908	0,000214
SARM1	2,782903	1,41E-06
RNASEL	2,781734	3,36E-08
SVIP	2,778076	8,53E-13
D2HGDH	2,777821	4,64E-16
MEPCE	2,776305	1,56E-49
ROGDI	2,767255	2,06E-10
CCDC85C	2,753491	4,90E-36
PSMG3-AS:	2,752711	2,28E-09
CELSR2	2,745222	3,65E-27
PRKAR2B	2,739588	1,97E-07
PIF1	2,733856	0,000109
NUDT12	2,733451	8,16E-06
DTWD1	2,732164	7,04E-18
STARD5	2,725723	0,000257
SMARCD2	2,715145	9,72E-36
KDM5B	2,710088	2,25E-18
ZNF815P	2,704942	0,00045
FAAH	2,698683	1,26E-12
SLC46A1	2,695357	1,33E-13
FZD3	2,693712	1,73E-12
FLJ37453	2,684489	7,16E-05
FAM86C2P	2,683803	1,17E-06
PEX1	2,683396	6,25E-19
CSDE1	2,676035	9,64E-31
MCM3AP-A	2,6751	2,22E-10
PCDHB10	2,674175	0,000614
FZD2	2,67124	6,91E-13

TRIM33	2,668185	4,95E-34
E2F2	2,66477	2,05E-05
INTS2	2,663598	4,99E-10
JHDM1D-A	2,655123	3,31E-09
MACROD1	2,653416	4,07E-14
MPZL3	2,652744	8,95E-09
LAMA5	2,650172	3,08E-32
SIAE	2,649546	1,75E-09
ADCY6	2,646084	5,08E-12
EPHA10	2,644792	8,07E-05
ZKSCAN1	2,640108	2,45E-24
FAM46B	2,638257	7,81E-08
CEBPA	2,635732	1,17E-06
LAGE3	2,632313	6,27E-21
C6orf226	2,630161	2,34E-05
ZFP90	2,629165	1,33E-20
C5	2,62631	1,64E-06
ZNF595	2,625787	7,41E-14
KIAA2018	2,610906	3,12E-13
MXD4	2,608826	1,82E-09
ACTR6	2,608781	3,40E-14
ZNF397	2,604951	3,04E-07
MKL2	2,603084	2,27E-23
BTD	2,596648	0,000214
FGFR4	2,591241	3,73E-13
NHP2	2,580577	1,02E-42
SLX4IP	2,578716	3,10E-05
HOXC-AS3	2,577343	0,000414
DANCR	2,577322	5,36E-19
PTPRS	2,576965	1,98E-30
FAM149B1	2,575786	2,22E-10
KANSL1L	2,567684	2,34E-08
RIMKLA	2,563227	0,000109
LEF1	2,562536	2,21E-18
MBTD1	2,558886	1,11E-11
SPA17	2,55885	1,31E-07
CCDC88C	2,556605	6,67E-13
SLC29A3	2,555627	4,29E-09
ZNF512B	2,554433	1,17E-21
MCTS2P	2,553339	2,23E-08
CCDC120	2,552838	2,59E-08
TRIM41	2,550487	7,35E-25
CRABP2	2,547083	1,28E-08
DNPH1	2,546381	2,96E-33
FAM212B	2,546327	1,03E-05
LINC01004	2,541812	0,000377
SPPL2A	2,53898	7,08E-29
DOLPP1	2,537035	3,03E-13
ZNF713	2,535318	9,65E-08
SNX27	2,533902	1,20E-29

ZSCAN21	2,530847	1,91E-19
TMEM238	2,530626	2,09E-06
PDE4D	2,52708	2,63E-10
USP30	2,525014	5,08E-11
WNT10B	2,523555	0,000145
GPR153	2,516307	2,60E-09
TRIM24	2,516287	2,07E-31
MIR600HG	2,515701	4,11E-06
TMEM168	2,511042	8,79E-22
SLC9A3R1	2,507766	3,35E-18
VPS26B	2,504643	3,39E-11
ALG10	2,500727	1,10E-05
G2E3	2,49835	7,82E-15
CYB561	2,483762	2,31E-40
MARVELD2	2,482745	5,43E-11
HMMR	2,479541	9,26E-15
ABCB6	2,476358	3,57E-14
FITM2	2,47524	9,96E-09
WDR34	2,474593	2,72E-36
OSBP2	2,470901	0,000133
SECTM1	2,468875	2,61E-05
ATOH8	2,46586	1,04E-05
ZFP62	2,465664	4,28E-27
PAXIP1-AS1	2,46563	2,16E-06
DLGAP5	2,464704	7,50E-31
PYGO2	2,462759	5,01E-25
NUDT4	2,460376	2,82E-19
DMXL2	2,458869	3,91E-27
ATG10	2,457487	1,11E-06
KIF27	2,455552	0,000181
SLC39A11	2,451406	5,61E-08
SEMA3F	2,451309	2,05E-15
ERVMER34	2,449118	7,07E-05
EARS2	2,448296	1,22E-25
ZNF785	2,443504	1,11E-06
SOBP	2,441052	0,00037
RNFT2	2,44053	6,75E-05
XPC	2,43871	5,96E-21
ZNF138	2,436094	5,50E-10
ZNF117	2,433573	4,17E-07
OR7E14P	2,43311	0,000174
DYNLL2	2,431858	9,11E-37
OARD1	2,43043	2,44E-18
TRIM45	2,4284	6,48E-08
LOC90784	2,428161	5,74E-20
SETMAR	2,425052	2,10E-14
ZNF76	2,417117	2,09E-13
FAM185A	2,414733	8,80E-09
FAM172A	2,412629	5,45E-08
PP7080	2,406461	8,47E-10

GSTA4	2,405666	3,27E-07
ZNF687	2,403817	2,88E-34
ZMYM2	2,399234	1,03E-30
LRRC45	2,398548	1,30E-21
ZBTB41	2,397348	5,85E-15
ABCA2	2,393398	9,27E-15
SNHG25	2,390219	2,02E-05
DET1	2,386704	4,69E-05
SYT12	2,380696	1,59E-08
GOLPH3L	2,379669	2,48E-10
SCRN2	2,376527	2,70E-05
SLC12A9	2,375397	3,75E-09
EPM2AIP1	2,372516	5,00E-11
NBPF3	2,369725	3,93E-05
TMEM256	2,36712	9,95E-06
DCAF7	2,36482	1,03E-26
ZNF48	2,35861	4,87E-08
TMEM184/	2,358176	2,15E-08
ALPK1	2,356604	8,08E-05
CLDN4	2,354052	3,82E-12
CHD6	2,351676	1,77E-29
ARHGAP19	2,351598	1,61E-08
COG7	2,34981	2,87E-13
NQO1	2,346836	4,43E-05
PPA2	2,345855	3,19E-16
ADCK3	2,344968	0,000374
GSTZ1	2,343687	8,36E-20
CCNG1	2,342651	2,48E-07
STXBP4	2,340323	4,56E-05
HES6	2,340209	0,000624
NDUFB10	2,337887	1,16E-29
KHK	2,337053	4,39E-14
PPM1L	2,333581	0,000137
EFEMP1	2,331765	1,45E-22
CMTM8	2,330094	3,94E-09
DTNB	2,325707	1,35E-13
DTD2	2,325165	2,37E-09
BPTF	2,322454	8,29E-37
ESRP2	2,321653	2,29E-21
PTRH2	2,31606	1,35E-19
SEMA4A	2,315749	4,61E-11
CACNB3	2,314858	4,04E-23
PRDX2	2,312474	2,38E-26
GMCL1	2,308947	2,41E-11
ZNF219	2,307645	4,56E-13
MMAB	2,306469	9,67E-26
SH2D3A	2,30561	1,86E-05
OSCP1	2,300715	0,000199
F11R	2,296762	7,55E-22
PUS7	2,2953	2,26E-16

ALAD	2,28842	4,12E-10
DHX40	2,287201	2,57E-17
AP3B1	2,281147	1,06E-22
MMACHC	2,280126	1,56E-06
ARHGAP44	2,278925	4,01E-08
MYO5B	2,278373	5,17E-17
GLCE	2,276647	1,42E-13
DMKN	2,276326	3,74E-06
PCDHGB1	2,274047	1,58E-05
TEAD3	2,273826	9,79E-13
SIAH2	2,273816	6,69E-17
MMP24-AS	2,272159	1,18E-08
TCAF1	2,271056	1,92E-13
SERTAD4	2,270469	4,99E-06
MCM9	2,267919	1,09E-09
DENND6A	2,265217	4,59E-12
RSAD1	2,262291	4,29E-13
FHDC1	2,261309	2,33E-05
ZSCAN2	2,260519	6,79E-16
TMEM37	2,260472	1,85E-06
KIAA0586	2,259601	6,70E-12
FAM86EP	2,257879	1,44E-05
TSPAN6	2,256491	1,09E-12
MIS18BP1	2,255365	1,16E-07
SPR	2,254647	1,57E-22
HDDC3	2,253072	1,29E-12
LHPP	2,252961	1,36E-10
RASGEF1A	2,251175	3,78E-09
ZNF771	2,250384	1,50E-06
IDUA	2,249851	3,76E-05
GCHFR	2,2479	0,000681
RMI1	2,241176	1,93E-07
HIPK1	2,241114	2,23E-29
MAGI1	2,24108	7,16E-11
CPNE7	2,23719	4,16E-06
FTSJ3	2,235419	6,96E-19
ABCC5	2,23332	1,24E-09
ZNF276	2,233034	2,24E-17
THUMPD3-	2,232422	6,24E-05
PHLPP1	2,23239	1,09E-18
SURF1	2,231948	4,12E-10
RBM47	2,231602	1,60E-10
NEBL	2,226872	3,36E-13
ARRB2	2,224137	2,94E-17
TOB1	2,221588	6,98E-12
TNFRSF19	2,220615	3,03E-18
ZNF362	2,218784	2,30E-08
GAREM	2,214658	9,03E-05
SNAPC5	2,211369	6,81E-12
LTBP1	2,210877	1,90E-13

NR1D2	2,206529	2,10E-15
DAAM1	2,204861	4,61E-17
STX6	2,19832	4,93E-20
ERMP1	2,198015	1,13E-07
PSRC1	2,19496	1,63E-11
PATZ1	2,19383	1,01E-14
C1QTNF9B-	2,19253	9,80E-06
ARID2	2,191102	4,12E-16
TBC1D9	2,1907	1,20E-17
MED13	2,190665	5,75E-25
PDF	2,189832	7,85E-06
TET2	2,186594	9,54E-17
GSE1	2,182786	9,53E-27
H1FX	2,181721	4,89E-15
IL17RD	2,178513	1,90E-06
EHMT2	2,176384	3,62E-25
DLD	2,176135	2,71E-13
CD9	2,175448	7,67E-17
C3orf14	2,17539	1,04E-15
KNOP1	2,174659	3,35E-24
ZFHX3	2,172832	1,97E-21
THOC7	2,17244	1,47E-27
ARSG	2,170585	1,12E-07
RAB3A	2,169848	0,000122
NT5C	2,169451	2,61E-10
CDC25C	2,168814	4,39E-06
KIAA1958	2,167692	1,14E-11
EGFL7	2,164945	1,00E-10
SLC22A23	2,156183	4,23E-16
BCDIN3D	2,155933	2,93E-05
IVD	2,154676	2,07E-15
CRYL1	2,154594	4,62E-05
TMEM183A	2,154071	7,60E-25
MGC57346	2,15258	1,34E-05
BBS4	2,152037	3,62E-07
LINC00938	2,149128	7,18E-07
PYCRL	2,148207	3,62E-11
OLMALINC	2,148042	4,62E-05
CNOT6	2,143538	1,30E-20
AHR	2,141976	2,74E-24
EAPP	2,140104	6,32E-13
IQCK	2,13866	5,68E-10
IDH2	2,134446	1,58E-23
UBN2	2,131958	1,35E-12
APRT	2,131371	6,26E-30
FANCE	2,125991	0,000645
ISOC1	2,124165	1,46E-19
THEM6	2,121643	1,68E-16
H1F0	2,121306	2,75E-06
RSBN1	2,118846	1,65E-14

LIG3	2,113982	8,41E-18
OSR2	2,113933	0,000106
WDR17	2,107635	0,000573
RNF122	2,106671	2,77E-06
MPND	2,105402	2,46E-11
SPICE1	2,102798	5,57E-10
KIF20A	2,100358	1,69E-18
NOL11	2,097412	1,77E-28
RCOR2	2,092604	7,34E-05
ATPAF2	2,092085	6,06E-09
AP1S1	2,091149	9,57E-10
ACTR3B	2,089261	3,90E-13
C2orf68	2,08673	4,27E-17
CARHSP1	2,082649	3,72E-19
MED20	2,080032	3,18E-08
MUT	2,079136	1,21E-08
PI4K2B	2,07909	6,05E-12
MED13L	2,078596	1,66E-17
KIF14	2,078207	3,88E-19
PCLO	2,076885	1,11E-10
MRPS25	2,076017	1,18E-20
AP4M1	2,073632	4,44E-12
C14orf2	2,070575	1,61E-22
TOP2B	2,070374	4,10E-26
MDM1	2,066916	3,44E-05
MUC1	2,06627	0,000226
DFNB31	2,065129	5,08E-07
TEAD2	2,063764	5,44E-13
TRIM4	2,06282	2,91E-14
EPB41L4A-/	2,060202	1,77E-06
TTC8	2,058287	7,10E-11
INTS4	2,056741	5,35E-12
ANGEL1	2,056503	2,77E-10
PARPBP	2,056435	2,67E-12
DNAJC19	2,056042	6,49E-10
LNX2	2,055337	4,79E-15
FAM50B	2,054642	9,76E-08
ZBTB5	2,053959	2,52E-11
EIF5AL1	2,053593	4,36E-08
USP8	2,053147	1,03E-25
WDR54	2,052941	3,21E-08
ANKS1A	2,05273	3,43E-17
CETN3	2,051963	3,38E-07
ARPP19	2,050835	2,54E-25
C9orf116	2,050161	1,47E-05
NCBP2-AS2	2,048272	2,08E-10
CTNNBIP1	2,046523	8,16E-10
THAP9	2,046201	0,00086
LINC01521	2,045241	0,000151
TMPO-AS1	2,037517	5,56E-05

ZNF3	2,031505	2,27E-18
PANK3	2,029899	1,29E-15
RNF208	2,029619	5,03E-08
BTBD6	2,02554	6,67E-08
PRKX	2,025419	8,66E-11
CINP	2,023568	6,01E-08
MDM4	2,022631	3,35E-09
EIF4B	2,02175	3,49E-31
ZNF252P	2,020998	1,28E-18
ILF3-AS1	2,020644	1,03E-07
ZMYND11	2,01959	4,00E-20
HMBS	2,018393	2,14E-19
TBC1D5	2,018124	6,77E-19
IFT27	2,017629	9,88E-08
MCCC2	2,015866	6,40E-16
SCAI	2,014994	3,55E-07
NMU	2,014965	0,000607
TPRN	2,014202	6,12E-12
SCAND2P	2,013952	0,000946
CNOT8	2,01389	4,48E-18
SMURF2	2,01382	2,77E-11
ALG6	2,012594	0,000112
DEPDC1B	2,011123	1,17E-08
MGAT4A	2,008719	4,05E-05
IFT22	2,007305	2,81E-18
MKS1	2,006108	1,16E-10
EZH2	2,004043	4,08E-10
TAMM41	2,002768	3,23E-12
BBS2	2,002129	3,67E-10
NSD1	2,000123	1,54E-29
ZNF624	1,998038	5,31E-05
SLC25A29	1,997946	4,32E-17
SKIDA1	1,996079	0,000239
HIST1H2BD	1,995616	0,000298
DENND2D	1,994185	1,50E-13
RFX5	1,994028	8,05E-08
ZMIZ1	1,99375	1,10E-13
PRUNE	1,993064	5,49E-09
ZNF618	1,991568	3,29E-23
NR1H3	1,991531	0,000252
ZNF74	1,987459	5,20E-16
GSTM3	1,98596	3,48E-05
LINC00674	1,983378	1,03E-12
IST1	1,983333	9,47E-17
SCD	1,982163	2,03E-29
FAM210B	1,981829	1,48E-09
MCM2	1,981739	4,38E-25
TMEM198B	1,980963	3,60E-05
ARHGEF3	1,979968	2,64E-09
SULF2	1,97995	5,79E-13

ZNF18	1,978354	0,000492
INSR	1,974233	0,000998
LYSMD2	1,97422	4,72E-11
SLC9A3R2	1,97058	1,13E-15
SETDB1	1,970197	1,11E-12
PSMD5-AS1	1,969318	4,98E-06
DPH1	1,969282	0,000102
CEP97	1,968487	1,21E-05
WDR6	1,966503	1,76E-13
ASMTL	1,965056	3,22E-08
LOC101927	1,96489	7,24E-05
PITPNA-AS1	1,963224	0,000243
ADAT1	1,962836	4,95E-17
SIGIRR	1,961264	4,66E-13
ARHGEF9	1,96125	3,08E-05
LMNB1	1,957719	4,93E-19
GDE1	1,954254	3,55E-15
SNHG19	1,951393	0,000162
IL27RA	1,951365	1,72E-05
TBCK	1,950823	9,46E-12
RPRD2	1,950583	4,66E-23
HSPA1A	1,950424	5,31E-06
CLNS1A	1,949675	5,86E-22
LOC730101	1,949147	0,000902
HMG20B	1,945369	4,69E-23
TMEM106C	1,944796	3,93E-13
COA3	1,944114	2,21E-10
FIS1	1,943142	1,00E-18
NUDT16	1,942676	3,52E-06
GIN1	1,940487	2,23E-06
C16orf46	1,93705	4,34E-05
GALNT3	1,936891	1,72E-06
ANKRD50	1,936362	3,55E-19
DCP2	1,936169	8,46E-14
ZCWPW1	1,936132	6,32E-05
DDX6	1,934929	5,78E-23
H2AFX	1,93456	4,60E-23
LMBRD1	1,931546	0,000382
BCAS2	1,928126	8,13E-19
ZNF845	1,928078	5,76E-09
SLC24A1	1,927858	1,47E-08
LOC645513	1,925706	0,000425
CENPBD1	1,925454	4,12E-10
ZNF277	1,925222	6,00E-11
MYEF2	1,922917	3,49E-07
SIKE1	1,922111	1,21E-20
PC	1,922052	0,000941
CDKN3	1,917677	1,02E-15
MVK	1,91755	4,90E-05
HIP1	1,916506	3,84E-12

ENSA	1,916195	2,31E-25
ZNF354A	1,91538	1,50E-08
MTHFD2	1,912545	3,65E-06
WDR89	1,912352	1,70E-10
NUCKS1	1,91186	4,73E-18
NEK11	1,911817	0,000461
ONECUT2	1,911399	2,72E-08
NEO1	1,90769	2,97E-14
P2RY2	1,907019	1,09E-08
UBL7-AS1	1,905989	0,000506
ZNF839	1,905101	1,72E-05
ZNF688	1,904465	2,29E-06
HAUS4	1,902657	9,93E-06
RPAIN	1,899882	7,96E-12
PDK2	1,89936	0,000438
RNF182	1,899269	0,000939
KIAA0100	1,899039	3,93E-10
TRNT1	1,898837	2,22E-07
TMEM161/	1,898144	3,52E-08
ACACB	1,895755	8,80E-08
ZFP1	1,895073	5,48E-11
TRRAP	1,894061	5,95E-26
CSAD	1,892558	0,000329
C11orf30	1,892064	2,43E-12
ZBED6	1,890034	0,000101
SPATA2L	1,886766	6,59E-11
ADRBK2	1,8863	0,00033
SUPT4H1	1,886089	6,53E-18
PGBD2	1,88535	5,71E-05
HOMEZ	1,884982	2,45E-07
TIGD2	1,88469	0,0008
GAN	1,882314	7,67E-06
PIGM	1,881763	5,62E-07
KLHDC3	1,878681	6,47E-11
CHURC1	1,878365	3,75E-11
KAT2A	1,877984	5,16E-20
BRWD1	1,877667	1,19E-10
FAM53B	1,876941	8,19E-17
OCLN	1,875707	1,25E-06
MAPK6	1,87364	8,08E-22
DDX59	1,873324	3,11E-06
NOA1	1,872962	3,27E-10
TRMT5	1,872895	2,03E-10
DHODH	1,87246	2,33E-10
CBLB	1,870345	2,92E-10
C6orf120	1,870185	7,34E-10
KCTD1	1,8701	2,69E-08
CD24	1,869747	2,66E-13
PVRL1	1,869125	6,94E-07
ZNF619	1,866254	0,000371

BRCA2	1,865189	1,66E-06
PCMTD2	1,864824	2,66E-07
GAS8	1,864658	3,69E-12
PLXNB1	1,864245	1,47E-13
TNS2	1,863907	2,08E-05
C12orf66	1,862493	6,58E-05
C10orf35	1,861893	0,000212
MAPK3	1,861204	5,58E-08
LEO1	1,860617	5,03E-23
CBR4	1,858207	5,60E-10
GCH1	1,857495	3,85E-08
PGM2L1	1,855357	5,89E-10
CLTC	1,85472	3,74E-14
CBX8	1,854249	4,62E-06
ZNF510	1,850307	3,35E-08
DCTPP1	1,848254	3,34E-16
PIGO	1,84817	6,86E-07
RPS3	1,84653	2,09E-15
C1QBP	1,844614	6,59E-19
AMOT	1,840886	3,19E-09
C12orf57	1,839899	1,56E-09
SIVA1	1,839305	7,48E-12
SNHG4	1,834751	0,000904
NUDT3	1,834494	6,05E-07
PAXIP1	1,832357	1,32E-11
C14orf93	1,831975	2,87E-05
GLRX5	1,830839	1,52E-13
AGFG2	1,830608	0,000559
ZNF552	1,828207	0,000167
COX7C	1,825253	2,60E-20
MRPL41	1,825119	2,10E-15
PAQR8	1,823404	2,69E-05
TMEM129	1,823043	6,46E-06
ZNF212	1,822999	4,12E-09
COIL	1,821578	1,30E-06
TGIF2	1,821516	1,54E-08
ARMC10	1,821151	1,30E-15
TMEM64	1,819807	6,58E-19
TMTC4	1,819782	7,15E-06
LRFN3	1,81922	2,01E-05
POLI	1,817593	0,000605
PAIP2	1,817553	1,94E-10
PAPOLG	1,817524	4,99E-05
CCP110	1,817135	9,10E-06
PGAP2	1,816811	1,31E-09
SLC35B2	1,815717	3,07E-10
EFCAB11	1,814845	9,84E-07
IMPACT	1,814725	3,46E-12
DDAH2	1,813744	6,02E-11
ELMOD2	1,81354	1,19E-06

RAB15	1,811328	9,28E-11
PIAS3	1,811046	1,63E-11
ZNF480	1,810523	1,67E-10
MTUS1	1,809785	6,56E-10
CIRBP	1,809545	2,91E-07
BRMS1L	1,809079	2,11E-06
ATP6V1C2	1,809021	0,00054
LMTK2	1,808107	5,57E-15
DHRS4	1,807398	9,76E-08
POGZ	1,805812	4,23E-10
OMA1	1,805486	4,62E-05
ZNF629	1,804484	1,27E-17
MPP7	1,804331	5,22E-11
BCL7A	1,802896	3,22E-12
RNASEH1-A	1,801181	3,00E-08
DBP	1,798736	5,60E-06
ATP5A1	1,798665	2,82E-20
PAQR4	1,798326	1,64E-20
PLK1	1,798269	4,99E-10
AP4E1	1,796751	8,16E-09
CDH24	1,796579	5,60E-11
ZNF33B	1,79585	1,00E-09
ARID4A	1,79581	2,30E-07
WDR83	1,795148	6,11E-07
SAYSD1	1,794276	1,07E-12
LRCH4	1,793514	2,25E-13
ZNF254	1,790305	8,39E-09
RPS25	1,787901	2,01E-15
FANCM	1,787473	7,91E-05
PRICKLE2	1,787447	1,36E-05
METTL2A	1,787083	5,17E-14
HEXDC	1,786851	1,34E-06
DHFR	1,785347	2,04E-11
CBS	1,784097	3,93E-07
LAMTOR4	1,78365	5,29E-14
ATXN1L	1,781752	3,54E-10
TMEM144	1,781664	0,000229
IDH1	1,781036	3,17E-21
SNHG8	1,780525	0,000336
IDI1	1,779901	3,96E-08
NME4	1,777864	2,24E-17
ACAT2	1,776825	1,87E-11
DMTF1	1,77649	3,30E-05
RMND5B	1,775296	4,63E-17
ISOC2	1,773746	3,54E-14
ZNF184	1,772642	2,41E-08
ZNF286A	1,771197	5,46E-12
SHPK	1,770773	9,10E-09
RPP25	1,770128	3,27E-08
NLN	1,769957	1,34E-18

PRADC1	1,769858	0,000741
PPCDC	1,768577	1,24E-06
HSD17B4	1,76796	2,59E-10
EZH1	1,767788	0,000243
SNRNP25	1,767131	3,03E-07
LMBR1	1,762024	6,62E-14
PDCD6	1,760457	5,21E-14
CCNF	1,758709	2,71E-15
TMEM183E	1,756443	9,69E-05
PAGR1	1,752512	5,68E-17
ZNF337	1,752309	0,000142
RAB3IP	1,750124	1,29E-16
ACAD8	1,749876	0,000214
HADH	1,748878	3,58E-08
MSRB1	1,748159	1,49E-06
C9orf114	1,746066	6,47E-11
ATXN3	1,745476	8,62E-08
ARHGEF39	1,744788	0,000862
KIF26A	1,743921	8,57E-05
C15orf61	1,743277	4,82E-05
WIBG	1,740556	8,21E-12
C14orf79	1,739315	0,000333
KLHDC2	1,737971	6,09E-06
HSPA1B	1,735859	7,07E-05
RNF169	1,733528	4,30E-09
ZSWIM7	1,733474	2,90E-07
IFT122	1,73175	7,29E-06
ABHD11	1,73108	1,06E-09
COPG2	1,730685	1,16E-09
NELFA	1,730384	6,93E-14
CASZ1	1,728421	1,34E-05
CYB5D2	1,726978	9,42E-06
FBXL19	1,72624	1,15E-12
MFSD3	1,72609	4,06E-12
TMPO	1,726011	5,55E-09
FBXO31	1,724073	8,83E-13
RGS12	1,72406	3,31E-08
NMRAL1	1,72365	1,60E-14
CORO1B	1,721865	6,12E-06
ARMCX6	1,7204	1,79E-08
C8orf82	1,719251	5,12E-12
RPS29	1,71895	4,45E-10
PLEKHA6	1,718805	1,15E-09
SLMAP	1,718589	1,35E-09
HSP90AA1	1,718451	7,50E-11
HEATR5A	1,717347	1,75E-09
HSDL1	1,716708	2,92E-08
ACSF2	1,715126	0,000357
TARBP2	1,712335	1,55E-12
CCPG1	1,711003	7,45E-05

PLEKHF2	1,709645	3,50E-12
CHCHD6	1,708917	5,88E-05
CCDC47	1,706468	2,23E-15
TBX3	1,706466	1,16E-06
FBXW9	1,704415	2,68E-08
BAG5	1,703326	5,79E-12
MCM7	1,703263	1,23E-14
CAND1	1,700891	7,48E-16
TARS2	1,698113	1,89E-10
HMGCS1	1,697279	2,22E-07
NFS1	1,696606	5,98E-09
NOP14-AS1	1,696443	2,69E-05
RSBN1L	1,695889	1,40E-07
KIAA1467	1,695826	0,000375
TRPM7	1,695769	9,14E-18
SSH3	1,694805	5,32E-08
RBSN	1,693486	8,98E-12
SMAD6	1,692367	0,000556
ZNF766	1,691099	3,70E-11
ZNF490	1,690533	9,28E-05
INPP4B	1,690199	3,69E-06
ULK1	1,69015	2,84E-07
NCBP2	1,689002	1,85E-14
PPM1A	1,68636	5,34E-08
SCAMP1	1,683781	3,02E-09
RALGAPA2	1,683431	2,90E-05
TMEM254	1,683095	5,60E-08
TMEM216	1,68018	0,000229
LOC93622	1,679393	3,74E-08
ZFP3	1,676905	1,20E-07
WWOX	1,676722	3,34E-08
GLTSCR1L	1,674471	1,01E-09
PPP1R9A	1,670392	4,41E-07
GABPB2	1,670117	1,68E-05
RPL17	1,668092	2,98E-09
TMEM134	1,66688	1,59E-06
BCL2L1	1,666191	4,51E-08
PIGQ	1,665733	5,84E-09
GABPB1	1,664993	2,17E-09
TP53	1,664072	2,30E-09
FAM175A	1,66393	1,15E-07
PODXL	1,66379	6,70E-08
ITPKB	1,663447	1,39E-06
LOC148413	1,662514	8,30E-07
FRMD6	1,658215	5,74E-10
SAPCD2	1,656808	2,13E-08
TRAF7	1,656195	2,82E-17
TACO1	1,656149	3,55E-15
ARNT	1,656114	3,50E-12
ZNF433	1,654812	0,000126

FAM111B	1,654396	1,07E-08
NAT1	1,654198	0,000473
PSTPIP2	1,652361	8,16E-06
TMCO6	1,651225	5,25E-05
ZNF500	1,649299	0,000716
ZNF282	1,646948	4,05E-14
MYO6	1,645444	1,66E-12
MAZ	1,645281	6,94E-16
PHACTR2	1,640453	1,39E-08
IRX5	1,640102	5,60E-08
GIGYF1	1,638922	0,000133
LOC728554	1,637712	3,09E-07
BTBD9	1,637574	0,00016
SECISBP2	1,63661	1,16E-08
ETFA	1,635494	1,72E-09
EEF2K	1,635415	5,83E-12
ATP11C	1,633504	2,44E-08
GTF2I	1,633442	5,53E-18
POP7	1,633241	1,87E-07
LRP5	1,633206	1,67E-11
C14orf80	1,632247	1,24E-09
NDUFAB1	1,630624	3,59E-08
AASDH	1,629913	0,000668
UQCRH	1,629495	1,32E-16
DIRAS1	1,628618	9,93E-09
MRE11A	1,627931	1,33E-06
SOS2	1,627651	1,49E-09
LRRC75A-A	1,627301	1,39E-06
NCAPD2	1,625919	1,20E-13
CECR5	1,625478	1,00E-10
FKBP3	1,624826	1,18E-10
POLR3K	1,623104	8,59E-06
NDUFB1	1,622386	1,07E-09
RALBP1	1,620907	5,64E-10
KBTBD4	1,620712	4,71E-07
ZNF786	1,620182	1,40E-05
MECOM	1,619245	1,66E-06
VGLL4	1,618019	2,50E-09
ZMYM3	1,617032	1,01E-10
NELFCD	1,616881	1,16E-12
ZNF33A	1,616562	1,39E-10
DNAAF2	1,616156	2,41E-10
INVS	1,615904	2,20E-07
NDC80	1,611135	2,95E-05
TMOD3	1,611064	2,87E-18
SPINT2	1,61073	4,61E-17
DICER1	1,610548	1,91E-07
WDR73	1,608068	5,86E-10
CYB561D1	1,60537	5,48E-06
TRMT10A	1,603902	0,000216

SKA2	1,603899	4,94E-11
FKBPL	1,6036	5,50E-07
RMND5A	1,603574	6,27E-12
SRPK2	1,599865	1,08E-12
SMG8	1,599627	8,06E-10
JRK	1,599064	1,82E-07
VPS72	1,598049	8,98E-08
MTERF1	1,596817	1,95E-05
IMPDH2	1,596411	2,47E-10
FBXO21	1,595206	2,35E-10
EXOC3	1,595074	7,30E-09
CAMSAP3	1,595057	7,26E-12
TAPT1	1,593933	0,000458
ZNF680	1,590513	3,94E-06
CHD3	1,588036	2,61E-13
IFT88	1,586817	2,06E-05
CRBN	1,585955	1,16E-05
NFIA	1,584741	5,60E-06
DLG3	1,584053	1,36E-11
AEN	1,58384	9,09E-10
ZNF429	1,583687	7,74E-05
KLHL12	1,58227	4,91E-09
AP1G1	1,580792	2,31E-08
NAPEPLD	1,580116	0,000806
SLC25A11	1,579914	5,77E-12
DECR1	1,579235	3,75E-11
KLF5	1,578457	4,63E-13
ZDHHC24	1,578153	2,52E-05
IER5L	1,573655	8,72E-05
GSK3B	1,57347	3,79E-13
AKT1	1,572954	1,38E-08
ZKSCAN5	1,572951	2,05E-09
PANK1	1,572893	1,05E-07
BRD8	1,572561	7,13E-07
TIMELESS	1,570645	8,30E-10
RALGAPA1	1,570056	1,33E-08
CBX1	1,568354	1,45E-10
TMEM260	1,567449	5,86E-05
YEATS4	1,566484	1,78E-05
TMEM14A	1,566232	6,60E-06
THAP5	1,565833	2,75E-07
ADGRA3	1,56565	7,77E-06
UBE3C	1,565538	5,59E-09
TATDN2	1,565316	5,56E-09
TOR3A	1,56394	7,79E-10
NET1	1,563598	6,19E-05
FUT8	1,562643	1,47E-11
G6PC3	1,561988	6,01E-10
C21orf58	1,560576	0,000691
FGD5-AS1	1,559635	9,09E-12

GINS2	1,559329	3,51E-12
PRDM15	1,558076	1,90E-06
CDS1	1,557831	9,69E-08
GNPNAT1	1,557579	6,42E-12
RPS3A	1,557509	3,45E-07
NCOA2	1,557459	8,46E-06
CTCF	1,557412	3,36E-13
HARS2	1,556996	6,75E-12
MTX3	1,55645	0,000833
UQCRC2	1,552832	1,42E-15
SMARCC1	1,552275	4,06E-17
UBTF	1,551935	2,15E-08
FUK	1,551481	0,000536
DCAF11	1,550907	4,03E-06
THUMPD1	1,550876	9,65E-11
ARFIP1	1,550876	3,79E-08
CTU2	1,54964	3,95E-09
RPL13	1,549544	3,70E-12
LRBA	1,546792	1,92E-07
ATP5D	1,546325	1,86E-16
CAMK2G	1,545143	1,14E-08
COQ7	1,544863	0,000165
FRS2	1,543178	1,58E-06
STYX	1,542021	3,47E-08
PBX2	1,541452	3,92E-15
MVD	1,541358	0,000105
SLC19A1	1,541313	6,39E-08
NBEAL2	1,540958	4,64E-07
PPAP2C	1,540694	5,81E-07
NUDCD2	1,540145	1,62E-08
ZDHHC12	1,539971	2,38E-07
BAMBI	1,538751	2,31E-15
MPV17L2	1,536652	2,93E-09
TAF11	1,536219	6,52E-10
KAT7	1,534244	1,77E-10
NRAS	1,533787	2,57E-09
MED28	1,532675	3,03E-08
ENTPD5	1,532599	0,000139
N4BP2	1,532472	1,10E-05
PSKH1	1,531849	3,60E-09
IREB2	1,531447	2,92E-14
NAA40	1,530941	2,03E-07
ATP5L	1,530691	7,79E-17
OXLD1	1,53001	7,18E-05
NARS2	1,528458	3,59E-07
CRIP2	1,528151	9,56E-13
SYPL1	1,527971	8,32E-09
CIPC	1,526829	1,43E-06
UNK	1,526779	2,59E-06
NDUFA5	1,526553	3,62E-07

NIPAL2	1,526329	0,001005
TRIAP1	1,521321	1,05E-07
DPAGT1	1,52034	1,03E-08
PRKRIR	1,51842	1,27E-10
ZC3H10	1,518136	0,000101
SNX18	1,51802	5,41E-06
C2CD3	1,517796	1,51E-08
BRF1	1,514499	6,08E-06
HAUS1	1,511718	2,52E-06
DBF4	1,511018	1,42E-10
PRIM1	1,508136	6,58E-06
SPAG5	1,507444	3,77E-09
NFIB	1,507135	1,03E-07
WDR59	1,506864	7,09E-08
SF3B3	1,506316	3,53E-10
SDHA	1,505773	1,84E-07
HELLS	1,505555	1,15E-07
WRB	1,504805	1,91E-06
CEP131	1,503016	2,51E-05
GBAP1	1,501805	0,000309
LYPLAL1	1,499304	0,000141
FAM83D	1,499034	1,68E-08
MEX3B	1,498633	6,27E-07
SRCAP	1,498364	7,39E-17
ACOT13	1,497944	2,37E-07
EAF1	1,497713	1,96E-07
TOP2A	1,497585	3,47E-08
POLD3	1,496776	8,68E-10
COA5	1,496459	3,70E-07
SPATA33	1,495411	1,59E-07
KCTD3	1,494785	3,87E-10
C9orf156	1,49197	1,45E-05
IFT74	1,491646	0,000151
HMGB3	1,490049	5,58E-12
KIAA0195	1,487168	1,35E-10
ZC3H6	1,486784	0,000698
TMEM132/	1,485725	1,31E-05
INTS5	1,485337	8,93E-06
RPUSD3	1,485247	5,66E-09
DCAF8	1,485166	1,54E-08
ARF3	1,484554	6,16E-11
SLC37A4	1,481307	2,73E-12
CBLL1	1,481154	8,20E-11
LYSMD1	1,4811	5,10E-05
C2CD2L	1,480729	0,000287
C11orf73	1,480689	6,64E-07
MTHFD1	1,480355	2,14E-17
MTIF3	1,48026	5,61E-07
PAN3	1,479539	1,14E-05
H3F3B	1,478748	1,36E-16

NLK	1,477695	3,06E-06
FBXO22	1,477249	5,25E-09
RPS15A	1,47702	4,02E-11
NAA50	1,475677	2,31E-15
STRBP	1,475344	3,67E-10
TCF25	1,474642	9,13E-11
TM7SF3	1,472773	8,17E-07
SEL1L	1,472073	9,23E-10
SMAD1	1,472049	4,96E-09
TRIM65	1,470091	0,000936
XPOT	1,469803	7,86E-09
ZNF165	1,468712	2,15E-06
CNIH1	1,467036	4,96E-10
SGOL1	1,466652	6,10E-07
EBPL	1,466524	0,000339
TMA16	1,465188	4,00E-06
BRD3	1,464941	1,22E-08
PIGS	1,463319	0,00056
ORC5	1,462211	4,33E-05
ALG8	1,461654	4,25E-05
ZNF43	1,461442	8,16E-06
GCDH	1,460469	8,29E-08
SREBF1	1,460291	4,48E-08
ASPM	1,459763	7,99E-08
C3orf38	1,459485	3,16E-08
TMEM186	1,457195	8,45E-05
ADCK1	1,45629	0,000102
RHOD	1,456277	0,000347
NFRKB	1,456031	3,02E-08
TMOD2	1,45544	0,000564
RALGDS	1,45498	7,52E-09
UBFD1	1,454744	3,44E-09
TRAPPC13	1,453642	4,70E-07
INSIG1	1,453364	1,95E-05
AKAP9	1,450365	3,10E-05
HMGB2	1,449195	2,42E-05
SIN3A	1,44846	3,15E-14
C17orf62	1,448233	1,44E-10
MPHOSPH9	1,447105	2,03E-09
GSKIP	1,446683	1,66E-06
TPX2	1,44655	3,73E-13
XRCC3	1,44542	0,000754
ZBTB22	1,444922	2,93E-05
GATAD1	1,44465	2,65E-06
ATG7	1,443812	0,000202
NARF	1,443655	0,000376
POLR2J	1,442296	1,16E-08
DHX29	1,441889	3,84E-08
PPP1R13B	1,441111	8,33E-07
ERCC4	1,440013	0,000252

TRIM28	1,439081	1,26E-12
TOP1	1,438726	1,01E-14
FAM134C	1,438041	8,71E-08
DHPS	1,437503	2,26E-10
ATP5I	1,437483	4,35E-13
TSPAN17	1,437025	1,14E-06
TMEM231	1,435783	1,06E-05
GEMIN5	1,435278	4,88E-12
WWP1	1,434779	0,000158
KBTBD6	1,434659	4,74E-08
GPN3	1,433713	7,67E-06
ATP8B1	1,433191	2,41E-07
PRMT5	1,432221	4,70E-12
DDB1	1,431467	3,12E-07
TSC22D1	1,431329	1,25E-06
ZNF273	1,431183	0,00041
MGMT	1,43071	1,86E-05
SLC5A6	1,430352	9,36E-06
PRDM4	1,428717	6,97E-15
ZNRF1	1,427932	2,28E-11
OAZ2	1,424151	4,13E-07
ATG14	1,423419	0,000155
TUFM	1,422227	3,02E-11
ALKBH7	1,422226	2,52E-08
SGPL1	1,420991	2,44E-10
INCENP	1,420979	2,46E-09
CNNM3	1,420368	4,90E-08
PACS2	1,415982	3,21E-11
CMTR2	1,415341	3,00E-07
AP5S1	1,415166	2,23E-07
RBM8A	1,414625	7,82E-12
SLC25A1	1,413472	6,83E-07
GCA	1,412324	4,78E-05
EIF4EBP2	1,412174	4,76E-06
NUBP2	1,410217	2,30E-10
SETD6	1,409432	0,000369
ATIC	1,408811	1,75E-14
CYP51A1	1,407572	3,63E-13
RINT1	1,404787	3,33E-06
ACAA1	1,404219	0,000197
DPY19L3	1,403122	2,22E-06
CLCC1	1,401977	5,14E-07
PNPO	1,399825	1,72E-09
FANCL	1,398884	0,000885
RXRB	1,39617	0,000356
MARCKSL1	1,395911	5,36E-15
USP38	1,393694	5,37E-06
KIAA0556	1,393675	1,89E-07
HRSP12	1,3936	0,000282
PRR11	1,393008	2,12E-08

RCOR1	1,390702	3,24E-08
TSPYL1	1,390692	3,16E-07
KIZ	1,390021	0,000562
NRDE2	1,389538	2,32E-05
PCGF3	1,388204	1,44E-10
CCDC117	1,386835	8,77E-05
PAK2	1,385159	1,59E-11
REST	1,384547	3,51E-06
GSTCD	1,383693	0,000106
ACSL3	1,38337	4,51E-05
DECR2	1,383308	2,25E-06
FXR1	1,381582	1,12E-07
LANCL1	1,380735	5,62E-07
PAX8-AS1	1,380526	4,90E-05
VMP1	1,379565	4,71E-07
FAM168A	1,379073	2,75E-06
C14orf1	1,377467	0,000711
SNRPE	1,375647	1,55E-13
CASP6	1,374165	1,32E-05
UBB	1,371921	3,99E-11
RPL35A	1,371857	2,89E-08
MDH2	1,368961	1,01E-09
THOC6	1,36739	0,000137
SARAF	1,367111	3,94E-08
CAPN7	1,365735	5,85E-08
METTL14	1,365124	0,000302
POLR3GL	1,364345	6,12E-05
NUBPL	1,362999	0,000143
PYCARD	1,362753	0,000665
APOA1BP	1,362523	7,48E-09
TMEM101	1,360488	1,24E-06
HEXIM1	1,35982	8,03E-06
BCL6	1,358281	0,000185
NDUFC1	1,358095	3,65E-07
SLC19A2	1,357057	0,000528
KIAA0101	1,355734	1,34E-07
MAPK8IP1	1,355351	2,03E-06
BIRC5	1,354321	3,94E-05
HS6ST1	1,352794	1,39E-07
PAPOLA	1,352557	1,05E-11
NOM1	1,349502	1,01E-06
CHTF8	1,348731	1,16E-09
TYSND1	1,348544	3,47E-08
CHD8	1,348377	3,38E-08
MXD3	1,347869	0,000107
TCTN1	1,347641	6,86E-06
TMEM251	1,347494	4,84E-07
CHMP2B	1,346928	1,30E-08
COX14	1,346842	5,21E-06
PEX13	1,346404	0,000255

HYAL2	1,34626	3,31E-09
PPP2R5E	1,345812	1,43E-11
BBS10	1,345413	0,000381
TRAF4	1,344913	1,72E-10
ZBTB6	1,344903	0,000189
TRIP11	1,342515	2,01E-08
NCAPG2	1,341047	2,71E-08
PSMD4	1,340787	3,66E-11
SS18L1	1,340449	0,000361
HECTD1	1,340403	2,85E-11
GPATCH2	1,339136	3,41E-06
SREBF2	1,338984	6,24E-10
LTA4H	1,337675	2,83E-07
ZNF592	1,337101	7,65E-07
XXYLT1	1,332432	1,27E-09
NUMA1	1,331581	2,42E-11
SLF1	1,330417	0,000406
NAAA	1,329781	0,001001
FBXO34	1,329406	8,75E-07
ETNK1	1,328154	3,60E-05
FADS2	1,327203	0,000199
TAF6L	1,326101	3,46E-05
ECE2	1,325018	2,38E-05
SLC25A40	1,324173	0,000182
NDUFB5	1,323333	1,75E-05
TRAP1	1,322545	4,57E-11
TELO2	1,321965	3,51E-07
SORD	1,321902	1,75E-11
KIAA0141	1,321662	7,65E-07
P4HTM	1,321523	1,50E-05
ZNF718	1,320868	0,000192
AURKA	1,320276	1,17E-08
LMLN	1,320231	0,000122
ARL6IP1	1,319145	3,12E-10
CDCA4	1,319108	0,000265
TECPR2	1,318749	2,30E-05
ZNF783	1,318257	0,00015
WDR83OS	1,316517	3,52E-10
ACOX3	1,315738	0,000236
TRMT2B	1,314075	1,52E-05
EDC3	1,313587	1,12E-06
ADIPOR1	1,313432	2,27E-05
RIOK2	1,313322	6,91E-08
KIF22	1,313112	6,99E-05
FLYWCH2	1,312794	1,46E-06
SLC20A2	1,310602	3,74E-06
GPD1L	1,310551	6,76E-05
HNRNPLL	1,310284	1,04E-07
ALDOC	1,30971	0,0007
RNMTL1	1,308092	6,80E-06

CPSF2	1,307952	3,55E-10
PCCB	1,307547	1,29E-07
SOCS7	1,306699	0,000976
DCUN1D1	1,306432	3,60E-05
CENPH	1,305474	0,000608
SNHG16	1,303693	9,11E-11
ARHGEF16	1,302929	0,000536
EVL	1,302679	2,09E-06
HMGCR	1,301461	2,82E-08
YLPM1	1,301027	1,36E-09
HMGN1	1,300111	5,69E-12
AKAP1	1,299846	4,02E-07
POLR2A	1,299807	1,67E-12
ITFG3	1,299671	9,97E-06
RBMX	1,297676	9,97E-08
ARFIP2	1,296568	8,71E-06
ALKBH2	1,296374	1,15E-06
EEF1G	1,296362	1,31E-13
GALK1	1,296142	1,74E-05
CRNKL1	1,295099	1,28E-05
ADPRHL1	1,294181	0,000865
THAP11	1,294111	1,78E-05
ZNF607	1,2936	0,000413
TNRC6A	1,292037	7,75E-07
SLC7A5	1,291531	8,91E-10
COX17	1,29045	0,000355
MIDN	1,290098	4,62E-09
GFOD2	1,289355	3,72E-05
PGP	1,288156	7,16E-08
METTL16	1,287127	1,88E-08
PLA2G12A	1,285576	1,57E-05
ZBTB1	1,284149	2,15E-08
SLC25A38	1,283504	3,23E-07
AMZ2	1,282364	4,90E-05
COX11	1,281683	0,000299
VPS25	1,281216	1,35E-08
CISD3	1,280727	0,000255
ZBTB9	1,280236	2,98E-07
MAP2K5	1,279888	4,04E-06
CMC1	1,279533	0,000256
ATP5J2	1,27656	1,55E-05
HIF1A	1,275344	2,71E-10
TOMM6	1,275223	3,73E-11
CBWD2	1,272563	1,95E-05
CPSF4	1,272375	0,000429
ARMT1	1,27085	6,44E-08
NCBP1	1,269894	8,21E-10
LPCAT1	1,268922	8,22E-07
SUCLG1	1,267811	1,30E-09
ZNF322	1,267427	0,000253

ZNF614	1,265344	0,000385
RPS27	1,265331	3,27E-05
STIM2	1,264542	1,31E-06
ATXN7	1,263339	4,75E-06
PLCB1	1,26273	0,000611
GEMIN2	1,262287	0,000267
CLOCK	1,262274	1,72E-06
TET3	1,260659	1,13E-08
VPS13A	1,259199	4,95E-05
NARFL	1,258766	5,68E-05
SCAP	1,255135	5,59E-06
EMC9	1,255015	0,000231
PIGX	1,254955	8,96E-08
CHCHD10	1,254216	9,72E-09
MAPK13	1,253686	2,81E-07
ATF6B	1,25243	7,87E-08
RHNO1	1,252408	2,63E-05
FAM122B	1,251379	1,52E-06
SHFM1	1,251091	3,74E-10
NUSAP1	1,251022	8,03E-06
FDFT1	1,250176	1,45E-05
MAML1	1,249439	1,86E-06
LEMD3	1,249196	1,85E-07
UBR7	1,248824	7,38E-08
C1orf43	1,248756	2,18E-09
TRAPPC9	1,248003	0,000617
CNOT2	1,247177	1,09E-09
ITPK1	1,246647	2,91E-11
RNF115	1,246283	2,66E-08
HDHD2	1,245454	0,000252
MAL2	1,245304	4,55E-08
PROSER3	1,244905	9,50E-05
TRMT10C	1,243779	6,59E-07
CUL7	1,243416	5,30E-05
HAGHL	1,243169	2,12E-05
POMT1	1,242573	0,000977
SH2B1	1,242175	0,000876
GEMIN4	1,241332	8,46E-08
OXNAD1	1,24086	5,13E-05
GATC	1,239344	4,06E-08
MSRB2	1,237691	8,42E-07
FANCD2	1,237158	1,58E-05
SUMO3	1,236086	7,68E-11
MSH3	1,235645	3,10E-06
MREG	1,23485	2,75E-06
FAM217B	1,233699	1,38E-05
PMS2P1	1,233199	0,000255
CCNB1	1,232873	1,83E-08
MTSS1L	1,232725	1,27E-05
UBP1	1,232712	6,30E-08

ATP5G2	1,232457	1,59E-10
RASA1	1,230847	4,70E-05
THOC3	1,230315	0,000123
JMJD8	1,230209	1,66E-07
LAMTOR2	1,229904	7,34E-07
RNF168	1,229775	1,69E-08
AUTS2	1,229445	0,000215
MKI67	1,228997	6,13E-09
PDCD4	1,228866	3,20E-06
DPY19L1	1,225994	0,000306
RNF123	1,225713	7,18E-05
PRKAR1A	1,22448	5,20E-09
SPATS2	1,223352	1,53E-06
LETMD1	1,222458	3,32E-07
POLR2G	1,220925	8,23E-09
DIS3L	1,220881	4,46E-05
GTF2A1	1,219848	1,20E-07
RFK	1,217075	1,22E-05
LRCH3	1,216121	8,63E-05
CENPE	1,214382	7,56E-05
ARF5	1,213618	4,62E-10
NDUFS2	1,210834	3,09E-05
NSF	1,210545	0,000489
MRPL12	1,210074	2,84E-09
ACSF3	1,210067	3,02E-07
PYCR1	1,20859	1,74E-05
ARVCF	1,206094	0,000351
PAFAH1B3	1,205097	9,37E-06
IFT46	1,204674	5,61E-07
CHRNA5	1,204471	1,84E-06
IER5	1,204003	4,53E-05
SMYD5	1,199818	4,18E-07
CDC42SE1	1,199488	6,21E-07
STX17	1,198314	3,94E-06
TMED3	1,196104	2,97E-06
VPRBP	1,194958	8,07E-07
SMARCC2	1,194805	1,31E-09
SCAF8	1,193184	0,000229
SFXN1	1,191878	3,69E-06
PCDH1	1,191505	0,000202
SOS1	1,190686	5,96E-06
CHMP1B	1,190306	4,92E-07
ZNF740	1,189712	0,000994
CUL3	1,188929	6,72E-08
PHF10	1,18739	8,53E-05
NDUFAF7	1,185858	0,000477
SCOC	1,185115	0,000257
ZNF646	1,184286	8,87E-07
RNF40	1,184128	7,96E-07
KDM3B	1,183471	1,31E-07

PRR14L	1,182613	4,17E-08
ELF2	1,181337	0,000159
GTF3C1	1,181256	0,000126
TXN2	1,181197	0,000157
LSM3	1,180799	2,69E-06
SLC25A13	1,179708	1,13E-05
ZNF260	1,17958	5,04E-05
UQCC2	1,179546	8,72E-05
MAVS	1,179132	2,43E-06
CSTF2T	1,178265	1,38E-05
TNK1	1,177751	9,90E-05
FAM120B	1,173693	0,000113
TMEM241	1,173473	0,000555
COA4	1,173259	9,42E-09
TMEM2	1,17293	2,92E-05
PPM1B	1,172509	6,01E-05
ATMIN	1,172473	8,39E-09
ZBTB33	1,17208	0,00054
PREPL	1,171741	4,46E-06
LSG1	1,171601	7,94E-08
WDR48	1,170793	0,000635
CCBL2	1,168214	0,000651
BCKDHA	1,166088	2,08E-05
RBM4	1,165711	3,92E-07
MRPS27	1,165555	5,20E-08
SNRNP48	1,165134	4,47E-05
MRPL42	1,164356	3,55E-05
RECQL5	1,161213	3,19E-05
RPL32	1,16078	2,57E-07
FANCG	1,160681	0,000289
HIGD2A	1,158269	1,91E-06
KCTD13	1,156896	0,000453
NAA38	1,156185	1,22E-06
SLC35E2B	1,156015	9,81E-06
H3F3AP4	1,15543	3,75E-06
SMTN	1,154575	7,79E-07
JTB	1,152911	1,54E-06
PRPF8	1,15152	7,35E-08
GPSM2	1,150346	1,74E-07
PSMC5	1,149171	4,20E-06
NUDT14	1,14489	6,98E-05
NBR1	1,1435	0,000422
TRAPPC4	1,143156	1,27E-05
TRIM56	1,143143	0,000676
ZNF627	1,142445	1,11E-05
ZMYND8	1,141562	1,33E-07
ZNF346	1,141179	0,000573
SLC25A15	1,140864	5,64E-06
DHX8	1,140549	0,000209
PPP4R3A	1,140546	3,74E-09

C16orf13	1,139215	2,91E-06
AVL9	1,138983	4,44E-08
RFX7	1,137923	8,65E-05
RPL21	1,137358	2,61E-07
DARS2	1,13711	3,91E-08
POLR1D	1,136978	1,73E-08
TNRC18	1,136708	1,49E-07
SETD5	1,135578	3,11E-05
SETD1A	1,13506	6,15E-06
GPHN	1,133969	2,65E-05
DCTN5	1,133418	0,000173
ELP5	1,133122	9,99E-07
PIK3R4	1,130281	5,31E-05
LDLR	1,130251	2,01E-06
UBE2T	1,129872	7,79E-07
DNAJC2	1,129665	3,04E-07
POP5	1,129207	0,000516
MED21	1,129081	0,000181
CHCHD4	1,128929	0,000988
ZNF689	1,127103	0,000348
IMP3	1,127061	2,32E-07
DNAJC30	1,127015	1,56E-05
SNRPA1	1,12647	1,00E-07
TDG	1,126411	1,60E-06
BCL9L	1,126139	0,000102
ETFB	1,124818	0,000715
CRACR2B	1,12473	0,000724
SNN	1,123573	0,000431
DAG1	1,123188	2,23E-05
FAM195A	1,121782	1,25E-06
MRPL53	1,121691	0,000761
METTL9	1,121533	2,03E-08
PDE7A	1,121482	0,000263
KITLG	1,121068	4,95E-06
OPA1	1,119001	1,43E-06
STRN3	1,117751	1,51E-05
CTDSPL	1,117594	0,000102
ATP2C1	1,116917	3,10E-08
WDR20	1,115811	1,59E-05
TRPT1	1,115072	0,000869
MRPL57	1,114994	2,43E-07
SFXN4	1,114673	4,20E-06
C20orf27	1,114515	5,35E-07
THOC2	1,114303	6,66E-07
ZNF701	1,11233	0,000878
METAP1	1,110596	2,91E-06
ALDH7A1	1,110244	1,23E-06
SNAPIN	1,108446	2,78E-05
NCAPD3	1,108087	0,000265
RPIA	1,107928	6,99E-05

PPP4R2	1,10526	1,47E-06
COQ2	1,104729	4,75E-05
TRMT112	1,104185	4,68E-05
MSH6	1,103499	1,45E-08
HMGN3	1,103493	5,91E-05
MCM4	1,103443	3,70E-07
MAT2B	1,103419	0,000825
C19orf54	1,103302	0,000197
VEZF1	1,102749	6,34E-06
ABCE1	1,102423	5,79E-07
BTBD3	1,101842	0,00065
AP5M1	1,100788	0,000686
ZNF638	1,100458	9,26E-07
ATPAF1	1,099165	4,17E-05
NTHL1	1,098877	0,000214
CENPU	1,098731	0,00096
FAM110A	1,098349	0,000491
TAF8	1,097753	0,000523
C17orf85	1,096165	9,66E-06
ANP32B	1,096142	4,92E-06
CDK8	1,09555	5,91E-06
ANKRD28	1,0941	1,44E-06
RRBP1	1,093572	0,000642
AHCY	1,091541	4,38E-05
BCL9	1,090854	2,47E-05
TACC3	1,089866	0,00012
CBX5	1,089753	9,92E-06
GOT2	1,088644	1,45E-05
ARFGAP2	1,087873	5,16E-06
SFI1	1,087567	0,000686
DCAF16	1,08713	0,000173
C16orf58	1,084876	1,07E-07
CMTM4	1,084477	2,78E-07
SPRYD3	1,082825	1,44E-05
NUP160	1,081675	8,21E-06
PDS5A	1,081151	2,78E-06
PMVK	1,080662	5,34E-05
RPSAP58	1,080164	7,53E-06
PMPCB	1,078867	1,19E-07
PSMB4	1,078137	3,27E-06
CLUH	1,075497	5,61E-07
DHX38	1,075474	2,51E-06
POLR1A	1,074099	8,08E-06
CKB	1,072861	0,000757
TCF3	1,072688	5,32E-08
FAF2	1,072512	5,56E-07
NADK2	1,07246	4,75E-05
SPIDR	1,071744	5,01E-05
AFG3L1P	1,069227	0,000622
IMPA2	1,069203	1,21E-05

SPAST	1,063121	0,00052
ASH1L	1,062572	1,87E-07
CFDP1	1,061949	2,64E-06
FANCA	1,061018	7,71E-06
MCM3	1,059622	8,77E-06
C5orf22	1,058821	4,71E-05
TMEM5	1,058431	6,27E-06
IPO4	1,057625	3,67E-06
CCND1	1,056248	9,44E-05
MEAF6	1,055935	0,00078
QTRTD1	1,055452	7,01E-05
CHMP1A	1,054981	0,000102
PPIL1	1,054627	2,53E-06
RPS18	1,0543	0,000518
USP22	1,053916	1,33E-06
PAPSS1	1,052014	0,00039
PSPC1	1,051442	5,17E-06
EXD2	1,051429	5,82E-06
RPL15	1,05007	7,76E-08
SNX5	1,049908	2,78E-05
CCAR2	1,049541	3,09E-05
MAGEF1	1,04911	8,20E-06
KPNA2	1,048603	9,43E-07
EHMT1	1,048385	3,95E-05
DEPDC5	1,047005	0,000209
TRIM36	1,04516	0,000955
NKRF	1,044793	1,49E-05
STON2	1,043812	8,16E-05
CSTF1	1,040708	4,04E-06
ZNF398	1,039126	1,64E-06
TNNT1	1,038009	5,22E-07
PDAP1	1,03787	2,64E-07
ANP32E	1,037738	3,87E-06
METTL6	1,037665	6,40E-05
KLHDC4	1,037369	1,44E-05
KMT2C	1,036212	4,03E-07
GRB2	1,035887	5,76E-08
RAB4A	1,035526	0,0001
LIG1	1,035297	2,08E-06
AGGF1	1,034894	1,47E-05
MRFAP1	1,033714	1,27E-06
MTA2	1,03322	2,16E-07
TATDN1	1,033108	5,47E-06
ERI2	1,032915	0,00031
ZNF616	1,032787	0,000191
RPL23A	1,032174	1,51E-07
UBQLN4	1,031384	1,50E-06
TSFM	1,029812	6,43E-05
SLC7A11	1,028523	0,000447
SP1	1,027297	3,68E-06

ERBB2IP	1,026729	1,14E-05
TCTN3	1,025199	7,84E-05
NDUFA2	1,023191	0,000148
SUPT16H	1,022944	2,37E-08
THADA	1,022529	0,000173
FAHD1	1,02243	2,66E-06
POLB	1,020936	0,000806
SLC25A39	1,020862	6,03E-08
SDHD	1,020516	0,000209
C6orf136	1,018946	0,000697
NFYA	1,017656	0,000255
TSEN54	1,017545	0,00011
SLC50A1	1,014836	3,15E-05
MRPL10	1,01411	7,15E-05
TOMM70A	1,01328	1,12E-06
DYM	1,01294	1,27E-05
CDC25B	1,012934	1,60E-05
ZNF764	1,012443	0,000904
CCNA2	1,011935	4,35E-06
FBXO9	1,011894	2,16E-05
WWP2	1,011845	0,00027
IQSEC1	1,011795	6,34E-05
PSMD12	1,010826	0,0003
GRHPR	1,009347	0,000781
RPP30	1,009223	0,000664
DCLRE1B	1,007766	0,000175
NDUFA3	1,007697	0,000236
MBD4	1,00757	0,00019
RAF1	1,006101	4,62E-06
FBXO45	1,005607	0,000691
ZCCHC14	1,005412	1,37E-06
HNRNPH1	1,005066	8,54E-05
RPSA	1,004963	2,54E-07
FLOT2	1,004713	0,000226
TRAPPC2L	1,004521	9,67E-06
TUBG1	1,002882	1,43E-05
ACTL6A	1,002552	1,24E-05
TMEM184(	1,001715	4,98E-05
GRK6	1,001278	0,000204
USP19	1,000682	0,000643
DUS1L	1,000559	8,59E-05
PSMD7	0,998745	4,98E-07
SOCS4	0,99533	0,000903
C7orf73	0,994679	6,75E-06
TROVE2	0,993835	0,000962
RPL34	0,993274	1,13E-05
C5orf30	0,992758	0,000338
SUV420H1	0,99239	1,14E-05
SEPHS1	0,992285	0,000119
WDR60	0,991881	0,000275

NFATC3	0,991524	1,66E-05
ZNF148	0,990391	1,32E-05
ZCRB1	0,990386	8,96E-05
MRPS7	0,99021	6,17E-05
TBC1D24	0,988809	0,000782
XPO1	0,988672	1,72E-05
FAM189B	0,987079	0,000634
HACD3	0,986369	1,59E-07
RNASEH2A	0,986225	1,85E-06
ARRDC1	0,983377	5,64E-05
HNRNPAB	0,981211	1,82E-07
RPL4	0,978881	3,19E-07
NSDHL	0,977942	3,55E-05
IP6K2	0,976103	0,000989
ATL3	0,975518	2,04E-06
NPM1	0,974338	1,50E-07
USP6NL	0,973844	3,44E-05
CPSF6	0,973359	2,14E-07
MSH2	0,973338	1,15E-05
TBK1	0,973136	0,000916
MLST8	0,972182	7,06E-06
UBAC1	0,97205	2,65E-05
HNRNPA0	0,969564	2,39E-05
TTLL5	0,969136	3,15E-05
RPS2	0,968945	1,15E-07
KIF2A	0,968795	4,39E-06
SYTL2	0,968772	0,000517
CDT1	0,968347	0,000905
UBR2	0,968076	5,82E-05
TTC19	0,967264	5,41E-05
KIF11	0,966865	5,52E-06
KRIT1	0,964069	0,000577
ZFYVE21	0,961989	0,000141
LDB1	0,958544	2,62E-05
G3BP2	0,9578	0,000321
PIP5K1A	0,955671	0,000555
SETD3	0,955643	8,41E-06
ASUN	0,955639	6,99E-05
SF3B4	0,955397	5,34E-08
HNRNPA1	0,955043	4,16E-07
TMEM167A	0,952657	0,000319
RMND1	0,952212	0,000126
SMG7	0,952203	2,69E-07
CETN2	0,951547	6,33E-05
VPS37C	0,950809	0,000253
KLHL42	0,950041	0,000553
USP39	0,947852	1,12E-06
RCN2	0,947265	3,86E-05
ATP5H	0,947121	7,07E-05
APH1A	0,946785	5,41E-06

KDM1A	0,946242	5,99E-06
SLC25A5	0,946022	0,000665
PHKG2	0,945826	0,000125
PCF11	0,945778	0,000665
CLCN3	0,945623	3,30E-05
ILF2	0,944779	1,30E-07
GMPR2	0,943688	0,00039
CREBBP	0,941467	0,000148
SLC6A6	0,940249	2,22E-05
CMTM6	0,939202	0,000443
HOXC10	0,937456	0,000291
METRN	0,937411	0,00027
NIT2	0,93741	0,000132
SUMO2	0,936673	1,24E-06
CUX1	0,935975	1,14E-05
FXR2	0,935114	6,04E-05
ERAL1	0,934722	1,83E-05
PIGG	0,934679	0,001012
NDUFB4	0,9337	1,78E-05
TBC1D9B	0,933636	9,05E-06
MATR3	0,932048	5,04E-07
TBC1D14	0,932007	0,000104
CUL9	0,931709	0,000299
USP42	0,930455	0,000398
POLR3E	0,928508	0,000137
PPP2R5C	0,928219	2,02E-05
FAM120AC	0,92715	0,000135
PNMA1	0,92702	0,000539
NDUFV3	0,926618	1,18E-05
DGCR6L	0,926341	3,64E-05
PLK4	0,925722	0,000535
MARCH6	0,925395	0,000268
UBE2K	0,924441	0,000236
SLC3A2	0,923597	6,02E-06
ARPC1A	0,923558	0,000248
PELP1	0,921783	9,66E-05
RANBP9	0,921616	0,000178
ZNF24	0,91892	1,84E-05
THUMPD3	0,916723	3,78E-05
PTK7	0,915465	4,94E-05
LDOC1L	0,915269	1,69E-05
AKAP11	0,913476	2,64E-05
NDUFS3	0,913096	3,56E-05
TEX2	0,912744	1,65E-05
DSC2	0,91132	0,000194
VPS33B	0,910764	0,000954
HDGF	0,908729	2,69E-07
CDCA3	0,908422	0,000115
CCDC90B	0,907717	0,00057
USP7	0,906089	8,78E-07

TPBG	0,905978	2,80E-05
ATP5B	0,904058	2,09E-05
CLPX	0,903883	0,000177
SPG21	0,903283	1,76E-06
MTA3	0,901535	0,000125
FAM120A	0,89682	0,000182
CPNE3	0,896782	3,89E-05
SNRPF	0,896608	3,97E-05
BRPF3	0,894383	6,61E-05
MANEAL	0,893085	0,000911
CSNK1G2	0,892617	0,000149
PPP6R3	0,889418	1,09E-05
QARS	0,889079	1,94E-05
POLA1	0,887416	0,000612
UQCC3	0,887072	0,00021
BRCA1	0,886475	0,000339
RMDN1	0,886435	0,000434
CCNI	0,884386	1,69E-06
CKAP5	0,884333	0,000293
RPL41	0,884149	1,29E-05
STK38	0,883626	0,000127
MRPS34	0,881822	7,74E-06
BCL7C	0,881185	0,000181
ZNF609	0,880111	0,000298
DHX9	0,87904	6,25E-06
NEDD1	0,878604	0,000122
RBM12	0,87801	0,000617
MCL1	0,877619	1,33E-06
SCO1	0,877402	0,00037
TDP1	0,875681	0,000202
ACAD9	0,875293	0,00096
ZNF664	0,875195	0,000186
PEMT	0,874931	0,000476
MDC1	0,874467	3,59E-05
PSMC2	0,872628	1,40E-05
INPPL1	0,872358	1,43E-05
SMARCA5	0,871532	5,19E-06
RHOT2	0,871464	9,43E-05
POLR2B	0,871054	1,43E-05
INTS3	0,870949	4,47E-05
RNF181	0,870684	0,000657
C14orf166	0,870147	1,49E-05
CYTH2	0,869585	7,34E-05
XPO7	0,866672	0,000238
FBL	0,866303	1,97E-06
DHX15	0,865379	0,000162
ECSIT	0,865244	0,000402
CSNK1G3	0,864845	0,00072
KCTD20	0,864471	2,11E-05
BCLAF1	0,860609	4,41E-06

AGAP3	0,860475	0,000741
RPL27	0,858281	5,84E-06
SENP5	0,857994	0,000225
ZNF444	0,857207	0,000761
FAM64A	0,855691	0,000772
APEX1	0,855536	7,20E-05
GTF3A	0,855228	8,52E-06
SUDS3	0,853151	0,000528
TCEA3	0,853033	0,000444
GPS1	0,852831	0,000135
RABGAP1	0,851494	0,000135
SRPK1	0,84989	3,02E-05
NAA30	0,84675	0,000577
AK3	0,845702	0,000471
MAD2L1	0,845141	0,000428
RABL6	0,843943	0,000139
ATP5F1	0,84367	9,06E-05
F12	0,841397	0,000453
MRFAP1L1	0,839793	0,000108
SHROOM3	0,839258	0,000981
GTPBP6	0,838257	0,000716
SFPQ	0,837331	6,45E-06
GNPTAB	0,835864	0,000557
XRN2	0,835717	2,92E-05
RNF26	0,832892	0,000505
TBCD	0,827394	0,000245
OXA1L	0,826768	0,000167
GCSH	0,825808	0,000872
VPS45	0,822875	0,000855
CTPS2	0,821583	0,000681
STAG2	0,820721	6,16E-05
PTMA	0,820173	6,17E-05
ASPSCR1	0,819895	0,000651
NDUFS6	0,81697	0,000482
FANCI	0,814383	0,000669
GPX4	0,812326	0,00016
RPL26	0,812281	6,34E-05
UMPS	0,810887	0,00033
PRR12	0,81044	0,000399
FUNDC2	0,808983	0,000546
RBM28	0,808209	0,000481
CDC42BPB	0,807512	9,79E-05
MRRF	0,807127	0,000828
SRGAP2	0,806911	0,000103
COX6C	0,806694	0,000332
CNOT7	0,803589	0,000157
ILVBL	0,802567	9,55E-05
CASC3	0,802126	0,000669
UQCRFS1	0,800936	6,76E-05
JAGN1	0,796114	0,000702

GID8	0,795571	0,000153
EIF4E	0,793637	0,000546
HTATSF1	0,79299	0,000484
BTBD7	0,792613	0,000641
IGF1R	0,792179	0,000397
KMT2D	0,791545	4,58E-05
SIPA1L1	0,78944	0,000175
RBBP5	0,788088	0,000473
BAG1	0,788033	0,000316
RTN3	0,785221	0,00097
AFG3L2	0,784678	0,000343
BRK1	0,783872	0,000418
VPS52	0,78311	8,05E-05
NT5C3B	0,780127	0,000909
RFWD2	0,779421	0,000918
MDK	0,778592	0,000246
SMARCB1	0,775794	0,000843
BTBD2	0,775457	0,000159
CANX	0,771887	0,000138
KARS	0,769304	0,000267
COX6A1	0,768972	7,05E-05
PSMB6	0,768404	0,000689
GON4L	0,768254	0,000719
MLLT6	0,760466	0,000431
FGFRL1	0,75295	0,000941
HN1L	0,752686	8,35E-05
COPS6	0,752608	0,000213
SMIM7	0,751516	0,000757
NOTCH1	0,749299	0,000351
MAPK1IP1l	0,742314	0,000641
SRRM2	0,740818	0,000141
MRPS18B	0,740362	0,000644
CNBP	0,740316	0,000499
RCC1	0,738706	0,000611
TLE3	0,736123	0,000161
SKP1	0,7339	0,000979
MBTPS1	0,733454	0,000938
ANKIB1	0,732342	0,000739
ESYT2	0,722508	0,000391
RPL7A	0,720842	6,57E-05
PSME3	0,720129	0,000265
H2AFZ	0,719822	0,000874
MORF4L1	0,719511	0,000688
ATXN7L3	0,718595	0,000312
SMCHD1	0,718125	0,000705
ANKRD17	0,717851	0,000238
CCDC85B	0,715672	0,000641
PHB	0,713663	0,000154
ZC3H18	0,712665	0,000869
TAF15	0,712628	0,000365

SRSF9	0,71175	0,000545
USP3	0,708795	0,000668
KPNB1	0,706643	0,0001
PRKCI	0,702541	0,000794
LLPH	0,70053	0,00068
COX8A	0,699362	0,000308
TUBB3	0,691647	0,000225
TADA3	0,689322	0,000936
RAD21	0,689247	0,000144
CASP2	0,688629	0,000871
SRSF2	0,68846	0,000482
RPL24	0,681562	0,000583
STUB1	0,676637	0,000741
MRPS21	0,675957	0,00066
ATP9A	0,674121	0,000984
ALYREF	0,671819	0,000459
CCT2	0,670583	0,000688
COX4I1	0,662996	0,000185
FDPS	0,656284	0,000633
PSMC1	0,648895	0,000954
SEC11A	0,647627	0,000715
DBN1	0,640073	0,000555
TAF7	0,637412	0,000989
RPL14	0,637393	0,000819
SMARCE1	0,635747	0,00102
HNRNPK	0,634571	0,000964
HNRNPA2B	0,631167	0,000643
PCBP2	0,617454	0,000655
EIF2S3	-0,61485	0,000861
CHCHD2	-0,62629	0,000829
EPRS	-0,62738	0,000534
RPS20	-0,63637	0,000261
HUWE1	-0,63796	0,000656
MAPK1	-0,65251	0,000973
AES	-0,66191	0,000646
PPIF	-0,67387	0,000845
YWHAH	-0,67707	0,000267
DDX1	-0,67779	0,00033
AATF	-0,68195	0,000707
MRPS15	-0,6842	0,00052
RAP1B	-0,68503	0,000653
BFAR	-0,68815	0,000776
C11orf58	-0,69501	0,000232
MORF4L2	-0,6957	0,000288
GTF2F1	-0,70567	0,000793
SLTM	-0,71003	0,000439
SGTA	-0,71186	0,000164
EDF1	-0,71355	0,00041
TBCB	-0,71569	0,000668
PTK2	-0,71847	0,000188

ESYT1	-0,72109	7,55E-05
HDAC1	-0,72261	0,000232
TMEM259	-0,72388	0,000628
DNAJC11	-0,72565	0,000385
COPB1	-0,72616	0,000368
TMEM248	-0,72971	0,000693
SERP1	-0,73248	3,90E-05
PYGB	-0,73362	0,000188
ZDHHC5	-0,73408	0,000333
PKN1	-0,7398	0,000337
MANF	-0,74221	0,000582
AZIN1	-0,74352	4,01E-05
CHMP4B	-0,74463	0,000721
CCT8	-0,74787	4,98E-05
SERF2	-0,75862	0,000169
NUP62	-0,7598	0,000954
ADRM1	-0,76559	8,64E-05
EIF3G	-0,7665	3,90E-05
ROCK2	-0,76943	0,000394
RLIM	-0,76978	0,000286
KHSRP	-0,77218	5,37E-05
CENPO	-0,77327	0,000487
SNX19	-0,77566	0,000535
TACC1	-0,77582	0,000867
NUDCD3	-0,77887	0,000498
ZDHHC7	-0,77921	0,000561
GFM1	-0,78045	0,000119
MTX2	-0,78265	0,000567
MTMR2	-0,78537	0,000878
VIMP	-0,78692	0,000139
CLSPN	-0,79071	0,000296
ARAP1	-0,79082	0,000769
PSMD13	-0,79232	6,85E-05
BAK1	-0,79894	0,000768
ANXA7	-0,79946	3,13E-05
TSTA3	-0,80014	2,60E-05
PISD	-0,80319	0,000401
BRMS1	-0,80324	0,000471
ZNF579	-0,80327	0,000143
METRNL	-0,8039	0,000977
COMMD7	-0,80857	0,000936
AK2	-0,81133	1,32E-05
PPP1R37	-0,81227	0,000735
SLC52A2	-0,81328	0,000211
EIF3I	-0,81597	0,000169
NSMAF	-0,81705	0,000647
UBA52	-0,82257	3,67E-06
OSGIN2	-0,82422	0,00097
ST13	-0,82465	8,89E-05
DCTD	-0,82597	0,000161

DDX39A	-0,82719	1,79E-05
PLOD3	-0,82748	0,000287
PPP1R9B	-0,82789	1,77E-05
NCKAP1	-0,82815	3,70E-05
BLOC1S3	-0,82869	0,000665
SMC6	-0,82903	0,000934
MED14	-0,83186	0,00018
SERPINH1	-0,83679	0,000139
LTBP3	-0,83724	0,000565
SSSCA1	-0,83823	0,000246
ATXN10	-0,83849	1,86E-05
RBM42	-0,83936	0,000472
DPM1	-0,84092	0,000176
GDI1	-0,84093	2,95E-05
ELOVL5	-0,84213	0,000125
CC2D1B	-0,84448	0,000727
SBNO2	-0,84485	0,000454
RPTOR	-0,84507	3,46E-05
NDOR1	-0,84709	0,000391
EIF3D	-0,84882	0,000124
PPP6R1	-0,84976	0,000104
SHCBP1	-0,85109	0,000756
ELOF1	-0,85163	0,000104
SLC4A1AP	-0,8525	0,000775
ARID1B	-0,85309	0,000505
MAP4	-0,85342	1,71E-06
ASCC2	-0,85378	7,69E-05
AKR1A1	-0,85751	0,000107
TMEM70	-0,86202	0,000317
TSSC4	-0,8636	0,000431
CAB39	-0,865	0,000405
FAR1	-0,86559	0,000667
DPP7	-0,86855	8,11E-06
TOR1B	-0,86933	0,000243
SLC26A2	-0,87008	0,000518
SUPT7L	-0,87433	0,000374
ATP2B1	-0,87459	0,000552
C6orf132	-0,87641	0,000215
PIN1	-0,87726	0,000333
FASTKD5	-0,87822	0,000711
APEX2	-0,87834	0,000247
USP4	-0,8785	0,000377
SEPT11	-0,87978	6,76E-06
PFN1	-0,88142	9,75E-07
C11orf24	-0,88169	5,39E-06
DTL	-0,88229	9,11E-05
NDUFAF3	-0,88435	0,000308
MARS	-0,88437	9,94E-07
CWC15	-0,88445	0,000546
ZNF410	-0,88486	1,53E-05

HMGXB3	-0,88552	7,17E-06
FNDC3A	-0,88701	0,000245
GPAA1	-0,88837	1,29E-05
TMUB1	-0,88984	0,000368
PTP4A1	-0,89241	2,16E-05
EBNA1BP2	-0,89292	3,19E-06
SCRN1	-0,89414	5,90E-06
HEATR1	-0,89467	7,74E-05
PIGT	-0,89516	0,000215
RAB3GAP1	-0,89614	8,17E-06
KIAA0196	-0,89642	9,96E-06
COPRS	-0,89659	0,000374
MAP4K4	-0,89727	2,34E-06
SMG5	-0,89744	3,99E-05
MAGT1	-0,89983	8,76E-05
PSMG3	-0,90029	0,000155
FAM219B	-0,90257	0,000667
PKM	-0,90347	4,98E-05
TTYH3	-0,90396	0,000107
DNM2	-0,90433	5,60E-06
MBOAT7	-0,90457	0,000496
ELMO2	-0,9047	0,000447
XAB2	-0,90592	0,000403
EIF2S2	-0,90626	0,000322
FKBP10	-0,90828	7,76E-07
NOP10	-0,90971	3,30E-05
RNF216	-0,91097	0,000214
TACC2	-0,91331	0,000254
PFN2	-0,91605	1,94E-06
TRAPPC3	-0,91635	0,000275
SHC1	-0,91701	9,38E-07
LAS1L	-0,91704	0,000382
CEP164	-0,9174	9,66E-05
TRIP13	-0,91893	0,000289
SLC9A1	-0,91937	2,65E-05
BICD2	-0,92113	2,13E-05
ORMDL1	-0,92223	0,000129
ZNF358	-0,92249	6,53E-05
FAM21A	-0,92302	6,10E-06
RHOQ	-0,9238	0,000553
POP1	-0,92539	0,00046
RBM3	-0,92737	4,92E-06
RASEF	-0,9277	0,000272
ENTPD6	-0,92966	4,76E-05
USP1	-0,93152	0,000841
FOXP1	-0,93195	0,000298
EP300	-0,93683	0,000204
STRADB	-0,93719	0,000566
OLA1	-0,93807	5,33E-06
AKAP8L	-0,93953	0,000823

TSG101	-0,93962	0,000258
ACADM	-0,94012	0,000392
C19orf47	-0,94205	0,000721
PDIA6	-0,94322	0,000373
MBD2	-0,94391	3,56E-05
FLCN	-0,94399	2,69E-05
CDC42	-0,9449	1,86E-05
MRTO4	-0,94608	1,06E-06
TPP2	-0,94655	0,000129
USP47	-0,94742	3,02E-05
GNL1	-0,9479	1,51E-05
SRSF4	-0,94842	0,000113
LRRC47	-0,95107	0,000993
ZC3H3	-0,95365	0,000345
DDHD2	-0,95481	6,07E-05
ARHGEF18	-0,95704	0,000139
NCOA1	-0,95785	0,00057
TULP3	-0,95889	0,000962
TRIM25	-0,96243	0,000116
NOL9	-0,96294	0,000794
PPFIBP1	-0,96383	2,46E-05
KIDINS220	-0,96398	9,83E-07
ACSL1	-0,96437	0,000172
RPS6KA4	-0,96767	1,31E-05
WARS	-0,96876	0,000605
TWSG1	-0,96951	0,000153
PEF1	-0,96962	3,35E-06
RGS10	-0,97024	0,000178
EIF3M	-0,97073	1,14E-06
SPIRE1	-0,97204	1,46E-06
PRKRA	-0,9722	1,86E-06
TMEM184B	-0,97241	1,70E-05
FIBP	-0,97306	1,48E-06
ZHX2	-0,97473	9,46E-05
EIF4ENIF1	-0,9773	0,000125
TMED4	-0,97743	2,38E-06
PBDC1	-0,97982	8,15E-05
PPP2CB	-0,98126	1,69E-05
MPC2	-0,98149	0,000135
SRP14	-0,98188	1,34E-05
MRPS22	-0,98199	1,24E-05
SEC23A	-0,98495	0,000531
RAB27A	-0,98508	0,00055
RAC1	-0,98571	5,85E-08
ATP11B	-0,98592	7,89E-06
ZHX1	-0,98604	5,92E-05
MTRF1L	-0,98703	0,000459
OSBPL9	-0,98782	5,14E-07
BNIP2	-0,9893	0,000228
NFU1	-0,99087	0,00039

CYP20A1	-0,99242	0,000463
PXN	-0,99416	0,000703
BLZF1	-0,99552	0,000246
YIF1B	-0,99694	0,000137
YAP1	-0,99808	5,88E-05
JMJD1C	-0,99877	5,88E-05
OPLAH	-0,99978	0,000162
EFCAB14	-1,00051	9,41E-07
ACBD3	-1,00195	1,45E-05
IBTK	-1,00235	5,95E-06
NT5C2	-1,00357	0,000283
HERPUD2	-1,00566	2,85E-05
NAP1L4	-1,00631	4,19E-07
SUN1	-1,00862	6,56E-07
NEDD4	-1,01027	0,000211
CEP63	-1,01207	1,31E-05
POMT2	-1,01311	1,57E-05
HTRA2	-1,01363	0,000119
RBM34	-1,01392	2,40E-06
RAB11B	-1,01415	6,67E-05
LGMN	-1,01472	2,71E-05
SCAF1	-1,01582	4,61E-05
ST7	-1,01616	0,000556
ARHGEF10I	-1,01813	0,000317
SUGT1	-1,01887	0,000552
PPIE	-1,02115	1,66E-06
SUV39H1	-1,02157	6,32E-05
WDR4	-1,02181	7,17E-05
COPA	-1,0221	3,16E-06
IAH1	-1,0223	7,50E-06
ENDOD1	-1,02535	0,000107
CREM	-1,02613	6,22E-05
PSMF1	-1,02653	6,08E-05
SKI	-1,02692	4,57E-07
BCCIP	-1,02726	2,94E-07
ATPIF1	-1,02777	5,74E-06
FAM188A	-1,02818	0,000279
HMGN4	-1,02848	2,20E-05
GOLIM4	-1,02903	3,10E-07
LAP3	-1,02951	5,82E-05
IQGAP1	-1,03004	3,60E-06
ASB6	-1,03052	7,25E-05
PSMD14	-1,03202	2,38E-06
GORASP2	-1,03206	1,39E-08
RABAC1	-1,03433	0,000521
UPF3A	-1,03483	5,96E-05
TIMM44	-1,03916	2,26E-06
NFKBIB	-1,03972	0,00064
PHF8	-1,04017	0,000105
MESDC1	-1,04017	0,000142

ARHGAP1	-1,04034	0,000606
NSMCE2	-1,04048	1,15E-05
HIAT1	-1,04137	8,90E-06
RNF126	-1,04164	0,000396
MAFK	-1,04215	3,83E-05
IGF2R	-1,04248	0,000139
RUFY3	-1,04292	0,000497
GUCY1A3	-1,04643	0,000421
SSX2IP	-1,04769	2,09E-05
JADE3	-1,0479	0,000103
PHRF1	-1,0489	4,21E-07
CWC22	-1,04907	3,85E-05
SLC35E1	-1,04926	8,11E-07
UTP11L	-1,05052	1,94E-05
GTF2A2	-1,05083	2,33E-06
USP9X	-1,05536	2,66E-05
TPD52	-1,05617	1,30E-06
EIF3J	-1,05626	2,12E-06
GCC2	-1,05672	0,00041
PCNXL2	-1,05729	0,000383
HIVEP1	-1,06002	2,66E-06
CEP104	-1,06212	1,62E-06
DYNLRB1	-1,06222	6,00E-06
LASP1	-1,06255	0,000274
PCGF5	-1,06294	2,65E-05
EIF6	-1,06363	1,03E-07
NUP35	-1,06694	0,000297
ACAP3	-1,06802	1,23E-05
DESI2	-1,06873	1,24E-05
HSP90B1	-1,06982	3,53E-07
ATF2	-1,07113	7,93E-05
OTUD5	-1,07243	3,24E-05
PEX3	-1,07255	0,000916
TCEB1	-1,07389	3,36E-07
ABHD12	-1,07391	2,99E-07
RAD51	-1,07398	3,08E-05
LTBR	-1,0741	3,09E-09
GNA11	-1,07544	1,34E-07
MEGF8	-1,07553	1,64E-06
LINC00116	-1,07738	0,000829
SLC35A3	-1,0788	0,000631
KATNAL1	-1,07901	0,00025
VPS37B	-1,08066	8,08E-06
TLN1	-1,08069	7,05E-07
GET4	-1,08087	2,68E-06
SACS	-1,08228	4,83E-07
DOCK7	-1,08324	2,72E-06
KIAA2013	-1,08522	2,36E-06
TPRA1	-1,08556	5,80E-06
YIPF6	-1,08657	5,84E-06

SDHB	-1,08705	1,10E-06
FAM3C	-1,08774	5,91E-06
GSTO1	-1,08877	0,000148
ITSN1	-1,08947	3,01E-06
TANK	-1,08991	9,53E-06
FER	-1,09108	0,000206
SHB	-1,09162	2,10E-06
CHSY1	-1,09208	6,46E-05
OSBPL2	-1,09392	9,49E-06
DSG2	-1,0941	3,44E-09
SIRT6	-1,09441	5,21E-05
UBA3	-1,09476	0,000113
GNL2	-1,09477	2,63E-07
TFPT	-1,09525	0,00041
ARL2	-1,09558	4,52E-06
TMEM54	-1,0958	3,15E-07
ABRACL	-1,0959	1,88E-05
HPS1	-1,09731	3,94E-06
MAGOHB	-1,0982	0,000128
NCOR2	-1,09875	3,60E-07
TRIO	-1,09959	1,18E-06
TBRG1	-1,09963	1,30E-05
SH3BP4	-1,10023	1,35E-05
DSCC1	-1,1008	0,000124
TXNDC12	-1,10137	4,64E-07
TEAD1	-1,10197	2,64E-07
ARHGAP27	-1,10336	2,98E-05
HPS3	-1,10337	6,06E-06
IL17RA	-1,10444	1,31E-05
MAGOH	-1,10539	2,63E-07
PTPN3	-1,10637	4,30E-06
SMARCAL1	-1,10737	2,41E-05
CLCN7	-1,10739	9,24E-07
PEPD	-1,10784	1,24E-05
SF3B2	-1,10796	3,74E-10
YBX1	-1,109	4,64E-05
C20orf24	-1,11083	3,73E-06
GBE1	-1,1109	0,000375
POLA2	-1,11215	0,000268
ANXA11	-1,11306	1,53E-06
HEXB	-1,11343	9,15E-06
SLC36A1	-1,11359	0,000125
CSPP1	-1,11544	0,000352
RPN1	-1,11666	2,24E-06
MIER1	-1,11785	0,000154
GTPBP1	-1,11939	1,43E-05
SMC4	-1,11965	1,11E-06
NIPA2	-1,12263	2,38E-05
VPS37A	-1,12313	6,76E-06
TBC1D12	-1,12473	0,000272

BBS7	-1,12478	0,000151
LY6E	-1,12556	0,000719
RHPN2	-1,12812	3,10E-05
DNAJC1	-1,12936	1,01E-08
ZNF343	-1,12993	6,89E-05
CUTA	-1,13194	2,30E-07
DVL1	-1,13308	1,07E-06
HSF1	-1,13514	2,64E-09
CBR1	-1,13564	6,87E-05
ZSWIM8	-1,13595	5,10E-05
MKL1	-1,13602	2,43E-07
RPS6KC1	-1,13605	1,79E-05
RTKN	-1,13714	2,48E-08
PITPNB	-1,13958	2,25E-08
GNB1	-1,13962	1,36E-09
TRMT6	-1,14055	1,19E-07
SEMA4D	-1,14068	0,000896
MACF1	-1,14071	6,11E-09
BABAM1	-1,14133	3,14E-07
MCFD2	-1,14296	2,67E-08
PRKAR2A	-1,14539	0,00064
NUDC	-1,1455	0,00025
SLC39A10	-1,14586	0,000813
MYL6	-1,14726	1,43E-10
CD81	-1,1499	4,35E-08
CEP290	-1,15109	3,34E-05
ADGRG1	-1,15286	5,41E-08
ACTN4	-1,15286	1,43E-05
KDSR	-1,1556	7,00E-08
AKIRIN1	-1,15566	5,13E-08
PLGRKT	-1,15568	0,000166
ZDHHC8	-1,15719	1,90E-05
PGLS	-1,15872	7,21E-07
ZNF414	-1,15896	7,06E-05
KIAA0355	-1,15927	0,00034
SAMD8	-1,15941	0,000156
NFKBIA	-1,16229	1,35E-06
HPS5	-1,16279	1,85E-05
STOM	-1,16341	1,02E-05
PHF1	-1,16381	0,000176
ADAM17	-1,16447	1,05E-05
CHST12	-1,16533	0,000459
ZNF395	-1,16547	7,96E-08
BAG2	-1,16561	1,20E-05
NUCB1	-1,16604	7,54E-07
MAP3K2	-1,16615	5,59E-07
M6PR	-1,16787	1,54E-05
QDPR	-1,16868	3,07E-06
MED15	-1,17019	6,24E-09
ASAP2	-1,17105	1,89E-08

MIEF1	-1,17233	2,04E-07
POFUT2	-1,17311	0,000715
GLUD1	-1,17335	1,53E-09
KANSL3	-1,17355	8,51E-07
TYRO3	-1,17434	3,34E-08
VAMP4	-1,17451	0,000801
SLC25A28	-1,17562	0,000304
RPS6KA3	-1,17591	6,58E-08
EIF2B2	-1,177	8,30E-09
SLC41A1	-1,17761	2,37E-06
LZTS2	-1,17768	9,41E-09
FMNL2	-1,17812	2,68E-06
MAP2K1	-1,18025	3,84E-08
CHN1	-1,18026	0,000656
EHBP1	-1,18029	1,29E-06
FERMT1	-1,18274	0,000132
USP16	-1,18345	2,44E-06
SAMD4B	-1,18367	2,61E-05
CLIP1	-1,18375	7,33E-09
PPP2R3A	-1,18457	0,000194
RBFOX2	-1,18489	5,27E-09
ZMIZ2	-1,18619	1,55E-10
CFL1	-1,18649	1,82E-09
RASA3	-1,18653	0,000763
SF3B6	-1,18698	1,86E-05
DERL1	-1,18761	2,50E-06
HAT1	-1,18817	3,71E-09
FAU	-1,19009	2,06E-11
SCARB1	-1,1921	3,16E-05
EHD1	-1,19245	0,000829
HK1	-1,19247	6,76E-05
SRPRB	-1,19349	7,97E-10
TMCO3	-1,19358	0,000546
ARF4	-1,19383	0,000811
RECQL	-1,19547	7,48E-08
ITPA	-1,19558	9,49E-06
ZNF584	-1,19788	8,31E-06
RBM19	-1,19874	0,000903
GNAI2	-1,19902	0,000125
SRM	-1,19914	1,96E-05
PSMB2	-1,1993	3,23E-11
LRRFIP2	-1,19983	2,53E-08
DMTN	-1,2002	4,74E-06
IQCB1	-1,20046	2,17E-05
MTDH	-1,20052	5,80E-06
ZCCHC17	-1,20531	8,78E-06
LRRC41	-1,20542	3,82E-08
TFE3	-1,20555	9,33E-09
DLGAP4	-1,20607	3,03E-08
ATP13A2	-1,20699	1,49E-05

TRIT1	-1,20719	9,82E-05
SEC61A1	-1,20752	0,000177
CTNNAL1	-1,20879	0,00044
ZDHHC13	-1,20932	4,44E-05
KLF4	-1,21018	8,77E-05
RABGEF1	-1,2111	1,58E-07
SVIL	-1,21149	1,73E-05
TWISTNB	-1,21278	2,58E-06
MOSPD1	-1,21304	7,78E-06
SLC2A8	-1,21333	0,000238
UGGT2	-1,21385	0,000992
BTBD10	-1,2182	2,06E-06
NCAPH2	-1,21841	7,23E-09
ARHGEF7	-1,22052	2,69E-06
CSNK2A1	-1,22162	5,45E-10
ENAH	-1,22244	1,26E-07
ACAT1	-1,22314	0,000138
RSU1	-1,22585	6,12E-10
RCBTB1	-1,22684	5,25E-05
DNAJC8	-1,22704	3,15E-07
SBF1	-1,2274	1,31E-08
CEBPD	-1,22742	0,000123
ATP6V1E1	-1,22862	2,67E-06
SLC16A1	-1,22988	1,29E-08
GFRA1	-1,23008	0,000153
MCM8	-1,23012	1,09E-07
C1orf52	-1,23029	1,53E-05
NENF	-1,23239	5,04E-08
ACVR1	-1,23346	0,000161
HAUS8	-1,23507	2,48E-06
CERS5	-1,23609	1,88E-06
UACA	-1,23639	6,38E-06
CCT6A	-1,23652	6,63E-11
NXPH4	-1,23733	2,46E-07
KCTD11	-1,23744	0,000759
MPZL1	-1,23762	3,69E-11
RPS6KA1	-1,23784	0,000234
YIF1A	-1,23823	5,93E-09
ERCC1	-1,23881	3,81E-08
TM2D1	-1,23969	0,00024
ECI2	-1,24034	3,92E-06
LDLRAP1	-1,24057	0,000933
STAT2	-1,24279	2,90E-06
ATAD3A	-1,24286	4,42E-10
DEDD2	-1,24288	0,000169
ATP6V1C1	-1,24339	0,00053
LPGAT1	-1,24399	9,51E-07
SLC10A3	-1,24435	6,41E-08
TMEM230	-1,24521	3,22E-08
ZBTB43	-1,24619	4,31E-07

MED27	-1,24652	1,38E-06
RHOG	-1,24684	2,68E-09
LOC100129	-1,2473	7,75E-05
TYK2	-1,24904	6,39E-05
AGTPBP1	-1,24925	1,56E-05
MRPL13	-1,25207	9,32E-08
DMPK	-1,25224	3,57E-05
TMEM87A	-1,25254	1,36E-08
STK24	-1,25431	1,18E-09
TYMS	-1,25446	4,58E-09
CD276	-1,25467	9,04E-06
STAM2	-1,25519	2,99E-07
AGRN	-1,25552	1,26E-06
RAB29	-1,25829	5,66E-05
SPAG9	-1,2589	1,14E-06
BZW1	-1,25989	2,41E-09
PIGK	-1,26132	2,86E-05
LAMB1	-1,26203	2,34E-11
SASS6	-1,26268	0,000786
PTS	-1,26389	0,000542
TMEM63A	-1,26512	0,000133
SELK	-1,26708	0,00074
CRY1	-1,26847	1,38E-06
MAP2K7	-1,27137	6,38E-09
STXBP2	-1,27154	1,25E-08
DPM2	-1,27172	1,40E-05
RB1	-1,27178	3,35E-07
STXBP5	-1,27189	1,03E-05
OPHN1	-1,2756	0,000736
PIK3C3	-1,27578	4,62E-05
MYSM1	-1,27579	0,000976
ELMSAN1	-1,27642	7,17E-05
LUZP1	-1,27643	0,000499
BET1L	-1,27873	8,50E-07
QKI	-1,27915	2,01E-05
EXO5	-1,27972	6,77E-05
RAB3GAP2	-1,27974	4,24E-09
LFNG	-1,28021	9,43E-05
FBXO27	-1,28086	9,11E-08
CD99	-1,28092	5,76E-08
B4GALT5	-1,28162	7,35E-09
DMAP1	-1,28189	0,00036
CDS2	-1,28417	3,27E-07
BLOC1S6	-1,28448	1,47E-08
LMAN2L	-1,2852	4,12E-07
PATL1	-1,28552	1,83E-10
LRRC42	-1,28665	4,89E-09
SATB2	-1,28677	2,28E-06
SLC31A1	-1,28697	8,14E-11
ZFAND2A	-1,28745	3,26E-06

FOXK1	-1,28919	3,40E-08
GPX8	-1,28945	1,53E-06
CPD	-1,29042	2,74E-09
FLNB	-1,29055	2,17E-06
DHDDS	-1,29069	0,000288
EXOSC10	-1,29135	5,06E-09
SIPA1L2	-1,29151	2,47E-09
CXorf40B	-1,29239	0,000468
CLTA	-1,2926	5,92E-10
ARHGAP21	-1,29268	7,01E-09
COPS8	-1,2928	4,70E-07
RCN1	-1,29337	8,02E-12
ZNF335	-1,29343	3,66E-06
ZNF841	-1,29462	7,97E-07
CA5BP1	-1,29512	2,37E-05
CLIC1	-1,29629	1,74E-07
PHC2	-1,29726	8,24E-07
NOTCH2	-1,29787	8,21E-12
TTPAL	-1,29866	1,40E-07
YKT6	-1,29892	2,17E-11
PITHD1	-1,29899	4,47E-08
CDC37	-1,29909	8,36E-07
TMEM201	-1,30176	4,17E-07
SAT2	-1,30199	1,96E-05
EMC1	-1,30252	7,66E-10
VDR	-1,30307	0,000393
SNX12	-1,30345	0,00027
LIMK1	-1,30404	6,33E-06
TRIOBP	-1,30421	4,77E-09
STAT3	-1,30568	0,000471
ACTB	-1,30668	2,44E-06
S100A11	-1,30885	2,67E-05
RNH1	-1,30956	6,26E-06
GFPT1	-1,30982	2,44E-11
TWF2	-1,30987	1,21E-08
MARK4	-1,31026	7,00E-06
RHBDF2	-1,3105	1,09E-08
BMP2K	-1,31073	5,29E-07
PITPNM3	-1,31207	0,000502
MAFG	-1,31355	5,88E-09
MAP7D3	-1,3136	1,13E-09
RRP12	-1,31567	6,76E-11
TPCN1	-1,31591	7,85E-09
SDF4	-1,31606	1,51E-05
HEBP2	-1,31681	3,74E-07
FAM110B	-1,317	0,000928
INTS6	-1,31885	5,29E-05
SEC14L1	-1,31913	9,93E-08
CXXC5	-1,32074	0,000162
ZRSR2	-1,32121	1,85E-07

EDEM1	-1,32208	1,58E-07
RAPH1	-1,32444	1,02E-05
PNPLA2	-1,32646	9,37E-06
SKAP2	-1,32661	2,93E-07
RPAP1	-1,32886	5,90E-10
ZBTB17	-1,32942	6,57E-07
HAUS2	-1,32971	0,000656
RNF144A	-1,33016	3,57E-05
FBXL6	-1,33113	0,000522
KIF1B	-1,33123	1,88E-05
NCLN	-1,33255	8,34E-12
USP18	-1,33704	0,000731
MELK	-1,33716	2,48E-11
RNF213	-1,33852	0,000215
NDUFA11	-1,33873	1,62E-11
PEX14	-1,33912	1,98E-06
PHF19	-1,33972	2,23E-09
RAB23	-1,33984	2,02E-08
TRAM1	-1,34018	5,00E-05
SCRN3	-1,34025	0,000143
RPF1	-1,34644	4,82E-08
LAMB2	-1,34656	5,92E-09
PTPN9	-1,34676	1,06E-08
HDAC7	-1,34677	1,47E-09
TRIM44	-1,34741	8,84E-13
YPEL5	-1,34816	8,44E-05
U2SURP	-1,35045	3,43E-11
TRIM35	-1,35313	7,15E-06
KIAA1715	-1,35333	2,18E-06
PPARA	-1,35517	0,000377
LRCH1	-1,35636	4,49E-09
ZNF706	-1,35792	1,36E-12
CEP89	-1,35856	1,05E-06
PSMA1	-1,35951	4,52E-12
PWWP2B	-1,35957	6,20E-11
C11orf95	-1,35959	1,05E-08
TJAP1	-1,35967	0,000126
HMGCL	-1,36034	9,04E-07
B4GALT6	-1,36455	0,000905
SDCCAG8	-1,36481	8,96E-05
ILK	-1,36535	1,02E-07
UFM1	-1,36664	2,09E-10
ENC1	-1,36711	1,41E-05
NVL	-1,36903	3,76E-08
WDFY2	-1,37049	7,19E-05
CAPN1	-1,37119	0,000695
CCDC9	-1,37148	2,58E-10
CDK16	-1,37246	6,36E-11
EPS15L1	-1,373	3,92E-08
TRMT1	-1,3752	1,19E-08

DUSP14	-1,37542	9,27E-09
NSFL1C	-1,37702	3,50E-10
UBE2E3	-1,38043	3,78E-09
TCIRG1	-1,38134	5,60E-06
ZNF823	-1,38145	9,12E-05
WDR91	-1,38239	3,50E-07
SGCB	-1,38392	7,81E-07
STX2	-1,38455	3,18E-06
EPS15	-1,38482	1,58E-09
SSFA2	-1,38497	6,47E-11
POFUT1	-1,3855	2,01E-09
ALKBH3	-1,38641	3,13E-08
YARS	-1,3871	7,76E-08
CCDC50	-1,38726	3,63E-07
CLSTN1	-1,38821	2,65E-08
MAP1S	-1,38887	1,43E-06
CASC4	-1,38968	1,49E-08
STK10	-1,39058	9,02E-11
PTDSS2	-1,39142	0,000435
GNA12	-1,3916	5,92E-11
AAED1	-1,39187	0,000508
TTC13	-1,39248	0,000117
DNAJC3	-1,3936	4,35E-05
ADAM10	-1,39376	4,20E-09
GTF2H1	-1,39455	5,90E-11
CRISPLD2	-1,39602	3,36E-06
MAPK11	-1,39931	4,37E-05
TRIM8	-1,4005	2,04E-09
ZNF114	-1,40063	2,47E-06
CNPY2	-1,4033	9,71E-13
SGK223	-1,40442	3,62E-08
GHDC	-1,40499	0,000288
VAC14	-1,40514	1,45E-13
FBXO28	-1,40622	8,23E-06
SLC12A7	-1,40658	5,74E-11
NRBF2	-1,4078	7,91E-10
PLCG2	-1,40937	3,59E-07
TSPYL2	-1,41019	5,67E-05
LRRC57	-1,41274	0,000387
IFI30	-1,41443	1,82E-05
GNPDA1	-1,41742	8,54E-07
ATF4	-1,41757	9,07E-07
KLHL7	-1,41763	9,27E-06
NECAP2	-1,41814	5,55E-09
DCLK1	-1,41863	6,11E-09
SDC3	-1,41987	1,14E-07
PLD3	-1,41999	3,26E-06
HEBP1	-1,42058	2,05E-10
DYNC1I2	-1,42458	2,70E-10
PNPLA6	-1,42503	0,000208

NPC1	-1,42561	4,93E-10
NEK1	-1,42809	0,000873
TNFRSF1A	-1,42824	9,67E-08
LPP	-1,42831	1,36E-08
EXTL3	-1,42935	8,44E-10
LAMP1	-1,43031	6,47E-09
TBC1D20	-1,43068	7,13E-10
MLF1	-1,4328	6,88E-06
SP140L	-1,43363	2,55E-08
PGAM1	-1,43456	3,12E-07
VAMP3	-1,43484	6,36E-05
USP11	-1,43624	2,34E-10
RELA	-1,43833	7,09E-13
NCSTN	-1,43872	7,84E-12
CCDC183-A	-1,43985	0,000156
TXLNA	-1,44072	4,17E-09
KCNH2	-1,44121	3,71E-06
SPTAN1	-1,44126	4,24E-12
TMEM65	-1,44147	2,48E-07
XPNPEP1	-1,44194	2,40E-08
RIC8A	-1,44553	1,46E-13
PVRL2	-1,44761	1,82E-09
PACSIN2	-1,44766	1,80E-09
ANKLE2	-1,44786	1,47E-11
KIF3C	-1,44806	2,27E-07
EDEM2	-1,45361	8,69E-09
FKBP9	-1,45611	7,40E-13
TUBA1C	-1,46016	9,82E-14
SLC12A8	-1,46074	1,23E-05
PDCD5	-1,4612	1,09E-14
B3GALNT1	-1,46238	4,51E-08
UAP1	-1,46276	6,37E-07
FIGNL1	-1,46348	6,75E-10
B3GNT4	-1,46367	0,000472
ZBTB8OS	-1,46486	1,84E-07
CTU1	-1,46753	0,000327
WDR13	-1,46755	0,000124
FZD6	-1,46782	3,31E-08
FAXC	-1,46864	5,67E-05
CREB3	-1,47004	1,72E-05
HSPA5	-1,47073	2,98E-17
ARNT2	-1,47076	1,23E-07
ZNF41	-1,47259	5,50E-07
DBNL	-1,47307	2,89E-06
PSMD8	-1,47405	9,30E-17
ERO1A	-1,47446	1,95E-10
LAPTM4A	-1,47465	9,10E-14
SLC25A32	-1,4749	1,88E-07
CALU	-1,475	1,45E-11
TOM1	-1,47536	1,29E-07

ZNF275	-1,47563	2,69E-09
BTN2A2	-1,47568	1,47E-05
CLK1	-1,47653	0,000133
CDC42BPA	-1,47768	1,86E-07
ZNF44	-1,47937	6,84E-06
CCZ1	-1,4818	6,46E-09
FBXO42	-1,48342	8,48E-07
EMC7	-1,4853	4,17E-08
HDAC6	-1,48536	6,00E-09
ATP1B3	-1,48633	1,29E-16
PDIA5	-1,48638	3,75E-10
GLRX3	-1,48768	2,80E-13
ANO6	-1,48794	5,92E-09
INAFM1	-1,48987	5,59E-06
FTL	-1,49055	6,72E-05
CNDP2	-1,49076	2,89E-10
B3GLCT	-1,49181	6,18E-05
PLXNA3	-1,49261	1,18E-08
NFATC1	-1,49308	0,000219
USP40	-1,49337	3,16E-09
RASAL2	-1,49351	6,08E-09
MAST2	-1,49679	9,66E-13
PIGH	-1,49844	3,44E-09
PRPS1	-1,49911	8,76E-09
FMNL3	-1,50049	4,12E-08
CST3	-1,50079	2,39E-09
PNKD	-1,50128	5,48E-08
SERAC1	-1,50153	0,00058
LGR4	-1,5026	0,000108
STAM	-1,50522	5,98E-08
NRSN2	-1,50665	3,57E-06
CREB3L2	-1,50674	9,33E-08
RAB7A	-1,50967	3,49E-06
TRNAU1AP	-1,51032	2,67E-06
PRKAG2	-1,51094	1,16E-11
ATP6AP2	-1,51103	5,58E-09
PPP1R12C	-1,51173	6,84E-11
CAPZB	-1,51271	8,82E-08
EOGT	-1,51336	0,000319
CCDC130	-1,51597	0,00012
DNTTIP2	-1,51606	1,01E-14
ENO1	-1,51706	2,05E-08
DPF2	-1,52109	2,26E-12
SMAP2	-1,52351	0,000543
POMGNT1	-1,52589	0,000191
KIAA1671	-1,52718	7,10E-11
ITPR3	-1,52789	1,48E-10
RNPEPL1	-1,5303	4,35E-08
RTN4	-1,53075	4,59E-13
RNF24	-1,53382	8,21E-06

SNX3	-1,53435	5,00E-10
FAM210A	-1,53521	2,83E-06
CCDC91	-1,53727	4,86E-06
ATP6V0B	-1,53864	3,50E-16
COL9A2	-1,54052	6,33E-05
GNB1L	-1,54105	4,72E-06
ST3GAL4	-1,54124	5,75E-05
RILPL1	-1,5421	1,03E-07
HSPBAP1	-1,54376	0,000116
NUAK1	-1,54571	8,06E-10
RIT1	-1,5462	8,64E-10
ACOT9	-1,54627	9,08E-08
TRPV3	-1,54665	0,000129
ARFGEF1	-1,54694	4,08E-12
TBC1D17	-1,54848	2,86E-06
TXN	-1,54978	4,75E-07
BNIP3L	-1,55369	0,000146
CD151	-1,55485	1,63E-13
TDRD7	-1,55515	0,000907
PRELID2	-1,55756	9,79E-07
SLC35C1	-1,55932	3,04E-13
SH3RF1	-1,56015	0,000129
EXT2	-1,56032	1,45E-07
EIF1AD	-1,56041	2,64E-14
TMED5	-1,56057	8,27E-08
PTP4A2	-1,56112	5,56E-14
SNX25	-1,56156	1,55E-05
SOD2	-1,5622	9,78E-12
PLD1	-1,56222	2,88E-06
WHAMM	-1,56247	9,77E-09
ARMC9	-1,56479	4,73E-09
RMDN3	-1,5648	1,01E-06
DR1	-1,56578	1,96E-14
DNTTIP1	-1,56988	3,31E-10
ARL2BP	-1,57031	4,98E-12
FAM49B	-1,57072	1,72E-16
TRIM14	-1,57164	0,000239
SEPT7	-1,57332	1,77E-13
GNPTG	-1,57603	1,78E-09
DCBLD1	-1,57609	0,000172
SLC2A10	-1,57642	4,22E-05
WDFY1	-1,57646	4,52E-13
ARHGEF10	-1,57653	0,000125
PIP5K1C	-1,57943	3,15E-07
SPRYD7	-1,58049	1,18E-06
PLEKHG2	-1,5813	2,65E-12
PLEKHA5	-1,58335	3,21E-10
ACADVL	-1,58615	3,84E-08
UBQLN2	-1,5866	3,28E-14
AP2A2	-1,58679	1,54E-12

TPD52L2	-1,58919	1,92E-15
TAF2	-1,59016	2,14E-16
PARP3	-1,59027	4,71E-07
BCL2L2	-1,59082	2,41E-08
ATP11A	-1,59119	5,48E-09
SEMA7A	-1,592	1,48E-07
ITSN2	-1,59268	3,94E-09
ZNF274	-1,59319	1,33E-08
STK40	-1,59382	9,84E-09
MAD1L1	-1,59445	1,03E-17
DNAJC10	-1,59604	9,53E-13
ITGAV	-1,5961	1,30E-09
UBA5	-1,59635	2,79E-09
CABLES1	-1,59699	1,51E-07
DMWD	-1,59701	1,76E-10
ZNF530	-1,59725	0,00071
JMJD6	-1,59756	2,91E-06
PARP8	-1,5977	1,18E-06
RNF19A	-1,59882	3,32E-09
NEK6	-1,59949	6,43E-12
FLVCR1	-1,6	8,35E-06
CTSC	-1,60513	3,13E-13
CNIH4	-1,60622	2,25E-12
ABL2	-1,60726	2,92E-16
WWC2	-1,60843	2,19E-10
FBXL12	-1,60879	2,18E-06
CARS	-1,60973	6,37E-09
RDH10	-1,61137	5,52E-05
BACH1	-1,61149	3,17E-05
PLEKHM1	-1,61211	3,41E-06
ATP6V1D	-1,61655	7,58E-05
AP1M1	-1,61696	5,93E-08
RNF216P1	-1,62124	5,58E-09
TMEM198	-1,62162	0,000126
GARS	-1,62213	3,77E-17
PHF20L1	-1,6239	1,78E-17
GUK1	-1,62612	1,32E-08
CCNL1	-1,62678	0,000327
DIXDC1	-1,62825	2,78E-06
NCS1	-1,62959	5,67E-16
ATP10D	-1,62996	0,000614
ANXA2	-1,6311	1,51E-21
NR6A1	-1,6314	3,62E-05
CKAP2L	-1,63141	9,66E-06
QRICH2	-1,63554	0,000118
IER3	-1,63589	2,19E-11
LOC646762	-1,63651	6,00E-05
GPR108	-1,63779	1,22E-09
PFDN2	-1,63839	1,14E-12
FZD7	-1,63951	6,47E-09

CHCHD7	-1,64108	2,19E-06
LINC01278	-1,64132	1,12E-10
PLEKHN1	-1,64385	5,27E-06
C19orf12	-1,64457	7,40E-09
ZNF440	-1,64483	3,08E-05
CYP2R1	-1,64492	0,000374
TESK1	-1,64663	1,45E-10
SNTB1	-1,64725	5,16E-18
CALR	-1,6478	5,24E-07
ATP6V1H	-1,65031	2,52E-11
DOCK5	-1,65045	1,03E-08
RASSF3	-1,65046	1,83E-08
FZD5	-1,65237	2,75E-11
ZC3H12A	-1,65244	1,54E-05
MYDGF	-1,65289	2,57E-11
FGF11	-1,65361	0,000287
CAST	-1,65442	1,83E-14
PQLC1	-1,65462	4,80E-09
TBCE	-1,65603	4,62E-11
COL18A1	-1,65668	2,35E-17
REXO2	-1,65728	1,09E-06
ATP9B	-1,65741	1,91E-06
SIPA1L3	-1,65749	5,64E-16
WDR47	-1,65762	2,74E-07
RER1	-1,65773	1,25E-16
JAK3	-1,65853	0,000109
CNKSR3	-1,65938	2,16E-08
ADCY3	-1,66074	1,69E-14
JRKL	-1,66138	3,50E-05
RASA2	-1,66404	1,86E-05
CFLAR	-1,66547	1,39E-13
MAP2	-1,66593	3,91E-05
SHARPIN	-1,6676	8,56E-19
SETD4	-1,6683	0,000589
ANXA5	-1,66855	1,19E-22
AKIP1	-1,67022	2,44E-09
BCL10	-1,67106	1,16E-07
PQBP1	-1,67194	1,99E-13
BTG1	-1,67213	2,14E-17
ALS2	-1,67369	3,33E-12
CELSR1	-1,67407	6,64E-17
MORC4	-1,67452	4,38E-13
INAFM2	-1,67532	0,000686
MARVELD1	-1,67638	6,19E-13
ARFGAP1	-1,67644	6,80E-16
ECHDC3	-1,67655	3,85E-07
AGPAT2	-1,67804	1,11E-13
ELK1	-1,67886	1,25E-14
LOC10013[illegible]	-1,67939	0,000858
NIPA1	-1,68107	6,08E-14

JOSD1	-1,68133	1,07E-16
KLC2	-1,68159	1,09E-17
TCF19	-1,68183	1,54E-16
HGSNAT	-1,68444	3,77E-06
HSPA13	-1,68518	7,93E-08
PLEKHA1	-1,68803	2,39E-09
KLF3	-1,68904	5,89E-09
SETDB2	-1,6901	5,14E-06
NOCT	-1,69141	1,49E-09
DONSON	-1,69409	2,29E-10
NCK2	-1,69494	3,60E-20
RGS19	-1,69888	1,15E-06
MPRIP	-1,69954	2,99E-13
CTNS	-1,70036	1,02E-12
ZNHIT6	-1,7004	4,21E-05
PPP3CC	-1,70111	1,88E-06
RIPK2	-1,70276	3,77E-20
C1GALT1	-1,70284	2,15E-08
FAM129B	-1,70475	5,00E-14
CXorf38	-1,70477	2,29E-08
LATS2	-1,7048	2,65E-12
RUNX1	-1,70678	5,65E-17
LRRFIP1	-1,70701	1,70E-19
ACOT7	-1,70714	6,06E-11
SLC35A2	-1,70755	1,98E-11
SLC20A1	-1,71007	4,41E-20
PTPN21	-1,7101	3,41E-09
CASP3	-1,7108	2,12E-07
DPP9	-1,71366	1,35E-13
PLCB4	-1,71373	3,02E-09
SPG20	-1,71466	4,14E-14
PTPRG	-1,71477	6,96E-10
SLFN5	-1,71586	4,27E-08
PARVB	-1,71785	5,92E-11
ARRDC3	-1,71881	9,26E-09
MAGED2	-1,71888	4,48E-17
SZT2	-1,7192	1,29E-08
UXT	-1,71925	7,52E-09
FNTA	-1,72161	6,13E-14
TSPO	-1,72301	4,55E-18
PGK1	-1,72591	8,97E-18
WASF2	-1,72641	6,53E-19
MTFR2	-1,7277	0,000497
SNAPC2	-1,72813	1,90E-08
GPKOW	-1,72843	1,00E-10
FAM65A	-1,72846	3,18E-16
TMEM178I	-1,72871	9,33E-09
DYNLT1	-1,72958	3,89E-15
ST6GALNA(	-1,73059	2,20E-08
HIVEP2	-1,73146	4,60E-14

HIC1	-1,73181	0,00064
PPIL3	-1,73194	3,17E-12
WDR45	-1,73518	1,20E-09
SLC25A12	-1,73615	1,58E-08
HIPK2	-1,73925	1,96E-06
ABCC4	-1,74016	8,97E-14
FUCA1	-1,7418	7,34E-08
TOLLIP	-1,74287	2,03E-06
SYNJ1	-1,74442	4,68E-06
ZNFX1	-1,74458	7,89E-09
ST3GAL3	-1,745	4,70E-05
SSR3	-1,74569	5,80E-15
OGFR	-1,74574	6,59E-13
C19orf33	-1,74623	1,08E-07
PI4K2A	-1,74914	1,21E-10
EPS8L2	-1,75173	1,57E-07
NFKB1	-1,75235	5,22E-08
NAPRT	-1,75346	2,45E-05
FBXO17	-1,75467	7,32E-07
LPCAT4	-1,75649	1,11E-05
MBOAT2	-1,76141	6,68E-10
CSRP2	-1,7618	1,70E-11
FAIM	-1,76428	1,45E-09
TIPARP	-1,76436	2,16E-10
EGLN1	-1,76457	1,01E-16
RHOC	-1,76473	9,39E-10
PDGFB	-1,76554	2,13E-08
MGAT5	-1,76665	3,52E-13
EML2	-1,76831	3,83E-08
SRGAP2D	-1,76899	0,000671
HCFC2	-1,76916	0,00013
SGMS2	-1,76991	0,00027
LENG8	-1,7725	3,28E-11
CD63	-1,77527	7,91E-10
UBE2J2	-1,77563	4,46E-19
CASP7	-1,7795	2,61E-08
PCBP4	-1,77958	1,98E-12
TIGD7	-1,7798	0,000134
ZNF563	-1,78003	0,000149
CASP8	-1,78033	1,53E-13
FAM198B	-1,78115	9,44E-12
WDR90	-1,78208	0,000261
TOMM34	-1,78225	7,27E-19
EVI5	-1,7829	6,12E-07
CPTP	-1,78392	2,85E-12
PLEKHA3	-1,78494	3,69E-10
AP5Z1	-1,7854	1,26E-17
NOD1	-1,78695	1,88E-07
TPP1	-1,78809	4,24E-12
RNF185	-1,79014	4,66E-15

PDLIM5	-1,79061	3,01E-09
AHNAK	-1,79125	3,03E-12
FADS3	-1,79158	4,90E-13
PLEKHM2	-1,79202	4,44E-17
FAAP20	-1,7923	5,59E-15
SNTA1	-1,79302	4,23E-10
NAPA	-1,7931	3,92E-08
PCED1A	-1,79592	1,44E-10
VPS13C	-1,79725	1,94E-08
ZNF644	-1,79795	2,33E-17
RBMS1	-1,79826	2,50E-11
EPHX1	-1,79969	3,66E-08
SEMA6A	-1,79986	1,40E-08
TMEM62	-1,80214	4,48E-11
CFL2	-1,80732	2,08E-12
ABHD5	-1,80748	8,93E-15
RALB	-1,80917	0,00016
ETV6	-1,80951	2,40E-11
BACE2	-1,81084	6,21E-12
MYO10	-1,81105	5,99E-17
UBA6	-1,81231	4,14E-18
CHST14	-1,81273	3,57E-08
FJX1	-1,81344	0,000284
RDX	-1,81362	1,30E-12
ITGB1BP1	-1,81423	6,07E-14
STK4	-1,81477	2,71E-16
SLC17A5	-1,81747	1,88E-05
SMS	-1,81943	5,44E-13
SERTAD2	-1,8199	3,29E-17
KDELC1	-1,82237	3,22E-07
MXD1	-1,82276	1,45E-07
FURIN	-1,82549	2,43E-13
KCNC4	-1,82555	9,81E-05
CDC42EP5	-1,82796	9,15E-05
PTER	-1,82803	5,76E-13
ZFP36L1	-1,82966	4,36E-13
PACS1	-1,83025	2,72E-17
GINS3	-1,83184	1,85E-07
RELL2	-1,83319	2,90E-08
RAPGEF1	-1,83419	5,61E-14
ARPIN	-1,83463	4,27E-05
F2R	-1,83678	4,13E-07
FAM76A	-1,83679	2,25E-05
DIAPH2	-1,83705	1,58E-07
AKAP17A	-1,83856	1,19E-08
UXS1	-1,83893	5,02E-21
FRMD8	-1,84284	4,36E-13
MIR4435-2	-1,8429	1,80E-09
ITPRIPL2	-1,84461	6,03E-15
LRIG1	-1,84567	4,57E-12

C4orf32	-1,84659	4,53E-06
GNPDA2	-1,84909	0,000165
ZFPL1	-1,85215	6,10E-18
LPIN2	-1,8525	1,60E-11
PJA1	-1,85391	1,95E-09
MT2A	-1,85427	4,61E-17
NABP1	-1,85435	7,36E-09
AMPD2	-1,85442	2,37E-12
TTLL11	-1,8551	0,000127
MYO9B	-1,85745	3,66E-10
TK2	-1,85912	7,06E-06
ATRNL1	-1,86258	0,000136
VOPP1	-1,86417	2,80E-12
KDM5C	-1,86476	2,47E-21
KDELR3	-1,86645	2,39E-05
KLF10	-1,86728	8,23E-07
SDC1	-1,86927	8,32E-16
ADAM9	-1,87035	1,54E-21
UBA6-AS1	-1,8706	2,04E-07
ANKRD13C	-1,87261	1,81E-12
CAPN10	-1,87335	1,08E-06
DGKD	-1,87388	6,83E-12
RLTPR	-1,87482	1,86E-06
CD58	-1,87563	3,66E-08
FKBP1A	-1,876	8,33E-16
OXCT1	-1,87621	4,49E-15
DESI1	-1,87639	6,75E-22
IL13RA1	-1,87677	1,83E-11
EIF5A2	-1,87807	1,32E-13
ZNF827	-1,88352	2,21E-05
SPRED2	-1,88552	1,05E-19
SLC33A1	-1,88603	1,05E-15
DOK3	-1,88689	2,76E-06
PRAF2	-1,89002	2,06E-07
RNF149	-1,89314	1,72E-22
DAPK3	-1,89437	1,48E-20
SIN3B	-1,89618	1,29E-19
TNIP1	-1,89797	4,67E-08
SLC35F6	-1,89902	1,53E-14
APH1B	-1,90003	8,21E-06
SLC8A1	-1,90484	4,24E-12
RP9P	-1,90533	1,43E-05
SNX9	-1,90686	8,54E-18
OAT	-1,90811	5,25E-06
SLC31A2	-1,90955	0,000617
MROH1	-1,91145	9,95E-16
SNX21	-1,91221	1,57E-10
AIDA	-1,91278	1,20E-10
IFI27L1	-1,91349	1,22E-06
BTG3	-1,91477	5,45E-05

HECTD3	-1,91586	2,60E-09
SLC15A4	-1,91751	2,23E-15
ZNF558	-1,91764	2,27E-08
TAPBP	-1,91969	9,57E-09
LGALS8	-1,92327	2,33E-14
AAK1	-1,92646	1,72E-10
NFIX	-1,92728	3,70E-08
CNTLN	-1,92801	1,84E-05
TCEA1	-1,9306	5,80E-15
CHIC1	-1,93317	0,00077
FAM107B	-1,93339	2,09E-06
GPR161	-1,93359	7,09E-11
PDIA3	-1,93439	3,83E-24
NBEA	-1,93757	3,25E-08
USB1	-1,93812	2,13E-08
RAB8B	-1,93899	8,59E-07
PHF11	-1,93919	1,25E-09
CD82	-1,93939	4,25E-10
FLOT1	-1,93995	3,76E-18
SEC61A2	-1,94168	1,62E-07
LIN37	-1,94326	6,84E-09
CMIP	-1,94447	5,55E-16
GLI3	-1,94459	2,30E-10
IMPA1	-1,94842	4,84E-09
CORO1C	-1,95116	1,88E-26
SEL1L3	-1,9517	6,73E-13
CPOX	-1,95364	7,84E-07
DOCK9	-1,95501	5,26E-15
FARP1	-1,9551	4,67E-18
MGST3	-1,95644	2,72E-10
SH3GL1	-1,95887	2,17E-17
TEX30	-1,95922	2,64E-09
MFSD12	-1,96027	1,12E-23
PLBD2	-1,96062	6,72E-10
ELF4	-1,96312	1,00E-10
FTSJ1	-1,96389	1,35E-22
ANXA2P2	-1,96402	0,000139
NPC2	-1,96539	3,07E-19
PKD1	-1,96617	0,000117
SHISA2	-1,96675	2,60E-05
C1orf198	-1,96726	1,18E-12
CTSA	-1,97017	1,35E-07
ECHDC1	-1,97204	4,30E-11
HIC2	-1,9729	6,37E-07
CBR3	-1,97388	1,43E-05
PTPRJ	-1,97421	3,68E-14
ITM2B	-1,97478	7,32E-18
DRAM1	-1,97534	4,96E-11
C12orf4	-1,97573	2,07E-13
CYTH3	-1,97653	1,02E-15

RCAN3	-1,97749	9,16E-06
C15orf41	-1,97916	2,30E-05
PRKXP1	-1,97963	0,000183
CDC42EP2	-1,98022	3,11E-13
LEPROT	-1,98334	3,37E-15
GCLM	-1,98645	2,31E-07
P4HA1	-1,98656	1,18E-21
HOXB7	-1,99075	2,18E-10
XRRA1	-1,99176	1,14E-10
PRKACB	-1,99374	1,04E-13
GADD45B	-1,99403	0,000991
KCNJ14	-1,99697	2,52E-05
VAT1	-1,99821	1,51E-10
EFNB1	-2,00207	7,37E-12
SLC39A8	-2,00432	1,18E-09
EIF2AK4	-2,00433	1,89E-22
GRAMD1A	-2,00711	1,18E-26
HCCS	-2,00891	3,55E-14
ME2	-2,00978	1,14E-20
CHFR	-2,01176	2,04E-17
IFNAR2	-2,01204	4,66E-12
USE1	-2,01417	4,05E-15
PAK1	-2,01611	1,01E-08
KIF13B	-2,01622	0,000208
NUMBL	-2,01772	2,32E-19
BIRC2	-2,0197	6,86E-11
GALE	-2,02584	2,79E-09
CAMK1	-2,026	2,16E-06
SNAPC1	-2,02644	2,74E-11
SYVN1	-2,02815	4,14E-16
ZNF486	-2,0285	1,66E-07
MITD1	-2,02874	2,11E-17
GOLGA7B	-2,03181	0,00011
PCDHGB7	-2,03201	0,000221
RIOK3	-2,03215	1,04E-20
SLC39A13	-2,03244	2,17E-16
PDGFA	-2,03383	2,24E-16
SEC61G	-2,0345	1,76E-08
TPK1	-2,03565	6,54E-06
IQCG	-2,03605	2,00E-07
OSTM1	-2,03668	0,000202
KLF13	-2,03732	4,87E-23
SORBS1	-2,03979	7,58E-07
SLC23A2	-2,04121	1,84E-19
PBX3	-2,04195	1,27E-13
TRIB3	-2,04319	7,92E-05
SOCS6	-2,04324	1,70E-14
ADPRHL2	-2,04332	2,74E-22
GATA6	-2,04367	2,50E-07
RBMS2	-2,0451	2,32E-07

SLC1A4	-2,04931	2,53E-06
TTC28	-2,05017	1,74E-11
LOC90768	-2,05136	3,05E-06
ASPHD1	-2,05192	6,04E-08
CLDN23	-2,05309	1,22E-07
TICAM1	-2,05319	1,57E-21
RALA	-2,05362	4,53E-28
SCN8A	-2,05412	0,000154
CASK	-2,05495	6,67E-23
SCYL1	-2,05591	1,92E-20
TMEM106A	-2,05627	0,000149
TRIM21	-2,05627	0,000503
QSOX1	-2,06057	2,53E-24
CDK2AP2	-2,0607	1,41E-26
TBKBP1	-2,0661	7,25E-06
TCF12	-2,06663	4,20E-13
MID2	-2,06767	3,93E-12
ITGB1	-2,0689	9,43E-30
NCEH1	-2,07003	2,69E-11
TNFRSF10B	-2,07117	1,07E-20
EFR3A	-2,07161	4,42E-10
MT1X	-2,07215	1,10E-12
PAM	-2,07263	6,23E-15
DOCK1	-2,07299	4,18E-16
TSHZ1	-2,07563	8,00E-12
SH3GLB1	-2,07579	3,65E-21
BTN2A1	-2,07687	5,19E-06
ARID5A	-2,08004	5,68E-10
FKBP14	-2,08126	6,71E-11
CCM2	-2,08378	7,84E-18
PIP4K2A	-2,0843	1,32E-24
CHRNB1	-2,08442	7,35E-06
PHLDB1	-2,08764	1,18E-21
DENND5B	-2,08849	2,44E-09
SRC	-2,08948	2,62E-20
NRBP2	-2,09174	8,00E-12
RTN2	-2,09344	1,34E-09
S1PR2	-2,09393	7,65E-11
ELOVL2	-2,09415	3,35E-10
C18orf54	-2,09502	9,18E-13
TRAM2	-2,09855	6,07E-28
PANK2	-2,10015	5,49E-20
CAMKK1	-2,10165	6,11E-09
BCL3	-2,1023	6,24E-12
CCNJL	-2,10299	1,22E-06
UBTD1	-2,10332	5,54E-06
SPTBN1	-2,10619	4,63E-36
MET	-2,10722	8,88E-32
SH3D21	-2,11202	1,12E-05
MIB2	-2,11273	3,79E-14

ZNF605	-2,11342	2,92E-06
SH2D4A	-2,11343	6,66E-19
ATP6V1B2	-2,11453	1,24E-24
PPP1R13L	-2,11526	8,40E-13
PGM3	-2,11552	1,39E-12
SCCPDH	-2,1175	2,27E-14
XKR8	-2,11988	6,92E-09
E2F7	-2,12008	1,18E-14
PKD2	-2,12418	4,45E-14
TTL	-2,12949	3,07E-22
HPCAL1	-2,13004	1,27E-23
PLK2	-2,13132	1,26E-08
ACTR3	-2,13342	4,05E-18
MOB3A	-2,1353	1,04E-20
ZNF512	-2,1371	5,60E-17
CAMK1D	-2,13738	3,59E-11
EHBP1L1	-2,14082	4,83E-08
HOOK3	-2,14357	3,98E-14
KCTD12	-2,14408	8,26E-07
GPAT3	-2,14464	3,55E-05
ZAK	-2,14513	1,88E-27
LINC00869	-2,14517	0,000321
NXPE3	-2,14568	0,000133
SBDS	-2,14574	1,57E-27
CDV3	-2,14708	1,73E-20
SHANK3	-2,14748	1,02E-09
DTX3L	-2,14876	1,97E-09
STOML1	-2,14889	1,44E-07
LRRC61	-2,15028	3,41E-13
HTRA1	-2,15139	1,17E-21
RCBTB2	-2,15302	4,23E-08
RPL13P5	-2,1577	0,000837
CLCN6	-2,15776	3,04E-05
UROD	-2,15848	7,38E-19
SDCBP	-2,15925	4,62E-11
CAMK2N1	-2,1595	2,81E-08
CPNE2	-2,16049	5,59E-08
MAP1LC3B	-2,16269	1,19E-26
SLC39A14	-2,16575	2,47E-08
KRBOX4	-2,17068	5,68E-10
KLHL15	-2,17126	4,96E-09
HSD17B12	-2,1723	1,90E-21
GNG12	-2,17266	8,13E-21
HSPB11	-2,17338	5,11E-12
FGD1	-2,17456	8,39E-22
RRAS2	-2,17535	4,64E-15
ANO10	-2,17604	3,83E-10
CSTB	-2,17751	1,79E-20
GPATCH11	-2,17758	7,66E-13
CITED2	-2,17824	2,16E-14

ZNF319	-2,18059	2,37E-05
BRI3	-2,18163	1,82E-33
PFKFB4	-2,18266	2,79E-06
CCDC109B	-2,19228	1,45E-15
MAPKBP1	-2,19234	5,57E-10
TGFB1	-2,19359	3,17E-21
DOPEY2	-2,19482	8,97E-32
IKBIP	-2,20227	1,66E-06
BIVM	-2,20253	2,31E-05
STPG1	-2,20408	1,56E-09
SH3BGRL3	-2,20544	4,93E-09
SLC16A9	-2,20547	6,32E-11
GLDC	-2,20736	7,17E-05
STARD3NL	-2,20767	1,87E-11
CDON	-2,20948	2,99E-05
ASAP1	-2,21095	4,57E-31
HSF4	-2,2131	2,55E-09
TSPAN9	-2,2167	5,66E-07
FAHD2B	-2,21722	4,75E-05
MKNK2	-2,21836	3,07E-24
SYNJ2	-2,21904	4,72E-23
CASP9	-2,22023	4,93E-07
FCRLB	-2,22527	0,000418
HERC3	-2,22542	0,000146
MEIS1	-2,22709	0,000644
TNS3	-2,22807	5,68E-11
PRKAA2	-2,23176	4,45E-16
BNIP3	-2,23653	1,52E-30
TUBB6	-2,23705	3,46E-21
LRRC37B	-2,23843	1,29E-07
STK17A	-2,23896	9,72E-22
DDA1	-2,23903	4,74E-24
ITPR1	-2,23918	4,33E-12
FOXC1	-2,2395	1,62E-19
PDLIM7	-2,2425	5,09E-23
EPHB2	-2,24268	3,47E-25
CCDC150	-2,24296	8,69E-10
SHOX2	-2,24343	8,68E-07
ARSA	-2,24366	3,52E-06
RAB9A	-2,24394	5,93E-20
ZNF296	-2,24439	2,86E-22
HS3ST3B1	-2,24679	2,74E-09
SLC30A7	-2,24943	2,37E-12
RGS14	-2,25129	1,87E-19
SHROOM1	-2,25194	6,14E-16
GSDMD	-2,2544	7,85E-06
APBA3	-2,25612	2,06E-18
LOC100419	-2,25625	2,63E-12
ADCY7	-2,25728	1,19E-18
GDPD5	-2,25951	0,000601

TSHZ3	-2,26171	0,000115
QPCTL	-2,26199	3,15E-14
SHISA4	-2,26763	0,000315
C2orf74	-2,27215	5,87E-10
ZDHHC14	-2,27469	4,40E-10
C20orf194	-2,27605	1,44E-11
FAM50A	-2,27732	1,79E-36
BEND7	-2,27891	1,48E-08
CUEDC1	-2,28575	6,05E-21
MAP3K10	-2,28578	2,79E-18
CCDC15	-2,28821	3,86E-06
GPR180	-2,29362	8,31E-18
SP110	-2,2959	0,000521
FLNA	-2,29967	3,05E-28
PAQR5	-2,30009	2,10E-16
NEDD4L	-2,30019	4,25E-17
IRF9	-2,30158	1,18E-08
RASSF2	-2,30248	8,86E-08
OTUB2	-2,30309	3,96E-05
MAPK12	-2,30866	3,03E-12
PRKD1	-2,30993	6,52E-05
SMYD3	-2,31113	2,18E-11
SNX8	-2,31183	4,41E-19
TNIK	-2,31325	3,65E-13
GALNT10	-2,31694	1,74E-29
CHST10	-2,32097	4,62E-09
TMEM51	-2,32369	1,33E-22
PRPS2	-2,32449	1,51E-21
PDLIM1	-2,33302	3,71E-35
STK39	-2,33427	1,38E-10
TRIM46	-2,33478	5,95E-05
SMPD1	-2,33573	7,96E-06
CYB5R3	-2,3363	4,16E-19
MYO1B	-2,33763	5,19E-41
MUS81	-2,33787	1,63E-09
ZC2HC1A	-2,34182	4,78E-08
STEAP3	-2,34245	1,93E-16
TNFRSF11A	-2,34303	5,19E-09
FOXO3	-2,34496	1,82E-12
TP53BP2	-2,3465	6,49E-22
ZNF266	-2,34695	3,50E-15
FAF1	-2,34826	2,14E-11
KIAA0930	-2,34827	3,23E-16
IL20RB	-2,35011	1,08E-05
HECTD2	-2,35201	1,73E-09
FAM13A	-2,35207	0,000155
NIPAL3	-2,35329	7,63E-09
SPEG	-2,35655	1,12E-06
EFNA5	-2,36004	8,75E-16
KIF5A	-2,36095	0,000445

DNMBP	-2,36798	7,23E-13
TRIB2	-2,37042	3,25E-09
MAPRE3	-2,37115	5,20E-08
IPO13	-2,37322	1,93E-13
EML5	-2,37998	0,000703
CHM	-2,38102	2,48E-07
NFKBIE	-2,38391	8,90E-20
PDE4A	-2,38561	9,23E-17
MICALL1	-2,38861	1,38E-30
TBC1D25	-2,38992	3,62E-10
VEGFB	-2,39201	1,96E-16
GPNMB	-2,39214	2,80E-14
MIR22HG	-2,39514	7,90E-08
ZNF462	-2,39642	9,46E-16
ACTN1	-2,39804	1,65E-20
ADM2	-2,39961	7,34E-06
SLC25A16	-2,40006	7,30E-12
FMN1	-2,40089	3,21E-06
PRTFDC1	-2,40165	4,35E-10
GNG5	-2,40343	1,94E-40
STIM1	-2,40409	6,73E-27
BEX4	-2,40541	4,26E-10
MEGF6	-2,40612	7,59E-10
SH3KBP1	-2,40949	5,13E-14
LOC100506	-2,40963	0,000685
PLK3	-2,41061	8,63E-09
ARNTL	-2,41204	8,36E-08
DDAH1	-2,41281	1,29E-11
PELI1	-2,41346	4,19E-06
DUSP7	-2,41627	1,66E-08
CARD10	-2,42015	8,25E-12
C3orf67	-2,42287	7,77E-06
SLC4A3	-2,42498	3,50E-12
HECW2	-2,4291	1,26E-22
LACC1	-2,43127	5,79E-08
GLIS2	-2,43292	2,59E-28
CCDC88B	-2,43307	0,000116
ETHE1	-2,43322	6,49E-16
RAI14	-2,43596	2,56E-21
SMARCD3	-2,43745	2,96E-12
SLC25A22	-2,43835	5,73E-32
DENND5A	-2,43887	1,71E-15
FSCN1	-2,43912	5,90E-32
SEMA4B	-2,4418	1,35E-29
LOC100130	-2,44296	7,40E-06
TMCC1-AS1	-2,44358	5,89E-05
FAM57A	-2,44454	8,09E-18
C11orf68	-2,44794	7,52E-31
KLF12	-2,4482	3,03E-12
CCDC93	-2,44859	2,72E-25

MAPK8IP3	-2,45092	3,45E-06
NAB1	-2,4575	8,29E-14
ANKRD13A	-2,46023	1,98E-11
DPY19L2P1	-2,46046	0,000122
ARMCX1	-2,4627	3,63E-06
SRGAP1	-2,4644	1,60E-14
APP	-2,46509	2,21E-48
CHST15	-2,46834	4,00E-30
KLHL21	-2,47145	4,03E-32
FERMT2	-2,47289	9,53E-30
TENM4	-2,47373	4,04E-09
PQLC2	-2,47717	1,82E-18
SERINC2	-2,4824	6,94E-13
SDC2	-2,48263	2,92E-22
WSCD1	-2,48619	4,25E-15
EHD4	-2,48952	1,99E-27
SIRT2	-2,49063	2,31E-15
LRRC8E	-2,4919	3,25E-19
TPST2	-2,49713	3,94E-14
UNC13A	-2,50031	0,000545
CAP1	-2,50033	1,05E-26
CHST11	-2,50162	6,60E-18
CXCR4	-2,50355	1,58E-15
WWTR1	-2,5054	3,24E-39
MYH9	-2,50774	7,58E-26
IFI27L2	-2,51135	0,000244
CCDC18	-2,51569	2,61E-08
SUSD6	-2,51809	2,15E-17
CAPRIN2	-2,51882	7,32E-17
KLF6	-2,52358	1,44E-07
CLIP2	-2,52888	2,50E-27
LHFPL2	-2,53131	3,77E-29
CSRNP1	-2,53316	8,53E-17
RBCK1	-2,53409	4,96E-47
INSIG2	-2,53468	1,37E-12
GANC	-2,53785	1,97E-12
STK17B	-2,54061	4,92E-17
SWAP70	-2,54358	2,59E-24
FAH	-2,54714	4,86E-31
WDR66	-2,54979	2,87E-05
HDAC4	-2,55449	0,000105
PLEC	-2,55463	1,10E-09
GALNT14	-2,55758	2,17E-36
GALNT2	-2,55775	5,62E-28
GALNT18	-2,56064	5,97E-21
GPC1	-2,56575	7,56E-20
STXBP1	-2,56668	1,25E-28
GPR157	-2,57136	4,87E-05
PML	-2,57494	8,57E-10
IFNLR1	-2,57532	1,14E-10

KLHL5	-2,57565	3,55E-27
MED8	-2,57625	1,07E-29
ZBED6CL	-2,58468	1,96E-14
MAMLD1	-2,58716	4,36E-09
DOCK10	-2,58738	5,28E-07
PITPNM2	-2,59049	5,22E-16
VASP	-2,59498	2,62E-42
LRP1	-2,59809	4,20E-06
DRAP1	-2,59829	4,11E-38
ERAP1	-2,60139	9,15E-30
KAZN	-2,60151	2,44E-20
PRSS53	-2,61254	1,01E-08
CAMK2D	-2,61362	4,15E-21
RAB31	-2,61597	9,22E-10
TNS1	-2,61812	1,14E-12
PDP1	-2,61875	3,39E-30
SLC22A4	-2,61976	4,37E-07
ATP2B4	-2,61986	5,85E-14
TMEM44	-2,62214	1,50E-15
PAQR3	-2,62623	7,82E-24
HOMER3	-2,62736	4,08E-43
TMEM86B	-2,62816	8,39E-09
TRAF5	-2,63019	1,18E-15
TPM4	-2,63072	8,80E-58
SCMH1	-2,63218	1,85E-08
NTN1	-2,63289	2,50E-13
FAM114A1	-2,63331	2,92E-09
MAP7D1	-2,63337	6,22E-26
FOLR1	-2,6386	1,46E-05
FGF18	-2,63972	1,19E-06
PLOD1	-2,65961	3,26E-29
SPAG4	-2,66077	4,55E-08
SPTBN4	-2,66094	2,22E-07
SYBU	-2,66483	2,85E-11
LBX2-AS1	-2,66602	1,88E-05
COLGALT1	-2,66764	1,96E-52
RBPMS	-2,67241	4,17E-40
CKAP4	-2,67605	5,13E-44
TAP2	-2,67853	2,31E-21
MALT1	-2,68128	6,15E-40
PTGER4	-2,68872	2,26E-06
PMEPA1	-2,68978	1,64E-31
TMX3	-2,69207	1,16E-23
RTTN	-2,69378	1,59E-13
TREX1	-2,69503	4,28E-11
LIMA1	-2,69699	3,77E-13
VCL	-2,69864	3,08E-16
ST5	-2,69901	2,03E-12
PALLD	-2,70032	4,51E-21
ABHD6	-2,70045	8,80E-11

SLC41A2	-2,7039	1,21E-16
TGFBR2	-2,70499	1,33E-16
LOC103091	-2,70529	2,18E-07
ZIC5	-2,7054	0,000361
BTBD11	-2,70651	8,07E-12
DDX58	-2,70727	7,60E-07
CDR2L	-2,7076	5,90E-16
PEA15	-2,70937	3,70E-33
AMIGO3	-2,71419	6,65E-17
RTCA	-2,71518	4,41E-38
BOLA3-AS1	-2,71676	1,44E-06
HTATSF1P2	-2,71999	8,60E-07
IFNGR2	-2,73052	2,82E-19
AK4	-2,73263	9,03E-42
LRP4	-2,73281	4,90E-12
CTSB	-2,7355	1,31E-17
IFFO2	-2,73621	1,82E-25
BMP1	-2,73821	3,26E-33
SMAD3	-2,73841	7,13E-17
LINC01128	-2,73896	1,94E-13
NKX3-1	-2,74438	2,29E-11
COTL1	-2,74916	9,08E-38
ACSL4	-2,74925	4,98E-23
PLP2	-2,75094	5,20E-44
MATN2	-2,75513	1,33E-31
ISM1	-2,75863	5,19E-10
ALDH3B1	-2,7593	9,87E-06
AMIGO2	-2,76059	9,03E-25
CDK14	-2,76096	6,42E-10
INHBB	-2,76422	6,45E-24
PRKCE	-2,76736	1,59E-07
FNDC3B	-2,7679	5,24E-47
LOC171391	-2,76794	8,94E-05
ANKMY2	-2,76851	3,33E-24
RAB3B	-2,77158	1,23E-12
LDHA	-2,7985	1,40E-54
LRRC6	-2,79877	2,38E-05
LMF2	-2,79974	2,32E-31
HSPG2	-2,81322	4,06E-28
GUCY1B3	-2,8134	2,33E-10
SOAT1	-2,81369	1,62E-28
IL6ST	-2,8188	6,51E-26
EVA1B	-2,82872	2,93E-09
PTGR1	-2,82996	1,21E-12
VLDLR	-2,83332	3,65E-07
PRDM11	-2,83432	9,61E-10
RP2	-2,83448	1,09E-12
FYN	-2,83681	3,86E-12
OSMR	-2,8371	1,17E-14
CD59	-2,83893	2,03E-15

FAM129A	-2,83946	5,00E-07
PDGFC	-2,84193	1,54E-07
SGPP2	-2,84448	9,16E-11
SGTB	-2,84631	6,10E-18
GBX2	-2,84935	0,000335
PITPNM1	-2,85052	1,34E-23
SH3TC1	-2,85303	2,16E-13
CACNA2D3	-2,85416	0,00019
DPY19L2P2	-2,85453	0,000412
ARL9	-2,85706	4,05E-05
CXCL12	-2,85954	3,75E-24
PLA2G4C	-2,86306	0,000903
GPR68	-2,86745	3,51E-12
SH3BGRL	-2,86787	2,76E-17
RPS6KA2	-2,86861	5,87E-19
CD27-AS1	-2,87064	0,000601
COL23A1	-2,87293	0,000117
C16orf62	-2,87389	1,54E-21
SLC8B1	-2,87433	2,27E-20
CARS2	-2,87832	1,33E-45
MCOLN1	-2,88047	7,01E-26
HNRNPU-A	-2,88309	2,59E-07
C9orf72	-2,88311	0,000981
ABTB2	-2,88496	6,41E-36
PPAP2B	-2,88509	3,28E-14
ZNF155	-2,88994	1,96E-08
SASH1	-2,89258	2,46E-09
LINC01116	-2,89575	3,97E-21
FUT4	-2,89591	4,17E-12
SDC4	-2,89702	4,73E-24
NUPL2	-2,89781	7,30E-10
SNHG15	-2,89983	4,77E-07
LAMA3	-2,90023	4,08E-28
LDLRAD4	-2,90699	0,001006
CYLD	-2,90948	2,08E-07
ALDH1A3	-2,91122	3,09E-05
FSTL3	-2,91175	2,23E-15
ACO1	-2,9146	1,07E-16
RRAGC	-2,92488	6,31E-36
FAM89A	-2,93067	4,57E-07
SPAG1	-2,93209	9,19E-13
ZNF774	-2,9341	6,90E-05
BICD1	-2,94013	7,29E-29
PPP1R18	-2,94463	4,25E-35
FHL3	-2,94484	3,37E-35
GOLM1	-2,94561	1,61E-49
IFITM3	-2,95377	1,07E-13
THEMIS2	-2,9549	2,16E-06
KLF8	-2,95922	1,66E-08
APBA1	-2,9664	8,05E-14

MICB	-2,97034	2,32E-14
ADGRE5	-2,97274	5,12E-16
LOC730102	-2,9745	5,78E-05
SLC2A1	-2,9753	3,27E-19
ERRFI1	-2,97756	3,28E-18
DNM1	-2,98098	1,68E-31
GATA2	-2,98746	3,13E-24
FAM126A	-2,98995	6,33E-24
DYRK3	-2,9955	4,55E-30
LOC100507	-2,99731	0,000176
SLC37A2	-3,00083	1,40E-05
RIMS2	-3,00285	1,50E-09
DCLK2	-3,00513	3,76E-08
ALS2CL	-3,00845	1,24E-11
ARFGAP3	-3,01659	5,66E-35
PLXNC1	-3,02377	0,000571
PPARG	-3,02461	6,49E-14
CTSL	-3,03016	1,45E-18
PFKP	-3,03103	2,08E-29
OGFRL1	-3,03242	1,71E-32
LYPD1	-3,03531	8,04E-20
FILIP1L	-3,03838	2,50E-09
RNF207	-3,03928	1,44E-10
NES	-3,04004	4,36E-37
DSE	-3,04032	1,29E-17
BHLHE40	-3,0436	1,69E-41
SH3PXD2B	-3,04375	1,20E-42
SORBS3	-3,04524	1,58E-55
ICAM5	-3,04841	8,88E-07
CYSTM1	-3,0531	2,68E-54
RAET1K	-3,05491	0,000242
FUT11	-3,05731	5,85E-25
KCNS3	-3,05809	1,57E-10
TCF7	-3,05815	7,96E-28
ADCY9	-3,06389	8,70E-21
MTSS1	-3,06804	3,64E-07
CCNYL1	-3,06988	1,04E-24
ZNF697	-3,07069	4,91E-24
PORCN	-3,07213	2,22E-15
LINC00623	-3,07267	4,29E-16
HOXA4	-3,07521	9,14E-11
RIN3	-3,07673	1,28E-15
PXDN	-3,07753	1,20E-31
NXN	-3,081	1,09E-39
LPCAT2	-3,08471	5,16E-16
DCBLD2	-3,08525	1,20E-26
MCC	-3,09118	1,20E-20
TPST1	-3,09191	1,08E-18
RENBP	-3,09281	5,98E-10
GPR39	-3,09634	2,21E-10

TAP1	-3,09844	1,85E-11
CDK6	-3,10065	5,45E-09
POU3F3	-3,10152	6,51E-14
ZMYM6NB	-3,10208	1,16E-13
ELL2	-3,10239	3,94E-22
OTUD1	-3,10392	9,49E-15
ARNTL2	-3,10904	2,60E-22
MB21D1	-3,11018	6,99E-19
PDK1	-3,11392	1,19E-17
ULBP3	-3,11737	0,000136
HOXC4	-3,11782	2,16E-09
MXRA7	-3,12052	4,92E-22
HES4	-3,12361	1,60E-33
CA12	-3,12425	4,26E-09
CEP112	-3,1272	1,91E-11
NFIL3	-3,1274	1,24E-09
ALOXE3	-3,12784	2,28E-12
TRPM2	-3,13351	7,48E-36
OCIAD2	-3,1338	4,69E-40
ARHGAP26	-3,13426	4,05E-10
MARCKS	-3,13469	8,96E-26
TMEM163	-3,1535	2,45E-17
GPRC5B	-3,155	3,74E-12
JAK1	-3,15504	7,04E-43
HACD1	-3,15536	8,87E-23
TSPAN10	-3,157	0,000312
LIMS1	-3,15856	1,79E-46
CCDC82	-3,16242	6,83E-12
SPRY2	-3,16755	4,72E-14
FOSL2	-3,17175	4,65E-40
WWC3	-3,1748	2,27E-52
NAGS	-3,17595	0,000471
PHLDA2	-3,1786	3,35E-11
TSPAN4	-3,18118	1,75E-21
HLA-F-AS1	-3,18152	0,000182
TFEB	-3,18288	2,84E-10
SNAI1	-3,18378	1,07E-11
DYNLT3	-3,18473	2,34E-13
C12orf75	-3,18614	1,28E-51
PFKFB3	-3,18918	1,04E-19
FABP5	-3,19366	1,29E-60
TRIM7	-3,19383	2,98E-10
SPNS2	-3,19536	6,74E-16
PVR	-3,19907	1,74E-18
AP1S2	-3,20064	1,08E-27
ZCCHC24	-3,2011	4,24E-12
PMAIP1	-3,20118	4,48E-19
EHD3	-3,20378	6,40E-22
HNMT	-3,20448	1,64E-08
PAOX	-3,21252	1,42E-10

LOC101927	-3,21283	1,41E-09
HSD17B14	-3,21335	2,23E-23
KLF7	-3,2245	1,22E-40
A4GALT	-3,22462	1,62E-08
WHAMMP3	-3,22512	5,80E-06
CDKL1	-3,22719	2,37E-15
HERC6	-3,22915	1,43E-07
MACROD2	-3,23321	8,42E-13
ANXA3	-3,24017	1,92E-20
TBC1D2	-3,25323	5,49E-13
ADPRH	-3,25871	1,37E-10
ZYX	-3,25978	2,68E-42
LRP12	-3,26014	3,64E-60
STARD8	-3,27107	1,67E-11
LETM2	-3,27344	2,44E-07
PPP2R5B	-3,27996	1,20E-33
TMEM249	-3,28346	0,000376
FMNL1	-3,28494	3,19E-40
MT1F	-3,28807	1,82E-16
CRIM1	-3,28814	6,51E-26
SCN1B	-3,29344	4,42E-07
OSBPL6	-3,29354	1,36E-09
BARX2	-3,2999	6,36E-06
TRIM6	-3,30621	0,000896
ZNF441	-3,30683	4,44E-07
SEC14L1P1	-3,30812	0,000643
LGALS1	-3,31619	3,36E-26
RIPK4	-3,31676	9,88E-46
SEC14L2	-3,32418	1,01E-13
P3H2	-3,32586	2,28E-46
EFNB2	-3,34394	5,38E-42
ITPRIP	-3,34405	9,29E-14
RCAN1	-3,34755	4,36E-24
IFIH1	-3,34899	1,04E-09
SLCO3A1	-3,35273	3,54E-36
THBD	-3,3541	8,60E-26
C3orf18	-3,35629	0,000102
NFKBID	-3,35689	4,04E-07
FOXD1	-3,35914	1,35E-07
ELK3	-3,35989	7,86E-18
ZBTB47	-3,37594	3,17E-07
STAT1	-3,37675	1,68E-19
IRF5	-3,37919	2,62E-19
PTPN14	-3,37921	2,68E-58
CHST7	-3,38078	4,06E-12
GADD45A	-3,38153	5,46E-42
KIAA0226L	-3,38266	0,000239
TMEM55A	-3,38482	5,80E-37
SMARCA1	-3,38546	9,77E-16
ISG20	-3,38888	2,35E-16

DAGLA	-3,39193	2,89E-22
TLN2	-3,39251	7,44E-29
APOBEC3B	-3,39403	5,16E-35
ZBTB38	-3,39482	2,68E-48
PARVA	-3,39582	1,53E-40
DLX2	-3,39792	4,23E-33
GLB1L	-3,40119	1,16E-16
VSTM2L	-3,40295	4,75E-15
CILP2	-3,40355	3,27E-13
PLOD2	-3,40504	3,88E-58
CTH	-3,40521	1,91E-17
PPP1R3B	-3,40536	2,05E-53
TMEM133	-3,40601	0,000752
MOCOS	-3,40741	2,35E-19
IGF2BP2	-3,40791	5,64E-40
SPATS2L	-3,41126	2,26E-56
ENPEP	-3,41377	2,12E-28
IFI35	-3,41587	6,67E-13
ELFN2	-3,41657	2,64E-08
GPC4	-3,41688	1,05E-44
HOXC8	-3,42685	0,000539
IRS2	-3,43127	4,60E-20
TMEM56	-3,43551	6,67E-20
RNASET2	-3,44018	5,18E-41
FNIP2	-3,44692	1,73E-10
EBF4	-3,45019	0,000268
LTBP2	-3,45045	4,92E-16
BDKRB2	-3,45169	0,000702
TTN-AS1	-3,45209	3,20E-17
CD44	-3,45936	9,96E-59
FRMD3	-3,46712	3,20E-10
POU6F1	-3,46787	8,78E-14
FRMPD3	-3,4727	1,21E-06
GLS	-3,47524	2,80E-43
GBP2	-3,47648	7,45E-05
CCDC106	-3,48118	1,28E-08
TUBB2B	-3,48203	1,85E-19
KCNIP3	-3,48209	3,14E-44
SLFN11	-3,48717	1,43E-21
CCDC88A	-3,48851	2,85E-38
SIX2	-3,49488	3,36E-10
STRIP2	-3,49565	2,03E-18
MFSD1	-3,50692	4,79E-33
TGFA	-3,51301	4,45E-21
HOTAIRM1	-3,51703	1,12E-06
SLC2A6	-3,51966	5,53E-12
NAV1	-3,52044	6,38E-16
GOLGA8B	-3,52476	5,54E-08
IL1R2	-3,5267	1,50E-06
PLXNA2	-3,52881	1,37E-07

SOX7	-3,53517	5,85E-05
NFKBIZ	-3,54171	1,01E-17
EGLN3	-3,54247	7,74E-35
PHYH	-3,54669	6,56E-34
NFKB2	-3,54892	3,15E-48
MIRLET7BH	-3,55038	5,53E-07
TMEM217	-3,55466	2,78E-09
STAMBPL1	-3,55602	1,60E-09
IL1RAP	-3,55696	1,55E-12
DEGS1	-3,55929	5,69E-37
ZNF513	-3,55999	1,41E-06
LINC01508	-3,56587	7,53E-07
FAM219A	-3,5719	1,85E-22
GRB10	-3,57563	7,60E-10
RAB6B	-3,57583	5,87E-26
BHLHA15	-3,57664	3,25E-08
TMSB4X	-3,58301	1,24E-92
EPB41L4A	-3,5862	3,62E-15
ZNF532	-3,58973	2,53E-52
PTPRE	-3,59245	6,13E-08
WNT5B	-3,59403	8,70E-10
HOXB6	-3,59753	0,000239
BISPR	-3,5984	3,90E-10
MAPK8IP2	-3,59929	3,87E-16
HELZ2	-3,60513	4,25E-15
FKBP9P1	-3,61066	2,60E-06
SAMD14	-3,61502	5,92E-07
ST3GAL1	-3,62455	7,28E-22
PRNP	-3,62643	2,79E-41
KLHL29	-3,62696	1,61E-20
PKP1	-3,62913	6,78E-13
APOL2	-3,63248	1,35E-09
CDK5R2	-3,63279	0,000993
FAM63B	-3,63419	1,87E-20
CD83	-3,63918	3,73E-36
SLCO4A1	-3,64491	1,82E-26
FAM71F2	-3,64598	1,60E-05
PPM1K	-3,65188	1,54E-11
LIMD2	-3,65768	6,69E-20
PLA2R1	-3,66763	0,000291
SAMHD1	-3,66853	6,41E-18
HAGLR	-3,67348	2,98E-34
FGFR1	-3,67545	1,11E-86
SLC35E4	-3,67677	1,03E-37
LINC01410	-3,68062	5,48E-05
SNX10	-3,68271	1,74E-22
P3H1	-3,68319	2,14E-67
SERPINB8	-3,68411	2,00E-20
HSPA12A	-3,68468	1,56E-37
PXDC1	-3,69329	7,08E-51

MTCL1	-3,70296	4,65E-30
RUSC2	-3,70324	1,72E-25
ADORA1	-3,70649	2,12E-18
LGALS3BP	-3,71644	0,000391
IFITM1	-3,72066	1,40E-10
ADORA2B	-3,73045	2,86E-25
HYAL3	-3,73359	2,31E-12
EFHD2	-3,74344	7,17E-56
RRAGD	-3,7497	7,52E-26
UBE2L6	-3,75192	6,03E-15
ARL4C	-3,75282	1,49E-77
GGT1	-3,75518	1,24E-62
LIMCH1	-3,76166	5,07E-11
SYDE1	-3,76373	2,41E-42
PITX2	-3,77118	2,34E-13
THRB	-3,77451	2,62E-23
IFIT5	-3,7805	4,31E-18
PLSCR1	-3,78337	2,13E-68
IL11	-3,78715	1,51E-05
DGCR5	-3,8003	3,42E-26
RASGRP1	-3,80199	5,14E-15
ATAD3B	-3,80655	9,33E-27
LOXL1	-3,80977	6,91E-36
POU2F2	-3,80983	2,73E-05
FOXO1	-3,81389	5,35E-21
IL4R	-3,82119	2,46E-26
HOXA11	-3,8215	3,72E-08
PROCR	-3,82802	5,05E-08
GLIPR2	-3,83006	1,33E-30
ENO2	-3,83424	2,90E-33
CREG2	-3,83527	7,44E-05
ADAMTS9	-3,83826	1,15E-10
LZTS3	-3,84147	2,81E-15
C14orf159	-3,84216	3,82E-20
TPM1	-3,85105	8,61E-109
PTRF	-3,85112	4,41E-61
GAB3	-3,85161	0,000408
AMZ1	-3,85306	3,12E-20
COL5A2	-3,85724	1,16E-39
HYI	-3,86201	1,33E-20
THBS3	-3,86318	1,62E-61
PLEKHA2	-3,8658	2,24E-32
TRNP1	-3,87669	1,96E-37
GFPT2	-3,87983	1,61E-30
RHEBL1	-3,8819	6,41E-09
FAM228B	-3,89046	8,43E-11
CLCF1	-3,8915	4,98E-09
VEPH1	-3,89705	4,32E-11
UAP1L1	-3,89759	2,31E-72
IRF1	-3,89962	1,72E-40

SPSB1	-3,90769	5,79E-18
CTTNBP2	-3,90945	0,000862
NPAS2	-3,91099	8,52E-37
TRANK1	-3,91207	6,54E-06
BST2	-3,91639	2,96E-12
CDKL5	-3,91644	1,10E-12
ODF3B	-3,91727	5,16E-09
ITPKA	-3,92387	1,95E-22
SMIM3	-3,92884	9,72E-68
FLT3LG	-3,93842	1,65E-12
LYST	-3,94039	1,03E-24
ROBO3	-3,94525	4,23E-18
TIMP2	-3,95413	1,88E-81
TNFSF9	-3,95821	2,28E-10
GABARAPL	-3,95988	1,61E-07
SETBP1	-3,96193	7,06E-13
SLC6A8	-3,96851	8,50E-26
ARL4D	-3,97109	4,11E-38
RAB11FIP5	-3,97715	8,49E-20
MYLK	-3,98047	8,96E-41
HBEGF	-3,98426	3,94E-26
FAM92A1	-3,98506	2,87E-16
IRF7	-3,98793	1,65E-24
ST6GALNA(	-3,99399	1,77E-26
RRAS	-3,99673	1,93E-28
APBB1	-4,00106	6,64E-19
ADAP2	-4,00335	1,13E-32
ITGA6	-4,00574	7,03E-53
ANK2	-4,00591	1,01E-13
PLBD1	-4,0096	2,41E-33
LMO1	-4,01005	2,09E-06
SLC16A3	-4,01052	4,68E-18
SFXN3	-4,01117	7,18E-47
ISPD	-4,01144	5,64E-06
GPER1	-4,02083	7,31E-18
PLEK2	-4,02171	3,97E-67
TGFB2	-4,02192	1,12E-10
COL14A1	-4,02457	9,47E-10
DDX11L2	-4,02767	3,94E-06
ASPHD2	-4,02798	1,14E-34
CYP4V2	-4,03026	9,96E-13
EREG	-4,03227	8,94E-05
KBTBD11	-4,03699	1,40E-22
HOXD8	-4,04133	1,63E-13
SUSD1	-4,04255	5,36E-55
GRK5	-4,04567	1,16E-23
PGM1	-4,05402	1,29E-50
PTGES	-4,05583	4,51E-21
MICAL1	-4,05894	3,94E-32
NCALD	-4,06069	4,13E-08

CRAT	-4,06085	1,94E-17
OAS3	-4,06853	0,000103
BCL2L15	-4,06914	6,37E-12
IL6R	-4,0769	4,83E-09
TBX19	-4,07822	0,000846
B3GNT5	-4,07938	4,64E-08
MAPRE2	-4,09006	1,01E-37
LCP1	-4,09159	5,00E-18
CMTM7	-4,09415	1,91E-28
GNAI1	-4,09852	9,75E-27
HPGD	-4,11329	3,35E-08
BTN3A3	-4,11369	1,99E-09
DNAH17	-4,11601	5,26E-08
CLTCL1	-4,12191	1,62E-41
CAMK4	-4,13368	1,49E-10
ARHGAP29	-4,13388	3,52E-37
SH3RF2	-4,1354	3,53E-17
MYADM	-4,14164	3,30E-34
PCOLCE2	-4,14741	3,56E-41
S100A6	-4,15312	1,82E-37
SCARF2	-4,159	2,28E-40
AMPD3	-4,15996	3,67E-24
PPP1R15A	-4,16071	4,60E-50
NMI	-4,16379	1,60E-29
WTIP	-4,16393	2,62E-34
P4HA2	-4,17683	2,39E-43
CMTM3	-4,17871	2,83E-33
RND3	-4,18262	4,74E-66
DENND3	-4,18443	7,17E-19
ECE1	-4,18809	2,59E-102
PARP12	-4,18835	3,97E-27
FABP6	-4,18981	3,03E-24
CPQ	-4,19062	1,61E-18
TOR4A	-4,19066	1,94E-43
REC8	-4,20882	8,41E-06
KIF26B	-4,21314	7,57E-36
CCDC68	-4,21676	4,14E-09
FAM171A1	-4,21895	1,31E-69
DDX60L	-4,22185	4,08E-26
ERO1B	-4,22286	4,38E-05
L1CAM	-4,22378	3,13E-37
NUAK2	-4,2269	3,07E-61
STEAP1	-4,23967	8,45E-17
CPM	-4,25133	3,48E-15
ADRA2A	-4,25863	5,06E-06
ASPH	-4,26162	1,34E-52
COLEC11	-4,26291	0,000217
HLA-C	-4,26559	1,55E-15
MTAP	-4,2717	8,80E-13
TBXAS1	-4,27478	3,93E-08

SERPING1	-4,28084	1,67E-05
C10orf54	-4,28087	0,000279
GPR3	-4,28101	0,000967
ADGRF4	-4,28482	3,02E-13
VAMP5	-4,28985	5,95E-11
TNFRSF11B	-4,29417	2,49E-27
HHEX	-4,29537	6,92E-26
FAM101B	-4,3004	2,71E-51
SIK1	-4,31395	3,00E-06
HIVEP3	-4,31934	2,12E-12
JAG1	-4,32246	2,70E-73
MDGA1	-4,3305	7,89E-20
P4HA3	-4,3335	0,000773
RASD2	-4,34206	4,48E-18
AFF3	-4,34401	1,01E-43
RASGEF1B	-4,34818	1,08E-15
SPTLC3	-4,34862	2,73E-09
SLC12A4	-4,35097	1,62E-13
MFGE8	-4,35213	2,11E-69
EPDR1	-4,36043	9,89E-49
UBASH3B	-4,36718	1,65E-28
AHNAK2	-4,36908	3,27E-83
TRIP10	-4,37082	6,84E-44
LGALS3	-4,37709	8,02E-35
C3orf52	-4,37786	1,76E-08
PBX4	-4,37929	1,95E-25
FAM20C	-4,38347	4,25E-95
B3GNT9	-4,38534	1,62E-11
ADAMTSL3	-4,38861	7,27E-28
DLGAP1-AS	-4,38945	4,57E-16
PXK	-4,39073	7,13E-21
RGAG1	-4,39937	8,93E-09
DMBX1	-4,40311	6,28E-08
BTN3A2	-4,40724	1,09E-12
SLC30A4	-4,41739	1,53E-08
HSPB6	-4,41999	5,99E-08
ZNF257	-4,4204	2,83E-11
LPXN	-4,4213	2,08E-56
SLC16A1-A	-4,4333	1,91E-06
CLCN4	-4,43591	4,34E-27
GNG4	-4,44031	2,39E-25
KIAA2022	-4,45098	2,44E-07
LRRC8C	-4,45219	2,75E-31
ARHGAP23	-4,45527	1,86E-21
NAV2	-4,45548	1,17E-78
TMEM140	-4,46493	8,06E-14
HOXA7	-4,46598	2,31E-09
NOD2	-4,46961	4,15E-11
MGLL	-4,47081	8,77E-28
MAP1B	-4,47226	3,63E-33

LINC00152	-4,47282	1,53E-27
CUBN	-4,47489	8,97E-06
B2M	-4,47811	3,18E-44
ISG15	-4,48094	5,19E-05
SIPA1	-4,48323	3,88E-58
RPS2P32	-4,4871	3,48E-05
SYNM	-4,49129	2,56E-49
PALM2	-4,49296	3,89E-17
DLK2	-4,49974	8,32E-09
FAM189A2	-4,5014	6,65E-14
NAP1L5	-4,50286	0,000258
FRMD5	-4,50468	2,87E-50
SEMA5B	-4,50902	0,000949
FNDC4	-4,51068	1,66E-10
TLL2	-4,51163	9,04E-19
STARD9	-4,52753	6,98E-17
ARAP3	-4,53007	1,89E-25
RGS2	-4,53276	4,96E-27
ALDH1L2	-4,54258	0,000864
IFI6	-4,54294	1,41E-05
AKAP12	-4,54535	2,38E-49
IL10RA	-4,5463	0,000382
LOC440028	-4,55184	0,000215
EDIL3	-4,56276	3,63E-12
NCR3LG1	-4,57213	2,95E-32
RIN2	-4,58192	3,09E-28
MYO1E	-4,58261	3,20E-37
TIMP1	-4,58479	1,11E-18
EVC2	-4,58687	7,11E-23
SOX9	-4,59726	1,54E-22
EXT1	-4,60437	4,32E-89
PGF	-4,60908	6,38E-11
AOX1	-4,63116	5,27E-08
LIF	-4,63719	5,95E-19
LRRK1	-4,6392	4,60E-37
LIX1L	-4,64081	7,47E-38
PRKCA	-4,65297	8,75E-49
ABCB1	-4,65831	7,38E-11
MAP3K8	-4,65962	2,45E-23
CHST1	-4,66203	2,51E-12
TM4SF1	-4,66965	4,29E-38
CYP26B1	-4,6753	2,75E-10
ITGA3	-4,67977	6,31E-139
TAPBPL	-4,68386	1,15E-18
CNTNAP1	-4,69867	1,86E-55
DSEL	-4,705	1,55E-22
ZNF264	-4,71778	9,74E-44
CACNB4	-4,73512	2,77E-08
NUDT11	-4,73645	7,37E-19
ROR1	-4,74582	4,81E-55

SERPINB9	-4,74587	8,00E-35
MRAS	-4,75403	5,03E-63
CD163L1	-4,75412	3,28E-11
SYNE1	-4,75481	7,00E-23
PLEKHF1	-4,75519	2,03E-26
XKR6	-4,75697	4,50E-06
FAT2	-4,75804	0,000266
PGBD5	-4,75944	6,85E-60
ULBP2	-4,76375	5,61E-11
TRIM47	-4,78666	5,95E-56
CHST3	-4,78742	1,62E-16
PTGS1	-4,79839	1,22E-06
EPHA2	-4,80074	2,31E-41
PPP4R4	-4,81081	6,12E-09
TMCC3	-4,81427	6,93E-29
TRPC1	-4,82096	1,04E-34
SMOX	-4,82449	0,000123
SQRDL	-4,82973	6,62E-40
CAV2	-4,83502	5,21E-63
SH2B3	-4,83738	9,83E-90
CFB	-4,84075	9,27E-12
ARMC4	-4,84987	2,82E-09
ECM1	-4,85087	1,11E-14
CD74	-4,85108	4,65E-12
MID1	-4,85288	2,76E-21
SPTBN5	-4,85575	9,45E-10
ZNF382	-4,86884	5,61E-16
PAK3	-4,87323	8,67E-05
SPRED1	-4,87491	1,85E-32
CREB5	-4,88	0,000506
RELB	-4,88351	1,39E-42
HERC5	-4,88408	1,09E-09
TUBB2A	-4,91018	5,27E-33
SPHK1	-4,91308	2,96E-102
ME3	-4,91796	3,12E-11
SNX7	-4,92406	1,00E-52
OAF	-4,93579	1,36E-90
DIRC3	-4,95669	7,83E-07
FAM46A	-4,96007	7,95E-54
CRIP1	-4,96218	1,12E-35
EMILIN2	-4,96226	5,67E-21
LINC00862	-4,9625	1,45E-07
PARP10	-4,97862	9,88E-46
GPR176	-4,98754	9,91E-32
OLFML2A	-4,99539	2,34E-32
S100A2	-5,00645	5,90E-14
CCDC80	-5,01356	2,24E-26
GFRA2	-5,01896	4,30E-23
SH2D5	-5,02914	2,87E-45
CNR1	-5,02985	1,09E-16

SEPT10	-5,0373	6,69E-36
CD22	-5,04261	0,000293
FXYD5	-5,06157	1,94E-96
MBP	-5,062	6,43E-59
MN1	-5,06762	1,57E-07
CALD1	-5,06879	9,76E-24
ENPP1	-5,0897	1,56E-28
FAM149A	-5,10117	2,36E-10
LAMB3	-5,10612	0,000573
YOD1	-5,11006	3,32E-22
IL1R1	-5,11752	5,97E-18
CAV1	-5,11891	2,41E-71
GCNT2	-5,12946	0,000835
PLEK	-5,13118	3,41E-08
PROS1	-5,13442	1,41E-17
GULP1	-5,14202	4,37E-61
ETV4	-5,15537	3,74E-13
ANKRD29	-5,15598	2,63E-18
PLBD1-AS1	-5,16359	0,000569
ZNF702P	-5,17291	5,58E-41
IRAK2	-5,1755	2,03E-06
SYNC	-5,18347	1,27E-44
COPZ2	-5,19287	2,11E-54
CPED1	-5,19554	1,92E-06
IQCA1	-5,19762	1,87E-05
CPEB1	-5,19802	1,05E-46
HOXA-AS2	-5,19862	1,01E-10
BCAR3	-5,20593	2,62E-118
NOV	-5,21107	0,000317
TUBA4A	-5,21356	1,69E-21
KIF7	-5,22834	0,000269
IFI44L	-5,23985	0,00012
LGALS9	-5,24126	2,06E-11
PRUNE2	-5,24301	4,69E-23
CORO2B	-5,24787	9,46E-13
IL15RA	-5,24801	8,11E-66
CAPG	-5,25327	1,95E-181
MAP7D2	-5,25728	5,15E-07
ARHGEF6	-5,26152	5,33E-57
MMP24	-5,26309	3,67E-30
RTN4RL2	-5,26339	4,89E-31
QPCT	-5,26371	6,89E-47
ITGAM	-5,26399	7,07E-05
CMPK2	-5,27252	1,00E-22
KCNMA1	-5,27324	5,41E-34
PRRT3-AS1	-5,27558	1,53E-05
SOWAHC	-5,28819	1,09E-60
ETV7	-5,29173	2,53E-12
PPP1R1A	-5,30395	2,36E-28
CRYBG3	-5,30956	1,69E-31

DUSP6	-5,3149	3,50E-10
ZBTB18	-5,33338	1,38E-17
COL16A1	-5,34473	3,43E-48
TAGLN	-5,35458	1,26E-51
HLA-F	-5,36138	1,64E-31
GJB2	-5,36227	1,36E-68
HOXB4	-5,37709	8,48E-06
MICAL2	-5,38548	4,01E-100
C19orf66	-5,3874	4,08E-29
WIPF1	-5,39381	8,45E-77
EHD2	-5,42174	2,80E-158
COL27A1	-5,42268	3,06E-41
DNM3	-5,4322	1,15E-12
ZNF883	-5,43657	0,000127
HEG1	-5,44047	0,000351
FHAD1	-5,44439	7,46E-12
CDC42EP3	-5,4455	5,86E-116
ST3GAL2	-5,45098	2,62E-58
STX11	-5,45381	2,96E-11
PLEKHG4B	-5,45772	3,02E-32
ARMCX4	-5,468	3,16E-06
MRC2	-5,4686	2,96E-42
TMEM52B	-5,47684	1,21E-06
SPX	-5,5081	0,000311
NOG	-5,52022	9,42E-12
LGI2	-5,53814	1,05E-13
UPP1	-5,53824	9,97E-23
SP100	-5,54397	5,66E-05
OAS2	-5,54673	7,82E-06
CD68	-5,54854	3,50E-31
TLR2	-5,55723	1,30E-07
EMP1	-5,5581	0,000727
MAGEB2	-5,57337	1,92E-11
NLRP3	-5,57464	0,000715
UGT2B4	-5,57581	0,00065
C10orf11	-5,5786	1,64E-21
MSRB3	-5,58027	1,84E-83
C8orf34	-5,58088	1,18E-07
PRR5L	-5,5855	9,52E-70
PTPN22	-5,58756	2,61E-18
PAG1	-5,59953	1,20E-73
C17orf51	-5,60827	1,18E-45
MARCH3	-5,61592	4,44E-17
AGPAT4	-5,62437	3,24E-51
TRPM8	-5,62841	0,000902
PTPRH	-5,63415	5,45E-19
C1QTNF2	-5,64047	9,27E-12
NPIPA5	-5,6627	4,69E-08
PLCE1	-5,66815	8,22E-22
PNMA2	-5,70033	6,00E-22

PRDM1	-5,70066	0,000405
GNG2	-5,71999	6,57E-10
SORCS2	-5,7223	3,24E-47
PLIN2	-5,7225	2,43E-95
EPB41L2	-5,72558	7,04E-137
SHC3	-5,72774	5,99E-26
OPTN	-5,72967	9,31E-30
EPHB1	-5,73356	7,61E-56
TRIM67	-5,73502	5,49E-11
CECR1	-5,74456	1,11E-06
MATN3	-5,74675	1,08E-46
TTLL6	-5,7508	0,000306
TMEM45A	-5,76479	3,99E-27
TRAF1	-5,76594	1,66E-51
SERPINE2	-5,8139	1,21E-94
HLA-A	-5,81819	3,41E-114
PLA2G16	-5,82185	1,96E-75
PIK3AP1	-5,83349	1,36E-26
KIFC3	-5,84135	1,30E-27
CASP10	-5,84448	6,85E-20
SLIT3	-5,85343	6,89E-07
SYN1	-5,85809	9,05E-19
HOXB8	-5,85851	3,70E-07
PAPSS2	-5,86209	1,87E-06
ZC3H12C	-5,87206	9,00E-40
CD109	-5,87409	2,73E-88
TRIM54	-5,88403	8,76E-06
ADAM8	-5,89371	4,34E-56
C2	-5,90495	4,38E-64
STAT5A	-5,90738	2,83E-13
C3orf80	-5,91035	1,24E-22
PTHLH	-5,93282	1,94E-82
HLA-DPA1	-5,9514	8,25E-10
POPDC3	-5,95141	3,25E-19
PQLC2L	-5,96873	3,91E-43
SLC2A3	-5,9708	6,42E-23
VIM-AS1	-5,97382	1,92E-74
LYN	-5,97898	1,82E-51
F3	-5,98449	1,61E-80
HOXA2	-5,9853	1,05E-12
OLFML2B	-5,9868	3,25E-21
RBMS3	-5,99062	2,41E-20
PMP22	-5,99615	8,71E-56
FBLN1	-6,00005	1,47E-09
ADAM19	-6,00092	1,02E-125
SH3PXD2A	-6,01954	4,55E-82
DYSF	-6,03617	4,29E-30
APLN	-6,03773	7,59E-20
SLC22A15	-6,04318	3,53E-42
GBP4	-6,04946	9,77E-05

MLLT11	-6,05195	6,00E-69
LCAT	-6,05211	1,08E-11
SH3RF3	-6,05816	6,53E-15
FHL1	-6,06665	7,48E-75
RSAD2	-6,06678	0,000666
LOC101928	-6,07185	2,15E-11
STEAP2	-6,07734	8,62E-11
GBP5	-6,07879	1,00E-09
KCNH1	-6,07939	3,75E-14
ZDHHC2	-6,0978	4,44E-37
PTPRB	-6,11654	3,06E-22
PYGL	-6,11993	1,69E-06
MFSD7	-6,12009	1,95E-06
IGFBP6	-6,12066	1,73E-41
SPRY4	-6,12531	3,40E-57
P2RX7	-6,12727	5,80E-11
ABLIM3	-6,13277	9,02E-65
BICC1	-6,14401	7,89E-56
ZNF415	-6,15082	4,19E-12
ZNF439	-6,1773	2,47E-05
MCAM	-6,1818	1,04E-58
EGFLAM	-6,20155	1,99E-39
MX2	-6,20391	6,82E-67
ZBTB20	-6,20965	9,26E-17
ADAMTS6	-6,21347	2,02E-05
RFTN1	-6,21381	8,07E-82
HAVCR2	-6,2343	3,92E-07
TNFAIP3	-6,24248	1,05E-78
PLAC8	-6,2592	7,13E-11
PRICKLE1	-6,26015	2,85E-39
DISP2	-6,26021	1,47E-05
SIRPB1	-6,26053	3,23E-10
DAB2	-6,26706	3,52E-21
CPA4	-6,27064	3,33E-07
CLDN1	-6,27983	1,60E-06
PIK3CD	-6,28941	1,21E-52
AEBP1	-6,29959	3,23E-90
C15orf52	-6,30119	7,84E-12
ARHGEF40	-6,30797	9,54E-43
XDH	-6,31145	2,70E-12
PLTP	-6,31586	2,43E-74
DAPK1	-6,31966	1,47E-21
CFH	-6,32111	9,07E-06
KIAA1462	-6,3275	3,14E-56
COL1A1	-6,33679	7,29E-183
GLIPR1	-6,33733	2,42E-48
ABHD8	-6,34709	1,02E-40
HCG4	-6,35492	2,11E-15
COL12A1	-6,35543	2,04E-41
GRASP	-6,36542	6,81E-08

ITGB8	-6,38821	8,13E-22
BDNF	-6,38939	8,11E-35
ZNF469	-6,3962	5,75E-44
NPB	-6,40113	5,63E-14
MEFV	-6,40123	1,35E-17
CAPN2	-6,4034	5,77E-52
NCF2	-6,41194	2,50E-05
EGFR	-6,42485	2,58E-141
SLC6A17	-6,42859	8,60E-06
LINC01605	-6,42865	4,46E-34
TRIM22	-6,4324	2,27E-26
FBXO32	-6,4377	7,65E-49
VEGFC	-6,4443	3,31E-48
SLCO2A1	-6,44609	2,90E-07
MAFF	-6,46658	6,92E-05
ZNF528	-6,49482	1,30E-21
SELM	-6,50593	7,75E-177
LOC100133	-6,53425	0,000723
HAPLN3	-6,53734	6,74E-14
CGNL1	-6,54739	3,79E-34
SDPR	-6,56507	4,31E-14
TRABD2A	-6,56901	3,86E-05
JAZF1	-6,56989	6,05E-42
ABI3	-6,59217	1,49E-06
COL6A1	-6,59629	6,18E-267
BATF2	-6,61076	1,00E-08
PLEKHA4	-6,62031	4,22E-48
CA2	-6,63891	3,11E-44
FHOD3	-6,65047	4,80E-34
LOXL4	-6,65369	2,63E-11
IL12A	-6,65553	3,67E-13
TNIP3	-6,66107	1,75E-06
JPH2	-6,67717	0,000437
SLC16A2	-6,67785	1,26E-71
SIRPA	-6,6889	1,69E-230
MMP13	-6,69342	3,69E-18
BANK1	-6,70332	0,000509
GLI2	-6,7039	3,51E-13
ZNF788	-6,7064	3,83E-09
IFI27	-6,71177	7,34E-10
LOC101927	-6,72252	0,000385
TRIM9	-6,72854	5,16E-29
TNF	-6,73672	3,04E-23
CCDC144A	-6,74747	0,000596
PLAUR	-6,75645	1,19E-49
HS3ST1	-6,75971	0,000324
TMEM200B	-6,77735	8,14E-17
ZNF365	-6,77996	9,74E-33
VILL	-6,78133	2,98E-09
TBX15	-6,78437	2,15E-27

PTGER1	-6,78842	0,000409
SLC15A3	-6,79461	6,96E-08
RASSF4	-6,7978	1,73E-48
HCP5	-6,80553	6,59E-21
BCL2A1	-6,81447	5,46E-07
HOXD1	-6,82638	4,51E-09
MCTP2	-6,83554	1,95E-22
GNB3	-6,84384	5,79E-18
GEM	-6,84727	1,75E-112
TRABD2B	-6,8494	6,13E-06
TSPAN2	-6,88041	5,28E-32
RAB32	-6,88325	1,37E-83
GPR146	-6,88644	0,000246
LINC00622	-6,88892	4,62E-07
PEG10	-6,89564	1,80E-09
SEC16B	-6,90322	3,36E-10
TCAF2	-6,91996	7,95E-17
FAM26F	-6,92621	1,44E-07
ROBO1	-6,9349	1,58E-77
DMGDH	-6,95521	0,000193
MIR614	-6,95631	0,00015
ETV5	-6,96321	2,03E-78
ACOT2	-6,96516	1,77E-30
ANTXR2	-6,96778	1,58E-29
PEAR1	-6,98174	0,000146
IL15	-7,00768	8,46E-11
SRSF12	-7,02436	3,62E-13
FHL2	-7,0526	2,28E-38
APOL3	-7,07555	1,03E-13
ZEB1	-7,07702	8,17E-50
ANGPTL4	-7,09851	2,34E-51
ILDR2	-7,11246	2,23E-23
MALL	-7,13991	3,69E-19
ANXA1	-7,17067	1,43E-218
FAM49A	-7,19008	1,25E-51
DKK3	-7,19605	6,95E-95
KIRREL3	-7,19874	7,34E-05
BST1	-7,20463	6,75E-12
PXDNL	-7,22108	5,71E-05
LOX	-7,23882	6,81E-180
LOC79160	-7,24939	1,23E-08
NLRC5	-7,25047	2,32E-63
CDKN1C	-7,25959	3,88E-12
STAT4	-7,2652	1,01E-08
SMO	-7,2704	3,63E-12
CRYM	-7,2705	4,75E-05
TYMP	-7,27586	1,85E-158
C1RL	-7,28959	1,86E-15
ZNF426	-7,29667	2,07E-18
DUSP10	-7,32242	1,39E-89

CABP4	-7,32583	4,69E-05
IGF2BP1	-7,33034	6,45E-71
INPP1	-7,33212	1,33E-07
SRPX	-7,33267	4,43E-89
RIN1	-7,33391	1,82E-110
CD6	-7,3509	0,001
SLC6A12	-7,3595	0,00092
MFNG	-7,3595	0,00092
CPA6	-7,36622	1,83E-08
FOXQ1	-7,36803	5,24E-18
IL2RG	-7,37691	0,000867
GPR85	-7,38416	2,74E-05
LINC00702	-7,38885	0,001008
LOC154761	-7,39304	3,84E-05
S100A3	-7,39406	3,14E-05
CLDN14	-7,39418	0,000862
BNC2	-7,39715	4,21E-19
NLRP1	-7,39798	2,69E-05
KCNQ3	-7,39971	0,000787
KIAA1755	-7,42215	0,000715
SYNPO	-7,42417	9,78E-71
ZNF717	-7,42661	2,78E-09
CNTN6	-7,42765	0,000707
CALCB	-7,42813	2,68E-09
BPIFC	-7,43304	0,000748
PILRA	-7,43849	2,75E-05
DUSP5P1	-7,44155	0,00067
PTN	-7,44425	0,000654
LOC101928	-7,44425	0,000654
SERPINE1	-7,4517	7,73E-07
MAF	-7,45309	8,87E-97
ITGA5	-7,45464	3,87E-257
RELN	-7,45539	1,73E-22
NEK9	-7,4576	8,40E-06
FAM26E	-7,45807	0,00067
SPON2	-7,45823	1,11E-90
UBQLNL	-7,46335	0,000612
AJAP1	-7,46587	0,00033
ACP5	-7,46901	3,96E-07
SLC16A12	-7,47135	0,000602
ITGA11	-7,47397	0,000618
HS1BP3-IT1	-7,47397	0,000618
PADI1	-7,47654	0,000646
SOGA3	-7,47842	4,11E-20
ZNF660	-7,47905	0,000684
ARHGAP22	-7,48096	1,20E-34
EVA1A	-7,48506	6,09E-71
THSD1	-7,48958	0,000124
PLSCR4	-7,48962	3,11E-05
MIR31HG	-7,49781	0,000601

ST3GAL6	-7,50371	1,89E-05
AFP	-7,50495	0,000758
CSF1	-7,52177	2,21E-140
TMEM255B	-7,52763	9,47E-33
FBXL21	-7,52943	0,00046
DPY19L2	-7,5296	1,39E-13
BIN1	-7,53605	1,34E-112
PLAG1	-7,53709	1,78E-12
INSC	-7,53941	0,000521
DGKG	-7,54873	1,02E-09
POSTN	-7,54882	0,000692
LINC01094	-7,55549	0,000499
INHBA-AS1	-7,56027	0,000431
LOXL2	-7,56212	7,18E-17
SPINK13	-7,56273	0,00041
ACSM5	-7,56307	0,000958
CTSS	-7,56487	0,00012
CP	-7,56723	2,77E-45
GPR132	-7,57097	1,31E-05
PHYHIP	-7,57332	0,000491
SPOCK1	-7,58112	4,69E-132
GNGT2	-7,5821	0,000491
AICDA	-7,58525	0,000363
PKLR	-7,59318	0,00044
COL5A1	-7,59382	2,43E-95
KRT222	-7,605	0,000332
TMEM173	-7,60541	3,03E-05
MYH13	-7,60743	0,00033
IGFBP7-AS1	-7,61057	0,000435
IL18R1	-7,61357	1,10E-38
LOC102477	-7,6146	0,000353
LINC01358	-7,61504	0,000364
SYTL3	-7,61782	1,95E-105
SPOCD1	-7,61906	2,22E-06
CARD16	-7,62132	0,000433
SYT11	-7,62261	1,76E-24
HRASLS	-7,62343	0,000476
LOC399715	-7,62779	0,000433
LINC01583	-7,63394	0,000329
FBXL7	-7,63394	0,000329
IGFBP3	-7,63682	2,15E-48
COL7A1	-7,63824	1,27E-116
LOC400940	-7,63917	0,000664
ACOT1	-7,65467	9,53E-06
IL36G	-7,65803	0,000269
MPP1	-7,66402	3,42E-70
ENG	-7,6656	4,73E-111
CDH16	-7,66963	0,000273
COL8A1	-7,67167	3,46E-10
TP73-AS1	-7,67259	0,000364

MESP2	-7,67453	0,000256
TFCP2	-7,68298	0,000358
PIK3R6	-7,68374	0,00024
NFE4	-7,68601	0,000246
ARPP21	-7,68825	0,000254
TLL1	-7,68825	0,000254
ITGAX	-7,6931	0,000234
ROR2	-7,71778	9,31E-38
UCA1	-7,72513	1,31E-18
ITGBL1	-7,73097	0,000275
SLC4A4	-7,7339	1,01E-21
CCL26	-7,73686	0,00025
CDH4	-7,73827	0,000436
ARL10	-7,74407	5,67E-06
MAEL	-7,74524	0,000186
FAM212A	-7,74548	0,000424
CA9	-7,74589	4,29E-18
C1QL1	-7,75434	2,10E-172
NOX4	-7,77003	0,000304
SLC26A7	-7,77306	0,000829
TTC22	-7,77374	0,000166
DPYSL3	-7,77501	4,97E-167
TM6SF2	-7,78187	0,000212
ADAMTS14	-7,78354	7,60E-26
LOC10106(	-7,7847	0,000149
LINC01138	-7,78854	1,27E-10
KRT75	-7,79144	0,000168
HOXB3	-7,79369	4,80E-15
LIPG	-7,79631	0,000826
ANO3	-7,79767	0,000143
LOC100131	-7,80093	0,00022
F2	-7,80272	0,000244
TRIM38	-7,81618	0,000186
MMP7	-7,81684	0,000129
PLB1	-7,82547	0,000139
TSHZ2	-7,82749	0,000131
PCDH20	-7,82954	0,000125
LAMC2	-7,83604	2,10E-172
CYS1	-7,83721	3,99E-06
CCIN	-7,84188	0,000127
COL6A2	-7,84663	1,69E-230
LINC01160	-7,87854	0,000127
ERAP2	-7,87878	3,02E-74
NR1H4	-7,88035	0,000138
LINC00857	-7,88189	6,91E-11
IGFBP1	-7,9031	9,10E-05
CD300LB	-7,90531	9,38E-05
ZNF69	-7,90877	0,000106
TNFAIP2	-7,91088	1,51E-69
ENPP2	-7,91188	9,22E-24

PSMB9	-7,91417	2,74E-106
XAF1	-7,91851	8,27E-07
PSORS1C3	-7,92114	8,58E-05
SFTA1P	-7,92273	9,35E-05
C1R	-7,92996	4,38E-142
CDH13	-7,93082	7,61E-05
ST6GAL2	-7,9322	2,17E-27
KCTD16	-7,94508	9,73E-05
NDUFA4L2	-7,95081	4,10E-128
ZNF521	-7,95733	5,86E-20
GRPR	-7,95896	0,000106
PAX8	-7,97394	8,69E-120
RGS4	-7,97417	0,000101
NTNG1	-7,98784	1,12E-20
ANKRD30B	-7,9884	5,71E-05
TAGLN3	-7,98902	7,96E-05
RFX8	-7,99575	6,24E-05
SLC17A9	-7,99667	1,56E-91
MME	-7,99893	2,49E-06
LOC101928	-8,0007	8,93E-05
ADAD2	-8,00184	1,87E-06
ZNF625-ZN	-8,01062	5,88E-05
HOXD11	-8,026	1,30E-39
PPP1R3G	-8,03438	1,57E-06
TMBIM1	-8,03608	2,06E-67
LOC101448	-8,03643	4,80E-05
PID1	-8,03788	8,74E-17
HLA-B	-8,04375	1,46E-73
LOC100289	-8,04377	4,36E-05
LOC102723	-8,04455	6,57E-05
BIRC3	-8,0554	4,51E-54
RNF217	-8,05782	7,40E-16
DNER	-8,06751	3,49E-16
SLC2A9	-8,0794	4,01E-05
ACSL5	-8,07959	1,11E-61
FOSL1	-8,08288	5,74E-99
ACTBL2	-8,08435	4,76E-05
SLC22A3	-8,08435	4,76E-05
APOBEC3C	-8,08582	2,35E-36
IL12RB1	-8,09004	3,56E-05
LINC00524	-8,0901	0,000127
BAIAP2L2	-8,09089	9,04E-27
HDAC9	-8,10938	1,69E-40
HOXB5	-8,12408	5,03E-05
HS3ST3A1	-8,14046	8,46E-55
ALDH2	-8,14741	4,77E-82
SAMD9L	-8,14743	0,00025
FBN1	-8,14796	5,69E-161
ABCC9	-8,15428	1,40E-10
GAPLINC	-8,15433	4,06E-05

KCNJ15	-8,15847	2,64E-05
ANKRD33B	-8,15963	2,72E-58
IFFO1	-8,1642	3,31E-17
PPP1R14C	-8,17142	1,14E-22
TMEM200(	-8,17492	2,73E-05
FAM71E1	-8,17682	2,25E-05
LINC01444	-8,17901	3,10E-05
DAW1	-8,18201	2,63E-05
C10orf90	-8,18381	2,08E-06
SCG2	-8,18652	2,46E-05
PTGS2	-8,20156	5,19E-22
HAS2	-8,22263	4,35E-24
LYNX1	-8,22396	1,85E-05
COL6A3	-8,22409	6,06E-41
FZD8	-8,23455	8,33E-94
CD177	-8,23667	1,72E-05
FAM27B	-8,23667	1,72E-05
RTL1	-8,24043	2,38E-05
RAB7B	-8,24551	7,51E-07
SLC7A7	-8,24555	1,15E-17
CH25H	-8,26794	1,43E-05
COL4A2-AS	-8,27217	3,45E-05
ARSJ	-8,27544	8,76E-18
CHRNA1	-8,28469	2,51E-05
C8orf34-AS	-8,28495	1,34E-05
VSIG1	-8,30316	1,19E-05
FAM150B	-8,31072	1,14E-05
NRP2	-8,31153	1,28E-122
PTGFR	-8,31614	4,13E-17
TM4SF19	-8,33818	1,62E-05
GALNT5	-8,34187	1,06E-05
LOC101928	-8,34602	1,24E-05
ZKSCAN7	-8,35092	1,05E-05
BCHE	-8,37421	1,58E-05
MARCO	-8,37537	1,75E-05
MMP10	-8,38073	1,08E-05
TGFB1I1	-8,38377	6,77E-09
TRIM55	-8,39356	2,84E-07
TRPC4	-8,39809	1,40E-05
APOL6	-8,40295	5,70E-106
DMBT1	-8,41542	8,12E-06
MAP1LC3A	-8,42077	6,59E-06
C2orf88	-8,42204	2,48E-07
GGT8P	-8,43263	9,65E-06
TENM2	-8,43743	0,000655
SNPH	-8,43907	1,84E-18
SCG5	-8,43995	2,53E-07
LOC115110	-8,45102	5,84E-06
SRGN	-8,45177	5,25E-07
DRGX	-8,45814	6,43E-06

USP32P1	-8,47445	4,98E-07
GRIA3	-8,50201	4,34E-06
MX1	-8,5024	2,88E-107
ZNF844	-8,51774	1,57E-07
TNFSF12	-8,52036	3,81E-06
C11orf45	-8,52165	3,86E-06
ICAM2	-8,52249	2,59E-07
ICAM1	-8,53645	9,91E-97
ZNF559	-8,53648	5,16E-06
LOC100507	-8,53847	3,42E-06
C22orf34	-8,53878	4,39E-06
SERP2	-8,54365	3,52E-06
PHLDB2	-8,54761	8,96E-41
C10orf67	-8,55887	3,07E-06
GABRE	-8,56073	4,80E-06
SOX2-OT	-8,56327	1,95E-07
OSR1	-8,57743	1,18E-07
GLIS3	-8,58328	2,24E-06
CD274	-8,58397	5,26E-26
UCHL1	-8,5876	1,68E-17
B3GALT1	-8,59884	2,50E-06
ENPP3	-8,60434	3,57E-06
LINC00899	-8,60815	3,46E-06
SGK2	-8,61127	4,39E-06
AMPH	-8,61149	1,46E-07
TLR6	-8,61409	3,31E-06
SRPX2	-8,61569	2,93E-06
GBP3	-8,61739	9,90E-08
CD69	-8,62823	2,37E-06
NPFFR2	-8,63057	2,18E-06
LINC01111	-8,64455	2,62E-06
ARHGAP25	-8,65811	3,42E-06
OSBPL3	-8,66993	1,74E-70
APOL1	-8,67175	1,20E-09
ADM	-8,68636	5,87E-107
SCN9A	-8,69062	3,65E-14
CTHRC1	-8,69691	2,06E-142
PSG5	-8,71415	1,90E-06
SIGLEC15	-8,72163	1,31E-06
NTRK3	-8,72736	1,38E-06
LOC101928	-8,75049	1,36E-06
LINC01436	-8,7598	1,27E-06
CLDN11	-8,76781	6,60E-08
ZBED2	-8,77936	1,02E-06
DNAH12	-8,78255	9,24E-07
BMPER	-8,78255	9,24E-07
KLRC1	-8,78447	1,12E-06
IL36B	-8,78578	8,98E-07
KRT34	-8,78833	1,44E-06
ZNF385D	-8,80114	1,05E-06

PINLYP	-8,80633	1,46E-06
FSTL1	-8,81057	4,09E-97
ABI3BP	-8,81114	1,07E-06
PTPRD-AS1	-8,8179	7,51E-07
LOC100506	-8,82398	1,00E-14
CCL20	-8,82739	7,17E-07
ITK	-8,83894	6,92E-07
ASB2	-8,84202	3,11E-21
C14orf105	-8,84916	7,42E-07
HOXA9	-8,8506	1,67E-06
OLAH	-8,87513	6,42E-07
DEPDC7	-8,89085	6,59E-07
GSTM1	-8,89231	4,44E-15
HECW1	-8,89618	4,88E-07
NUDT16P1	-8,90727	5,12E-07
FAM43B	-8,92693	4,12E-07
AQP9	-8,92774	4,85E-07
LINC01224	-8,93275	4,24E-07
ZNF625	-8,93379	4,00E-07
C15orf48	-8,93396	5,32E-08
GJC1	-8,9419	2,88E-27
FSD2	-8,95609	3,54E-07
ADAMTS2	-8,96023	2,84E-15
GPR1	-8,96944	3,39E-07
RRN3P1	-8,97135	6,04E-07
NEXN	-8,97437	4,89E-22
FAM27E3	-9,00562	4,01E-07
PPP1R3C	-9,01163	2,64E-15
CLIP4	-9,01169	5,09E-36
RTP4	-9,02221	3,20E-07
PAPPA2	-9,02501	1,34E-08
CDH2	-9,04026	1,31E-111
PRDM8	-9,05923	2,30E-07
GIPC2	-9,06311	2,75E-07
SPRR2D	-9,06923	2,80E-07
BRINP1	-9,07128	9,29E-16
DOCK2	-9,07582	1,23E-22
NEFM	-9,07682	1,88E-07
KHDRBS3	-9,07721	1,12E-07
HABP2	-9,08301	1,79E-07
CR1L	-9,08569	1,68E-07
LINC01411	-9,08925	1,75E-07
PHACTR1	-9,09051	8,04E-16
ECM2	-9,1032	1,59E-07
LOC541472	-9,11338	1,71E-07
KLHDC7A	-9,11679	5,49E-16
LZTS1	-9,1324	1,30E-07
LBH	-9,1356	5,51E-16
S1PR1	-9,16268	1,14E-07
LRRC15	-9,18874	1,31E-07

PSG1	-9,192	1,98E-07
IL7	-9,19322	9,26E-08
CARD6	-9,19489	9,33E-08
PRKCQ-AS1	-9,1998	9,43E-08
CLMP	-9,20236	2,74E-30
MYO7B	-9,2078	8,92E-08
FOXL1	-9,20873	1,40E-07
LOC72997(	-9,21273	8,65E-08
SKAP1	-9,22086	1,26E-07
ZNF71	-9,23207	1,95E-16
FGF2	-9,23322	9,41E-31
LUM	-9,23467	1,04E-15
ZMIZ1-AS1	-9,24294	6,96E-08
PDE10A	-9,26493	1,50E-08
MSR1	-9,27723	5,65E-08
ACTN2	-9,27738	6,55E-08
EPAS1	-9,28374	5,62E-181
SLFN13	-9,28715	5,68E-08
NID1	-9,29614	6,23E-08
SLAMF7	-9,30322	5,16E-08
GAL3ST1	-9,33549	4,75E-08
LINC01320	-9,33556	7,60E-08
IL34	-9,33753	4,06E-08
HLA-H	-9,35696	8,69E-17
C10orf10	-9,37166	5,78E-17
MUC12	-9,37258	3,39E-08
TIMP4	-9,39717	2,06E-09
CXCL3	-9,40901	6,71E-09
NACAD	-9,42116	3,19E-08
GBP1P1	-9,42278	2,61E-08
DKFZp434J	-9,42354	2,47E-08
IL1A	-9,43289	3,09E-08
ATP6V0D2	-9,43667	2,30E-08
ZNF501	-9,4442	2,31E-08
ADCY8	-9,44617	2,48E-08
SP140	-9,45109	2,10E-08
LINC00958	-9,46926	1,67E-32
LONRF3	-9,47076	1,89E-17
TNFSF14	-9,47481	1,89E-08
GCNT4	-9,47481	1,89E-08
ASB5	-9,48638	2,11E-08
LHFP	-9,48759	3,30E-61
HLA-G	-9,50453	2,26E-08
C3	-9,50736	1,11E-87
BATF3	-9,51196	1,32E-09
SERPINB4	-9,51271	1,72E-08
IL32	-9,51626	1,93E-16
ITPRIPL1	-9,5182	1,42E-09
GDF6	-9,51884	1,30E-09
HLA-DPB1	-9,53463	1,10E-17

ZC3HAV1L	-9,55093	1,56E-08
MT1G	-9,56075	1,15E-08
PNMAL1	-9,56105	1,50E-08
TSLP	-9,56835	1,07E-08
CATSPER1	-9,57899	1,01E-08
KY	-9,58091	1,10E-09
WFDC21P	-9,58325	5,58E-18
LINC00900	-9,58719	1,56E-08
UCN2	-9,58807	7,91E-10
CASP1	-9,58895	9,57E-09
APOBEC3D	-9,59517	9,26E-09
LOC283683	-9,60145	9,96E-09
ARHGAP6	-9,61118	1,32E-08
CXCL6	-9,61182	9,00E-09
SSC5D	-9,62852	1,32E-55
CNN1	-9,6312	7,86E-09
KCNIP1	-9,64255	7,00E-09
TMEM47	-9,65611	8,62E-18
GALNT9	-9,6715	9,13E-09
ZNF665	-9,67497	6,11E-09
CCBE1	-9,67681	9,62E-10
TNFRSF14	-9,68371	3,68E-18
ADGRE1	-9,69051	9,00E-09
CYP7B1	-9,69362	5,18E-09
TFPI2	-9,69891	2,56E-08
IL31RA	-9,70642	5,55E-09
IL6	-9,72794	4,91E-93
GATA2-AS1	-9,73578	3,98E-10
CYB5R2	-9,754	3,83E-09
TLR3	-9,75424	5,96E-09
TPM2	-9,81023	1,38E-197
C1RL-AS1	-9,82789	4,48E-09
MIOX	-9,83678	2,32E-09
GPC6	-9,8413	3,73E-27
LOC554223	-9,84343	2,49E-09
PAQR9	-9,85094	2,21E-09
LINC00520	-9,856	2,03E-09
LTK	-9,89294	2,70E-10
SUN3	-9,89818	1,57E-09
MDFIC	-9,90491	1,51E-27
ITGA1	-9,90915	1,50E-10
MIR155HG	-9,92744	1,33E-09
TKTL1	-9,92985	1,32E-09
ZNF215	-9,94842	1,22E-09
ZNF528-AS	-9,95847	1,53E-09
HOXD3	-9,96653	1,55E-09
SULT1C2	-9,97327	1,40E-09
HACD4	-9,97922	1,55E-09
ARHGAP28	-9,98121	1,07E-09
PTPRR	-9,98295	1,10E-09

HLX	-10,0079	1,06E-19
IL4I1	-10,0141	6,20E-07
SLC43A3	-10,0206	2,56E-05
SPAG16	-10,031	9,76E-10
PTX3	-10,0337	2,49E-08
CARD11	-10,038	1,40E-09
KCNE4	-10,0492	6,50E-10
PSG4	-10,057	9,67E-10
ZNF502	-10,0587	2,44E-09
C8orf31	-10,0729	7,60E-10
LIMS2	-10,0812	4,34E-38
CREB3L1	-10,0874	9,89E-126
TNFSF15	-10,0892	5,14E-10
C1QTNF1	-10,1053	1,78E-08
PRKCQ	-10,1062	4,65E-10
ZNF826P	-10,1151	5,13E-10
APCDD1L-A	-10,1203	3,20E-20
KCNS1	-10,1464	3,67E-10
EDNRA	-10,169	3,23E-10
LINC00460	-10,1702	3,22E-10
ZNF280B	-10,1715	6,65E-10
KMO	-10,1769	3,15E-10
CGB8	-10,177	3,52E-10
GLT8D2	-10,1847	3,67E-10
DOK6	-10,1955	3,33E-10
TGFBI	-10,2152	1,66E-99
VCAN	-10,2231	7,96E-154
TMEM220	-10,2282	2,36E-10
EBF1	-10,2351	2,80E-10
HOXD10	-10,2404	1,52E-20
GBP1	-10,2409	1,19E-16
BDKRB1	-10,2418	2,19E-10
HOXD4	-10,2606	1,90E-10
CD209	-10,2748	1,86E-10
ZNF470	-10,2838	1,71E-10
GRIN2A	-10,2885	1,75E-10
LINC01426	-10,2885	1,75E-10
ZNF280A	-10,2899	2,55E-10
ADAMTS5	-10,2999	1,67E-10
TNFRSF10D	-10,3013	1,48E-10
SYT13	-10,3019	7,63E-15
KANK4	-10,3133	1,67E-10
CA13	-10,3146	1,59E-10
ALOX5	-10,3227	1,79E-11
POU5F1	-10,3355	1,31E-10
ADAMTS16	-10,3635	1,06E-10
SLAMF8	-10,3637	1,11E-10
MEOX2	-10,3798	9,44E-11
SPNS3	-10,3875	1,03E-10
ARSE	-10,3911	8,96E-11

NTF3	-10,393	8,84E-11
HAS2-AS1	-10,394	1,29E-10
PDCD1LG2	-10,4014	8,31E-11
AGPS	-10,4021	4,45E-68
TLR4	-10,4043	8,21E-11
TDRP	-10,4044	1,57E-10
SEC14L6	-10,4099	7,92E-11
GDNF	-10,4103	7,86E-11
SDHAF3	-10,4314	9,56E-11
UBA7	-10,452	6,59E-11
TDRD9	-10,4738	8,62E-12
MT1L	-10,4751	6,42E-11
MMP14	-10,5038	1,90E-05
IL7R	-10,521	9,10E-11
ZNF334	-10,5317	3,82E-11
PPARGC1A	-10,5532	4,07E-11
SERPINB3	-10,5615	3,26E-11
DENND2A	-10,5678	3,60E-11
KCP	-10,5827	3,32E-11
IPW	-10,6055	3,19E-11
EMP3	-10,6067	4,77E-111
LRRC34	-10,6093	2,40E-11
DMD	-10,6214	2,24E-11
HOXD-AS2	-10,6372	2,39E-11
SLCO2B1	-10,6412	2,11E-11
GLIS1	-10,644	2,31E-11
TLE4	-10,6626	2,83E-12
RARRES1	-10,6767	2,15E-11
C1S	-10,6989	3,65E-87
APOBEC3G	-10,7152	1,65E-11
FGF5	-10,7213	1,63E-11
MSN	-10,7261	1,62E-10
NCAM1	-10,7301	4,74E-71
ZNF90	-10,7445	1,11E-11
GNE	-10,7755	9,68E-12
DPYD	-10,7774	8,87E-12
ROBO4	-10,7797	1,87E-11
NTM	-10,7891	9,45E-12
C3AR1	-10,8021	8,00E-12
ZFP82	-10,8237	7,44E-12
RAB33A	-10,8389	6,77E-12
CCNYL2	-10,8847	5,52E-12
CLEC2B	-10,8919	4,45E-12
C1orf186	-10,9004	4,25E-12
FABP3	-10,9012	4,23E-12
CSF2	-10,926	3,98E-12
LY96	-10,9265	6,12E-12
SERPINB7	-10,9401	5,35E-12
EBI3	-10,9431	3,61E-12
NGF	-10,9494	3,56E-12

HOXA10-AS	-10,9594	3,02E-12
HOXD13	-10,981	2,70E-12
P3H3	-10,9982	2,40E-12
KL	-11,0334	9,06E-24
MXRA5	-11,0383	7,11E-09
ADAMTS3	-11,0572	2,18E-12
PLA1A	-11,0711	1,54E-12
SLC35F3	-11,0757	1,57E-12
PTGIS	-11,0815	2,91E-12
TSPAN18	-11,0863	3,37E-13
MAMDC2	-11,1102	1,24E-12
CDH11	-11,1139	1,21E-12
ZFPM2-AS1	-11,1205	1,18E-12
FBLN7	-11,1312	1,77E-12
CCL28	-11,1365	1,04E-12
NPR2	-11,1387	2,16E-13
MYOM3	-11,1409	1,07E-12
AIM2	-11,161	9,28E-13
CXCL1	-11,1668	8,76E-13
NRN1	-11,2129	8,65E-13
COL4A2	-11,2133	5,60E-144
RAB42	-11,2237	7,09E-13
LINC00941	-11,2272	6,15E-13
ADAM12	-11,2464	7,59E-70
LINC00944	-11,2555	5,11E-13
TNFSF10	-11,2596	1,63E-13
CXCL2	-11,269	5,01E-13
C7orf31	-11,2722	5,31E-13
ANKRD1	-11,289	9,39E-14
PKIA	-11,2906	4,20E-13
TINAGL1	-11,2909	4,51E-13
ZNF518B	-11,3181	4,47E-13
BHLHE41	-11,3221	7,41E-14
CYBRD1	-11,3236	3,88E-13
ZNF626	-11,3271	4,07E-13
PAPPA	-11,3358	3,41E-13
MMP3	-11,3363	3,26E-13
RGS20	-11,3378	3,32E-13
ZNF175	-11,3405	3,59E-13
IL1RL2	-11,3545	2,90E-13
MT1A	-11,3556	4,58E-13
ARHGAP31	-11,3585	5,88E-14
LOC61326	-11,3618	2,89E-13
HRH1	-11,3675	3,02E-13
C2CD2	-11,386	2,35E-13
PDZK1IP1	-11,3986	2,40E-13
GSTP1	-11,4077	2,08E-169
GSPT2	-11,4082	2,08E-13
WT1	-11,4267	2,32E-13
AFAP1L1	-11,4294	3,17E-26

CPNE8	-11,4374	1,73E-13
TGM2	-11,4375	4,92E-173
COL11A1	-11,4379	2,07E-13
LINC00942	-11,489	2,83E-14
TOX	-11,5192	1,77E-13
PSMB8	-11,5212	8,31E-51
EPSTI1	-11,5383	8,90E-27
ZNF506	-11,5523	1,11E-13
MYHAS	-11,5796	8,90E-14
PLAT	-11,5847	7,05E-14
HAVCR1	-11,5903	7,02E-14
FRG1CP	-11,5959	6,59E-14
HKDC1	-11,6067	1,22E-39
FIGN	-11,6099	6,07E-14
ZNF300	-11,6234	6,28E-14
ZNF542P	-11,6235	5,84E-14
THBS2	-11,6306	5,39E-14
DPP4	-11,6348	5,25E-75
TCF4	-11,6415	9,15E-14
SAA1	-11,6434	6,79E-14
HOXD9	-11,6537	1,19E-13
LOC100126	-11,6614	4,51E-14
GCNT3	-11,6817	4,00E-14
UGT2B7	-11,684	8,06E-14
BAALC	-11,6905	3,77E-14
NEURL3	-11,7099	3,35E-14
LY6K	-11,7178	3,46E-14
HMGA2	-11,737	7,03E-15
KCNJ12	-11,7463	2,87E-14
CTSZ	-11,7917	8,56E-162
CDIP1	-11,8046	1,86E-14
CLEC4E	-11,8118	2,51E-14
GPX7	-11,8156	1,79E-14
TNC	-11,8294	3,83E-105
DGKI	-11,8809	1,20E-14
STK33	-11,8861	1,25E-14
SIGMAR1	-11,8976	1,27E-28
SPINK1	-11,9095	1,53E-14
CACNA1C	-11,9211	2,59E-15
GNG11	-11,9392	9,04E-29
C5orf42	-11,9482	8,09E-15
CNRIP1	-11,9495	7,78E-15
HOXA10	-11,9647	6,96E-15
FLG	-11,9655	7,89E-15
ZCCHC11	-11,9802	7,81E-15
HSPB2	-11,9985	5,80E-15
RAB38	-12,0208	5,27E-15
COL5A3	-12,0254	6,60E-15
ZNF773	-12,0318	5,32E-15
SERPINB2	-12,0419	8,12E-15

AFAP1-AS1	-12,0562	4,04E-15
CDHR1	-12,0832	4,80E-15
TRPA1	-12,1117	2,83E-15
AXL	-12,1402	1,85E-43
SPP1	-12,1505	1,75E-09
SEMA5A	-12,156	2,18E-15
CDK15	-12,1574	2,59E-15
ADRA1B	-12,1612	2,10E-15
CD40	-12,1758	1,99E-15
PVRL3	-12,1773	2,16E-15
LINC01322	-12,1952	1,86E-15
MARCH4	-12,1975	1,75E-15
KCNK3	-12,2106	4,47E-30
PDPN	-12,2136	1,85E-15
SMIM10	-12,2334	1,46E-15
SNHG18	-12,24	1,68E-15
MSC-AS1	-12,297	9,07E-16
C8orf88	-12,306	8,47E-16
PPAPDC1A	-12,3076	8,87E-16
LPAR1	-12,3167	7,95E-16
SLITRK2	-12,3389	6,89E-16
LINC00667	-12,348	6,56E-16
DZIP1	-12,3689	6,01E-16
ITGB3	-12,3767	1,68E-16
ZFHX4	-12,385	5,92E-16
ZNF320	-12,4327	4,14E-16
MAGI2-AS3	-12,4382	5,16E-16
PTPRD	-12,4712	3,81E-16
CDH6	-12,5229	8,53E-17
AGT	-12,5602	2,16E-16
TCEAL8	-12,5792	1,87E-16
RUNX1T1	-12,5902	1,54E-16
CHRDL1	-12,655	1,00E-16
C10orf128	-12,6674	8,93E-17
CST7	-12,7402	6,73E-17
MOXD1	-12,7699	5,13E-17
MT1M	-12,8442	2,94E-17
NRG1	-12,8681	2,50E-17
VCAM1	-12,8701	3,75E-17
SFRP2	-12,8766	2,44E-17
LINC00839	-12,8942	2,24E-17
CD70	-12,8984	1,52E-33
NID2	-12,9062	2,63E-17
IFI16	-12,9866	4,48E-34
COL4A1	-13,0038	6,25E-50
AKT3	-13,0789	6,91E-18
RRAD	-13,0799	6,57E-18
PRKCDBP	-13,0801	6,61E-18
ZEB2	-13,0848	6,33E-18
LAMA1	-13,1172	9,05E-18

VSTM4	-13,1245	4,91E-18
PAX6	-13,1605	4,09E-18
AK5	-13,1737	3,70E-18
IGF2BP3	-13,1822	3,40E-18
SLC35G2	-13,2084	2,87E-18
NRIP3	-13,2106	3,11E-18
EVC	-13,2346	2,43E-18
IGFBP7	-13,2746	2,41E-18
MMP2	-13,2778	2,56E-52
MT1E	-13,3191	1,45E-18
ADAMTS1	-13,3417	4,13E-19
ABCA1	-13,4328	8,21E-19
G0S2	-13,4424	6,32E-19
CHST2	-13,4509	6,01E-19
SALL1	-13,4516	6,04E-19
SGCE	-13,4661	5,46E-19
NT5E	-13,5257	3,70E-19
COL1A2	-13,5417	3,98E-19
PREX2	-13,5597	2,96E-19
STAC	-13,6085	2,22E-19
LAYN	-13,6136	2,06E-19
KIAA1644	-13,6265	1,91E-19
STK32B	-13,6277	1,91E-19
MMP1	-13,6351	1,80E-19
DFNA5	-13,7717	9,23E-20
BHMT2	-13,8131	5,89E-20
PLAU	-13,821	7,77E-20
APCDD1L	-13,831	5,24E-20
NIPAL4	-13,9025	3,04E-20
ATP10A	-13,9469	2,33E-20
AKR1B1	-13,9741	3,08E-109
EPB41L3	-13,975	1,88E-20
GJB6	-14,0054	1,60E-20
GPX1	-14,0139	1,59E-20
RAC2	-14,0753	9,69E-21
PTPRM	-14,079	1,05E-20
MSC	-14,1435	6,27E-21
CXCL5	-14,3169	2,47E-21
HNF1B	-14,3342	1,66E-21
EMC10	-14,343	2,02E-21
RARRES2	-14,5124	5,19E-22
ALPK2	-14,5369	4,10E-22
MXRA8	-14,5795	3,28E-22
CNN3	-14,5938	9,24E-23
NNMT	-14,6016	2,74E-22
SNAI2	-14,6086	2,50E-22
EFEMP2	-14,7732	9,09E-23
APBB1IP	-14,9877	2,23E-23
WBP5	-15,1818	4,36E-24
KIRREL	-15,1852	4,27E-24

ETS1	-15,1972	3,95E-24
GYPC	-15,2451	2,79E-24
ANGPTL2	-15,2539	2,83E-24
SPARC	-15,2721	2,30E-24
INHBA	-15,3525	2,01E-24
LRRN4	-15,3565	1,24E-24
NEFL	-15,4352	7,02E-25
GREM1	-15,4358	6,98E-25
CRYAB	-15,6792	1,16E-25
TNFRSF9	-15,8655	3,56E-26
IGFN1	-16,1128	4,70E-27
MYL9	-16,3182	9,98E-28
FLNC	-16,9895	6,22E-30
ANPEP	-17,3129	4,62E-31

SF539

Gene	log2FoldCh	FDR
CDH1	16,24549	3,32E-27
MYH14	15,35603	1,56E-23
FREM2	15,14729	6,40E-24
FBP1	14,94056	4,52E-23
TFF1	14,61192	4,69E-16
MEST	14,59194	2,96E-22
CLDN7	14,58601	3,29E-22
LDOC1	14,53042	1,19E-21
FOXA1	14,5052	5,34E-22
ST14	14,45569	3,13E-21
PROM2	14,43087	1,62E-13
TSPYL5	14,39724	1,15E-21
PTEN	14,25457	4,15E-21
TACSTD2	14,24217	5,33E-21
ABCA3	14,10189	5,63E-20
PRSS8	14,09338	8,85E-21
GRHL2	14,09299	2,21E-20
RAB25	14,05204	1,78E-20
FAM83H	14,04163	3,38E-21
EVPL	13,75986	1,30E-19
EPN3	13,74184	1,51E-19
TJP3	13,55396	5,14E-18
SBK1	13,48524	4,96E-19
FLRT3	13,26207	2,57E-18
TMEM30B	13,26057	3,03E-18
ZNF467	13,24221	2,97E-18
CLDN3	13,14161	1,16E-18
DSCAM-AS	13,11244	2,10E-10
RHOD	13,08213	3,59E-17
CAMSAP3	13,0443	8,95E-18
TC2N	13,01729	3,38E-17
VPS9D1-AS	12,89786	6,72E-18
RTN4RL1	12,88886	2,46E-17
SLC27A2	12,87346	3,07E-17
BSPRY	12,84256	4,64E-17
LRRC61	12,74533	1,47E-16
GGT6	12,73926	1,09E-15
WDR72	12,71727	7,32E-17
CDH3	12,6764	9,27E-48
DLX3	12,55459	6,46E-16
PRR15L	12,55326	5,68E-16
KIAA1324L	12,52434	2,50E-16
MGMT	12,50213	5,40E-16
LONRF2	12,47675	2,04E-15
IRX2	12,4731	3,56E-16
MAL2	12,42992	3,90E-31
AK7	12,38216	6,38E-16
ALDH3B2	12,37771	6,87E-15

KCNC3	12,33815	1,38E-15
SYK	12,24432	1,52E-15
TSTD1	12,22836	1,43E-15
KIF26A	12,14861	1,05E-14
KREMEN2	12,10932	8,30E-16
KRTAP5-AS	12,09343	1,46E-05
PCDHA11	12,07078	7,17E-15
GALNT6	12,03779	3,04E-15
CBFA2T3	12,03195	4,87E-15
RHPN1	12,0286	6,39E-15
RBBP8NL	12,00634	9,02E-15
CDC42BPG	12,00387	1,34E-14
SHANK2	11,98659	6,24E-15
OPLAH	11,94905	9,26E-15
CYP2S1	11,92965	9,65E-14
ARHGEF5	11,86049	2,77E-14
DNAJA4	11,83448	2,83E-14
SULT2B1	11,82982	3,01E-14
PCSK1N	11,81584	1,86E-14
OLFM1	11,8071	1,85E-14
SCN4A	11,80472	2,86E-05
GLB1L2	11,79534	2,04E-14
ATAD1	11,77741	3,71E-15
SULT1A1	11,73659	3,38E-07
HENMT1	11,7178	3,25E-14
HOOK1	11,69374	3,64E-14
FAM213A	11,62268	1,28E-36
BCAS1	11,62091	0,000106
NCAM2	11,60922	2,09E-13
ESPN	11,60716	8,04E-13
ACTA2	11,58733	0,000115
ALOX15	11,58112	2,39E-12
TMEM45B	11,56227	8,02E-14
KDF1	11,56133	9,79E-14
RASGEF1A	11,51015	1,68E-13
GALNT14	11,50929	1,10E-13
ATP6V0E2	11,48138	1,12E-35
SMIM22	11,46826	2,15E-13
EPHA1	11,43819	2,54E-13
ALG1L	11,42674	1,34E-12
MINPP1	11,42559	2,06E-13
MNX1	11,41998	2,03E-13
EPCAM	11,36311	7,94E-97
ASCL2	11,34691	1,26E-12
C5orf38	11,33847	5,62E-13
PCP4	11,33512	3,90E-13
FGD3	11,30596	2,94E-12
MNX1-AS1	11,30108	1,06E-12
OVOL1	11,2161	2,06E-12
PACSIN1	11,2046	3,87E-12

SYT17	11,20404	6,80E-13
CRB3	11,20301	2,04E-12
ENTPD2	11,20015	2,14E-12
ARHGDIG	11,18929	7,66E-13
BMP7	11,1617	1,28E-98
TMPRSS13	11,14819	1,74E-12
TDRD1	11,14106	9,97E-13
ILDR1	11,13534	1,05E-12
RDH16	11,09279	1,80E-12
PKP2	11,06412	9,06E-13
RANBP17	11,06342	2,72E-12
DPYSL5	11,02111	3,35E-12
CACNA1D	11,02053	2,02E-12
KCNK15	11,00214	2,73E-12
SUSD3	10,99938	4,34E-11
FXYD3	10,98706	9,05E-12
CST1	10,9382	4,11E-12
C5AR2	10,93437	1,79E-11
MB	10,92883	3,48E-12
ELF3	10,90146	8,93E-12
RGMA	10,88557	8,01E-12
ESRP1	10,87263	7,92E-79
KNDC1	10,84753	8,00E-11
SPDEF	10,84685	1,28E-10
LRP2	10,81226	3,10E-06
MAPT	10,80644	1,45E-12
PLEKHG6	10,80455	3,67E-11
HPN	10,79524	1,28E-11
POF1B	10,78759	5,43E-12
ARHGAP44	10,7714	9,38E-12
RET	10,76546	9,36E-11
EPPK1	10,74729	7,05E-20
PLXNA4	10,74637	2,05E-11
DSP	10,72046	3,70E-23
BCL11B	10,71768	5,11E-23
SPESP1	10,70694	2,31E-11
KCNH2	10,69945	1,35E-11
CCDC64B	10,68501	2,65E-11
RAB17	10,66524	3,08E-11
KRT19	10,65595	0,000397
ERBB3	10,6468	3,99E-53
TMEM125	10,64219	1,91E-11
FUOM	10,58554	3,09E-11
PDE3B	10,56627	3,63E-11
ODAM	10,56	3,08E-11
CEACAM21	10,55097	3,60E-11
ARHGEF35	10,5462	5,20E-11
CLDN4	10,5436	4,39E-12
KRT8	10,53244	9,07E-214
TDRD5	10,52688	5,81E-11

TSPEAR	10,51904	1,01E-10
MYRIP	10,51759	9,54E-11
FAM83B	10,50541	4,95E-12
TMC5	10,4947	6,08E-11
SH2D3A	10,47394	1,05E-10
MPZL2	10,43594	1,47E-10
PCDHA10	10,43252	8,62E-11
LXN	10,42774	3,84E-09
PLEKHB1	10,41082	2,03E-10
KCNJ11	10,40865	2,24E-10
DEF6	10,39038	8,19E-11
ABCG1	10,38103	1,01E-11
CASC9	10,35849	9,97E-11
LAD1	10,35666	6,90E-09
WSCD1	10,32848	1,57E-10
EPHX2	10,29792	2,60E-10
PCDHA12	10,29065	2,38E-10
CHRM1	10,28237	6,90E-10
HPDL	10,27349	1,74E-10
HLA-DQB1	10,26236	9,71E-09
ANXA9	10,25541	6,21E-11
FAS	10,25158	1,35E-08
C21orf91	10,24855	1,88E-10
CNTNAP2	10,24791	2,70E-10
CXCR4	10,22588	2,44E-10
NSUN7	10,20824	2,50E-10
ENPP4	10,19344	5,19E-10
KRTAP3-1	10,18923	1,40E-09
PRR15	10,18636	4,12E-10
AIF1L	10,1858	1,01E-06
RNLS	10,18416	2,90E-10
TFF3	10,16653	3,98E-10
DLGAP3	10,16469	3,06E-10
C18orf63	10,15903	4,52E-10
TNK1	10,15043	3,18E-11
RERG	10,15012	3,39E-10
ADAMTS19	10,11059	3,20E-20
EN2	10,10013	4,45E-10
IL17RB	10,08906	7,94E-10
CEACAM1	10,08416	5,03E-10
LINC00925	10,08174	5,54E-10
OVOL2	10,07423	9,01E-09
ERBB4	10,07289	8,35E-10
MLXIPL	10,06914	8,83E-10
KRT17	10,05944	6,41E-09
PCSK6	10,04867	2,13E-11
S100A14	10,04019	1,65E-05
PRR36	10,03609	1,45E-86
ZNF732	10,00847	1,56E-09
BATF	9,991254	2,08E-09

SLAIN1	9,986032	1,44E-09
ASCL1	9,972194	2,10E-09
GLS2	9,944475	1,10E-09
LOC11323(	9,938825	1,12E-10
C12orf56	9,925934	1,37E-09
PADI2	9,913465	2,39E-08
EPHA6	9,912908	1,38E-09
FAR2P1	9,903022	1,16E-08
SLC9A4	9,892358	1,16E-08
DNAH14	9,891693	4,71E-09
DEGS2	9,883147	2,05E-09
LOC72861:	9,879187	1,68E-09
UPK2	9,87641	3,95E-08
KIF1A	9,868218	4,17E-19
PRIMA1	9,85349	9,16E-09
P2RY6	9,841918	3,42E-05
COBL	9,838785	2,72E-10
OR7E14P	9,830027	2,49E-09
SYT3	9,824775	9,88E-09
ANO9	9,822885	2,72E-09
KCNQ1OT1	9,809243	1,18E-08
PNPLA4	9,797638	2,59E-09
CHST8	9,792985	2,66E-09
S100P	9,759217	5,58E-07
NOVA1	9,753331	5,68E-09
SPINT1	9,746166	4,97E-80
AKR1E2	9,728527	1,73E-08
RNF125	9,720576	2,93E-08
TBC1D30	9,715823	6,02E-43
ESRP2	9,71092	2,16E-49
KLLN	9,706916	3,52E-08
NUP210	9,695019	9,16E-21
MAPK4	9,663409	1,05E-07
TUBA3E	9,658495	1,08E-08
EPHA10	9,640385	6,35E-09
SPATA18	9,63693	1,46E-07
ZG16B	9,636582	6,88E-08
LINC00960	9,634994	1,19E-08
ECHDC3	9,616254	7,32E-09
C10orf82	9,614468	1,24E-08
MUC5B	9,607157	8,08E-09
ZNF492	9,598681	4,41E-08
CBLC	9,59315	1,08E-08
SPINK5	9,589268	4,73E-08
ENPEP	9,588043	8,94E-09
DOCK8	9,579589	9,66E-09
HID1	9,561197	1,48E-56
YBX2	9,554859	3,20E-40
CHRM3	9,55406	1,07E-08
SLITRK4	9,546149	1,53E-08

PLEK2	9,540143	1,13E-08
IRX4	9,516748	2,70E-08
FAM19A5	9,499786	2,50E-08
PRSS22	9,493024	3,50E-08
SPINT2	9,476063	8,70E-156
PLBD1	9,45903	3,21E-08
POU3F3	9,442036	5,03E-08
FUT9	9,441243	1,98E-08
LRRC37A4F	9,435716	2,87E-08
GAL	9,433057	5,93E-33
AP4B1-AS1	9,418947	1,47E-09
TUBA3D	9,415037	4,73E-08
PAX9	9,389909	2,71E-08
KCNK5	9,369404	1,43E-07
C2orf54	9,368902	3,83E-07
PLA2G4F	9,367317	1,81E-06
ALDH3A1	9,365262	1,27E-06
LOC100506	9,354236	7,56E-08
KLRG2	9,335728	4,82E-08
ACTL8	9,325149	8,26E-08
GCHFR	9,324896	4,52E-08
ARHGEF34I	9,316949	5,33E-08
SFN	9,310413	4,99E-57
CCDC78	9,306331	5,42E-08
WISP2	9,29414	3,84E-06
ANKRD18A	9,288214	4,94E-08
FCGR1A	9,266354	3,82E-07
RASAL1	9,261119	2,15E-07
COL9A3	9,259139	6,93E-08
EMID1	9,244538	6,84E-08
SLC22A31	9,243672	7,13E-08
LOC100505	9,239323	9,38E-08
PVRL4	9,236668	1,42E-16
RGS6	9,222958	1,27E-07
COL4A5	9,188706	1,97E-07
BGN	9,186107	0,000108
CNGB3	9,175837	1,28E-07
B3GALT4	9,17425	6,80E-07
CACNG4	9,170101	6,21E-23
DNAAF3	9,167051	2,76E-16
FAM201A	9,163549	9,59E-08
SFMBT2	9,157277	1,74E-07
RIC3	9,15066	1,84E-07
NDRG4	9,14276	2,04E-08
SHH	9,139951	1,85E-07
LINC00669	9,127202	1,80E-07
FSTL4	9,115844	2,48E-07
BLNK	9,107003	4,89E-06
LINC00992	9,099839	1,57E-07
ZNF876P	9,095636	1,42E-07

NRG3	9,087924	2,62E-07
SHANK1	9,074903	5,33E-07
PTCHD2	9,067349	9,12E-07
DTX4	9,066259	9,99E-09
C6orf132	9,064545	6,72E-23
RAB36	9,058453	1,06E-08
GNG13	9,052773	5,17E-07
JAK3	9,050269	1,84E-07
TNFRSF11A	9,046808	2,05E-07
RSPH1	9,045658	5,29E-07
C1orf210	9,041124	3,67E-07
CHGA	9,038466	9,76E-09
FOXP2	9,025431	3,52E-07
LINC00858	9,024098	5,64E-07
GSG1L	9,020048	2,31E-07
KLHDC9	9,011353	2,57E-07
IGSF5	9,006076	2,86E-07
FAM110C	9,003721	1,44E-06
CRABP1	8,996291	8,41E-07
SOWAHB	8,967281	3,50E-07
MAOB	8,956808	1,31E-06
GRIK3	8,956733	1,58E-06
ZNF695	8,946648	3,71E-07
RPRM	8,946495	1,04E-06
LOC100996	8,94231	4,40E-07
LRRC37A8P	8,941442	3,34E-07
S100A9	8,936744	7,94E-05
FAM83H-A	8,934869	2,23E-06
ABCA12	8,934548	2,99E-08
AGR2	8,932756	7,79E-06
HTR2C	8,928403	3,66E-07
LHX1	8,925827	4,45E-07
KIAA1257	8,909756	6,85E-07
LOC399815	8,909589	4,54E-07
MATK	8,909455	4,68E-57
ICA1	8,905167	1,10E-05
CNNM1	8,894167	4,28E-07
AEBP1	8,893221	7,27E-07
RADIL	8,892404	1,79E-06
PCDHA5	8,890315	4,55E-07
SVIP	8,887158	5,20E-15
CALCR	8,884156	6,98E-07
LOC100288	8,879691	5,63E-07
KCNMB2-A	8,878243	4,67E-07
LRRC26	8,874724	2,22E-08
KCNV1	8,87141	1,77E-06
AARD	8,859064	2,44E-08
HUNK	8,844953	1,61E-21
SERPINB5	8,839418	1,40E-05
LCN2	8,835751	3,23E-06

PRODH	8,83185	7,43E-05
MCF2L	8,831685	6,02E-07
TMPRSS4	8,830699	1,04E-06
CDC42EP5	8,827426	6,38E-07
DMC1	8,825102	4,69E-06
MMP11	8,823969	6,28E-07
MPPED2	8,814946	2,67E-06
INPP5D	8,813986	5,84E-05
TPTE	8,808062	7,99E-07
EDAR	8,807918	2,64E-06
LOC644919	8,794305	1,15E-06
PKP1	8,792592	3,89E-12
PLD6	8,790537	1,54E-18
DSCAM	8,787981	2,15E-06
ADAMTS19	8,787722	8,07E-07
PPL	8,758003	6,47E-11
ARHGEF37	8,75682	5,03E-08
SLC25A21-/	8,746969	1,39E-05
KRT23	8,734418	1,03E-06
LINC00664	8,731125	2,32E-06
IGSF9B	8,725468	3,88E-06
FRMPD1	8,724555	1,28E-06
CCDC83	8,722003	1,84E-06
SIGIRR	8,71851	1,71E-07
KCNN2	8,714131	1,15E-06
EFS	8,710633	2,89E-14
FAM109B	8,707853	5,20E-08
FAAH2	8,683932	1,89E-06
TMEM139	8,663365	3,15E-06
CARD14	8,63387	3,17E-06
LOC728739	8,628402	2,05E-06
DACH1	8,628135	1,82E-06
FCHO1	8,62472	5,49E-48
MYRFL	8,610504	3,64E-05
FAM81B	8,602409	3,02E-06
ATP2C2	8,599968	0,000146
GREB1L	8,568589	1,03E-06
TM7SF2	8,56803	8,93E-52
MYO7A	8,552665	2,71E-06
SMIM1	8,547134	9,55E-06
LINC01006	8,535568	3,18E-06
NPDC1	8,531769	9,86E-118
PRRT4	8,522529	3,78E-06
TMEM132E	8,494611	0,000215
FAM155B	8,480267	1,87E-13
EDA	8,470083	4,16E-06
TNFSF13	8,450621	4,24E-07
HLA-DRB5	8,446628	8,11E-06
PAX2	8,443431	0,00015
SLC22A11	8,438736	2,35E-05

PVALB	8,434103	1,60E-05
RASL11B	8,433983	2,88E-13
CES3	8,43121	6,76E-06
TCHHL1	8,425585	0,000332
NMUR2	8,423593	5,52E-06
FAM95C	8,414881	6,01E-06
TMC4	8,412787	1,29E-05
GUCY1B2	8,403746	2,06E-05
RUNDC3A	8,400241	1,67E-05
ADGRB1	8,399198	9,19E-25
PIP5K1B	8,38748	8,03E-06
IGFL1	8,368845	0,00032
GATA5	8,368775	7,94E-06
IGSF9	8,36805	4,58E-97
CCDC64	8,366617	5,07E-13
ATP2A3	8,36593	2,76E-05
SERPINA3	8,354908	1,92E-05
CRACR2A	8,354833	5,05E-05
TESC	8,353503	9,39E-06
NODAL	8,350848	1,48E-05
PPP2R2C	8,343402	1,10E-74
WIF1	8,342002	5,88E-05
LOC102723	8,335792	9,33E-06
SLC6A14	8,327831	9,64E-06
MIR4697H(	8,297854	2,56E-05
CRACR2B	8,2887	4,53E-18
TRPM2-AS	8,287204	1,08E-05
GPR37L1	8,27392	1,45E-05
HIST1H2BH	8,270525	1,31E-05
FOXA2	8,262029	1,22E-05
AKR1C2	8,261782	4,76E-10
PTK6	8,259555	4,96E-07
SORL1	8,256916	1,98E-12
HS6ST3	8,253478	1,49E-05
MAPK15	8,239561	3,35E-05
TARP	8,234812	7,05E-05
NELL2	8,234098	2,29E-05
F7	8,232471	6,15E-05
PAH	8,227019	1,91E-05
LIPA	8,225091	5,91E-05
UNC5A	8,223509	1,71E-05
SLC52A3	8,220589	2,63E-05
KLHL35	8,210848	1,80E-05
C2CD4D	8,205005	1,92E-05
LOC728673	8,180486	0,00011
LOC100996	8,164347	2,66E-05
LRG1	8,161452	0,000121
FLT3	8,149887	2,16E-05
TTC39A	8,147831	5,43E-05
CKMT1A	8,1474	1,08E-22

TRPV6	8,146199	2,32E-05
TEX19	8,146145	8,57E-07
RENBP	8,143655	3,54E-05
WFDC2	8,134983	4,75E-05
FOXI3	8,109154	2,98E-05
VAMP8	8,099225	3,43E-34
FA2H	8,096887	1,09E-09
POU6F2	8,091036	3,27E-05
COLCA1	8,084504	3,58E-05
IFIT3	8,082923	9,03E-05
RAPGEF5	8,062148	4,03E-05
MSI1	8,050544	2,06E-55
IGSF11	8,045618	3,56E-05
TTYH1	8,037787	0,000168
NOXA1	8,032895	0,000109
APOA1	8,028889	4,58E-05
MARVELD3	8,027258	2,86E-30
SLC52A1	8,006725	0,000156
MGC32805	8,005685	0,000145
TENM4	7,998212	2,59E-06
STK31	7,992194	6,83E-05
GATA3-AS1	7,985983	5,56E-05
CD8A	7,977994	1,61E-06
TFR2	7,971348	2,03E-06
TH	7,966514	9,09E-05
RDM1	7,948966	7,13E-05
XG	7,946875	0,000756
ADAMTSL3	7,945766	8,20E-05
ADGRE2	7,941957	2,28E-06
TMEM105	7,93714	0,000106
HTR6	7,935358	6,19E-05
PMEL	7,930398	1,42E-05
LOC440461	7,927139	0,000147
ARL11	7,925278	7,06E-05
BARX2	7,912836	0,0003
EMCN	7,911873	7,86E-05
LINC00885	7,908124	0,000373
EWSAT1	7,895836	0,000134
IGSF1	7,892342	0,000389
LRRC10B	7,878794	0,000161
FAM3B	7,875932	5,09E-16
ATP8A1	7,870906	0,000114
KRTAP4-1	7,860407	0,00015
GPM6B	7,854019	8,99E-05
SIDT1	7,832202	6,78E-11
CCT6B	7,830771	3,60E-06
SEPP1	7,825066	0,000387
EPHB6	7,824542	0,000103
CYP4F22	7,82295	0,00014
UCP2	7,81979	2,80E-109

C19orf33	7,815112	7,62E-19
CALML5	7,813271	0,000195
GJB5	7,813129	0,000581
CCSER1	7,810611	0,000138
C17orf104	7,805871	1,16E-05
RNF223	7,796929	1,24E-10
SGCG	7,796395	0,000293
TLX1NB	7,789734	0,000121
GJD3	7,778159	0,000206
HBA1	7,777769	7,44E-06
TMC6	7,761111	2,64E-31
TTC6	7,749204	0,000161
KLB	7,749204	0,000161
LOC101929	7,744315	0,000189
ARHGEF38	7,743087	0,000198
KIF12	7,735278	0,000162
PRRG2	7,72974	5,54E-10
CAPN9	7,727874	0,0002
DEFB126	7,721212	0,000167
FAM84B	7,719291	5,00E-60
VANGL2	7,714496	0,000182
LMX1B	7,711987	7,61E-15
SMTNL2	7,700231	0,000208
LY6G5C	7,695686	0,000216
CELF3	7,673878	0,000318
PNMA6A	7,673535	0,000275
C2orf15	7,672674	3,14E-22
PDGFB	7,672541	1,44E-18
FCGBP	7,66317	0,000477
ACTR3C	7,640533	7,31E-06
SYTL1	7,639905	7,84E-22
SMPDL3B	7,634803	4,66E-42
GOLT1A	7,63441	3,46E-10
NEBL	7,634259	1,06E-37
COL26A1	7,630878	0,000708
TTC9	7,627016	9,61E-46
SLC5A5	7,626837	0,000568
CEACAM5	7,609003	0,000641
KCNJ4	7,608023	0,0003
PLEKHA6	7,601151	1,01E-36
NUP62CL	7,600453	1,13E-05
LINC00494	7,597256	0,000287
LRRIQ1	7,593199	0,000301
GADD45G	7,576983	1,41E-11
PGM5	7,574964	1,42E-05
CNTNAP3	7,574928	0,000324
IRF6	7,572653	3,26E-20
LLGL2	7,572583	2,73E-99
SMIM10L2A	7,569862	0,000715
KRTCAP3	7,569617	2,93E-05

CA4	7,565268	0,000413
ZMAT1	7,560997	0,000134
SAMD12-A	7,555879	0,000425
FAM69B	7,550317	6,51E-10
LINC00959	7,537105	1,21E-05
FAM83F	7,531069	0,000854
LINC01132	7,530793	4,18E-05
MIPOL1	7,527291	1,69E-17
RGS16	7,523659	1,78E-15
ADIRF	7,515006	4,75E-22
RNF208	7,512477	4,34E-25
BRINP2	7,507628	0,000793
PARD6A	7,494912	2,53E-09
NMNAT3	7,484871	0,000616
SRCIN1	7,477111	1,48E-05
KLF8	7,469626	0,000571
MIR4664	7,463123	0,00051
MARC1	7,449949	6,27E-37
MMP25	7,445639	0,001008
GJB2	7,43331	4,55E-15
GALNT12	7,428349	2,06E-25
PLA2G3	7,418329	8,10E-05
SLC29A2	7,393181	2,35E-72
IGFBP2	7,385031	4,01E-95
LRFN5	7,379804	0,000724
FBXO15	7,375015	2,41E-05
ARHGEF4	7,370379	2,44E-05
SSPO	7,359208	0,001006
PACRG	7,347401	0,000852
SOX11	7,343542	0,00093
MYO1D	7,342558	1,96E-48
PDIA2	7,330594	0,000874
SERPINB9	7,318514	0,000985
CHDH	7,306898	9,05E-14
C19orf81	7,302843	3,24E-05
SYT7	7,283569	6,65E-121
PRSS16	7,274596	5,68E-19
CTSH	7,271008	3,53E-30
PTPN6	7,236856	9,64E-30
MTMR7	7,234617	4,15E-05
MFAP3L	7,215599	6,11E-17
ESR1	7,213263	2,91E-90
HES2	7,188075	8,56E-11
ASCL4	7,183905	0,000159
GRTP1	7,163469	6,81E-22
TMEM178E	7,144543	6,96E-16
AREG	7,140333	2,70E-22
AP1G2	7,130318	5,95E-46
STXBP2	7,130315	1,78E-50
PCDH19	7,127594	0,000118

NUP210L	7,124306	3,49E-21
TGFA	7,119404	2,55E-10
SGPP2	7,095343	9,81E-05
RND2	7,087736	2,23E-14
MACROD2	7,080658	0,000122
APCDD1	7,073009	2,28E-150
MYH10	7,061379	2,90E-95
SLC9A2	7,053841	8,16E-07
SHC2	7,025935	0,000198
TEX40	7,016979	0,000103
XKRX	6,995134	0,00019
BMP8B	6,992383	0,000116
JUP	6,980856	6,45E-77
CYFIP2	6,980282	9,45E-26
ASCL5	6,979145	0,000116
TAPT1-AS1	6,977603	0,000118
SLC24A3	6,971819	6,58E-11
MAP7	6,943895	3,30E-58
RUNDC3A-/	6,925675	1,01E-08
DENND2D	6,920858	2,69E-31
SNAR-E	6,91235	0,000281
PEX11G	6,910168	0,00024
CAMK2B	6,905645	1,37E-10
NPY1R	6,890461	1,21E-26
RIMKLA	6,888163	4,86E-13
PRPH	6,880054	1,52E-07
ITGB4	6,877874	1,07E-54
SLC25A21	6,866657	0,000197
PKIB	6,861312	9,83E-66
ZNF730	6,858894	2,46E-07
P2RY2	6,847294	1,29E-53
ZNF98	6,844458	0,000337
PLEKHA7	6,838324	5,28E-38
SLC26A4-A	6,837449	3,14E-10
MYO15B	6,824341	0,000235
LINC00649	6,815252	0,000222
AMIGO1	6,813703	2,30E-07
MUC1	6,81276	2,85E-31
GYLTL1B	6,812605	1,71E-41
GRB7	6,800173	0,000349
HCN4	6,787267	2,04E-12
NCKAP5	6,77418	9,00E-10
CPT1C	6,769598	1,74E-06
LOC28333	6,766516	0,000526
LAMA5	6,747195	7,49E-119
PCSK9	6,741894	5,53E-08
CERS4	6,735718	2,18E-15
NEB	6,712771	2,67E-32
KRT18	6,69601	1,25E-75
N4BP3	6,689326	2,70E-38

ERVMER34	6,688441	1,35E-11
MAML3	6,659202	2,80E-09
BCAS3	6,656402	2,69E-111
GRID1	6,63447	0,000518
CYP1A1	6,610682	2,87E-08
MARVELD2	6,607205	1,03E-46
ZFP2	6,59261	0,000837
PTP4A3	6,591209	1,04E-33
SPTSSB	6,540157	2,72E-25
LYPD3	6,524072	3,44E-25
PCDHB3	6,490967	3,17E-35
ADGRV1	6,486729	2,49E-09
ELF5	6,47932	5,44E-08
MYO5C	6,471855	9,63E-111
RHOV	6,460515	1,85E-24
PDE9A	6,450285	4,00E-06
POTEF	6,442483	0,000929
ARHGEF16	6,431107	2,55E-38
PARD6B	6,421064	8,01E-90
LOC14870S	6,399633	3,43E-08
DDIT4L	6,394367	8,99E-15
NUTM2A-A	6,386043	1,92E-13
FLT4	6,379815	2,98E-05
TPD52L1	6,362162	2,25E-64
MALRD1	6,359698	1,23E-08
TMEM238	6,355039	3,48E-13
KHK	6,345511	4,79E-47
FDXR	6,340828	3,59E-27
ARVCF	6,336343	2,49E-41
F11R	6,323727	1,64E-125
TP53TG1	6,322496	3,21E-25
RAP1GAP2	6,311451	8,70E-43
FAM174B	6,30983	9,86E-26
C9orf152	6,309106	1,59E-27
SYNE4	6,251443	5,70E-34
LMTK3	6,250339	1,71E-13
C19orf57	6,24983	5,03E-05
CGREF1	6,236894	1,81E-49
C4orf19	6,23135	1,61E-05
TNFAIP8L3	6,199209	3,18E-24
NUDT7	6,196253	1,55E-05
ZNF185	6,170264	3,48E-21
ZP3	6,165715	5,28E-38
CCDC88C	6,161807	1,03E-55
MTSS1	6,152723	2,65E-13
VRK2	6,124443	1,36E-33
BIK	6,124198	1,25E-07
CGN	6,112954	1,89E-48
ADCY1	6,111209	1,80E-21
INHA	6,104441	0,000352

SORBS1	6,094391	2,89E-05
KLHL29	6,08606	4,80E-05
NES	6,070829	1,33E-19
FAM83E	6,06971	9,52E-05
CKMT1B	6,069641	3,94E-46
MIR4737	6,063405	6,37E-05
C2orf81	6,038763	6,82E-10
FGFR4	6,0372	2,46E-59
DLG3	6,004633	4,99E-74
ADAMTS13	5,996542	1,99E-06
TMEM52	5,991969	3,25E-10
CTXN1	5,974623	2,54E-61
STARD10	5,962318	7,72E-57
KYNU	5,905472	2,19E-23
LLPH-AS1	5,905095	8,60E-05
ALDOC	5,899758	1,37E-26
ZSCAN12P1	5,898727	4,96E-09
PCDH1	5,882769	5,82E-37
ACSS3	5,842497	2,49E-113
ANXA3	5,839988	8,47E-08
CELF5	5,831815	1,40E-06
GSTO2	5,829613	1,53E-37
ENTPD1	5,828168	1,10E-13
ZNF385C	5,8187	5,33E-06
CSPG5	5,796263	1,44E-08
HLA-DRB1	5,796008	2,10E-07
PAIP2B	5,794132	2,05E-26
RBM47	5,787323	5,15E-51
XK	5,784482	1,88E-06
DMKN	5,777436	2,76E-18
TENM1	5,775708	2,03E-05
NRXN3	5,772173	1,90E-07
CELSR2	5,76953	1,10E-85
RIMS4	5,756278	3,63E-13
GLDN	5,7511	0,000244
ACBD4	5,749028	0,000131
WNK2	5,739795	1,04E-43
NRARP	5,733828	2,72E-23
ITGA2B	5,72789	6,04E-07
VAV1	5,717267	3,54E-10
ZNF296	5,697858	8,96E-25
STAP2	5,695248	2,08E-59
EHF	5,68955	2,44E-14
LSR	5,67794	1,86E-111
LOC101927	5,661457	3,19E-28
PCDHA13	5,658009	1,99E-05
AGMAT	5,657983	5,48E-17
TMEM191A	5,656108	2,75E-29
MAPK13	5,654328	1,04E-34
NPNT	5,647971	9,64E-31

PPM1E	5,646595	1,82E-20
CCDC170	5,637127	1,00E-11
KCNN1	5,615851	0,000787
DNAH3	5,615153	5,30E-06
DHRS2	5,613245	1,76E-32
HSD11B2	5,609266	6,07E-13
C2CD4C	5,588484	0,000793
TRPV3	5,578756	1,77E-07
FHIT	5,570938	5,05E-10
F12	5,570236	2,76E-87
PPP1R9A	5,566522	1,88E-47
MYT1	5,556103	1,36E-07
RAI2	5,503829	0,000138
TNFRSF18	5,488142	1,94E-05
CEBPA	5,482538	3,89E-16
KLHL13	5,480272	5,66E-07
RAB3D	5,476338	1,37E-63
AMZ1	5,461607	3,29E-05
EGFL7	5,458521	1,18E-29
SLC37A1	5,458501	1,56E-26
SLC27A5	5,454692	6,63E-36
PKP3	5,448347	1,91E-71
LPAR2	5,445682	2,87E-23
GGT1	5,432148	3,10E-21
LINGO1	5,43182	3,75E-44
SLC2A4	5,41729	1,65E-05
OCLN	5,414574	6,19E-34
PPFIBP2	5,413136	4,33E-20
ERG	5,393232	1,61E-05
SLC27A3	5,386939	3,41E-43
AP1M2	5,386193	5,40E-106
KCNJ3	5,384381	3,94E-06
PRAME	5,373242	8,39E-10
SPTBN2	5,367158	1,75E-56
PRKCZ	5,365196	4,73E-44
MIF4GD	5,361971	1,27E-11
LMF1	5,359163	6,04E-25
CACNA2D2	5,350919	0,0009
ACTL10	5,350673	6,10E-06
ISYNA1	5,34267	2,18E-57
CNIH2	5,335135	1,20E-18
ELMO3	5,331805	3,22E-32
NAALADL2	5,32935	4,55E-18
DGAT2	5,328111	2,96E-17
DBNDD1	5,319144	1,72E-83
HS6ST2	5,300247	2,63E-31
ZFHX2	5,280343	0,000189
TMEM254-	5,277538	2,82E-07
CRABP2	5,250061	5,81E-33
EBP	5,246229	8,21E-24

ARRDC4	5,242336	1,30E-24
PLCH1	5,240381	3,99E-45
MISP	5,239051	5,86E-32
MYO5B	5,238126	6,16E-74
PAX5	5,233822	0,000641
CTSV	5,229754	2,65E-08
RNF43	5,221651	1,97E-104
SYNPO2	5,213977	1,67E-21
NUDT8	5,211426	1,52E-26
TMEM163	5,204875	2,87E-05
RASGRF1	5,201005	5,05E-28
ACBD7	5,195201	6,93E-20
OGDHL	5,186127	7,19E-14
FES	5,179713	0,000393
PROSER2	5,177551	3,84E-13
EFNA3	5,174805	3,20E-28
EEF1A2	5,167518	1,52E-71
SEMA4A	5,151566	4,43E-18
ASS1	5,147417	1,87E-40
ZFYVE28	5,145059	7,73E-14
CDS1	5,138911	1,86E-51
JMJD8	5,126135	1,93E-76
GRB14	5,121981	1,31E-24
LGR6	5,118988	3,15E-05
GPLD1	5,115898	1,07E-06
PRLR	5,110059	3,09E-45
MBP	5,096394	0,0002
CYP2R1	5,079998	7,65E-05
PLEKHH1	5,074594	9,96E-30
ABLIM2	5,073226	6,27E-05
ENPP5	5,072214	9,81E-06
FUT1	5,071605	1,80E-10
PTPRO	5,06379	1,16E-13
EXPH5	5,059249	5,20E-11
BTG2	5,058607	2,75E-26
MANSC1	5,055516	5,91E-20
GALNT18	5,053014	4,48E-11
TTYH2	5,04902	1,95E-07
SP6	5,043349	4,38E-29
CXCL16	5,042359	1,52E-25
FGFBP3	5,039527	0,000125
RAB39B	5,035586	0,000109
TP73	5,032576	1,40E-09
SP5	5,023961	3,15E-66
SUSD2	5,02225	5,11E-08
ZNF681	5,020147	8,64E-28
SMKR1	5,014137	2,02E-18
PEX1	5,01121	3,11E-37
CCDC74B	5,00704	5,67E-06
ZNF165	5,000207	1,84E-38

RAB26	4,995313	5,09E-09
RNASET2	4,977333	2,17E-32
JAG2	4,973696	9,87E-39
SELENBP1	4,963395	1,22E-48
ZNF837	4,961305	5,21E-13
ATP1A3	4,947622	5,14E-18
ZNF610	4,946489	0,000376
NFE2	4,883423	8,44E-07
GATA3	4,87812	4,89E-85
C15orf59	4,877496	8,31E-14
KRT15	4,874518	2,81E-05
IL17RE	4,842322	9,39E-05
ARRDC1	4,803396	1,94E-53
FRAS1	4,796146	2,32E-28
GOLGA7B	4,792678	2,07E-11
ALDH5A1	4,772294	4,30E-49
DCDC2	4,754487	7,32E-10
DKK1	4,745271	4,59E-15
DMTN	4,735985	2,04E-22
ABAT	4,735043	3,74E-12
CDK18	4,728275	1,76E-20
RPARP-AS1	4,710362	4,19E-09
NRCAM	4,702775	4,75E-22
TMEM102	4,69674	7,13E-19
RIPK4	4,695382	1,15E-14
CADM4	4,67969	2,27E-25
PCDHB4	4,675611	1,08E-05
LOC10013[illegible]	4,670682	6,81E-05
AQP3	4,65706	0,000415
RNFT2	4,654341	2,80E-10
RPP25	4,644324	1,86E-42
SH2B2	4,641624	4,69E-23
CPNE7	4,633803	1,11E-12
CDKN1A	4,630765	5,00E-21
NPW	4,630559	3,42E-06
MAST1	4,622833	4,20E-11
LOC43999[illegible]	4,613594	6,46E-07
ZNF607	4,609864	1,20E-30
ABCA2	4,592688	1,60E-50
FMO5	4,588665	1,47E-05
STMND1	4,57571	0,000457
EFNB3	4,564878	2,24E-14
PRSS27	4,563786	1,60E-05
TRAPPC6A	4,556654	1,53E-25
SYNGR1	4,551718	1,19E-10
ADRA2C	4,550039	2,20E-43
ACE	4,548918	0,000316
MARCH1	4,533785	6,96E-07
DOCK6	4,529995	1,39E-50
SEMA4G	4,511691	1,28E-06

HOOK2	4,491354	2,03E-57
PCDHGB5	4,484824	6,91E-06
TNNT1	4,48329	3,91E-98
H1F0	4,47659	1,01E-24
LDHD	4,471135	3,88E-06
ROGDI	4,470557	2,98E-38
KLHDC7B	4,467543	1,07E-06
LINC00052	4,457884	6,48E-06
CRYL1	4,450999	2,82E-27
RAB3IP	4,445008	3,94E-79
SCNN1A	4,425829	7,32E-06
MACROD1	4,414834	9,51E-30
CHMP4C	4,413778	7,33E-22
CYB561	4,403265	1,37E-120
ZNF711	4,402449	7,90E-10
FAM117B	4,379272	1,90E-27
NAT8L	4,378904	2,86E-18
ZNF630	4,378285	1,02E-06
SAMD10	4,374494	1,34E-08
GATS	4,371032	7,29E-13
FBXO27	4,37084	8,24E-15
TCEA3	4,365674	6,13E-50
SEMA6B	4,362857	5,81E-22
GALNT3	4,360324	3,86E-45
DPYSL4	4,35557	1,76E-08
TLE2	4,355058	2,18E-40
NKAIN1	4,352504	3,06E-21
SEMA4D	4,347635	4,48E-17
RAB11FIP4	4,332295	9,35E-52
ADGRG6	4,325911	4,93E-10
FGD4	4,318837	1,59E-16
SLC4A11	4,311527	5,11E-06
HIST1H3G	4,311418	0,000413
VAV3	4,310812	1,39E-45
IL17RD	4,307114	8,48E-14
UFSP1	4,299812	0,000151
HSD17B8	4,288258	4,67E-14
CYP4F11	4,28745	1,36E-05
TGM1	4,287363	4,61E-06
RHOF	4,287153	7,75E-33
ROCK1P1	4,275441	7,49E-05
ARSG	4,271836	4,95E-10
PRTFDC1	4,258824	1,77E-05
TMEM134	4,254002	8,27E-28
CFAP53	4,252282	3,32E-05
GJA3	4,251743	2,93E-07
MFSD6	4,246338	3,44E-34
CNKSR1	4,24002	4,49E-13
MYLIP	4,238204	5,92E-33
GSTZ1	4,238029	1,90E-64

ZDHHC11	4,23689	0,000604
LIN7A	4,236046	1,11E-26
GAA	4,235217	5,41E-23
DYRK1B	4,234565	1,03E-24
CNFN	4,229779	0,000631
IDNK	4,226784	8,06E-14
DENND6B	4,224962	1,22E-08
C11orf80	4,224581	1,32E-25
PCDHA6	4,214077	1,14E-45
SYNGR3	4,211778	3,24E-06
SYBU	4,203832	3,05E-08
SULF2	4,189164	1,21E-51
GREB1	4,189105	6,71E-43
CACNB3	4,184531	4,14E-61
MTL5	4,181216	1,59E-18
DDR1	4,180633	4,73E-26
BEND5	4,167405	1,42E-08
LOC729737	4,165817	0,000356
GDF15	4,138572	4,10E-16
TUSC3	4,136439	5,37E-26
ADAM11	4,125426	1,81E-14
SHISA9	4,123604	1,08E-09
MYEF2	4,123079	1,33E-26
NOTCH3	4,122304	9,34E-75
KSR2	4,114508	2,02E-06
C6orf52	4,111551	1,61E-06
RHOBTB1	4,105011	2,60E-89
CA8	4,102387	3,63E-05
GRHL3	4,100644	4,76E-17
SEPT3	4,099905	3,03E-20
DCLK1	4,079961	2,55E-34
PTGES	4,07284	2,40E-07
ST8SIA4	4,051114	8,03E-10
MFAP2	4,049109	4,87E-16
EPB41L4B	4,03142	1,59E-24
ADAM22	4,031126	4,30E-28
ELOVL7	4,027111	8,99E-34
CSTA	4,026083	1,42E-05
THEM6	4,020077	1,65E-28
ELL3	4,000791	4,70E-18
LRRC75B	3,996958	5,70E-14
ZBTB42	3,993681	4,97E-38
REPS2	3,993446	0,000178
RASD1	3,993297	0,000372
TKFC	3,992868	3,71E-59
ZSCAN16	3,990178	1,92E-25
LAMP3	3,989213	5,65E-05
FSD1	3,988371	4,17E-08
CREB3L4	3,980307	9,59E-40
MTUS1	3,98025	3,41E-38

ISM1	3,976567	1,83E-07
ZNF554	3,976508	3,49E-07
DLL1	3,975002	7,79E-05
PIK3C2B	3,960614	3,47E-42
JAM2	3,959632	0,000352
KIAA1324	3,958033	6,95E-15
ALDH4A1	3,955705	5,38E-13
KIFC2	3,955485	2,59E-29
PALM3	3,9522	2,33E-05
ZNF385A	3,950695	1,03E-56
INHBB	3,944276	6,51E-09
ZNF204P	3,94091	3,21E-06
HAGHL	3,938422	1,73E-21
ZNF48	3,932605	2,33E-17
LIPT2	3,928181	3,51E-09
KCNK6	3,927264	1,80E-13
DOK4	3,92708	2,76E-23
C3orf14	3,920241	4,26E-45
ZBED6CL	3,916398	0,000462
TUBB4A	3,915554	7,51E-07
GLUD2	3,909687	7,64E-14
HOXC-AS3	3,909417	0,000814
TP53	3,907762	5,12E-35
PRDX2	3,906912	1,45E-75
PROCA1	3,901501	2,44E-05
TMPRSS2	3,89875	5,88E-05
EFEMP1	3,898045	3,24E-57
DYNC1I1	3,894529	6,33E-37
ZNF737	3,885203	2,35E-14
CBS	3,88321	1,36E-21
RGAG4	3,882523	7,76E-07
FRMD4B	3,882365	3,24E-05
AGAP2	3,881316	4,16E-16
SH3GL3	3,879884	1,04E-05
OLFM2	3,877211	4,40E-30
FHDC1	3,873453	1,16E-11
IDUA	3,870702	1,09E-11
TMEM229E	3,858512	2,85E-06
RALGPS1	3,858468	4,34E-12
C17orf96	3,821624	5,26E-38
TSPAN13	3,819946	2,15E-42
KCNJ8	3,818901	2,99E-25
JPH1	3,815418	2,51E-10
SCAMP5	3,810859	2,18E-15
NBEAL2	3,809646	2,02E-26
EPS8L2	3,793789	6,95E-21
ZNF793	3,785283	1,07E-16
TCHH	3,77985	0,000502
MGAT4A	3,77425	6,83E-15
PLCG2	3,769274	5,14E-18

NCOA3	3,765591	3,33E-57
CEL	3,764773	0,000412
PTPRG-AS1	3,760699	4,85E-09
MPP7	3,752157	3,41E-33
SIAE	3,748566	2,65E-23
SORT1	3,744569	1,15E-35
SREBF1	3,741134	7,75E-48
SPATA17	3,739926	4,15E-10
KIAA0040	3,737245	1,27E-15
ASAH2	3,737019	2,09E-15
MFSD3	3,733817	6,86E-23
PPM1D	3,733174	2,30E-50
CYBA	3,729792	1,02E-50
SEPT1	3,729676	1,72E-07
GSTM3	3,727438	6,80E-15
ONECUT1	3,71951	0,000317
HSD3B7	3,719388	1,27E-35
VGF	3,715256	4,78E-16
PHLPP1	3,714968	6,43E-35
FAM86C2P	3,712518	6,98E-11
TRIM37	3,702672	4,57E-102
BAMBI	3,702493	1,29E-83
RCOR2	3,692093	5,59E-12
CCDC113	3,681156	7,27E-05
STMN3	3,6771	6,93E-24
ARHGAP4	3,676746	7,78E-07
RPS6KL1	3,672593	9,38E-12
C10orf35	3,662996	2,33E-11
HEYL	3,650698	0,000203
FRAT1	3,643884	0,000301
GCH1	3,641799	2,40E-20
SALL2	3,640196	2,33E-22
GSTM4	3,632268	3,89E-30
PLEKHG1	3,632138	2,69E-07
SH3YL1	3,620881	1,91E-29
GRHL1	3,619196	4,36E-07
LCA5L	3,597153	0,000135
SLC15A2	3,590664	0,000223
CRMP1	3,589936	7,09E-08
GFRA1	3,589909	4,63E-25
FIRRE	3,575327	1,80E-05
ATHL1	3,574632	1,51E-05
TGFBR3L	3,564776	8,19E-07
BBS2	3,561155	4,38E-16
PRR5	3,560064	7,70E-10
BOLA1	3,551888	8,58E-28
PBX1	3,548846	1,35E-12
CACNA1H	3,541923	8,93E-41
OSR2	3,539272	1,83E-07
VPS37D	3,537172	2,53E-05

RINL	3,53272	3,38E-06
NAAA	3,531647	1,61E-18
EGLN3	3,530178	8,51E-06
NEURL1B	3,529009	1,35E-25
TP53INP1	3,523578	5,41E-09
SLC7A2	3,521774	3,81E-36
CASZ1	3,521165	1,88E-18
FAM78A	3,520008	0,000615
ERV3-1	3,519024	1,25E-21
LOC101927	3,517277	3,32E-09
ZFP14	3,512765	1,45E-08
GDPD1	3,511466	6,49E-06
GPR160	3,508119	9,13E-13
MYH7B	3,505632	0,000334
C1orf233	3,505392	7,12E-32
SLC37A4	3,500777	5,07E-26
ZCWPW1	3,500593	2,08E-10
BCAM	3,500114	1,46E-30
LAMA3	3,496438	3,87E-12
BRSK1	3,495453	2,92E-10
CCDC74A	3,492653	6,70E-06
SAMD12	3,491229	2,24E-11
PGAP2	3,488295	2,12E-28
ZNF443	3,488164	3,00E-12
ZNF860	3,482466	3,55E-05
ZNF311	3,477321	0,000239
TMEM154	3,465695	0,000332
TNS1	3,462473	4,15E-08
TINCR	3,461212	3,21E-05
RMND5B	3,460782	6,37E-43
GPC2	3,459063	2,43E-06
BMP2	3,443976	1,35E-31
CITED4	3,43842	6,77E-05
ANKRD18B	3,4383	4,66E-08
SLC24A1	3,431104	2,27E-19
KCNH3	3,427806	0,0006
DOC2A	3,426183	8,11E-06
GUCY1A3	3,423913	5,89E-15
LRP5	3,421335	2,69E-59
LOC339862	3,417703	8,35E-06
ACTG2	3,417346	0,000539
RPL39L	3,415582	1,34E-27
FAM227B	3,415172	2,26E-11
NDRG2	3,402297	9,63E-12
PCDHB13	3,40222	2,10E-13
PRICKLE2-A	3,397186	0,000103
METRN	3,391093	2,33E-29
PDCD4	3,382	3,39E-35
TPD52	3,381861	2,56E-35
ZNF287	3,375658	2,34E-07

ALDH6A1	3,371426	6,48E-38
CASKIN1	3,36947	2,83E-13
RHPN1-AS1	3,368878	0,000192
CABYR	3,367216	1,10E-07
ZCCHC12	3,362867	6,18E-05
USP32	3,360569	1,26E-28
TRPM4	3,35615	5,52E-24
LENG9	3,346232	4,69E-18
MDK	3,34433	1,42E-54
TLCD2	3,33986	2,29E-08
AKTIP	3,32044	1,39E-22
DHTKD1	3,319216	5,50E-46
LHPP	3,316584	8,74E-14
PACS2	3,313253	6,60E-43
EFNA2	3,309908	0,000484
JMY	3,300897	2,97E-08
ZNF219	3,300032	1,26E-21
ARFGEF3	3,297298	6,80E-14
ERICH2	3,287124	3,14E-13
SH3GLB2	3,285931	2,73E-40
TLCD1	3,285004	7,62E-31
DNMT3B	3,280487	2,33E-32
SYNGR2	3,276716	1,52E-24
WASF3	3,276466	4,05E-17
HIST3H2A	3,274164	5,65E-08
PCAT7	3,27284	0,000169
DAAM1	3,271013	3,59E-22
ZNF397	3,262968	2,39E-17
CCDC24	3,262652	5,53E-06
PSD4	3,26213	5,53E-25
OLMALINC	3,253451	6,41E-12
USP43	3,2519	3,57E-07
RAP1GAP	3,250463	1,08E-08
GLIDR	3,243191	1,42E-05
ZNF329	3,243104	1,64E-12
ISG20	3,240825	0,000602
TRIM24	3,237319	2,14E-42
SHF	3,230161	2,56E-08
C5	3,227935	6,08E-09
WNT7B	3,220912	4,52E-11
ZDHHC24	3,219684	2,39E-11
SUV420H2	3,212977	1,87E-13
CYP2J2	3,211534	4,85E-11
TMTC2	3,210446	3,27E-10
PGM2L1	3,209226	7,33E-22
LOC10099€	3,201696	5,75E-06
MOSPD3	3,200444	1,14E-15
GAD1	3,199791	5,42E-13
RMI2	3,188307	4,57E-17
APRT	3,186623	1,18E-68

APPBP2	3,185957	1,43E-41
SYT1	3,180869	1,58E-11
AMOT	3,176284	9,59E-23
GATSL3	3,170654	4,91E-05
LRRC73	3,167136	2,86E-06
TFAP2A	3,16382	1,14E-60
LRRC24	3,163703	6,38E-06
RNF44	3,161581	4,68E-40
SSH3	3,153327	7,35E-24
HCG11	3,152104	1,51E-09
BRIP1	3,142944	3,15E-47
RB1	3,139358	1,17E-17
THNSL2	3,136891	5,95E-10
CD9	3,122986	1,70E-35
MEX3A	3,122893	2,10E-50
MARCH9	3,119395	8,58E-20
C1orf116	3,117685	0,00092
MPND	3,116772	2,96E-18
USP18	3,115994	1,36E-08
MACC1	3,115723	0,000639
COL9A2	3,113186	1,44E-08
B3GNT4	3,106236	0,000638
PODXL2	3,106032	9,64E-34
ATP6V1E2	3,10342	4,81E-08
EFHD1	3,102404	1,25E-09
SPSB4	3,102099	0,000493
HEATR6	3,094853	3,76E-54
GSE1	3,070245	1,91E-65
SPATA2L	3,069449	1,26E-22
ASNS	3,068524	2,09E-14
SLC2A11	3,068289	7,42E-13
DUS4L	3,063131	1,84E-23
PSMD6	3,059933	6,08E-49
TDRKH	3,056885	2,13E-14
THAP9	3,047331	0,000143
PSTPIP2	3,042228	1,18E-14
MAP3K9	3,037207	5,01E-21
GAB2	3,035133	2,18E-11
NMB	3,030343	2,12E-13
CELSR3	3,026715	3,09E-15
EPS8L1	3,024499	7,65E-08
EDA2R	3,02407	0,000122
LCP1	3,020039	6,90E-09
BAIAP3	3,017077	9,86E-05
CD24	3,016522	8,40E-35
ADSSL1	3,012331	0,000723
DNPH1	3,011221	1,49E-42
ACSS1	3,011059	4,07E-06
RTN4R	3,003009	3,78E-08
MOAP1	3,001403	5,68E-18

SCD	3,000034	1,20E-62
ITPK1	2,998252	4,57E-45
C16orf46	2,998033	2,73E-09
HOMER2	2,996591	1,63E-34
TBC1D9	2,995385	2,77E-31
LRRC8B	2,993984	2,71E-20
MEIS3	2,976854	4,82E-05
C1orf226	2,975763	4,64E-05
ZNF347	2,97543	3,31E-08
MREG	2,973183	8,06E-20
IQCH-AS1	2,971418	2,59E-12
ALKBH2	2,970535	3,14E-25
TMEM254	2,966261	6,27E-18
TMEM132A	2,964082	7,98E-17
ARHGEF19	2,957853	1,98E-11
TMEM141	2,956929	5,32E-39
PCK2	2,955989	2,94E-07
LOC284023	2,95585	0,000782
PAN2	2,952552	1,79E-06
PQLC3	2,951952	3,79E-11
NHS	2,951413	4,22E-08
SEMA3F	2,946458	5,19E-22
SNHG9	2,946383	0,000427
SMIM14	2,946186	5,67E-07
LHX4	2,943774	6,40E-22
VSIG10L	2,9434	1,67E-08
BBC3	2,942846	1,18E-09
DHRS13	2,939547	5,79E-17
PPP1R13B	2,93659	6,10E-22
LNX2	2,931609	1,93E-26
ARF5	2,926053	6,36E-45
IGFBP5	2,919536	2,18E-13
MEPCE	2,919533	1,66E-64
PPDPF	2,91402	3,00E-41
ZNF799	2,910756	3,65E-05
CA11	2,90839	0,000751
PPM1L	2,906251	2,27E-06
SLC22A18	2,899796	5,74E-11
CPLX1	2,899548	9,36E-08
LOC645513	2,886863	1,84E-06
C20orf96	2,884477	6,11E-06
C9orf116	2,867544	2,11E-09
ZNF43	2,86594	6,99E-29
THBS3	2,865464	9,77E-08
CCDC121	2,864713	3,83E-05
HIST1H2BD	2,864074	2,74E-07
PAQR4	2,859677	3,51E-48
SLC16A13	2,857827	2,93E-08
IMPA2	2,852908	2,14E-23
EXD2	2,85174	3,06E-34

CCNG2	2,846286	3,62E-09
ZNF217	2,842716	4,05E-34
RASSF2	2,839258	0,000152
ERMARD	2,838391	8,45E-10
RAD51C	2,837551	2,86E-39
RAB30	2,828326	1,40E-15
ADAM15	2,823849	5,06E-44
PI4K2B	2,82378	2,71E-16
FAM171A2	2,821379	1,87E-25
SIAH2	2,815622	1,72E-27
ZNF425	2,807035	5,37E-12
DTWD1	2,806669	2,18E-18
PFDN4	2,796228	8,93E-41
DNASE2	2,793924	1,03E-28
PCDHA7	2,792683	0,000222
HCFC1R1	2,791878	1,59E-19
TRPT1	2,790439	1,59E-13
TMEM170E	2,787093	4,50E-06
LOC10023	2,786533	9,72E-06
B4GALNT4	2,784012	8,04E-11
CACFD1	2,773756	1,01E-14
ZNF354A	2,773344	3,23E-16
KRT7	2,770591	0,00017
SH3BP5	2,769539	3,07E-38
ZNF33B	2,769071	7,56E-16
SLC39A4	2,768749	6,34E-17
TRAPPC9	2,768691	2,57E-14
APOE	2,765318	0,000173
STK32C	2,765146	6,76E-14
PAQR8	2,764043	6,17E-08
PLXNB1	2,761175	2,42E-16
TUBD1	2,760993	7,56E-16
MSX2	2,757377	1,23E-16
CHCHD5	2,754531	6,90E-15
COMTD1	2,749623	1,35E-20
MLLT3	2,749575	2,90E-09
MAN2B1	2,748986	1,71E-25
PLEKHF2	2,745576	9,57E-30
ZNF497	2,743875	0,000136
NOTCH1	2,736793	8,66E-41
CCNO	2,727183	1,23E-06
LRRC20	2,721543	1,02E-08
ACOT4	2,715962	5,63E-05
FLJ23867	2,715659	1,57E-13
DHCR7	2,715245	4,13E-22
TNFRSF19	2,708826	9,66E-26
ANKIB1	2,70353	2,10E-35
ZNF836	2,700808	7,96E-09
NR1H3	2,698818	1,38E-06
ZNF391	2,698801	1,42E-05

SLC25A23	2,696868	2,22E-13
TFAP2C	2,693958	1,06E-25
SLC47A1	2,693937	0,000131
ZC3H6	2,687605	9,37E-09
AP1S1	2,686875	6,07E-16
RPS6KB1	2,685357	5,68E-54
ZNF253	2,68384	9,68E-23
FAM213B	2,680266	8,71E-15
FGF12	2,676728	7,56E-07
MMAB	2,676188	5,04E-35
CYP51A1	2,672525	1,63E-46
PITPNC1	2,670276	4,63E-37
NXPH4	2,669331	1,53E-19
RHBDF1	2,668326	3,25E-13
C16orf74	2,666871	1,96E-05
PTPRU	2,663977	4,31E-16
BTBD11	2,662652	0,000118
ZNF525	2,660633	1,15E-15
C21orf33	2,66014	2,23E-20
ZNF620	2,658566	1,01E-09
GOLGA2P7	2,658339	5,36E-07
CLU	2,657316	2,26E-11
FAM102B	2,656839	2,34E-28
AP4M1	2,652934	1,98E-17
ACVR2B	2,650949	2,39E-07
MYCL	2,650312	6,55E-05
AR	2,649434	4,13E-09
NANOS1	2,646529	5,90E-15
GATAD1	2,644443	3,92E-17
PCBD1	2,642522	7,36E-27
SNHG5	2,641256	3,24E-09
ZNF736	2,640936	2,94E-13
PRMT6	2,640198	2,59E-36
C14orf28	2,640161	9,98E-05
PSMG3-AS:	2,639537	4,47E-09
NIPSNAP1	2,638794	2,59E-31
SOCS1	2,638756	5,68E-05
GALNT16	2,635999	0,000775
COBLL1	2,635989	6,47E-14
ABLIM1	2,635893	3,78E-19
CIART	2,635165	2,30E-08
TYSND1	2,63342	2,80E-26
DHRS12	2,630791	1,00E-05
NME3	2,63004	9,49E-09
RMND5A	2,624094	1,37E-22
CMTM4	2,623173	1,65E-31
ATP7B	2,622214	0,000457
ZFP3	2,620007	8,41E-08
KLC3	2,618862	0,000352
GPRC5C	2,616087	9,17E-13

NIPAL1	2,612584	0,000611
CCDC120	2,612527	2,29E-08
ARHGAP27	2,610202	1,12E-12
PRKX	2,607039	3,14E-16
LOC72921\u0038	2,605968	1,99E-05
KBTBD7	2,604204	5,48E-12
CTPS2	2,603418	7,29E-23
JHDM1D-A	2,59681	2,40E-09
MARCH2	2,595806	5,02E-07
IRX5	2,593569	4,89E-18
NKD1	2,592509	3,54E-21
SDC3	2,590638	2,82E-15
ARMC10	2,589766	3,73E-32
CARF	2,588392	0,000141
GCA	2,576913	2,86E-12
FBXW4	2,575333	7,18E-22
ZNF286B	2,574386	3,83E-06
THNSL1	2,572089	3,49E-21
NHP2	2,571124	4,29E-49
IDH2	2,5684	2,52E-43
LINC00665	2,564591	1,40E-12
PHF10	2,563501	2,64E-19
FZD3	2,562939	3,14E-08
ASNA1	2,562073	4,64E-35
RALGAPA1	2,561042	1,92E-19
LOC10192\u0038	2,560598	0,000612
EFNA4	2,557189	5,38E-22
DGKE	2,554872	5,21E-08
ATG16L2	2,553907	0,000287
ATXN7L3B	2,550695	8,58E-41
HOXC11	2,546662	2,65E-06
TMTC4	2,543358	6,23E-12
NUDT14	2,543045	9,44E-19
TSNARE1	2,542798	8,68E-06
ETNK2	2,539932	2,10E-09
RALGDS	2,534513	9,05E-15
NMU	2,533225	1,88E-05
HIST2H2BE	2,533049	1,75E-07
NR4A1	2,532517	1,34E-06
SOX13	2,530278	1,67E-10
SYNE2	2,528506	3,22E-19
IMMP2L	2,524579	7,10E-08
ANKRD34A	2,523667	3,57E-09
THOC6	2,523174	5,70E-09
DUSP2	2,513408	9,44E-05
LRBA	2,511847	8,08E-18
KCTD1	2,509268	8,00E-13
INADL	2,507357	1,76E-20
TMEM184/	2,505825	1,27E-09
MGST2	2,503656	1,42E-07

EPB41L5	2,502738	6,76E-25
CCND1	2,501926	9,18E-23
GRIP1	2,501557	3,92E-06
FLJ10038	2,499819	4,36E-11
ZNF74	2,496784	9,45E-18
FUK	2,494556	4,64E-07
TMEM231	2,492532	2,31E-11
BCL2L1	2,492423	6,91E-17
LPIN3	2,487283	9,65E-14
RPL17	2,476115	4,20E-11
SAPCD2	2,475548	1,55E-17
DOPEY2	2,47507	2,15E-21
TSPAN14	2,473843	5,75E-21
SLC29A3	2,473	1,32E-06
KIF16B	2,471875	4,74E-07
KRIT1	2,470855	2,04E-21
KIAA1958	2,470012	1,45E-14
ZFP62	2,467946	1,15E-29
NUDT3	2,46789	1,79E-11
SLC4A8	2,467699	0,000426
PTPRF	2,462745	8,29E-32
FITM2	2,462649	1,04E-10
ZNF33A	2,459326	3,09E-22
HILPDA	2,457827	2,40E-14
IDH1	2,452268	1,43E-32
CHD6	2,445018	5,37E-19
XBP1	2,443145	3,19E-09
UNC93B1	2,442622	9,26E-17
PYCARD	2,441698	1,85E-09
ANKRD13B	2,440941	6,99E-19
FAM221A	2,437263	0,000393
SBF2-AS1	2,435412	6,03E-05
MAP2K6	2,42488	2,65E-05
DECR2	2,421561	1,76E-15
SLC29A4	2,420967	1,20E-14
PRPF40B	2,420561	7,29E-09
ECI1	2,41686	7,37E-29
TRIM33	2,416377	3,00E-24
DSC2	2,413805	1,52E-23
RAB33B	2,413183	1,48E-07
KIAA1211	2,411958	2,37E-10
H2AFX	2,409042	1,12E-39
PRKAR2B	2,406566	5,95E-06
NEK8	2,404945	0,000314
PCYOX1L	2,404623	6,95E-09
MTERF1	2,404232	3,60E-10
PRRT3	2,404216	0,000469
OBFC1	2,402658	8,78E-10
CUX1	2,402645	1,05E-28
WRB	2,40247	3,22E-13

MYB	2,401653	0,001007
ZHX2	2,398241	4,18E-14
ZNF117	2,396171	3,14E-13
PDZD4	2,39564	1,37E-06
TEAD2	2,395486	1,05E-17
DUSP8	2,393882	4,91E-05
DDB2	2,388354	2,70E-09
HYKK	2,386481	0,000918
FIS1	2,380999	6,56E-29
ZNF433	2,380529	7,19E-08
MAPK11	2,379392	2,13E-05
LINC00886	2,377127	0,000159
IFT22	2,375835	9,89E-26
SALL4	2,374744	8,24E-05
JUND	2,372224	7,17E-12
SCAMP1	2,371945	2,08E-18
SLC25A40	2,371444	1,15E-10
IQCK	2,371088	2,46E-10
MMP15	2,370882	9,07E-13
RAC3	2,370323	1,14E-14
ZNF879	2,367183	0,000395
TRIM36	2,363002	7,45E-10
TMEM116	2,361588	1,04E-05
TMEM54	2,361434	1,63E-15
GMCL1	2,35655	4,64E-12
H2AFY2	2,355861	1,50E-15
HDDC3	2,34888	7,99E-14
DYM	2,348617	2,55E-24
LAMTOR4	2,34264	4,28E-25
FAM185A	2,340786	6,82E-09
TSPAN31	2,339792	2,70E-07
ZSCAN21	2,336758	1,13E-17
CUL7	2,336587	1,24E-14
DPP7	2,330094	4,86E-27
ABCA7	2,329972	3,06E-06
IFT140	2,329708	6,58E-07
PODXL	2,328166	1,58E-17
TGIF1	2,327117	5,31E-14
FAM86EP	2,325164	5,02E-06
SEZ6L2	2,321717	3,24E-12
ADPRHL1	2,321332	5,99E-09
ZFP90	2,321214	2,91E-16
ZC3H8	2,318695	1,02E-07
GTPBP10	2,318173	4,75E-15
TUBA1A	2,317211	1,33E-29
ZKSCAN1	2,31568	8,95E-28
MBNL3	2,307545	6,11E-12
GALK1	2,307185	1,01E-17
C1QTNF9B-	2,307011	1,15E-05
TCEA2	2,305845	7,99E-09

MAPK8IP2	2,304389	8,75E-05
TMEM87B	2,30102	4,52E-14
BCAS4	2,300165	2,86E-07
OARD1	2,297713	3,92E-14
ZNF786	2,297652	2,42E-18
SLC7A5	2,2914	2,84E-30
DUSP9	2,290664	3,09E-05
SLC25A29	2,289634	7,71E-19
ATG10	2,288009	1,21E-09
H2AFJ	2,287956	1,11E-12
MSRB2	2,287851	7,69E-18
PTGR2	2,286797	1,98E-10
EFR3B	2,283129	5,53E-10
PCDHB8	2,282386	9,54E-11
ATP8B1	2,281898	7,98E-16
TMEM198B	2,28122	2,80E-05
WWOX	2,281003	1,20E-13
FECH	2,279936	1,29E-05
ZNF839	2,278965	5,46E-09
PALM	2,273402	4,03E-08
FAM117A	2,273284	6,50E-10
ZNF3	2,273077	2,23E-24
LONRF1	2,272944	7,24E-08
ZNF184	2,272674	4,14E-17
ATP6V1C2	2,272078	1,36E-07
P2RX4	2,271419	7,71E-18
TNFAIP8L1	2,270344	3,63E-07
SMARCD2	2,268583	1,02E-26
ABHD11	2,263761	6,73E-16
DDAH2	2,26259	1,18E-17
OBSCN	2,257052	1,85E-05
ZNF652	2,254577	3,09E-21
ZBTB12	2,25435	1,91E-06
PRDX5	2,251075	2,22E-36
GRAMD4	2,250362	6,29E-06
PIK3R3	2,249706	9,80E-10
HIST1H2BK	2,248168	3,11E-05
MAFIP	2,248163	8,80E-07
GPR27	2,246181	0,000667
RTKN	2,244324	5,63E-13
ENTPD5	2,243949	1,53E-07
THSD4	2,240914	7,97E-08
SIRT5	2,239556	1,82E-14
CECR5	2,238754	7,60E-24
IGFLR1	2,238747	2,43E-05
EVL	2,236433	2,10E-18
TRIB1	2,23457	2,64E-12
TMCO6	2,231896	5,44E-07
FBXW9	2,229421	1,96E-11
GAMT	2,226624	1,48E-11

MDM2	2,225816	3,76E-09
WDR24	2,222755	8,11E-09
PHKA1	2,221661	2,15E-07
TRPS1	2,220883	3,62E-22
CBX2	2,217371	8,00E-25
ZNF114	2,216568	3,90E-09
GATSL2	2,215509	3,28E-19
LOC100506	2,214319	3,81E-05
EPHB4	2,213832	1,95E-34
PREX1	2,213316	7,08E-19
NADSYN1	2,212743	5,45E-15
GCDH	2,212438	4,51E-18
GPT2	2,211957	6,19E-06
CPNE3	2,211156	1,50E-20
MAP3K14-A	2,206722	0,00035
CEMIP	2,206581	9,98E-07
DBP	2,206097	5,94E-11
ZNF813	2,197734	1,69E-07
LTBP4	2,196821	5,64E-10
PEMT	2,192313	9,98E-18
SLC39A11	2,191716	1,82E-07
L2HGDH	2,191514	9,64E-14
ZNF517	2,187403	0,000551
ENOSF1	2,187311	9,92E-15
LRRC23	2,185248	0,000538
CXADR	2,185136	8,10E-09
SPPL2A	2,184598	4,46E-23
DPP3	2,184258	1,05E-15
RNF157	2,183741	0,000168
SPTLC2	2,183715	1,91E-20
S100A16	2,183319	1,68E-10
CROT	2,178706	0,00048
JOSD2	2,176924	1,99E-06
MED12L	2,174542	5,60E-05
TMEM183B	2,173988	2,82E-07
LYPLAL1	2,17268	3,89E-08
TMEM159	2,171748	2,44E-09
TBCK	2,171619	4,82E-14
SLCO4A1	2,169869	7,91E-06
SERINC5	2,168557	1,63E-10
NMRAL1	2,163633	1,97E-21
SFI1	2,160392	3,42E-06
TMEM205	2,160051	4,13E-14
TMBIM4	2,158273	8,15E-14
HSPB8	2,155464	1,24E-08
PKN1	2,155328	7,49E-28
MRPS25	2,154394	4,66E-23
CBR4	2,153718	1,21E-11
ZFYVE21	2,153047	2,24E-11
ALKBH4	2,15247	1,55E-17

ARRB2	2,152402	4,76E-17
WDR83	2,151794	1,27E-12
ANGEL1	2,147876	2,36E-18
MXI1	2,145883	9,76E-08
SOX4	2,14442	6,78E-23
PACSIN3	2,142783	3,27E-22
MMAA	2,142189	0,000294
PUS10	2,141655	3,89E-05
DBF4	2,139736	7,48E-20
FLOT2	2,138985	8,54E-17
FAM50B	2,135868	9,02E-08
MCM3AP-A	2,13555	6,29E-08
SLC25A39	2,132645	3,20E-35
NPL	2,131583	3,51E-06
SLC48A1	2,130848	1,38E-06
SPATA7	2,129072	7,38E-05
FIBP	2,125954	4,72E-24
SERTAD4	2,125505	1,13E-05
FBRSL1	2,124709	9,35E-26
SPATA20	2,120514	6,05E-17
FAM173A	2,116408	4,03E-10
PPP1R21	2,114971	3,83E-08
SLC43A2	2,113561	3,74E-11
TET2	2,111283	4,25E-12
P4HTM	2,106627	4,49E-11
GAS8	2,105839	1,01E-10
STUB1	2,103974	3,52E-28
RNF215	2,103581	0,000197
MYL12A	2,099602	1,06E-23
XPC	2,098413	2,46E-17
ZFP1	2,09494	6,06E-12
GABPB1-AS	2,093046	8,78E-06
COPG2	2,091579	1,42E-14
PRR14	2,090174	2,99E-19
COQ2	2,087201	6,48E-11
TMEM180	2,086691	9,37E-06
SLC9A3R1	2,086148	1,52E-13
SH3BGR	2,086072	0,000404
PLEKHH3	2,079477	2,18E-05
ZNF888	2,076895	1,11E-08
C1orf115	2,075794	3,22E-07
PXN-AS1	2,06517	5,58E-06
IMPDH1	2,063952	1,19E-19
ATP9A	2,062245	1,51E-22
MYL12B	2,061831	1,04E-29
SLC44A1	2,061724	1,01E-20
MYO18A	2,061403	6,67E-09
IQSEC1	2,054221	3,43E-17
ACTR1B	2,05391	1,99E-05
D2HGDH	2,052212	6,87E-08

NUDT4	2,051178	2,90E-15
RILP	2,050827	4,63E-05
RAB11FIP1	2,05048	9,97E-13
CAMK2N1	2,049931	7,99E-05
RBSN	2,049141	1,20E-14
PNRC1	2,04814	1,72E-10
B9D1	2,047831	4,04E-05
RALBP1	2,047563	3,71E-15
C8orf82	2,046148	2,23E-14
CXorf23	2,044912	1,15E-07
C9orf16	2,04393	4,20E-13
BET1	2,042569	1,83E-07
PCDHB6	2,038169	0,000827
PHKB	2,037131	3,06E-07
GLCE	2,036132	5,05E-18
TMEM168	2,035624	4,35E-17
SNHG8	2,033567	3,09E-05
FAHD1	2,033139	2,20E-18
NRSN2-AS1	2,032845	3,01E-05
CMBL	2,032363	9,67E-06
DUSP16	2,031998	4,09E-19
DLD	2,028155	1,05E-12
ZYG11A	2,025623	0,000975
ZNRF3	2,020852	5,09E-29
STC1	2,019696	2,36E-12
C2orf68	2,018387	6,33E-17
KPTN	2,017309	1,45E-06
MBLAC2	2,016765	0,000496
ITPR2	2,015499	1,42E-09
HMG20B	2,012787	9,12E-26
NTHL1	2,011542	1,83E-11
KATNAL2	2,01126	0,000424
PDF	2,011182	4,37E-06
HOXC10	2,010998	1,01E-14
PLA2G12A	2,009136	6,94E-12
CCDC85C	2,009036	1,06E-20
FAM210B	2,004319	3,58E-08
RAB6A	2,004296	7,08E-14
TMEM183/	2,004038	1,63E-22
ZNF783	2,004001	1,79E-07
HAUS4	2,003035	6,35E-06
ZNF688	2,000232	8,95E-07
FAM195A	1,999075	3,24E-18
CAPN1	1,998882	3,87E-07
C4orf48	1,996691	6,21E-07
ZMYM2	1,994258	1,28E-22
TET3	1,993538	6,16E-25
GCSH	1,992323	6,17E-17
DCXR	1,990985	2,44E-20
OSBPL10	1,985807	7,70E-12

SEMA6A	1,983625	7,49E-05
PFKL	1,98315	8,41E-19
KRBA1	1,983034	1,68E-07
CFD	1,981082	0,000661
PHLDA3	1,978925	0,000309
ZNF641	1,978184	6,00E-07
FBXO31	1,977104	3,53E-19
EPB41L4A-/	1,975209	3,71E-06
ZNRF1	1,971	1,01E-20
TMEM150/	1,966107	2,20E-06
SEMA3C	1,962173	2,18E-20
RNASEH1-/	1,962078	3,36E-10
ZNF276	1,961753	1,43E-14
TUBB3	1,960246	1,03E-28
BCR	1,958598	2,53E-09
ZNF664	1,957711	1,23E-16
CSDE1	1,956805	1,66E-16
TSPAN17	1,956699	1,85E-13
ITFG3	1,956052	1,50E-11
RPUSD3	1,955138	2,57E-14
CORO1B	1,953091	7,57E-08
MGC72080	1,952673	1,41E-09
C16orf13	1,949542	4,58E-19
MVD	1,949524	3,68E-07
APLP2	1,948641	5,91E-12
DALRD3	1,94768	3,22E-07
ULK3	1,947483	9,91E-11
TTC30B	1,946677	0,000212
ZNF595	1,946319	1,26E-07
ZNF239	1,941304	0,000499
ANKRD16	1,940581	3,64E-06
POLI	1,939565	0,00014
C7orf13	1,9394	1,93E-05
LOC10496{	1,939395	0,000375
SLC25A10	1,937869	1,75E-19
DTD1	1,935195	2,08E-14
RNF130	1,932586	1,40E-14
BRCA2	1,932489	4,38E-07
CC2D1A	1,929781	1,51E-12
C14orf169	1,929414	1,37E-10
PMS2P1	1,929255	1,99E-07
SPR	1,9206	7,71E-18
KLHDC4	1,919695	5,60E-17
ALPK1	1,916213	3,55E-05
NT5C	1,912349	8,03E-09
PEX11B	1,911073	1,10E-12
ZSWIM7	1,910325	2,84E-09
MTHFD2	1,909982	2,56E-06
DTNA	1,909242	2,13E-07
CLMN	1,908993	2,57E-08

TPCN2	1,90484	0,000312
PLD2	1,90433	3,39E-05
ADCK2	1,904253	4,29E-11
CD320	1,904199	1,36E-15
LMBR1	1,902846	4,10E-15
TMEM216	1,901014	2,63E-05
TRAP1	1,89938	3,97E-26
GIGYF1	1,898956	3,89E-05
HAGH	1,894491	2,11E-07
MAGI1	1,894322	5,48E-09
SNX27	1,893894	9,24E-19
FAM53B	1,893867	1,15E-21
HBP1	1,893151	1,49E-05
SEPW1	1,892617	1,83E-08
UBN2	1,885754	1,59E-11
SLC22A5	1,885237	3,07E-07
DHODH	1,884965	1,22E-10
RASA1	1,883772	2,11E-09
KIAA1549	1,881756	4,74E-09
SFXN2	1,880868	2,08E-07
HOXC13	1,880363	2,89E-05
GSTK1	1,879914	3,30E-08
PIK3R2	1,8799	5,09E-10
GNB2	1,879444	4,60E-20
MKS1	1,877969	1,74E-08
UNKL	1,876338	1,36E-09
THOC7	1,875239	5,26E-21
TBC1D5	1,874357	9,30E-13
PAQR5	1,873603	6,19E-05
PIEZO1	1,87308	4,32E-13
ACP6	1,872481	4,71E-07
RNASEH2A	1,870628	5,52E-20
TES	1,870369	4,41E-08
FAM149B1	1,868862	3,62E-06
UHRF1BP1	1,868771	1,88E-09
RBM48	1,868029	5,04E-05
ATXN3	1,865296	7,76E-07
AADAT	1,86182	1,37E-07
NADK2	1,85876	1,24E-11
WIBG	1,856223	4,09E-13
MYO6	1,854099	3,02E-20
USP6NL	1,853609	5,13E-12
TTC19	1,85162	7,04E-12
PPCDC	1,851452	2,30E-07
C20orf27	1,849498	1,64E-20
CYB5D2	1,849045	2,92E-08
TRAPPC2L	1,847447	3,37E-15
SPATA33	1,846886	1,96E-09
SPIRE2	1,845057	0,000265
RBM38	1,842843	2,12E-14

HMHA1	1,837955	7,94E-06
ZNF572	1,835975	0,000659
RPS3	1,835934	1,68E-15
LINC01521	1,834249	0,000685
IFI30	1,833452	1,94E-08
ZFP69B	1,83134	4,63E-05
ZNF552	1,82973	0,000907
SETMAR	1,828047	1,86E-10
N4BP2	1,827022	3,87E-06
ZNF37BP	1,825624	8,89E-05
PRSS23	1,823838	6,95E-09
FAM222A	1,823275	7,72E-12
LINC01004	1,817787	0,000739
DANCR	1,817711	4,00E-10
PVRL1	1,817504	8,65E-07
NFXL1	1,815723	7,76E-05
CHCHD10	1,814073	4,17E-19
CBLL1	1,813919	8,57E-13
PXYLP1	1,81364	0,000195
SNHG25	1,809447	2,44E-05
SUMF1	1,808913	2,50E-07
MCM7	1,807716	1,33E-16
FAM134B	1,807421	0,000856
MRPL11	1,806468	1,49E-20
ZNF138	1,8051	6,20E-10
SLC50A1	1,803743	1,20E-14
GLUL	1,803293	2,27E-18
AXIN2	1,802006	7,62E-19
TRIAP1	1,8017	1,33E-10
PATZ1	1,800782	1,89E-10
MZT2A	1,799285	3,68E-07
SFT2D3	1,798247	4,51E-06
MAN2A2	1,796217	9,47E-06
LYSMD2	1,796023	1,04E-09
ACTR6	1,795106	6,88E-11
PPP1R16A	1,795033	4,08E-09
PEX10	1,789174	1,44E-13
ERMP1	1,788889	2,49E-07
ATP5S	1,78787	0,000138
SLC25A15	1,786721	1,38E-08
EHMT2	1,785871	2,13E-24
WDR59	1,782655	4,99E-10
PECR	1,780711	4,75E-05
NFIA	1,780319	1,62E-06
GNPTAB	1,780076	1,95E-14
C14orf1	1,779046	4,56E-06
KIF27	1,777702	0,000414
ZRANB3	1,777016	2,34E-05
TET1	1,775689	0,000315
TLE3	1,772646	1,75E-21

SLC12A9	1,769796	6,06E-06
CCS	1,769106	0,00028
RCOR1	1,766899	1,81E-14
PPAP2A	1,764088	4,48E-07
ARPC1B	1,763175	5,10E-10
WDR83OS	1,760768	7,74E-19
ARHGEF3	1,760545	1,67E-08
TMEM243	1,759703	4,03E-05
ZFHX3	1,758663	4,54E-11
ZNF524	1,756572	7,16E-06
ZFAS1	1,756349	0,000978
TMEM170A	1,75631	9,39E-05
EMB	1,755324	7,13E-11
KLHDC2	1,754786	9,60E-08
WDR60	1,753781	6,82E-07
VTI1B	1,753633	7,25E-11
CRNDE	1,751787	0,000118
CASP6	1,751759	1,54E-08
PRKAR1B	1,750418	1,21E-16
ATMIN	1,750178	5,93E-16
ADD3	1,749647	4,19E-09
GPR153	1,746415	9,06E-06
LRRC75A-A	1,746218	1,05E-07
C12orf66	1,745607	0,000101
ZNF687	1,745124	3,16E-20
CRIP2	1,744888	3,30E-16
SRD5A3	1,744536	2,42E-09
HMGN1	1,74279	1,52E-20
COA5	1,740389	9,38E-10
PSMD5-AS1	1,738834	4,80E-05
GINS2	1,734278	8,93E-11
RSBN1	1,734077	1,90E-11
XPOT	1,733912	9,31E-12
ACACB	1,733896	0,000135
ATXN7L1	1,733583	9,85E-08
MLYCD	1,732661	2,63E-07
HIPK1	1,729947	4,30E-16
TMEM129	1,729452	2,18E-05
NDUFAF7	1,72832	9,73E-08
COG5	1,72825	2,28E-14
ZNF766	1,72766	1,22E-12
TCF25	1,72755	3,48E-15
DCLRE1B	1,727541	2,68E-10
MTHFR	1,725476	1,46E-05
ZNF624	1,724365	0,000976
POLR2J	1,723672	1,37E-11
TRNT1	1,723652	6,57E-05
GPHN	1,720603	5,31E-12
ISOC1	1,72057	3,78E-12
RNF187	1,720464	2,61E-08

FAM172A	1,715962	1,17E-07
PRICKLE2	1,715857	2,28E-05
C14orf2	1,714192	6,49E-18
ATP5D	1,711968	2,96E-18
SLC26A11	1,711233	0,000151
SLC2A4RG	1,7096	9,99E-15
ZNF485	1,704718	0,000194
ENDOG	1,703823	4,60E-09
PPM1A	1,703019	6,29E-07
TMEM37	1,702418	8,79E-06
TST	1,701228	0,000567
GRK6	1,700232	1,86E-19
HEATR5A	1,698098	6,57E-08
FAM189B	1,697484	8,85E-10
DTNB	1,696495	7,52E-09
SHROOM3	1,693848	2,41E-11
ATP5A1	1,693419	2,87E-23
PYCR1	1,692536	1,62E-11
KIAA0195	1,690288	7,87E-09
LOXL3	1,690267	0,000224
SPATA13	1,687815	1,55E-06
FERMT1	1,686332	8,03E-05
ISOC2	1,686328	2,76E-19
PTPRS	1,685469	1,74E-13
RPSAP58	1,685406	3,22E-15
SCML2	1,684653	0,0007
ACAA1	1,683916	3,42E-06
PUS7	1,683416	1,81E-10
SAYSD1	1,68158	5,51E-08
ENSA	1,681421	1,07E-22
MARCH8	1,681049	1,28E-05
ANK3	1,680761	6,55E-08
EIF2D	1,675928	4,62E-13
NBPF1	1,6743	1,53E-06
GTF3A	1,674053	1,07E-18
TSPAN33	1,67395	9,38E-05
MANEAL	1,673615	1,35E-09
CDC25B	1,673612	6,97E-14
PPP1R35	1,673349	2,36E-06
ICAM3	1,672661	0,000314
ATP5J2	1,671828	8,17E-10
ZNF816	1,671338	5,34E-05
TRPM7	1,669232	1,93E-19
DNAJC30	1,667505	2,39E-07
FUT8	1,667096	1,50E-14
TRIM4	1,666799	4,77E-07
LAMTOR2	1,665688	1,82E-12
NLN	1,665655	2,12E-14
SHTN1	1,665494	5,59E-15
ENDOV	1,664402	2,44E-06

CAMK2N2	1,663811	0,000651
FBXL19	1,663763	2,66E-13
ULK1	1,663334	5,13E-08
NOL3	1,661308	1,13E-07
TTC12	1,659495	0,000623
MPG	1,659007	3,42E-11
G6PC3	1,657362	1,83E-11
RBL2	1,653021	5,87E-09
EFCAB11	1,652874	9,10E-06
NIPAL2	1,651711	0,000437
ZNF14	1,650307	0,000154
ZNF277	1,648854	8,74E-07
LTBP3	1,648382	6,01E-08
FAM175A	1,647399	9,76E-08
TRAF4	1,646999	8,00E-13
PLXDC2	1,646834	1,51E-05
CCDC115	1,64499	7,70E-08
CTC1	1,644876	9,17E-08
ABHD17C	1,644094	0,000626
RPL22L1	1,643909	6,20E-07
CHST15	1,643672	2,01E-09
TMOD2	1,64313	4,44E-05
DCTPP1	1,642036	2,16E-13
ALDH3A2	1,640401	1,84E-17
RALGPS2	1,639781	6,46E-09
HMBS	1,638692	2,23E-12
VPS72	1,635462	2,98E-08
KNOP1	1,635214	1,86E-09
EZH1	1,635135	0,000769
ZSCAN2	1,635122	9,09E-09
ZNF720	1,635019	0,000123
IER5L	1,633869	3,39E-05
CALM1	1,632352	7,93E-10
LRRC1	1,632016	2,04E-08
PNMA1	1,630979	1,06E-10
CHCHD6	1,630505	8,04E-06
PAPSS1	1,629269	1,77E-05
GID8	1,627188	2,16E-17
BDH1	1,626953	2,44E-11
C14orf80	1,626784	1,08E-10
INPP4B	1,626274	1,92E-05
ZNF821	1,625996	7,19E-05
CHMP1B	1,623617	1,82E-11
GABBR1	1,623241	0,00079
GPX4	1,622008	5,29E-20
CDH24	1,621851	8,06E-09
RPL13	1,61737	8,67E-14
DPAGT1	1,615532	2,09E-12
MFSD9	1,615493	4,83E-05
NSD1	1,615293	6,24E-16

C12orf73	1,613635	0,000195
EBAG9	1,612681	1,91E-08
ERMAP	1,611004	0,000899
WDR34	1,61057	5,92E-17
MSRB1	1,609883	3,78E-06
ZFPM1	1,60938	3,72E-06
YEATS4	1,608328	6,96E-06
NDUFV1	1,606715	8,68E-14
SMPD2	1,606566	0,000497
SNAPC5	1,60576	2,53E-06
LIG1	1,605663	3,30E-13
SLC39A6	1,605592	7,10E-08
HDHD2	1,605339	3,49E-06
SOS2	1,604938	6,49E-07
CTAGE5	1,604838	0,000951
CDK2AP2	1,60342	6,78E-11
SETDB1	1,601843	1,30E-12
AKAP9	1,601383	6,22E-07
HEXDC	1,600995	0,000229
ADRBK2	1,60074	1,66E-05
BPHL	1,600008	1,50E-07
MAPK8IP1	1,595586	1,10E-08
ZFP30	1,587723	0,000235
ASMTL	1,587099	6,82E-06
RNF103	1,586805	9,15E-06
TMEM120/	1,585463	1,12E-06
SEC11C	1,585223	1,17E-06
RAB1B	1,584323	1,90E-06
ZNF91	1,584207	3,55E-09
BCAS2	1,584164	1,20E-16
IQGAP3	1,584156	0,000236
NPM3	1,583593	9,95E-08
SMAGP	1,582926	2,02E-09
ACSF3	1,581826	1,56E-10
LYRM1	1,58173	3,46E-06
MXD3	1,581474	8,57E-06
ZNF490	1,574291	0,000348
CHCHD4	1,574256	4,87E-06
PDCD2	1,573336	1,36E-14
ONECUT2	1,572192	3,78E-06
ANXA4	1,571865	1,03E-08
NARS2	1,570482	1,28E-07
PCDHB16	1,570138	0,000133
HSPA4L	1,568353	0,000522
ACTR3B	1,567716	1,56E-08
TMEM265	1,565851	9,68E-06
ZNF429	1,56551	2,94E-06
PBX2	1,562145	2,15E-13
PPFIA3	1,561481	7,52E-11
SMDT1	1,55971	7,89E-05

ZNF764	1,558599	1,02E-05
CYB5A	1,556601	3,70E-06
EAPP	1,554809	3,91E-09
DGCR6L	1,551807	4,83E-11
PNPO	1,551323	2,57E-08
RPLP0	1,549083	3,69E-18
TCTN1	1,545753	2,86E-07
ANKEF1	1,544823	1,15E-07
GDE1	1,544799	4,89E-10
AHCY	1,543915	2,72E-10
PPP1R3D	1,543339	2,79E-05
EBPL	1,542646	0,000122
TMOD3	1,542362	9,50E-12
PFKFB2	1,542327	1,86E-06
THAP5	1,541725	2,06E-09
CUL9	1,540075	2,51E-07
METTL4	1,537601	7,37E-05
SQLE	1,53663	1,23E-12
MLST8	1,536595	5,50E-14
PCLO	1,536313	7,58E-06
FAM102A	1,535864	7,72E-12
RHOB	1,532373	2,99E-09
MAPK3	1,530696	1,18E-05
B3GNT2	1,529971	7,09E-05
ZNF616	1,528642	9,84E-09
TPBG	1,528623	7,80E-14
ILVBL	1,527468	4,14E-14
HR	1,527185	5,41E-09
AK4	1,526671	5,05E-08
RPS29	1,525823	1,38E-07
PCNXL3	1,525462	7,25E-11
CLNS1A	1,524606	4,57E-15
ECHS1	1,524305	1,89E-12
ZNF627	1,523422	5,37E-09
CIRBP	1,523411	6,72E-06
SOX12	1,522995	2,09E-15
ZBTB25	1,521851	0,000474
PCYOX1	1,521459	5,25E-08
RABIF	1,521343	8,39E-08
FAR2	1,520309	3,58E-07
ADCY3	1,519559	7,73E-10
ZNF675	1,516391	2,06E-06
SIKE1	1,516336	1,34E-13
BCL7C	1,515885	1,53E-10
PSEN1	1,51418	5,55E-11
LMTK2	1,508559	2,15E-11
MAP3K11	1,506943	6,33E-06
SLC22A23	1,505695	1,06E-12
PTPMT1	1,504631	1,71E-10
SLC25A1	1,504579	5,21E-08

ZNF205	1,504514	7,03E-09
CHD3	1,503458	1,37E-12
PPP1CA	1,499916	1,49E-18
NDUFB10	1,498652	1,56E-12
INTS3	1,49795	4,02E-10
INTS2	1,496846	1,24E-08
DNAAF2	1,495762	6,98E-10
PDE4DIP	1,494538	1,06E-05
ITGA2	1,494087	9,95E-05
PSME1	1,492963	7,28E-10
ZNF704	1,492468	2,78E-07
FRAT2	1,492144	0,000513
ELMOD2	1,489231	7,44E-08
VPS13A	1,488285	8,86E-08
EXOC6	1,487957	8,05E-06
ZNHIT1	1,487181	5,27E-05
SIMC1	1,484965	0,000306
ZNF84	1,484252	1,16E-08
RDH13	1,48391	2,17E-08
CCDC71L	1,483587	0,000769
ADRBK1	1,483144	4,62E-08
EPM2AIP1	1,480913	3,04E-05
C6orf120	1,480514	4,56E-07
ADAT1	1,47927	2,13E-11
NUBP2	1,478648	8,77E-12
ADI1	1,477876	1,41E-11
POLR1D	1,476078	1,86E-14
TLDC1	1,475403	3,29E-05
CCPG1	1,475025	6,71E-05
ANAPC16	1,472985	1,92E-11
ORC5	1,472818	4,09E-05
IMPACT	1,472815	4,64E-07
SYPL1	1,472575	7,44E-11
PCDHB14	1,471393	4,31E-05
CFAP36	1,469565	2,36E-06
BRWD1	1,469072	4,08E-09
PKMYT1	1,468509	4,71E-08
MDM4	1,466459	2,28E-05
HIP1R	1,465527	1,04E-07
ARSD	1,464476	0,000567
SCARB2	1,461615	3,08E-09
RABL2B	1,461185	0,000371
CNOT6	1,460618	2,39E-11
CCNJ	1,460076	2,50E-08
GLI4	1,458828	0,000759
CASKIN2	1,458788	2,82E-10
TMEM187	1,458302	0,000982
STRBP	1,457851	3,38E-09
FRS2	1,456657	5,76E-06
MED13L	1,455878	1,67E-07

MTMR14	1,45435	9,84E-06
PAXIP1-AS1	1,452012	0,00058
ZNF718	1,451624	3,46E-05
ZKSCAN5	1,45098	2,65E-07
MTA3	1,450309	7,78E-09
PIGQ	1,448367	7,80E-07
PTBP3	1,447328	7,39E-09
CPT1A	1,447157	3,56E-13
OS9	1,443966	1,70E-05
C4orf3	1,443024	5,33E-05
JUNB	1,442203	0,000198
MTIF3	1,441651	8,31E-07
FAM120B	1,441401	1,18E-06
SOS1	1,440184	2,74E-09
DTD2	1,438529	0,000387
TMEM241	1,438004	5,00E-07
DMXL2	1,437112	8,99E-11
VPS4B	1,43643	1,28E-09
TMEM186	1,436155	3,11E-05
ZNF703	1,435863	9,62E-13
SETD6	1,435755	6,41E-05
TRIM59	1,435709	7,66E-05
PP7080	1,43563	9,47E-06
NUDCD2	1,434983	1,50E-07
AKT1	1,434803	6,66E-08
EIF4EBP2	1,433774	9,05E-06
CABLES2	1,432036	9,99E-07
HAUS1	1,430817	4,08E-06
AP4E1	1,430691	9,82E-08
COX18	1,429547	1,30E-05
MCCC2	1,428335	2,53E-08
COX6C	1,428309	1,23E-10
RETSAT	1,427911	3,08E-07
ATP13A2	1,426477	2,58E-06
OXSM	1,425818	0,000115
RPS25	1,425259	4,18E-10
GPD1L	1,425129	1,82E-05
ZNF480	1,42404	3,18E-06
ZNF286A	1,423224	2,82E-08
KDM5B	1,423219	2,92E-06
ESYT2	1,421289	6,55E-12
TGIF2	1,420834	7,28E-06
H3F3AP4	1,420488	2,06E-09
SMYD2	1,420216	0,000322
FAM86C1	1,420025	1,43E-06
VPS35	1,418895	1,27E-13
ADCK1	1,418661	7,61E-05
DBN1	1,418411	1,12E-15
UNG	1,416136	3,08E-10
ZDHHC12	1,416053	3,78E-06

LOC90784	1,412956	1,00E-07
REL	1,412032	8,33E-05
PCMTD2	1,41152	0,000787
KIAA1522	1,411177	2,17E-13
APOA1BP	1,41102	1,73E-09
C22orf29	1,409395	0,000173
BRK1	1,408909	2,92E-11
MIPEP	1,408843	8,57E-08
MZT2B	1,408597	2,76E-08
NFIB	1,406256	1,30E-06
SUV420H1	1,404569	6,38E-11
RPLP1	1,404294	5,45E-06
COX14	1,403056	1,43E-06
TRMT10A	1,402448	0,00094
ADGRL1	1,402058	4,08E-11
FAM45A	1,401654	3,35E-06
ACADSB	1,401202	3,47E-05
MLLT4	1,40021	3,17E-06
ZNF710	1,400147	6,75E-08
KCTD3	1,399346	1,21E-11
YIF1A	1,399211	3,74E-12
NAMPT	1,396604	8,12E-09
PRUNE	1,39609	1,01E-09
SRR	1,393544	0,000557
MFSD10	1,389426	4,71E-08
SYNJ2BP	1,38836	0,000195
CEP68	1,387581	8,03E-06
SIPA1L1	1,386683	2,79E-11
NPR3	1,386128	0,000368
FBXW8	1,385672	4,25E-08
HSCB	1,383644	2,35E-05
FAM110A	1,383427	0,000118
MXD4	1,383272	0,000929
MED28	1,382231	8,05E-08
UBAC1	1,381132	1,69E-11
S100A10	1,380711	2,49E-09
LRFN4	1,380081	2,55E-09
DHPS	1,379804	8,72E-08
SFXN4	1,379125	8,84E-08
SCARB1	1,377498	9,12E-07
GRN	1,376396	0,000113
HSD17B4	1,376273	1,93E-07
SRI	1,37416	8,90E-07
ZNF254	1,374146	2,34E-05
TNPO2	1,373216	5,15E-05
METTL8	1,372768	3,24E-05
LY6E	1,372409	2,93E-05
CTBP2	1,371843	3,07E-12
TM7SF3	1,371455	1,44E-06
TRMT112	1,368434	1,13E-08

ZNF394	1,368214	0,000645
NSMCE4A	1,367228	3,41E-06
STX6	1,366745	1,51E-10
WDR54	1,366023	4,77E-05
MAPK6	1,364969	1,66E-11
POLR1A	1,364301	1,30E-08
STX10	1,363146	2,98E-06
SGPL1	1,362901	2,33E-09
KREMEN1	1,362842	0,000204
VIPAS39	1,362097	1,71E-07
TCF3	1,360946	5,26E-13
ANKRD50	1,360407	1,33E-06
ABHD14B	1,360277	8,12E-06
RPL41	1,360262	4,01E-12
NEDD1	1,359517	1,28E-07
PARP16	1,358969	0,000132
PCCB	1,358779	3,81E-11
AGA	1,358565	0,000949
PLEKHA1	1,358066	0,000669
LIG3	1,357278	2,98E-09
NAPG	1,356531	1,25E-05
ARPP19	1,35592	1,46E-13
GABPB1	1,355614	8,22E-07
VPS50	1,35335	0,000229
CHEK2	1,352619	9,92E-05
CCDC53	1,350969	0,000546
KIAA1671	1,349825	2,34E-05
CCNI	1,348452	1,77E-08
LEF1	1,347016	5,29E-06
RAB11FIP3	1,345601	6,19E-05
ESCO1	1,345598	4,66E-07
EXOC4	1,343408	4,35E-08
AHR	1,342473	6,09E-10
FAM120AC	1,340297	3,23E-06
PIP4K2C	1,340205	1,92E-05
POLR2G	1,340152	1,11E-10
RPAIN	1,336928	1,65E-08
TMEM64	1,336395	2,47E-10
SAC3D1	1,335956	6,02E-07
TAB3	1,335508	0,000101
KRCC1	1,335024	8,66E-06
FLYWCH2	1,334891	9,85E-07
LPCAT3	1,332333	0,000121
NDUFB1	1,32956	4,19E-07
NDUFS8	1,329363	8,40E-11
MRM1	1,328454	0,000449
MIS18BP1	1,328224	0,000116
TMEM251	1,32697	5,50E-07
KDM6B	1,326799	1,59E-08
CENPH	1,326506	0,000394

ASB13	1,325568	2,50E-06
TCAF1	1,325269	5,50E-06
KLHL36	1,32288	1,31E-08
HIGD2A	1,321822	5,89E-13
PAFAH1B3	1,320679	7,66E-10
PPAP2C	1,320635	9,67E-06
PSRC1	1,319806	2,53E-05
ISCU	1,319281	1,67E-07
DHX40	1,319265	1,48E-09
CCDC90B	1,318993	4,77E-05
BBS10	1,318799	0,000447
MYD88	1,3185	1,60E-05
MSH3	1,316345	7,33E-06
DNMT3A	1,315263	1,10E-08
ACVR1B	1,314888	6,79E-07
NLRX1	1,314639	6,28E-05
DDX6	1,313418	1,25E-07
LINC01184	1,3112	6,13E-05
MSMO1	1,307317	0,000779
HDHD3	1,30702	2,20E-05
CIPC	1,305592	5,41E-05
CTDSPL	1,305335	2,00E-06
POLR2J3	1,305099	0,00099
GABPB2	1,304988	0,000186
MSL3P1	1,304961	0,000311
BCL7A	1,304773	1,62E-07
LINC00938	1,303275	0,000963
LEO1	1,299512	1,36E-11
CHMP4A	1,297951	7,27E-06
PEX26	1,296685	1,05E-08
C19orf60	1,295582	1,68E-06
GTF2I	1,295255	1,57E-11
FBXL15	1,295053	0,000164
FAM133B	1,294892	0,000108
PICK1	1,294728	6,77E-05
PANK1	1,293338	1,26E-05
COX20	1,292111	6,70E-06
BLVRB	1,29186	0,000742
RNF169	1,289095	1,16E-05
PLXNA3	1,288459	0,000268
ZMYND11	1,288326	8,33E-06
PIGV	1,288209	0,000594
RPS15A	1,287591	2,06E-08
ERP29	1,287022	1,34E-06
SIVA1	1,286808	4,38E-07
IGSF3	1,286523	0,000466
PTRHD1	1,28582	5,14E-05
COX5B	1,285643	1,92E-10
TRIM41	1,285045	2,52E-06
MVK	1,283493	0,000464

PHKA2	1,282427	3,17E-05
USP8	1,282385	5,79E-12
CAT	1,28111	1,05E-07
ATP5G2	1,280033	2,95E-11
DEPDC1B	1,279336	9,88E-06
COL18A1	1,278955	6,74E-09
AFTPH	1,278944	1,43E-07
SRPK2	1,278799	4,42E-10
RPS3A	1,278333	2,44E-05
NDUFA3	1,277125	1,38E-06
TARS2	1,276639	1,45E-06
FMNL2	1,27526	4,70E-06
RHBDD2	1,274024	5,08E-07
TMEM161A	1,27358	8,54E-05
NBPF3	1,271929	0,000498
ALDH16A1	1,271799	2,14E-06
PRDM4	1,271686	3,14E-12
BCKDK	1,271599	3,43E-08
CCDC85B	1,270974	3,29E-10
RAB40C	1,270699	4,49E-05
ZNF768	1,269273	1,10E-08
PAWR	1,269038	0,000354
GSKIP	1,268565	4,76E-05
TRIM56	1,268552	0,000126
SELO	1,268534	8,58E-06
MDH2	1,268188	3,31E-09
C11orf73	1,267401	3,44E-06
COX7C	1,267023	2,82E-12
YIPF2	1,266281	3,55E-07
DCP2	1,26421	5,76E-09
NOA1	1,261262	3,81E-05
TTC39C	1,260415	4,51E-05
YDJC	1,260406	1,67E-06
CARM1	1,259114	1,28E-09
ARF3	1,255603	1,56E-08
SMUG1	1,253212	7,75E-05
TTC8	1,252787	4,41E-05
TAMM41	1,252624	3,78E-06
SEH1L	1,251557	2,12E-07
CTNNBIP1	1,24822	1,09E-05
KIAA0100	1,247515	4,07E-05
ATXN1L	1,247326	5,32E-09
COX4I1	1,244938	1,31E-11
TRMT5	1,244457	7,57E-08
ARL8A	1,241869	8,32E-09
NLK	1,240583	6,17E-05
GPATCH2	1,240273	4,75E-07
SLC25A13	1,239923	4,80E-06
TMEM160	1,23824	0,00091
BANF1	1,238064	6,46E-08

TMED3	1,234651	8,69E-07
FASN	1,233407	0,000561
NAGLU	1,23316	4,25E-05
PDCL3	1,232179	3,33E-06
CMTM8	1,230932	0,000475
MAP2K5	1,230211	7,47E-06
MAZ	1,229903	9,19E-10
SEL1L	1,228654	3,88E-11
PSMD7	1,228027	1,27E-11
UQCRC2	1,227683	8,01E-12
ARL2	1,227584	0,000139
ANAPC1	1,22668	4,03E-09
CHRNA5	1,226287	1,29E-06
CNNM4	1,220082	6,47E-06
TOB1	1,21928	6,48E-05
MKL2	1,218097	4,25E-06
GNAS	1,21703	1,61E-11
MRPS33	1,216835	8,85E-07
BCL2L11	1,216296	2,33E-05
KAT2A	1,215664	3,16E-09
NRAS	1,214509	8,06E-07
PPA2	1,213836	2,12E-06
NIT2	1,21322	0,000152
RPIA	1,211951	1,35E-05
TMEM260	1,210796	0,000981
RPS2	1,210586	1,17E-13
NDUFB7	1,210264	5,70E-10
YES1	1,208687	2,66E-06
POGZ	1,207278	2,62E-05
ARHGAP39	1,20722	0,000349
ITPKB	1,206918	0,00062
NDST2	1,205224	0,000567
RASSF7	1,20451	0,000313
ZBTB41	1,199786	0,000231
PTPN18	1,198427	7,59E-08
ZNF83	1,197672	0,000157
HADH	1,197671	0,000143
ABCB6	1,19658	0,00061
NBN	1,195848	7,26E-09
ZMIZ1	1,19577	1,88E-05
PAAF1	1,195536	1,22E-06
NR2F2	1,191998	1,49E-06
EFTUD1	1,188514	2,65E-05
AFG3L2	1,188442	5,17E-10
FAM207A	1,187486	1,18E-07
PRKRIR	1,186497	6,31E-08
HMGA1	1,18581	5,97E-08
ARFRP1	1,185502	2,34E-05
LRSAM1	1,183968	0,000534
ZNF107	1,183475	1,28E-06

WDR48	1,182382	0,000702
IFT43	1,182074	6,97E-05
KDM2A	1,181242	2,99E-08
TRRAP	1,18067	3,03E-10
ATP11C	1,17982	2,45E-05
GCC1	1,17953	1,91E-07
SLC19A1	1,179092	2,43E-08
C12orf57	1,17871	0,000181
DDX41	1,178672	9,78E-10
ZNHIT3	1,177838	0,000109
PANK3	1,177112	3,96E-05
PIGM	1,175116	0,000358
SETD5	1,17488	1,23E-05
SREBF2	1,174314	3,18E-09
HMGB2	1,173745	0,00062
ZNF444	1,172698	2,44E-06
HMGCS1	1,171385	0,000451
TMED10	1,171221	1,50E-07
GNPNAT1	1,169639	7,45E-07
LPCAT1	1,168685	3,72E-06
TSEN54	1,16861	7,54E-05
BAG5	1,168262	1,88E-06
KAT8	1,167139	0,000563
TMEM97	1,166472	9,30E-08
TMEM106(	1,165785	1,32E-08
KLF5	1,165713	3,04E-06
ZNF680	1,158871	0,000651
PSPC1	1,157804	4,64E-06
LLGL1	1,157745	0,000265
LRCH4	1,156144	1,97E-06
FAM89B	1,156115	3,64E-06
PROSER1	1,154066	1,36E-09
STX17	1,152959	4,21E-05
NDUFC1	1,15289	8,23E-05
MED21	1,151209	3,98E-05
EMP2	1,150063	0,000375
RPL32	1,149064	3,37E-07
EZH2	1,148959	0,000319
RINT1	1,147859	0,000118
DGKZ	1,147452	1,20E-06
SMCHD1	1,145716	2,76E-06
CRBN	1,145158	0,000223
MIB1	1,143133	9,60E-06
RBM4	1,142697	5,99E-07
POP7	1,14261	0,000289
RBM8A	1,138522	6,49E-09
FNBP1L	1,137775	1,05E-06
ZNF160	1,136983	0,000103
ZNF213	1,134405	9,02E-06
BRF1	1,132477	0,000763

COX6A1	1,132275	5,87E-10
NUP107	1,131013	1,56E-05
SETD1B	1,130488	5,76E-07
NPRL3	1,130262	2,40E-08
OXNAD1	1,129799	0,000546
JTB	1,128527	1,56E-07
ACSL3	1,128116	0,00066
PRR7	1,127059	9,12E-05
C11orf30	1,126697	2,73E-05
CENPBD1	1,125591	9,80E-05
LTA4H	1,125326	4,87E-06
SPIDR	1,123675	2,78E-06
IGF1R	1,123396	4,36E-07
MLLT6	1,123267	8,57E-07
LDB1	1,123077	1,06E-06
MRPL57	1,122237	8,76E-08
EEF1G	1,120717	5,27E-11
KARS	1,120598	1,37E-07
BRD7	1,119594	3,70E-07
USP54	1,119239	2,16E-05
ZNF689	1,117082	0,000155
AP5S1	1,116507	2,97E-05
TRAPPC4	1,113173	3,96E-05
PHPT1	1,113136	2,18E-07
IRX3	1,112743	4,98E-06
SPG21	1,112516	4,95E-08
PPP1R26	1,112499	0,000332
RPRD1A	1,110543	2,63E-05
HSDL1	1,108184	0,000269
AEN	1,1079	1,46E-08
PDAP1	1,107832	5,51E-08
RPS6KB2	1,107646	3,49E-05
PRDM15	1,107356	0,000208
C7orf73	1,104022	1,87E-07
SLC44A2	1,103166	3,37E-06
RNF149	1,102819	4,61E-06
TXLNG	1,102365	0,000161
CMTR2	1,101752	3,01E-05
C16orf58	1,101658	1,84E-08
EEF2K	1,101624	2,60E-06
HMGB3	1,101622	8,19E-10
RGL2	1,101375	4,79E-05
CBX5	1,101168	5,00E-06
METTL9	1,098987	7,50E-07
ZFP36L2	1,098478	0,000945
FANCD2	1,097068	0,000196
C9orf114	1,096502	2,65E-05
GLTSCR1L	1,096394	4,19E-05
DICER1	1,091426	0,000277
ZNF592	1,091135	4,54E-06

ZNF518A	1,088821	0,000317
VPS52	1,086363	6,31E-08
MLH3	1,085281	0,000247
TBC1D9B	1,083073	4,05E-08
RAD23A	1,082126	1,88E-07
RBCK1	1,081933	2,26E-07
NUMA1	1,080998	2,23E-07
EEF1B2	1,080138	2,73E-05
TBL2	1,079487	2,55E-06
SSSCA1	1,078989	0,000648
VPS51	1,077312	1,21E-06
SLC9A3R2	1,076564	1,20E-05
UBQLN4	1,076159	1,68E-07
KIAA1109	1,07615	2,82E-05
EIF2B2	1,075791	2,17E-07
MTHFD1	1,074766	2,85E-10
RFX7	1,074153	7,47E-05
ADGRG1	1,073976	1,86E-06
TMEM258	1,073789	7,61E-05
PPP4R3A	1,073723	2,30E-07
BNIP3	1,073357	2,71E-06
STRA13	1,072092	6,83E-07
NAA30	1,069888	1,75E-05
LUC7L2	1,068389	0,000809
RPRD2	1,067892	7,04E-07
CBLB	1,067084	0,00025
DHRS7	1,06575	1,01E-05
PEX13	1,064849	0,000217
GOT1	1,064783	8,50E-07
SMIM20	1,063712	0,000486
TATDN1	1,063499	1,07E-05
NEO1	1,062883	1,18E-06
LSS	1,06196	0,000408
DBI	1,061353	9,83E-08
TSFM	1,061244	4,10E-06
PEBP1	1,060848	1,34E-08
CLN6	1,05914	6,54E-08
USP34	1,059055	2,05E-08
MLH1	1,058634	3,78E-06
SRSF8	1,058024	0,000448
APH1A	1,056461	7,58E-09
ATP5L	1,055816	1,21E-07
TOP1MT	1,055697	9,00E-08
GLRX5	1,055507	7,79E-05
TEX264	1,055287	3,47E-05
RBFA	1,053769	0,000118
DNPEP	1,052996	4,68E-07
PYCRL	1,05122	8,46E-05
MED13	1,050917	3,70E-08
EIF4B	1,050278	5,28E-10

VPS25	1,050161	1,73E-06
SGSM2	1,049582	0,000562
PHKG2	1,049461	0,000222
TEAD3	1,049099	0,000445
BUD31	1,048871	1,09E-07
KBTBD6	1,047985	0,000244
CAMKK2	1,043704	2,06E-06
BRD3	1,043665	1,86E-05
NUCKS1	1,042526	1,61E-07
ZNF32	1,039959	0,000244
HECTD4	1,0396	0,000111
RMDN1	1,038781	2,60E-05
SLC38A1	1,036229	1,55E-07
RFNG	1,036015	0,000884
TAF6	1,034226	9,20E-07
PSMB1	1,03396	5,40E-06
DAG1	1,033374	6,69E-05
FKBP5	1,033039	0,000221
NETO2	1,032448	4,09E-06
SLC29A1	1,031361	3,75E-05
RPL31	1,029152	8,20E-06
RTN3	1,028056	5,30E-06
PACS1	1,026227	1,11E-05
NDUFB2	1,025784	1,01E-06
COPS6	1,025062	7,88E-08
IDE	1,024741	2,34E-05
FAM111B	1,024129	0,000497
TTLL5	1,022598	3,68E-05
RHBDF2	1,021422	0,000172
ZNF629	1,021301	4,37E-06
AGAP2-AS1	1,018671	0,000284
TUBGCP2	1,016625	3,60E-06
ZNF252P	1,014933	2,98E-06
SHFM1	1,0144	8,89E-07
PTMS	1,014124	9,62E-05
ANAPC15	1,013963	1,14E-05
TOR3A	1,013155	9,29E-06
SAMD1	1,01256	4,91E-05
ASCC1	1,011985	2,97E-05
ALDH7A1	1,011626	2,22E-06
MICU2	1,010552	3,44E-05
TP53I13	1,009589	0,000876
HMGCR	1,008632	9,69E-06
MON1B	1,005775	3,98E-05
SP1	1,004844	1,77E-06
PAXIP1	1,004021	0,000157
MIF	1,003984	4,95E-07
GATC	1,002518	6,24E-05
WBSCR22	1,002485	8,04E-07
KMT2E	1,002206	1,97E-05

PHB2	1,001821	8,79E-08
TRAPPC13	1,001748	0,00057
C5orf30	0,999577	0,000139
FAM136A	0,998542	5,86E-07
PFDN5	0,996741	5,60E-07
IREB2	0,995943	0,000116
TPT1	0,994465	5,56E-05
POR	0,993755	2,42E-05
PPM1B	0,992503	0,000681
TRIP6	0,9923	5,68E-06
PCNX	0,991025	2,75E-05
ZNF282	0,991009	2,67E-06
PPIL2	0,990156	4,91E-05
ARID2	0,986893	0,000459
METTL2A	0,986141	8,19E-05
ZNF358	0,984665	0,000105
TIMELESS	0,984533	3,53E-05
FOXRED2	0,984423	0,000149
ARMT1	0,984301	4,25E-05
SMURF1	0,983237	8,26E-06
OTUB1	0,980276	2,73E-06
TADA3	0,978652	1,04E-06
GIPC1	0,977224	0,000599
RNF115	0,976446	6,37E-05
RPL36AL	0,974743	7,84E-05
ECSIT	0,974272	3,19E-05
MRPL41	0,972891	1,80E-05
RPL37	0,972885	0,000259
MED20	0,970206	0,000277
PMVK	0,969769	0,000376
FBXO21	0,966976	0,000336
MTMR12	0,966476	0,000622
MTMR3	0,966009	0,000249
TRAK2	0,965676	0,000177
PCF11	0,965644	0,000546
SERPINB6	0,960136	4,95E-07
IFT46	0,960054	3,30E-05
ABHD16A	0,957914	0,000335
POLR1B	0,955994	4,13E-05
HSPB1	0,955718	0,000203
TOP2B	0,955612	8,18E-07
SMARCA4	0,955064	0,000195
ATP5I	0,954863	1,48E-06
GPR137	0,94989	0,000525
CELSR1	0,947332	0,000785
RNF181	0,947309	0,000141
TELO2	0,947038	3,03E-05
BCKDHA	0,94577	0,000222
SS18	0,943029	1,18E-05
FGFR1OP	0,942938	0,000555

FBXL14	0,94258	0,000875
SLC3A2	0,941889	5,68E-07
LMNB1	0,941614	1,72E-05
MTMR4	0,941188	4,87E-05
LTBP1	0,941018	7,69E-06
DNAJA2	0,940069	0,000973
ZBTB1	0,939398	8,62E-06
POLR2I	0,93912	8,72E-05
AGPAT3	0,935995	0,000185
ARPC1A	0,935697	0,000133
VPS37C	0,932631	0,000282
CFDP1	0,931638	2,94E-05
MBTPS1	0,930695	1,82E-06
AAAS	0,930616	1,28E-05
PRPSAP1	0,930435	9,92E-05
ORC2	0,929908	0,000732
MRPL49	0,929882	4,06E-05
FAM127B	0,927955	3,38E-06
NELFCD	0,927428	6,11E-05
KAT5	0,926862	0,00013
PSMD9	0,926124	9,94E-05
IQCE	0,926089	2,32E-05
ESD	0,924728	8,72E-07
JAGN1	0,924704	8,30E-05
TSC2	0,924692	0,000173
ZFP41	0,924189	0,000418
SSNA1	0,9241	9,72E-05
IMMT	0,917551	1,15E-06
EPB41L1	0,914588	0,000472
MRPL21	0,912379	2,13E-05
RUSC1	0,911025	0,000237
MRPL16	0,910663	0,000641
TIMM10B	0,910281	0,000155
CNOT11	0,910182	1,28E-05
CANX	0,909323	3,24E-06
MRPL35	0,908403	6,01E-05
BTG1	0,908324	0,000232
IMP3	0,906248	1,13E-05
TRAF7	0,905173	2,09E-06
KIAA0196	0,905044	1,83E-05
CHD1L	0,904507	0,000386
LAGE3	0,903135	0,000125
ZNF24	0,902879	0,000192
HIP1	0,902595	0,000164
LRRC45	0,902299	0,000894
PRKAG1	0,901408	0,000229
SMARCC1	0,898987	2,80E-06
DIRAS1	0,898094	0,000925
DDB1	0,897938	0,000447
TUG1	0,89793	7,42E-06

PGP	0,897834	0,000161
TCEB2	0,896393	0,00037
PIGU	0,896053	0,000286
SEPHS2	0,894707	1,47E-05
DNAJC2	0,893511	0,000116
MYBL2	0,890702	6,32E-07
NUDT16L1	0,890466	0,000466
HNRNPF	0,888554	9,39E-06
CAMSAP1	0,887987	2,68E-05
SEPHS1	0,887025	0,000761
C19orf43	0,88638	4,36E-07
ZNF618	0,885956	5,08E-06
PGLS	0,885387	0,000348
CCZ1B	0,882088	0,000188
CFLAR	0,879666	0,000618
NDUFB9	0,879323	0,000939
ATP5H	0,875075	0,000417
ATRAID	0,873051	2,15E-05
RPS23	0,868589	0,000612
SPAG7	0,864686	0,000644
ZNF398	0,863334	8,16E-05
GGCX	0,861674	0,000158
ESYT1	0,85753	1,90E-06
PMPCB	0,857222	9,16E-05
MRGBP	0,856449	0,000349
MRPS21	0,855905	8,33E-06
LAMTOR1	0,855674	0,000823
CNOT2	0,855237	2,26E-05
GIT1	0,854883	2,98E-05
DNAJC10	0,853824	0,000389
CDC42SE2	0,853447	0,000212
DECR1	0,852164	0,000271
UBXN4	0,851857	1,90E-05
SYAP1	0,851815	0,000238
CDC42BPB	0,850083	1,27E-05
CTCF	0,849429	0,00023
COX8A	0,848171	1,34E-05
DLST	0,840485	0,000137
RNMTL1	0,837469	0,000816
PTPN2	0,837246	0,000295
RUFY1	0,834904	0,0007
ANXA7	0,834265	1,98E-05
SORD	0,834089	1,80E-05
TSTA3	0,833885	5,26E-05
RPL34	0,8336	0,000162
RPSA	0,831109	8,05E-07
ARFGAP2	0,829647	0,000223
TAF11	0,827676	0,000649
FKBP3	0,827524	8,42E-05
CASP2	0,825571	0,000185

SUDS3	0,825054	0,000514
ADK	0,820405	0,000203
FAU	0,818349	9,29E-06
HSBP1	0,816899	2,84E-05
MARCKSL1	0,814719	5,95E-06
DYNLL2	0,813319	2,94E-05
PRRC2B	0,811679	0,000393
ARL6IP1	0,811285	0,000114
CNOT8	0,811104	0,000308
TMEM167/	0,808604	0,000974
FNBP1	0,803591	0,000464
TK1	0,803222	4,10E-05
CUEDC2	0,801401	0,000218
MKKS	0,800866	0,000431
RFC2	0,80081	5,22E-05
MKRN1	0,800318	0,000536
RPL3	0,79624	0,000414
SRCAP	0,793808	8,46E-06
IMP4	0,78941	3,29E-05
KIAA1191	0,788241	0,000106
WDR61	0,785348	0,000456
DLG5	0,783641	5,98E-05
SNTB1	0,781619	0,000726
AP3B1	0,780533	0,000879
RPL14	0,77918	0,000162
MRPL4	0,775387	0,000144
GTPBP6	0,774347	0,0007
SPATS2	0,773552	0,000856
RABL6	0,773093	0,000296
KMT2D	0,771455	3,54E-05
LLPH	0,769458	0,000225
CAND1	0,764578	0,000292
MRPS34	0,76367	0,000185
NAA60	0,761237	0,000359
PPP1R14B	0,760681	0,000184
YIPF3	0,76062	0,000322
SMYD5	0,760582	0,000942
DCAF7	0,758469	0,000581
CFL1	0,758327	0,000162
SNRPE	0,755952	3,96E-05
C12orf10	0,739937	0,001003
NSUN5	0,73922	0,001
SIN3A	0,735559	0,000393
NDUFV3	0,734394	0,000432
SCAF11	0,730003	0,000531
FEM1B	0,728481	0,000593
NGRN	0,723681	0,000512
SAP130	0,714698	0,000242
ZMYND8	0,712124	0,000779
RPL8	0,708618	2,71E-05

FDPS	0,69666	0,000176
PSMC2	0,695901	0,00056
ATL3	0,693759	0,00076
TDG	0,692034	0,000735
HN1L	0,691199	0,000223
H3F3B	0,690924	0,000143
IST1	0,690065	0,000572
GDI2	0,689826	0,000833
CTNNA1	0,685507	0,000181
NDUFA13	0,680234	0,000629
LRPPRC	0,669189	0,000151
COX5A	0,667661	0,000847
CCNF	0,658456	0,001008
LAPTM4B	0,644746	0,000742
FBRS	0,641975	0,000534
TOP1	0,641314	0,000216
BPTF	0,639529	0,000439
PSMD4	0,637512	0,000361
EIF1AX	0,633959	0,000774
USP39	0,633232	0,000806
GNB2L1	0,63186	0,000478
HMGB1	0,619785	0,000389
PABPC1	0,605979	0,000403
CCT5	-0,61262	0,00056
AATF	-0,61427	0,000939
PSMD11	-0,62376	0,000798
ETF1	-0,63006	0,000694
TIMM50	-0,63618	0,000724
SRF	-0,64048	0,000836
SYNCRIP	-0,64329	0,000843
UBE2M	-0,64342	0,000867
C11orf58	-0,64462	0,000584
SAE1	-0,65015	0,000413
KXD1	-0,65023	0,000927
BOP1	-0,65236	0,000422
PPIA	-0,65698	0,000144
RRP36	-0,65988	0,000705
NCKAP1	-0,67624	0,000557
NDC1	-0,67766	0,000892
RPL11	-0,68088	8,19E-05
ZBTB4	-0,6876	0,000999
PAF1	-0,69443	0,000149
HSF1	-0,69548	8,83E-05
NUP93	-0,69981	0,000404
RRP7A	-0,69984	0,000752
HNRNPA2B	-0,7046	8,26E-05
PSMD13	-0,70629	0,000261
GMPS	-0,7073	0,00035
ASXL1	-0,71032	0,00025
STIP1	-0,71112	0,000437

HNRNPU	-0,71833	0,000991
SLC35E1	-0,7188	0,000792
IDH3A	-0,71897	0,000519
SEC63	-0,721	0,000115
ADIPOR2	-0,72358	0,000579
PSMD3	-0,73079	4,00E-05
PPP1R37	-0,73422	0,000624
MDN1	-0,73617	0,00037
PARP1	-0,73819	0,000181
MFAP3	-0,7412	0,000513
CTNNBL1	-0,74162	0,000233
EIF4G3	-0,74176	0,000409
MFSD12	-0,74901	0,000319
FSCN1	-0,74965	0,000406
TOMM40	-0,75182	1,84E-05
ARPC5L	-0,75272	0,000326
SAR1A	-0,75325	0,000223
C6orf89	-0,75466	0,000392
YTHDF2	-0,75521	7,48E-05
MAP4	-0,75789	4,95E-05
SLC31A1	-0,75803	0,000262
MAGED2	-0,75831	0,000365
RTN4	-0,75954	0,000402
PHRF1	-0,75984	7,92E-05
SNRNP70	-0,76055	0,000182
TFG	-0,76241	8,16E-05
DHX30	-0,76385	0,000177
RANGAP1	-0,76461	9,80E-05
DDX52	-0,76947	0,000995
SENP5	-0,7711	0,000172
COPS3	-0,77156	0,000228
SF3A1	-0,77527	0,00015
GAPVD1	-0,77707	0,00024
H2AFV	-0,77807	0,000429
BAK1	-0,78021	0,000153
EIF2S3	-0,78168	9,72E-06
PSMA7	-0,78293	1,21E-05
DOT1L	-0,78326	0,000618
BABAM1	-0,78608	0,000824
NPC1	-0,78775	0,000538
UQCC1	-0,78965	0,000999
GARS	-0,79127	4,11E-05
EEF1A1	-0,79127	1,81E-05
PAFAH1B1	-0,79159	2,01E-05
SEC62	-0,79207	7,21E-05
CD99	-0,7929	0,000899
POLRMT	-0,79328	0,00014
DTL	-0,7936	0,000797
RERE	-0,79451	5,43E-05
DHX37	-0,79518	3,43E-05

XPO7	-0,79853	0,000393
COX7A2	-0,80113	0,000708
KIF3B	-0,80124	0,000848
ENTPD6	-0,80216	0,000321
TPD52L2	-0,80257	4,60E-05
MTCH1	-0,80278	0,000121
CCAR2	-0,80289	2,27E-05
DHX36	-0,8033	0,000154
SMG9	-0,8035	0,000128
B4GALT1	-0,80719	2,50E-05
PRIM2	-0,80724	0,000758
FMR1	-0,8109	0,000964
MTDH	-0,81108	0,000286
NUP50	-0,81302	0,000478
TSSC4	-0,81656	0,000823
PDCD5	-0,81692	0,000156
USP48	-0,81754	0,000281
PSMB3	-0,81978	0,000173
SYF2	-0,82159	0,00073
TGS1	-0,82296	0,000247
COX7B	-0,82606	4,86E-05
PPIH	-0,82654	0,00034
NCAPH2	-0,82728	5,23E-05
MRPL34	-0,828	0,000222
RAB27A	-0,82892	0,000675
RANBP3	-0,82941	0,000213
SLC20A1	-0,82987	7,06E-05
RCN1	-0,83204	1,28E-05
CENPO	-0,83384	0,000567
ZNF106	-0,83465	2,80E-05
TANC1	-0,83628	0,000154
GTSE1	-0,83684	0,000619
SRP14	-0,83706	0,000185
RARS	-0,83781	0,000368
CHKA	-0,83948	0,000113
SENP6	-0,84055	0,000631
LEMD2	-0,84096	1,44E-05
GSK3A	-0,84228	2,61E-05
IFT57	-0,84648	0,000121
ATR	-0,84858	0,000164
DBR1	-0,84859	0,000581
SERBP1	-0,85005	0,000395
KBTBD2	-0,85176	5,28E-05
HOMER3	-0,8521	0,000168
TRIM32	-0,8522	0,001005
GTPBP1	-0,85383	0,000813
ATXN2	-0,85396	0,000217
DCAF10	-0,85411	0,000493
MAP2K1	-0,85474	2,66E-05
RCC1	-0,85491	1,39E-05

EIF3I	-0,85558	6,80E-05
RTF1	-0,85562	3,21E-05
DCUN1D5	-0,85634	0,000299
RRP9	-0,85669	6,81E-05
POLR2H	-0,85716	4,59E-05
GTF2H1	-0,85813	0,000221
AGPAT5	-0,85845	0,000285
EIF3M	-0,86032	1,22E-05
VEZF1	-0,86181	3,46E-05
RALA	-0,86192	0,000306
ERO1A	-0,86305	0,000165
NEDD4	-0,86363	0,000996
DDHD2	-0,86375	0,00057
NCSTN	-0,86411	0,000506
RSPRY1	-0,86712	0,000433
MRPL20	-0,86931	0,000309
MLLT1	-0,86996	1,51E-05
QRSL1	-0,8706	0,000597
SNRPB2	-0,87282	9,34E-06
GTF2F1	-0,87366	2,40E-05
PSMC4	-0,87381	7,33E-07
NCL	-0,87394	7,70E-07
TYMS	-0,87402	1,55E-05
TNRC6A	-0,87505	0,000317
STOML2	-0,87545	2,56E-06
ACKR3	-0,87701	1,54E-05
BCCIP	-0,8781	7,14E-06
RNF111	-0,88005	0,000634
FAM3C	-0,88089	0,000205
APIP	-0,88121	0,000963
DUSP3	-0,8813	8,33E-05
VBP1	-0,88138	2,80E-05
GDI1	-0,88209	2,72E-05
UMPS	-0,88238	9,02E-05
HIF1A	-0,88301	3,75E-05
SLC35F6	-0,88392	0,000739
PPIE	-0,88446	9,79E-05
ATG12	-0,88453	0,000289
TAB2	-0,88463	0,000286
PSMC3IP	-0,88576	0,000774
NUP62	-0,88665	7,12E-05
LINC01420	-0,88706	0,000522
TMED4	-0,88748	7,00E-05
COPS5	-0,88775	0,000596
UBAP2	-0,88811	0,000238
ZSWIM6	-0,88972	0,000693
ZZZ3	-0,88977	0,000508
NOC4L	-0,89036	5,84E-05
PELO	-0,89121	0,000215
TMEM2	-0,89201	0,000936

TBC1D20	-0,89324	0,00056
CARKD	-0,89426	0,000587
KPNA2	-0,89441	1,41E-05
ERP44	-0,8951	3,30E-05
PRPF4	-0,89622	6,24E-06
RAC1	-0,89669	1,53E-07
GLIS2	-0,897	0,000493
CUL1	-0,89761	1,40E-05
TMEM57	-0,89807	0,000754
PSMA1	-0,89903	5,16E-06
TOR1B	-0,90038	0,000283
KIF20B	-0,90203	3,90E-05
PROSC	-0,90241	0,000707
RNF4	-0,90285	7,14E-06
DVL1	-0,90315	7,02E-05
TUBA1C	-0,90577	1,85E-06
RPF2	-0,90748	0,000233
ACER3	-0,90794	0,000213
TBRG4	-0,90924	0,000306
GAK	-0,91028	1,38E-06
DESI1	-0,91035	2,64E-05
HEBP1	-0,91323	0,000122
NRD1	-0,91475	1,21E-05
FAM50A	-0,91484	6,25E-06
UBE2A	-0,91524	4,28E-06
EXOSC9	-0,91583	0,000342
CDCA8	-0,91589	0,000874
ZDHHC5	-0,91593	3,02E-06
TAF12	-0,91593	0,000192
VPS39	-0,9162	0,000788
GOLGA7	-0,91633	6,38E-05
USP12	-0,91763	0,000431
DLGAP4	-0,91856	2,16E-05
SGTA	-0,91868	9,93E-07
SRRM1	-0,91881	8,95E-05
GSR	-0,91986	0,000177
SSBP3	-0,9206	8,41E-05
CWC22	-0,92138	0,000166
HAUS8	-0,92151	0,000942
HGS	-0,92252	2,85E-06
XPO6	-0,92285	3,74E-07
ZNF644	-0,92311	3,92E-05
CCDC97	-0,92343	1,31E-05
KHSRP	-0,92466	3,28E-06
MCM8	-0,92507	0,000103
ATG16L1	-0,92603	0,000883
ITSN1	-0,92625	0,001003
TMEM222	-0,929	5,26E-05
STRAP	-0,93011	2,38E-05
UBQLN1	-0,93018	9,52E-07

NDEL1	-0,9316	0,000127
RBPMS	-0,93167	0,00012
SIK3	-0,93287	2,67E-05
TUBB6	-0,93746	0,000128
LRRC59	-0,93782	6,80E-06
DUSP14	-0,93791	7,50E-05
ANKRD27	-0,93865	7,07E-05
BET1L	-0,93988	0,00025
ZHX1	-0,94032	0,000799
ABCF1	-0,94142	0,000164
MRPL13	-0,94283	7,89E-05
PLEKHO1	-0,94307	9,11E-06
RAB3GAP2	-0,94429	2,96E-05
URGCP	-0,94448	0,000987
LSM10	-0,94494	0,00045
GMNN	-0,94582	3,42E-05
SNRPA1	-0,94633	1,18E-06
UBE2E3	-0,9476	1,39E-05
RYK	-0,94813	0,00021
UBN1	-0,94827	1,86E-06
NEK2	-0,9483	0,000569
SLC39A7	-0,94835	7,17E-05
PEF1	-0,94852	3,87E-06
RGS3	-0,94922	0,000802
RNGTT	-0,94954	0,000184
ZW10	-0,94969	0,000261
SF3B6	-0,95055	0,000508
PEAK1	-0,9506	0,000148
ZNF584	-0,95075	0,000602
PTDSS1	-0,9512	0,00063
C9orf3	-0,95134	0,000551
GPATCH4	-0,9516	1,78E-05
MOSPD1	-0,95243	0,000395
RPP30	-0,95278	4,82E-05
DDX17	-0,95368	7,62E-08
SMC5	-0,95768	0,000584
NUDCD3	-0,95769	2,66E-06
PLXNA1	-0,95822	2,64E-05
ARHGEF1	-0,959	2,14E-05
TMED7	-0,96136	1,09E-05
DDX47	-0,96328	3,05E-06
MTG2	-0,96386	8,19E-06
TIMM13	-0,96592	7,87E-06
RHOA	-0,96614	4,42E-07
MED27	-0,96714	0,000166
UBR3	-0,96741	0,000613
ORMDL1	-0,96753	2,18E-05
FBXL3	-0,96813	4,21E-05
CDCA2	-0,96879	0,000607
ANKRD40	-0,96966	0,000106

TRMT2A	-0,9739	2,72E-05
TXNDC12	-0,97467	3,53E-06
YBX1	-0,97539	0,000714
PARK7	-0,97557	2,98E-05
COASY	-0,97573	2,30E-06
HERC2	-0,97575	4,18E-07
KIDINS220	-0,97583	3,77E-07
C1orf174	-0,97707	0,000851
LACTB2	-0,97728	0,000981
MTMR9	-0,97748	0,00013
GNA11	-0,97768	3,25E-06
LPGAT1	-0,97786	0,000495
MUM1	-0,97823	0,00012
TRMT61B	-0,97973	0,000729
TAGLN2	-0,97988	1,89E-08
CNOT7	-0,98027	3,23E-06
ATAD3A	-0,98059	1,05E-07
PRKD3	-0,98074	2,98E-05
NDFIP2	-0,98113	0,000282
AUTS2	-0,98136	1,26E-05
BFAR	-0,98164	9,98E-06
B3GALNT1	-0,98245	0,000325
TBC1D13	-0,98247	0,000177
MAP1S	-0,98323	0,000857
PDIA5	-0,98419	0,000106
SPOP	-0,98454	0,000201
TTI2	-0,98472	0,000592
FRG1	-0,98581	0,0008
DYNLRB1	-0,98587	1,39E-05
NFIC	-0,98606	2,25E-07
TP53BP1	-0,98855	0,000102
ZMPSTE24	-0,98876	0,000239
GOSR1	-0,9895	2,63E-05
NUAK1	-0,98977	0,000984
NDUFA10	-0,99101	2,82E-07
URB1	-0,99329	3,10E-06
IFNGR1	-0,9935	0,000483
CUL4A	-0,99434	2,06E-05
PTPN1	-0,99501	1,96E-05
WDFY1	-0,99525	2,03E-05
VKORC1L1	-0,9953	8,29E-07
UBLCP1	-0,99545	9,81E-06
OPA3	-0,99562	2,46E-05
ENAH	-0,99609	0,000296
FH	-0,99726	6,98E-07
MCFD2	-0,99787	8,19E-07
BRI3	-0,99833	2,40E-06
TUSC1	-0,99846	0,000283
CHMP5	-0,99933	0,00025
SZRD1	-0,99935	5,11E-05

USE1	-1,00086	0,000731
FABP5	-1,00105	2,76E-06
GOLGA4	-1,00141	3,67E-05
ISCA1	-1,00153	1,09E-05
EXOSC2	-1,00226	1,15E-06
UBE2J1	-1,00327	7,96E-05
DNTTIP1	-1,00362	0,000111
PARP2	-1,00406	1,29E-05
HPCAL1	-1,00497	9,84E-06
TCP1	-1,00532	2,48E-07
FAH	-1,00604	1,33E-05
COPB1	-1,0062	1,95E-07
DSCR3	-1,0062	0,000199
FBXO46	-1,00732	0,000195
FTSJ1	-1,00846	3,05E-06
CHMP7	-1,00984	8,02E-07
AK2	-1,01003	2,11E-08
FBXL18	-1,01012	9,39E-06
FARP1	-1,01098	1,59E-05
ENDOD1	-1,01103	0,00014
DAGLB	-1,01269	4,83E-06
HMGXB4	-1,01382	6,08E-06
SACS	-1,016	5,69E-05
TM9SF2	-1,01746	0,000856
ZNF503	-1,01906	6,87E-07
TRMT6	-1,0194	2,01E-05
SLC35A2	-1,01976	0,000242
C19orf47	-1,02276	0,000166
CCDC93	-1,02418	0,000364
NCAPH	-1,02545	1,14E-07
UBE2R2	-1,02614	1,24E-07
SDF4	-1,0269	0,000641
TBX3	-1,02947	2,24E-06
NOCT	-1,02987	0,000774
MFN1	-1,0306	0,000283
UBIAD1	-1,0313	5,30E-05
IGBP1	-1,03247	0,000252
PBX3	-1,03258	0,000798
ATP6V1E1	-1,03318	5,79E-05
SUPT5H	-1,03378	3,18E-06
KDELR2	-1,03415	3,11E-05
PDLIM1	-1,03529	2,02E-07
CYCS	-1,03689	1,51E-07
RHOBTB3	-1,0373	5,51E-06
NDUFAF4	-1,04062	0,000129
RBM26	-1,04245	1,29E-05
SEC24A	-1,04255	5,20E-05
DFFA	-1,04259	4,07E-07
ATG5	-1,04389	1,53E-05
MZT1	-1,04523	0,000211

MPLKIP	-1,04655	0,000457
C4orf46	-1,04733	0,000866
ORC1	-1,04742	0,000103
PDHX	-1,05045	0,000126
RBM42	-1,05065	4,42E-07
CCNC	-1,05134	0,000618
UHRF1	-1,05169	0,000433
CSTF2	-1,05173	3,51E-07
SLBP	-1,05248	3,06E-06
FBXL5	-1,05359	0,000158
LRRC42	-1,05378	2,48E-06
HSPG2	-1,05384	0,000787
ARHGAP11	-1,0542	1,51E-05
FAM122A	-1,05526	0,000808
SMIM12	-1,05579	6,17E-06
PLS3	-1,05627	5,31E-06
YIPF5	-1,05711	1,91E-05
NOP10	-1,0582	1,42E-07
AIG1	-1,05897	5,79E-06
SLC4A1AP	-1,06019	5,30E-06
COPG1	-1,0602	1,57E-06
RPS6KC1	-1,06148	8,23E-05
TMEM41A	-1,06162	2,97E-06
PQLC1	-1,06205	3,25E-05
STK25	-1,06391	8,09E-07
ANKRD54	-1,06414	0,000111
QRICH1	-1,06416	2,23E-07
TEAD1	-1,06496	4,15E-06
RNF219	-1,06561	0,000138
RPAP1	-1,0657	9,78E-07
PSMD8	-1,06606	3,51E-09
RBMS1	-1,06618	9,61E-05
HSPA5	-1,06743	4,77E-10
TMEM70	-1,0681	1,73E-05
PPIL4	-1,07098	6,19E-06
NOSIP	-1,07207	4,05E-08
ITGB1BP1	-1,07359	1,89E-05
C9orf40	-1,07372	0,000427
ARHGAP17	-1,07429	3,45E-07
TOMM5	-1,07467	6,42E-07
IKBKG	-1,07572	2,17E-05
DCAF12	-1,07603	1,51E-06
EIF2B5	-1,07693	8,46E-06
RNF25	-1,07757	2,94E-06
HPS5	-1,0786	0,000898
FOXJ3	-1,07927	4,63E-07
ZNF292	-1,08096	1,18E-05
GNL3L	-1,0814	1,47E-06
RABL3	-1,08142	0,000158
POLR2C	-1,08201	2,95E-08

PFDN2	-1,08323	1,84E-06
RRM2	-1,08474	3,03E-08
YARS	-1,08484	1,78E-05
RAB34	-1,08554	6,41E-05
MGAT1	-1,08666	4,07E-05
PHF19	-1,08731	2,36E-06
ZCCHC17	-1,08863	5,05E-05
NOC2L	-1,0897	3,14E-06
NCDN	-1,0906	1,56E-07
CHAF1A	-1,09122	2,57E-06
CSNK1A1	-1,09142	2,21E-06
KLHL18	-1,09236	0,000191
QKI	-1,093	0,000952
TANK	-1,09409	9,17E-06
SEC61A1	-1,09457	0,00052
TBPL1	-1,09468	0,00032
RLF	-1,09598	3,83E-06
WDR45B	-1,09817	9,62E-05
RAB2A	-1,09985	1,84E-07
CTPS1	-1,10167	4,40E-07
PJA2	-1,10175	9,20E-06
SNX4	-1,10187	1,42E-06
ITCH	-1,10324	1,59E-08
ST6GALNA(	-1,10483	0,000733
TBCCD1	-1,10495	0,00037
SHC1	-1,10578	3,98E-09
SMG5	-1,10637	1,64E-09
NFX1	-1,10894	1,82E-08
TMEM263	-1,10991	4,10E-05
LMNB2	-1,11212	0,000168
SEP15	-1,11262	5,01E-08
PIGK	-1,11277	0,000473
MFHAS1	-1,1131	2,24E-06
USP24	-1,11512	1,87E-06
CPTP	-1,11526	1,53E-05
VDAC3	-1,11528	2,40E-06
AP1S2	-1,11566	0,000719
USP11	-1,11652	3,64E-07
FUCA2	-1,11678	0,000195
ANAPC13	-1,11688	0,000888
AP2M1	-1,1169	1,71E-05
ACTG1	-1,11743	0,000212
SNX18	-1,11881	0,000195
MARK4	-1,11934	0,000208
SIK2	-1,1211	0,000126
DTYMK	-1,12167	0,000165
GNB1	-1,12215	1,37E-10
CTSC	-1,12311	3,44E-07
TAF2	-1,12325	7,99E-08
SUPT7L	-1,12453	9,99E-07

PDIA3	-1,12505	1,16E-06
APOOL	-1,12528	1,05E-05
PSMD1	-1,12578	1,37E-07
OGFOD3	-1,12834	1,82E-06
RPN1	-1,12839	1,03E-06
TRIP12	-1,12938	0,000374
BLMH	-1,12952	3,82E-07
WDR62	-1,13066	2,09E-05
DYNC2H1	-1,13083	0,000428
CCT8	-1,13191	7,84E-11
SUGT1	-1,13388	6,93E-05
USP13	-1,13405	2,75E-07
VCP	-1,13745	4,90E-05
WBP11	-1,13788	1,74E-08
MTFR1L	-1,13791	1,24E-05
SP140L	-1,13797	2,96E-05
ACADM	-1,13833	9,94E-06
SAMD8	-1,13841	5,16E-05
TNKS	-1,13905	8,88E-07
COPS2	-1,1394	1,33E-06
ELOVL1	-1,13962	5,04E-05
GNL1	-1,14003	1,47E-08
FAM72B	-1,14216	0,000616
FAM57A	-1,14237	0,00037
UTP18	-1,14308	9,85E-08
EPS15L1	-1,14354	3,04E-06
ATP13A3	-1,14397	5,05E-10
COQ10B	-1,14496	0,000293
ELK1	-1,14535	6,51E-06
TNPO1	-1,14551	1,16E-08
TRAPPC3	-1,14608	2,34E-06
MAK16	-1,14629	0,000288
AGFG1	-1,14698	2,14E-06
MED15	-1,14763	8,79E-08
PSMA2	-1,14776	9,83E-10
FLNA	-1,14867	4,07E-09
ACTR3	-1,14906	6,55E-06
SNX3	-1,14914	1,07E-05
RNF126	-1,1493	5,51E-05
CCNE2	-1,14951	3,83E-05
RAB23	-1,14961	1,57E-05
FN3KRP	-1,14999	2,95E-07
SAMD4B	-1,15133	3,16E-05
MINOS1	-1,15167	0,000356
SERPINH1	-1,15196	4,25E-08
SERINC1	-1,1527	5,73E-05
NDUFAF3	-1,15443	5,28E-08
SLC33A1	-1,15449	1,69E-06
WWTR1	-1,15576	4,41E-08
FBXO30	-1,15642	0,00022

RBM18	-1,15691	3,78E-06
LTV1	-1,15736	0,000659
TMEM259	-1,15745	3,69E-08
ZBTB43	-1,15844	3,38E-06
DNAJC13	-1,15895	1,40E-05
GRAMD1A	-1,15995	8,25E-09
EIF2A	-1,16064	2,11E-06
LASP1	-1,16255	4,12E-05
UBE2W	-1,16442	3,84E-06
PLCB4	-1,16535	0,000122
CIC	-1,16554	3,76E-09
PPP1R8	-1,16558	9,62E-09
USP33	-1,16588	6,84E-06
PKNOX1	-1,16593	7,54E-06
SURF4	-1,16604	1,77E-06
PWP2	-1,16643	1,53E-06
SIN3B	-1,16666	1,78E-07
CDK17	-1,16698	0,000606
MFAP1	-1,16803	4,92E-05
HPS3	-1,16814	1,27E-06
ACTB	-1,16818	2,15E-05
TFDP2	-1,1682	3,69E-07
DPP9	-1,16832	7,19E-07
RND3	-1,16854	5,07E-05
FKBP15	-1,16907	1,00E-07
SUN1	-1,16916	1,54E-09
DHDDS	-1,16959	0,001001
PDLIM7	-1,17054	2,21E-06
DNAJC8	-1,17099	1,92E-08
EIF3D	-1,17107	4,61E-08
DCTN6	-1,17372	1,79E-06
PRPS2	-1,175	3,48E-05
ALYREF	-1,17543	2,00E-10
LPP	-1,17649	2,62E-06
DNAJC16	-1,17748	5,05E-05
AKIRIN2	-1,17816	1,53E-05
DMWD	-1,17897	3,41E-06
HIAT1	-1,17902	1,91E-07
MTMR2	-1,17967	9,04E-06
NUS1	-1,18289	7,28E-07
CAPZB	-1,18308	2,47E-05
HK2	-1,18332	0,000791
PHF20L1	-1,18359	1,54E-09
TSR2	-1,18399	1,52E-08
SHB	-1,18514	4,79E-10
TMCO1	-1,1852	6,22E-05
MRPL47	-1,18521	3,19E-07
MYDGF	-1,18609	2,28E-06
PTAR1	-1,18682	0,000115
FOXK1	-1,18757	1,84E-07

INTS9	-1,18982	2,17E-06
STARD3	-1,19084	0,000383
ARID4B	-1,191	8,77E-05
SERTAD2	-1,19116	6,55E-08
DIEXF	-1,19186	0,000417
HEATR1	-1,19255	5,59E-07
PAK1IP1	-1,19274	2,36E-06
DIS3	-1,19443	2,01E-06
ORMDL3	-1,1992	0,000119
NCAPD3	-1,1998	1,08E-06
STK24	-1,20144	1,69E-09
PHACTR4	-1,20166	4,73E-10
TM2D2	-1,20218	6,13E-07
NONO	-1,20239	1,05E-08
ACOT7	-1,20251	5,83E-06
LDHA	-1,20275	3,12E-09
GPKOW	-1,20348	0,000113
YAP1	-1,20363	1,71E-06
MAP3K3	-1,20371	0,00013
MAD2L2	-1,20388	0,000189
EXT2	-1,20461	6,81E-05
SIPA1L3	-1,20534	1,05E-09
RWDD1	-1,20627	8,97E-07
TRAF3IP1	-1,2079	2,69E-07
BAP1	-1,20854	1,58E-10
ZMYM6	-1,20935	0,000239
ATG3	-1,20979	6,25E-09
HAT1	-1,21002	6,65E-10
UBAC2	-1,21015	2,62E-07
CC2D1B	-1,21037	1,49E-06
EXO5	-1,2105	0,000232
RNF220	-1,21182	8,51E-10
USP4	-1,21223	2,34E-05
CHCHD2	-1,21247	7,67E-13
CASP7	-1,21359	0,000282
KANK2	-1,21436	1,13E-07
CCZ1	-1,21447	1,67E-06
KIF18A	-1,21457	5,14E-05
FAN1	-1,21493	0,000681
KAT6A	-1,2163	3,36E-06
PI4K2A	-1,21776	5,41E-05
DDX10	-1,21864	3,58E-09
CAP2	-1,21885	1,67E-07
ALG3	-1,21938	6,33E-05
PHC2	-1,21963	2,45E-06
LAPTM4A	-1,21971	9,41E-10
TMEM51	-1,22021	1,61E-05
OXCT1	-1,22379	1,54E-06
ERCC2	-1,22602	1,39E-05
DAB2IP	-1,22622	7,53E-09

NF2	-1,2273	4,95E-07
MKL1	-1,22793	8,68E-09
INTS10	-1,22845	7,64E-07
UTP6	-1,23103	7,77E-09
CDV3	-1,23114	4,75E-07
CEP152	-1,23137	0,000846
MORC3	-1,23166	0,000254
SLC36A1	-1,23174	1,43E-05
STX2	-1,23341	9,26E-05
PLEKHG2	-1,23363	3,64E-07
DDX56	-1,23425	4,24E-10
ARHGEF7	-1,23453	2,20E-06
EXOSC3	-1,23534	1,34E-06
RAB7A	-1,23805	0,000109
PITPNB	-1,23836	4,31E-11
GLMN	-1,23956	0,000456
NEK7	-1,23987	3,22E-06
ZFAND5	-1,23988	2,58E-09
ARIH2	-1,24053	3,20E-09
SLC25A46	-1,24054	2,84E-06
BCL3	-1,24109	0,000127
BNIP2	-1,24138	1,27E-05
ITGB1	-1,24327	2,95E-07
TRMU	-1,24362	1,61E-05
ASCC3	-1,245	0,000332
ISY1	-1,24576	2,15E-05
TMEM38B	-1,24587	2,97E-05
TMEM248	-1,24626	2,09E-09
THAP4	-1,24628	5,04E-09
HK1	-1,24733	1,59E-05
ACTN4	-1,24734	1,69E-06
GNL3	-1,24803	3,38E-05
CSPP1	-1,24943	0,000265
KDM5C	-1,24961	1,41E-10
HDHD1	-1,24966	0,000861
TSC22D2	-1,24967	1,77E-06
RIC8A	-1,2503	8,96E-12
PHIP	-1,25177	1,18E-07
DEDD2	-1,25336	6,67E-05
BAG4	-1,25368	2,44E-05
GPATCH3	-1,25513	0,000306
UBE3A	-1,25593	2,90E-10
FAM49B	-1,25639	7,87E-10
RUNX2	-1,25768	1,24E-07
AP2A2	-1,25933	5,61E-09
CCDC14	-1,26133	0,000394
GAS6	-1,26183	0,000236
PRPF38A	-1,26198	4,77E-11
FLVCR1	-1,26275	0,000314
APTX	-1,2634	1,66E-06

CD99L2	-1,26348	2,69E-05
MAP3K7	-1,26619	1,65E-08
CDK11B	-1,26704	5,73E-07
DNTTIP2	-1,26956	2,32E-10
TRIO	-1,26972	5,75E-09
TMEM138	-1,27089	3,70E-08
SNTA1	-1,27301	2,83E-05
SOCS6	-1,27322	1,47E-05
LMNA	-1,27329	0,000111
UHRF2	-1,27432	0,000554
SSFA2	-1,27579	1,06E-09
CDC37L1	-1,27586	2,45E-05
HAUS6	-1,27693	6,29E-07
TJAP1	-1,27853	0,000374
CHAF1B	-1,27919	6,06E-07
DHX16	-1,27963	1,38E-10
HDAC1	-1,27982	7,56E-11
PPP5C	-1,28107	7,88E-09
MMS22L	-1,28294	9,95E-06
RAP1A	-1,28354	1,24E-07
RLIM	-1,2846	9,00E-11
APEX2	-1,28534	8,76E-09
NIPA2	-1,28553	5,53E-07
MALSU1	-1,28634	3,66E-05
PNP	-1,28673	8,14E-10
ERCC6L	-1,28677	2,49E-07
TTK	-1,28776	1,73E-07
EFCAB2	-1,28799	0,000981
TOM1L2	-1,28894	0,000159
TFB2M	-1,28937	5,43E-05
BNIP3L	-1,29027	0,000905
HSPA13	-1,29032	5,39E-05
EPHX1	-1,29052	0,000121
MTO1	-1,29108	9,78E-07
AIDA	-1,29139	4,37E-05
EBNA1BP2	-1,29221	1,74E-13
ZRANB2	-1,29222	1,30E-10
NOL4L	-1,29247	1,82E-07
HIATL1	-1,29289	9,22E-09
MORF4L2	-1,29364	3,65E-12
PHF8	-1,29467	1,31E-07
BAHD1	-1,29476	4,83E-07
C20orf24	-1,29507	5,41E-08
SLC9A1	-1,29678	6,34E-11
KIAA2013	-1,29687	7,60E-09
HSD17B12	-1,29695	2,00E-09
SATB2	-1,29869	1,64E-06
DNAJB2	-1,30179	8,06E-09
CEP89	-1,30194	3,18E-06
ACVR1	-1,30251	4,75E-05

SKI	-1,30274	7,45E-10
KIAA0020	-1,30412	2,44E-05
RABGEF1	-1,30647	3,62E-08
SBNO2	-1,30716	7,70E-08
SDHB	-1,30754	3,67E-09
MRPL32	-1,30952	6,52E-08
CASP8AP2	-1,31071	2,55E-06
IPO5	-1,31086	1,44E-13
MLPH	-1,31291	3,65E-09
CADM1	-1,3159	2,30E-08
C1orf112	-1,31649	1,34E-06
SLC25A22	-1,31657	2,88E-07
ARHGEF18	-1,31711	1,16E-08
IKBKB	-1,3173	0,00021
UBA2	-1,3185	3,14E-08
CASC4	-1,31904	8,33E-09
ZNF234	-1,31939	0,000678
UBE2V2	-1,31969	3,64E-06
PFKP	-1,31995	5,68E-07
ITGAV	-1,31998	5,08E-06
MYCBP2	-1,32113	1,35E-08
MIER2	-1,32149	4,42E-06
MRTO4	-1,32229	1,11E-12
DONSON	-1,3236	2,75E-06
CWC25	-1,32391	5,83E-07
TOPBP1	-1,32422	2,11E-11
EIF4A2	-1,32554	0,00036
ZNF275	-1,32629	2,15E-07
ANKLE2	-1,32729	5,68E-10
DOCK7	-1,32859	4,57E-09
ACBD3	-1,3292	5,44E-09
AHCTF1	-1,32964	5,86E-06
ARFGAP1	-1,32968	5,96E-08
TIMM17A	-1,33171	1,12E-07
ACTN1	-1,33179	6,04E-07
PLEKHM2	-1,33206	3,00E-12
OSBPL2	-1,33333	5,93E-09
MSANTD3	-1,33432	2,14E-06
WASF2	-1,33434	2,35E-13
ERI1	-1,33456	2,89E-08
GPATCH1	-1,3349	3,75E-07
CDC42	-1,33807	9,64E-10
KPNA1	-1,33844	2,83E-08
TXN	-1,33867	1,10E-05
RAPH1	-1,3404	1,07E-05
MFN2	-1,34078	9,17E-13
S1PR2	-1,34189	0,000608
RAI14	-1,34441	1,72E-06
NASP	-1,34585	1,06E-14
MRPS22	-1,34614	4,18E-09

ADAM17	-1,34641	7,53E-10
CBFB	-1,34671	1,30E-10
REXO1	-1,34673	5,25E-12
C1orf216	-1,34687	6,66E-06
SLC23A2	-1,34752	2,50E-08
CCDC58	-1,3476	6,44E-08
PJA1	-1,34809	0,000137
CAPZA1	-1,35026	2,71E-11
ATP1B3	-1,35056	1,67E-14
CITED2	-1,35122	4,06E-05
COPA	-1,35123	1,61E-10
TMEM87A	-1,3538	4,32E-11
QSOX2	-1,35435	1,64E-05
RNF216P1	-1,35441	1,50E-06
DNAJC11	-1,35479	9,87E-13
PLP2	-1,35551	8,62E-11
NCLN	-1,35679	1,07E-12
MIER1	-1,35682	3,88E-08
IRS1	-1,35682	4,18E-10
SYMPK	-1,35902	6,83E-14
SPG20	-1,35911	3,67E-07
DENND5A	-1,36	3,60E-05
LEPROTL1	-1,36011	6,11E-06
KIFAP3	-1,36115	4,42E-06
TOE1	-1,36168	0,000107
UBAP1	-1,3647	2,05E-08
SLC9A6	-1,36507	0,000666
ANXA5	-1,3651	3,23E-13
KIAA1524	-1,36548	8,31E-09
DBNL	-1,36569	1,18E-05
MDC1	-1,36569	7,12E-12
ZNF512	-1,36656	9,34E-07
MPZL1	-1,36674	5,95E-14
SSH1	-1,36882	5,71E-10
SEMA7A	-1,36952	3,64E-06
TMEM30A	-1,37189	2,64E-05
SUV39H1	-1,37189	2,50E-09
FAXC	-1,37341	0,000224
NECAP2	-1,3786	1,42E-09
B3GALT6	-1,37888	3,10E-08
BLOC1S6	-1,37899	3,55E-09
EXOSC10	-1,3805	1,70E-10
KLHL2	-1,38056	0,000162
MYADM	-1,38064	0,000163
CDC42BPA	-1,38072	1,56E-06
BRF2	-1,38112	2,78E-07
ATG4B	-1,38119	5,75E-09
GNAI2	-1,3819	7,96E-06
DEPDC1	-1,38202	3,17E-07
UBXN8	-1,38215	2,77E-05

LSM1	-1,38247	1,14E-06
SLC25A32	-1,38276	1,03E-06
KIF4A	-1,38299	2,41E-12
PBDC1	-1,38403	8,03E-09
AP1M1	-1,38649	1,75E-06
RNF170	-1,38668	0,000407
SRPRB	-1,38679	1,17E-13
EXO1	-1,39012	1,22E-09
SHQ1	-1,3902	7,46E-07
TMEM39B	-1,39049	6,49E-06
TCEB1	-1,39146	1,99E-12
ATG9A	-1,3939	2,45E-06
MTF2	-1,39456	2,86E-06
HDAC7	-1,39477	2,84E-10
PDGFA	-1,39504	1,64E-07
SAT2	-1,39533	1,73E-06
GFM1	-1,39662	4,68E-12
GTF2B	-1,39691	6,76E-07
GADD45A	-1,39712	4,78E-07
HBS1L	-1,39903	2,40E-12
MRPL37	-1,39999	6,24E-08
REXO2	-1,40182	2,32E-05
CLSTN1	-1,40335	1,24E-09
VPS37B	-1,40349	6,05E-10
WDR47	-1,40531	8,56E-06
NUDT16	-1,40718	2,16E-06
ASB6	-1,40814	2,56E-09
C16orf70	-1,40932	1,62E-05
GPN2	-1,40943	6,40E-10
TMED5	-1,41061	5,64E-08
TRAM1	-1,41109	1,49E-05
PPARA	-1,41138	0,000167
SLC39A13	-1,41525	7,58E-07
LYRM2	-1,41763	1,13E-10
LDAH	-1,41795	5,91E-08
BUB1B	-1,41888	1,14E-05
ERICH1	-1,41905	3,62E-07
TMEM59	-1,42199	2,17E-06
KPNA6	-1,42441	1,62E-11
EIF3J	-1,42458	4,53E-12
MGAT5	-1,42518	3,74E-09
ARFGEF1	-1,42601	3,35E-10
VAMP3	-1,42728	5,10E-05
IPO9	-1,42782	5,34E-10
EMC1	-1,42848	6,08E-09
PLK2	-1,42879	0,000171
TMCO3	-1,42973	6,13E-06
TOMM40L	-1,43021	2,59E-06
TAF5L	-1,43094	3,83E-10
EFNB1	-1,4315	5,97E-06

SLC16A9	-1,43409	0,000238
ITGA6	-1,43492	2,18E-05
ASAP1	-1,43549	2,82E-12
BBX	-1,43564	1,52E-07
COPB2	-1,43657	2,40E-07
GTF2A2	-1,43695	1,85E-12
KIF2C	-1,43896	2,35E-09
TBC1D23	-1,43906	5,01E-07
CCM2	-1,43965	4,71E-09
YEATS2	-1,44017	0,000258
CEP85	-1,44164	0,000277
SYTL2	-1,44175	3,36E-08
MICA	-1,44469	2,11E-06
BCL2L2	-1,44516	8,14E-07
SKIL	-1,44596	9,05E-08
SH3BP5L	-1,44618	5,15E-11
XPNPEP1	-1,44878	2,14E-07
RAD54L	-1,4493	1,81E-06
SLC20A2	-1,44963	3,61E-11
PTS	-1,45005	1,78E-05
CREB3	-1,45106	1,59E-05
PLEC	-1,45246	0,000705
LYPD1	-1,45284	0,000585
FAM65A	-1,45425	1,37E-11
NME7	-1,45457	6,08E-05
IMPAD1	-1,4554	5,85E-12
LOC101929	-1,45668	0,001006
ARID1B	-1,45985	4,37E-10
TBC1D2B	-1,46214	1,71E-09
ABCD3	-1,46272	1,72E-10
RRAS2	-1,463	9,28E-06
TBL1XR1	-1,46496	1,43E-08
INPP5B	-1,46567	0,000281
UBR1	-1,4663	0,000226
ZNF283	-1,46686	0,00023
CENPW	-1,46731	3,46E-07
MYEOV2	-1,4674	3,46E-10
NCS1	-1,47111	4,94E-15
LBR	-1,47159	5,90E-10
SH3GL1	-1,47415	2,24E-10
SQSTM1	-1,47442	3,37E-05
STK4	-1,47469	4,21E-13
DMAP1	-1,47569	2,27E-05
ABCC4	-1,47579	6,84E-10
LPIN1	-1,47806	0,000786
MELK	-1,47905	2,87E-14
LANCL2	-1,48078	5,19E-12
STOM	-1,48092	1,02E-10
PLEKHA3	-1,48144	3,44E-05
PIP4K2A	-1,48369	2,02E-07

NFAT5	-1,48375	7,60E-06
SSH2	-1,48499	6,32E-07
FAM76A	-1,48512	0,00099
TBC1D25	-1,4853	0,000695
ADAM10	-1,48561	2,37E-12
SMU1	-1,48745	1,03E-13
LRRC47	-1,48757	1,14E-08
TMEM50A	-1,48827	2,51E-11
MPRIP	-1,48976	1,75E-11
MAGOH	-1,49095	2,15E-13
ZYX	-1,49219	4,66E-10
EDEM1	-1,49367	4,61E-10
ICMT	-1,4939	2,56E-15
MANEA	-1,49439	0,000116
SGCB	-1,4946	5,31E-09
NSMAF	-1,49464	7,52E-11
GINS3	-1,49571	5,21E-05
YIPF6	-1,49733	4,64E-12
LMF2	-1,49757	3,72E-08
FUCA1	-1,49945	1,46E-05
DOCK1	-1,50068	1,78E-08
GNL2	-1,50073	2,93E-13
MAD1L1	-1,50181	1,50E-16
GBF1	-1,50184	2,66E-06
HIC2	-1,50227	0,000448
NUP43	-1,50395	2,18E-08
PFN2	-1,50431	6,19E-17
PSMB2	-1,50445	8,06E-18
FOXC1	-1,50514	4,85E-08
SDCCAG8	-1,50522	4,58E-06
ANXA6	-1,50905	2,06E-05
B4GALT4	-1,51037	7,35E-10
GOPC	-1,5121	4,67E-12
YRDC	-1,51281	1,32E-08
DDAH1	-1,51299	0,000142
TOX2	-1,51305	3,76E-06
KRBOX4	-1,51313	0,00013
DNAJB11	-1,51375	6,31E-15
SNAP23	-1,51515	2,54E-07
SOWAHC	-1,5159	0,000547
UTP11L	-1,5161	3,38E-11
ATP11B	-1,51839	8,76E-13
PGS1	-1,51861	5,87E-07
GBE1	-1,52097	4,56E-07
KLHL5	-1,52215	8,66E-09
MIR4435-2	-1,52279	1,73E-06
ENO1	-1,52358	4,81E-09
PPP1R9B	-1,52436	4,57E-17
ERC2	-1,52481	4,20E-05
MIA3	-1,52495	6,42E-13

HOXB7	-1,52597	5,71E-07
NOTCH2	-1,52611	6,13E-16
FNIP1	-1,52842	7,92E-12
IP6K1	-1,52852	3,63E-14
TNFRSF12A	-1,52883	0,000234
ETV6	-1,52945	4,65E-09
CDC20	-1,531	9,77E-20
PCBP4	-1,53179	3,14E-09
PRDM2	-1,53193	1,62E-09
MIB2	-1,53235	4,06E-06
BACH1	-1,53334	3,42E-06
YAE1D1	-1,53372	3,22E-05
ARID5A	-1,53516	8,13E-06
TRIM5	-1,53539	0,000679
FAM129B	-1,53697	8,17E-13
OSBPL5	-1,53793	2,62E-06
EIF5A2	-1,53822	5,25E-08
NOD1	-1,53834	8,73E-06
LRRC40	-1,53938	1,90E-06
TNK2	-1,54003	9,60E-05
TMEM181	-1,54368	4,04E-11
PPP2R2A	-1,54451	8,37E-15
SGK223	-1,54663	2,15E-08
ALG14	-1,54874	0,000163
ARRDC1-AS	-1,55022	3,84E-05
LUZP1	-1,55186	1,50E-05
MAGT1	-1,55232	2,12E-14
TOLLIP	-1,5527	2,21E-05
WDR76	-1,55352	3,61E-07
ARMCX3	-1,55462	3,33E-08
UBQLN2	-1,55565	3,04E-13
NSRP1	-1,55566	3,02E-11
ATP6V0B	-1,55663	1,92E-17
HDLBP	-1,55682	1,28E-09
PLEKHA5	-1,55723	7,78E-09
VASP	-1,55866	4,49E-16
COPS7B	-1,55913	7,70E-09
ZAK	-1,56111	4,48E-11
LATS2	-1,56116	1,99E-07
ANTXR1	-1,56135	7,16E-17
CCDC137	-1,56189	4,21E-08
SEC23A	-1,56434	2,38E-08
RASA2	-1,56471	0,000108
RER1	-1,56473	2,05E-15
EIF2B3	-1,5651	3,14E-06
HMGXB3	-1,56519	2,55E-18
ESCO2	-1,56637	3,73E-09
SNRNP40	-1,56677	2,22E-12
AMPD2	-1,56766	1,00E-08
CXXC5	-1,56829	4,04E-06

MPDZ	-1,56829	1,67E-06
DIAPH3	-1,56902	3,88E-05
UCHL3	-1,56944	1,03E-06
LY6G5B	-1,57088	0,000473
IL10RB	-1,57192	8,46E-05
GTF2E2	-1,57196	1,20E-15
FAM73A	-1,57334	1,63E-05
CLN8	-1,57472	2,99E-10
SEPT2	-1,57639	1,10E-17
TPM4	-1,57893	3,67E-18
NEDD4L	-1,57898	1,72E-07
SEPN1	-1,57914	8,03E-17
RBFOX2	-1,58351	1,61E-16
LAS1L	-1,58552	2,78E-11
STXBP1	-1,5862	3,53E-09
GINS4	-1,58802	4,54E-12
HUS1	-1,58824	2,54E-07
BAG3	-1,58983	3,35E-06
NMNAT1	-1,59025	7,42E-06
ZBTB17	-1,59055	1,45E-11
TLN1	-1,59092	1,23E-13
TRIT1	-1,59328	1,49E-08
GNG5	-1,59489	2,83E-14
SPAG9	-1,59637	1,85E-10
BICD2	-1,59927	3,97E-12
STXBP5	-1,59963	1,31E-06
SNTB2	-1,60156	1,46E-09
HSPA12A	-1,60347	0,000134
POLQ	-1,60505	9,90E-07
TBC1D4	-1,6056	0,000339
MSX1	-1,60706	2,57E-06
PGK1	-1,60835	4,55E-17
QSOX1	-1,60867	3,15E-11
ITPRIPL2	-1,60979	4,75E-11
POLR3D	-1,61001	1,66E-08
KCTD9	-1,61038	1,09E-10
PRKACB	-1,61322	3,46E-07
PDLIM2	-1,61362	7,28E-06
CHSY1	-1,61568	1,48E-09
TMEM39A	-1,61625	2,63E-07
SHCBP1	-1,62311	4,81E-14
PTPN21	-1,62843	5,04E-09
LOXL1-AS1	-1,63063	0,000836
MTERF4	-1,63134	7,12E-10
HIPK2	-1,63266	1,11E-05
LEPROT	-1,63321	7,43E-12
ATP6V1H	-1,63507	7,48E-12
DNAJC24	-1,63791	2,73E-05
CLDND1	-1,63873	4,11E-10
NR2F1	-1,63897	2,38E-05

MLF1	-1,64047	3,45E-08
CD58	-1,64159	9,22E-07
HCFC2	-1,6421	0,000459
GCLM	-1,64211	3,50E-05
OTUD3	-1,64247	2,98E-07
PAM	-1,64407	2,17E-08
MBTPS2	-1,64644	1,86E-11
STAT3	-1,64798	3,31E-06
APOBEC3B	-1,65166	2,20E-05
FGFR2	-1,65523	5,91E-06
EMC7	-1,6553	6,97E-10
R3HCC1	-1,65587	1,27E-09
DR1	-1,66195	2,78E-10
HGSNAT	-1,66215	3,50E-06
TTC28	-1,66249	7,65E-06
TYRO3	-1,66266	6,38E-17
CHPF2	-1,66283	1,35E-08
CRISPLD2	-1,66393	9,52E-09
NUMBL	-1,665	3,12E-13
PLEKHM1	-1,66988	1,18E-06
ARF4	-1,67302	1,56E-06
YKT6	-1,67334	2,42E-19
CYB5R3	-1,67336	1,72E-10
LOC100506	-1,67765	0,000381
HIVEP2	-1,67886	1,17E-08
CMPK1	-1,68012	6,69E-09
RIC1	-1,68051	6,00E-08
JAG1	-1,68239	1,43E-06
DAPK3	-1,6834	1,41E-16
RAPGEF1	-1,68387	2,04E-12
CSNK1D	-1,68634	7,42E-17
SRSF4	-1,68772	3,98E-14
NABP1	-1,68902	2,49E-08
MYH9	-1,68934	2,46E-12
HTRA1	-1,6905	8,08E-11
NCEH1	-1,69218	1,61E-06
POMK	-1,69266	0,000639
PPARG	-1,69331	0,000956
DIXDC1	-1,6934	1,44E-05
TAF9B	-1,69427	1,46E-06
SLC16A1	-1,69561	3,14E-16
MCPH1	-1,69637	8,30E-13
PIBF1	-1,69744	2,82E-05
SVIL-AS1	-1,69875	7,37E-11
UBA3	-1,69999	1,43E-13
FAM135A	-1,70097	0,000331
ASB1	-1,70169	3,56E-14
AKIRIN1	-1,70286	1,13E-15
TRIM35	-1,70307	1,09E-09
POLR1E	-1,70338	1,23E-13

LRIG1	-1,70393	5,28E-10
ELP3	-1,70411	6,94E-17
DMPK	-1,7042	2,93E-06
CLTA	-1,70623	1,30E-17
FXR1	-1,70746	6,35E-13
C1orf52	-1,71045	7,37E-09
PRKAR2A	-1,71151	1,75E-08
FAM20C	-1,71389	1,86E-10
B4GALT5	-1,71607	1,85E-14
LDLRAP1	-1,71715	8,28E-07
SLC36A4	-1,71843	1,78E-05
C2orf74	-1,71944	5,89E-06
USP16	-1,72019	1,95E-17
SNX30	-1,72042	2,45E-11
PML	-1,72081	4,42E-05
BICD1	-1,72204	8,78E-07
PSMD2	-1,72421	1,14E-16
HCCS	-1,72524	1,02E-10
PANK2	-1,72666	5,01E-12
PTP4A2	-1,72737	2,12E-18
TTLL7	-1,72855	1,34E-05
PLIN3	-1,72944	8,85E-05
KCNG1	-1,73077	2,15E-05
PLOD1	-1,7325	3,77E-12
FBXO10	-1,733	5,02E-07
BRWD3	-1,73376	0,000183
SSR3	-1,73419	3,24E-16
PHF13	-1,7342	6,20E-14
ATAD5	-1,73454	7,95E-06
MAMLD1	-1,73639	0,000359
SUN2	-1,73936	5,13E-17
CD83	-1,73956	2,01E-05
PTBP2	-1,73971	2,78E-06
CAB39	-1,74037	1,03E-17
SWAP70	-1,74175	9,34E-10
SDC1	-1,74243	3,34E-15
TYW3	-1,74268	4,05E-16
MBOAT2	-1,74492	1,11E-11
PTPRG	-1,74929	2,87E-10
HDAC8	-1,74992	7,49E-07
AHI1	-1,7505	2,84E-07
ITGB5	-1,75392	4,51E-06
GNA12	-1,75552	4,96E-19
WWC2	-1,76331	1,66E-09
WSB2	-1,76695	2,96E-05
FURIN	-1,76757	1,24E-16
FHL3	-1,76863	2,86E-11
ZMYM6NB	-1,7697	0,000723
ACSL1	-1,77058	1,42E-14
MCOLN1	-1,77108	7,69E-09

E2F7	-1,77113	8,26E-10
NOL6	-1,77139	7,13E-13
TEX15	-1,77179	1,46E-07
GANC	-1,77542	9,10E-06
ANXA2	-1,77557	1,04E-26
COPRS	-1,7773	4,31E-18
ZNF266	-1,77961	1,25E-07
PPP1R7	-1,78137	5,47E-10
ELOVL5	-1,78269	1,96E-23
STIM1	-1,78407	9,17E-17
USP1	-1,78475	8,72E-12
CEP170	-1,7881	8,64E-13
FAM195B	-1,78974	2,13E-08
NPLOC4	-1,79126	1,00E-22
SEC14L1	-1,79136	3,17E-12
ANKRD10	-1,79202	2,76E-09
ZNF462	-1,79293	6,36E-09
DAGLA	-1,79323	0,00016
XYLT1	-1,79478	9,78E-10
GPAT3	-1,79609	0,000855
NUDC	-1,7965	2,51E-10
BIN3	-1,79712	2,17E-09
SH3GLB1	-1,79957	1,15E-15
C1GALT1	-1,80162	5,21E-14
RPF1	-1,80431	1,02E-14
NTN4	-1,80485	0,000748
OAT	-1,80546	1,23E-05
RAB29	-1,80568	3,32E-09
POFUT1	-1,80767	2,25E-16
MAP7D3	-1,81178	2,74E-21
ACOT9	-1,81456	8,00E-13
TFDP1	-1,81564	1,59E-23
TSPAN4	-1,81614	9,34E-07
ANO6	-1,81822	2,04E-13
SF3A3	-1,81964	3,11E-10
PEX14	-1,82155	1,48E-13
USB1	-1,82225	2,82E-07
BMF	-1,82302	1,00E-05
TMEM201	-1,82651	2,71E-15
SLC35G1	-1,82703	0,000899
RASL10B	-1,82741	0,000357
GOLIM4	-1,83058	1,47E-13
MUL1	-1,83095	1,14E-15
LRRC41	-1,83201	1,32E-21
SLC41A1	-1,83284	4,73E-15
RANBP6	-1,83287	7,19E-09
UBE2J2	-1,83698	6,66E-23
NKX6-1	-1,8389	0,000563
KAZN	-1,83945	1,31E-09
PPARD	-1,84024	5,03E-08

TGFB1	-1,843	3,49E-15
BAG2	-1,8463	2,86E-14
LPCAT4	-1,84672	2,51E-06
LRRC8E	-1,8505	3,35E-09
TPP2	-1,85105	7,61E-18
ARHGDIA	-1,85106	1,50E-13
DTX3L	-1,85119	2,49E-07
RMDN3	-1,85316	5,17E-10
ZNF274	-1,85374	7,23E-12
SNX25	-1,85384	5,59E-08
SLC25A12	-1,85491	1,72E-10
UBE2F	-1,85651	6,00E-10
LOC28378{	-1,85919	1,81E-05
RASAL2	-1,85968	9,37E-12
EEF1E1	-1,86171	0,000706
AFF3	-1,86212	0,000181
CNST	-1,86438	7,46E-08
DNAJA1	-1,86465	2,60E-11
EHD4	-1,86553	7,03E-15
ENTHD2	-1,86564	2,25E-06
ZNF827	-1,86892	9,67E-06
RELT	-1,86992	1,04E-09
CCNJL	-1,87267	1,45E-05
TOMM34	-1,87397	3,61E-16
LMAN2L	-1,87428	1,65E-15
ATP10D	-1,8744	5,44E-05
GRIN2D	-1,87593	2,47E-05
B3GLCT	-1,87633	6,82E-08
SLIT2	-1,87793	0,000544
HECA	-1,88114	3,49E-06
TRMT13	-1,88166	0,000693
C19orf12	-1,88326	3,68E-13
EVI5	-1,88386	1,80E-07
CENPQ	-1,88438	4,97E-08
TMEM56	-1,88535	2,32E-05
C12orf4	-1,88546	4,93E-11
ILKAP	-1,88836	7,74E-11
TPRA1	-1,88929	1,68E-20
ZNF230	-1,8899	0,000287
FAM214B	-1,89017	1,48E-08
IFIT5	-1,89034	0,0003
SPATS2L	-1,89217	3,14E-14
CCDC18	-1,89262	0,000144
WIPI1	-1,89591	3,65E-06
UROD	-1,89632	6,04E-15
CDC14B	-1,89693	6,89E-10
C16orf45	-1,89887	3,95E-05
PQLC2	-1,90464	1,71E-10
RNF145	-1,90514	1,08E-06
SHROOM1	-1,90637	1,09E-10

IFNGR2	-1,90679	3,22E-09
IKBIP	-1,90701	4,95E-05
RRAGC	-1,91311	2,05E-14
ERCC1	-1,91612	1,45E-20
GPAT4	-1,91651	1,50E-12
MPP6	-1,91764	2,79E-16
MAML2	-1,91783	4,63E-05
ANXA2P2	-1,91791	0,00012
NFATC4	-1,91908	4,92E-07
MACF1	-1,92064	1,42E-23
MYO9B	-1,92238	4,77E-11
TWISTNB	-1,92392	1,82E-18
SGK1	-1,92419	0,000217
CTNNAL1	-1,92721	1,14E-09
TNFRSF10B	-1,93185	4,88E-19
ALAS1	-1,93229	1,91E-13
UAP1	-1,93517	3,35E-12
CAPN10	-1,93528	3,13E-07
LOC100129	-1,94118	9,97E-12
AHDC1	-1,94475	1,55E-18
ELF4	-1,94951	2,46E-10
COTL1	-1,95139	2,83E-19
SLC45A3	-1,95253	2,72E-08
WNT11	-1,95635	0,000376
ITPKA	-1,9568	0,000203
TGFB3	-1,9582	0,000513
KIF1B	-1,95841	5,66E-12
CCDC50	-1,96251	1,40E-13
DLC1	-1,96298	5,02E-14
SLFN5	-1,96361	2,14E-08
SIRT2	-1,96644	1,12E-09
EIF2AK4	-1,9668	3,12E-22
C12orf75	-1,96952	6,60E-14
RDX	-1,96993	5,87E-13
ABL2	-1,97009	1,37E-20
CDK6	-1,97025	0,000495
ST3GAL4	-1,97454	4,80E-08
PLSCR1	-1,97473	5,37E-15
DOCK5	-1,97536	1,25E-12
IPO13	-1,97628	2,00E-09
DNMBP	-1,97907	1,42E-08
USP40	-1,98023	3,15E-17
ANLN	-1,98238	2,18E-22
ETHE1	-1,9862	5,55E-10
ARNTL	-1,98672	1,12E-05
TXLNA	-1,98679	3,60E-17
AAK1	-1,9891	1,24E-11
PANK4	-1,991	1,21E-13
FMNL3	-1,99328	4,46E-17
FBLIM1	-1,99446	4,16E-07

SCCPDH	-1,99603	3,07E-12
TMX3	-1,99741	3,21E-13
TSHZ3	-2,00004	0,000939
CARD10	-2,00109	1,67E-08
DCTN3	-2,0023	6,18E-15
RRAS	-2,00445	0,000102
CALU	-2,00603	1,51E-20
TUBGCP3	-2,00735	2,02E-20
NOL9	-2,00737	4,01E-14
ODF2L	-2,00813	2,05E-15
LCA5	-2,00936	0,000823
BACE1	-2,01315	2,22E-13
DDA1	-2,01331	4,23E-20
SNX9	-2,01599	6,16E-20
ADORA2B	-2,01822	7,04E-05
PPP2CB	-2,01865	2,30E-24
SEPT7	-2,01875	1,57E-25
RASSF5	-2,01968	0,000512
KANK1	-2,02231	2,08E-14
ARFGAP3	-2,02565	4,12E-12
SNHG3	-2,02573	2,02E-06
SH3BGRL3	-2,02577	8,86E-08
ZSCAN20	-2,02654	1,24E-05
FGD1	-2,02658	7,06E-24
ABHD6	-2,02844	2,17E-05
U2SURP	-2,02906	1,08E-32
PYGO1	-2,02966	2,81E-05
LOC103611	-2,0316	0,000631
HDAC5	-2,03296	4,82E-16
RBM24	-2,03473	3,29E-06
PBK	-2,03475	6,70E-12
CHST14	-2,0354	6,59E-13
LRRFIP1	-2,03677	3,66E-29
PITHD1	-2,03915	1,56E-19
VPS37A	-2,04192	2,76E-19
KAT2B	-2,04298	1,01E-05
ZNF530	-2,04304	1,90E-06
ADAM9	-2,04535	6,74E-21
SDC2	-2,0478	5,32E-20
CYLD	-2,0563	0,000743
RDH10	-2,05655	3,48E-09
BIRC2	-2,06012	8,01E-12
RELB	-2,06262	2,57E-06
MEF2D	-2,06936	4,96E-11
CCDC126	-2,06957	2,61E-05
DLEU2	-2,07049	0,000678
SCMH1	-2,07371	1,83E-05
LACTB	-2,07407	0,000178
PSD3	-2,07738	2,41E-19
SNX8	-2,07755	8,20E-16

PDE4A	-2,08025	1,61E-11
EFCAB7	-2,08034	4,69E-05
TCF19	-2,0879	1,65E-27
CLCN6	-2,08808	8,30E-05
SZT2	-2,09285	2,62E-11
SBDS	-2,09394	8,46E-27
UBA5	-2,09524	3,40E-21
PPP3CC	-2,09749	3,74E-14
IL13RA1	-2,1034	3,00E-15
FAT1	-2,10539	3,07E-12
JPH3	-2,10594	3,00E-12
AKR1C1	-2,10787	0,000709
ABCB7	-2,1094	2,56E-14
TCEB3	-2,1097	6,49E-24
FOXP1	-2,11064	1,62E-21
OGFRL1	-2,12269	3,80E-12
HECTD2	-2,12416	4,18E-07
CTSB	-2,12797	3,47E-11
LPXN	-2,12819	5,12E-07
ARL16	-2,13232	4,10E-15
ST5	-2,13343	3,58E-08
SEC61G	-2,13514	1,22E-09
STK17A	-2,13567	2,28E-21
SLC25A20	-2,13904	6,96E-06
PMEPA1	-2,14107	1,45E-20
SLC35D1	-2,14231	3,59E-14
SLC2A10	-2,14371	1,20E-10
HSPB11	-2,14443	4,73E-13
CHORDC1	-2,14924	5,76E-24
GPRC5B	-2,15213	0,000287
LINC00623	-2,15383	3,27E-07
GNG12	-2,15618	2,42E-17
TAP2	-2,15677	1,49E-14
SHISA4	-2,15948	0,000732
PGM3	-2,1661	2,56E-14
SLC25A37	-2,16849	3,07E-14
IMPA1	-2,16933	2,21E-15
STARD8	-2,16976	0,000406
C15orf41	-2,17229	1,06E-06
PMAIP1	-2,17532	2,00E-08
GADD45B	-2,17825	0,000213
CERK	-2,17965	6,24E-10
PITPNM2	-2,18003	5,00E-10
RBPMS2	-2,18007	3,30E-11
TMEM44	-2,1803	1,13E-09
SMC4	-2,1818	4,31E-28
CCND3	-2,18217	3,61E-07
HOXC4	-2,18263	0,000183
SMYD3	-2,18338	1,27E-09
TUBB2A	-2,18358	6,47E-06

STEAP1	-2,18406	0,000687
SUSD1	-2,18711	4,54E-10
VCL	-2,18713	3,94E-11
TMEM104	-2,18801	1,89E-13
FGFRL1	-2,18858	1,02E-33
TK2	-2,18925	2,08E-08
FOXO3	-2,19143	1,41E-09
CORO1C	-2,19201	1,11E-29
HLA-F	-2,19425	0,000779
CDK14	-2,19433	2,05E-05
CSRNP1	-2,19435	7,17E-14
RIMS3	-2,2001	1,23E-05
SLC30A7	-2,20704	1,21E-15
ENTPD4	-2,20834	2,95E-23
SERPINB8	-2,20981	1,84E-06
TYMP	-2,21175	0,000234
CCT6A	-2,21501	2,66E-31
CYTH3	-2,21598	1,12E-20
ANKH	-2,21682	3,52E-19
RTCA	-2,21699	4,29E-22
PFKFB3	-2,21864	4,47E-10
JMJD6	-2,22357	3,16E-12
LOC101927	-2,23239	0,000327
CPEB4	-2,24004	1,59E-05
TSHZ1	-2,2405	1,72E-12
HACD1	-2,24505	8,19E-10
TMEM55A	-2,2473	4,80E-12
ITPR1	-2,24965	6,27E-13
ARL4C	-2,25299	1,27E-22
S100A6	-2,25311	1,13E-11
PCDHGC3	-2,25496	7,45E-15
MEIS1	-2,2582	0,000797
SLC17A5	-2,26314	6,75E-09
LTBP2	-2,26563	5,74E-06
HYI	-2,26683	4,19E-06
CLSPN	-2,26741	8,05E-21
TIGD7	-2,26808	1,17E-07
WDR90	-2,26919	0,000998
TCEAL3	-2,27344	3,44E-15
SORBS3	-2,27361	3,13E-23
NEK6	-2,27376	5,80E-29
TTN-AS1	-2,27618	1,90E-05
MOCOS	-2,27734	2,52E-08
LOC10013C	-2,27873	0,000417
GPR180	-2,27976	1,71E-19
DLX1	-2,28571	1,06E-12
COLGALT1	-2,28772	1,42E-39
PAK1	-2,2938	1,17E-11
CAP1	-2,29385	8,14E-24
EOGT	-2,29536	1,61E-09

FZD7	-2,29635	1,22E-18
SH2D5	-2,29708	0,000312
ADCY9	-2,30274	9,45E-15
TMSB4X	-2,30452	5,75E-35
FMNL1	-2,30575	1,67E-15
DYRK3	-2,30789	3,73E-15
ZC2HC1A	-2,30916	8,32E-08
MAPKBP1	-2,30969	1,55E-10
SAMD11	-2,31125	5,95E-06
FAM114A1	-2,31586	3,89E-07
TREX1	-2,31763	7,60E-08
ST3GAL3	-2,31774	1,65E-08
SMARCD3	-2,31931	5,07E-13
HHLA3	-2,32535	3,27E-13
RLTPR	-2,32606	1,00E-10
MFGE8	-2,32611	6,04E-12
ATP8B2	-2,32973	3,03E-24
COPS8	-2,32997	1,47E-26
C11orf63	-2,33181	2,14E-09
FNTA	-2,33184	2,02E-29
SFXN3	-2,33253	4,45E-11
ANKRD13C	-2,33799	3,03E-20
LIMD2	-2,33922	1,94E-05
METRNL	-2,34178	3,75E-34
CHM	-2,34251	1,36E-07
POMZP3	-2,34252	1,51E-14
FNDC3B	-2,34376	7,57E-22
SMS	-2,34411	7,44E-22
FERMT2	-2,34907	4,65E-26
JAK1	-2,35013	1,41E-20
STOML1	-2,35027	1,44E-09
KCNJ14	-2,35356	4,61E-06
TMCC1-AS1	-2,35932	0,000163
RGS5	-2,36812	6,08E-05
TEX30	-2,36941	1,08E-14
TRAM2	-2,3719	2,83E-39
BHLHE40	-2,37235	4,29E-26
BTG3	-2,38085	1,28E-07
BIVM	-2,38156	2,28E-06
EXTL3	-2,38425	2,97E-33
PCDHGB2	-2,38918	3,56E-07
FGF18	-2,38952	0,000194
CHST7	-2,39288	3,98E-05
IRF1	-2,39433	7,71E-13
MED8	-2,39539	6,36E-26
GPX8	-2,39848	1,14E-24
FTL	-2,39888	3,02E-11
ECHDC1	-2,40079	1,18E-17
KCNQ5	-2,40351	4,51E-05
ZNF223	-2,40539	0,000135

LINC01128	-2,407	6,08E-10
MTCL1	-2,40791	7,83E-12
SLC12A8	-2,40881	5,83E-16
SBF2	-2,41162	5,99E-08
GFPT2	-2,41674	2,03E-09
TUB	-2,4198	2,14E-13
SCRN1	-2,42432	6,33E-37
NXPE3	-2,42617	6,27E-06
ENC1	-2,4271	6,08E-18
RABGGTB	-2,43454	6,67E-28
SPSB1	-2,43786	8,68E-07
GOLM1	-2,44098	1,98E-35
GPR68	-2,44406	4,92E-08
EFHD2	-2,44476	3,01E-23
CPOX	-2,44775	5,33E-11
POFUT2	-2,449	3,26E-17
TCF12	-2,44936	1,93E-17
SDC4	-2,44969	2,95E-18
PHLDB1	-2,45086	5,05E-32
ARMC9	-2,45646	1,71E-25
FAF1	-2,45805	5,69E-13
CAPRIN2	-2,46388	4,70E-17
CFL2	-2,46456	1,73E-19
RTTN	-2,46833	3,71E-10
AHNAK2	-2,46886	2,30E-15
KLF13	-2,47342	5,37E-38
OSMR	-2,47381	3,10E-11
IL6ST	-2,47696	4,17E-19
DUSP7	-2,48431	1,67E-09
TIPARP	-2,48493	1,91E-20
CAMK2D	-2,48825	6,45E-19
MYO10	-2,49005	9,64E-30
BEND6	-2,49115	1,08E-05
EVA1B	-2,49492	7,57E-07
FAM89A	-2,49558	1,38E-05
DLX2	-2,49663	7,00E-14
DCLK2	-2,49768	0,000106
KLHL21	-2,49781	2,14E-34
PLAGL1	-2,4984	2,57E-10
SDCBP	-2,49847	6,81E-16
TACC1	-2,49862	9,00E-38
HOXC9	-2,50045	5,53E-06
PRPS1	-2,50095	1,71E-25
CHST12	-2,50432	7,06E-24
MYO1E	-2,51007	5,87E-10
NAV2	-2,51706	8,82E-18
CD59	-2,5174	1,20E-12
CKAP4	-2,51766	7,64E-50
SEC14L2	-2,52034	4,54E-07
ARNTL2	-2,52789	3,92E-13

LACC1	-2,52831	8,12E-09
PDP1	-2,52987	2,76E-28
GLS	-2,53261	5,02E-31
MAFG	-2,53667	1,70E-27
PLEKHO2	-2,53696	3,12E-14
SRGAP2D	-2,54075	8,05E-09
RIN3	-2,54574	4,44E-10
INAFM2	-2,54698	4,69E-09
TIAM2	-2,54944	3,02E-11
QPCT	-2,55168	5,77E-07
SETBP1	-2,55565	0,000272
IFFO2	-2,55867	2,88E-21
CIRBP-AS1	-2,56025	0,000439
TPST2	-2,56299	1,60E-15
KLF12	-2,56348	3,50E-13
SHROOM2	-2,56822	9,53E-14
DGKD	-2,56889	8,85E-29
PRNP	-2,57325	3,30E-20
NFIL3	-2,57418	1,14E-06
MFSD1	-2,57837	9,64E-18
ARPIN	-2,58162	2,29E-11
PARVA	-2,58176	8,62E-22
RILPL2	-2,58646	2,53E-05
SLC35E4	-2,59108	7,64E-15
ABHD2	-2,59128	3,83E-23
GULP1	-2,59252	1,86E-05
SEPT10	-2,59612	4,14E-08
TIMP3	-2,59833	3,13E-13
DNAJC3	-2,60028	6,05E-18
FKBP7	-2,60241	3,54E-11
PRKD1	-2,60407	2,92E-06
ATL1	-2,60419	0,000106
KDELC1	-2,60647	1,77E-15
MT2A	-2,6086	2,70E-38
ZIC2	-2,60911	2,26E-12
RECK	-2,61141	5,65E-05
LRP12	-2,61466	1,97E-28
ADGRE5	-2,61779	9,00E-13
PHEX	-2,62029	0,000679
SMAP2	-2,62033	7,84E-11
TFPI	-2,6216	6,72E-06
TTC14	-2,62183	1,36E-17
CAPG	-2,62328	8,83E-36
TRIM7	-2,6258	6,32E-06
KCTD7	-2,62633	6,71E-13
PVR	-2,62908	2,58E-13
TAPBPL	-2,63144	0,000402
LIMA1	-2,63149	1,23E-13
ANKMY2	-2,63261	2,94E-20
RUNX1	-2,63323	1,40E-38

SYNJ1	-2,63512	1,06E-16
KIAA0355	-2,63791	1,44E-22
ITM2C	-2,63902	5,02E-21
MEG3	-2,6416	0,00066
STEAP3	-2,66356	1,54E-22
LOC103091	-2,66763	4,35E-07
STARD3NL	-2,67029	3,19E-17
UAP1L1	-2,6712	3,02E-19
GLIPR2	-2,67156	1,19E-10
SLCO3A1	-2,67859	2,63E-21
HOXC6	-2,68654	6,43E-09
ZNF222	-2,68738	3,01E-05
GRB10	-2,68828	4,82E-06
IL1RAP	-2,69091	3,88E-06
RBMS2	-2,69134	1,24E-14
B3GNTL1	-2,69241	4,46E-10
MICAL1	-2,71079	2,28E-09
PPAP2B	-2,71403	2,06E-12
DAAM2	-2,71418	1,87E-08
C8orf48	-2,71481	4,85E-08
LINC00641	-2,71504	1,29E-12
ID3	-2,71516	2,28E-14
OAS2	-2,71719	0,000923
CDKN2B	-2,72269	6,57E-15
ZBTB47	-2,72539	0,000108
CLTCL1	-2,72575	8,57E-13
SOCS3	-2,729	7,91E-18
SCN1B	-2,73002	8,63E-05
LOC90768	-2,73547	1,06E-12
MGAT5B	-2,73772	1,80E-10
LHFPL2	-2,73857	2,16E-32
APBA1	-2,73874	6,33E-11
DNAJC3-AS	-2,74005	3,51E-05
TOR4A	-2,74109	9,61E-13
RGL1	-2,74494	2,04E-15
RTN4RL2	-2,74869	1,94E-05
SERINC2	-2,75879	1,08E-16
BASP1	-2,76458	1,79E-07
GALNT2	-2,76578	1,30E-43
SMCO4	-2,76728	1,87E-08
LGALS3	-2,77378	2,81E-12
SMIM3	-2,77592	7,51E-27
ADORA1	-2,77655	1,99E-08
WNT5A	-2,77889	6,25E-19
MCC	-2,78092	2,21E-15
CEP19	-2,78644	5,86E-11
DNAJB5	-2,79072	1,42E-08
SDCBP2-AS	-2,80055	3,15E-05
CPM	-2,80344	4,52E-05
KIF13B	-2,80582	1,11E-08

CLN5	-2,80905	9,31E-13
L3MBTL3	-2,81076	8,01E-16
SMAD3	-2,81329	7,27E-19
C14orf159	-2,8143	1,04E-07
HOXA4	-2,81522	8,38E-09
LGALS1	-2,81637	1,56E-19
BTN2A1	-2,82112	4,74E-11
CTSL	-2,8224	5,49E-17
PORCN	-2,82763	1,10E-10
IL15RA	-2,82905	9,39E-09
BTN3A1	-2,82984	0,000188
TIAM1	-2,83299	1,22E-17
GNPDA2	-2,83846	2,49E-13
EYA2	-2,84375	2,93E-08
CCDC82	-2,84775	2,38E-10
KCNMA1	-2,85122	1,79E-08
NUPL2	-2,85169	3,31E-09
SRPX	-2,85814	0,000518
KIF26B	-2,85976	2,92E-11
NXN	-2,86706	9,27E-43
CNTLN	-2,87148	6,91E-17
OSTM1	-2,87251	1,17E-08
CPQ	-2,87506	2,03E-06
FAM92A1	-2,88067	2,13E-08
SOX9	-2,88397	2,53E-05
PPP1R18	-2,88863	7,08E-34
MAMSTR	-2,89644	3,64E-06
P4HA2	-2,89771	7,06E-21
ANKRD44	-2,89998	4,15E-05
OPTN	-2,90045	2,17E-07
TRIP10	-2,90082	1,97E-18
PTPN14	-2,90416	1,19E-41
AKAP7	-2,90418	4,71E-05
MIR22HG	-2,90566	4,36E-11
ZFAT	-2,91393	1,33E-31
IL6R	-2,91765	0,000255
FRRS1	-2,92091	7,90E-06
CAV2	-2,93639	1,40E-17
HERC3	-2,93772	8,19E-10
PCOLCE2	-2,93835	7,09E-14
ITGA3	-2,94032	5,72E-56
TIMP1	-2,94142	4,00E-08
HOXB9	-2,94565	0,00062
HIVEP3	-2,94787	2,35E-05
B2M	-2,94991	3,38E-20
HNMT	-2,95041	1,16E-06
HTATSF1P2	-2,95342	1,12E-07
CLCF1	-2,95846	6,15E-05
ST6GALNA(	-2,95916	1,20E-12
CHIC1	-2,96313	8,82E-10

CDKN2C	-2,96347	3,20E-43
DEGS1	-2,96539	2,26E-25
TGFBR2	-2,97049	1,19E-21
ZNF155	-2,97498	1,20E-08
SOAT1	-2,97605	5,44E-41
SNORA73A	-2,97791	4,91E-05
CD82	-2,98559	1,87E-24
ARHGEF10	-2,98601	3,60E-17
KIAA1549L	-2,99429	8,86E-10
SH3BGRL	-2,99469	4,94E-24
ADGRB2	-2,99589	2,03E-17
ANK2	-3,00156	1,32E-06
SH2D4A	-3,00248	9,02E-46
MX2	-3,00427	1,43E-07
FLOT1	-3,00649	2,41E-52
FAM219A	-3,00652	9,56E-15
ATP6V1B2	-3,00709	6,46E-57
SQRDL	-3,00836	1,25E-11
DENND5B	-3,01756	3,15E-22
LOC389831	-3,0197	3,42E-15
FGFR1	-3,02053	1,74E-58
ITPRIP	-3,02269	7,05E-11
IGFBP6	-3,02852	4,60E-05
GPATCH11	-3,02859	6,67E-33
VEPH1	-3,02912	2,33E-06
PTHLH	-3,03324	5,11E-10
GYG2	-3,04013	1,80E-10
HMGN5	-3,04619	0,000357
AMIGO3	-3,05141	7,72E-18
PLOD2	-3,05751	7,83E-57
TNFAIP3	-3,06335	6,72E-08
KIAA0930	-3,06364	1,87E-29
FAM13A	-3,06884	1,60E-08
SH3BP4	-3,07788	4,98E-41
ZIC5	-3,07832	2,32E-05
DNM1	-3,07981	2,09E-30
PPP1R15A	-3,08103	3,01E-29
FNIP2	-3,08889	3,45E-08
SLC22A15	-3,08969	7,83E-05
SYNPO	-3,09677	3,64E-10
SLC37A2	-3,097	6,86E-06
ARHGAP24	-3,09798	2,25E-07
FAM49A	-3,10003	0,000683
PEA15	-3,10311	4,97E-45
CMTM7	-3,10875	1,45E-12
TMEM140	-3,11048	5,57E-05
EPHX4	-3,11112	3,80E-05
LRRC8C	-3,1177	1,10E-12
HOOK3	-3,12525	2,11E-31
FMN1	-3,13188	1,47E-10

HSPA6	-3,1395	5,93E-05
OAF	-3,14036	9,78E-25
EPHB2	-3,14279	1,63E-64
DDX60L	-3,16222	3,57E-13
KIAA1024	-3,16315	1,38E-06
LDLRAD4	-3,1676	0,000162
GPC4	-3,16849	1,02E-37
LMO7	-3,18128	6,45E-43
ROR1	-3,18201	1,43E-13
SLC39A14	-3,18252	1,61E-17
NIPAL3	-3,18362	1,99E-18
RGS2	-3,18547	2,78E-09
AKR1B10	-3,20333	0,00063
PEX6	-3,20665	1,24E-11
ARAP3	-3,20742	6,58E-09
CD44	-3,21251	6,91E-53
ZBTB38	-3,21845	2,61E-42
FAM126A	-3,21944	1,09E-25
ARMCX1	-3,22048	9,20E-12
EXTL2	-3,22331	3,42E-26
TRIM47	-3,22526	2,32E-21
CRIM1	-3,22804	1,47E-25
DCBLD1	-3,24234	5,21E-28
HS3ST3B1	-3,24533	4,73E-23
MAP7D1	-3,24966	8,50E-43
P3H2	-3,25244	2,41E-43
STPG1	-3,25683	1,38E-26
CYP4V2	-3,2572	3,64E-07
ANKRD13A	-3,25898	1,68E-21
PDGFC	-3,25939	4,03E-10
FSTL3	-3,25965	1,28E-19
STK17B	-3,26844	4,82E-40
NKX3-1	-3,26929	2,23E-17
GXYLT2	-3,26963	1,33E-13
PGM1	-3,27006	4,32E-33
ZFP37	-3,28436	3,69E-06
ECE1	-3,28712	6,24E-53
PALM2	-3,29498	1,20E-06
WNK3	-3,30404	0,000383
C16orf62	-3,31257	2,18E-31
SLC7A8	-3,32626	2,75E-20
ETV4	-3,33406	0,000156
NAV1	-3,33458	4,98E-14
DDX11L2	-3,33846	0,000896
LINC00152	-3,33997	1,17E-11
HNRNPU-A	-3,34238	3,25E-10
FAM225A	-3,34625	0,000704
LOC10192٩	-3,34777	0,000596
CARS2	-3,34844	2,55E-68
THRB	-3,34926	1,93E-16

HOXD8	-3,35115	3,20E-06
LOC344887	-3,35148	0,001006
GPC1	-3,35423	9,41E-52
GABARAPL	-3,35811	3,46E-05
PCOLCE	-3,36205	2,09E-36
ANO7	-3,36221	2,12E-05
ADGRL2	-3,36488	2,20E-38
DLG4	-3,36893	9,67E-13
SNAI1	-3,36986	6,84E-16
AHRR	-3,37398	2,61E-08
ALPL	-3,37659	1,16E-07
RCAN2	-3,39687	3,74E-05
GALNT10	-3,40887	8,15E-71
RRAGD	-3,41302	4,55E-20
LRRK1	-3,41435	2,26E-15
PKDCC	-3,4194	0,000284
SMPD1	-3,43822	1,09E-13
STRIP2	-3,44243	3,65E-17
CMTM3	-3,44517	2,49E-21
ZNF532	-3,45683	5,33E-55
C3	-3,46684	0,000566
LINC-PINT	-3,46939	2,08E-05
SPEG	-3,47269	1,42E-19
RHOBTB2	-3,47434	1,07E-38
ZNF697	-3,48161	1,73E-33
FAM198B	-3,49064	1,16E-49
FKBP14	-3,49483	4,87E-49
ZNF382	-3,50902	1,16E-05
SLC6A8	-3,51672	1,50E-21
SPRY2	-3,51717	2,24E-18
DCBLD2	-3,52407	3,63E-41
TLN2	-3,52452	9,36E-33
GOLGA8B	-3,52469	9,24E-06
SH3RF2	-3,52749	4,70E-11
MITF	-3,52851	9,49E-06
PCDHB17P	-3,52915	9,10E-06
CPEB1	-3,53066	4,93E-15
MAP3K14	-3,53293	1,48E-21
COL6A1	-3,53357	6,43E-72
CRAT	-3,53359	2,05E-12
MT1X	-3,5365	7,03E-58
SLFN11	-3,54345	7,26E-23
ACO1	-3,544	4,84E-27
SH3PXD2B	-3,54512	5,22E-59
LCTL	-3,55029	1,76E-06
CCR1	-3,55178	8,42E-05
SIPA1	-3,55758	1,61E-32
PITX2	-3,55819	7,29E-13
CEP112	-3,55832	6,64E-17
ZCCHC24	-3,55915	2,41E-16

CUEDC1	-3,55962	2,34E-68
LAMB3	-3,55982	0,000199
HLA-A	-3,56923	1,15E-41
UBASH3B	-3,57276	1,02E-16
PALLD	-3,57492	3,10E-33
TNS3	-3,57524	3,69E-31
FAM13C	-3,57696	6,63E-05
MAP1B	-3,57702	1,29E-22
CADPS2	-3,57936	0,000165
DNAJB4	-3,5845	3,28E-12
EXT1	-3,58455	6,41E-51
NDRG1	-3,586	1,47E-42
MICB	-3,59409	5,70E-23
SIK1	-3,59551	0,000332
SYNJ2	-3,59604	4,38E-68
ARMC4	-3,6001	0,000572
LRP1	-3,6006	7,42E-11
CCDC88A	-3,61209	1,89E-47
PXDN	-3,61588	2,82E-46
BTN3A3	-3,61687	1,07E-06
PTPRB	-3,61776	0,000276
ACSL4	-3,62118	3,61E-42
DYSF	-3,63922	4,84E-07
WTIP	-3,64055	3,37E-23
BMP1	-3,6408	1,31E-60
ELL2	-3,64403	4,98E-33
RARB	-3,65022	6,31E-09
AGPAT4	-3,65305	1,08E-12
ATAD3B	-3,65323	8,62E-25
PLEKHF1	-3,65645	9,29E-14
ULBP3	-3,66286	1,72E-08
TPM1	-3,66671	2,04E-98
ELFN2	-3,68181	9,36E-11
SYDE1	-3,6829	5,74E-41
GLRB	-3,68856	4,83E-09
FOSL2	-3,70529	2,30E-56
GBP2	-3,70853	1,04E-05
PLXND1	-3,71261	1,20E-39
LYST	-3,71783	3,56E-20
TRIM22	-3,71797	0,000248
GNB4	-3,72158	1,04E-45
RAB31	-3,72355	2,04E-21
PXDC1	-3,72693	3,73E-53
FRMD5	-3,73425	2,85E-26
SASH1	-3,73653	1,20E-18
VOPP1	-3,7397	1,14E-60
PLEKHA2	-3,74471	1,49E-31
MB21D1	-3,77563	4,16E-33
PCDHB5	-3,77924	3,20E-05
MRAS	-3,78045	1,67E-36

PROCR	-3,78255	5,63E-08
LIF	-3,8009	1,46E-11
EVC2	-3,80308	5,15E-13
GLI3	-3,80411	1,84E-43
ARL4D	-3,81837	1,67E-31
MAPRE2	-3,82033	3,43E-32
SHOX2	-3,82124	1,55E-35
TMEM133	-3,82624	4,04E-05
HDAC4	-3,82672	1,78E-11
RPLP0P2	-3,83043	2,50E-11
SEC14L1P1	-3,83107	6,80E-06
SLC12A4	-3,83267	2,08E-10
RAB11FIP5	-3,83849	3,72E-19
ISPD	-3,85119	1,40E-05
CCDC106	-3,86041	1,91E-11
IL6	-3,86244	0,000234
CGNL1	-3,86263	7,65E-07
TCF7	-3,86487	5,22E-46
SH2B3	-3,86954	1,46E-50
EHD2	-3,86964	7,72E-66
RASA3	-3,8791	2,11E-48
COPZ2	-3,88535	3,08E-21
FAM171A1	-3,90173	2,11E-55
AMIGO2	-3,90287	1,48E-65
TRPC1	-3,90355	1,33E-17
MXRA7	-3,90956	1,08E-38
COL27A1	-3,92025	6,45E-15
IL4R	-3,93292	7,43E-27
C5orf56	-3,93653	2,53E-05
AKAP12	-3,94018	8,98E-36
GPNMB	-3,94093	1,06E-33
UPP1	-3,94351	1,76E-11
TRIM6	-3,94933	9,68E-06
SPHK1	-3,95457	9,26E-62
SPOCK1	-3,95684	2,56E-22
SUGCT	-3,96763	3,07E-08
ELK3	-3,977	1,72E-28
MAFB	-3,98747	3,10E-37
TRIB2	-3,98849	1,82E-41
DSC1	-3,99617	1,89E-08
MARCKS	-4,00348	1,76E-75
CHSY3	-4,02352	4,12E-22
DACT3	-4,0259	2,80E-05
HOXA1	-4,04308	2,48E-15
TBXA2R	-4,04478	0,00049
FAM72C	-4,055	9,82E-10
PRICKLE1	-4,06162	7,34E-10
IL1R1	-4,06203	3,21E-11
HOXA7	-4,06792	2,66E-07
CHST3	-4,07249	5,29E-12

RCAN1	-4,08598	6,85E-40
PPM1K	-4,0968	5,59E-20
UCHL1	-4,11548	5,68E-08
FOXD1	-4,12038	3,78E-12
ICAM1	-4,12178	9,35E-18
PTRF	-4,12421	1,62E-70
HOXA-AS2	-4,1257	4,53E-05
ZNF264	-4,12973	3,49E-30
IGFBP3	-4,13859	3,07E-14
BCAR3	-4,14113	2,20E-64
APLN	-4,14736	1,39E-05
SYNDIG1	-4,14823	4,08E-06
TAGLN	-4,15251	6,48E-22
FAM101B	-4,15382	9,01E-49
RAB3B	-4,16447	3,24E-36
ELMOD1	-4,16495	1,16E-05
PCDHB7	-4,17204	0,000137
TMEM255I	-4,17597	5,64E-05
CDC42EP3	-4,17844	1,23E-49
P3H1	-4,17953	1,10E-80
APBB1	-4,18303	2,26E-22
MX1	-4,19587	1,62E-19
TIMP2	-4,20348	2,54E-93
PAG1	-4,20449	3,71E-30
HOXC8	-4,21169	3,06E-08
F3	-4,2196	4,55E-25
FHOD3	-4,22999	2,81E-11
COL24A1	-4,23078	0,000276
ARHGEF6	-4,23575	1,96E-28
FZD8	-4,24149	1,55E-10
HMCN1	-4,24459	3,91E-37
NCF2	-4,25259	1,99E-05
CUBN	-4,26548	5,84E-05
TGFB2	-4,26797	1,54E-12
HBEGF	-4,28068	3,02E-42
SEMA3A	-4,28884	1,12E-10
BTN3A2	-4,29105	4,27E-12
CLIC2	-4,29483	8,79E-06
GPER1	-4,29536	1,45E-20
ZNF880	-4,29653	6,47E-14
KRT81	-4,31227	3,84E-05
PELI2	-4,31887	6,54E-05
S100A4	-4,31909	1,78E-50
CLCN4	-4,32167	3,33E-27
ITGB2	-4,33929	4,14E-10
SYNM	-4,34355	4,61E-41
APOL1	-4,36225	2,06E-06
NDNF	-4,3722	1,77E-39
MGLL	-4,3797	3,92E-27
EPHA2	-4,38078	6,37E-35

MEF2C	-4,39084	1,54E-26
PTGR1	-4,40329	2,70E-38
SLC16A3	-4,40509	1,26E-22
RUSC2	-4,41873	3,09E-44
MSRB3	-4,42689	4,42E-39
FAT4	-4,43277	6,37E-14
BIRC3	-4,4371	1,47E-09
IGF2BP2	-4,44229	1,48E-81
PCDH10	-4,44903	1,91E-07
SORBS2	-4,4581	1,01E-10
P2RX7	-4,45983	0,000505
EPDR1	-4,46229	5,97E-53
STARD9	-4,47031	2,41E-14
FILIP1L	-4,47055	4,10E-31
TBC1D2	-4,47344	6,76E-29
PLIN2	-4,47631	1,36E-57
SLC16A7	-4,49895	8,32E-12
ZNF221	-4,50344	3,11E-05
MEFV	-4,50876	3,14E-05
APOLD1	-4,52063	4,46E-08
C10orf11	-4,52885	1,26E-09
SLC2A3	-4,52978	1,28E-11
CPVL	-4,54273	8,96E-12
HOTAIRM1	-4,54842	6,14E-14
C17orf51	-4,55346	3,82E-25
PLEKHA4	-4,57246	1,77E-17
KCTD12	-4,57606	1,56E-44
CYP26B1	-4,58434	3,66E-10
ABCC9	-4,5883	5,61E-06
SNX7	-4,60947	1,13E-42
HDX	-4,64724	1,95E-08
SAMD9L	-4,64859	0,000215
ARMCX2	-4,6608	1,52E-30
LETM2	-4,66313	5,82E-22
WIPF1	-4,67487	1,30E-43
ZNF365	-4,69329	5,43E-09
ZNF513	-4,69425	3,14E-16
MICAL2	-4,70688	3,64E-72
PROS1	-4,71472	8,86E-11
GNB3	-4,72801	0,00089
LCAT	-4,73643	0,000151
ASPH	-4,7532	3,97E-70
TSPAN10	-4,77092	6,31E-15
VASN	-4,77361	1,31E-21
PCDHGC5	-4,77636	1,85E-08
TRIM9	-4,7779	5,24E-08
PTPN22	-4,78185	7,41E-11
CAV1	-4,79642	6,41E-62
DSE	-4,7983	6,47E-70
CAMK4	-4,80963	3,01E-14

PRRT3-AS1	-4,81588	0,000487
DUSP10	-4,81635	1,08E-25
KIAA1614	-4,81764	0,000443
ABLIM3	-4,81807	5,79E-37
PXK	-4,8213	1,47E-26
HOXB6	-4,83413	5,17E-10
SOX30	-4,84387	0,000824
DOCK10	-4,84912	2,42E-36
SH3RF3	-4,85431	7,61E-07
S100A2	-4,8559	6,86E-13
LOXL1	-4,87333	4,40E-71
UGT8	-4,88373	6,19E-05
ADAMTS15	-4,88848	1,03E-23
MCAM	-4,89488	4,60E-36
HLA-B	-4,89575	5,75E-27
NAP1L5	-4,89738	8,52E-06
C1R	-4,90316	4,88E-26
YOD1	-4,9058	1,06E-18
SELM	-4,91552	4,68E-89
ZNF284	-4,92084	3,24E-05
THBS1	-4,92542	1,24E-42
XKR6	-4,93088	1,91E-06
FAM46A	-4,9347	1,80E-53
PRR5L	-4,9361	3,64E-50
MLLT11	-4,94826	7,55E-40
ELFN1	-4,96944	1,36E-38
WLS	-4,99401	8,15E-64
LINC00673	-5,00378	3,10E-07
GNAI1	-5,01362	5,67E-51
SP100	-5,02581	0,000276
CORO2B	-5,02685	5,98E-11
EPB41L2	-5,04989	1,57E-100
GPR176	-5,05299	1,08E-34
ZDHHC2	-5,05872	2,46E-20
SYNE1	-5,06384	1,70E-31
SIRPA	-5,0767	1,80E-108
EDIL3	-5,08344	8,82E-17
CDKN1C	-5,10359	0,000981
ANGPTL4	-5,10505	8,25E-21
TPST1	-5,10702	5,36E-88
PGF	-5,14161	2,60E-15
MCOLN3	-5,1719	2,17E-06
NKX3-2	-5,17576	2,95E-12
SYTL3	-5,19074	4,85E-39
ST3GAL2	-5,2062	2,13E-55
KIF7	-5,21619	0,000243
GNG2	-5,21754	1,38E-06
EEPD1	-5,22012	8,38E-30
ULBP2	-5,22019	3,91E-15
RIN1	-5,22045	8,31E-38

PCSK5	-5,22414	0,000113
RNASEH2B-	-5,22915	0,000145
SPRED1	-5,22946	1,61E-41
NEK10	-5,24067	7,90E-05
DNM3	-5,24117	1,55E-10
IRAK2	-5,24288	1,10E-06
FHL2	-5,2435	9,95E-20
ZC3H12C	-5,24755	8,98E-28
PLCE1	-5,25187	2,00E-17
PSMB9	-5,25622	1,99E-30
MN1	-5,25623	1,90E-08
IL15	-5,25794	0,00044
MRVI1	-5,25805	5,36E-06
SPRY4	-5,26824	4,30E-32
PMP22	-5,27766	1,46E-37
TRO	-5,28248	3,59E-06
LYN	-5,28681	1,41E-34
C3orf52	-5,29382	6,33E-15
COL16A1	-5,29428	8,34E-32
FXYD5	-5,2951	5,81E-108
HCP5	-5,31298	5,88E-09
NTNG1	-5,31311	5,09E-05
EGFR	-5,3154	5,48E-83
TRABD2A	-5,32098	7,56E-09
ANKRD33B	-5,32506	8,59E-16
DSEL	-5,34848	4,85E-39
CAPN2	-5,35547	2,99E-37
APOL3	-5,36835	3,38E-05
COL5A2	-5,37812	2,33E-99
SLC1A3	-5,38516	2,07E-79
STEAP2	-5,42249	5,14E-07
SPTLC3	-5,43443	1,75E-17
GRASP	-5,44644	0,000153
SMARCA1	-5,44737	5,64E-52
PIK3CD	-5,45822	2,72E-36
CTSS	-5,46936	6,96E-07
CD274	-5,47644	7,17E-07
CHN2	-5,47803	2,20E-07
EVA1A	-5,48421	3,73E-24
CRYBG3	-5,48819	3,98E-35
VIM-AS1	-5,5211	8,88E-74
RFTN1	-5,52427	4,30E-63
WNT5B	-5,52458	3,70E-40
LIX1L	-5,53403	1,25E-68
LAMC2	-5,53631	5,27E-65
POPDC3	-5,5481	2,10E-15
BAIAP2L2	-5,57297	6,64E-08
ARHGEF40	-5,57486	3,01E-31
PTGFR	-5,59798	0,000159
TGM2	-5,60552	2,49E-24

IL21R	-5,60556	2,65E-24
ANGPT1	-5,61289	7,90E-27
TM4SF1	-5,62071	4,31E-73
ZNF528	-5,62437	2,00E-13
CD109	-5,63872	1,20E-55
ME3	-5,65962	2,17E-15
SHISA2	-5,67418	2,08E-71
COL6A2	-5,6894	3,68E-128
MTAP	-5,7036	1,34E-29
EGFLAM	-5,71655	6,53E-29
PDZRN3	-5,71904	6,49E-118
SERPINE2	-5,72005	1,09E-100
CALD1	-5,72025	1,13E-31
FPR1	-5,72498	1,31E-07
LOX	-5,73454	2,62E-88
RBMS3	-5,75336	3,87E-17
PNPLA3	-5,76167	3,53E-06
ENPP1	-5,77104	6,67E-45
LOC79160	-5,77412	0,00037
APOL6	-5,80119	2,00E-44
HAS2	-5,80453	2,43E-07
DRAXIN	-5,80835	9,47E-06
C1QTNF2	-5,80836	2,93E-13
HTR7	-5,81065	5,42E-25
LAPTM5	-5,81286	2,77E-06
GLIPR1	-5,81445	1,80E-41
ARHGAP23	-5,83787	1,34E-44
APCDD1L-A	-5,84021	0,00021
MAF	-5,84186	8,50E-50
VEGFC	-5,85169	4,91E-32
B3GNT9	-5,85201	3,34E-26
ARHGAP22	-5,85416	3,72E-13
SLC7A7	-5,85457	7,43E-06
NFASC	-5,86013	4,18E-10
C10orf10	-5,86733	0,000182
PYGL	-5,88987	4,53E-06
SEC16B	-5,89772	1,33E-05
RAB32	-5,90382	1,51E-51
IGF2BP1	-5,91253	2,47E-35
CD68	-5,94497	7,74E-39
TUBA4A	-5,98675	3,03E-30
TP73-AS1	-5,99693	1,29E-10
LOC101927	-6,001	1,78E-17
LOXL2	-6,00256	2,20E-28
TMEM45A	-6,01648	9,51E-34
SCARF2	-6,0499	1,36E-103
NLRC5	-6,05032	1,36E-34
HOXA2	-6,05785	2,09E-13
SYNC	-6,06042	1,17E-67
DACT1	-6,06401	5,24E-22

MAP1A	-6,06771	3,65E-36
KLHL4	-6,07588	4,95E-05
ADAMTS6	-6,0812	0,000105
RCN3	-6,09623	9,36E-56
AOX1	-6,13341	9,50E-06
FHL1	-6,14218	1,16E-79
GAS1	-6,14825	2,52E-19
MCTP2	-6,15525	4,82E-16
SNPH	-6,15785	6,49E-07
PLA2G4A	-6,16101	8,44E-05
PLCL2	-6,16533	2,20E-11
RIN2	-6,18639	4,89E-72
ACAN	-6,20956	2,04E-05
PHACTR1	-6,23358	2,25E-05
C1S	-6,24673	2,83E-14
DNM3OS	-6,24868	2,93E-07
ADAM19	-6,27079	6,52E-142
ECM1	-6,27968	4,98E-31
ZNF469	-6,31945	2,87E-42
MYLK	-6,32017	1,02E-135
PLEKHG4B	-6,32998	1,80E-51
CSF1	-6,33165	2,25E-85
SLC4A4	-6,3371	6,21E-11
GEM	-6,34737	5,08E-89
EMILIN2	-6,36931	2,39E-46
HLA-H	-6,39662	5,49E-06
TMEM200E	-6,40084	2,62E-13
FENDRR	-6,44181	1,33E-13
MRC2	-6,45537	1,56E-55
FOSL1	-6,47656	3,43E-64
COL7A1	-6,50659	1,43E-39
SOGA3	-6,51368	5,70E-12
CUX2	-6,5221	2,98E-06
C1RL	-6,52601	1,72E-10
GRIK2	-6,53113	2,81E-06
CTHRC1	-6,55624	1,14E-69
PRRX2	-6,57181	4,29E-37
LMO7-AS1	-6,57331	2,84E-15
BEAN1	-6,57441	0,000733
CSF1R	-6,57523	1,06E-10
GABRQ	-6,58273	1,19E-11
PLAUR	-6,5891	4,63E-50
C11orf70	-6,5988	0,000657
NRROS	-6,60863	4,76E-12
P2RY1	-6,61232	1,61E-06
DOCK2	-6,61403	6,60E-09
ROBO1	-6,62579	3,86E-68
ADM	-6,63322	1,10E-48
FLJ22447	-6,63344	2,10E-27
DPP4	-6,63643	1,87E-17

LINC00862	-6,64979	3,37E-25
CYS1	-6,66726	0,000681
GJA1	-6,69949	8,53E-13
CDH2	-6,70258	2,80E-50
ANTXR2	-6,70861	2,77E-27
SLIT3	-6,73095	1,28E-11
BICC1	-6,75315	1,50E-56
SPOCD1	-6,78642	4,00E-05
ACOT2	-6,79218	2,57E-29
ZNF426	-6,79293	2,07E-13
NLRP1	-6,79478	0,000387
CRYGS	-6,79508	3,01E-14
SVEP1	-6,79719	1,20E-17
SH3PXD2A	-6,80971	4,76E-120
HAPLN3	-6,83208	5,75E-16
BEX1	-6,83778	0,000279
ROR2	-6,87068	4,43E-24
PRKCA	-6,88573	1,16E-170
CGB5	-6,88718	0,000222
TGFB1I1	-6,89036	2,44E-06
MID1	-6,89723	2,64E-72
PTGS2	-6,90154	2,10E-11
FBXO32	-6,90842	2,49E-63
TNFRSF11B	-6,92891	4,07E-125
LINC00857	-6,95448	1,34E-07
TDO2	-6,96113	0,00017
INPP1	-6,99545	5,57E-07
ZNF717	-6,99946	1,27E-07
LOC101928	-7,014	2,78E-19
DKK3	-7,0309	4,22E-94
NEK9	-7,05967	2,84E-05
TMBIM1	-7,06794	4,43E-45
HOXD11	-7,06841	7,45E-28
KIFC3	-7,06918	6,19E-50
ITGA5	-7,07486	1,07E-232
SPANXB1	-7,09995	0,000102
COL5A1	-7,111	2,34E-86
IL11	-7,13434	4,61E-43
OLFML2B	-7,13886	3,26E-40
BDNF	-7,14259	9,21E-48
ETV5	-7,14888	2,62E-88
EPAS1	-7,15406	3,05E-86
LINC01605	-7,16723	7,77E-47
C1QTNF1	-7,16929	1,59E-19
TNFAIP2	-7,16999	7,60E-54
MPP1	-7,17991	3,11E-60
ABHD8	-7,18051	2,29E-61
BIN1	-7,18395	1,30E-97
ST6GAL2	-7,1907	2,74E-19
ZBTB18	-7,20582	6,82E-49

PAPPA2	-7,21227	6,66E-05
TMTC1	-7,22537	1,21E-11
TBX15	-7,23373	3,40E-37
ZNF521	-7,25236	3,07E-15
PLXDC1	-7,25889	6,44E-05
FMN2	-7,31118	4,31E-20
ALDH2	-7,31425	1,24E-61
DNER	-7,31682	3,31E-12
BRINP1	-7,33328	9,11E-09
ENPP2	-7,3371	9,82E-19
TMEM173	-7,33785	6,58E-05
DAB2	-7,34277	3,47E-40
MILR1	-7,35876	4,01E-05
GSTP1	-7,37214	4,75E-58
DPYSL3	-7,38107	1,38E-148
GIPC2	-7,38795	0,000997
ZEB1	-7,39875	1,50E-49
NALCN	-7,40245	0,000943
HEG1	-7,40948	7,98E-07
ADRA1D	-7,41557	2,61E-05
MIR221	-7,41638	0,000817
PRSS12	-7,4168	6,49E-13
BMPER	-7,42087	0,000814
ZBTB20	-7,42134	1,46E-36
SV2A	-7,43046	3,47E-79
RRAD	-7,43771	0,000811
KCNJ12	-7,44866	0,000757
ANXA1	-7,44953	1,56E-246
ADAMTS10	-7,45305	0,000691
MUC13	-7,45609	0,00068
TNIP3	-7,46121	2,50E-09
JAZF1	-7,47226	6,17E-66
PLSCR4	-7,47436	2,04E-05
FGF2	-7,47633	3,29E-16
UCN2	-7,47946	2,75E-05
LINC01291	-7,48452	0,000608
ZNF69	-7,48582	0,000662
PPP1R3C	-7,49029	2,75E-09
ZNF788	-7,49066	2,56E-13
PPP1R3G	-7,49407	2,25E-05
GCNT2	-7,50485	7,05E-13
MICU3	-7,50653	0,000554
PWAR5	-7,5124	0,000552
LOC101927	-7,52003	0,000823
FLJ31356	-7,52657	0,000599
COL5A3	-7,53111	0,000501
VCAN	-7,53707	1,66E-67
LURAP1L-A	-7,54304	1,89E-05
CYP27C1	-7,54728	0,000811
ZNF660	-7,54958	0,00046

CDA	-7,55693	0,000589
HLX	-7,56282	1,04E-09
ZSCAN23	-7,57224	0,000492
ABCA13	-7,59415	0,00038
CSRP3	-7,60405	0,000507
LINC00693	-7,60791	0,000383
GAPLINC	-7,60911	0,000362
LINC00622	-7,61944	6,22E-10
LINC01204	-7,62001	0,000342
LYNX1	-7,62158	0,000433
SELPLG	-7,6258	7,48E-19
IL10	-7,63044	0,000422
ROS1	-7,63083	0,000351
TMEM236	-7,63315	0,000397
DCN	-7,63521	0,000549
BAALC	-7,63892	0,000376
ECM2	-7,63892	0,000376
LOC100128	-7,64391	0,000329
FLJ32255	-7,65448	8,90E-22
SEMA5A	-7,65455	0,000294
ARHGAP20	-7,65605	0,000329
ITK	-7,66733	0,000395
KCNT2	-7,67299	0,000292
ABI3BP	-7,67299	0,000292
GCNT4	-7,67823	0,000304
FOXQ1	-7,67976	5,23E-22
HS3ST2	-7,69233	0,000265
PRDM8	-7,69233	0,000265
FAM225B	-7,69455	0,000307
NOX4	-7,69491	0,000269
DHRS9	-7,69604	0,000244
LOC101929	-7,7093	0,00055
LINC01138	-7,71013	2,81E-10
JPH2	-7,71673	7,48E-06
CLIP4	-7,72071	5,19E-23
LINC00942	-7,72092	7,67E-06
CPNE5	-7,72477	0,000329
ZNF625-ZN	-7,72732	0,000307
PAQR9	-7,74992	0,000197
C7orf57	-7,74992	0,000197
TFCP2	-7,75602	0,000325
ACTC1	-7,76007	0,000581
CARD16	-7,76334	0,000197
HEPH	-7,76432	0,000272
KLHL38	-7,76616	0,000232
IL7	-7,77908	0,000192
COL4A2-AS	-7,78491	0,000239
CFI	-7,79552	0,000283
GABRA3	-7,79832	0,000875
MT1L	-7,80602	0,000349

LINC00460	-7,82324	0,00014
HRCT1	-7,82324	0,00014
PADI1	-7,83233	0,000157
PDGFD	-7,83834	0,000129
MME	-7,8439	4,27E-06
C10orf90	-7,84785	4,00E-06
LRCH2	-7,84867	0,000123
SLFN13	-7,85098	0,000121
NRP2	-7,85586	2,41E-108
KCNJ2	-7,86447	0,000147
HHIP-AS1	-7,8648	0,00012
AIM2	-7,86541	0,000279
BHLHE41	-7,86806	3,91E-06
VGLL2	-7,86938	0,000123
DOK5	-7,87265	0,000109
RNF217	-7,874	1,66E-15
KLHL3	-7,87684	0,000157
HOXB3	-7,87946	1,67E-15
ADTRP	-7,88141	0,000138
LOC101928	-7,884	0,000111
HCRTR2	-7,90296	0,000103
TICAM2	-7,90839	9,22E-05
ARHGAP31	-7,91725	9,25E-05
APCDD1L	-7,91857	0,000125
BTBD19	-7,92574	9,47E-06
TSLP	-7,92922	8,42E-05
COL12A1	-7,94168	1,97E-65
CD70	-7,94306	4,93E-11
MMP1	-7,94725	0,00011
APOBEC3C	-7,94797	1,01E-34
HSPB3	-7,96239	7,74E-05
CXCL5	-7,96832	0,000143
LINC01239	-7,97342	0,000104
PID1	-7,97563	1,89E-16
GPR85	-7,97676	3,04E-06
FBN1	-7,97736	6,91E-159
DGKG	-7,97817	2,96E-11
TMEM100	-7,98193	0,000148
OSBPL3	-7,98402	5,20E-56
APBA2	-7,98438	0,000642
TMEM26	-7,99418	7,13E-05
LOC101060	-7,99628	7,33E-05
AGTR1	-8,00482	3,73E-06
CCBE1	-8,02678	3,56E-25
ZNF625	-8,02911	6,23E-05
KRT34	-8,04069	7,99E-05
FOXF1	-8,04214	1,13E-05
LOC152225	-8,04284	0,000142
DMD	-8,04347	8,23E-05
NPR2	-8,04431	1,65E-06

SMO	-8,04479	1,84E-16
FOXC2	-8,04536	1,61E-06
CAND2	-8,04763	1,65E-06
C15orf52	-8,06515	2,40E-06
ADAMTS14	-8,07758	1,80E-06
LOC101927	-8,0915	3,81E-05
TRIM38	-8,10926	8,69E-05
SCUBE1	-8,1118	3,54E-05
LINC00944	-8,11565	3,61E-05
FAM155A	-8,1165	3,37E-05
RGCC	-8,1201	9,23E-84
GNG11	-8,12319	7,66E-12
PDE3A	-8,15987	2,87E-05
PDZRN3-AS	-8,15987	2,87E-05
CACNA1A	-8,16409	4,12E-05
CHI3L1	-8,16441	2,65E-05
HDAC9	-8,16463	1,21E-38
ZNF582	-8,16971	3,46E-05
LINC00702	-8,16998	2,63E-05
C11orf45	-8,19002	2,35E-05
PTX3	-8,20124	6,39E-06
USP44	-8,20718	2,15E-05
LOC102467	-8,22235	1,99E-05
LRRC17	-8,22693	1,33E-32
CTB-113P1	-8,23281	2,22E-05
RAB38	-8,23459	2,14E-05
LHX8	-8,23587	4,58E-05
GCNT3	-8,23659	2,09E-05
DUSP5P1	-8,23814	1,99E-05
CRYBA2	-8,23991	1,93E-05
CXCL2	-8,24127	3,61E-05
RAB7B	-8,24898	7,00E-07
CST7	-8,25077	2,67E-05
APOBEC3G	-8,29324	1,49E-05
EMILIN1	-8,29719	4,88E-112
HS3ST3A1	-8,30528	5,29E-59
PRDM1	-8,31137	1,37E-12
TNFAIP6	-8,31427	1,26E-05
LAT	-8,3192	1,43E-08
TM4SF19	-8,32239	1,57E-05
ENG	-8,3389	2,53E-149
EPHA5-AS1	-8,34868	2,94E-05
TUBA3FP	-8,35585	1,53E-05
IFFO1	-8,36636	3,39E-17
HHIP	-8,36683	1,30E-05
C20orf197	-8,36955	1,39E-05
C5orf17	-8,37234	1,07E-05
TMEM220	-8,38268	9,72E-06
ZNF501	-8,38568	9,13E-06
LOC101926	-8,40313	8,24E-06

ZNF844	-8,4146	7,08E-07
HACD4	-8,42486	7,16E-06
CHRDL1	-8,42621	8,62E-06
CNTN1	-8,4385	2,98E-07
CRYBB1	-8,45585	8,74E-06
LINC01411	-8,49535	1,20E-05
UBA7	-8,50443	5,13E-06
PSORS1C1	-8,51012	4,56E-06
LINC01561	-8,52117	5,19E-06
NLGN1	-8,52533	4,13E-06
LRRN3	-8,52823	4,07E-06
WT1-AS	-8,54697	8,54E-06
CNKSR2	-8,55221	8,38E-06
ZFHX4-AS1	-8,55936	3,84E-06
DDO	-8,56277	4,48E-06
ZNF577	-8,56561	8,52E-06
SSC5D	-8,56777	1,87E-40
ZNF667	-8,56987	1,23E-05
SCG5	-8,57997	1,92E-07
SLCO1B3	-8,58183	3,29E-06
AFP	-8,58862	3,02E-06
KLRC2	-8,58922	3,49E-06
RAET1E	-8,58939	3,04E-06
CCDC80	-8,60065	1,35E-150
CYP7B1	-8,60577	2,71E-06
ADRA1B	-8,60792	3,07E-06
LBH	-8,62224	7,43E-14
SLC16A2	-8,63169	3,04E-142
TENM2	-8,63293	0,000509
VSNL1	-8,63832	6,60E-14
ITGA1	-8,63896	1,58E-07
LONRF3	-8,64216	8,20E-14
MEIS2	-8,64327	5,54E-05
FBLN1	-8,64459	2,68E-06
GBP5	-8,65615	1,73E-06
LHFP	-8,66422	1,70E-48
TMEM255/	-8,66721	2,57E-06
SAA1	-8,68048	2,35E-06
TNFSF12	-8,68531	2,18E-06
NETO1	-8,69052	2,44E-06
DOK6	-8,70485	3,86E-06
LPAR4	-8,70559	1,60E-06
LOC72997(	-8,70815	1,56E-06
ADGRE1	-8,70815	1,56E-06
ZC3HAV1L	-8,74207	1,47E-06
TMEM200(	-8,76009	2,10E-06
FGF5	-8,8063	1,19E-06
PINLYP	-8,80978	6,13E-06
ITGB3	-8,81617	4,26E-08
PDGFRA	-8,81648	5,41E-21

SULT1C2	-8,81761	1,48E-06
PPARGC1A	-8,82664	8,62E-07
FAM196B	-8,83215	5,71E-08
ZNF671	-8,85121	1,39E-06
ZC4H2	-8,85393	6,33E-15
LPPR4	-8,85395	7,61E-07
C10orf55	-8,86095	1,28E-06
BNC2	-8,87849	2,47E-08
ZNF280B	-8,89401	5,73E-07
ARMCX4	-8,90446	2,04E-08
MAB21L1	-8,9187	5,86E-07
MDFI	-8,93692	9,40E-07
AMPH	-8,94551	5,16E-08
LINC01094	-8,94713	4,95E-07
PHLDB2	-8,94818	1,49E-45
ZNF71	-8,94938	3,38E-15
FAM133A	-8,96668	9,06E-07
SULF1	-8,9775	1,90E-08
ANKRD1	-8,97884	1,90E-08
CD40	-8,9799	3,54E-07
ZKSCAN7	-8,98036	3,95E-07
ZFP28	-8,98096	3,50E-07
CATSPER1	-8,98564	3,52E-07
GBP1	-8,98568	4,69E-13
MAMDC2	-8,99073	4,39E-07
ARHGAP28	-9,02853	2,99E-07
SLC43A3	-9,03019	0,000172
HOXB5	-9,04163	2,63E-07
EMP3	-9,07238	1,70E-74
TNFSF10	-9,07819	1,15E-08
IPW	-9,089	1,98E-07
RUNX1T1	-9,09167	2,55E-07
RGS4	-9,1035	3,84E-07
ATP6V0D2	-9,1246	1,55E-07
ADAMTS5	-9,12473	1,81E-07
COL3A1	-9,1295	1,79E-141
LINC00601	-9,13337	2,25E-07
CFH	-9,17538	4,44E-15
PTPRR	-9,22608	1,21E-07
LOC10037S	-9,23952	8,11E-08
BVES	-9,26158	9,11E-08
ARSJ	-9,27403	1,50E-23
CDO1	-9,28823	6,11E-08
RGS20	-9,28896	7,70E-08
PDCD1LG2	-9,30511	5,51E-08
MDFIC	-9,30664	4,39E-22
ZNF528-AS	-9,31471	1,20E-07
MIR646HG	-9,31857	1,51E-07
SLC2A5	-9,32622	5,56E-08
ANXA8L1	-9,34467	5,14E-08

NDN	-9,35006	4,26E-08
LOC100507	-9,35026	6,29E-08
SLA	-9,35124	4,38E-08
GJC1	-9,35172	1,01E-31
PDLIM4	-9,35836	6,79E-125
FOXL2NB	-9,35857	4,07E-08
SGCD	-9,35857	4,07E-08
EDNRA	-9,37165	5,11E-08
ZNF503-AS	-9,37662	3,66E-08
RUNX3	-9,38388	1,76E-31
ZNF667-AS	-9,38932	3,40E-08
MPP4	-9,39047	3,45E-08
CACNA1C	-9,40322	2,27E-09
TNC	-9,42416	3,02E-56
SLC9A9	-9,42487	2,85E-08
C3AR1	-9,42866	2,74E-08
WFDC21P	-9,44244	3,82E-17
EMP1	-9,44999	2,24E-17
LUM	-9,45173	8,57E-17
HOXD-AS2	-9,45392	2,60E-08
ZFPM2-AS1	-9,46802	2,55E-08
PAPPA	-9,47417	3,80E-08
HOXD10	-9,48904	1,86E-17
RFX8	-9,48915	1,17E-07
LOC102477	-9,49188	1,88E-08
GPX7	-9,49693	3,15E-08
FGF1	-9,512	1,69E-08
IL31RA	-9,52076	1,84E-08
ILDR2	-9,52153	5,59E-55
OSR1	-9,54387	1,14E-09
ALX4	-9,54888	1,35E-08
SLITRK3	-9,57726	1,15E-08
SDHAF3	-9,62158	1,00E-08
NGF	-9,62629	1,46E-08
GALNT13	-9,63663	9,38E-09
EVI2B	-9,64026	9,62E-09
DKK2	-9,64504	1,45E-08
GLIS3	-9,65169	1,19E-07
NTF3	-9,65285	8,42E-09
GPC6	-9,65625	1,71E-26
MAB21L2	-9,65758	7,45E-09
ZNF470	-9,66013	1,30E-08
PSMB8	-9,69702	1,15E-34
AGMO	-9,69862	5,70E-09
CASP1	-9,70018	5,76E-09
ADAMTS16	-9,70083	5,70E-09
ZCCHC5	-9,71162	5,34E-09
ZNF229	-9,7167	5,17E-09
EPSTI1	-9,7175	1,79E-18
FLI1	-9,71799	5,83E-10

FN1	-9,74477	1,08E-195
PDE1C	-9,7486	5,48E-09
SERPINB7	-9,75142	5,08E-09
TOX	-9,77003	5,17E-09
SNCA	-9,8097	3,00E-09
CLMP	-9,81177	9,13E-35
TLR6	-9,82805	3,16E-09
NTM	-9,84028	2,72E-09
COL8A1	-9,8542	5,10E-19
MAP1LC3A	-9,8681	2,13E-09
GIPC3	-9,88233	2,28E-09
ANKRD30B	-9,88631	2,53E-09
SERPINE1	-9,91486	3,01E-11
FOXL1	-9,91506	1,75E-09
PTPRN	-9,92118	2,13E-10
ZFPM2	-9,92547	1,71E-09
ADAMTS3	-9,92554	1,82E-09
DGKI	-9,92982	2,08E-09
SCG2	-9,9308	1,47E-09
LAMA4	-9,95567	1,76E-09
GDF6	-9,95995	1,55E-10
HOXD13	-9,96317	1,50E-09
LPAR3	-9,97018	1,16E-09
MIR31HG	-9,97496	1,14E-09
ZNF662	-9,98712	1,35E-09
ZNF626	-10,0259	8,34E-10
ZMIZ1-AS1	-10,0329	9,71E-10
LINC00941	-10,0373	1,34E-09
BARX1	-10,0607	7,51E-10
AGPS	-10,0694	2,80E-59
ETV1	-10,0847	6,61E-11
GSPT2	-10,0852	8,05E-10
CDKN2A	-10,0855	5,60E-20
TMEM200/	-10,0936	7,07E-10
NEXN	-10,1082	2,40E-29
LHFPL4	-10,1523	5,29E-10
ZNF804A	-10,1554	7,11E-10
NEGR1	-10,1607	3,87E-10
AFAP1L1	-10,1608	1,95E-20
KCNJ15	-10,1651	3,74E-10
CCDC152	-10,1763	4,00E-10
TMEM108	-10,1775	3,57E-10
SYT14	-10,179	6,62E-10
CSPG4	-10,1813	1,20E-07
MIR100HG	-10,1815	5,56E-11
ZNF300P1	-10,1841	3,76E-10
BDKRB1	-10,1847	4,11E-10
ZNF132	-10,1909	3,21E-10
LDHB	-10,1964	0,000484
CDH13	-10,1987	3,82E-10

ZNF502	-10,2002	3,08E-10
NEFL	-10,2023	3,36E-10
NID2	-10,2088	3,68E-10
HOXD9	-10,2147	7,90E-10
TWIST1	-10,218	4,29E-30
P3H3	-10,2184	8,58E-11
S1PR1	-10,2209	4,74E-10
ZNF320	-10,2257	4,11E-10
PSG5	-10,2405	2,46E-10
IGFN1	-10,2454	6,84E-10
FAM26E	-10,2579	2,37E-10
DEPDC7	-10,2736	1,95E-10
CREB3L1	-10,2958	2,11E-129
FNDC1	-10,3104	1,13E-09
ZNF559	-10,3139	1,56E-10
MKRN3	-10,3311	1,42E-10
SLFN12	-10,3382	1,40E-10
TPM2	-10,3608	5,45E-227
ARSE	-10,4006	1,20E-10
PKIA	-10,416	9,48E-11
STK33	-10,4248	1,10E-10
ZNF175	-10,4385	1,24E-10
CPA4	-10,4446	1,84E-31
AKR1B1	-10,4465	1,79E-59
ZNF583	-10,4582	8,88E-11
LY96	-10,4633	6,89E-11
GPR183	-10,4814	5,88E-11
PDPN	-10,4855	5,96E-11
LINC00565	-10,4887	5,46E-11
PLAT	-10,4973	8,16E-11
ZNF773	-10,5363	4,14E-11
BCHE	-10,5638	3,50E-11
GLT8D2	-10,5901	3,80E-11
SALL1	-10,6047	3,08E-11
ZNF649	-10,6067	4,61E-11
CTSZ	-10,6076	5,17E-133
LDB2	-10,6104	4,14E-11
LRRC4	-10,6337	2,35E-11
LINC01279	-10,6437	2,74E-11
ADAMTS2	-10,6595	9,86E-23
CA13	-10,6953	1,84E-11
GBP3	-10,7233	2,28E-12
PTN	-10,7256	1,84E-11
ZNF506	-10,7352	2,52E-11
DMRTA2	-10,7516	1,89E-12
CARD6	-10,7584	1,29E-11
MOXD1	-10,7597	1,26E-11
SPAG16	-10,7732	1,01E-11
ZFP82	-10,7748	1,25E-11
COL4A2	-10,8125	9,47E-130

ADAM12	-10,8429	9,49E-65
STK32B	-10,857	6,11E-12
AASS	-10,8859	5,16E-12
FIGN	-10,8971	5,40E-12
TWIST2	-10,9094	4,48E-12
SIGLEC15	-10,93	4,03E-12
EVI2A	-10,9312	4,05E-12
LINC01444	-10,9362	3,83E-12
TNFRSF1B	-10,9369	3,83E-12
SP140	-10,9786	3,82E-12
CLEC2B	-10,9795	3,99E-12
KCNE4	-10,99	4,44E-12
POSTN	-10,991	3,89E-12
FSTL1	-10,9948	2,43E-166
DLX6	-10,9951	2,95E-12
FRG1CP	-11,011	2,45E-12
AGT	-11,0151	2,90E-12
CDIP1	-11,0165	2,83E-12
CHST2	-11,0791	1,75E-12
ITGBL1	-11,1165	1,42E-12
ZNF542P	-11,1306	1,33E-12
PRRX1	-11,1734	9,31E-13
PRKCDBP	-11,1854	8,64E-13
LINC00667	-11,1869	9,73E-13
GREM2	-11,2219	8,14E-13
HMGA2	-11,2274	1,42E-13
SERPINB2	-11,2411	8,25E-13
GYPC	-11,2704	5,21E-13
FOXL2	-11,2767	5,15E-13
C5orf42	-11,2816	4,91E-13
TGFBI	-11,289	2,97E-40
ARSI	-11,3232	4,20E-13
C7orf31	-11,328	5,27E-13
PIEZO2	-11,3389	3,92E-13
SRGN	-11,3513	7,33E-14
ZNF300	-11,357	4,56E-13
GPR1	-11,3584	3,06E-13
WT1	-11,3653	2,97E-13
FLRT2	-11,3862	7,42E-14
SMIM10	-11,4256	2,28E-13
TLE4	-11,4343	4,77E-14
MARCH4	-11,4412	2,01E-13
LPAR1	-11,4489	2,24E-13
CGB8	-11,4637	1,62E-13
MMP14	-11,4802	3,15E-06
EBF1	-11,4924	2,55E-13
MSC-AS1	-11,4935	1,46E-13
PSG4	-11,5123	1,35E-13
THBS2	-11,5155	1,23E-13
STAC	-11,6086	7,19E-14

HECW1	-11,6152	9,29E-14
PAX6	-11,6155	6,91E-14
MAGI2-AS3	-11,6544	5,23E-14
MEOX2	-11,6565	5,37E-14
EYA1	-11,6947	4,48E-14
ZIK1	-11,6976	4,66E-14
GDNF	-11,7075	3,74E-14
INHBA	-11,7094	4,34E-14
NKX2-5	-11,7153	3,89E-14
SRPX2	-11,7155	5,29E-14
C8orf88	-11,7297	3,26E-14
DPYD	-11,7616	2,77E-14
EPHA5	-11,7799	2,48E-14
LAMA1	-11,9017	2,12E-14
TNFRSF10D	-11,9178	1,18E-14
MSN	-11,9413	1,04E-12
FAP	-11,9807	2,78E-14
CDH11	-12,0131	8,61E-15
GNE	-12,0409	5,82E-15
PTPRM	-12,0443	6,56E-15
IFI16	-12,0507	2,28E-29
MMP2	-12,0628	9,85E-43
CYTL1	-12,0899	4,82E-15
ARHGAP31	-12,09	1,13E-15
VGLL3	-12,1194	8,53E-15
COL4A1	-12,1502	1,24E-43
LRRC15	-12,1697	2,34E-15
DFNA5	-12,1794	2,06E-15
EFEMP2	-12,2019	1,90E-15
GRAMD1B	-12,2084	1,74E-15
EMC10	-12,2538	1,87E-15
EYA4	-12,2702	1,21E-15
FBXL7	-12,2788	1,20E-15
GLIS1	-12,2811	1,58E-15
EPHA3	-12,3067	9,65E-16
ZCCHC11	-12,3412	7,90E-16
VSTM4	-12,3593	7,06E-16
HRH1	-12,4047	5,27E-16
CYBRD1	-12,4117	5,48E-16
ZNF518B	-12,4262	4,96E-16
SLC35G2	-12,4474	3,99E-16
FOXG1	-12,4691	4,46E-16
IGFBP7	-12,4873	7,73E-16
HOXA10	-12,4915	2,96E-16
GALNT5	-12,5189	2,96E-16
ALPK2	-12,5458	2,15E-16
ITGA4	-12,5491	2,14E-16
DDR2	-12,5532	2,01E-16
PVRL3	-12,587	1,76E-16
KANK4	-12,5922	1,66E-16

AK5	-12,5943	1,59E-16
ATP10A	-12,6394	1,18E-16
MMP3	-12,6585	2,24E-16
LZTS1	-12,6955	8,82E-17
TLR4	-12,7471	8,61E-17
NRN1	-12,7494	6,52E-17
SIGMAR1	-12,7552	6,07E-33
PDGFRB	-12,8091	4,01E-17
SGCE	-12,8374	3,36E-17
C2CD2	-12,84	3,30E-17
ITGA11	-12,8567	4,00E-17
MSC	-12,9708	1,50E-17
EVC	-12,9758	1,52E-17
TCF4	-13,0621	8,29E-18
CNRIP1	-13,1081	6,77E-18
SUSD5	-13,1115	6,75E-18
ZEB2	-13,1449	8,01E-18
NRIP3	-13,2443	2,60E-18
TCEAL8	-13,2478	2,50E-18
AKT3	-13,251	3,95E-18
MXRA8	-13,2648	2,18E-18
IL7R	-13,3025	1,73E-18
LAYN	-13,326	1,71E-18
IGF2BP3	-13,3443	1,31E-18
PPAPDC1A	-13,4133	8,29E-19
RAC2	-13,4229	7,75E-19
WISP1	-13,4272	8,80E-19
AXL	-13,547	1,05E-53
ANGPTL2	-13,5528	3,71E-19
NNMT	-13,5733	2,91E-19
KIAA1644	-13,5996	2,49E-19
TMEM246	-13,6746	1,56E-19
TNFRSF9	-13,7662	8,08E-20
EPB41L3	-13,769	9,24E-20
MT1E	-13,7799	7,70E-20
ZFHX4	-13,8183	5,75E-20
ADAMTS1	-13,8528	1,54E-20
NID1	-13,9073	3,54E-20
FOXF2	-13,9445	2,46E-20
ANPEP	-14,0927	1,01E-20
ETS1	-14,2401	3,69E-21
COL6A3	-14,2872	7,57E-139
HSPB7	-14,3247	1,92E-21
WBP5	-14,339	1,78E-21
NRG1	-14,5311	5,07E-22
PLAU	-14,5707	3,55E-22
DZIP1	-14,5835	3,35E-22
CNN3	-14,6273	8,72E-23
MYL9	-14,6755	1,70E-22
GPX1	-14,7816	1,06E-22

CDK15	-14,7895	9,11E-23
NT5E	-15,0776	1,02E-23
KIRREL	-15,1678	5,89E-24
SNAI2	-15,3398	1,74E-24
COL1A2	-16,6956	7,50E-29
FLNC	-17,0246	4,70E-30
SPARC	-17,7422	1,25E-32

UO31

Gene	log2FoldCh	FDR
APCDD1	16,25276	1,73E-27
BMP7	15,74618	5,48E-26
FBP1	14,90931	4,17E-23
TFF1	14,57842	6,32E-21
TSPYL5	14,36734	1,06E-21
CDH3	14,23128	1,64E-21
SYT7	14,18583	3,80E-21
GRHL2	14,06451	2,26E-20
RAB25	14,02088	1,64E-20
ATP2A3	13,93789	8,17E-07
MYH14	13,88023	1,40E-19
ESRP1	13,84411	3,83E-20
TJP3	13,52048	5,42E-18
TMEM30B	13,22945	2,70E-18
LRFN4	13,14899	3,19E-18
UQCRH	13,08733	2,67E-18
DSCAM-AS	13,07804	1,71E-10
RTN4RL1	12,85922	2,19E-17
BSPRY	12,81131	4,10E-17
CACNA1H	12,75613	3,85E-17
S100A14	12,72701	8,81E-31
GGT6	12,70549	1,10E-15
LINGO1	12,52785	1,80E-16
IRX2	12,44365	3,06E-16
SYNE4	12,4388	2,65E-16
FXYD3	12,39029	9,13E-09
ALDH3B2	12,34419	6,58E-15
SPDEF	12,34387	1,17E-14
SPTSSB	12,32359	1,11E-14
SYK	12,21346	1,24E-15
CKMT1B	12,17336	1,43E-15
KIF26A	12,12105	9,78E-15
PCSK6	12,0779	3,39E-15
ATP2C2	12,07105	4,97E-15
TMEM150(	12,00861	6,71E-15
CBFA2T3	12,00219	3,92E-15
RBBP8NL	11,97759	7,71E-15
FAM83G	11,95046	5,25E-15
NOTCH3	11,92484	1,24E-65
FAM83B	11,91498	6,52E-15
C15orf59	11,91128	4,68E-14
HUNK	11,84707	9,47E-15
YBX2	11,84007	5,77E-15
DNAJA4	11,80285	2,29E-14
OLFM1	11,77709	1,43E-14
HENMT1	11,68735	2,50E-14
ZFP3	11,67774	2,99E-14
GYLTL1B	11,66021	2,46E-14

PCDHB8	11,57181	5,80E-14
CKMT1A	11,55224	5,48E-14
ALOX15	11,54656	2,36E-12
TMEM45B	11,53212	6,10E-14
CLDN7	11,52895	1,75E-39
RASGEF1A	11,47873	1,31E-13
HSPA1A	11,46364	7,94E-33
SMIM22	11,43684	1,69E-13
AQP3	11,34447	4,71E-07
ASCL2	11,31949	1,13E-12
C5orf38	11,30999	4,64E-13
PCP4	11,30407	2,96E-13
ASCL4	11,25651	3,83E-13
OVOL1	11,18839	1,79E-12
ARHGDIG	11,15954	5,70E-13
NUP210L	11,13421	7,35E-13
EFS	11,12882	7,99E-13
TDRD1	11,11119	7,37E-13
PRR15L	11,07817	7,45E-13
DPYSL5	10,98947	2,53E-12
KCNK15	10,97296	2,07E-12
PGR	10,92583	1,66E-10
C5AR2	10,90125	1,48E-11
MB	10,89862	2,48E-12
NRXN2	10,87858	3,00E-12
SLC9A2	10,86741	1,29E-33
PCDHB13	10,82165	4,47E-12
NAALADL2	10,81204	6,86E-12
LRP2	10,80176	2,86E-06
FUT1	10,76197	1,06E-10
RET	10,7316	8,06E-11
LMX1B	10,72053	7,14E-12
CDH1	10,68521	1,88E-137
SPESP1	10,67511	1,70E-11
KCNH2	10,66951	9,47E-12
CCDC64B	10,65317	1,95E-11
TMEM125	10,61178	1,32E-11
ASS1	10,5756	2,05E-95
WNT11	10,56267	3,46E-10
ODAM	10,52964	2,11E-11
CEACAM21	10,52011	2,48E-11
TDRD5	10,49831	4,33E-11
TSPEAR	10,48663	7,59E-11
CHGA	10,45211	3,26E-11
DPYSL4	10,42426	1,35E-10
SLC9B2	10,42333	4,70E-11
PCDHA10	10,40117	6,03E-11
SLC1A2	10,39742	1,30E-11
ADGRB1	10,36658	1,35E-11
FAR2	10,35474	1,42E-11

SCN4A	10,33945	2,48E-09
CASC9	10,32816	6,69E-11
AARD	10,27272	9,36E-11
CHRM1	10,24918	5,37E-10
HIST3H2A	10,24293	1,09E-10
HR	10,2218	6,02E-21
RNF223	10,2205	1,67E-10
CNTNAP2	10,21922	1,91E-10
PRR15	10,15782	2,93E-10
KRTAP3-1	10,1558	1,10E-09
C18orf63	10,13036	3,17E-10
RERG	10,12034	2,22E-10
XYLT1	10,04083	3,59E-10
ZNF732	9,980511	1,14E-09
TC2N	9,959165	1,73E-26
BATF	9,958711	1,48E-09
FAR2P1	9,876881	9,42E-09
EPHB3	9,871924	2,78E-10
KCNC3	9,864927	7,73E-19
SLC9A4	9,85833	9,09E-09
SLC26A4-A	9,85432	1,01E-09
CXCL12	9,851755	1,17E-09
DEGS2	9,851737	1,33E-09
MISP	9,845735	5,90E-08
KIF1A	9,838404	2,98E-19
PRIMA1	9,826784	7,10E-09
NMU	9,817473	1,74E-09
CHST8	9,762624	1,64E-09
POF1B	9,757512	6,67E-17
ZNF311	9,746658	1,19E-08
MUCL1	9,737752	1,13E-06
MATK	9,728849	2,45E-43
S100P	9,722392	5,54E-07
BCL11B	9,689256	2,92E-35
JPH3	9,68321	2,58E-09
PTK6	9,673988	3,26E-09
TMPRSS13	9,673394	7,35E-10
ILDR1	9,663267	4,97E-10
TUBA3E	9,630514	7,32E-09
KLHDC7B	9,623463	3,53E-09
ZG16B	9,602035	5,48E-08
PCDHB6	9,600011	5,90E-09
UQCRHL	9,591384	5,05E-09
C10orf82	9,5863	8,23E-09
FSD1	9,583216	5,17E-09
MUC5B	9,576451	4,83E-09
ACTG2	9,555175	1,58E-07
PPM1E	9,539995	3,60E-20
CHRM3	9,523483	6,36E-09
MYT1	9,509067	6,95E-09

MANSC4	9,498872	1,54E-06
RIMS4	9,498207	4,41E-09
TNFAIP8L3	9,497655	1,19E-15
ADAMTS19	9,496364	8,97E-26
GPR158	9,491156	8,23E-09
IRX4	9,489016	1,82E-08
DLX5	9,488963	1,04E-17
FAM19A5	9,467611	1,60E-08
C9orf152	9,459761	1,39E-16
TMC4	9,457185	2,95E-24
PCDHGB5	9,430857	1,04E-08
PPP2R2C	9,416208	1,57E-55
FUT9	9,411018	1,16E-08
CRLF1	9,402744	1,73E-08
SUSD2	9,402116	4,10E-08
DDX43	9,397957	1,85E-08
CD8A	9,394124	1,27E-08
GPR27	9,391538	1,75E-08
FIRRE	9,387858	5,95E-08
TUBA3D	9,387318	3,15E-08
ESR1	9,374839	7,74E-53
SULT2B1	9,356721	9,96E-17
ADGRE2	9,347491	2,09E-08
KCNK5	9,342983	1,05E-07
PLA2G4F	9,330872	1,64E-06
PRSS8	9,299197	1,40E-74
GPX2	9,290648	9,67E-06
LRP3	9,282291	1,44E-07
ZNF354C	9,256252	3,30E-08
FCGR1A	9,23195	2,85E-07
C17orf104	9,229277	2,27E-07
CELF5	9,206995	9,25E-08
AKR1C2	9,206805	5,28E-08
CPT1C	9,202431	1,51E-07
EYA2	9,195503	2,07E-07
HBA1	9,193176	1,05E-07
RGS6	9,19055	7,91E-08
TLE2	9,184745	3,64E-24
FGB	9,176661	2,53E-05
ONECUT1	9,174621	1,16E-07
LGR6	9,174178	7,56E-08
CA8	9,1738	7,85E-08
INHA	9,148659	1,20E-05
CNGB3	9,143975	7,62E-08
LOC339862	9,141717	6,14E-08
RIC3	9,122826	1,16E-07
PALM3	9,098637	6,63E-08
LINC00992	9,07085	9,07E-08
BLNK	9,070655	4,29E-06
ADIRF	9,068311	8,38E-18

DNAH3	9,067209	1,27E-07
CXXC4	9,065586	1,12E-07
NRG3	9,055512	1,60E-07
C1orf210	9,008528	2,26E-07
LINC00858	8,990878	3,65E-07
ADRA2C	8,978619	5,92E-49
CRABP1	8,969711	5,75E-07
TACSTD2	8,960975	4,11E-82
SPSB4	8,958987	3,96E-07
GRIK3	8,930797	1,14E-06
NPNT	8,922948	7,91E-21
MAOB	8,922788	9,01E-07
DEF6	8,917976	1,77E-08
RPRM	8,912911	6,87E-07
S100A9	8,898911	8,14E-05
HTR2C	8,898862	2,01E-07
KIAA1257	8,882001	4,19E-07
RADIL	8,866194	1,24E-06
PCDHA5	8,860842	2,49E-07
CALCR	8,851969	4,08E-07
KCNMB2-A	8,848239	2,52E-07
LOC10028[illegible]	8,84821	3,15E-07
SNCAIP	8,842129	2,61E-07
KCNV1	8,837568	1,18E-06
PLA2G3	8,835851	2,85E-06
ESRP2	8,805211	3,88E-67
SERPINB5	8,803137	1,18E-05
TMPRSS4	8,798217	6,20E-07
MPPED2	8,788784	1,84E-06
TPTE	8,779205	4,46E-07
LOC64491[illegible]	8,766351	6,83E-07
FLT4	8,765186	1,95E-06
ERG	8,763363	4,03E-07
ADAMTS1[illegible]	8,756884	4,35E-07
DSCAM	8,754499	1,38E-06
TENM1	8,733281	9,49E-07
STMND1	8,73324	5,18E-07
PACSIN1	8,728872	2,52E-13
ENTPD2	8,725112	7,55E-14
CCL5	8,706746	1,31E-05
FRMPD1	8,695789	7,11E-07
LINC00052	8,693484	4,71E-07
CCDC83	8,689507	1,08E-06
MARVELD3	8,657178	2,44E-27
FAAH2	8,651887	1,07E-06
LOC10013[illegible]	8,621515	1,00E-06
OVOL2	8,607221	7,33E-07
LOC72873[illegible]	8,599485	1,12E-06
TBC1D30	8,543722	1,12E-62
BCAS1	8,543395	0,00018

MSI1	8,538763	1,26E-51
IGF1	8,535202	1,48E-06
PCDH19	8,528231	3,61E-06
FAM83E	8,510019	4,59E-06
ASCL1	8,500992	2,44E-07
PRRT4	8,493896	2,08E-06
BRSK2	8,493859	2,74E-06
RFTN2	8,487245	3,61E-06
NOTUM	8,480507	9,46E-13
TMEM132E	8,471908	0,000201
LOC10013C	8,464261	2,41E-06
CST1	8,463119	1,42E-13
NPR3	8,462136	7,41E-14
ZNF704	8,459974	1,30E-39
CEBPA-AS1	8,435804	5,38E-06
HLA-DRB5	8,413875	4,76E-06
RGMA	8,411193	3,89E-13
SLC22A11	8,403938	1,67E-05
PVALB	8,40001	1,06E-05
ASCL5	8,399247	2,70E-06
NMUR2	8,392816	2,89E-06
TCHHL1	8,387889	0,000317
HRK	8,385101	0,00011
UNC5C	8,380102	1,40E-05
GUCY1B2	8,377821	1,40E-05
EPPK1	8,377734	1,43E-34
TOX2	8,374672	2,22E-07
TNNT1	8,372155	5,00E-148
ARHGAP4	8,367415	3,25E-07
EPN3	8,36132	4,33E-73
SOX18	8,355776	3,39E-06
LLPH-AS1	8,345854	3,54E-06
GATA5	8,339897	4,26E-06
ITGA2B	8,338746	8,44E-07
SNAR-E	8,338447	1,14E-05
IGFL1	8,331303	0,000299
SERPINA3	8,321177	1,22E-05
HPN	8,319268	9,61E-13
NODAL	8,317743	8,92E-06
WIF1	8,317348	4,47E-05
LOC102723	8,306885	5,02E-06
SLC6A14	8,296569	5,14E-06
CLCA2	8,29237	0,000134
INHBB	8,257057	5,20E-18
HOXD12	8,247269	3,80E-05
CILP2	8,234898	1,14E-05
RORC	8,231287	8,99E-06
MYO15B	8,226968	7,35E-06
HS6ST3	8,224865	8,13E-06
SLC27A3	8,223498	2,57E-56

FES	8,219784	6,80E-06
FAM46C	8,219013	7,57E-14
F7	8,207175	4,37E-05
TARP	8,199625	5,13E-05
LINC01124	8,194949	1,40E-05
GLDN	8,193448	9,85E-06
DHRS2	8,189261	5,10E-30
SLC52A3	8,187579	1,56E-05
BST2	8,185768	3,73E-21
IGFBP2	8,182129	1,80E-84
EPHA10	8,168242	5,61E-07
PART1	8,144717	1,32E-05
LOC100996	8,136356	1,51E-05
ESPN	8,132849	7,95E-18
TRIL	8,132481	2,04E-05
LRG1	8,125873	9,04E-05
PTP4A3	8,123533	1,30E-27
TEX19	8,118844	8,20E-07
CBLC	8,118273	8,55E-07
TRPV6	8,117058	1,23E-05
FOXI3	8,0776	1,61E-05
AR	8,066918	4,85E-23
POU6F2	8,062405	1,78E-05
KCNN1	8,061609	3,55E-05
COLCA1	8,052622	1,98E-05
KRTAP5-AS	8,047778	8,72E-15
C2CD4C	8,040382	3,51E-05
CYP1A1	8,040202	1,13E-09
GHR	8,033816	1,78E-05
VAV3	8,022216	4,13E-46
PRDM6	8,017171	1,29E-06
IGSF11	8,015611	1,87E-05
TTYH1	8,002457	0,000123
CEBPA	7,993017	7,39E-23
CLDN9	7,976742	4,53E-05
MGC32805	7,970889	1,00E-04
POU3F3	7,967444	2,82E-06
ACOXL	7,967365	7,20E-05
ZNF467	7,963579	3,45E-60
KRT16	7,958857	0,000137
MAFB	7,952646	6,64E-19
SNCB	7,92035	0,000319
LAMC3	7,912812	3,23E-05
XG	7,909792	0,000646
TMEM105	7,903823	6,41E-05
ICOS	7,894799	3,87E-05
ARL11	7,893829	3,82E-05
LOC440461	7,893034	9,51E-05
ALDH3A1	7,888136	3,27E-05
BARX2	7,888115	0,00022

EMCN	7,880152	4,30E-05
LOC10050€	7,879759	3,85E-06
SP5	7,878204	8,42E-102
LINC00885	7,872246	0,000284
EWSAT1	7,869114	8,29E-05
IGSF1	7,856491	0,000295
LRRC10B	7,852378	0,000102
BEX5	7,851691	0,000179
JAKMIP3	7,849668	4,75E-05
LRRC26	7,844723	2,13E-11
FAM3B	7,842274	3,59E-16
BAMBI	7,841072	9,17E-146
KRTAP4-1	7,827021	9,17E-05
CLDN3	7,824307	1,31E-126
WISP2	7,812155	7,60E-05
AP3B2	7,811108	8,05E-06
SYT5	7,800115	5,82E-05
CYP4F22	7,790406	8,03E-05
CALML5	7,779694	0,000121
HCN4	7,777574	3,85E-11
GJB5	7,777065	0,000447
ALOX12P2	7,766611	0,000296
TLX1NB	7,7599	6,44E-05
PROM2	7,749756	3,78E-41
GJD3	7,744883	0,000125
KLB	7,720561	8,86E-05
C8orf48	7,720494	9,24E-05
DRD1	7,710831	0,0008
CAPN9	7,699946	0,000114
B3GALT4	7,695406	2,08E-05
MUC20	7,693011	0,000694
DEFB126	7,691606	8,95E-05
SNAR-G1	7,672652	0,000138
S1PR5	7,669532	0,000111
OXGR1	7,663141	0,000153
CELF3	7,640592	0,000194
TTN-AS1	7,636287	0,000122
ANXA9	7,632594	4,24E-23
LINC00925	7,611925	2,03E-10
WNT9A	7,604457	0,000231
IBSP	7,601147	0,000139
LOC11323(	7,589262	3,64E-22
GNG13	7,577917	1,67E-05
KCNJ4	7,576427	0,000167
CEACAM5	7,574311	0,000436
GADD45G	7,571515	2,73E-12
LINC00494	7,567395	0,000155
PRDM16	7,567335	0,000337
C19orf45	7,549072	0,000168
CEACAM6	7,541216	0,00078

CA4	7,537524	0,000241
HIST1H3E	7,514146	0,000467
FAM83F	7,496364	0,000583
ZSCAN12P1	7,49523	6,06E-10
ETNPPL	7,482815	0,00092
BRINP2	7,473422	0,00052
COL3A1	7,469725	0,000417
GPC3	7,447513	0,000725
PDE9A	7,442886	1,55E-05
KCNK2	7,430157	0,000629
PRLR	7,40943	2,86E-45
ELF5	7,401332	4,88E-08
PVRL4	7,400557	3,79E-30
SLC17A7	7,36364	0,000604
LRFN5	7,349145	0,000403
ARHGEF4	7,345756	2,02E-05
SSPO	7,331869	0,000608
EDAR	7,329582	5,79E-05
HID1	7,309101	3,20E-65
HLA-DRB1	7,302209	2,57E-08
PDIA2	7,300713	0,000488
MIR2052H(	7,299358	0,000894
RDH16	7,295616	5,25E-19
RIMKLA	7,290784	3,24E-13
HSD17B14	7,279286	2,57E-05
IZUMO1	7,270308	0,000629
PAQR5	7,268815	2,63E-13
MCF2L-AS1	7,267507	0,000697
LINC00659	7,267507	0,000697
MB21D1	7,265861	3,00E-05
FGF13	7,264692	0,000788
NKD2	7,257563	2,98E-13
HAGLROS	7,256226	0,000584
PTPRQ	7,253415	0,000599
SIX2	7,249134	0,000892
PCDHA13	7,248368	2,99E-05
SLC22A23	7,245945	6,23E-91
PCBP3	7,237219	1,38E-12
ABCC8	7,233457	0,00064
DLL4	7,221909	0,000858
MYCN	7,214668	0,000724
WNK2	7,207631	1,17E-41
LPAR5	7,207377	0,000182
LRRC16B	7,205968	0,000725
HLA-DQB1	7,205871	2,75E-08
SUSD3	7,199285	2,30E-14
MAPK4	7,180564	6,20E-07
NOX5	7,1704	0,00096
CARD14	7,159692	6,46E-05
EPHA1	7,157308	5,45E-26

PAX5	7,156047	0,000203
SYT17	7,142549	1,95E-21
FAM155B	7,129903	2,25E-18
MYRFL	7,128783	0,000421
SPINK5	7,124435	1,74E-07
PRSS27	7,118386	5,33E-05
TFF3	7,113244	7,01E-12
TYRP1	7,099464	1,15E-08
FGF12	7,059273	1,93E-17
PCDHB14	7,056352	1,09E-27
WNT6	7,027619	6,77E-05
MLXIPL	7,009584	4,80E-11
KCNJ3	6,98948	3,89E-08
TEX40	6,977465	8,22E-05
EOMES	6,973531	1,98E-08
SLC24A3	6,962464	1,55E-11
ALG1L	6,952308	2,35E-20
UCP2	6,935456	1,28E-126
SYTL1	6,913169	3,43E-28
C2orf54	6,896589	2,60E-06
ALDOC	6,877706	1,35E-26
RIPPLY3	6,866875	0,000134
PRPH	6,846488	1,43E-07
CSTA	6,814488	3,88E-10
TRPM2-AS	6,812822	0,000154
RGS16	6,79261	4,93E-15
PDE3B	6,773377	6,22E-17
TINCR	6,762009	3,11E-15
TP53I11	6,746541	9,52E-66
ELL3	6,743801	1,08E-20
FIBCD1	6,715264	2,49E-14
PLXNA4	6,689089	1,77E-17
SFMBT2	6,686852	3,33E-07
ABCG1	6,649338	1,07E-35
KDF1	6,632163	4,65E-30
PCDHA11	6,598794	8,80E-36
GFRA3	6,597	0,000979
GAL	6,596618	2,39E-71
HES2	6,561154	4,14E-10
BNIPL	6,557687	1,06E-05
APOA1	6,5559	0,000448
ZNF418	6,550254	0,000447
GALNT16	6,52511	5,77E-14
GATA3-AS1	6,513061	0,000517
IGSF9	6,500515	1,12E-121
NFE2	6,49918	1,58E-13
EPS8L1	6,490023	9,30E-19
TH	6,488408	0,000768
LYPD3	6,484153	1,13E-22
RNF150	6,468015	7,22E-07

HTR6	6,463518	0,00055
PCDHB9	6,45763	6,80E-13
SEPT3	6,447867	5,55E-25
POTEF	6,423365	0,00069
ABLIM2	6,417344	8,87E-07
PCDHA6	6,412895	2,59E-64
ZNF385C	6,406103	8,42E-07
FAM178B	6,379906	2,87E-05
FBXL16	6,378843	5,73E-24
PCDHB3	6,364356	1,70E-33
ADAM11	6,358188	5,76E-17
ENTPD1	6,346953	6,31E-15
PAX9	6,336048	1,36E-08
DNAAF3	6,330092	4,66E-33
COLEC12	6,304834	2,02E-27
EGR3	6,286542	1,13E-05
KRTCAP3	6,274912	3,07E-32
CYP1B1	6,257272	7,70E-30
C19orf57	6,246395	1,51E-05
MAPT	6,245593	8,20E-38
GRHL3	6,243581	4,65E-18
KCNN2	6,237601	4,81E-06
MYRIP	6,237233	3,49E-15
LLGL2	6,227653	1,79E-47
PCDHA12	6,224555	9,90E-12
SIDT1	6,207313	3,76E-12
BEND5	6,201918	2,32E-09
ZSWIM5	6,195281	4,99E-10
DIRAS1	6,192872	1,57E-30
HAGLR	6,188252	3,46E-06
TCHH	6,175848	1,23E-05
TFAP2C	6,160046	8,71E-104
GALM	6,150547	6,45E-14
TMOD2	6,139437	8,34E-20
KAZALD1	6,136316	4,12E-08
FAM81B	6,122833	1,72E-05
CFD	6,122319	3,20E-12
KYNU	6,113509	1,28E-29
ADGRV1	6,111649	4,15E-09
DLGAP3	6,103759	4,73E-14
CCDC170	6,088898	1,46E-20
FHDC1	6,081805	8,21E-20
DYNC1I1	6,070003	2,54E-56
PCDHB4	6,068783	1,21E-06
OR7E14P	6,031421	1,35E-11
MNX1-AS1	6,027925	7,43E-20
ERBB3	6,020312	9,74E-117
REEP1	6,019927	7,93E-08
LIN7A	6,01283	3,53E-30
BEST1	5,982743	0,000218

CRMP1	5,982242	7,09E-24
LOC14870¦	5,974029	1,42E-08
MUC1	5,949507	8,02E-26
NKAIN1	5,942051	1,07E-27
AP4B1-AS1	5,928064	1,51E-21
RUNDC3A	5,923599	6,62E-05
PRRG2	5,911371	9,08E-10
HOXC13-A¦	5,889124	3,77E-07
NCAM2	5,887442	5,52E-26
FLRT3	5,885295	1,10E-69
TMEM191/	5,863297	4,05E-31
LTBP1	5,854787	1,14E-90
ACTL8	5,852724	3,33E-08
PCDHB10	5,84419	1,25E-06
RNF43	5,830116	1,58E-111
LINC00649	5,798	7,21E-05
OLFM2	5,79635	7,17E-38
CXADR	5,78231	1,12E-43
CDC42EP5	5,774756	2,29E-06
DMC1	5,774713	2,01E-05
KCNJ11	5,769346	1,89E-14
PAH	5,757734	0,000127
PCDHGA4	5,750418	0,000125
FGD3	5,73821	6,48E-17
DOC2A	5,717034	1,51E-13
GRTP1	5,71399	1,40E-24
CCM2L	5,706974	1,86E-09
PRR36	5,692946	5,39E-117
N4BP3	5,623465	2,53E-34
RGAG4	5,612255	3,59E-07
CIART	5,591331	3,61E-21
GUCY1A3	5,562459	2,94E-18
NEURL1B	5,557183	1,92E-46
DLX3	5,554126	1,63E-24
GATA3	5,544602	1,12E-123
NPL	5,54189	1,50E-12
EFNA2	5,527698	1,50E-05
SPINT1	5,527494	1,01E-91
KHK	5,503337	6,76E-47
ADSSL1	5,503238	7,59E-08
LINC01132	5,500872	7,46E-06
ZNF114	5,495385	2,76E-19
PCDHGA1	5,48308	0,000379
HPDL	5,481971	9,67E-16
CEL	5,469543	1,83E-07
AGR2	5,464643	0,000349
PCDHGB7	5,457154	0,000473
ELMO3	5,4566	4,41E-62
LINC01547	5,456245	2,77E-10
SLC22A31	5,446397	2,62E-08

GOLT1A	5,445639	4,95E-15
KREMEN2	5,437581	7,45E-66
RCAN3	5,420547	1,88E-10
GREB1	5,407617	1,79E-74
FAM83H-A	5,39582	2,14E-54
ARHGAP44	5,391058	6,42E-22
GOLGA7B	5,38782	6,14E-13
GPM6B	5,381555	0,00028
TIMP3	5,378529	2,71E-12
XKRX	5,375533	0,000145
ACE	5,356598	9,76E-05
LRRC73	5,345477	1,10E-10
SLC30A3	5,344767	7,72E-07
ADCY1	5,32144	3,47E-37
ZFYVE28	5,308415	7,36E-24
PLCB1	5,305955	2,52E-26
RAP1GAP	5,304541	1,03E-15
IFITM1	5,303452	5,00E-10
EPHA6	5,271792	4,21E-13
NEB	5,26763	2,09E-42
LHX4	5,266111	2,10E-34
PDZD4	5,265849	1,26E-12
DLX4	5,262897	1,57E-05
MYO5C	5,255173	1,19E-81
GJB2	5,250607	6,57E-19
ARC	5,238356	2,33E-06
MUC3A	5,228116	3,53E-06
PDK3	5,216941	3,13E-34
TMEM229E	5,206124	2,10E-14
PALM	5,17319	2,21E-29
CCDC64	5,170568	1,56E-22
ERBB4	5,14034	6,40E-13
EFR3B	5,126864	2,48E-27
WDR72	5,093029	1,31E-59
F12	5,074979	1,43E-92
NMNAT2	5,069453	1,45E-12
RAB3D	5,064505	1,05E-59
NUPR1	5,045574	1,73E-15
VGF	5,039764	1,91E-28
CLIC3	5,033512	1,07E-05
HNRNPLL	5,032476	2,13E-34
BCAS3	5,02216	3,20E-76
MYCL	5,019182	2,01E-10
PTCHD2	5,010804	5,16E-06
HIST1H3G	5,009903	0,000112
RPL39L	5,009228	3,50E-45
SOBP	5,007625	3,93E-09
TEX14	5,005147	8,83E-05
WNT10B	5,003472	1,58E-09
NEBL	4,996028	1,56E-47

FREM2	4,992439	1,07E-143
LMTK3	4,981188	2,64E-16
MARK1	4,960962	0,000244
HLF	4,960235	0,000162
UPK2	4,935891	4,59E-07
PTPRO	4,934877	2,09E-21
STON2	4,929003	1,24E-59
PKP1	4,919883	1,46E-11
PCDHGA5	4,909438	0,000222
TBX2-AS1	4,898252	2,07E-11
JUP	4,895337	1,62E-41
RHOV	4,888801	2,29E-22
PCDHB16	4,878507	2,56E-27
KLRG2	4,85735	3,16E-08
PKIB	4,855494	7,99E-50
CBX2	4,853516	5,40E-103
RORA	4,85114	1,14E-26
SRRM3	4,8473	4,80E-07
NRARP	4,832727	1,70E-21
TMPRSS2	4,830942	3,93E-09
RAB26	4,829262	3,57E-09
RPP25	4,815145	6,44E-60
MPP7	4,813533	1,01E-47
AKR1E2	4,809411	3,30E-06
C15orf65	4,800786	2,04E-08
SBK1	4,793009	4,38E-91
ARTN	4,789856	3,44E-06
DKK1	4,777282	7,64E-14
TMEM254-	4,756818	1,15E-07
LYSMD2	4,753732	5,95E-45
SPATA17	4,716789	1,61E-23
TSTD1	4,713959	3,21E-40
MALRD1	4,712122	1,85E-08
UNC5B	4,708758	1,76E-08
SLC27A2	4,696577	1,37E-43
PCDHGA10	4,693503	0,000136
LDHD	4,688177	1,24E-06
ZBTB7C	4,683896	1,81E-05
STXBP6	4,673735	1,00E-12
FAAH	4,670414	1,20E-25
ST14	4,655744	7,33E-47
SELM	4,65334	1,82E-06
ADM2	4,649062	5,27E-13
DOCK8	4,640511	2,91E-08
CLUHP3	4,63431	0,000381
LYPD6B	4,622548	2,08E-06
CGREF1	4,61558	3,78E-28
AEBP1	4,609508	1,38E-06
ZNF204P	4,592671	5,89E-06
RNF157	4,583556	9,30E-11

ISYNA1	4,582215	7,96E-86
KIF5C	4,580017	6,71E-06
CALHM2	4,562518	3,43E-14
MAP10	4,55105	5,20E-05
CYB561	4,543841	3,38E-138
PPFIA3	4,534016	5,17E-35
SH3YL1	4,525652	1,48E-48
LRRC41	4,524694	9,98E-27
AIF1L	4,521972	5,61E-85
STK31	4,520906	0,000515
GRHL1	4,507454	3,66E-16
EHF	4,503518	3,55E-14
LRRC75B	4,474299	1,88E-15
FRAT1	4,459431	1,10E-05
MFAP2	4,458415	1,23E-22
C2orf15	4,454452	7,33E-30
JHDM1D-A	4,452623	1,23E-12
EMID1	4,452582	2,02E-08
NRCAM	4,446369	4,99E-23
NELL2	4,43658	0,000326
HIST1H3H	4,415333	1,16E-05
PCSK1N	4,414672	1,63E-33
KRT17	4,413753	5,68E-08
C1orf21	4,412862	2,34E-13
SEMA4A	4,412042	7,74E-28
IFITM10	4,407075	0,000151
MGAT4A	4,403843	2,08E-19
ERICH2	4,394114	1,61E-20
RHOB	4,393092	2,28E-70
RASD1	4,392781	3,27E-06
KCNJ8	4,384684	2,81E-50
TRPV3	4,378235	1,12E-07
LCP1	4,374179	3,15E-15
IFI30	4,371441	8,71E-42
ZNF165	4,367751	3,55E-36
CEMIP	4,363461	3,99E-16
HOXC13	4,360755	3,84E-20
ATP1A3	4,358551	2,02E-15
GPLD1	4,358538	1,33E-06
ADGRL1	4,350616	2,11E-104
ZNF879	4,350355	9,18E-07
EPCAM	4,348497	2,38E-93
EFHD1	4,341371	5,25E-19
SEMA6A	4,341098	2,76E-15
PLEKHA7	4,338117	9,79E-46
HIST2H2AC	4,337836	0,000259
FBXO15	4,335376	1,29E-06
ABCC2	4,329267	2,27E-06
KIAA1211L	4,324069	0,000442
EFNA3	4,313914	1,07E-26

HIST2H2BE	4,311449	5,82E-15
ADAMTS17	4,307017	3,92E-05
SMPDL3B	4,306185	1,91E-45
REEP6	4,302961	3,07E-40
ZBTB42	4,293847	1,11E-43
C1orf233	4,288149	6,27E-47
ALDH5A1	4,285657	4,19E-46
SYT1	4,28516	4,64E-19
TUB	4,277657	2,10E-06
STARD10	4,273569	1,46E-32
CDH18	4,267716	0,000259
BGN	4,264109	2,35E-08
S100A4	4,255078	0,000193
CACNG4	4,244274	4,27E-26
TP73	4,233575	1,13E-06
C21orf58	4,232305	9,91E-17
MARCH1	4,22542	9,77E-06
ARHGEF16	4,221823	2,28E-31
YPEL1	4,20095	4,55E-05
MYH7B	4,200136	6,69E-06
ASAH2	4,191448	1,60E-19
RAB3IL1	4,154187	1,87E-15
PODXL2	4,148177	1,79E-59
CNKSR1	4,141298	1,56E-14
C17orf96	4,136378	1,84E-30
ACKR3	4,133332	2,76E-53
NCOA3	4,132875	1,15E-78
ADPRHL1	4,130645	4,59E-21
HCN2	4,110127	2,73E-18
PCDHGB2	4,109858	0,000441
HIST1H2BD	4,107942	3,10E-13
SYT3	4,102478	6,97E-08
SERPINI1	4,09737	1,66E-06
FGFR4	4,095316	8,00E-36
SOX2	4,089004	2,43E-18
USP32	4,077312	1,57E-43
RASL11B	4,049234	2,45E-17
CPLX1	4,049026	6,17E-16
SAPCD2	4,047526	6,02E-43
RUNDC3A-/	4,045775	1,16E-07
TMEM238	4,037224	7,41E-15
GRID1	4,034844	0,000437
RND2	4,028203	6,86E-15
MAGEH1	4,023033	1,46E-10
HIST1H2AC	4,012819	1,39E-08
MIR4697H(	4,012304	0,000448
LRRC37A8F	4,008044	6,18E-07
C12orf56	3,992825	8,64E-12
NANOS1	3,990586	4,39E-29
SLC27A5	3,983608	2,97E-32

NHLRC1	3,967162	3,65E-05
CRABP2	3,966487	1,65E-19
LOC100505	3,962591	3,03E-07
IMPA2	3,95525	3,32E-41
AK7	3,954617	8,15E-42
PCDHA7	3,949523	4,99E-07
RAB39B	3,94817	0,000487
SLC15A2	3,943918	9,93E-06
HIST1H2BK	3,939591	2,36E-13
RMND5B	3,93272	6,95E-60
ZNF790-AS	3,931081	1,05E-11
MAP2K6	3,92919	1,97E-07
GABPB1-AS	3,926283	5,05E-16
LINC01006	3,892855	2,35E-05
SPPL2A	3,883393	6,51E-57
COL9A3	3,883268	1,66E-06
WFDC2	3,852664	0,000862
DGAT2	3,84665	1,62E-15
MAML3	3,843805	7,60E-10
SLC16A14	3,842402	1,06E-07
S1PR3	3,842087	8,16E-13
IRX3	3,836737	2,28E-35
IGSF10	3,831219	0,000138
FOXA1	3,830582	1,58E-96
SELENBP1	3,821968	1,68E-39
CLDN4	3,821422	1,59E-31
FRMD6-AS	3,820461	3,19E-06
MAPK8IP2	3,811875	1,19E-10
FMO5	3,810205	3,81E-05
RNF125	3,795984	5,75E-06
SLC43A2	3,789647	1,50E-31
CREB3L4	3,787559	1,48E-42
MAST1	3,780597	1,72E-11
SRCIN1	3,750903	1,48E-06
PCDH1	3,732306	6,28E-27
HIST1H1C	3,73096	5,63E-07
FAM78A	3,730262	0,000104
TTC39A	3,729005	2,50E-11
RPS6KL1	3,719453	1,06E-16
GLUD2	3,707191	2,42E-13
TUBD1	3,7046	5,19E-33
CEACAM1	3,704261	3,20E-12
DTWD1	3,703059	7,70E-29
APPBP2	3,700496	2,13E-95
PKP3	3,698591	3,13E-38
COL9A2	3,692701	6,92E-12
CCDC74B	3,672276	6,47E-05
PSMG3-AS	3,669083	6,15E-16
CCDC78	3,668795	1,00E-06
FAM227B	3,666267	4,16E-14

HOOK2	3,665886	1,91E-64
IRX5	3,662356	2,67E-33
RHOBTB1	3,662181	1,72E-83
PGBD5	3,653893	1,05E-06
RINL	3,64486	5,72E-05
ADAMTS13	3,618169	9,36E-05
FLJ10038	3,614934	2,75E-21
LENG9	3,614481	3,34E-26
HS3ST3B1	3,598227	8,12E-06
NGEF	3,597016	0,000738
FAM222A	3,59608	7,41E-31
FA2H	3,594132	4,05E-06
RNF165	3,591736	1,01E-07
CXCR4	3,579725	3,26E-11
CPT1A	3,577461	1,50E-75
SCML2	3,574119	1,38E-12
ABCA12	3,570582	4,20E-18
C19orf81	3,564877	1,22E-05
TFAP2A	3,558483	6,22E-94
EDA	3,537204	6,80E-05
RNF144A	3,534372	8,02E-15
AGAP2	3,526119	1,77E-15
SIAH2	3,520993	6,50E-42
C19orf33	3,520213	3,30E-15
LINC00960	3,515671	1,12E-07
TBX2	3,511265	5,38E-25
LOC283335	3,506812	2,74E-17
FAM174B	3,506803	1,12E-12
RASGRF1	3,506656	2,14E-21
CELSR3	3,505649	1,88E-22
MYB	3,503059	8,15E-09
GNG7	3,500444	1,57E-05
TTC9	3,500257	2,20E-27
GDPD3	3,499187	1,71E-05
TCF7L1	3,496759	3,36E-10
SAMD11	3,495837	3,96E-07
EML6	3,486963	2,51E-05
PPFIBP2	3,474035	3,55E-16
LOC100128	3,46098	0,00035
ZBTB46	3,449533	0,000587
RRAGD	3,448773	7,56E-08
VAV1	3,439466	6,73E-09
C9orf172	3,438607	9,72E-06
SLC2A4	3,43395	1,50E-05
MYL5	3,433811	3,48E-07
ADGRL2	3,433547	9,69E-10
P2RY6	3,431714	0,000382
PRSS22	3,431487	1,91E-06
PCDHGB1	3,426397	1,27E-09
TRPS1	3,420781	1,74E-73

TRAPPC6A	3,41464	8,42E-27
TGM1	3,41395	0,000202
ANKRD34A	3,401497	6,84E-11
SLC6A9	3,390732	6,43E-08
FOXP2	3,382397	3,14E-05
ZSCAN31	3,380589	1,26E-15
C7orf13	3,379778	2,86E-15
STOX1	3,375958	1,15E-05
PPM1D	3,374625	8,01E-61
FGFR3	3,368207	1,18E-19
FAM46B	3,364071	1,30E-13
MREG	3,357187	4,77E-36
SOX13	3,341751	1,72E-17
MFAP3L	3,337929	7,03E-11
KCNMB4	3,329226	2,85E-05
IRF6	3,327678	6,31E-18
TRIM37	3,324236	1,87E-86
SYT12	3,322977	3,66E-13
RBPMS2	3,318744	1,79E-07
DUSP2	3,317625	3,72E-07
LINC01012	3,306756	0,00026
CDS1	3,296751	5,21E-34
GSE1	3,291457	6,94E-84
MAST4	3,291213	1,58E-07
SLC9A3R2	3,282602	1,96E-44
JAG2	3,276742	3,77E-21
EPB41L5	3,27543	2,21E-45
BRIP1	3,274908	5,41E-55
C1QTNF9B-	3,274016	3,67E-11
RNF44	3,27153	5,68E-47
LCMT2	3,267496	8,18E-22
OBSCN	3,26679	3,61E-11
RPS6KB1	3,258127	2,73E-75
ICOSLG	3,257999	2,69E-08
LEO1	3,257922	2,31E-73
TM7SF2	3,256218	1,02E-14
SYNPO2	3,249664	1,94E-10
MAPK6	3,247095	1,72E-75
ZNF497	3,242809	7,60E-09
VASH2	3,238978	9,15E-09
PLEKHB1	3,237132	1,30E-08
JMY	3,229979	1,64E-08
ZNF433	3,226089	1,43E-12
USP8	3,225557	2,00E-64
LRRC37A3	3,224642	1,00E-06
PLEKHG6	3,222883	2,68E-08
ZNF354A	3,217498	2,58E-22
GPC2	3,216181	8,61E-07
MZF1-AS1	3,211841	1,10E-07
TSPAN13	3,207037	4,11E-37

ZP3	3,202662	5,77E-25
CRTC1	3,196219	1,10E-32
CGN	3,196044	1,53E-30
MAP7	3,195099	1,85E-45
HEYL	3,185108	0,000491
ZSCAN16	3,182325	3,66E-21
SMA4	3,181908	0,000183
RALGPS1	3,175	3,07E-18
RSPH1	3,17404	0,000222
HEATR6	3,168136	1,17E-59
DGKE	3,16313	1,80E-11
LINC01004	3,157075	9,68E-08
DBP	3,151616	1,28E-21
FAM102B	3,151221	6,95E-46
SULT1A1	3,148429	2,94E-06
C6orf52	3,143871	9,78E-06
RTN1	3,128605	0,000793
AP4E1	3,126384	1,58E-30
XBP1	3,122667	2,56E-14
FAM214A	3,122186	4,53E-08
RAB11FIP4	3,11974	1,99E-38
CFAP44	3,114629	0,000233
TSPAN33	3,112764	8,98E-13
USP18	3,102776	4,38E-07
C12orf57	3,089241	1,33E-24
PDCD4	3,086742	1,30E-31
DUSP9	3,08674	2,58E-09
ACSS3	3,0833	4,17E-57
KLF5	3,077283	3,90E-47
PREX1	3,071571	5,07E-33
RASSF5	3,063677	0,000599
LOC100134	3,062947	0,000762
LEF1	3,062295	1,10E-24
PIK3C2B	3,047819	1,26E-30
ATP6V1C2	3,043943	1,73E-12
CAMK2N2	3,04044	5,16E-13
GRIN2D	3,039382	1,04E-06
PRDX2	3,038257	3,79E-51
ADAM22	3,038242	1,34E-16
TNFRSF19	3,034088	1,10E-33
DLL1	3,033533	0,00043
FOS	3,026324	0,00025
NUDT8	3,02555	7,41E-15
C14orf169	3,022917	2,11E-19
TMEM216	3,018865	8,56E-10
DHRS13	3,017365	2,38E-18
BOLA1	3,017251	5,12E-24
SEMA3F	3,012534	4,11E-25
ZCCHC12	3,009224	0,000266
SLC22A17	3,008271	3,61E-05

GSTZ1	3,006892	4,91E-37
LHX2	2,985015	0,000308
TNRC6C	2,981762	1,49E-37
PITPNC1	2,979948	4,35E-52
TPBG	2,978153	1,61E-43
C1orf115	2,975455	4,95E-12
KCNK6	2,974521	1,52E-10
MROH6	2,974014	2,67E-11
VPS9D1-AS	2,972787	4,06E-32
GJA3	2,970489	9,15E-08
DAAM1	2,968514	3,06E-33
GAD1	2,96292	9,61E-13
PCDH18	2,959993	1,15E-06
LRRC24	2,959435	4,11E-06
DYRK1B	2,957247	1,00E-25
GGT1	2,956185	1,94E-18
ARHGEF19	2,952293	1,82E-15
CASKIN1	2,951793	1,67E-11
ACBD7	2,947091	7,75E-14
ST8SIA4	2,93774	4,54E-06
TGFB3	2,923492	0,000619
PFDN4	2,922798	4,49E-64
PDE4DIP	2,921863	8,77E-19
AMH	2,901752	4,09E-05
HOXC-AS3	2,896669	0,00021
NPDC1	2,888749	6,14E-50
IGFBP5	2,888261	3,77E-14
PIK3R3	2,885542	1,75E-16
HCG11	2,879715	8,39E-13
TRPM7	2,876323	2,73E-59
A1BG-AS1	2,873333	4,22E-09
LAGE3	2,871279	1,97E-34
TOB2P1	2,870313	0,000615
CAMK2B	2,862869	1,20E-08
SERINC5	2,860044	4,78E-18
SDHAF1	2,851624	2,26E-17
GSG1L	2,849657	4,99E-05
CSAD	2,847382	1,62E-08
HECW2	2,846463	8,41E-12
KRT8	2,845808	2,26E-18
KLHDC9	2,837808	5,01E-05
RABL2A	2,837319	5,88E-06
BCAS2	2,836136	2,41E-62
P2RY2	2,831311	5,58E-20
IGSF5	2,828348	0,000143
MAP3K14-/	2,826681	1,43E-07
FUT8-AS1	2,826678	5,97E-05
A1BG	2,826123	1,53E-06
MDM4	2,822768	4,89E-17
RAD51C	2,81663	2,42E-50

CABYR	2,814396	7,97E-06
PAIP2B	2,81391	2,37E-12
CNIH2	2,813232	2,73E-12
ALDH3A2	2,806942	1,49E-52
PAX8-AS1	2,798683	2,48E-13
SMIM14	2,795273	3,14E-07
LOC90784	2,794811	5,03E-30
HSD3B7	2,789312	6,75E-30
RAB3A	2,782729	1,21E-06
ZBTB12	2,77781	7,25E-09
RNF122	2,773511	2,86E-12
PSMD6	2,770866	1,09E-43
G6PD	2,769616	3,26E-06
SUV420H2	2,767876	1,39E-16
KALRN	2,76395	2,16E-06
RTN4R	2,761517	2,42E-10
BSN	2,759337	0,000247
GLUL	2,756833	4,97E-71
C10orf35	2,75194	3,63E-08
CSPG5	2,751609	1,48E-07
LAMP3	2,750296	0,000201
CLU	2,745295	9,50E-13
ABCB6	2,744693	3,16E-18
PCAT7	2,743471	3,56E-05
KCNS3	2,739181	0,00053
SDHAP2	2,733153	5,78E-08
NXPH4	2,728804	1,58E-21
SLC48A1	2,71683	7,51E-10
SPIRE2	2,714698	1,85E-07
HSD11B2	2,70515	7,69E-08
LONRF2	2,703525	5,38E-10
SHF	2,69968	1,41E-06
ACTL10	2,690634	2,67E-05
KNDC1	2,687612	0,000109
OSR2	2,679434	4,73E-07
DENND2C	2,674613	0,000175
CACFD1	2,672958	1,28E-16
RNF208	2,672209	1,65E-14
DMXL2	2,668567	8,17E-34
EPB41L4B	2,666593	7,94E-20
DDIT4L	2,666255	2,13E-07
YBEY	2,665017	8,22E-07
ZNF703	2,660797	2,89E-34
C22orf46	2,646439	1,06E-16
ZNF397	2,638027	6,99E-15
DDAH2	2,633352	3,80E-23
MARVELD2	2,632863	1,05E-15
SLC26A11	2,631401	2,67E-09
PRSS16	2,62947	1,55E-09
DCXR	2,628687	6,81E-36

SMKR1	2,627022	1,47E-09
MYLIP	2,623517	1,90E-17
SH3BP5	2,62265	7,88E-41
RNASEL	2,614255	7,57E-13
SIGIRR	2,604935	1,93E-23
KRT18	2,597355	2,74E-12
MEX3A	2,594273	1,82E-52
KLHDC2	2,594211	5,92E-15
FAM69B	2,591689	2,71E-06
B3GNT4	2,590538	3,32E-05
TXNIP	2,589387	3,74E-05
TFR2	2,585652	2,68E-05
TSPAN15	2,5849	1,79E-13
SESN3	2,582043	5,23E-05
PANX2	2,5808	0,000466
PRICKLE2-A	2,580566	0,000521
CRYL1	2,577968	2,65E-19
ZNF385A	2,576257	9,84E-33
PRMT6	2,573421	3,48E-45
ICA1	2,573333	4,52E-18
MED12L	2,572764	3,31E-07
MSX2	2,572078	2,32E-15
EDN1	2,568117	7,43E-07
CENPBD1	2,565132	5,09E-17
GABPB1	2,562733	3,52E-21
ETS2	2,560641	1,21E-25
COBL	2,56013	1,18E-10
HEIH	2,555355	5,01E-10
PLEKHF2	2,549929	1,65E-31
EGFL7	2,543019	9,21E-17
SLC29A3	2,541892	2,50E-09
SALL4	2,538152	3,43E-06
TLCD1	2,526538	2,84E-27
ZNF793	2,522245	1,25E-11
GPR153	2,513291	2,33E-12
CXCL16	2,513202	3,68E-13
GCH1	2,510149	2,13E-16
ZMIZ1	2,509308	6,02E-23
PTGES	2,501548	1,07E-05
WDR83	2,499766	1,66E-18
SOX4	2,497254	1,23E-30
IQCH-AS1	2,496979	5,43E-12
TMOD3	2,496762	4,08E-46
LNX2	2,487886	6,04E-31
FRAS1	2,487232	6,48E-14
TEAD2	2,485486	6,62E-19
ADRBK2	2,485228	5,91E-10
SCAMP5	2,484553	2,55E-07
ISM1	2,482841	0,000125
ZBED9	2,481031	1,44E-07

TMEM183E	2,478751	1,29E-10
CCDC57	2,475797	2,66E-15
ENPP5	2,475242	8,16E-05
KIAA1211	2,474011	7,65E-10
ISOC2	2,472649	7,82E-41
NPW	2,471566	0,000428
TLCD2	2,470805	5,40E-07
KBTBD7	2,468439	1,98E-13
FAM110A	2,467388	2,46E-09
FUOM	2,46705	3,71E-09
SP6	2,457958	1,24E-10
FAM171A2	2,450381	2,40E-27
ARRDC4	2,449157	8,08E-08
NIPAL2	2,447387	1,91E-08
CASKIN2	2,445484	2,56E-27
NAT8L	2,437331	6,71E-12
KIAA1324L	2,434519	1,51E-21
ITPK1	2,434229	2,37E-35
ZNF253	2,4339	1,00E-19
SLC39A4	2,430337	3,02E-15
LIN7B	2,42938	1,62E-05
TMEM52	2,427843	0,000281
SLC4A8	2,423074	0,0003
ATXN7L2	2,421176	3,47E-10
RLTPR	2,419231	0,000463
PPCDC	2,416785	6,26E-11
TET3	2,415168	7,96E-40
TRIM36	2,415055	1,16E-14
FRAT2	2,414768	1,41E-08
TRPM4	2,41275	6,34E-23
LSR	2,40489	2,85E-26
ZNF239	2,400309	2,33E-05
PNRC1	2,396631	8,23E-16
PLEKHH1	2,395303	1,25E-20
DNASE2	2,39137	1,02E-30
NHP2	2,389932	6,32E-49
BAHCC1	2,38761	2,43E-05
MTIF3	2,386428	1,31E-17
SMAD6	2,384647	8,49E-07
KRBA2	2,38447	0,000896
B4GALNT4	2,383864	1,31E-09
TMC6	2,383427	3,18E-15
MDH2	2,382753	1,56E-32
TMEM44-A	2,381935	0,000149
MEST	2,381458	1,69E-41
ZNF711	2,380725	1,62E-10
BLVRB	2,375308	1,89E-10
LPAR2	2,371817	2,32E-27
RAC3	2,362147	3,89E-15
ZNF737	2,352056	2,17E-07

C5	2,348149	6,04E-06
CCDC71L	2,346679	7,81E-08
SH2D3A	2,341491	1,83E-06
MPND	2,341477	7,27E-16
CSDE1	2,341375	7,42E-27
FAM53B	2,34105	1,26E-36
ALKBH2	2,340665	3,30E-18
ZSWIM7	2,340525	1,61E-13
ZNF391	2,336516	0,000597
RCOR2	2,335875	7,01E-06
ANO8	2,334518	3,78E-12
ZFP14	2,331344	6,82E-09
SLC29A2	2,327156	2,82E-14
IL17RE	2,318659	0,000279
SEMA4C	2,317932	1,06E-17
ZNF32	2,308615	1,39E-16
ZNF630	2,308412	2,83E-05
MEPCE	2,308175	5,16E-49
UFSP1	2,307231	0,000298
C21orf33	2,304218	8,54E-18
UBE2Q2P2	2,30392	0,000251
ZNF217	2,300805	3,51E-41
MNX1	2,299428	1,98E-10
C2orf76	2,298511	3,20E-05
TMEM121	2,296943	0,000442
ENDOV	2,294306	6,39E-14
ZNF836	2,291937	1,82E-10
JUNB	2,291179	3,05E-09
GGACT	2,290097	0,00011
DHTKD1	2,282393	3,24E-24
TRIM24	2,280655	1,97E-27
FBLIM1	2,279257	1,92E-05
SYNE2	2,272688	6,59E-16
VSIG10L	2,272142	2,18E-08
LMF1	2,271141	3,67E-09
KATNAL2	2,27009	6,11E-05
TDRKH	2,269986	1,23E-08
ECE2	2,268901	2,53E-10
MBNL3	2,266239	4,01E-24
PYCR1	2,265347	9,01E-20
LINC00886	2,263939	0,000222
MRPL41	2,262427	7,15E-26
GLS2	2,260062	4,95E-06
AP1M2	2,258509	4,35E-24
WDR83OS	2,258252	7,73E-33
FAM134B	2,256528	1,91E-05
HSPA2	2,255231	9,21E-05
MAFG-AS1	2,254798	1,21E-05
B3GNT3	2,2536	0,00032
NIPBL-AS1	2,252857	7,89E-09

MOAP1	2,252822	4,82E-13
SPATA2L	2,252664	6,48E-16
PCDHB2	2,251345	6,66E-12
HS6ST1	2,249424	2,87E-23
NPY1R	2,246735	7,05E-11
AUTS2	2,244529	2,50E-17
GSTM4	2,241635	2,20E-17
CCT6B	2,241038	0,000264
CAMSAP3	2,239875	1,65E-22
CELSR2	2,238995	3,40E-23
SLC7A5	2,233229	1,86E-32
ZFP62	2,232677	4,16E-29
KDM7A	2,230303	2,66E-12
GLCE	2,228967	3,84E-27
SMARCD2	2,228314	1,55E-40
ZNF232	2,222564	9,56E-07
TAF11	2,220905	4,08E-22
ONECUT2	2,219912	2,11E-11
SFT2D3	2,211349	6,17E-08
FAM212B	2,210973	3,41E-06
TNS2	2,210437	3,30E-12
LIPT2	2,20881	8,75E-05
TRIM33	2,20505	2,39E-28
CCDC88C	2,203548	6,09E-11
MXD4	2,201948	1,64E-07
TET2	2,198468	6,42E-21
ULK3	2,192532	3,16E-14
HIPK1	2,190295	5,41E-31
H1F0	2,188784	1,61E-06
TMEM251	2,187755	4,70E-18
LOC10099€	2,186116	0,000221
TEAD3	2,185564	2,60E-14
DNLZ	2,183937	1,47E-18
WRB	2,178134	1,40E-13
HAGHL	2,176627	2,71E-13
ARPP19	2,175136	1,46E-35
IL17RB	2,172556	7,66E-05
RNASEH2A	2,171704	5,85E-29
EN2	2,167564	1,16E-06
ZNF276	2,16697	1,25E-17
MCM3AP-/	2,164452	1,01E-07
EPB41L4A-.	2,163602	4,38E-07
AXIN2	2,163132	2,24E-27
ASPSCR1	2,158787	1,24E-21
TMEM183/	2,158763	6,64E-30
TMEM184/	2,155808	4,46E-07
IDUA	2,154566	2,36E-06
DDR1	2,146472	1,03E-07
AGAP9	2,144319	0,000521
SLC7A8	2,14416	1,68E-06

PER3	2,133599	9,26E-05
CBX4	2,132729	7,65E-07
AIM1	2,13118	3,74E-07
ZNF713	2,126988	3,72E-07
POLR3C	2,121657	9,39E-09
RALGAPA1	2,11858	3,71E-19
SPTBN2	2,11775	3,47E-13
C15orf39	2,117	2,76E-24
JDP2	2,113403	8,69E-05
ZNF137P	2,112793	0,000472
CTXN1	2,110894	2,15E-16
ZNRF2	2,110482	3,83E-10
SAMD15	2,110305	0,000579
VPS72	2,109258	8,22E-13
AREG	2,108719	6,29E-06
ZNF682	2,108461	1,70E-06
C2orf68	2,103768	2,02E-21
HMGB3	2,103521	1,48E-37
HES6	2,102407	7,91E-05
MZF1	2,1024	0,000251
LOC72855[illegible]	2,100545	1,10E-11
WDR34	2,093554	2,37E-34
KCNK1	2,093245	4,90E-07
EFNA4	2,092331	5,76E-16
PHLPP1	2,090378	5,27E-19
NES	2,089661	6,51E-08
DSP	2,087628	1,62E-26
LOC72861[illegible]	2,087552	4,44E-05
LOXL3	2,085424	7,45E-06
ZCWPW1	2,083027	1,32E-06
PPP1R3F	2,077444	1,55E-05
SLC37A1	2,072089	6,12E-06
RANBP17	2,067143	7,37E-07
SCAND2P	2,066415	0,000518
ZFYVE19	2,060477	7,75E-14
DCLRE1B	2,060127	9,33E-16
SETDB1	2,059031	1,88E-19
ZC3H8	2,058472	2,03E-06
DNPH1	2,054215	1,42E-27
ZNF74	2,053554	9,77E-17
COX6C	2,051815	2,24E-24
HIGD2A	2,045639	2,21E-32
THOC6	2,044359	1,86E-09
CRB3	2,041261	4,04E-06
NMB	2,041243	1,40E-07
AHR	2,039354	3,64E-27
ALDH4A1	2,038374	0,000256
SNHG25	2,036844	2,68E-08
TET1	2,033618	1,99E-05
ENSA	2,030845	7,81E-37

ST20	2,030552	5,06E-05
ROGDI	2,030395	8,37E-16
DBNDD1	2,028349	7,77E-18
TCEA3	2,027118	7,71E-19
MC1R	2,025978	4,39E-06
ZNF687	2,024515	1,94E-28
TBC1D9	2,02346	1,79E-17
SIPA1L2	2,022969	1,69E-15
RABIF	2,022271	9,34E-17
TMEM141	2,018944	3,02E-22
SAYSD1	2,01787	2,86E-16
GABPB2	2,016853	3,68E-09
SRD5A3	2,009213	4,35E-13
PXN-AS1	2,007676	1,76E-07
ABHD11	2,007575	3,97E-14
ZNF829	2,007343	2,22E-08
MPC1	2,005527	1,19E-06
CCNG2	2,004081	8,68E-06
LOC101927	2,003296	1,75E-05
ECH1	2,002881	9,33E-17
ZNF572	1,994035	5,19E-05
PDE4A	1,993362	3,85E-06
THEM6	1,9929	3,66E-17
CCDC121	1,986166	0,000601
ABCA2	1,985621	1,88E-12
SEPW1	1,984439	5,29E-10
MIR600HG	1,982662	2,81E-05
KREMEN1	1,976824	1,18E-12
ELOVL7	1,975195	6,71E-13
LOC100506	1,973964	1,17E-05
VWA1	1,968207	4,82E-08
ZNRF3	1,960734	1,02E-29
ZNF595	1,960423	7,60E-10
ZNF48	1,959576	6,31E-06
POGZ	1,957371	4,98E-12
SIKE1	1,956016	8,85E-26
DDX59	1,954075	1,80E-06
GUCY1A2	1,953944	7,28E-09
PDE4D	1,95343	2,24E-06
IPO5P1	1,951811	1,63E-06
STRA13	1,951806	4,64E-21
FAM86EP	1,951769	2,38E-05
ERV3-1	1,949722	3,83E-09
MANSC1	1,94751	6,37E-06
MSRB2	1,946047	3,80E-16
COA5	1,945783	7,74E-13
FBXW9	1,944484	9,13E-15
RBM8A	1,943696	2,37E-27
IGFLR1	1,942304	0,00038
GPR160	1,935428	4,00E-06

SNAPC5	1,934273	8,01E-12
CCPG1	1,929971	3,25E-10
IDNK	1,929051	7,77E-07
KDM6B	1,928833	5,56E-18
PRKCH	1,928279	4,38E-08
KDM5B	1,92559	3,30E-11
APRT	1,922983	1,31E-29
ZBTB7B	1,922673	5,11E-23
ZNRF1	1,919419	5,26E-22
PITPNA-AS:	1,918554	0,000148
EMP2	1,916966	8,75E-10
FLJ37453	1,916827	0,000521
LYSMD1	1,914946	7,37E-11
RNF115	1,912538	4,20E-22
FKBPL	1,911745	1,36E-10
ADI1	1,907981	4,48E-20
ZNF117	1,907665	9,95E-10
FBXW4	1,906891	9,61E-14
MAPK8IP1	1,901347	8,55E-13
GAS5	1,900394	4,56E-05
ZSCAN29	1,899377	2,36E-09
PRR19	1,898526	0,00046
C16orf46	1,898082	8,20E-06
CCDC24	1,89647	3,44E-05
ISL2	1,8955	0,00029
ZFHX3	1,895056	2,93E-21
MTL5	1,893583	1,99E-06
TSEN54	1,893139	1,72E-15
ANKRD16	1,889692	3,28E-06
NSUN7	1,888577	3,25E-05
OBFC1	1,888482	5,88E-07
EVPL	1,884409	1,01E-13
C14orf80	1,8824	2,16E-15
ZSCAN21	1,882254	3,79E-14
FZD1	1,87683	4,48E-13
TST	1,872894	1,15E-06
PACS2	1,869821	1,28E-21
CBX8	1,869051	6,75E-07
OCLN	1,865536	6,19E-07
C6orf48	1,86144	1,66E-05
PARD6B	1,85881	9,91E-22
ATP6V1E2	1,858808	0,000551
C9orf16	1,858239	2,42E-12
ETNK2	1,857154	4,93E-06
ZNF43	1,85598	7,47E-17
PWWP2B	1,855338	2,99E-14
METRNL	1,854535	9,53E-14
ZFAS1	1,853275	0,000629
HOMER2	1,850528	3,79E-15
C3orf14	1,850205	2,93E-13

TNPO2	1,84809	3,80E-08
ADAL	1,845786	3,66E-07
SPINT2	1,843521	3,54E-27
ATAD2B	1,842863	2,26E-07
PATZ1	1,8419	3,88E-11
PGM2L1	1,841595	2,43E-10
CSK	1,839011	7,48E-10
GABBR1	1,834992	4,37E-05
KLF4	1,833229	7,15E-07
OARD1	1,833143	4,37E-13
RHOD	1,830081	1,71E-06
GLI4	1,828022	6,02E-06
PCOLCE	1,827466	7,55E-05
PP7080	1,827343	3,14E-10
PSMD5-AS:	1,826244	3,31E-06
GBAP1	1,825275	7,88E-06
C8orf82	1,825195	3,08E-13
THNSL2	1,823537	4,05E-05
HDAC5	1,82076	2,15E-06
ABCD1	1,818409	0,00057
SLC50A1	1,818105	1,41E-16
ZNF252P	1,814197	1,09E-17
OGDHL	1,813466	7,63E-07
RAB27B	1,811788	1,22E-08
ARG2	1,811146	0,000147
COX5B	1,808161	2,99E-25
TPRN	1,807805	2,97E-11
NQO2	1,805988	5,96E-15
ASNS	1,805845	1,52E-05
PLEKHO1	1,805004	6,22E-16
EPHX2	1,804143	0,000635
COMTD1	1,801942	3,08E-11
ZNF627	1,801036	3,28E-13
WWOX	1,797486	3,05E-09
SCD5	1,788683	2,56E-07
LRRC75A-A	1,782981	9,87E-08
CYTH2	1,781121	2,75E-19
PLEKHH3	1,780553	0,000303
HDDC3	1,778903	1,48E-08
CYP2J2	1,776492	4,22E-05
ZFAND2A	1,775403	6,32E-08
SPATC1L	1,774551	4,32E-05
ATXN7L3B	1,77364	1,10E-28
TMEM254	1,772268	1,09E-11
GPR89B	1,772157	7,47E-05
ZNF3	1,771917	5,85E-16
PCBD2	1,771361	1,58E-05
SLC25A10	1,770013	1,24E-19
PPM1L	1,769984	0,000267
SLC22A18	1,767483	3,14E-06

PAQR4	1,767117	3,08E-22
DLD	1,767111	2,57E-10
TRAF4	1,76566	1,07E-23
E2F2	1,763265	0,000663
TACO1	1,763086	2,39E-23
FAM171B	1,761884	1,27E-08
ALDH6A1	1,760956	1,25E-14
ZIC1	1,758828	1,28E-05
RSBN1	1,753511	6,99E-11
NOL3	1,751978	9,09E-10
KCTD15	1,751904	4,64E-05
JARID2	1,751395	1,76E-18
RHPN1	1,749609	2,74E-09
ZNF184	1,749495	2,40E-12
TDG	1,74883	1,82E-21
PNPLA8	1,746555	3,81E-05
ATXN7L1	1,7454	2,82E-08
PTPRU	1,744021	1,29E-09
PSD4	1,743549	8,26E-10
NFYA	1,737901	3,80E-11
ZNF287	1,73742	3,27E-05
HARS2	1,736682	2,24E-17
CBLL1	1,734769	2,10E-16
MZT2B	1,734738	4,34E-12
NAPG	1,733509	2,84E-09
ZNF821	1,729042	2,22E-05
SLC16A13	1,728545	0,000528
CREG1	1,726799	4,21E-15
SMG7	1,725565	6,84E-25
NUDT14	1,725548	4,04E-12
ARHGEF35	1,72518	0,000143
EPHB4	1,723866	3,38E-27
NUDT3	1,722233	8,96E-08
PLXDC2	1,72149	6,77E-08
RPP40	1,719833	2,68E-10
UBE2T	1,719245	9,73E-16
RAB3IP	1,715905	5,40E-19
ALDH16A1	1,715161	2,35E-12
ZNF786	1,713578	3,65E-13
RAB15	1,711611	6,98E-13
GALNT18	1,709604	0,000192
RPRD1A	1,709456	1,05E-18
SLC29A4	1,706703	3,16E-10
DGCR6L	1,705664	1,37E-16
ALKBH4	1,704876	2,25E-08
HSCB	1,704364	1,47E-08
ZNF85	1,702434	4,24E-10
ZNF653	1,699191	5,97E-05
EXD2	1,698477	2,07E-16
SLC3A2	1,698118	2,41E-21

PRSS23	1,69808	4,48E-08
MIF4GD	1,694949	1,91E-07
SERTAD3	1,69461	9,27E-07
NSD1	1,6938	3,90E-26
GPR89A	1,692762	1,04E-07
ISOC1	1,692276	2,51E-17
FLJ42627	1,689627	0,000331
ZNF425	1,687662	3,14E-06
LOC104968	1,686623	0,000414
HEXDC	1,685924	5,92E-07
BRWD1	1,684039	1,76E-15
DANCR	1,682698	7,50E-10
GSTA4	1,681126	3,10E-05
HILPDA	1,675813	7,45E-08
MXD3	1,67566	8,74E-08
WDR73	1,675025	5,19E-15
SNRPE	1,672792	2,83E-23
GTF3A	1,669102	9,85E-23
HBP1	1,665234	9,72E-05
DMPK	1,663759	4,96E-07
NRAS	1,663023	4,82E-15
DHPS	1,662845	4,24E-14
MAML1	1,659999	2,87E-11
CDC25C	1,656653	0,000205
CHMP1B	1,655761	1,06E-13
BCKDHA	1,652844	1,61E-11
KIF27	1,652026	0,000724
TMC5	1,648705	0,000411
DOCK6	1,648425	4,01E-12
CBWD2	1,646989	6,08E-10
TATDN1	1,646135	6,33E-16
RNF5	1,645704	6,71E-09
ZNF569	1,644548	2,10E-05
TRIM41	1,642613	6,40E-13
CDC25B	1,641436	1,02E-14
ZNF14	1,639776	0,000421
PARP6	1,639656	4,37E-08
TRIM45	1,63943	3,68E-05
RMND5A	1,638852	1,90E-13
C15orf61	1,63643	1,87E-05
MPV17L2	1,635705	6,81E-11
ZNF628	1,634885	1,88E-09
N4BP2	1,634299	2,07E-07
CERCAM	1,634183	5,88E-05
PRADC1	1,632957	2,78E-05
DTNB	1,631973	1,06E-08
FCHO1	1,631875	1,69E-15
LDOC1	1,630616	2,08E-09
ACACB	1,629948	2,45E-09
RAB33B	1,628877	0,000218

EDC3	1,628271	9,71E-11
FAM117B	1,627442	3,66E-09
ARRB2	1,625844	1,11E-14
RAB30	1,62417	2,22E-06
MEX3B	1,623727	4,80E-11
PGBD2	1,622252	0,000125
TPD52L1	1,62217	1,55E-06
PRDM4	1,620696	2,68E-23
TMEM168	1,6128	2,06E-13
IRF2BP1	1,611058	4,27E-10
EZH2	1,609127	5,56E-07
ICAM3	1,606861	9,05E-05
MAZ	1,605506	5,86E-16
GPATCH2	1,598111	1,02E-09
GALNT6	1,596687	7,12E-06
DYNLL2	1,596291	7,56E-20
ABHD16A	1,595853	1,33E-12
CHD6	1,595619	9,84E-20
UPF3B	1,59479	7,05E-08
RPRD2	1,59466	2,57E-17
UBL3	1,594314	3,85E-08
ENDOG	1,593361	6,25E-10
ABCC10	1,592008	1,39E-08
MRPS21	1,590781	1,58E-18
SLC25A29	1,586224	6,16E-13
GTF2IRD2B	1,584579	2,79E-07
TLE3	1,583143	1,11E-21
PHYKPL	1,582337	0,000106
P3H4	1,581888	2,84E-14
INTS3	1,581884	6,23E-19
KIFC2	1,580916	1,89E-07
SLC25A39	1,576125	1,53E-23
ATP6V0E2	1,571539	1,24E-06
PRUNE	1,569745	9,00E-13
ZNF816	1,569723	8,98E-07
ZNF618	1,569634	5,76E-18
SLC9A3R1	1,56699	2,17E-08
ARRDC1	1,566696	1,04E-11
SEC11C	1,566489	7,05E-07
DUSP23	1,566329	6,93E-09
NGRN	1,564702	7,08E-21
PAFAH1B3	1,563703	5,84E-15
DSC2	1,561074	1,02E-11
C14orf2	1,559491	1,01E-15
HAGH	1,559293	5,54E-06
LRRC45	1,556621	5,81E-12
ZNF480	1,554855	5,83E-09
CDC42BPG	1,554421	1,29E-05
TRMT12	1,554306	5,79E-10
RUSC1	1,550263	2,48E-11

PNMA1	1,549977	7,66E-11
NAAA	1,545764	2,65E-05
LRFN3	1,545355	5,72E-06
PPP1R13B	1,544826	1,62E-08
ECI1	1,543203	1,11E-13
KCTD1	1,542665	1,93E-06
OIP5-AS1	1,542148	3,16E-09
SULF2	1,541609	4,33E-09
IDH2	1,53825	2,09E-19
HOXC10	1,537991	1,49E-10
ZC3H10	1,533814	1,08E-05
NDUFB9	1,533112	1,20E-10
EFNA1	1,533018	0,000307
MYL12B	1,532813	1,29E-18
NUDT4	1,531713	5,29E-09
ACSF3	1,5313	2,15E-13
RPAIN	1,530425	1,22E-11
ZKSCAN1	1,52871	3,19E-15
PDE7A	1,528217	2,14E-07
JOSD2	1,527757	0,000115
TOB1	1,527733	1,56E-07
JTB	1,525035	7,58E-15
ID1	1,524201	0,000122
ZNF138	1,524171	3,28E-09
RAD23A	1,523598	2,38E-17
RNASEH1-A	1,523381	7,58E-08
TOR3A	1,523363	2,54E-14
ZSCAN2	1,523296	1,53E-09
GRK6	1,52009	3,48E-16
KCTD13	1,51858	9,87E-08
COIL	1,517426	2,73E-05
ADIPOR1	1,51726	2,81E-07
EXOSC5	1,517195	1,48E-06
MALAT1	1,516656	3,72E-06
DUS4L	1,513572	7,28E-08
RAB24	1,509832	3,67E-07
FAM50B	1,508813	1,18E-07
TRIB1	1,507822	3,92E-08
RP9	1,50756	1,49E-06
SRPK2	1,506643	2,24E-15
PYCARD	1,506346	6,22E-05
METTL2A	1,505545	5,73E-12
NECAB3	1,505224	3,13E-05
CASZ1	1,50189	0,000172
RRBP1	1,500525	8,36E-07
BPTF	1,498528	1,16E-19
ATP5I	1,49839	1,76E-16
POLR2J	1,49771	5,04E-10
ATP5S	1,496317	0,000379
BMP2	1,496263	3,22E-10

FAM193B	1,495981	3,10E-06
PACSIN3	1,495808	3,84E-14
DDX49	1,494249	4,50E-14
TPCN2	1,493935	6,88E-06
ORC5	1,491379	2,63E-05
CEP76	1,491207	0,000303
FLJ23867	1,490059	5,13E-06
BCL7A	1,489947	8,42E-10
TBC1D9B	1,489875	2,98E-16
DNAAF2	1,486262	2,54E-11
ATXN3	1,485747	3,56E-07
ILVBL	1,483438	1,67E-16
POLR1D	1,482147	5,56E-17
L2HGDH	1,480757	7,12E-12
FUNDC2	1,480443	6,90E-11
TARS2	1,476779	2,61E-09
FIS1	1,476166	1,90E-13
PDCL3	1,47549	2,95E-09
ABCA7	1,475094	0,000757
CCDC97	1,473313	4,70E-12
PRR3	1,473248	1,68E-05
RALGDS	1,469956	5,42E-11
C6orf47	1,466296	1,36E-12
C19orf43	1,465943	1,04E-18
KANSL1L	1,465389	0,000273
ZNF398	1,464471	8,89E-15
H2AFY2	1,463608	6,17E-08
LINC00674	1,463297	2,01E-08
TBC1D7	1,460546	9,25E-08
COBLL1	1,460093	4,41E-06
SORD	1,459832	1,80E-16
PRPF3	1,4587	1,50E-06
ZNF710	1,456546	7,54E-09
CROCC	1,456025	1,51E-05
GIN1	1,45588	6,54E-05
ATG4D	1,454948	3,57E-06
PIF1	1,454519	0,000546
FBXO31	1,453293	1,88E-12
SFXN4	1,45255	2,05E-10
ZFP36	1,451689	8,65E-06
HMG20B	1,451188	1,85E-17
SEPHS2	1,450793	1,02E-15
TYSND1	1,450791	1,41E-12
SNHG7	1,450144	3,95E-10
CCDC101	1,449379	1,28E-06
ACTR6	1,446743	1,05E-08
PPIP5K1	1,4444	5,04E-06
ATMIN	1,443798	3,13E-15
THAP7	1,443259	6,02E-08
ZNF33B	1,442767	1,77E-07

SLC25A43	1,442365	2,74E-09
MTMR4	1,4413	4,16E-14
NUCKS1	1,440085	6,17E-16
WDR60	1,439334	4,03E-08
ACOX3	1,438806	1,44E-05
SAMD1	1,438533	2,93E-09
ZBTB22	1,436147	2,26E-07
BCAM	1,43502	7,43E-08
F8A1	1,434772	5,73E-06
SMDT1	1,434168	4,71E-06
MFSD2A	1,43197	2,55E-05
FITM2	1,431775	3,05E-05
UBN2	1,428582	7,00E-09
STMN3	1,428122	3,57E-06
ASNA1	1,426468	1,24E-17
FAM120AC	1,426232	8,93E-11
ZNF514	1,425998	4,04E-05
FLAD1	1,4232	5,07E-15
FAM206A	1,422883	3,09E-06
EPC1	1,421949	2,61E-11
ITPKB	1,421372	2,21E-05
C14orf132	1,421068	6,77E-08
SURF1	1,419919	2,13E-06
STX6	1,419762	6,26E-13
LINC00938	1,416554	0,000611
KIAA1958	1,4161	2,04E-07
PSMD4	1,415487	2,09E-19
SLC27A4	1,414754	6,56E-06
ZBTB5	1,412679	4,16E-07
CGRRF1	1,41163	0,000627
CENPB	1,409602	4,98E-17
ANKRD13B	1,407724	1,07E-08
RARG	1,407569	1,56E-08
CNNM3	1,402915	7,94E-08
TMED3	1,402225	4,55E-09
BBS10	1,40215	1,51E-05
STK19	1,401946	2,93E-05
MAP3K6	1,401428	9,44E-08
CDC42SE1	1,398722	4,39E-10
PPAP2A	1,397232	1,67E-05
C1orf43	1,396714	1,87E-13
UBAC1	1,396665	1,22E-13
NARF	1,39158	0,000406
EIF4EBP2	1,390731	2,57E-06
TBCC	1,39002	9,78E-08
RUFY1	1,389827	4,57E-09
SLC44A2	1,389177	1,66E-11
CNOT8	1,388763	2,16E-11
ZNF592	1,387019	1,03E-09
CBS	1,386494	1,58E-08

NR2F6	1,385107	8,86E-07
DCP2	1,384963	2,35E-12
CHRNA5	1,382184	2,29E-09
VTI1B	1,376404	1,56E-07
ZBTB9	1,376013	7,97E-09
FAM72D	1,37587	0,000957
PARP16	1,375123	3,10E-05
MORC2	1,372516	3,79E-12
AMER1	1,372117	1,37E-06
PPIC	1,371718	5,79E-08
MRPS18A	1,369796	1,12E-12
TMBIM4	1,369615	2,23E-07
VARS2	1,365918	3,74E-08
ANKRD39	1,364679	2,99E-05
FLOT2	1,364621	4,51E-08
WIBG	1,364158	3,10E-08
APH1A	1,364087	2,79E-15
CKB	1,364005	9,40E-06
LMNB1	1,362999	1,66E-10
C19orf60	1,362383	4,69E-08
ZNF718	1,362311	5,79E-05
ZNF254	1,36112	1,75E-07
CRIP2	1,360585	1,62E-12
ZNF607	1,360134	8,41E-07
C14orf142	1,356756	1,28E-05
PIGM	1,356154	2,84E-05
MPZL3	1,355451	0,000235
PIGB	1,354884	4,01E-06
ZFP1	1,353978	4,52E-07
TEF	1,353923	0,000605
DDX41	1,353748	2,06E-15
CLN6	1,353063	4,03E-15
TMEM79	1,351197	2,77E-05
CHCHD5	1,35111	4,55E-07
BNIP3	1,348363	4,04E-10
BOD1	1,347495	2,75E-06
GOLPH3L	1,347056	0,000227
IDH1	1,346768	1,09E-16
C7orf43	1,346686	1,47E-06
LAMTOR2	1,344209	4,18E-11
ZNF766	1,344175	9,17E-10
HAX1	1,340957	5,41E-15
RALBP1	1,338897	7,78E-08
RPS18	1,335588	7,28E-06
SNX27	1,335085	1,47E-10
TEX2	1,333428	8,08E-10
PTRH2	1,332454	5,49E-09
SH2B2	1,332168	0,000162
PCDHGC3	1,331745	0,000268
H1FX	1,33097	1,63E-06

C9orf114	1,329363	3,79E-07
KIAA0195	1,327181	5,29E-10
MIF-AS1	1,327119	7,03E-06
CINP	1,326595	0,00019
KLHDC3	1,325751	6,10E-07
COX6B1	1,325317	1,39E-09
TRAPPC13	1,322413	3,13E-07
MARK3	1,321916	1,41E-07
EIF3K	1,321342	2,01E-08
IFT22	1,320591	1,86E-11
ZSCAN9	1,319561	0,000571
ARHGAP39	1,318249	9,25E-05
SURF2	1,317449	7,43E-09
TRIM39	1,317446	0,000174
ENTPD5	1,315403	0,000457
SLIRP	1,314654	2,32E-07
CCDC112	1,314605	4,05E-05
PPP4R3A	1,313882	4,82E-13
TMEM161/	1,313745	2,03E-05
DHFRL1	1,311467	1,82E-05
SCYL3	1,310067	0,000164
FAM213A	1,309754	2,63E-05
TMEM205	1,309164	1,89E-06
ZNF431	1,308815	1,16E-05
ZNF557	1,308411	9,64E-05
ARL4A	1,30814	1,47E-09
MYO1D	1,30562	8,61E-06
AP5M1	1,305431	2,59E-07
GAA	1,305145	5,04E-06
DHRS7B	1,304552	1,59E-06
PSMA3-AS:	1,304373	0,000112
PMVK	1,303454	3,45E-08
TNK1	1,302765	2,12E-06
HNRNPAB	1,302352	3,87E-15
DYM	1,300774	1,00E-11
TMEM134	1,298619	7,44E-05
SIPA1L1	1,297727	3,36E-11
SNRPA1	1,296739	1,20E-11
GCLC	1,296571	3,04E-07
RPS29	1,296345	2,95E-06
BNIP1	1,296174	0,000756
CACNB3	1,294821	5,67E-10
ZNF93	1,294771	5,55E-07
ZNF839	1,2942	0,000291
GADD45GI	1,292725	1,89E-05
SIVA1	1,292304	1,33E-07
SPHK2	1,292007	1,02E-06
PFKL	1,290659	4,23E-10
SGPL1	1,289452	4,00E-10
RINT1	1,28878	8,94E-06

VMP1	1,288239	1,38E-08
PUS7	1,287764	9,65E-07
XXYLT1	1,285903	6,38E-11
ZNF675	1,284236	1,47E-05
HSPB1	1,283988	2,14E-07
MYL12A	1,283186	1,70E-10
TMEM185E	1,280664	7,85E-09
H3F3B	1,276933	5,57E-16
RBAK	1,27681	2,65E-09
TMEM64	1,2758	6,29E-12
ZNF524	1,275505	9,93E-05
ICK	1,274747	3,88E-09
FARSA	1,274338	1,22E-07
TIGD6	1,273967	0,000322
TLK2	1,273486	0,000186
PITX1	1,273249	8,86E-09
MRPS18C	1,272793	5,15E-08
STAP2	1,271932	2,68E-06
RNMTL1	1,27127	1,29E-07
PYGO2	1,271099	6,75E-10
ZNF771	1,270292	0,000648
OTUD7B	1,270072	2,03E-11
SIX4	1,265772	2,22E-06
TRIM26	1,264596	2,39E-09
BCAP31	1,264373	1,49E-08
SERF2	1,264052	4,67E-08
ADCK2	1,26366	5,82E-06
CTBP1-AS2	1,262355	2,81E-05
PAN2	1,261705	0,000122
NDUFA13	1,261085	1,92E-12
CXorf23	1,255612	0,000254
FLYWCH2	1,254953	3,07E-06
NELFE	1,253657	3,56E-13
UHRF1BP1	1,253474	2,55E-05
SIRT5	1,251422	3,95E-07
AIMP2	1,250537	3,90E-11
TPD52	1,249715	2,22E-10
TP53TG1	1,249601	0,000179
THOC7	1,249052	6,97E-12
CALM1	1,248208	1,54E-06
AADAT	1,248081	0,000306
RBM4	1,247185	4,48E-09
MED13	1,246786	3,65E-14
ZNF616	1,246582	2,97E-07
IGF1R	1,244291	7,23E-10
OXLD1	1,244122	0,000253
MEA1	1,243672	2,04E-12
CYB5D2	1,243315	2,95E-05
FRS2	1,243175	4,72E-05
FAM3A	1,24313	4,55E-07

ZNF219	1,242669	5,60E-06
RPS14	1,241822	1,12E-09
D2HGDH	1,241553	1,18E-05
NKRF	1,239361	4,19E-08
SQLE	1,238515	1,50E-09
FZD3	1,236343	0,000183
ZNF212	1,234692	1,66E-06
FAM83H	1,233496	8,49E-09
DARS2	1,233013	2,67E-11
IQCK	1,232595	3,19E-05
PVT1	1,231738	0,000132
PCLO	1,231017	4,81E-05
C19orf52	1,230564	1,43E-05
CCDC47	1,230343	6,40E-10
ZRANB1	1,22812	6,25E-08
THNSL1	1,227878	1,13E-08
OBSL1	1,22742	0,00025
CUL7	1,227213	1,13E-05
SUPT4H1	1,226992	4,27E-10
FEM1B	1,226906	7,28E-10
ETFA	1,226015	1,60E-06
MAP2K5	1,225737	6,94E-07
ARF5	1,225282	2,81E-12
LINC00665	1,223395	4,15E-05
ZNF429	1,222025	3,17E-05
DLG3	1,221698	3,88E-09
ZNF286A	1,220687	2,31E-07
ZNF33A	1,219815	1,15E-06
RPS11	1,219385	1,59E-07
CUX1	1,21863	1,44E-09
NFKBIL1	1,218572	2,80E-07
DPY19L3	1,215584	2,87E-06
KRTCAP2	1,214514	7,10E-11
DTWD2	1,213619	0,000186
NOL11	1,212341	1,85E-12
CTSH	1,212002	7,44E-05
CNNM4	1,211538	3,58E-06
SLC5A6	1,211157	0,000112
BRPF3	1,210367	4,32E-08
ELP5	1,210053	4,11E-08
ZNF620	1,209545	0,000702
RPS27	1,209133	7,23E-05
ATP5D	1,209044	5,46E-12
ZNF688	1,20864	0,000527
EFCAB11	1,208074	0,000108
OXR1	1,207993	2,16E-06
MED7	1,207745	0,000505
PPL	1,206584	0,000429
KNSTRN	1,205641	0,000391
PCGF3	1,204711	2,41E-09

NSUN5	1,204435	7,10E-08
UFC1	1,203503	0,000284
WDR62	1,203137	5,65E-07
HKR1	1,202247	2,81E-05
RPL17	1,202198	1,59E-06
CKS1B	1,201859	0,000249
PTRHD1	1,201371	0,000175
ACP2	1,201133	5,00E-05
RFXANK	1,200478	1,61E-09
FUT8	1,199375	2,22E-09
ZNF600	1,198505	0,00044
ZMYM2	1,196812	5,61E-10
ZNF140	1,196658	2,92E-05
ZNF91	1,196344	6,81E-06
BLOC1S3	1,195558	5,16E-06
MAPK13	1,195414	6,81E-11
PSMB4	1,195133	6,12E-09
ZDHHC24	1,190346	0,001005
UBE2Q1	1,189947	4,81E-10
SLC52A2	1,188135	7,56E-09
ZNF444	1,187195	7,58E-07
GPSM2	1,186792	2,94E-09
SS18L1	1,186622	0,00092
CDYL	1,186273	1,39E-08
PPP1R13L	1,185847	0,00039
ZNF768	1,184559	1,59E-07
TUBB3	1,18444	5,96E-13
USF2	1,183906	3,43E-11
ATXN7L3	1,183861	5,01E-11
POLR2I	1,183618	1,44E-08
PPDPF	1,182207	3,77E-08
PTGR2	1,181889	0,000336
ACOT13	1,181692	8,53E-06
CD24	1,181403	8,65E-07
MRPL57	1,180846	1,53E-09
SHTN1	1,180714	8,81E-09
ZNF525	1,180115	1,73E-05
DUS1L	1,180113	2,86E-07
TAMM41	1,179963	3,21E-06
GLRX2	1,178972	3,67E-05
ARNT	1,176738	3,82E-10
TDRD3	1,172031	7,85E-05
COPE	1,172003	1,78E-10
ANGEL1	1,17198	4,19E-08
RDH13	1,17102	1,97E-06
ILF3-AS1	1,170629	0,000498
C11orf80	1,169342	0,000126
C7orf26	1,169013	1,02E-08
KRT10	1,168858	0,000383
ARID4A	1,167786	3,44E-05

NUSAP1	1,167658	1,53E-05
MRFAP1L1	1,165937	2,05E-09
ARMC10	1,165823	2,69E-08
RNF113A	1,165387	8,89E-06
NAA10	1,164329	3,66E-11
EXOSC4	1,163997	1,66E-08
MRPL9	1,163947	2,03E-06
CCZ1	1,163923	9,91E-05
STK38	1,162787	3,79E-08
PEX1	1,162727	3,83E-06
SEC11A	1,162198	2,96E-12
ZFP36L2	1,160649	0,000432
SPATA5L1	1,159729	0,000322
ATP5A1	1,159415	7,72E-14
TPT1	1,159005	1,50E-06
KIF20A	1,158913	1,18E-06
SLC35B2	1,157203	3,76E-05
RNF130	1,157175	3,56E-06
PRELID1	1,156566	5,43E-10
MTERF1	1,156521	0,000127
SIX1	1,156111	0,000107
CCDC85C	1,1549	1,78E-10
YIF1A	1,154567	4,11E-10
CISD3	1,153285	0,000265
C1orf27	1,151741	2,75E-05
SF3B4	1,150994	2,46E-12
NT5C	1,150777	0,000139
GNB2L1	1,150722	2,90E-13
DFNB31	1,15047	0,000919
ZNF888	1,146011	0,000222
CTNNBIP1	1,145051	1,08E-05
HCFC1R1	1,144724	4,58E-08
MBTD1	1,142643	0,000419
ARL8A	1,141311	5,16E-09
DENND2D	1,140864	4,96E-07
PLCH1	1,140688	1,39E-06
EPC2	1,14058	3,45E-06
NDUFB7	1,14025	7,60E-11
ZNF777	1,138696	0,000154
IMP4	1,137457	2,23E-11
TSPAN17	1,13737	1,47E-05
VANGL1	1,135268	1,21E-05
NUDT12	1,134553	0,00072
IMP3	1,132458	8,40E-09
CC2D1A	1,131987	9,67E-06
GGCT	1,130412	9,97E-11
H2AFJ	1,125467	0,000103
DNMT3A	1,125311	3,48E-08
ZNF511	1,124341	2,38E-05
SSR4	1,124127	5,69E-08

OFD1	1,123445	1,60E-05
ALKBH7	1,122279	7,78E-07
MACROD1	1,121271	1,08E-05
GOT1	1,119167	7,14E-07
MRPS12	1,119122	4,63E-07
RFWD2	1,117103	3,91E-08
LENG1	1,115837	0,000976
KLHL25	1,115422	7,32E-05
SSNA1	1,115195	1,12E-06
SMG8	1,115073	8,82E-07
RPL18A	1,114696	3,31E-07
FAM127B	1,113995	2,42E-09
EVL	1,113245	7,31E-07
LDB1	1,113149	3,81E-09
COX6A1	1,111442	9,55E-12
EHMT2	1,111426	1,21E-11
EMC9	1,111	0,000347
RCOR1	1,109622	1,18E-08
LMAN2	1,109396	7,68E-08
ZNF721	1,108542	7,37E-07
R3HCC1L	1,107205	1,52E-05
EIF2B2	1,106704	3,13E-09
MCUR1	1,106585	6,76E-05
HTRA1	1,10105	5,88E-06
ATP6AP1	1,100916	6,40E-05
IQCE	1,099982	1,01E-07
GATAD1	1,098679	0,000315
RPS19	1,098594	3,98E-07
RPLP1	1,098446	0,000475
TKFC	1,098276	1,09E-07
NDUFAF7	1,09814	0,000218
ZNF787	1,097921	1,12E-07
ORC2	1,096729	3,12E-05
MRPS36	1,09671	0,000111
HMGN1	1,095538	1,14E-09
YIPF2	1,095404	1,75E-06
NDUFA1	1,09502	2,12E-07
SEPHS1	1,095011	1,19E-05
ECHS1	1,09385	7,62E-08
SRP19	1,092837	1,48E-06
KNOP1	1,091947	8,06E-09
TMEM97	1,091508	1,07E-08
CHD3	1,090558	7,21E-08
CCNJ	1,090491	1,50E-05
FAM58A	1,090235	8,58E-06
PRPSAP2	1,090006	0,000567
TRMT2B	1,089556	0,000165
COX7C	1,089051	6,76E-11
PIP5K1A	1,087698	5,88E-05
CYB5A	1,087095	0,000482

KIFC1	1,085756	2,23E-05
ZNF260	1,084164	3,34E-06
NDUFB1	1,083106	2,27E-06
ZNF701	1,083022	0,000578
SPG21	1,082458	2,98E-10
DNAJC2	1,080828	1,69E-09
DBN1	1,079693	1,76E-11
PROSER1	1,079553	2,08E-10
CECR5	1,078596	2,68E-08
BRD2	1,078086	4,17E-05
ZNF316	1,077122	9,31E-09
C7orf50	1,076295	1,52E-06
NPM3	1,075615	1,29E-07
ATP5H	1,072828	9,49E-07
MARC1	1,070507	1,74E-05
SREBF1	1,070243	1,66E-05
PTPN18	1,070185	1,74E-07
PSRC1	1,068838	0,000576
SLC39A9	1,068167	1,76E-09
STK36	1,067138	1,02E-05
NDUFA3	1,06694	3,86E-05
PGGT1B	1,06591	2,01E-05
VPS52	1,065669	2,07E-09
FAM195A	1,065639	4,41E-07
PRR7	1,06491	1,72E-06
CNOT6	1,063613	1,95E-07
UQCC2	1,063548	0,000222
ATAT1	1,062613	0,00045
C19orf24	1,062087	2,87E-05
PANK3	1,060915	8,75E-08
PSMC5	1,060497	3,74E-06
CHMP4A	1,059652	6,90E-05
GLRX5	1,057494	4,86E-06
ECSIT	1,056573	1,53E-06
NSDHL	1,056435	1,29E-06
TMEM11	1,054716	4,29E-07
PPP1R11	1,054686	4,11E-06
VPS45	1,052955	2,93E-06
ZMYM5	1,052898	0,000449
NDUFA2	1,052716	2,73E-06
EMC4	1,051856	6,22E-05
SUCLG1	1,051605	2,94E-08
TAF7	1,050984	4,66E-09
YDJC	1,049834	3,24E-05
GAS8	1,049157	2,37E-05
RAE1	1,047321	7,13E-07
ANKS1A	1,047013	7,18E-07
LRWD1	1,045152	0,000241
TRAPPC6B	1,044828	5,30E-05
GLTSCR1	1,044678	0,000324

RSAD1	1,044532	1,58E-05
GPRC5C	1,04393	0,000689
NKAP	1,043919	0,000129
ATG10	1,043887	0,00092
PWWP2A	1,04328	3,18E-05
MAD2L1BP	1,042736	0,000306
ZNF100	1,042659	4,91E-05
EBAG9	1,041249	0,00019
SOS1	1,03875	2,42E-07
SYAP1	1,038466	7,15E-07
MED6	1,037259	7,02E-05
ZNF652	1,035869	2,09E-06
PBX2	1,03216	4,90E-08
PSEN1	1,03154	2,03E-06
S100A13	1,030536	0,000123
NKD1	1,030399	0,000115
FAF2	1,028519	7,49E-09
ZNF576	1,027581	0,00046
CDKN2C	1,02714	0,000707
IDH3G	1,026526	1,56E-07
FAM89B	1,024747	7,84E-07
SNX18	1,022173	0,000124
VIMP	1,022124	5,23E-07
SSR2	1,021429	2,88E-05
FXR2	1,020772	1,75E-07
EML1	1,020443	3,55E-05
RNF139	1,019528	6,70E-07
DGKZ	1,019516	4,32E-06
RPS17	1,018586	5,88E-05
NRBP1	1,01711	1,90E-07
SLC25A5	1,01565	8,81E-05
USP39	1,013339	3,17E-09
THAP5	1,011092	4,52E-07
LAMA5	1,010407	8,39E-09
PAIP2	1,010282	0,000294
NOP16	1,009898	1,22E-08
SYTL2	1,008827	0,000112
RNF181	1,008794	6,35E-07
ZNF510	1,008034	0,000551
AFG3L1P	1,007253	0,000356
TRIM28	1,006888	3,32E-09
SOS2	1,006102	7,33E-05
MAN2B1	1,005707	5,05E-05
ILF2	1,005499	2,59E-10
C9orf156	1,004504	0,000458
FAHD1	1,003932	3,72E-07
HDHD3	1,003538	0,000297
TOMM40	1,002676	3,06E-10
ZNF83	1,002619	0,000783
ARVCF	1,002019	7,32E-05

MRPL24	1,00175	6,46E-05
SRGAP2	1,000732	2,20E-08
MCM3AP	1,000096	1,04E-07
DPP7	1,000057	6,54E-08
ZDHHC9	0,999744	0,000307
CTPS2	0,999318	1,21E-06
GRB14	0,998643	0,000351
CFAP36	0,995041	0,000161
ANK3	0,994939	0,000491
IFT43	0,994718	0,000375
ANAPC2	0,994406	9,47E-05
TRAK2	0,993526	5,62E-09
RRN3	0,993153	3,23E-07
ACBD6	0,992356	4,87E-08
FTSJ3	0,992252	2,64E-05
SPR	0,992145	7,89E-07
CTC1	0,991095	0,000266
ZNF544	0,990198	2,96E-06
IVD	0,989218	5,26E-05
GNB5	0,988961	0,000274
DDX20	0,988869	1,72E-05
MRPL21	0,98832	4,18E-07
ZC3H11A	0,987865	0,000873
TMEM87A	0,98658	1,24E-05
LHPP	0,986514	0,000794
KCTD3	0,984752	6,37E-07
COX5A	0,984671	4,27E-08
LAMTOR4	0,984494	1,71E-06
HCG18	0,983973	1,41E-05
PRR14L	0,982547	2,05E-07
RPL37	0,982187	0,000108
GPX4	0,981843	2,83E-09
LRRC8B	0,981794	0,000852
CNOT3	0,9812	2,07E-05
MCL1	0,978679	1,16E-09
GTF2IRD1	0,97795	2,67E-05
BAG5	0,977259	5,82E-05
STK32C	0,975898	0,000441
TP53I13	0,975232	0,000155
CCDC115	0,974605	0,000135
ZNF205	0,97434	9,02E-05
CDK8	0,973828	3,35E-05
FAM189B	0,973278	0,000529
ZNF304	0,97294	0,00036
SETD3	0,972708	1,07E-06
RBBP5	0,971216	6,86E-07
TRMT5	0,97106	4,10E-06
ZNF362	0,970758	0,000738
DOLK	0,97025	0,000383
SYPL1	0,969505	7,20E-06

RPL30	0,967371	0,000571
ZBTB1	0,966882	1,99E-06
RPL13	0,96623	1,29E-05
FIBP	0,966061	2,24E-07
RPL31	0,965808	9,29E-06
SLC25A13	0,965507	0,000188
SLC25A11	0,964586	3,07E-06
ERCC3	0,962141	5,65E-07
VPS28	0,961452	7,93E-06
SLC10A3	0,960473	0,000194
AHSA1	0,959109	1,52E-06
FAM53C	0,957808	0,000246
ZNF778	0,955773	0,0007
CTBP2	0,955206	3,67E-07
TMED10	0,954572	7,80E-06
CUTC	0,954462	0,000693
ALKBH1	0,954444	0,000275
EAPP	0,953645	7,05E-05
HNRNPA0	0,953554	1,35E-05
KMT2E	0,952828	3,35E-05
CBR4	0,9524	0,000102
UFD1L	0,952059	9,69E-07
TUG1	0,952032	3,31E-07
GPATCH4	0,951372	1,65E-05
MED21	0,951282	0,00051
BOLA3	0,950928	8,93E-05
PTPN2	0,947897	5,39E-06
TOMM6	0,947566	1,71E-06
XPOT	0,94685	0,000207
RNF167	0,946067	1,23E-05
GNPNAT1	0,9451	1,07E-05
FBXO45	0,944722	0,00026
PPIL3	0,944236	0,000641
AP4B1	0,942685	7,52E-05
DHX34	0,942408	2,41E-06
ARMCX6	0,941122	0,000261
AFG3L2	0,940707	6,55E-08
NDUFB10	0,938981	1,31E-06
DLGAP5	0,938831	5,01E-07
FAM207A	0,938054	1,33E-05
MRPL12	0,937528	1,25E-07
NSMCE2	0,936885	0,000242
SARS2	0,934582	6,18E-06
GCDH	0,933717	1,55E-05
PIAS4	0,931389	0,000784
PPP2R5C	0,930797	1,35E-06
C19orf53	0,930456	2,96E-06
OPLAH	0,928913	0,000878
BYSL	0,927787	0,000492
AIFM1	0,927617	9,16E-05

RBM38	0,927068	3,92E-06
PPP2R5E	0,926382	1,03E-07
SNHG6	0,926032	0,000632
DECR1	0,924851	2,80E-05
RPS23	0,923043	0,000221
SAP130	0,922851	8,39E-08
RANBP9	0,922437	3,11E-05
RPL39	0,922132	0,000718
UBTD2	0,921821	1,52E-05
AFTPH	0,920979	2,49E-05
HEATR5A	0,919727	0,000663
PIAS2	0,919623	5,96E-06
RPL18	0,919296	6,06E-07
CYC1	0,918549	5,60E-07
NDUFS6	0,918306	8,61E-06
AP4M1	0,916485	0,000403
RPS2	0,916294	1,44E-09
DNAJC1	0,916145	0,000133
CDKN3	0,915	0,000137
ULK1	0,914843	1,61E-05
TMEM179E	0,913146	7,94E-05
ALG5	0,912499	5,58E-05
GLTSCR1L	0,912239	0,000294
PKMYT1	0,909268	0,00065
WDR89	0,909033	0,000297
GCC1	0,908116	1,61E-05
CLOCK	0,907612	9,97E-05
NKIRAS2	0,905177	0,000467
CMC2	0,904735	0,000359
ELMOD2	0,903991	0,000221
DSTYK	0,903785	5,98E-05
PEMT	0,901762	5,37E-05
ATP5C1	0,900746	6,33E-07
LOC93622	0,899741	0,00055
PPIL1	0,898993	7,90E-06
EEF1B2	0,89844	0,000634
MRPL30	0,898103	1,63E-06
C14orf166	0,897832	1,63E-07
RXRA	0,897734	0,000796
STAG2	0,897455	1,75E-07
AP3B1	0,896771	1,43E-05
PCCB	0,896506	3,52E-07
UQCRQ	0,896274	1,00E-06
LRCH4	0,896034	6,10E-05
NCBP2-AS2	0,895771	0,000325
ABT1	0,895259	9,66E-05
SNAPIN	0,893717	0,000169
TUBA1A	0,892694	1,38E-05
HSPA9	0,892388	0,000521
UQCRFS1	0,891828	6,06E-07

PPP1R15B	0,891465	0,000776
C1orf122	0,890776	0,000697
KIF5B	0,890355	5,32E-08
NDUFAF6	0,890305	0,000329
INTS2	0,889553	5,42E-05
MAPK9	0,888888	9,04E-06
C9orf64	0,888131	0,000892
NDUFS2	0,887117	0,000644
MMAB	0,887079	3,26E-06
UBE2M	0,885781	7,70E-07
ATP13A1	0,885679	6,24E-06
EXOC3	0,885558	0,000786
PSMA4	0,883992	0,000205
ALG13	0,883042	0,000169
WDR20	0,882607	6,19E-05
NDUFS3	0,881621	1,54E-05
NDUFC1	0,881501	0,000296
MARCH5	0,880498	6,14E-06
MPDU1	0,879814	0,00019
C12orf45	0,87939	0,000538
FRA10AC1	0,879198	0,000432
F11R	0,877721	1,41E-06
ZNF410	0,876063	3,08E-05
MRPL27	0,872855	3,07E-05
H2AFX	0,871722	8,27E-07
DUSP16	0,871463	2,57E-05
RPL26L1	0,87058	6,84E-05
MRPL22	0,869973	0,0006
ENY2	0,869643	0,00062
CHCHD10	0,868608	7,36E-05
UQCR10	0,867812	0,00055
PRCC	0,867502	8,36E-07
ZDHHC4	0,86709	1,86E-05
ZMYND11	0,865697	1,08E-05
KIAA0101	0,864137	0,000849
SRA1	0,85992	0,000611
MAF1	0,859768	0,000407
NAA38	0,857663	0,000177
TCF25	0,85661	3,11E-06
MIDN	0,856145	7,56E-05
CNPY3	0,855934	0,000266
GNAS	0,853685	5,11E-07
RMDN1	0,851954	0,000157
MRPS5	0,850613	2,13E-05
WDR55	0,848943	4,32E-05
ago-02	0,847712	0,000146
GALK1	0,847193	0,000212
UBQLN4	0,846532	1,10E-05
PDHA1	0,846452	0,000444
CERS2	0,846097	2,16E-07

NLN	0,845431	2,30E-06
KAT2A	0,845236	4,32E-06
SMIM7	0,84497	2,07E-05
ZBTB41	0,84411	0,000293
PSMG3	0,843437	0,00055
PIAS3	0,841313	1,07E-05
ANKRD50	0,840027	9,03E-06
RIOK2	0,838104	0,00017
CABIN1	0,835746	0,000707
FAM127C	0,830789	0,000613
PHAX	0,829539	5,31E-06
IREB2	0,828987	3,59E-06
SLC2A4RG	0,827206	0,000131
PPP4R3B	0,826459	2,19E-06
ZNF282	0,825801	4,38E-05
CLCC1	0,824719	0,000314
WRNIP1	0,824074	0,000805
LARS	0,823546	1,50E-05
NBPF15	0,823295	0,000349
SLC25A15	0,823039	0,000521
MICU2	0,822185	0,000168
SUV420H1	0,821922	5,87E-05
ZNF346	0,820794	0,000779
ZFYVE21	0,820688	0,00037
MRPS25	0,820328	4,50E-05
PAK4	0,819508	0,000182
MED13L	0,819435	0,000854
DDX39A	0,818673	1,00E-05
HTATSF1	0,817669	0,000184
PRPSAP1	0,816291	0,000813
RBM17	0,815868	0,000277
ZNF579	0,812396	0,000941
SNRPF	0,811122	3,00E-05
PELP1	0,810163	0,000529
CCDC85B	0,808882	4,08E-05
SMG9	0,807472	0,000145
TSEN34	0,805847	0,000949
ZC3H13	0,805105	0,000392
RPS3	0,804709	0,000707
ZNF561	0,804374	0,00039
DBF4	0,804178	0,00042
ACAA2	0,803519	1,39E-05
SLC35B1	0,801761	0,00025
MLX	0,801745	0,000101
TMEM167/	0,8007	0,000266
INADL	0,800048	0,000356
CAMLG	0,79974	0,000123
GNA13	0,799353	9,65E-06
MFSD3	0,798074	0,000581
ISG20L2	0,797752	3,05E-05

CFDP1	0,797463	0,000162
PPP1R16A	0,797411	0,00048
NCBP2	0,797139	2,47E-06
NCAPG2	0,79651	0,000779
GPS2	0,796508	7,07E-06
ADRBK1	0,796386	0,000707
SEC16A	0,795311	2,45E-05
GCSH	0,793836	0,000292
POLR2D	0,793419	0,000901
PSMC4	0,792568	3,56E-06
KCMF1	0,792513	0,001003
S100A10	0,792237	0,000375
SCD	0,790182	9,38E-07
SLC25A38	0,788391	5,90E-05
MDK	0,788125	3,80E-05
EIF2D	0,788031	0,000496
KARS	0,787161	4,62E-05
CLPX	0,786442	0,000395
PRR14	0,786329	7,57E-05
SCARB2	0,78531	0,000685
YIPF3	0,783879	3,73E-05
PSMC2	0,782672	1,72E-05
EMD	0,77629	0,000269
ZBTB10	0,775086	0,000805
LSM4	0,772964	2,13E-05
CYBA	0,772602	0,00079
LIG3	0,771732	0,000237
ATP5F1	0,771702	9,47E-05
WDR33	0,770247	2,81E-05
KCTD20	0,768679	1,62E-05
NR3C1	0,768368	0,000642
WDR18	0,768121	0,000261
POLR2K	0,766978	0,00051
MRPL4	0,76613	6,70E-05
USMG5	0,765556	0,000938
CHD7	0,764999	0,000599
CARM1	0,762975	6,95E-05
C17orf62	0,761661	0,000194
GNL1	0,759741	0,000211
MIF	0,758661	0,000156
TOP1	0,756221	1,64E-06
DNPEP	0,755761	4,80E-05
ANAPC16	0,75383	7,86E-05
MRPL11	0,753084	5,03E-05
PIH1D1	0,753016	0,000999
DAP3	0,752051	0,000121
COX4I1	0,749034	2,38E-06
MRPL35	0,746881	0,000205
SECISBP2	0,744878	0,000379
RDH11	0,74477	0,000632

BDH1	0,74446	0,000569
ARMT1	0,743979	0,000272
STUB1	0,743073	3,49E-05
DDX6	0,740716	2,42E-05
AAGAB	0,739966	7,47E-05
EAF1	0,739161	9,68E-05
CCNK	0,738665	0,000793
CANX	0,73841	5,89E-05
HMBS	0,737524	0,000299
PEX10	0,737099	0,000705
ZNF629	0,736814	5,14E-05
MTMR3	0,735175	0,000291
RPS19BP1	0,734597	0,000423
THG1L	0,734419	0,000721
TUBGCP2	0,733836	0,000144
RAD50	0,732791	3,42E-05
MRPL14	0,731681	0,000216
GID8	0,730198	3,17E-05
REXO4	0,729255	0,000233
STRBP	0,728722	0,000718
COX20	0,728153	0,000844
RPL21	0,727909	0,000961
GLTSCR2	0,725947	0,000151
C12orf10	0,718932	0,00022
RAD23B	0,718866	3,36E-05
RPL8	0,716547	3,07E-06
C1QBP	0,716265	0,000229
GNB2	0,715645	0,000384
MECP2	0,708642	0,000294
WDR61	0,708374	0,000404
NDUFA6	0,708034	0,000189
DCAF7	0,70594	0,000709
MRPL2	0,702599	0,000383
PAGR1	0,701715	0,00016
EIF2S1	0,700894	0,000375
NDUFS8	0,699105	0,000277
NACC1	0,697166	0,000434
CAMSAP1	0,689931	0,000108
BUD31	0,689595	4,60E-05
SNRPC	0,688921	0,000206
KMT2D	0,686033	0,00013
PAIP1	0,679327	0,000397
C7orf73	0,678996	0,000309
MORF4L1	0,677513	0,000124
HACD3	0,677368	0,000144
EIF2AK1	0,677217	2,94E-05
CSRNP2	0,676407	0,000985
NUP88	0,671652	0,00052
RPL4	0,670807	0,00031
PPP2CA	0,668151	0,000122

EDF1	0,667675	7,70E-05
TADA3	0,666186	0,000442
CPNE3	0,664527	0,000955
PRDX4	0,66432	0,000414
RBM15B	0,662042	9,71E-05
PKN1	0,659731	0,00093
SHFM1	0,649253	0,000409
SIN3A	0,649004	0,000216
RPL19	0,647367	0,000394
ASH1L	0,644029	0,000702
RPL35	0,643317	0,000494
PSMD7	0,640761	0,00022
SART1	0,636228	0,000744
PLCG1	0,635228	0,000879
C20orf27	0,633264	0,000855
AP2S1	0,632779	0,000961
MRPS2	0,632761	0,00028
PSMA6	0,630599	0,000278
RPS24	0,629044	0,000193
GTF3C5	0,620107	0,000817
EIF1AX	0,619418	0,000254
MYO6	0,612282	0,000644
ERAL1	0,607314	0,00092
FBL	0,605049	0,000364
RPL7A	0,600538	0,000125
H2AFY	0,594807	0,000847
SERPINB6	0,593142	0,000917
GRB2	0,588154	0,000749
MARCKSL1	0,578235	0,000741
NBN	0,575164	0,000864
CSNK2B	0,562769	0,000993
HMGB1	0,542306	0,000616
DDX5	0,539688	0,001007
TUBA1B	-0,51889	0,000879
HGS	-0,58483	0,000707
EIF4H	-0,58846	0,000376
CDC20	-0,58916	0,000402
NASP	-0,58919	0,000855
NME4	-0,59184	0,00054
MAP4K4	-0,59888	0,000186
HNRNPD	-0,59959	0,000324
POLD2	-0,59984	0,000827
TPM4	-0,60694	0,000137
DHX30	-0,60948	0,000409
YKT6	-0,61127	0,000789
STAU1	-0,61483	0,000601
DNAJA2	-0,61528	0,000682
EIF6	-0,62372	0,000118
EPRS	-0,63586	0,000162
SPCS1	-0,64266	0,00073

SF3A1	-0,6446	0,000405
CTNNBL1	-0,64599	0,000464
TEX261	-0,6475	0,000707
NAA25	-0,65089	0,000747
SPATS2	-0,65299	0,000775
GNB1	-0,65826	5,45E-05
AP3D1	-0,65845	6,95E-05
POLR2C	-0,65964	0,000409
CAT	-0,6648	0,000923
VEZF1	-0,66483	0,000932
ANKRD28	-0,6657	0,000439
MGAT4B	-0,66614	0,00017
APMAP	-0,6713	9,21E-05
USP19	-0,67466	0,000529
DDX19A	-0,67468	0,000456
PTGES3	-0,67509	0,00026
NCL	-0,67574	1,29E-05
RANGAP1	-0,67577	8,97E-05
MED15	-0,67762	0,0005
DCTN1	-0,67851	0,000214
MFHAS1	-0,67933	0,000707
EBNA1BP2	-0,67948	6,89E-05
KTN1	-0,68231	0,000336
CD164	-0,68337	0,000389
BOP1	-0,68366	5,43E-05
AKIRIN1	-0,68382	0,000929
YBX3	-0,68516	0,000701
ATXN10	-0,68553	0,000191
TXNDC12	-0,68587	0,00061
DNAJC14	-0,68639	0,000887
CNPY2	-0,68925	0,000274
TMEM164	-0,69985	0,00088
IARS2	-0,7024	4,36E-05
JOSD1	-0,70266	0,000404
RANBP3	-0,70445	0,000163
BCL2L13	-0,70522	0,000835
HN1L	-0,70601	1,92E-05
G3BP1	-0,70635	1,47E-05
UBXN6	-0,7079	0,000186
ATG3	-0,708	0,000273
KDSR	-0,7104	0,000541
LAPTM4A	-0,71123	0,000108
MFN2	-0,71249	9,92E-05
ITPA	-0,71571	0,000609
UBE2J1	-0,71714	0,000163
PANX1	-0,71726	0,000426
GART	-0,71796	4,68E-05
MLH1	-0,72313	0,000211
XRN2	-0,72485	3,65E-05
KIF21A	-0,72504	0,000279

SLC20A2	-0,72735	0,00073
MKL1	-0,73091	0,000512
ATP2C1	-0,73321	8,25E-05
PTPN12	-0,73329	3,91E-05
DSN1	-0,73385	0,000705
DDX52	-0,73396	0,000786
FBXO21	-0,73505	0,000442
GOPC	-0,74182	0,000743
RAC1	-0,74212	3,18E-06
PCGF5	-0,74244	0,00027
NPC1	-0,74248	0,000561
MAP3K7	-0,74255	0,000247
ECT2	-0,74352	2,01E-05
VAV2	-0,74566	0,000482
HERC2	-0,74612	4,75E-05
CDC42	-0,74736	0,000103
C2CD3	-0,74813	0,000281
GSPT1	-0,74872	6,72E-05
KPNA1	-0,7489	0,000846
RER1	-0,74933	0,000136
USP16	-0,75049	0,000722
RNF185	-0,75376	0,000951
GFM1	-0,7541	5,63E-05
RPS6KA3	-0,75428	0,000208
COX15	-0,7551	6,70E-05
COPS7A	-0,75589	0,000261
MPZL1	-0,75733	2,45E-05
SLC35B4	-0,75753	0,000271
VPS35	-0,75915	5,33E-06
VPS36	-0,75938	0,000279
CRTAP	-0,76013	2,26E-06
PPP1CC	-0,76163	0,000317
ASXL1	-0,76305	3,78E-05
NPRL3	-0,76391	1,68E-05
ATF2	-0,7679	0,000241
U2SURP	-0,77089	5,93E-06
IMPAD1	-0,77161	2,49E-05
CEP41	-0,77221	0,000313
TWSG1	-0,77232	0,000258
USP33	-0,77563	0,000289
HUWE1	-0,77631	1,91E-06
CMIP	-0,77741	0,000407
SMARCC1	-0,7782	1,58E-06
CCT8	-0,77882	2,03E-06
RUNX1	-0,77911	0,000189
TFDP1	-0,77957	9,61E-06
ELP6	-0,77999	0,000275
DIS3	-0,782	0,000138
CFLAR	-0,78273	0,000101
DENR	-0,78317	9,78E-06

COASY	-0,78507	1,67E-05
NONO	-0,78522	0,000206
SLC33A1	-0,78523	0,00084
PPP1R9B	-0,78798	5,51E-06
CD2AP	-0,78811	8,50E-05
DNM1L	-0,7893	4,90E-06
PDPR	-0,79051	0,000121
RAD18	-0,79104	0,000108
CYFIP1	-0,79349	8,08E-06
PDZD8	-0,79358	1,66E-05
IFRD2	-0,79398	4,79E-06
RPS26	-0,79406	0,000979
ZMYM4	-0,79426	1,05E-05
GXYLT1	-0,79536	0,00048
EIF3M	-0,79592	1,61E-05
SMC1A	-0,79698	0,000208
HMGXB4	-0,79773	0,000137
ZFAND5	-0,79844	9,10E-05
FAM192A	-0,79861	4,93E-05
CNP	-0,79862	0,000648
C11orf84	-0,79997	0,000166
COL18A1	-0,80002	1,13E-05
FPGS	-0,8019	0,000301
QRICH1	-0,80208	7,64E-05
TAF5L	-0,80259	0,000246
SH3GLB1	-0,80298	0,000521
ARHGAP21	-0,80327	0,000235
NCAPD2	-0,80361	8,43E-05
CLDN12	-0,80402	0,000293
NUS1	-0,80581	0,000496
AHDC1	-0,80651	0,000721
GOLGA4	-0,80662	0,000269
OSBPL10	-0,80671	3,76E-05
RABEP1	-0,80795	0,000282
STT3A	-0,80886	0,000426
ARFGEF2	-0,80897	8,78E-06
RFC1	-0,80953	3,66E-05
UBE2E3	-0,80957	0,00027
SLC12A7	-0,81062	0,000101
CLSPN	-0,8112	3,18E-05
MAGI1	-0,81132	0,000348
TMEM230	-0,81147	0,000204
ALKBH3	-0,81151	0,0008
EIF4G3	-0,8129	6,37E-06
CIAPIN1	-0,81504	2,11E-05
ZYG11B	-0,8151	0,000449
UBAC2	-0,81522	0,000346
RHOBTB3	-0,81709	5,55E-05
TTLL4	-0,81817	0,000719
BAIAP2L1	-0,81818	6,39E-05

C22orf29	-0,8201	0,000256
NPC2	-0,82109	0,000143
ZFP91	-0,82151	4,92E-05
LRRC42	-0,82232	0,000187
FAH	-0,82248	0,000172
SUN2	-0,826	0,000162
TUSC2	-0,82682	0,000854
AGPAT5	-0,82689	1,70E-05
TERF2	-0,82821	0,000443
IFT57	-0,82853	4,98E-05
MCPH1	-0,82866	0,000838
DESI2	-0,82915	0,000247
GLRX3	-0,8302	7,40E-06
OSBP	-0,83042	5,58E-05
UBQLN2	-0,83104	6,20E-05
TRIM25	-0,83175	0,000447
OGFR	-0,83209	0,000773
REV3L	-0,83289	0,000117
KHSRP	-0,83292	1,97E-07
GORASP1	-0,83396	0,000363
TRMT6	-0,83533	7,51E-05
NFKBIA	-0,83561	0,000412
PPP3CA	-0,83575	0,000146
PSMB2	-0,83639	1,15E-06
ANKRD40	-0,83911	0,000768
PUM1	-0,83949	8,61E-06
CAPRIN1	-0,83989	7,56E-08
DMTN	-0,84043	0,000695
KIAA1468	-0,84304	0,000258
GNPAT	-0,84358	3,44E-05
NSL1	-0,84419	0,000385
DR1	-0,84446	3,54E-05
DYNC1I2	-0,845	4,47E-05
FEN1	-0,84552	1,28E-06
AGPAT3	-0,84581	0,000222
ATP6V1H	-0,84723	0,000189
YIPF6	-0,84845	9,27E-05
FAM208A	-0,84916	0,000187
FGD6	-0,84932	0,000567
TSG101	-0,8497	0,000375
DCTD	-0,84981	6,82E-06
TXNDC11	-0,85012	6,02E-05
PARP2	-0,85107	8,36E-05
SPAG9	-0,85112	0,000785
SUZ12	-0,85116	1,87E-06
LAMP1	-0,85129	0,000143
STAT2	-0,85129	0,000853
LAP3	-0,85254	0,000392
RGS10	-0,85402	0,000327
SNX3	-0,8546	0,00041

ADD1	-0,85486	9,51E-05
COPRS	-0,85776	4,01E-05
GLUD1	-0,85828	3,58E-07
PSMA2	-0,85877	8,44E-07
RNF26	-0,8593	2,40E-05
KANK2	-0,85978	8,69E-05
DCPS	-0,86001	0,000134
MYO5B	-0,86028	0,000323
DDA1	-0,86063	0,000187
TMEM41A	-0,86169	5,43E-05
OXSR1	-0,86226	0,000133
PFN2	-0,86344	6,31E-07
VPS41	-0,86351	0,000722
FGFR1OP2	-0,86354	0,000359
MAP1LC3B	-0,86405	3,49E-05
TPRA1	-0,86809	1,97E-05
UBE2J2	-0,86865	5,39E-06
ASAP2	-0,87025	8,58E-06
NCKAP1	-0,87111	6,37E-07
LPGAT1	-0,8713	0,000317
SMARCA2	-0,87147	0,000582
LTBR	-0,87221	1,24E-07
PPTC7	-0,87223	0,000285
ACD	-0,87276	0,000116
CDK2	-0,87333	3,18E-05
TMED7	-0,87481	7,63E-05
SHROOM3	-0,87482	7,26E-06
TUBGCP3	-0,87519	0,00025
RALGAPA2	-0,87538	0,000582
SH3PXD2B	-0,87579	0,000334
NUP37	-0,87689	0,000128
TMEM201	-0,87694	0,000482
UACA	-0,87867	0,000735
SRC	-0,88019	7,20E-06
RNF216	-0,88026	0,000127
PICALM	-0,88142	2,59E-06
SERTAD2	-0,88227	2,73E-05
AP2M1	-0,88248	0,000492
SMURF2	-0,88262	8,91E-05
ST13	-0,88318	4,20E-06
PDHB	-0,88357	0,000722
PLCG2	-0,88475	0,000965
GALNT1	-0,88597	5,52E-07
WNK1	-0,88677	3,20E-08
TANC1	-0,88792	1,77E-06
ALG9	-0,88823	9,68E-05
MANF	-0,89141	1,20E-06
SAAL1	-0,89154	1,10E-05
PEBP1	-0,89249	1,06E-08
FBXO30	-0,893	0,000825

NUTF2	-0,89346	4,36E-05
TSPO	-0,89349	1,06E-06
PTK2	-0,89623	1,69E-07
ATRN	-0,89642	8,86E-05
CCDC34	-0,89818	0,000305
KCTD9	-0,89833	0,000131
MDN1	-0,89837	6,20E-07
EGLN1	-0,89932	4,27E-05
ENO1	-0,89966	0,000551
SMAD4	-0,90131	6,03E-06
NOL4L	-0,90135	0,000306
DDX19B	-0,9014	1,90E-05
RHOT1	-0,90235	2,34E-05
VPRBP	-0,90277	2,42E-06
CCDC93	-0,90305	0,000623
PTPN9	-0,90402	0,000108
TMEM184I	-0,90412	1,62E-05
BROX	-0,90474	1,46E-05
CWC15	-0,90612	8,33E-05
NAGPA	-0,90621	0,000503
TBC1D22A	-0,90627	0,00049
TRIM32	-0,9063	0,000105
LAMC1	-0,90632	1,17E-06
ESCO2	-0,9073	0,000774
EXOSC7	-0,90905	3,43E-05
ELP4	-0,90976	0,000638
HIP1R	-0,91073	7,47E-06
FANCD2	-0,9131	0,000417
LRRC14	-0,91405	0,000268
CEP164	-0,91436	8,42E-06
TMEM109	-0,91609	1,43E-06
UFL1	-0,91636	0,00074
USP31	-0,91825	0,000129
NLE1	-0,92055	0,000465
FBXL5	-0,92166	4,42E-05
RAP1GDS1	-0,92207	1,16E-05
NIN	-0,92436	6,57E-05
HAUS6	-0,92453	2,86E-05
ARID4B	-0,92741	0,000828
GNA11	-0,92907	1,34E-06
DHX32	-0,92965	0,000327
WASL	-0,92966	1,04E-06
DUSP14	-0,9297	1,96E-05
GANAB	-0,93087	1,47E-09
NCLN	-0,9316	2,52E-08
TAB2	-0,93263	1,79E-07
SSRP1	-0,93348	2,64E-07
TOPBP1	-0,93584	1,09E-06
MINA	-0,93673	1,71E-05
DENND4C	-0,94341	3,63E-05

URB1	-0,94405	2,82E-06
ATP5G1	-0,94483	1,87E-05
ZDHHC7	-0,94488	7,42E-06
MCU	-0,94566	0,000231
SUCLA2	-0,94597	5,60E-05
TNKS	-0,94608	3,39E-06
BAG4	-0,94755	3,32E-05
SMARCD1	-0,94784	8,69E-08
EIF3D	-0,94837	2,53E-06
GTF2H3	-0,94892	0,000146
SBNO2	-0,94933	2,27E-05
ENTPD6	-0,94937	1,68E-07
DNTTIP1	-0,94941	9,25E-05
OSBPL9	-0,95008	1,32E-07
SLC20A1	-0,95091	6,18E-08
ASXL2	-0,95098	8,99E-05
RIMKLB	-0,95124	3,97E-05
GLB1	-0,95126	0,000109
C11orf58	-0,9519	8,48E-09
MUL1	-0,9534	2,82E-05
VASP	-0,95381	3,03E-07
ADGRG1	-0,95399	2,62E-07
ITPR3	-0,95567	6,01E-05
BNIP2	-0,95656	8,92E-05
KIAA1524	-0,95718	4,64E-05
USP47	-0,95846	4,61E-06
TMEM261	-0,95857	9,73E-06
INF2	-0,95971	2,70E-05
FAM168A	-0,96101	0,000162
PTER	-0,96154	0,000195
ATG16L1	-0,96201	2,47E-05
PDHX	-0,96403	0,000171
SH3GL1	-0,96418	4,03E-05
RAB34	-0,96461	0,000371
ATP8B1	-0,96486	7,57E-07
DYNC1H1	-0,96541	4,92E-07
CMSS1	-0,96542	3,20E-05
DEXI	-0,96732	0,000614
RNH1	-0,96752	0,000604
RPUSD4	-0,96764	0,000288
TMF1	-0,96955	0,000494
RALA	-0,97152	5,15E-08
PTPN3	-0,97204	1,15E-05
ALDH1B1	-0,9728	7,88E-08
P4HA1	-0,97293	2,64E-06
UBA6	-0,9741	2,90E-07
IBTK	-0,97432	5,42E-07
TNPO1	-0,97434	2,87E-08
PNP	-0,97509	1,30E-06
CHAF1A	-0,97609	1,60E-05

PDCD6IP	-0,97711	2,28E-09
TBC1D5	-0,97715	7,24E-07
STT3B	-0,97793	1,16E-05
NCOR2	-0,97805	9,39E-07
MIA3	-0,97837	9,19E-07
KDM5C	-0,97948	2,20E-08
HPS3	-0,98013	1,01E-05
SNN	-0,98021	2,94E-05
DNAJC10	-0,98024	5,34E-06
APLP2	-0,98066	2,50E-10
RETSAT	-0,98125	5,78E-07
PLEKHM2	-0,98188	1,48E-07
USP15	-0,98285	4,99E-07
HBS1L	-0,98312	2,10E-07
PLCB3	-0,98374	1,83E-05
LRRFIP2	-0,98519	3,16E-06
PITPNB	-0,9871	1,66E-08
LRP6	-0,98804	8,10E-06
UTP20	-0,98854	7,88E-06
PCNP	-0,98859	8,76E-07
CLDND1	-0,98908	0,000177
ITCH	-0,98974	4,15E-08
ZGPAT	-0,99099	2,15E-05
IRF2	-0,9917	0,00027
ZDHHC8	-0,99268	0,000203
DYNLT1	-0,99281	2,21E-06
PKN2	-0,99319	0,000183
GOLIM4	-0,99413	8,13E-08
LMAN1	-0,99458	1,68E-05
PKM	-0,99461	8,98E-07
XPO6	-0,99545	5,34E-10
PXMP2	-0,99558	0,000437
ZNF343	-0,99638	0,000192
XRRA1	-0,99784	0,000978
PHF19	-0,99791	3,43E-06
GPKOW	-0,9993	0,000293
GNPDA1	-0,99952	0,000381
DDHD2	-1,0016	2,50E-06
TBC1D23	-1,00183	0,000421
C11orf57	-1,00189	9,90E-06
BTBD3	-1,00201	4,51E-05
EP400	-1,00274	6,19E-09
PROSC	-1,00345	1,11E-05
ANKS6	-1,00371	8,11E-07
DIS3L2	-1,0042	3,03E-06
TFDP2	-1,00434	1,08E-06
STK24	-1,0067	9,76E-10
DIRC2	-1,00727	0,000722
HMOX2	-1,00781	0,000251
CNOT6L	-1,00863	1,65E-05

DCP1B	-1,00888	0,000575
PIK3R4	-1,0118	2,09E-06
C6orf132	-1,01236	8,85E-07
ADCY3	-1,01343	5,76E-08
SNRK	-1,01481	1,52E-05
SNTB1	-1,01554	2,30E-08
SPOP	-1,01566	3,20E-05
NUDC	-1,01589	0,00046
RNF111	-1,01631	6,05E-07
TTC7B	-1,01676	8,96E-05
RRM1	-1,01826	2,36E-06
PRPS1	-1,01843	0,000133
EIF5A2	-1,01888	0,00011
IFT172	-1,0194	0,000224
NOL6	-1,01992	4,35E-05
NUP93	-1,02029	4,10E-09
CRLS1	-1,02122	6,89E-06
SH3BP5L	-1,02136	1,61E-06
PRDX3	-1,02348	4,82E-07
C11orf31	-1,02372	3,22E-07
KIAA0319L	-1,02373	0,000981
CIRH1A	-1,02412	0,000222
TBC1D2B	-1,02485	9,75E-06
TOMM20	-1,02539	6,35E-10
TBCEL	-1,02575	0,000354
POLA2	-1,02601	0,000626
LIMD1	-1,02633	6,89E-05
EPS8	-1,0266	2,34E-07
MT1X	-1,02748	0,000158
NAP1L4	-1,02839	7,21E-10
SMTN	-1,03053	1,31E-07
KITLG	-1,03105	1,01E-06
MED14	-1,03135	1,09E-07
RYK	-1,03168	1,07E-05
COMMD7	-1,03261	3,81E-06
FRMD8	-1,03454	6,38E-05
TNFRSF21	-1,03703	8,70E-08
PDP2	-1,03761	0,000107
IMPA1	-1,03829	0,000928
BRD7	-1,03864	5,27E-08
CD46	-1,0398	1,22E-06
STK17B	-1,04044	0,000692
APEH	-1,04067	2,75E-06
METTL3	-1,04106	6,31E-06
ABL2	-1,04128	1,41E-08
TLDC1	-1,04161	0,000617
NCOA4	-1,04169	1,19E-06
FAM200B	-1,042	0,000704
POLE	-1,04315	4,21E-07
NUDCD3	-1,04328	3,64E-08

PTP4A2	-1,04331	3,97E-08
MGAT5	-1,04374	1,17E-05
SC5D	-1,0442	0,00074
MED8	-1,04438	4,72E-05
HIATL1	-1,04441	1,42E-06
CENPW	-1,04568	0,000293
PCED1A	-1,04588	0,000537
MAP3K4	-1,04706	6,19E-06
ZNF330	-1,04803	8,69E-08
NFU1	-1,04944	4,47E-05
WDR4	-1,04998	1,23E-05
PACS1	-1,0502	3,92E-08
MEN1	-1,05046	9,99E-08
ELOVL5	-1,05209	1,23E-09
EPG5	-1,05248	5,91E-05
CNKSR3	-1,05364	0,000632
COPS7B	-1,05385	5,62E-06
PTPN23	-1,05477	3,87E-09
RRP9	-1,05497	1,31E-07
ACTN4	-1,05612	3,14E-05
FOCAD	-1,05866	6,01E-08
BOK	-1,05931	2,09E-05
PHRF1	-1,05983	3,42E-10
BIN3	-1,05987	0,000851
USP13	-1,06038	4,09E-08
PANK4	-1,0613	7,31E-05
SLC25A26	-1,06259	0,000281
PNPO	-1,06259	3,14E-08
MAGT1	-1,06262	1,70E-08
CLEC16A	-1,06338	2,27E-07
OTUD4	-1,06483	4,44E-05
PRKAA1	-1,06667	0,000198
WWC1	-1,06843	0,000161
FGD1	-1,06875	2,89E-07
MSANTD4	-1,06944	0,000629
DLG1	-1,0725	9,40E-08
ARID1B	-1,07373	1,25E-06
POLR3G	-1,07384	0,000948
FXR1	-1,07426	2,91E-06
NLRX1	-1,07685	2,65E-06
RBL2	-1,07696	1,04E-07
HSDL2	-1,07716	0,00014
MGRN1	-1,07852	3,62E-09
CTDSP2	-1,07931	3,44E-05
FTSJ1	-1,07942	2,87E-08
SNX30	-1,07981	4,68E-05
ARL14EP	-1,08015	6,29E-05
CMTM6	-1,0809	7,83E-08
ABRACL	-1,08109	2,42E-06
ETNK1	-1,08173	1,69E-06

SUN1	-1,08331	7,25E-09
C21orf2	-1,08357	0,000217
UXS1	-1,08681	1,79E-08
FNIP1	-1,08716	7,89E-07
FGFRL1	-1,08738	4,29E-10
SPRED2	-1,08757	8,37E-07
GADD45A	-1,08781	0,000104
ITSN2	-1,08784	9,59E-05
KPNA3	-1,08813	1,17E-05
MIR4435-2	-1,08858	0,000973
SPTAN1	-1,08964	1,05E-08
ATXN2	-1,08993	5,02E-08
KPNA6	-1,09135	1,44E-08
STK25	-1,09148	2,25E-09
FAM49B	-1,09158	2,30E-09
SVIL	-1,09163	6,08E-05
RAB3GAP2	-1,09295	6,59E-08
NOCT	-1,0939	8,20E-05
PTGFRN	-1,09491	5,28E-09
NAE1	-1,0951	1,52E-09
CLP1	-1,09923	0,000178
BET1L	-1,09984	4,12E-06
TALDO1	-1,10004	3,86E-07
USP24	-1,10016	1,65E-07
POLR2L	-1,10223	4,58E-07
VPS26B	-1,10303	1,19E-05
SLC6A6	-1,10356	4,51E-10
VAC14	-1,10415	3,67E-10
CSNK2A1	-1,10454	2,95E-10
GSTO1	-1,1048	1,19E-05
KNTC1	-1,10505	0,000837
SLBP	-1,10595	3,42E-07
HIBADH	-1,10673	2,94E-08
FLII	-1,10721	3,96E-11
GTPBP1	-1,10759	4,38E-07
COMMD9	-1,10763	0,000209
TRAF3	-1,10856	1,74E-07
B3GALNT2	-1,10932	2,29E-06
MELK	-1,1097	4,54E-09
ST7	-1,11012	2,68E-05
DRAP1	-1,11069	9,65E-09
PDIA5	-1,11129	1,33E-06
FER	-1,1114	2,52E-05
SMS	-1,11249	5,18E-06
HTATIP2	-1,11274	0,000225
PARP4	-1,11303	5,58E-11
DOCK7	-1,1131	1,70E-08
TMEM219	-1,11371	2,05E-05
CNOT1	-1,11427	1,69E-11
XPC	-1,11463	3,23E-08

EXOC6B	-1,11479	0,000183
DCUN1D5	-1,11532	8,09E-08
WASF2	-1,11664	1,78E-11
RRAGC	-1,11667	1,89E-05
MBOAT2	-1,11683	1,57E-05
USP9X	-1,11717	1,68E-06
BCL9L	-1,118	2,29E-05
TRAM1	-1,11961	0,00024
CDC25A	-1,11998	2,72E-06
STARD4	-1,12081	8,80E-06
PRELID2	-1,12093	0,000617
LDLR	-1,12132	4,12E-09
ZBTB44	-1,12256	3,27E-05
SIK3	-1,12296	2,93E-08
SNX33	-1,12319	1,87E-06
MSRB1	-1,12409	4,99E-05
COPS8	-1,12494	4,11E-07
TMEM104	-1,12568	0,000341
CLTA	-1,12589	4,82E-09
ATOX1	-1,12628	0,000668
TMEM41B	-1,12634	1,82E-05
SH3KBP1	-1,12651	0,000941
TARBP1	-1,1301	0,000414
TMEM50B	-1,13052	0,000456
ENKD1	-1,13085	0,000375
LRRC16A	-1,13096	0,000659
NUP133	-1,13128	4,84E-09
PJA1	-1,13291	0,000402
PGM2	-1,13483	1,09E-07
TMED4	-1,13642	1,04E-09
RNF19A	-1,13692	2,79E-05
TMEM245	-1,13958	1,95E-08
CHFR	-1,14023	1,54E-06
OSBPL11	-1,14028	3,13E-05
TM7SF3	-1,14072	1,18E-05
THYN1	-1,14074	1,24E-05
RIC8A	-1,14112	5,86E-12
SMC4	-1,14163	1,56E-08
ANXA5	-1,14443	2,25E-13
ERI1	-1,1446	1,49E-07
DCP1A	-1,14478	0,000367
SBDS	-1,14561	2,61E-09
TTC17	-1,14585	5,62E-10
ADIPOR2	-1,14665	7,83E-11
PAFAH1B2	-1,14675	1,46E-09
USP53	-1,14699	2,55E-06
PDLIM7	-1,14969	3,52E-07
RABGEF1	-1,1509	2,33E-08
ACAD10	-1,15215	3,79E-07
AAK1	-1,15346	0,00017

PLEKHA6	-1,15443	8,67E-08
CLCN7	-1,15669	5,96E-09
SLC41A1	-1,15963	9,11E-07
LRRC40	-1,16053	7,95E-06
TGOLN2	-1,16069	1,82E-08
AGRN	-1,16096	2,25E-06
NCSTN	-1,16101	7,46E-11
ATP1B3	-1,1611	1,28E-12
ATP11A	-1,16164	2,55E-05
DNAJC5	-1,16176	5,08E-06
MKLN1	-1,16223	2,12E-10
ESYT1	-1,1625	1,49E-13
C4orf46	-1,16276	3,80E-05
NECAP2	-1,16288	1,48E-08
ATR	-1,16292	4,44E-09
TRAF3IP2	-1,16359	1,88E-05
CEP57	-1,16643	7,19E-05
TCTN2	-1,16655	1,49E-06
DZIP3	-1,16688	1,26E-07
RB1	-1,16693	4,75E-08
GNPTAB	-1,16877	2,05E-09
SHQ1	-1,16912	4,47E-06
SP140L	-1,16958	2,06E-06
HEATR3	-1,17036	5,29E-05
SLC9A1	-1,17084	5,34E-10
TAPBP	-1,17177	0,000468
NFKBIE	-1,17226	2,09E-05
MAP3K1	-1,17227	3,01E-08
QDPR	-1,17361	1,16E-07
SKI	-1,17514	5,08E-11
EFNB2	-1,1774	0,000221
SLC2A1	-1,1793	0,000588
MYL6	-1,17935	1,31E-14
ARHGAP17	-1,1797	6,45E-11
B4GALT4	-1,17971	2,63E-07
TPD52L2	-1,18039	1,63E-11
AP1M1	-1,18068	3,49E-05
PHF13	-1,18111	5,34E-07
CHAF1B	-1,18155	1,89E-06
RANBP6	-1,1819	0,000135
LATS2	-1,18347	2,64E-07
TYW3	-1,18465	1,74E-08
LTBP3	-1,18487	1,77E-08
TAB3	-1,18717	4,01E-05
ROCK2	-1,18762	1,69E-10
ATP11B	-1,18768	1,18E-09
PGAM1	-1,18817	8,34E-06
TMED5	-1,18853	2,98E-06
CASP8	-1,19039	3,73E-07
CCDC109B	-1,19058	0,000109

NISCH	-1,19092	1,03E-08
ANO10	-1,19129	0,000917
ARHGEF26	-1,19217	6,28E-06
SLC15A4	-1,19242	6,54E-07
FAM198B	-1,19287	5,06E-06
NDFIP2	-1,19408	5,71E-07
IQGAP1	-1,19422	2,03E-09
ERLIN2	-1,19692	1,27E-10
HCCS	-1,19705	6,97E-06
PKI55	-1,19706	0,000865
POT1	-1,19786	7,36E-07
FAM73A	-1,20016	0,000799
CIT	-1,20059	0,000353
FEZ2	-1,20071	1,93E-08
SEPN1	-1,20267	4,43E-11
ZDHHC5	-1,20282	3,15E-12
NUP43	-1,2044	7,17E-06
KIAA1671	-1,20501	1,35E-08
ABHD12	-1,20546	5,07E-10
RFT1	-1,20835	4,45E-10
PRKACB	-1,20906	3,66E-06
STRAP	-1,20935	3,48E-09
PSTPIP2	-1,20991	3,73E-07
CHEK1	-1,21166	5,76E-11
CAB39	-1,21415	8,17E-11
ZC3H7B	-1,21417	9,04E-11
MROH1	-1,21579	1,62E-07
PRKD3	-1,21583	1,20E-08
NNT	-1,21592	1,30E-07
RNPEPL1	-1,21655	6,06E-06
RFC5	-1,21767	2,07E-07
MIB2	-1,2186	5,57E-05
SLC35F2	-1,21887	4,45E-05
WDR82	-1,22249	8,14E-12
POLK	-1,22292	1,38E-06
MLEC	-1,22315	7,59E-07
NOL9	-1,2235	1,12E-06
KIF1B	-1,224	1,47E-05
PPP2CB	-1,22452	9,28E-10
MYO1B	-1,22471	7,71E-14
C11orf1	-1,2249	0,000772
RECQL	-1,22506	5,84E-10
BICD2	-1,22594	8,66E-11
NSMAF	-1,22615	2,49E-09
HMGA1	-1,22659	2,77E-09
DNAJC16	-1,22718	3,25E-06
GLT8D1	-1,22788	2,17E-08
PLSCR1	-1,22945	4,05E-07
USP4	-1,23056	8,89E-10
PRKDC	-1,2312	4,68E-09

ANKRD13C	-1,23239	2,38E-06
TMEM115	-1,23295	0,000202
CC2D1B	-1,23303	5,18E-09
PIP4K2B	-1,23534	2,60E-10
DIEXF	-1,23586	0,000108
ADARB1	-1,23672	3,57E-07
HPCAL1	-1,23697	1,08E-09
LTN1	-1,23923	0,000102
PRKCD	-1,23938	0,000399
CKAP2L	-1,24236	0,000472
CAST	-1,24467	1,28E-09
LMO7	-1,247	6,84E-07
CD63	-1,24731	1,03E-05
HSP90B1	-1,24734	5,00E-11
NUP98	-1,24925	7,71E-15
DAPK3	-1,24961	1,08E-10
RAD54L2	-1,25089	4,85E-05
PTPRF	-1,25258	1,85E-15
ZMYM6	-1,25453	1,60E-05
SMC5	-1,25583	1,52E-09
TTC28	-1,25647	9,65E-05
EMC1	-1,258	1,31E-12
KIF18A	-1,2589	2,98E-06
COL4A3BP	-1,25906	3,48E-08
PPME1	-1,26017	2,12E-07
PGS1	-1,26099	1,76E-05
BRI3	-1,26168	7,48E-11
PHC1	-1,26226	1,16E-07
PQLC1	-1,26229	1,56E-07
LRRCC1	-1,26255	1,42E-06
AASDHPPT	-1,26263	1,40E-07
HIAT1	-1,26272	9,08E-10
RHBDF2	-1,26514	2,20E-09
CSNK2A2	-1,266	1,17E-11
RPS6KA1	-1,26616	6,22E-05
HERPUD2	-1,26641	2,47E-09
TRPC4AP	-1,26656	1,78E-08
ABHD5	-1,26835	2,14E-08
DPP9	-1,26871	6,46E-09
TANGO6	-1,26973	1,22E-05
MLXIP	-1,27125	2,16E-14
AHNAK2	-1,27151	6,57E-06
SLX4	-1,27364	8,10E-08
RGS3	-1,27376	9,78E-07
DEAF1	-1,27395	9,28E-08
NRSN2	-1,27396	5,70E-05
CCT6A	-1,28022	8,61E-15
NSFL1C	-1,28074	6,21E-11
RBL1	-1,28131	4,25E-05
SF3A3	-1,2819	9,26E-06

CC2D2A	-1,28221	8,38E-07
CLIP1	-1,28282	5,68E-13
CERS5	-1,28354	1,66E-09
FMNL1	-1,28357	7,16E-05
FZD7	-1,28379	3,57E-06
CD81	-1,284	4,57E-15
UBA5	-1,28558	1,25E-08
PTPRA	-1,28717	5,00E-07
PPAP2C	-1,28787	2,24E-09
ZW10	-1,28822	2,56E-09
VAMP3	-1,28855	0,00024
PSMD13	-1,28929	5,86E-15
ARL16	-1,28985	2,91E-06
TLN2	-1,29256	0,000376
RTCA	-1,29295	3,29E-09
SSH2	-1,29338	5,13E-06
IPO5	-1,29378	1,78E-16
PEX14	-1,2963	1,98E-07
GTF2H1	-1,29784	2,21E-11
PPARA	-1,2983	0,000322
PSD3	-1,29875	1,79E-08
CEP290	-1,2992	2,64E-08
USP54	-1,30213	4,36E-12
ELF4	-1,30396	1,38E-05
M6PR	-1,30631	2,15E-07
RBM19	-1,30641	7,23E-05
CEP162	-1,30674	5,28E-05
OPN3	-1,3092	3,12E-07
CES2	-1,30948	3,54E-09
PLOD1	-1,30972	7,54E-09
ALS2	-1,31021	1,40E-09
TMEM50A	-1,31091	3,79E-09
MLLT3	-1,31094	1,71E-06
ARHGEF18	-1,31095	5,83E-11
METTL21B	-1,31167	4,50E-07
CHST14	-1,31206	8,15E-06
NRGN	-1,31209	0,000301
SLC41A2	-1,31211	9,52E-05
ARL13B	-1,31238	8,54E-09
NPTXR	-1,31279	1,88E-09
IFT80	-1,3148	0,000222
STAG3L2	-1,31496	9,66E-05
STRADB	-1,31528	8,21E-10
NUDT16	-1,31657	4,28E-06
CTDSPL	-1,31797	8,07E-12
BBC3	-1,31823	1,75E-05
TACC1	-1,3183	3,47E-11
TJP1	-1,3183	1,67E-06
VPS54	-1,31901	1,01E-06
BFAR	-1,31999	1,65E-14

HDAC7	-1,32112	5,96E-13
PCSK7	-1,32479	0,00029
IPO7	-1,32598	2,71E-14
KIAA0930	-1,32708	3,87E-06
ANXA11	-1,3277	3,16E-10
CTBS	-1,32998	3,20E-05
TBC1D20	-1,33081	4,20E-10
SLC4A7	-1,33098	1,12E-07
NDUFAF3	-1,33234	1,19E-11
DFFA	-1,33238	3,92E-13
TTL	-1,33299	1,57E-09
CAP2	-1,33526	1,39E-10
SLC25A37	-1,33632	5,87E-06
BTN2A2	-1,33974	2,45E-05
RPS6KA4	-1,34144	7,98E-12
AMPD2	-1,34206	4,44E-07
CCBL1	-1,34225	0,000664
KRT80	-1,34238	2,83E-07
EPS15	-1,34249	2,34E-10
STAT3	-1,34379	0,000173
QSER1	-1,3442	2,58E-14
PANK2	-1,3445	1,32E-08
CNST	-1,34586	0,000194
CHID1	-1,34648	9,21E-09
NBAS	-1,34672	9,31E-11
MAPKBP1	-1,34869	0,000606
HEATR1	-1,3499	1,19E-10
PHYH	-1,35011	2,78E-05
DONSON	-1,35026	2,59E-07
PTPN13	-1,35082	9,13E-08
ZNF512	-1,35229	1,43E-07
C2orf74	-1,35234	0,000302
SACM1L	-1,35345	1,54E-09
RNF213	-1,35463	6,21E-05
ABCB7	-1,35563	2,38E-06
PFAS	-1,35766	1,59E-16
LIN7C	-1,35775	5,38E-06
PAQR7	-1,35848	1,48E-06
TMEM248	-1,3593	2,39E-12
PRCP	-1,36169	2,16E-09
ARFGEF3	-1,36322	3,71E-05
GLYR1	-1,36335	1,81E-15
BAZ2A	-1,36373	1,24E-16
FCHSD2	-1,36381	6,00E-06
BCKDHB	-1,36623	0,000149
TUBB6	-1,36672	1,87E-09
WBP11	-1,36686	5,07E-16
TBCE	-1,36742	1,71E-08
SRI	-1,36812	4,96E-14
PGM3	-1,36831	1,34E-06

PIP4K2A	-1,37116	2,98E-14
TBL1XR1	-1,37137	7,73E-09
DUSP3	-1,37217	6,85E-12
FLYWCH1	-1,37279	1,16E-05
FAM214B	-1,37341	2,99E-05
SNTB2	-1,3747	4,88E-08
ICMT	-1,37509	3,92E-17
NGLY1	-1,37557	8,37E-09
PPP1R18	-1,37613	3,37E-09
DCLK1	-1,3767	1,25E-10
COLGALT1	-1,37792	1,31E-15
MYCBP2	-1,37934	8,91E-11
BAG2	-1,37995	5,47E-09
HSPB11	-1,38037	1,08E-05
PARP8	-1,38077	1,93E-05
SLC16A1	-1,38151	7,65E-12
PIK3C3	-1,38329	3,32E-07
ABCC1	-1,38345	1,14E-14
WDR35	-1,38392	1,93E-06
TBRG1	-1,38696	1,01E-11
PLXNB2	-1,38769	1,45E-06
CD99L2	-1,38829	1,15E-06
CDV3	-1,38876	1,95E-09
PLS1	-1,38935	7,44E-08
GALNT14	-1,38952	1,99E-10
NACC2	-1,3904	2,05E-10
CYSTM1	-1,39042	9,21E-11
FUT11	-1,39098	0,000292
EFCAB14	-1,39131	4,81E-15
CNDP2	-1,39148	6,41E-13
GIT2	-1,39633	2,78E-14
ABCD3	-1,39803	3,40E-11
BAP1	-1,39817	2,98E-14
ACTR8	-1,39829	5,89E-11
PPP2R5B	-1,39923	1,00E-04
METTL15	-1,39948	2,11E-08
GPX8	-1,40071	1,40E-08
ACER3	-1,40159	2,36E-11
TJP2	-1,40283	2,61E-05
RASAL2	-1,40293	4,59E-09
KLHL15	-1,40393	0,000471
DPYSL2	-1,40413	1,00E-05
CTSO	-1,40474	0,000774
ANKH	-1,40485	1,23E-07
NCS1	-1,40712	6,88E-16
ZNF445	-1,40725	6,75E-06
FNDC3A	-1,40875	1,64E-12
DIXDC1	-1,40895	3,43E-05
HMHA1	-1,41218	3,31E-07
SUSD6	-1,41301	5,57E-06

ANKLE2	-1,41636	1,02E-11
MLLT4	-1,41748	1,63E-07
NPAS2	-1,41866	0,000122
PTAR1	-1,4187	3,03E-09
ERO1A	-1,41884	1,65E-12
MARVELD1	-1,41949	2,48E-11
LONP2	-1,41978	6,14E-13
PLS3	-1,41991	1,22E-11
PDGFA	-1,42016	4,46E-09
AIDA	-1,42151	5,40E-07
GANC	-1,42178	0,000298
ANXA6	-1,42227	5,78E-05
CREBL2	-1,42335	1,43E-05
ARFGAP1	-1,42376	4,24E-15
FKBP11	-1,42385	1,33E-07
C20orf194	-1,42687	7,52E-05
PPP2R3A	-1,42711	1,27E-06
RAB9A	-1,42823	1,32E-08
SPIN4	-1,42857	0,000529
PLXND1	-1,42882	5,35E-06
TP53BP2	-1,43088	1,08E-08
EIF4A2	-1,43373	5,11E-05
ENTPD7	-1,43418	3,19E-06
DDX17	-1,43472	2,65E-19
VEGFB	-1,435	1,40E-06
CDC42EP2	-1,43585	1,25E-07
KIAA1147	-1,43709	8,06E-09
EFNA5	-1,4371	6,00E-08
GNL3	-1,4377	3,26E-07
RHPN2	-1,43829	1,06E-10
RHOA	-1,44009	9,45E-17
UAP1L1	-1,44271	4,58E-08
AJUBA	-1,44398	0,000951
ORAI2	-1,44505	6,46E-08
TNFRSF1A	-1,44519	1,05E-08
IGF2R	-1,44575	8,72E-10
CHCHD7	-1,44594	1,31E-05
CPNE7	-1,44791	4,48E-09
CDC42BPA	-1,44826	1,90E-07
COPB1	-1,44832	4,18E-17
FKBP1A	-1,44901	5,34E-11
PTPN21	-1,44911	1,73E-07
ARHGAP1	-1,44987	7,59E-08
PCBP4	-1,44992	7,04E-10
MECOM	-1,45078	6,25E-10
NUDT21	-1,45287	9,39E-16
CNEP1R1	-1,45409	1,43E-05
SEPT2	-1,45546	1,78E-16
CXorf38	-1,45547	1,23E-07
CAMKK1	-1,45664	7,50E-05

RABL3	-1,45681	2,23E-09
LPCAT3	-1,457	1,32E-07
UHRF1	-1,45856	9,15E-08
APPL1	-1,45872	1,35E-16
IP6K1	-1,45885	3,02E-15
HMGCL	-1,46079	2,15E-09
GHDC	-1,46104	2,11E-05
SACS	-1,46186	2,74E-15
SGTB	-1,46652	6,93E-05
TPP2	-1,46753	2,72E-12
CRY1	-1,46846	6,05E-11
SDC3	-1,47002	3,63E-09
TTC33	-1,47059	8,80E-05
NRBP2	-1,47235	2,52E-06
YEATS2	-1,47299	0,00017
P4HA2	-1,47313	3,93E-06
BACE1	-1,47583	8,22E-08
DGKD	-1,47633	2,59E-09
TUBA1C	-1,4783	1,24E-17
SH3D19	-1,47952	2,19E-13
DESI1	-1,48044	4,35E-16
ZC3H12A	-1,48109	0,000103
RHOBTB2	-1,48154	2,86E-06
DNAJC3	-1,48566	2,71E-06
RAB11FIP1	-1,48598	5,12E-15
PSMA1	-1,486	8,21E-17
ITFG1	-1,48647	1,95E-07
PDLIM5	-1,48656	1,65E-07
MRE11A	-1,48761	1,32E-12
CBL	-1,48787	8,84E-12
ZNF395	-1,48825	9,49E-15
KATNB1	-1,48848	1,41E-10
FAM111A	-1,48992	1,41E-11
TERF1	-1,49036	7,71E-11
DDX10	-1,49179	1,78E-16
NFIX	-1,49225	2,69E-05
GBAS	-1,49339	0,00042
PTPN11	-1,49719	3,07E-20
NEK7	-1,49812	7,30E-11
WEE1	-1,49908	5,61E-05
PLEKHM1	-1,50315	5,49E-06
ARMC9	-1,50433	4,87E-10
FAM43A	-1,50942	8,88E-08
RRM2B	-1,51271	4,66E-05
KIAA1143	-1,51386	1,10E-08
TIAM2	-1,51456	0,000578
ZFP36L1	-1,52036	1,45E-09
LOC64516[illegible]	-1,52373	0,000347
AP2A2	-1,52382	8,16E-15
PIK3R1	-1,52492	7,71E-11

MYO9B	-1,52548	1,16E-07
SEC22C	-1,52548	3,55E-12
LUZP1	-1,52695	1,17E-05
LCOR	-1,52771	5,09E-13
TRIO	-1,52889	1,67E-13
SLC36A4	-1,53035	7,18E-05
PDE12	-1,5322	2,31E-15
MYOF	-1,53272	8,35E-05
C11orf54	-1,53278	0,000111
GALE	-1,53308	4,84E-06
KLHL7	-1,53536	8,62E-08
ATP13A3	-1,5372	3,60E-21
UROD	-1,53728	2,95E-11
TBC1D25	-1,53832	0,000153
COL5A2	-1,53855	9,88E-05
FAM109A	-1,54175	2,06E-08
DBNL	-1,54252	2,19E-07
CRYZL1	-1,54303	1,92E-06
XPNPEP1	-1,5435	2,20E-10
TRPM2	-1,54427	6,12E-08
SNX8	-1,5449	1,67E-09
MAP4	-1,54539	2,37E-23
MOCS1	-1,54634	0,000143
TMEM263	-1,54671	1,71E-10
PRMT3	-1,54685	3,49E-09
NETO2	-1,54873	3,80E-20
ANKRD18B	-1,5489	2,44E-06
PNPLA2	-1,5504	2,55E-08
LPCAT1	-1,5518	5,49E-14
LARS2	-1,55383	8,11E-14
SLFN5	-1,55485	1,52E-08
SH3BGRL3	-1,55501	3,93E-05
PI4K2A	-1,55657	1,14E-09
GPNMB	-1,55711	1,68E-07
ATM	-1,55785	6,27E-12
C4orf32	-1,55801	6,88E-05
DDX47	-1,55833	1,17E-18
RSPRY1	-1,55923	1,44E-16
RAB29	-1,56106	2,41E-08
SHPRH	-1,56112	1,01E-06
MCTP1	-1,56165	8,55E-09
CYB5R3	-1,56208	1,01E-09
SOD2	-1,56328	8,78E-15
TFB1M	-1,5641	2,68E-05
PHKB	-1,56416	7,35E-06
GINS4	-1,56433	9,64E-15
CD83	-1,56466	5,82E-05
XRN1	-1,5673	4,52E-06
SEMA4B	-1,56878	8,39E-14
ARFGEF1	-1,57137	3,56E-14

APH1B	-1,57206	0,000167
ACTN1	-1,57233	2,10E-10
ATRNL1	-1,57365	0,000855
GINS3	-1,57424	7,38E-06
EHBP1L1	-1,57904	6,43E-05
API5	-1,58011	1,01E-16
MOK	-1,58053	1,04E-07
PAQR3	-1,58315	3,50E-09
HGSNAT	-1,58496	6,40E-06
TMEM30A	-1,58563	3,76E-07
AP2B1	-1,58591	2,31E-18
NUAK1	-1,58728	7,92E-12
NPAT	-1,58841	3,92E-10
UAP1	-1,58876	6,26E-09
FAM118B	-1,58981	3,26E-09
KLHL18	-1,59128	1,27E-11
PACSIN2	-1,59543	9,52E-14
GNA12	-1,59563	2,46E-18
RCBTB1	-1,59577	1,99E-10
TNFRSF12A	-1,59641	0,000166
FAR1	-1,59716	2,09E-15
IER3	-1,59826	4,92E-13
SNX21	-1,59903	3,82E-08
GBE1	-1,60104	5,85E-09
BHLHE40	-1,60142	2,07E-13
NEIL2	-1,60354	1,17E-11
PLCXD1	-1,60604	5,48E-05
FNTA	-1,60627	1,39E-14
DOK4	-1,60675	9,86E-15
HEXB	-1,60679	3,90E-13
TRAM2	-1,60756	5,70E-20
HIGD1A	-1,60774	2,88E-13
EIF2AK4	-1,60822	3,78E-17
LFNG	-1,61161	1,64E-07
ABHD10	-1,61282	1,29E-10
MBNL2	-1,61302	6,16E-13
SMARCD3	-1,61303	4,44E-07
HIF1A	-1,61535	2,37E-20
PIK3C2A	-1,61587	9,63E-23
SLC2A8	-1,6178	1,26E-07
POMZP3	-1,61861	1,77E-08
CPPED1	-1,62	1,36E-14
FHL3	-1,62069	2,71E-11
OXCT1	-1,6232	6,94E-13
MAP4K2	-1,6252	8,97E-09
NOTCH2	-1,62553	2,01E-21
DTL	-1,63106	7,85E-19
ALKBH8	-1,63143	1,41E-05
FOXP1	-1,63382	2,62E-16
PLEKHA1	-1,63484	1,07E-10

LMNB2	-1,63608	1,86E-08
USP28	-1,63623	2,82E-12
MSANTD3	-1,6366	4,74E-10
OAT	-1,63709	7,27E-05
SORL1	-1,63742	7,82E-10
MIER1	-1,6378	4,41E-16
ITSN1	-1,6381	4,38E-15
FGGY	-1,63916	0,000127
CAPN10	-1,64075	6,56E-06
MAP2	-1,64123	1,14E-05
ALAS1	-1,64165	1,29E-10
STK4	-1,64208	5,51E-19
RTN4	-1,64212	2,43E-17
IL13RA1	-1,64416	4,06E-10
STXBP5	-1,64615	8,54E-11
PXN	-1,64963	6,94E-10
SOCS6	-1,64987	1,79E-11
SEC23A	-1,65093	2,62E-13
FURIN	-1,65274	6,82E-17
ZDHHC14	-1,65326	7,93E-06
NTN4	-1,65439	0,00062
NFKB2	-1,65442	9,85E-12
SEPT7	-1,65458	4,13E-21
RBCK1	-1,65605	3,05E-24
CAP1	-1,65654	8,17E-14
TYMS	-1,65772	4,15E-21
VKORC1L1	-1,65772	2,26E-22
ZDBF2	-1,65815	0,000411
SNX19	-1,65854	9,30E-20
ARHGEF12	-1,65943	6,12E-16
TNS1	-1,66159	1,91E-05
FAM216A	-1,66204	1,99E-07
KANK1	-1,6652	3,47E-10
TRAK1	-1,6654	1,82E-14
EDEM2	-1,66995	1,37E-15
PTDSS2	-1,67427	6,19E-06
SCMH1	-1,67492	0,000906
FGF11	-1,67651	4,42E-05
KLHL2	-1,6766	5,24E-08
LPIN2	-1,67668	3,33E-10
SDCCAG8	-1,67822	7,37E-09
LGALS3	-1,684	6,46E-05
B2M	-1,68413	2,65E-07
LASP1	-1,68704	4,14E-10
LZTFL1	-1,68719	3,63E-09
FOXK1	-1,68881	2,86E-16
FIGNL1	-1,6894	5,56E-16
RPGRIP1L	-1,69126	4,76E-11
LINC01116	-1,69206	1,92E-07
PCNA	-1,69255	4,56E-26

ARIH2	-1,69336	7,30E-21
STXBP1	-1,69417	3,94E-13
LIMK1	-1,69575	3,57E-10
CEP250	-1,697	1,82E-09
ACSS1	-1,69738	1,99E-05
PVR	-1,69759	7,75E-06
SIPA1L3	-1,69774	2,94E-22
EHD1	-1,69796	4,01E-07
SGCB	-1,69878	6,59E-14
LOC10012S	-1,70025	2,57E-09
ANXA2	-1,70096	3,89E-28
MPP6	-1,70236	5,24E-15
SEC24D	-1,70371	0,000183
MYH10	-1,70448	2,31E-20
TOMM34	-1,71079	2,26E-21
FAM160A1	-1,7122	5,65E-06
TCF12	-1,71295	1,97E-09
ARF4	-1,71425	3,03E-07
MMD	-1,71497	0,000335
CDC14B	-1,71534	1,89E-09
SNX29	-1,71637	3,15E-06
OSBPL5	-1,71667	1,08E-10
FLNA	-1,7176	2,48E-21
HACE1	-1,7178	6,14E-06
CPD	-1,71946	7,92E-20
CTR9	-1,72013	2,26E-10
HPS5	-1,72023	3,80E-14
FHOD1	-1,72206	4,90E-20
TTYH3	-1,72429	1,36E-16
SPTBN1	-1,72458	6,57E-29
SYNJ1	-1,72565	1,01E-06
ARSK	-1,72727	4,30E-05
SNORA40	-1,72998	3,26E-05
PTPN1	-1,73087	1,17E-16
ARHGEF28	-1,73141	1,13E-14
DSG2	-1,73198	1,48E-25
ENTPD4	-1,73379	3,38E-16
MCM8	-1,73511	5,15E-17
SDC1	-1,73854	6,53E-17
PYGB	-1,73875	1,46E-27
DNAJC18	-1,7396	8,03E-05
TRIM68	-1,74071	4,35E-05
NT5DC3	-1,74157	1,78E-08
TMEM106E	-1,74317	1,29E-17
SLC45A3	-1,74323	3,85E-08
COL6A1	-1,74347	2,76E-17
ATAD5	-1,74368	4,73E-07
EXTL2	-1,74384	1,76E-06
KCTD7	-1,7439	1,28E-05
ABR	-1,74575	6,41E-19

NABP1	-1,74593	1,08E-10
DYNC2H1	-1,7512	2,72E-11
ZNFX1	-1,75127	8,31E-10
EFEMP1	-1,75219	2,75E-20
RELT	-1,75278	7,85E-09
C4orf19	-1,75488	7,74E-05
DNAJC13	-1,75515	5,09E-13
CNNM1	-1,75716	2,47E-06
C1orf106	-1,75886	6,08E-05
FERMT2	-1,75909	1,85E-17
ERC1	-1,75975	3,72E-23
NEDD4	-1,76047	5,93E-20
CDS2	-1,76406	2,94E-16
SERAC1	-1,76492	1,75E-06
ZNF827	-1,76762	2,78E-05
MICALL1	-1,76834	3,19E-19
SPG20	-1,76982	3,72E-19
MTMR2	-1,77048	1,17E-22
ASPHD1	-1,7718	8,32E-07
FZD6	-1,77381	3,44E-14
WNT5A	-1,77655	4,55E-08
ZC2HC1A	-1,7767	7,66E-05
HOXC6	-1,7772	0,000486
AP1S2	-1,77876	5,69E-11
CLSTN1	-1,77929	8,21E-18
UBTD1	-1,78224	9,07E-05
SRGAP1	-1,78246	3,02E-08
SPAG1	-1,78444	6,13E-05
TRIM44	-1,78576	7,10E-28
CDCA7	-1,78845	3,76E-05
PPP1R15A	-1,7886	8,51E-10
SLC39A8	-1,78967	1,04E-08
SNX25	-1,79254	1,16E-08
SLC39A14	-1,79578	5,64E-06
MANEA	-1,79938	2,04E-07
PLD1	-1,80064	6,38E-10
THSD4	-1,80191	6,20E-09
FYN	-1,80212	4,48E-05
TBC1D1	-1,804	3,55E-16
SLC4A3	-1,80529	5,70E-07
NCAPD3	-1,80721	5,09E-15
DLX1	-1,80879	2,49E-08
TMEM120[illegible]	-1,8088	4,65E-16
ILK	-1,80975	1,03E-14
FAF1	-1,81173	2,52E-07
RBFOX2	-1,81221	5,71E-25
RBM7	-1,81235	1,24E-09
AEBP2	-1,81411	1,53E-13
LITAF	-1,81437	1,08E-10
IFNAR2	-1,81572	1,04E-10

TIAM1	-1,81634	2,43E-07
LAMB2	-1,81743	9,97E-19
CRYZ	-1,81807	1,96E-08
CAPRIN2	-1,8185	1,40E-09
RCN1	-1,81955	1,18E-26
ITPR1	-1,82097	5,60E-09
POLQ	-1,82112	8,07E-09
STIM1	-1,82152	1,18E-22
TMCO3	-1,82227	8,69E-10
CCNYL1	-1,82499	5,04E-09
GNAI2	-1,82629	3,04E-10
CAMK1	-1,82717	1,57E-05
CDKN2B	-1,82794	1,18E-06
CARNMT1	-1,82939	1,17E-10
CKAP4	-1,83064	3,31E-30
RELL1	-1,83164	4,75E-08
ARL2BP	-1,83241	3,66E-20
DNAJB5	-1,83244	0,000851
STAT6	-1,83257	1,25E-16
VSIG10	-1,83342	5,93E-18
TSPAN14	-1,8361	2,36E-18
LRRK2	-1,83948	0,000421
LINC00641	-1,84032	2,15E-06
DIAPH3	-1,84037	7,90E-07
MCFD2	-1,84133	5,72E-27
ANXA2P2	-1,8416	6,40E-05
B3GLCT	-1,84184	2,64E-08
CD151	-1,84193	3,65E-29
C1GALT1	-1,84198	1,23E-17
ENC1	-1,84233	5,88E-11
TRMT11	-1,84375	0,000604
LOC103611	-1,8473	0,000913
SSH1	-1,84842	1,28E-19
PFKFB3	-1,8512	4,23E-07
DLX2	-1,85142	9,91E-08
HFE	-1,85144	0,000668
PIGK	-1,85199	2,21E-13
BTBD10	-1,85222	5,04E-18
SSR3	-1,85236	2,78E-20
RASA2	-1,85344	1,27E-07
PATL1	-1,85358	1,82E-25
PEX6	-1,85531	0,000404
NFAT5	-1,85647	3,34E-09
MORC4	-1,85853	7,63E-20
CLN5	-1,86054	1,14E-05
KLF3	-1,86063	6,54E-12
CHORDC1	-1,8688	9,21E-21
ZHX3	-1,87037	1,77E-06
LRRC8E	-1,87105	1,59E-11
PHC2	-1,8729	3,00E-14

PMEPA1	-1,87451	3,50E-16
EXT2	-1,87577	7,35E-12
C16orf70	-1,87601	1,81E-11
PSMD2	-1,87728	2,06E-21
EEA1	-1,8777	2,29E-18
TLN1	-1,87826	2,74E-22
LRRFIP1	-1,87837	1,85E-30
E2F7	-1,88328	4,90E-12
C12orf4	-1,88527	6,33E-14
SLC39A10	-1,88767	5,22E-18
TXLNA	-1,88855	2,82E-17
HCFC2	-1,88945	3,91E-06
CBFB	-1,89311	2,01E-26
FAM86DP	-1,89436	1,84E-11
INPP5B	-1,89454	1,52E-10
ANLN	-1,89739	1,81E-32
PLEKHA5	-1,89826	7,52E-18
ADGRE5	-1,89881	4,16E-07
ITM2B	-1,90102	1,32E-20
SLC25A22	-1,90207	7,99E-19
DDAH1	-1,90304	1,92E-07
ZNF185	-1,90576	1,41E-09
AGTPBP1	-1,90597	8,44E-18
CPS1	-1,90725	1,02E-11
MAML2	-1,90769	4,33E-05
QSOX1	-1,90881	6,10E-32
TWISTNB	-1,91065	7,07E-18
SGK1	-1,91127	1,98E-06
CALU	-1,91189	3,65E-24
MICAL1	-1,91377	5,74E-06
DDX58	-1,91561	0,000765
LINC01123	-1,91577	3,17E-09
SNAP25	-1,91618	2,78E-05
CASP3	-1,91829	4,46E-10
BMP1	-1,91848	1,07E-15
SUCLG2	-1,91974	1,86E-21
FAHD2B	-1,92017	0,000652
RNF141	-1,92093	2,15E-14
KLF6	-1,92163	9,21E-05
DIAPH2	-1,92212	2,27E-12
PON2	-1,92233	1,84E-09
TOLLIP	-1,92235	2,15E-08
TMEM123	-1,92373	1,03E-33
DNAJC24	-1,92386	7,11E-10
EXTL3	-1,92633	6,15E-25
RAB8B	-1,9276	1,46E-07
LDHA	-1,92885	8,18E-32
ACACA	-1,92889	8,95E-33
ME2	-1,93088	1,86E-21
FAM129B	-1,93094	2,04E-22

ZNF319	-1,93204	0,000222
LMF2	-1,93226	1,40E-15
SCARF2	-1,93339	1,49E-05
HTR7P1	-1,93469	0,000846
UBA3	-1,93507	5,08E-21
ZAK	-1,93662	8,96E-26
BACH1	-1,93794	4,02E-13
NMT2	-1,93799	1,91E-13
STX2	-1,94351	1,02E-15
PTPRK	-1,94397	4,71E-24
TAF9B	-1,94565	3,10E-09
SLC16A4	-1,94569	4,21E-05
MYO10	-1,94732	4,69E-23
LAMB1	-1,94783	1,21E-34
ZNF275	-1,95083	1,72E-20
TNIP1	-1,95281	6,26E-09
RNF170	-1,95398	4,34E-09
TMEM135	-1,95577	2,05E-11
CEP170	-1,95779	1,15E-25
BEND6	-1,95894	0,000627
DOCK9	-1,96415	8,45E-17
NFKB1	-1,96597	3,03E-10
WDR91	-1,97026	8,62E-18
PDDC1	-1,9732	8,68E-17
ZNF853	-1,97474	0,000429
ARFGAP3	-1,97539	7,99E-15
FGFR1	-1,9779	2,50E-25
SNX10	-1,97976	4,02E-08
EFR3A	-1,98012	6,13E-10
POFUT2	-1,98141	2,79E-11
SLC17A5	-1,98377	2,22E-07
GPR180	-1,9842	1,69E-16
LPP	-1,98614	7,04E-20
PXDC1	-1,98826	3,73E-12
NAB1	-1,99036	1,42E-11
CREB3L2	-1,99135	7,59E-15
C11orf68	-1,99315	6,76E-21
WSB2	-1,99408	1,63E-06
CARS2	-1,99943	9,53E-24
SPSB1	-2,00183	8,59E-05
EFNB1	-2,00547	4,70E-13
STX1A	-2,00592	2,33E-15
NR2F2	-2,00819	1,77E-21
ARAP1	-2,00877	2,56E-31
ASCC3	-2,00966	6,45E-11
ECHDC1	-2,00974	3,69E-13
SLC12A8	-2,01276	6,49E-12
CDK17	-2,01292	1,14E-18
ACAT1	-2,01359	9,33E-12
TEAD1	-2,01676	8,62E-34

SOAT1	-2,02124	5,45E-20
SLC2A10	-2,02477	1,05E-10
KCNQ5	-2,0267	0,000747
ASAP1	-2,02754	1,23E-33
BMP2K	-2,02758	1,73E-20
BBX	-2,02819	8,22E-20
KSR1	-2,02911	1,10E-05
APP	-2,03004	4,39E-39
VPS37B	-2,0319	1,51E-25
EVI5	-2,0344	3,59E-10
TNFRSF10B	-2,03735	6,91E-24
ABCB10	-2,04421	2,25E-15
LPIN1	-2,04668	6,31E-07
MFSD1	-2,04814	1,21E-11
FKBP7	-2,04894	3,09E-07
MACF1	-2,04947	3,60E-34
MAPK8IP3	-2,04987	0,000137
UBE2H	-2,05129	2,76E-10
NUPL2	-2,05251	4,77E-05
TAP2	-2,05327	2,83E-14
EZR	-2,05408	1,59E-17
FTO	-2,05592	4,30E-24
CCL2	-2,05704	9,45E-05
FBXL2	-2,05778	1,00E-06
FDXACB1	-2,06042	5,53E-07
HEBP1	-2,06107	9,83E-28
IFNGR2	-2,06188	7,87E-12
GLYCTK	-2,06787	3,47E-05
SCRN1	-2,06955	2,99E-36
ADGRG6	-2,07212	3,79E-18
ZBED5-AS1	-2,07447	3,72E-07
ZNF532	-2,07503	4,11E-17
RP2	-2,07646	5,99E-08
TIPARP	-2,08379	6,89E-17
KLHL21	-2,08401	6,88E-26
PRKAA2	-2,08443	1,76E-16
HACD1	-2,08543	3,35E-09
TRIM14	-2,09198	1,58E-07
ADAP2	-2,09327	1,64E-05
GFRA1	-2,09327	1,10E-11
PFKP	-2,09335	5,26E-17
FAT1	-2,09357	2,48E-12
DERA	-2,09411	3,64E-28
POFUT1	-2,09439	1,13E-22
EOGT	-2,0945	6,61E-09
SEPT9	-2,09493	5,00E-08
SLC2A6	-2,09623	0,000183
RBM24	-2,09794	9,62E-07
CEP85L	-2,09933	2,86E-05
FRMD4A	-2,10146	5,04E-10

MPRIP	-2,10269	2,29E-24
UTRN	-2,10272	1,96E-12
TPST1	-2,10949	5,22E-08
TWF2	-2,11103	2,03E-30
USP40	-2,11241	2,17E-24
SDK1	-2,1173	2,54E-11
EXOG	-2,11744	0,000189
SH3RF1	-2,11959	2,60E-08
ADCY7	-2,12259	9,40E-19
SMCO4	-2,12517	6,18E-05
FABP5	-2,12551	2,97E-33
CEP112	-2,12833	7,14E-05
VLDLR	-2,13017	0,000278
ITGA6	-2,13077	3,64E-13
ADAM8	-2,13305	0,000404
FAM219A	-2,13831	9,15E-08
LGALS1	-2,13961	5,59E-12
NFE2L3	-2,14049	1,35E-26
SHCBP1	-2,14207	2,07E-30
SH3TC1	-2,14337	1,45E-07
PPFIBP1	-2,14499	5,03E-28
SH2D4A	-2,14572	1,35E-23
DYRK3	-2,14868	2,75E-15
VPS13C	-2,14967	7,39E-15
HOXB7	-2,15055	1,39E-16
PPM1F	-2,1513	3,12E-09
CYTH3	-2,15277	2,08E-22
DOCK1	-2,1532	2,10E-19
S100A6	-2,15382	3,22E-11
MLPH	-2,15384	4,60E-29
TMEM55A	-2,15399	5,79E-13
LOC101927	-2,15456	0,000216
PINK1	-2,15752	2,91E-05
PPAP2B	-2,16241	1,05E-07
GPR63	-2,16785	0,000414
LHFPL2	-2,16856	5,73E-25
LRRC20	-2,17149	9,21E-11
GATA6	-2,17262	9,06E-10
RASSF3	-2,17304	9,43E-19
GLIS2	-2,18098	7,38E-29
BACE2	-2,18185	3,58E-19
MOCOS	-2,18331	8,45E-08
GLDC	-2,18873	3,15E-06
RIPK4	-2,18877	5,32E-20
STK17A	-2,19069	1,75E-26
CAMK2D	-2,19154	1,19E-15
C16orf45	-2,19296	6,93E-08
QKI	-2,19826	1,86E-15
FUCA1	-2,2	5,41E-15
IL17RD	-2,20359	1,22E-21

ZNF792	-2,20586	1,60E-08
DNAJC22	-2,20817	2,78E-16
C18orf54	-2,20943	3,93E-17
LOC389831	-2,20961	1,26E-07
WDFY1	-2,21068	9,14E-34
GAB2	-2,21316	8,41E-22
TPCN1	-2,21523	2,95E-29
SLC30A7	-2,21534	6,84E-18
FYCO1	-2,21544	4,42E-12
B4GALT6	-2,21624	1,11E-12
ST6GALNAC	-2,21801	2,40E-19
LOC90768	-2,21979	2,08E-08
MKNK2	-2,2221	2,77E-29
GMPR	-2,22222	0,000116
AP1S3	-2,22612	1,27E-13
AKIP1	-2,23128	2,08E-19
LIMS1	-2,23546	1,70E-45
FAM20C	-2,23834	4,09E-22
NMI	-2,23966	7,13E-08
CFL2	-2,24293	5,54E-24
OAS3	-2,24318	0,000717
TNFRSF11A	-2,24429	3,34E-11
CEP19	-2,24619	2,84E-07
SORBS3	-2,24752	5,29E-33
LAMA3	-2,2478	2,83E-17
SCCPDH	-2,25241	2,55E-19
STARD3NL	-2,25478	4,87E-13
LIMA1	-2,25483	1,74E-10
MAP3K8	-2,2589	0,000563
FAM46A	-2,26065	1,64E-06
SPATS2L	-2,26193	2,11E-27
GAS6	-2,26392	1,17E-15
FNDC3B	-2,2651	7,97E-37
EDA2R	-2,26569	3,07E-07
LOC100129	-2,2713	6,81E-05
ARHGAP18	-2,27192	3,50E-19
RASL10B	-2,27399	1,36E-08
STEAP3	-2,27522	2,83E-17
HIVEP2	-2,27815	2,50E-31
TMEM51	-2,27929	3,12E-24
IFI27L2	-2,2796	0,000962
FJX1	-2,27966	2,50E-06
INAFM2	-2,28105	2,26E-08
FADS1	-2,28879	1,20E-16
OCIAD2	-2,28986	2,19E-21
GSDMD	-2,29243	4,08E-06
TPP1	-2,29335	1,83E-22
A4GALT	-2,29548	0,000285
PCOLCE2	-2,29846	1,57E-10
TMEM8B	-2,29951	3,51E-07

SKAP2	-2,29996	4,32E-26
ZNF462	-2,30075	2,02E-16
PAPLN	-2,30181	8,90E-05
SHISA4	-2,30387	0,000126
EHHADH	-2,30507	3,40E-17
CLCN6	-2,30627	3,61E-06
DYNLT3	-2,31006	2,80E-07
EDEM1	-2,31382	5,49E-28
SSFA2	-2,31503	1,22E-40
LEPROT	-2,31515	5,91E-30
PMAIP1	-2,31528	5,46E-10
CDCA7L	-2,31597	1,07E-09
ERAP1	-2,32068	7,21E-26
ATP10D	-2,32236	2,98E-08
ABCC4	-2,32281	2,35E-32
FARP1	-2,3278	2,22E-31
FAM3C	-2,3281	9,59E-35
RGL1	-2,32821	3,81E-12
STOML1	-2,33224	2,38E-10
PARP12	-2,33349	5,00E-08
SLC8B1	-2,33534	4,00E-14
IFFO2	-2,33797	2,87E-20
PARVB	-2,33871	9,04E-30
SLC35E4	-2,33883	4,81E-13
SEC14L1	-2,33907	8,01E-30
DRAM1	-2,33926	8,22E-18
CHM	-2,34851	8,47E-08
APAF1	-2,35152	2,25E-08
PDLIM1	-2,35352	1,96E-46
LBR	-2,3564	1,26E-30
TMEM56	-2,35698	1,03E-11
FLNB	-2,35796	2,93E-21
CYFIP2	-2,36549	1,84E-09
BEX4	-2,37065	2,65E-12
ARHGAP26	-2,37272	8,38E-06
PAG1	-2,37479	1,98E-06
PELI1	-2,37499	1,75E-06
GLI3	-2,37718	5,22E-18
FAM65A	-2,37729	9,38E-33
STX3	-2,38035	5,34E-19
ARNT2	-2,38303	3,81E-26
MAPKAPK3	-2,3857	2,54E-23
DENND5A	-2,38743	2,33E-15
PTPRG	-2,389	4,21E-25
FAM107B	-2,39025	2,34E-09
MPDZ	-2,39327	7,22E-17
LCA5	-2,39365	1,10E-05
HDAC4	-2,40194	0,00024
PAM	-2,40797	1,48E-26
ZMYM6NB	-2,40957	3,52E-09

DTX3L	-2,41092	3,70E-13
GRB10	-2,41465	7,69E-05
ABHD2	-2,41517	4,11E-21
CSRNP1	-2,41861	1,42E-18
TMEM44	-2,41946	7,03E-14
CLIP2	-2,42757	1,06E-29
OPHN1	-2,42917	5,22E-19
PITPNM1	-2,43052	1,64E-18
P3H1	-2,43461	2,81E-29
CITED4	-2,43491	1,84E-14
RHOF	-2,43563	3,55E-40
TUBB2B	-2,43856	6,77E-10
KLF12	-2,44067	2,34E-14
FAM114A1	-2,44146	2,32E-08
SNX9	-2,44208	5,26E-34
SIK2	-2,44235	6,43E-29
SLC25A12	-2,44378	5,61E-24
SLCO3A1	-2,44753	4,75E-19
MAMLD1	-2,44855	1,42E-08
SGK223	-2,44917	2,07E-30
TIMP1	-2,4515	9,28E-06
NEK6	-2,45515	2,06E-38
HOXA5	-2,45641	7,93E-07
KDELC1	-2,45797	1,74E-18
KCND1	-2,46263	0,000528
BICD1	-2,4634	4,01E-24
SH3BP4	-2,46591	8,83E-32
FAM126A	-2,47	1,86E-17
HNMT	-2,4712	8,00E-05
LOC441666	-2,47132	1,82E-06
DCBLD1	-2,47356	1,70E-16
MMP16	-2,4755	2,33E-11
PITPNM2	-2,47696	2,09E-17
LOC654342	-2,47939	8,46E-12
ANXA2P3	-2,48041	4,04E-05
YAP1	-2,48089	1,83E-36
PLEKHG4	-2,48313	1,38E-14
ITPRIPL2	-2,4833	5,71E-33
TSPAN9	-2,48591	1,71E-09
GALNT7	-2,48658	5,78E-16
PEA15	-2,4875	2,62E-29
PLAGL1	-2,48925	5,07E-14
CENPV	-2,49332	6,76E-08
LACTB	-2,49523	2,03E-06
ASRGL1	-2,49538	9,34E-17
ZCCHC24	-2,49539	1,42E-07
STRIP2	-2,49766	1,64E-08
SDCBP	-2,4977	1,56E-16
CLTCL1	-2,49992	7,94E-12
CIB2	-2,50327	0,000644

USB1	-2,50777	1,01E-14
MAP7D1	-2,50849	4,80E-27
RNF145	-2,5092	5,86E-11
FMNL3	-2,5111	3,71E-35
AMOTL1	-2,51427	4,08E-41
ST3GAL3	-2,51703	4,71E-14
DEGS1	-2,51949	5,58E-19
ZNF488	-2,51965	0,000347
TBC1D8B	-2,52131	1,73E-05
SMPD1	-2,52306	2,19E-07
ZNF697	-2,52349	5,83E-19
SDC2	-2,5241	1,22E-38
CHD9	-2,52436	1,39E-27
TNIK	-2,52484	2,91E-16
ETHE1	-2,52757	6,89E-21
KIAA0754	-2,52817	2,04E-16
MATN2	-2,5284	2,70E-30
ACSL1	-2,53215	1,36E-36
NF2	-2,53293	1,45E-34
WWC2	-2,53653	8,82E-34
PARP3	-2,53845	1,14E-26
CNTLN	-2,54064	4,39E-14
NPTX1	-2,5422	0,000598
COL4A5	-2,54467	5,69E-09
EVA1B	-2,5459	2,06E-07
QPRT	-2,54599	2,46E-12
PRDM5	-2,5508	3,02E-05
KBTBD8	-2,55119	0,000141
PML	-2,55601	3,33E-10
SYNPO	-2,55607	6,34E-07
PLCB4	-2,55759	2,30E-24
SLC5A3	-2,55898	1,02E-39
FAM92A1	-2,55962	1,59E-06
MTMR10	-2,56216	7,01E-23
WWTR1	-2,56248	3,06E-48
CDKL1	-2,56412	2,71E-09
FAS	-2,5664	0,000277
TMOD1	-2,57046	1,25E-09
RFX2	-2,5705	1,58E-18
PRTG	-2,5722	6,46E-15
UGCG	-2,57305	2,22E-31
TMX3	-2,5745	1,95E-28
ETV6	-2,57704	1,62E-34
PTPRJ	-2,57848	9,65E-30
HHAT	-2,58136	1,92E-14
FOSL2	-2,58193	2,23E-27
ARHGEF25	-2,58235	6,07E-16
FKBP1B	-2,5847	0,000444
SGPP2	-2,58529	1,74E-09
ADAM10	-2,58571	1,11E-47

MYADM	-2,59019	1,39E-14
PLOD2	-2,59128	5,53E-50
LZTS3	-2,59736	2,75E-06
ARHGEF10	-2,59766	6,87E-13
BIRC2	-2,60725	2,15E-20
ABTB2	-2,60814	5,26E-34
ZMAT3	-2,60893	5,75E-07
RASA3	-2,61186	8,77E-21
RPS6KA6	-2,61362	6,92E-05
RIN3	-2,62209	4,63E-12
DLC1	-2,62486	3,32E-30
FAM229B	-2,62583	8,82E-06
SLC26A2	-2,62636	2,29E-50
DOCK4	-2,62946	2,90E-14
CORO1C	-2,62949	3,17E-60
RPS6KA2	-2,63035	4,66E-16
TREX1	-2,63185	1,41E-11
SEPT11	-2,6327	1,73E-62
RTTN	-2,6328	1,09E-14
GNPDA2	-2,63323	7,82E-12
STARD9	-2,63406	0,000593
SEPT4	-2,63822	9,32E-10
SERPINB8	-2,64695	3,87E-10
CUEDC1	-2,64703	1,77E-38
FAM135A	-2,64944	3,84E-14
PKD2	-2,6546	3,92E-32
RRAS2	-2,65536	2,61E-24
MET	-2,65836	1,23E-63
FAM110B	-2,6585	3,44E-18
SMAD3	-2,65913	8,51E-17
RAB31	-2,66333	1,14E-10
SPNS2	-2,66455	3,51E-11
ATAD3B	-2,66595	9,33E-13
NPHP3	-2,67121	0,000378
KCTD17	-2,67136	9,49E-13
LOC730102	-2,68071	0,000353
KDELC2	-2,68722	2,31E-31
FLT3LG	-2,68895	9,73E-05
NKX3-1	-2,69354	2,30E-12
DENND5B	-2,70378	2,95E-19
ITGA2	-2,70507	3,04E-39
CPOX	-2,70636	8,65E-15
VOPP1	-2,71132	2,46E-32
MICALCL	-2,71269	1,93E-05
CADM1	-2,71329	6,12E-44
FSTL3	-2,71461	1,46E-13
PRRG1	-2,71513	5,50E-07
CCDC50	-2,71542	2,26E-31
SGMS2	-2,71632	8,70E-12
FERMT1	-2,71726	1,10E-35

THRB	-2,72266	9,77E-11
FOXO3	-2,72787	1,59E-17
CCDC88B	-2,72819	4,03E-08
CPNE2	-2,73066	1,39E-13
PRAME	-2,73262	4,54E-09
BIVM	-2,73529	1,31E-09
ZYX	-2,73739	8,35E-35
APOL2	-2,73804	1,08E-05
ADGRL3	-2,7434	0,000216
DOCK5	-2,74484	5,95E-32
DPY19L2P2	-2,76021	0,000184
PPP2R2B	-2,76113	0,000547
HSD17B12	-2,76257	2,67E-59
HIVEP3	-2,76308	7,82E-05
LRRC6	-2,76312	9,79E-06
ANO6	-2,76633	9,34E-37
L3MBTL3	-2,767	2,51E-18
LRP12	-2,76865	3,91E-50
RBPMS	-2,7711	1,68E-51
RCAN1	-2,77157	7,15E-17
GALNT10	-2,77649	9,39E-53
PLLP	-2,77702	0,000944
ITPRIP	-2,77805	2,17E-09
REXO2	-2,77834	1,71E-21
EHD2	-2,7797	7,53E-38
GXYLT2	-2,78296	1,33E-10
PLEC	-2,78704	1,13E-11
TRIM5	-2,78745	1,35E-12
RDX	-2,79028	5,53E-37
MGAT5B	-2,79392	3,91E-12
OSMR	-2,79412	2,42E-15
CHIC1	-2,7959	1,67E-11
PIDD1	-2,79651	8,24E-42
HOPX	-2,79758	0,000283
THBS1	-2,805	2,49E-13
SAMHD1	-2,81228	7,65E-11
KIAA1462	-2,81964	3,54E-07
PRKCE	-2,82142	4,09E-08
ALCAM	-2,82162	2,51E-34
KIF17	-2,82217	4,08E-05
SWAP70	-2,82461	1,28E-39
GPRC5A	-2,83016	6,47E-06
KAT2B	-2,83113	7,61E-13
WDR90	-2,84212	1,49E-13
RASSF2	-2,84638	1,16E-13
SLC9A7	-2,84756	2,55E-13
RECK	-2,85058	1,73E-06
EPS8L2	-2,85481	2,08E-20
ARAP2	-2,85734	8,80E-27
PYGO1	-2,85791	4,68E-11

RELB	-2,85804	2,59E-13
CMTM1	-2,85875	0,00025
WNT7B	-2,8594	4,44E-17
TCF7	-2,86642	6,16E-25
BEND7	-2,87712	3,47E-20
BTC	-2,87753	9,02E-06
TMCC1-AS1	-2,87756	8,90E-09
CAMK1D	-2,87761	6,85E-28
PRPS2	-2,87816	7,17E-39
OGFRL1	-2,88095	7,98E-34
MRAS	-2,88233	5,85E-20
PDZD2	-2,88671	0,000456
CAPG	-2,8892	7,06E-53
ATP6V1B2	-2,88952	3,08E-64
PLK3	-2,89131	1,01E-14
GPR39	-2,8954	8,51E-10
SEMA7A	-2,89968	1,61E-33
SORBS1	-2,90155	1,41E-19
OTUB2	-2,90283	3,32E-08
SH3BGRL	-2,90421	1,45E-25
HOOK3	-2,90591	3,54E-37
SLC37A2	-2,90765	1,64E-05
SLC1A3	-2,90795	1,54E-14
SERPINA1	-2,90832	4,00E-17
NUAK2	-2,91078	1,32E-24
FAM189A2	-2,9136	0,000255
SIM2	-2,91913	6,85E-24
SLC39A13	-2,92324	1,29E-48
ALS2CL	-2,92358	2,40E-12
HOXA1	-2,92545	4,78E-07
HLA-C	-2,92664	6,14E-08
ATP2B4	-2,92963	1,86E-18
FADS3	-2,93229	1,70E-46
KLHL29	-2,93363	5,92E-13
CHST7	-2,93417	1,04E-10
NEDD4L	-2,93949	1,07E-32
CACNA2D1	-2,94395	5,51E-42
CYLD	-2,94396	7,86E-08
FKBP14	-2,94627	4,49E-35
KLHL5	-2,9492	5,29E-52
PQLC2L	-2,95174	3,76E-05
SPEG	-2,95264	7,99E-15
CAPN5	-2,95828	8,39E-09
HOXA11	-2,95869	0,0001
SLC23A2	-2,96304	1,57E-55
TNS3	-2,96638	1,21E-21
SPON2	-2,97499	1,55E-05
GPRC5B	-2,97873	9,00E-12
TMEM154	-2,98053	2,83E-13
MICB	-2,98467	3,54E-16

PHLDA2	-2,9856	3,63E-10
PGM5	-2,99117	4,20E-16
AHNAK	-2,99593	1,26E-36
QPCT	-2,99778	8,28E-12
CTSC	-3,00049	1,29E-56
MARCKS	-3,00366	8,03E-63
DHRS3	-3,00634	2,14E-13
KIF13B	-3,01299	1,95E-10
COCH	-3,01712	3,99E-13
TSPAN4	-3,01781	1,78E-19
SOWAHC	-3,01961	1,62E-18
STOM	-3,02347	1,12E-58
PHLDB1	-3,03358	8,01E-61
CCDC82	-3,03455	4,95E-12
ANKMY2	-3,03493	9,62E-37
KCNIP3	-3,03807	5,84E-36
ANKRD29	-3,04023	0,000198
GRK5	-3,04653	1,57E-11
LOC101927	-3,05005	0,000249
GDPD5	-3,05078	6,75E-07
GOLM1	-3,05082	2,14E-69
MYH9	-3,05085	6,05E-45
PBX3	-3,05472	2,49E-43
VCL	-3,05877	3,50E-22
PARP10	-3,06302	1,51E-15
TNFSF9	-3,06348	3,96E-06
IKBIP	-3,06886	4,13E-13
CASK	-3,07835	1,49E-68
PDP1	-3,07939	2,71E-54
MALT1	-3,08043	4,45E-67
GPAT3	-3,08125	1,50E-11
CCDC68	-3,08219	0,000132
MKLN1-AS	-3,08475	3,58E-05
MYO1E	-3,08597	9,77E-17
C19orf66	-3,08933	2,05E-07
FNIP2	-3,0935	1,01E-08
CARD10	-3,09473	8,15E-29
CTNNAL1	-3,09747	7,13E-25
APOBEC3B	-3,10454	5,26E-32
KAZN	-3,10997	3,59E-33
RAI14	-3,11227	4,75E-37
ANK1	-3,11342	0,000322
PRKD1	-3,11348	2,84E-11
HTR7	-3,12103	0,000576
TK2	-3,12149	4,67E-22
RRAS	-3,12536	7,97E-15
PAK1	-3,12604	2,23E-23
JAG1	-3,12927	3,74E-36
NXN	-3,13038	1,37E-58
ST6GALNA(	-3,13251	2,74E-15

CTSB	-3,13589	9,57E-26
NXPE3	-3,14007	5,05E-10
EPB41L4A	-3,14644	8,63E-12
RUSC2	-3,14879	7,62E-20
AKAP3	-3,15082	4,92E-05
B4GALT5	-3,15232	6,86E-67
LIMD2	-3,15259	1,13E-13
ENDOD1	-3,15263	2,09E-59
RAPGEF5	-3,15592	4,81E-15
CPQ	-3,15766	3,83E-09
ZNF880	-3,15808	3,07E-06
TMSB4X	-3,15816	5,72E-81
ITGB1	-3,15936	4,81E-84
PRR5L	-3,1653	2,66E-13
ITGAV	-3,16633	3,17E-46
IL6ST	-3,16811	2,91E-50
MAPK10	-3,16896	4,85E-05
COL16A1	-3,17049	1,39E-12
TPM2	-3,17188	1,40E-07
CHRNB1	-3,17291	6,32E-17
DOCK10	-3,17303	1,59E-12
GPATCH11	-3,1817	5,31E-44
GPR37	-3,19067	1,51E-08
TPM1	-3,19357	5,01E-86
OAF	-3,19781	1,24E-30
AKAP7	-3,19793	4,62E-07
ZNF513	-3,19819	1,51E-05
PRKAR2A	-3,20308	3,28E-38
SPATA18	-3,20431	4,14E-07
DHCR24	-3,20558	1,80E-31
TRPV2	-3,20666	0,000178
CRACR2A	-3,21947	3,67E-06
SDC4	-3,22301	1,32E-33
PCDHB5	-3,22375	0,0006
GPC1	-3,22778	1,74E-53
STAMBPL1	-3,23517	7,56E-08
CD163L1	-3,23545	0,000172
EFHD2	-3,24689	2,01E-46
FXYD5	-3,24898	1,60E-40
LINC01515	-3,25089	6,95E-05
GLS	-3,25273	2,24E-66
HIPK2	-3,2579	7,92E-23
UPP1	-3,26199	1,11E-07
DNM1	-3,26251	2,08E-46
DNAJB4	-3,26427	3,81E-10
PLEK2	-3,26614	5,41E-44
CRIP1	-3,26805	9,05E-12
LOC100288	-3,26858	2,38E-05
C1R	-3,27035	5,57E-10
SYNM	-3,27062	4,49E-21

PGM1	-3,27101	2,95E-34
GNG12	-3,27249	2,12E-60
SPHK1	-3,27336	8,79E-40
COL1A1	-3,28091	2,30E-41
BTBD11	-3,28182	6,65E-30
HCAR1	-3,28382	7,56E-10
JAK1	-3,28498	1,47E-53
PXDN	-3,28529	4,70E-44
LGR4	-3,29107	3,38E-33
ABHD6	-3,30476	2,74E-19
ANKRD13A	-3,30542	5,76E-23
VWA5A	-3,30655	0,00019
PTPRH	-3,30768	0,000158
CD82	-3,3142	9,56E-35
AFF3	-3,31554	3,82E-22
HYI	-3,32041	2,82E-15
UBE2L6	-3,32554	4,33E-12
FAM167A	-3,32617	8,22E-05
ALDH3B1	-3,33444	6,15E-09
SERPINE2	-3,33464	4,25E-21
TEX15	-3,34624	2,32E-47
MACC1	-3,3508	1,46E-10
SYNJ2	-3,3552	1,18E-75
SYDE1	-3,35741	1,98E-33
GALNT2	-3,35964	6,76E-92
PALLD	-3,36194	2,34E-34
ST5	-3,36495	1,25E-22
PPM1K	-3,3905	2,12E-12
TRIP10	-3,39742	2,01E-27
CLCF1	-3,42535	7,05E-07
SPRY2	-3,42555	5,47E-21
PALM2	-3,42697	2,08E-08
PITX2	-3,42847	1,14E-12
COPZ2	-3,42991	1,85E-16
TUBB2A	-3,43092	7,37E-16
ARHGAP24	-3,43162	3,16E-11
ADGRB2	-3,44646	2,79E-27
NRK	-3,4477	6,91E-07
DLG4	-3,45199	5,59E-16
CDK14	-3,45267	5,63E-19
GPR176	-3,4589	1,71E-13
PRTFDC1	-3,46787	1,55E-27
C12orf75	-3,46864	4,78E-70
PRNP	-3,47213	1,22E-38
SLC6A8	-3,47383	4,02E-21
NCR3LG1	-3,4781	1,08E-15
ARPIN	-3,48075	1,19E-28
IRF1	-3,48142	2,65E-35
SYTL5	-3,48149	1,70E-14
FEZ1	-3,48271	6,63E-24

CNTNAP1	-3,48621	5,29E-24
PLIN2	-3,48767	6,05E-35
TUBB4A	-3,49164	7,58E-23
COL5A1	-3,49548	9,45E-17
RILPL2	-3,49863	2,99E-13
SLC16A2	-3,50037	5,69E-10
KCTD12	-3,50782	1,23E-26
SLC16A3	-3,50908	4,06E-14
DDX60L	-3,51163	1,68E-19
SBF2	-3,51413	1,04E-18
CITED2	-3,51568	3,72E-55
CACNA2D4	-3,51628	5,26E-05
HOXA4	-3,52966	4,38E-15
KCCAT333	-3,53078	0,000302
NAV1	-3,53219	1,04E-16
CLCN4	-3,53275	3,25E-16
SERINC2	-3,53491	4,56E-29
ZDHHC8P1	-3,53721	8,46E-09
ABCB4	-3,53831	1,46E-09
WIPF1	-3,54297	2,71E-24
SLC9A5	-3,5446	3,30E-08
ZBTB38	-3,55459	5,96E-69
COL27A1	-3,55495	4,14E-14
MATN3	-3,55717	2,99E-11
FAM63B	-3,56061	1,49E-21
ZNF264	-3,56216	2,02E-21
IFI27	-3,56326	5,97E-07
ADPRH	-3,57474	2,89E-16
SLC2A3	-3,57718	1,51E-06
MLLT11	-3,58314	8,50E-17
SORBS2	-3,60133	8,42E-06
GRIK4	-3,60263	0,000345
ACSL4	-3,62889	1,07E-52
PLCE1	-3,63253	8,14E-06
SIPA1	-3,63538	1,86E-38
DCDC2	-3,6387	6,88E-66
TMEM178E	-3,64001	1,90E-82
GABARAPL	-3,64231	2,27E-06
PTGR1	-3,64807	1,33E-25
SLC1A1	-3,65016	6,35E-08
SUSD1	-3,65036	2,91E-50
LOXL1	-3,65364	1,59E-36
MAP1B	-3,65891	1,78E-23
LRP1	-3,66262	6,11E-12
LYST	-3,66482	1,06E-22
WLS	-3,66588	5,19E-32
PARVA	-3,66659	7,51E-55
APOLD1	-3,67131	9,24E-05
TSPAN2	-3,67749	5,75E-05
SEC14L2	-3,68146	1,43E-19

FOXD1	-3,68548	2,67E-09
SFXN3	-3,69401	7,41E-46
DPY19L2P1	-3,69531	6,76E-15
FADS2	-3,69888	1,64E-77
F2R	-3,70183	3,22E-73
PMP22	-3,70398	3,50E-14
SQRDL	-3,70682	8,94E-22
HBEGF	-3,70969	1,59E-31
AFAP1L2	-3,71685	3,55E-36
TBC1D2	-3,71878	2,65E-19
ZBTB47	-3,72175	3,86E-09
STEAP1	-3,72305	1,68E-12
DNM3	-3,72409	0,000264
ATP8B3	-3,72696	0,000272
ISPD	-3,72741	7,53E-06
FCGRT	-3,73428	1,94E-12
CACNA2D3	-3,73989	2,18E-10
HLA-A	-3,74	2,70E-55
HOGA1	-3,74063	4,06E-11
SLFN11	-3,74354	4,65E-30
CD59	-3,74807	1,19E-28
TYW1B	-3,7482	1,19E-09
FILIP1L	-3,75254	2,73E-22
HNF4G	-3,75435	3,20E-09
C3orf18	-3,76425	1,08E-07
SLC16A1-A	-3,76458	0,000176
TPK1	-3,77082	4,64E-29
DUSP7	-3,77854	9,75E-24
CRAT	-3,7818	3,30E-15
AIM1L	-3,78954	6,50E-23
MAF	-3,79673	3,42E-14
B3GNT9	-3,79857	3,84E-08
MFGE8	-3,80543	5,21E-71
MAPRE2	-3,81738	1,46E-36
HMGN5	-3,8197	1,28E-06
ZNF382	-3,83396	4,12E-08
CCDC88A	-3,83533	1,05E-85
WNK4	-3,83536	9,59E-13
PDGFC	-3,83604	4,12E-14
ARHGAP42	-3,84275	3,47E-13
IL4R	-3,84674	4,78E-28
SLC15A3	-3,84773	1,72E-05
TSPAN1	-3,85151	1,36E-10
SLC30A4	-3,85299	1,66E-06
DNMBP	-3,85726	5,12E-50
IL1RAP	-3,87064	2,60E-17
UNC13D	-3,87904	0,000317
PPARG	-3,87924	9,10E-30
MT2A	-3,88119	5,73E-94
HDX	-3,88811	3,62E-06

ADORA2B	-3,89946	7,84E-33
ADAM9	-3,89987	2,89E-135
TMEM45A	-3,90774	1,69E-08
LRRC8C	-3,90786	2,56E-28
BTN3A1	-3,90937	6,42E-11
IL11	-3,91071	2,60E-06
CD27-AS1	-3,91345	1,11E-08
MGC70870	-3,91416	3,58E-07
P3H2	-3,91902	6,59E-76
SYTL3	-3,93643	1,24E-17
STARD8	-3,95355	1,85E-25
C10orf54	-3,95711	0,000744
GBP2	-3,96402	1,09E-06
ANOS1	-3,97721	5,54E-10
HSPA12A	-3,97883	2,40E-51
FOXO1	-3,97913	1,04E-27
PTPN14	-3,98269	1,86E-107
SASH1	-3,99091	1,24E-26
MGLL	-3,99412	8,58E-23
B3GALT5	-3,99707	1,98E-13
KBTBD11	-3,99979	1,48E-23
RP1L1	-4,00921	0,000744
HOXB13	-4,01088	1,12E-06
ADORA1	-4,01838	1,51E-28
HGD	-4,02108	2,13E-05
C10orf11	-4,02963	5,81E-08
PTRF	-4,04627	3,46E-77
CD68	-4,0485	3,52E-14
CMTM3	-4,05857	3,81E-32
TRAF1	-4,06593	6,67E-19
LRP4	-4,06927	1,44E-32
LINC00152	-4,07008	1,32E-21
IL15RA	-4,07069	1,60E-33
EXT1	-4,07201	4,81E-75
HLA-DPA1	-4,07969	0,000775
TFPI	-4,08261	1,27E-17
ADCY9	-4,085	2,96E-72
TUBA4A	-4,08854	5,75E-13
MCC	-4,0906	5,39E-51
PLEKHA2	-4,10422	3,17E-43
DSEL	-4,12413	4,38E-18
CADPS2	-4,12879	1,33E-06
ZNF257	-4,13294	7,95E-10
PTPRB	-4,13923	1,35E-06
C1S	-4,14854	0,000238
FUT4	-4,15591	1,69E-40
ASPH	-4,15608	5,30E-55
NCEH1	-4,15795	1,38E-55
HOTAIRM1	-4,16569	3,95E-12
LOC10192[illegible]	-4,17531	0,000629

FAM149A	-4,17866	3,37E-06
GNB4	-4,18028	2,36E-97
TRIM47	-4,1847	1,11E-46
STAT5A	-4,21253	6,53E-05
ULBP3	-4,21435	5,41E-15
SUGCT	-4,21832	7,97E-11
FGF18	-4,21868	4,29E-34
STX1B	-4,22442	6,37E-09
PORCN	-4,22875	1,66E-40
BTN3A3	-4,24597	7,66E-12
COTL1	-4,24627	8,15E-101
EPDR1	-4,25278	1,87E-51
TGFA	-4,25708	3,45E-33
PALD1	-4,26368	2,85E-42
SSC5D	-4,26952	0,000138
PDE2A	-4,27446	4,35E-17
SHISA2	-4,28196	4,91E-37
TRPC1	-4,2853	1,78E-27
C16orf62	-4,2859	3,17E-88
ARL4C	-4,28711	7,48E-122
FAM13A	-4,28958	5,83E-25
HCG4	-4,29021	6,62E-05
CCDC106	-4,29835	3,64E-18
RDH10	-4,29974	5,20E-81
TNFRSF11B	-4,30251	5,93E-33
MX1	-4,30678	3,00E-21
CYP4V2	-4,31698	4,60E-16
CACNG8	-4,3175	0,000663
ARHGAP29	-4,32476	3,91E-49
RARB	-4,32538	3,98E-19
EPHX4	-4,33045	7,44E-16
ADAMTS9	-4,33533	2,16E-14
HMCN1	-4,3378	4,22E-53
FRMD5	-4,34552	1,52E-52
CAMK2N1	-4,34643	1,47E-38
GAS6-AS2	-4,34764	9,12E-05
POU2F2	-4,35395	7,40E-08
THBD	-4,35923	6,23E-58
VAMP5	-4,36313	1,14E-11
RHBDL2	-4,36676	3,86E-05
SHISA9	-4,36822	1,67E-64
SCARF1	-4,37059	0,000147
MTCL1	-4,37795	6,53E-50
CHST3	-4,38628	1,22E-13
TGFBR2	-4,39314	6,95E-57
ALDH1A3	-4,40002	0,000106
SPRED1	-4,40777	6,83E-26
GAS6-AS1	-4,40908	1,67E-06
PLK2	-4,41074	1,45E-41
FAM171A1	-4,41629	2,90E-92

WNT5B	-4,43077	6,11E-23
WSCD1	-4,4328	1,13E-81
CD44	-4,43534	5,68E-115
RAB11FIP5	-4,43631	3,36E-27
F2RL1	-4,43925	6,09E-64
VANGL2	-4,45775	7,19E-44
ABLIM3	-4,4614	4,51E-34
CDK6	-4,46692	1,40E-19
CFAP54	-4,47554	2,41E-05
FHL1	-4,47633	1,39E-32
GLIPR2	-4,4903	1,17E-59
FBN2	-4,49488	9,41E-36
ARL4D	-4,51608	7,68E-65
IL6	-4,52141	2,35E-07
ERO1B	-4,52671	1,17E-06
HOXB6	-4,53858	1,66E-08
TCN2	-4,5416	0,00027
AMPD3	-4,5425	2,09E-38
ARNTL2	-4,55873	3,18E-65
GULP1	-4,56017	1,23E-51
COL13A1	-4,56462	0,000183
ROR1	-4,58596	7,30E-54
RBMS2	-4,58825	1,74E-69
OXTR	-4,58951	4,57E-39
EPHB1	-4,60528	2,07E-30
HOXB-AS1	-4,62061	0,000196
OPTN	-4,6248	2,26E-19
HCP5	-4,63031	4,40E-06
DSE	-4,63193	2,39E-71
PLEKHA4	-4,63427	1,76E-18
LBX2	-4,64793	9,32E-05
YOD1	-4,6484	2,78E-17
MXRA7	-4,65213	1,44E-66
C6orf99	-4,66586	2,12E-10
SYNC	-4,67392	2,38E-35
LRRK1	-4,67546	5,94E-41
SYCE1L	-4,67711	8,88E-05
DPP4	-4,68447	5,57E-06
SIK1	-4,68884	5,00E-08
SERPINB9	-4,69615	2,00E-41
LETM2	-4,69652	4,20E-25
ST6GAL2	-4,70192	0,000233
TMEM255E	-4,71403	3,24E-08
SNX7	-4,71756	1,21E-51
EPHB2	-4,726	1,73E-188
CDC42EP3	-4,72714	9,71E-94
ULBP2	-4,73674	9,73E-12
SH3PXD2A	-4,74027	9,00E-42
TENM4	-4,7405	1,02E-47
ERICH5	-4,78015	0,00068

ADAMTSL1	-4,78177	3,85E-08
PNMA2	-4,78186	5,65E-16
MAL	-4,78457	5,25E-07
PLA2G4A	-4,78594	2,25E-07
MAFF	-4,79249	1,10E-06
LIF	-4,79709	4,14E-21
TOR4A	-4,80273	4,24E-78
PTGS1	-4,80601	4,04E-07
B3GNT5	-4,80631	2,74E-12
BEST3	-4,80943	6,54E-16
HSPG2	-4,80951	2,94E-101
GLRB	-4,82242	1,26E-24
COL6A3	-4,8276	6,71E-08
KIF7	-4,83119	3,74E-09
ZNF702P	-4,84505	1,77E-37
TBX15	-4,84677	5,80E-10
ETV4	-4,85651	1,46E-11
GLRX	-4,85912	3,03E-10
IL18	-4,86126	3,05E-11
CX3CL1	-4,87329	1,42E-06
ANK2	-4,87579	7,23E-28
B3GNT7	-4,87901	3,62E-05
ELFN2	-4,87909	3,46E-24
ICAM1	-4,8832	6,24E-28
NUDT11	-4,89762	2,05E-22
SEMA3E	-4,89897	3,79E-13
TBXAS1	-4,89965	5,13E-15
ECE1	-4,90941	1,43E-176
SP100	-4,91081	2,93E-10
TMEM133	-4,91131	1,45E-12
MRC2	-4,92235	6,90E-34
SMARCA1	-4,9275	2,45E-40
C17orf51	-4,93637	3,42E-34
TRABD2A	-4,94089	2,26E-07
EPHA2	-4,94358	3,67E-47
SLC12A4	-4,95185	1,53E-18
TRNP1	-4,95321	1,42E-93
ALPK3	-4,95348	2,09E-19
ARMCX2	-4,96049	1,64E-39
LINC00472	-4,96459	1,11E-09
ADAM19	-4,96677	2,52E-80
DRAXIN	-4,96833	0,000808
FLJ32255	-4,97184	6,76E-05
BCAR3	-4,98914	1,94E-123
LTBP2	-5,00513	6,33E-46
LDLRAD2	-5,01702	0,000188
BTN3A2	-5,01827	1,57E-18
ANXA3	-5,02553	1,63E-69
SEMA3A	-5,02594	6,96E-16
CAV2	-5,03138	1,65E-104

VIM-AS1	-5,04365	2,99E-54
DACT1	-5,04824	1,85E-12
FAM101B	-5,05821	4,16E-98
AMPH	-5,06257	1,37E-05
BCL2L15	-5,10303	8,64E-34
LYPD1	-5,10645	9,85E-73
TAGLN	-5,10996	3,99E-49
CMTM7	-5,11201	9,40E-71
TMEM92	-5,11656	9,60E-11
FBXO32	-5,12514	1,32E-25
SHC3	-5,12738	1,63E-19
NDRG1	-5,14677	7,72E-131
ANKLE1	-5,14841	1,62E-06
SSPN	-5,14851	1,83E-54
IGF2BP2	-5,1503	1,23E-147
EEPD1	-5,15638	6,37E-30
ZNF528	-5,16946	4,95E-11
SYNE1	-5,16983	4,66E-37
CRIM1	-5,17649	5,31E-84
NAP1L5	-5,18004	1,67E-07
APLN	-5,18249	5,98E-14
DENND1C	-5,18357	4,31E-11
HOXA2	-5,18606	1,76E-08
ST3GAL2	-5,18763	4,80E-61
LOX	-5,18837	5,09E-83
DUSP6	-5,19693	1,11E-09
SPRY4	-5,20081	1,76E-36
LINC00673	-5,20156	4,82E-08
NFKBIZ	-5,20257	2,43E-69
MARCH3	-5,20376	6,34E-14
GPR143	-5,20479	1,04E-51
HOXA-AS3	-5,20871	4,08E-07
DAGLA	-5,21465	6,98E-102
CNIH3	-5,22294	1,04E-09
CREB3L1	-5,23049	4,44E-20
LIX1L	-5,23057	4,44E-60
TLL2	-5,23647	3,57E-34
DENND3	-5,2379	2,12E-43
VEGFC	-5,24513	3,72E-27
MITF	-5,25449	3,63E-17
HLA-B	-5,2557	9,13E-32
LCAT	-5,25728	4,07E-08
ADRB2	-5,25823	2,89E-06
MEIS2	-5,25853	8,98E-07
SEPT10	-5,26625	6,71E-40
SLC16A7	-5,26639	5,56E-22
ADAMTS15	-5,26718	5,26E-34
TIMP2	-5,26722	1,30E-192
PLXNC1	-5,26782	1,41E-23
RASSF4	-5,27044	2,18E-23

PXK	-5,27069	3,00E-35
ST6GAL1	-5,27291	5,53E-47
DGKA	-5,2773	7,84E-28
ARMC4	-5,29726	1,25E-17
ITGB6	-5,30556	1,78E-06
GSTM2	-5,31184	0,000144
ACO1	-5,31196	2,70E-70
CAMK4	-5,31361	8,80E-28
MBP	-5,32066	1,74E-78
LYN	-5,32289	8,39E-40
MSRB3	-5,3236	2,93E-83
PCDHGB4	-5,32463	2,34E-15
MTAP	-5,32638	3,33E-24
ST6GALNA(	-5,33605	1,62E-06
SLITRK5	-5,34091	1,98E-07
PLEKHG4B	-5,3519	2,01E-32
VNN2	-5,3777	8,69E-05
TAPBPL	-5,38466	1,09E-38
DUSP10	-5,39196	1,73E-41
ADAMTS2	-5,39304	0,000847
GNAI1	-5,40077	8,18E-69
EDN2	-5,40545	6,31E-07
ATP8A2	-5,4065	1,81E-32
AGPAT4	-5,41088	7,06E-52
ANXA8	-5,41797	1,51E-07
GRIK2	-5,41919	0,000906
PLAUR	-5,42194	1,27E-29
C14orf159	-5,42649	9,35E-71
ZNF788	-5,4572	4,22E-05
ABHD8	-5,45729	1,26E-27
LBH	-5,45883	0,000592
NAV2	-5,45906	8,57E-144
DSCAML1	-5,4903	1,71E-10
NAP1L3	-5,49355	0,000524
GATA4	-5,49609	2,60E-20
CREB5	-5,50396	8,76E-05
C3orf52	-5,5105	1,33E-16
SATB1	-5,5349	1,47E-11
VILL	-5,54097	3,24E-05
PROS1	-5,56284	3,36E-25
LIMCH1	-5,56302	2,60E-33
TMCC3	-5,56522	2,75E-53
SIRPA	-5,57215	4,45E-170
PAPSS2	-5,58737	5,38E-06
HOXA7	-5,58918	4,37E-17
NLRC5	-5,62313	1,88E-30
ELK3	-5,63457	1,06E-70
TGFB2	-5,63475	8,07E-27
TLR2	-5,63876	1,96E-08
ARAP3	-5,64119	3,33E-56

DOCK3	-5,64744	3,03E-21
PLA2G16	-5,65715	6,23E-77
KCNMA1	-5,65882	1,64E-41
PPP4R4	-5,66113	5,94E-17
TNFAIP3	-5,66484	4,14E-65
CASP10	-5,67618	3,37E-21
ZNF415	-5,69203	1,47E-09
ITGA5	-5,69452	2,27E-145
ACSL5	-5,69674	3,92E-19
DZIP1L	-5,70729	1,56E-09
HOXA-AS2	-5,71217	1,99E-16
PLTP	-5,72443	2,40E-68
RAB32	-5,73597	1,99E-52
ZNF439	-5,73758	0,000149
SOX7	-5,73814	9,95E-28
GEM	-5,74633	9,03E-75
NOG	-5,75128	1,02E-14
SAMD9L	-5,7522	2,76E-09
HSPB6	-5,75276	3,70E-17
INPP1	-5,76856	2,82E-14
APBB1	-5,77664	5,73E-70
MN1	-5,77779	1,86E-11
FNDC4	-5,77962	3,81E-29
EDIL3	-5,78302	2,28E-23
DAB2	-5,78868	3,09E-17
LONRF3	-5,79482	0,000128
IGFBP6	-5,80159	1,92E-37
TMEM173	-5,80917	6,86E-10
ZPLD1	-5,81251	9,16E-05
CAV1	-5,81552	6,67E-115
TRIM6	-5,82253	1,16E-17
HOXC8	-5,84132	1,96E-26
B3GALT5-A	-5,85358	2,15E-29
LOC101927	-5,8577	9,95E-05
FAT4	-5,85874	2,49E-41
KHDRBS3	-5,8687	0,000906
GRIK5	-5,87345	1,72E-14
SH2D5	-5,88545	3,68E-84
LPXN	-5,88584	3,79E-143
AOX1	-5,89307	2,02E-15
PTPN22	-5,89333	8,85E-27
DAPK1	-5,89809	2,64E-18
RFTN1	-5,89958	7,98E-86
SFRP4	-5,90177	1,56E-06
GNA15	-5,90966	3,65E-13
CPED1	-5,91726	6,40E-11
RIN2	-5,92037	4,45E-67
CSF1	-5,92673	3,18E-78
LOC400043	-5,93301	2,73E-09
SPOCD1	-5,9448	4,94E-13

ADM	-5,95902	1,65E-36
SRPX	-5,96198	8,29E-57
TBX20	-5,99354	1,18E-09
AMIGO2	-6,00824	1,11E-229
CRYBG3	-6,02086	3,56E-52
CASP4	-6,03457	0,00064
TRANK1	-6,03772	2,56E-32
SH3RF2	-6,04489	4,03E-52
LAT	-6,04688	3,55E-15
CFH	-6,06013	2,26E-05
CD109	-6,06958	1,49E-109
S100A2	-6,08311	2,67E-27
RAB3B	-6,10696	5,11E-104
PRKCA	-6,11068	5,82E-124
FHOD3	-6,11526	8,26E-28
LIPE	-6,11667	1,65E-05
COL7A1	-6,1234	1,72E-78
TTLL6	-6,12849	1,51E-05
PYGL	-6,14354	1,39E-06
CTTNBP2	-6,14676	6,28E-15
MCAM	-6,1509	2,30E-58
PIK3CD	-6,17541	1,24E-54
SH2B3	-6,17875	1,30E-201
GJA1	-6,18619	8,64E-28
PPP1R3C	-6,19335	1,26E-05
ARHGAP23	-6,20115	6,19E-51
CALD1	-6,22628	2,12E-37
SV2A	-6,23355	3,08E-49
CACNG7	-6,24829	7,17E-06
SOX9	-6,25767	1,41E-68
LINC01138	-6,26826	6,83E-06
ARMCX4	-6,27377	3,11E-11
ACOT2	-6,31556	4,52E-24
EPSTI1	-6,31954	4,59E-06
EVA1A	-6,32309	2,81E-44
PNPLA3	-6,32446	4,81E-10
CPM	-6,33794	9,90E-54
KIAA1549L	-6,3397	2,14E-123
JAZF1	-6,35759	1,04E-41
ZC3H12C	-6,36653	1,88E-58
DCBLD2	-6,38735	3,66E-156
AKAP12	-6,39469	1,10E-123
UBASH3B	-6,39533	1,33E-99
TIMP4	-6,40864	0,000813
CHST1	-6,41114	1,30E-40
TRO	-6,41722	2,49E-14
APOL6	-6,42314	2,02E-67
PIK3AP1	-6,42562	4,15E-41
ACY3	-6,42873	0,000724
EPB41L2	-6,45231	8,32E-209

SIRPB1	-6,46234	5,11E-12
ARHGEF40	-6,48282	5,26E-47
KCNH1	-6,50496	8,86E-19
LINC01605	-6,53498	1,07E-38
APOL3	-6,53892	2,35E-11
ENTPD3	-6,55007	1,52E-12
PRICKLE1	-6,55028	1,26E-49
MICAL2	-6,55117	4,66E-177
IRAK3	-6,57546	0,000464
LOC101928	-6,58329	0,000485
CLDN1	-6,58331	4,13E-07
PPP1R3G	-6,58748	0,000418
LAMC2	-6,5896	5,21E-117
CD274	-6,59104	4,94E-13
GATA2-AS1	-6,59997	0,000449
CCBE1	-6,60813	1,43E-13
PROCR	-6,61296	8,85E-28
PSMB9	-6,61754	2,29E-70
BEAN1	-6,61828	0,000377
PLAG1	-6,61918	1,58E-09
TRPM8	-6,6236	6,02E-07
KCNE3	-6,62802	0,000354
ANTXR2	-6,62818	5,52E-27
ITGAM	-6,65569	2,29E-10
LOC102724	-6,65587	4,57E-07
EGFR	-6,6561	4,90E-173
ZEB1	-6,66462	2,49E-44
ARL10	-6,6806	0,000314
RAG1	-6,69323	7,11E-16
ZNF426	-6,74785	4,98E-15
APOL1	-6,76836	2,42E-06
IL15	-6,77333	2,59E-10
MILR1	-6,7911	0,000234
SLC4A4	-6,82016	1,30E-15
ZDHHC2	-6,83158	3,93E-59
DPY19L2	-6,83422	1,63E-10
HPGD	-6,84985	1,04E-46
CGNL1	-6,85762	4,85E-47
FHL2	-6,86034	6,99E-37
CAPN2	-6,87173	6,05E-63
ANXA1	-6,87337	1,62E-223
RASSF6	-6,88192	7,09E-11
SCN5A	-6,89013	4,69E-115
CCDC181	-6,90383	4,81E-08
PPP1R14C	-6,9193	1,72E-14
HLX	-6,93535	5,60E-08
LOXL2	-6,94379	1,49E-37
TMEM98	-6,94645	9,15E-104
FLJ45513	-6,95009	0,000107
MID1	-6,95334	5,27E-79

PLSCR4	-6,9756	9,70E-05
ALDH1A1	-6,97607	1,13E-20
NRG2	-7,00981	4,88E-19
IGF2BP1	-7,02565	6,67E-67
ALOX5	-7,05918	7,88E-05
CDKN1C	-7,06624	9,35E-12
BDNF	-7,06776	2,09E-51
TMEM200I	-7,07343	4,04E-22
SPOCK1	-7,09518	1,79E-135
ASB2	-7,09591	8,34E-12
CAND2	-7,11725	6,46E-05
APCDD1L-A	-7,11936	1,37E-08
BATF3	-7,12519	5,29E-05
FBN1	-7,13849	2,63E-125
BANK1	-7,13878	6,91E-05
HAPLN3	-7,14681	1,29E-19
CYP24A1	-7,15192	8,22E-302
PIWIL4	-7,1531	4,88E-05
GLIPR1	-7,1534	2,10E-83
CLSTN2	-7,16039	2,48E-37
THSD1	-7,18946	0,000264
BIN1	-7,20088	1,39E-107
HAS3	-7,20999	4,59E-215
GBP1	-7,21704	6,61E-33
PWARSN	-7,22303	0,001009
PIK3R6	-7,22581	0,000911
GCNT4	-7,22789	0,000921
TSLP	-7,22858	0,000941
RGN	-7,22927	0,000969
PLCL2	-7,22953	2,73E-20
MME	-7,23354	2,22E-05
ABCB1	-7,23972	7,99E-51
FAM49A	-7,24399	7,59E-60
TICAM2	-7,25133	0,000918
ST3GAL6	-7,26935	3,43E-05
MPP1	-7,2761	7,23E-68
MIR614	-7,27782	2,94E-05
KLRC1	-7,28175	0,000734
GCKR	-7,28242	0,000725
HBD	-7,30828	0,00068
ABCA13	-7,31089	0,000647
LINC00667	-7,31548	0,000807
PTCHD4	-7,31914	3,02E-27
LTB	-7,31934	1,71E-09
C1QTNF1	-7,3204	1,50E-22
VAT1L	-7,32194	2,21E-16
FMN2	-7,32362	2,70E-21
TP73-AS1	-7,33423	0,000667
NYX	-7,33818	0,000582
ZNF69	-7,36053	0,000585

SERPINA6	-7,3618	0,000545
KCNT2	-7,36243	0,000532
TWIST1	-7,36351	2,66E-13
CCND2-AS1	-7,36558	0,000532
PTPRR	-7,36558	0,000532
IQCA1	-7,3789	3,54E-23
PIANP	-7,38111	4,49E-17
CACNG6	-7,38175	1,99E-13
CH17-408N	-7,38628	0,000535
ZNF556	-7,38815	0,000482
PAQR9	-7,38938	0,000467
KCP	-7,39062	0,000467
STXBP5-AS	-7,3931	0,000513
HHIPL1	-7,39558	0,000633
SPNS3	-7,40787	0,000744
CYTH4	-7,41341	0,000439
TGM5	-7,41523	0,000419
CORO2B	-7,42032	7,05E-41
HEG1	-7,42277	7,89E-07
ZNF717	-7,42811	1,22E-09
DYSF	-7,42883	1,28E-57
SSTR1	-7,43704	0,000431
FOSL1	-7,43762	4,91E-87
PAK3	-7,43836	1,32E-26
LUM	-7,44421	1,06E-09
IRAK2	-7,44878	0,000981
ENG	-7,44911	4,57E-117
NEK9	-7,45492	9,62E-06
PDGFD	-7,45729	0,000648
ZNF582	-7,46382	0,000347
LINC00312	-7,46793	0,000366
PSG5	-7,47029	0,000442
ALDH2	-7,47262	7,60E-68
ETV5	-7,47729	6,62E-106
TGFB1I1	-7,4789	2,92E-07
GLYATL2	-7,49012	0,000306
KLHDC7A	-7,49456	1,21E-09
SOGA3	-7,50527	1,46E-21
HIF3A	-7,50565	0,000519
PPAPDC3	-7,51026	0,000307
TMEM132I	-7,51651	0,000307
CABP4	-7,52556	1,03E-05
ZNF844	-7,53468	9,87E-06
PIK3CG	-7,53852	0,000257
TRIM38	-7,54162	0,00026
ZNF280A	-7,54301	0,000379
INHBA-AS1	-7,5591	0,000226
KIFC3	-7,55986	3,05E-60
IL4I1	-7,5656	0,000238
C1RL	-7,56719	2,12E-18

LOXL4	-7,57096	2,12E-18
RRN3P1	-7,57939	0,00023
ME3	-7,5864	2,94E-48
LURAP1	-7,59115	8,82E-06
SFTA1P	-7,60475	0,000184
DGKG	-7,60584	2,63E-10
GRIN2B	-7,62123	0,00025
SUCLG2-AS	-7,62307	8,37E-06
IL7	-7,62336	0,000196
CDH10	-7,62652	0,000167
LINC00654	-7,62914	0,000177
C11orf70	-7,63155	7,60E-06
MMP24	-7,63167	7,61E-108
PLA1A	-7,67215	0,000145
CHST4	-7,67317	0,000155
KLHL3	-7,67573	0,000204
PAX8	-7,67804	1,10E-118
HOXB4	-7,68077	5,36E-23
RIN1	-7,68239	2,33E-141
ARSI	-7,68891	0,000133
ST3GAL6-A	-7,68942	0,000129
LOC102724	-7,68992	0,000127
CFI	-7,69293	0,00013
TFPI2	-7,70062	1,18E-05
PRSS12	-7,70324	3,49E-15
LINC00880	-7,71193	0,000113
GIMAP2	-7,71639	0,000147
KIAA1755	-7,71739	0,000167
SULT1C2	-7,72971	0,000113
KCNS1	-7,73313	0,000104
LIN28B	-7,75691	0,000122
MGARP	-7,76678	6,40E-11
CFTR	-7,77176	8,61E-05
CDH5	-7,78894	5,31E-11
NACAD	-7,82738	7,51E-05
MT1M	-7,82967	6,57E-05
DLL3	-7,83103	6,53E-05
TWIST2	-7,83103	6,53E-05
GPC6	-7,84154	9,92E-16
ITPRIPL1	-7,84773	2,71E-06
RNF217	-7,85942	5,09E-16
LINGO2	-7,86761	5,46E-05
KCTD19	-7,8685	5,46E-05
IGFBP7-AS1	-7,86983	5,73E-05
ANKRD7	-7,87161	6,60E-05
DMGDH	-7,87714	2,22E-06
NPM2	-7,88314	5,85E-05
ZNF132	-7,88621	4,99E-05
EVI2A	-7,88665	4,99E-05
DPYSL3	-7,88958	3,18E-200

DKK3	-7,91409	3,07E-139
LOC101928	-7,92058	4,42E-05
ST6GALNA(	-7,9579	3,54E-05
FAM189A1	-7,95808	4,37E-50
GAS7	-7,96197	1,16E-11
LOC729987	-7,96292	5,45E-05
LINC01094	-7,97208	3,70E-05
LAMB3	-7,97235	3,16E-08
B3GALT1	-7,97291	3,47E-05
LOC100130	-7,97291	3,47E-05
LINC00899	-7,97538	3,26E-05
ADAMTS14	-7,97689	7,72E-30
IFFO1	-7,98828	1,06E-16
TNFRSF14	-7,98917	9,99E-12
ZBTB20	-7,98995	1,10E-51
ZBTB18	-8,01148	1,28E-72
LHFP	-8,01589	1,65E-40
ILDR2	-8,01775	3,68E-35
SDPR	-8,01822	3,29E-27
CA2	-8,02618	2,42E-87
EPAS1	-8,03483	1,29E-141
COL12A1	-8,03962	9,03E-104
KANK4	-8,03972	2,56E-05
LRRC34	-8,04011	2,49E-05
ADTRP	-8,04051	2,43E-05
RAB42	-8,04248	2,35E-05
LINC01444	-8,04287	2,37E-05
KAAG1	-8,05546	2,49E-05
UGT8	-8,0563	3,77E-05
KRT34	-8,05897	2,17E-05
RBMS3	-8,06967	2,14E-55
FAM196B	-8,07095	1,00E-06
ARHGAP22	-8,07419	3,53E-09
ZNF214	-8,0895	1,86E-05
SMO	-8,09303	2,16E-17
PRDM8	-8,10584	1,70E-05
SCN4B	-8,10697	1,71E-05
PEG10	-8,10743	1,96E-14
ACOT1	-8,1086	8,11E-07
IL1RL2	-8,12013	1,64E-05
LOC102724	-8,12051	1,61E-05
FOXC2-AS1	-8,13577	1,51E-05
LOC100128	-8,13798	1,47E-05
HS3ST3A1	-8,14737	3,27E-61
CHRDL1	-8,1494	1,59E-05
MAP3K7CL	-8,15488	1,51E-05
LOC100507	-8,16653	1,27E-05
WT1	-8,16834	1,26E-05
ERAP2	-8,18038	2,59E-106
LINC00857	-8,18666	1,67E-12

TM4SF1	-8,19047	7,96E-222
LRRC15	-8,19807	1,09E-05
FAM65C	-8,19948	1,18E-05
GPRIN2	-8,20019	1,26E-05
MYOM2	-8,21218	9,99E-06
VSIG1	-8,21358	1,05E-05
NAV3	-8,21513	0,000664
CCL28	-8,22408	1,03E-05
ARHGAP28	-8,22512	9,59E-06
CTHRC1	-8,2364	2,17E-142
RBMS3-AS3	-8,23691	1,15E-05
PHACTR1	-8,24035	1,04E-12
LINC01204	-8,24206	8,90E-06
ZNF677	-8,24309	9,58E-06
EMP1	-8,24381	1,09E-12
RELN	-8,2443	3,37E-33
ANKRD33B	-8,24635	5,16E-65
TLX2	-8,25304	8,65E-06
CYP11A1	-8,26493	1,04E-05
CD74	-8,26888	3,88E-87
ASIC2	-8,28209	7,05E-06
TNFSF12	-8,28276	6,94E-06
ADAM12	-8,28413	8,30E-35
C1RL-AS1	-8,29276	8,98E-06
SYT11	-8,29432	9,36E-34
IL31RA	-8,29673	6,45E-06
LOC100507	-8,29739	6,50E-06
LRRN3	-8,29739	6,50E-06
EPHA5	-8,29838	6,79E-06
ITGB8	-8,30167	8,52E-61
CXCL3	-8,31077	3,18E-07
HOXD-AS2	-8,32299	5,74E-06
BST1	-8,32379	1,20E-18
RGS4	-8,32493	5,71E-06
TRIM22	-8,32935	1,70E-74
C15orf52	-8,33199	5,01E-07
TMEM220	-8,33465	6,17E-06
PROM1	-8,33979	5,87E-06
TFCP2	-8,34354	9,35E-05
TMBIM1	-8,3529	2,34E-81
SLIT3	-8,359	1,83E-24
ZNF655	-8,36531	0,00031
TNFAIP2	-8,36583	5,85E-89
NDP	-8,36748	5,95E-06
CDO1	-8,37378	5,30E-06
MSC	-8,37566	4,41E-06
RTP4	-8,39094	4,06E-06
SERP2	-8,39217	4,44E-06
MIR100HG	-8,3985	2,31E-07
CDHR1	-8,40176	3,79E-06

PWAR5	-8,40207	3,75E-06
SLC34A2	-8,40268	3,71E-06
JPH2	-8,41181	2,04E-07
NLRP3	-8,41943	1,95E-13
FAM150B	-8,42709	3,32E-06
ZFP92	-8,43131	4,11E-06
ZNF229	-8,43884	3,25E-06
TM4SF4	-8,44538	0,000355
ZNF625	-8,45257	2,86E-06
LINC00704	-8,45317	2,86E-06
LINC01391	-8,45405	2,94E-06
ROBO1	-8,45429	5,32E-144
LYNX1	-8,45613	3,59E-06
COL4A6	-8,46015	1,64E-07
PHYHD1	-8,46814	2,75E-06
HOXB3	-8,47269	1,43E-19
MYRF	-8,48095	6,66E-09
ZNF71	-8,48595	1,04E-13
CSDC2	-8,52813	2,42E-06
LINC01561	-8,53601	1,86E-06
POU4F1	-8,53797	1,90E-06
STEAP2	-8,53999	2,98E-06
ZNF577	-8,55993	1,62E-06
CNN1	-8,56021	1,63E-06
ZFPM2-AS1	-8,57093	1,53E-06
COL4A2-AS	-8,58482	1,63E-06
NEURL3	-8,59321	1,37E-06
ADGRF1	-8,60396	1,31E-06
GPR15	-8,60529	1,29E-06
BCO1	-8,60635	1,35E-06
SNPH	-8,60788	2,13E-20
MDFI	-8,61489	1,24E-06
ZNF671	-8,61647	1,22E-06
UCN2	-8,61779	7,58E-08
ICAM2	-8,62577	7,27E-08
DIRAS3	-8,62915	1,29E-06
BICC1	-8,63505	7,18E-155
ZNF501	-8,63624	1,15E-06
GGT8P	-8,63781	1,08E-06
APCDD1L	-8,66037	9,83E-07
NRP2	-8,66216	2,17E-146
PTX3	-8,66526	1,43E-06
VSTM1	-8,6691	9,24E-07
RARRES1	-8,68081	8,56E-07
LPL	-8,69118	8,09E-07
MMP2	-8,72003	4,06E-21
MMP7	-8,72184	6,86E-07
ZC4H2	-8,72948	1,13E-14
ZNF583	-8,73386	6,92E-07
FAM90A1	-8,74265	6,14E-07

USP44	-8,7441	6,62E-07
SAA1	-8,75595	7,96E-07
PHYHIP	-8,76197	5,54E-07
ZNF502	-8,76268	5,52E-07
LIPC	-8,767	9,26E-07
SMIM6	-8,78079	5,23E-07
ZIK1	-8,78457	5,74E-07
GABRE	-8,80217	4,47E-07
PSG4	-8,81032	4,35E-07
PDE10A	-8,81148	9,70E-08
GCNT2	-8,81221	1,17E-21
UNC13C	-8,82163	4,07E-07
ANXA8L1	-8,83218	4,13E-07
UCA1	-8,83628	4,50E-28
KLHL4	-8,86759	2,99E-15
NTNG1	-8,86985	1,57E-28
NIPAL4	-8,8765	2,99E-07
EMP3	-8,88687	4,25E-76
ARHGAP31	-8,88709	2,86E-07
CATSPER1	-8,88731	2,89E-07
MICU3	-8,92958	2,38E-07
FGFBP1	-8,93298	2,39E-07
SGIP1	-8,94021	2,09E-07
NYAP2	-8,94021	2,09E-07
BIRC3	-8,94162	3,29E-105
SLC2A2	-8,9455	2,76E-07
SAMD5	-8,94761	2,08E-07
UCHL1	-8,95423	4,93E-62
UPK1B	-8,95895	2,01E-07
ZNF518B	-8,97538	1,74E-07
LOC152225	-8,99021	1,72E-07
BAIAP2L2	-8,99158	2,61E-36
RNF128	-8,99719	3,26E-06
HSPB2	-9,00711	1,56E-07
ITGA1	-9,02464	1,08E-08
ZNF385D	-9,02899	1,88E-07
CLIP4	-9,03635	3,12E-37
ATP6V1B1-	-9,04406	1,37E-07
SRPX2	-9,04781	1,35E-07
CCDC80	-9,05139	2,64E-184
HOXB8	-9,0754	1,40E-29
IL32	-9,07742	4,58E-15
ZKSCAN7	-9,08547	1,28E-07
ZNF826P	-9,11169	9,36E-08
KCTD4	-9,11376	8,01E-08
ITGB3	-9,12468	6,56E-09
PPARGC1A	-9,12909	7,40E-08
BNC2	-9,12924	5,99E-09
FBLN7	-9,13075	7,79E-08
PTPRD-AS1	-9,13466	8,54E-08

CLMP	-9,16006	1,21E-30
FOXC2	-9,16992	5,11E-09
NALCN	-9,18042	6,27E-08
TRPC4	-9,1986	5,37E-08
ZNF559	-9,21236	4,69E-08
IL7R	-9,21832	4,57E-08
ZFP82	-9,22373	5,42E-08
C3AR1	-9,2272	4,39E-08
HOXB5	-9,24018	4,00E-08
CDH2	-9,24983	1,62E-129
NLGN1	-9,25389	3,81E-08
FMOD	-9,26544	5,16E-08
EBI3	-9,27656	3,28E-08
AJAP1	-9,28496	5,19E-06
GREM2	-9,29226	3,66E-08
TSPAN18	-9,30148	2,74E-09
PTPN20	-9,30318	2,80E-08
VGLL3	-9,31745	2,58E-08
HHIP-AS1	-9,3194	3,03E-08
POU5F1	-9,32428	2,48E-08
FBLN1	-9,32706	1,79E-07
C8orf31	-9,33077	2,38E-08
OSR1	-9,34942	2,32E-09
BVES	-9,34989	2,18E-08
ADAMTS16	-9,35163	2,20E-08
RNF175	-9,35673	2,08E-08
NKX2-4	-9,36339	2,00E-08
MPP4	-9,38161	1,99E-08
TGFBI	-9,38428	3,27E-86
KCCAT198	-9,39945	2,33E-08
SCN9A	-9,40773	1,93E-17
KCNJ2	-9,40989	1,57E-08
LINC01322	-9,40989	1,57E-08
TNFRSF1B	-9,41555	1,48E-08
ST8SIA2	-9,42771	1,50E-09
S1PR1	-9,43283	1,44E-08
KCNJ16	-9,43298	1,42E-08
TIE1	-9,43929	1,36E-08
NCAM1	-9,445	2,91E-55
KCNQ3	-9,46383	1,20E-08
ZNF215	-9,48345	1,01E-08
SERPINE1	-9,4878	1,70E-10
ZNF667-AS	-9,50711	8,82E-09
DSC3	-9,51561	5,21E-18
SLAMF7	-9,51725	1,50E-08
MSC-AS1	-9,52475	7,99E-09
ZNF470	-9,53769	7,75E-09
ZNF667	-9,57011	6,43E-09
GLI2	-9,57467	6,67E-42
TMEM47	-9,57604	4,05E-18

C11orf45	-9,57613	6,07E-09
HDAC9	-9,59006	5,01E-65
ZNF660	-9,5994	5,22E-09
NEFL	-9,61476	5,03E-09
NPR2	-9,62044	5,51E-10
PLAT	-9,62098	4,64E-09
EREG	-9,62983	4,99E-11
OSBPL3	-9,6452	1,49E-94
ADRA1B	-9,66179	4,98E-09
MPV17L	-9,68069	3,29E-09
TNC	-9,68958	6,24E-74
NEXN	-9,70331	2,79E-27
MIR31HG	-9,71616	2,75E-09
ZNF665	-9,71677	2,68E-09
FGF1	-9,72073	2,80E-09
PSG1	-9,72885	2,83E-09
SERPINB7	-9,72922	3,00E-09
MT1A	-9,74714	2,27E-09
SLFN12	-9,74726	2,26E-09
C10orf55	-9,74907	2,53E-09
GPX7	-9,75089	2,42E-09
SLC43A3	-9,75251	4,27E-05
KCNJ12	-9,76268	2,08E-09
MT1G	-9,79399	2,08E-09
NUDT16P1	-9,79587	1,77E-09
GLIS3	-9,79657	3,94E-08
APOBEC3D	-9,7975	1,74E-09
PRKCQ-AS1	-9,81553	1,55E-09
FSTL1	-9,81758	4,05E-133
SIM1	-9,8339	1,47E-09
DEPDC7	-9,83959	1,34E-09
CCRL2	-9,84038	1,35E-09
GSTP1	-9,84836	2,59E-138
GJC1	-9,85276	1,08E-36
ADAMTS18	-9,85318	2,29E-09
SEC14L6	-9,85733	1,29E-09
MALL	-9,86437	2,26E-09
CSMD3	-9,86504	1,34E-09
GLYATL1	-9,86906	1,21E-09
SLC16A12	-9,87096	1,67E-09
ZNF90	-9,87339	1,15E-09
KLHL14	-9,87384	1,20E-09
PRKCQ	-9,87551	1,21E-09
LY6K	-9,87838	1,16E-09
APOBEC3C	-9,88182	5,12E-62
SFRP1	-9,89247	1,40E-09
NOX4	-9,89499	9,82E-10
BCHE	-9,8963	1,01E-09
CHST9	-9,92229	8,35E-10
ZNF773	-9,92722	8,21E-10

ZNF334	-9,95665	7,08E-10
NLRP1	-9,96291	1,03E-10
P3H3	-9,98177	1,33E-10
CD70	-9,98696	9,18E-20
P2RY8	-10,0063	5,95E-10
CYS1	-10,0158	7,30E-11
IPW	-10,0228	5,25E-10
SUSD5	-10,0294	4,52E-10
LOC72997(	-10,0408	4,46E-10
GBP3	-10,0472	6,63E-11
ZFP28	-10,0696	3,67E-10
C3	-10,0732	9,74E-104
ZNF626	-10,0797	3,72E-10
ITGBL1	-10,1192	2,99E-10
MDFIC	-10,131	6,33E-30
GSPT2	-10,1372	2,43E-10
PEAR1	-10,1579	3,51E-11
AGPS	-10,1583	3,46E-66
CYGB	-10,1696	8,97E-21
CCND2	-10,1704	1,28E-180
SLC22A3	-10,172	2,05E-10
EBF3	-10,1747	1,96E-10
SP140	-10,1922	1,91E-10
PIEZO2	-10,1951	1,97E-10
NXPH2	-10,2005	1,70E-10
SRGN	-10,2007	2,86E-11
MYH16	-10,2088	2,75E-11
PDCD1LG2	-10,226	1,46E-10
NETO1	-10,2315	1,72E-10
ZC3HAV1L	-10,2443	1,31E-10
SNHG18	-10,2938	9,96E-11
HACD4	-10,3098	9,09E-11
TOX	-10,3119	9,95E-11
ARSJ	-10,3246	3,02E-31
DLGAP1	-10,3274	8,09E-11
TLR6	-10,3281	8,34E-11
CPA4	-10,3328	2,72E-31
FAM155A	-10,34	7,67E-11
RARRES2	-10,3404	7,53E-11
DOCK2	-10,3478	1,97E-31
FOXG1	-10,3545	7,05E-11
PSMB8	-10,362	4,65E-41
APOBEC3G	-10,3681	6,79E-11
EYA4	-10,3693	8,13E-11
FGF2	-10,3701	3,12E-41
FOXL1	-10,3824	6,20E-11
TLR3	-10,4218	7,17E-11
CASP1	-10,4649	3,65E-11
LINC01111	-10,4921	3,13E-11
SIGLEC15	-10,4984	3,00E-11

CARD11	-10,5022	3,71E-11
AFAP1L1	-10,5377	2,08E-22
ETV1	-10,5534	4,29E-12
ZFPM2	-10,5536	2,20E-11
MMP14	-10,5653	1,79E-05
GIPC2	-10,5685	2,00E-11
LINC01411	-10,586	1,86E-11
LZTS1	-10,6103	1,56E-11
ZNF320	-10,6104	1,56E-11
BMPER	-10,6133	1,54E-11
GREM1	-10,6134	1,55E-11
CARD6	-10,6206	1,52E-11
ACTBL2	-10,6307	1,50E-11
GABRB3	-10,6494	2,73E-12
ZNF506	-10,6572	1,21E-11
CSF2	-10,6652	1,41E-11
MSN	-10,6879	1,63E-10
DENND2A	-10,6885	9,96E-12
CCAT1	-10,6926	1,03E-11
UBA7	-10,7096	8,92E-12
LINC00958	-10,711	3,64E-44
SKAP1	-10,7219	8,20E-12
ZNF300	-10,7468	7,20E-12
KLK11	-10,7575	6,67E-12
HECW1	-10,7756	6,29E-12
CNRIP1	-10,7958	5,61E-12
ZNF649	-10,8226	4,66E-12
LDHB	-10,8337	0,000189
SENCR	-10,8575	3,96E-12
PAPPA	-10,8651	4,35E-12
BNC1	-10,8924	3,08E-12
INA	-10,8985	3,06E-12
PDGFRB	-10,9023	2,84E-12
CCDC8	-10,9326	2,48E-12
QRFPR	-10,9382	2,34E-12
CXCL2	-10,9438	2,28E-12
ITGA11	-10,9496	2,42E-12
RGS20	-10,9499	2,30E-12
IL1A	-10,9559	2,12E-12
GAL3ST1	-10,9684	2,48E-12
SPP1	-10,9767	5,43E-08
SLC35F3	-10,9776	2,07E-12
VCAN	-10,9843	6,44E-197
ARSE	-10,9886	1,82E-12
SERPINB2	-10,9896	2,17E-12
FIGN	-11,0151	1,97E-12
ZNF280B	-11,0255	1,38E-12
SMIM10	-11,0542	1,16E-12
C7orf31	-11,0737	1,17E-12
SDHAF3	-11,0934	9,63E-13

C1orf186	-11,0943	9,20E-13
GNG11	-11,1149	4,11E-25
PHLDB2	-11,1452	4,85E-80
TAGLN3	-11,1467	6,74E-13
CHST2	-11,1645	6,45E-13
CLEC4E	-11,1652	7,05E-13
SLC35G2	-11,1708	6,74E-13
NTF3	-11,1739	6,00E-13
ANKRD1	-11,2108	1,21E-13
SGK2	-11,217	4,47E-13
HOXA9	-11,2233	4,71E-13
LINC01279	-11,2338	4,05E-13
TLE4	-11,2497	1,00E-13
TDRP	-11,2508	3,71E-13
SPAG16	-11,2535	3,65E-13
CTSZ	-11,2953	1,36E-157
SLFN13	-11,2973	2,84E-13
ZNF175	-11,3014	2,75E-13
GYPC	-11,3052	2,65E-13
CA13	-11,3154	3,00E-13
KCNK3	-11,3466	4,11E-26
SYT14	-11,3576	1,96E-13
HOXA10-A:	-11,3631	1,95E-13
CXCL6	-11,3639	1,88E-13
LPAR1	-11,3667	2,06E-13
SOX17	-11,3679	1,92E-13
ZNF542P	-11,3715	1,83E-13
SLC6A15	-11,3741	1,79E-13
TRHDE-AS1	-11,388	1,88E-13
FGF5	-11,4281	1,47E-13
CD200	-11,4318	1,30E-13
ZSCAN18	-11,4484	1,14E-13
PADI1	-11,4489	1,17E-13
DMD	-11,4576	1,08E-13
STK33	-11,4577	1,08E-13
TLR4	-11,4754	1,05E-13
LINC00839	-11,4981	9,49E-14
TNFRSF9	-11,5702	7,89E-14
HKDC1	-11,5911	5,47E-40
PNMAL1	-11,6086	4,63E-14
FLI1	-11,6184	1,21E-14
UGT2B7	-11,639	4,01E-14
GDNF	-11,6975	2,54E-14
LOC10012€	-11,6985	2,54E-14
GLIS1	-11,7212	2,31E-14
CPNE8	-11,7277	2,19E-14
ADAMTS5	-11,7328	2,12E-14
MYOM3	-11,7455	1,96E-14
C8orf88	-11,7499	1,86E-14
ANGPTL2	-11,7629	1,79E-14

NID2	-11,7678	1,70E-14
IFI16	-11,7834	2,56E-28
LINC00941	-11,8021	1,38E-14
CLDN2	-11,8541	1,07E-14
PRKCDBP	-11,8599	1,02E-14
CYTL1	-11,8647	9,38E-15
FBXL7	-11,9003	7,61E-15
NEGR1	-11,9124	7,47E-15
BHMT2	-11,9353	6,11E-15
ARHGAP31	-11,9584	1,72E-15
PDZK1IP1	-11,9711	5,80E-15
CXCL1	-11,9821	4,62E-15
GRAMD1B	-11,9931	4,33E-15
TNFRSF10D	-11,9959	4,53E-15
TMEM200A	-11,9965	4,35E-15
DAW1	-12,0519	3,03E-15
CRYAB	-12,0657	2,83E-15
GNE	-12,072	3,03E-15
F2RL2	-12,0952	2,32E-15
TCF4	-12,1191	2,03E-15
C5orf42	-12,1703	1,47E-15
GCNT3	-12,1941	1,28E-15
HRH1	-12,1976	1,26E-15
ADAMTS3	-12,2004	1,24E-15
DPYD	-12,2305	1,03E-15
FOXL2NB	-12,2377	1,01E-15
HHIP	-12,242	9,68E-16
ZEB2	-12,2422	9,86E-16
PREX2	-12,2575	8,64E-16
SEMA5A	-12,2621	8,43E-16
FRG1CP	-12,271	8,03E-16
C14orf105	-12,2855	9,17E-16
FOXE1	-12,2959	7,15E-16
TMEM25	-12,3097	6,29E-16
AASS	-12,3217	5,89E-16
COL4A2	-12,3332	2,96E-196
KCNIP1	-12,3335	5,51E-16
AKR1B1	-12,3338	1,04E-86
NRG1	-12,3398	5,57E-16
COL8A1	-12,3452	4,35E-31
ABCA1	-12,3583	5,16E-16
TNFSF10	-12,3727	1,51E-16
CLDN16	-12,3738	4,27E-16
ZFHX4	-12,3865	3,93E-16
ANPEP	-12,3944	3,83E-16
CDH6	-12,4006	1,27E-16
ZCCHC11	-12,4301	3,50E-16
SIGMAR1	-12,4322	1,47E-31
EDNRA	-12,449	2,79E-16
CDA	-12,4701	2,36E-16

RRAD	-12,4731	2,33E-16
GALNT5	-12,5072	1,95E-16
ZBED2	-12,5264	1,66E-16
EVC	-12,5459	1,52E-16
BHLHE41	-12,5629	4,93E-17
TMEM246	-12,575	1,25E-16
ATP10A	-12,5791	1,28E-16
MMP1	-12,649	7,83E-17
SNAI2	-12,6496	7,82E-17
ADAMTS1	-12,6541	2,81E-17
CDIP1	-12,6789	6,95E-17
EMC10	-12,6821	7,97E-17
RAB38	-12,7007	5,65E-17
TCEAL8	-12,7522	4,16E-17
DZIP1	-12,7556	4,11E-17
HOXA10	-12,7697	3,68E-17
C2CD2	-12,8043	3,14E-17
LAYN	-12,8317	2,63E-17
CD40	-12,8351	2,51E-17
SGCE	-12,868	2,09E-17
PTPRD	-12,9177	1,47E-17
CELF2	-13,0558	6,00E-18
PPAPDC1A	-13,0596	6,06E-18
MARCH4	-13,0696	5,72E-18
AKT3	-13,0839	5,03E-18
PKIA	-13,0938	4,79E-18
MAGI2-AS3	-13,1407	3,53E-18
STAC	-13,2119	2,21E-18
PVRL3	-13,2333	1,94E-18
MAMDC2	-13,2789	1,46E-18
FOXL2	-13,2956	1,35E-18
PAX6	-13,2967	1,31E-18
TM4SF18	-13,3286	1,14E-18
AXL	-13,3458	2,68E-53
AK5	-13,3736	8,33E-19
MOXD1	-13,3937	7,21E-19
WBP5	-13,3974	6,68E-19
GDF5	-13,4078	6,65E-19
JAM3	-13,4088	6,82E-19
CXCL5	-13,4181	6,21E-19
HAVCR1	-13,4391	6,23E-19
TGM2	-13,4464	9,59E-255
CLDN11	-13,6307	6,35E-20
CYBRD1	-13,6679	1,16E-19
NRIP3	-13,6772	1,08E-19
NTM	-13,7647	6,20E-20
G0S2	-13,8042	4,65E-20
COL4A1	-13,811	4,29E-57
IGFBP7	-13,8128	4,53E-20
NT5E	-13,8278	3,98E-20

HNF1B	-13,8694	3,01E-20
KIAA1644	-13,9174	2,19E-20
PTPRM	-13,9213	2,14E-20
LAMA1	-13,9564	1,73E-20
STK32B	-13,9595	1,69E-20
TRHDE	-13,9987	1,30E-20
RAC2	-14,0043	1,25E-20
MYL9	-14,0711	8,13E-21
NNMT	-14,099	6,42E-21
EFEMP2	-14,1494	4,58E-21
TRIM55	-14,1497	1,86E-21
NID1	-14,1539	4,44E-21
IGF2BP3	-14,2306	2,66E-21
APBB1IP	-14,2869	1,84E-21
CDH13	-14,4374	7,45E-22
MT1L	-14,5179	3,84E-22
LAMA4	-14,5901	2,24E-22
HMGA2	-14,6683	5,23E-23
CNN3	-14,6704	5,07E-23
VCAM1	-14,6711	1,32E-22
DFNA5	-14,7218	9,04E-23
SALL1	-14,7612	6,82E-23
TINAGL1	-14,8158	4,83E-23
INHBA	-14,9327	2,06E-23
MT1E	-14,9636	1,64E-23
ALPK2	-15,0137	1,19E-23
CDH4	-15,1178	5,39E-24
PDPN	-15,1213	5,39E-24
ETS1	-15,1838	3,38E-24
GPX1	-15,2205	2,60E-24
IGFN1	-15,3042	1,45E-24
KIRREL	-15,3837	8,03E-25
THBS2	-15,6934	8,13E-26
PLAU	-15,7782	4,48E-26
SPARC	-16,4677	2,32E-28
LRRN4	-17,162	9,63E-31
PTGIS	-17,5742	3,29E-32
FLNC	-18,4392	2,31E-35
